# Characterization of glycosyl dioxolenium ions and their role in glycosylation reactions

**DOI:** 10.1038/s41467-020-16362-x

**Published:** 2020-05-29

**Authors:** Thomas Hansen, Hidde Elferink, Jacob M. A. van Hengst, Kas J. Houthuijs, Wouter A. Remmerswaal, Alexandra Kromm, Giel Berden, Stefan van der Vorm, Anouk M. Rijs, Hermen S. Overkleeft, Dmitri V. Filippov, Floris P. J. T. Rutjes, Gijsbert A. van der Marel, Jonathan Martens, Jos Oomens, Jeroen D. C. Codée, Thomas J. Boltje

**Affiliations:** 1 Leiden University, Leiden Institute of Chemistry, Einsteinweg 55, 2333 CC Leiden, The Netherlands; 2 Radboud University Institute for Molecules and Materials, Heyendaalseweg 135, 6525 AJ Nijmegen, The Netherlands; 3 Radboud University Institute for Molecules and Materials, FELIX Laboratory, Toernooiveld 7-c, 6525 ED Nijmegen, The Netherlands

**Keywords:** Carbohydrate chemistry, Reaction mechanisms, Computational chemistry

## Abstract

Controlling the chemical glycosylation reaction remains the major challenge in the synthesis of oligosaccharides. Though 1,2-*trans* glycosidic linkages can be installed using neighboring group participation, the construction of 1,2-*cis* linkages is difficult and has no general solution. Long-range participation (LRP) by distal acyl groups may steer the stereoselectivity, but contradictory results have been reported on the role and strength of this stereoelectronic effect. It has been exceedingly difficult to study the bridging dioxolenium ion intermediates because of their high reactivity and fleeting nature. Here we report an integrated approach, using infrared ion spectroscopy, DFT computations, and a systematic series of glycosylation reactions to probe these ions in detail. Our study reveals how distal acyl groups can play a decisive role in shaping the stereochemical outcome of a glycosylation reaction, and opens new avenues to exploit these species in the assembly of oligosaccharides and glycoconjugates to fuel biological research.

## Abstract (web summary)

Dioxolenium ion intermediates formed from remote positions are hypothesized to direct stereoselective glycosylations. Herein we combine infrared ion spectroscopy, DFT calculations and synthetic work to characterize and study these dioxolenium ions and their role in stereoselective glycosylation reactions.

## Introduction

**Fig. 1:**
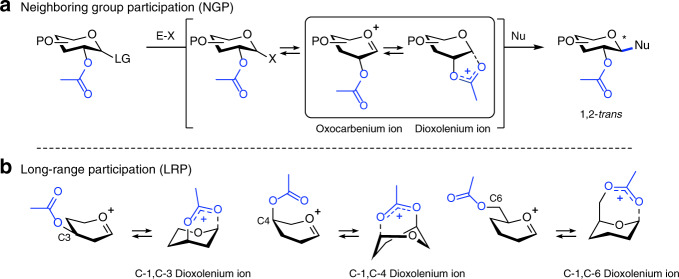
NGP and LRP in glycosylation reactions offers an opportunity to control the stereoselectivity of glycosylations. Schematic representation of possible reactive intermediates in NGP (**a**) and LRP (**b**). P protection group, E–X promoter system, Nu nucleophile.

The principle challenge in chemical oligosaccharide synthesis is the stereoselective installation of glycosidic bonds^1–4^. Glycosidic bonds connecting monosaccharides can either exist as 1,2-*trans* or 1,2-*cis* diastereomers and the nature of the linkage has a profound influence on the structure and function of the glycans. The most common approach to chemically create glycosidic bonds is a nucleophilic substitution reaction between a glycosyl donor carrying an anomeric leaving group, and a glycosyl acceptor containing a nucleophilic alcohol. The stereochemical outcome of glycosylation reactions can be controlled using neighboring group participation (NGP)^5,6^. Acyl groups at the C-2 position of glycosyl donors can engage in NGP affording bicyclic C-1,C-2 dioxolenium ion intermediates that react in a stereospecific manner with glycosyl acceptors to afford 1,2-*trans* products (Fig. 1a)^7^. NGP of an *O*- or *N*-acyl functionality at C-2 applies to a wide variety of monosaccharides, has enabled the stereoselective synthesis of oligosaccharides both in solution and on solid support and is one of the pillars upon which chemical oligosaccharide synthesis stands^8,9^.

By definition, NGP by a C-2 acyl group only allows access to 1,2-*trans* glycosides and is not applicable to the synthesis of C-2-deoxy or 1,2-*cis* glycosides. Long-range participation (LRP) of acyl functionalities farther away from the anomeric center, i.e., placed on the C-3, C-4, or C-6 hydroxyl groups, has also been suggested to direct the stereoselectivity of glycosylation reactions (Fig. 1b). Importantly, LRP potentially allows for the utilization of the relative stereochemistry of C-3, C-4, or C-6 groups to control the facial selectivity in glycosylation reactions thereby enabling the stereoselective synthesis of C-2-deoxy and 1,2-*cis* glycosides. However, contradictory results have been reported and there is an ongoing debate as to the role and strength of this stereoelectronic effect^10–24^. Indirect proof for LRP has been derived from the stereochemical outcome of glycosylation reactions and studies using model systems^24^. The ability of acyl substituents positioned at C-3, C-4, or C-6 to engage in LRP likely depends on their distance and stereochemical orientation with respect to the cationic center. In addition, the relative configuration of the neighboring substituents may influence LRP by steric and electronic effects. Hence, differences in LRP of acyl groups in glycosyl donors are to be expected. Due to the instability of the intermediate dioxolenium ions and their short lifetime in solution, it is exceedingly difficult to detect and characterize them and has only been reported with respect to NGP^25,26^. Although glycosyl cations have been characterized in superacid solution by nuclear magnetic resonance (NMR), protonation of the acetyl groups under these conditions prevents the assessment of their ability to engage in NGP and LRP^27,28^. This hampers our fundamental understanding of LRP and prevents its systematic development to advance stereoselective oligosaccharide synthesis^27–30^.

Herein, we report the use of infrared ion spectroscopy (IRIS) to characterize glucosyl, mannosyl, and galactosyl dioxolenium ions formed via LRP and show how the stability and reactivity of these species depend on the position and configuration of the acyl group. Using density–functional theory (DFT) calculations, the conformational energy landscape (CEL) of these glycosyl cations was systematically mapped in gas and solution-phase. Finally, through a series of glycosylation reactions, employing a set of model nucleophiles of gradually decreasing nucleophilicity, we mapped the importance of the remote dioxolenium ions in glycosylation reactions. The combination of these techniques established the strength of LRP as 3-Ac-Man » 4-Ac-Gal > 3-Ac-Glu ∼ 3-Ac-Gal > 4-Ac-Glu > 4-Ac-Man ∼ 6-Ac-Glc/Gal/Man. The establishment of dioxolenium ion intermediates as possible reactive intermediates in glycosylation reactions opens up avenues to exploit these species for more stereoselective glycosylations.

## Results

### IRIS of glycosyl cations

**Fig. 2:**
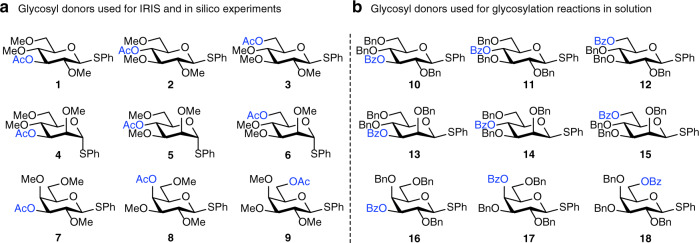
*S*-phenyl donors equipped with ester protection groups on systematically varied positions on the ring. **a** Glycosyl donors used for IRIS experiments and DFT computations. Donors **3**–**9** and **13**–**15** were converted into their corresponding sulfoxides prior for the IRIS experiments to improve the yield of the glycosyl cation generation^33^. **b** Glycosyl donors used for chemical glycosylation reaction in solution.

Recently, we and others have employed IRIS to characterize both glycosyl oxocarbenium and dioxolenium ions in the gas-phase^31–34^. In this method, glycosyl donors are introduced into the mass spectrometer via electrospray ionization (ESI) and, in a tandem-mass spectrometric (MS^2^) scheme, glycosyl cations are formed from the isolated donors by collision-induced dissociation. This allowed us to generate the “naked” glycosyl cations in the absence of a counter ion and solvent molecules and characterized them using multi photon IRIS^32,33^. The IR spectra showed diagnostic vibrational bands and were used to characterize both C-1,C-2 dioxolenium ions and oxocarbenium ions^32^. In addition, we provided the first example of dioxolenium ions formed by LRP in uronic acid derivatives^33^. To systematically investigate whether LRP plays a role in glycosylation reactions we assembled two sets of glycosyl donors, derived from the most commonly used pyranosides, d-glucose, d-mannose, and d-galactose donors, and equipped these with an acyl group at either the C-3, C-4, or C-6 hydroxyl group. The first set (Fig. 2a) comprises *S*-phenyl donors equipped with methyl ethers and acetyl esters (**1**–**9**) used for the IRIS studies and computational studies to minimize computing costs. The second set (Fig. 2b) features benzyl ethers and benzoate esters (**10**–**18**) used in a matrix of model glycosylation reactions, as these represent the most commonly used protecting groups in synthetic carbohydrate chemistry. The benzyl ether and benzoate esters are structurally very similar, while differing significantly in electronic properties and their ability to stabilize an oxocarbenium ion^35,36^.

**Fig. 3:**
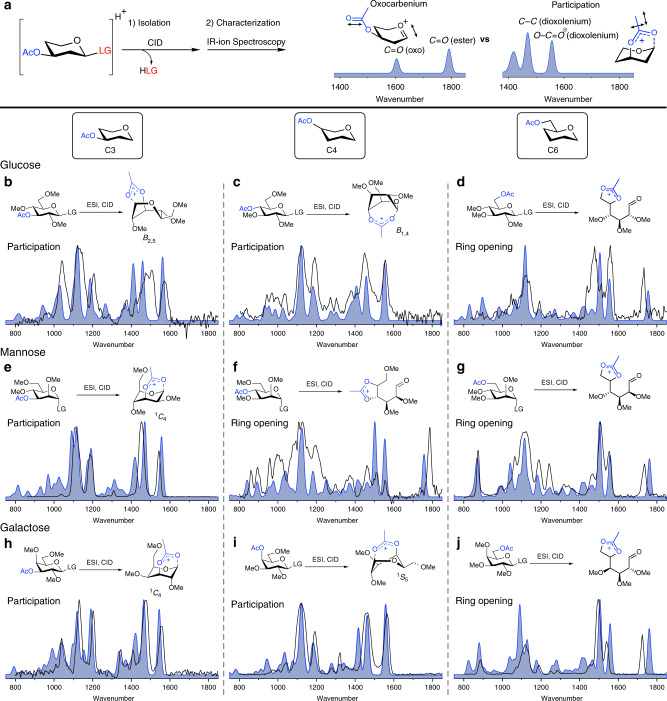
Infrared ion spectroscopy of glycosyl cations. **a** Oxocarbenium ions and dioxolenium ions give different diagnostic peaks (in blue). Overview of a comparison of the computed IR-ion spectra (filled blue) and the measured IR-ion spectra (black line) of the glycosyl cations derived from glucosyl (**b**–**d**), mannosyl (**e**–**g**) and galactosyl (**h**–**j**) donors **1**–**9**. Ring-opening of donors **3**, **5**, **6**, and **9** have been presented as accessible structures, their exact conformation is presented in Supplementary Figs. 3, 5, 6, and 9 and coordinates presented in the Supplementary Data file.

Glycosyl cations derived from **1** to **9** were formed from precursor ions using tandem-MS (see Supplementary Figs. 1–9). IRIS of the glycosyl cations was carried out using the FELIX infrared free electron laser (IR-FEL) operating in the 700–1850 cm^−1^ frequency range which is well suited to assign oxocarbenium and dioxolenium ion structures (Fig. 3a)^37^. For example, oxocarbenium ions derived from **1** to **9** can be assigned on the basis of their characteristic C_1_=O_5_^*+*^ stretch (∼1600 cm^−1^) and preservation of the acetyl C=O stretch near 1800 cm^−1^. Conversely, the formation of a dioxolenium ion leads to the absence of the acetyl C=O stretch and the C_1_=O_5_^+^ stretch and appearance of a dioxolenium ion O-C=O^+^ stretch- (∼1550 cm^−1^) and bending mode (∼1500 cm^−1^). Accurate spectral assignments were made by comparing the experimental IR spectra with computed IR spectra obtained by high-level DFT calculations (B3LYP/6–31+ +G(d,p))^38^. The experimental IR spectra of **1**–**9** are presented in Fig. 3b–j (black line) together with the best matching calculated spectra (blue filled).

The IR spectra of the gluco-, manno-, and galacto*-*C-3 acetyl derivatives **1**, **4**, and **7** all confirm LRP of the C-3 acetyl group (Fig. 3b, e, h), as indicated by a characteristic dioxolenium O-C=O^+^ stretch (∼1550 cm^−1^) and the absence of an oxocarbenium C_1_=O_5_^+^ or acetyl C=O stretch. The DFT calculated IR spectra of the formed dioxolenium ions matched well with the experimentally obtained spectra, while the computational spectra of the possible oxocarbenium ions derived from **1**, **4**, and **7** did not (Supplementary Figs. 1, 4, and 7). The calculated energy difference between the dioxolenium ions and the corresponding oxocarbenium ions also indicate LRP to be favorable for the C-3-acyl glycosides as all C-1,C-3 dioxolenium ions are lower in energy than their oxocarbenium ion counterparts (vide infra). From these experiments, it is clear that LRP of the equatorial C3-acetyl group of gluco-, manno-, and galactopyranosides is favorable.

IRIS of C-4 acetyl derivatives **2**, **5**, and **8** also produced characteristic IR spectra (Fig. 3c, f, i, black line). The IRIS spectra of glucoside **2** and galactoside **8** showed an absence of a C=O ester stretch and instead showed diagnostic dioxolenium ion signals. In these cases, the agreement between the experimental and calculated IR spectra clearly indicate LRP. Alternative structures lacking LRP were calculated but did not match the experimental spectrum (Supplementary Figs. 2 and 8). The IRIS spectrum of the ion resulting from C-4 acetyl mannose donor **5** showed distinctive dioxolenium absorptions at 1550 and 1500 cm^−1^ and a significant band at 1790 cm^−1^ suggesting the presence of a carbonyl functionality. A mixture of dioxolenium ions formed by LRP and oxocarbenium ions could explain this observation. However, comparing and mixing the DFT calculated spectra of these ions did not lead to a good match with the experimental spectrum (Supplementary Fig. 5). The only structure that was in good agreement with the experimental spectrum is the dioxolenium ion, formed by attack of the C-4 acetyl on the C-5 of the initially formed oxocarbenium ion (Fig. 3f). This leads to ring opening and the formation of the C-4,C-5 dioxolenium ion with an aldehyde functionality at C-1. To the best of our knowledge, this type of rearrangement has not been reported before and the experimental conditions likely promote the formation of this species. Hence, we conclude that the C-4 acetyl in mannose does not directly engage in LRP at the anomeric center. Taken together, these results show that the axial and equatorial C-4 esters in the glucose and galactose ions can engage in LRP, while the mannose ion provides a C-4,C-5 dioxolenium ion.

Finally, the cations of C-6 acetyl glycosides **3**, **6**, and **9** were investigated (Fig. 3d, g, j, respectively). In all cases, a strong absorption near 1730 cm^−1^ was observed indicating the presence of a carbonyl functionality. However, a clear dioxolenium ion signature (∼1550 cm^−1^) was also observed. Again, neither the DFT calculated IR spectra of the oxocarbenium ion or LRP dioxolenium ion nor a mixture of the two matched with the experimental spectrum (Supplementary Figs. 3, 6, and 9, respectively). Similar to the C-4 acetyl mannose donor **5**, the experimental spectrum was matched best with calculated spectra corresponding to the ring-opened structures featuring a C-5,C-6 dioxolenium ion, producing both the aldehyde C = O and the dioxolenium O-C=O^+^ stretching bands. This suggests that C-6 acetyls are unlikely to provide LRP.

To verify that LRP in the methyl ether/acetyl ester protected series is similar to their benzyl ether/benzoyl ester counterparts we performed IRIS experiments with the mannosyl set **13**–**15** (Supplementary Figs. 10–12) and compared their spectra to those of **4**–**6** (Fig. 3e–g). These experiments confirmed that the LRP behavior of these two sets of glycosyl donors is the same in the IRIS experiments.

### Computing CEL of glycosyl cations

**Fig. 4:**
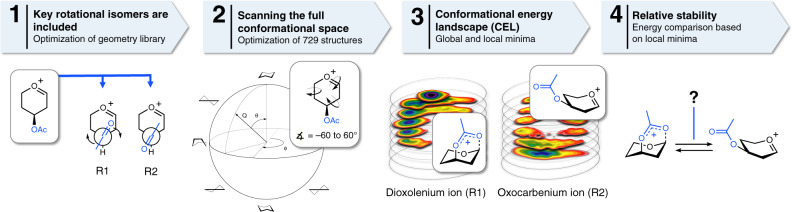
Overview of the workflow to map the relative stability of glycosyl dioxolenium- and oxocarbenium ions. (1) Two rotamers are used to probe long-range participation: R1 makes it geometrically feasible to form dioxolenium ions, where R2 generates the free oxocarbenium ion. (2) The complete conformational space of the 6-membered rings was scanned by computing 729 prefixed structures per rotamer; A few canonical conformations (chair, half-chair, envelope, and boat) are depicted. (3) The associated energies were graphed on slices dividing the Cremer–Pople sphere; the CEL map of the R1 rotamer and CEL map of the R2 rotamer. (4) Based on the CEL maps of R1 and R2 the relative stability of both intermediates can be evaluated.

**Fig. 5:**
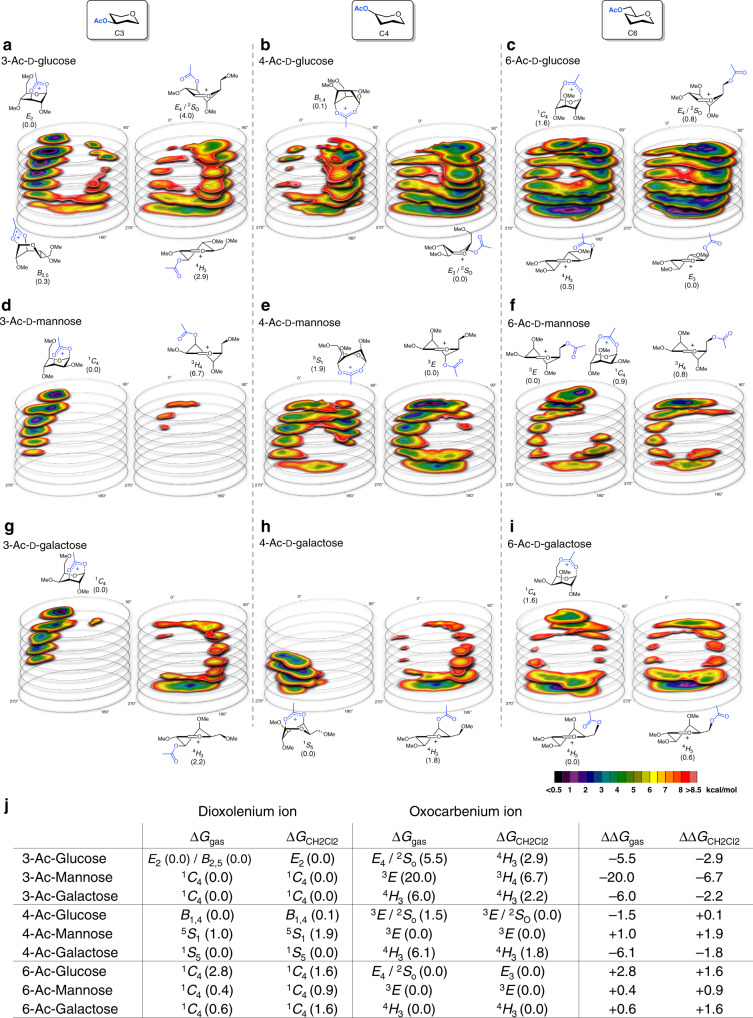
CEL maps of selected glycosyl cations in which the local minima identified are shown with their respective energy^42^. Two acetyl ester rotamers (R1 and R2) were considered for all computed glycosyl cations generating two sperate CEL maps. All energies are as computed at PCM(CH_2_Cl_2_)-B3LYP/6-311G(d,p) at 213.15 K and expressed as solution-phase Gibbs free energy. CEL maps for C3-acetyl pyranosyl ions (**a**–**c**), C4-acetyl pyranosyl ions (**d**–**f**), C6-acetyl pyranosyl ions (**g**–**i**). **j** Table summarizing the relative energy of the dioxolenium and oxocarbenium ion conformers in the gas- and solution-phase.

To understand why some acetyl esters engage in LRP and lead to the formation of bicyclic dioxolenium ions from the parent oxocarbenium ions, while others do not, we computationally investigated their relative stability. We recently developed a DFT protocol to compute the relative energy of a large ensemble of oxocarbenium ion conformers, filling the complete conformational space these cations can occupy and plotted their relative energy to afford CEL maps^29,39–41^. Employing this method, we are able to find low energy conformers and relevant (conformational) pathways which connect these on the CEL. We adopted this method here (Fig. 4) to evaluate the relative stability of the oxocarbenium and dioxolenium ions derived from **1** to **9** (Fig. 5). Two rotamers of the acetyl ester were taken into account and separately visualized as a CEL map: rotamer 1 (R1) in which the acetyl is pointing toward C-1 making LRP geometrically feasible, and rotamer 2 (R2) in which the acetyl points away from C-1 making the inspection of the oxocarbenium ion possible. The geometry of all the conformers was optimized by DFT using the hybrid functional B3LYP and the basis set 6–311 G(d,p), which presents an practical trade-off between computing time and accuracy (for more information see Supplementary Methods). To probe the difference in glycosyl cation structure between the IRIS gas-phase experiments devoid of solvent and glycosylation experiments performed in solution (vide infra), CEL maps were generated for ions formed in the gas-phase at room temperature and in solvent (computationally evaluated using a polarizable continuum model, see SI) at −60 ^o^C at which the experimental glycosylations take place, respectively. Figure 5a–i depicts the maps for the solution-phase ions, while Fig. 5j summarizes the relative energy of the structures found in the solution-phase and the gas-phase (all gas-phase CEL maps can be found in Supplementary Fig. 25).

Figure 5a–c shows the solution-phase CEL maps of the C-3 acetyl protected glucosyl, mannosyl, and galactosyl dioxolenium (R1) and oxocarbenium ions (R2). The computed local minima (in dark and purple), show that formation of the dioxolenium ions is energetically favorable. For the gas-phase, the maps are similar but with larger energy differences between the lowest energy dioxolenium and oxocarbenium ions (Fig. 5j). The comparison shows that the energy difference is largest for the C-3 acetyl mannose system, with the most favorable mannose C-1,C-3 dioxolenium ion adopting a ^1^*C*_4_-conformation. The glucose and galactose ions benefit from LRP with the *E*_2_-glucose and ^1^*C*_4_-galactose C-1,C-3 dioxolenium ions being 2–3 kcal/mol more stable than the corresponding lowest energy ^4^*H*_3_-oxocarbenium ions. In the mannose system the lowest energy oxocarbenium and dioxolenium ions are close in conformational space, indicating that only a small conformational change is required for the formation of the dioxolenium ion from the oxocarbenium ion. In the glucose and galactose systems, the lowest energy oxocarbenium and dioxolenium ions are found in different regions of the conformational space. Hence, transition from the initially formed oxocarbenium ions into the more stable dioxolenium ions requires a conformational change accompanied by the crossing of a significant energy barrier. Overall the CEL maps suggest that participation by a C-3 acyl group can be favorable for all three diastereoisomeric ions studied, which is corroborated by the IRIS experiments and is most beneficial in the mannose configured C-3 acetyl system.

A similar analysis of the C-4 acetyl systems (Fig. 5d–f) reveals important differences between the glucose, mannose and galactose systems, again consistent with the IRIS experiments. The participation of the C-4 acetyl in the mannose ion is unfavorable, while the C-4 acetyl glucose and galactose systems benefit from LRP. The formation of the galactosyl C-1,C-4 dioxolenium ion, adopting a ^1^*S*_5_-like structure, from the ^4^*H*_3_-oxocarbenium ion requires only a minimal adjustment of the sugar ring conformation and is therefore facile. For the glucosyl C-4 acetyl case this requires significantly more structural rearrangement from the *E*_3_-oxocarbenium ion to the lowest energy *B*_1,4_-structure. Furthermore, the computations indicate that the glucose C-1,C-4 dioxolenium ion is more stable than the oxocarbenium ion in the gas-phase, while the relative energy of both ions is similar in solution. The relative instability of the mannosyl C-1,C-4 dioxolenium ion may be due to the *pseudo*-axial orientation of all substituents in the ^5^*S*_1_-like structure, with the C-2 and C-3 substituents experiencing unfavorable eclipsing interactions that are not present in the glucose and galactose dioxolenium ions.

Finally, the C6-acetyl systems were probed. As can be seen from Fig. 5g–i, the energy difference between the two acyl rotamers (R1 and R2) is small, indicating that LRP does not lead to significant stabilization of the ions.

### Model glycosylation reactions

Finally, to correlate the IRIS and CEL map findings to solution-phase experiments, we probed the influence of LRP in glycosylation reactions using **10**–**18** (Table 1b, entry 2–4, 6–8, and 10–12, respectively). To this end, we performed a matrix of glycosylation reactions with a set of model alcohol nucleophiles of gradually decreasing nucleophilicity^43,44^. The trends observed relate to changes from an S_N_2-type substitution reaction of the covalent intermediate (e.g., a glycosyl triflate) for the most nucleophilic alcohols, to reactions involving more oxocarbenium character for the poorest nucleophiles (Table 1a). The glycosylation reactions were performed under pre-activation conditions using diphenyl sulfoxide (Ph_2_SO)/triflic anhydride (Tf_2_O) as an activator and the results compared to donors bearing solely benzyl ether protecting groups (Table 1b, entry 1, 5, and 9)^45^.

**Table 1:**
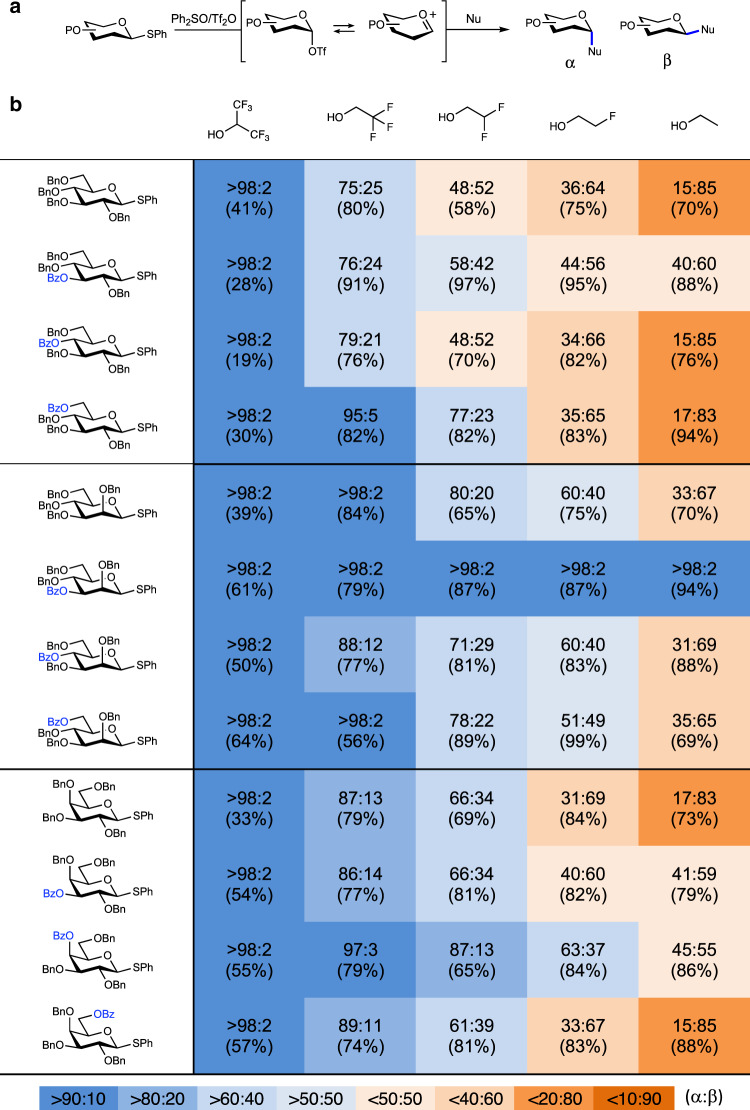
Experimentally found stereoselectivities for model glycosylation reactions.

Experimental conditions: pre-activation based glycosylation conditions; nucleophile (2 eq.), Tf_2_O (1.3 eq.), Ph_2_SO (1.3 eq.), TTBP (2.5 eq.), DCM (0.05 M), −80 to −60 °C. The stereoselectivity of the reaction is expressed as alpha:beta and based on ^1^H-NMR of the purified compounds. In all cases, the NMR spectra for both the crude and purified compounds are compared to analyze whether the measured stereoselectivity did not alter upon purification.

The glycosylations of the C-3 benzoyl donors reveal a shift in stereoselectivity with respect to their C-3 benzyl counterparts towards the side of the α-products, formed on the S_N_1-side of the reaction mechanism spectrum. The change in stereoselectivity between the benzoyl/benzyl donors can be explained as arising from the LRP of the C-3 acyl groups. This shift is most pronounced in the mannose series, where all glycosylations proceed to give solely the α-product (this is also observed in the glycosylation with the methyl/acetyl protected donor analog, Supplementary Table 1). These observations are in excellent agreement with the IRIS and the CEL maps results which indicate that C-1,C-3 dioxolenium ion formation is possible and most pronounced in the mannose system. For the glucose and galactose donors (Table 1b, entry 2 and 6), C-3 LRP provides less stabilization, which is reflected by the smaller impact on the α-selectivity in these cases.

In contrast, the stereoselectivity of the C-4 benzoate glucose and mannose donors is virtually identical to the selectivity of the C-4 benzyl glucose and mannose donors, revealing little influence of the group present at C-4 position. The IRIS experiments and CEL maps revealed that the formation of the mannosyl C-1,C-4 dioxolenium ion is not favorable. The C-4 acyl group in mannose thus has relatively little effect on the position of the mechanistic continuum at which the substitution reactions take place and LRP can be excluded. While IRIS and the CEL maps have shown that the formation of the glucosyl C-1,C-4 dioxolenium ion is favorable in the gas-phase, the glycosylation reactions of the glucosyl donor appear to be unaffected by the nature of the C-4 substituent. The solution-phase CEL maps have revealed the C-1,C-4 dioxolenium ion, and oxocarbenium ions to be of similar energy. This may account for the moderate effect of the C-4 acetyl group on the stereochemical outcome of the glucosylation reactions. In contrast to the C-4 acyl glucose and mannose series, the C-4 acyl group in the galactose donor is capable of LRP. Both IRIS and the CEL maps provide support for the formation of the bridged C-1,C-4 dioxolenium ion. The stability of this ion translates into the formation of more α-product in the glycosylations of the C-4 acyl galactosides.

Finally, the IRIS spectra of the C-6 acyl gluco-, manno-, and galactosyl donors provide no evidence for LRP of this functional group and also the CEL maps indicate that the formation of the dioxolenium ion bridging the C-1 and C-6 positions does not lead to significant stabilization of the ions. The matrix of glycosylation reactions indeed shows little influence of the C-6 acyl groups. Based on this combined dataset, LRP by C-6 acyl appears to have little influence on glycosylation reactions.

In conclusion, we report a systematic evaluation of LRP in glucosyl, mannosyl and galactosyl donors bearing an acyl protecting group at their C-3, C-4, or C-6 hydroxyl group functionality. A three-pronged approach consisting of IRIS, CEL computations, and glycosylation reactions was used to assess the effect of LRP in these glycosyl donors. These studies confirm that LRP can play a decisive role in shaping the stereochemical outcome of a glycosylation reaction. LRP plays a major role in glycosylations of C-3 acyl mannosides and to a somewhat lesser extent C-4 acyl galactosides. C-3 acyl groups in glucose and galactosyl donors can engage in LRP but this anchimeric assistance has relatively little influence on the stereochemical course of glycosylations of these donors. No important role for C-6 acyl LRP has been found. The strength of LRP thus follows the order: 3-Ac-Man » 4-Ac-Gal > 3-Ac-Glu ∼ 3-Ac-Gal > 4-Ac-Glu > 4-Ac-Man ∼ 6-Ac-Glc/Gal/Man. The establishment of dioxolenium ion intermediates as possible reactive intermediates in glycosylation reactions opens up avenues to enhance and exploit this effect to gain stereocontrol^14,46,47^. These are expected to accelerate the assembly of glycoconjugates to fuel biological research.

## Methods

### Ion spectroscopy in a modified ion trap mass spectrometer

The experimental apparatus is based on a modified 3D quadrupole ion trap mass spectrometer (Bruker, AmaZon Speed ETD) coupled to the beamline of the FELIX infrared free electron laser (IR-FEL)^37^. Glycosyl precursor cations ([M + NH_4_]^+^ or [M + H]^+^) were generated by ESI from 10^−6^ M solutions (in 50:50 acetonitrile:water) containing 2% ammonium acetate. An *m/z* of interest was isolated and collisionally activated for 40 ms in order to generate the relevant oxonium ions. Following the fragmentation reaction, the product ion *m/z* of interest was isolated and subsequently irradiated by the tunable mid-infrared beam. Following the absorption of a sufficient number of photons, typically occurring during a single macropulse, unimolecular dissociation results and is monitored as a frequency-dependent fragmentation (in this case, MS^3^) signal in the mass spectrum. Relating the precursor *m/z* intensity to the sum of all fragment *m/z* intensities in the mass spectrum recorded at each frequency position generates an infrared vibrational spectrum. IR spectra were linearly corrected for frequency-dependent variation of the laser pulse energy and the frequency was calibrated using a grating spectrometer.

### Simulation of IR spectra

Chemical structures of the oxocarbenium, dioxolenium and rearranged ions were defined using SMILES notation for input to our workflow using the cheminformatics toolbox RDKit^48^. A conformational search was performed using a distance geometry algorithm, yielding 500 random 3D-conformations, which were minimized using a classical forcefield^49^. A maximum of 40 conformations was selected based on root-mean-squared deviation of atomic positions between geometries. Selected conformations were then submitted to Gaussian 16^50^ for geometry optimization and vibrational analysis at a semi-empirical PM6 level. Based on the comparison of relative energies, structures were filtered using an energy cut-off of 40 kJ mol^−1^ (for pyranosyl cations, the cut-off was increased to 80 kJ mol^−1^). Remaining structures were reoptimized at the B3LYP/6-31 + +G(d,p) level of theory and thereafter a harmonic frequency calculation was performed (frequencies were scaled by 0.975). In order to facilitate comparison to the experimental spectra, a 20 cm^−1^ Gaussian broadening function was applied to the calculated vibrational lines. Finally, for each structure, electronic energies (based on the B3LYP optimized geometries) were calculated using second-order Møller–Plesset perturbation theory (MP2) and combined with enthalpy and entropy contributions resulting from the B3LYP vibrational analysis to give Gibbs free energies (*T* = 298 K).

### CEL calculations

A complete survey of the possible conformational space of the glycosyl cation was done by scanning three dihedral angles ranging from −60° to 60°, including the C1–C2–C3–C4, C3–C4–C5–O, and C5–O–C1–C2^29^. The resolution of this survey is determined by the step size which was set to 15° per puckering parameter, giving a total of 729 prefixed conformations per glycosyl cation spanning the entire conformational landscape. All other internal coordinates were unconstrained. The 729 structures were computed with Gaussian 09 Rev. D.01^51^ with DFT/B3LYP/6-311G(d,p). Furthermore, where applicable solvation effects of CH_2_Cl_2_ were taken into account with a polarizable continuum model. For this specific study, two rotamers were taken into account of the C-O bond rotamer of the ring carbon and the oxygen of one of the substituents which is protected by an acyl protecting group. The CEL maps were separately computed and visualized. The final denoted energy is expressed as free Gibbs energy. The thermodynamic corrections were computed using the quasi-harmonic approximation in the gas-phase^52,53^. All optimized structures were checked for the absence of imaginary frequencies. To visualize the energy levels of the conformers on the Cremer–Pople sphere, we have generated slices dissecting the sphere that combine closely associated conformers. The OriginPro software was employed to produce the energy heat maps, contoured at 0.5 kcal/mol^42^.

### Pre-activation Tf_2_O/Ph_2_SO-based *O*-glycosylation experiments

A solution of the donor (100 μmol), Ph_2_SO (26 mg, 130 μmol, 1.3 eq.) and TTBP (62 mg, 250 μmol, 2.5 eq.) in DCM (2 mL, 0.05 M) was stirred over activated 3 Å molecular sieves (rods, size 1/16 in., Sigma-Aldrich) for 30 min under an atmosphere of N_2_. The solution was cooled to −80 °C and Tf_2_O (22 μl, 130 μmol, 1.3 eq.) was slowly added to the reaction mixture. The reaction mixture was allowed to warm to −60 °C in approximately 45 min, followed by cooling to −80 °C and the addition of the acceptor (200 μmol, 2 eq.) in DCM (0.4 mL, 0.5 M). The reaction was allowed to warm up to −60 °C and stirred for an additional 4–18 h at this temperature until full reaction completion was observed. The reaction was quenched with sat. aq. NaHCO_3_ at −60 °C and diluted with DCM (5 mL). The resulting mixture was washed with H_2_O and brine, dried over MgSO_4_, filtered and concentrated under reduced pressure. Purification by column chromatography yielded the corresponding *O-*coupled glycoside.

## Supplementary information

Supplementary Information

Description of Additional Supplementary Files

Supplementary Data 1

## Data Availability

The authors declare that the data supporting the findings of this study are available within the paper and its supplementary method and data files.
