# Supplementary material for: Characterization of glycosyl dioxolenium ions and their role in glycosylation reactions
Source: Nat Commun. 2020 May 29;11:2664. doi: 10.1038/s41467-020-16362-x (PMC7260182; doi:10.1038/s41467-020-16362-x)
Supplement: Supplementary file 1 — Supplementary Information [file 41467_2020_16362_MOESM1_ESM.pdf]

Supplementary Information

"Characterization of Glycosyl Dioxolenium Ions and Their Role in Glycosylation Reactions"

Hansen *et al.*

Contents

Supplementary Methods *3*

Tandem-MS combined with IR ion spectroscopy **3**

General procedure I: Ion spectroscopy in a modified ion trap mass spectrometer 3
General procedure II: Simulation of IR spectra 3
CID- and IR-spectra 4

DFT calculations **16**

General procedure III: conformational energy landscape calculation of pyranosyl oxocarbenium ions 16
Multi-substituted pyranosyl cations 20
Gas-phase CEL maps 29

Organic synthesis **30**

General experimental procedures 30
General procedure IV: acetylation procedure 30
General procedure V: Methylation procedure 30
General procedure VI: *S*-oxidation procedure 31
General procedure VII: pre-activation Tf_2O/Ph_2SO based *O*-glycosylation 31
Preparation of the donors for the model glycosylation reactions 32
Model glycosylation reactions 51

NMR spectra of new and selected compounds **83**

Supplementary References *269*

Supplementary Methods

Tandem-MS combined with IR ion spectroscopy

General procedure I: Ion spectroscopy in a modified ion trap mass spectrometer

The experimental apparatus is based on a modified 3D quadrupole ion trap mass spectrometer (Bruker, AmaZon Speed ETD) that has been coupled to the beam line of the FELIX infrared free electron laser (IR-FEL).[1] Ammonium adducts of each thioether compound ($[M+NH_4]^+$) or protonated adducts from each thiosulfinyl compound ($[M+H]^+$) were generated by electrospray ionization from solutions of 10^{-6} M (in 50:50 acetonitrile:water) containing 2% ammonium acetate infused at 2 µl min^{-1}. The mass-isolated ions of interest were collisionally activated for 40 ms in order to generate the relevant oxonium products. These fragment ions were subsequently mass isolated and irradiated by the tunable mid-infrared beam. The FEL was operated to provide 5 µs optical pulses at 10 Hz having 60-120 mJ pulse energy over the entire IR frequency range (bandwidth ~0.4% of the center frequency). The actual pulse energy used for measurements was appropriately attenuated in order to avoid saturation of the signal. When a sufficient number of photons is absorbed, typically during a single macropulse, unimolecular dissociation occurs generating frequency-dependent fragment ion intensities in the mass spectrum. Relating the precursor ion intensity to the total fragmentation intensity in the observed mass spectrum (yield=ΣI(fragment ions)/ΣI(parent+fragment ions)) for each frequency position generates an infrared vibrational spectrum. The yield is obtained from several averaged mass spectra and is linearly corrected for laser power; the IR frequency is calibrated using a grating spectrometer. A frequency step size of 3 cm^{-1} was used in all spectra reported here.

General procedure II: Simulation of IR spectra

For the calculation of gas-phase geometries and corresponding IR spectra we have used a workflow that has been reported previously.[2] The SMILES structure format of the oxocarbenium, dioxolenium and rearranged ions were used as input for the workflow using the cheminformatics toolbox RDKit.[3] A conformational search was performed using a distance geometry algorithm, yielding 500 random 3D-conformations, which were minimized using a classical forcefield.[4] A maximum of 40 conformations were selected by hierarchical on the root means squared distance between geometries.[5] These selected conformations were then submitted to Gaussian 16 for geometry optimization and frequency calculations using the semi-empirical PM6 level.[6] By comparing relative energies (electronic and thermal), unfavorable conformations were filtered by using an energy cut-off of 40 kJ/mol. When generating pyranosyl cations, this cut-off was increased to 80 kJ/mol, as the oxocarbenium ions would otherwise be filtered out because dioxolenium ions that were formed in the optimization were much lower in energy. Additionally, similar geometries were filtered based on (close to) identical calculated frequencies and corresponding intensities. After these filtering steps, the remaining structures were reoptimized using the B3LYP density functional and 6-31++G(d,p) basis set and thereafter a frequency calculation was performed. Harmonic vibrational frequencies were scaled by 0.975. To aid comparison to experimental spectra, Gaussian broadening (20 cm^{-1} at full width half maximum) was applied to the calculated vibrational lines. To obtain reliable energies the thermal energy of the frequency calculation was combined with the electronic energies calculated using second order Møller-Plesset perturbation theory and the 6-31++G(d,p) basis set.

CID- and IR-spectra

Ac/Me-*O*-protected glycosyl donors

3-*O*-Acetyl-2,4,6-tri-*O*-methyl-gluco-D-pyranosyl cation

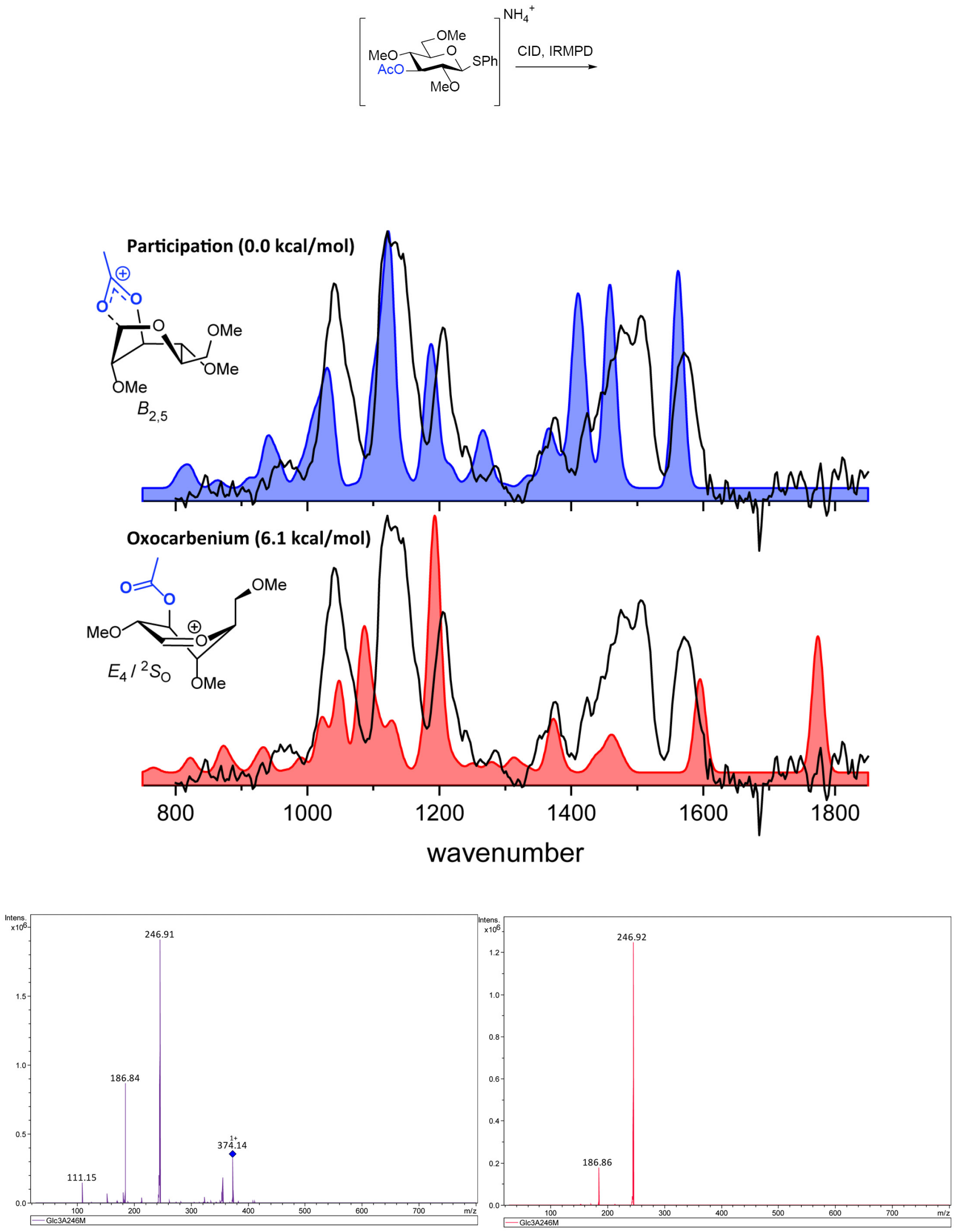

Supplementary Figure 1. Comparison of the computed IR-spectra of low energy structures (filled) with the measured IR-ion spectrum (black line) of the m/z = 247 CID fragment of compound **1** (top structure). The computed IR-spectrum in blue was assigned as best match with the measured spectrum. Fragment m/z = 247 (bottom right) was generated by CID of the parent ion ammonium adduct (bottom left).

4-*O*-Acetyl-2,3,6-tri-*O*-methyl-gluco-D-pyranosyl cation

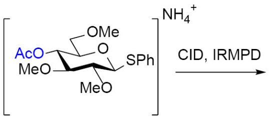

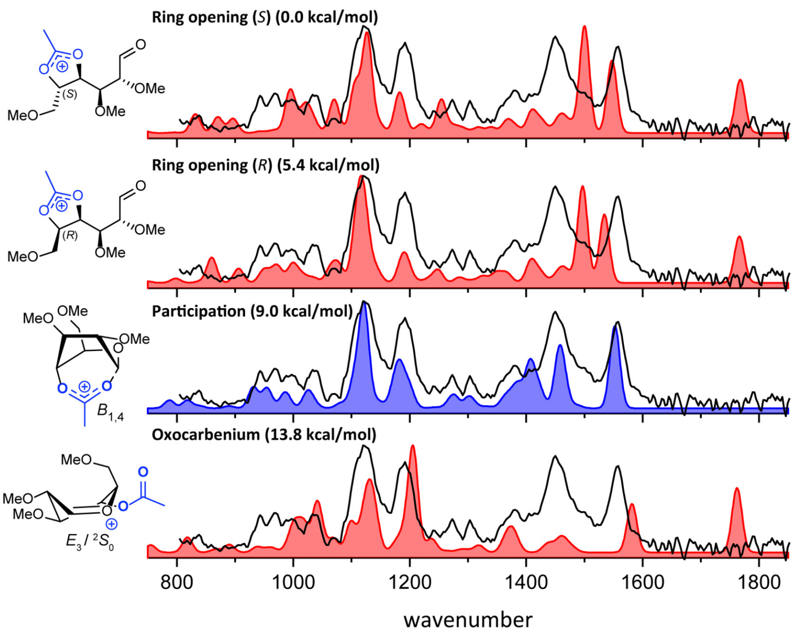

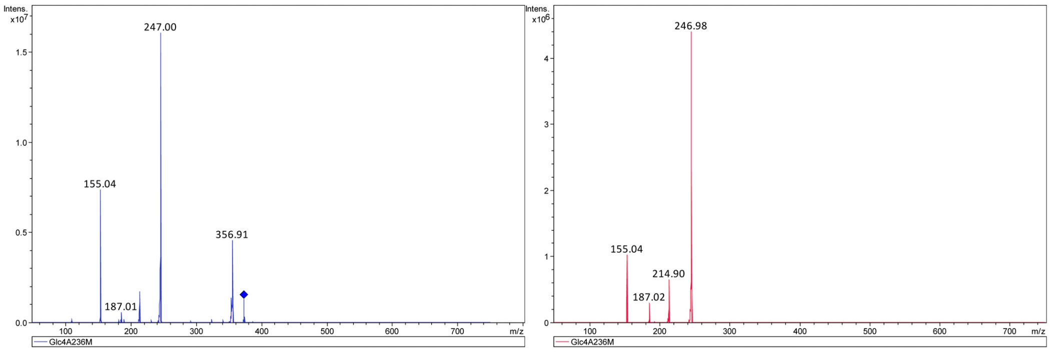

Supplementary Figure 2. Comparison of the computed IR-spectra of low energy structures (filled) with the measured IR-ion spectrum (black line) of the m/z = 247 CID fragment of compound **2** (top structure). The computed IR-spectrum in blue was assigned as best match with the measured spectrum. Fragment m/z = 247 (bottom right) was generated by CID of the parent ion ammonium adduct (bottom left).

6-*O*-Acetyl-2,3,4-tri-*O*-methyl-gluco-D-pyranosyl cation

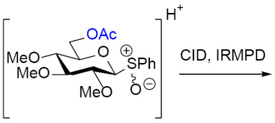

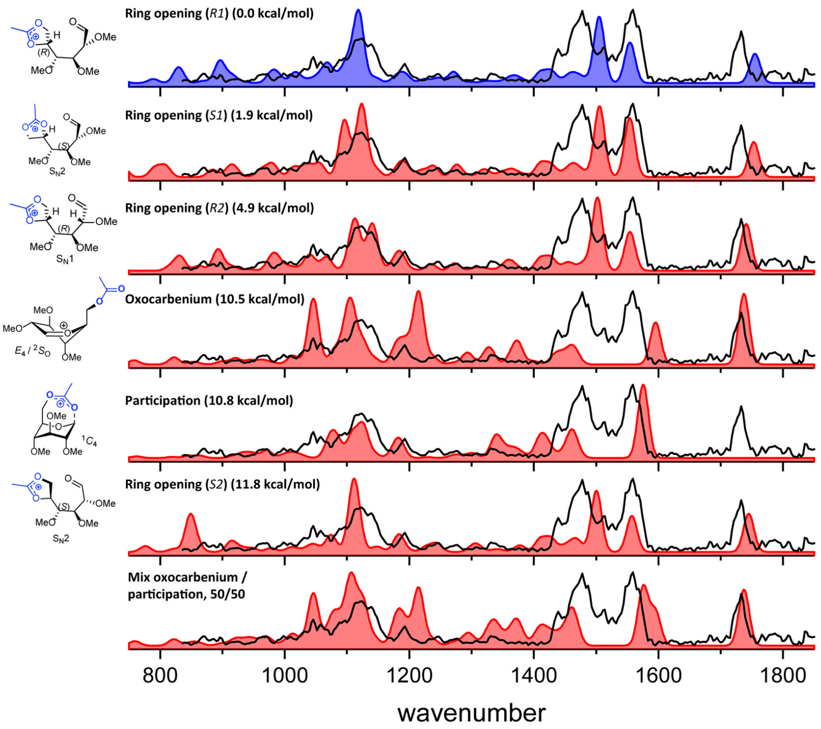

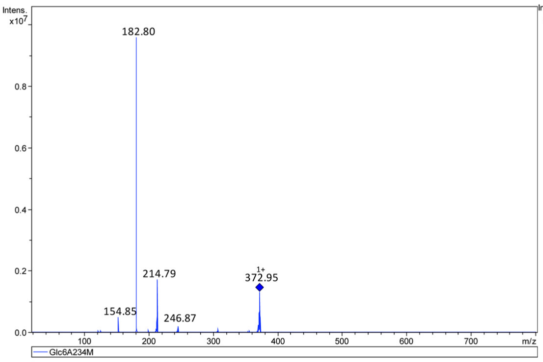

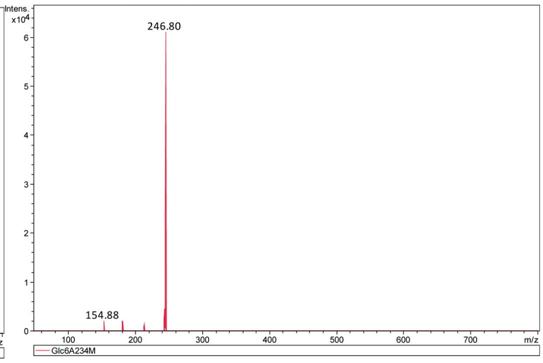

Supplementary Figure 3. Comparison of the computed IR-spectra of low energy structures (filled) with the measured IR-ion spectrum (black line) of the m/z = 247 CID fragment of compound **3** (top structure). The computed IR-spectrum in blue was assigned as best match with the measured spectrum. Fragment m/z = 247 (bottom right) was generated by CID of the parent ion proton adduct (bottom left).

3-*O*-Acetyl-2,4,6-tri-*O*-methyl-manno-D-pyranosyl cation

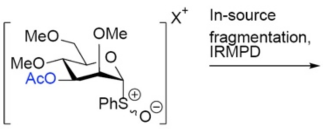

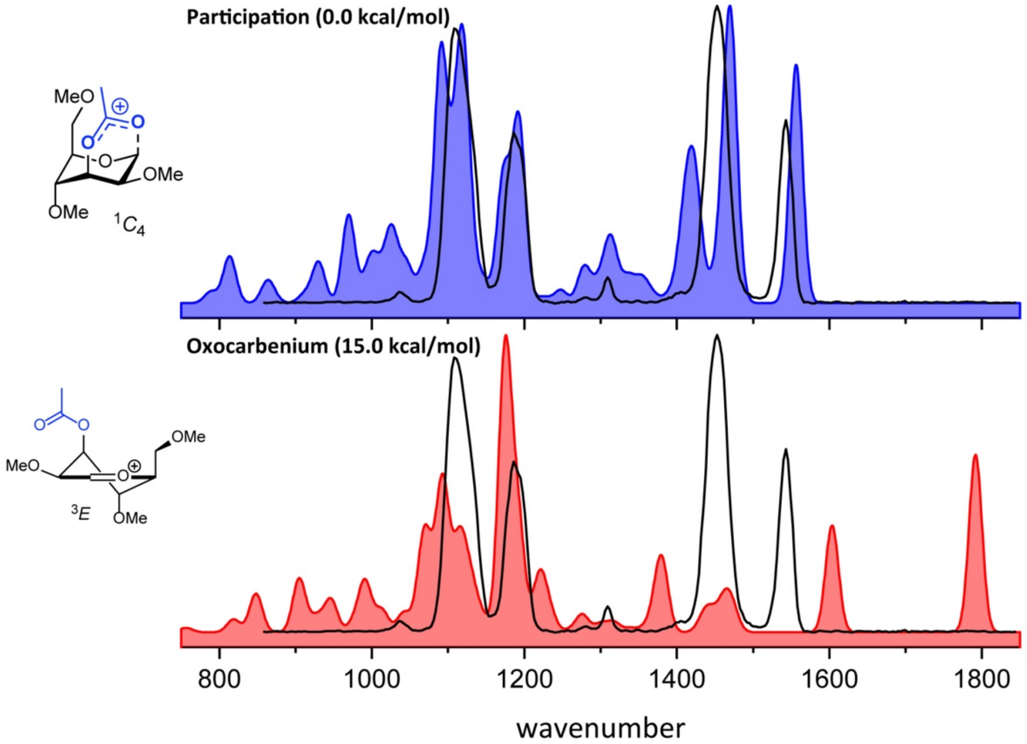

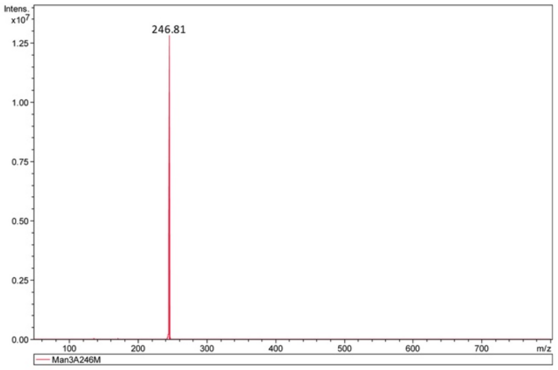

Supplementary Figure 4. Comparison of the computed IR-spectra of low energy structures (filled) with the measured IR-ion spectrum (black line) of the m/z = 247 CID fragment of compound **4** (top structure). The computed IR-spectrum in blue was assigned as best match with the measured spectrum. Fragment m/z = 247 (bottom right) was generated in source (bottom figure).

4-*O*-Acetyl-2,3,6-tri-*O*-methyl-manno-D-pyranosyl cation

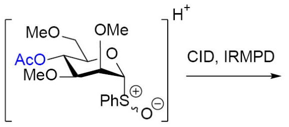

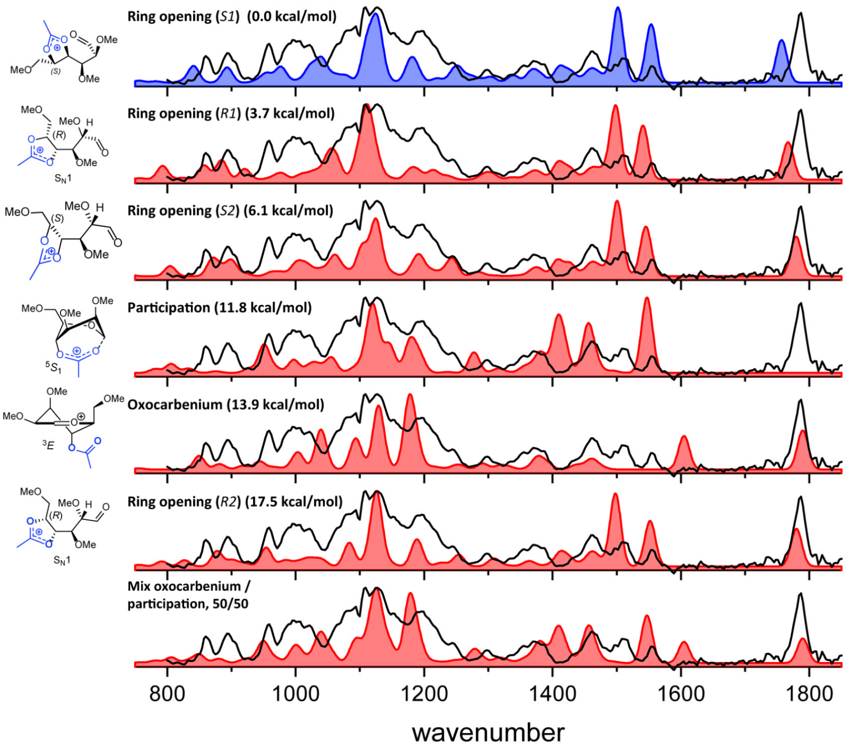

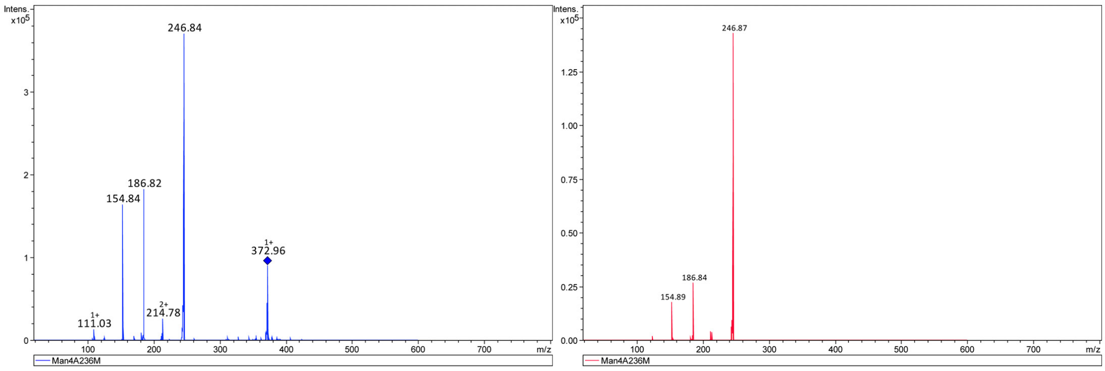

Supplementary Figure 5. Comparison of the computed IR-spectra of low energy structures (filled) with the measured IR-ion spectrum (black line) of the m/z = 247 CID fragment of compound **5** (top structure). The computed IR-spectrum in blue was assigned as best match with the measured spectrum. Fragment m/z = 247 (bottom right) was generated by CID of the parent ion proton adduct (bottom left).

6-*O*-Acetyl-2,3,4-tri-*O*-methyl-manno-D-pyranosyl cation

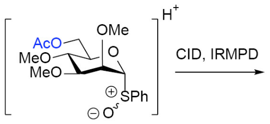

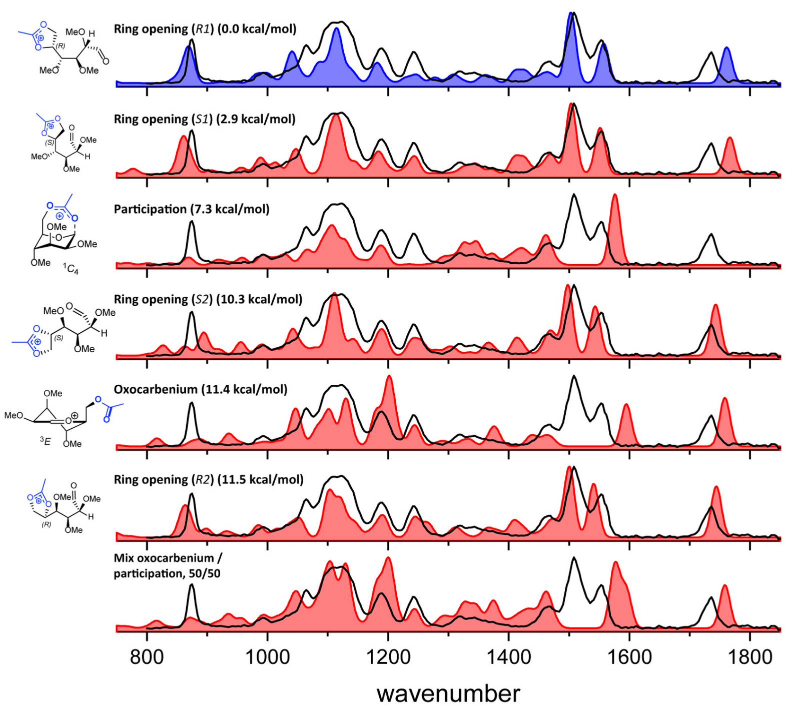

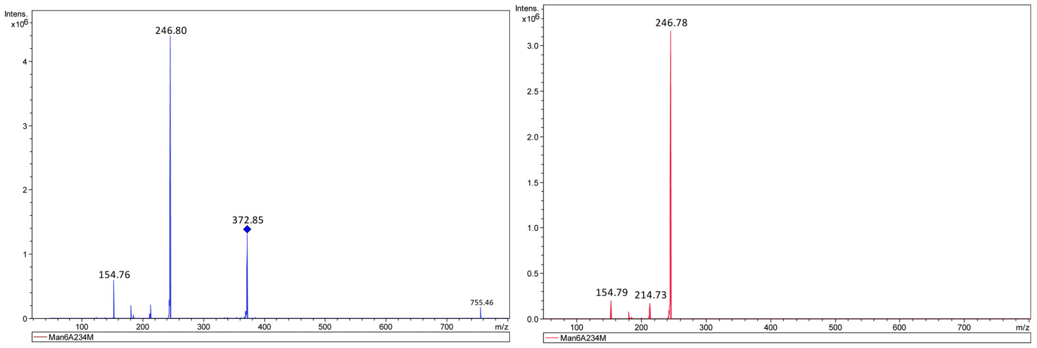

Supplementary Figure 6. Comparison of the computed IR-spectra of low energy structures (filled) with the measured IR-ion spectrum (black line) of the m/z = 247 CID fragment of compound **6** (top structure). The computed IR-spectrum in blue was assigned as best match with the measured spectrum. Fragment m/z = 247 (bottom right) was generated by CID of the parent ion proton adduct (bottom left).

3-*O*-Acetyl-2,4,6-tri-*O*-methyl-galacto-D-pyranosyl cation

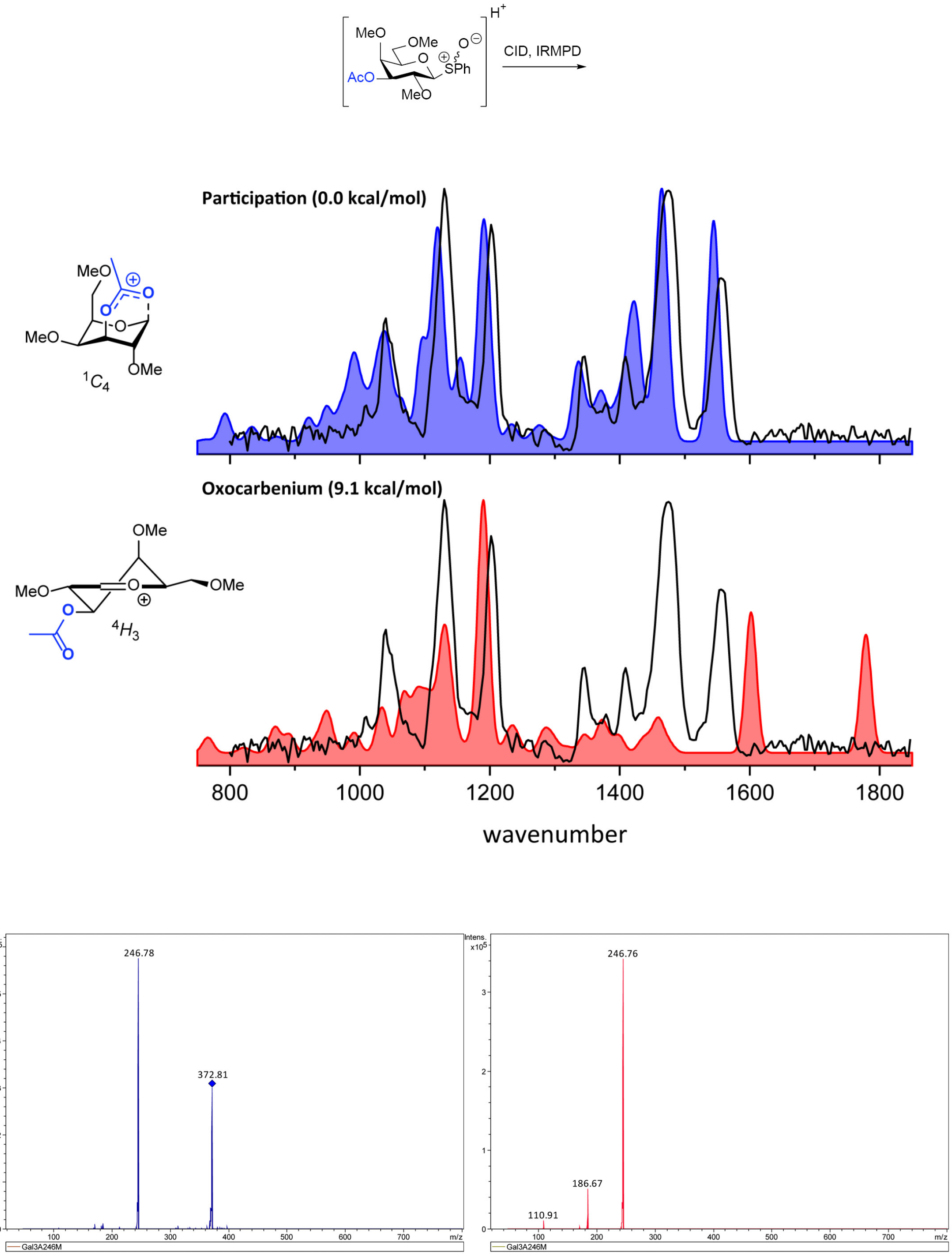

Supplementary Figure 7. Comparison of the computed IR-spectra of low energy structures (filled) with the measured IR-ion spectrum (black line) of the m/z = 247 CID fragment of compound **7** (top structure). The computed IR-spectrum in blue was assigned as best match with the measured spectrum. Fragment m/z = 247 (bottom right) was generated by CID of the parent ion proton adduct (bottom left).

4-*O*-Acetyl-2,3,6-tri-*O*-methyl-galacto-D-pyranosyl cation

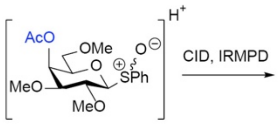

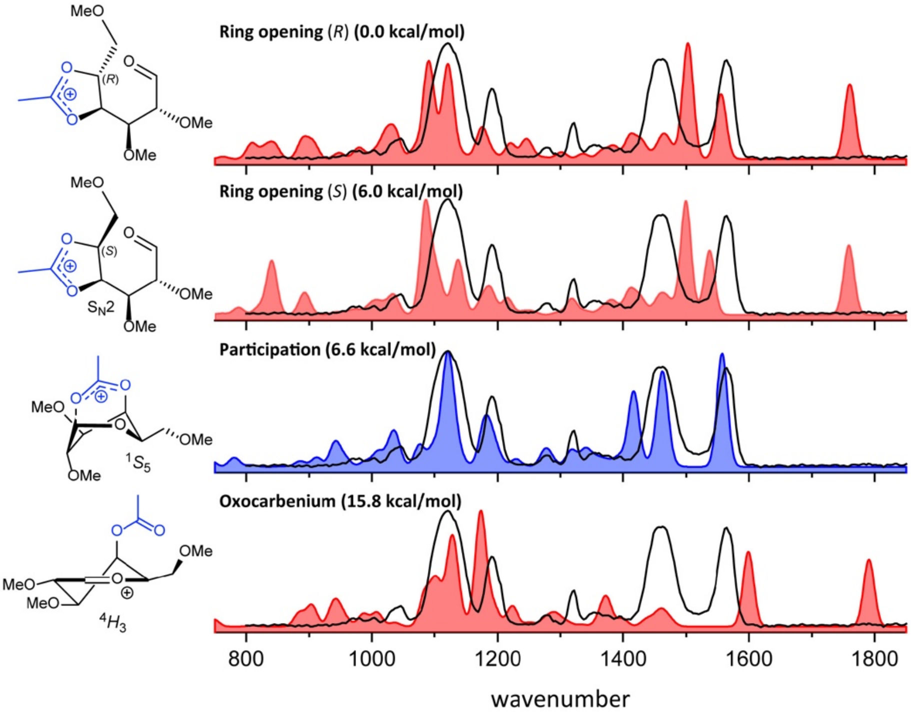

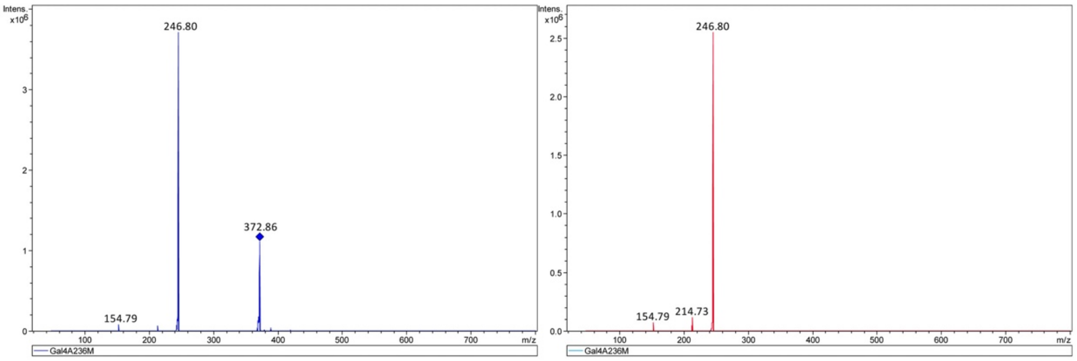

Supplementary Figure 8. Comparison of the computed IR-spectra of low energy structures (filled) with the measured IR-ion spectrum (black line) of the m/z = 247 CID fragment of compound **8** (top structure). The computed IR-spectrum in blue was assigned as best match with the measured spectrum. Fragment m/z = 247 (bottom right) was generated by CID of the parent ion proton adduct (bottom left).

6-*O*-Acetyl-2,3,4-tri-*O*-methyl-galacto-D-pyranosyl cation

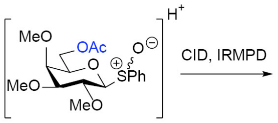

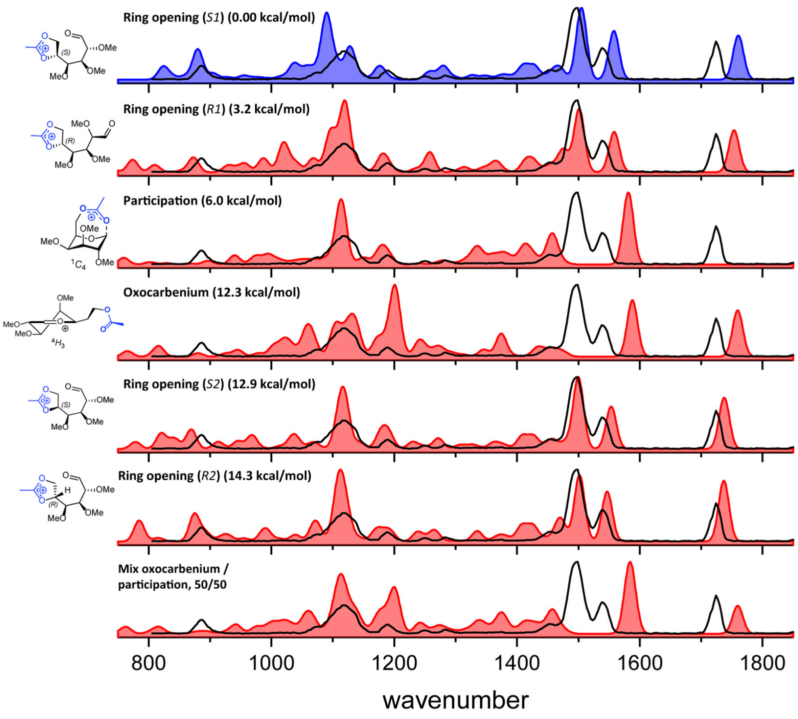

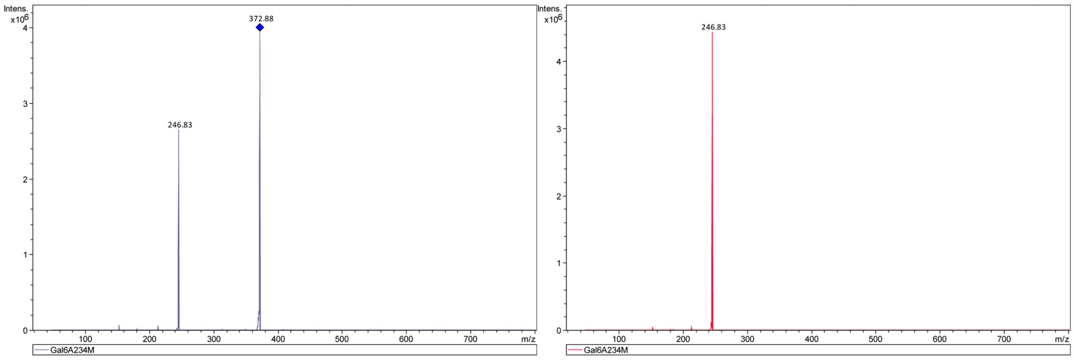

Supplementary Figure 9. Comparison of the computed IR-spectra of low energy structures (filled) with the measured IR-ion spectrum (black line) of the m/z = 247 CID fragment of compound **9** (top structure). The computed IR-spectrum in blue was assigned as best match with the measured spectrum. Fragment m/z = 247 (bottom right) was generated by CID of the parent ion proton adduct (bottom left).

Bz/Bn-*O*-protected glycosyl donors

3-*O*-Benzoyl-2,4,6-tri-*O*-benzyl-manno-D-pyranosyl cation

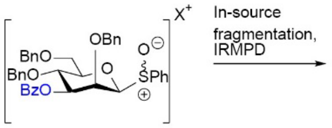

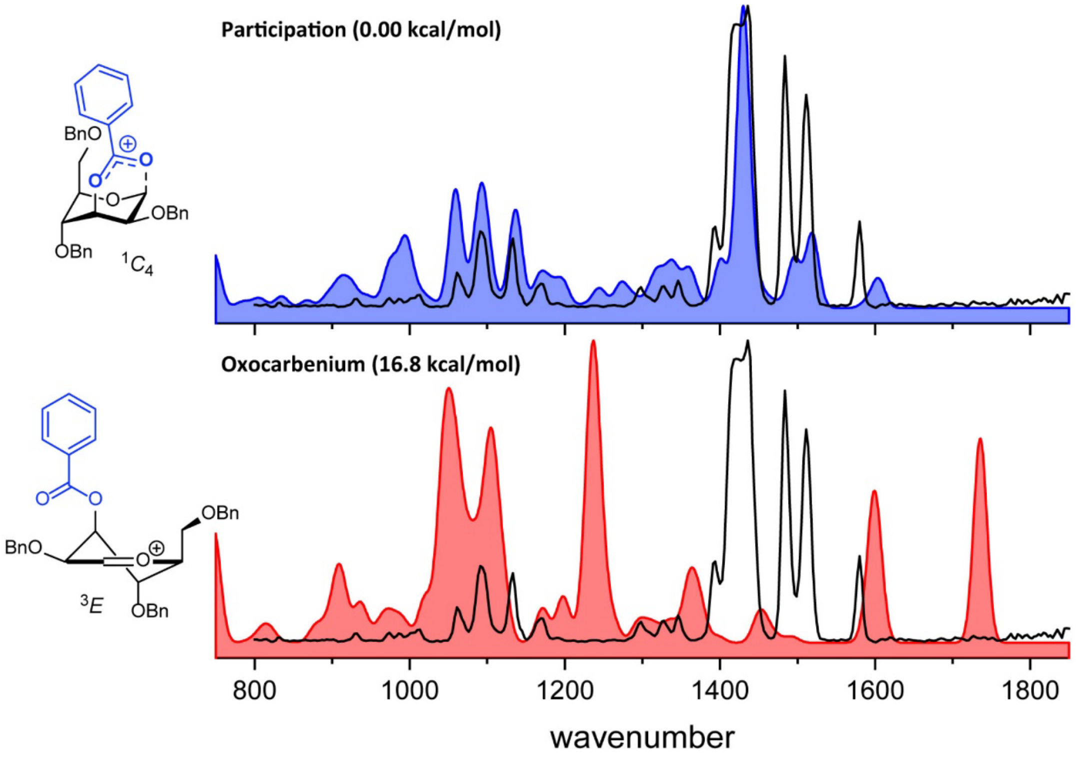

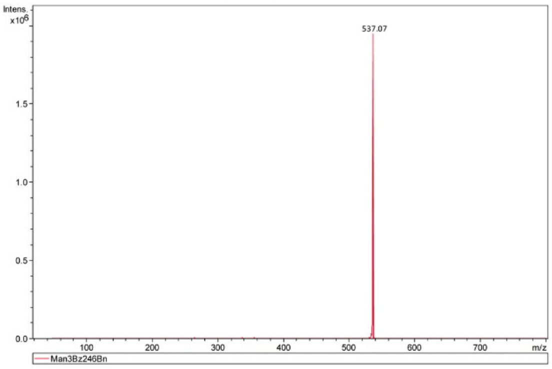

Supplementary Figure 10. Comparison of the computed IR-spectra of low energy structures (filled) with the measured IR-ion spectrum (black line) of the m/z = 537 CID fragment of compound **13** (top structure). The computed IR-spectrum in blue was assigned as best match with the measured spectrum. Fragment m/z = 537 (bottom left) was generated by in source fragmentation of the parent ion.

4-*O*-Benzoyl-2,3,6-tri-*O*-benzyl-manno-D-pyranosyl cation

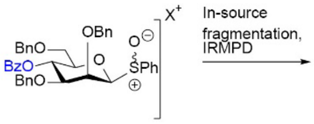

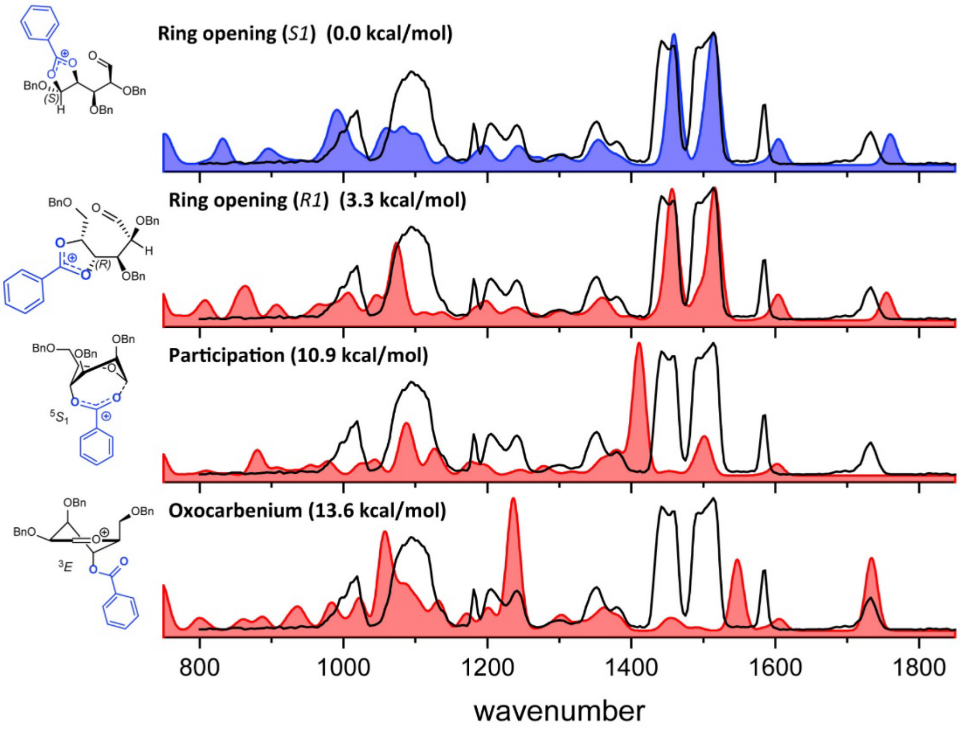

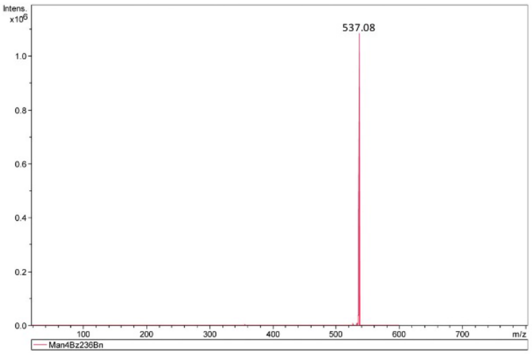

Supplementary Figure 11. Comparison of the computed IR-spectra of low energy structures (filled) with the measured IR-ion spectrum (black line) of the m/z = 537 CID fragment of compound **14** (top structure). The computed IR-spectrum in blue was assigned as best match with the measured spectrum. Fragment m/z = 537 (bottom left) was generated by in source fragmentation of the parent ion.

6-*O*-Benzoyl-2,3,4-tri-*O*-benzyl-manno-D-pyranosyl cation

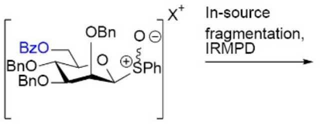

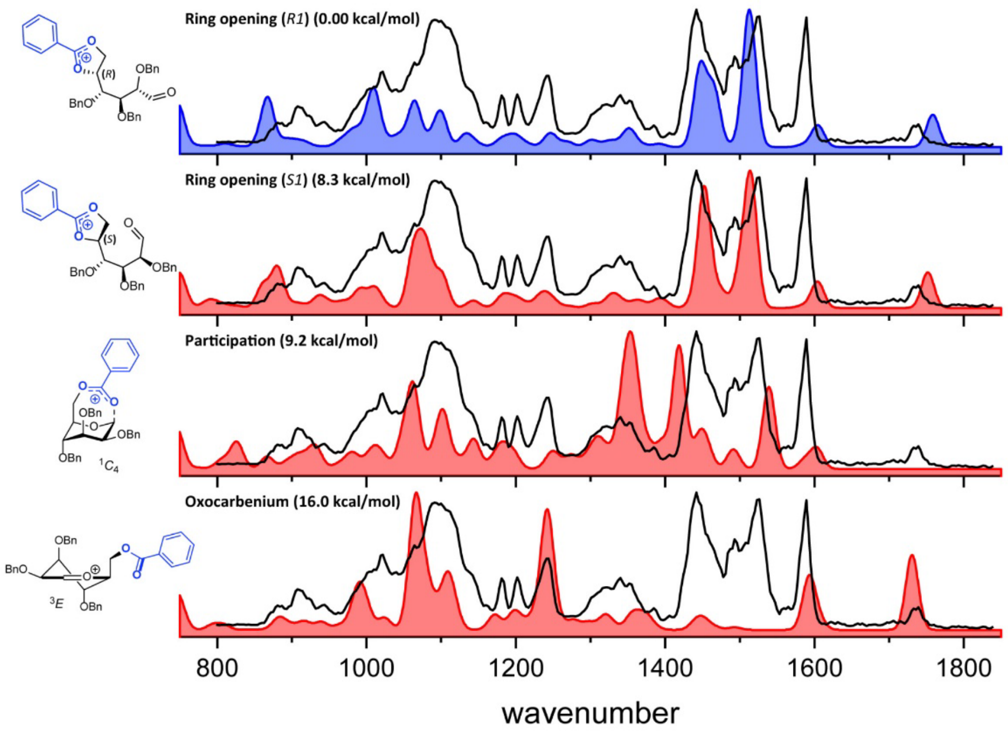

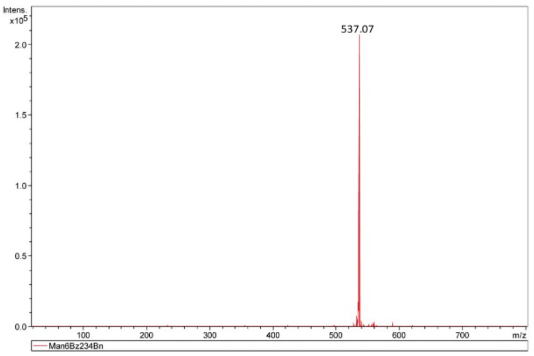

Supplementary Figure 12. Comparison of the computed IR-spectra of low energy structures (filled) with the measured IR-ion spectrum (black line) of the m/z = 537 CID fragment of compound **15** (top structure). The computed IR-spectrum in blue was assigned as best match with the measured spectrum. Fragment m/z = 537 (bottom left) was generated by in source fragmentation of the parent ion.

DFT calculations

General procedure III: conformational energy landscape calculation of pyranosyl oxocarbenium ions

To keep the calculation time manageable, large protection groups (OBn) were substituted with electronic comparable smaller groups (OMe). The initial structure for the conformational energy landscape (CEL) mapping of the six-membered glycosyl cation was optimised by starting from a 'conformer distribution search' option included in the Spartan 10 program by utilising DFT as the level of theory and B3LYP as hybrid functional in gas phase with 6-31G(d) as the basis set. All generated gas-phase geometries were re-optimized with Gaussian 09 Rev. D.01 by using B3LYP/6-311G(d,p), after which a vibrational analysis was computed to obtain the thermodynamic properties. The gas-phase structures were then solvated by using the PCM implicit solvation model, with CH_2Cl_2 as solvent. Solvent effects were explicitly used in solving the SCF equations and during the optimization of the geometry. The geometry with the lowest energy was selected as the starting point for the CEL. A complete survey of the possible conformational space was done by scanning three dihedral angles ranging from –60° to 60°, including the C1-C2-C3-C4 (D1), C3-C4-C5-O (D3) and C5-O-C1-C2 (D5). The resolution of this survey is determined by the step size which was set to 15° per puckering parameter, giving a total of 729 pre-fixed conformations per glycosyl cation spanning the entire conformational landscape. All other internal coordinates were unconstrained. Except when a C2-substituent was present on the oxocarbenium ring of interest, then the C2-H2 bond length was fixed based on the optimised structure to counteract rearrangements occurring for higher energy conformers. The 729 structures were computed with Gaussian 09 Rev. D.01 again with a two-step procedure. First, the structures were optimized in the gas-phase with B3LYP/6-311G(d,p), after which a vibrational analysis was computed to obtain the thermodynamic properties. The gas-phase structures were then solvated by using the PCM implicit solvation model, with CH_2Cl_2 as solvent. For glycosyl cation bearing a C5-C6 substituent three separate staggered rotamers (*gg*, *gt*, *tg*) of the O5-C5-C6-O6 dihedral angle (-65°, 65°, 175°) were considered. Earlier work showed the importance of these rotamers and their crucial impact on the selectivity and reactivity of the ion.[7–10] The CEL maps were computed separately and the starting geometry was obtained from the method described above in which the lowest energy generated rotamers were used. For this specific study two extra rotamers were taken into account of the C-O bond rotamer of the ring carbon and the oxygen of one of the substituents which is protected by an acyl protecting group, bringing the total conformations for each glycosyl cation ion configuration to 4374 geometries. CEL maps were separately computed and visualized: rotamer 1 (R1) in which the acetyl is positioned in such a way that dioxolenium ion formation is geometrically feasible and rotamer 2 (R2) in which the acetyl points away and the free oxocarbenium ion can be found. The final denoted free Gibbs energy was calculated using Equation (1) in which ΔE_{gas} is the gas-phase energy (electronic energy), $\Delta G^{T}_{gas,QH}$ (T = reaction temperature, p = 1 atm. and C = 1 M) is the sum of corrections from the electronic energy to free Gibbs energy in the quasi-harmonic oscillator approximation also including ZPE, and ΔG_{solv} is their corresponding free solvation Gibbs energy. The $\Delta G^{T}_{gas,QH}$ were computed using the quasi-harmonic approximation in the gas phase according to the work of Truhlar.[11]

$$\Delta G^{T}_{\text{in solution}} = \Delta E_{gas} + \Delta G^{T}_{gas,QH} + \Delta G_{solv} \qquad \text{(Supplementary Equation 1)}$$

$$= \Delta G^{T}_{gas} + \Delta G_{solv}$$

The quasi-harmonic approximation is the same as the harmonic oscillator approximation except that vibrational frequencies lower than 100 cm^{-1} were raised to 100 cm^{-1} as a way to correct for the breakdown of the harmonic oscillator model for the free energies of low-frequency vibrational modes.

All optimized structures were checked for the absence of imaginary frequencies. To visualise the energy levels of the conformers on the Cremer-Pople sphere, we have generated slices dissecting the sphere that combine closely associated conformers. The OriginPro software was employed to produce the energy heat maps, contoured at 0.5 kcal/mol.[12] For ease of visualisation, the Cremer-Pople globe is turned 180° with respect to its common representation.

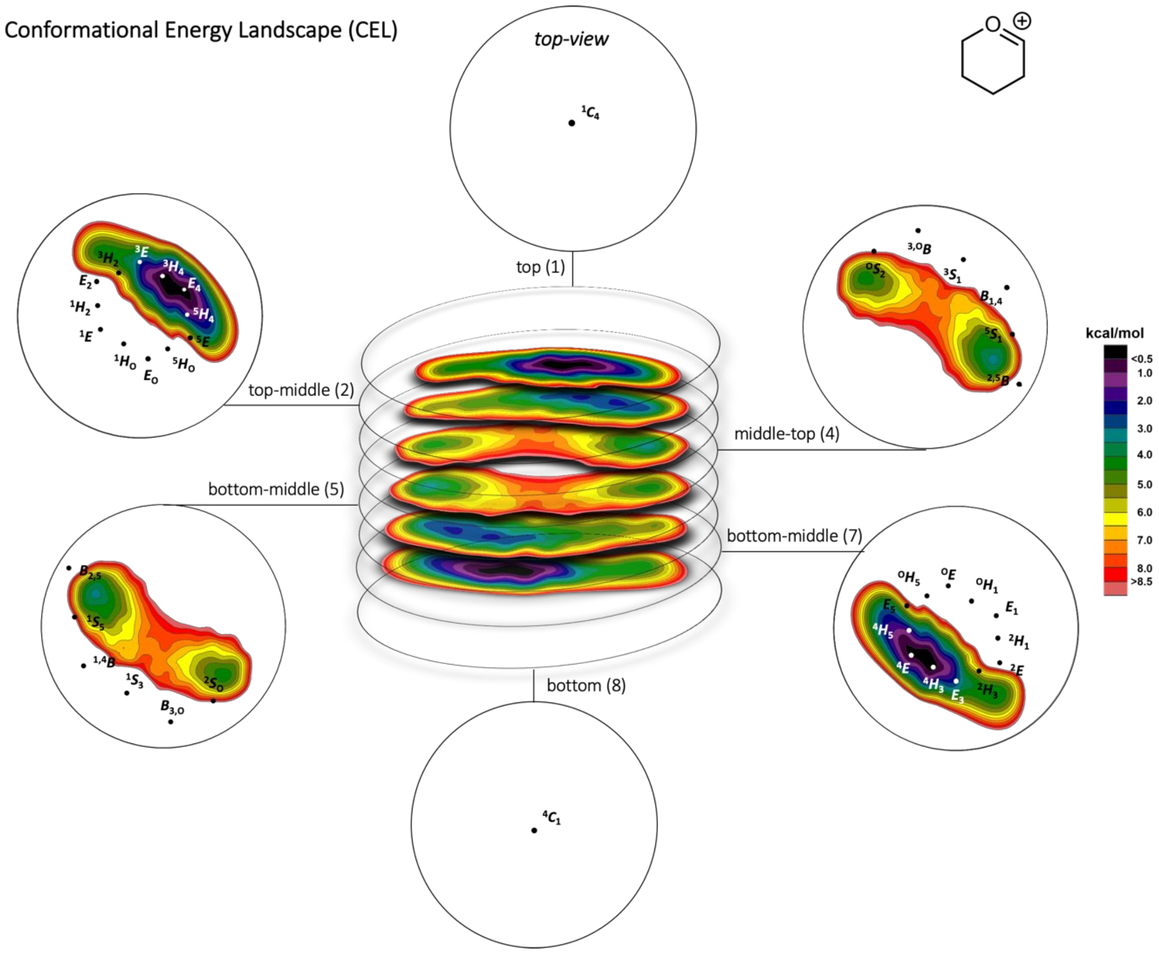

Supplementary Figure 13. "Deconvolution" of the CEL map of the pyranosyl oxocarbenium ion showing a top view of the most important slices that have been combined to generate the complete CEL map.[12]

Mono-substituted pyranosyl cations

Ac/Me-*O*-substituted pyranosyl cations

3-Acetyloxy-pyranosyl cation

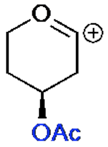

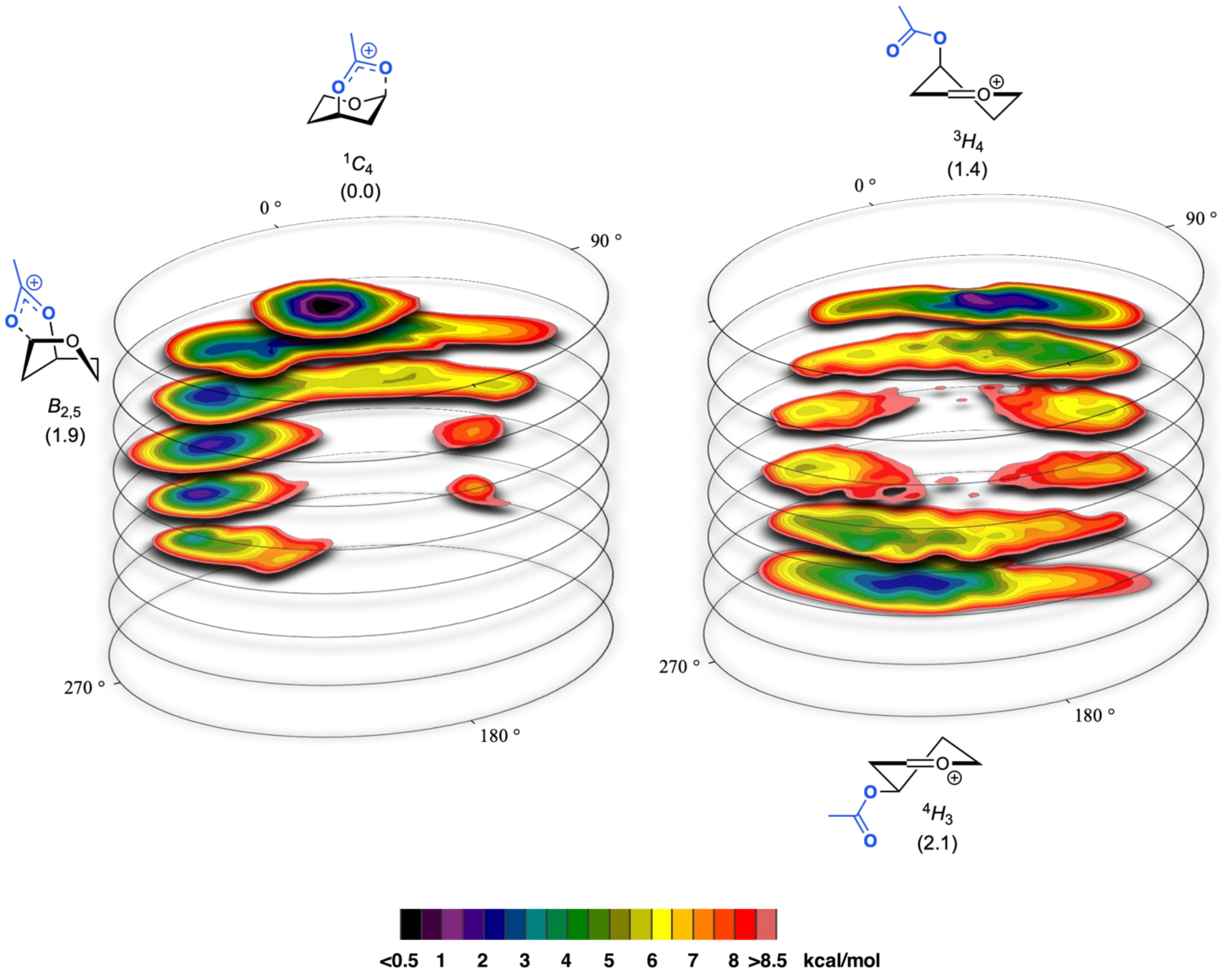

Supplementary Figure 14. CEL map of 3-acetyloxy-pyranosyl cation. All energies are computed at PCM(CH_2Cl_2)-B3LYP/6-311G(d,p) at *T*=213.15 K and expressed as solution-phase Gibbs free energy.[12]

4-Acetyloxy-pyranosyl cation

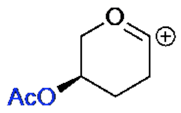

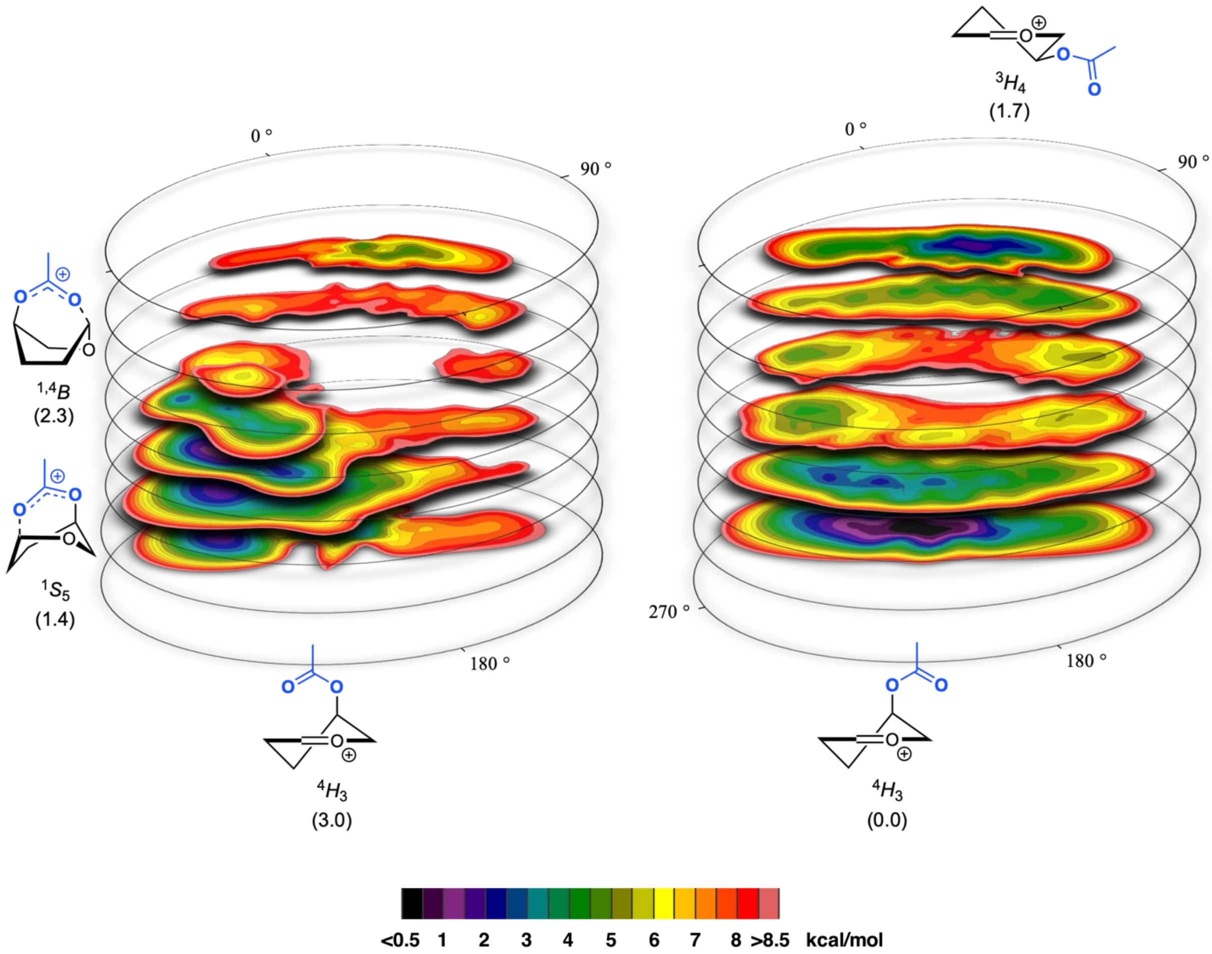

Supplementary Figure 15. CEL map of 4-acetyloxy-pyranosyl cation. All energies are computed at PCM(CH_2Cl_2)-B3LYP/6-311G(d,p) at T=213.15 K and expressed as solution-phase Gibbs free energy.[12]

Multi-substituted pyranosyl cations

Ac/Me-*O*-substituted cations

3-*O*-Acetyl-2,4,6-tri-*O*-methyl-gluco-D-pyranosyl cation

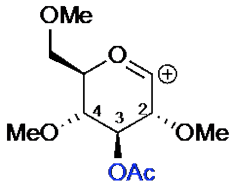

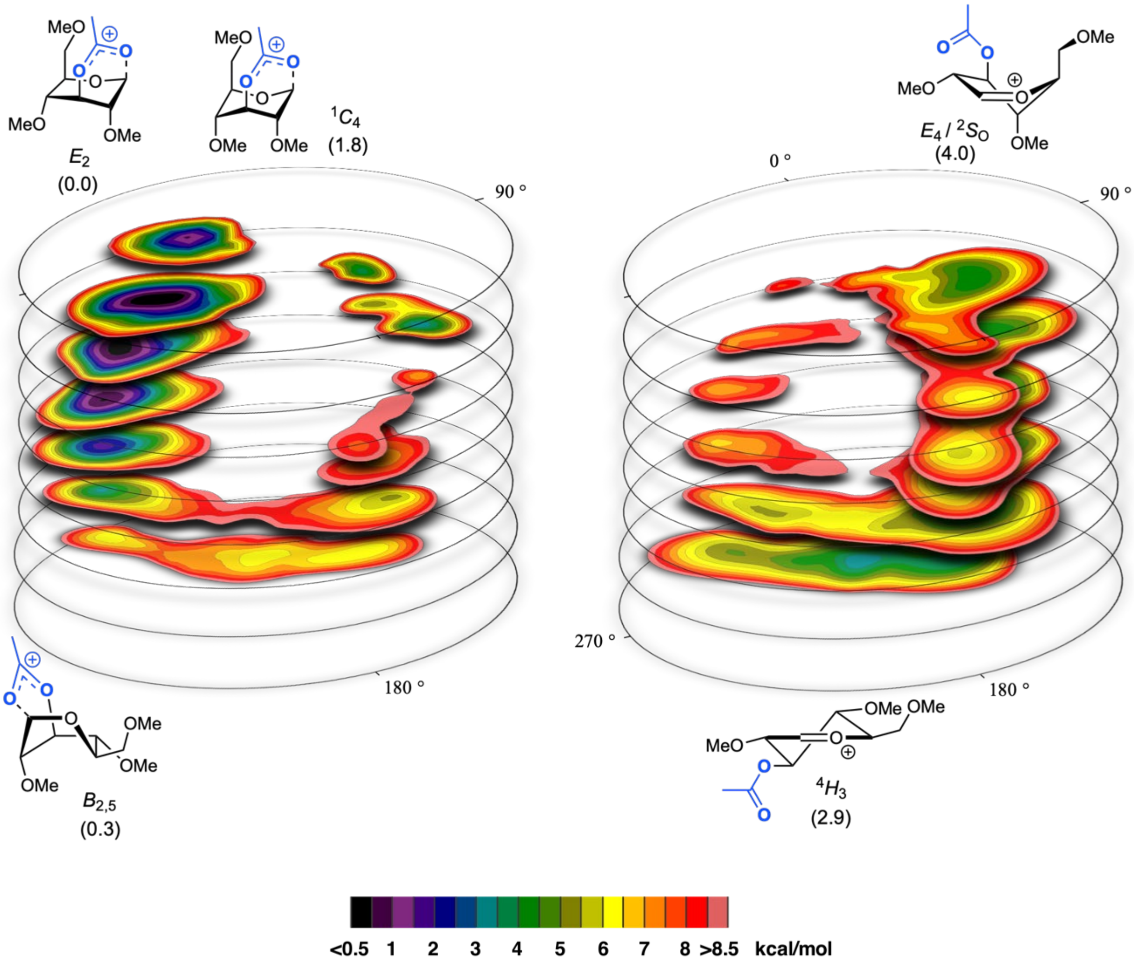

Supplementary Figure 16. CEL map of 3-*O*-acetyl-2,4,6-tri-*O*-methyl-gluco-D-pyranosyl cation. All energies are computed at PCM(CH_2Cl_2)-B3LYP/6-311G(d,p) at T=213.15 K and expressed as solution-phase Gibbs free energy.[12]

3-*O*-Acetyl-2,4,6-tri-*O*-methyl-manno-D-pyranosyl cation

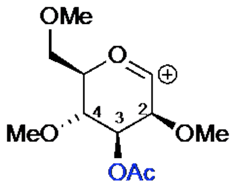

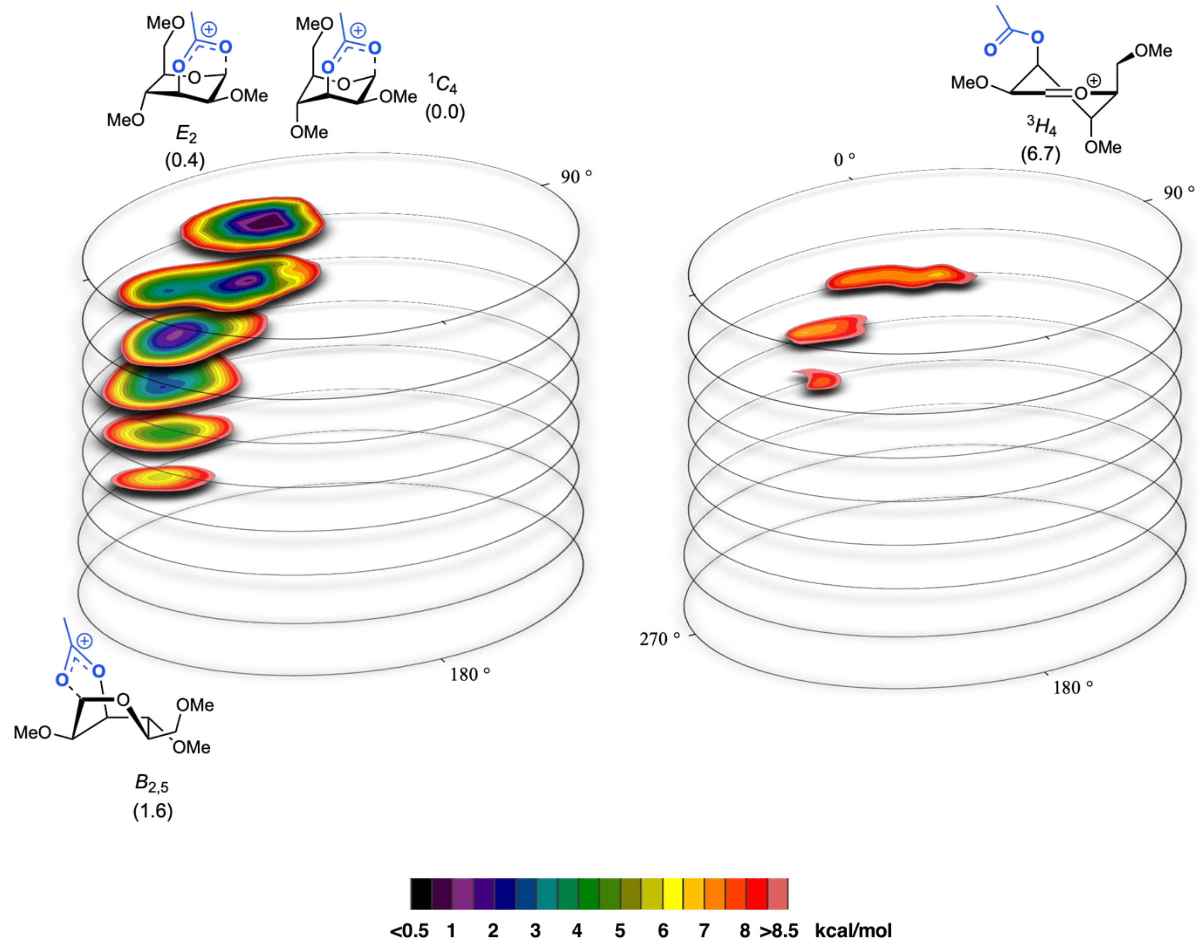

Supplementary Figure 17. CEL map of 3-*O*-acetyl-2,4,6-tri-*O*-methyl-manno-D-pyranosyl cation. All energies are computed at PCM(CH_2Cl_2)-B3LYP/6-311G(d,p) at *T*=213.15 K and expressed as solution-phase Gibbs free energy.[12]

3-*O*-Acetyl-2,4,6-tri-*O*-methyl-galacto-D-pyranosyl cation

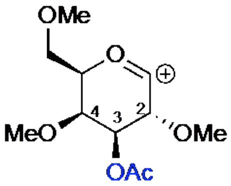

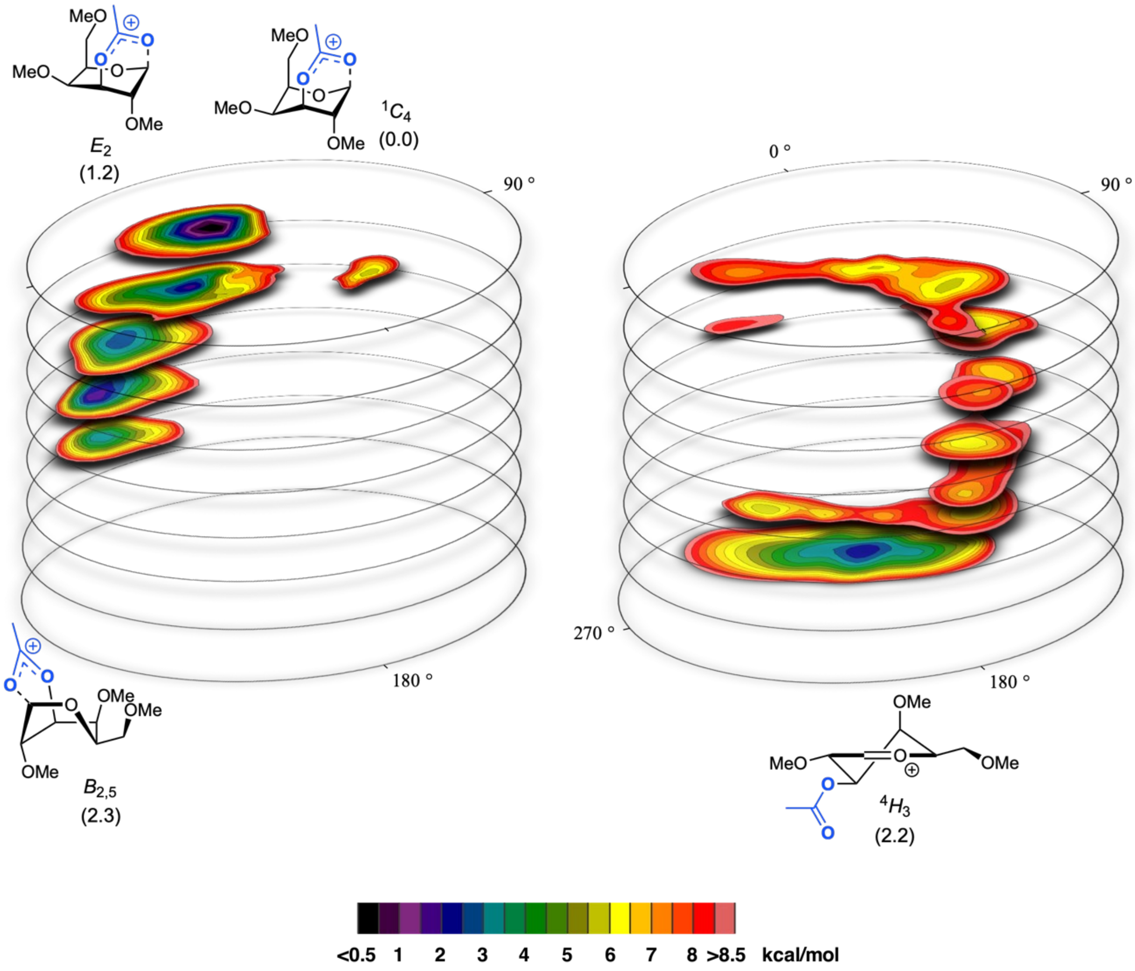

Supplementary Figure 18. CEL map of 3-*O*-acetyl-2,4,6-tri-*O*-methyl-galacto-D-pyranosyl cation. All energies are computed at PCM(CH_2Cl_2)-B3LYP/6-311G(d,p) at *T*=213.15 K and expressed as solution-phase Gibbs free energy.[12]

4-*O*-Acetyl-2,3,6-tri-*O*-methyl-gluco-D-pyranosyl cation

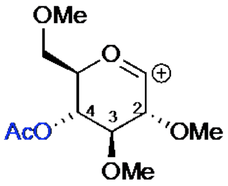

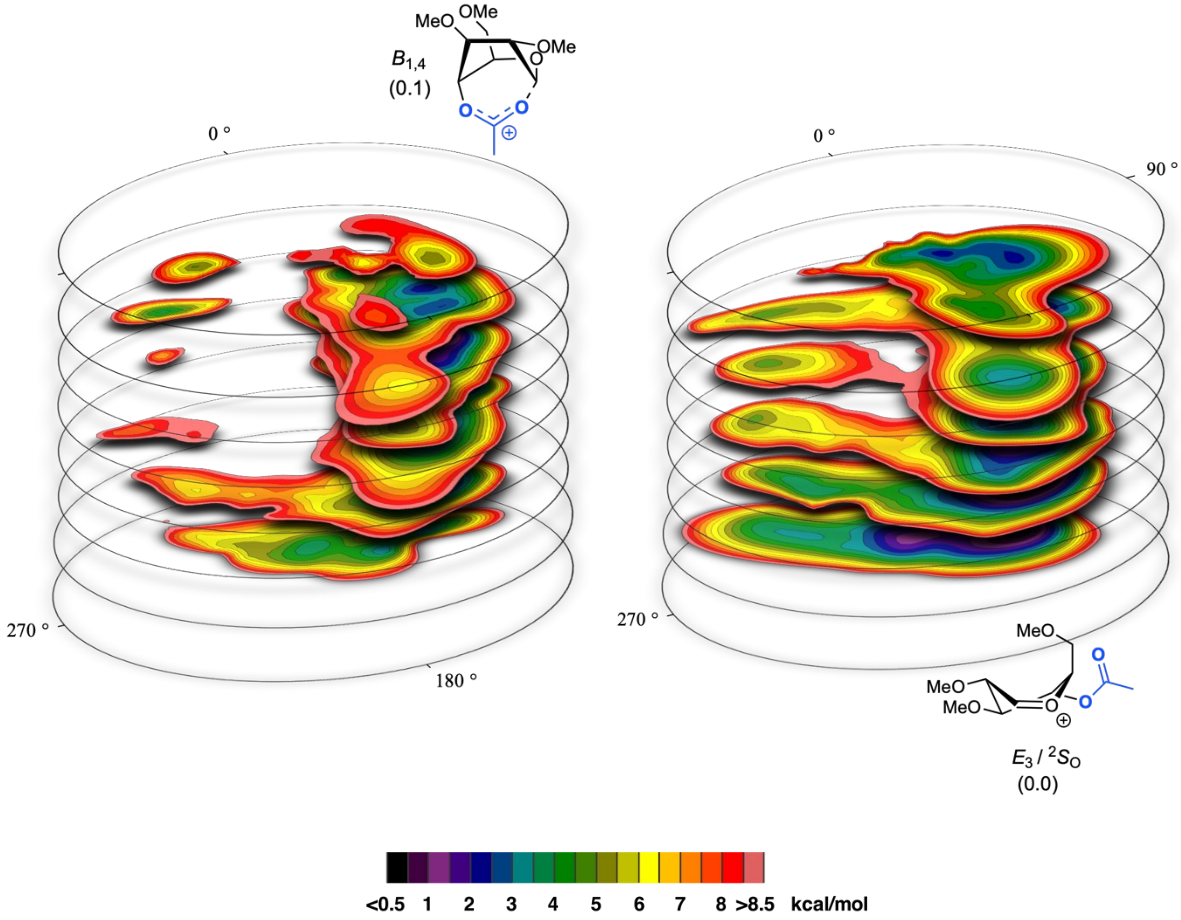

Supplementary Figure 19. CEL map of 4-*O*-acetyl-2,3,6-tri-*O*-methyl-gluco-D-pyranosyl cation. All energies are computed at PCM(CH_2Cl_2)-B3LYP/6-311G(d,p) at *T*=213.15 K and expressed as solution-phase Gibbs free energy.[12]

4-*O*-Acetyl-2,3,6-tri-*O*-methyl-manno-D-pyranosyl cation

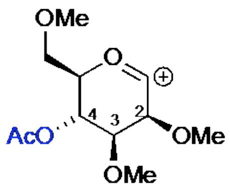

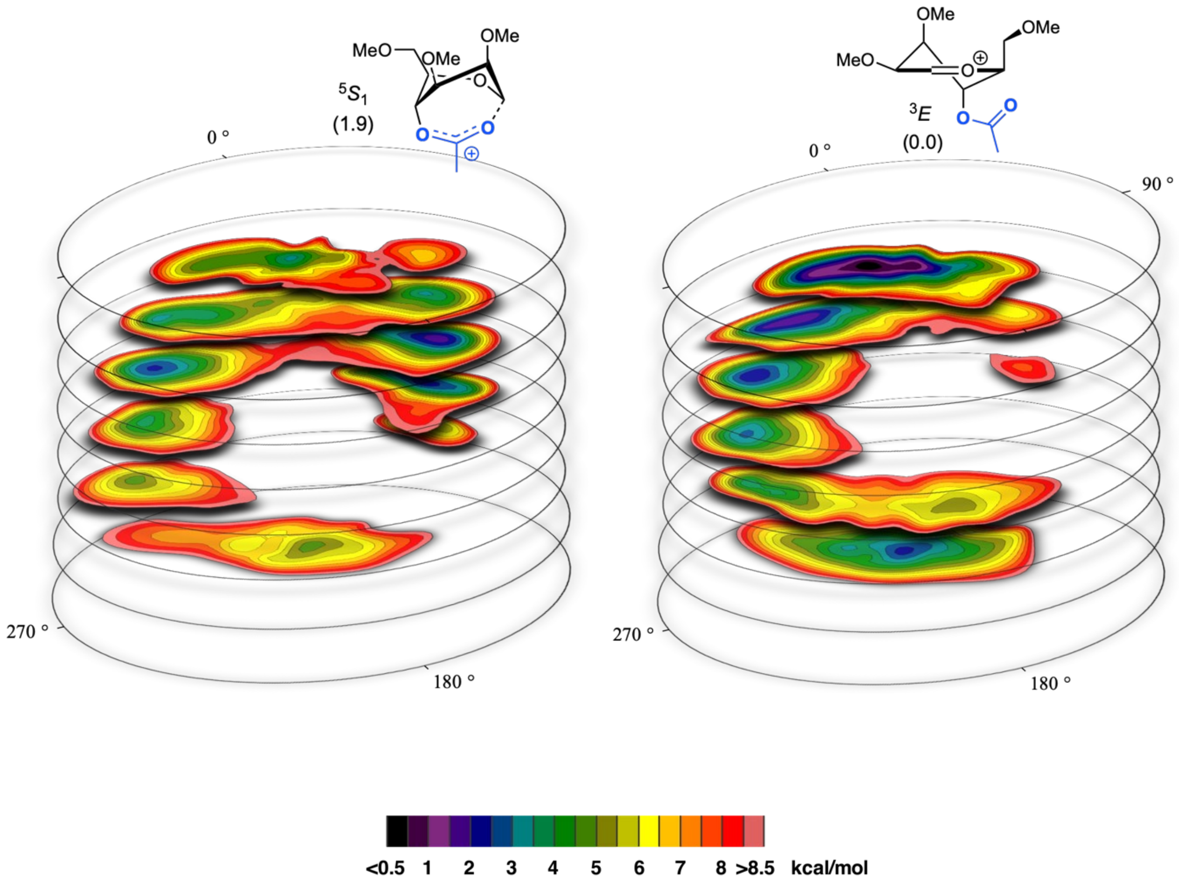

Supplementary Figure 20. CEL map of 4-*O*-acetyl-2,3,6-tri-*O*-methyl-manno-D-pyranosyl cation. All energies are computed at PCM(CH_2Cl_2)-B3LYP/6-311G(d,p) at *T*=213.15 K and expressed as solution-phase Gibbs free energy.[12]

4-*O*-Acetyl-2,3,6-tri-*O*-methyl-galacto-D-pyranosyl cation

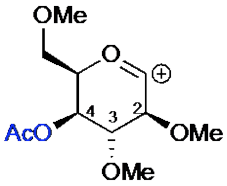

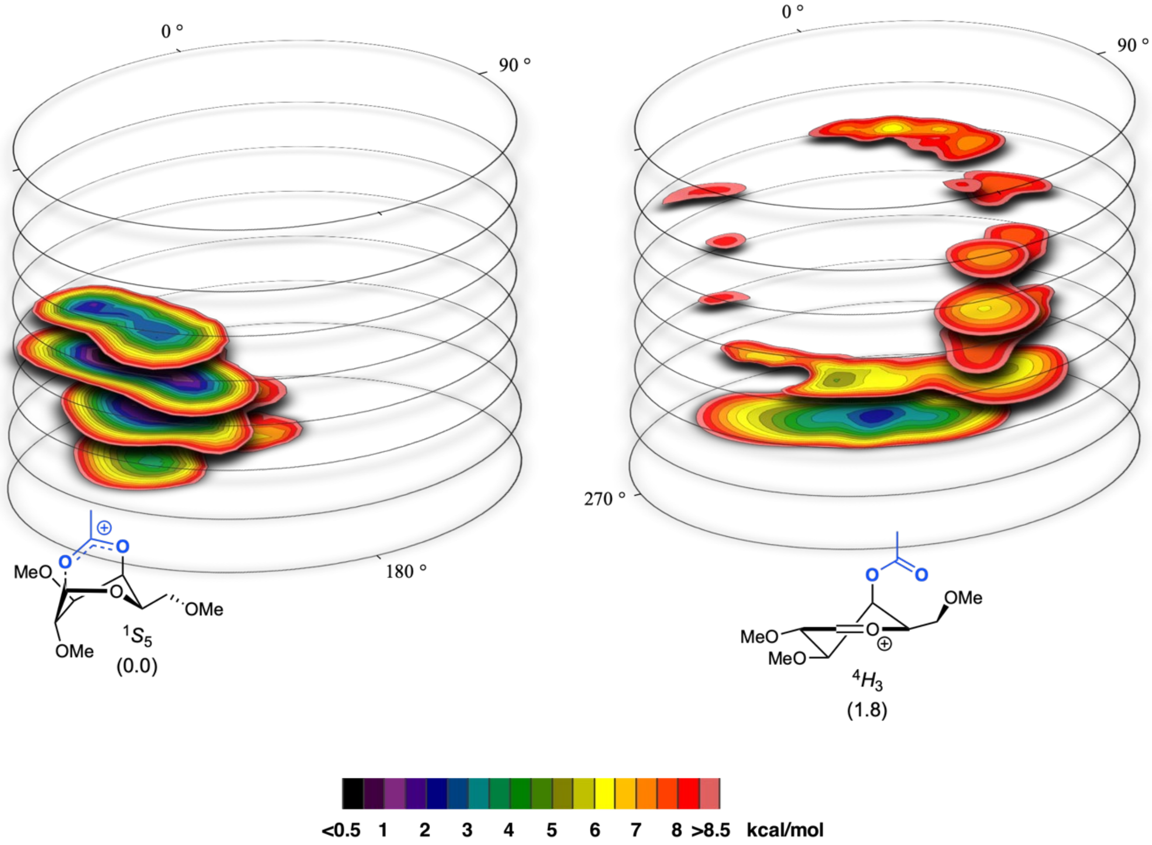

Supplementary Figure 21. CEL map of 4-*O*-acetyl-2,3,6-tri-*O*-methyl-galacto-D-pyranosyl cation. All energies are computed at PCM(CH_2Cl_2)-B3LYP/6-311G(d,p) at T=213.15 K and expressed as solution-phase Gibbs free energy.[12]

6-*O*-Acetyl-2,3,4-tri-*O*-methyl-gluco-D-pyranosyl cation

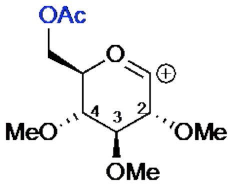

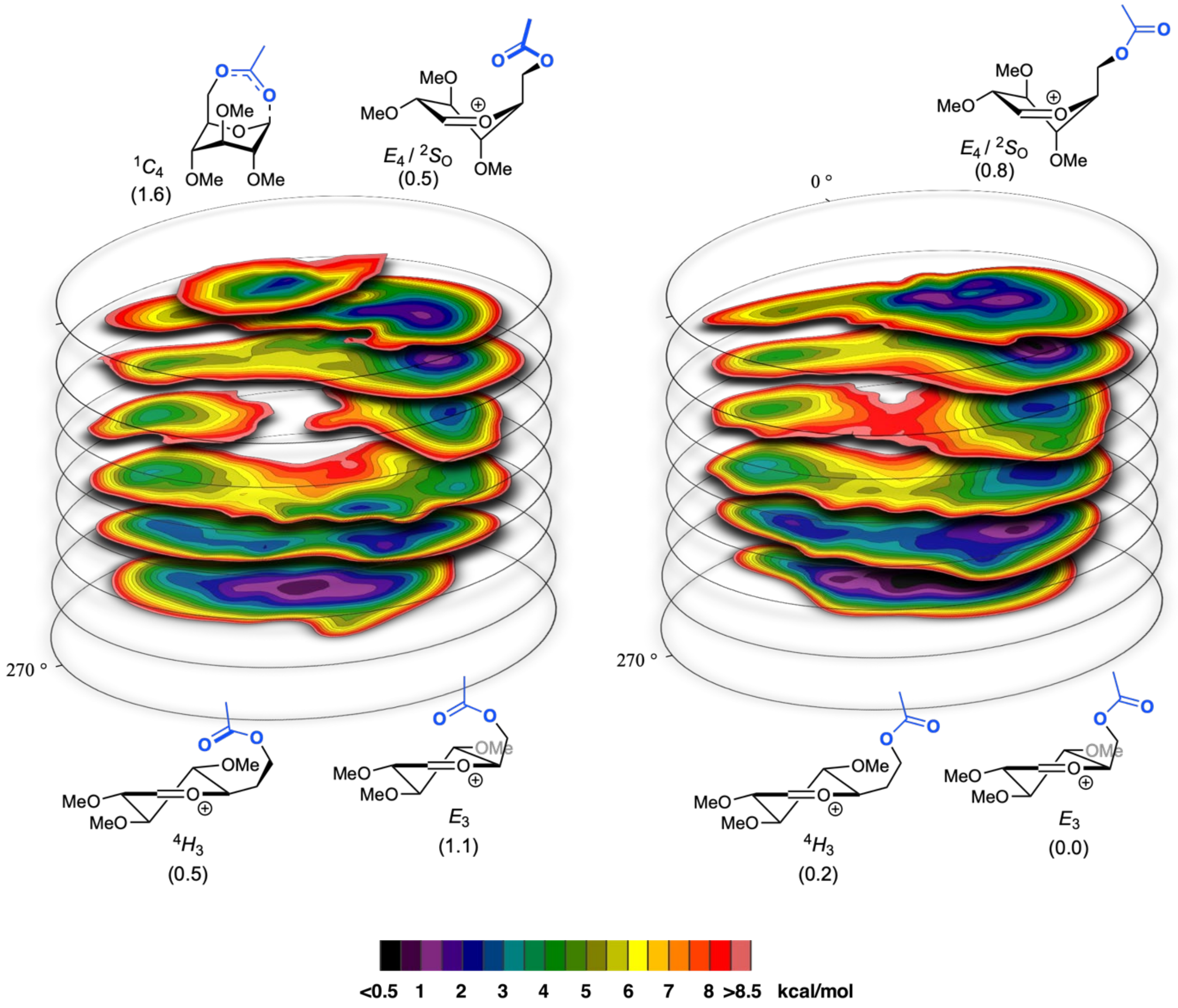

Supplementary Figure 22. CEL map of 6-*O*-acetyl-2,3,4-tri-*O*-methyl-gluco-D-pyranosyl cation. All energies are computed at PCM(CH_2Cl_2)-B3LYP/6-311G(d,p) at *T*=213.15 K and expressed as solution-phase Gibbs free energy.[12]

6-*O*-Acetyl-2,3,4-tri-*O*-methyl-manno-D-pyranosyl cation

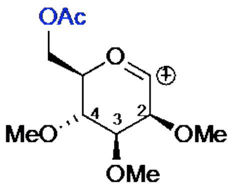

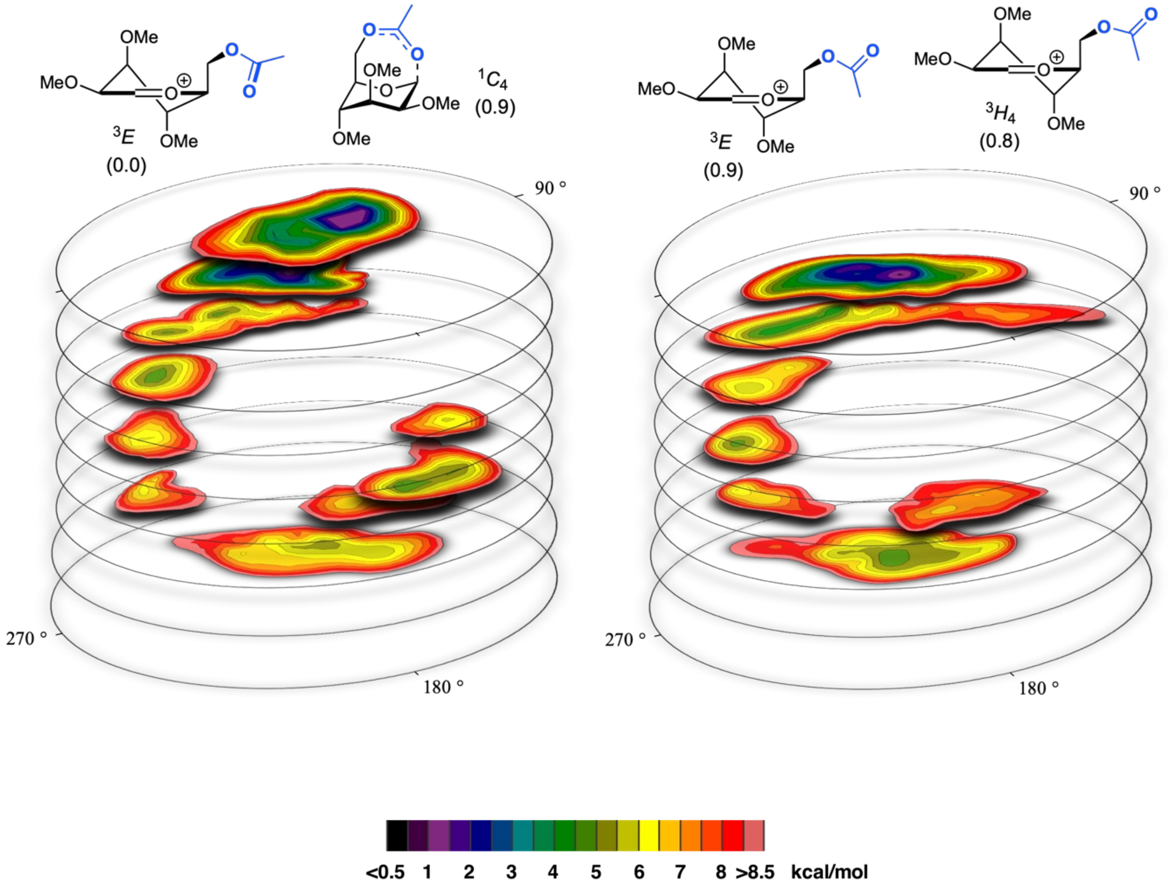

Supplementary Figure 23. CEL map of 6-*O*-acetyl-2,3,4-tri-*O*-methyl-manno-D-pyranosyl cation. All energies are computed at PCM(CH_2Cl_2)-B3LYP/6-311G(d,p) at T=213.15 K and expressed as solution-phase Gibbs free energy.[12]

6-*O*-Acetyl-2,3,4-tri-*O*-methyl-galacto-D-pyranosyl cation

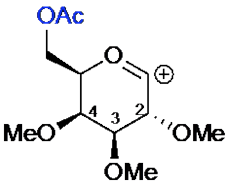

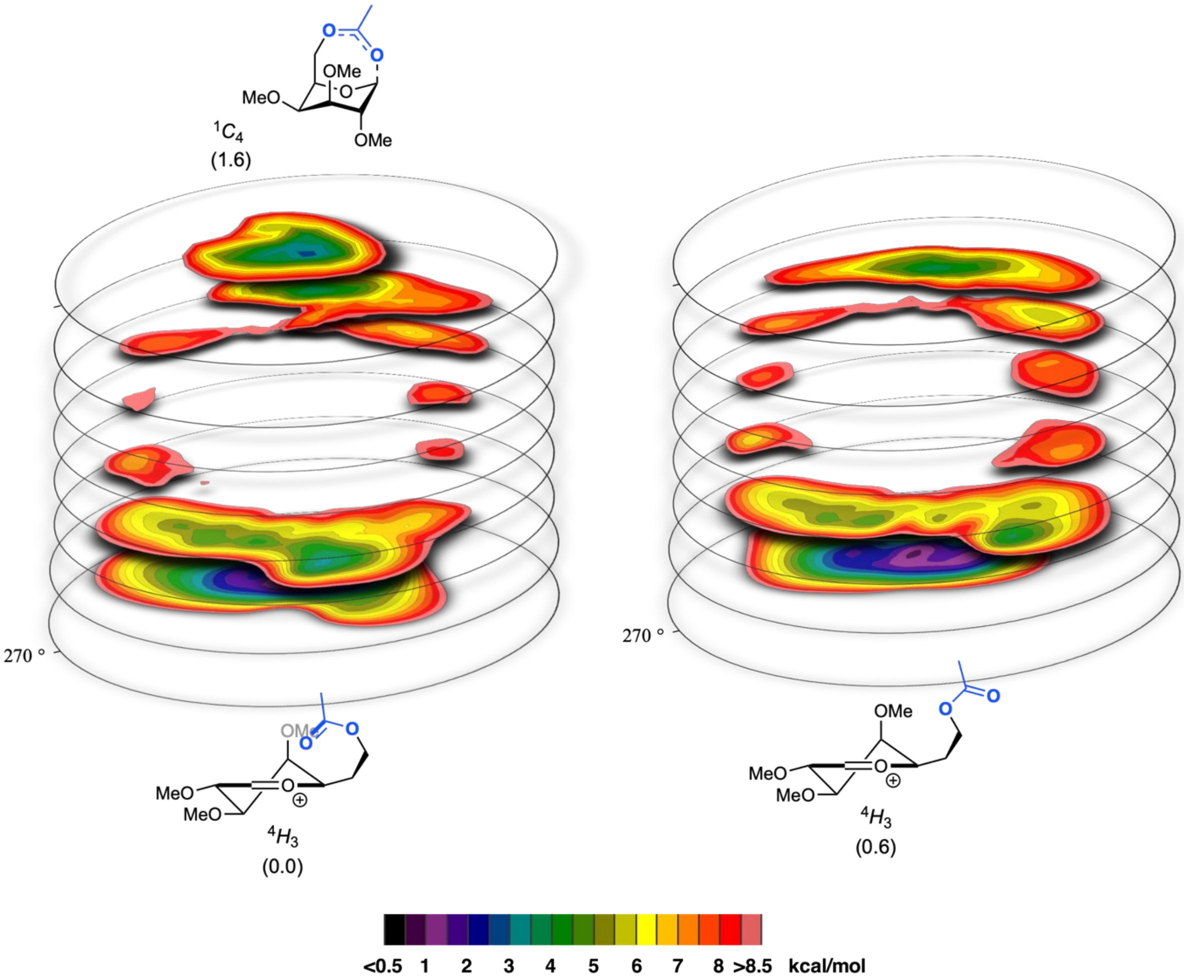

Supplementary Figure 24. CEL map of 6-*O*-acetyl-2,3,4-tri-*O*-methyl-galacto-D-pyranosyl cation. All energies are computed at PCM(CH_2Cl_2)-B3LYP/6-311G(d,p) at *T*=213.15 K and expressed as solution-phase Gibbs free energy.[12]

Gas-phase CEL maps

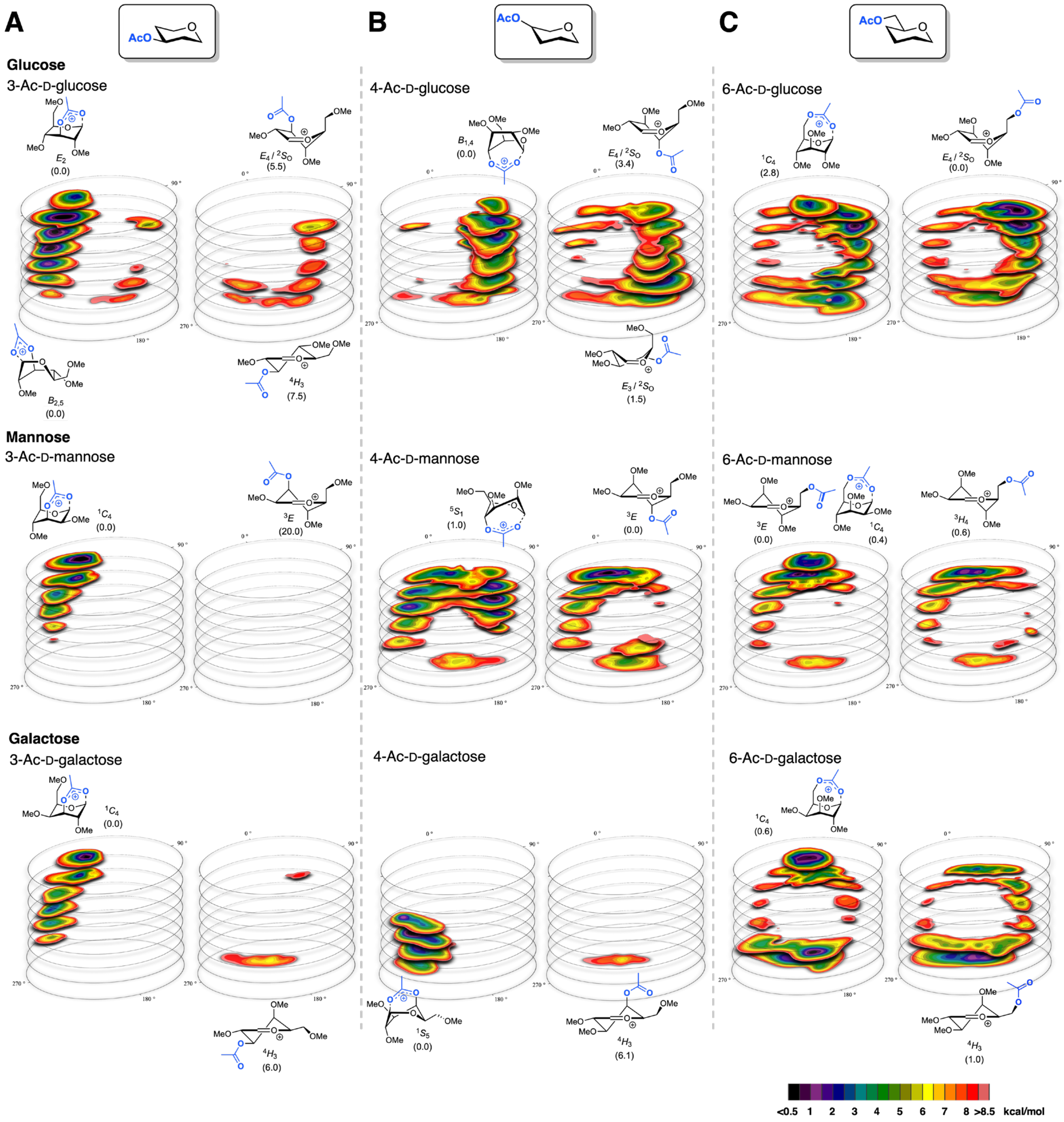

Supplementary Figure 25. CEL maps of selected pyranosyl oxocarbenium ions in which the found local minima are shown and indicated with their respective energy.[12] Two acetyl ester rotamers (R1; left and R2; right) were considered for all computed oxocarbenium ions generating two CEL maps. All energies are as calculated in the gas-phase at B3LYP/6-311G(d,p) at T=293.15 K and expressed as Gibbs free energy. A) CEL maps for the C3-acetyl pyranosyl ions; B) CEL maps for the C4-acetyl pyranosyl ions; C) CEL maps for the C6-acetyl pyranosyl ions.

Organic synthesis

General experimental procedures

All chemicals (Acros, Fluka, Merck, and Sigma-Aldrich) were used as received unless stated otherwise. Dichloromethane was stored over activated 4 Å molecular sieves (beads, 8-12 mesh, Sigma-Aldrich). Before use traces of water present in the donor, diphenyl sulfoxide (Ph_2SO) and tri-*tert*-butylpyrimidine (TTBP) were removed by co-evaporation with dry toluene. The acceptors were stored in stock solutions (DCM, 0.5 M) over activated 3 Å molecular sieves (rods, size 1/16 in., Sigma-Aldrich). Trifluoromethanesulfonic anhydride (Tf_2O) was distilled over P_2O_5 and stored at –20 °C under a nitrogen atmosphere. Overnight temperature control was achieved by an FT902 Immersion Cooler (Julabo). Column chromatography was performed on silica gel 60 Å (0.04 – 0.063 mm, Screening Devices B.V.). Size exclusion chromatography was carried out on Sephadex™ (LH-20, GE Healthcare Life Sciences) by isocratic elution with DCM:MeOH (1:1, v:v). TLC-analysis was conducted on TLC Silica gel 60 (Kieselgel 60 F_{254}, Merck) with UV detection by (254 nm) and by spraying with 20% sulfuric acid in ethanol followed by charring at ± 150 °C or by spraying with a solution of $(NH_4)_6Mo_7O_{24}\cdot H_2O$ (25 g/l) and $(NH_4)_4Ce(SO_4)_4\cdot 2H_2O$ (10 g/l) in 10% sulfuric acid in water followed by charring at ± 250 °C. High-resolution mass spectra were recorded on a Thermo Finnigan LTQ Orbitrap mass spectrometer equipped with an electrospray ion source in positive mode (source voltage 3.5 kV, sheath gas flow 10, capillary temperature 275 °C) with resolution R=60.000 at m/z=400 (mass range = 150-4000). ^1H and ^{13}C NMR spectra were recorded on a Bruker AV-400 NMR instrument (400 and 101 MHz respectively), a Bruker AV-500 NMR instrument (500 and 126 MHz respectively), or a Bruker AV-600 NMR instrument (600 and 151 MHz respectively). For samples measured in $CDCl_3$ chemical shifts (δ) are given in ppm relative to tetramethylsilane as an internal standard or the residual signal of the deuterated solvent. Coupling constants (*J*) are given in Hz. To get better resolution of signals with small coupling constants or overlapping signals a gaussian window function (LB ± -1 and GB ± 0.5) was used on the ^1H NMR spectrum. All given ^{13}C APT spectra are proton decoupled. NMR peak assignment was made using COSY, HSQC. If necessary additional NOESY, HMBC and HMBC-GATED experiments were used to elucidate the structure further. The anomeric product ratios were based on the integration of ^1H NMR. If the stereochemistry of the coupled product was not completely clear a deprotection step was used to verify the stereochemistry. IR spectra were recorded on a Shimadzu FTIR-8300 IR spectrometer with a resolution of 4 cm^{-1} and are reported in cm^{-1}. Specific rotations were measured on an MCP 100 Anton Paar polarimeter in $CHCl_3$ (10 mg/mL) at 589 nm unless stated otherwise.

General procedure IV: acetylation procedure

To a solution of the glycoside in pyridine (0.10 M), Ac_2O (10 eq.) and cat. DMAP was added. The mixture was stirred to completion before being concentrated *in vacuo*. The resulting crude was dissolved in EtOAc and washed with 1 M aq. HCl solution, sat. aq. $NaHCO_3$ and brine. The organic layer was dried and the resulting solvent evaporated under reduced pressure to obtain the acetylated sugar.

General procedure V: Methylation procedure

To a solution of the glycoside in DMF (0.20 M), NaH (60 wt% in mineral oil, 1.5 eq. per hydroxyl) and MeI (1.1 eq. per hydroxyl group) were added at room temperature under inert atmosphere. The mixture was allowed to stir to completion after which it was quenched by dropwise addition of methanol. The resulting suspension was taken up in diethyl ether and washed once with 5% aq. LiCl solution and brine. The resulting aqueous layer was extracted once with DCM. The combined organic layers were dried ($MgSO_4$), filtered and conc. *in vacuo*. The resulting residue was purified by crystallization or silica column chromatography.

General procedure VI: *S*-oxidation procedure

Based on a protocol by Gómez *et al.*[13] a solution of the thioglycoside in DCM (0.050 mM) was cooled to –78 °C under inert atmosphere and then *m*-CPBA (1.1 eq., 75 wt%) was added. The reaction was stirred for 3 h, diluted with DCM (30 mL) and washed with 10% aq. $Na_2S_2O_3$ solution, sat. aq. $NaHCO_3$ and brine. The organic layer was dried ($MgSO_4$), filtered, concentrated *in vacuo*. The resulting crude mixture was used directly for IRMPD experiments.

General procedure VII: pre-activation Tf_2O/Ph_2SO based *O*-glycosylation

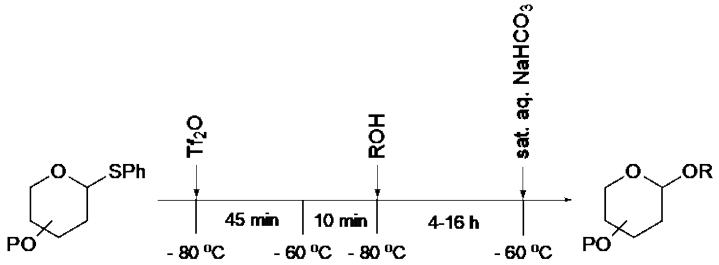

Supplementary Figure 26. Schematic representation of the reaction procedure during pre-activation Ph_2SO/Tf_2O mediated glycosylation.

A solution of the donor (100 µmol), Ph_2SO (26 mg, 130 µmol, 1.3 eq.) and TTBP (62 mg, 250 µmol, 2.5 eq.) in DCM (2 mL, 0.05 M) was stirred over activated 3 Å molecular sieves (rods, size 1/16 in., Sigma-Aldrich) for 30 min under an atmosphere of N_2. The solution was cooled to –80 °C and Tf_2O (22 µl, 130 µmol, 1.3 eq.) was slowly added to the reaction mixture. The reaction mixture was allowed to warm to –60 °C in approximately 45 min, followed by cooling to –80 °C and the addition of the acceptor (200 µmol, 2 eq.) in DCM (0.4 mL, 0.5 M). The reaction was allowed to warm up to –60 °C and stirred for an additional 4-18 h at this temperature till full reaction completion was observed. The reaction was quenched with sat. aq. $NaHCO_3$ at –60 °C and diluted with DCM (5 mL). The resulting solution was washed with H_2O and brine, dried over $MgSO_4$, filtered and concentrated under reduced pressure. Purification by column chromatography yielded the corresponding *O*-coupled glycoside.

Preparation of the donors for the model glycosylation reactions

Bz/Bn-*O*-protected glycosyl donors

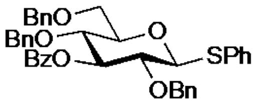

Phenyl 3-*O*-benzoyl-2,4,6-tri-*O*-benzyl-1-thio-β-D-glucopyranoside (10). The title compound was prepared according to literature procedure.[14] ^{1}H NMR (400 MHz, $CDCl_3$, HH-COSY, HSQC): δ 8.00 – 7.91 (m, 2H, CH_{arom}), 7.62 – 7.57 (m, 2H, CH_{arom}), 7.57 – 7.52 (m, 1H, CH_{arom}), 7.44 – 7.24 (m, 10H, CH_{arom}), 7.14 – 7.07 (m, 8H, CH_{arom}), 7.02 (td, *J* = 4.7, 2.9 Hz, 2H, CH_{arom}), 5.58 (t, *J* = 9.2 Hz, 1H, H-3), 4.81 – 4.74 (m, 2H, H-1, C*H*H Bn), 4.64 (d, *J* = 12.0 Hz, 1H, C*H*H Bn), 4.58 – 4.51 (m, 2H, 2x CH*H* Bn), 4.51 – 4.45 (m, 2H, CH_2 Bn), 3.83 (t, *J* = 9.6 Hz, 1H, H-4), 3.80 – 3.73 (m, 2H, 2x H-6), 3.64 – 3.58 (m, 2H, H-2, H-5); ^{13}C NMR (101 MHz, $CDCl_3$, HSQC): δ 165.6 (C=O Bz), 138.2, 137.5, 137.4, 133.7 (C_q), 133.2, 132.2 (CH_{arom}), 130.1 (C_q), 129.9, 129.1, 128.5, 128.5, 128.4, 128.4, 128.3, 128.2, 127.9, 127.9, 127.8, 127.8, 127.7 (CH_{arom}), 87.6 (C-1), 79.0 (C-2/C-5), 78.7 (C-2/C-5), 78.1 (C-3), 75.9 (C-4), 74.9 (CH_2 Bn), 74.6 (CH_2 Bn), 73.6 (CH_2 Bn), 68.8 (C-6); HRMS: $[M+Na]^+$ calcd for $C_{40}H_{38}NaO_6S$ 669.22813, found 669.22756.

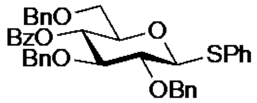

Phenyl 4-*O*-benzoyl-2,3,6-tri-*O*-benzyl-1-thio-β-D-glucopyranoside (11). The title compound was prepared according to literature procedure.[14] ^{1}H NMR (400 MHz, $CDCl_3$, HH-COSY, HSQC): δ 8.23 – 8.18 (m, 1H, CH_{arom}), 8.03 – 7.95 (m, 2H, CH_{arom}), 7.76 – 7.70 (m, 1H, CH_{arom}), 7.67 – 7.63 (m, 2H, CH_{arom}), 7.62 – 7.55 (m, 2H, CH_{arom}), 7.47 (ddd, *J* = 8.1, 6.2, 1.6 Hz, 4H, CH_{arom}), 7.43 – 7.34 (m, 3H, CH_{arom}), 7.33 – 7.23 (m, 8H, CH_{arom}), 7.19 – 7.08 (m, 4H, CH_{arom}), 5.38 – 5.31 (m, 1H, H-4), 4.96 (d, *J* = 10.3 Hz, 1H, C*H*H Bn), 4.85 – 4.76 (m, 3H, H-1, C*H*H Bn, CH*H* Bn), 4.68 (d, *J* = 11.0 Hz, 1H, CH*H* Bn), 4.53 (s, 2H, CH_2 Bn), 3.88 (t, *J* = 9.0 Hz, 1H, H-3), 3.82 (dt, *J* = 10.1, 4.5 Hz, 1H, H-5), 3.72 – 3.61 (m, 3H, H-2, 2x H-6); ^{13}C NMR (101 MHz, $CDCl_3$, HSQC): δ 165.5 (C=O Bz), 138.0, 138.0, 137.8 (C_q), 134.7 (CH_{arom}), 133.7 (C_q), 133.4, 132.0, 130.7, 129.9 (CH_{arom}), 129.7 ($C_{q\text{-}arom}$), 129.1, 129.0, 128.6, 128.6, 128.4, 128.4, 128.1, 128.1, 127.8, 127.7, 127.6 (CH_{arom}), 87.7 (C-1), 83.9 (C-3), 80.8 (C-2), 78.0 (C-5), 75.7, 75.6, 73.7 (CH_2 Bn), 71.4 (C-4), 69.9 (C-6); HRMS: $[M+Na]^+$ calcd for $C_{40}H_{38}NaO_6S$ 669.22813, found 669.22815.

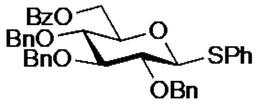

Phenyl 6-*O*-benzoyl-2,3,4-tri-*O*-benzyl-1-thio-β-D-glucopyranoside (12). The title compound was prepared according to literature procedure.[15] ^{1}H NMR (400 MHz, $CDCl_3$, HH-COSY, HSQC): δ 8.07 – 8.00 (m, 2H, CH_{arom}), 7.65 – 7.56 (m, 1H, CH_{arom}), 7.58 – 7.51 (m, 2H, CH_{arom}), 7.51 – 7.42 (m, 2H, CH_{arom}), 7.45 – 7.38 (m, 2H, CH_{arom}), 7.40 – 7.22 (m, 13H, CH_{arom}), 7.25 – 7.16 (m, 1H, CH_{arom}), 7.16 – 7.10 (m, 2H, CH_{arom}), 4.97 – 4.83 (m, 4H, 2x C*H*H Bn, CH_2 Bn), 4.75 (d, *J* = 10.2 Hz, 1H, CH*H* Bn), 4.72 – 4.64 (m, 2H, H-1, H-6), 4.62 (d, *J* = 10.8 Hz, 1H, CH*H* Bn), 4.44 (dd, *J* = 11.9, 4.9 Hz, 1H, H-6), 3.77 (t, *J* = 8.6 Hz, 1H, H-3), 3.73 – 3.60 (m, 2H, H-4, H-5), 3.53 (dd, *J* = 9.8, 8.7 Hz, 1H, H-2); ^{13}C NMR (101 MHz, $CDCl_3$, HSQC): δ 166.2 (C=O Bz), 138.2, 138.0, 137.6, 133.3 ($C_{q\text{-}arom}$), 133.3, 132.4 (CH_{arom}), 130.0 (C_q), 129.9, 129.0, 128.7, 128.6, 128.5, 128.3, 128.2, 128.2, 128.1, 128.1, 128.0, 127.8 (CH_{arom}), 87.4 (C-1), 86.9 (C-3), 80.8 (C-2), 77.7 (C-4), 77.2 (C-5), 76.1, 75.6, 75.3 (CH_2 Bn), 63.7 (C-6); HRMS: $[M+Na]^+$ calcd for $C_{40}H_{38}NaO_6S$ 669.22813, found 669.22788.

Preparation of Donor 13

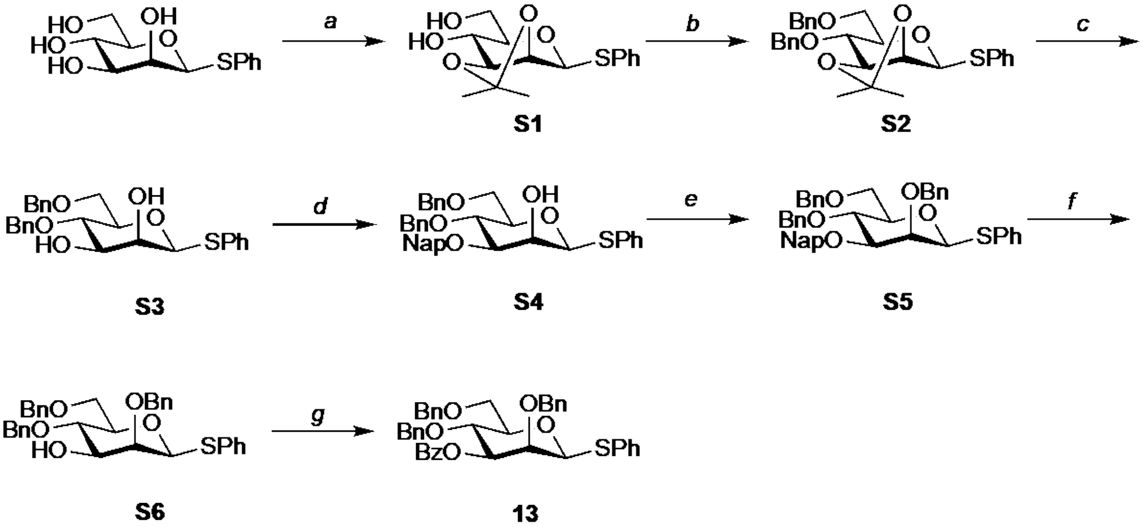

Supplementary Figure 27. Donor 13 synthesis. *Reagents and conditions:* a) i) 2,2-dimethoxypropane, acetone, $Sc(OTf)_3$, ii) AcOH, MeOH, DCM, reflux, S1: 56%; b) BnBr, NaH, DMF, S2: 92%; c) PTSA, MeOH, 50 °C, S3: 99%; d) i) di-n-butyltin(IV) oxide, toluene reflux, ii) NapBr, CsF, DMF, S4: 79%; e) BnBr, NaH, DMF, S5: 76%; f) DDQ, DCM, H_2O, S6: 62%; g) BzCl, pyridine, 13: 100%.

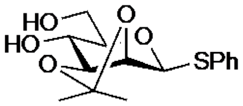

Phenyl 2,3-*O*-isopropylidene-1-thio-β-D-mannopyranoside (S1). According to a modified literature procedure.[16] phenyl 1-thio-β-D-mannopyranoside[17] (4.62 g, 17.0 mmol) was suspended in 80 mL 2,2-dimethoxypropane and 25 mL acetone. 50 mg $Sc(OTf)_3$ was added, and the suspension was stirred until everything was dissolved. The solution was concentrated to 25% of the original volume, 50 mL acetone was added and the reaction was stirred for 1 h, before being quenched with 0.3 mL triethylamine and concentrated under reduced pressure. The residue was dissolved in DCM and washed with water. The organic phase was dried with $MgSO_4$ and concentrated to give the crude diisopropylidene as yellowish powder, which was used without further purification. The crude product was dissolved in 5:13:7 DCM/MeOH/AcOH and heated to a vigorous reflux until the title compound was the major product together with a few percent unreacted starting material. The reaction mixture was diluted with toluene, concentrated under reduced pressure and co-evaporated with toluene two more times. The residue was purified over silica (30% ⟶ 50% acetone in pentane) yielding the title compound (2.97 g, 9.5 mmol, 56%) as off-white powder. TLC: R_f 0.35, (pentane:acetone, 60:40, v:v); $[\alpha]_D^{25}$ -114.1° (*c* 0.27, $CHCl_3$); IR (thin film, cm^{-1}): 733, 1066, 1090, 1217, 1483, 3449; ^1H NMR (400 MHz, $CDCl_3$, HH-COSY, HSQC): δ 7.53 – 7.48 (m, 2H, CH_{arom}), 7.35 – 7.26 (m, 3H, CH_{arom}), 5.11 (d, *J* = 2.2 Hz, 1H, H-1), 4.45 (dd, *J* = 5.5, 2.2 Hz, 1H, H-2), 4.10 (dd, *J* = 7.2, 5.5 Hz, 1H, H-3), 3.96 – 3.89 (m, 1H, H-6), 3.86 – 3.78 (m, 2H, H-4, H-6), 3.32 (ddd, *J* = 9.7, 5.1, 3.6 Hz, 1H, H-5), 2.89 (d, *J* = 3.7 Hz, 1H, 4-OH), 2.31 (t, *J* = 6.6 Hz, 1H, 6-OH), 1.59 (s, 3H, CH_3 isopropylidene), 1.42 (s, 3H, CH_3 isopropylidene); ^{13}C NMR (101 MHz, $CDCl_3$, HSQC): δ 134.7 (C_q), 130.9, 129.3, 127.7 (CH_{arom}), 111.0 (C_q isopropylidene), 84.2 (C-1), 80.3 (C-3), 78.5 (C-5), 76.1 (C-2), 70.1 (C-4), 62.8 (C-6), 28.2, 26.5 (CH_3 isopropylidene); HRMS: $[M+Na]^+$ calcd for $C_{15}H_{20}NaO_5S$ 335.09237, found 335.09217.

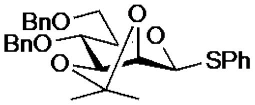

Phenyl 4,6-di-*O*-benzyl-2,3-*O*-isopropylidene-1-thio-β-D-mannopyranoside (S2). **S1** (2.94 g, 9.41 mmol) was dissolved in DMF, and benzyl bromide (3.35 mL, 28.2 mmol, 3 eq.) and sodium hydride (60% dispersion in mineral oil, 1.13 g, 28.2 mmol, 3 eq) were added. When TLC shows full conversion, the reaction mixture was quenched with water and extracted twice with diethyl ether. Combined organic phases were dried with $MgSO_4$ and concentrated under reduced pressure. The residue was purified over silica (5% → 10% acetone in pentane) yielding the title compound (4.26 g, 8.6 mmol, 92%) as white powder. TLC: R_f 0.25, (pentane:acetone, 90:10, v:v); $[\alpha]_D^{25}$ -84.0° (*c* 0.43, $CHCl_3$); IR (thin film, cm^{-1}): 695, 736, 1059, 1216, 1380; ^1H NMR (400 MHz, $CDCl_3$, HH-COSY, HSQC): δ 7.58 – 7.53 (m, 2H, CH_{arom}), 7.35 – 7.18 (m, 13H, CH_{arom}), 5.07 (d, *J* = 2.1 Hz, 1H, H-1), 4.81 (d, *J* = 11.6 Hz, 1H, C*H*H Bn), 4.58 (d, *J* = 11.6 Hz, 1H, CH*H* Bn), 4.55 (s, 2H, CH_2 Bn), 4.46 (dd, *J* = 5.8, 2.1 Hz, 1H, H-2), 4.35 – 4.28 (m, 1H, H-3), 3.85 (dd, *J* = 10.2, 1.8 Hz, 1H, H-6), 3.70 – 3.63 (m, 1H, H-6), 3.63 – 3.56 (m, 2H, H-4, H-5), 1.57 (s, 3H, CH_3 isopropylidene), 1.42 (s, 3H, CH_3 isopropylidene); ^{13}C NMR (101 MHz, $CDCl_3$, HSQC): δ 138.5, 138.0, 135.5 (C_q), 130.5, 129.1, 128.5, 128.4, 128.1, 127.9, 127.8, 127.6, 127.2 (CH_{arom}), 110.7 (C_q isopropylidene), 84.4 (C-1), 79.8 (C-3), 78.5 (C-4), 76.1 (C-2), 75.4 (C-5), 73.6, 72.7 (CH_2 Bn), 70.4 (C-6), 27.9, 26.4 (CH_3 isopropylidene); HRMS: $[M+NH_4]^+$ calcd for $C_{29}H_{36}NO_5S$ 515.23087, found 515.23058.

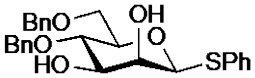

Phenyl 4,6-di-*O*-benzyl-1-thio-β-D-mannopyranoside (S3). **S2** (4.20 g, 8.53 mmol) and PTSA (162 mg, 0.853 mmol, 0.1 eq) were dissolved in 75 mL methanol and heated to 50 °C. When the reaction was complete, the title compound was precipitated from the solution. Triethylamine (0.24 mL, 1.71 mmol, 0.2 eq.) was added and the mixture was cooled to -30 °C. The product was collected by filtration and washed with a few mL of very cold methanol, yielding the title compound (2.72 g, 6.0 mmol, 71%) as fluffy white solid (mp: 146 °C). The concentrated mother liquor contained ca. 1.1 g (28%) of slightly impure product of sufficient quality to use in subsequent reactions. TLC: R_f 0.15, (pentane:EtOAc, 60:40, v:v); $[\alpha]_D^{25}$ – 114.1 ° (*c* 0.27, $CHCl_3$); IR (thin film, cm^{-1}): 733, 1066, 1217, 3449 ; ^1H NMR (400 MHz, $CDCl_3$, HH-COSY, HSQC): δ 7.61 – 7.46 (m, 2H, CH_{arom}), 7.38 – 7.16 (m, 13H, CH_{arom}), 4.84 (d, *J* = 1.1 Hz, 1H, H-1), 4.78 (d, *J* = 11.3 Hz, 1H, C*H*H Bn), 4.66 – 4.60 (m, 2H, C*H*H Bn, CH*H* Bn), 4.56 (d, *J* = 11.9 Hz, 1H, CH*H* Bn), 4.17 – 4.10 (m, 1H, H-2), 3.82 (dd, *J* = 10.9, 2.0 Hz, 1H, H-6), 3.75 (dd, *J* = 10.9, 5.1 Hz, 1H, H-6), 3.72 – 3.65 (m, 2H, H-3, H-4), 3.47 (dq, *J* = 7.4, 3.1, 2.5 Hz, 1H, H-5), 2.72 (d, *J* = 5.8 Hz, 1H, 2-OH), 2.61 – 2.53 (m, 1H, 3-OH); ^{13}C NMR (101 MHz, $CDCl_3$, HSQC): δ 138.2, 138.2, 134.4 (C_q), 131.3, 129.2, 128.7, 128.5, 128.2, 128.1, 128.0, 127.8, 127.6 (CH_{arom}), 87.1 (C-1), 79.6 (C-5), 75.8, 75.4 (C-3/C-4), 75.0, 73.7 (CH_2 Bn), 72.7 (C-2), 69.3 (C-5); HRMS: $[M+NH_4]^+$ calcd for $C_{26}H_{32}NO_5S$ 470.19957, found 470.19932.

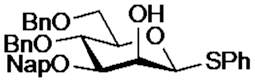

Phenyl 3-*O*-(2-naphthyl)methyl-4,6-di-*O*-benzyl-1-thio-β-D-mannopyranoside (S4). Diol **S3** (3.82 g, 8.44 mmol) and di-n-butyltin oxide (2.73 g, 11.0 mmol, 1.3 eq.) were refluxed in toluene for 2 h, while removing water using a Dean-Stark setup. The mixture was cooled to room temperature and concentrated under reduced pressure. The residue was dissolved in DMF, CsF (1.67 g, 11.0 mmol, 1.3 eq) and napthyl bromide (2.43 g, 11.0 mmol, 1.3 eq) were added. After overnight reaction, water and diethyl ether were added, causing the product to precipitate as white solid (3.36 g) Silica chromatography of the concentrated organic phase yielded an additional 590 mg product, bringing the total yield to 3.95 g, 6.7 mmol, 79%. TLC: R_f 0.40, ($CHCl_3$:acetone, 90:10, v:v);

$[\alpha]_D^{25}$ –32.2° (*c* 1.12, $CHCl_3$); IR (thin film, cm^{-1}):698, 738, 1027, 1074, 1089, 1120; ^1H NMR (400 MHz, $CDCl_3$, HH-COSY, HSQC): δ 7.85 – 7.72 (m, 4H, CH_{arom}), 7.55 – 7.43 (m, 5H, CH_{arom}), 7.35 – 7.18 (m, 13H, CH_{arom}), 4.93 – 4.87 (m, 2H, 2x C*H*H Bn/Nap), 4.82 (d, *J* = 11.8 Hz, 1H, CH*H* Bn/Nap), 4.79 – 4.78 (m, 1H, H-1), 4.62 – 4.57 (m, 2H, C*H*H Bn/Nap, CH*H* Bn/Nap), 4.54 (d, *J* = 11.9 Hz, 1H, CH*H* Bn/Nap), 4.30 (t, *J* = 2.9 Hz, 1H, H-2), 3.90 – 3.79 (m, 2H, H-4, H-6), 3.72 (dd, *J* = 10.9, 5.9 Hz, 1H, H-6), 3.66 (dd, *J* = 9.0, 3.3 Hz, 1H, H-3), 3.50 (ddd, *J* = 9.8, 5.9, 1.9 Hz, 1H, H-5), 2.72 (d, *J* = 3.0 Hz, 1H, OH); ^{13}C NMR (101 MHz, $CDCl_3$, HSQC): δ 138.4, 138.2, 135.0, 135.0, 133.3, 133.2 (C_q), 131.0, 129.1, 128.6, 128.5, 128.4, 128.1, 128.1, 127.9, 127.9, 127.9, 127.7, 127.4, 127.0, 126.4, 126.3, 125.9 (CH_{arom}), 86.8 (C-1), 82.6 (C-3), 79.8 (C-5), 75.4 (CH_2 Bn/Nap), 74.4 (C-4), 73.6, 72.1 (CH_2 Bn/Nap), 70.2 (C-4), 69.5 (C-6); HRMS: $[M+NH_4]^+$ calcd for $C_{37}H_{40}NO_5S$ 610.26217, found 610.26172.

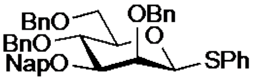

Phenyl 3-*O*-(2-naphthyl)methyl-2,4,6-tri-*O*-benzyl-1-thio-β-D-mannopyranoside (S5). S4 (3.30 g, 5.57 mmol) was dissolved in DMF at 0 °C. Benzyl bromide (0.99 mL, 8.35 mmol, 1.5 eq) and NaH (60% in mineral oil, 334 mg, 8.35 mmol, 1.5 eq) were added and the reaction mixture was allowed to warm to rt. When TLC showed full conversion, the reaction was quenched with water and extracted with diethyl ether. The organic phase was dried with $MgSO_4$ and concentrated under reduced pressure. The residue was purified over silica (15% diethyl ether in pentane) yielding the title compound (2.89 g, 4.2 mmol, 76%) as white solid. TLC: R_f 0.30, (pentane:Et_2O), 80:20, v:v); $[\alpha]_D^{25}$ –40.2° (*c* 0.59, $CHCl_3$); IR (thin film, cm^{-1}): 696, 738, 1026, 1072, 1122; ^1H NMR (400 MHz, $CDCl_3$, HH-COSY, HSQC): δ 7.85 – 7.76 (m, 3H, CH_{arom}), 7.73 – 7.67 (m, 1H, CH_{arom}), 7.54 – 7.41 (m, 8H, CH_{arom}), 7.38 – 7.15 (m, 17H, CH_{arom}), 5.09 (d, *J* = 11.5 Hz, 1H, C*H*H Bn/Nap), 4.96 – 4.89 (m, 2H, C*H*H Bn/Nap, CH*H* Bn/Nap), 4.87 (d, *J* = 12.0 Hz, 1H, C*H*H Bn/Nap), 4.82 (d, *J* = 12.0 Hz, 1H, CH*H* Bn/Nap), 4.77 (d, *J* = 1.1 Hz, 1H, H-1), 4.64 – 4.58 (m, 2H, C*H*H Bn/Nap, CH*H* Bn/Nap), 4.55 (d, *J* = 11.7 Hz, 1H, CH*H* Bn/Nap), 4.17 (dd, *J* = 3.0, 1.1 Hz, 1H, H-2), 3.98 (t, *J* = 9.6 Hz, 1H, H-4), 3.86 (dd, *J* = 11.0, 1.9 Hz, 1H, H-6), 3.76 (dd, *J* = 10.9, 6.5 Hz, 1H, H-6), 3.68 (dd, *J* = 9.4, 2.9 Hz, 1H, H-3), 3.54 (ddd, *J* = 9.8, 6.5, 1.9 Hz, 1H, H-5); ^{13}C NMR (101 MHz, $CDCl_3$, HSQC): δ 138.6, 138.4, 138.3, 135.8, 135.6, 133.3, 133.1 (C_q), 130.6, 129.0, 128.5, 128.4, 128.4, 128.3, 128.1, 128.0, 127.9, 127.8, 127.7, 127.5, 127.1, 126.5, 126.3, 126.1, 125.7 (CH_{arom}), 87.7 (C-1), 84.3 (C-3), 80.2 (C-5), 77.7 (C-2), 75.3, 75.2 (CH_2 Bn/Nap), 75.1 (C-4), 73.6, 72.7 (CH_2 Bn/Nap), 69.9 (C-6); HRMS: $[M+NH_4]^+$ calcd for $C_{44}H_{46}NO_5S$ 700.30967, found 700.30861.

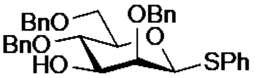

Phenyl 2,4,6-tri-*O*-benzyl-1-thio-β-D-mannopyranoside (S6). S5 (2.86 g, 4.19 mmol) was dissolved in 25mL 9:1 DCM/H_2O. DDQ (1.90 g, 8.38 mmol, 2 eq.) was added and the reaction was stirred at room temperature until TLC showed full conversion. The mixture was diluted with DCM and washed twice with sat. aq. $NaHCO_3$. The organic phase was dried with $MgSO_4$ and concentrated under reduced pressure. The residue was purified over silica (15% acetone in pentane), yielding the title compound (1.40 g, 2.58 mmol, 62%) as white solid. TLC: R_f 0.32, (pentane:acetone, 80:20, v:v); $[\alpha]_D^{25}$ –95.6° (*c* 0.95, $CHCl_3$); IR (thin film, cm^{-1}): 695, 732, 1027, 1062, 1073, 1089, 1452; ^1H NMR (400 MHz, $CDCl_3$, HH-COSY, HSQC): δ 7.57 – 7.51 (m, 2H, CH_{arom}), 7.48 – 7.44 (m, 2H, CH_{arom}), 7.41 – 7.20 (m, 16H, CH_{arom}), 5.03 (d, *J* = 11.5 Hz, 1H, C*H*H Bn), 4.82 (d, *J* = 1.1 Hz, 1H, H-1), 4.80 – 4.74 (m, 2H, C*H*H Bn, CH*H* Bn), 4.63 (d, *J* = 11.7 Hz, 1H, C*H*H Bn), 4.60 – 4.53 (m, 2H, 2x CH*H* Bn), 4.05 (dd, *J* = 3.5, 1.1 Hz, 1H, H-2), 3.86 (dd, *J* = 10.9, 2.0 Hz, 1H, H-6), 3.76 (dd, *J* = 11.0, 6.3 Hz, 1H, H-6), 3.73 – 3.69 (m, 1H, H-3), 3.65 (t, *J* = 9.3 Hz, 1H, H-4), 3.49 (ddd, *J* = 9.4, 6.1, 2.0 Hz, 1H, H-5), 2.18 (d, *J* = 7.8 Hz, 1H, OH); ^{13}C NMR (101 MHz, $CDCl_3$, HSQC): δ

138.5, 138.2, 138.1, 135.4 (C_q), 130.7, 129.1, 128.7, 128.6, 128.4, 128.4, 128.2, 128.2, 128.0, 128.0, 127.6, 127.3 (CH_{arom}), 87.8 (C-1), 80.7 (C-2), 79.8 (C-5), 76.5 (C-4), 76.3 (CH_2 Bn), 75.8 (C-3), 74.9, 73.6 (CH_2 Bn), 69.8 (C-6); HRMS: $[M+NH_4]^+$ calcd for $C_{33}H_{38}NO_5S$ 560.24652, found 560.24624.

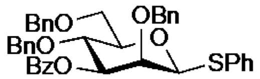

Phenyl 3-*O*-benzoyl-2,4,6-tri-*O*-benzyl-1-thio-β-D-mannopyranoside (13). S6 (1.36 g, 2.50 mmol) and benzoyl chloride (0.44 mL, 3.75 mmol, 1.5 eq) were dissolved in 5 mL pyridine. When TLC shows full conversion, the reaction mixture was diluted with ethyl acetate and washed twice with 1 M aq. HCl and once with sat. aq. $NaHCO_3$. The organic phase was dried with $MgSO_4$ and concentrated under reduced pressure. The residue was purified over silica (5% → 10% acetone in pentane), yielding the title compound in quantitative yield as colourless oil. TLC: R_f 0.28, (pentane:acetone, 90:10, v:v); $[\alpha]_D^{25}$ –74.7° (*c* 1.33, $CHCl_3$); IR (thin film, cm^{-1}): 695, 713, 733, 1025, 1062, 1089, 1266, 1452, 17165, 1718; ^1H NMR (400 MHz, $CDCl_3$, HH-COSY, HSQC): δ 8.03 – 7.97 (m, 2H, CH_{arom}), 7.61 – 7.52 (m, 3H, CH_{arom}), 7.46 – 7.41 (m, 2H, CH_{arom}), 7.35 (ddt, *J* = 8.0, 4.2, 2.3 Hz, 4H, CH_{arom}), 7.33 – 7.28 (m, 3H, CH_{arom}), 7.27 – 7.20 (m, 7H, CH_{arom}), 7.16 (dd, *J* = 5.0, 1.9 Hz, 3H, CH_{arom}), 7.10 – 7.05 (m, 2H, CH_{arom}), 5.26 (dd, *J* = 9.9, 3.2 Hz, 1H, H-3), 4.96 (d, *J* = 1.1 Hz, 1H, H-1), 4.82 (d, *J* = 11.4 Hz, 1H, C*H*H Bn), 4.74 (d, *J* = 11.4 Hz, 1H, CH*H* Bn), 4.71 – 4.64 (m, 2H, 2x C*H*H Bn), 4.60 – 4.54 (m, 2H, 2x CH*H* Bn), 4.37 (dd, *J* = 3.3, 1.1 Hz, 1H, H-2), 4.19 (t, *J* = 9.8 Hz, 1H, H-4), 3.89 – 3.78 (m, 2H, 2x H-6), 3.64 (ddd, *J* = 9.7, 5.4, 2.2 Hz, 1H, H-5); ^{13}C NMR (101 MHz, $CDCl_3$, HSQC): δ 165.9 (C=O Bz), 138.5, 137.8, 137.7, 135.2 (C_q), 133.5, 131.2, 129.9 (CH_{arom}), 129.6 (C_q), 129.1, 128.7, 128.4, 128.4, 128.3, 128.2, 128.1, 128.0, 127.9, 127.8, 127.6, 127.4 (CH_{arom}), 87.8 (C-1), 80.1 (C-5), 78.6 (C-2), 77.9 (C-3), 75.9, 75.2, 73.7 (CH_2 Bn), 73.4 (C-4), 69.6 (C-6); HRMS: $[M+NH_4]^+$ calcd for $C_{40}H_{42}NO_6S$ 664.27274, found 669.27257

Preparation of Donor 14

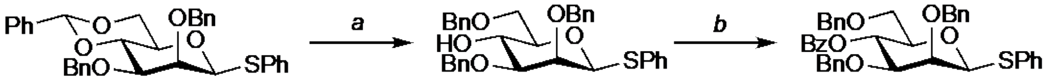

S7 **14**

Supplementary Figure 28. Donor **14** synthesis. *Reagents and conditions:* a) TES-H, TFA, DCM, 0 °C, **S7**: 71%; b) BzCl, pyridine, **14**: 97%.

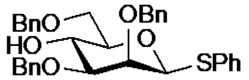

Phenyl 2,3,6-tri-*O*-benzyl-1-thio-β-D-mannopyranoside (S7). Phenyl 2,3-di-O-benzyl-4,6-O-benzylidene-1-thio-β-D-mannopyranoside[17] (2.00 g, 3.70 mmol) was dissolved in dichloromethane and cooled to 0 °C, after which TES-H (5.9 mL, 37.0 mmol, 10 eq) and TFA (2.8 mL, 37.0 mmol, 10 eq) were added. When TLC shows full conversion, the reaction is quenched with sat. aq. $NaHCO_3$ and diluted with DCM. Phases were separated and the aquatic phase was extracted with DCM. Combined organic phases were dried with $MgSO_4$ and concentrated under reduced pressure. The residue was purified over silica (15% acetone in pentane), yielding the title compound (1.42 g, 2.62 mmol, 71%) as waxy white solid. TLC: R_f 0.20, (pentane:EtOAc, 85:15, v:v); $[\alpha]_D^{25}$ –71.6° (*c* 0.83, $CHCl_3$); IR (thin film, cm^{-1}): 695, 732, 1026, 1064, 1128; ^1H NMR (400 MHz, $CDCl_3$, HH-COSY, HSQC): δ 7.53 – 7.44 (m, 4H, CH_{arom}), 7.38 – 7.25 (m, 13H, CH_{arom}), 7.25 – 7.18 (m, 3H, CH_{arom}), 4.98 (d, *J* = 11.4 Hz, 1H, C*H*H Bn), 4.86 (d, *J* = 11.4 Hz, 1H, CH*H* Bn), 4.80 (d, *J* = 1.1 Hz, 1H, H-1), 4.73 (d, *J* = 11.8 Hz, 1H, C*H*H Bn), 4.59 (d, *J* = 11.8 Hz, 1H, CH*H* Bn), 4.57 (s, 2H, CH_2 Bn), 4.15 (dd, *J* = 3.0, 1.1 Hz, 1H, H-2), 4.06 (td, *J* = 9.5, 1.9 Hz, 1H, H-4),

3.90 (dd, J = 10.4, 4.1 Hz, 1H, H-6), 3.80 (dd, J = 10.4, 6.4 Hz, 1H, H-6), 3.51 (ddd, J = 9.4, 6.3, 4.0 Hz, 1H, H-5), 3.44 (dd, J = 9.4, 3.0 Hz, 1H, H-3), 2.71 (d, J = 2.0 Hz, 1H, 4-OH); ^{13}C NMR (101 MHz, $CDCl_3$, HSQC): δ 138.2, 137.9, 135.6 (C_q), 130.8, 129.0, 128.7, 128.5, 128.5, 128.3, 128.1, 127.9, 127.9, 127.8, 127.7, 127.3 (CH_{arom}), 87.9 (C-1), 83.6 (C-3), 79.2 (C-5), 76.8 (C-2), 75.2, 73.8, 72.4 (CH_2 Bn), 71.2 (C-6), 68.7 (C-4); HRMS: $[M+NH_4]^+$ calcd for $C_{33}H_{38}NO_5S$ 560.24652, found 560.24596

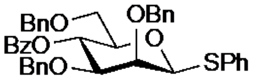

Phenyl 4-*O*-benzoyl-2,3,6-tri-*O*-benzyl-1-thio-β-D-mannopyranoside (14). S7 (1.40 g, 2.58 mmol) was dissolved in 5 mL pyridine with benzoyl chloride (0.45 mL, 3.87 mmol, 1.5 eq.) When TLC shows full conversion, the reaction mixture was diluted with ethyl acetate and washed with 1 M aq. HCl and sat. aq. $NaHCO_3$. The organic phase was dried and concentrated. The residue was purified over silica (10 ⟶ 15% EA in pentane) yielding the title compound (1.62 g, 2.51 mmol, 97%) as white amorphous solid. TLC: R_f 0.22, (pentane:EtOAc, 85:15, v:v); $[\alpha]_D^{25}$ –69.1° (c 1.01, $CHCl_3$); IR (thin film, cm^{-1}): 695, 711, 1027, 1068, 1109, 1266, 1452, 1724; ^{1}H NMR (400 MHz, $CDCl_3$, HH-COSY, HSQC): δ 8.00 – 7.91 (m, 2H, CH_{arom}), 7.61 – 7.47 (m, 6H, CH_{arom}), 7.43 (t, J = 7.8 Hz, 2H, CH_{arom}), 7.38 – 7.33 (m, 2H, CH_{arom}), 7.32 – 7.28 (m, 1H, CH_{arom}), 7.23 – 7.12 (m, 13H, CH_{arom}), 5.66 (t, J = 9.6 Hz, 1H, H-4), 5.08 (d, J = 11.5 Hz, 1H, C*H*H Bn), 4.87 (d, J = 11.6 Hz, 1H, CH*H* Bn), 4.84 (d, J = 1.1 Hz, 1H, H-1), 4.63 (d, J = 12.2 Hz, 1H, C*H*H Bn), 4.52 – 4.48 (m, 2H, C*H*H Bn, CH*H* Bn), 4.43 (d, J = 11.4 Hz, 1H, CH*H* Bn), 4.22 (dd, J = 3.0, 1.1 Hz, 1H, H-2), 3.83 – 3.75 (m, 2H, H-5, H-6), 3.74 – 3.66 (m, 2H, H-3, H-6); ^{13}C NMR (101 MHz, $CDCl_3$, HSQC): δ 165.7 (C=O Bz), 138.3, 138.0, 137.5, 135.5 (C_q), 133.3 , 130.8, 129.9 (CH_{arom}), 129.8 (C_q), 129.0, 128.7, 128.5, 128.5, 128.4, 128.3, 128.0, 127.9, 127.9, 127.7, 127.5, 127.3 (CH_{arom}), 87.9 (C-1), 80.9 (C-3), 78.9 (C-5), 76.5 (C-2), 75.1, 73.7, 72.2 (CH_2 Bn), 70.6 (C-6), 69.5 (C-4); HRMS: $[M+NH_4]^+$ calcd for $C_{40}H_{42}NO_6S$ 664.27274, found 669.27235.

Preparation of Donor 15

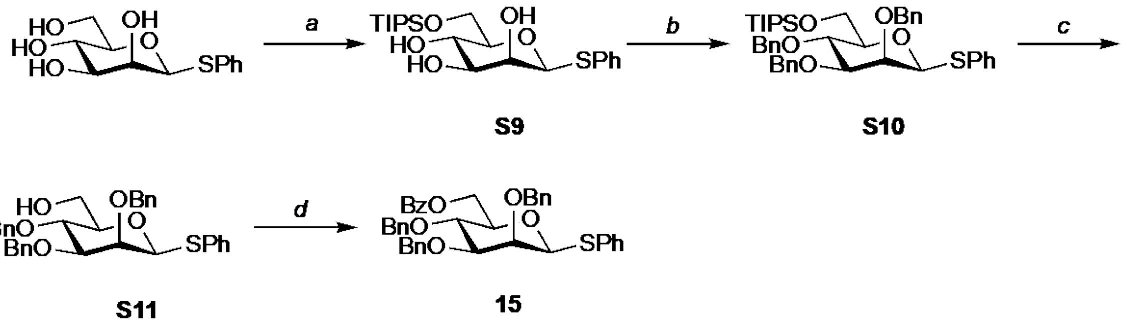

Supplementary Figure 29. Donor **15** synthesis. *Reagents and conditions:* a) TIPS-Cl, imidazole, **S10**: 83%; b) BnBr, NaH, DMF, **S9**: 81%; c) TFA, THF, water, **S11**: 93%; d) BzCl, pyridine, **15**: 78%.

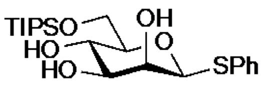

Phenyl 6-*O*-triisopropylsilyl-1-thio-β-D-mannopyranoside (S9). Phenyl 1-thio-β-D-mannopyranoside[17] (5.45 g, 20 mmol) was dissolved in DMF after which imidazole (3.4 g, 50 mmol, 2.5 eq) and triisopropylsilyl chloride (5.4 mL, 25 mmol, 1.25 eq) were added. After reacting overnight, excess reagent was destroyed by the addition of 7.5 mL methanol. After stirring for an additional 30 min, the reaction mixture was concentrated under reduced pressure, dissolved in diethyl ether and washed with water. The organic phase was dried with $MgSO_4$ and

concentrated, the residue was purified over silica (20% acetone in pentane) yielding the title compound (6.29 g, 14.7 mmol, 73%) as yellowish oil. TLC: R_f 0.40, (pentane:acetone, 60:40, v:v); $[\alpha]_D^{25}$ −83.6° (*c* 0.73, $CHCl_3$); IR (thin film, cm^{-1}): 688, 734, 882, 946, 1264, 2865 ; ^1H NMR (400 MHz, $CDCl_3$, HH-COSY, HSQC): δ 7.55 – 7.40 (m, 2H, CH_{arom}), 7.30 – 7.22 (m, 3H, CH_{arom}), 4.87 (d, *J* = 1.1 Hz, 1H, H-1), 4.21 (t, *J* = 4.3 Hz, 1H, H-2), 4.07 – 3.98 (m, 2H, 2x H-6), 3.96 (d, *J* = 2.0 Hz, 1H, 3-OH), 3.91 – 3.81 (m, 2H, H-4, 4-OH), 3.65 (ddd, *J* = 9.1, 5.5, 3.3 Hz, 1H, H-3), 3.45 – 3.35 (m, 2H, H-5, 2-OH), 1.15 – 1.02 (m, 21H, 3x CH TIPS, 6x CH_3 TIPS); ^{13}C NMR (101 MHz, $CDCl_3$, HSQC): δ 134.8 (C_q), 131.0, 129.1, 127.4 (CH_{arom}), 87.3 (C-1), 78.7 (C-5), 75.1 (C-3), 72.2 (C-2), 70.8 (C-4), 65.5 (C-6), 18.0 (CH_3 TIPS), 11.8 (CH TIPS); HRMS: $[M+Na]^+$ calcd for $C_{21}H_{36}NaO_5SSi$ 451.19449, found 451.19430.

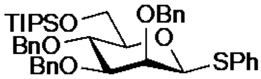

Phenyl 6-*O*-triisopropylsilyl-2,3,4-tri-*O*-benzyl-1-thio-β-D-mannopyranoside (S10). S9 (4.07 g, 9.50 mmol) was dissolved in DMF and cooled to 0 °C, after which benzyl bromide (4.2 mL, 35.6 mmol, 3.75 eq) and NaH (60% dispersion in mineral oil, 1.43 g, 35.6 mmol, 3.75 eq) were added after which the reaction mixture was slowly allowed to warm to RT. When TLC showed full conversion, the reaction was quenched with water. The aqueous phase was extracted twice with diethyl ether, combined organic phases were dried with sodium sulfate and concentrated under reduced pressure. The residue was purified over silica (10% diethyl ether in pentane) to yield the title compound (5.40 g, 7.7 mmol, 81%) as waxy white solid. TLC: R_f 0.40, (pentane:Et_2O, 85:15, v:v); $[\alpha]_D^{25}$ −30.8° (*c* 0.59, $CHCl_3$); IR (thin film, cm^{-1}):695, 732, 1062, 1088, 1134, 1453; ^1H NMR (400 MHz, $CDCl_3$, HH-COSY, HSQC): δ 7.54 – 7.46 (m, 4H, CH_{arom}), 7.38 – 7.24 (m, 13H, CH_{arom}), 7.24 – 7.16 (m, 3H, CH_{arom}), 5.05 (d, *J* = 11.4 Hz, 1H, C*H*H Bn), 4.90 (d, *J* = 11.0 Hz, 1H, C*H*H Bn), 4.84 (d, *J* = 11.4 Hz, 1H, CH*H* Bn), 4.78 – 4.72 (m, 2H, H-1, C*H*H Bn), 4.69 (d, *J* = 11.8 Hz, 1H, CH*H* Bn), 4.65 (d, *J* = 11.0 Hz, 1H, CH*H* Bn), 4.13 (dd, *J* = 3.0, 1.1 Hz, 1H, H-2), 3.99 (dd, *J* = 10.9, 1.8 Hz, 1H, H-6), 3.92 (t, *J* = 8.2 Hz, 1H, H-4), 3.88 (dd, *J* = 9.6, 4.9 Hz, 1H, H-6), 3.63 (dd, *J* = 9.5, 3.0 Hz, 1H, H-3), 3.38 (ddd, *J* = 9.7, 6.2, 1.8 Hz, 1H, h-5), 1.44 – 0.80 (m, 21H, 6x CH_3 TIPS, 3x CH TIPS); ^{13}C NMR (101 MHz, $CDCl_3$, HSQC): δ 138.6, 138.4, 138.3, 136.5 (C_q), 130.4, 128.9, 128.6, 128.5, 128.5, 128.3, 128.2, 128.1, 127.9, 127.9, 127.7, 127.6, 126.9 (CH_{arom}), 87.9 (C-1), 84.4 (C-3), 81.8 (C-5), 78.0 (C-2), 75.4, 75.1 (CH_2 Bn), 74.9 (C-4), 72.7 (CH_2 Bn), 63.4 (C-6), 18.2, 18.1 (CH_3 TIPS), 12.1 (CH TIPS); HRMS: $[M+NH_4]^+$ calcd for $C_{42}H_{58}NO_5SSi$ 716.37995, found 716.37934.

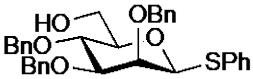

Phenyl 2,3,4-tri-*O*-benzyl-1-thio-β-D-mannopyranoside (S11). S10 (5.24 g, 7.50 mmol) was dissolved in 30 mL THF, to which 10 mL and 10 mL TFA were added. The reaction mixture was stirred at room temperature until TLC showed full conversion. The solution was diluted with water, neutralised with 20 g K_2CO_3, partially concentrated to remove organic solvents and extracted twice with ethyl acetate. Combined organic phases were dried and concentrated under reduced pressure. The residue was purified over silica (15% acetone in pentane), yielding the title compound (3.77 g, 7.0 mmol, 93%) as colourless oil. TLC: R_f 0.35, (pentane:acetone, 80:20, v:v); $[\alpha]_D^{25}$ −19.7° (*c* 0.67, $CHCl_3$); IR (thin film, cm^{-1}): 695, 732, 1026, 1072, 1452; ^1H NMR (400 MHz, $CDCl_3$, HH-COSY, HSQC): δ 7.50 – 7.23 (m, 20H, CH_{arom}), 5.05 (d, *J* = 11.3 Hz, 1H, C*H*H Bn), 4.91 (d, *J* = 10.9 Hz, 1H, C*H*H Bn), 4.83 (d, *J* = 11.3 Hz, 1H, CH*H* Bn), 4.80 (d, *J* = 1.1 Hz, 1H, H-1), 4.78 – 4.68 (m, 2H, CH_2 Bn), 4.66 (d, *J* = 10.9 Hz, 1H, CH*H* Bn), 4.14 (dd, *J* = 2.9, 1.2 Hz, 1H, H-2), 3.99 (t, *J* = 9.5 Hz, 1H, H-4), 3.87 (ddd, *J* = 12.0, 6.9, 2.9 Hz, 1H, H-6), 3.74 (ddd, *J* = 12.1, 7.0, 5.7 Hz, 1H, H-6), 3.65 (dd, *J* = 9.5, 2.9 Hz, 1H, H-3), 3.37 (ddd, *J* = 9.6, 5.7, 2.9 Hz, 1H, H-5), 2.22 (t, *J* = 6.9 Hz, 1H, OH); ^{13}C NMR (101 MHz, $CDCl_3$, HSQC): δ 138.2, 138.1, 138.1, 135.2 (C_q), 130.6, 129.1,

128.6, 128.5, 128.4, 128.3, 128.3, 128.0, 127.9, 127.8, 127.7, 127.4 (CH_{arom}), 87.8 (C-1), 84.2 (C-3), 80.1 (C-5), 77.7 (C-2), 75.4, 75.4 (CH_2 Bn), 74.8 (C-4), 72.7 (CH_2 Bn), 62.6 (C-6); HRMS: $[M+Na]^+$ calcd for $C_{33}H_{34}NaO_5S$ 565.20192, found 565.20152.

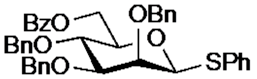

phenyl 6-benzoyl-2,3,4-tri-O-benzyl-1-thio-β-D-mannopyranoside (15). **S11** (2.98 g, 5.50 mmol) was dissolved in 10 mL pyridine, to which benzoyl chloride (0.96 mL, 8.25 mmol, 1.5 eq) was added. When TLC showed full conversion of the starting material, the reaction mixture was diluted with ethyl acetate and washed twice with 1 M aq. HCl and with sat. aq. $NaHCO_3$. The organic phase was dried and concentrated under reduced pressure. The residue was purified by recrystallization from ethyl acetate/pentane, obtaining the title compound (2.79 g, 4.3 mmol, 78%) as fluffy white solid. (melting point: 137 °C); TLC: R_f 0.20, (pentane:EtOAc, 90:10, v:v); $[\alpha]_D^{25}$ – 44.1° (*c* 0.85, $CHCl_3$); IR (thin film, cm^{-1}): 697, 713, 1027, 1070, 1090, 1120, 1274, 1720; ^1H NMR (400 MHz, $CDCl_3$, HH-COSY, HSQC): δ 8.05 – 8.00 (m, 2H, CH_{arom}), 7.59 – 7.53 (m, 1H, CH_{arom}), 7.52 – 7.47 (m, 4H, CH_{arom}), 7.42 – 7.26 (m, 14H, CH_{arom}), 7.21 – 7.08 (m, 3H, CH_{arom}), 5.07 (d, *J* = 11.4 Hz, 1H, C*H*H Bn), 4.95 (d, *J* = 10.9 Hz, 1H, C*H*H Bn), 4.87 (d, *J* = 11.3 Hz, 1H, CH*H* Bn), 4.79 (d, *J* = 1.1 Hz, 1H, H-1), 4.77 (d, *J* = 12.0 Hz, 1H, C*H*H Bn), 4.75 – 4.69 (m, 2H, H-6, CH*H* Bn), 4.66 (d, *J* = 10.9 Hz, 1H, CH*H* Bn), 4.39 (dd, *J* = 11.7, 7.5 Hz, 1H, H-6), 4.19 (dd, *J* = 2.9, 1.1 Hz, 1H, H-2), 4.00 (t, *J* = 9.5 Hz, 1H, H-4), 3.74 – 3.67 (m, 2H, H-3, H-5); ^{13}C NMR (101 MHz, $CDCl_3$, HSQC): δ 166.4 (C=O Bz), 138.3, 137.9, 137.9, 135.6 (C_q), 133.1, 130.8 CH_{arom}), 130.2 (C_q), 129.9, 128.9, 128.7, 128.6, 128.4, 128.4, 128.3, 128.1, 128.0, 127.8, 127.8, 127.2 (CH_{arom}), 87.8 (C-1), 84.3 (C-3), 77.9 (C-5), 77.6 (C-2), 75.5, 75.3 (CH_2 Bn), 74.8 (C-4), 72.7 (CH_2 Bn), 64.5 (C-6); HRMS: $[M+Na]^+$ calcd for $C_{40}H_{38}NaO_6S$ 669.22813, found 669.22749.

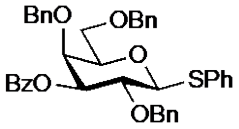

Phenyl 3-*O*-benzoyl-2,4,6-tri-*O*-benzyl-1-thio-β-D-galactopyranoside (16). Compound was prepared according to literature procedure.[14] ^1H NMR (400 MHz, $CDCl_3$, HH-COSY, HSQC): δ 7.99 – 7.92 (m, 2H, CH_{arom}), 7.62 – 7.57 (m, 2H, CH_{arom}), 7.57 – 7.53 (m, 1H, CH_{arom}), 7.43 – 7.38 (m, 2H, CH_{arom}), 7.34 – 7.19 (m, 13H, CH_{arom}), 7.16 – 7.13 (m, 5H, CH_{arom}), 5.28 (dd, *J* = 9.6, 3.0 Hz, 1H, H-3), 4.80 (d, *J* = 10.6 Hz, 1H, C*H*H Bn), 4.77 (d, *J* = 9.6 Hz, 1H, H-1), 4.67 (d, *J* = 11.5 Hz, 1H, C*H*H Bn), 4.59 (d, *J* = 10.5 Hz, 1H, CH*H* Bn), 4.54 – 4.46 (m, 2H, C*H*H Bn, CH*H* Bn), 4.43 (d, *J* = 11.7 Hz, 1H, CH*H* Bn), 4.16 (dd, *J* = 3.1, 1.0 Hz, 1H, H-4), 4.11 (t, *J* = 9.7 Hz, 1H, H-2), 3.84 (ddd, *J* = 7.0, 5.7, 1.0 Hz, 1H, H-5), 3.72 – 3.63 (m, 2H, 2x H-6); ^{13}C NMR (101 MHz, $CDCl_3$) δ 165.8 (C=O Bz), 138.2, 137.9, 137.8, 134.0 (C_q), 133.4, 131.6, 129.9 (CH_{arom}), 129.7 (C_q), 129.0, 128.6, 128.5, 128.3, 128.3, 128.3, 127.9, 127.9, 127.8, 127.7, 127.3 (CH_{arom}), 87.8 (C-1), 77.6 (C-3), 77.1 (C-5), 75.5 (C-2), 75.5 (CH_2 Bn), 75.0 (CH_2 Bn), 74.7 (C-4), 73.6 (CH_2 Bn), 68.4 (C-6); HRMS: $[M+NH_4]^+$ calcd for $C_{40}H_{42}NO_6S$ 664.27274, found 664.27236.

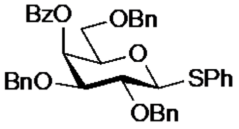

Phenyl 4-*O*-benzoyl-2,3,6-tri-*O*-benzyl-1-thio-β-D-galactopyranoside (17). The title compound was prepared according to literature procedure.[14] ^1H NMR (400 MHz, $CDCl_3$, HH-COSY, HSQC): δ 8.02 – 7.96 (m, 2H, CH_{arom}), 7.66 – 7.63 (m, 2H, CH_{arom}), 7.62 – 7.56 (m, 1H, CH_{arom}), 7.49 – 7.42 (m, 2H, CH_{arom}), 7.41 – 7.25 (m, 15H, CH_{arom}), 7.23 – 7.20 (m, 4H, CH_{arom}), 5.89 (dd, *J* = 3.1, 1.0 Hz, 1H, H-4), 4.85 (d, *J* = 11.1 Hz, 1H, C*H*H Bn), 4.73 (s, 2H, CH_2

Bn), 4.70 (d, *J* = 9.4 Hz, 1H, H-1), 4.55 – 4.49 (m, 2H, C*H*H Bn, CH*H* Bn), 4.44 (d, *J* = 11.7 Hz, 1H, CH*H* Bn), 3.89 (td, *J* = 6.4, 5.9, 1.0 Hz, 1H, H-5), 3.76 (dd, *J* = 9.1, 3.1 Hz, 1H, H-3), 3.72 – 3.66 (m, 2H, H-2, H-6), 3.58 (dd, *J* = 9.6, 6.8 Hz, 1H, H-6); ^{13}C NMR (101 MHz, $CDCl_3$, HSQC): δ 165.8 (C=O Bz), 138.4, 137.7 (C_q), 133.3, 133.0 (CH_{arom}), 130.1 (C_q), 129.9, 129.0, 128.5, 128.5, 128.5, 128.4, 128.3, 128.0, 127.9, 127.9, 127.8 (CH_{arom}), 87.3 (C-1), 81.6 (C-3), 76.6 (C-2), 76.4 (C-5), 75.8, 73.8, 71.9 (CH_2 Bn), 68.5 (C-6), 67.4 (C-4); HRMS: $[M+NH_4]^+$ calcd for $C_{40}H_{42}NO_6S$ 664.27274, found 664.27250.

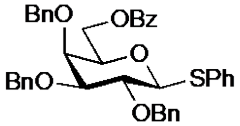

Phenyl 6-*O*-benzoyl-2,3,4-tri-*O*-benzyl-1-thio-β-D-galactopyranoside (18). Phenyl 2,3,4-tri-*O*-benzyl-1-thio-β-D-galactopyranoside[18] (1.45 g, 2.67 mmol) was dissolved in 10 mL pyridine after which benzoyl chloride (0.47 mL, 4.01 mmol, 1.5 eq) was added. When TLC shows full conversion, the reaction mixture was diluted with ethyl acetate and washed with 1M aq. HCl and sat. aq. $NaHCO_3$. The organic phase was dried and concentrated. The residue was purified over silica (10% ethyl acetate in pentane) yielding the title compound (1.28 g, 2.0 mmol, 74%) as white amorphous solid. TLC: R_f 0.30, (pentane:EtOAc, 90:10, v:v); $[\alpha]_D^{25}$ –18.1° (*c* 0.42, $CHCl_3$); IR (thin film, cm^{-1}): 698, 713, 736, 1026, 1070, 1271, 1452, 1718 ; ^{1}H NMR (400 MHz, $CDCl_3$, HH-COSY, HSQC) δ 8.00 – 7.92 (m, 2H, CH_{arom}), 7.56 (ddt, *J* = 6.7, 3.7, 1.7 Hz, 3H, CH_{arom}), 7.47 – 7.22 (m, 17H, CH_{arom}), 7.19 – 7.05 (m, 3H, CH_{arom}), 5.02 (d, *J* = 11.6 Hz, 1H, C*H*H Bn), 4.86 (d, *J* = 10.2 Hz, 1H, C*H*H Bn), 4.82 – 4.72 (m, 3H, C*H*H Bn, 2x CH*H* Bn), 4.71 – 4.63 (m, 2H, H-1, CH*H* Bn) 4.51 (dd, *J* = 11.3, 7.3 Hz, 1H, H-6), 4.37 (dd, *J* = 11.3, 5.2 Hz, 1H, H-6), 3.98 (t, *J* = 9.5 Hz, 1H, H-2), 3.90 (d, *J* = 2.8 Hz, 1H, H-4), 3.74 (dd, *J* = 7.2, 5.4 Hz, 1H, H-5), 3.63 (dd, *J* = 9.2, 2.8 Hz, 1H, H-3); ^{13}C NMR (101 MHz, $CDCl_3$, HSQC) δ 166.2 (C=O), 138.3, 138.2, 134.4 (C_q), 133.2, 131.5 (CH_{arom}), 129.8 (C_q), 128.8, 128.6, 128.5, 128.4, 128.4, 128.2, 127.9, 127.8, 127.7, 127.2 (CH_{arom}), 88.1 (C-1), 84.2 (C-3), 77.6 (C-2), 76.2 (C-5), 75.8, 74.5 (CH_2 Bn), 73.6 (C-4), 73.3 (CH_2 Bn), 64.0 (C-6); HRMS: $[M+NH_4]^+$ calcd for $C_{40}H_{42}NO_6S$ 664.27274, found 664.27231.

Ac/Me-*O*-protected glycosyl donors

Preparation of Donors 1-3

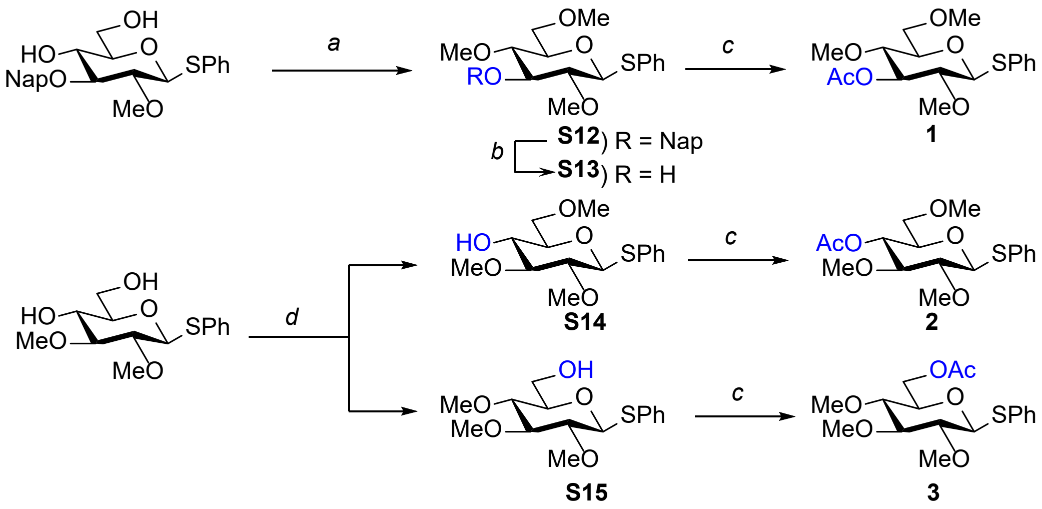

Supplementary Figure 30. Synthesis of mono-acetyl glucosides **1-3**. Reagents and conditions: a) MeI, NaH, DMF, **S12**: 89%; b) DDQ, H_2O/DCM, **S13**: 81%; c) Ac_2O, Pyridine, **1**: 97%, **2**: 57%. **3**: 97%; d) MeI, NaH, DMF, **S14**: 21%, **S15**: 32%.

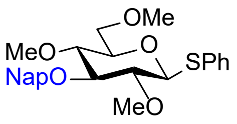

Phenyl 3-*O*-(2-methylnaphthyl)-2,4,6-tri-*O*-methyl-1-thio-β-D-glucopyranose S12: *Via* general methylation protocol starting with phenyl 3-*O*-(2-methylnaphthyl)-2-*O*-methyl-1-thio-β-D-glucopyranose[19] (85 mg, 0.21 mmol). The residue was purified by crystallization (MeOH) to afford the product **S12** (83 mg, 89%) as white needles; TLC: (EtOAc/n-heptane, 1/4, v/v): R_f = 0.23; ^{1}H NMR (400 MHz, Chloroform-*d*) δ 7.83 (dd, *J* = 6.0, 3.5 Hz, 4H, 4 x C*H* Ar), 7.65 – 7.38 (m, 5H, 5 x C*H* Ar), 7.36 – 7.15 (m, 3H, 3 x C*H* Ar), 5.03 (d, *J* = 11.2 Hz, 1H, C*H*H Nap), 4.99 (d, *J* = 11.2 Hz, 1H, CH*H* Nap), 4.53 (d, *J* = 9.8 Hz, 1H, H-1), 3.66 (dd, *J* = 10.9, 1.8 Hz, 1H, H-6a), 3.63 (s, 3H, -OC*H*3), 3.62 – 3.50 (m, 5H, H-6b, H-3, -OC*H*3), 3.40 (s, 3H, -OC*H*3), 3.37 – 3.26 (m, 2H, H-5, H-4), 3.18 (dd, *J* = 9.8, 8.8 Hz, 1H, H-2); ^{13}C NMR (101 MHz, Chloroform-*d*) δ 136.2, 134.1, 133.5, 133.1, 131.9, 129.0, 128.2, 128.1, 127.8, 127.5, 126.7, 126.2, 126.2, 126.0, 87.7 (C-1), 86.8 (C-3), 82.9 (C-2), 79.6 (C-4), 79.1 (C-5), 75.7, 71.5 (C-6), 61.2, 60.8, 59.5; HRMS *(m/z)*: $[M+Na]^+$ calcd for $C_{26}H_{30}O_5S$, 477.1712; found, 477.1694.

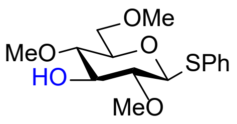

Phenyl 2,4,6-tri-*O*-methyl-1-thio-β-D-glucopyranose S13: To a well stirred emulsion of **S12** (75 mg, 0.16 mmol) in DCM and H_2O (7/1, v/v, 1.6 mL) was added DDQ (56 mg, 0.25 mmol) and the suspension was protected from light and stirred at room temperature for 1.5h. The mixture was diluted with DCM (20 mL) and washed (2 x 10 mL) with 10% $Na_2S_3O_3$ in H_2O w/w, to reduce the remaining DDQ. The organic layer was dried over $MgSO_4$, filtered and concentrated *in vacuo*. Column chromatography of the residue afforded **S13** as white solid (42 mg, 81%); TLC: (EtOAc/n-heptane, 2/3, v/v): R_f = 0.35; ^{1}H NMR (500 MHz, Chloroform-*d*) δ 7.59 – 7.44 (m, 2H, 2 x C*H* Ar), 7.39 – 7.20 (m, 3H, 3 x C*H* Ar), 4.60 – 4.48 (m, 1H, H-1), 3.68 – 3.56 (m, 6H, H-6a, H-6b, -OC*H*3, H-3), 3.56 (s, 3H, -OC*H*3), 3.40 (s, 3H, -OC*H*3), 3.33 (ddd, *J* = 9.7, 4.5, 2.0 Hz, 1H, H-5), 3.26 – 3.20 (m, 1H, H-4), 3.08 (dd, *J* = 9.7, 8.8 Hz, 1H, H-2), 2.67 (s, 1H, -OH); ^{13}C NMR (126 MHz, $CDCl_3$) δ 134.1, 132.0, 131.8, 129.1, 129.0, 127.5, 87.2 (C-1), 82.5 (C-2), 79.1 (C-4), 78.8 (C-5), 78.6 (C-3), 71.6 (C-6), 62.4, 61.2, 60.7, 59.5; HRMS *(m/z)*: $[M+Na]^+$ calcd for $C_{15}H_{22}O_5S$, 337.1086; found, 337.1086.

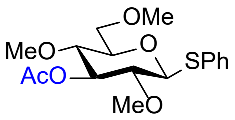

Phenyl 3-*O*-acetyl-2,4,6-tri-*O*-methyl-1-thio-β-ᴅ-glucopyranose 1: *Via* general acetylation protocol starting with **S13** (40 mg, 0.13 mmol). The product **1** (44 mg, 97%) was obtained as a pale oil; TLC: (EtOAc/n-heptane, 2/3, v/v): R_f = 0.49; ^{1}H NMR (500 MHz, Chloroform-*d*) δ 7.60 – 7.47 (m, 2H, 2 x C*H* Ar), 7.38 – 7.20 (m, 4H, 3 x C*H* Ar), 5.13 (t, *J* = 9.0 Hz, 1H, H-3), 4.58 (d, *J* = 9.8 Hz, 1H, H-1), 3.67 – 3.57 (m, 3H, H-6a, H-6b), 3.50 (s, 3H, -OCH_3), 3.42 (s, 3H, -OCH_3), 3.40 (s, 3H, -OCH_3), 3.38 (dd, *J* = 3.8, 1.8 Hz, 1H, H-5), 3.37 – 3.32 (m, 1H, H-4), 3.16 (t, *J* = 9.5 Hz, 1H, H-2), 2.14 (s, 3H, -CH_3 Ac) ; ^{13}C NMR (126 MHz, $CDCl_3$) δ 170.1, 133.7, 132.2, 129.0, 128.9, 127.7, 87.4 (C-1), 80.6 (C-2), 78.7 (C-5), 77.5 (C-4), 77.5 (C-3), 71.2 (C-6), 60.3, 60.1, 59.5, 21.2; HRMS *(m/z)*: $[M+Na]^+$ calcd for $C_{17}H_{24}O_6S$, 379.1191; found, 379.1205.

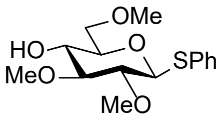

Phenyl 2,3,6-tri-*O*-methyl-1-thio-β-ᴅ-glucopyranoside S14: To a solution of phenyl 2,3-di-*O*-methyl-1-thio-β-ᴅ-glucopyranoside[20] (100 mg, 0.33 mmol) in DMF (3.0 mL), NaH (33 mg, 0.83 mmol, 60 wt% in mineral oil) was added. The mixture was allowed to stir for 5 minutes before MeI (21 µL, 0.33 mmol) was added and was left at room temperature for 1 hour under argon. The reaction was quenched by addition of sat. aq. NH_4Cl (0.5 mL), diluted with EtOAc (15 mL) and washed once with water (10 mL) and once with brine. The organic layer was dried ($MgSO_4$), filtered and concentrated *in vacuo*. Silica column chromatography of the crude (30% EtOAc in *n*-heptane) afforded Phenyl 2,3,6-tri-*O*-methyl-1-thio-β-ᴅ-glucopyranoside **S14** (34 mg, 32%) as a clear oil. Phenyl 2,3,4-tri-*O*-methyl-1-thio-β-ᴅ-glucopyranoside (22 mg, 21%) was obtained as main byproduct; TLC: (EtOAc/n-heptane, 1/1, v/v): R_f = 0.35; ^{1}H NMR (400 MHz, Chloroform-*d*) δ 7.68 – 7.40 (m, 2H, 2 x C*H* Ar), 7.40 – 7.13 (m, 3H, 3 x C*H* Ar), 4.55 (d, *J* = 9.7 Hz, 1H, H-1), 3.66 (pd, *J* = 4.1 Hz, 5H, H-6A, H-6B, -OCH_3), 3.61 (s, 3H, -OCH_3), 3.57 – 3.49 (m, 1H, H-4), 3.48 – 3.34 (m, 4H, -OCH_3, H-5), 3.18 (t, *J* = 8.8 Hz, 1H, H-3), 3.08 (dd, *J* = 9.7, 8.7 Hz, 1H, H-2), 2.83 (d, *J* = 2.1 Hz, 1H, 4-O*H*); ^{13}C NMR (101 MHz, Chloroform-*d*) δ 133.9, 132.0, 129.0, 128.6, 88.0 (C-3), 87.7 (C-1), 82.5 (C-2), 77.8 (C-5), 73.0 (C-6), 71.7 (C-4), 61.2, 60.8, 59.7; HRMS *(m/z)*: $[M+Na]^+$ calcd for $C_{15}H_{22}O_5S$, 337.1086; found, 337.1084.

Phenyl 2,3,4-tri-*O*-methyl-1-thio-β-ᴅ-glucopyranoside S15: Afforded as main byproduct in the synthesis of

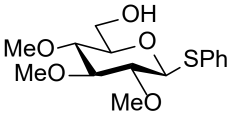

phenyl 2,3,6-tri-*O*-methyl-1-thio-β-ᴅ-glucopyranoside. TLC: (EtOAc/n-heptane, 1/1, v/v): R_f = 0.44; ^{1}H NMR (400 MHz, Chloroform-*d*) δ 7.53 – 7.46 (m, 2H, 2 x C*H* Ar), 7.35 – 7.24 (m, 3H, 3 x C*H* Ar), 4.55 (d, *J* = 9.8 Hz, 1H, H-1), 3.92 – 3.78 (m, 1H, H-6A), 3.74 – 3.64 (m, 4H, H-6B, -OCH_3), 3.62 (s, 3H, -OCH_3), 3.55 (s, 3H, -OCH_3), 3.29 – 3.20 (m, 2H, H-5, H-3), 3.17 – 3.08 (m, 1H, H-4), 3.03 (dd, *J* = 9.8, 8.6 Hz, 1H, H-4), 1.92 (t, *J* = 6.7 Hz, 1H, 6-O*H*); ^{13}C NMR (101 MHz, Chloroform-*d*) δ 132.0, 129.1, 127.8, 88.6 (C-3), 87.2 (C-1), 82.8 (C-2), 79.7 (C-4), 79.2 (C-5), 62.4 (C-6), 61.1, 61.0, 60.7; HRMS *(m/z)*: $[M+Na]^+$ calcd for $C_{15}H_{22}O_5S$, 337.1086; found, 337.1083.

Phenyl 4-*O*-acetyl-2,3,6-tri-*O*-methyl-1-thio-β-ᴅ-glucopyranoside 2: *Via* general acetylation protocol starting

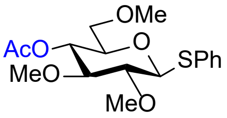

with **S14** (20 mg, 0.064 mmol). **2** (13 mg, 57%) was obtained as a white amorphous solid. TLC: (EtOAc/n-heptane, 2/3, v/v): R_f = 0.49; ^{1}H NMR (400 MHz, Chloroform-*d*) δ 7.65 – 7.47 (m, 1H), 7.41 – 7.19 (m, 2H, 2 x C*H* Ar), 4.85 (t, *J* = 9.6 Hz, 1H, H-4), 4.53 (d, *J* = 9.8 Hz, 1H, H-1), 3.59 (s, 3H, -OCH_3), 3.54 (s, 3H, -OCH_3), 3.50 (ddd, *J* = 9.8, 5.9, 3.2 Hz, 1H, H-5), 3.46 – 3.42 (m, 2H, H-6A, H-6B), 3.35 – 3.26 (m, 4H, H-3, -OCH_3), 3.13 (dd, *J* = 9.8, 8.7 Hz, 1H, H-2), 2.09 (s, 1H, -CH_3 Ac); ^{13}C NMR (101 MHz, Chloroform-*d*) δ 170.0, 132.2, 129.0, 127.7, 87.4 (C-1), 86.0 (C-3), 82.2 (C-2), 77.5 (C-5), 72.2 (C-6), 70.9 (C-4), 61.0, 60.9, 59.6, 21.1; HRMS *(m/z)*: $[M+Na]^+$ calcd for $C_{17}H_{24}O_6S$, 379.1191; found, 379.1193.

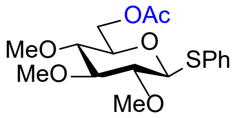

Phenyl 6-*O*-acetyl-2,3,4-tri-*O*-methyl-1-thio-β-D-glucopyranoside 3: *Via* general acetylation protocol starting with **S15** (33 mg, 0.10 mmol). **3** (36 mg, 96%) was obtained as a white amorphous solid. TLC: (EtOAc/n-heptane, 2/3, v/v): R_f = 0.50; ^{1}H NMR (400 MHz, Chloroform-*d*) δ 7.66 – 7.50 (m, 2H, 2 x C*H* Ar), 7.41 – 7.20 (m, 3H, 3 x C*H* Ar), 4.49 (d, *J* = 9.8 Hz, 1H, H-1), 4.35 (dd, *J* = 11.8, 2.2 Hz, 1H, H-6A), 4.20 (dd, *J* = 11.8, 6.2 Hz, 1H, H-6B), 3.65 (s, 3H, -OC*H*3), 3.61 (s, 3H, -OC*H*3), 3.52 (s, 3H, -OC*H*3), 3.41 (ddd, *J* = 9.9, 6.2, 2.1 Hz, 1H, H-5), 3.24 (t, *J* = 8.8 Hz, 1H, H-3), 3.06 (pddd, *J* = 9.9, 8.8, 7.9 Hz, 2H, H-4, H-2), 2.08 (s, 3H, -C*H*3 Ac); ^{13}C NMR (101 MHz, Chloroform-*d*) δ 170.9, 133.7, 132.1, 128.9, 127.7, 88.7 (C-3), 87.2 (C-1), 82.7 (C-2), 79.9 (C-4), 76.9 (C-5), 63.7 (C-6), 61.2, 61.0, 60.8, 21.0; HRMS *(m/z)*: [M+Na]$^+$ calcd for $C_{17}H_{24}O_6S$, 379.1191; found, 379.1198.

Preparation of Donor 4-6

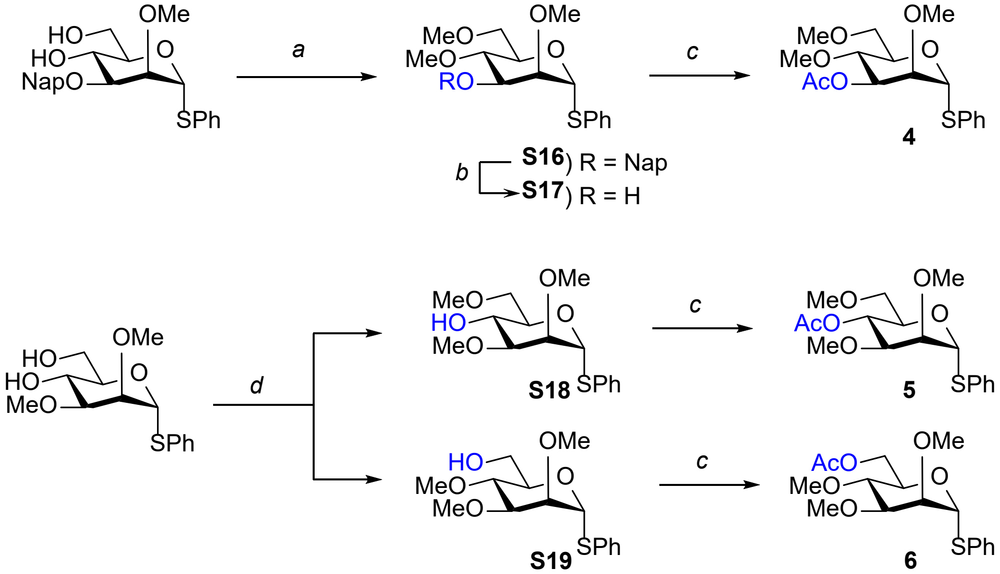

Supplementary Figure 31. Synthesis of mono-acetyl mannosides **4-6**. Reagents and conditions: a) MeI, NaH, DMF, **S16**: 94%; b) DDQ, H_2O/DCM, **S17**: 86%; c) Ac_2O, Pyridine, **4**: 98%, **5**: 97%, **6**: *quant*.; d) MeI, NaH, DMF, **S18**: 11%, **S19**: 43%.

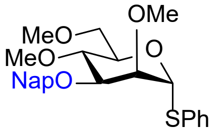

Phenyl 3-O-(2-methylnaphthyl)-2,4,6-tri-O-methyl-1-thio-α-D-mannoyranose S16: *Via* general methylation protocol starting with phenyl 3-*O*-(2-methylnaphthyl)-2-*O*-methyl-1-thio-α-D-mannopyranose[19] (350 mg, 0.821 mmol). The residue was purified by silica column chromatography (20% EtOAc in toluene) to afford the product **S19** (352 mg, 94%) as a white amorphous solid. TLC: (EtOAc/n-heptane, 1/4, v/v): R_f = 0.19; ^{1}H NMR (400 MHz, Chloroform-*d*) δ 7.97 – 7.78 (m, 4H,4 x C*H* Ar), 7.63 – 7.38 (m, 5H, 5 x C*H* Ar), 7.35 – 7.12 (m, 3H, 3 x C*H* Ar), 5.59 (d, *J* = 1.5 Hz, 1H, H-1), 4.92 (d, *J* = 12.2 Hz, 1H, C*H*H Nap), 4.87 (d, *J* = 12.2 Hz, 1H, C*H*H Nap), 4.09 (ddd, *J* = 9.7, 4.6, 1.9 Hz, 1H, H-5), 3.80 (dd, *J* = 9.4, 3.1 Hz, 1H, H-3), 3.76 – 3.63 (m, 3H, H-2, H-4, H-6A), 3.62 – 3.59 (m, 4H, -OC*H*3, H-6B), 3.43 (s, 3H, -OC*H*3), 3.38 (s, 1H, -OC*H*3); ^{13}C NMR (101 MHz, Chloroform-*d*) δ 135.9, 134.7, 133.5, 133.2, 131.2, 129.1, 128.3, 128.1, 127.9, 127.4, 126.7, 126.3, 126.1, 126.0, 85.1 (C-1), 80.0 (C-3), 79.8 (C-2), 76.5 (C-4), 72.7, 72.6 (C-5), 71.5 (C-6), 61.0, 59.3, 58.6; HRMS *(m/z)*: [M+Na]$^+$ calcd for $C_{26}H_{30}O_5S$, 477.1712; found, 477.1694.

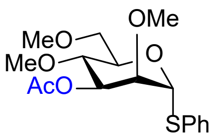

Phenyl 3-*O*-acetyl-2,4,6-tri-*O*-methyl-1-thio-α-D-mannopyranoside 4: To a well stirred emulsion of **S16** (330 mg, 0.73 mmol) in DCM and H_2O (7/1, v/v, 7.3 mL) was added DDQ (247 mg, 1.1 mmol) and the suspension was protected from light and stirred at room temperature for 1.5h. The mixture was diluted with DCM (100 mL) and washed (2 x 20 mL) with 10% $Na_2S_3O_3$ in H_2O w/w, to reduce the remaining DDQ. The organic layer was dried over $MgSO_4$, filtered and concentrated *in vacuo*. Column chromatography (30% EtOAc in n-heptane) of the residue afforded **S17** as a clear oil (0.196 mg, 86%). TLC: (EtOAc/n-heptane, 2/3, v/v): R_f = 0.13; HRMS *(m/z)*: $[M+Na]^+$ calcd for $C_{15}H_{22}O_5S$, 337.1086; found, 337.1106. **S17** was directly acetylated *via* general acetylation protocol starting with **S17** (189 mg, 0.60 mmol). The product **4** (210 mg, 98%) was obtained as a pale oil. TLC: (EtOAc/n-heptane, 1/1, v/v): R_f = 0.67; ^{1}H NMR (500 MHz, Chloroform-*d*) δ 7.55 – 7.45 (m, 2H, 2 x C*H* Ar), 7.33 – 7.28 (m, 3H, 3 x C*H* Ar), 5.60 (d, *J* = 1.9 Hz, 1H, H-1), 5.11 (dd, *J* = 9.6, 3.3 Hz, 1H, H-3), 4.16 (ddd, *J* = 9.8, 4.3, 2.0 Hz, 1H, H-5), 3.88 (dd, *J* = 3.3, 1.9 Hz, 1H, H-2), 3.75 (t, *J* = 9.7 Hz, 1H, H-4), 3.67 (dd, *J* = 10.8, 4.2 Hz, 1H, H-6A), 3.58 (dd, *J* = 10.7, 2.0 Hz, 1H, H-6B), 3.49 (s, 3H, -OC*H*3), 3.42 (s, 3H, -OC*H*3), 3.39 (s, 3H, -OC*H*3), 2.16 (s, 3H, C*H*3 Ac); ^{13}C NMR (126 MHz, Chloroform-*d*) δ 170.5, 134.6, 131.3, 129.2, 127.4, 84.9 (C-1), 79.9 (C-2), 74.7 (C-4), 74.0 (C-3), 72.3 (C-5), 71.2 (C-6), 60.6, 59.4, 58.6, 21.3; HRMS *(m/z)*: $[M+Na]^+$ calcd for $C_{17}H_{24}O_6S$, 379.1191; found, 379.1199.

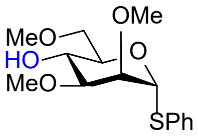

Phenyl 2,3,6-tri-*O*-methyl-1-thio-α-D-mannopyranoside S18: To a solution of phenyl 2,3-di-*O*-methyl-1-thio-α-D-mannopyranoside[21] (196 mg, 0.653 mmol) in DMF (6.5 mL), NaH (78 mg, 1.96 mmol, 60 wt% in mineral oil) was added. The mixture was allowed to stir for 5 minutes before MeI (41 µL, 0.653 mmol) was added and was left at room temperature for 1 hour under argon. The reaction was quenched by addition of sat. aq. NH_4Cl (0.5 mL), diluted with EtOAc (15 mL) and washed once with water (10 mL) and once with brine. The organic layer was dried ($MgSO_4$), filtered and concentrated *in vacuo*. Silica column chromatography of the crude (40% Et_2O in toluene) afforded phenyl 2,3,6-tri-*O*-methyl-1-thio-α-D-mannopyranoside **S22** (88 mg, 43%) as a clear oil. Phenyl 2,3,4-tri-*O*-methyl-1-thio-β-D-glucopyranoside (22 mg, 11%) was obtained as byproduct; TLC: (Et_2O/Toluene, 2/3, v/v): R_f = 0.28; ^{1}H NMR (500 MHz, Chloroform-*d*) δ 7.60 – 7.51 (m, 2H, 2 x C*H* Ar), 7.39 – 7.21 (m, 3H, 3 x C*H* Ar), 5.69 (d, *J* = 1.5 Hz, 1H, H-1), 4.24 (dt, *J* = 9.2, 4.3 Hz, 1H, H-5), 3.97 (t, *J* = 9.6 Hz, 1H, H-4), 3.91 (dd, *J* = 3.1, 1.6 Hz, 1H, H-2), 3.80 – 3.67 (m, 2H, H-6A, H-6B), 3.53 (s, 3H, -OC*H*3), 3.49 – 3.44 (m, 4H, -OC*H*3, H-3), 3.41 (s, 3H, -OC*H*3), 2.71 (s, 1H, 4-O*H*); ^{13}C NMR (126 MHz, Chloroform-*d*) δ 134.6, 131.4, 129.2, 127.6, 85.2 (C-1), 81.4 (C-3), 77.7 (C-2), 72.4 (C-5), 71.9 (C-6), 67.8 (C-4), 59.6, 58.3, 57.3; HRMS *(m/z)*: $[M+Na]^+$ calcd for $C_{15}H_{22}O_5S$, 337.1086; found, 337.1090.

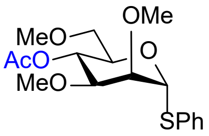

Phenyl 4-*O*-acetyl-2,3,6-tri-*O*-methyl-1-thio-α-D-mannopyranoside 5: *Via* general acetylation protocol starting with **S18** (80 mg, 0.25 mmoL). The title compound (88 mg, 97%) was obtained as a pale oil. TLC: (EtOAc/n-heptane, 1/1, v/v): R_f = 0.63; ^{1}H NMR (500 MHz, Chloroform-*d*) δ 7.63 – 7.49 (m, 2H, C*H* Ar), 7.46 – 7.15 (m, 3H, C*H* Ar), 5.63 (d, *J* = 1.9 Hz, 1H, H-1), 5.26 (t, *J* = 9.7 Hz, 1H, H-4), 4.30 (dt, *J* = 9.5, 4.5 Hz, 1H, H-5), 3.89 (dd, *J* = 3.1, 1.9 Hz, 1H, H-2), 3.56 (dd, *J* = 9.5, 3.1 Hz, 1H, H-3), 3.52 – 3.46 (m, 5H, H-6A, H-6B, -OC*H*3), 3.45 (s, 3H, -OC*H*3), 3.33 (s, 3H, -OC*H*3), 2.10 (s, 3H, C*H*3 Ac); ^{13}C NMR (126 MHz, Chloroform-*d*) δ 170.0, 134.3, 131.7, 129.2, 127.8, 85.3 (C-1), 79.2 (C-3), 78.3 (C-2), 72.0 (C-6), 71.0 (C-5), 68.9 (C-4), 59.5, 58.5, 57.9, 21.1; HRMS *(m/z)*: $[M+Na]^+$ calcd for $C_{17}H_{24}O_6S$, 379.1191; found, 379.1201.

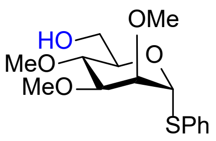

Phenyl 2,3,4-tri-*O*-methyl-1-thio-α-D-mannopyranoside S19: Isolated as main byproduct in the synthesis of Phenyl 2,4,6-tri-*O*-methyl-1-thio-α-D-mannopyranoside[22] TLC: (Et_2O/Toluene, 2/3, v/v): R_f = 0.25; ^1H NMR (500 MHz, Chloroform-*d*) δ 7.51 – 7.40 (m, 2H, 2 x C*H* Ar), 7.40 – 7.16 (m, 3H, 3 x C*H* Ar), 5.61 (d, *J* = 1.8 Hz, 1H, H-1), 4.04 (ddd, *J* = 9.5, 4.7, 2.9 Hz, 1H, H-5), 3.88 – 3.73 (m, 3H, H-2, H-6A, H-6B), 3.57 (s, 3H, H-4, -OCH_3), 3.55 – 3.49 (m, 4H, H-3, -OCH_3), 3.48 (s, 3H, -OCH_3), 1.98 (s, 1H, 6-O*H*); ^{13}C NMR (126 MHz, Chloroform-*d*) δ 134.2, 131.8, 129.3, 127.8, 85.0 (C-1), 81.7 (C-3), 78.8 (C-2), 76.6 (C-4), 73.1 (C-5), 62.3 (C-6), 61.0, 58.4, 57.9.

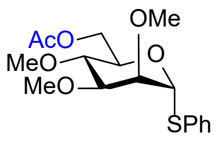

Phenyl 6-*O*-acetyl-2,3,4-tri-*O*-methyl-1-thio-α-D-mannopyranoside 6: *Via* general acetylation protocol starting with **S19** (22 mg, 70 µmol). Obtained **6** (25 mg, *quant.*) as a clear oil. TLC: (EtOAc/n-heptane, 1/1, v/v): R_f = 0.63; ^1H NMR (500 MHz, Chloroform-*d*) δ 7.60 – 7.38 (m, 2H, 2 x C*H* Ar), 7.40 – 7.16 (m, 3H, 3 x C*H* Ar), 5.64 (d, *J* = 1.6 Hz, 1H, H-1), 4.37 – 4.28 (m, 2H, H-6A, H-6B), 4.23 (ddd, *J* = 8.5, 5.1, 3.1 Hz, 1H, H-5), 3.86 (dd, *J* = 3.0, 1.7 Hz, 1H, H-2), 3.54 (s, 3H, -OCH_3), 3.53 – 3.49 (m, 4H, H-3, -OCH_3), 3.47 (s, 4H, H-4, -OCH_3), 2.06 (s, 3H, CH_3 Ac); ^{13}C NMR (126 MHz, Chloroform-*d*) δ 170.9, 134.2, 131.6, 129.2, 127.7, 84.6 (C-1), 81.7 (C-3), 78.5 (C-2), 76.7 (C-4) 70.8 (C-5), 63.6 (C-6), 61.0, 58.2, 57.8, 21.0; HRMS *(m/z)*: $[M+Na]^+$ calcd for $C_{17}H_{24}O_6S$, 379.1191; found, 379.1202.

Preparation of Donors 7-9

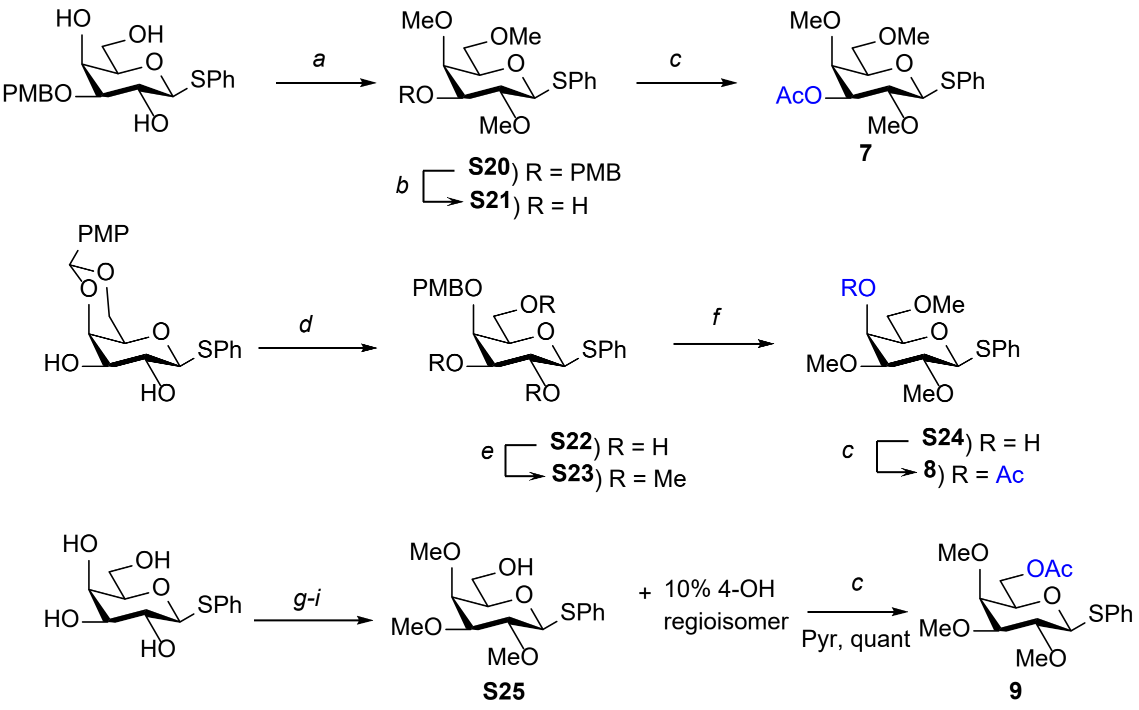

Supplementary Figure 32. Synthesis of mono-acetyl gactosides **7-9**. Reagents and conditions: a) MeI, NaH, DMF, **S20**: 99%; b) *p*TsOH, MeOH, **S21**: 81%; c) Ac_2O, pyridine, **7**: *quant*; **8**: *quant*, **9**: *quant*; d) BH_3, TMSOTf, THF, **S22**: 75%; e) MeI, NaH, DMF, **S23**: 97%; f) *p*TsOH, MeOH, **S24**: 75%; g) TBS-Cl, imidazole, DMF; h) MeI, NaH, DMF; i) TBAF, THF, **S25**: 27% over three steps.

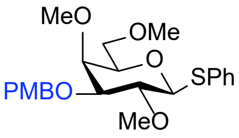

Phenyl 2,4,6-tri-*O*-methyl-3-*O*-*p*-methoxybenzyl-1-thio-β-D-galactopyranoside S20: *Via* general methylation protocol starting with phenyl 3-*O*-*p*-methoxybenzyl-1-thio-β-D-galactopyranoside (327 mg, 0.833 mmol). The residue was purified by silica column chromatography (30% EtOAc in *n*-heptane) to obtain the product **S25** (359 mg, 99%) as a white amorphous solid. TLC: (EtOAc/n-heptane, 3/7, v/v): R_f = 0.31; ^{1}H NMR (400 MHz, Chloroform-*d*) δ 7.62 – 7.46 (m, 2H, 2 x C*H* Ar), 7.40 – 7.13 (m, 5H, 5 x C*H* Ar), 6.97 – 6.81 (m, 2H, C*H* Ar), 4.70 – 4.63 (m, 2H, 2 x C*H*H PMB), 4.49 (d, *J* = 9.3 Hz, 1H, H-1), 3.81 (s, 3H, -OC*H*3), 3.62 – 3.58 (m, 4H, H-4, -OC*H*3), 3.58 – 3.55 (m, 4H, H-6A, -OC*H*3), 3.55 – 3.45 (m, 4H, H-6B, H-2, H-5), 3.42 (dd, *J* = 9.2, 2.9 Hz, 1H, H-3), 3.36 (s, 3H, -OC*H*3); ^{13}C NMR (101 MHz, Chloroform-*d*) δ 134.3, 131.6, 130.4, 129.3, 128.7, 127.1, 113.9, 87.9 (C-1), 83.2 (C-3), 79.4 (C-2), 77.0 (C-5), 76.0 (C-4), 72.3, 70.7 (C-6), 61.3, 61.3, 59.2, 55.3; HRMS *(m/z)*: $[M+Na]^+$ calcd for $C_{23}H_{30}O_6S$, 457.1661; found, 457.1658.

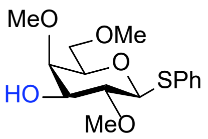

Phenyl 2,4,6-tri-*O*-methyl-1-thio-β-D-galactopyranoside S21: S20 (359 mg, 0.826 mmol) was suspended in methanol (4.13 mL) and heated to 50°C before *p*TsOH (157 mg, 0.826 mmol) was added. The suspension stirred for 18 hours before being neutralized by addition of TEA. The mixture was concentrated in *vacuo*. Silica column chromatography (50% ethyl acetate in n-heptane) of the residue obtained the product **S21** (210 mg, 81%) as a clear oil; TLC: (EtOAc/n-heptane, 1/1, v/v): R_f = 0.23; ^{1}H NMR (500 MHz, Chloroform-*d*) δ 7.59 – 7.47 (m, 1H, 2 x C*H* Ar), 7.37 – 7.14 (m, 3H, 3 x C*H* Ar), 4.50 (d, *J* = 9.6 Hz, 1H, H-1), 3.71 – 3.50 (m, 11H, 2 x -OC*H*3, H-3, H-4, H-5, H-6A, H-6B), 3.37 (s, 3H, -OC*H*3), 3.29 (ddd, *J* = 9.7, 7.2, 3.1 Hz, 1H, H-2), 2.47 (d, *J* = 6.9 Hz, 1H, 4-OH); ^{13}C NMR (126 MHz, Chloroform-*d*) δ 134.3,

131.7, 128.9, 127.4, 87.7 (C-1), 80.8 (C-2), 78.4 (C-5), 77.3 (C-3), 75.8 (C-4), 70.7 (C-6), 61.9, 61.5, 59.3; **HRMS** ***(m/z)***: $[M+Na]^+$ calcd for $C_{15}H_{22}O_5S$, 337.1086; found, 337.1104.

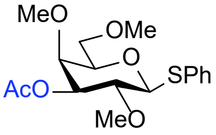

Phenyl 3-*O*-acetyl-2,4,6-tri-O-methyl-1-thio-β-D-galactopyranoside 7: *Via* general acetylation protocol starting with **S21** (150 mg, 0.477 mmol). **7** (170 mg, quant.) was obtained as a pale oil. TLC: (EtOAc/n-heptane, 1/1, v/v): R_f = 0.64; ^{1}H NMR (400 MHz, Chloroform-*d*) δ 7.60 – 7.48 (m, 2H, 2 x C*H* Ar), 7.40 – 7.11 (m, 3H, 3 x C*H* Ar), 4.87 (dd, *J* = 9.7, 3.1 Hz, 1H, H-3), 4.57 (d, *J* = 9.8 Hz, 1H, H-1), 3.71 (dd, *J* = 3.2, 1.0 Hz, 1H, H-4), 3.68 – 3.45 (m, 10H, 2 x -OC*H*₃, H-2, H-5, H-6A, H-6B), 3.34 (s, 3H, -OC*H*₃), 2.15 (s, 3H, C*H*₃ Ac); ^{13}C NMR (101 MHz, Chloroform-*d*) δ 170.4, 134.0, 131.8, 128.9, 128.9, 127.4, 87.9 (C-1), 77.4 (C-2), 77.3 (C-3), 76.7 (C-5), 76.6 (C-4), 70.5 (C-6), 61.4, 61.1, 59.3, 21.2; HRMS *(m/z)*: $[M+Na]^+$ calcd for $C_{17}H_{24}O_6S$, 379.1191; found, 379.1211.

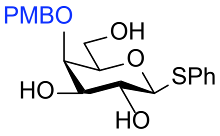

Phenyl 4-*O*-*p*-methoxybenzyl-1-thio-β-D-galactopyranoside S22: Phenyl 4,6-*O*-*p*-methoxybenzylidene-1-thio-β-D-galactopyranoside (1.0 g, 2.56 mmol) was dissolved in DCM (26 mL) after which 1 M BH_3 in THF (18 mL, 18 mmol) was added at 0 °C . The mixture was allowed to warm up to room temperature in 20 minutes before TMSOTf (46 µL, 0.26 mmol) was added. After full conversion was observed after 3 hours, the reaction was cooled to 0°C and triethylamine (0.5 mL) was added. The mixture was quenched by careful addition of methanol and subsequently concentrated *in vacuo*. Silica column chromatography (70% ethyl acetate in n-heptane) of the residue gave the product **S22** (756 mg, 75%) as a white amorphous solid; TLC: (EtOAc/n-heptane, 4/1, v/v): R_f = 0.15; ^{1}H NMR (400 MHz, Acetone-d_6) δ 7.71 – 7.46 (m, 2H, 2 x C*H* Ar), 7.40 – 7.10 (m, 5H, 5 x C*H* Ar), 7.01 – 6.81 (m, 2H, 2 x C*H* Ar), 4.89 (d, *J* = 11.0 Hz, 1H, C*H*H PMB), 4.70 – 4.46 (m, 2H, H-1, C*H*H PMB), 4.24 (dd, *J* = 4.5, 1.3 Hz, 1H, H-4), 4.11 – 4.04 (m, 1H, 2-O*H*), 3.92 (dd, *J* = 2.8, 0.8 Hz, 1H, 3-O*H*), 3.79 (s, 3H, -OC*H*₃), 3.78 – 3.60 (m, 5H, H-2, H-3, H-5, H-6A, H-6B); ^{13}C NMR (101 MHz, Acetone-d_6) δ 160.1, 135.8, 132.5, 131.6, 131.6, 130.0, 129.5, 127.4, 114.3, 89.0, 89.0, 80.3, 80.3, 77.3, 77.2, 77.1, 77.0, 75.2, 71.0, 70.9, 70.9, 62.0, 61.8, 55.5, 55.0; HRMS *(m/z)*: $[M+Na]^+$ calcd for $C_{20}H_{24}O_6S$, 415.1191; found, 415.1187.

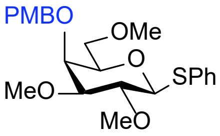

Phenyl 2,3,6-tri-*O*-methyl-4-*O*-*p*-methoxybenzyl-1-thio-β-D-galactopyranoside S23: *Via* general methylation protocol starting with **S22** (316 mg, 0.81 mmol). Crystallization of the residue in methanol gave **S23** (340 mg, 97%) as a white solid. TLC: (EtOAc/n-heptane, 1/1, v/v): R_f = 0.60; ^{1}H NMR (400 MHz, Chloroform-*d*) δ 7.63 – 7.42 (m, 2H, 2 x C*H* Ar), 7.33 – 7.25 (m, 2H, 2 x C*H* Ar), 7.24 – 7.18 (m, 3H, 3 x C*H* Ar), 6.93 – 6.83 (m, 2H, 2 x C*H* Ar), 4.83 (d, *J* = 11.3 Hz, 1H, C*H*H PMB), 4.55 (d, *J* = 11.3 Hz, 1H, CH*H* PMB), 4.50 (d, *J* = 9.6 Hz, 1H, H-1), 3.91 (d, *J* = 2.8 Hz, 1H, H-4), 3.82 (s, 3H, -OC*H*₃), 3.57 (s, 3H, -OC*H*₃), 3.55 – 3.41 (m, 7H, -OC*H*₃, H-6A, H-6B, H-2, H-5), 3.28 (s, 3H, -OC*H*₃), 3.22 (dd, *J* = 9.2, 2.8 Hz, 1H, H-3); ^{13}C NMR (101 MHz, Chloroform-*d*) δ 159.3, 134.3, 131.7, 131.1, 129.6, 128.8, 127.1, 113.7, 87.7 (C-1), 86.6 (C-3), 79.0 (C-2), 77.3 (C-5), 74.1, 72.2 (C-4), 71.1 (C-6), 61.2, 59.3, 58.4, 55.5; HRMS *(m/z)*: $[M+Na]^+$ calcd for $C_{23}H_{30}O_6S$, 457.1661; found, 457.1661.

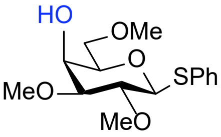

Phenyl 2,3,6-tri-*O*-methyl-1-thio-β-D-galactopyranoside S24: S23 (315 mg, 0.725 mmol) was suspended in methanol (3.62 mL) and heated to 50°C before *p*-TsOH (138 mg, 0.725 mmol) was added. The suspension stirred for 18 hours before being neutralized by addition of TEA. The mixture was concentrated in *vacuo*. Silica column chromatography (50% ethyl acetate in n-heptane) of the residue obtained the product **S24** (171 mg, 75%) as a clear oil. TLC: (EtOAc/n-heptane, 1/1, v/v): R_f = 0.31; ^{1}H NMR (500 MHz, Chloroform-*d*) δ 7.65 – 7.41 (m, 2H, 2 x

C*H* Ar), 7.38 – 7.13 (m, 3H, 3 x C*H* Ar), 4.51 (d, *J* = 9.7 Hz, 1H, H-1), 4.12 (s, 1H, H-4), 3.72 (dd, *J* = 10.0, 5.8 Hz, 1H, H-6A), 3.64 (dd, *J* = 10.0, 5.6 Hz, 1H, H-6B), 3.57 (s, 3H, -OCH_3), 3.54 (t, *J* = 5.4 Hz, 1H, H-5), 3.51 (s, 3H, -OCH_3), 3.39 (s, 3H, -OCH_3), 3.35 (t, *J* = 9.3 Hz, 1H, H-2), 3.23 (dd, *J* = 8.9, 3.2 Hz, 1H, H-3), 2.61 – 2.39 (m, 1H, 4-O*H*); ^{13}C NMR (126 MHz, Chloroform-*d*) δ 133.9, 132.0, 128.9, 127.5, 87.6 (C-1), 84.8 (C-3), 78.6 (C-2), 76.9 (C-5), 71.9 (C-6), 66.2 (C-4), 61.3, 59.6, 57.7; HRMS *(m/z)*: [M+Na]$^+$ calcd for $C_{15}H_{22}O_5S$, 337.1086; found, 337.1092.

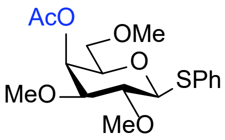

Phenyl 4-*O*-acetyl-2,3,6-tri-O-methyl-1-thio-β-D-galactopyranoside 8: *Via* general acetylation protocol starting with **S24** (171 mg, 0.544 mmol). The product **8** (193 mg, quant.) was obtained as a pale oil. TLC: (EtOAc/n-heptane, 1/1, v/v): R_f = 0.50; ^{1}H NMR (500 MHz, Chloroform-*d*) δ 7.57 (dt, *J* = 8.6, 2.1 Hz, 2H, 2 x C*H* Ar), 7.36 – 7.16 (m, 3H, 3 x C*H* Ar), 5.48 (d, *J* = 1.6 Hz, 1H, H-4), 4.64 – 4.48 (m, 1H, H-1), 3.70 – 3.64 (m, 1H, H-5), 3.57 (s, 3H, -OCH_3), 3.50 (dd, *J* = 9.9, 6.1 Hz, 1H, H-6A), 3.42 (s, 3H, -OCH_3), 3.41 – 3.38 (m, 1H, H-6B), 3.33 (s, 3H, -OCH_3), 3.28 (dd, *J* = 5.5, 1.5 Hz, 2H, H-2, H-3), 2.13 (s, 3H, -CH_3 Ac); ^{13}C NMR (126 MHz, Chloroform-*d*) δ 170.4, 133.8, 132.0, 128.9, 127.6, 87.6 (C-1), 83.6 (C-3), 78.5 (C-2), 76.1 (C-5), 71.1 (C-6), 66.6 (C-4), 61.4, 59.5, 57.9, 21.0; HRMS *(m/z)*: [M+Na]$^+$ calcd for $C_{17}H_{24}O_6S$, 379.1191; found, 379.1201.

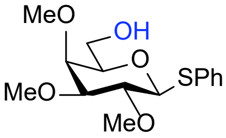

Phenyl 2,3,4-tri-*O*-methyl-1-thio-β-D-galactopyranoside S25: To a mixture of phenyl 1-thio-β-galactopyranoside (1.00 g, 1 eq, 3.67 mmol) in DMF (18.4 mL) was added imidazole (375 mg, 5.51 mmol) and TBS-Cl (664 mg, 4.41 mmol). The reaction was stirred for 2 hours before being quenched by the addition of 0.5 mL of MeOH. The mixture was partitioned between H_2O and Et_2O, and the aqueous layer was extracted. The combined organic phases were subsequently washed with aq. 1 M HCl, sat aq. $NaHCO_3$ and brine before being dried over $MgSO_4$, filtered, and conc. *in vacuo.* The crude product was dissolved in DMF (18.4 mL) and to this solution were added MeI (1.82 g, 804 µL, 3.5 eq, 12.9 mmol) and NaH (0.73 g, 18.4 mmol, 60 wt% in mineral oil) at 0 ºC. The reaction stirred at ambient temperature for 18 hours before being quenched by careful addition of MeOH (0.5 mL) at 0ºC. The residue was taken up in Et_2O and washed with 5% aq. LiCl and brine. The organic layer was dried ($MgSO_4$), filtered and concentrated *in vacuo*. The residue was dissolved in 4 mL of THF and treated with 7.3 mL of 1.0 M TBAF (in THF, 2 eq, 7.3 mmol). The mixture was stirred for 3 h and subsequently taken up in EtOAc and H_2O. The water layer was further extracted with EtOAc, and the combined organic layers were dried (MgSO4), filtered and concentrated *in vacuo*. Purification of the residue by Silica column chromatography (50 % ethyl acetate in *n*-heptane) afforded S25 (302 mg, 26 %) as a white amorphous solid. In addition, the 4-OH regio-isomer (113 mg, 10 %) was obtained. TLC: (EtOAc/n-heptane, 1/1, v/v): R_f = 0.13 ^{1}H NMR (500 MHz, Chloroform-*d*) δ 7.52 (dt, *J* = 8.5, 2.0 Hz, 2H, 2 x C*H* Ar), 7.35 – 7.13 (m, 3H, 3 x C*H* Ar), 4.53 (d, *J* = 9.7 Hz, 1H, H-1), 3.92 (dd, *J* = 11.2, 7.6 Hz, 1H, H-6A), 3.75 – 3.66 (m, 1H, H-6B), 3.64 (d, *J* = 2.8 Hz, 1H, H-4), 3.60 (s, 3H, -OCH_3), 3.55 (s, 3H, -OCH_3), 3.54 (s, 3H, -OCH_3), 3.48 – 3.35 (m, 2H, H-2, H-5), 3.22 (dd, *J* = 9.2, 3.0 Hz, 1H, H-3), 2.01 (d, *J* = 7.4 Hz, 1H, 6-O*H*); ^{13}C NMR (126 MHz, Chloroform-*d*) δ 134.1, 131.8, 129.0, 127.4, 87.7 (C-1), 86.2 (C-3), 79.4 (C-2), 79.0 (C-5), 76.0 (C-4), 62.5 (C-6), 61.4, 61.3, 58.5; HRMS *(m/z)*: [M+Na]$^+$ calcd for $C_{15}H_{22}O_5S$, 337.1086; found, 337.1093.

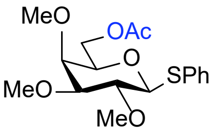

Phenyl 6-*O*-acetyl-2,3,4-tri-*O*-methyl-1-thio-β-D-galactopyranoside 9: *Via* general acetylation protocol starting with **S25** (205 mg, 0.65 mmol). Product **9** (233 mg, *quant.*) was obtained as a pale amorphous solid. TLC: (EtOAc/n-heptane, 1/1, v/v): R_f = 0.58 ^{1}H NMR (500 MHz, Chloroform-*d*) δ 7.63 – 7.45 (m, 2H, 2 x C*H* Ar), 7.35 – 7.18 (m, 3H, 3 x C*H* Ar), 4.49 (d, *J* = 9.7 Hz, 1H, H-1), 4.31 (dd, *J* = 11.3, 7.2 Hz, 1H, H-6A), 4.24 (dd, *J* = 11.3, 5.4 Hz, 1H, H-6B), 3.61

(m, 1H, H-4), 3.60 (s, 3H, -OC*H*$_3$), 3.59 – 3.56 (m, 1H, H-5), 3.56 (s, 3H, -OC*H*$_3$), 3.54 (s, 3H), 3.46 – 3.39 (m, 1H, H-2), 3.21 (dd, *J* = 9.2, 3.0 Hz, 1H, H-3), 2.07 (s, 3H, C*H*$_3$ Ac); ^{13}C NMR (126 MHz, Chloroform-*d*) δ 170.8, 134.3, 131.9, 128.8, 127.4, 87.9 (C-1), 86.1 (C-3), 79.3 (C-2), 76.1 (C-5), 75.7 (C-4), 63.5 (C-6), 61.5, 61.3, 58.6, 21.0; HRMS *(m/z)*: [M+Na]$^+$ calcd for $C_{17}H_{24}O_6S$, 379.1191; found, 379.1195.

Oxidized donors

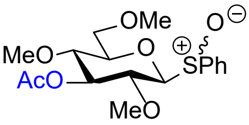

Phenyl 3-*O*-acetyl-2,4,6-tri-*O*-methyl-1-thiosulfinyl-β-D-glucopyranoside S26: *Via* general S-oxidation protocol starting with **1** (10 mg, 0.028 mmol). HRMS *(m/z)*: [M+Na]$^+$ calcd for $C_{17}H_{17}O_7S$, 395.1140; found, 395.1150.

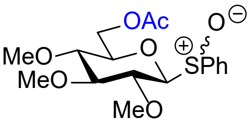

Phenyl 6-*O*-acetyl-2,3,4-tri-*O*-methyl-1-thiosulfinyl-β-D-glucopyranoside S27: *Via* general S-oxidation protocol starting with **2** (10 mg, 0.028 mmol). HRMS *(m/z)*: [M+Na]$^+$ calcd for $C_{17}H_{17}O_7S$, 395.1140; found, 395.1153.

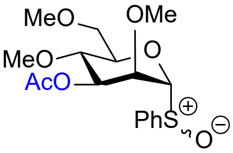

Phenyl 3-*O*-acetyl-2,4,6-tri-*O*-methyl-1-thiosulfinyl-α-D-mannopyranoside S28: *Via* general S-oxidation protocol starting with **3** (17 mg, 0.048 mmol). HRMS *(m/z)*: [M+Na]$^+$ calcd for $C_{17}H_{24}O_7S$, 395.1140; found, 379.1151.

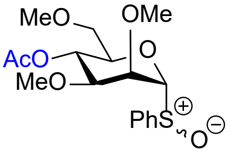

Phenyl 4-*O*-acetyl-2,3,6-tri-*O*-methyl-1-thiosulfinyl-α-D-mannopyranoside S29: *Via* general S-oxidation protocol starting with **4** (10 mg, 0.028 mmol). HRMS *(m/z)*: [M+Na]$^+$ calcd for $C_{17}H_{24}O_7S$, 395.1140; found, 379.1150.

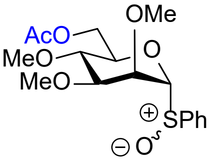

Phenyl 6-*O*-acetyl-2,3,4-tri-*O*-methyl-1-thiosulfinyl-α-D-mannopyranoside S30: *Via* general S-oxidation protocol starting with **5** (25 mg, 0.070 mmol). HRMS *(m/z)*: [M+Na]$^+$ calcd for $C_{17}H_{17}O_7S$, 395.1140; found, 395.1146.

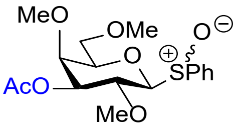

Phenyl 3-*O*-acetyl-2,4,6-tri-*O*-methyl-1-thiosulfinyl-β-D-galactopyranoside S31: *Via* general S-oxidation protocol starting with **6** (10 mg, 0.028 mmol). HRMS *(m/z)*: [M+Na]$^+$ calcd for $C_{17}H_{17}O_7S$, 395.1140; found, 395.1147.

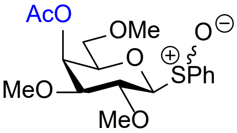

Phenyl 4-*O*-acetyl-2,3,6-tri-*O*-methyl-1-thiosulfinyl-β-D-galactopyranoside S32: *Via* general S-oxidation protocol starting with **7** (13 mg, 0.035 mmol); HRMS *(m/z)*: [M+Na]$^+$ calcd for $C_{17}H_{17}O_7S$, 395.1140; found, 395.1153.

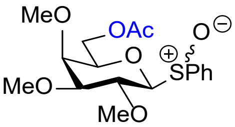

Phenyl 6-*O*-acetyl-2,3,4-tri-*O*-methyl-1-thiosulfinyl-β-D-galactopyranoside S33: *Via* general S-oxidation protocol starting with **8** (12 mg, 0.033 mmol); HRMS *(m/z)*: [M+Na]$^+$ calcd for $C_{17}H_{17}O_7S$, 395.1140; found, 395.1151.

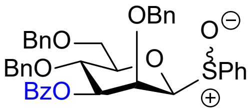

Phenyl 3-*O*-benzoyl-2,4,6-tri-*O*-benzyl-1-thiosulfinyl-β-D-mannopyranoside S34: *Via* general S-oxidation protocol starting with **13** (20 mg, 0.031 mmol). HRMS *(m/z)*: [M+Na]$^+$ calcd for $C_{40}H_{38}O_7S$, 685.2236; found, 685.2208.

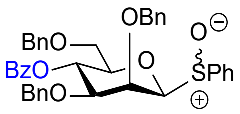

Phenyl 4-*O*-benzoyl-2,3,6-tri-*O*-benzyl-1-thiosulfinyl-β-D-mannopyranoside S35: *Via* general S-oxidation protocol starting with **14** (20 mg, 0.031 mmol). **HRMS** *(m/z)*: $[M+Na]^+$ calcd for $C_{40}H_{38}O_7S$, 685.2236; found, 685.2206.

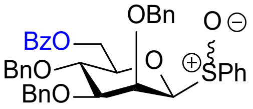

Phenyl 6-*O*-benzoyl-2,3,4-tri-*O*-benzyl-1-thiosulfinyl-β-D-mannopyranoside S36: *Via* general S-oxidation protocol starting with **15** (20 mg, 0.031 mmol). **HRMS** *(m/z)*: $[M+Na]^+$ calcd for $C_{40}H_{38}O_7S$, 685.2236; found, 685.2208.

Model glycosylation reactions

Bn/Bz-protected glycosyl donors

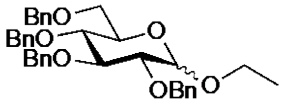

Ethyl 2,3,4,6-tetra-*O*-benzyl-α/β-D-glucopyranoside (S37). The title compound was prepared according to general procedure VII. Column chromatography (100:0 → 90:10, pentane:EtOAc) yielded the title compound (40 mg, 58 µmol, 70%, colourless oil, α:β; 15:85). TLC: R_f 0.50 (pentane:EtOAc, 90:10, v:v); IR (thin film, cm^{-1}): 733, 1026, 1065, 1358, 1452, 1497, 2864, 2901; Data of the major stereoisomer (β product): ^{1}H NMR (400 MHz, $CDCl_3$, HH-COSY, HSQC): δ 7.39 – 7.08 (m, 20H, CH_{arom}), 5.00 – 4.89 (m, 2H, C*H*H Bn, C*H*H Bn), 4.82 (d, *J* = 10.9 Hz, 1H, C*H*H Bn), 4.79 (d, *J* = 11.0 Hz, 1H, CH*H* Bn), 4.72 (d, *J* = 10.9 Hz, 1H, CH*H* Bn), 4.64 – 4.55 (m, 2H, C*H*H Bn), 4.52 (d, *J* = 10.6 Hz, 1H, CH*H* Bn), 4.40 (d, *J* = 7.8 Hz, 1H, H-1), 4.01 (dq, *J* = 9.5, 7.1 Hz, 1H, C*H*HCH_3 Et), , 1H, H-6), 3.68 (t, *J* = 5.5 Hz, 1H, H-6), 3.63 (t, *J* = 8.7 Hz, 1H, H-3), 3.72 – 3.54 (m, 1H, CH*H*CH_3 Et) 3.57 (t, *J* = 9.2 Hz, 1H, H-4), 3.45 (dd, *J* = 8.9, 7.8 Hz, 1H, H-2), 3.50 – 3.41 (m, 1H, H-5), 1.29 (t, *J* = 7.1 Hz, 3H, CH_3 Et); ^{13}C NMR ($CDCl_3$, 101 MHz, HSQC): δ 138.8, 138.7, 138.3, 138.2 ($C_{q\text{-}arom}$), 128.5, 128.5, 128.5, 128.5, 128.3, 128.2, 128.1, 128.1, 128.0, 127.9, 127.9, 127.8, 127.7, 127.7 (CH_{arom}), 103.6 (C-1), 84.8 (C-3), 82.4 (C-2), 78.1 (C-4), 75.8 (CH_2 Bn), 75.1 (CH_2 Bn), 75.0 (C-5), 74.9 (CH_2 Bn), 73.6 (CH_2 Bn), 69.2 (C-6), 65.7 (*C*H_2CH_3 Et), 15.5 (CH_3 Et); Diagnostic signals of the minor stereoisomer (α product): ^{1}H NMR (400 MHz, $CDCl_3$, HH-COSY, HSQC): δ 5.00 (d, *J* = 10.9 Hz, 1H, C*H*H Bn), 4.76 (d, *J* = 3.7 Hz, 1H, H-1), 4.46 (d, *J* = 11.7 Hz, 1H, CH*H* Bn); ^{13}C NMR (101 MHz, $CDCl_3$, HSQC): δ 96.7 (C-1); HRMS: $[M+Na]^+$ calcd for $C_{36}H_{40}O_6$ 591.27171, found 591.27077.

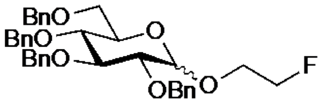

2-Fluoroethyl 2,3,4,6-tetra-*O*-benzyl-α/β-D-glucopyranoside (S38). The title compound was prepared according to general procedure VII. Column chromatography (97:3 → 90:10, pentane:EtOAc) yielded the title compound (44 mg, 75 µmol, 75%, colourless oil, α:β; 36:64). TLC: R_f 0.34, 0.46 (pentane:EtOAc, 85:15, v:v); IR (thin film, cm^{-1}): 695, 734, 1027, 1065, 1360, 1453, 1497, 2901; Data of the major stereoisomer (α product): ^{1}H NMR (400 MHz, $CDCl_3$, HH-COSY, HSQC): δ 7.41 – 7.08 (m, 20H, CH_{arom}), 5.01 – 4.90 (m, 2H, C*H*H Bn, CH*H* Bn), 4.87 – 4.42 (m, 9H, H-1, C*H*H Bn, CH*H* Bn, C*H*H Bn, CH*H* Bn, C*H*H Bn, CH*H* Bn, CH_2C*H*HF, CH_2CH*H*F), 4.12 (dddd, *J* = 33.2, 12.1, 4.5, 2.5 Hz, 1H, C*H*HCH_2F), 3.94 – 3.54 (m, 5H, H-3, H-4, H-6, H-6, CH*H*CH_2F), 3.49 (dd, *J* = 8.9, 7.8 Hz, 1H, H-2) 3.52 – 3.43 (m, 1H, H-5); ^{13}C NMR (101 MHz, $CDCl_3$, HSQC): ^{13}C NMR (101 MHz, $CDCl_3$) δ 138.9, 138.7, 138.5, 138.3, 138.2, 138.1, 138.0 ($C_{q\text{-}arom}$), 128.6, 128.5, 128.3, 128.1, 128.1, 128.0, 128.0, 128.0, 127.9, 127.8, 127.8, 127.8, 127.8, 127.7 (CH_{arom}), 103.9 (C-1), 84.7 (C-3), 82.7 (d, *J* = 169.8 Hz, CH_2F), 82.2 (C-2), 77.8 (C-4), 75.8 (C-5), 75.2 (CH_2 Bn), 75.0 (CH_2 Bn), 74.9 (CH_2 Bn), 73.6 (CH_2 Bn), 69.0 (d, *J* = 20.0 Hz, *C*H_2CH_2F), 68.9 (C-6); Diagnostic signals of the minor stereoisomer (β product): ^{1}H NMR (400 MHz, $CDCl_3$, HH-COSY, HSQC): ^{1}H NMR (400 MHz, $CDCl_3$, HH-COSY, HSQC): δ 4.79 (d, *J* = 3.7 Hz, 1H, H-1), 4.01 (t, *J* = 9.3 Hz, 1H, H-3); ^{13}C NMR (101 MHz, $CDCl_3$, HSQC): δ 97.5 (C-1), 82.6 (d, *J* = 169.9 Hz, CH_2F), 67.1 (d, *J* = 20.2 Hz, *C*H_2CH_2F); HRMS: $[M+Na]^+$ calcd for $C_{36}H_{39}FO_6$ 609.2628, found 609.2638.

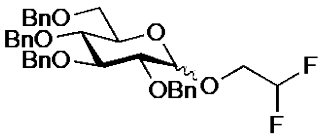

2,2-Difluoroethyl 2,3,4,6-tetra-*O*-benzyl-α/β-D-glucopyranoside (S39). The title compound was prepared according to general procedure VII. Column chromatography (97:3 → 90:10, pentane:EtOAc) yielded the title compound (35 mg, 58 μmol, 58%, colourless oil, α:β; 48:52). TLC: R_f 0.31 (pentane:EtOAc, 90:10, v:v); IR (thin film, cm-1): 695, 733, 1027, 1066, 1360, 1453, 1497, 2865; Data of the major stereoisomer (β product): ^{1}H NMR (400 MHz, $CDCl_3$, HH-COSY, HSQC): δ 7.42 – 7.09 (m, 20H, CH_{arom}), 5.96 (tt, *J* = 55.1, 4.2 Hz, 1H, CHF_2), 4.93 (d, *J* = 10.9 Hz, 1H, C*H*H Bn), 4.90 (d, *J* = 11.0 Hz, 1H, C*H*H Bn), 4.84 – 4.74 (m, 2H, C*H*H Bn, CH*H* Bn), 4.70 (d, *J* = 10.8 Hz, 1H, C*H*H Bn), 4.63 (d, *J* = 12.1 Hz, 1H, C*H*H Bn), 4.60 (d, *J* = 11.1 Hz, 1H, CH*H* Bn), 4.53 (d, *J* = 12.2 Hz, 1H, CH*H* Bn), 4.43 (d, *J* = 7.6 Hz, 1H, H-1), 4.03 (dddd, *J* = 19.8, 11.8, 10.8, 3.4 Hz, 1H, CH*H*CHF_2), 3.86 – 3.55 (m, 5H, H-3, H-4, H-6, H-6, C*H*HCHF_2), 3.48 (dd, *J* = 9.0, 7.7 Hz, 1H, H-2) 3.48 – 3.44 (m, 1H, H-5); ^{13}C NMR (101 MHz, $CDCl_3$, HSQC): δ ^{13}C NMR (101 MHz, $CDCl_3$) δ 138.8, 138.6, 138.3, 138.1 ($C_{q\text{-}arom}$), 128.6, 128.5, 128.5, 128.2, 128.1, 128.0, 127.9, 127.9 (CH_{arom}), 114.2 (t, *J* = 241.3 Hz, CHF_2), 104.1 (C-1), 84.5 (C-3), 82.0 (C-2), 77.6 (C-4), 76.8 (CH_2 Bn), 75.9 (CH_2 Bn), 75.1 (CH_2 Bn), 75.0 (C-5), 73.6 (CH_2 Bn), 70.7, 68.8 (C-6), 67.3 (t, *J* = 28.8 Hz, *C*H_2CHF_2); Diagnostic signals of the minor stereoisomer (α product): ^{1}H NMR (400 MHz, $CDCl_3$, HH-COSY, HSQC): δ 4.75 (d, *J* = 3.6 Hz, 1H, H-1), 3.96 (t, *J* = 9.0 Hz, 1H, H-3); ^{13}C NMR (101 MHz, $CDCl_3$, HSQC): δ 98.0 (C-1), 73.6 (CH_2 Bn), 68.8 (t, *J* = 28.9 Hz, *C*H_2CHF_2) 68.3 (C-6); HRMS: $[M+Na]^+$ calcd for $C_{36}H_{38}F_2O_6$ 627.2534, found 627.2538.

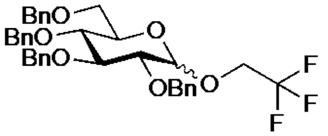

2,2,2-Trifluoroethyl 2,3,4,6-tetra-*O*-benzyl-α/β-*D*-glucopyranoside (S40). The title compound was prepared according to general procedure VII. Column chromatography (97:3 → 90:10, pentane:EtOAc) yielded the title compound (50 mg, 80 μmol, 80%, colourless oil, α:β; 72:28). TLC: R_f 0.36 (pentane:EtOAc, 90:10, v:v); IR (thin film, cm^{-1}): 695, 734, 1027, 1047, 1070, 1154, 1277, 1361, 1453, 1497, 2899; Data of the major stereoisomer (α product): ^{1}H NMR (400 MHz, $CDCl_3$, HH-COSY, HSQC): δ 7.61 – 6.74 (m, 20H, CH_{arom}), 4.98 (d, *J* = 12.0 Hz, 1H, C*H*H Bn), 4.83 (d, *J* = 11.9 Hz, 1H, C*H*H Bn), 4.82 – 4.76 (m, 2H, C*H*H Bn, CH*H* Bn), 4.80 (d, *J* = 2.2 Hz, 1H, H-1), 4.63 (d, *J* = 12.0 Hz, 1H, CH*H* Bn), 4.59 (d, *J* = 12.1 Hz, 1H C*H*H Bn), 4.47 (d, *J* = 11.7 Hz, 1H, CH*H* Bn), 4.46 (d, *J* = 12.1 Hz, 1H, CH*H* Bn), 3.98 (dd, *J* = 9.7, 8.9 Hz, 1H, H-3), 3.88 (q, *J* = 8.7 Hz, 2H, C*H*$_2CF_3$), 3.81 – 3.61 (m, 3H, H-4, H-6, H-6), 3.59 (dd, *J* = 9.6, 3.6 Hz, 1H, H-2); ^{13}C NMR (101 MHz, $CDCl_3$, HSQC): δ 138.8, 138.6, 138.2, 138.1, 138.0, 137.8 ($C_{q\text{-}arom}$), 128.6, 128.6, 128.6, 128.5, 128.2, 128.1, 128.1, 128.0, 127.9, 127.9, 127.8, 127.8 (CH_{arom}), 128.2 (q, *J* = 279.5 Hz, CH_2*C*F_3), 97.9 (C-1), 81.7 (C-3), 79.8 (C-2), 77.3 (C-4), 75.9 (CH_2 Bn), 75.3 (CH_2 Bn), 73.6 (CH_2 Bn), 73.5 (CH_2 Bn), 71.0 (C-5), 68.2 (C-6), 64.8 (q, *J* = 34.9 Hz, *C*H_2CF_3); ^{13}C-GATED NMR (101 MHz, $CDCl_3$) δ 97.9 ($J_{H1\text{-}C1}$ = 171 Hz, α) Diagnostic signals of the minor stereoisomer (β product): ^{1}H NMR (400 MHz, $CDCl_3$, HH-COSY, HSQC): δ 4.93 (d, *J* = 10.9 Hz, 1H), 4.92 (d, *J* = 10.6 Hz, 1H, CHH Bn), 4.68 (d, *J* = 10.7 Hz, 1H, CHH Bn), 4.50 (d, *J* = 7.7 Hz, 1H, H-1), 4.22 (dq, *J* = 12.4, 8.7 Hz, 1H, C*H*$_2CF_3$), 3.50 (dd, *J* = 9.0, 7.6 Hz, 1H, H-2); ^{13}C NMR (101 MHz, $CDCl_3$, HSQC): δ 103.8 (C-1), 81.8 (C-3), 75.2 (CH_2 Bn), 75.1 (CH_2 Bn), 68.7 (C-6), 66.2 (q, *J* = 34.9 Hz, *C*H_2CF_3);^{13}C-GATED NMR (101 MHz, $CDCl_3$) δ 103.8 ($J_{H1\text{-}C1}$ = 159 Hz, β); HRMS: $[M+Na]^+$ calcd for $C_{36}H_{37}F_3O_6$ 645.2440, found 645.2455.

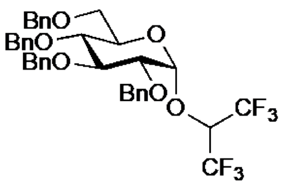

1,1,1,3,3,3-Hexafluoro-2-propyl 2,3,4,6-tetra-*O*-benzyl-α-*D*-glucopyranoside (S41). The title compound was prepared according to general procedure VII. Column chromatography (100:0 ⟶ 80:20, pentane:Et_2O) yielded the title compound (29 mg, 41 µmol, 41%, colourless oil, α:β; >98:2). TLC: R_f 0.13 (pentane:EtOAc, 95:5, v:v); $[\alpha]_D^{20}$ 20.5° (*c* 1, $CHCl_3$); IR (thin film, cm^{-1}): 687, 696, 736, 1103, 1219, 1287, 1369, 1454, 1498, 2917; ^{1}H NMR (500 MHz, $CDCl_3$, HH-COSY, HSQC, HMBC-Gated, ^{1}H-^{19}F decoupled) δ 7.36 – 7.25 (m, 20H, CH_{arom}), 5.14 (d, *J* = 3.8 Hz, 1H, H-1), 4.96 (d, *J* = 10.8 Hz, 1H, C*H*H Bn), 4.82 (dd, *J* = 10.7, 5.5 Hz, 2H, C*H*H Bn, CH*H* Bn), 4.70 (s, 2H, CH_2 Bn), 4.60 (d, *J* = 12.1 Hz, 1H, C*H*H Bn), 4.50 – 4.39 (m, 3H, CH*H* Bn, CH*H* Bn, C*H*(CF_3)$_2$), 3.96 (t, *J* = 9.5 Hz, 1H, H-3), 3.86 (dt, *J* = 10.1, 2.6 Hz, 1H, H-5), 3.76 (dd, *J* = 10.8, 3.1 Hz, 1H, H-6), 3.63 (dd, *J* = 9.8, 3.8 Hz, 1H, H-2), 3.60 (dd, *J* = 10.8, 2.1 Hz, 1H, H-6); ^{13}C NMR (126 MHz, $CDCl_3$, HSQC, HMBC-Gated) δ 138.7, 138.1, 137.8, 137.7 ($C_{q\text{-}arom}$), 128.6, 128.6, 128.5, 128.2, 128.1, 128.1, 128.0, 128.0, 127.8 (CH_{arom}), 99.6 (C-1), 81.3 (C-3), 79.0 (C-2), 77.1 (C-4), 75.9, 75.4, 73.7, 73.5 (CH_2 Bn), 72.9 (p, *J* = 33.2, *C*H(CF_3)$_2$), 72.0 (C-5), 67.9 (C-6); ^{13}C-GATED NMR (126 MHz, $CDCl_3$) δ 99.6 ($J_{H1\text{-}C1}$ = 172 Hz, α); HRMS: $[M+Na]^+$ calcd for $C_{37}H_{36}F_6O_6$ 713.2314, found 713.2329.

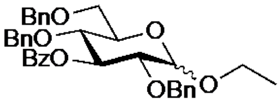

Ethyl 3-*O*-benzoyl-2,4,6-tri-*O*-benzyl-α/β-D-glucopyranoside (S42). The title compound was prepared according to general procedure VII. Column chromatography (95:5 ⟶ 75:25, pentane:Et_2O) yielded the title compound (51 mg, 88 µmol, 88%, colourless oil, α:β; 40:60). TLC: R_f 0.15, (pentane:Et_2O, 80:20, v:v); IR (thin film, cm^{-1}): 696, 711, 740, 1027, 1047, 1070, 1267, 1452, 1720, 2916, 3031; Data of the major stereoisomer (β product): ^{1}H NMR (500 MHz, $CDCl_3$, HH-COSY, HSQC): δ 7.95 – 6.93 (m, 20H, CH_{arom}), 5.49 (t, *J* = 9.5 Hz, 1H, H-4), 4.79 (d, *J* = 11.8 Hz, 1H, C*H*H Bn), 4.58 – 4.34 (m, 6H, H-1, CH*H* Bn, C*H*H Bn, CH*H* Bn, C*H*H Bn, CH*H* Bn), 4.03 (dq, *J* = 9.5, 7.1 Hz, 1H, C*H*HCH_3 Et), 3.87 – 3.53 (m, 4H, H-4, H-5, H-6, CH*H*CH_3 Et), 3.45 (dd, *J* = 9.6, 7.8 Hz, 1H, H-2), 1.31 (t, *J* = 7.1 Hz, 3H, CH_3 Et); ^{13}C NMR (126 MHz, $CDCl_3$, HSQC): δ 165.7 (C=O Bz), 138.1, 138.0, 137.6 ($C_{q\text{-}arom}$), 133.1, 131.2, 130.2, 129.9, 129.4, 128.5, 128.5, 128.4, 128.2, 128.2, 127.8, 127.5, 124.9 (CH_{arom}), 103.6 (C-1), 78.7 (C-2), 76.4 (C-3), 76.2 (C-4), 74.7 (C-5), 74.6 (CH_2 Bn), 73.9 (CH_2 Bn), 73.7 (CH_2 Bn), 68.7 (C-6), 65.8 (*C*H_2CH_3 Et), 15.5 (CH_3 Et); Diagnostic signals of the minor stereoisomer (α product): ^{1}H NMR (500 MHz, $CDCl_3$, HH-COSY, HSQC): δ 8.03 (dd, *J* = 8.4, 1.4 Hz, 2H, CH_{arom} Bz), 5.82 (dd, *J* = 10.0, 9.1 Hz, 1H, H-3), 4.87 (d, *J* = 3.5 Hz, 1H, H-1), 1.25 (t, *J* = 7.1 Hz, 3H, CH_3 Et); ^{13}C NMR (126 MHz, $CDCl_3$, HSQC): δ 165.6 (C=O Bz), 96.5 (C-1), 68.3 (CH_2 Bn), 63.7 (C-5), 15.1 (CH_3 Et); HRMS: $[M+NH_4]^+$ calcd for $C_{36}H_{38}O_7$ 600.29558, found 600.29556.

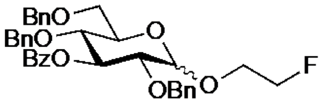

2-Fluoroethyl 3-*O*-benzoyl-2,4,6-tri-*O*-benzyl-α/β-D-glucopyranoside (S43). The title compound was prepared according to general procedure VII. Column chromatography (95:5 ⟶ 75:25, pentane:Et_2O) yielded the title compound (57 mg, 95 µmol, 95%, colourless oil, α:β; 44:56). TLC: R_f 0.15, (pentane:Et_2O, 70:30, v:v); IR (thin film, cm^{-1}): 697, 711, 742, 1026, 1046, 1070, 1090, 1269, 1452, 1724, 2869, 3030; Data of the major stereoisomer (β product): ^{1}H NMR (500 MHz, $CDCl_3$, HH-COSY, HSQC): δ 8.09 – 6.85 (m, 20H, CH_{arom}), 5.50 (t, *J* = 9.4 Hz, 1H, H-

3), 4.80 (d, J = 11.7 Hz, 1H, C*H*H Bn), 4.57 (d, J = 7.6 Hz, 1H, H-1), 4.74 – 4.33 (m, 7H, CH*H* Bn, C*H*H Bn, CH*H* Bn, C*H*H Bn, CH*H* Bn, CH_2C*H*HF, CH_2CH*H*F), 4.14 (dddd, J = 32.2, 12.1, 4.8, 2.5 Hz, 1H, C*H*HCH_2F), 4.00 – 3.69 (m, 4H, CH*H*CH_2F, H-4, H-6, H-6), 3.56 (dt, J = 9.8, 3.1 Hz, 1H, H-5), 3.50 (dd, J = 9.6, 7.7 Hz, 1H, H-2); ^{13}C NMR (126 MHz, $CDCl_3$, HSQC): δ 165.7 (C=O Bz), 138.0, 137.9, 137.9, 137.8, 137.6, 137.5, 133.1, 133.0, 129.9, 129.9, 129.4, 128.5, 128.5, 128.4, 128.4, 128.3, 128.3, 128.3, 128.2, 128.1, 128.0, 128.0, 128.0, 127.9, 127.9, 127.8, 127.8, 127.6 (CH_{arom}), 103.9 (C-1), 82.7 (d, J = 170.1 Hz, CH_2F), 78.4 (C-2), 76.3 (C-3), 76.0 (C-4), 74.8 (C-5), 74.6 (CH_2 Bn), 74.6 (CH_2 Bn), 73.7 (CH_2 Bn), 69.1 (CH_2 Bn), 69.0 (d, J = 20.1 Hz, CH_2*C*H_2F), 68.5 (C-6); Diagnostic signals of the minor stereoisomer (α product): ^{1}H NMR (500 MHz, $CDCl_3$, HH-COSY, HSQC): δ 5.82 (dd, J = 10.0, 9.2 Hz, 1H, H-3), 4.92 (d, J = 3.5 Hz, 1H, H-1); ^{13}C NMR (126 MHz, $CDCl_3$, HSQC): δ 165.6 (C=O Bz), 97.2 (C-1), 82.7 (d, J = 169.9 Hz, CH_2F), 68.2 (C-6), 67.3 (d, J = 20.2 Hz, *C*H_2CH_2F); HRMS: $[M+NH_4]^+$ calcd for $C_{36}H_{37}FO_7$ 618.28616, found 618.28601.

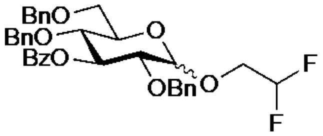

2,2-Difluoroethyl 3-*O*-benzoyl-2,4,6-tri-*O*-benzyl-α/β-D-glucopyranoside (S44). The title compound was prepared according to general procedure VII. Column chromatography (95:5 → 70:30, pentane:Et_2O) yielded the title compound (60 mg, 97 µmol, 97%, colourless oil, α:β; 58:42). TLC: R_f 0.10, (pentane:Et_2O, 80:20, v:v); IR (thin film, cm^{-1}): 696, 711, 743, 1027, 1070, 1090, 1268, 1452, 1720, 2871, 3031; Data of the major stereoisomer (α product): ^{1}H NMR (500 MHz, $CDCl_3$, HH-COSY, HSQC): δ 8.17 – 6.80 (m, 20H, CH_{arom}), 5.97 (tt, J = 55.5, 4.3 Hz, 1H, CHF_2), 5.78 (dd, J = 10.0, 9.1 Hz, 1H, H-3), 4.87 (d, J = 3.6 Hz, 1H, H-1), 4.69 – 4.35 (m, 6H, C*H*H Bn, CH*H* Bn, C*H*H Bn, CH*H* Bn, C*H*H Bn, CH*H* Bn), 3.93 – 3.65 (m, 6H, H-4, H-5, H-6, H-6, C*H*$HCHF_2$, CH*H*CHF_2), 3.63 (dd, J = 6.5, 3.5 Hz, 1H, H-2); ^{13}C NMR (126 MHz, $CDCl_3$, HSQC): δ 165.6 (C=O, Bz), 137.9, 137.7, 137.7, 137.4 ($C_{q\text{-}arom}$), 133.1, 131.2, 130.3, 129.9, 129.4, 128.6, 128.6, 128.4, 128.3, 128.2, 128.1, 128.1, 128.0, 127.9, 127.9, 127.7 (CH_{arom}), 114.2 (t, J = 242.0 Hz, CHF_2), 97.7 (C-1), 77.0 (C-2), 76.1 (C-3), 75.8 (C-4), 74.7, 73.8, 72.8 (CH_2 Bn), 70.5 (C-5), 68.1, (C-6) 67.5 (t, J = 29.1 Hz, *C*H_2CHF_2); Diagnostic signals of the minor stereoisomer (β product): ^{1}H NMR (500 MHz, $CDCl_3$, HH-COSY, HSQC): δ 5.49 (t, J = 9.4 Hz, 1H, H-3), 4.76 (d, J = 11.7 Hz, 1H, C*H*H Bn), 4.69 – 4.35 (m, 6H, CH*H* Bn, C*H*H Bn, CH*H* Bn, C*H*H Bn, CH*H* Bn, H-1), 4.05 (dtd, J = 18.6, 11.5, 3.3 Hz, 1H, C*H*$HCHF_2$), 3.50 (dd, J = 9.5, 7.6 Hz, 1H, H-2); ^{13}C NMR (126 MHz, $CDCl_3$, HSQC): δ 165.6 (C=O Bz), 114.2 (t, J = 242.0 Hz, CHF_2), 104.1 (C-1), 78.4 (C-2), 76.1 (C-3), 68.8 (dd, J = 29.8, 27.4 Hz, *C*H_2CHF_2), 68.3 (C-6); HRMS: $[M+NH_4]^+$ calcd for $C_{36}H_{36}F_2O_7$ 636.27674, found 636.27659.

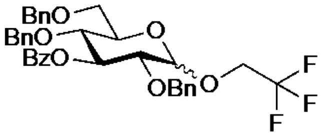

2,2,2-Trifluoroethyl 3-*O*-benzoyl-2,4,6-tri-*O*-benzyl-α/β-D-glucopyranoside (S45). The title compound was prepared according to general procedure VII. Column chromatography (95:5 → 70:30, pentane:Et_2O) yielded the title compound (58 mg, 91 µmol, 91%, colourless oil, α:β; 76:24). TLC: R_f 0.2, (pentane:Et_2O, 80:20, v:v); IR (thin film, cm^{-1}): 696, 711, 745, 1027, 1072, 1093, 1161, 1270, 1452, 1720, 2926, 3032; Data of the major stereoisomer (α product): ^{1}H NMR (500 MHz, $CDCl_3$, HH-COSY, HSQC): δ 8.11 – 6.77 (m, 20H, CH_{arom}), 5.79 (dd, J = 10.0, 8.8 Hz, 1H, H-3), 4.89 (d, J = 3.6 Hz, 1H, H-1), 4.68 – 4.42 (m, 5H, C*H*H Bn, CH*H* Bn, C*H*H Bn, CH*H* Bn, C*H*H Bn), 4.38 (d, J = 10.8 Hz, 1H, CH*H* Bn), 3.90 (q, J = 8.7 Hz, 2H, C*H*$_2CF_3$), 3.88 – 3.86 (m, 1H, H-5), 3.84 (dd, J = 10.1, 8.9 Hz, 1H, H-4), 3.77 (dd, J = 10.8, 3.0 Hz, 1H, H-6), 3.65 (dd, J = 10.1, 3.6 Hz, 1H, H-2), 3.64 (dd, J = 9.0, 5.4 Hz, 1H, H-6); ^{13}C NMR (126 MHz, $CDCl_3$, HSQC): δ 165.6 (C=O Bz), 137.9, 137.8, 137.7, 137.5 ($C_{q\text{-}arom}$), 130.3, 130.0,

128.6, 128.6, 128.5, 128.4, 128.3, 128.2, 128.2, 128.2, 128.1, 128.0, 128.0, 127.9, 127.9 (CH_{arom}), 123.7 (q, *J* = 279.0 Hz, CF_3) 97.8 (C-1), 76.8 (C-2), 75.7 (C-4), 74.7 (CH_2 Bn), 73.9 (C-3), 73.8 (CH_2 Bn), 72.7 (CH_2 Bn), 70.8 (C-5), 68.0 (C-6), 65.2 (q, *J* = 35.0 Hz, *C*H_2CF_3); Diagnostic signals of the minor stereoisomer (β product): ^{1}H NMR (500 MHz, $CDCl_3$, HH-COSY, HSQC): δ 5.49 (t, *J* = 9.4 Hz, 1H, H-3), 4.77 (d, *J* = 11.7 Hz, 1H, C*H*H Bn), 4.68 – 4.42 (m, 5H, C*H*H Bn, CH*H* Bn, C*H*H Bn, CH*H* Bn, C*H*H Bn), 4.60 (d, *J* = 7.6 Hz, 1H, H-1), 4.41 (d, *J* = 10.8 Hz, 1H, CH*H* Bn), 4.24 (dt, *J* = 12.1, 8.7 Hz, 1H, C*H*$_2CF_3$), 3.97 (dq, *J* = 12.1, 8.4 Hz, 1H, C*H*$_2CF_3$), 3.51 (dd, *J* = 9.5, 7.6 Hz, 1H, H-2); ^{13}C NMR (126 MHz, $CDCl_3$, HSQC): δ 165.6 (C=O Bz), 103.7 (C-1), 76.0 (C-3), 74.6 (CH_2 Bn), 73.9 (CH_2 Bn), 73.7 (CH_2 Bn), 68.3 (C-6), 66.3 (q, *J* = 35.1 Hz, *C*H_2CF_3); HRMS: $[M+NH_4]^+$ calcd for $C_{36}H_{35}F_3O_7$ 654.26731, found 654.26721.

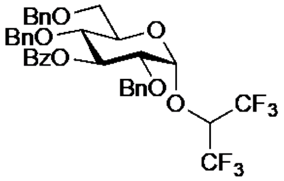

1,1,1,3,3,3-Hexafluoro-2-propyl 3-*O*-benzoyl-2,4,6-tri-*O*-benzyl-α-D-glucopyranoside (S46). The title compound was prepared according to general procedure VII. Column chromatography (100:0 → 80:20, pentane:Et_2O) yielded the title compound (20 mg, 28 µmol, 28%, colourless oil, α:β; >98:2). TLC: R_f 0.1, (pentane:Et_2O, 90:10, v:v); $[\alpha]_D^{25}$ 7.6° (*c* 1, $CHCl_3$); IR (thin film, cm^{-1}): 590, 867, 697, 710, 736, 1027, 1070, 1195, 1219, 1266, 1285, 1368, 1452, 1720, 2921, 3032; ^{1}H NMR (500 MHz, $CDCl_3$, HH-COSY, HSQC): δ 8.14 – 6.82 (m, 20H, CH_{arom}), 5.75 (t, *J* = 9.7 Hz, 1H, H-3), 5.21 (d, *J* = 3.8 Hz, 1H, H-1), 4.63 (d, *J* = 12.0 Hz, 1H, C*H*H Bn), 4.61 (d, *J* = 12.4 Hz, 1H, C*H*H Bn), 4.51 – 4.42 (m, 4H, C*H*H Bn, CH*H* Bn, CH*H* Bn, C*H*(CF_3)$_2$), 4.39 (d, *J* = 10.8 Hz, 1H, CH*H* Bn), 3.98 (dt, *J* = 10.0, 2.3 Hz, 1H, H-5), 3.85 (t, *J* = 9.7 Hz, 1H, H-4), 3.80 (dd, *J* = 10.9, 2.8 Hz, 1H, H-6), 3.71 (dd, *J* = 10.1, 3.8 Hz, 1H, H-2), 3.62 (dd, *J* = 10.9, 2.1 Hz, 1H, H-6); ^{13}C NMR (126 MHz, $CDCl_3$, HSQC): δ 137.6, 137.4 (C_{q-arom}), 133.3, 133.2, 130.0, 128.7, 128.5, 128.4, 128.3, 128.2, 128.2, 128.1, 127.9 (CH_{arom}), 99.3 (C-1), 75.8 (C-2), 75.4 (C-4), 74.9, 73.9 (CH_2 Bn), 73.6 (C-3), 72.5 (CH_2 Bn), 71.7 (C-5), 67.7 (C-6); HRMS: $[M+NH_4]^+$ calcd for $C_{37}H_{34}F_6O_7$ 722.25470, found 722.25415.

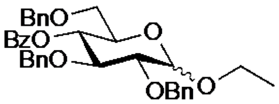

Ethyl 4-*O*-benzoyl-2,3,6-tri-*O*-benzyl-α/β-D-glucopyranoside (S47). The title compound was prepared according to general procedure VII. Column chromatography 95:5 → 80:20, pentane:Et_2O) yielded the title compound (44 mg, 76 µmol, 76%, colourless oil, α:β; 15:85). TLC: R_f 0.30, (pentane:Et_2O, 80:20, v:v); IR (thin film, cm^{-1}): 696, 711, 735, 1027, 1043, 1266, 1452, 1720, 2869, 3031; Data of the major stereoisomer (β product): ^{1}H NMR (500 MHz, $CDCl_3$, HH-COSY, HSQC): δ 8.00 – 7.00 (m, 20H, CH_{arom}), 5.24 (dd, *J* = 10.0, 9.3 Hz, 1H, H-4), 4.96 (d, *J* = 10.9 Hz, 1H, C*H*H Bn), 4.77 (d, *J* = 11.2 Hz, 1H, C*H*H Bn), 4.74 (d, *J* = 10.9 Hz, 1H, CH*H* Bn), 4.61 (d, *J* = 11.2 Hz, 1H, CH*H* Bn), 4.50 (d, *J* = 7.8 Hz, 1H, H-1), 4.47 (m, 2H, C*H*H Bn, CH*H* Bn), 4.11 – 3.98 (m, 1H, C*H*HCH_3 Et), 3.75 (t, *J* = 9.3 Hz, 1H, H-3), 3.72 – 3.59 (m, 4H, H-5, CH*H*CH_3 Et, H-6, H-6), 3.56 (dd, *J* = 9.2, 7.8 Hz, 1H, H-2), 1.31 (t, *J* = 7.1 Hz, 3H, CH_3 Et); ^{13}C NMR (126 MHz, $CDCl_3$, HSQC): δ 165.5 (C=O Bz), 138.5, 138.4, 138.3, 138.1, 138.0, 137.9 (C_{q-arom}), 133.3, 129.9, 129.8, 128.5, 128.5, 128.3, 128.3, 128.3, 128.3, 128.2, 128.1, 127.9, 127.8, 127.6, 127.6, 127.5 (CH_{arom}), 103.5 (C-1), 82.2 (C-2), 81.6 (C-3), 75.2, 75.1 (CH_2 Bn), 73.8 (C-5), 73.8 (CH_2 Bn), 71.6 (C-4), 70.0 (C-6), 65.9 (*C*H_2CH_3 Et), 15.5 (CH_3 Et); Diagnostic signals of the minor stereoisomer (α product): ^{1}H NMR (500 MHz, $CDCl_3$, HH-COSY, HSQC): δ 5.32 (dd, *J* = 10.3, 9.3 Hz, 1H, H-4), 4.85 (d, *J* = 11.2 Hz, 1H, C*H*H Bn), 4.79 (d, *J* = 3.6 Hz, 1H, H-1), 4.66 (d, *J* = 12.1 Hz, 1H, CH*H* Bn); ^{13}C NMR (126 MHz, $CDCl_3$, HSQC): δ 165.4 (C=O Bz), 96.7 (C-1),

79.8, 79.5 (CH_2 Bn), 71.3 (C-4), 69.2 (C-6), 63.7 (*C*H_2CH_3 Et), 15.1 (CH_3 Et); HRMS: $[M+NH_4]^+$ calcd for $C_{36}H_{38}O_7$ 600.29558, found 600.29548.

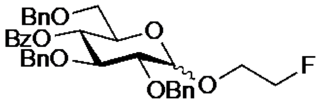

2-Fluoroethyl 4-*O*-benzoyl-2,3,6-tri-*O*-benzyl-α/β-D-glucopyranoside (S48). The title compound was prepared according to general procedure VII. Column chromatography (90:10 → 60:40, pentane:Et_2O) yielded the title compound (49 mg, 82 µmol, 82%, colourless oil, α:β; 34:66). TLC: R_f 0.2, (pentane:Et_2O, 70:30, v:v); IR (thin film, cm^{-1}): 696, 711, 740, 1027, 1042, 1090, 1268, 1452, 1720, 2867, 3030; Data of the major stereoisomer (β product): ^{1}H NMR (500 MHz, $CDCl_3$, HH-COSY, HSQC): 7.97 – 7.12 (m, 20H, CH_{arom}), 5.25 (dd, *J* = 10.0, 9.3 Hz, 1H, H-4), 4.98 (d, *J* = 11.8 Hz, 1H, C*H*H Bn), 4.87 – 4.81 (m, 1H, CH*H* Bn), 4.78 (d, *J* = 11.2 Hz, 1H, C*H*H Bn), 4.73 (d, *J* = 11.8 Hz, 1H, CH*H* Bn), 4.71 – 4.57 (m, 4H, C*H*H Bn, CH*H* Bn, CH_2C*H*HF, CH_2CH*H*F), 4.55 (d, *J* = 7.8 Hz, 1H, H-1), 4.22 – 3.78 (m, 2H, C*H*HCH_2F, CH*H*CH_2F), 3.76 (t, *J* = 9.2 Hz, 1H, H-3), 3.75 – 3.65 (m, 1H, H-5), 3.64 – 3.57 (m, 3H, H-6, H-6, H-2); ^{13}C NMR (126 MHz, $CDCl_3$, HSQC): δ 165.5 (C=O Bz), 138.3, 138.0, 137.9 ($C_{q\text{-}arom}$), 133.3, 133.2, 131.2, 129.9, 129.7, 129.4, 128.6, 128.5, 128.4, 128.3, 128.1, 128.0, 127.9, 127.8, 127.8, 127.8, 127.7, 127.6, 127.6, 127.5 (CH_{arom}), 103.8 (C-1), 81.7 (d, *J* = 73.4 Hz, CH_2F), 81.5 (C-2), 75.2, 75.1 (CH_2 Bn), 73.8 (C-5), 73.7 (CH_2 Bn), 71.3 (C-4), 69.8 (C-6), 67.0 (d, *J* = 15.2 Hz, *C*H_2CH_2F); Diagnostic signals of the minor stereoisomer (α product): ^{1}H NMR (500 MHz, $CDCl_3$, HH-COSY, HSQC): δ 5.35 (dd, *J* = 10.3, 9.3 Hz, 1H, H-4), 4.09 (t, *J* = 9.4 Hz, 1H, H-3), 4.84 – 4.80 (m, 2H, CH*H* Bn, H-1), 3.51 (dd, *J* = 10.9, 5.0 Hz, 1H, H-2); ^{13}C NMR (126 MHz, $CDCl_3$, HSQC): δ 165.4 (C=O Bz), 97.5 (C-1), 79.5 (d, *J* = 56.2 Hz, CH_2F), 71.0 (C-4), 67.4 (d, *J* = 19.9 Hz, *C*H_2CH_2F); HRMS: $[M+NH_4]^+$ calcd for $C_{36}H_{37}FO_7$ 618.28616, found 618.28582.

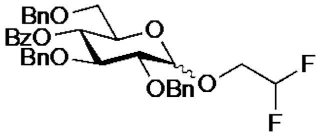

2,2-Difluoroethyl 4-*O*-benzoyl-2,3,6-tri-*O*-benzyl-α/β-D-glucopyranoside (S49). The title compound was prepared according to general procedure VII. Column chromatography (95:5 →70:30, pentane:Et_2O) yielded the title compound (43 mg, 70 µmol, 70%, colourless oil, α:β; 48:52). TLC: R_f 0.25, (pentane:Et_2O, 80:20, v:v); IR (thin film, cm^{-1}): 697, 711, 738, 1027, 1093, 1268, 1452, 1720, 2869, 3032; Data of the major stereoisomer (β product): ^{1}H NMR (500 MHz, $CDCl_3$, HH-COSY, HSQC): δ 8.20 – 7.03 (m, 20H, CH_{arom}), 6.04 (tt, *J* = 55.5, 4.5 Hz, 1H, CHF_2), 5.33 (dd, *J* = 10.3, 9.3 Hz, 1H, H-4), 4.86 – 4.59 (m, 6H, CH_2 Bn, CH_2 Bn, CH_2 Bn, CH_2 Bn, CH_2 Bn, CH_2 Bn), 4.53 (d, *J* = 7.8 Hz, 1H, H-1), 4.13 – 3.96 (m, 1H, C*H*HCHF_2), 3.91 – 3.65 (m, 2H, CH*H*CHF_2, H-5), 3.75 (t, *J* = 9.2 Hz, 1H, H-3), 3.64 – 3.53 (m, 3H, H-6, H-6, H-2); ^{13}C NMR (126 MHz, $CDCl_3$, HSQC): δ 165.4 (C=O Bz), 138.2, 138.2, 138.0, 137.9, 137.8, 137.7 ($C_{q\text{-}arom}$), 129.8, 129.7, 128.6, 128.5, 128.5, 128.4, 128.4, 128.4, 128.4, 128.3, 128.3, 128.3, , 128.0, 127.7, 127.6 (CH_{arom}), 114.2 (t, *J* = 241.3 Hz, CHF_2), 104.0 (C-1), 81.9 (C-2), 81.4 (C-3), 75.6 (CH_2 Bn), 75.3 (CH_2 Bn), 73.9 (C-5), 73.8 (CH_2 Bn), 71.2 (C-4), 68.8 (C-6), 67.7 (t, *J* = 28.8 Hz, *C*H_2CHF_2); Diagnostic signals of the minor stereoisomer (α product): ^{1}H NMR (500 MHz, $CDCl_3$, HH-COSY, HSQC): δ 6.0 (dddd, *J* = 56.0, 54.8, 5.3, 3.0 Hz, 1H, CHF_2), 5.3 (dd, *J* = 10.0, 9.3 Hz, 1H, H-4), 4.91 (d, *J* = 11.8 Hz, 1H, C*H*H Bn), 4.78 (d, *J* = 3.7 Hz, 1H, H-1), 4.04 (t, *J* = 9.5 Hz, 1H, H-3); ^{13}C NMR (126 MHz, $CDCl_3$, HSQC): δ 165.4 (C=O Bz), 114.2 (dd, *J* = 242.2, 239.9 Hz, CHF_2), 98.3 (C-1), 70.8 (C-4), 69.6 (C-6), 68.9 (dd, *J* = 30.4, 26.6 Hz, *C*H_2CHF_2); HRMS: $[M+NH_4]^+$ calcd for $C_{36}H_{36}F_2O_7$ 636.27674, found 636.27675.

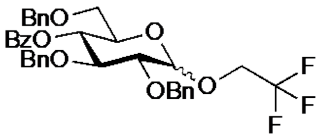

2,2,2-Trifluoroethyl 4-*O*-benzoyl-2,3,6-tri-*O*-benzyl-α/β-D-glucopyranoside (S50). The title compound was prepared according to general procedure VII. Column chromatography (95:5 → 80:20, pentane:Et_2O) yielded the title compound (49 mg, 76 µmol, 76%, colourless oil, α:β; 79:21). TLC: R_f 0.4, (pentane:Et_2O, 80:20, v:v); IR (thin film, cm^{-1}): 697, 711, 738, 1027, 1070, 1093, 1161, 1270, 1452, 1720, 2959, 2910, 3032; Data of the major stereoisomer (α product): ^{1}H NMR (500 MHz, $CDCl_3$, HH-COSY, HSQC): δ 8.07 – 6.88 (m, 20H, CH_{arom}), 5.35 (dd, *J* = 10.3, 9.3 Hz, 1H, H-4), 4.87 – 4.78 (m, 3H, C*H*H Bn, CH*H* Bn, H-1), 4.67 – 4.60 (m, 2H, C*H*H Bn, CH*H* Bn), 4.50 – 4.41 (m, 2H, C*H*H Bn, CH*H* Bn), 4.06 (t, *J* = 9.5 Hz, 1H, H-3), 4.00 – 3.87 (m, 3H, C*H*$_2$CF$_3$, H-5), 3.70 (dd, *J* = 9.6, 3.6 Hz, 1H, H-2), 3.55 (dd, *J* = 10.9, 2.7 Hz, 1H, H-6), 3.50 (dd, *J* = 10.9, 5.0 Hz, 1H, H-6); ^{13}C NMR (126 MHz, $CDCl_3$, HSQC): ^{13}C NMR (126 MHz, $CDCl_3$) δ 165.3 (C=O Bz), 138.2, 138.0, 137.7 ($C_{q\text{-}arom}$), 129.7, 128.6, 128.5, 128.4, 128.3, 128.2, 128.2, 128.0, 127.8, 127.7, 127.6, 127.2, 123.9 (q, *J* = 278.7 Hz, CF_3), 97.9 (C-1), 79.5 (C-2), 78.9 (C-3), 75.6, 73.8, 73.6 (CH_2 Bn), 70.6 (C-4), 69.9 (C-5), 68.7 (C-6), 64.9 (q, *J* = 34.9 Hz, *C*H$_2$CF$_3$); Diagnostic signals of the minor stereoisomer (β product): ^{1}H NMR (500 MHz, $CDCl_3$, HH-COSY, HSQC): δ 5.26 (dd, *J* = 10.0, 9.3 Hz, 1H, H-4), 4.93 (d, *J* = 10.6 Hz, 1H, C*H*H Bn), 4.60 (d, *J* = 7.1 Hz, 1H, H-1), 3.75 (t, *J* = 9.2 Hz, 1H, H-3); ^{13}C NMR (126 MHz, $CDCl_3$, HSQC): δ 103.6 (C-1), 81.7 (C-2), 81.2 (C-3), 71.0 (C-4) 69.4 (C-6); HRMS: $[M+NH_4]^+$ calcd for $C_{36}H_{35}F_3O_7$ 654.26731, found 654.26708.

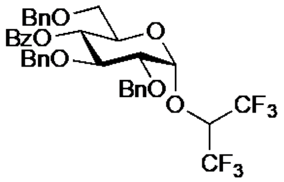

1,1,1,3,3,3-Hexafluoro-2-propyl 4-*O*-benzoyl-2,3,6-tri-*O*-benzyl-α-D-glucopyranoside (S51). The title compound was prepared according to general procedure VII. Column chromatography (100:0 → 80:20, pentane:Et_2O) yielded the title compound (13 mg, 19 µmol, 19%, colourless oil, α:β; >98:2). TLC: R_f 0.1, (pentane:Et_2O, 90:10, v:v); $[\alpha]_D^{25}$ 0.7° (*c* 1, $CHCl_3$); IR (thin film, cm^{-1}): 538, 688, 696, 711, 741, 1027, 1068, 1104, 1196, 1219, 1264, 1286, 1452, 1720, 2924, 3032; ^{1}H NMR (500 MHz, CDCl3, HH-COSY, HSQC): δ 8.42 – 6.93 (m, 20H, CH_{arom}), 5.42 (dd, *J* = 10.3, 9.4 Hz, 1H, H-4), 5.16 (d, *J* = 3.7 Hz, 1H, H-1), 4.82 (d, *J* = 11.1 Hz, 1H, C*H*H Bn), 4.74 (d, *J* = 11.6 Hz, 1H, C*H*H Bn), 4.70 (d, *J* = 11.6 Hz, 1H, CH*H* Bn), 4.61 (d, *J* = 11.1 Hz, 1H, CH*H* Bn), 4.55 – 4.47 (m, 2H, CH*H* Bn, C*H*(CF$_3$)$_2$), 4.42 (d, *J* = 12.0 Hz, 1H, CH*H* Bn), 4.13 – 4.05 (m, 1H, H-5), 4.04 (t, *J* = 9.6 Hz, 1H, H-3), 3.75 (dd, *J* = 9.8, 3.8 Hz, 1H, H-2), 3.55 (dd, *J* = 11.0, 2.6 Hz, 1H, H-6), 3.50 (dd, *J* = 11.0, 4.4 Hz, 1H, H-6); ^{13}C NMR (126 MHz, $CDCl_3$, HSQC): δ 165.3 (C=O Bz), 133.4, 131.2 ($C_{q\text{-}arom}$), 129.9, 129.5, 128.6, 128.6, 128.4, 128.3, 128.2, 128.2, 128.1, 127.9, 127.7, 127.7 (CH_{arom}), 99.5 (C-1), 77.2 (C-2), 76.9 (C-3), 75.6, 73.8, 73.7 (CH_2 Bn), 70.8 (C-5), 70.1 (C-4), 68.2 (C-6); HRMS: $[M+NH_4]^+$ calcd for $C_{37}H_{34}F_6O_7$ 722.25470, found 722.25434.

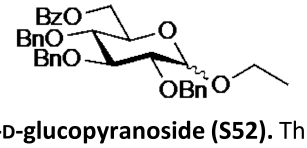

Ethyl 6-*O*-benzoyl-2,3,4-tri-*O*-benzyl-α/β-D-glucopyranoside (S52). The title compound was prepared according to general procedure VII. Column chromatography (95:5 → 75:25, pentane:Et_2O) yielded the title compound (55 mg, 94 µmol, 94%, colourless oil, α:β; 17:83). TLC: R_f 0.30, (pentane:Et_2O, 80:20, v:v); IR (thin film, cm^{-1}): 695, 711, 735, 1026, 1066, 1272, 1720, 2904, 3030; Data of the major stereoisomer (β product): ^{1}H NMR (500 MHz,

$CDCl_3$, HH-COSY, HSQC): δ 8.27 – 7.12 (m, 20H, CH_{arom}), 4.98 (d, *J* = 11.0 Hz, 1H, C*H*H Bn), 4.97 (d, *J* = 10.8 Hz, 1H, C*H*H Bn), 4.89 (d, *J* = 10.7 Hz, 1H, C*H*H Bn), 4.82 (d, *J* = 10.7 Hz, 1H, CH*H* Bn), 4.75 (d, *J* = 10.8 Hz, 1H, CH*H* Bn), 4.60 (m, 2H, CH*H* Bn, H-6), 4.54 – 4.42 (m, 2H, H-6, H-1), 3.98 (dq, *J* = 9.3, 7.2 Hz, 1H, CH_2CH_3 Et), 3.71 (t, *J* = 8.6 Hz, 1H, H-3), 3.71 – 3.55 (m, 3H, CH_2CH_3 Et, H-4, H-5), 3.49 (t, *J* = 8.3 Hz, 1H, H-2), 1.28 (t, *J* = 7.2 Hz, 3H, CH_2CH_3 Et); ^{13}C NMR (126 MHz, $CDCl_3$, HSQC): 13C NMR (126 MHz, CDCl3) δ 166.4 (C=O Bz), 133.2, 133.1, 130.2 ($C_{q\text{-}arom}$), 129.8, 129.8, 128.6, 128.6, 128.6, 128.5, 128.5, 128.3, 128.2, 128.2, 128.2, 128.1, 128.1, 127.9, 127.9 (CH_{arom}), 103.7 (C-1), 84.8 (C-3), 82.4 (C-2), 77.8 (C-4), 76.0, 75.3, 75.0 (CH_2 Bn), 73.1 (C-5), 65.9 (CH_2CH_3 Et), 63.7 (C-6), 15.5 (CH_3 Et); Diagnostic signals of the minor stereoisomer (α product): ^1H NMR (500 MHz, $CDCl_3$, HH-COSY, HSQC): δ 5.04 (d, *J* = 10.6 Hz, 1H, C*H*H Bn), 4.92 (d, *J* = 10.7 Hz, 1H, C*H*H Bn), 4.85 (d, *J* = 10.5 Hz, 1H, CH*H* Bn), 4.77 (signal overlaps with major isomer, 1H, H-1), 4.67 (d, *J* = 12.1 Hz, 1H, CH*H* Bn), 4.08 (t, *J* = 9.2 Hz, 1H, H-3); ^{13}C NMR (126 MHz, $CDCl_3$, HSQC): δ 96.5 (C-1), 82.3 (C-3), 80.3 (C-2), 77.8 (C-4), 76.1, 75.4, 73.4 (CH_2 Bn), 68.9 (C-5), 63.6 (CH_2CH_3 Et), 63.6 (C-6), 15.1 (CH_3 Et); HRMS: $[M+NH_4]^+$ calcd for $C_{36}H_{38}O_7$ 600.29558, found 600.29595.

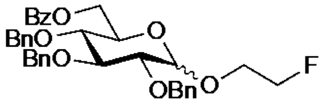

2-Fluoroethyl 6-*O*-benzoyl-2,3,4-tri-*O*-benzyl-α/β-D-glucopyranoside (S53). The title compound was prepared according to general procedure VII. Column chromatography (90:10 → 60:40, pentane:Et_2O) yielded the title compound (50 mg, 83 µmol, 83%, colourless oil, α:β; 35:65). TLC: R_f 0.2, (pentane:Et_2O, 70:30, v:v); IR (thin film, cm^{-1}): 696, 712, 736, 1026, 1067, 1154, 1273, 1452, 1720; Data of the major stereoisomer (β product): ^1H NMR (500 MHz, $CDCl_3$, HH-COSY, HSQC): δ 8.12 – 6.99 (m, 20H, CH_{arom}), 4.98 (m, 2H, C*H*H Bn, C*H*H Bn), 4.87 (m, 1H, CH*H* Bn), 4.85 – 4.78 (m, 3H, C*H*H Bn, CH*H* Bn, CH*H* Bn), 4.69 – 4.48 (m, 4H, H-6, H-6, CH_2CHHF, CH_2CHHF), 4.50 (d, *J* = 7.7 Hz, 1H, H-1), 4.13 – 4.00 (m, 1H, C*H*HCH_2F), 3.92 – 3.75 (m, 1H, CH*H*CH_2F), 3.72 (t, *J* = 8.8 Hz, 1H, H-3), 3.69 – 3.58 (m, 2H, H-4, H-5), 3.53 (dd, *J* = 8.9, 7.8 Hz, 1H, H-2); ^{13}C NMR (126 MHz, $CDCl_3$, HSQC): δ 166.3 (C=O Bz), 138.5, 138.4, 137.7 ($C_{q\text{-}arom}$), 129.8, 128.6, 128.6, 128.6, 128.5, 128.5, 128.5, 128.3, 128.1, 128.1, 127.9 (CH_{arom}), 104.0 (C-1), 84.7 (C-3), 82.7 (d, *J* = 169.9 Hz, CH_2F) 82.2 (C-2), 77.6 (C-4), 76.0, 75.3, 73.2 (CH_2 Bn), 69.1 (d, *J* = 19.8 Hz, CH_2CH_2F), 63.5 (C-6); Diagnostic signals of the minor stereoisomer (α product): ^1H NMR (500 MHz, $CDCl_3$, HH-COSY, HSQC): δ 5.03 (d, *J* = 10.6 Hz, 1H, C*H*H Bn), 4.81 (d, *J* = 2.0 Hz, 1H, H-1), 4.08 (t, *J* = 9.4 Hz, 1H, H-3); ^{13}C NMR (126 MHz, $CDCl_3$, HSQC): 97.3 (C-1), 82.6 (d, *J* = 170.2 Hz, CH_2F), 67.2 (d, *J* = 20.2 Hz, CH_2CH_2F), 63.5 (C-6); HRMS: $[M+NH_4]^+$ calcd for $C_{36}H_{37}FO_7$ 618.28616, found 618.28612.

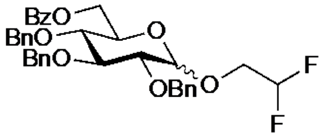

2,2-Difluoroethyl 6-*O*-benzoyl-2,3,4-tri-*O*-benzyl-α/β-D-glucopyranoside (S54). The title compound was prepared according to general procedure VII. Column chromatography (95:5 → 75:25, pentane:Et_2O) yielded the title compound (51 mg, 82 µmol, 82%, colourless oil, α:β; 77:23). TLC: R_f 0.30, (pentane:Et_2O, 80:20, v:v); IR (thin film, cm^{-1}): 696, 711, 737, 1274, 1452, 1720, 2917, 3032; Data of the major stereoisomer (α product): ^1H NMR (500 MHz, $CDCl_3$, HH-COSY, HSQC): δ 8.07 – 7.04 (m, 20H, CH_{arom}), 5.97 (tt, *J* = 55.5, 4.3 Hz, 1H, CHF_2), 5.01 (d, *J* = 10.6 Hz, 1H, C*H*H Bn), 4.92 (d, *J* = 10.8 Hz, 1H, C*H*H Bn), 4.85 (d, *J* = 10.6 Hz, 1H, CH*H* Bn), 4.81 (d, *J* = 11.9 Hz, 1H CH*H* Bn), 4.75 (d, *J* = 3.6 Hz, 1H, H-1), 4.68 – 4.59 (m, 2H, C*H*H Bn, CH*H* Bn), 4.54 (dd, *J* = 12.0, 2.2 Hz, 1H, H-6), 4.52 – 4.43 (m, 1H, H-6), 4.04 (t, *J* = 9.3 Hz, 1H, H-3), 4.00 (ddd, *J* = 10.2, 4.7, 2.1 Hz, 1H, H-5), 3.86 – 3.56

(m, 4H, H-2, H-4, C*H*HCHF$_2$, CH*H*CHF$_2$); ^{13}C NMR (126 MHz, CDCl$_3$, HSQC): δ 166.3 (C=O Bz), 138.6, 138.1, 137.7 (C$_{q\text{-arom}}$), 129.7, 128.7, 128.6, 128.6, 128.5, 128.4, 128.2, 128.2, 128.2, 127.9 (CH$_{arom}$), 114.1 (t, *J* = 241.4 Hz, CHF$_2$), 97.8 (C-1), 81.9 (C-3), 80.1 (C-2), 77.5 (C-4), 76.1, 75.4, 73.7 (CH$_2$ Bn), 69.5 (C-5), 67.3 (t, *J* = 28.8 Hz, *C*H$_2$CHF$_2$), 63.4 (C-6); Diagnostic signals of the minor stereoisomer (β product): ^{1}H NMR (500 MHz, CDCl$_3$, HH-COSY, HSQC): δ 6.12 – 5.64 (m, 1H CHF$_2$), 4.97 (d, *J* = 10.8 Hz, 1H, C*H*H Bn), 4.73 (d, *J* = 10.9 Hz, 1H, CH*H* Bn), 4.49 (d, *J* = 7.8 Hz, 1H, H-1), 3.51 (dd, *J* = 9.0, 7.7 Hz, 1H, H-2); ^{13}C NMR (126 MHz, CDCl$_3$, HSQC): δ 104.2 (C-1), 84.6 (C-3), 82.1 (C-2), 76.0, 75.3, 75.1 (CH$_2$ Bn), 68.9 (dd, *J* = 29.8, 27.3 Hz, *C*H$_2$CHF$_2$), 63.3 (C-6); HRMS: [M+NH$_4$]$^+$ calcd for $C_{36}H_{36}F_2O_7$ 636.27674, found 636.27645.

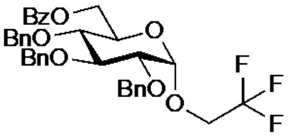

2,2,2-Trifluoroethyl 3-*O*-benzoyl-2,3,4-tri-*O*-benzyl-α-D-glucopyranoside (S55). The title compound was prepared according to general procedure VII. Column chromatography (95:5 → 80:20, pentane:Et$_2$O) yielded the title compound (52 mg, 82 µmol, 82%, colourless oil, α:β; 95:5). TLC: R$_f$ 0.45, (pentane:Et$_2$O, 80:20, v:v); $[\alpha]_D^{25}$ 40.2° (*c* 1, CHCl$_3$); IR (thin film, cm^{-1}): 696, 711, 736, 1027, 1070, 1273, 1452, 1720, 2920, 3032; Data of the major stereoisomer (α product): ^{1}H NMR (500 MHz, CDCl$_3$, HH-COSY, HSQC): δ 8.71 – 6.95 (m, 20H, CH$_{arom}$), 5.02 (d, *J* = 10.6 Hz, 1H, C*H*H Bn), 4.92 (d, *J* = 10.8 Hz, 1H, C*H*H Bn), 4.85 (d, *J* = 10.6 Hz, 1H, CH*H* Bn), 4.85 (d, *J* = 3.6 Hz, 1H, H-1) 4.82 (m, 1H, CH*H* Bn), 4.64 (m, 1H, C*H*H Bn, CH*H* Bn) 4.54 (dd, *J* = 12.0, 2.2 Hz, 1H, H-6), 4.47 (dd, *J* = 12.1, 4.5 Hz, 1H, H-6), 4.05 (dd, *J* = 9.7, 8.9 Hz, 1H, H-3), 3.97 (ddd, *J* = 10.1, 4.5, 2.2 Hz, 1H, H-5), 3.89 (qd, *J* = 8.7, 7.0 Hz, 2H, C*H*$_2$CF$_3$), 3.63 (t, *J* = 10.0 Hz, 1H, H-4), 3.60 (dd, *J* = 9.6, 3.7 Hz, 1H, H-2); ^{13}C NMR (126 MHz, CDCl$_3$, HSQC): δ 166.2 (C=O Bz), 138.6, 138.1, 137.7 (C$_{q\text{-arom}}$), 129.9, 129.7, 128.7, 128.7, 128.6, 128.6, 128.6, 128.5, 128.3, 128.2, 128.2, 128.2, 128.2, 128.1, 128.1, 128.0, 127.1 (CH$_{arom}$), 123.8 (q, *J* = 278.8 Hz, CF$_3$), 97.7 (C-1), 81.7 (C-3), 80.0 (C-2), 77.3 (C-4), 76.1, 75.4, 73.5 (CH$_2$ Bn), 65.3 (C-5), 64.9 (q, *J* = 35.0 Hz, *C*H$_2$CF$_3$), 63.2 (C-6); Diagnostic signals of the minor stereoisomer (β product): ^{1}H NMR (500 MHz, CDCl$_3$, HH-COSY, HSQC): 1H NMR (500 MHz, Chloroform-d) δ 5.06 (d, *J* = 10.7 Hz, 1H, C*H*H Bn), 4.70 (d, *J* = 10.6 Hz, 1H, CH*H* Bn), 4.18 (dq, *J* = 12.3, 8.8 Hz, 1H, C*H*$_2$CF$_3$), 3.53 (dd, *J* = 8.7, 7.7 Hz, 1H, H-2); ^{13}C NMR (126 MHz, CDCl$_3$, HSQC): 103.8 (C-1), 84.5 (C-3), 81.9 (C-2); HRMS: [M+NH$_4$]$^+$ calcd for $C_{36}H_{35}F_3O_7$ 654.26731, found 654.26723.

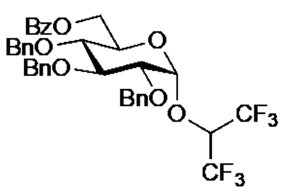

1,1,1,3,3,3-Hexafluoro-2-propyl 6-*O*-benzoyl-2,3,4-tri-*O*-benzyl-α-D-glucopyranoside (S56). The title compound was prepared according to general procedure VII. Column chromatography (100:0 → 80:20, pentane:Et$_2$O) yielded the title compound (21 mg, 30 µmol, 30%, colourless oil, α:β; >98:2). TLC: R$_f$ 0.1, (pentane:Et$_2$O, 90:10, v:v); $[\alpha]_D^{25}$ 49.0° (*c* 1, CHCl$_3$); IR (thin film, cm^{-1}): 595, 698, 712, 750, 1027, 1105, 1198, 1278, 1454, 1720, 2933, 3035; ^{1}H NMR (500 MHz, CDCl$_3$, HH-COSY, HSQC): δ 8.07 – 7.16 (m, 20H, CH$_{arom}$), 5.15 (d, *J* = 3.8 Hz, 1H, H-1), 5.00 (d, *J* = 10.6 Hz, 1H, C*H*H Bn), 4.93 (d, *J* = 10.7 Hz, 1H, C*H*H Bn), 4.85 (d, *J* = 10.6 Hz, 1H, CH*H* Bn), 4.76 – 4.66 (m, 2H, C*H*H Bn, CH*H* Bn), 4.63 (d, *J* = 10.7 Hz, 1H, CH*H* Bn), 4.56 (dd, *J* = 12.1, 2.1 Hz, 1H, H-6), 4.46 (m, 2H, H-6, C*H*(CF$_3$)$_2$), 4.09 (ddd, *J* = 10.2, 4.3, 2.1 Hz, 1H, H-5), 4.04 (dd, *J* = 9.8, 9.0 Hz, 1H, H-3), 3.78 – 3.59 (m, 2H, H-4, H-2); ^{13}C NMR (126 MHz, CDCl$_3$, HSQC): δ 166.2 (C=O Bz), 138.4, 137.6, 137.5 (C$_{q\text{-arom}}$),

133.4, 129.8, 129.7, 128.7, 128.6, 128.6, 128.3, 128.3, 128.3, 128.2, 128.2, 128.2, 128.0, 127.7 (CH_{arom}), 99.2 (C-1), 81.2 (C-3), 79.2 (C-2), 77.4 (C-4), 76.9, 76.1, 73.7 (CH_2 Bn), 72.9 (p, *J* = 33.6 Hz, *C*H(CF_3)$_2$), 62.9 (C-6); HRMS: $[M+NH_4]^+$ calcd for C37H34F6O7 722.25470, found 722.25454.

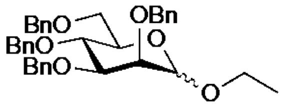

Ethyl 2,3,4,6-tetra-*O*-benzyl-α/β-D-mannopyranoside (S57). The title compound was prepared according to general procedure VII. Column chromatography (100:0 → 90:10, pentane:EtOAc) yielded the title compound (40 mg, 70 µmol, 70%, colourless oil, α:β; 33:67). TLC: R_f 0.25 (pentane:EtOAc, 90:10, v:v); Data of the major stereoisomer (β product): ^{1}H NMR (400 MHz, $CDCl_3$, HH-COSY, HSQC): δ 7.50 – 7.14 (m, 20H, CH_{arom}), 4.99 (d, *J* = 12.5 Hz, 1H, C*H*H Bn), 4.93 – 4.84 (m, 2H, C*H*H Bn, CH*H* Bn), 4.69 – 4.58 (m, 4H, C*H*H Bn, CH*H* Bn, C*H*H Bn, CH*H* Bn), 4.44 (d, J = 11.8 Hz, 1H, CH*H* Bn), 4.38 (s, 1H, H-1), 4.07 – 3.97 (m, 1H, C*H*HCH$_3$ Et), 3.97 – 3.67 (m, 5H, CH*H*CH$_3$ Et, H-2, H-4, H-6, H-6), 3.57 – 3.36 (m, 3H, CH*H*CH$_3$ Et, H-5, H-3), 1.27 (t, *J* = 7.0 Hz, 3H, CH_3 Et); ^{13}C NMR (101 MHz, $CDCl_3$, HSQC): δ 139.0, 138.6, 138.5, 138.3 ($C_{q\text{-}arom}$), 128.6, 128.5, 128.4, 128.4, 128.2, 128.2, 128.0, 127.9, 127.9, 127.8, 127.7, 127.7, 127.6, 127.6, 127.6, 127.5 (CH_{arom}), 101.6 (C-1), 82.5 (C-3), 76.1 (C-5), 75.3 (CH_2 Bn), 75.1 (C-4), 73.9 (CH_2 Bn), 73.9 (H-2), 73.6 (CH_2 Bn), 71.5 (CH_2 Bn), 69.9 (C-6), 65.4 (*C*H$_2$CH$_3$ Et), 15.4 (CH_3 Et); ^{13}C-GATED NMR (101 MHz, $CDCl_3$): δ 101.6 ($J_{H1\text{-}C1}$ = 153 Hz, β); Diagnostic signals of the minor stereoisomer (α product): ^{1}H NMR (400 MHz, $CDCl_3$, HH-COSY, HSQC): δ 4.93 – 4.84 (signals overlap with major isomer, 1H, H-1), 4.76 (d, *J* = 12.5 Hz, 1H, C*H*H Bn), 4.72 (d, *J* = 12.5 Hz, 1H, CH*H* Bn), 1.15 (t, *J* = 7.1 Hz, 3H, CH_3 Et); ^{13}C NMR (101 MHz, $CDCl_3$, HSQC): 97.8 (C-1), 80.5 (C-3), 69.5 (C-6), 63.0 (*CH$_2$*CH$_3$ Et), 15.1 (CH_3 Et); ^{13}C-GATED NMR (101 MHz, $CDCl_3$): δ 97.8 ($J_{H1\text{-}C1}$ = 168 Hz, α); HRMS: $[M+Na]^+$ calcd for $C_{36}H_{40}O_6Na$ 591.27171, found 591.27096.

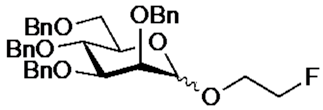

2-Fluoroethyl 2,3,4,6-tetra-*O*-benzyl-α/β-D-mannopyranoside (S58). The title compound was prepared according to general procedure VII. Column column chromatography (97:3 → 90:10, pentane:EtOAc) yielded the title compound (44 mg, 75 µmol, 75%, colourless oil, α:β; 60:40). TLC: R_f 0.24, 0.34 (pentane:EtOAc, 85:15, v:v); IR (thin film, cm^{-1}): 695, 734, 1026, 1073, 1362, 1453, 1496, 2910; Data of the major stereoisomer (α product): ^{1}H NMR (400 MHz, $CDCl_3$, HH-COSY, HSQC): 7.57 – 7.07 (m, 20H, CH_{arom}), 4.98 – 4.83 (m, 2H, C*H*H Bn, CH*H* Bn), 4.93 (d, *J* = 1.9 Hz, 1H, H-1), 4.76 (d, *J* = 12.4 Hz, 1H, C*H*H Bn), 4.72 (d, *J* = 12.5 Hz, 1H, CH*H* Bn), 4.68 – 4.39 (m, 6H, C*H*H Bn, CH*H* Bn, C*H*H Bn, CH*H* Bn, CH$_2$C*H*HF, CH$_2$CH*H*F), 4.04 – 3.59 (m, 12H, H-2, H-3, H-4, H-5, H-6, H-6, C*H*HCH$_2$F, CH*H*CH$_2$F); ^{13}C NMR (101 MHz, $CDCl_3$, HSQC): δ 138.7, 138.6, 138.4, 138.4 ($C_{q\text{-}arom}$), 128.5, 128.4, 128.4, 128.1, 127.7, 127.6, 127.6, 127.6, 127.6, 127.5 (CH_{arom}), 98.2 (C-1), 82.6 (d, *J* = 169.8 Hz, CH_2F), 80.2 (C-3), 75.3 (CH_2 Bn), 74.9 (C-2), 74.6 (C-4), 73.5, 72.8, 72.2 (CH_2 Bn), 71.9 (C-5), 69.2 (C-6), 66.6 (d, *J* = 19.8 Hz, *C*H$_2$CH$_2$F); ^{13}C-HMBC-GATED NMR (101 MHz, $CDCl_3$): δ 98.2 ($J_{H1\text{-}C1}$ = 170 Hz, α); Diagnostic signals of the minor stereoisomer (β product): ^{1}H NMR (400 MHz, $CDCl_3$, HH-COSY, HSQC): δ 4.98 (d, *J* = 12.4 Hz, 1H, C*H*H Bn), 4.45 (s, 1H, H-1), 4.11 (dddd, *J* = 35.8, 12.1, 3.9, 2.3 Hz, 1H, C*H*HCH$_2$F), 3.50 (dd, *J* = 9.4, 3.0 Hz, 1H, H-3), 3.46 (ddd, *J* = 9.7, 5.8, 2.1 Hz, 1H, H-5); ^{13}C NMR (101 MHz, $CDCl_3$, HSQC): δ 101.7 (C-1), 83.0 (d, *J* = 169.3 Hz, CH_2F), 82.2 (C-3), 69.7 (C-6), 68.7 (d, *J* = 19.7 Hz, *C*H$_2$CH$_2$F); ^{13}C-HMBC-GATED NMR (101 MHz, $CDCl_3$): δ 98.2 ($J_{H1\text{-}C1}$ = 153 Hz, β); HRMS: $[M+Na]^+$ calcd for $C_{36}H_{39}FO_6$ 609.2628, found 609.2635.

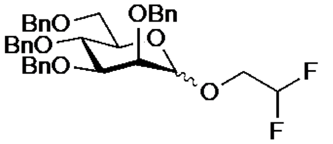

2,2-Difluoroethyl 2,3,4,6-tetra-*O*-benzyl-α/β-D-mannopyranoside (S59). The title compound was prepared according to general procedure VII. Column chromatography (97:3 → 90:10, pentane:EtOAc) yielded the title compound (39 mg, 65 µmol, 65%, colourless oil, α:β; 80:20). TLC: R_f 0.19, 0.31 (pentane:EtOAc, 90:10, v:v); IR (thin film, cm^{-1}): 695, 734, 1027, 1064, 1363, 1453, 1496, 2916; Data of the major stereoisomer (α product): ^1H NMR (400 MHz, $CDCl_3$, HH-COSY, HSQC): δ 7.71 – 7.10 (m, 20H, CH_{arom}), 5.86 (tdd, *J* = 55.3, 4.8, 3.4 Hz, 1H, CHF_2), 4.95 – 4.80 (m, 3H, C*H*H Bn, CH*H* Bn, C*H*H Bn), 4.90 (d, *J* = 2.0 Hz, 1H, H-1), 4.76 (d, *J* = 12.4 Hz, 1H, CH*H* Bn), 4.70 (d, *J* = 12.4 Hz, 1H, C*H*H Bn), 4.68 – 4.38 (m, 4H, C*H*H Bn, CH*H* Bn, C*H*H Bn, CH*H* Bn), 3.98 (t, *J* = 9.1 Hz, 1H, H-4), 4.13 – 3.61 (m, 7H, H-2, H-3, H-5, H-6, H-6, C*H*HCHF$_2$, CH*H*CHF$_2$); ^{13}C NMR (101 MHz, $CDCl_3$, HSQC): δ 138.6, 138.5, 138.4, 138.3 ($C_{q\text{-}arom}$), 129.5, 128.5, 128.5, 128.0, 127.9, 127.8, 127.8, 127.8, 127.7, 127.7, 127.6 (CH_{arom}), 114.1 (t, *J* = 241.1 Hz, CHF_2), 99.0 (C-1), 80.0 (C-3), 75.3 (C-4), 74.8 (C-2), 74.5, 73.5, 72.9, 72.4 (CH_2 Bn), 72.4 (C-5), 69.2 (C-6), 66.8 (t, *J* = 28.2 Hz, *C*H$_2$CHF$_2$); ^{13}C-HMBC-GATED NMR (101 MHz, $CDCl_3$): δ 99.0 ($J_{H1\text{-}C1}$ = 171 Hz, α); Diagnostic signals of the minor stereoisomer (β product): ^1H NMR (400 MHz, $CDCl_3$, HH-COSY, HSQC): δ 5.94 (dddd, *J* = 56.3, 54.7, 5.6, 2.7 Hz, 1H, CHF_2), 4.94 (d, *J* = 12.4 Hz, 1H, C*H*H Bn), 4.68 – 4.38 (signals overlap with major isomer, 1H, H-1), 3.50 (dd, *J* = 9.3, 3.0 Hz, 1H, H-3), 3.45 (ddd, *J* = 9.8, 5.8, 2.2 Hz, 1H, H-5); ^{13}C NMR (101 MHz, $CDCl_3$, HSQC): δ 114.4 (dd, *J* = 242.1, 240.0 Hz, CHF_2), 101.8 (C-1), 82.1, 68.5 (dd, *J* = 30.6, 26.2 Hz, *C*H$_2$CHF$_2$); ^{13}C-HMBC-GATED NMR (101 MHz, $CDCl_3$): δ 101.8 ($J_{H1\text{-}C1}$ = 153 Hz, β); HRMS: $[M+Na]^+$ calcd for $C_{36}H_{38}F_2O_6$ 627.2534, found 627.2541.

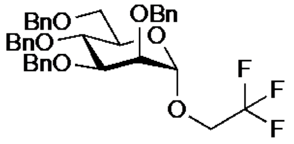

2,2,2-Trifluoroethyl 2,3,4,6-tetra-*O*-benzyl-α-D-mannopyranoside (S60). The title compound was prepared according to general procedure VII. Column chromatography (97:3 → 90:10, pentane:EtOAc) yielded the title compound (52 mg, 84 µmol, 84%, colourless oil, α:β; >98:2). TLC: R_f 0.45 (pentane:EtOAc, 90:10, v:v); $[\alpha]_D^{20}$ –16.6° (*c* 1, $CHCl_3$); IR (thin film, cm^{-1}): 667, 695, 985, 1069, 1165, 1279, 1362, 1454, 2867; ^1H NMR (400 MHz, $CDCl_3$, HH-COSY, HSQC): δ 8.07 – 5.74 (m, 20H, CH_{arom}), 4.94 (d, *J* = 1.9 Hz, 1H, H-1), 4.86 (d, *J* = 10.7 Hz, 1H, C*H*H Bn), 4.76 (d, *J* = 12.4 Hz, 1H, C*H*H Bn), 4.69 (d, *J* = 12.3 Hz, 1H, CH*H* Bn), 4.69 – 4.59 (m, 3H, CH*H* Bn, C*H*H Bn, C*H*H Bn), 4.53 (d, *J* = 12.2 Hz, 1H, CH*H* Bn), 4.49 (d, *J* = 10.7 Hz, 1H, CH*H* Bn), 4.00 (t, *J* = 9.1 Hz, 1H, H-4), 3.96 – 3.64 (m, 7H, H-2, H-3, H-5, H-6, H-6, C*H*HCF$_3$, CH*H*CF$_3$); ^{13}C NMR (101 MHz, $CDCl_3$, HSQC): δ 138.4, 138.3, 138.3, 138.1 ($C_{q\text{-}arom}$), 128.5, 128.5, 128.5, 128.1, 128.1, 127.9, 127.8, 127.8, 127.8, 127.8, 127.7, 127.7, 127.7 (CH_{arom}), 122.4 (q, *J* = 277.6 Hz, CF_3), 98.7 (C-1), 79.8 (C-3), 75.3 (CH_2 Bn), 74.6 (C-4), 74.4 (C-2), 73.6, 73.5 (CH_2 Bn), 73.1 (C-5), 72.7 (CH_2 Bn), 72.5 (C-6), 64.1 (q, *J* = 34.8 Hz, *C*H$_2$CF$_3$); HRMS: $[M+Na]^+$ calcd for $C_{36}H_{37}F_3O_6$ 645.2440, found 645.2452.

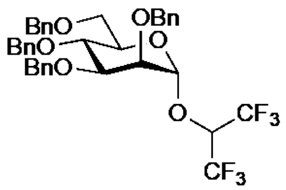

1,1,1,3,3,3-Hexafluoro-2-propyl 2,3,4,6-tetra-*O*-benzyl-α-D-mannopyranoside (S61). The title compound was prepared according to general procedure VII. Column chromatography (100:0 → 80:20, pentane:Et_2O) yielded the title compound (26 mg, 39 µmol, 39%, colourless oil, α:β; >98:2). TLC: R_f 0.23 (pentane:EtOAc, 95:5, v:v); $[\alpha]_D^{20}$ 35.7° (*c* 1, $CHCl_3$); IR (thin film, cm^{-1}): 695, 734, 900, 964, 1027, 1101, 1195, 1219, 1288, 1367, 1454, 2917; ^1H NMR (500 MHz, $CDCl_3$, HH-COSY, HSQC, HMBC-Gated, ^1H-^{19}F Decoupled) δ 7.38 – 7.12 (m, 20H, CH_{arom}), 5.07 (d, *J* = 1.8 Hz, 1H, H-1), 4.81 (d, *J* = 10.7 Hz, 1H, C*H*H Bn), 4.75 (d, *J* = 12.3 Hz, 1H, C*H*H Bn), 4.69 – 4.60 (m, 4H, C*H*H Bn, CH*H* Bn, C*H*H Bn, CH*H* Bn), 4.50 – 4.39 (m, 3H, CH*H* Bn, CH*H* Bn, C*H*(CF_3)$_2$), 4.05 (td, *J* = 9.8, 1.4 Hz, 1H, H-4), 3.87 – 3.79 (m, 3H, H-2, H-3, H-5), 3.77 (dd, *J* = 10.9, 4.5 Hz, 1H, H-6), 3.65 (dd, *J* = 10.9, 1.9 Hz, 1H, H-6); ^{13}C NMR (126 MHz, $CDCl_3$, HSQC, HMBC-Gated) δ 138.3, 138.3, 138.2, 137.8 ($C_{q\text{-}arom}$), 128.6, 128.6, 128.5, 128.5, 128.2, 128.1, 128.0, 127.9, 127.9, 127.7 (CH_{arom}), 100.7 (C-1), 79.2 (C-3), 75.1 (CH_2 Bn), 74.5 (C-2), 74.3 (C-4), 73.5 (C-5), 73.5, 73.3, 72.8 (CH_2 Bn), 72.1 (p, *J* = 32.7 Hz, *C*H(CF_3)$_2$), 68.7 (C-6); ^{13}C-GATED NMR (126 MHz, $CDCl_3$) δ 100.7 ($J_{H1\text{-}C1}$ = 174 Hz, α); HRMS: $[M+Na]^+$ calcd for $C_{37}H_{36}F_6O_6$ 713.2314, found 713.2335.

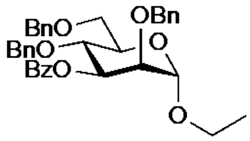

Ethyl 2,3,4,6-tetra-*O*-benzyl-α-D-mannopyranoside (S62). The title compound was prepared according to general procedure VII. Column chromatography (95:5 → 75:25, pentane:Et_2O) yielded the title compound (55 mg, 94 µmol, 94%, colourless oil, α:β; >98:2). TLC: R_f 0.30, (pentane:Et_2O, 80:20, v:v); $[\alpha]_D^{25}$ –3.6° (*c* 1, $CHCl_3$); IR (thin film, cm^{-1}): 695, 735, 1026, 1059, 1093, 1268, 1452, 1720, 2867, 3030; ^1H NMR (500 MHz, $CDCl_3$, HH-COSY, HSQC): δ 8.67 – 6.65 (m, 20H, CH_{arom}), 5.55 (dd, *J* = 9.6, 3.3 Hz, 1H, H-3), 4.93 (d, *J* = 1.9 Hz, 1H, H-1), 4.72 (d, *J* = 12.1 Hz, 1H, C*H*H Bn), 4.69 – 4.64 (m, 2H, C*H*H Bn, C*H*H Bn), 4.59 (d, *J* = 12.3 Hz, 1H, CH*H* Bn), 4.54 (d, *J* = 12.1 Hz, 1H, CH*H* Bn), 4.50 (d, *J* = 10.8 Hz, 1H, CH*H* Bn), 4.22 (t, *J* = 9.7 Hz, 1H, H-4), 3.97 (dd, *J* = 3.4, 1.9 Hz, 1H, H-2), 3.91 (ddd, *J* = 9.8, 4.5, 1.9 Hz, 1H, H-5), 3.83 (dd, *J* = 10.8, 4.5 Hz, 1H, H-6), 3.81 – 3.70 (m, 2H, H-6, C*H*HCH_3 Et), 3.49 (dq, *J* = 9.7, 7.1 Hz, 1H, CH*H*CH_3 Et), 1.20 (t, *J* = 7.1 Hz, 3H, CH_3 Et); ^{13}C NMR (126 MHz, $CDCl_3$, HSQC): δ 165.8 (C=O Bz), 138.4, 138.1, 138.0 ($C_{q\text{-}arom}$), 130.2, 128.5, 128.4, 128.4, 128.0, 127.8, 127.8, 127.7, 127.7, 127.7, 127.7 (CH_{arom}), 97.8 (C-1), 76.4 (C-2), 75.0 (CH_2 Bn), 74.8 (C-3), 73.8 (C-4), 73.6, 73.1 (CH_2 Bn), 71.6 (C-5), 69.2 (C-6), 63.3 (*C*H_2CH_3 Et), 15.1 (CH_3 Et); HRMS: $[M+NH_4]^+$ calcd for $C_{36}H_{38}O_7$ 600.29558, found 600.29561.

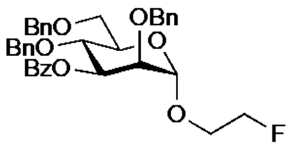

2-Fluoroethyl 3-*O*-benzoyl-2,4,6-tri-*O*-benzyl-α-D-mannopyranoside (S63). The title compound was prepared according to general procedure VII. Column chromatography (90:10 → 60:40, pentane:Et_2O) yielded the title compound (52 mg, 87 µmol, 87%, colourless oil, α:β; >98:2). TLC: R_f 0.20, (pentane:Et_2O, 70:30, v:v); $[\alpha]_D^{25}$ –2.6° (*c* 1, $CHCl_3$); IR (thin film, cm^{-1}): 539, 697, 714, 738, 1026, 2046, 170, 1270, 1452 1496, 1601, 1720, 2914, 3031; ^1H NMR (500 MHz, $CDCl_3$, HH-COSY, HSQC): 8.28 – 6.76 (m, 20H, CH_{arom}), 5.54 (dd, *J* = 9.4, 3.3 Hz, 1H, H-3), 4.98 (d, *J* = 2.0 Hz, 1H, H-1), 4.74 (d, *J* = 11.0 Hz, 1H, C*H*H Bn), 4.67 (d, *J* = 10.8 Hz, 1H, C*H*H Bn), 4.67 (d, *J* = 12.2 Hz,

1H, C*H*H Bn), 4.60 (d, *J* = 12.3 Hz, 1H, CH*H* Bn), 4.62 – 4.46 (m, 2H, C*H*HF, CH*H*F), 4.53 (d, *J* = 12.1 Hz, 1H, CH*H* Bn), 4.50 (d, *J* = 10.7 Hz, 1H, CH*H* Bn), 4.22 (t, *J* = 9.6 Hz, 1H, H-4), 4.04 (dd, *J* = 3.4, 2.0 Hz, 1H, H-2), 3.93 (ddt, *J* = 11.8, 6.7, 2.5 Hz, 2H, H-5), 3.82 (dd, *J* = 10.9, 4.5 Hz, 1H, H-6), 3.91 – 3.67 (m, 2H, C*H*HCH$_2$F, CH*H*CH$_2$F), 3.73 (dd, *J* = 10.9, 1.9 Hz, 1H, H-6); ^{13}C NMR (126 MHz, CDCl$_3$, HSQC): δ 165.7 (C=O Bz), 138.3, 137.9, 133.2, 131.2 (C$_{q\text{-}arom}$), 129.9, 129.4, 128.5, 128.4, 128.4, 128.4, 128.4, 128.0, 128.0, 127.8, 127.8, 127.8, 127.7, 127.7, (CH$_{arom}$) 98.4 (C-1), 82.6 (d, *J* = 169.9 Hz, CH$_2$F), 76.1 (C-2), 74.9 (CH$_2$ Bn), 74.4 (C-3), 73.6 (CH$_2$ Bn), 73.6 (C-4), 73.2 (CH$_2$ Bn), 71.7 (C-5), 69.0 (C-6), 66.8 (d, *J* = 20.2 Hz, *C*H$_2$CH$_2$F); HRMS: [M+NH$_4$]$^+$ calcd for $C_{36}H_{37}FO_7$ 618.28616, found 618.28583.

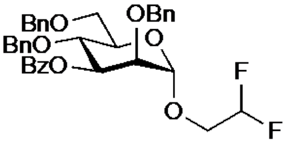

2,2-Difluoroethyl 3-*O*-benzoyl-2,4,6-tri-*O*-benzyl-α-D-mannopyranoside (S64). The title compound was prepared according to general procedure VII. Column chromatography (95:5 → 75:25, pentane:Et$_2$O) yielded the title compound (54 mg, 87 µmol, 87%, colourless oil, α:β; >98:2). TLC: R$_f$ 0.3, (pentane:Et$_2$O, 80:20, v:v); $[\alpha]_D^{25}$ 1.1° (*c* 1, CHCl$_3$); IR (thin film, cm^{-1}): 696, 737, 1269, 1452, 1720, 2926, 3032; ^{1}H NMR (500 MHz, CDCl$_3$, HH-COSY, HSQC): δ 8.40 – 6.70 (m, 20H, CH$_{arom}$), 5.92 (tdd, *J* = 55.5, 4.8, 3.7 Hz, 1H, CHF$_2$), 5.49 (dd, *J* = 9.2, 3.3 Hz, 1H, H-3), 4.96 (d, *J* = 2.1 Hz, 1H, H-1), 4.69 (d, *J* = 12.3 Hz, 1H, C*H*H Bn), 4.69 (d, *J* = 11.2 Hz, 1H, C*H*H Bn), 4.65 (d, *J* = 12.2 Hz, 1H, C*H*H Bn), 4.59 (d, *J* = 12.2 Hz, 1H, CH*H* Bn), 4.53 (d, *J* = 12.0 Hz, 1H, CH*H* Bn), 4.50 (d, *J* = 10.9 Hz, 1H, CH*H* Bn), 4.20 (t, *J* = 9.5 Hz, 1H, H-4), 4.03 (dd, *J* = 3.4, 2.1 Hz, 1H, H-2), 3.93 – 3.68 (m, 5H, H-6, H-6, H-5, C*H*HCH$_2$F, CH*H*CH$_2$F); ^{13}C NMR (126 MHz, CDCl$_3$, HSQC): ^{13}C NMR (126 MHz, CDCl$_3$) δ 165.8 (C=O Bz), 138.2, 137.8, 137.7, 133.3, 130.0, 129.9, 128.6, 128.5, 128.4, 128.4, 128.0, 127.8, 127.8 (CH$_{arom}$), 114.1 (t, *J* = 241.2 Hz, CHF$_2$), 98.9 (C-1), 75.8 (C-2), 74.9 (CH$_2$ Bn), 74.1 (C-3), 73.6 (CH$_2$ Bn), 73.4 (C-4), 73.3 (CH$_2$ Bn), 72.1 (C-5), 68.9 (C-6), 66.9 (t, *J* = 28.7 Hz, *C*H$_2$CHF$_2$); HRMS: [M+NH$_4$]$^+$ calcd for $C_{36}H_{36}F_2O_7$ 636.27674, found 636.27663.

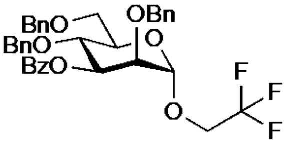

2,2,2-Trifluoroethyl 3-*O*-benzoyl-2,4,6-tri-*O*-benzyl-α-D-mannopyranoside (S65). The title compound was prepared according to general procedure VII. Column chromatography (95:5 → 80:20, pentane:Et$_2$O) yielded the title compound (51 mg, 79 µmol, 79%, colourless oil, α:β; >98:2). TLC: R$_f$ 0.40, (pentane:Et$_2$O, 80:20, v:v); $[\alpha]_D^{25}$ 5.6° (*c* 1, CHCl$_3$); IR (thin film, cm^{-1}): 698, 738, 1027, 1035, 1166, 1253, 1452, 1720, 2866, 3030; Data of the major stereoisomer (α product): ^{1}H NMR (500 MHz, CDCl$_3$, HH-COSY, HSQC): δ 8.53 – 6.76 (m, 20H, CH$_{arom}$), 5.50 (dd, *J* = 9.1, 3.3 Hz, 1H, H-3), 5.00 (d, *J* = 2.1 Hz, 1H, H-1), 4.69 (d, *J* = 12.1 Hz, 1H, C*H*H Bn), 4.68 (d, *J* = 10.9 Hz, 1H, C*H*H Bn), 4.65 (d, *J* = 12.1 Hz, 1H, C*H*H Bn), 4.61 (d, *J* = 12.1 Hz, 1H, CH*H* Bn), 4.52 (d, *J* = 12.1 Hz, 1H, CH*H* Bn), 4.50 (d, *J* = 10.9 Hz, 1H, CH*H* Bn), 4.21 (t, *J* = 9.4 Hz, 1H, H-4), 4.07 (dd, *J* = 3.4, 2.2 Hz, 1H, H-2), 4.06 – 3.83 (m, 3H, C*H*HCF$_3$, CH*H*CF$_3$, H-5), 3.80 (dd, *J* = 10.9, 4.5 Hz, 1H, H-6), 3.71 (dd, *J* = 10.9, 1.9 Hz, 1H, H-6); ^{13}C NMR (126 MHz, CDCl$_3$, HSQC): δ 165.7 (C=O Bz), 138.2, 137.8, 137.6 (C$_{q\text{-}arom}$), 130.0, 128.6, 128.5, 128.4, 128.1, 128.0, 128.0, 128.0, 127.9, 127.8, 127.8, 127.1 (CH$_{arom}$), 123.8 (q, *J* = 278.4 Hz, CF$_3$), 98.8 (C-1), 75.6 (C-2), 74.9 (CH$_2$ Bn), 73.9 (C-3), 73.7 (CH$_2$ Bn), 73.4 (C-4), 73.4 (CH$_2$ Bn), 72.3 (C-5), 68.8 (C-6), 64.3 (q, *J* = 35.0 Hz, *C*H$_2$CF$_3$); HRMS: [M+NH$_4$]$^+$ calcd for $C_{36}H_{35}F_3O_7$ 654.26731, found 654.26711.

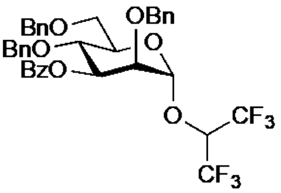

1,1,1,3,3,3-Hexafluoro-2-propyl 3-*O*-benzoyl-2,4,6-tri-*O*-benzyl-α-D-mannopyranoside (S66). The title compound was prepared according to general procedure VII. Column chromatography (100:0 → 80:20, pentane:Et_2O) yielded the title compound (43 mg, 61 µmol, 61%, colourless oil, α:β; >98:2). TLC: R_f 0.20, (pentane:Et_2O, 90:10, v:v); $[\alpha]_D^{25}$ 19.2° (*c* 1, $CHCl_3$); IR (thin film, cm^{-1}): 688, 696, 712, 738, 974, 1027, 1101, 1195, 1220, 1315, 1367, 1452, 1720, 2926, 3032; ^1H NMR (500 MHz, $CDCl_3$, HH-COSY, HSQC): δ 8.22 – 7.03 (m, 20H, CH_{arom}), 5.47 (dd, *J* = 7.9, 3.3 Hz, 1H, H-3), 5.15 (d, *J* = 2.7 Hz, 1H, H-1), 4.71 (d, *J* = 11.0 Hz, 1H, C*H*H Bn), 4.66 (d, *J* = 12.0 Hz, 1H, C*H*H Bn), 4.63 (s, 2H, CH_2 Bn), 4.50 (d, *J* = 10.8 Hz, 1H CH*H* Bn), 4.56 – 4.47 (m, 1H, $CH(CF_3)_2$), 4.44 (d, *J* = 12.0 Hz, 1H, CH*H* Bn), 4.22 (dd, *J* = 9.6, 7.9 Hz, 1H, H-4), 4.10 (dd, *J* = 3.3, 2.7 Hz, 1H, H-2), 3.92 (ddd, *J* = 9.6, 3.9, 2.1 Hz, 1H, H-5), 3.76 (dd, *J* = 11.0, 3.8 Hz, 1H, H-6), 3.66 (dd, *J* = 11.0, 2.1 Hz, 1H, H-6); ^{13}C NMR (126 MHz, $CDCl_3$, HSQC): δ 165.8 (C=O Bz), 138.0, 137.6, 137.2 ($C_{q\text{-}arom}$), 129.9, 128.6, 128.6, 128.5, 128.2, 128.1, 128.1, 128.0, 127.9, 127.8 (CH_{arom}), 100.9 (C-1), 75.1 (C-2), 74.5, 73.6, 73.4 (CH_2 Bn), 73.3 (C-4), 72.9 (C-5), 72.9 (C-3), 72.0 (p, *J* = 34.2 Hz, *C*$H(CF_3)_2$), 68.4 (C-6); ^{13}C-GATED NMR (126 MHz, $CDCl_3$) δ 100.9 ($J_{H1\text{-}C1}$ = 178 Hz, α); HRMS: $[M+NH_4]^+$ calcd for $C_{37}H_{34}F_6O_7$ 722.25470, found 722.25433.

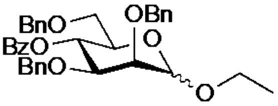

Ethyl 4-*O*-benzoyl-2,3,6-tri-*O*-benzyl-α/β-D-mannopyranoside (S67). The title compound was prepared according to general procedure VII. Column chromatography (95:5 → 70:30, pentane:Et_2O) yielded the title compound (51 mg, 88 µmol, 88%, colourless oil, α:β; 31:69). TLC: R_f 0.20, (pentane:Et_2O, 80:20, v:v); IR (thin film, cm^{-1}): 595, 695, 735, 1026, 1105, 1266, 1452, 1720, 2869, 3030; Data of the major stereoisomer (β product): ^1H NMR (500 MHz, $CDCl_3$, HH-COSY, HSQC): δ 7.61 – 7.06 (m, 20H, CH_{arom}), 5.55 (t, *J* = 9.7 Hz, 1H, H-4), 4.99 (d, *J* = 12.7 Hz, 1H, C*H*H Bn), 4.90 (d, *J* = 12.6 Hz, 1H, CH*H* Bn), 4.58 – 4.41 (m, 4H, C*H*H Bn, CH*H* Bn, CH*H* Bn, H-1), 4.24 (d, *J* = 12.5 Hz, 1H, CH*H* Bn), 4.05 (dq, *J* = 9.3, 7.1 Hz, 1H, C*H*HCH_3 Et), 3.94 (dd, *J* = 3.0, 0.8 Hz, 1H, H-2), 3.81 – 3.60 (m, 3H, H-5, H-6, H-6), 3.56 (dd, *J* = 9.8, 2.9 Hz, 1H, H-3), 3.59 – 3.44 (m, 1H, CH*H*CH_3 Et), 1.30 (t, *J* = 7.0 Hz, 3H, CH_3 Et); ^{13}C NMR (126 MHz, $CDCl_3$, HSQC): δ 165.7 (C=O Bz), 138.6, 138.2, 137.7 ($C_{q\text{-}arom}$), 129.9, 128.6, 128.4, 128.4, 128.4, 128.2, 127.7, 127.7, 127.6 (CH_{arom}), 101.3 (C-1), 78.8 (C-3), 74.8 (C-5), 74.0, 73.8 (CH_2 Bn), 73.2 (C-2), 71.0 (CH_2 Bn), 70.7 (C-6), 69.8 (C-4), 65.6 (*C*H_2CH_3 Et), 15.3 (CH_3 Et); Diagnostic signals of the minor stereoisomer (α product): ^1H NMR (500 MHz, $CDCl_3$, HH-COSY, HSQC): δ 5.64 (t, *J* = 9.8 Hz, 1H, H-4), 4.92 (d, *J* = 2.0 Hz, 1H, H-1), 4.80 (d, *J* = 12.5 Hz, 1H, C*H*H Bn), 4.73 (d, *J* = 12.5 Hz, 1H, CH*H* Bn), 3.84 (dd, *J* = 3.1, 2.0 Hz, 1H, H-2); ^{13}C NMR (126 MHz, $CDCl_3$, HSQC): δ 98.2 (C-1), 77.5 (C-3), 74.4 (C-2), 73.6, 73.0, 71.9 (CH_2 Bn), 63.4 (*C*H_2CH_3 Et), 15.1 (CH_3 Et); HRMS: $[M+NH_4]^+$ calcd for $C_{36}H_{38}O_7$ 600.29558, found 600.29575.

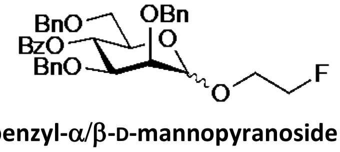

2-Fluoroethyl 4-*O*-benzoyl-2,3,6-tri-*O*-benzyl-α/β-D-mannopyranoside (S68). The title compound was prepared according to general procedure VII. Column chromatography (90:10 → 60:40, pentane:Et_2O) yielded the title compound (50 mg, 83 µmol, 83%, colourless oil, α:β; 60:40). TLC: R_f 0.20, (pentane:Et_2O, 70:30, v:v); IR (thin

film, cm^{-1}): 537, 695, 711, 738, 1027, 1043, 1069, 1088, 1266, 1452, 1724, 2867, 3030; Data of the major stereoisomer (α product): ^{1}H NMR (600 MHz, $CDCl_3$, HH-COSY, HSQC): δ 8.05 – 6.98 (m, 20H, CH_{arom}), 5.65 (t, *J* = 9.8 Hz, 1H, H-4), 4.96 (d, *J* = 2.0 Hz, 1H, H-1), 4.80 (d, *J* = 12.4 Hz, 1H, C*H*H Bn), 4.72 (d, *J* = 12.5 Hz, 1H, CH*H* Bn), 4.67 – 4.42 (m, 6H, C*H*H Bn, CH*H* Bn, C*H*H Bn, CH*H* Bn, C*H*HF, CH*H*F), 4.01 – 3.79 (m, 2H, C*H*HCH_2F, CH*H*CH_2F), 3.90 (dd, *J* = 3.0, 2.0 Hz, 1H, H-2), 3.79 – 3.64 (m, 3H, H-5, H-6, H-6), 3.62 (dd, *J* = 10.8, 2.8 Hz, 1H, H-2); ^{13}C NMR (151 MHz, $CDCl_3$, HSQC): δ 165.7 (C=O Bz), 138.5, 138.3, 138.2, 138.1, 137.7 ($C_{q\text{-}arom}$), 131.2, 130.0, 129.9, 128.6, 128.4, 128.4, 128.4, 128.4, 128.3, 127.7, 127.5 (CH_{arom}), 98.8 (C-1), 82.6 (d, *J* = 169.6 Hz, *C*H_2F), 77.3 (C-3), 74.3 (C-2), 73.6, 73.1, 72.1 (CH_2 Bn), 70.9 (C-5), 70.1 (C-6), 69.5 (C-4), 66.9 (d, *J* = 19.7 Hz, *C*H_2CH_2F); Diagnostic signals of the minor stereoisomer (β product): ^{1}H NMR (600 MHz, $CDCl_3$, HH-COSY, HSQC): δ 5.56 (t, *J* = 9.7 Hz, 1H, H-4), 4.98 (d, *J* = 12.5 Hz, 1H, C*H*H Bn), 4.89 (d, *J* = 12.5 Hz, 1H, CH*H* Bn), 4.67 – 4.42 (signals overlap with major isomer, 1H, H-1), 4.26 (d, *J* = 12.5 Hz, 1H, CH*H* Bn), 4.13 (dddd, *J* = 35.8, 12.2, 3.9, 2.3 Hz, 1H, C*H*HCH_2F), 3.58 (dd, *J* = 9.7, 3.0 Hz, 1H, H-2); ^{13}C NMR (151 MHz, $CDCl_3$, HSQC): δ 101.5 (C-1), 82.9 (d, *J* = 169.4 Hz, *C*H_2F), 74.2, 73.7, 71.1 (CH_2 Bn), 70.5 (C-6), 69.6 (C-4), 68.7 (d, *J* = 19.7 Hz, *C*H_2CH_2F); HRMS: $[M+NH_4]^+$ calcd for $C_{36}H_{37}FO_7$ 618.28616, found 618.28620.

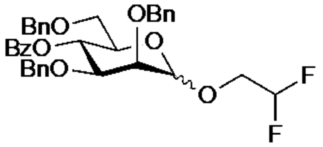

2,2-Difluoroethyl 4-*O*-benzoyl-2,3,6-tri-*O*-benzyl-α/β-D-mannopyranoside (S69). The title compound was prepared according to general procedure VII. Column chromatography (95:5 → 70:30, pentane:Et_2O) yielded the title compound (50 mg, 81 µmol, 81%, colourless oil, α:β; 71:29). TLC: R_f 0.20, (pentane:Et_2O, 80:20, v:v); IR (thin film, cm^{-1}): 697, 711, 738m 1027, 1066, 1105, 1266, 1452, 1720, 2870, 3030; Data of the major stereoisomer (α product): ^{1}H NMR (600 MHz, $CDCl_3$, HH-COSY, HSQC): δ 8.22 – 6.63 (m, 20H, CH_{arom}), 5.94 (tdd, *J* = 55.3, 4.8, 3.4 Hz, 1H, C*H*F_2), 5.63 (t, *J* = 9.7 Hz, 1H, H-4), 4.93 (d, *J* = 2.1 Hz, 1H, H-1), 4.79 (d, *J* = 12.3 Hz, 1H, C*H*H Bn), 4.70 (d, *J* = 12.4 Hz, 1H, CH*H* Bn), 4.60 – 4.42 (m, 4H, C*H*H Bn, CH*H* Bn, C*H*H Bn, CH*H* Bn), 3.99 (ddd, *J* = 9.7, 6.5, 2.7 Hz, 1H, H-5), 3.94 (dd, *J* = 9.3, 3.0 Hz, 1H, H-3), 3.87 (t, *J* = 2.6 Hz, 1H, H-2), 3.85 – 3.64 (m, 3H, H-6, C*H*HCHF_2, CH*H*CHF_2), 3.61 (dd, *J* = 10.8, 2.8 Hz, 1H, H-6); ^{13}C NMR (151 MHz, $CDCl_3$, HSQC): δ 165.7 (C=O Bz), 138.4, 138.2, 138.1, 138.1, 138.0, 137.7 ($C_{q\text{-}arom}$), 130.0, 128.6, 128.5, 128.5, 128.5, 128.5, 128.4, 128.4, 128.4, 127.9, 127.9, 127.8, 127.8, 127.8, 127.8, 127.8, 127.7 (CH_{arom}), 114.1 (t, *J* = 241.1 Hz, *C*HF_2), 99.6 (C-1), 77.2 (C-3), 74.3 (C-2), 73.7, 73.3, 72.2 (CH_2 Bn), 71.4 (C-5), 70.0 (C-6), 69.3 (C-4), 67.1 (t, *J* = 28.1 Hz, *C*H_2CHF_2); Diagnostic signals of the minor stereoisomer (β product): ^{1}H NMR (600 MHz, $CDCl_3$, HH-COSY, HSQC): δ 5.55 (t, *J* = 9.5 Hz, 1H, H-4), 4.85 (d, *J* = 12.5 Hz, 1H, CH*H* Bn), 4.53 (s, 1H, H-1), 4.28 (d, *J* = 12.5 Hz, 1H, CH*H* Bn), 4.07 (dddd, *J* = 21.7, 12.1, 9.5, 2.7 Hz, 1H, C*H*HCHF_2), 3.58 (dd, *J* = 9.6, 3.0 Hz, 1H, H-3); ^{13}C NMR (151 MHz, $CDCl_3$, HSQC): δ 114.5 (dd, *J* = 242.2, 239.7 Hz, *C*HF_2), 101.6 (C-1), 74.3, 73.8, 71.3 (CH_2 Bn), 70.4 (C-6), 69.5 (C-4), 68.6 (dd, *J* = 31.0, 25.9 Hz, *C*H_2CHF_2); HRMS: $[M+NH_4]^+$ calcd for $C_{36}H_{36}F_2O_7$ 636.27674, found 636.27674.

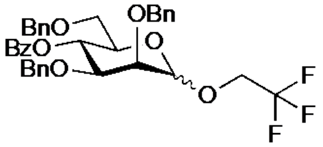

2,2,2-Trifluoroethyl 4-*O*-benzoyl-2,3,6-tri-*O*-benzyl-α/β-D-mannopyranoside (S70). The title compound was prepared according to general procedure VII. Column chromatography (95:5 → 85:15, pentane:Et_2O) yielded the title compound (49 mg, 77 µmol, 77%, colourless oil, α:β; 88:12). TLC: R_f 0.40, (pentane:Et_2O, 80:20, v:v); IR

(thin film, cm^{-1}): 697, 735, 1027, 1081, 1266, 1452, 1720, 2870, 3031; Data of the major stereoisomer (α product): ^{1}H NMR (400 MHz, $CDCl_3$, HH-COSY, HSQC): δ 8.47 – 6.78 (m, 20H, CH_{arom}), 5.65 (t, *J* = 9.6 Hz, 1H, H-4), 4.97 (d, *J* = 2.1 Hz, 1H, H-1), 4.80 (d, *J* = 12.3 Hz, 1H, C*H*H Bn), 4.69 (d, *J* = 12.3 Hz, 1H, CH*H* Bn), 4.6 (d, *J* = 12.0 Hz, 1H, C*H*H Bn), 4.53 – 4.41 (m, 3H, C*H*H Bn, CH*H* Bn, CH*H* Bn), 4.09 – 3.83 (m, 5H, H-3, H-4, H-5, C*H*HCF3, CH*H*CF3), 3.67 (dd, *J* = 10.8, 6.2 Hz, 1H, H-6), 3.60 (dd, *J* = 10.8, 2.9 Hz, 1H, H-6); ^{13}C NMR (101 MHz, $CDCl_3$, HSQC): δ 165.6 (C=O Bz), 138.1, 138.0, 137.9, 137.5 ($C_{q\text{-}arom}$), 128.4, 127.9, 127.8, 127.8, 127.7, 127.6 (CH_{arom}), 122.5 (q, *J* = 280.0 Hz, *C*F3), 99.1 (C-1), 76.9 (C-3), 74.0 (C-2), 73.6, 73.3, 72.2 (CH_2 Bn), 71.6 (C-5), 69.8 (C-6), 69.1 (C-4), 64.3 (q, *J* = 34.9 Hz, *C*H_2CF_3); Diagnostic signals of the minor stereoisomer (β product): ^{1}H NMR (400 MHz, $CDCl_3$, HH-COSY, HSQC): δ 5.56 (t, *J* = 9.5 Hz, 1H, H-4), 4.87 (d, *J* = 12.5 Hz, 1H, CH*H* Bn), 4.61 – 4.53 (signals overlap with major isomer, 1H, H-1); ^{13}C NMR (126 MHz, $CDCl_3$, HSQC): 101.1 (C-1), 78.5 (C-3), 70.3 (C-6), 69.3 (C-4), 64.3 (q, *J* = 34.9 Hz, *C*H_2CF_3); HRMS: $[M+NH_4]^+$ calcd for $C_{36}H_{35}F_3O_7$ 654.26731, found 654.26732.

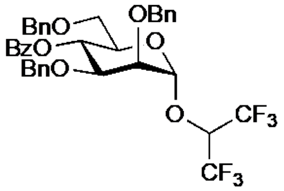

1,1,1,3,3,3-Hexafluoro-2-propyl 4-*O*-benzoyl-2,3,6-tri-*O*-benzyl-α-D-mannopyranoside (S71). The title compound was prepared according to general procedure VII. Column chromatography (100:0 → 80:20, pentane:Et_2O) yielded the title compound (35 mg, 50 µmol, 50%, colourless oil, α:β; >98:2). TLC: R_f 0.10, (pentane:Et_2O, 90:10, v:v); $[\alpha]_D^{25}$ 17.0° (*c* 1, $CHCl_3$); IR (thin film, cm^{-1}): 689, 711, 1027, 1068, 1452, 1727, 2861, 3032; ^{1}H NMR (500 MHz, $CDCl_3$, HH-COSY, HSQC): δ 8.08 – 7.09 (m, 20H, CH_{arom}), 5.69 (dd, *J* = 9.9, 8.1 Hz, 1H, H-4), 5.12 (d, *J* = 2.1 Hz, 1H, H-1), 4.77 (d, *J* = 12.3 Hz, 1H, C*H*H Bn), 4.65 (d, *J* = 12.2 Hz, 1H, CH*H* Bn), 4.59 (d, *J* = 12.0 Hz, 1H, C*H*H Bn), 4.53 (d, *J* = 12.0 Hz, 1H, CH*H* Bn), 4.52 – 4.45 (m, 3H, C*H*H Bn, CH*H* Bn, C*H*(CF_3)$_2$), 4.03 (ddd, *J* = 10.0, 5.6, 2.8 Hz, 1H, H-5), 3.93 – 3.84 (m, 2H, H-2, H-3), 3.64 (dd, *J* = 11.0, 5.6 Hz, 1H, H-6), 3.59 (dd, *J* = 11.0, 2.9 Hz, 1H, H-6); ^{13}C NMR (126 MHz, $CDCl_3$, HSQC): δ 165.5 (C=O Bz), 137.9, 137.7, 137.6 ($C_{q\text{-}arom}$), 129.8, 128.5, 128.5, 128.4, 128.4, 128.2, 128.1, 127.9, 127.8, 127.6, 127.6 (CH_{arom}), 100.8 (C-1), 76.5 (C-3), 74.0 (C-2), 73.6, 73.5, 72.4 (CH_2 Bn), 72.3 (C-5), 71.9 (p, *J* = 32.5 Hz, H-1, *C*H(CF_3)$_2$), 69.3 (C-6), 68.6 (C-4); ^{13}C-GATED NMR (126 MHz, $CDCl_3$) δ 100.8 ($J_{H1\text{-}C1}$ = 176 Hz, α); HRMS: $[M+NH_4]^+$ calcd for $C_{37}H_{34}F_6O_7$ 722.25470, found 722.25449.

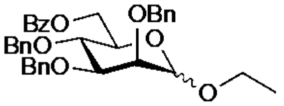

Ethyl 6-*O*-benzoyl-2,3,4-tri-*O*-benzyl-α/β-D-mannopyranoside (S72). The title compound was prepared according to general procedure VII. Column chromatography (95:5 → 75:25, pentane:Et_2O) yielded the title compound (40 mg, 69 µmol, 69%, colourless oil, α:β; 35:65). TLC: R_f 0.30, (pentane:Et_2O, 80:20, v:v); IR (thin film, cm^{-1}): 696, 712, 737, 1027, 1066, 1104, 1274, 1452, 1496, 1720, 2869, 3030; Data of the major stereoisomer (β product): ^{1}H NMR (500 MHz, $CDCl_3$, HH-COSY, HSQC): δ 8.41 – 7.08 (m, 20H, CH_{arom}), 5.04 (d, *J* = 12.3 Hz, 1H, C*H*H Bn), 5.00 (d, *J* = 10.7 Hz, 1H, C*H*H Bn), 4.91 (d, *J* = 12.3 Hz, 1H, CH*H* Bn), 4.73 – 4.51 (m, 5H, H-6, H-6, C*H*H Bn, CH*H* Bn, CH*H* Bn), 4.47 (s, 1H, H-1), 4.07 (t, *J* = 9.4 Hz, 1H, H-4), 4.05 – 3.99 (m, 1H, C*H*HCH3), 3.97 (d, *J* = 3.1 Hz, 1H, H-2), 3.66 – 3.62 (m, 1H, H-5), 3.61 (dd, *J* = 9.3, 3.0 Hz, 1H, H-3), 3.54 (dq, *J* = 9.8, 7.2 Hz, 1H, CH*H*CH3), 1.28 (t, *J* = 7.1 Hz, 3H, C*H*3); ^{13}C NMR (126 MHz, $CDCl_3$, HSQC): δ 166.5 (C=O Bz), 139.0, 138.6, 138.2, 138.2 ($C_{q\text{-}arom}$), 130.3, 130.2, 128.5, 128.5, 128.4, 128.4, 128.4, 127.9, 127.9, 127.8, 127.7, 127.7, 127.5 (CH_{arom}), 101.7 (C-1), 82.4 (C-3), 75.4 (CH_2 Bn), 74.8 (C-4), 74.0 (C-2), 73.9 (CH_2 Bn), 73.9 (C-5), 71.6 (CH_2 Bn), 65.6 (*C*H_2CH_3), 64.3

(C-6), 15.3 (CH_3); ^{13}C-GATED NMR (126 MHz, $CDCl_3$) δ 101.7 (J_{H1-C1} = 153 Hz, β); Diagnostic signals of the minor stereoisomer (α product): ^{1}H NMR (500 MHz, $CDCl_3$, HH-COSY, HSQC): δ 4.94 (d, J = 1.9 Hz, 1H, H-1), 4.82 (d, J = 12.2 Hz, 1H, C*H*H Bn), 4.14 (t, J = 9.6 Hz, 1H, H-4), 3.87 (dd, J = 3.1, 1.9 Hz, 1H, H-2), 3.76 (dq, J = 9.8, 7.1 Hz, 1H, C*H*HCH$_3$), 3.53 – 3.45 (m, 1H, CH*H*CH$_3$), 1.21 (t, J = 7.1 Hz, 3H, C*H*$_3$); ^{13}C NMR (126 MHz, $CDCl_3$, HSQC): δ 97.7 (C-1), 80.5 (C-3), 75.2 (C-2), 73.9, 72.8, 72.3 (CH_2 Bn), 64.0 (*C*H$_2$CH$_3$), 63.3 (C-6), 15.1 (*C*H$_3$); ^{13}C-GATED NMR (126 MHz, $CDCl_3$) δ 97.7 (J_{H1-C1} = 171 Hz, α); HRMS: $[M+NH_4]^+$ calcd for $C_{36}H_{38}O_7$ 600.29558, found 600.29587.

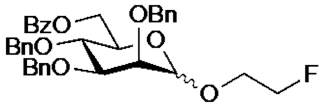

2-Fluoroethyl 6-*O*-benzoyl-2,3,4-tri-*O*-benzyl-α/β-D-mannopyranoside (S73). The title compound was prepared according to general procedure VII. Column chromatography (90:10 → 60:40, pentane:Et_2O) yielded the title compound (59 mg, 99 µmol, 99%, colourless oil, α:β; 51:49). TLC: R_f 0.20, (pentane:Et_2O, 70:30, v:v); IR (thin film, cm^{-1}): 538, 712, 1027, 1209, 1274, 1720, 2871, 3032; Data of the major stereoisomer (α product): ^{1}H NMR (500 MHz, $CDCl_3$, HH-COSY, HSQC): δ 8.39 – 6.92 (m, 20H, CH_{arom}), 4.99 (d, J = 12.2 Hz, 1H, C*H*H Bn), 4.94 (d, J = 1.9 Hz, 1H, H-1), 4.87 (d, J = 12.2 Hz, 1H, CH*H* Bn), 4.73 – 4.44 (m, 8H, C*H*H Bn, CH*H* Bn, C*H*H Bn, CH*H* Bn, H-6, H-6, C*H*HF, CH*H*F), 4.12 (t, J = 9.6 Hz, 1H, H-4), 4.02 (dd, J = 9.2, 3.0 Hz, 3H, H-3), 3.90 (dd, J = 3.1, 1.9 Hz, 1H, H-2), 3.86 – 3.63 (m, 2H, C*H*HCH$_2$F, CH*H*CH$_2$F); ^{13}C NMR (126 MHz, $CDCl_3$, HSQC): δ 166.4 (C=O Bz), 138.4, 138.1, 138.1, 138.0 (C_{q-arom}), 131.2, 130.2, 129.8, 128.5, 128.5, 128.4, 128.3, 128.3, 128.2, 127.9, 127.9, 127.8, 127.7, 127.7, 127.5 (CH_{arom}), 98.3 (C-1), 82.9 (d, J = 169.3 Hz, *C*H$_2$F), 80.3 (C-3), 75.4 (CH_2 Bn), 75.0 (C-2), 74.5 (C-4), 74.1, 74.0, 72.9 (CH_2 Bn), 70.5 (C-5), 66.8 (d, J = 20.0 Hz, *C*H$_2$CH$_2$F), 64.1 (C-6); ^{13}C-GATED NMR (126 MHz, $CDCl_3$) δ 98.3 (J_{H1-C1} = 171 Hz, α); Diagnostic signals of the minor stereoisomer (β product): ^{1}H NMR (500 MHz, $CDCl_3$, HH-COSY, HSQC): δ 4.95 (Signal overlaps with major isomer, 1H, H-1), 4.87 (d, J = 12.2 Hz, 1H, CH*H* Bn), 4.80 (d, J = 3.2 Hz, 1H, H), 4.05 (t, J = 9.5 Hz, 1H, H-4); ^{13}C NMR (126 MHz, $CDCl_3$, HSQC): 101.9 (C-1), 82.5 (d, J = 170.2 Hz, *C*H$_2$F), 68.7 (d, J = 19.8 Hz, *C*H$_2$CH$_2$); ^{13}C-GATED NMR (126 MHz, $CDCl_3$) δ 101.9 (J_{H1-C1} = 155 Hz, β); HRMS: $[M+NH_4]^+$ calcd for $C_{36}H_{37}FO_7$ 618.28616, found 618.28604.

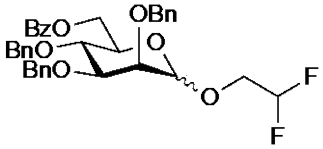

2,2-Difluoroethyl 6-*O*-benzoyl-2,3,4-tri-*O*-benzyl-α/β-D-mannopyranoside (S74). The title compound was prepared according to general procedure VII. Column chromatography (95:5 → 80:20, pentane:Et_2O) yielded the title compound (55 mg, 89 µmol, 89%, colourless oil, α:β; 78:22). TLC: R_f 0.30, (pentane:Et_2O, 80:20, v:v); IR (thin film, cm^{-1}): 696, 1027, 1069, 1273, 1452, 1720, 2924, 3031; Data of the major stereoisomer (α product): ^{1}H NMR (500 MHz, $CDCl_3$, HH-COSY, HSQC): δ 8.32 – 6.85 (m, 20H, CH_{arom}), 5.87 (tdd, J = 55.3, 4.6, 3.6 Hz, 1H, C*H*F$_2$), 4.93 (d, J = 10.7 Hz, 1H, C*H*H Bn), 4.91 (d, J = 2.0 Hz, 1H, H-1), 4.79 (d, J = 12.1 Hz, 1H, C*H*H Bn), 4.72 – 4.46 (m, 5H, C*H*H Bn, CH*H* Bn, CH*H* Bn, H-6, H-6), 4.10 (t, J = 9.5 Hz, 1H, H-4), 3.96 (dd, J = 9.3, 3.1 Hz, 1H, H-3), 3.93 (ddd, J = 9.8, 4.3, 2.5 Hz, 1H, H-5), 3.86 (dd, J = 3.1, 2.0 Hz, 1H, H-2), 3.80 (dddd, J = 15.3, 14.1, 11.8, 3.6 Hz, 1H, C*H*HCHF$_2$), 3.68 (tdd, J = 13.2, 11.8, 4.6 Hz, 1H, CH*H*CHF$_2$); ^{13}C NMR (126 MHz, $CDCl_3$, HSQC): δ 166.4 (C=O Bz), 138.6, 138.3, 138.2, 137.9 (C_{q-arom}), 130.1, 128.6, 128.5, 128.5, 128.4, 128.3, 128.3, 128.3, 128.0, 128.0, 127.9, 127.9, 127.7, 127.6 (CH_{arom}), 114.0 (t, J = 241.2 Hz, *C*HF$_2$), 98.8 (C-1), 80.0 (C-3), 75.4 (CH_2 Bn), 74.8 (C-2), 74.3 (C-4), 73.1, 72.5 (CH_2 Bn), 70.9 (C-5), 66.8 (t, J = 28.1 Hz, *C*H$_2$CHF$_2$), 63.7 (C-6); ^{13}C-GATED NMR (126 MHz, $CDCl_3$) δ 98.8 (J_{H1-C1} = 170 Hz, α); Diagnostic signals of the minor stereoisomer (β product): ^{1}H NMR (500 MHz, $CDCl_3$, HH-

COSY, HSQC): δ 6.04 – 5.77 (m, 1H, C*H*F_2), 4.84 (d, *J* = 12.1 Hz, 1H, CH*H* Bn), 4.50 (signal overlaps with major isomer, 1H, H-1), 4.03 (t, *J* = 9.4 Hz, 1H, H-4); ^{13}C NMR (126 MHz, $CDCl_3$, HSQC): δ 101.9 (C-1), 74.4, 74.1, 73.7 (CH_2 Bn), 68.5 (dd, *J* = 31.1, 25.9 Hz, *C*H_2CHF_2), 63.9 (C-6); HRMS: $[M+NH_4]^+$ calcd for $C_{36}H_{36}F_2O_7$ 636.27674, found 636.27662.

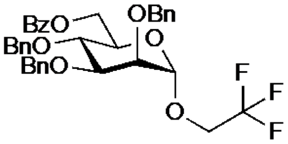

2,2,2-Trifluoroethyl 3-*O*-benzoyl-2,3,4-tri-*O*-benzyl-α/β-D-mannopyranoside (S75). The title compound was prepared according to general procedure VII. Column chromatography (95:5 → 80:20, pentane:Et_2O) yielded the title compound (36 mg, 56 µmol, 56%, colourless oil, α:β; >98:2). TLC: R_f 0.40, (pentane:Et_2O, 80:20, v:v); $[\alpha]_D^{25}$ 34.6° (*c* 1, $CHCl_3$); IR (thin film, cm^{-1}): 697, 712, 1027, 1070, 1166, 1274, 1452, 1720, 2867, 3032; ^{1}H NMR (500 MHz, $CDCl_3$, HH-COSY, HSQC): δ 8.45 – 6.83 (m, 20H, CH_{arom}), 4.93 (s, 1H, H-1), 4.93 (d, *J* = 10.5 Hz, 1H, C*H*H Bn), 4.79 (d, *J* = 12.1 Hz, 1H, C*H*H Bn), 4.73 – 4.64 (m, 3H, C*H*H Bn, CH*H* Bn, CH*H* Bn), 4.61 (d, *J* = 10.8 Hz, 1H, CH*H* Bn), 4.58 – 4.51 (m, 2H, H-6, H-6), 4.11 (t, *J* = 9.5 Hz, 1H, H-4), 4.01 – 3.80 (m, 5H, H-2, H-3, H-5, C*H*HCF_3, CH*H*CF_3); ^{13}C NMR (126 MHz, $CDCl_3$, HSQC): δ 166.4 (C=O Bz), 138.3, 138.1, 138.0 ($C_{q\text{-}arom}$), 130.1, 129.8, 128.6, 128.6, 128.5, 128.3, 128.0, 127.9, 127.9, 127.8, 127.1 (CH_{arom}), 123.8 (q, *J* = 278.3 Hz, *C*F_3), 98.7 (C-1), 79.8 (C-3), 75.4 (CH_2 Bn), 74.8 (C-2), 74.2 (C-4), 73.3, 72.6 (CH_2 Bn), 71.2 (C-5), 64.4 (q, *J* = 35.0 Hz, *C*H_2CF_3), 63.6 (C-6); HRMS: $[M+NH_4]^+$ calcd for $C_{36}H_{35}F_3O_7$ 654.26731, found 654.26715.

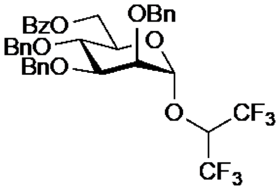

1,1,1,3,3,3-Hexafluoro-2-propyl 6-*O*-benzoyl-2,3,4-tri-*O*-benzyl-α-D-mannopyranoside (S76). The title compound was prepared according to general procedure VII. Column chromatography (100:0 → 85:15, pentane:Et_2O) yielded the title compound (45 mg, 64 µmol, 64%, colourless oil, α:β; >98:2). TLC: R_f 0.25, (pentane:Et_2O, 90:10, v:v); $[\alpha]_D^{25}$ 39.9° (*c* 1, $CHCl_3$); IR (thin film, cm^{-1}): 595, 698, 1027, 1103, 1274, 1720, 2937, 3034; Data of the major stereoisomer (α product): ^{1}H NMR (500 MHz, $CDCl_3$, HH-COSY, HSQC): δ 8.37 – 6.41 (m, 20H, CH_{arom}), 5.04 (d, *J* = 2.1 Hz, 1H, H-1), 4.90 (d, *J* = 10.7 Hz, 1H, C*H*H Bn), 4.78 (d, *J* = 12.1 Hz, 1H, C*H*H Bn), 4.72 (d, *J* = 11.5 Hz, 1H, C*H*H Bn), 4.68 (d, *J* = 11.6 Hz, 1H, CH*H* Bn), 4.64 – 4.58 (m, 2H, CH*H* Bn, CH*H* Bn), 4.55 (dd, *J* = 12.0, 2.3 Hz, 1H, H-6), 4.51 (dd, *J* = 12.0, 4.3 Hz, 1H, H-6), 4.41 (p, *J* = 5.9 Hz, 1H, C*H*$(CF_3)_2$), 4.11 (t, *J* = 9.3 Hz, 1H, H-4), 3.99 (ddd, *J* = 9.8, 4.1, 2.4 Hz, 1H, H-5), 3.92 (dd, *J* = 8.9, 3.0 Hz, 1H, H-3), 3.86 (dd, *J* = 3.0, 2.1 Hz, 1H, H-2); ^{13}C NMR (126 MHz, $CDCl_3$, HSQC): δ 166.1 (C=O Bz), 137.8, 137.5, 137.5 ($C_{q\text{-}arom}$), 129.8, 129.5, 128.4, 128.4, 128.4, 127.9, 127.8, 127.7, 127.7, 127.5 (CH_{arom}), 100.3 (C-1), 79.0 (C-3), 77.2 (CH_2 Bn), 76.9 (C-2), 76.7 (C-4), 75.1, 74.4 (CH_2 Bn), 72.2 (p, *J* = 32.7 Hz, *C*H$(CF_3)_2$) 71.9 (C-5), 63.1 (C-6); HRMS: $[M+NH_4]^+$ calcd for $C_{37}H_{34}F_6O_7$ 722.25470, found 722.25433.

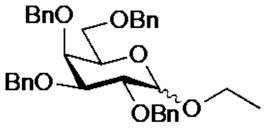

Ethyl 2,3,4,6-tetra-*O*-benzyl-α/β-D-galactopyranoside (S77). The title compound was prepared according to general procedure VII. Column chromatography (100:0 → 90:10, pentane:EtOAc) yielded the title compound (42 mg, 73 µmol, 73%, colourless oil, α:β; 17:83). TLC: R_f 0.46 (pentane:EtOAc, 90:10, v:v); IR (thin film, cm^{-1}): 731, 1026, 1067, 1092, 1360, 1452, 1497, 2868, 2914; Data of the major stereoisomer (β product): ^{1}H NMR (400 MHz, $CDCl_3$, HH-COSY, HSQC): δ 7.39 – 7.22 (m, 20H, CH_{arom}), 4.97 – 4.90 (m, 2H, C*H*H Bn, C*H*H Bn), 4.76 (m, 2H, CH*H* Bn, C*H*H Bn), 4.73 – 4.67 (m, 1H, C*H*H Bn), 4.62 (d, *J* = 11.7 Hz, 1H, CH*H* Bn), 4.49 – 4.34 (m, 2H, C*H*H Bn, CH*H* Bn), 4.36 (d, *J* = 7.7 Hz, 1H, H-1), 4.03 – 3.92 (m, 1H, C*H*HCH_3), 3.88 (d, *J* = 2.7 Hz, 1H, H-4), 3.81 (t, *J* = 8.7 Hz, 1H, H-2), 3.62 – 3.55 (m, 3H, H-6, H-6, C*H*HCH_3), 3.56 – 3.46 (m, 2H, H-3, H-5), 1.26 (t, *J* = 7.1 Hz, 3H, CH_3); ^{13}C-APT NMR ($CDCl_3$, 101 MHz, HSQC): δ 139.0, 138.8, 138.7, 138.1 ($C_{q\text{-}arom}$), 128.5, 128.5, 128.5, 128.4, 128.4, 128.4, 128.3, 128.3, 128.2, 128.0, 127.9, 127.7, 127.6, 127.6 (CH_{arom}), 103.9 (C-1), 82.3 (C-3), 79.8 (C-2), 75.3 (CH_2 Bn), 74.6 (CH_2 Bn), 73.7 (CH_2 Bn), 73.6 (C-4), 73.5 (C-5), 73.2 (CH_2 Bn), 69.1 (C-6), 65.6 (CH_2CH_3), 15.4 (CH_3); Diagnostic signals of the minor stereoisomer (α product): δ 4.86 (d, *J* = 11.9 Hz, 1H, C*H*H Bn), 4.82 (d, *J* = 4.6 Hz, 1H, H-1), 3.70 (dqd, *J* = 10.1, 7.2, 1.0 Hz, 1H, C*H*HCH_3); ^{13}C-APT NMR ($CDCl_3$, 101 MHz, HSQC): δ 97.3 (C-1), 76.7 (C-2), 74.9, 73.4, 69.2 (CH_2 Bn), 63.4 (C-6), 15.1 (CH_3); HRMS: $[M+Na]^+$ calcd for $C_{36}H_{40}O_6Na$ 591.27171, found 591.2710.

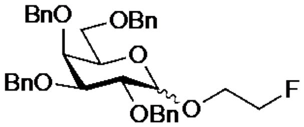

2-Fluoroethyl 2,3,4,6-tetra-*O*-benzyl-α/β-D-galactopyranoside (S78). The title compound was prepared according to general procedure VII. Column column chromatography (97:3 → 85:15, pentane:EtOAc) yielded the title compound (49 mg, 84 µmol, 84%, colourless oil, α:β; 31:69). TLC: R_f 0.36 (pentane:EtOAc, 85:15, v:v); IR (thin film, cm^{-1}): 696, 734, 1027, 1079, 1095, 1347, 1453, 1496, 2914; Data of the major stereoisomer (β product): ^{1}H NMR (500 MHz, $CDCl_3$, HH-COSY, HSQC, HMBC-Gated): δ 7.67 – 7.17 (m, 20H, CH_{arom}), 4.98 – 4.36 (m, 11H, CH_2 Bn, CH_2 Bn, CH_2 Bn, CH_2 Bn, H-1, CH_2C*H*HF, CH_2CH*H*F), 4.13 – 4.01 (m, 1H, C*H*HCH_2F), 3.60 – 3.55 (m, 3H, H-2, H-4, CH*H*CH_2F), 3.55 – 3.49 (m, 3H, H-6, H-6, H-3); ^{13}C NMR (126 MHz, $CDCl_3$, HSQC, HMBC-Gated): δ 138.8, 138.7, 138.6, 138.0 ($C_{q\text{-}arom}$), 131.1, 129.4, 128.5, 128.5, 128.5, 128.5, 128.4, 128.3, 128.0, 127.7, 127.7, 127.6, 124.9 (CH_{arom}), 104.2 (C-1), 82.7 (d, *J* = 169.8 Hz, CH_2F), 82.2 (C-3), 79.5 (C-2), 75.3, 74.6, 73.7 (CH_2 Bn), 73.6 (C-4), 73.6 (C-4), 73.5, 73.2 (CH_2 Bn), 69.0 (C-6), 68.8 (d, *J* = 20.2 Hz, CH_2CH_2F); ^{13}C-GATED NMR (126 MHz, $CDCl_3$): δ 104.2 ($J_{H1\text{-}C1}$ = 159 Hz, β); Diagnostic signals of the minor stereoisomer (α product): ^{1}H NMR (500 MHz, $CDCl_3$, HH-COSY, HSQC, HMBC-Gated): 4.86 (d, *J* = 3.6 Hz, 1H, H-1), 4.11 (ddd, *J* = 12.1, 4.4, 2.5, 1H, C*H*HCH_2F); ^{13}C NMR (126 MHz, $CDCl_3$, HSQC, HMBC-Gated): δ 98.1 (C-1), 82.7 (d, *J* = 169.8 Hz, CH_2F), 76.6 (C-2), 75.3, 74.9, 73.4 (CH_2 Bn), 69.5 (C-5), 69.1 (C-6), 67.1 (d, *J* = 20.3 Hz, CH_2CH_2F); ^{13}C-GATED NMR (126 MHz, $CDCl_3$): δ 98.1 ($J_{H1\text{-}C1}$ = 171 Hz, α); HRMS: $[M+Na]^+$ calcd for $C_{36}H_{39}FO_6$ 609.2628, found 609.2637.

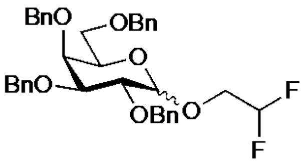

2,2-Difluoroethyl 2,3,4,6-tetra-*O*-benzyl-α/β-D-galactopyranoside (S79). The title compound was prepared according to general procedure VII. Column column chromatography (97:3 → 85:15, pentane:EtOAc) yielded the

title compound (42 mg, 69 µmol, 69%, colourless oil, α:β; 66:34). TLC: R_f 0.21, 0.31 (pentane:EtOAc, 90:10, v:v); IR (thin film, cm^{-1}): 696, 736, 1028, 1056, 1094, 1361, 1453, 1497, 2917; Data of the major stereoisomer (α product): ^1H NMR (500 MHz, $CDCl_3$, HH-COSY, HSQC, HMBC-Gated): δ 7.40 – 7.24 (m, 20H, CH_{arom}), 5.97 (tt, *J* = 55.6, 4.4 Hz, 1H, C*H*F_2), 4.94 (dd, *J* = 11.5, 1.3 Hz, 1H, C*H*H Bn), 4.86 – 4.79 (m, 3H, C*H*H Bn, CH*H* Bn, H-1), 4.73 (d, *J* = 11.6 Hz, 1H, C*H*H Bn), 4.65 (d, *J* = 11.9 Hz, 1H, CH*H* Bn), 4.56 (d, *J* = 11.4 Hz, 1H, CH*H* Bn), 4.46 (d, *J* = 11.8 Hz, 1H, C*H*H Bn), 4.43 – 4.37 (m, 1H, CH*H* Bn), 4.05 (dd, *J* = 10.0, 3.7 Hz, 1H, H-2), 3.98 – 3.90 (m, 3H, H-3, H-4, H-5), 3.73 (ddd, *J* = 14.7, 13.0, 4.4 Hz, 1H, C*H*HCHF_2), 3.80 – 3.66 (m, 1H, CH*H*CHF_2), 3.60 – 3.46 (m, 2H, H-6, H-6); ^{13}C NMR (126 MHz, $CDCl_3$, HSQC, HMBC-Gated): δ 138.8, 138.6, 138.6, 138.0 ($C_{q\text{-}arom}$), 128.5, 128.4, 128.4, 128.2, 127.8, 127.8, 127.7, 127.6 (CH_{arom}), 114.3 (t, *J* = 241.2 Hz, *C*HF_2), 98.9 (C-1), 78.9 (C-3), 76.5 (C-2), 75.1 (C-4), 74.9, 73.7, 73.6, 73.4 (CH_2 Bn), 69.9 (C-5), 69.1 (C-6), 67.5 (t, *J* = 28.9 Hz, *C*H_2CHF_2); ^{13}C-GATED NMR (126 MHz, $CDCl_3$): δ 98.9 ($J_{H1\text{-}C1}$ = 169 Hz, α); Diagnostic signals of the minor stereoisomer (β product): ^1H NMR (500 MHz, $CDCl_3$, HH-COSY, HSQC, HMBC-Gated): δ 5.91 (dddd, *J* = 56.3, 54.7, 5.4, 3.1 Hz, *C*HF_2), 4.88 (d, *J* = 10.8 Hz, 1H, C*H*H Bn), 4.61 (d, *J* = 11.6 Hz, 1H, CH*H* Bn), 4.39 (d, *J* = 7.6 Hz, 1H, H-1), 3.83 (dd, *J* = 9.7, 7.6 Hz, 1H, H-2); ^{13}C NMR (126 MHz, $CDCl_3$, HSQC, HMBC-Gated): δ 114.4 (dd, *J* = 242.1, 239.7 Hz, *C*HF_2), 104.3 (C-1), 79.3 (C-2), 75.4, 74.7 (CH_2 Bn), 73.8 (C-3), 73.7, 73.3 (CH_2 Bn), 68.9 (C-6); ^{13}C-GATED NMR (126 MHz, $CDCl_3$): δ 104.3 ($J_{H1\text{-}C1}$ = 159 Hz, β); HRMS: $[M+Na]^+$ calcd for $C_{36}H_{38}F_2O_6$ 627.2534, found 627.2542.

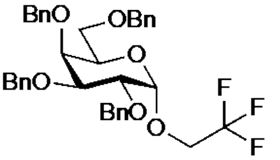

2,2,2-Trifluoroethyl 2,3,4,6-tetra-*O*-benzyl-α/β-D-galactopyranoside (S80). The title compound was prepared according to general procedure VII. Column chromatography (97:3 → 90:10, pentane:EtOAc) yielded the title compound (49 mg, 79 µmol, 79%, colourless oil, α:β; 87:13). TLC: R_f 0.28, 0.47 (pentane:EtOAc, 90:10, v:v); $[\alpha]_D^{20}$ −27.5° (*c* 1, $CHCl_3$); IR (thin film, cm^{-1}): 696, 735, 1079, 1153, 1278, 1351, 1453, 1497, 2915; Data of the major stereoisomer (α product): ^1H NMR (400 MHz, $CDCl_3$, HH-COSY, HSQC, ^{13}C-HMBC-Gated): δ 7.52 – 7.05 (m, 20H, CH_{arom}), 4.94 (d, *J* = 11.4 Hz, 1H, C*H*H Bn), 4.88 (d, *J* = 3.7 Hz, 1H, H-1), 4.87 – 4.79 (m, 2H, C*H*H Bn, CH*H* Bn), 4.73 (d, *J* = 11.6 Hz, 1H, CH*H* Bn), 4.65 (d, *J* = 11.9 Hz, 1H, CH*H* Bn), 4.56 (d, *J* = 11.4 Hz, 1H, C*H*H Bn), 4.46 (d, *J* = 11.9 Hz, 1H, C*H*H Bn), 4.39 (d, *J* = 11.8 Hz, 1H, CH*H* Bn), 4.07 (dd, *J* = 10.0, 3.7 Hz, 1H, H-2), 4.00 – 3.87 (m, 5H, C*H*HCF_3, CH*H*CF_3, H-3, H-4, H-5), 3.55 – 3.48 (m, 2H, H-6, H-6); ^{13}C NMR (101 MHz, $CDCl_3$, HSQC, ^{13}C-HMBC-Gated): δ 138.8, 138.6, 138.5, 138.0 ($C_{q\text{-}arom}$), 128.6, 128.6, 128.0, 128.0, 127.9, 127.9, 127.8, 127.8, 127.7, 127.6 (CH_{arom}), 122.6 (q, *J* = 281.9 Hz, *C*F_3), 98.3 (C-1), 78.7 (C-3), 76.3 (C-2), 75.0 (CH_2 Bn), 74.9 (C-4), 73.6, 73.5, 73.5 (CH_2 Bn), 70.2 (C-5), 68.9 (C-6), 64.6 (q, *J* = 34.7 Hz, *C*H_2CF_3); ^{13}C-GATED NMR (101 MHz, $CDCl_3$): δ 98.3 ($J_{H1\text{-}C1}$ = 172 Hz, α); Diagnostic signals of the minor stereoisomer (β product): ^1H NMR (400 MHz, $CDCl_3$, HH-COSY, HSQC, ^{13}C-HMBC-Gated): 4.17 (dq, *J* = 12.4, 8.8 Hz, 1H, C*H*HCF_3); ^{13}C NMR (101 MHz, $CDCl_3$, HSQC, ^{13}C-HMBC-Gated): 104.1 (C-1), 66.0 (q, *J* = 34.8 Hz, *C*H_2CF_3); HRMS: $[M+Na]^+$ calcd for $C_{36}H_{37}F_3O_6$ 645.2440, found 645.2445.

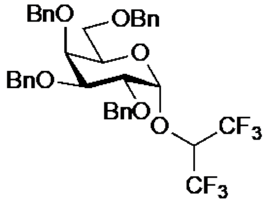

1,1,1,3,3,3-Hexafluoro-2-propyl 2,3,4,6-tetra-*O*-benzyl-α-D-galactopyranoside (S81). The title compound was prepared according to general procedure VII. Column chromatography (100:0 → 80:20, pentane:Et_2O) yielded the title compound (23 mg, 33 µmol, 33%, colourless oil, α:β; >98:2). TLC: R_f 0.38 (pentane:EtOAc, 95:5, v:v); $[\alpha]_D^{20}$ 58.8° (*c* 1, $CHCl_3$); IR (thin film, cm^{-1}): 689, 696, 734, 1028, 1051, 1102, 1195, 1218, 1287, 1169, 1454, 1497, 2926; ^{1}H NMR (500 MHz, $CDCl_3$, HH-COSY, HSQC, HMBC-Gated, ^{1}H-^{19}F Decoupled): δ 7.39 – 7.23 (m, 20H, CH_{arom}), 5.15 (d, *J* = 3.8 Hz, 1H, H-1), 4.94 (d, *J* = 11.3 Hz, 1H, C*H*H Bn), 4.84 (d, *J* = 11.5 Hz, 1H, C*H*H Bn), 4.79 – 4.66 (m, 3H, CH*H* Bn, CH*H* Bn, C*H*H Bn), 4.56 (d, *J* = 11.3 Hz, 1H, CH*H* Bn), 4.50 – 4.39 (m, 3H, C*H*H Bn, CH*H* Bn, C*H*$(CF_3)_2$), 4.13 (dd, *J* = 10.2, 3.8 Hz, 1H, H-2), 4.07 – 4.00 (m, 2H, H-4, H-5), 3.94 (dd, *J* = 10.2, 2.7 Hz, 1H, H-3), 3.57 – 3.46 (m, 2H, H-6, H-6); ^{13}C NMR (126 MHz, $CDCl_3$, HSQC, HMBC-Gated): δ 138.7, 138.5, 138.2, 138.0 (C_{q-arom}), 128.6, 128.5, 128.5, 128.4, 128.3, 128.0, 127.9, 127.9, 127.8, 127.8, 127.7, 127.6 (CH_{arom}), 99.9 (C-1), 78.4 (C-3), 75.5 (C-2), 75.0 (CH_2 Bn), 74.7 (C-4), 73.6, 73.6, 73.4 (CH_2 Bn), 72.5 (p, *J* = 33.3 Hz, C*H*$(CF_3)_2$), 70.9 (C-5), 68.4 (C-6); ^{13}C-GATED NMR (126 MHz, $CDCl_3$): δ 99.9 (J_{H1-C1} = 174 Hz, α); HRMS: $[M+Na]^+$ calcd for $C_{37}H_{36}F_6O_6$ 713.2314, found 713.2319.

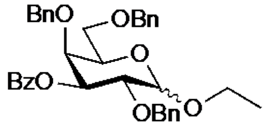

Ethyl 3-*O*-benzoyl-2,4,6-tri-*O*-benzyl-α/β-D-galactopyranoside (S82). The title compound was prepared according to general procedure VII. Column chromatography (100:0 → 85:15, pentane:Et_2O) yielded the title compound (46 mg, 79 µmol, 79%, colourless oil, α:β; 41:59). TLC: R_f 0.25, (pentane:Et_2O, 80:20, v:v); IR (thin film, cm^{-1}): 695, 735, 1026, 1069, 1270, 1720, 2869, 3063; Data of the major stereoisomer (β product): ^{1}H NMR (400 MHz, $CDCl_3$, HH-COSY, HSQC): δ 8.24 – 6.93 (m, 20H, CH_{arom}), 5.19 (dd, *J* = 10.2, 3.1 Hz, 1H, H-3), 4.86 (d, *J* = 11.5 Hz, 1H, C*H*H Bn), 4.70 (d, *J* = 11.4 Hz, 1H, CH*H* Bn), 4.66 – 4.60 (m, 2H, C*H*H Bn, CH*H* Bn), 4.54 – 4.39 (m, 3H, H-1, C*H*H Bn, CH*H* Bn), 4.22 – 4.12 (m, 1H, H-5), 4.07 (dd, *J* = 3.2, 1.0 Hz, 1H, H-4), 4.02 (dq, *J* = 9.5, 7.1 Hz, 1H, C*H*HCH_3), 3.93 (dd, *J* = 10.2, 7.7 Hz, 1H, H-2), 3.69 – 3.58 (m, 2H, H-6, CH*H*CH_3), 3.58 – 3.49 (m, 1H, H-6), 1.29 (t, *J* = 7.1 Hz, 3H, CH_3); ^{13}C NMR (101 MHz, $CDCl_3$, HSQC): δ 166.0 (C=O Bz), 138.4, 138.2, 138.0, 138.0 (C_{q-arom}), 130.1, 129.9, 128.5, 128.4, 128.4, 128.3, 128.1, 128.1, 127.9, 127.9, 127.9, 127.8, 127.8, 127.8, 127.8, 127.8, 127.7, 127.6 (CH_{arom}), 103.9 (C-1), 76.7 (C-2), 75.7 (C-3), 75.0, 74.7 (CH_2 Bn), 74.5 (C-4), 73.6 (CH_2 Bn), 68.8 (C-5), 68.6 (C-6), 65.8 (*C*H_2CH_3), 15.4 (*C*H_3); Diagnostic signals of the minor stereoisomer (α product): ^{1}H NMR (400 MHz, $CDCl_3$, HH-COSY, HSQC): δ 5.57 (dd, *J* = 10.5, 3.1 Hz, 1H, H-3), 4.90 (d, *J* = 3.6 Hz, 1H, H-1), 1.27 (t, *J* = 7.1 Hz, 3H, CH_3); ^{13}C NMR (101 MHz, $CDCl_3$, HSQC): δ 97.2 (C-1), 75.3 (CH_2 Bn), 74.3 (C-2), 73.5, 73.0 (CH_2 Bn), 68.8 (C-3), 68.7 (C-6), 63.7 (*C*H_2CH_3), 15.2 (*C*H_3); HRMS: $[M+NH_4]^+$ calcd for $C_{36}H_{38}O_7$ 600.29558, found 600.29551.

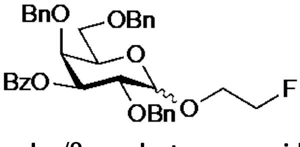

2-Fluoroethyl 3-*O*-benzoyl-2,4,6-tri-*O*-benzyl-α/β-D-galactopyranoside (S83). The title compound was prepared according to general procedure VII. Column chromatography (90:10 → 60:40, pentane:Et_2O) yielded the title

compound (49 mg, 82 µmol, 82%, colourless oil, α:β; 40:60). TLC: R_f 0.20, (pentane:Et_2O, 70:30, v:v); IR (thin film, cm^{-1}): 538, 696, 711, 735, 1026, 1043, 1070, 1077, 1270, 1452, 1720, 1870, 3030; Data of the major stereoisomer (β product): ^1H NMR (400 MHz, $CDCl_3$, HH-COSY, HSQC): δ 8.26 – 6.97 (m, 20H, CH_{arom}), 5.19 (dd, *J* = 10.2, 3.1 Hz, 1H, H-3), 4.87 (d, *J* = 11.4 Hz, 1H, C*H*H Bn), 4.76 – 4.58 (m, 3H, C*H*H Bn, C*H*H Bn, C*H*HCH2F), 4.55 (d, *J* = 7.7 Hz, 1H, H-1), 4.59 – 4.39 (m, 4H, CH*H* Bn, CH*H* Bn, CH*H* Bn, CH*H*CH2F), 4.19 – 4.05 (m, 1H, C*H*HF), 4.08 (d, *J* = 2.7 Hz, 1H, H-4), 3.96 (dd, *J* = 10.2, 7.6 Hz, 1H, H-2), 3.94 – 3.81 (m, 1H, CH*H*F), 3.77 (dd, *J* = 7.4, 6.1 Hz, 1H, H-5), 3.60 (t, *J* = 6.3 Hz, 2H, H-6), 3.55 (dd, *J* = 6.6, 1.3 Hz, 1H, H-6); ^{13}C NMR (101 MHz, $CDCl_3$, HSQC): δ 165.9 (C=O Bz), 138.2, 138.1, 138.0, 137.9 ($C_{q\text{-}arom}$), 130.0, 129.9, 129.7, 129.4, 128.5, 128.4, 128.4, 128.4, 128.3, 128.3, 128.2, 128.2, 128.1, 127.6 (CH_{arom}), 104.2 (C-1), 82.7 (d, *J* = 169.8 Hz, *C*H2F), 76.4 (C-2), 75.5 (C-3), 75.1, 74.7 (CH_2 Bn), 74.3 (C-4), 73.6 (CH_2 Bn), 73.4 (C-5), 69.0 (d, *J* = 20.1 Hz, *C*H2CH2F), 68.5 (C-6); Diagnostic signals of the minor stereoisomer (α product): ^1H NMR (400 MHz, $CDCl_3$, HH-COSY, HSQC): δ 5.59 (dd, *J* = 10.6, 3.1 Hz, 1H, H-3), 4.95 (d, *J* = 3.6 Hz, 1H, H-1), 4.18 (d, *J* = 3.7 Hz, 1H, H-4); ^{13}C NMR (101 MHz, $CDCl_3$, HSQC): δ 97.8 (C-1), 82.7 (d, *J* = 169.7 Hz, *C*H2F), 75.3, 73.4, 73.0 (CH_2 Bn), 67.2 (d, *J* = 20.2 Hz, *C*H2CH2F); HRMS: $[M+NH_4]^+$ calcd for $C_{36}H_{37}FO_7$ 618.28616, found 618.28638.

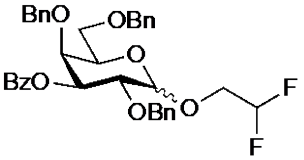

2,2-Difluoroethyl 3-*O*-benzoyl-2,4,6-tri-*O*-benzyl-α/β-D-galactopyranoside (S84). The title compound was prepared according to general procedure VII. Column chromatography (95:5 → 75:25, pentane:Et_2O) yielded the title compound (50 mg, 81 µmol, 81%, colourless oil, α:β; 66:34). TLC: R_f 0.20, (pentane:Et_2O, 80:20, v:v); IR (thin film, cm^{-1}): 695, 1026, 1069, 1093, 1270, 1452, 1720, 2869, 3030; Data of the major stereoisomer (α product): ^1H NMR (400 MHz, $CDCl_3$, HH-COSY, HSQC): δ 8.32 – 6.86 (m, 20H, CH_{arom}), 5.99 (tt, *J* = 55.5, 4.3 Hz, 1H, C*H*F2), 5.55 (dd, *J* = 10.6, 3.0 Hz, 1H, H-3), 4.91 (d, *J* = 3.7 Hz, 1H, H-1), 4.75 – 4.58 (m, 3H, C*H*H Bn, C*H*H Bn, CH*H* Bn), 4.56 – 4.36 (m, 3H, CH*H* Bn, C*H*H Bn, CH*H* Bn), 4.23 – 4.11 (m, 3H, H-2, H-3, H-4), 3.89 – 3.68 (m, 2H, C*H*HCHF2, CH*H*CHF2), 3.67 – 3.49 (m, 1H, H-6), 3.53 (dd, *J* = 6.5, 5.1 Hz, 2H, H-6); ^{13}C NMR (101 MHz, $CDCl_3$, HSQC): δ 165.9 (C=O Bz), 138.1, 138.0, 137.9, 137.8 ($C_{q\text{-}arom}$), 129.9, 129.7, 128.6, 128.5, 128.4, 128.4, 128.3, 128.3, 128.1, 128.0, 128.0, 128.0, 128.0, 127.9, 127.9, 127.8, 127.8 (CH_{arom}), 114.3 (t, *J* = 241.2 Hz, *C*HF2), 98.4 (C-1), 75.4 (C-2), 75.3 (CH_2 Bn), 74.0 (C-4), 73.5, 73.3 (CH_2 Bn), 72.7 (C-3), 69.4 (C-5), 68.6 (C-6), 67.5 (t, *J* = 29.0 Hz, *C*H2CHF2); Diagnostic signals of the minor stereoisomer (β product): ^1H NMR (400 MHz, $CDCl_3$, HH-COSY, HSQC): δ 5.19 (dd, *J* = 10.2, 3.2 Hz, 1H, H-3), 4.82 (d, *J* = 11.4 Hz, 1H, C*H*H Bn), 4.54 (d, *J* = 7.6 Hz, 1H, H-1), 4.08 (d, *J* = 3.2 Hz, 1H, H-4), 3.96 (dd, *J* = 10.2, 7.6 Hz, 1H, H-2); ^{13}C NMR (101 MHz, $CDCl_3$, HSQC): δ 114.3 (dd, *J* = 242.0, 240.0 Hz, *C*HF2), 104.3 (C-1), 76.4 (C-2), 75.1, 74.8, 73.6 (CH_2 Bn), 68.8 (dd, *J* = 30.4, 27.1 Hz, *C*H2CHF2), 68.4 (C-6); HRMS: $[M+NH_4]^+$ calcd for $C_{36}H_{36}F_2O_7$ 636.27674, found 636.27661.

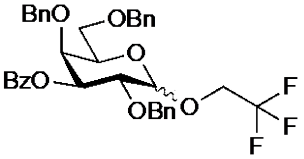

2,2,2-Trifluoroethyl 3-*O*-benzoyl-2,4,6-tri-*O*-benzyl-α/β-D-galactopyranoside (S85). The title compound was prepared according to general procedure VII. Column chromatography (95:5 → 80:20, pentane:Et_2O) yielded the title compound (49 mg, 77 µmol, 77%, colourless oil, α:β; 86:14). TLC: R_f 0.35, (pentane:Et_2O, 80:20, v:v); IR

(thin film, cm^{-1}): 695, 711, 735, 989, 1026, 1096, 1154, 1270, 1452, 1496, 1720, 2923, 3032; Data of the major stereoisomer (α product): ^{1}H NMR (400 MHz, $CDCl_3$, HH-COSY, HSQC): δ 8.16 – 7.12 (m, 20H, CH_{arom}), 5.53 (dd, *J* = 10.5, 3.1 Hz, 1H, H-3), 4.96 (d, *J* = 3.7 Hz, 1H, H-1), 4.71 (d, *J* = 12.3 Hz, 1H, C*H*H Bn), 4.63 (d, *J* = 11.5 Hz, 1H, C*H*H Bn), 4.62 (d, *J* = 12.2 Hz, 1H, CH*H* Bn), 4.50 (d, *J* = 11.9 Hz, 1H, C*H*H Bn), 4.43 (d, *J* = 11.4 Hz, 1H, CH*H* Bn), 4.47 – 4.39 (m, 1H, CH*H* Bn), 4.27 – 4.17 (m, 2H, H-2, H-4), 4.13 (td, *J* = 6.5, 1.2 Hz, 1H, H-5), 4.03 – 3.85 (m, 2H, C*H*HCF_3, CH*H*CF_3), 3.53 (dd, *J* = 6.6, 2.0 Hz, 2H, H-6, H-6); ^{13}C NMR (101 MHz, $CDCl_3$, HSQC): δ 165.9 (C=O Bz), 138.1, 137.9, 137.9, 137.8 ($C_{q\text{-}arom}$), 129.6, 128.6, 128.6, 128.5, 128.4, 128.4, 128.3, 128.0, 128.0, 128.0, 127.9 (CH_{arom}), 123.9 (q, *J* = 278.7 Hz, *C*F_3), 98.2 (C-1), 75.3 (CH_2 Bn), 75.3 (C-2), 73.8 (C-4), 73.5, 73.1 (CH_2 Bn), 72.7 (C-3), 69.7 (C-5), 68.4 (C-6), 64.9 (q, *J* = 34.9 Hz, *C*H_2CF_3); Diagnostic signals of the minor stereoisomer (β product): ^{1}H NMR (400 MHz, $CDCl_3$, HH-COSY, HSQC): δ 5.18 (dd, *J* = 10.2, 3.1 Hz, 1H, H-3), 4.84 (d, *J* = 11.3 Hz, 1H, C*H*H Bn), 4.60 (d, *J* = 7.5 Hz, 1H, H-1), 4.08 (dd, *J* = 3.2, 1.0 Hz, 1H, H-4); ^{13}C NMR (101 MHz, $CDCl_3$, HSQC): δ 104.1 (C-1), 76.1 (C-2), 75.2, 74.7, 73.6 (CH_2 Bn), 68.3 (C-6), 66.2 (d, *J* = 35.1 Hz, *C*H_2CF_3); HRMS: $[M+NH_4]^+$ calcd for $C_{36}H_{35}F_3O_7$ 654.26731, found 654.26738.

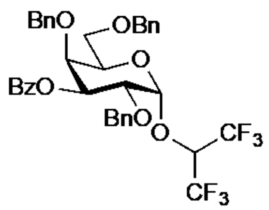

1,1,1,3,3,3-Hexafluoro-2-propyl 3-*O*-benzoyl-2,4,6-tri-*O*-benzyl-α-D-galactopyranoside (S86). The title compound was prepared according to general procedure VII. Column chromatography (100:0 → 80:20, pentane:Et_2O) yielded the title compound (38 mg, 55 µmol, 55%, colourless oil, α:β; >98:2). TLC: R_f 0.15, (pentane:Et_2O, 90:10, v:v); $[\alpha]_D^{25}$ 42.2° (*c* 1, $CHCl_3$); 687, 697, 735, 1105, 1196, 1219, 1315, 1452, 1496, 1720, 2363, 2868, 2936, 3065; ^{1}H NMR (500 MHz, $CDCl_3$, HH-COSY, HSQC): δ 8.22 – 7.13 (m, 20H, CH_{arom}), 5.51 (dd, *J* = 10.7, 3.0 Hz, 1H, H-3), 5.24 (d, *J* = 3.8 Hz, 1H, H-1), 4.69 (d, *J* = 12.1 Hz, 1H, C*H*H Bn), 4.63 (d, *J* = 12.0 Hz, 1H, CH*H* Bn), 4.61 (d, *J* = 11.3 Hz, 1H, C*H*H Bn), 4.50 (d, *J* = 12.1 Hz, 1H, CH*H* Bn), 4.49 – 4.41 (m, 3H, C*H*H Bn, CH*H* Bn, C*H*$(CF_3)_2$), 4.28 (dd, *J* = 10.7, 3.8 Hz, 1H, H-2), 4.26 – 4.22 (m, 2H, H-4, H-5), 3.60 – 3.48 (m, 2H, H-6, H-6); ^{13}C NMR (126 MHz, $CDCl_3$, HSQC): δ 165.9 (C=O Bz), 137.9, 137.6 ($C_{q\text{-}arom}$), 129.8, 128.6, 128.6, 128.5, 128.5, 128.2, 128.1, 128.0, 127.9, 127.9, 127.7 (CH_{arom}), 99.7 (C-1), 75.4 (CH_2 Bn), 75.0 (C-4), 73.5, 73.1 (CH_2 Bn), 72.9 (C-2), 72.4 (C-3), 70.3 (C-5), 68.0 (C-6); HRMS: $[M+NH_4]^+$ calcd for $C_{37}H_{34}F_6O_7$ 722.25470, found 722.25438.

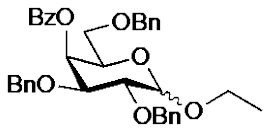

Ethyl 4-*O*-benzoyl-2,3,6-tri-*O*-benzyl-α/β-D-galactopyranoside (S87). The title compound was prepared according to general procedure VII. Column chromatography (95:5 → 70:30, pentane:Et_2O) yielded the title compound (50 mg, 86 µmol, 86%, colourless oil, α:β; 45:55). TLC: R_f 0.25, (pentane:Et_2O, 80:20x, v:v); IR (thin film, cm^{-1}): 696, 711, 1026, 1068, 1271, 1173, 1720, 2866, 3032; Data of the major stereoisomer (β product): ^{1}H NMR (500 MHz, $CDCl_3$, HH-COSY, HSQC): δ 8.29 – 7.03 (m, 20H, CH_{arom}), 5.83 (d, *J* = 1.9 Hz, 1H, H-4), 4.92 – 4.80 (m, 2H, C*H*H Bn, C*H*H Bn), 4.73 (d, *J* = 10.8 Hz, 1H, CH*H* Bn), 4.57 (d, *J* = 11.6 Hz, 1H, CH*H* Bn), 4.51 (d, *J* = 11.8 Hz, 1H, CH*H* Bn), 4.49 (d, *J* = 11.8 Hz, 1H, C*H*H Bn), 4.45 (d, *J* = 7.6 Hz, 1H, H-1), 4.04 (dq, *J* = 9.6, 7.1 Hz, 1H, C*H*HCH_3), 3.82 (ddd, *J* = 7.0, 5.9, 1.1 Hz, 1H, H-5), 3.71 – 3.57 (m, 3H, H-2, H-3, CH*H*CH_3), 3.53 (m, 2H, H-6, H-6), 1.32 (t, *J* = 7.1 Hz, 3H, C*H*$_3$); ^{13}C NMR (126 MHz, $CDCl_3$, HSQC): δ 166.0 (C=O Bz), 138.8, 138.7, 138.1, 137.8 ($C_{q\text{-}arom}$), 128.5,

128.4, 128.4, 128.4, 128.3, 128.3, 128.3, 128.2, 128.1, 128.0, 127.7, 127.7, 127.5 (CH_{arom}), 103.9 (C-1), 79.5 (C-3/C-2), 76.7 (C-2/C-3), 75.5, 73.9 (CH_2 Bn), 72.6 (C-5), 72.0 (CH_2 Bn), 68.6 (C-6), 67.6 (C-4), 66.1 ($\mathit{C}H_2CH_3$), 15.5 ($\mathit{C}H_3$); Diagnostic signals of the minor stereoisomer (α product): ^{1}H NMR (500 MHz, $CDCl_3$, HH-COSY, HSQC): δ 5.86 (dd, *J* = 3.5, 1.2 Hz, 1H, H-4), 4.92 – 4.80 (signal overlaps with major isomer, 1H, H-1), 4.65 (d, *J* = 12.1 Hz, 1H, CH*H* Bn), 4.22 (td, *J* = 6.3, 1.1 Hz, 1H, H-5), 4.10 (dd, *J* = 10.0, 3.3 Hz, 1H, H-3), 3.89 (dd, *J* = 10.0, 3.7 Hz, 1H, H-2), 3.76 (dq, *J* = 10.0, 7.1 Hz, 1H, C*H*HCH$_3$), 1.28 (t, *J* = 7.1 Hz, 3H, CH_3); ^{13}C NMR (126 MHz, $CDCl_3$, HSQC): δ 97.7 (C-1), 76.7 (C-3), 75.3 (C-2), 73.7, 73.6, 72.1 (CH_2 Bn), 68.9 (C-6), 68.1 (C-4), 15.1 ($\mathit{C}H_3$); HRMS: $[M+NH_4]^+$ calcd for $C_{36}H_{38}O_7$ 600.29558, found 600.29569.

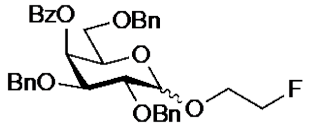

2-Fluoroethyl 4-*O*-benzoyl-2,3,6-tri-*O*-benzyl-α/β-D-galactopyranoside (S88). The title compound was prepared according to general procedure VII. Column chromatography (90:10 → 60:40, pentane:Et_2O) yielded the title compound (50 mg, 84 µmol, 84%, colourless oil, α:β; 63:37). TLC: R_f 0.20, (pentane:Et_2O, 70:30, v:v); IR (thin film, cm^{-1}): 536, 712, 740, 1090, 1270, 1720, 2870, 3032; Data of the major stereoisomer (α product): ^{1}H NMR (500 MHz, $CDCl_3$, HH-COSY, HSQC): δ 8.15 – 7.02 (m, 20H, CH_{arom}), 5.87 (dd, *J* = 3.4, 1.3 Hz, 1H, H-4), 4.95 (d, *J* = 3.7 Hz, 1H, H-1), 4.87 – 4.80 (m, 2H, C*H*H Bn, C*H*H Bn), 4.69 – 4.54 (m, 4H, CH*H* Bn, CH*H* Bn, C*H*HF, CH*H*F), 4.53 – 4.38 (m, 2H, C*H*H Bn, CH*H* Bn), 4.27 (td, *J* = 6.3, 1.3 Hz, 1H, H-5), 4.11 (dd, *J* = 10.1, 3.2 Hz, 1H, H-3), 3.92 (dd, *J* = 9.9, 3.7 Hz, 1H, H-2), 3.90 – 3.74 (m, 2H, C*H*HCH$_2$F, CHHCH$_2$F), 3.60 – 3.49 (m, 2H, H-6, H-6); ^{13}C NMR (126 MHz, $CDCl_3$, HSQC): δ 165.9 (C=O Bz), 138.6, 138.5, 138.0, 137.8 ($C_{q\text{-}arom}$), 129.9, 128.5, 128.5, 128.4, 128.4, 128.4, 128.4, 128.3, 128.2, 128.1, 127.8, 127.8, 127.7, 127.6 (CH_{arom}), 98.4 (C-1), 82.8 (d, *J* = 169.8 Hz, $\mathit{C}H_2F$), 76.5 (C-3), 75.2 (C-2), 73.7, 73.6, 72.1 (CH_2 Bn), 68.8 (C-4), 68.8 (C-6), 68.2 (C-5), 67.3 (d, *J* = 20.1 Hz, $\mathit{C}H_2CH_2F$); Diagnostic signals of the minor stereoisomer (β product): ^{1}H NMR (500 MHz, $CDCl_3$, HH-COSY, HSQC): δ 5.82 (dd, *J* = 3.3, 1.1 Hz, 1H, H-4), 4.50 (d, *J* = 7.4 Hz, 1H, H-1); ^{13}C NMR (126 MHz, $CDCl_3$, HSQC): 104.2 (C-1), 69.3 (d, *J* = 20.1 Hz, $\mathit{C}H_2CH_2F$), 67.5 (C-4); HRMS: $[M+NH_4]^+$ calcd for $C_{36}H_{37}FO_7$ 618.28616, found 618.28617.

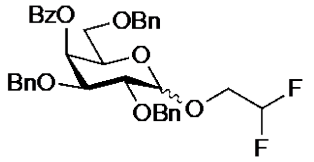

2,2-Difluoroethyl 4-*O*-benzoyl-2,3,6-tri-*O*-benzyl-α/β-D-galactopyranoside (S89). The title compound was prepared according to general procedure VII. Column chromatography (95:5 → 80:20, pentane:Et_2O) yielded the title compound (40 mg, 65 µmol, 65%, colourless oil, α:β; 44:56). TLC: R_f 0.30, (pentane:Et_2O, 80:20, v:v); IR (thin film, cm^{-1}): 738, 1026, 1267, 1720, 2869, 3032; Data of the major stereoisomer (α product): ^{1}H NMR (400 MHz, $CDCl_3$, HH-COSY, HSQC): δ 8.44 – 7.02 (m, 20H, CH_{arom}), 6.02 (tt, *J* = 55.5, 4.3 Hz, 1H, C*H*F$_2$), 5.86 (dd, *J* = 3.4, 1.2 Hz, 1H, H-4), 4.91 (d, *J* = 3.7 Hz, 1H, H-1), 4.85 (d, *J* = 11.2 Hz, 1H, C*H*H Bn), 4.83 (d, *J* = 11.9 Hz, 1H, C*H*H Bn), 4.64 (d, *J* = 11.9 Hz, 1H, CH*H* Bn), 4.58 (d, *J* = 11.2 Hz, 1H, CH*H* Bn), 4.49 (d, *J* = 11.8 Hz, 1H, C*H*H Bn), 4.41 (d, *J* = 11.9 Hz, 1H, CH*H* Bn), 4.21 (td, *J* = 6.3, 1.3 Hz, 1H, H-5), 4.07 (dd, *J* = 10.1, 3.2 Hz, 1H, H-3), 3.92 (dd, *J* = 10.1, 3.6 Hz, 1H, H-2), 3.80 (m, 2H, C*H*HCHF$_2$, CH*H*CHF$_2$), 3.53 (m, 2H, H-6, H-6); ^{13}C NMR (101 MHz, $CDCl_3$, HSQC): δ 165.8 (C=O Bz), 138.3, 138.1, 137.7 ($C_{q\text{-}arom}$), 128.5, 128.5, 128.4, 128.4, 128.4, 128.2, 128.1, 128.0, 127.9, 127.8, 127.8, 127.6 (CH_{arom}), 114.3 (t, *J* = 241.3 Hz, $\mathit{C}HF_2$), 99.1 (C-1), 76.3 (C-3), 75.1 (C-2), 73.9, 73.7, 72.1 (CH_2 Bn), 68.7 (C-6), 68.6 (C-5), 67.7 (t, *J* = 28.9 Hz, $\mathit{C}H_2CHF_2$); Diagnostic signals of the minor stereoisomer (β product): ^{1}H NMR

(400 MHz, $CDCl_3$, HH-COSY, HSQC): δ 5.82 (dd, *J* = 2.9, 1.1 Hz, 1H, H-4), 4.72 (d, *J* = 10.7 Hz, 1H, CH*H* Bn), 4.52-4.46 (signal overlaps with major isomer, 1H, H-1); ^{13}C NMR (101 MHz, $CDCl_3$, HSQC): δ 104.3 (C-1), 72.1 (CH_2 Bn); HRMS: $[M+NH_4]^+$ calcd for $C_{36}H_{36}F_2O_7$ 636.27674, found 636.27679.

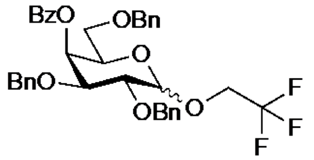

2,2,2-Trifluoroethyl 4-*O*-benzoyl-2,3,6-tri-*O*-benzyl-α/β-D-galactopyranoside (S90). The title compound was prepared according to general procedure VII. Column chromatography (95:5 → 85:15, pentane:Et_2O) yielded the title compound (51 mg, 79 µmol, 79%, colourless oil, α:β; 97:3). TLC: R_f 0.40, (pentane:Et_2O, 80:20, v:v); IR (thin film, cm^{-1}): 596, 696, 735, 1054, 1267, 1720, 2873, 3032; Data of the major stereoisomer (α product): ^{1}H NMR (500 MHz, $CDCl_3$, HH-COSY, HSQC): δ 8.43 – 6.88 (m, 20H, CH_{arom}), 5.87 (dd, *J* = 3.4, 1.2 Hz, 1H, H-4), 4.97 (d, *J* = 3.7 Hz, 1H, H-1), 4.85 (d, *J* = 11.1 Hz, 1H, C*H*H Bn), 4.82 (d, *J* = 11.9 Hz, 1H, C*H*H Bn), 4.63 (d, *J* = 12.0 Hz, 1H, CH*H* Bn), 4.58 (d, *J* = 11.1 Hz, 1H, CH*H* Bn), 4.49 (d, *J* = 11.9 Hz, 1H, C*H*H Bn), 4.41 (d, *J* = 11.9 Hz, 1H, CH*H* Bn), 4.19 (td, *J* = 6.3, 1.3 Hz, 1H, H-5), 4.08 (dd, *J* = 10.1, 3.3 Hz, 1H, H-3), 4.00 – 3.91 (m, 3H, H-2, C*H*HCF$_3$, CH*H*CF$_3$), 3.60 – 3.46 (m, 2H, H-6, H-6); ^{13}C NMR (126 MHz, $CDCl_3$, HSQC): δ 165.8 (C=O Bz), 138.4, 138.2, 137.7 ($C_{q\text{-}arom}$), 130.0, 128.5, 128.5, 128.5, 128.4, 128.4, 128.3, 128.1, 128.0, 127.9, 127.9, 127.8, 127.8, 127.2 (CH_{arom}), 123.9z (q, *J* = 278.8 Hz, *C*F$_3$), 98.7 (C-1), 76.1 (C-3), 75.0 (C-2), 73.7, 73.7, 72.2 (CH_2 Bn), 68.9 (C-5), 68.6 (C-6), 68.5 (C-4), 64.9 (q, *J* = 34.9 Hz, *C*H$_2$CF$_3$); Diagnostic signals of the minor stereoisomer (β product): ^{1}H NMR (500 MHz, $CDCl_3$, HH-COSY, HSQC): δ 5.72 (d, *J* = 3.9 Hz, 1H, H-4), 4.55 (d, *J* = 7.4 Hz, 1H, H-1); ^{13}C NMR (126 MHz, $CDCl_3$, HSQC): δ 104.0 (C-1), 75.6, 73.9 (CH_2 Bn); HRMS: $[M+NH_4]^+$ calcd for $C_{36}H_{35}F_3O_7$ 654.26731, found 654.26699.

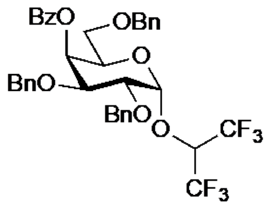

1,1,1,3,3,3-Hexafluoro-2-propyl 4-*O*-benzoyl-2,3,6-tri-*O*-benzyl-α-D-galactopyranoside (S91). The title compound was prepared according to general procedure VII. Column chromatography (100:0 → 85:15, pentane:Et_2O) yielded the title compound (39 mg, 55 µmol, 55%, colourless oil, α:β; >98:2). TLC: R_f 0.20, (pentane:Et_2O, 90:10, v:v); $[\alpha]_D^{25}$ 4.5° (*c* 1, $CHCl_3$); IR (thin film, cm^{-1}): 538, 685, 749, 1101, 1269, 1720, 2850, 3032; ^{1}H NMR (500 MHz, $CDCl_3$, HH-COSY, HSQC): δ 8.55 – 7.03 (m, 20H, CH_{arom}), 5.99 – 5.88 (m, 1H, H-4), 5.22 (d, *J* = 3.8 Hz, 1H, H-1), 4.86 (d, *J* = 10.9 Hz, 1H, C*H*H Bn), 4.78 (d, *J* = 11.8 Hz, 1H, C*H*H Bn), 4.66 (d, *J* = 11.7 Hz, 1H, CH*H* Bn), 4.58 (d, *J* = 10.9 Hz, 1H, CH*H* Bn), 4.53 – 4.46 (m, 2H, C*H*H Bn, C*H*(CF$_3$)$_2$), 4.41 (d, *J* = 11.9 Hz, 1H, CH*H* Bn), 4.30 (dd, *J* = 7.0, 5.7 Hz, 1H, H-5), 4.09 (dd, *J* = 10.2, 3.2 Hz, 1H, H-3), 3.98 (dd, *J* = 10.2, 3.8 Hz, 1H, H-2), 3.57 – 3.46 (m, 2H, H-6, H-6); ^{13}C NMR (126 MHz, $CDCl_3$, HSQC): δ 136.7, 133.8 ($C_{q\text{-}arom}$), 130.0, 129.6, 128.9, 128.6, 128.5, 128.5, 128.4, 128.1, 128.1, 127.9, 127.9, 127.8, 127.7, 127.7 (CH_{arom}), 100.2 (C-1), 76.0 (C-3), 74.3 (C-2), 73.9, 73.7, 72.1 (CH_2 Bn), 69.6 (C-5), 68.2 (C-4), 68.2 (C-6); HRMS: $[M+NH_4]^+$ calcd for $C_{37}H_{34}F_6O_7$ 722.25470, found 722.25409.

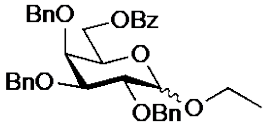

Ethyl 6-*O*-benzoyl-2,3,4-tri-*O*-benzyl-α/β-D-galactopyranoside (S92). The title compound was prepared according to general procedure VII. Column chromatography (95:5 → 75:25, pentane:Et_2O) yielded the title compound (51 mg, 88 µmol, 88%, colourless oil, α:β; 15:85). TLC: R_f 0.25, (pentane:Et_2O, 80:20, v:v); IR (thin film, cm^{-1}): 696, 1027, 1070, 1270, 1452, 1720, 2871, 3032; Data of the major stereoisomer (β product): ^{1}H NMR (500 MHz, $CDCl_3$, HH-COSY, HSQC): δ 8.54 – 7.08 (m, 20H, CH_{arom}), 5.00 (d, *J* = 11.6 Hz, 1H, C*H*H Bn), 4.95 (d, *J* = 10.9 Hz, 1H, C*H*H Bn), 4.84 (d, *J* = 11.7 Hz, 1H, C*H*H Bn), 4.78 (d, *J* = 10.9 Hz, 1H, CH*H* Bn), 4.74 (d, *J* = 11.8 Hz, 1H, CH*H* Bn), 4.70 (d, *J* = 11.6 Hz, 1H, CH*H* Bn), 4.48 (dd, *J* = 11.0, 6.5 Hz, 1H, H-6), 4.39 (d, *J* = 7.7 Hz, 1H, H-1), 4.33 (dd, *J* = 11.0, 6.5 Hz, 1H, H-6), 3.99 (dq, *J* = 9.5, 7.0 Hz, 1H, C*H*HCH_3), 3.87 (dd, *J* = 9.7, 7.7 Hz, 1H, H-2), 3.85 (dd, *J* = 2.9, 1.1 Hz, 1H, H-4), 3.67 (td, *J* = 6.5, 1.1 Hz, 1H, H-5), 3.65 – 3.57 (m, 1H, CH*H*CH_3), 3.55 (dd, *J* = 9.8, 2.9 Hz, 1H, H-3); ^{13}C NMR (126 MHz, $CDCl_3$, HSQC): δ 166.2 (C=O Bz), 138.9, 138.6, 138.4 ($C_{q\text{-}arom}$), 130.0, 128.6, 128.6, 128.5, 128.5, 128.4, 128.4, 128.3, 128.2, 127.8, 127.8, 127.7, 127.7, 127.7 (CH_{arom}), 104.0 (C-1), 82.4 (C-3), 79.7 (C-2), 75.3, 74.5, 73.7 (CH_2 Bn), 73.3 (C-4), 72.1 (C-5), 65.7 (*C*H_2CH_3), 63.4 (C-6), 15.4 (CH_3); Diagnostic signals of the minor stereoisomer (α product): ^{1}H NMR (500 MHz, $CDCl_3$, HH-COSY, HSQC): δ 4.92 (d, *J* = 11.8 Hz, 1H, C*H*H Bn), 4.86 – 4.81 (signal overlaps with major isomer, 1H, H-1), 4.66 (d, *J* = 11.6 Hz, 1H, CH*H* Bn), 4.26 (dd, *J* = 11.1, 5.7 Hz, 1H, H-6); ^{13}C NMR (126 MHz, $CDCl_3$, HSQC): δ 97.3 (C-1), 74.7, 73.8, 73.6 (CH_2 Bn), 15.1 (*C*H_3); HRMS: $[M+NH_4]^+$ calcd for $C_{36}H_{38}O_7$ 600.29558, found 600.29557.

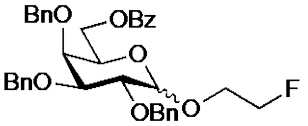

2-Fluoroethyl 6-*O*-benzoyl-2,3,4-tri-*O*-benzyl-α/β-D-galactopyranoside (S93). The title compound was prepared according to general procedure VII. Column chromatography (95:5 → 60:40, pentane:Et_2O) yielded the title compound (50 mg, 81 µmol, 81%, colourless oil, α:β; 33:67). TLC: R_f 0.20, (pentane:Et_2O, 70:30, v:v); IR (thin film, cm^{-1}): 711, 734, 1026, 1270, 1452, 1720, 2900; Data of the major stereoisomer (β product): ^{1}H NMR (500 MHz, $CDCl_3$, HH-COSY, HSQC): δ 8.01 – 7.03 (m, 20H, CH_{arom}), 5.00 (d, *J* = 11.7 Hz, 1H, C*H*H Bn), 4.97 (d, *J* = 10.8 Hz, 1H, C*H*H Bn), 4.85 (d, *J* = 11.8 Hz, 1H, C*H*H Bn), 4.80 – 4.55 (m, 5H, CH*H* Bn, CH*H* Bn, CH*H* Bn, C*H*HF, CH*H*F), 4.48 (dd, *J* = 11.1, 6.5 Hz, 1H, H-6), 4.45 (d, *J* = 7.7 Hz, 1H, H-1), 4.31 (dd, *J* = 11.1, 6.4 Hz, 1H, H-6), 3.90 (dd, *J* = 9.8, 7.7 Hz, 1H, H-2), 3.89 – 3.73 (m, 2H, C*H*HCH_2F, CH*H*CH_2F), 3.85 (dd, *J* = 2.8, 0.8 Hz, 1H, H-4), 3.68 (td, *J* = 6.5, 1.1 Hz, 1H, H-5), 3.56 (dd, *J* = 9.7, 2.9 Hz, 1H, H-3); ^{13}C NMR (126 MHz, $CDCl_3$, HSQC): δ 166.2 (C=O Bz), 138.9, 138.7, 138.6, 138.5, 138.3, 138.2 ($C_{q\text{-}arom}$), 130.0, 129.9, 128.6, 128.5, 128.5, 128.5, 128.4, 128.2, 127.9, 127.8, 127.8, 127.8, 127.7 (CH_{arom}), 104.3 (C-1), 82.8 (d, *J* = 169.7 Hz, *C*H_2F), 82.2 (C-3), 79.5 (C-2), 75.4, 74.5, 73.7 (CH_2 Bn), 73.2 (C-4), 72.3 (C-5), 68.9 (d, *J* = 20.2 Hz, *C*H_2CH_2F), 63.3 (C-6); Diagnostic signals of the minor stereoisomer (α product): ^{1}H NMR (500 MHz, $CDCl_3$, HH-COSY, HSQC): δ 4.92 (d, *J* = 11.7 Hz, 1H, C*H*H Bn), 4.88 (d, *J* = 3.6 Hz, 1H, H-1), 4.41 (dd, *J* = 11.2, 7.1 Hz, 1H, H-6), 4.25 (dd, *J* = 11.2, 5.5 Hz, 1H, H-6), 4.02 (dd, *J* = 10.1, 2.8 Hz, 1H, H-2), 3.96 (dd, *J* = 2.8, 1.3 Hz, 1H, H-4).; ^{13}C NMR (126 MHz, $CDCl_3$, HSQC): δ 98.1 (C-1), 82.7 (d, *J* = 169.8 Hz, *C*H_2F), 76.6 (C-2), 74.8, 73.8, 73.7 (CH_2 Bn), 67.2 (d, *J* = 20.2 Hz, *C*H_2CH_2F), 64.0 (C-6); HRMS: $[M+NH_4]^+$ calcd for $C_{36}H_{37}FO_7$ 618.28616, found 618.28635.

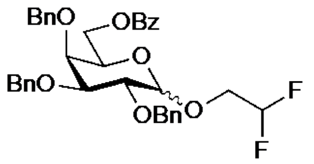

2,2-Difluoroethyl 6-*O*-benzoyl-2,3,4-tri-*O*-benzyl-α/β-D-galactopyranoside (S94). The title compound was prepared according to general procedure VII. Column chromatography (95:5 → 75:25, pentane:Et_2O) yielded the title compound (50 mg, 81 µmol, 81%, colourless oil, α:β; 61:39). TLC: R_f 0.30, (pentane:Et_2O, 80:20, v:v); IR (thin film, cm^{-1}): 711, 735, 1270, 1452, 1717, 2916, 3032; Data of the major stereoisomer (α product): ^1H NMR (500 MHz, $CDCl_3$, HH-COSY, HSQC): δ 8.47 – 6.72 (m, 20H, CH_{arom}), 5.95 (tt, *J* = 55.4, 4.3 Hz, 1H, C*H*F_2), 5.00 (d, *J* = 10.9 Hz, 1H, C*H*H Bn), 4.91 (d, *J* = 11.7 Hz, 1H, C*H*H Bn), 4.85 (d, *J* = 12.1 Hz, 1H, CH*H* Bn), 4.77 (d, *J* = 11.6 Hz, 1H, C*H*H Bn), 4.67 (d, *J* = 12.0 Hz, 1H, CH*H* Bn), 4.65 (d, *J* = 11.4 Hz, 1H, CH*H* Bn), 4.43 (d, *J* = 7.7 Hz, 1H, H-1), 4.41 (dd, *J* = 11.3, 7.3 Hz, 1H, H-6), 4.24 (dd, *J* = 11.3, 5.1 Hz, 1H, H-6), 4.14 – 4.05 (m, 2H, H-2, H-5), 4.00 – 3.94 (m, 2H, H-3, H-4), 3.80 – 3.65 (m, 2H, C*H*HCHF_2, CH*H*CHF_2); ^{13}C NMR (126 MHz, $CDCl_3$, HSQC): δ 166.3 (C=O Bz), 138.7, 138.5, 138.4, 138.1 ($C_{q\text{-}arom}$), 129.9, 129.8, 128.6, 128.6, 128.6, 128.6, 128.5, 128.5, 128.5, 128.2, 128.0, 127.9, 127.8, 127.8, 127.8, 127.7 (CH_{arom}), 114.2 (t, *J* = 241.3 Hz *C*HF_2), 98.7 (C-1), 78.9 (C-3), 76.4 (C-2), 74.9 (C-4), 74.8, 73.9, 73.8 (CH_2 Bn), 69.2 (C-5), 67.5 (t, *J* = 28.9 Hz, *C*H_2CHF_2), 64.1 (C-6); Diagnostic signals of the minor stereoisomer (β product): ^1H NMR (500 MHz, $CDCl_3$, HH-COSY, HSQC): δ 4.47 (dd, *J* = 11.2, 6.7 Hz, 1H, H-6), 4.43 (d, *J* = 7.7 Hz, 1H, H-1), 4.32 (dd, *J* = 11.2, 6.1 Hz, 1H, H-6), 3.90 (dd, *J* = 9.7, 7.6 Hz, 1H, H-2), 3.85 (dd, *J* = 2.9, 1.1 Hz, 1H, H-4), 3.56 (dd, *J* = 9.7, 2.8 Hz, 1H, H-3); ^{13}C NMR (126 MHz, $CDCl_3$, HSQC): δ 114.4 (dd, *J* = 241.1, 239.3 Hz, *C*HF_2), 104.4 (C-1), 82.2 (C-3), 79.3 (C-2), 75.5, 74.6, 73.8 (CH_2 Bn), 73.2 (C-4), 68.8 (dd, *J* = 30.8, 26.5 Hz, *C*H_2CHF_2), 63.3 (C-6); HRMS: $[M+NH_4]^+$ calcd for $C_{36}H_{36}F_2O_7$ 636.27674, found 636.27668.

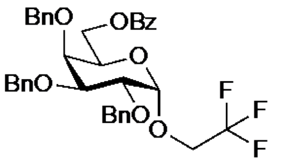

2,2,2-Trifluoroethyl 3-*O*-benzoyl-2,3,4-tri-*O*-benzyl-α/β-D-galactopyranoside (S95). The title compound was prepared according to general procedure VII. Column chromatography (95:5 → 80:20, pentane:Et_2O) yielded the title compound (47 mg, 74 µmol, 74%, colourless oil, α:β; 89:11). TLC: R_f 0.40, (pentane:Et_2O, 80:20, v:v); $[\alpha]_D^{25}$ 6.7° (*c* 1, $CHCl_3$); IR (thin film, cm^{-1}): 549, 711, 735, 1026, 1101, 1273, 1452, 1720, 2917, 3030; Data of the major stereoisomer (α product): ^1H NMR (500 MHz, $CDCl_3$, HH-COSY, HSQC): δ 8.34 – 7.02 (m, 20H, CH_{arom}), 5.00 (d, *J* = 11.4 Hz, 1H, C*H*H Bn), 4.91 (d, *J* = 11.4 Hz, 1H, C*H*H Bn), 4.89 (d, *J* = 3.7 Hz, 1H, H-1), 4.84 (d, *J* = 11.9 Hz, 1H, C*H*H Bn), 4.77 (d, *J* = 11.7 Hz, 1H, CH*H* Bn), 4.67 (d, *J* = 11.9 Hz, 1H, CH*H* Bn), 4.65 (d, *J* = 11.4 Hz, 1H, CH*H* Bn), 4.42 (dd, *J* = 11.3, 7.1 Hz, 1H, H-6), 4.24 (dd, *J* = 11.3, 5.4 Hz, 1H, H-6), 4.12 (dd, *J* = 9.8, 3.7 Hz, 1H, H-2), 4.06 (ddd, *J* = 6.8, 5.1, 1.1 Hz, 1H, H-5), 4.02 – 3.94 (m, 2H, H-3, H-4), 3.94 – 3.83 (m, 2H, C*H*HCF_3, CH*H*CF_3); ^{13}C NMR (126 MHz, $CDCl_3$, HSQC): δ 166.2 (C=O Bz), 138.7, 138.5, 138.4, 138.1 ($C_{q\text{-}arom}$), 129.8, 129.8, 129.7, 128.6, 128.6, 128.5, 128.5, 128.4, 128.1, 127.9, 127.9, 127.9, 127.7, 127.7, 127.7, 127.2 (CH_{arom}), 123.9 (q, *J* = 278.6 Hz, *C*F_3), 98.4 (C-1), 78.7 (C-3), 76.3 (C-2), 74.8 (CH_2 Bn), 74.8 (C-4), 73.9, 73.7 (CH_2 Bn), 64.7 (q, *J* = 34.9 Hz, *C*H_2CF_3), 63.8 (C-6); Diagnostic signals of the minor stereoisomer (β product): ^1H NMR (500 MHz, $CDCl_3$, HH-COSY, HSQC): δ 4.51 (d, *J* = 7.6 Hz, 1H, H-1), 3.56 (dd, *J* = 9.7, 2.9 Hz, 1H, H-3); ^{13}C NMR (126 MHz, $CDCl_3$, HSQC): δ 104.1 (C-1), 75.5, 74.6, 73.8 (CH_2 Bn), 66.0 (q, *J* = 35.0 Hz, CH_2CF_3), 63.2 (C-6); HRMS: $[M+NH_4]^+$ calcd for $C_{36}H_{35}F_3O_7$ 654.26731, found 654.26715.

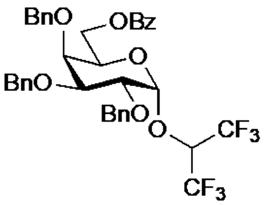

1,1,1,3,3,3-Hexafluoro-2-propyl 6-*O*-benzoyl-2,3,4-tri-*O*-benzyl-α-D-galactopyranoside (S96). The title compound was prepared according to general procedure VII. Column chromatography (100:0 → 85:15, pentane:Et_2O) yielded the title compound (40 mg, 57 µmol, 57%, colourless oil, α:β; >98:2). TLC: R_f 0.15, (pentane:Et_2O, 90:10x, v:v); $[\alpha]_D^{25}$ −47.0° (*c* 1, $CHCl_3$); IR (thin film, cm^{-1}): 538, 595, 697, 1027, 1104, 1196, 1274, 1452, 1720, 2924, 3032; Data of the major stereoisomer (α product): ^1H NMR (500 MHz, $CDCl_3$, HH-COSY, HSQC): δ 8.07 – 7.03 (m, 20H, CH_{arom}), 5.21 (d, *J* = 3.8 Hz, 1H, H-1), 5.00 (d, *J* = 11.3 Hz, 1H, C*H*H Bn), 4.91 (d, *J* = 11.5 Hz, 1H, C*H*H Bn), 4.79 – 4.69 (m, 3H, C*H*H Bn, CH*H* Bn, CH*H* Bn), 4.65 (d, *J* = 11.3 Hz, 1H, CH*H* Bn), 4.47 (dt, *J* = 11.9, 5.9 Hz, 1H, C*H*(CF_3)$_2$), 4.42 (dd, *J* = 11.4, 7.2 Hz, 1H, H-6), 4.25 (dd, *J* = 11.5, 4.9 Hz, 1H, H-6), 4.21 – 4.15 (m, 2H, H-2, H-5), 4.01 (t, *J* = 1.9 Hz, 1H, H-4), 3.99 (dd, *J* = 10.0, 2.7 Hz, 1H, H-2); ^{13}C NMR (126 MHz, $CDCl_3$, HSQC): δ 166.2 (C=O Bz), 138.5, 138.0 ($C_{q\text{-}arom}$), 129.7, 129.6, 128.9, 128.6, 128.6, 128.5, 128.1, 128.0, 127.7, 127.6 (CH_{arom}), 99.8 (C-1), 78.3 (C-3), 75.4 (C-2), 74.9 (CH_2 Bn), 74.7 (C-4), 73.8, 73.7 (CH_2 Bn), 70.5 (C-5), 64.0 (C-6); HRMS: $[M+NH_4]^+$ calcd for $C_{37}H_{34}F_6O_7$ 722.25470, found 722.25408.

Me/Ac-protected glycosyl donors

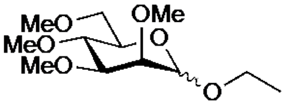

Ethyl 2,3,4,6-tetra-*O*-methyl-α/β-D-mannopyranoside (S97). The title compound was prepared according to general procedure VII. Column chromatography (80:20 → 70:30, pentane:EtOAc) yielded the title compound (18.2 mg, 69 μmol, 69%, colourless oil, α:β; 51:49). TLC: R_f 0.30, (pentane:EtOAc, 60:40, v:v); IR (thin film, cm^{-1}): 926, 1069, 1110, 1377, 2973; Data of the major stereoisomer (α product): ^1H NMR (400 MHz, $CDCl_3$, HH-COSY, HSQC): δ 4.92 (d, *J* = 1.6 Hz, 1H, H-1), 3.79 – 3.71 (m, 1H, C*H*H Et), 3.70 – 3.46 (m, 18H, H-2, H-3, H-4, H-5, 2x H-6, CH*H* Et, 3x CH_3 Me), 3.40 (s, 3H, CH_3 Me), 1.22 – 1.18 (m, 3H, CH_3 Et); ^{13}C NMR (101 MHz, $CDCl_3$, HSQC): δ 96.6 (C-1), 81.3 (C-3), 77.4, 77.1 (C-2/C-4), 71.9 (C-6), 71.3 (C-5), 63.1 (CH_2 Et), 60.9, 59.4, 59.3, 57.8 (CH_3 Me), 15.1 (CH_3 Et); ^{13}C-HMBC-GATED NMR (101 MHz, $CDCl_3$): δ 96.6 (J_{H1-C1} = 167 Hz, α); Diagnostic signals of the minor stereoisomer (β product): ^1H NMR (400 MHz, $CDCl_3$, HH-COSY, HSQC): δ 4.39 (d, *J* = 0.8 Hz, 1H, H-1), 3.99 (dq, *J* = 9.4, 7.1 Hz, 1H, C*H*H Et), 3.64 (s, 3H, CH_3 Me), 3.19 (dd, *J* = 8.9, 3.2 Hz, 1H, H-3), 1.26 – 1.22 (m, 3H, CH_3 Et); ^{13}C NMR (101 MHz, $CDCl_3$, HSQC): δ 101.3 (C-1), 84.2 (C-3), 72.2 (C-6), 65.3 (CH_2 Et), 15.2 (CH_3 Et); ^{13}C-HMBC-GATED NMR (101 MHz, $CDCl_3$): δ 101.3 (J_{H1-C1} = 154 Hz, β) ; HRMS: $[M+NH_4]^+$ calcd for $C_{13}H_{28}NO_7$ 282.19111, found 282.19093.

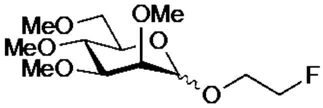

2-Fluoroethyl 2,3,4,6-tetra-*O*-methyl-α/β-D-mannopyranoside (S98). The title compound was prepared according to general procedure VII. Column chromatography (70:30 → 60:40, pentane:EtOAc) yielded the title compound (21 mg, 74 μmol, 74%, colourless oil, α:β; 64:36). TLC: R_f 0.21, (pentane:EtOAc, 60:40, v:v); IR (thin film, cm^{-1}): 730, 912, 1083, 1110, 2898; Data of the major stereoisomer (α product): ^1H NMR (400 MHz, $CDCl_3$, HH-COSY, HSQC): δ 4.96 (d, *J* = 1.8 Hz, 1H, H-1), 4.63 (dd, *J* = 4.9, 3.4 Hz, 1H, CH_2C*H*HF), 4.51 (dd, *J* = 4.8, 3.5 Hz, 1H, CH_2CH*H*F), 3.97 – 3.82 (m, 1H, C*H*HCH_2F), 3.81 – 3.67 (m, 2H, H-4, CH*H*CH_2F), 3.64 – 3.61 (m, 1H, H-2), 3.60 – 3.58 (m, 3H, H-5, 2x H-6), 3.58 – 3.53 (m, 1H, H-3), 3.53 (s, 3H, CH_3 Me) 3.51 (s, 3H, CH_3 Me), 3.49 (s, 3H, CH_3 Me), 3.40 (s, 3H, CH_3 Me); ^{13}C NMR (101 MHz, $CDCl_3$): δ 97.2 (C-1), 82.6 (d, *J* = 169.6 Hz, CH_2*C*H_2F), 81.2 (C-3), 77.1 (C-2), 76.5 (C-4), 71.8 (C-6), 71.5 (C-5), 66.7 (d, *J* = 19.7 Hz, *C*H_2CH_2F), 60.8, 59.4, 59.1, 57.9 (CH_3 Me); ^{13}C-HMBC-GATED NMR (101 MHz, $CDCl_3$): δ 97.2 (J_{H1-C1} = 169 Hz, α); Diagnostic signals of the minor stereoisomer (β product): ^1H NMR (400 MHz, $CDCl_3$ HH-COSY, HSQC): δ 4.46 (d, *J* = 0.8 Hz, 1H, H-1), 4.10 (dddd, *J* = 36.7, 12.2, 3.7, 2.3 Hz, 1H, C*H*HCH_2F), 3.65 (s, 3H, CH_3 Me), 3.52 (s, 3H, CH_3 Me), 3.50 (s, 2H, CH_3 Me), 3.39 (s, 3H, CH_3 Me), 3.20 (dd, *J* = 8.9, 3.2 Hz, 1H, H-3); ^{13}C NMR (101 MHz, $CDCl_3$) δ 101.6 (C-1), 83.0 (d, *J* = 169.1 Hz, CH_2*C*H_2F), 82.2 (C-3), 68.7 (d, *J* = 19.6 Hz, *C*H_2CH_2F) 61.9, 60.9, 59.4, 57.5 (4x CH_3 Me); ^{13}C-HMBC-GATED NMR (101 MHz, $CDCl_3$): δ 101.6 (J_{H1-C1} = 155 Hz, β); HRMS: $[M+NH_4]^+$ calcd for $C_{13}H_{27}NO_7F$ 300.18169, found 300.18153.

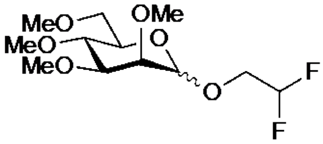

2,2-Difluoroethyl 2,3,4,6-tetra-*O*-methyl-α/β-D-mannopyranoside (S99). The title compound was prepared according to general procedure VII. Column chromatography (85:15 → 75:25, pentane:EtOAc) yielded the title

compound (15.5 mg, 52 μmol, 52%, colourless oil, α:β; 76:24). TLC: R_f 0.35, (pentane:EtOAc, 60:40, v:v); IR (thin film, cm^{-1}): 730, 909, 1072, 1111; Data of the major stereoisomer (α product): ^1H NMR (400 MHz, $CDCl_3$, HH-COSY, HSQC): δ 5.92 (tdd, *J* = 55.3, 4.8, 3.4 Hz, 1H, CH_2CHF_2), 4.95 (d, *J* = 1.9 Hz, 1H, H-1), 3.90 – 3.64 (m, 2H, CH_2CHF_2), 3.63 – 3.57 (m, 5H, H-2, H-3, H-5, 2x H-6), 3.52 (s, 3H, CH_3 Me), 3.51 (s, 3H, CH_3 Me), 3.50 – 3.47 (m, 4H, H-4, CH_3 Me), 3.40 (s, 3H, CH_3 Me); ^{13}C NMR (101 MHz, $CDCl_3$, HSQC): δ 117.5 – 110.0 (m, CH_2CHF_2), 97.9 (C-1), 81.0 (C-4), 76.8, 76.3 (C-2/C-3), 71.9 (C-5), 71.7 (C-6), 66.8 (t, J = 28.0 Hz, CH_2CHF_2), 60.8, 59.4, 59.2, 58.0 (CH_3 Me): ^{13}C-HMBC-GATED NMR (101 MHz, $CDCl_3$): δ 97.9 ($J_{H1\text{-}C1}$ = 170 Hz, α); Diagnostic signals of the minor stereoisomer (β product): ^1H NMR (400 MHz, $CDCl_3$, HH-COSY, HSQC): δ 6.10 – 5.76 (m, 1H, CH_2CHF_2), 4.46 (d, *J* = 0.8 Hz, 1H, H-1), 4.06 (dddd, *J* = 22.5, 11.7, 9.9, 2.7 Hz, 1H, $CHHCHF_2$), 3.50 (s, 3H, CH_3 Me), 3.19 (dd, *J* = 9.1, 3.1 Hz, 1H, H-3); ^{13}C NMR (101 MHz, $CDCl_3$) δ 101.8 (C-1), 83.9 (C-3), 68.8 – 68.0 (m, CH_2CHF_2), 61.9, 60.9, 59.4, 57.7 (4x CH_3 Me); ^{13}C-HMBC-GATED NMR (101 MHz, $CDCl_3$): δ 101.8 ($J_{H1\text{-}C1}$ = 156 Hz, β); HRMS: $[M+Na]^+$ calcd for $C_{13}H_{22}NaO_7F_2$ 323.12767, found 323.12723.

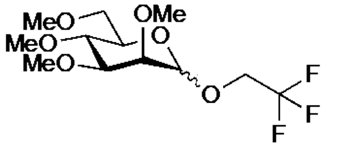

2,2,2-Trifluoroethyl 2,3,4,6-tetra-*O*-methyl-α/β-D-mannopyranoside (S100). The title compound was prepared according to general procedure VII. Column chromatography (85:15 pentane:EtOAc) yielded the title compound (18 mg, 57 μmol, 57%, colourless oil, α:β 93:7). TLC: R_f 0.40, (pentane:EtOAc, 60:40, v:v); IR (thin film, cm^{-1}): 732, 985, 1081, 1164, 1280; Data of the major stereoisomer (α product): ^1H NMR (400 MHz, $CDCl_3$, HH-COSY, HSQC): δ 4.99 (d, *J* = 1.8 Hz, 1H, H-1), 4.04 – 3.94 (m, 1H, $CHHCF_3$), 3.94 – 3.84 (m, 1H, $CHHCF_3$), 3.66 – 3.62 (m, 1H, H-2), 3.63 – 3.53 (m, 3H, H-5, H-6, H-6), 3.53 (s, 3H, CH_3 Me), 3.52 (s, 3H, CH_3 Me), 3.51 – 3.45 (m, 5H, H-2, H-4, CH_3 Me), 3.40 (s, 3H, CH_3 Me); ^{13}C NMR (101 MHz, $CDCl_3$, HSQC): δ 123.9 (q, *J* = 278.2 Hz, CH_2CF_3), 97.7 (C-1), 80.9 (C-4), 76.8 (C-2), 76.2 (C-3), 72.2 (C-5), 71.6 (C-6), 64.2 (q, *J* = 34.8 Hz, CH_2CF_3), 60.8, 59.4, 59.3, 58.1 (CH_3 Me); HMBC-GATED NMR (101 MHz, $CDCl_3$): δ 97.7 ($J_{H1\text{-}C1}$ = 170 Hz, α); Diagnostic signals of the minor stereoisomer (β product): ^1H NMR (500 MHz, $CDCl_3$, HH-COSY, HSQC): δ 4.52 (s, 1H, H-1), 4.21 (dq, *J* = 12.6, 8.9 Hz, 1H, $CHHCF_3$), 3.76 (dd, *J* = 3.1, 0.8 Hz, 1H, H-2), 3.19 (dd, *J* = 9.1, 3.2 Hz, 1H, H-3) ^{13}C NMR (101 MHz, $CDCl_3$, HSQC): δ 101.4 (C-1), 61.9, 59.4, 57.7 (3x CH_3 Me); HRMS: $[M+Na]^+$ calcd for $C_{13}H_{21}NaO_7F_3$ 341.11824, found 341.11811.

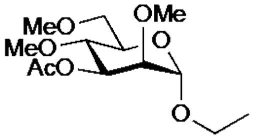

Ethyl 3-*O*-acetyl-2,4,6-tri-*O*-methyl-α-D-mannopyranoside (S101). The title compound was prepared according to general procedure VII. Column chromatography (85:15 → 75:25, pentane:EtOAc) yielded the title compound (19.1 mg, 65 μmol, 65%, colourless oil, α:β >98:2). TLC: R_f 0.29, (pentane:EtOAc, 60:40, v:v); $[\alpha]_D^{25}$ 69.0° (*c* 0.10 $CHCl_3$) IR (thin film, cm^{-1}): 730, 909,1067, 1101, 1238, 1733; ^1H NMR (400 MHz, $CDCl_3$, HH-COSY, HSQC): δ 5.17 (dd, *J* = 9.5, 3.4 Hz, 1H, H-3), 4.87 (d, *J* = 1.9 Hz, 1H, H-1), 3.75 (dt, *J* = 9.8, 7.1 Hz, 1H, *C*HH Et), 3.71 – 3.66 (m, 1H, H-6), 3.64 – 3.56 (m, 4H, H-2, H-4, H-5 ,CH*H* Et), 3.52 – 3.47 (m, 1H, H-6), 3.46 (s, 3H, CH_3 Me), 3.44 (s, 3H, CH_3 Me), 3.41 (s, 3H, CH_3 Me), 2.15 (s, 3H, CH_3 Ac), 1.20 (t, *J* = 7.1 Hz, 3H, CH_3 Et); ^{13}C NMR (101 MHz, $CDCl_3$, HSQC): δ 170.5 (C=O), 97.0 (C-1), 78.7 (C-2), 74.9 (C-4), 74.0 (C-3), 71.5 (CH_2 Et), 71.1 (C-5), 63.3 (C-6), 60.5, 59.4, 59.3

(CH$_3$ Me), 21.4 (CH$_3$ Ac), 15.1 (CH$_3$ Et); HMBC-GATED NMR (101 MHz, CDCl$_3$): δ 97.0 (J_{H1-C1} = 169 Hz, α); HRMS: [M+Na]$^+$ calcd for $C_{13}H_{24}NaO_7$ 315.14142, found 315.14126.

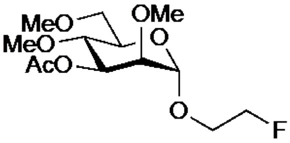

2-Fluoroethyl 3-*O*-acetyl-2,4,6-tri-*O*-met hyl-α-D-mannopyranoside (S102). The title compound was prepared according to general procedure VII. Column chromatography (80:20 → 60:40, pentane:EtOAc) yielded the title compound (24.7 mg, 80 µmol, 80%, colourless oil, α:β >98:2). TLC: R$_f$ 0.23, (pentane:EtOAc, 60:40, v:v); $[\alpha]_D^{25}$ 52.4° (*c* 0.29 CHCl$_3$); IR (thin film, cm^{-1}): 1047, 1101, 1123, 1238, 1369, 1739; ^{1}H NMR (400 MHz, CDCl$_3$, HH-COSY, HSQC): δ 5.17 (dd, *J* = 9.5, 3.4 Hz, 1H, H-3), 4.92 (d, *J* = 1.9 Hz, 1H, H-1), 4.65 – 4.60 (m, 1H, CH$_2$C*H*HF), 4.54 – 4.48 (m, 1H, CH$_2$CH*H*F), 3.97 – 3.82 (m, 1H, C*H*HCH$_2$F), 3.81 – 3.69 (m, 2H, H-6, CH*H*CH$_2$F), 3.68 – 3.56 (m, 4H, H-2, H-4, H-5, H-6), 3.46 (s, 3H, CH$_3$ Me), 3.44 (s, 3H, CH$_3$ Me), 3.41 (s, 3H, CH$_3$ Me), 2.15 (s, 3H, CH$_3$ Ac); ^{13}C NMR (101 MHz, CDCl$_3$, HSQC): δ 170.4 (C=O), 97.6 (C-1) 82.6 (d, *J* = 169.9 Hz, CH$_2$*C*H$_2$F), 78.4 (H-2), 74.7 (C-4), 73.7 (C-3), 71.4 (C-6), 71.3 (C-5), 66.8 (d, *J* = 20.0 Hz, *C*H$_2$CH$_2$F), 60.5, 59.4, 59.4 (CH$_3$ Me), 21.4 (CH$_3$ Ac); HMBC-GATED NMR (101 MHz, CDCl$_3$): δ 97.6 (J_{H1-C1} = 170 Hz, α); HRMS: [M+Na]$^+$ calcd for $C_{13}H_{23}NaO_7F$ 333.13200, found 333.13176.

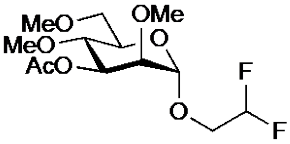

2,2-Difluoroethyl 3-*O*-acetyl-2,4,6-tri-*O*-methyl-α-D-mannopyranoside (S103). The title compound was prepared according to general procedure VII. Column chromatography (90:10 → 75:25, pentane:EtOAc) yielded the title compound (18.6 mg, 57 µmol, 57%, colourless oil, α:β >98:2). TLC: R$_f$ 0.35, (pentane:EtOAc, 60:40, v:v); $[\alpha]_D^{25}$ 59.3° (*c* 0.14 CHCl$_3$); IR (thin film, cm^{-1}): 731, 909, 1070, 1100, 1240, 1739; ^{1}H NMR (400 MHz, CDCl$_3$, HH-COSY, HSQC): δ 5.93 (tdd, *J* = 55.4, 4.8, 3.6 Hz, 1H, CH$_2$C*H*F$_2$), 5.12 (dd, *J* = 8.9, 3.3 Hz, 1H, H-3), 4.91 (d, *J* = 2.0 Hz, 1H, H-1), 3.90 – 3.78 (m, 1H, C*H*HCHF$_2$), 3.78 – 3.70 (m, 1H, CH*H*CHF$_2$), 3.69 – 3.57 (m, 5H, H-2, H-4, H-5, H-6, H-6), 3.46 (s, 3H, CH$_3$ Me), 3.44 (s, 3H, CH$_3$ Me), 3.41 (s, 3H, CH$_3$ Me), 2.15 (s, 3H, CH$_3$ Ac) ^{13}C NMR (101 MHz, CDCl$_3$, HSQC): δ 170.4 (C=O), 114.1 (t, *J* = 241.1 Hz, CH$_2$*C*HF$_2$), 98.2 (C-1), 78.1 (C-2), 74.6 (C-4), 73.5 (C-3), 71.7 (C-5), 71.3 (C-6), 66.9 (t, *J* = 28.6 Hz, *C*H$_2$CHF$_2$), 60.5, 59.5, 59.4 (CH$_3$ Me), 21.3 (CH$_3$ Ac); HMBC-GATED NMR (101 MHz, CDCl$_3$): δ 98.2 (J_{H1-C1} = 170 Hz, α); HRMS: [M+Na]$^+$ calcd for $C_{13}H_{22}NaO_7F_2$ 351.12258, found 351.12233.

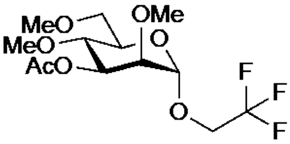

2,2,2-Trifluoroethyl 3-*O*-acetyl-2,4,6-tri-*O*-methyl-α-D-mannopyranoside (S104). The title compound was prepared according to general procedure VII. Column chromatography (85:15, pentane:EtOAc) yielded the title compound (15.5 mg, 45 µmol, 45%, colourless oil, α:β >98:2). TLC: R$_f$ 0.39, (pentane:EtOAc, 60:40, v:v); $[\alpha]_D^{25}$ 59.0° (*c* 0.5 CHCl$_3$) IR (thin film, cm^{-1}): 730, 910, 1083, 1103, 1238, 1745, 2933; ^{1}H NMR (400 MHz, CDCl$_3$, HH-COSY, HSCQ): δ 5.17 – 5.11 (m, 1H, H-3), 4.95 (d, *J* = 2.1 Hz, 1H, H-1), 3.95 (qq, *J* = 12.4, 8.6 Hz, 2H, C*H$_2$*CF$_3$), 3.69 (dd, *J* = 3.4, 2.1 Hz, 1H, H-2), 3.67 – 3.63 (m, 2H, H-4, H-5), 3.63 – 3.56 (m, 2H, H-6, H-6), 3.47 (s, 3H, CH$_3$ Me), 3.45 (s, 3H, CH$_3$ Me), 3.41 (s, 3H, CH$_3$ Me), 2.15 (s, 3H, CH$_3$ Ac); ^{13}C NMR (101 MHz, CDCl$_3$, HSQC): δ 170.4 (C=O),

98.1 (C-1), 77.9 (C-2), 74.5 (C-4), 73.2 (C-3), 71.9 (C-5), 71.2 (C-6), 64.3 (q, J = 35.0 Hz, CH_2CF_3), 60.5, 59.6, 59.4 (CH_3 OMe), 21.3 (OMe); HMBC-GATED NMR (101 MHz, $CDCl_3$): δ 98.1 (J_{H1-C1} = 170 Hz, α); HRMS: $[M+Na]^+$ calcd for $C_{13}H_{21}NaO_7F_3$ 369.11316, found 369.11324.

Supplementary Table 1. Stereoselectivity for glycosylation reactions for Ac-Me-protected donors

Supplementary Table 1. Experimentally found stereoselectivity for glycosylation reactions for Ac-Me-protected donors with model acceptors. The stereoselectivity is expressed as α:β-ratios and were established by ^{1}H-NMR spectroscopy of the crude and purified reaction mixtures.

Entry	Donor	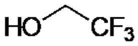	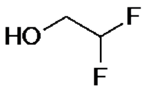	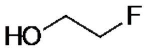	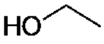
1	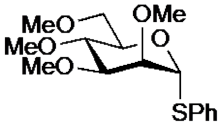	93:7 (57%)	76:24 (52%)	64:36 (74%)	51:49 (69%)
2	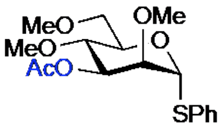	>98:2 (45%)	>98:2 (57%)	>98:2 (80%)	>98:2 (65%)

>90:10	>80:20	>60:40	>50:50	<50:50	<40:60	<20:80	<10:90	(α:β)

NMR spectra of new and selected compounds

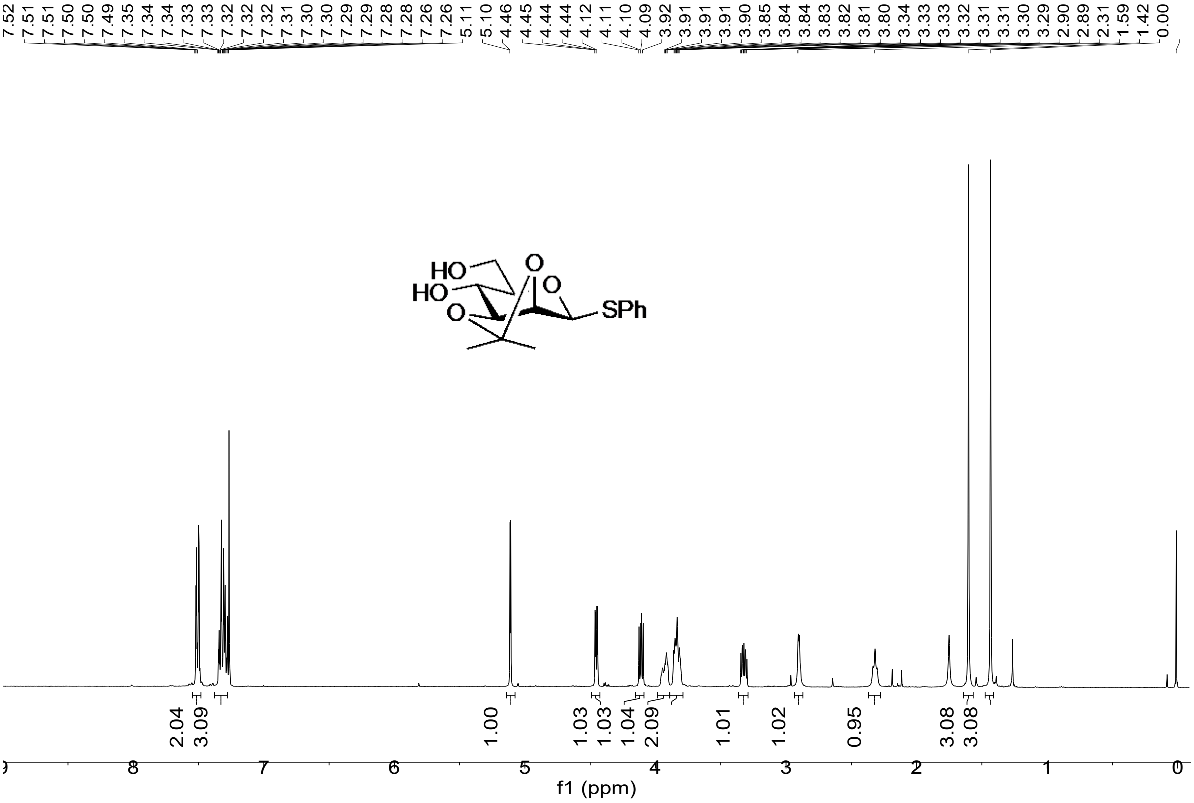

Supplementary Figure 33. ^{1}H NMR, 400 MHz, $CDCl_3$ of compound **S1**

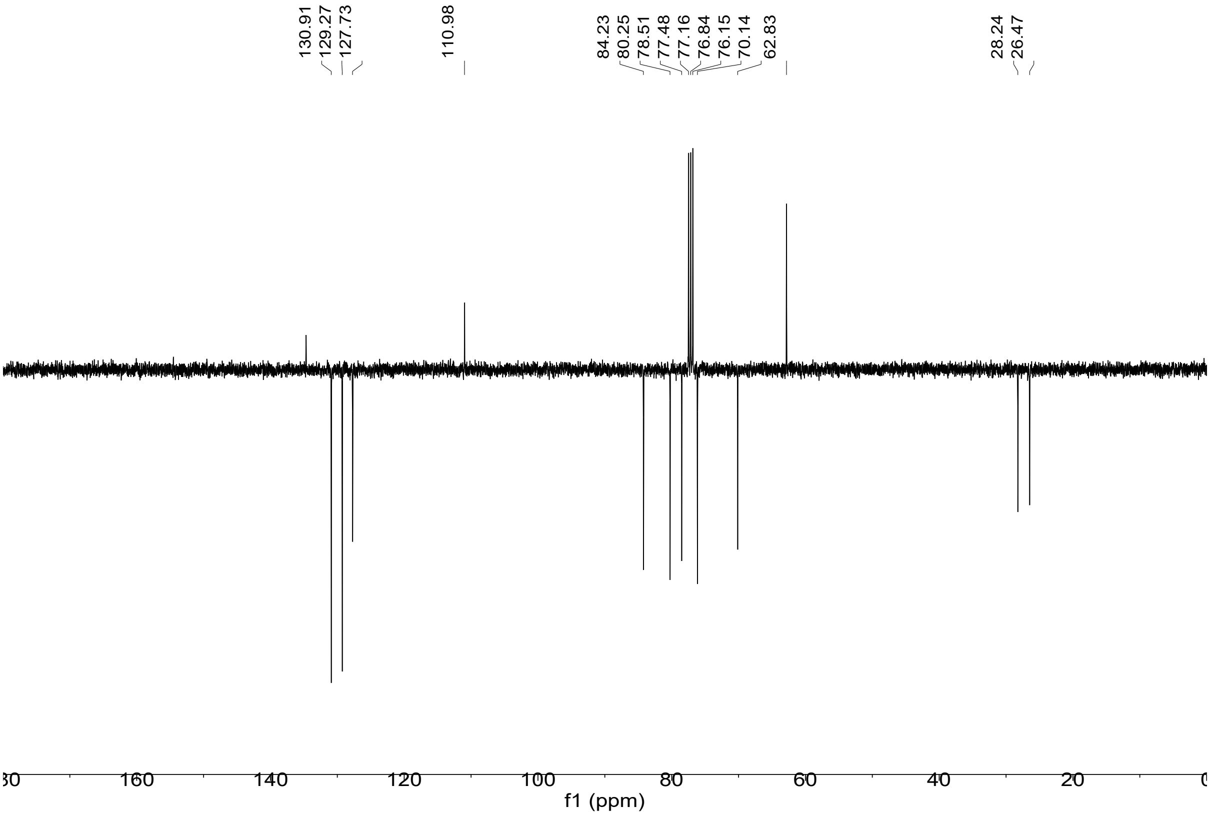

Supplementary Figure 34. ^{13}C NMR, 101 MHz, $CDCl_3$ of compound **S1**

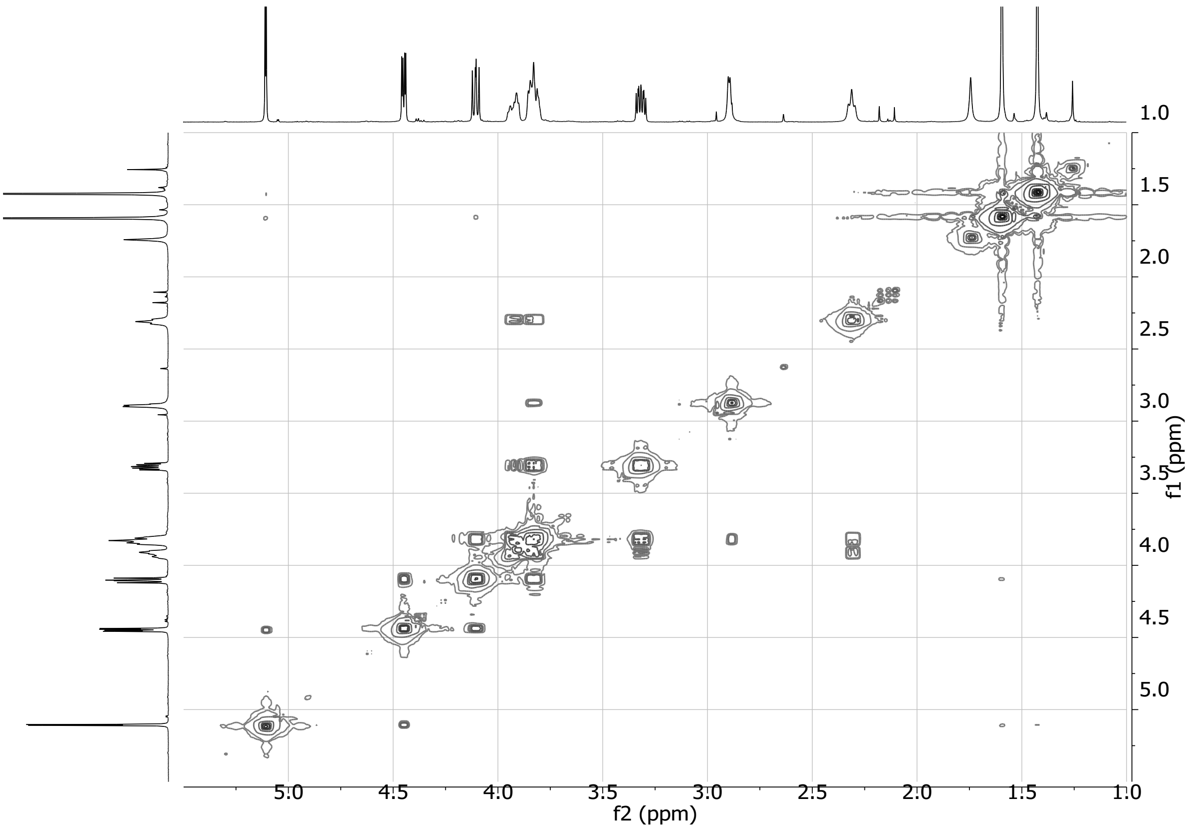

Supplementary Figure 35. HH-COSY NMR, $CDCl_3$ of compound **S1**

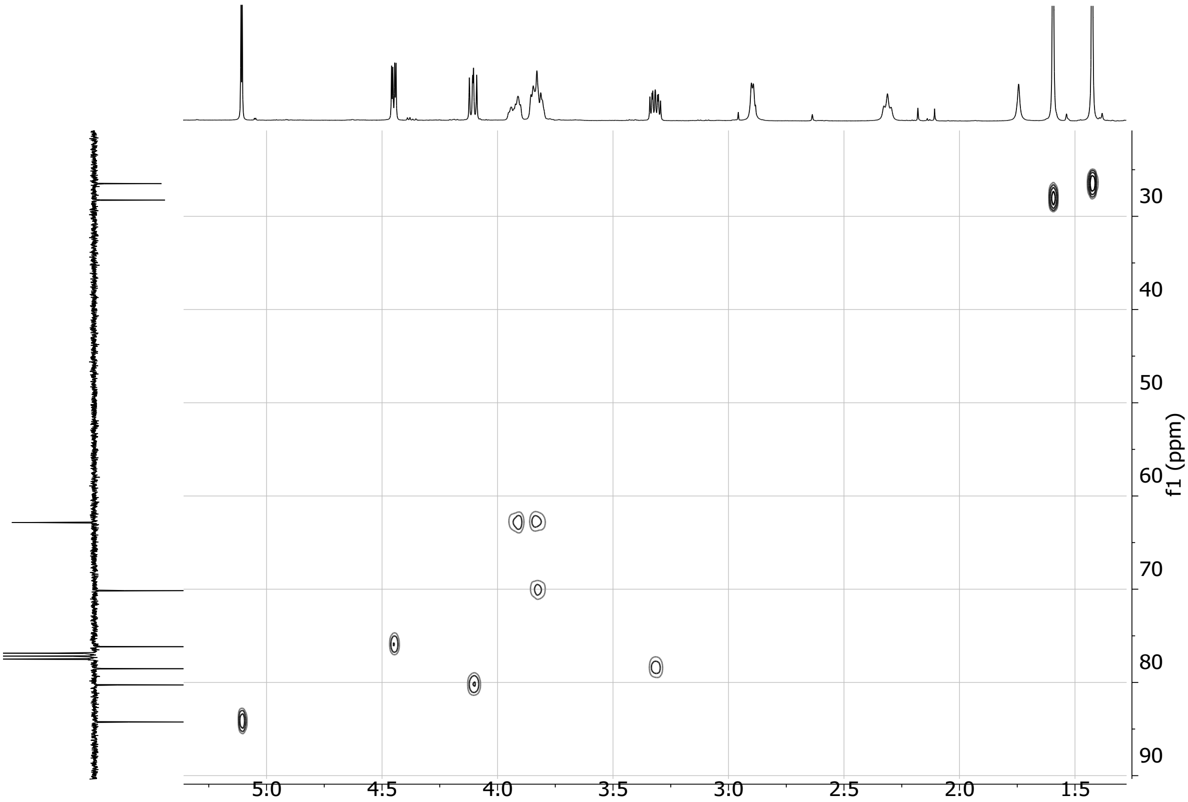

Supplementary Figure 36. HSQC NMR, $CDCl_3$ of compound **S1**

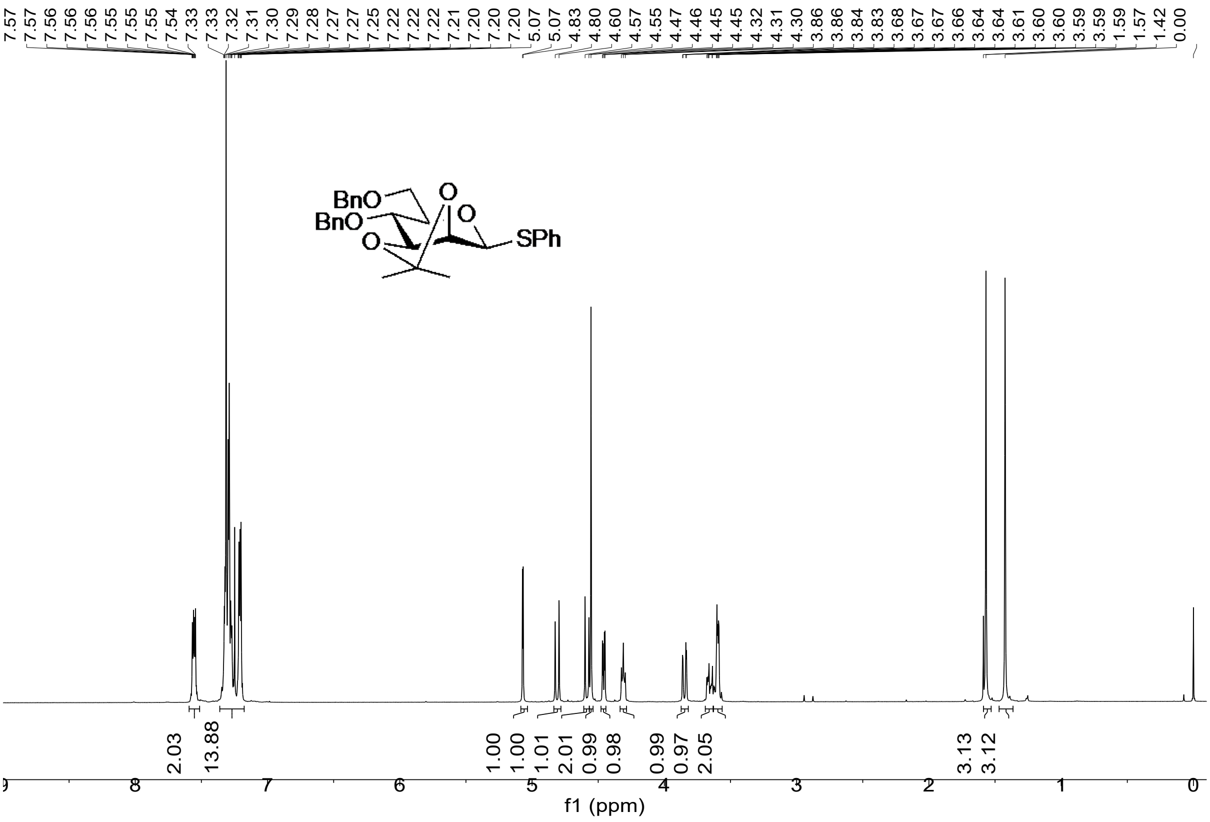

Supplementary Figure 37. ^{1}H NMR, 400 MHz, $CDCl_3$ of compound **S2**

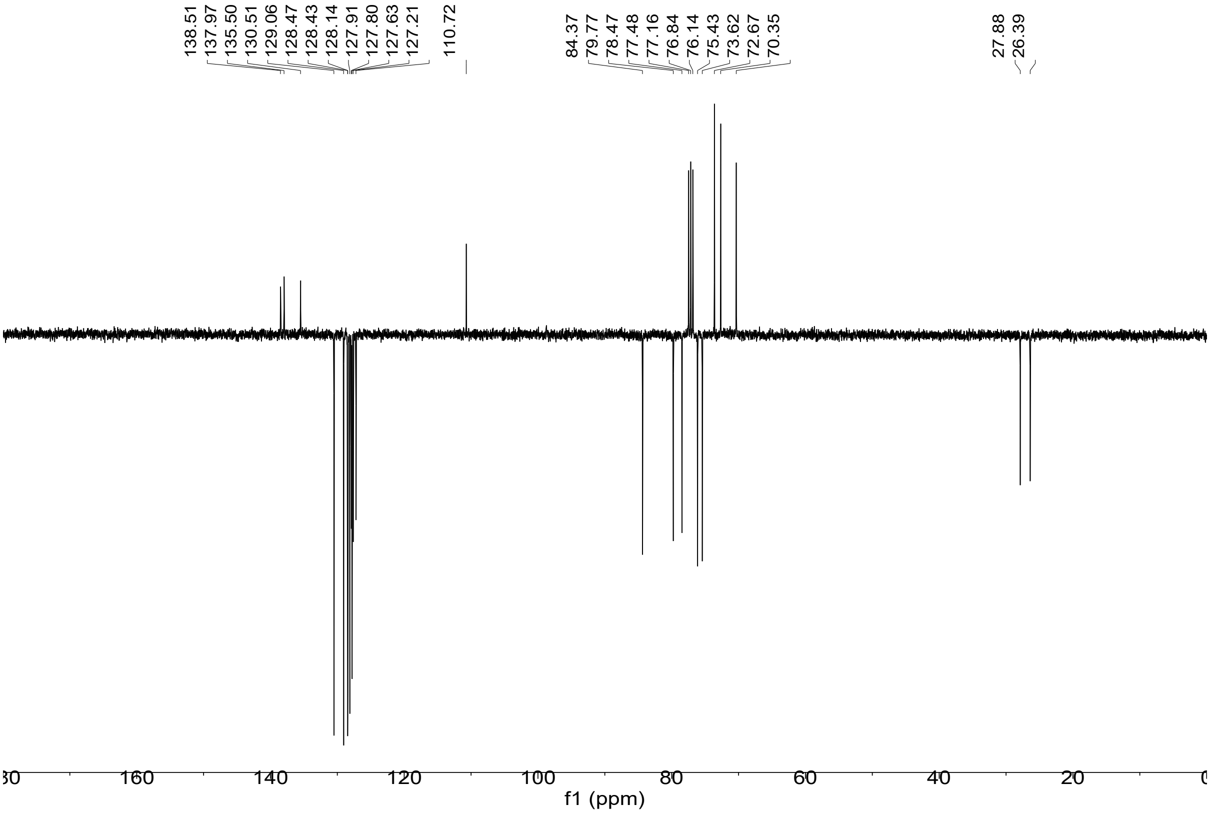

Supplementary Figure 38. ^{13}C NMR, 101 MHz, $CDCl_3$ of compound **S2**

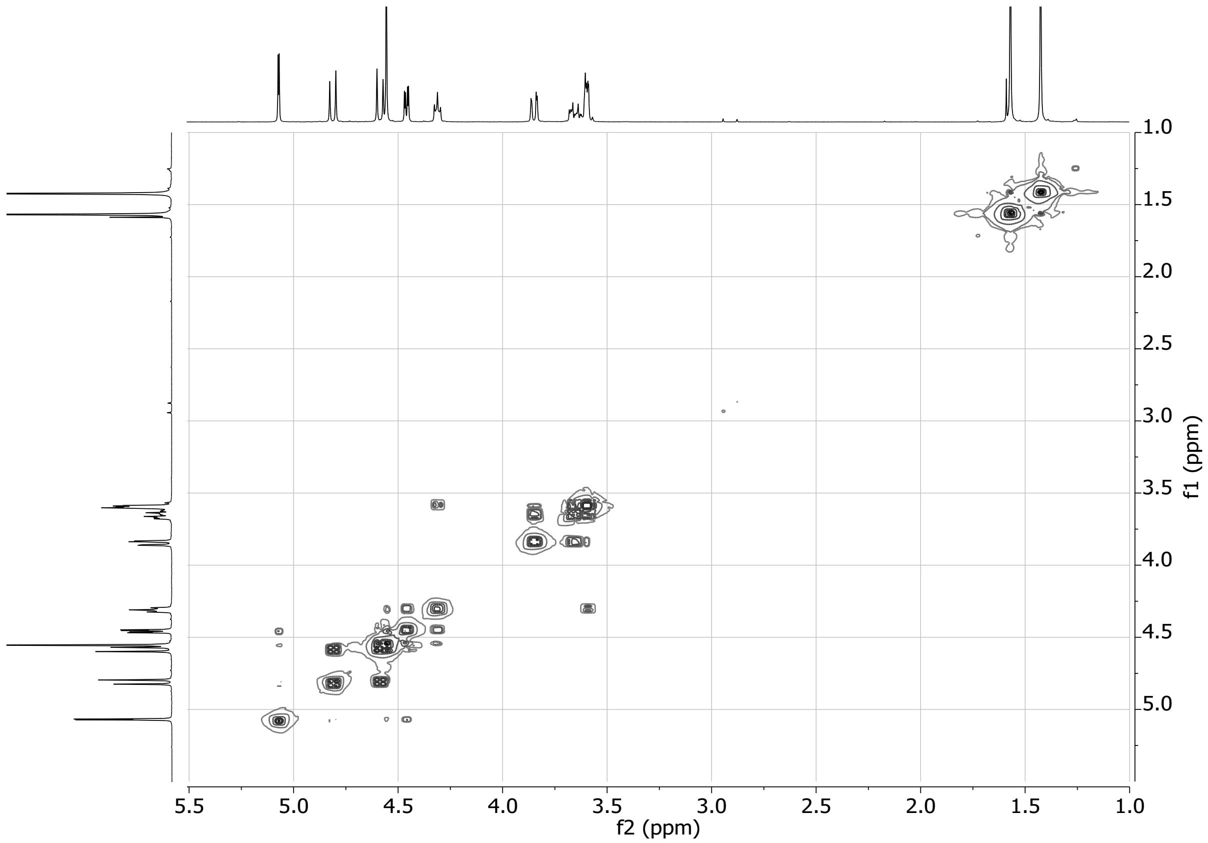

Supplementary Figure 39. HH-COSY NMR, $CDCl_3$ of compound **S2**

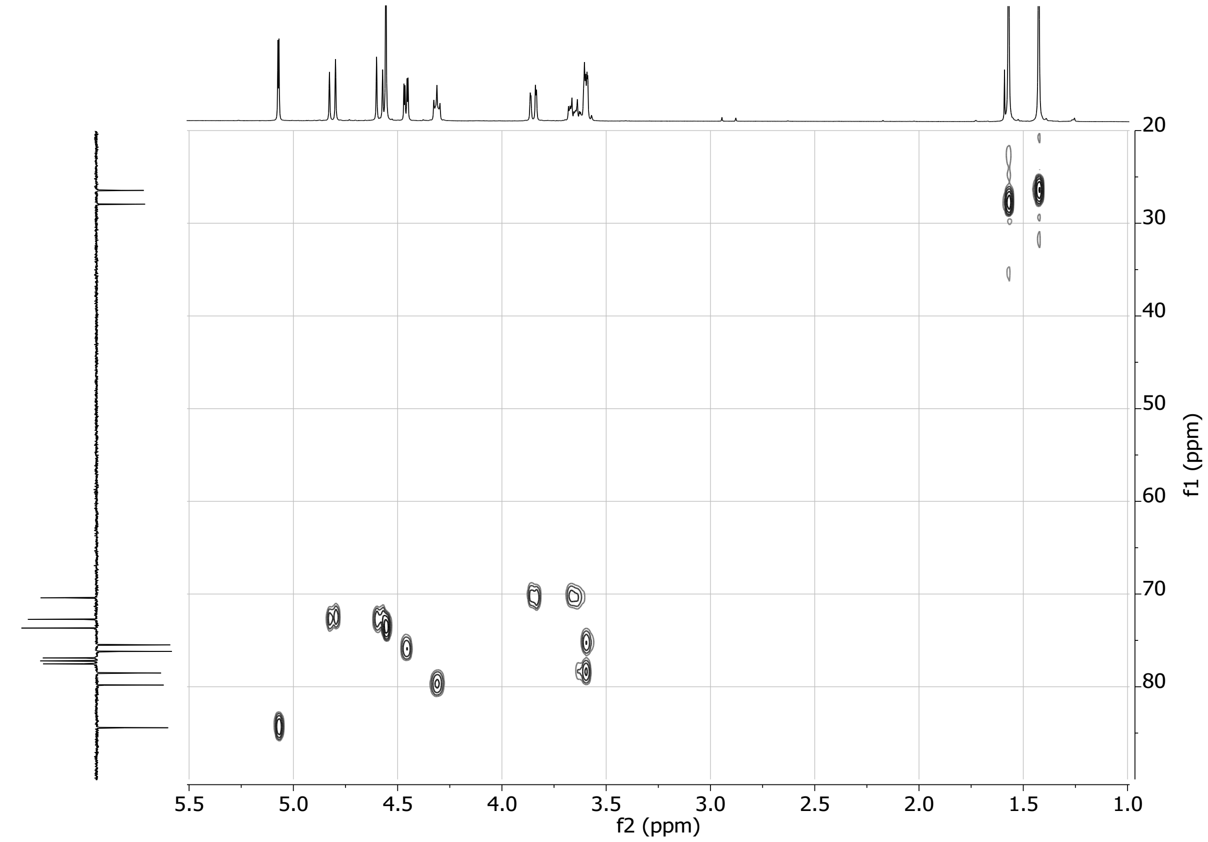

Supplementary Figure 40. HSQC NMR, $CDCl_3$ of compound **S2**

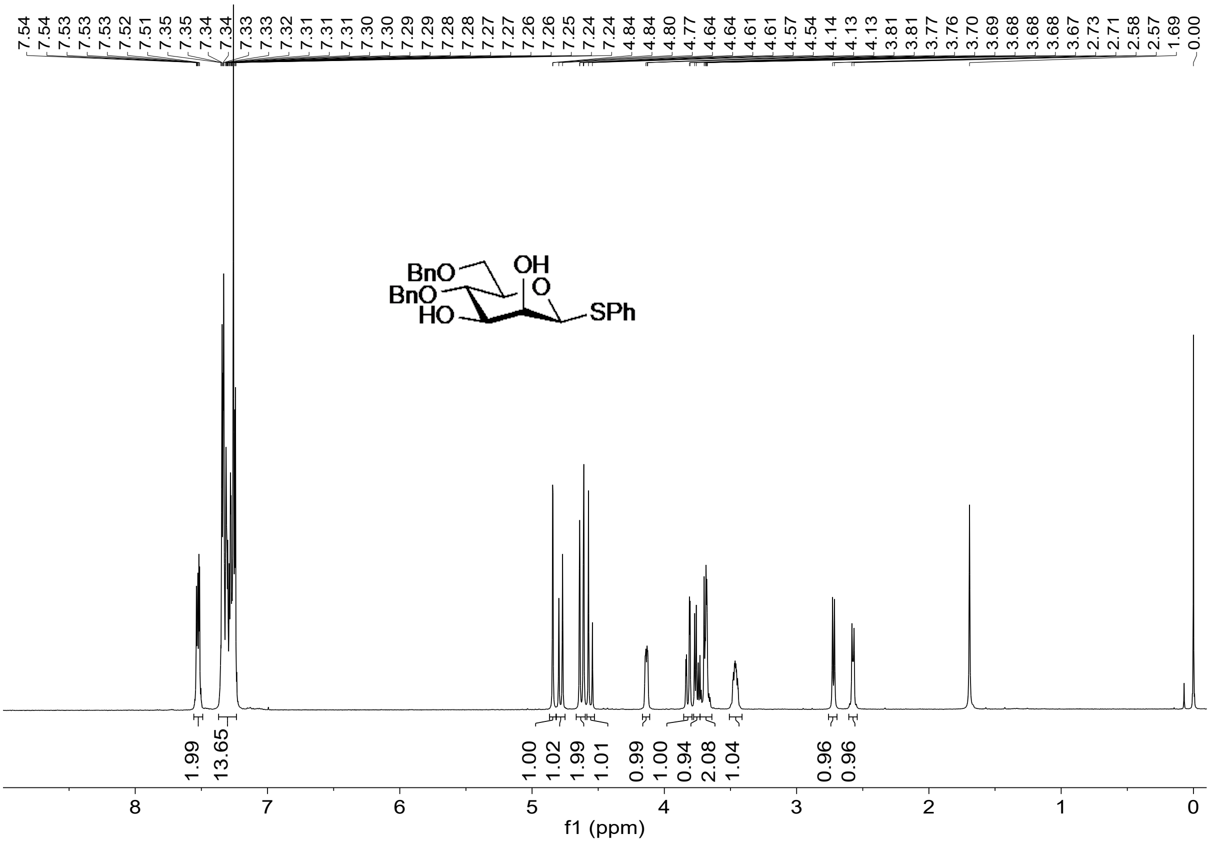

Supplementary Figure 41. ^{1}H NMR, 400 MHz, $CDCl_3$ of compound **S3**

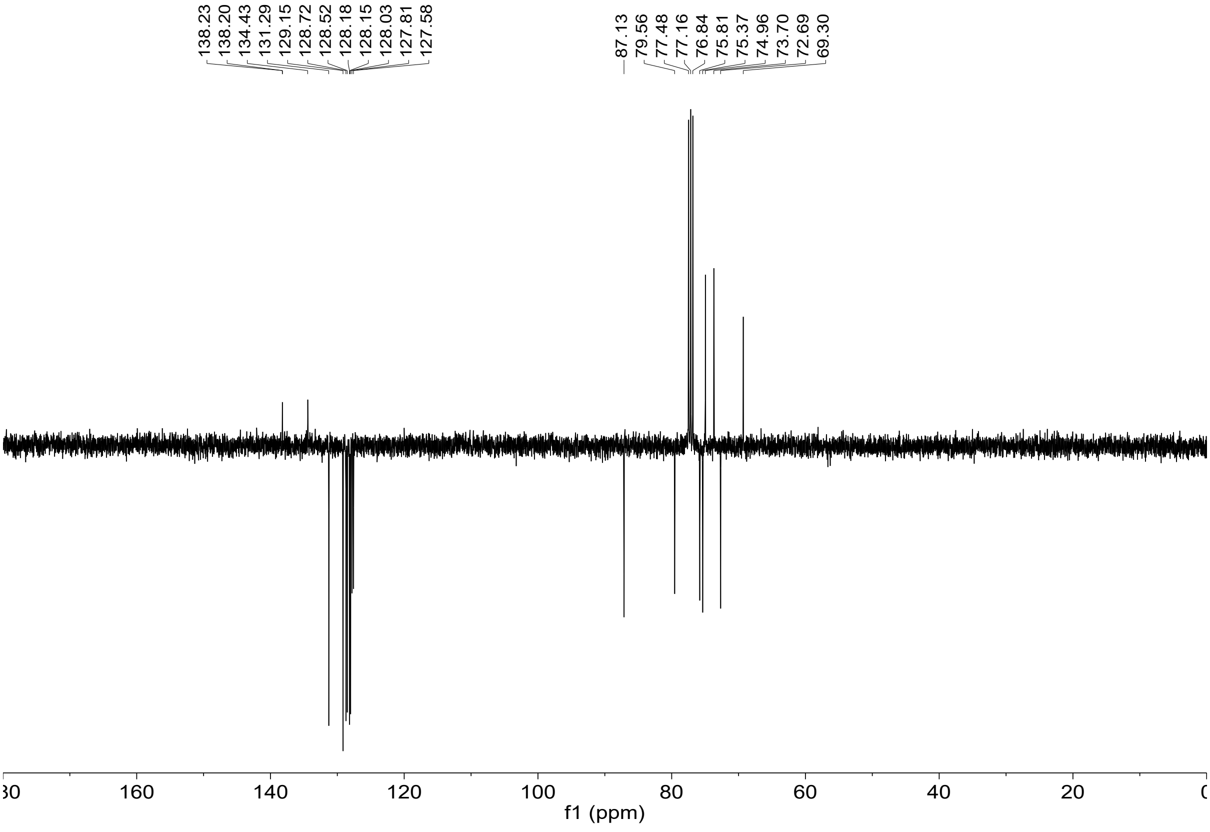

Supplementary Figure 42. ^{13}C NMR, 101 MHz, $CDCl_3$ of compound **S3**

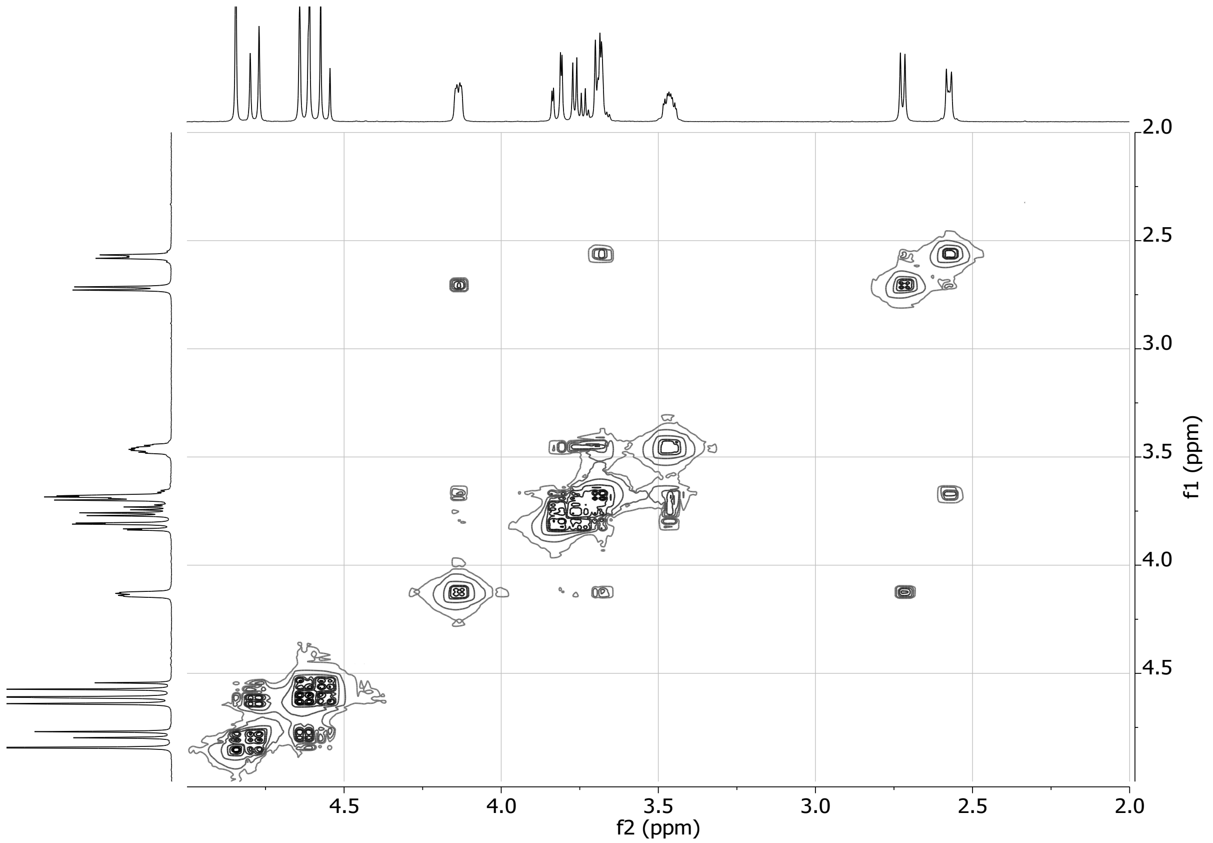

Supplementary Figure 43. HH-COSY NMR, $CDCl_3$ of compound **S3**

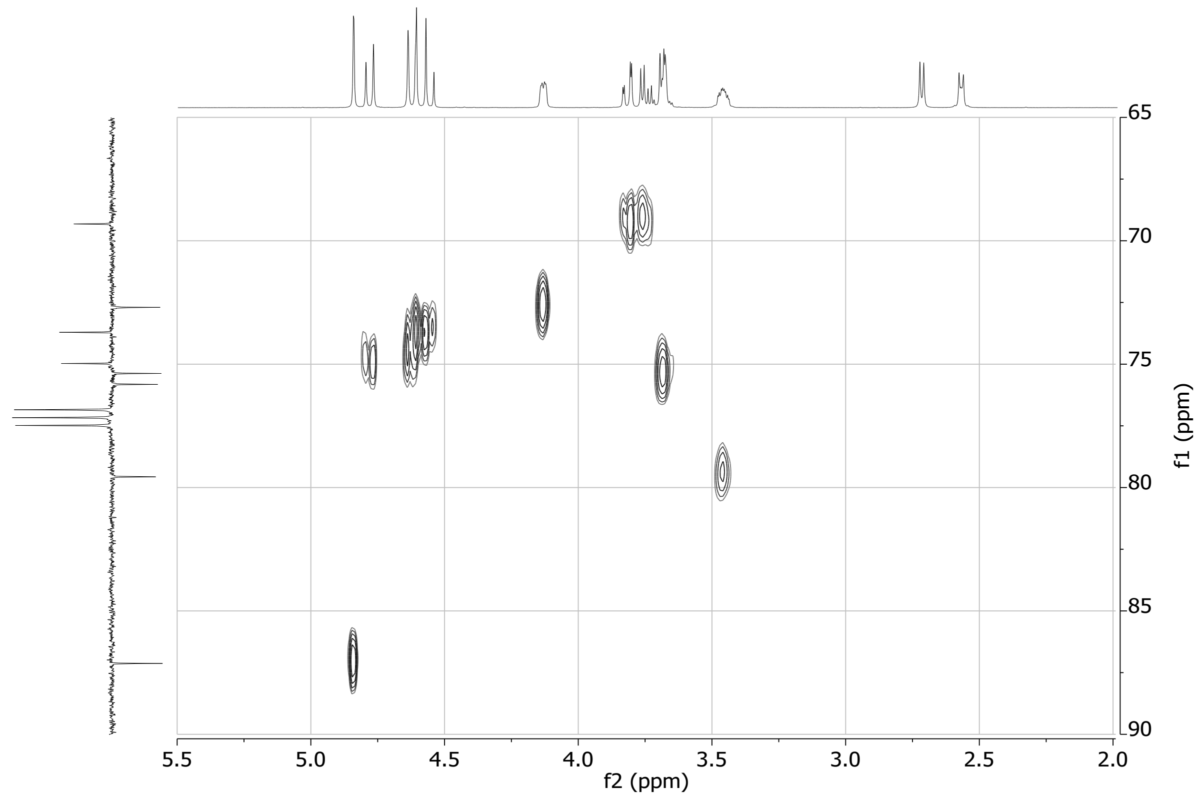

Supplementary Figure 44. HSQC NMR, $CDCl_3$ of compound **S3**

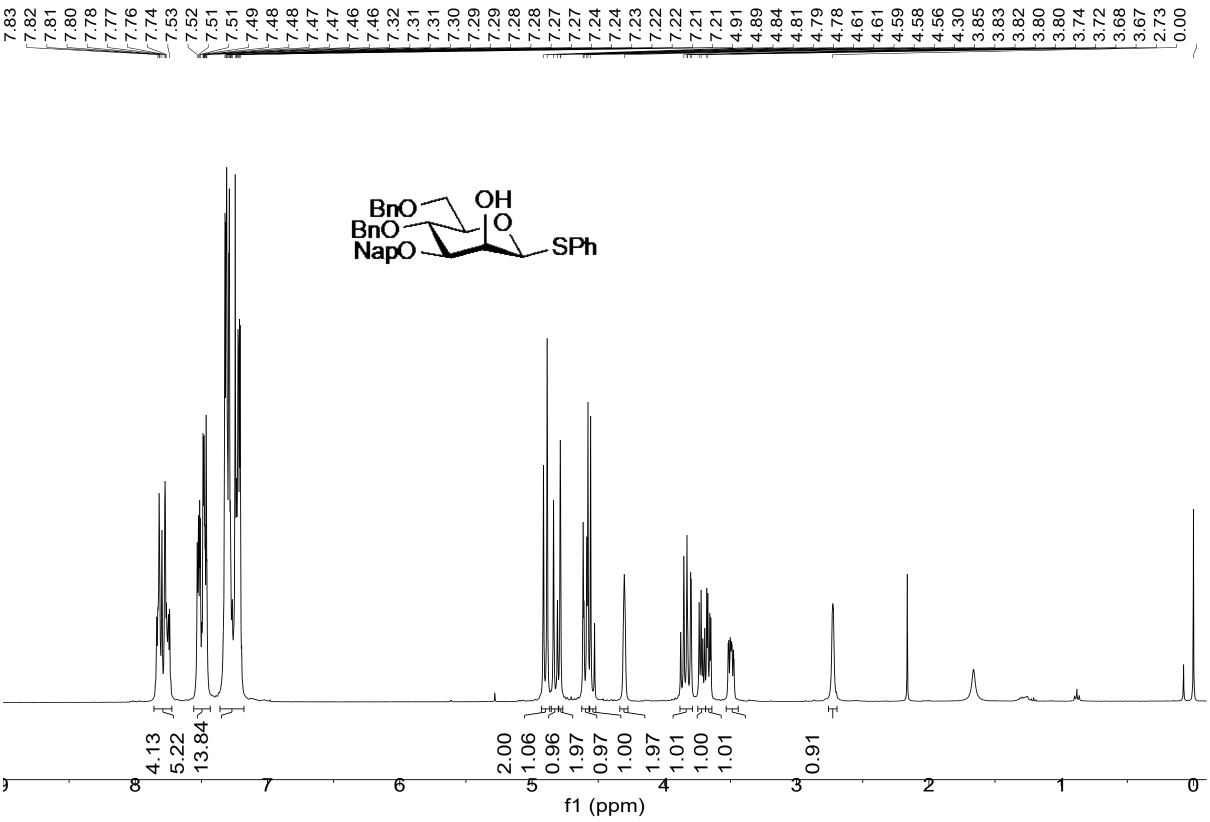

Supplementary Figure 45. ^{1}H NMR, 400 MHz, $CDCl_3$ of compound **S4**

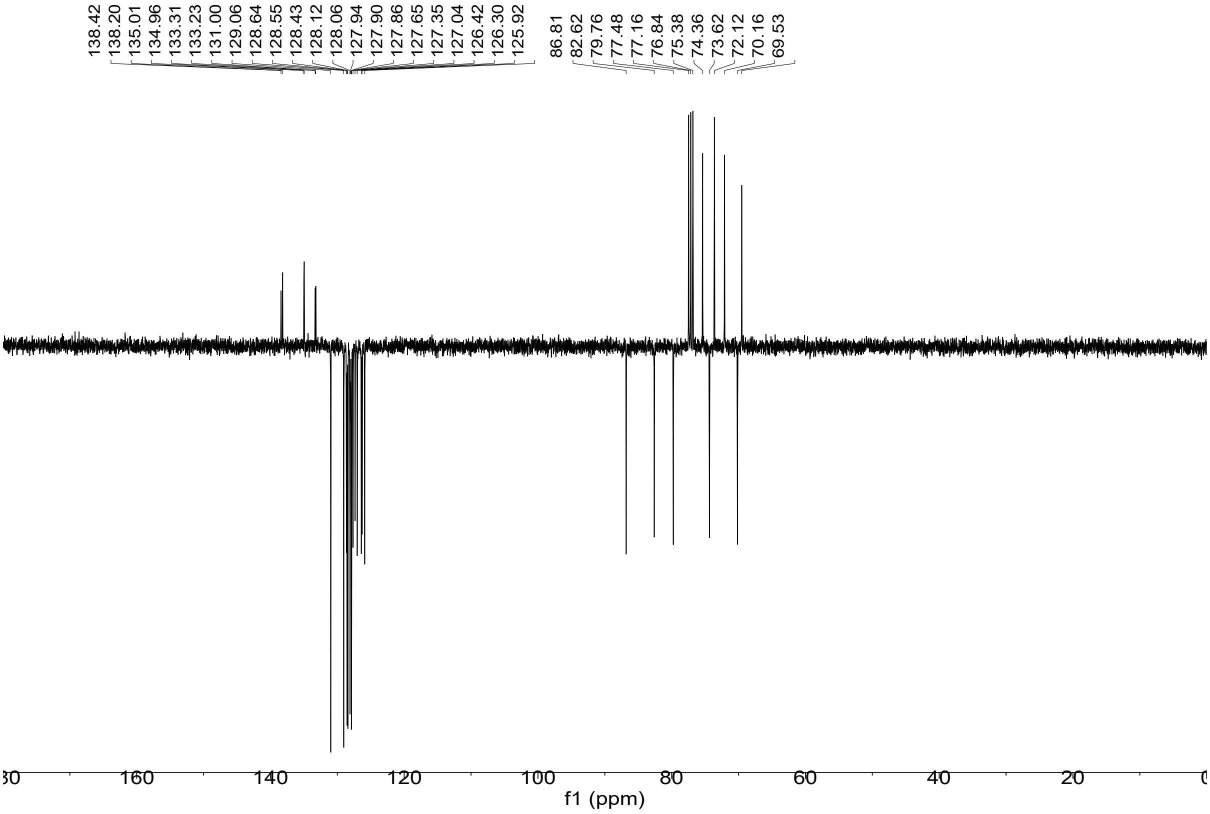

Supplementary Figure 46. ^{13}C NMR, 101 MHz, $CDCl_3$ of compound **S4**

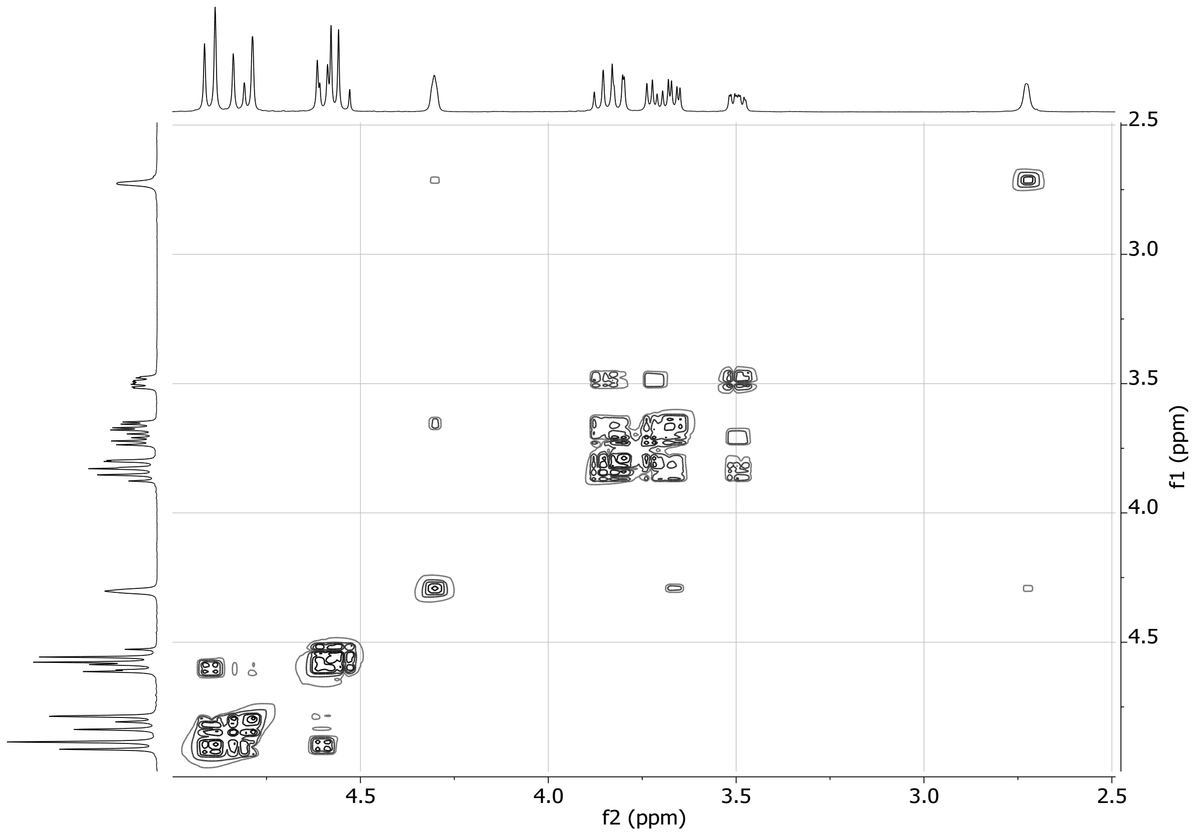

Supplementary Figure 47. HH-COSY NMR, $CDCl_3$ of compound **S4**

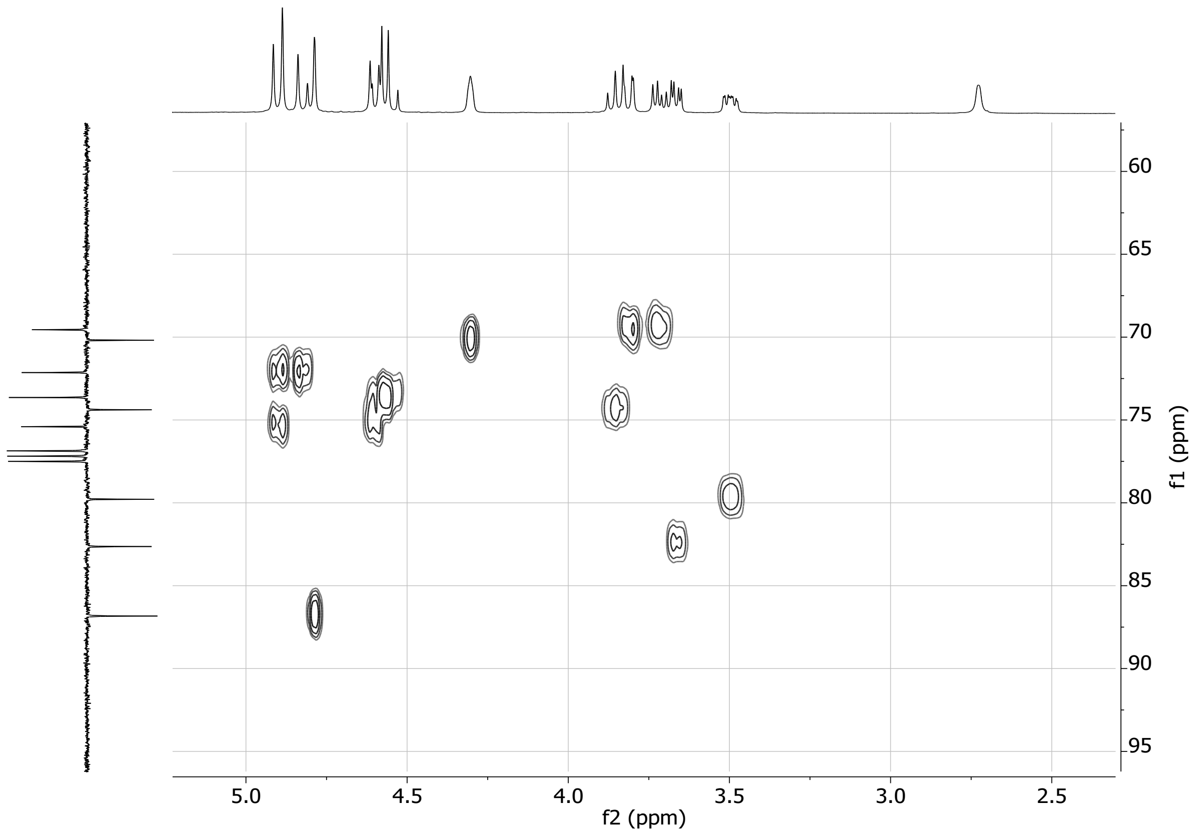

Supplementary Figure 48. HSQC NMR, $CDCl_3$ of compound **S4**

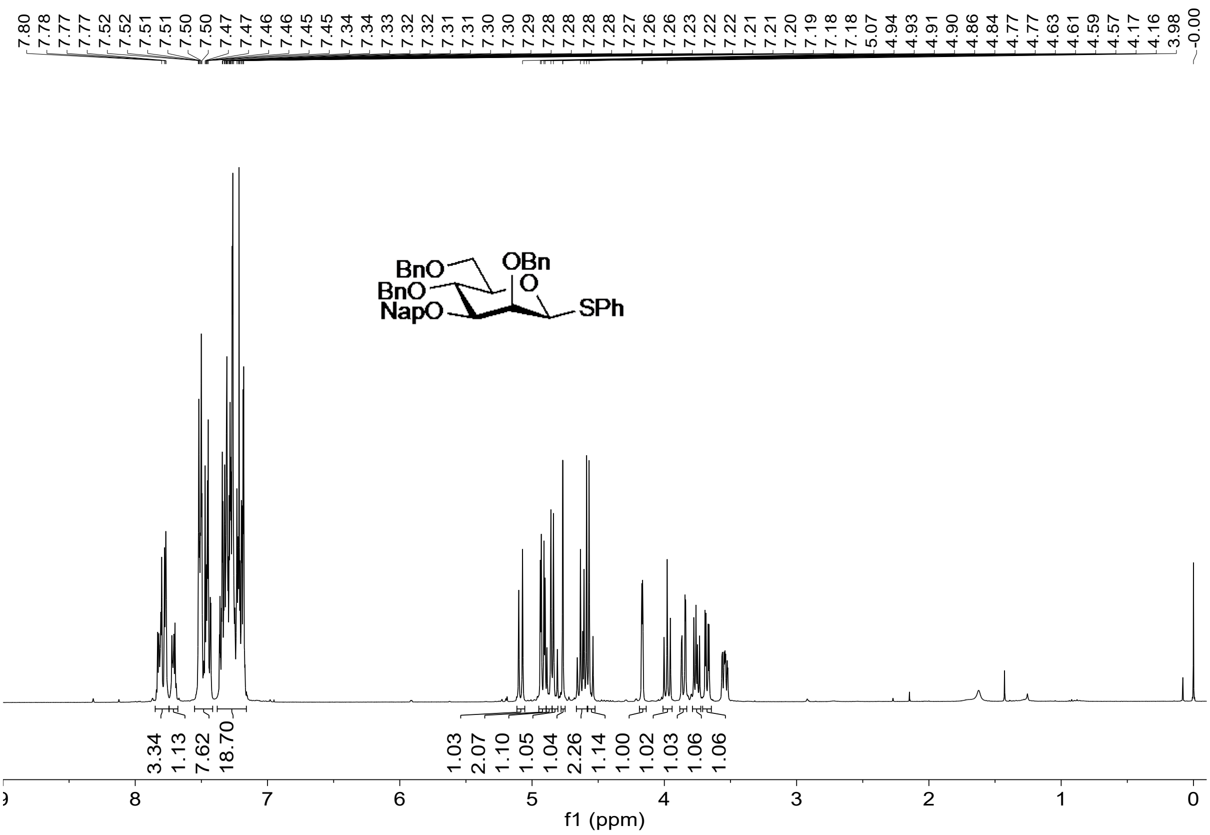

Supplementary Figure 49. ^{1}H NMR, 400 MHz, $CDCl_3$ of compound **S5**

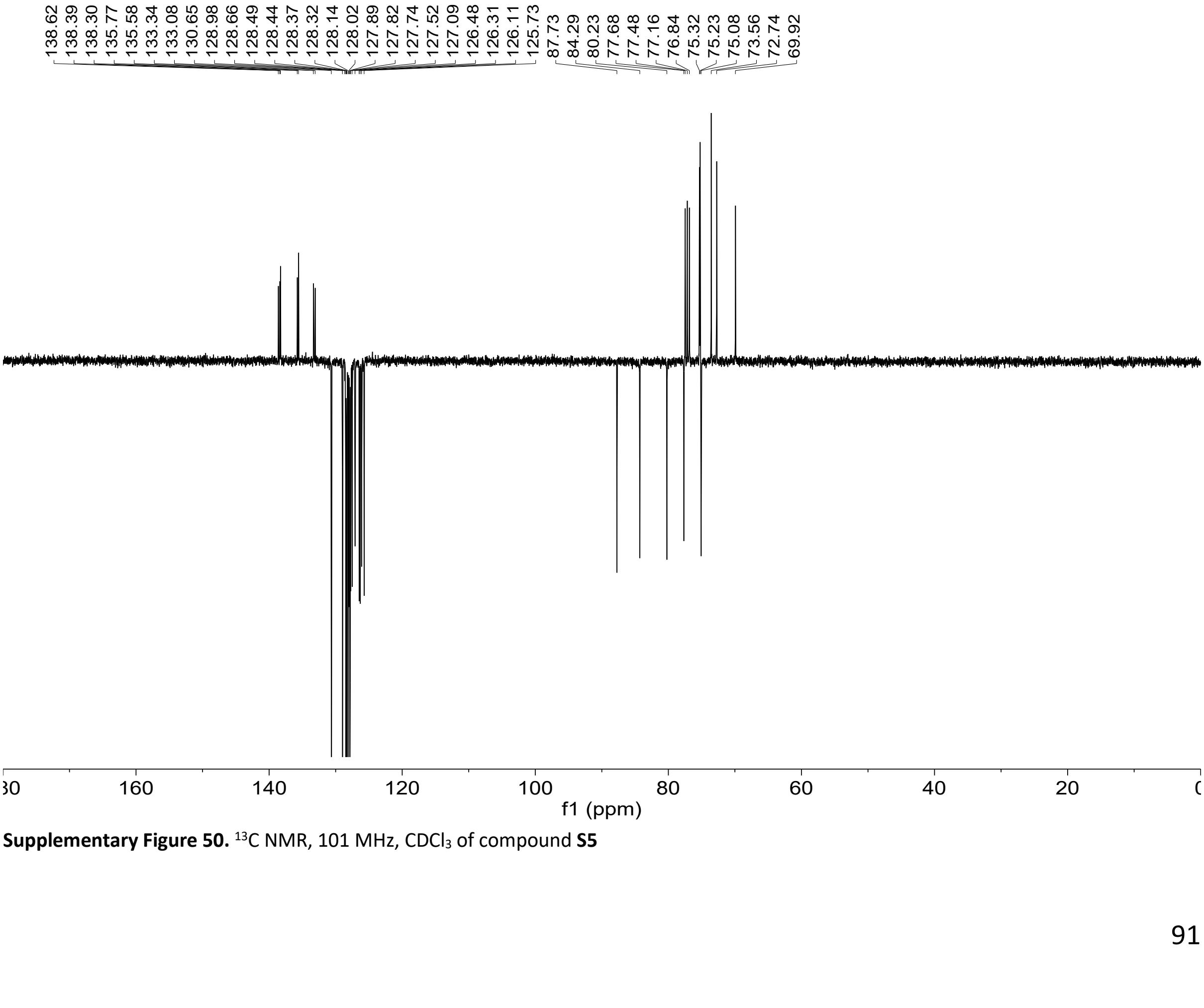

Supplementary Figure 50. ^{13}C NMR, 101 MHz, $CDCl_3$ of compound **S5**

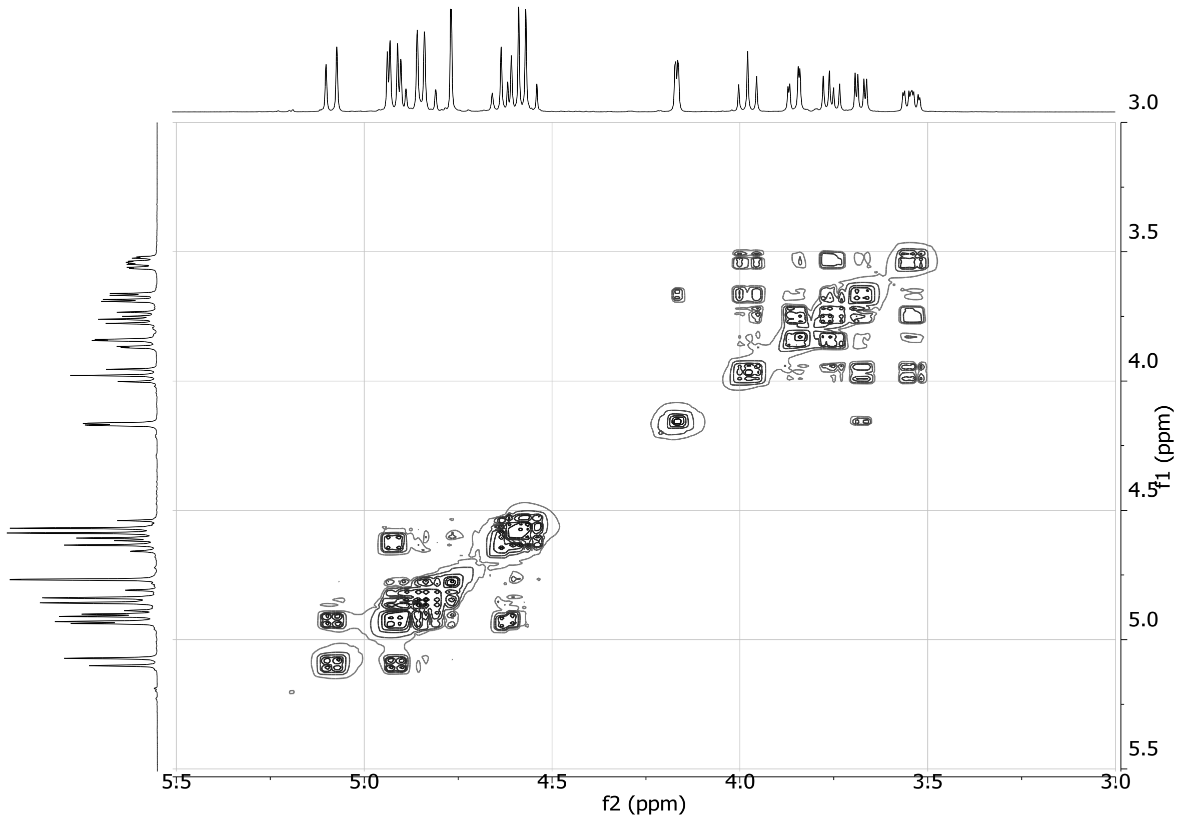

Supplementary Figure 51. HH-COSY NMR, $CDCl_3$ of compound **S5**

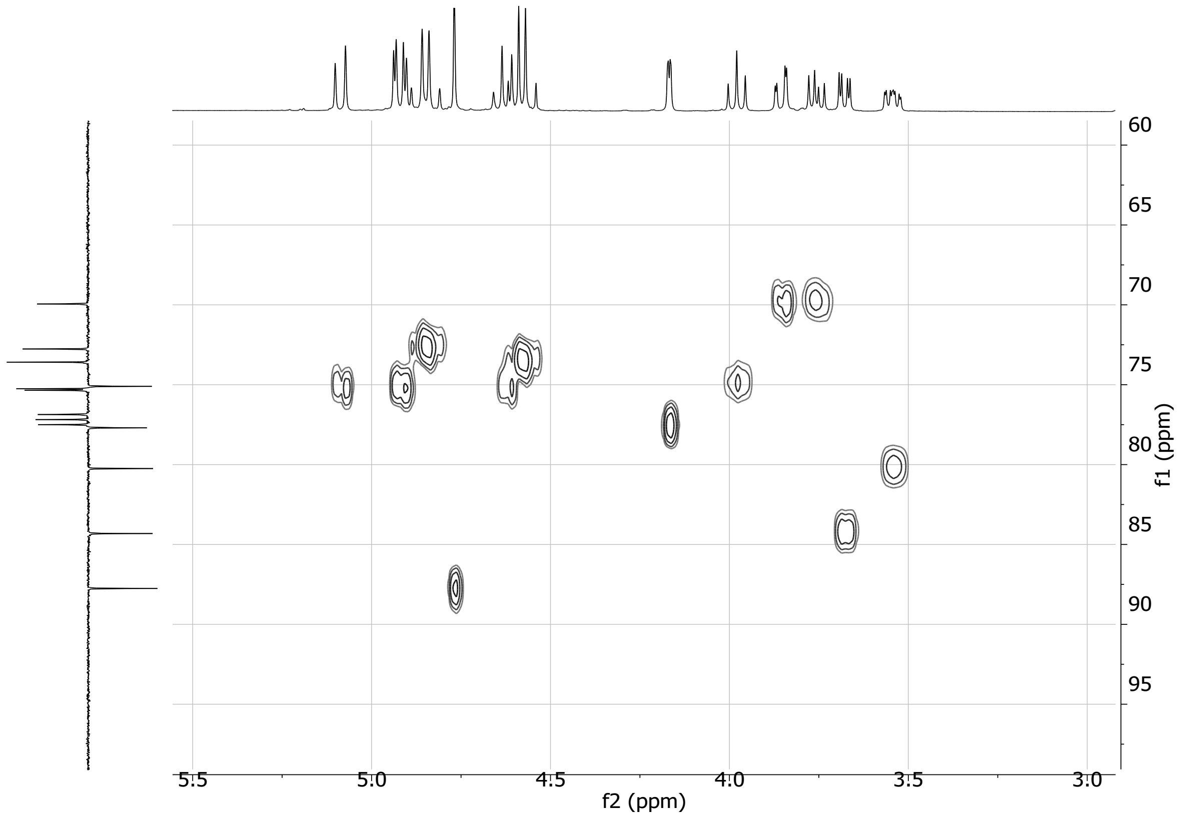

Supplementary Figure 52. HSQC NMR, $CDCl_3$ of compound **S5**

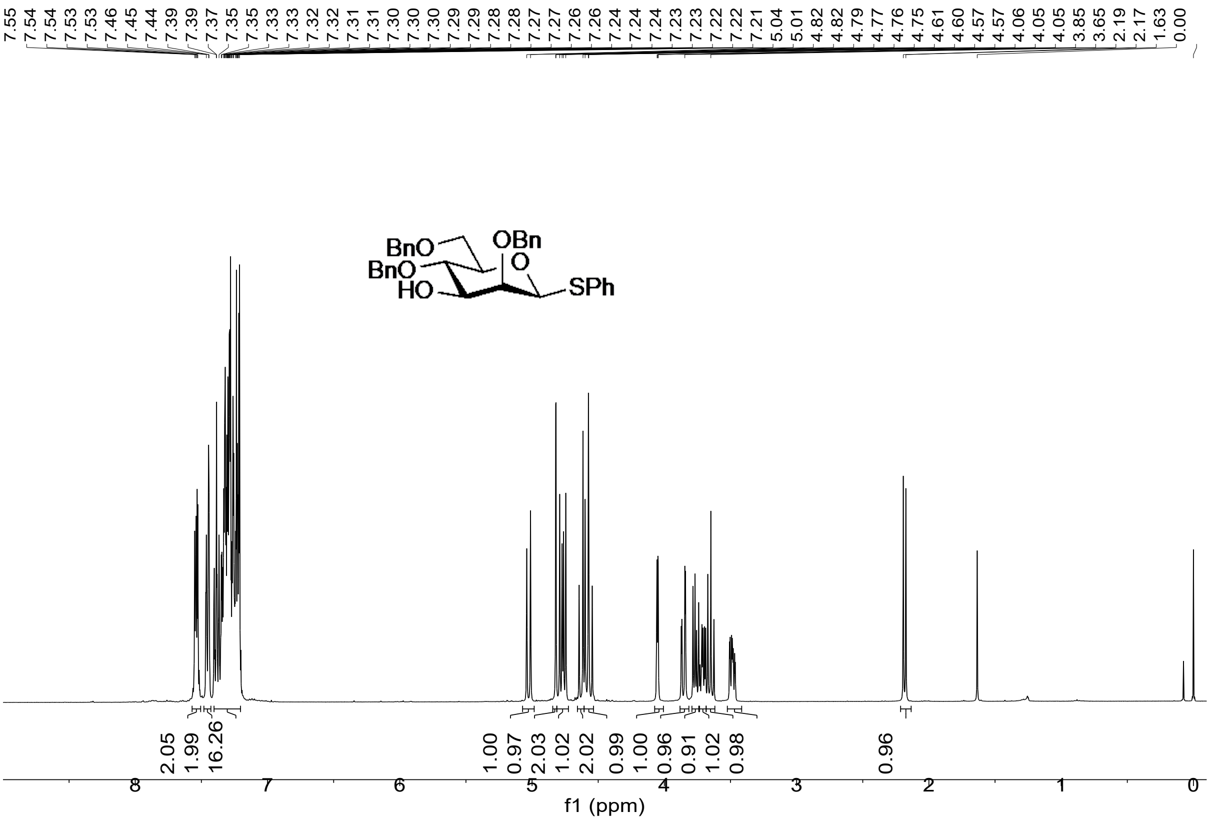

Supplementary Figure 53. ^{1}H NMR, 400 MHz, $CDCl_3$ of compound **S6**

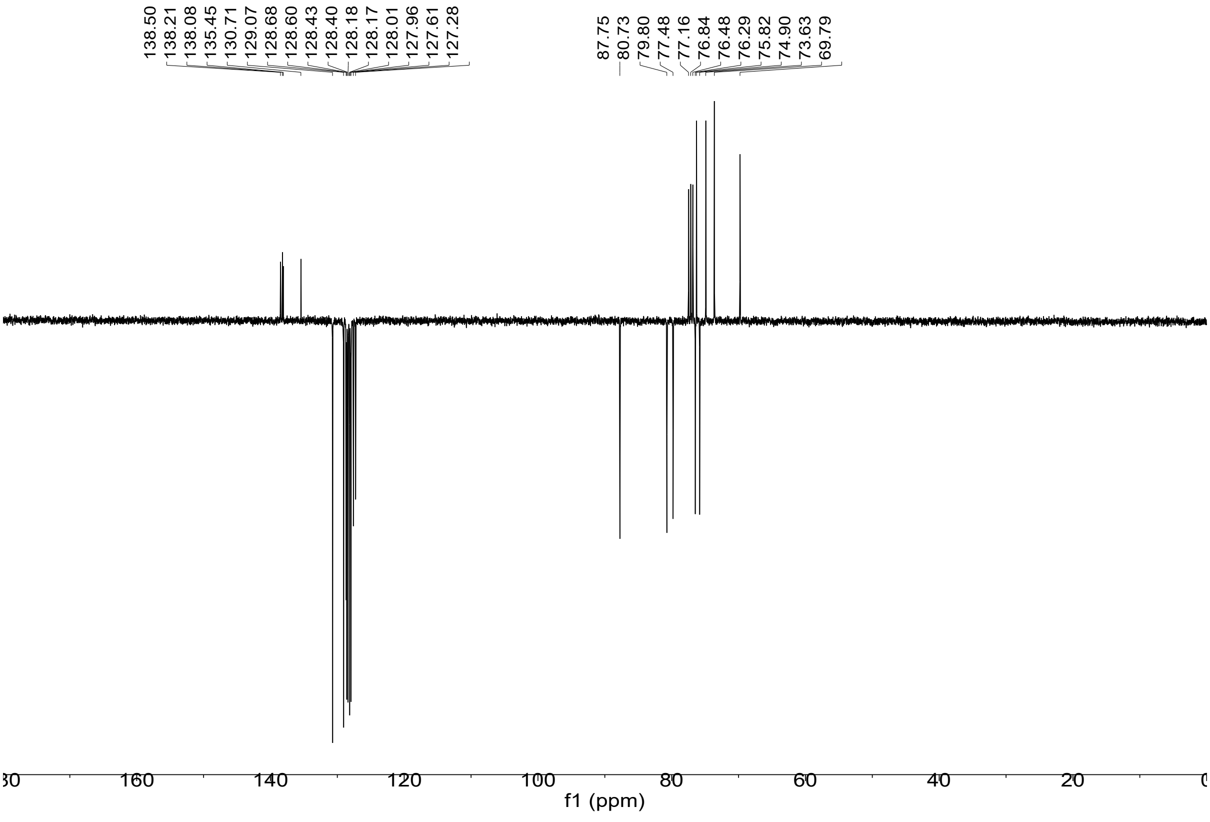

Supplementary Figure 54. ^{13}C NMR, 101 MHz, $CDCl_3$ of compound **S6**

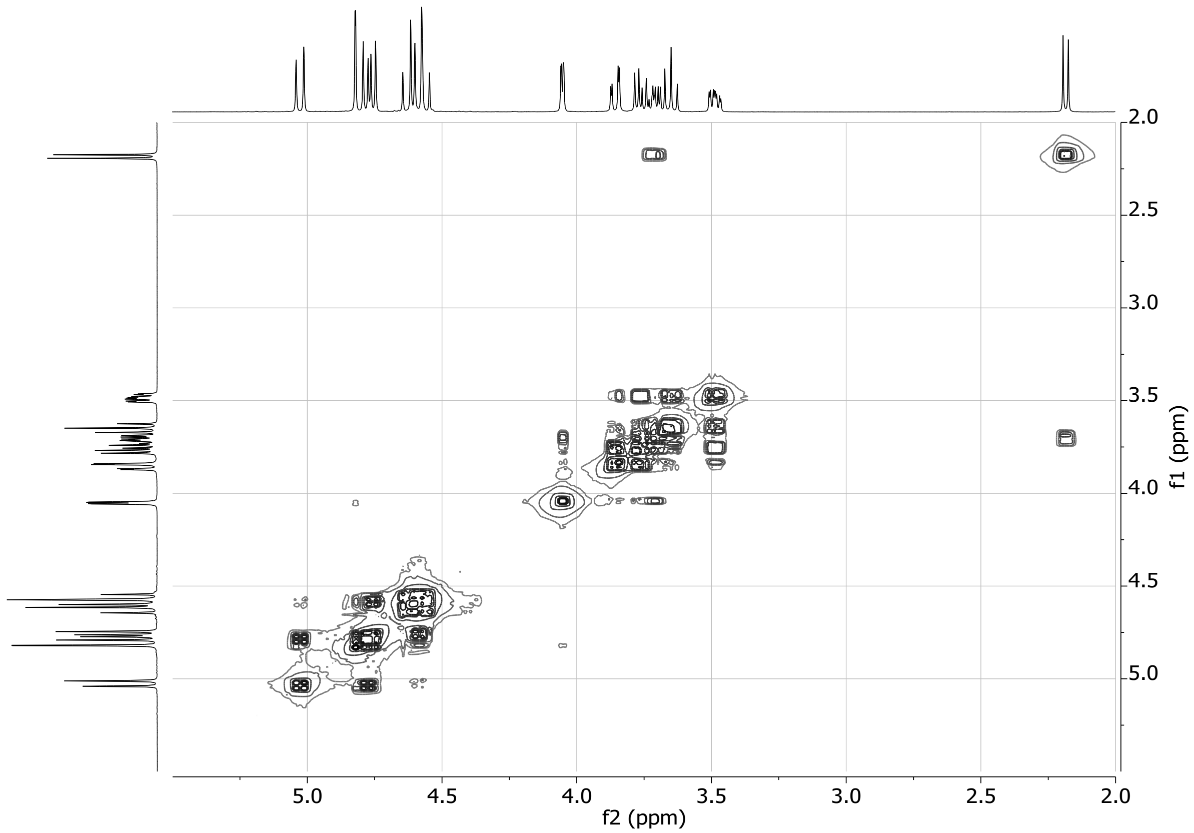

Supplementary Figure 55. HH-COSY NMR, $CDCl_3$ of compound **S6**

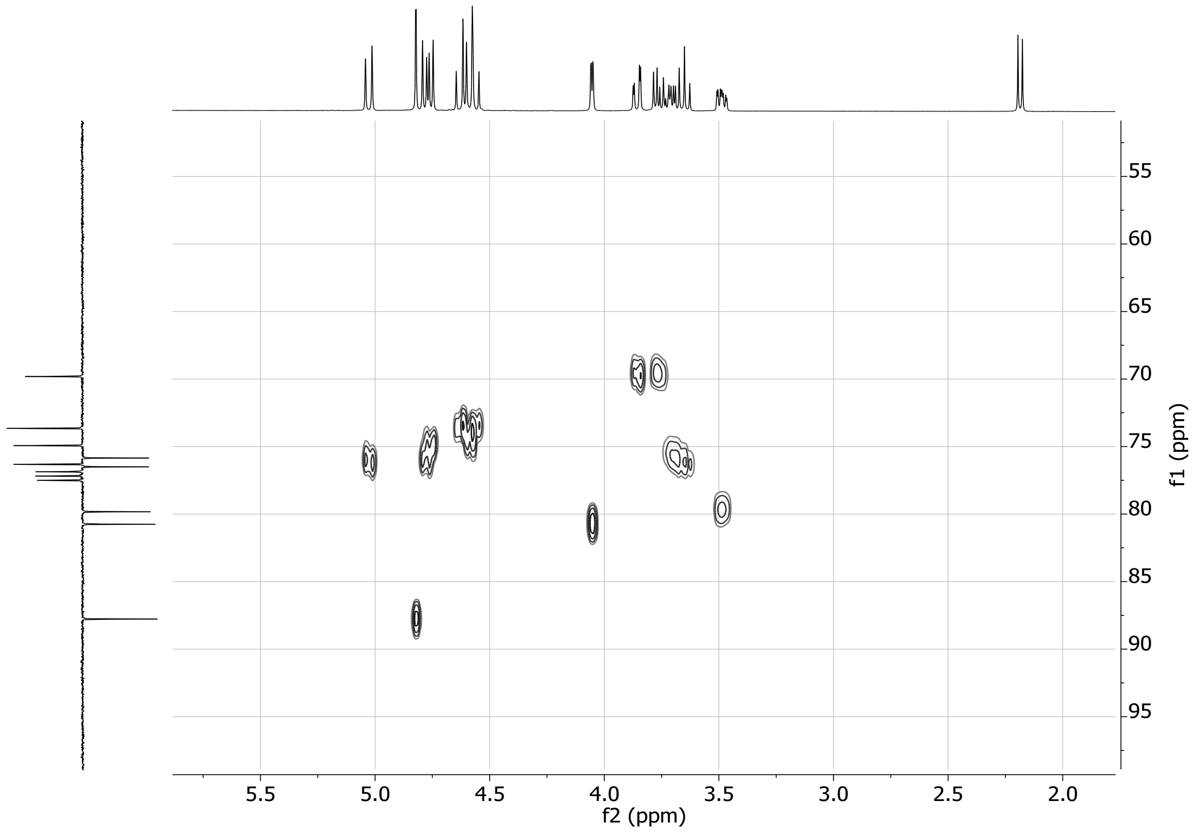

Supplementary Figure 56. HSQC NMR, $CDCl_3$ of compound **S6**

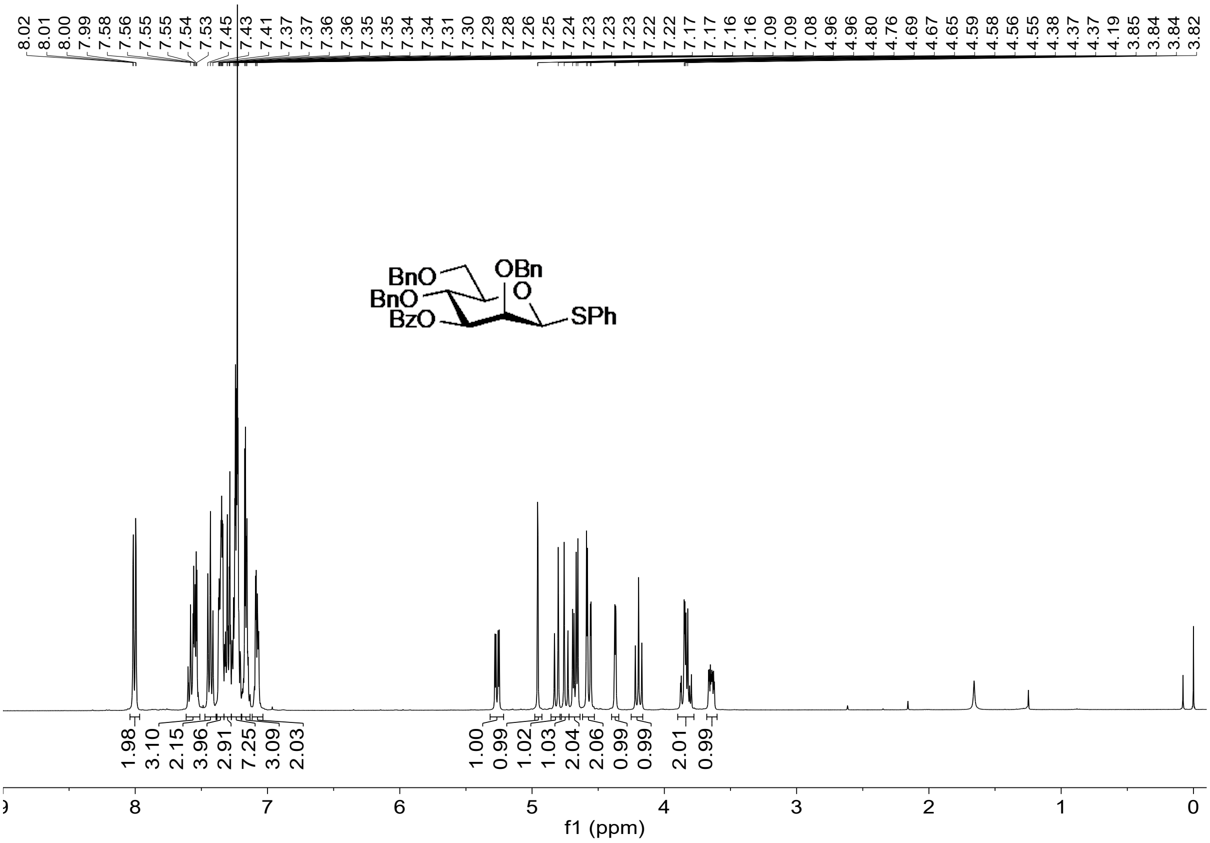

Supplementary Figure 57. ^{1}H NMR, 400 MHz, $CDCl_3$ of compound **13**

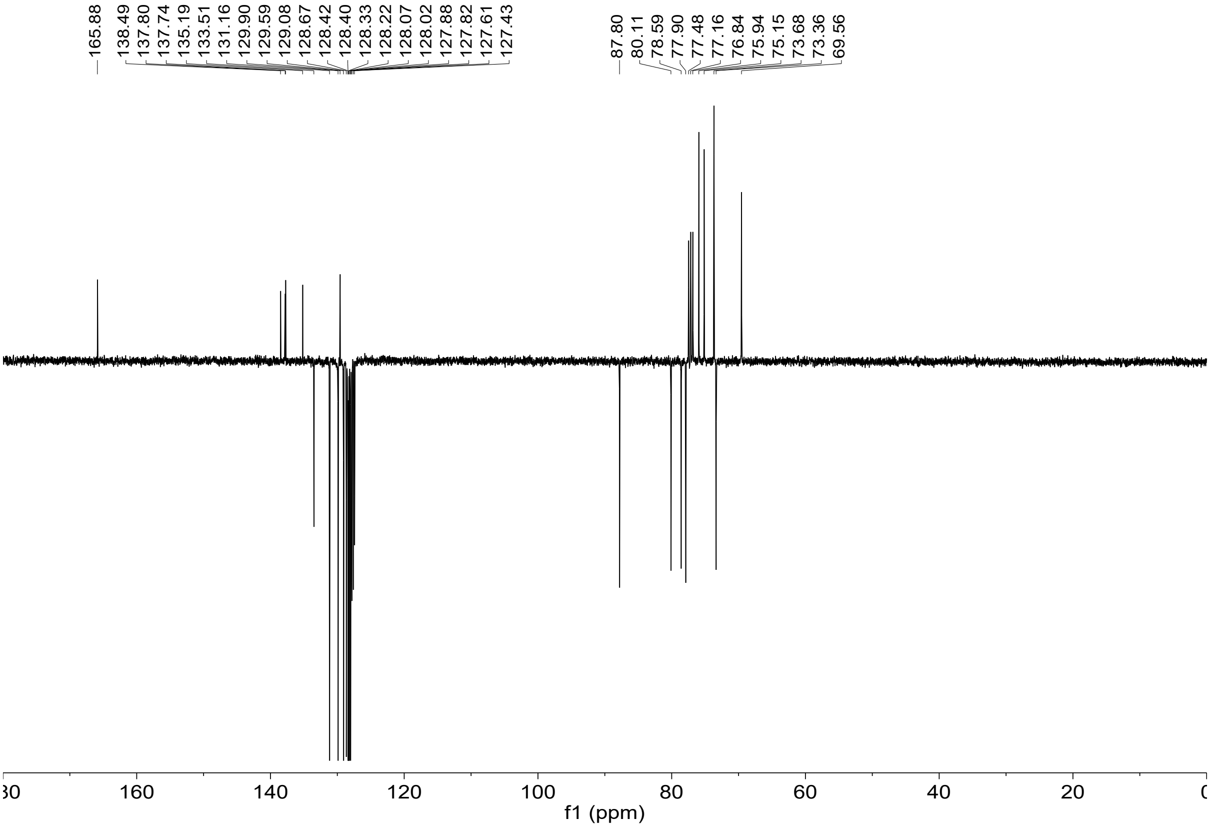

Supplementary Figure 58. ^{13}C NMR, 101 MHz, $CDCl_3$ of compound **13**

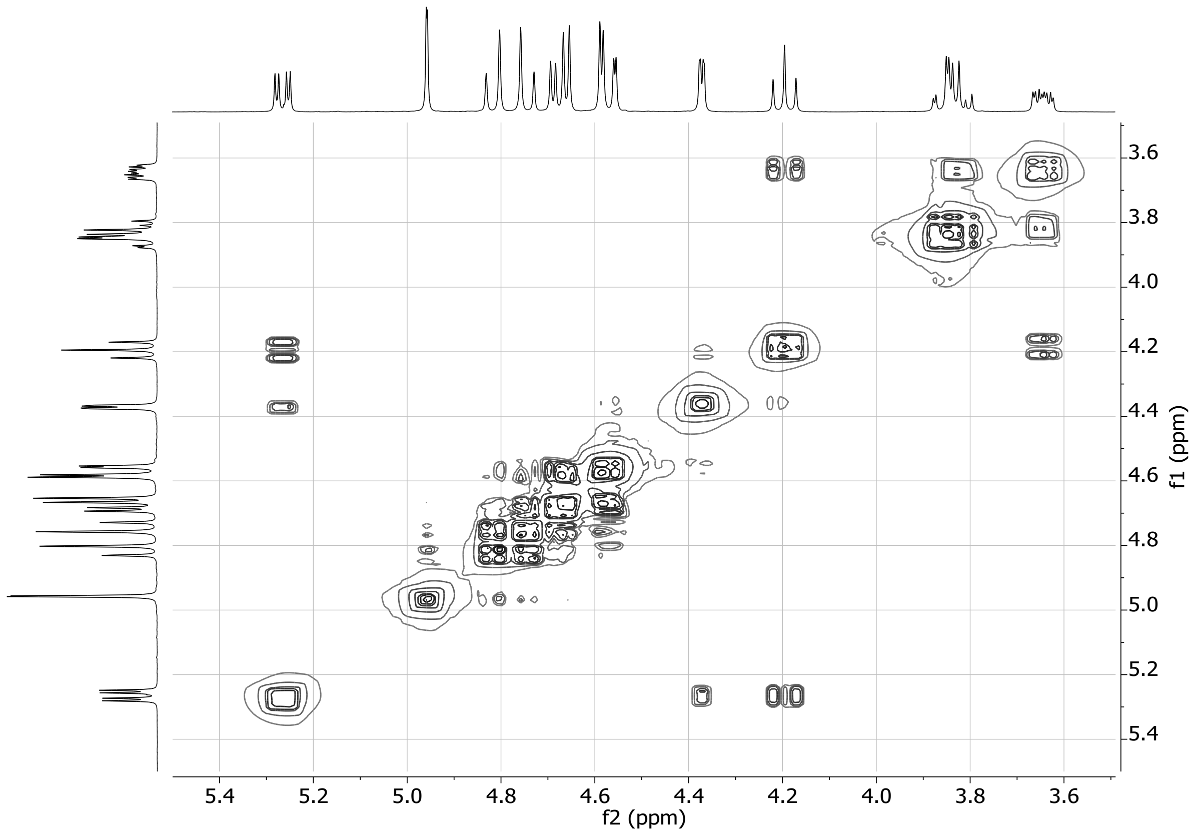

Supplementary Figure 59. HH-COSY NMR, $CDCl_3$ of compound **13**

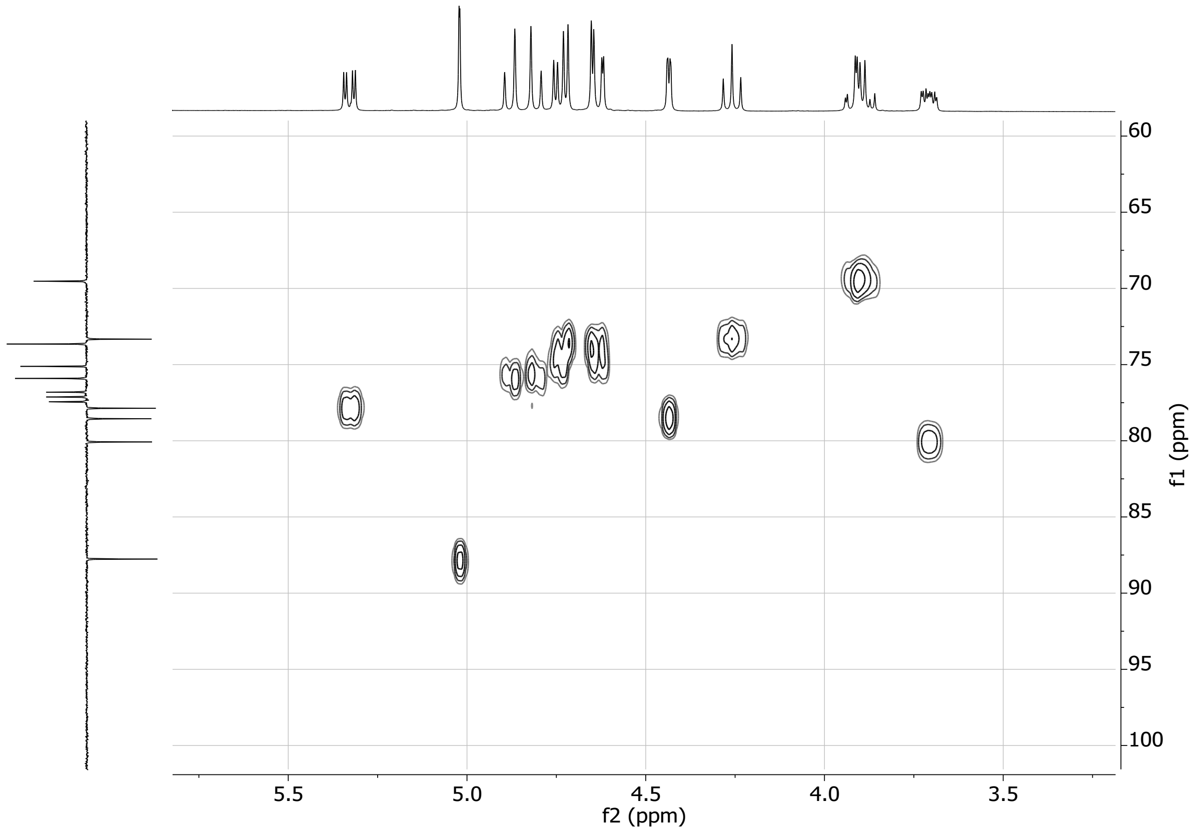

Supplementary Figure 60. HSQC NMR, $CDCl_3$ of compound **13**

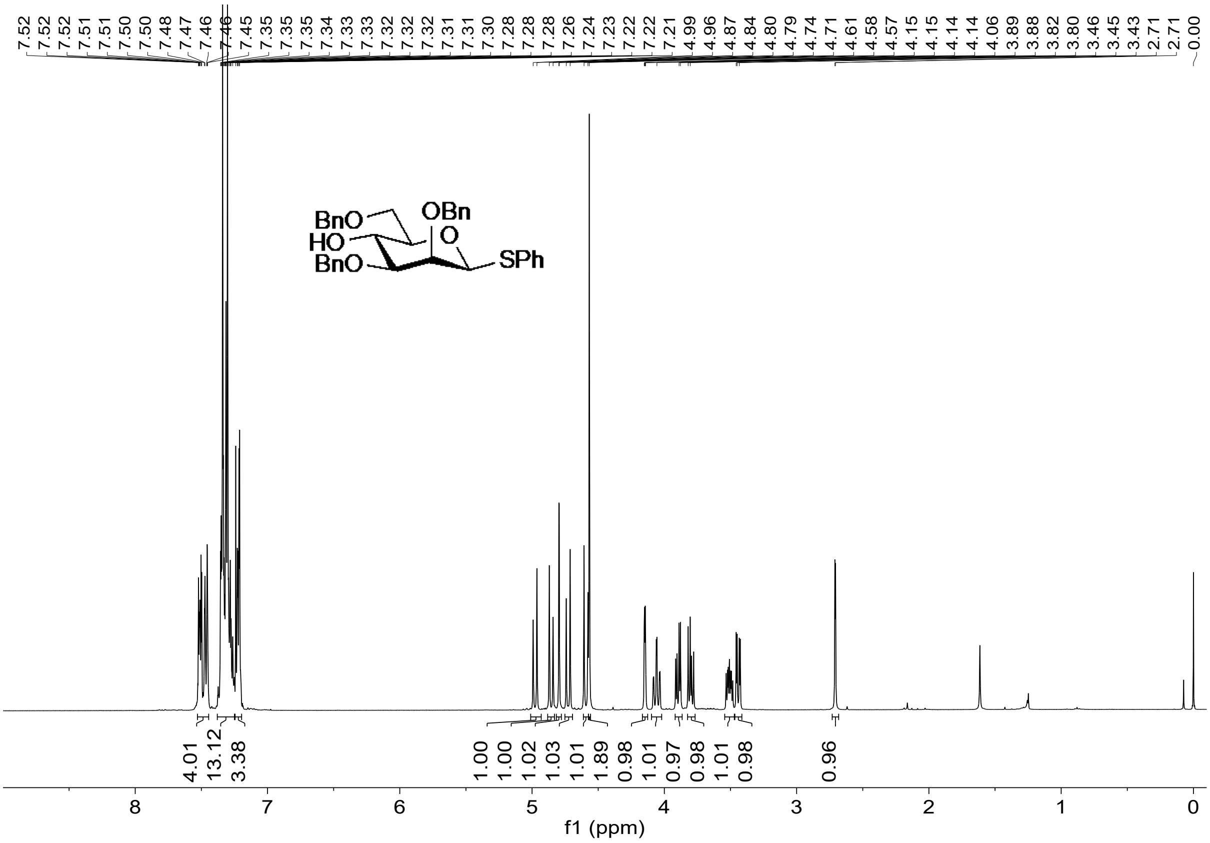

Supplementary Figure 61. ^{1}H NMR, 400 MHz, $CDCl_3$ of compound **S7**

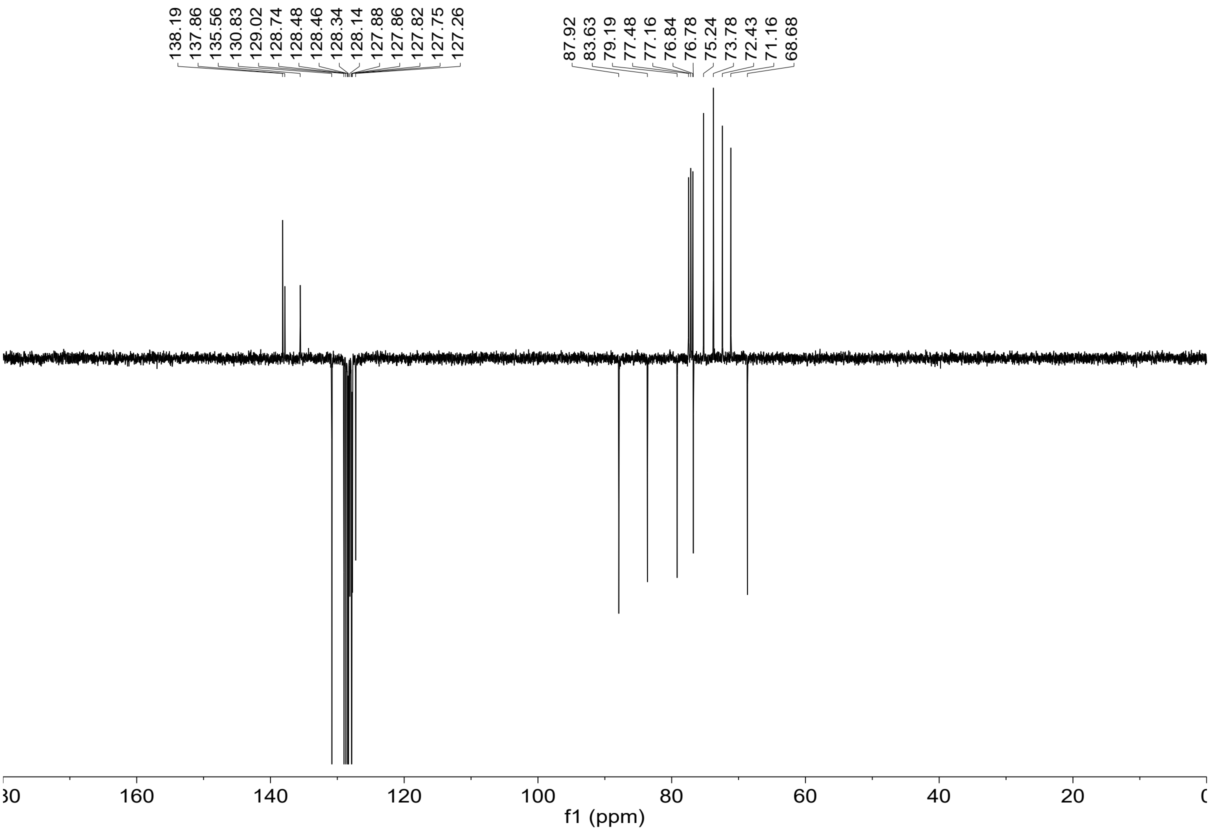

Supplementary Figure 62. ^{13}C NMR, 101 MHz, $CDCl_3$ of compound **S7**

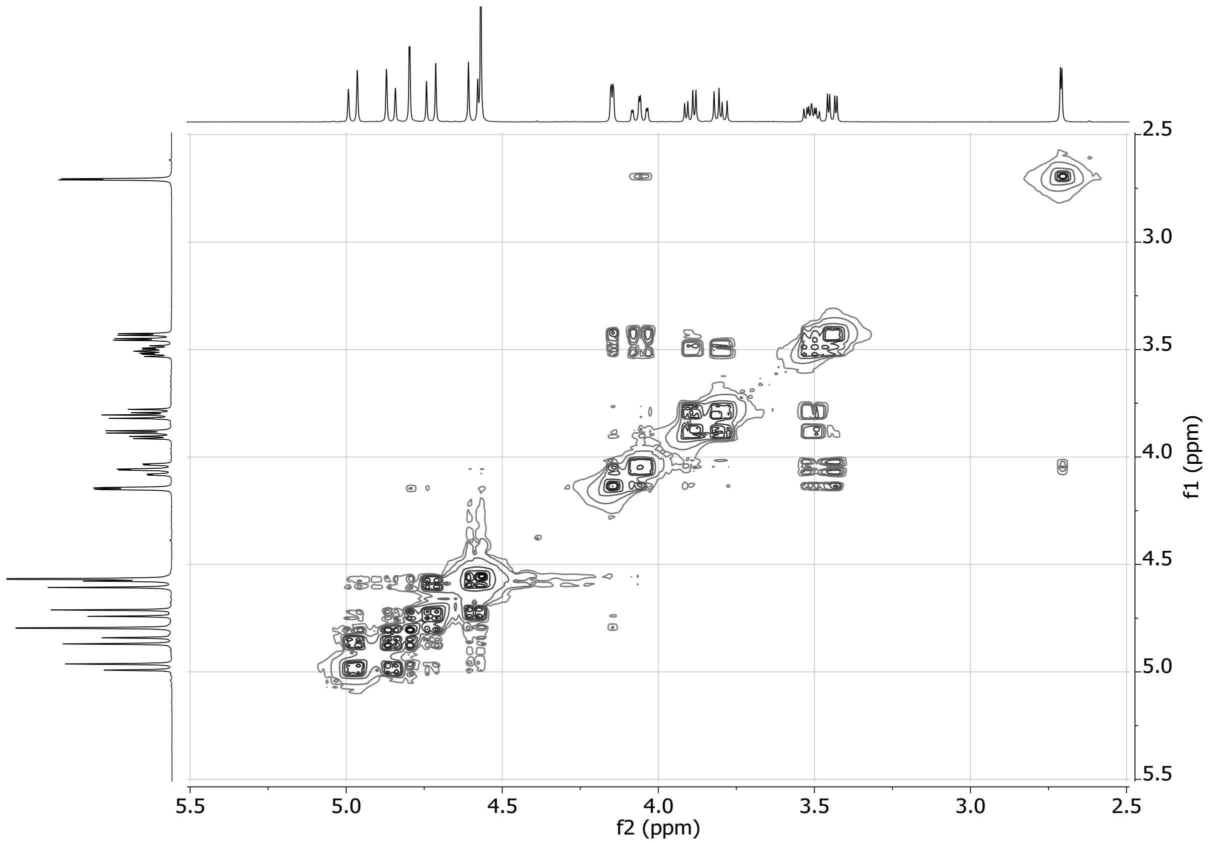

Supplementary Figure 63. HH-COSY NMR, $CDCl_3$ of compound **S7**

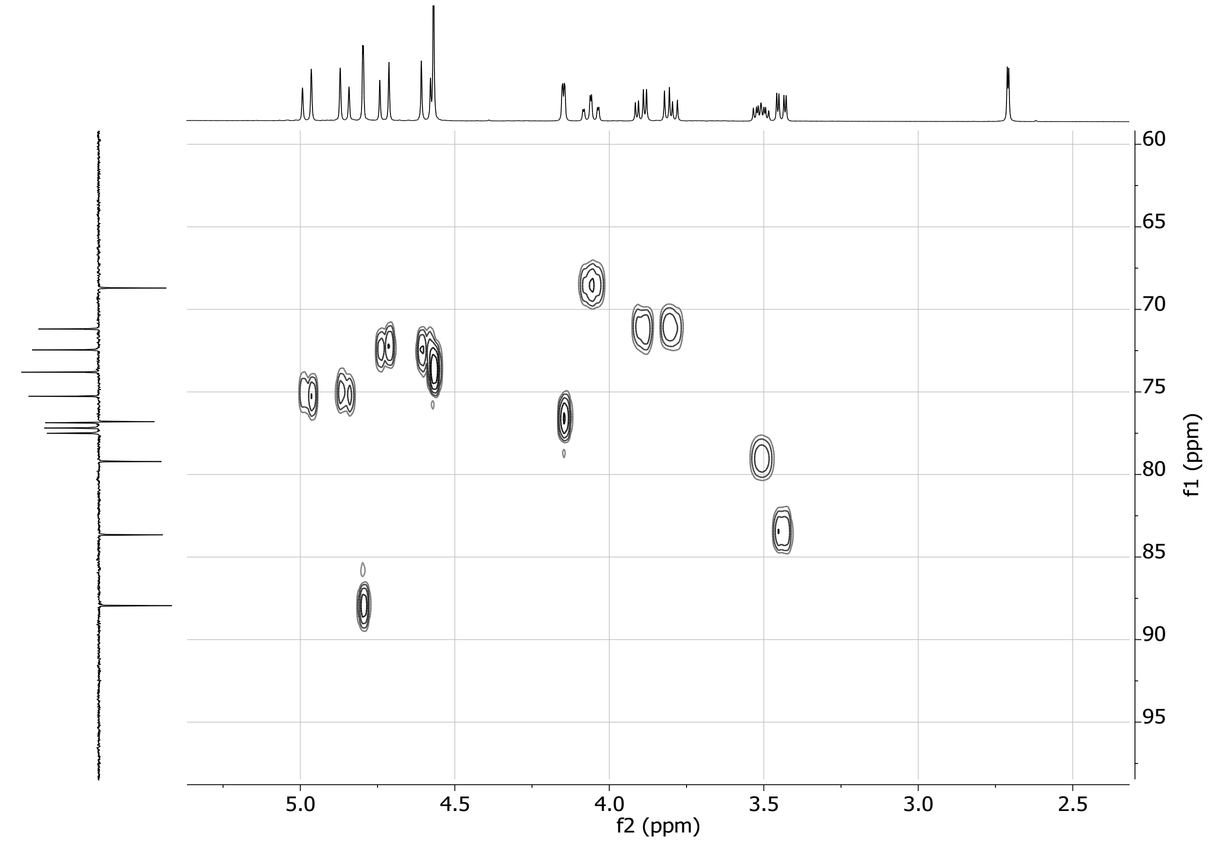

Supplementary Figure 64. HSQC NMR, $CDCl_3$ of compound **S7**

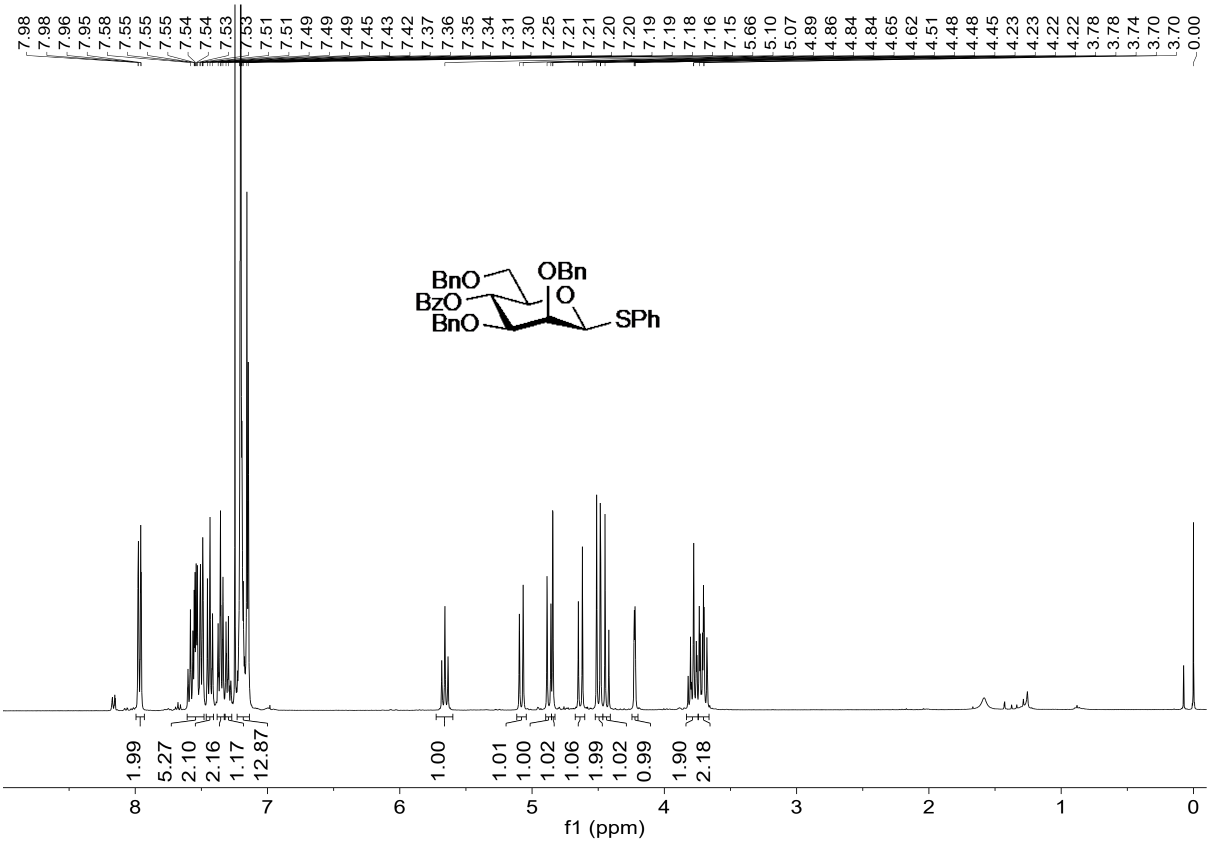

Supplementary Figure 65. ^{1}H NMR, 400 MHz, $CDCl_3$ of compound **14**

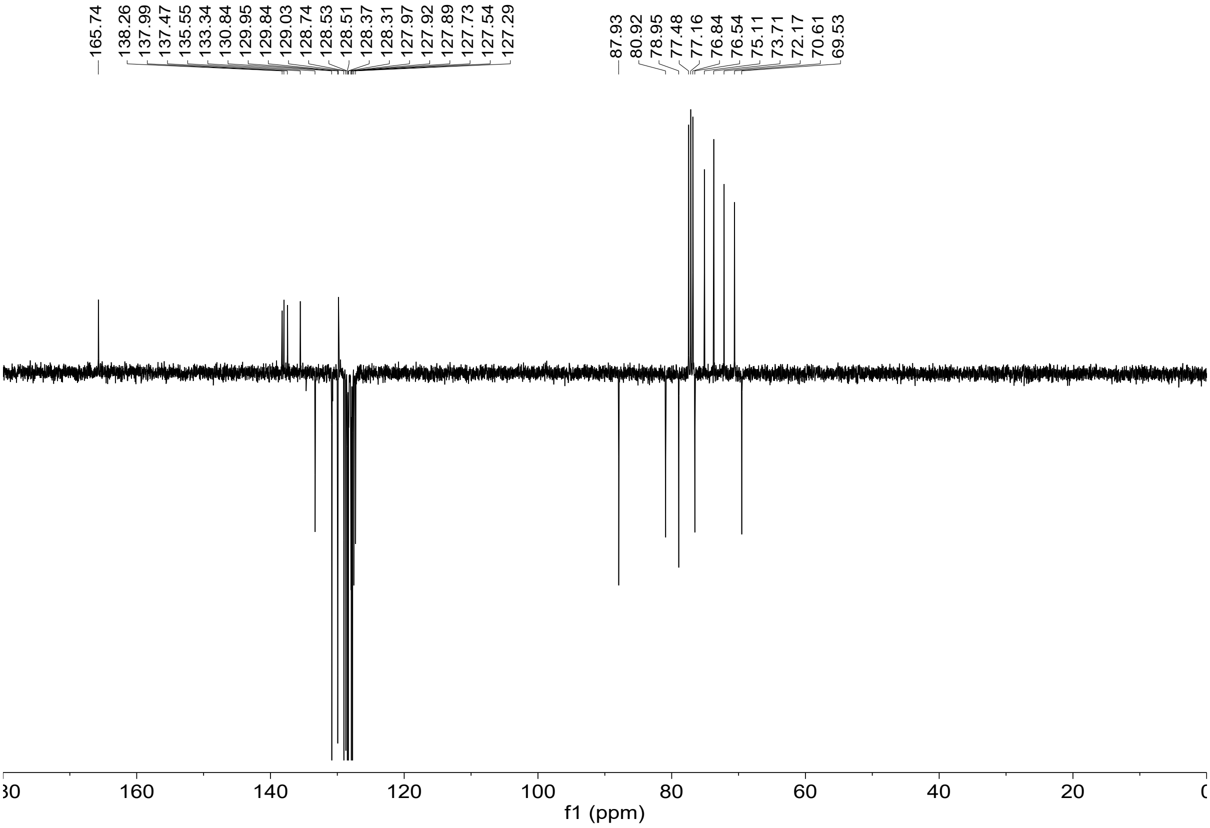

Supplementary Figure 66. ^{13}C NMR, 101 MHz, $CDCl_3$ of compound **14**

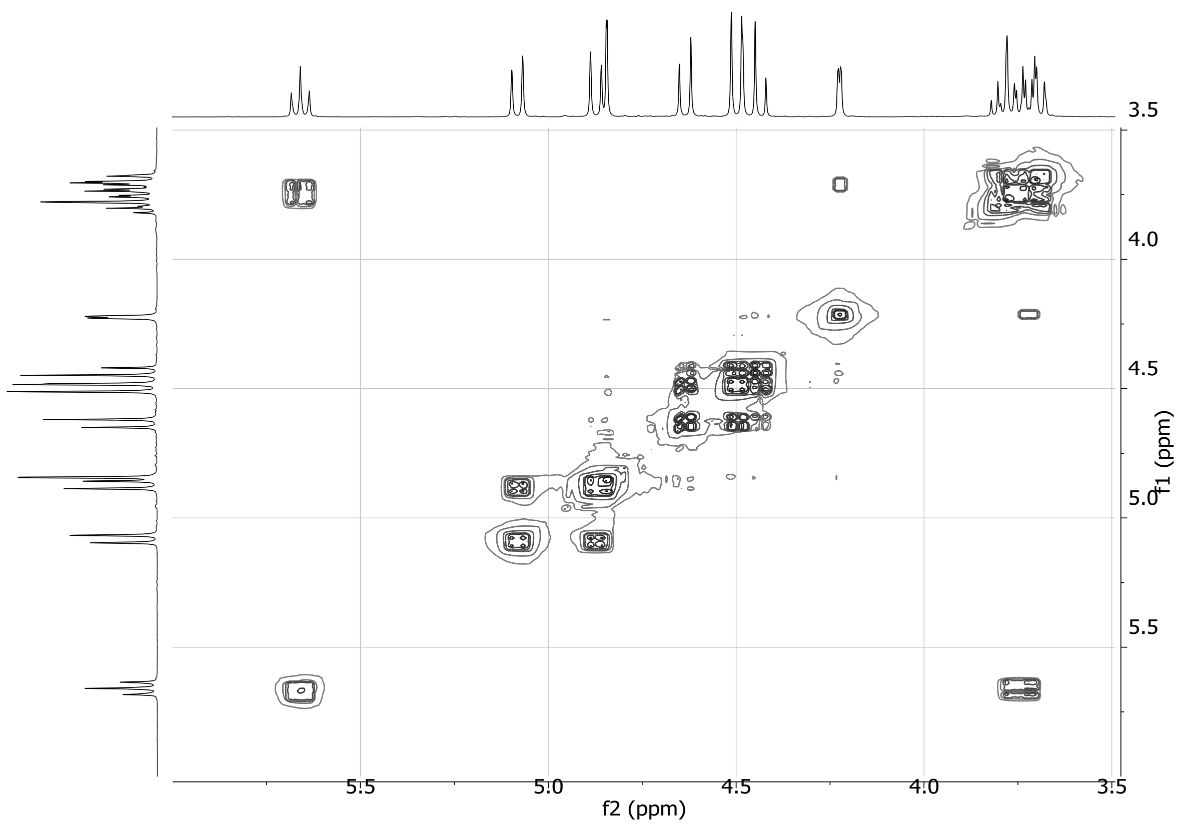

Supplementary Figure 67. HH-COSY NMR, $CDCl_3$ of compound **14**

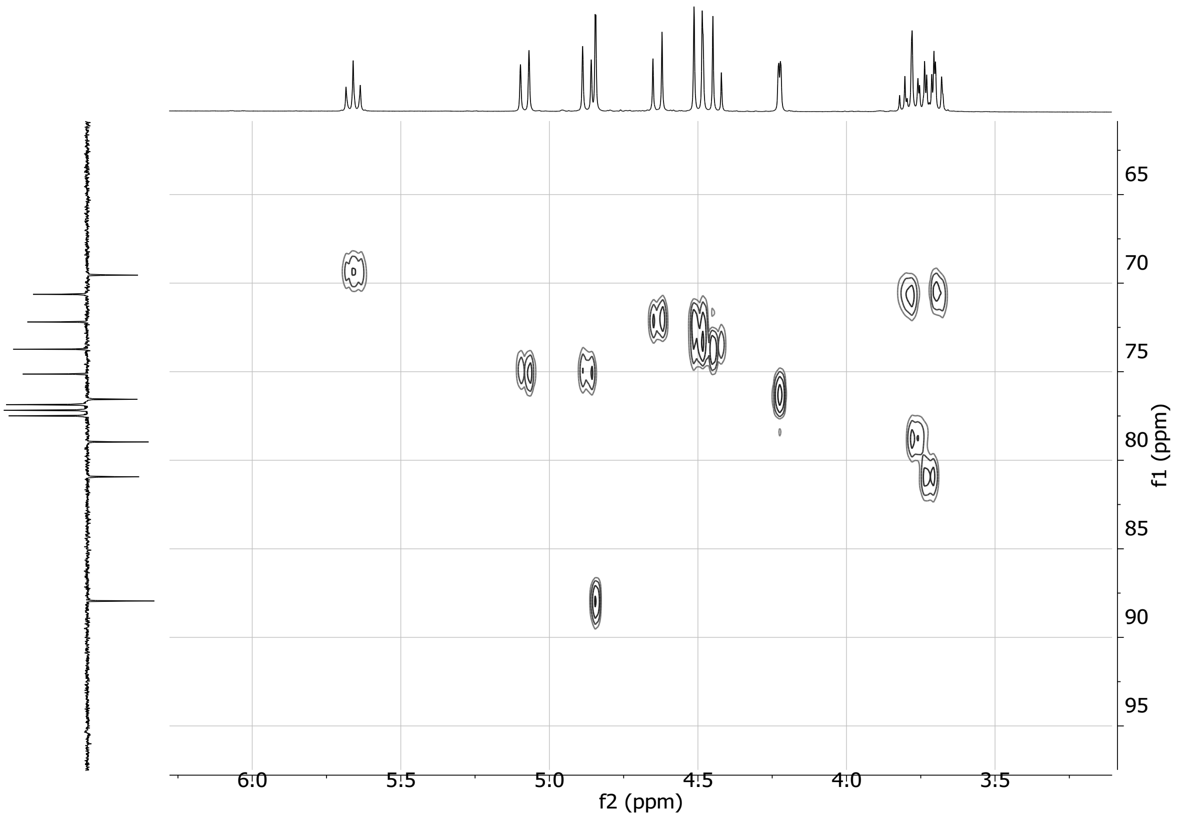

Supplementary Figure 68. HSQC NMR, $CDCl_3$ of compound **14**

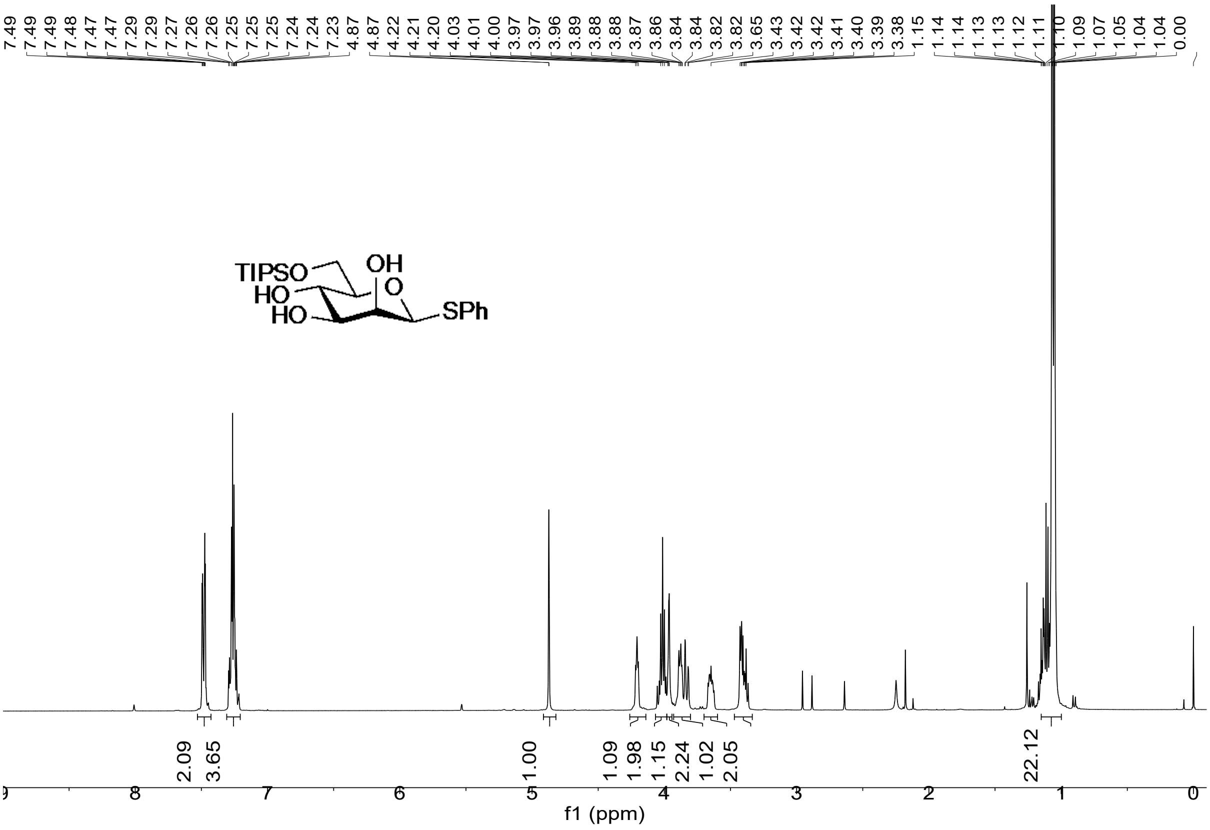

Supplementary Figure 69. ^{1}H NMR, 400 MHz, $CDCl_3$ of compound **S9**

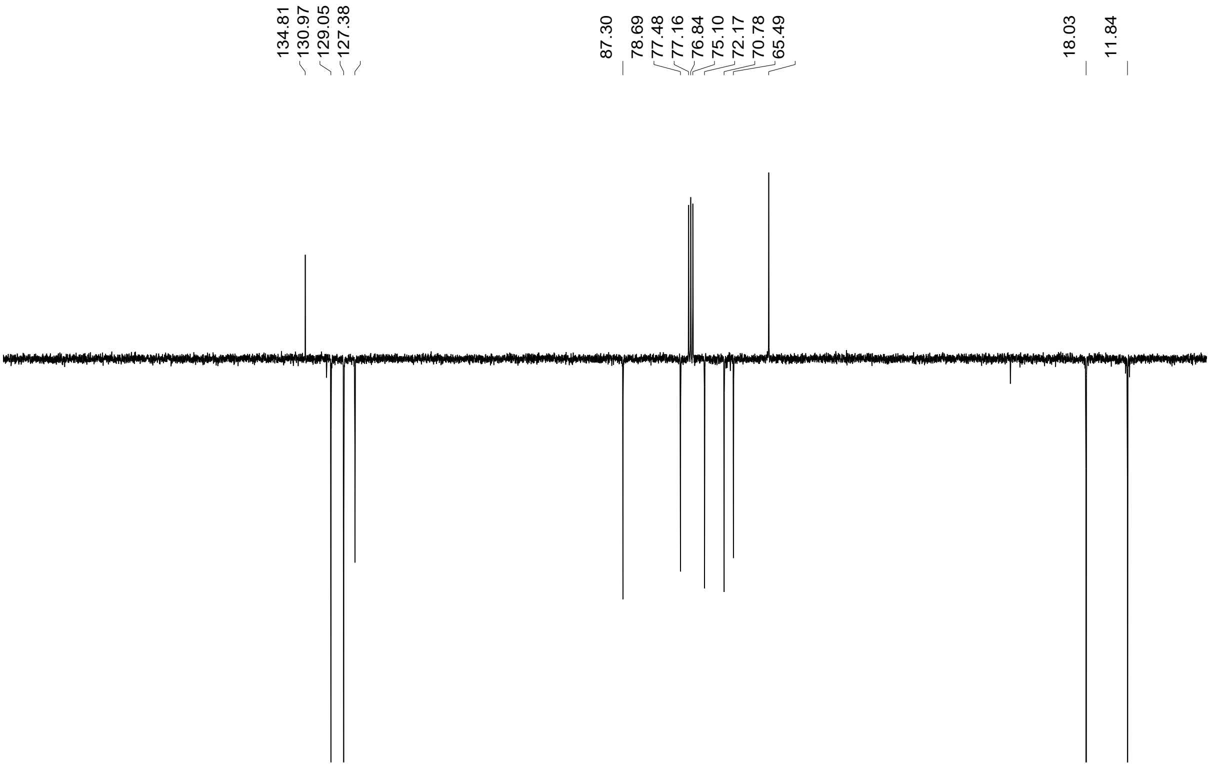

Supplementary Figure 70. ^{13}C NMR, 101 MHz, $CDCl_3$ of compound **S9**

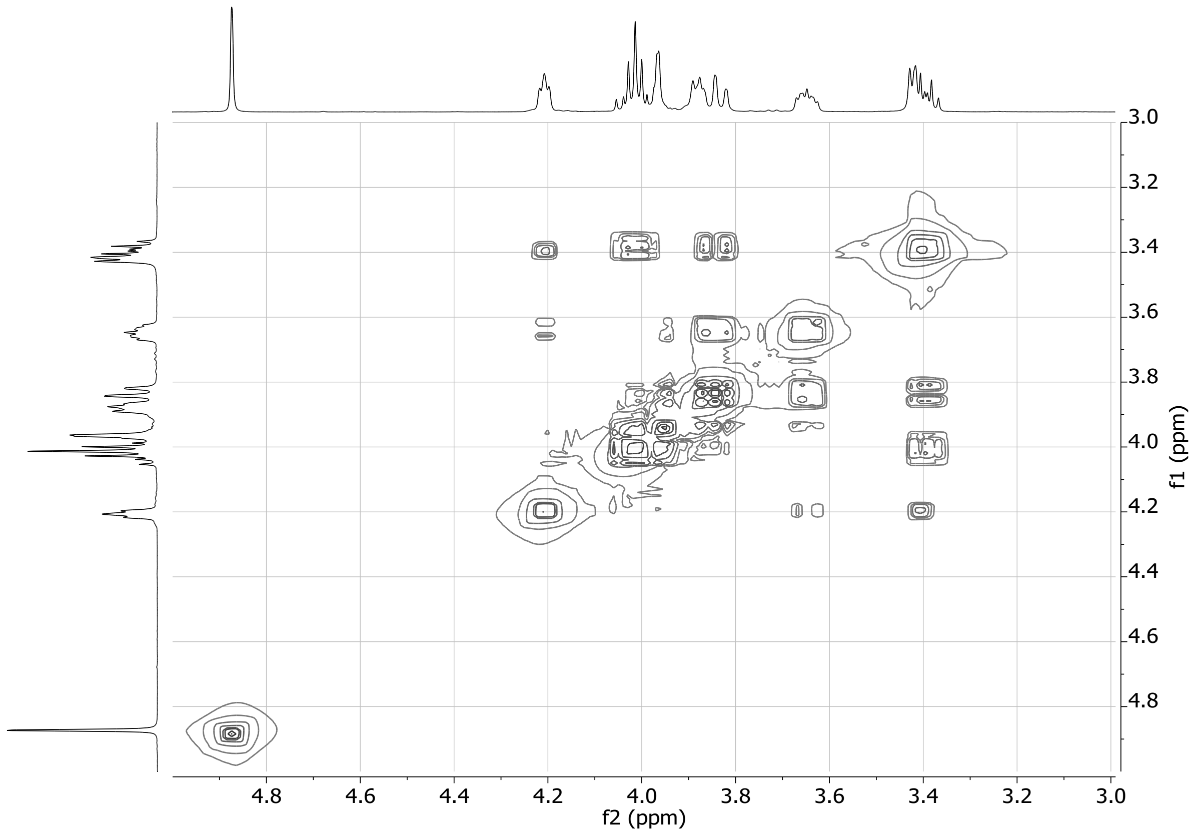

Supplementary Figure 71. HH-COSY NMR, $CDCl_3$ of compound **S9**

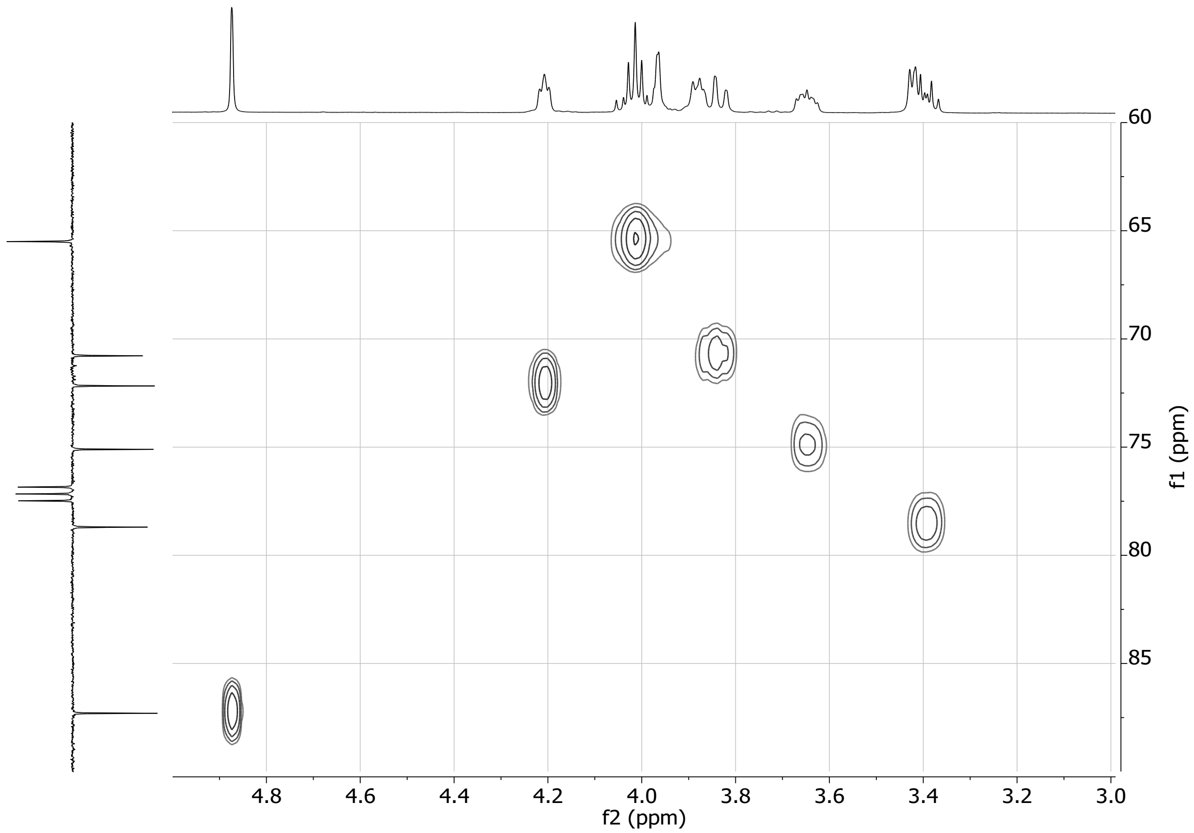

Supplementary Figure 72. HSQC NMR, $CDCl_3$ of compound **S9**

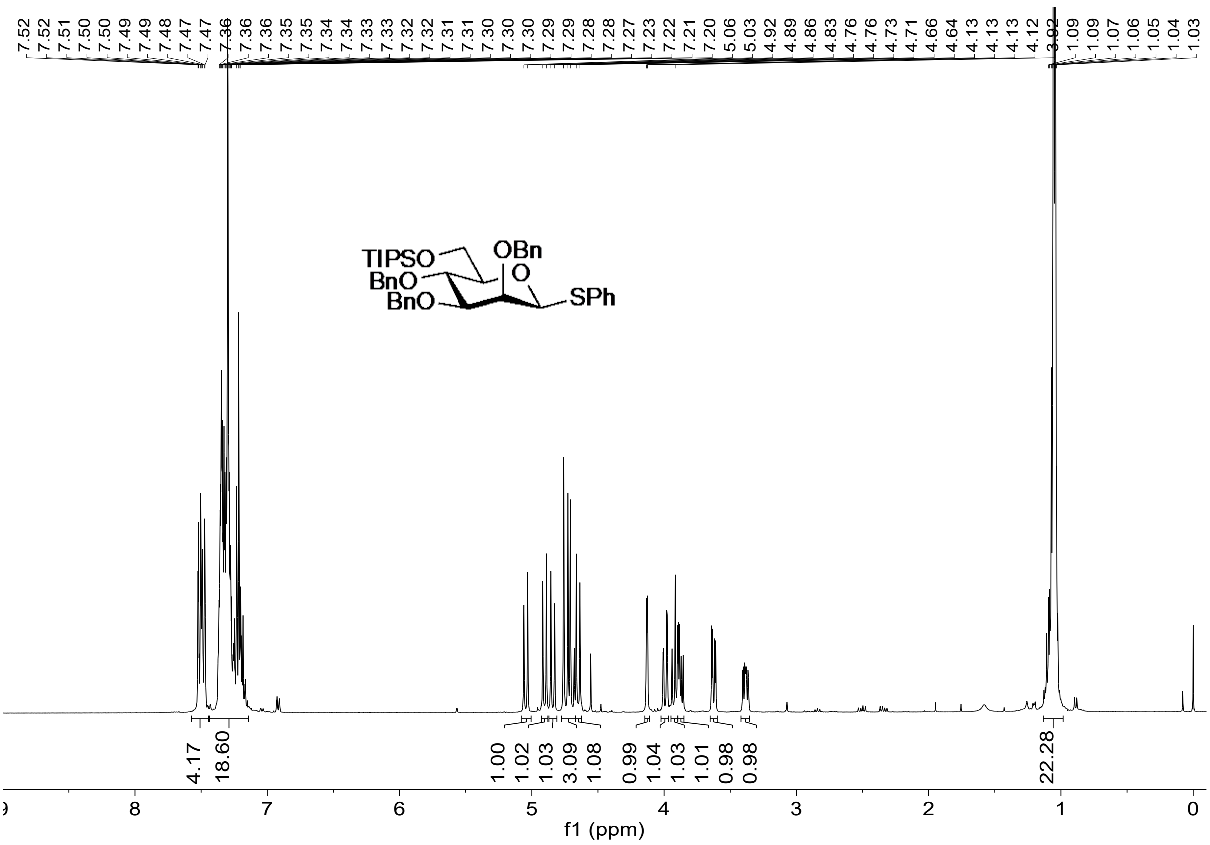

Supplementary Figure 73. ^{1}H NMR, 400 MHz, $CDCl_3$ of compound **S10**

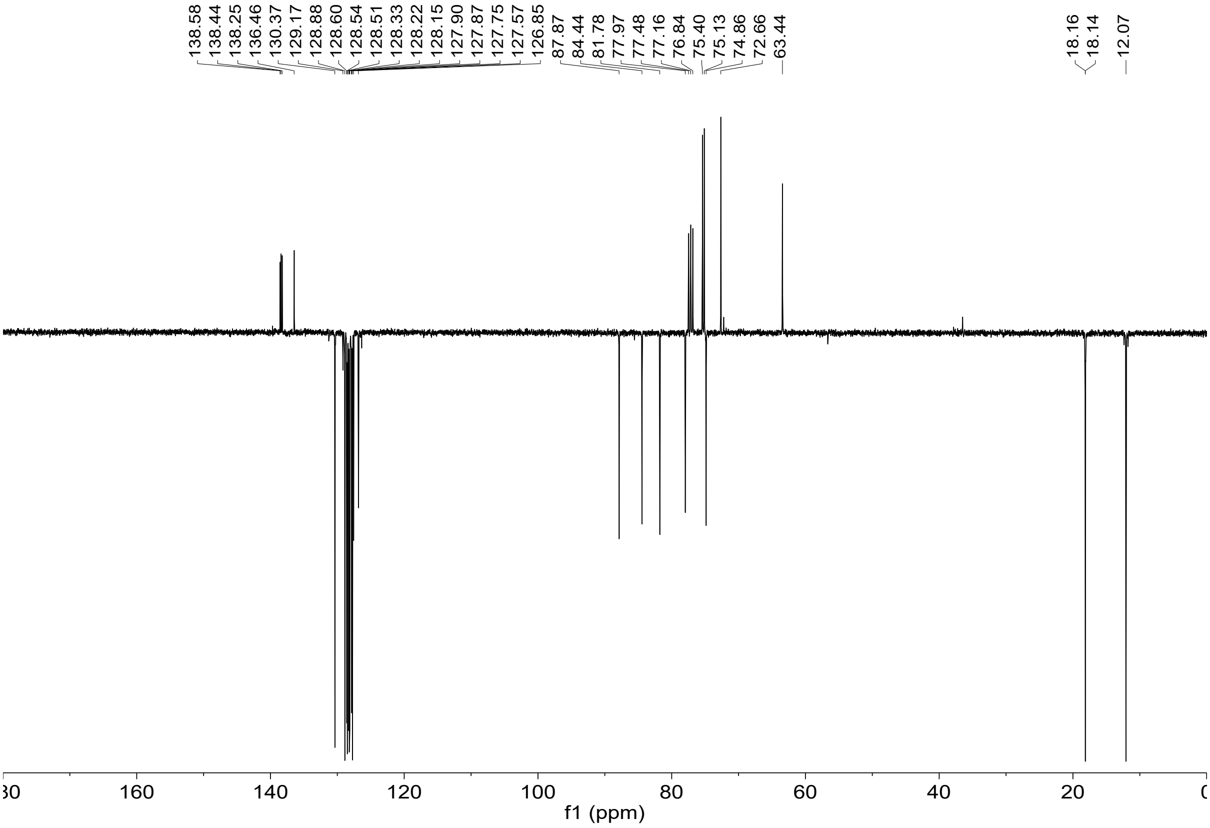

Supplementary Figure 74. ^{13}C NMR, 101 MHz, $CDCl_3$ of compound **S10**

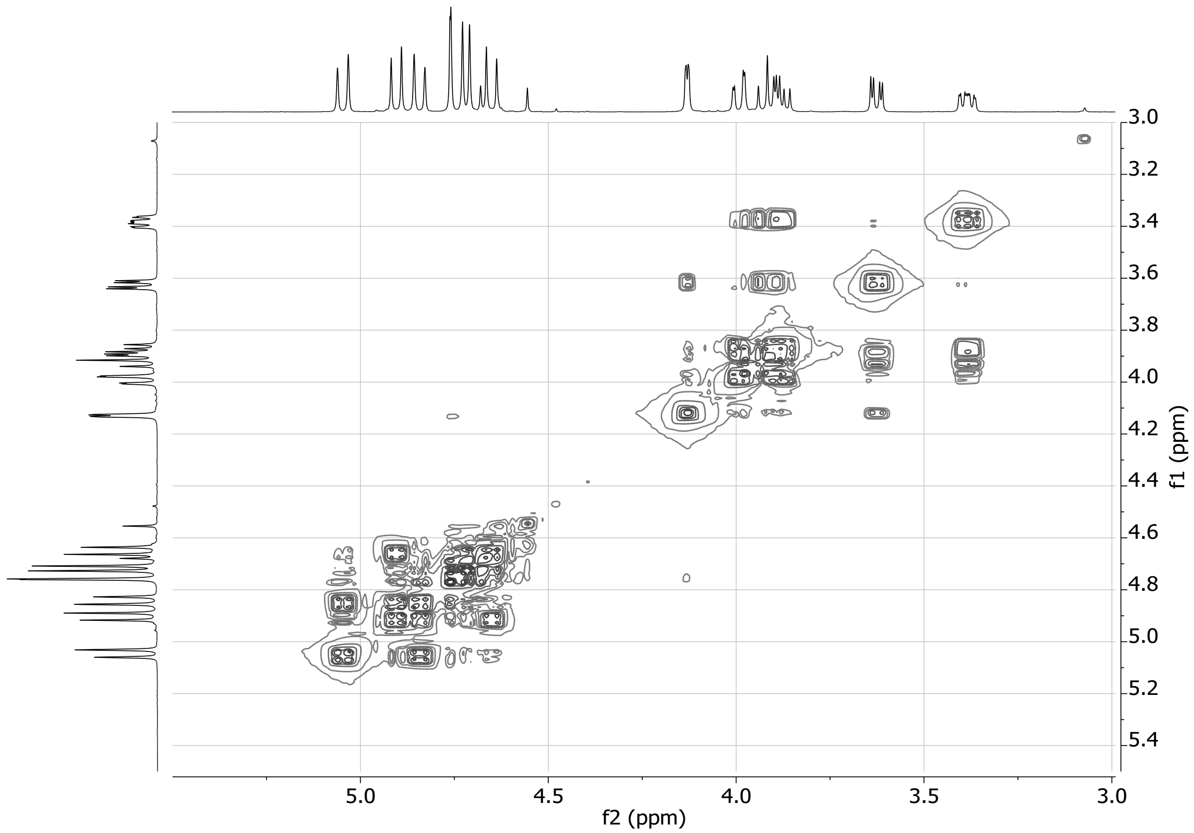

Supplementary Figure 75. HH-COSY NMR, $CDCl_3$ of compound **S10**

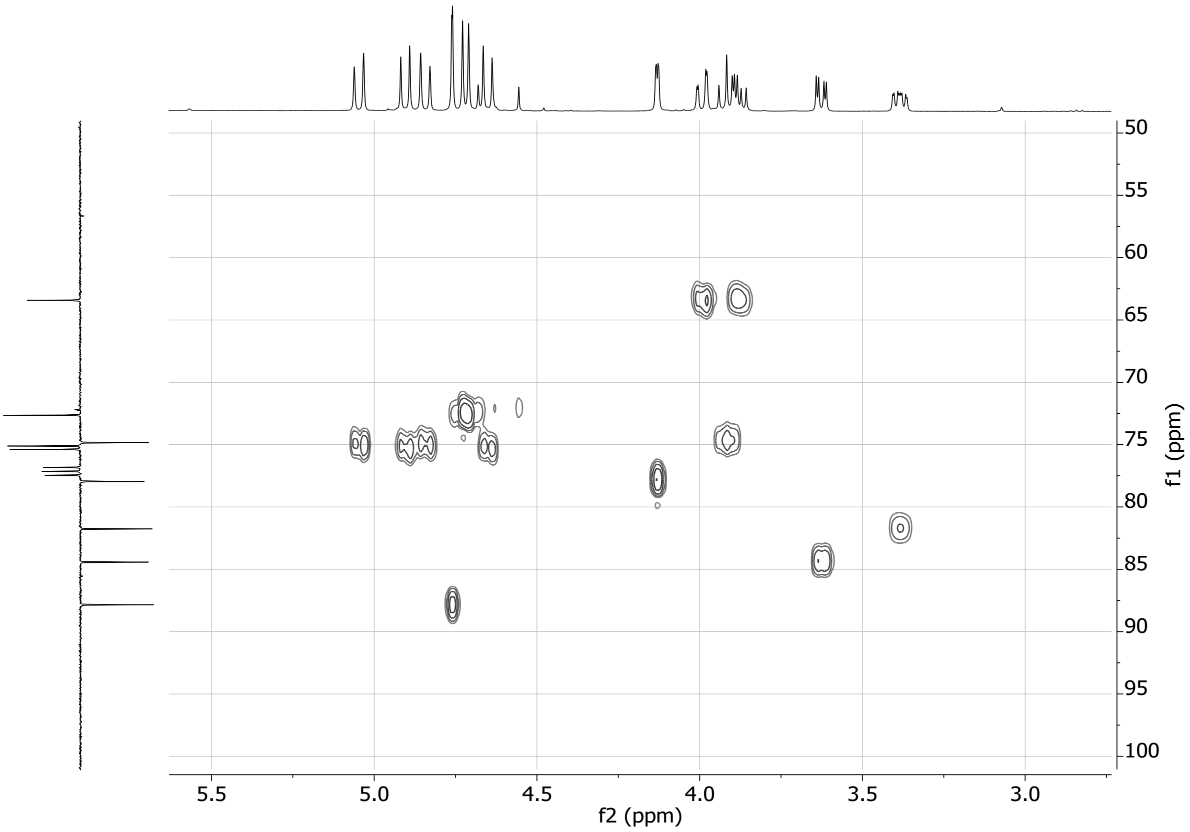

Supplementary Figure 76. HSQC NMR, $CDCl_3$ of compound **S10**

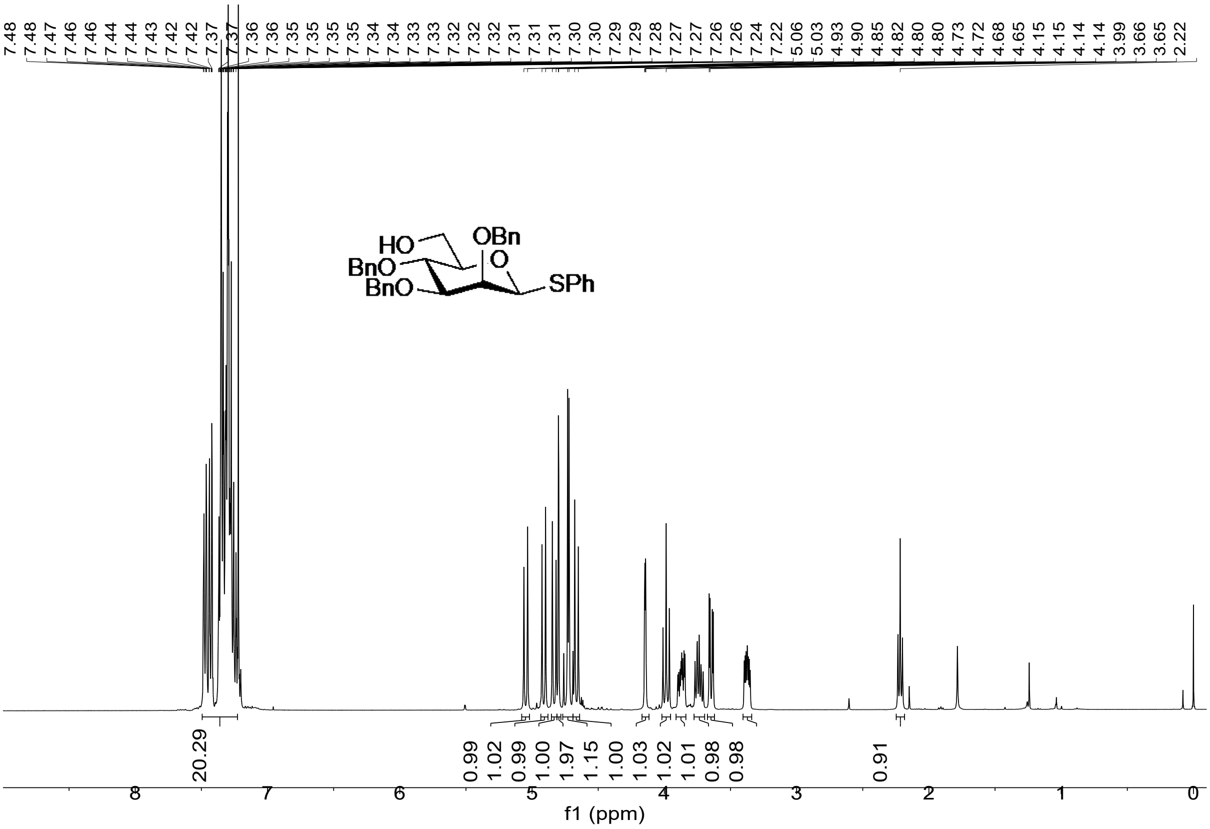

Supplementary Figure 77. ^{1}H NMR, 400 MHz, $CDCl_3$ of compound **S11**

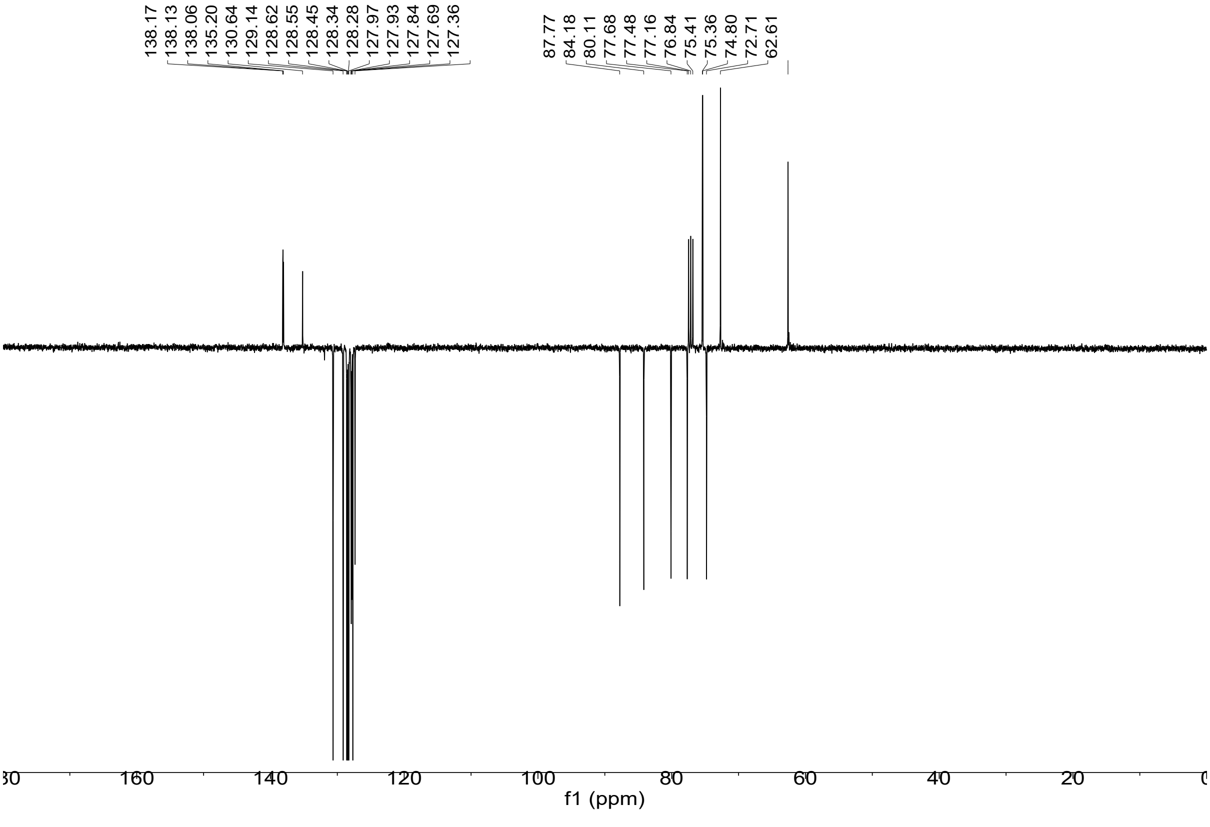

Supplementary Figure 78. ^{13}C NMR, 101 MHz, $CDCl_3$ of compound **S11**

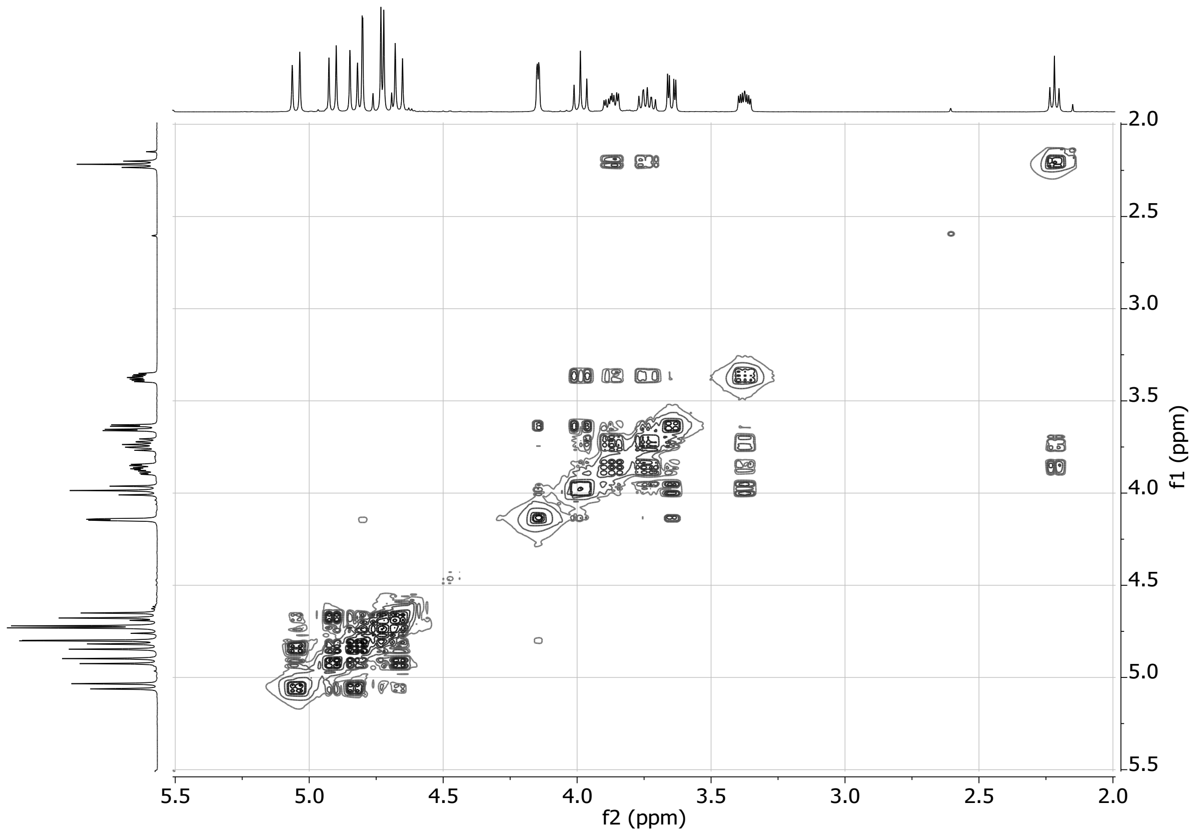

Supplementary Figure 79. HH-COSY NMR, $CDCl_3$ of compound **S11**

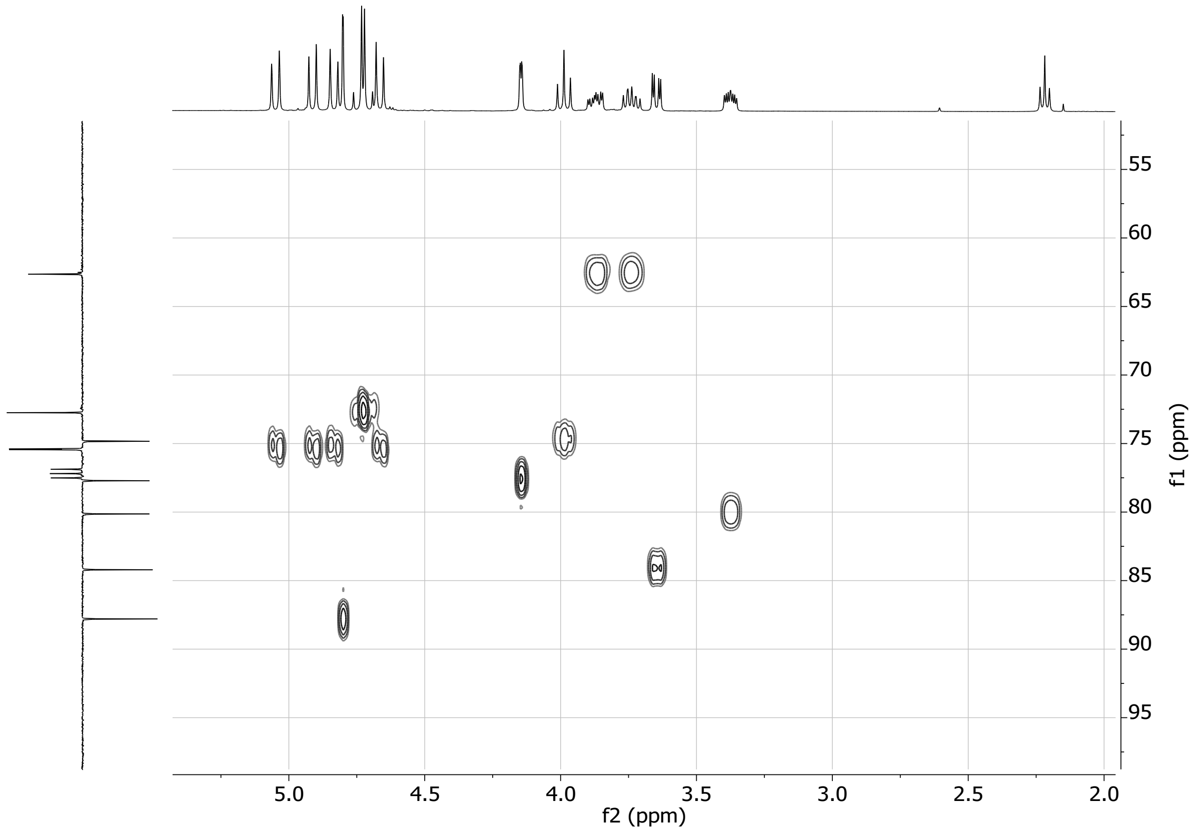

Supplementary Figure 80. HSQC NMR, $CDCl_3$ of compound **S11**

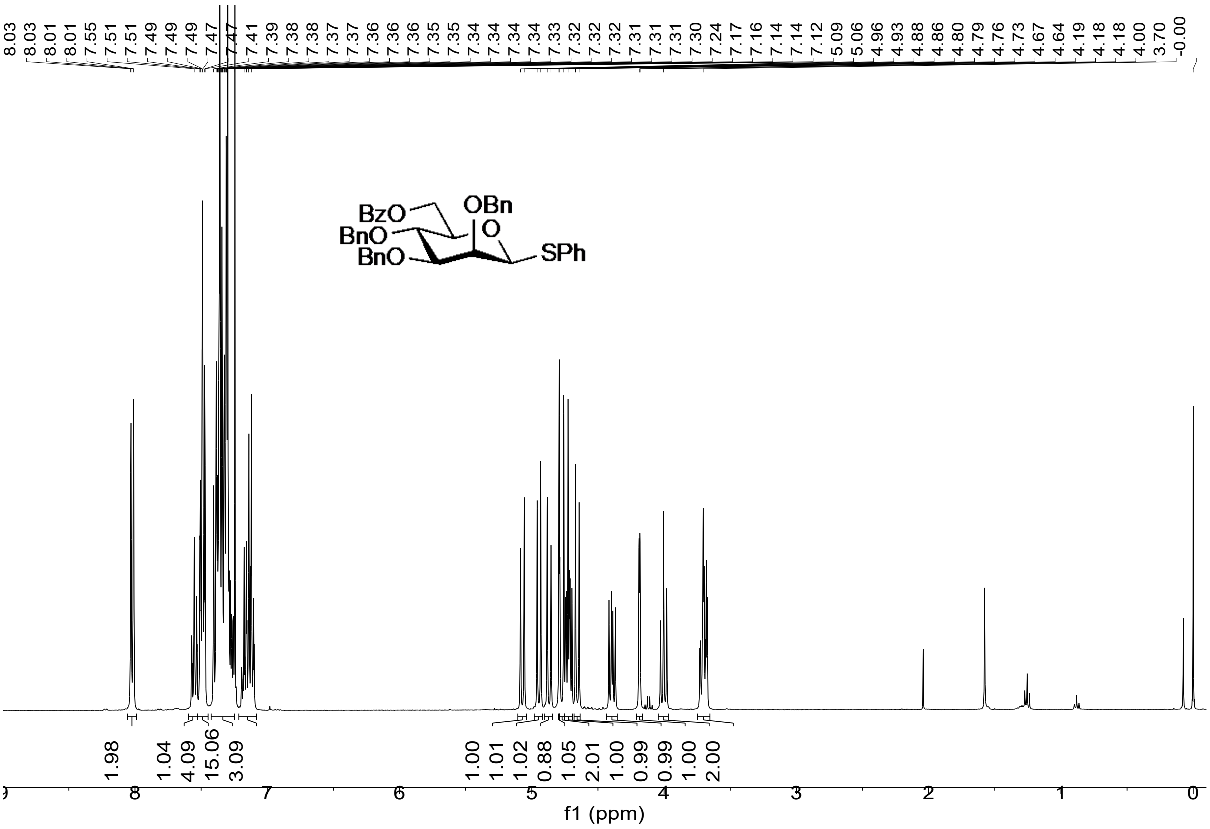

Supplementary Figure 81. ^{1}H NMR, 500 MHz, $CDCl_3$ of compound **15**

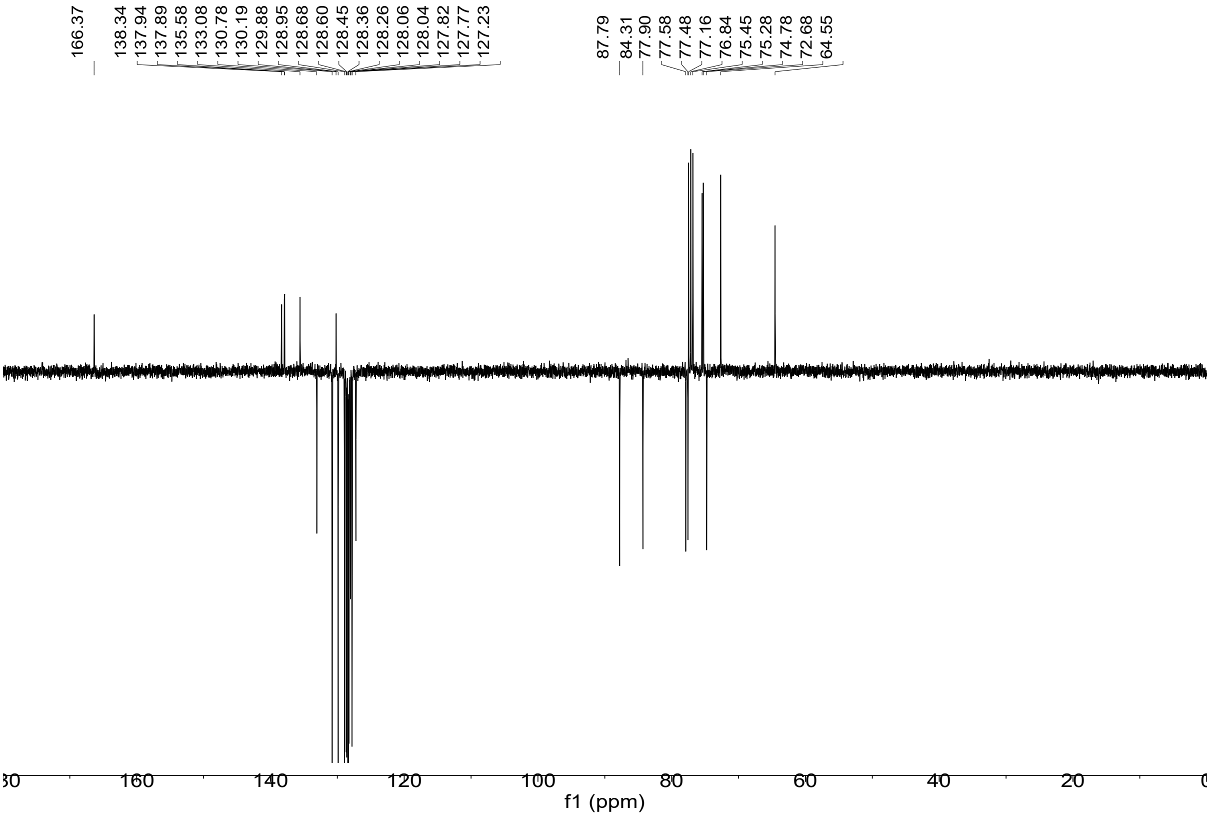

Supplementary Figure 82. ^{13}C NMR, 126 MHz, $CDCl_3$ of compound **15**

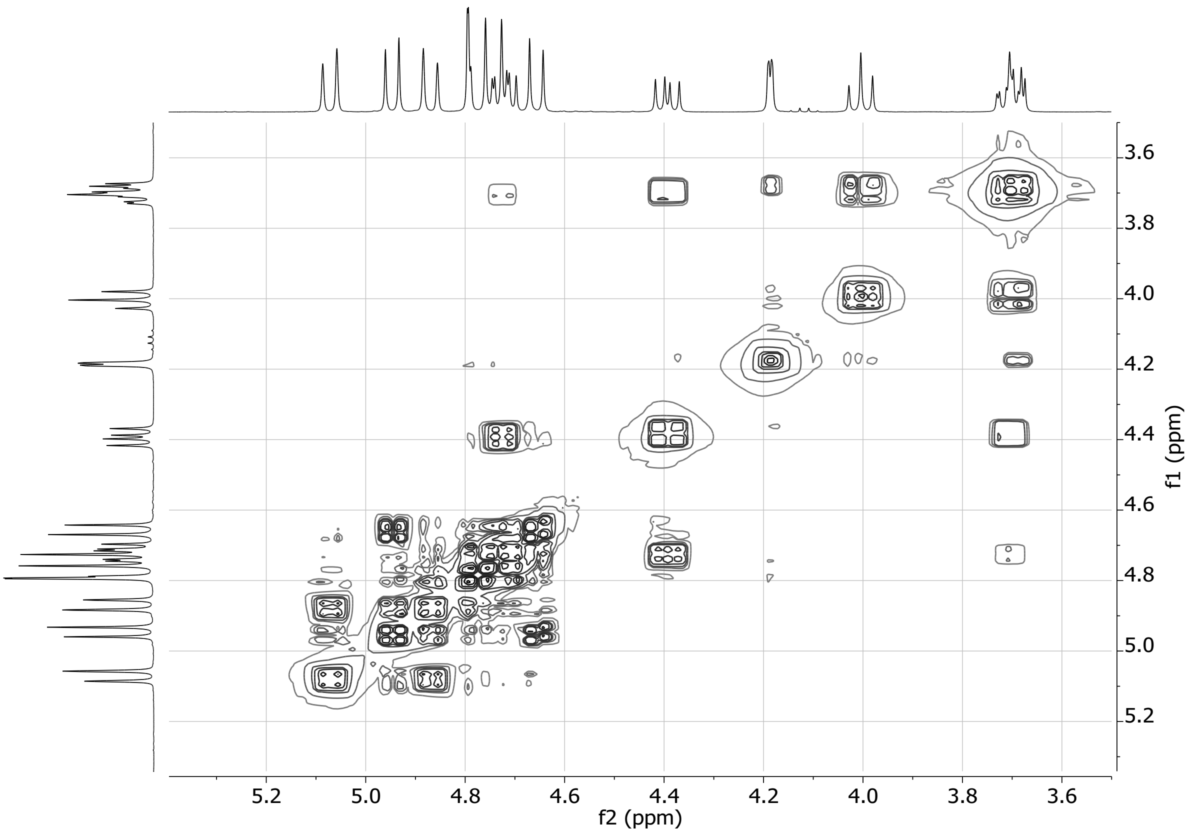

Supplementary Figure 83. HH-COSY NMR, $CDCl_3$ of compound **15**

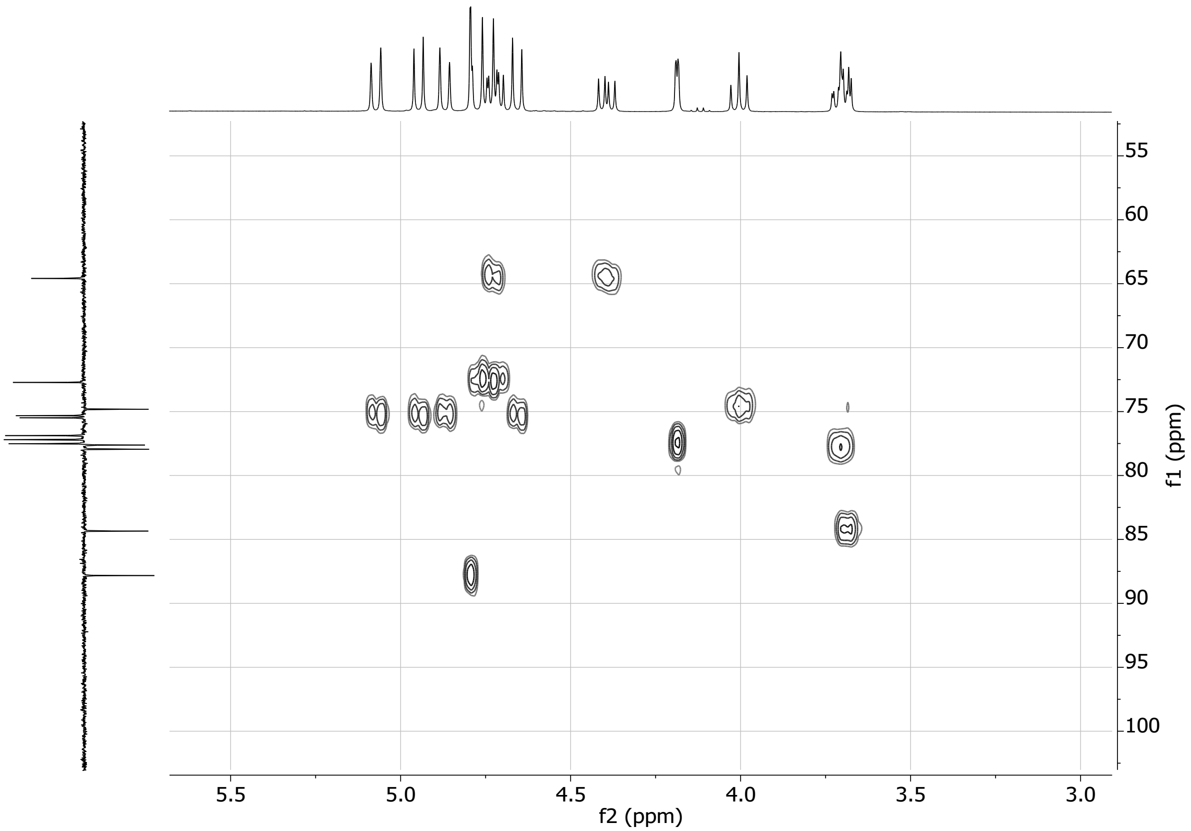

Supplementary Figure 84. HSQC NMR, $CDCl_3$ of compound **15**

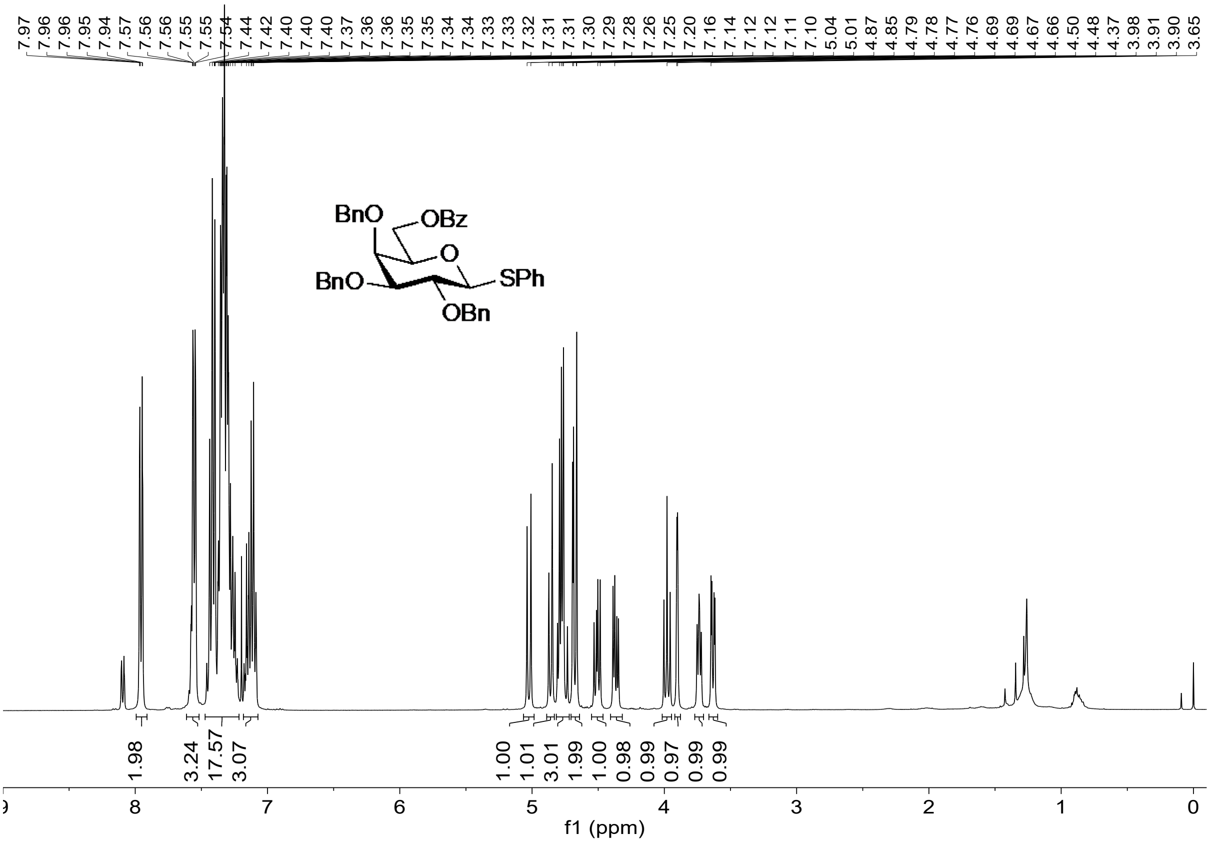

Supplementary Figure 85. ^{1}H NMR, 400 MHz, $CDCl_3$ of compound **18**

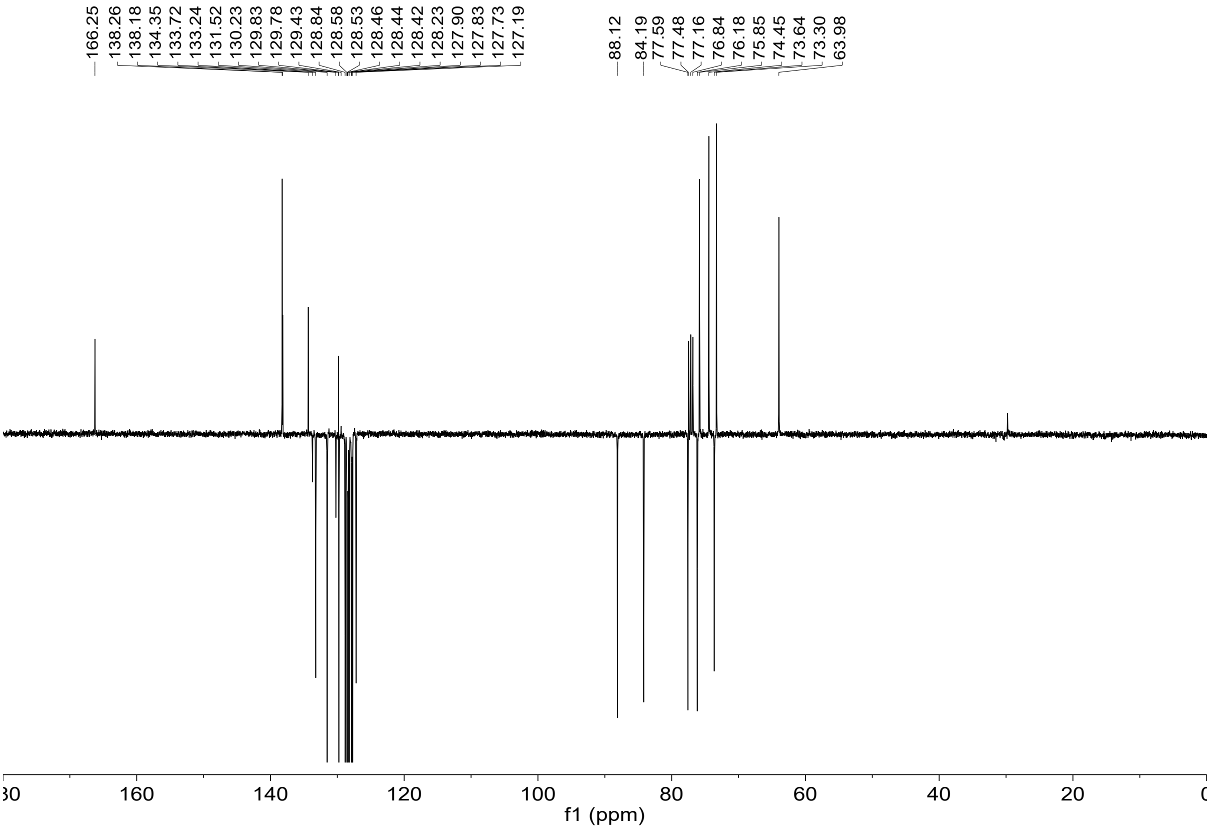

Supplementary Figure 86. ^{13}C NMR, 101 MHz, $CDCl_3$ of compound **18**

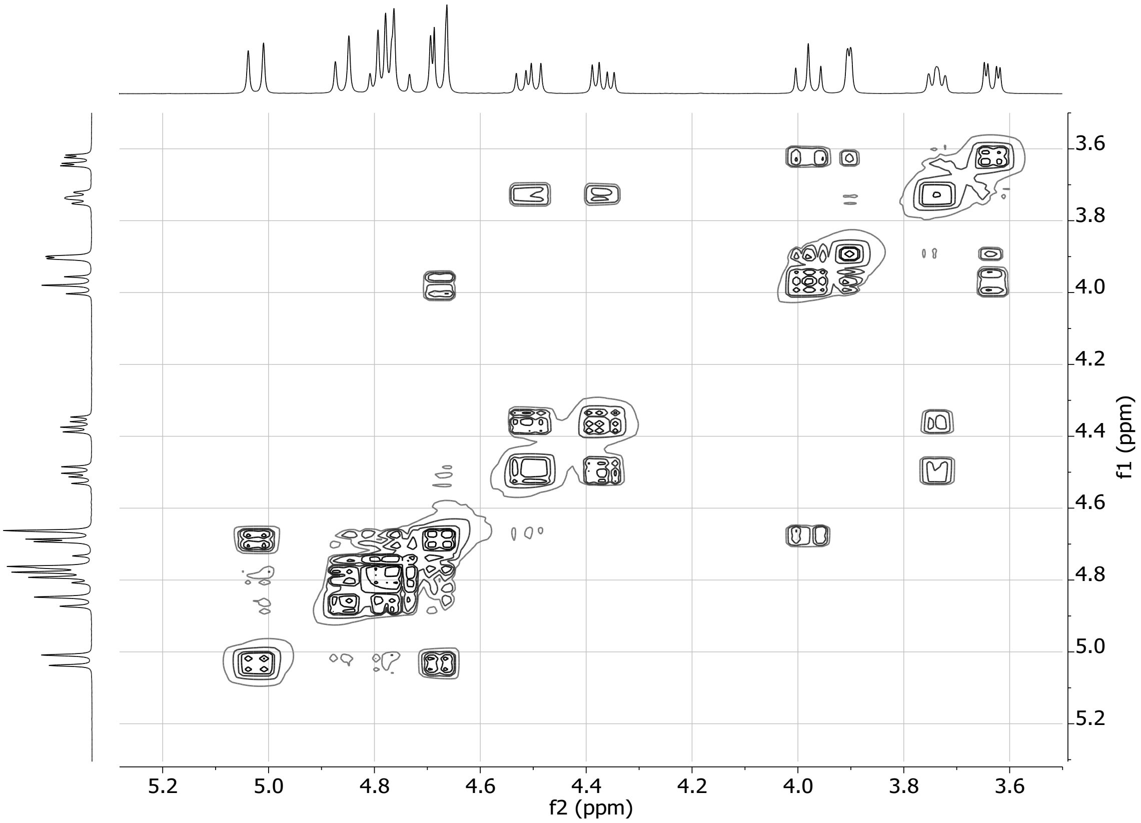

Supplementary Figure 87. HH-COSY NMR, $CDCl_3$ of compound **18**

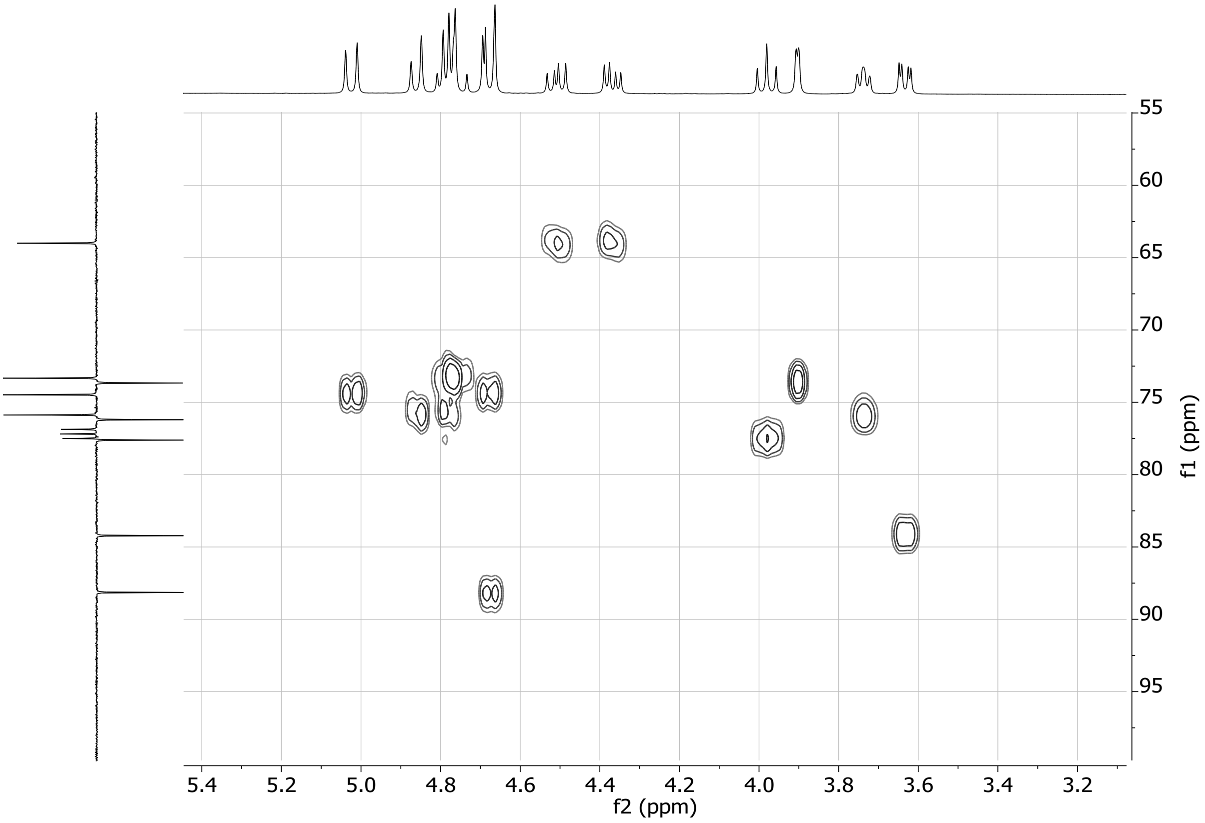

Supplementary Figure 88. HSQC NMR, $CDCl_3$ of compound **18**

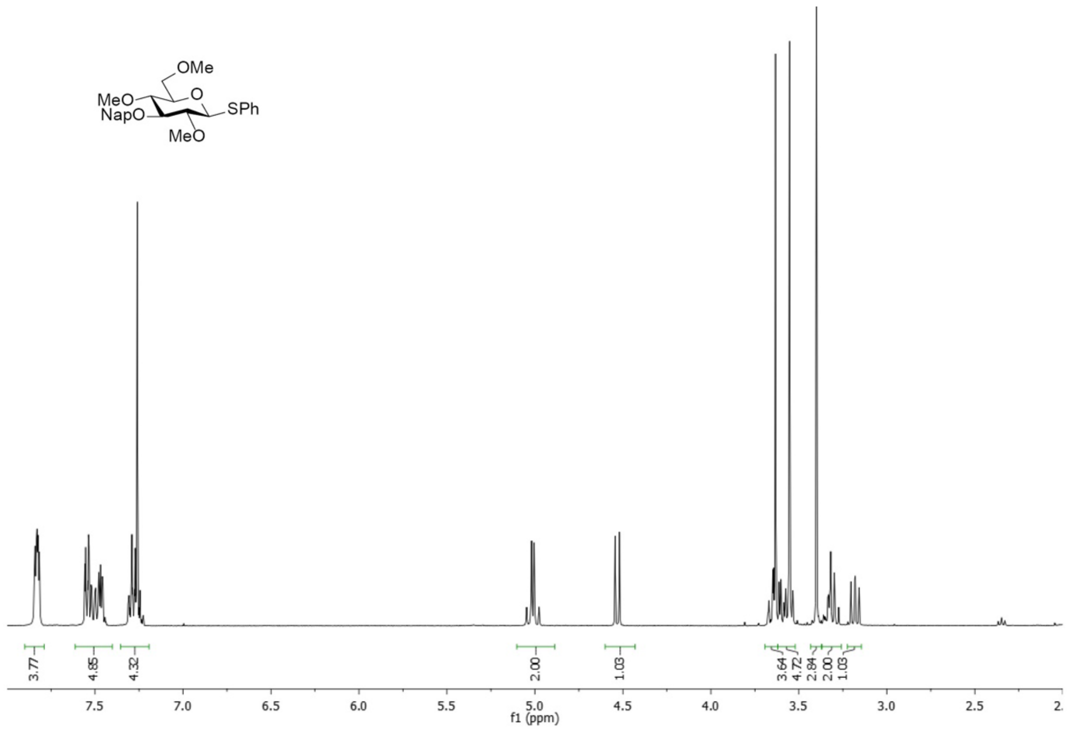

Supplementary Figure 89. ^{1}H NMR, 400 MHz, $CDCl_3$ of compound **S12**

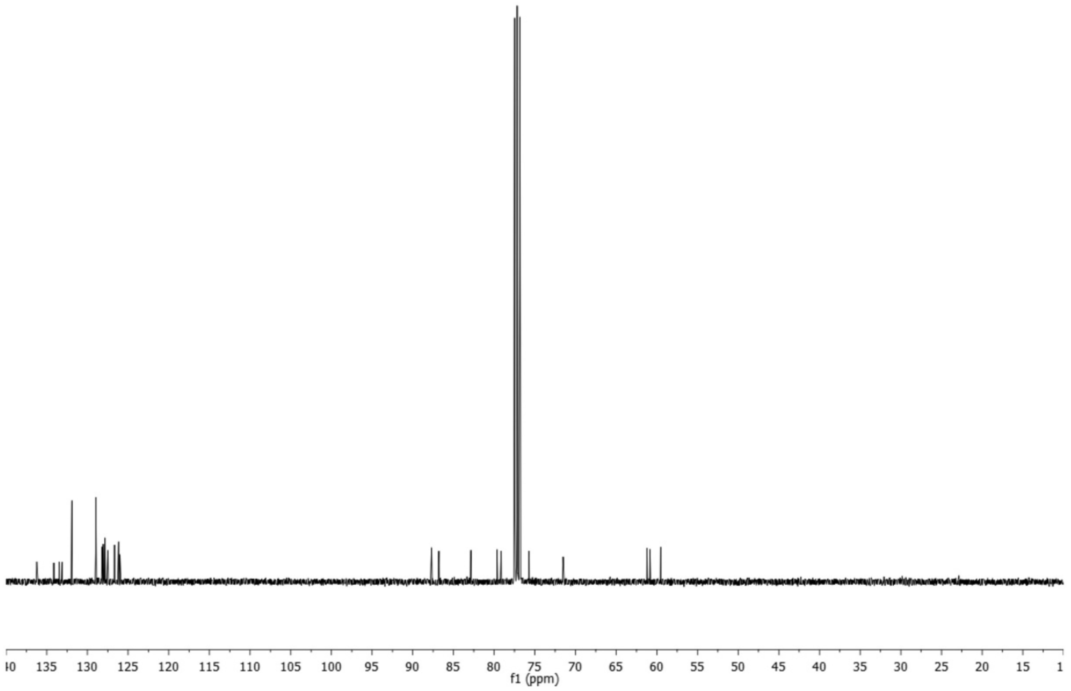

Supplementary Figure 90. ^{1}H NMR, 500 MHz, $CDCl_3$ of compound **13**

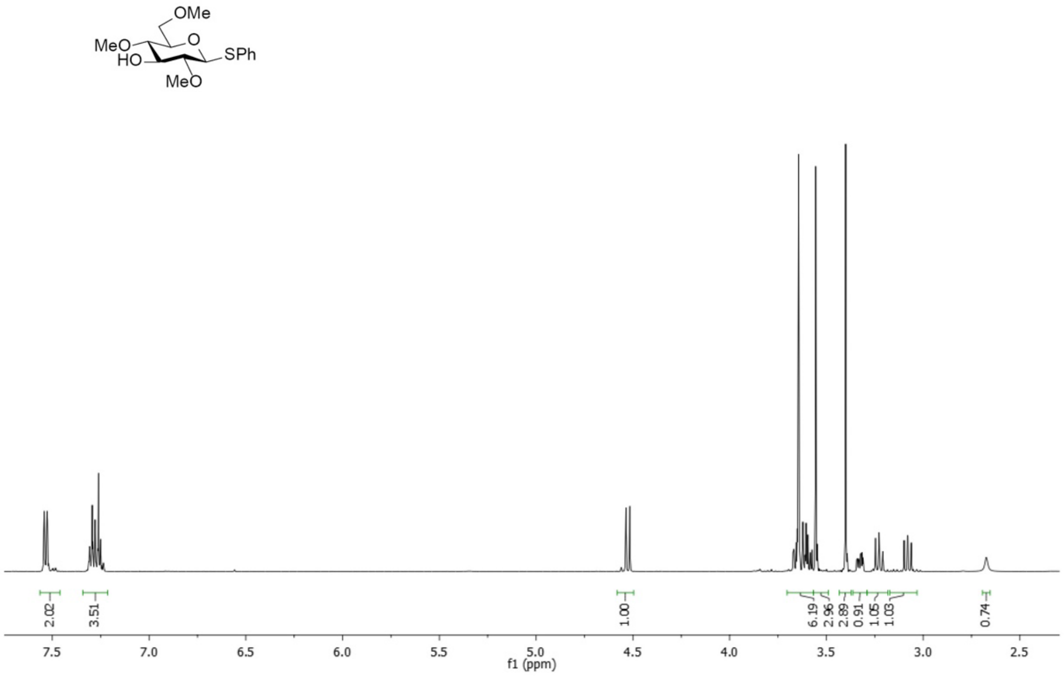

Supplementary Figure 91. ^{13}C NMR, 126 MHz, $CDCl_3$ of compound **13**

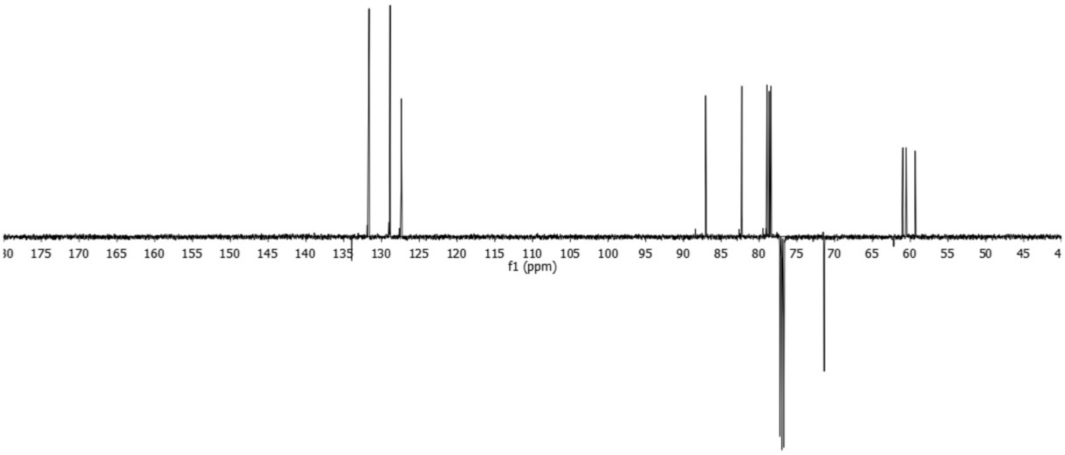

Supplementary Figure 92. ^{13}C NMR, 101 MHz, $CDCl_3$ of compound S12

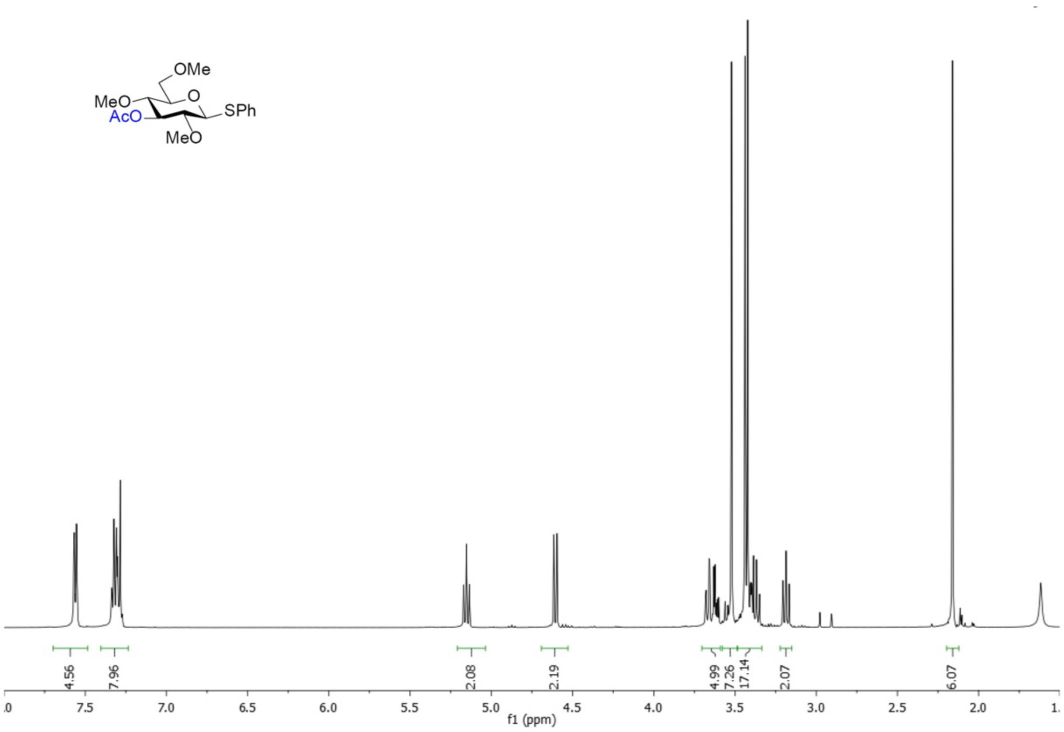

Supplementary Figure 93. ^{1}H NMR, 500 MHz, $CDCl_3$ of compound **1**

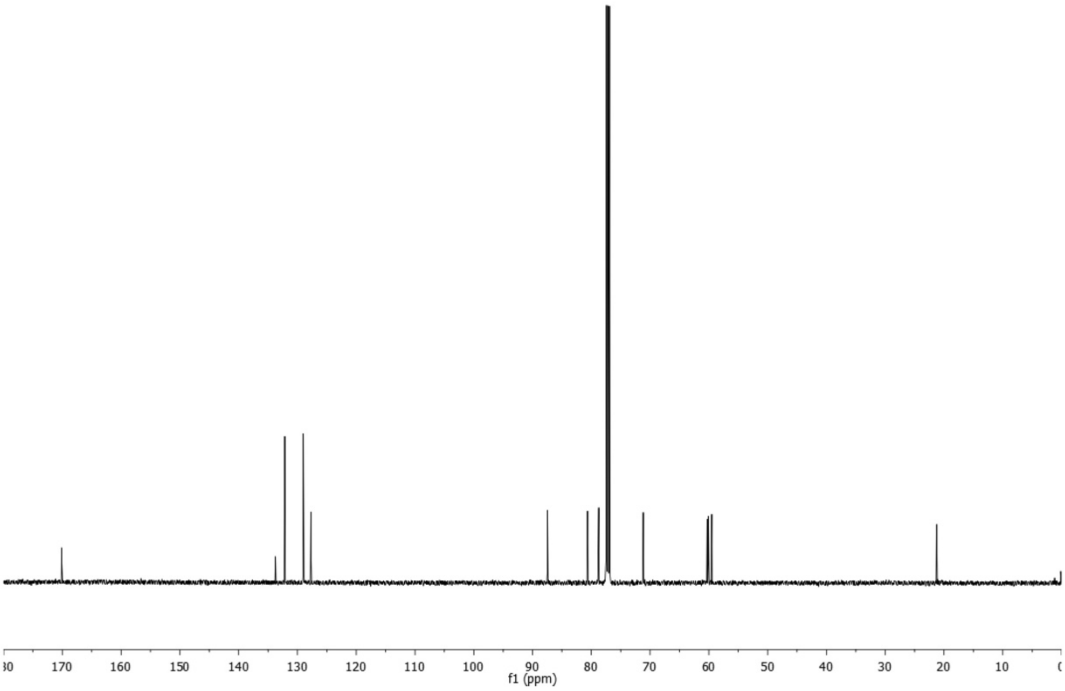

Supplementary Figure 94. ^{13}C NMR, 126 MHz, $CDCl_3$ of compound **1**

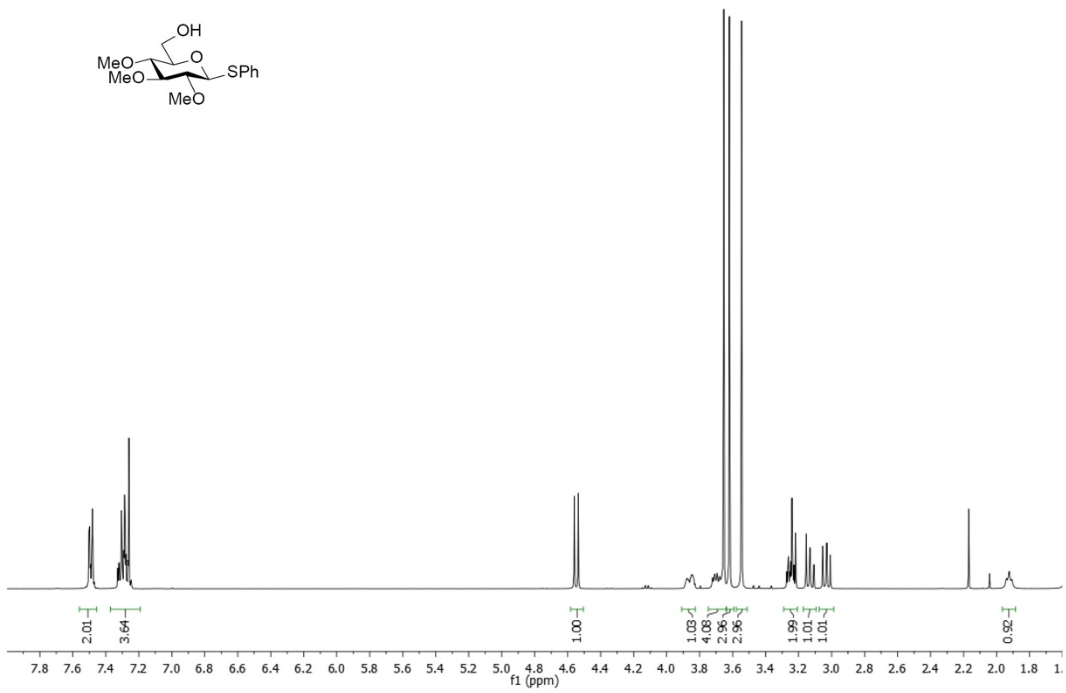

Supplementary Figure 95. ^{1}H NMR, 400 MHz, $CDCl_3$ of compound **S15**

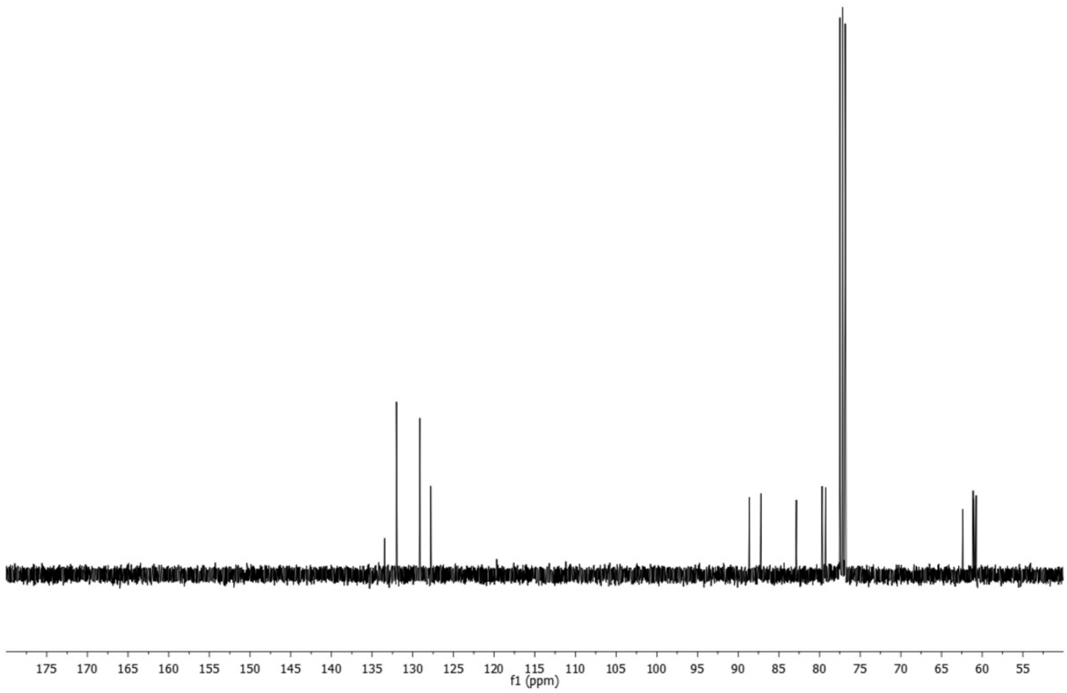

Supplementary Figure 96. ^{13}C NMR, 101 MHz, $CDCl_3$ of compound **S15**

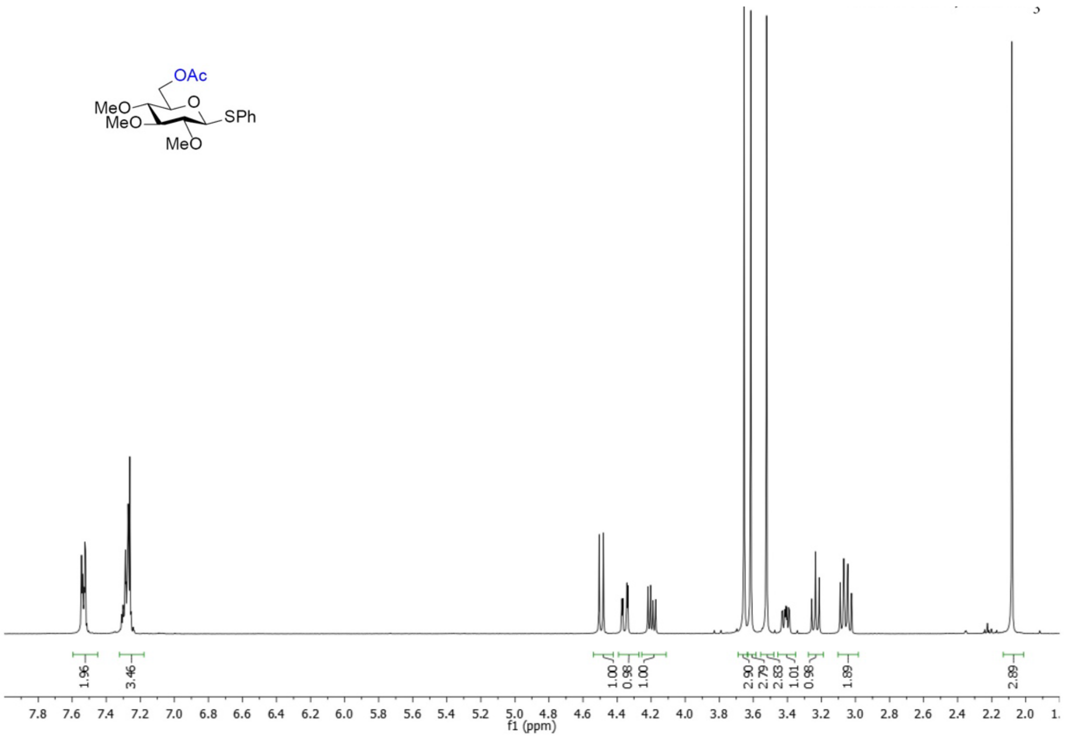

Supplementary Figure 97. ^{1}H NMR, 400 MHz, $CDCl_3$ of compound **3**

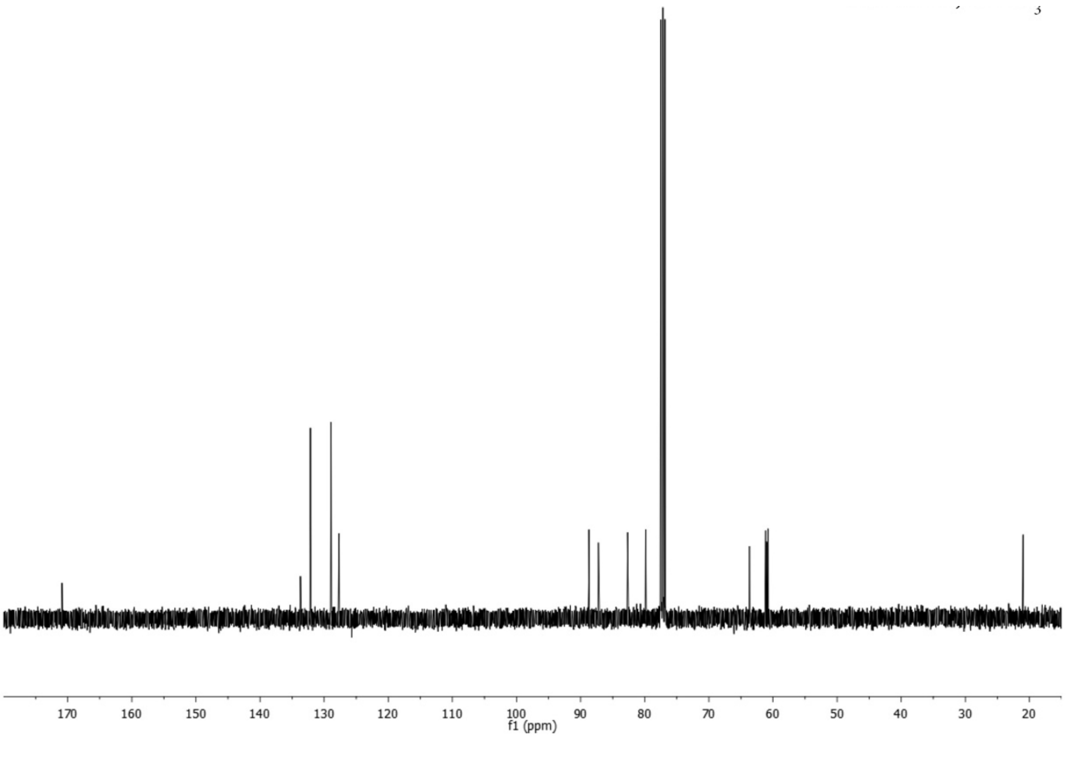

Supplementary Figure 98. ^{13}C NMR, 101 MHz, $CDCl_3$ of compound **3**

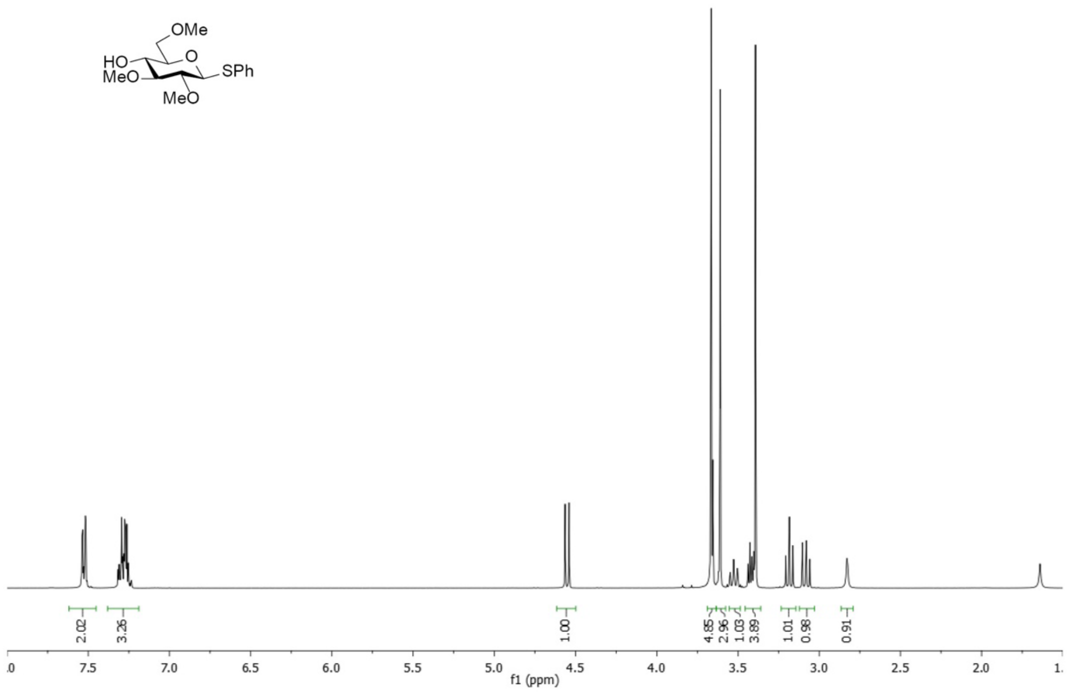

Supplementary Figure 99. ^{1}H NMR, 400 MHz, $CDCl_3$ of compound **S14**

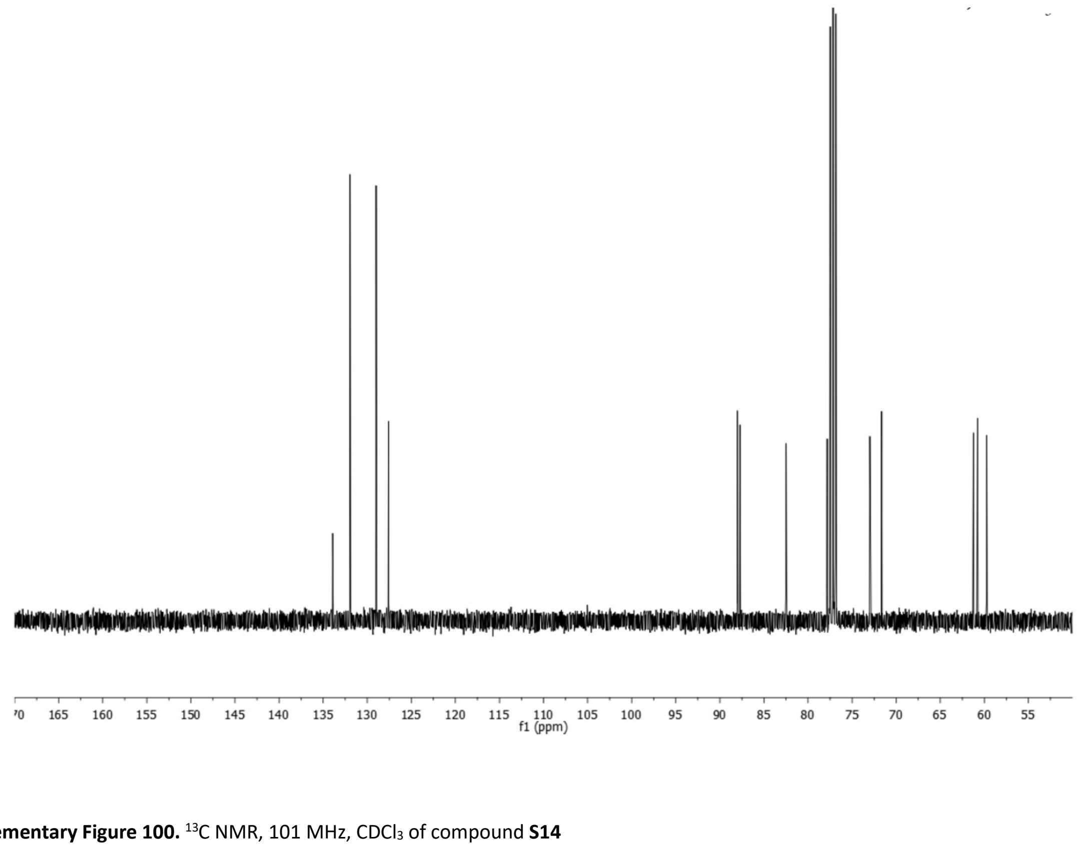

Supplementary Figure 100. ^{13}C NMR, 101 MHz, $CDCl_3$ of compound **S14**

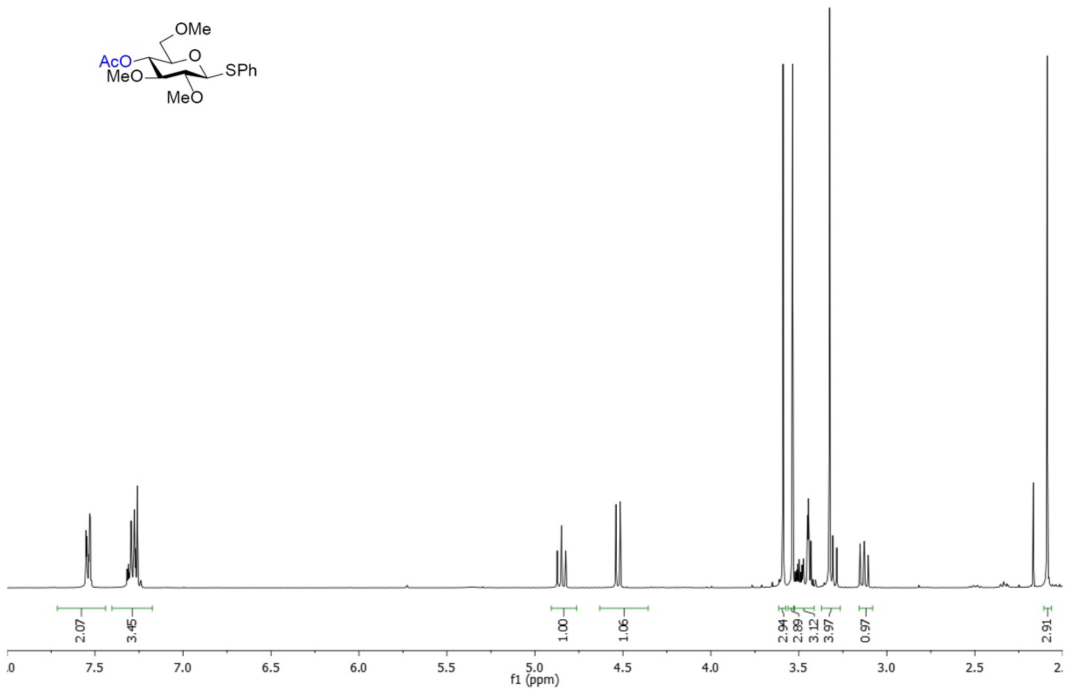

Supplementary Figure 101. ^{1}H NMR, 400 MHz, $CDCl_3$ of compound **2**

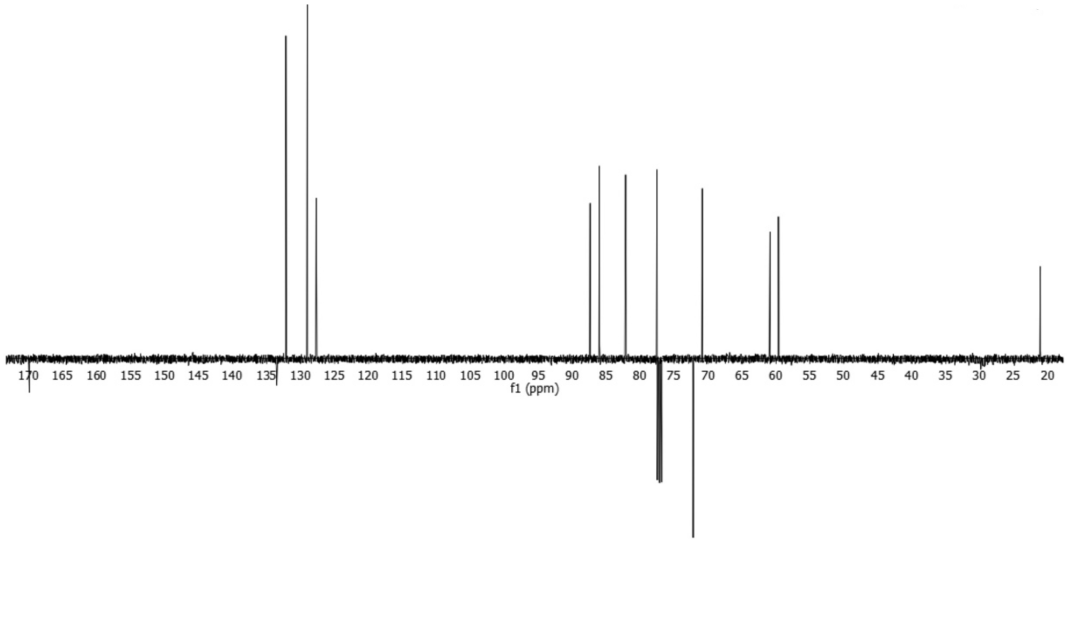

Supplementary Figure 102. ^{13}C NMR, 101 MHz, $CDCl_3$ of compound **2**

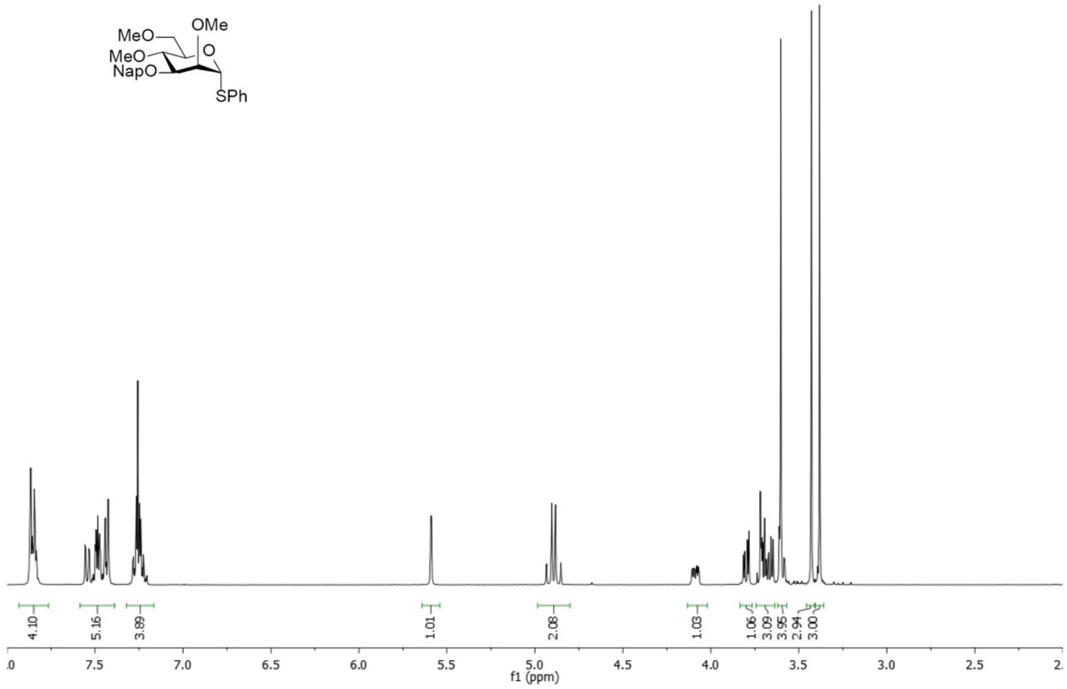

Supplementary Figure 103. ^{1}H NMR, 400 MHz, $CDCl_3$ of compound **S16**

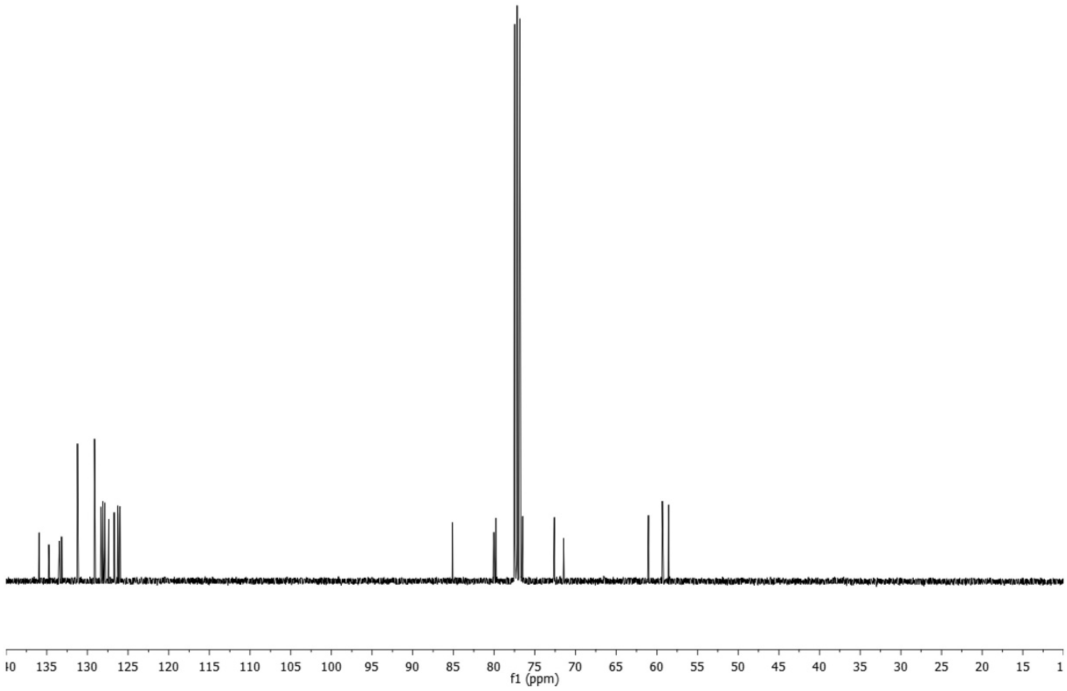

Supplementary Figure 104. ^{13}C NMR, 101 MHz, $CDCl_3$ of compound **S16**

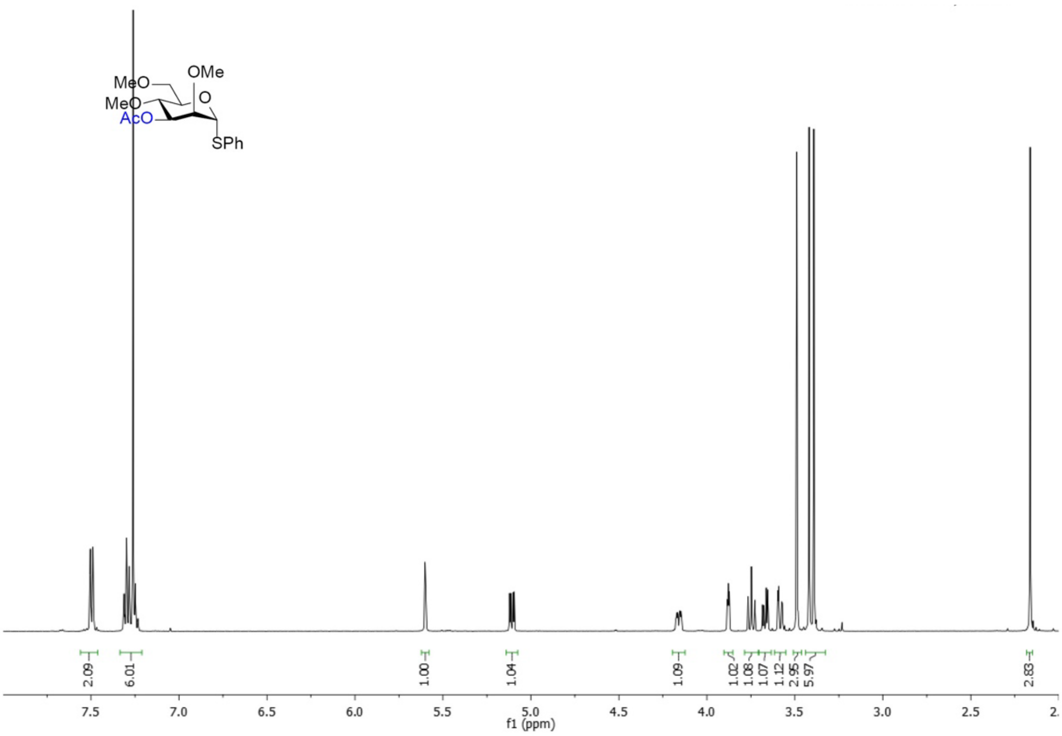

Supplementary Figure 105. ^{1}H NMR, 500 MHz, $CDCl_3$ of compound **4**

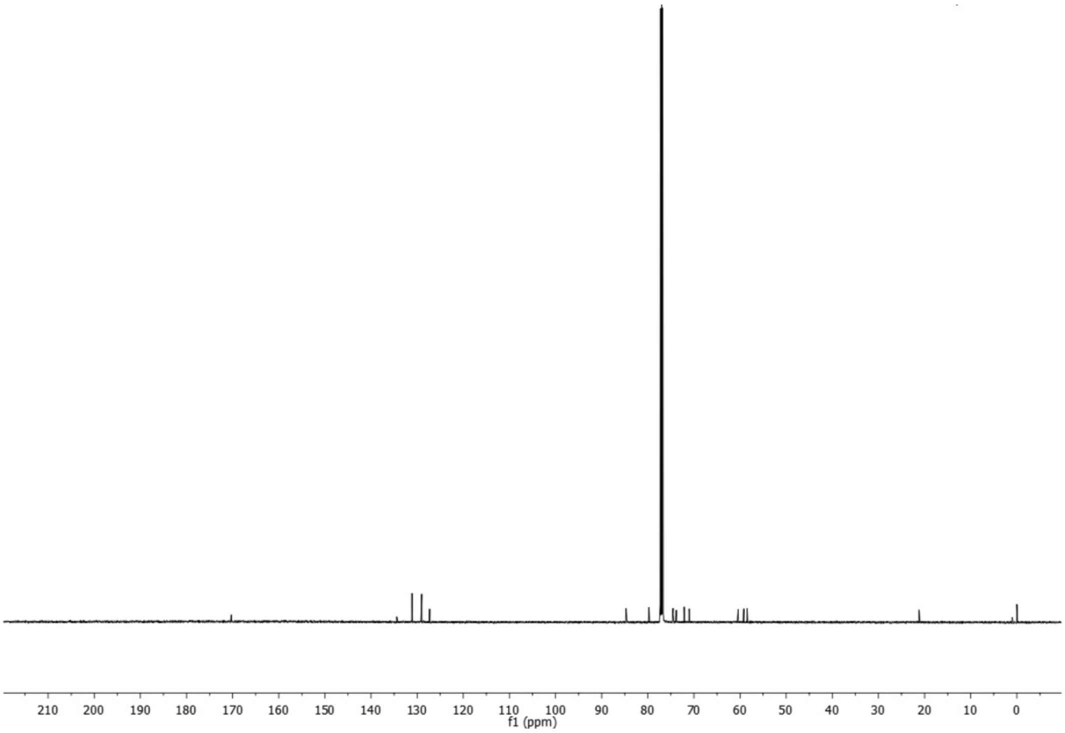

Supplementary Figure 106. ^{13}C NMR, 126 MHz, $CDCl_3$ of compound **4**

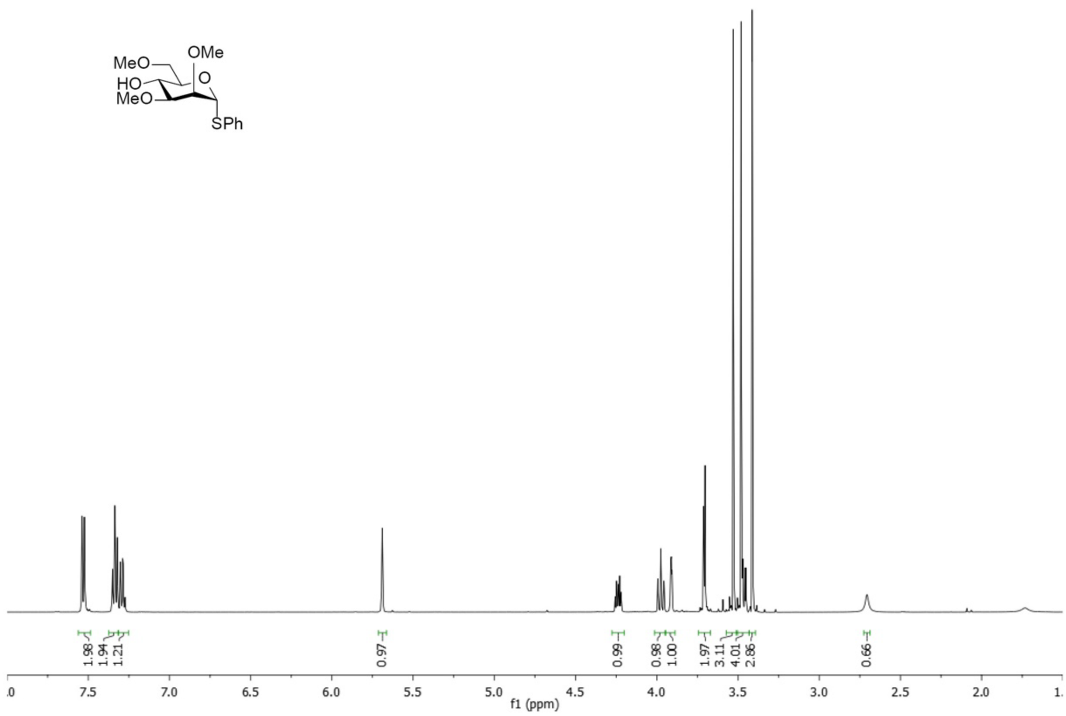

Supplementary Figure 107. ^{1}H NMR, 500 MHz, $CDCl_3$ of compound **S18**

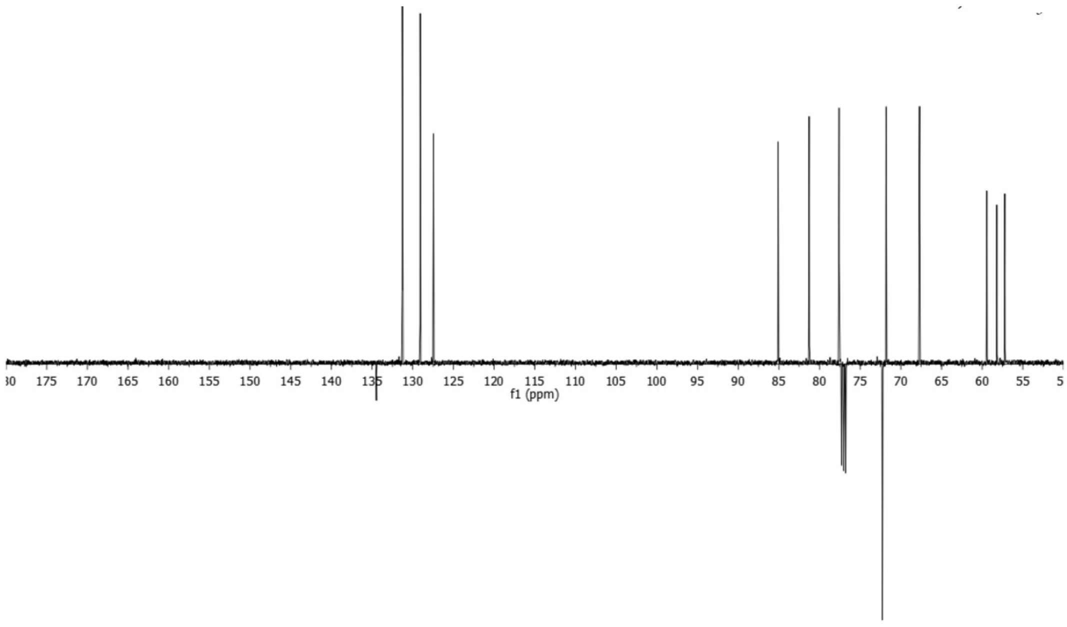

Supplementary Figure 108. ^{13}C NMR, 126 MHz, $CDCl_3$ of compound **S18**

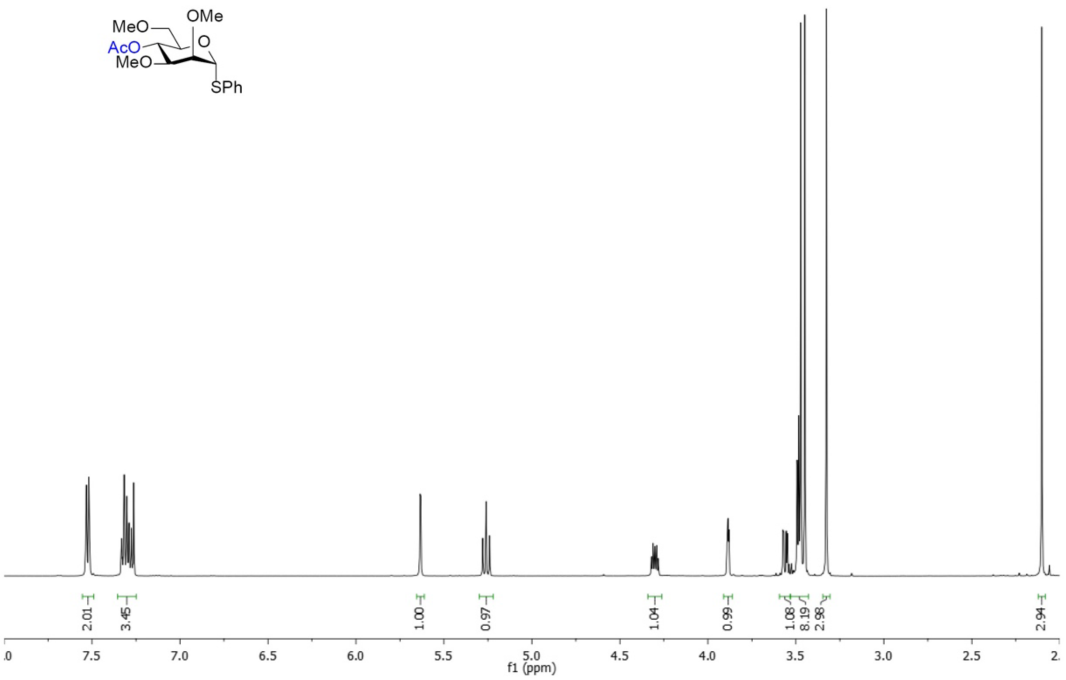

Supplementary Figure 109. ^{1}H NMR, 500 MHz, $CDCl_3$ of compound **5**

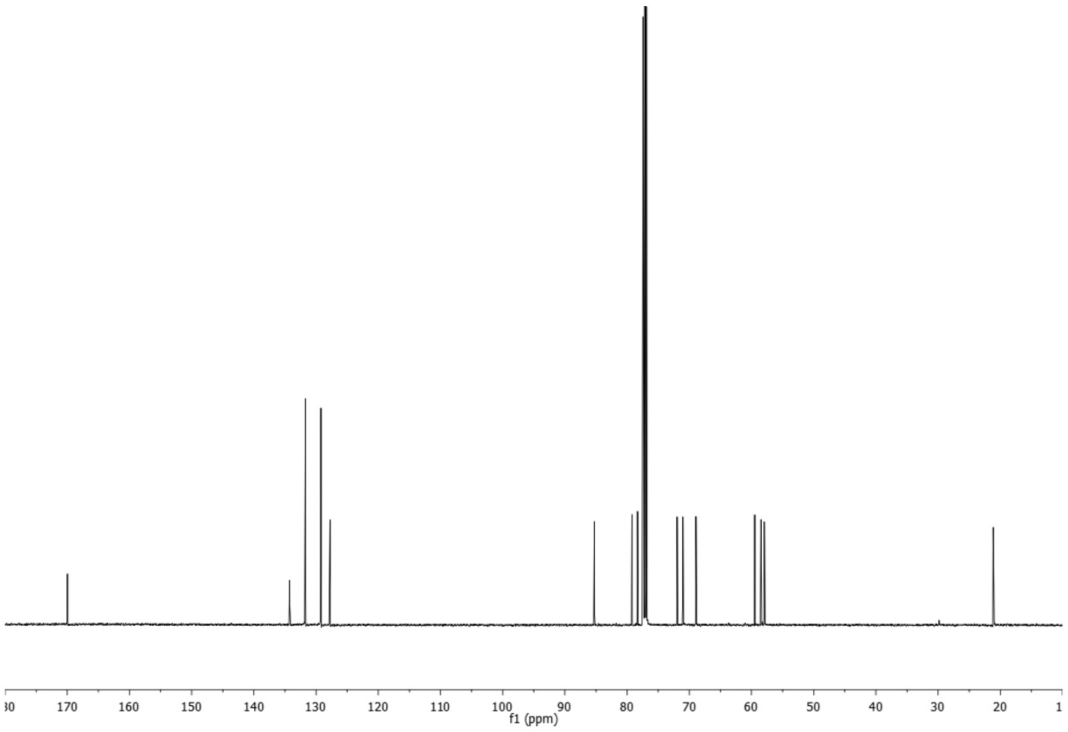

Supplementary Figure 110. ^{13}C NMR, 126 MHz, $CDCl_3$ of compound **5**

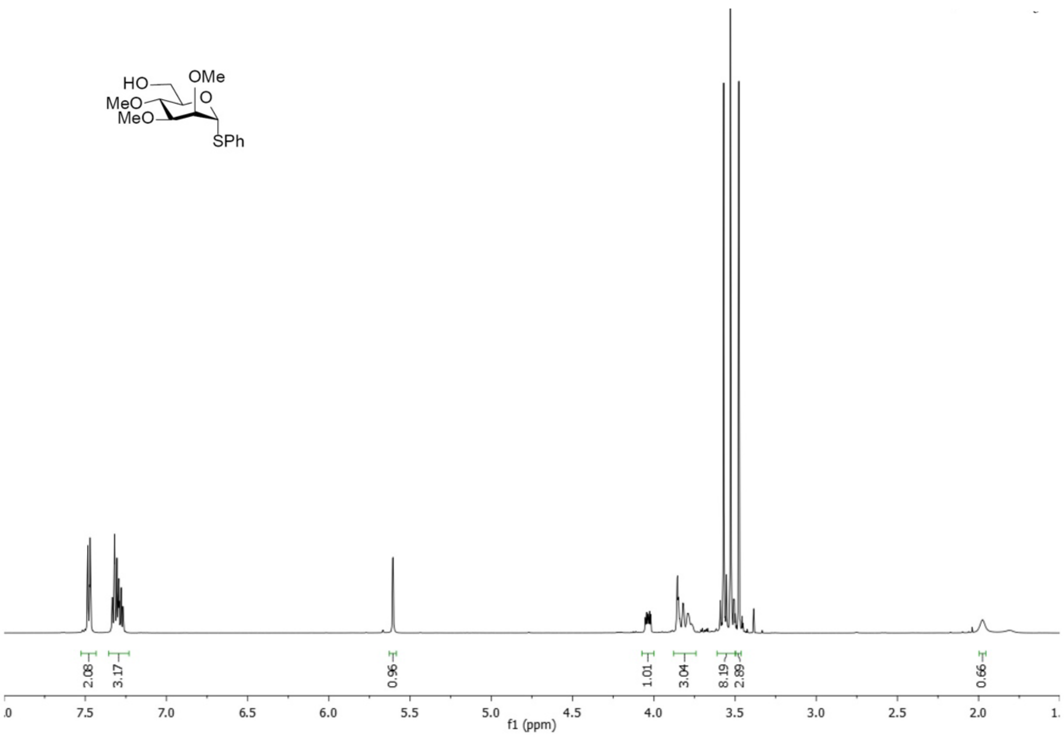

Supplementary Figure 111. ^{1}H NMR, 500 MHz, $CDCl_3$ of compound **S19**

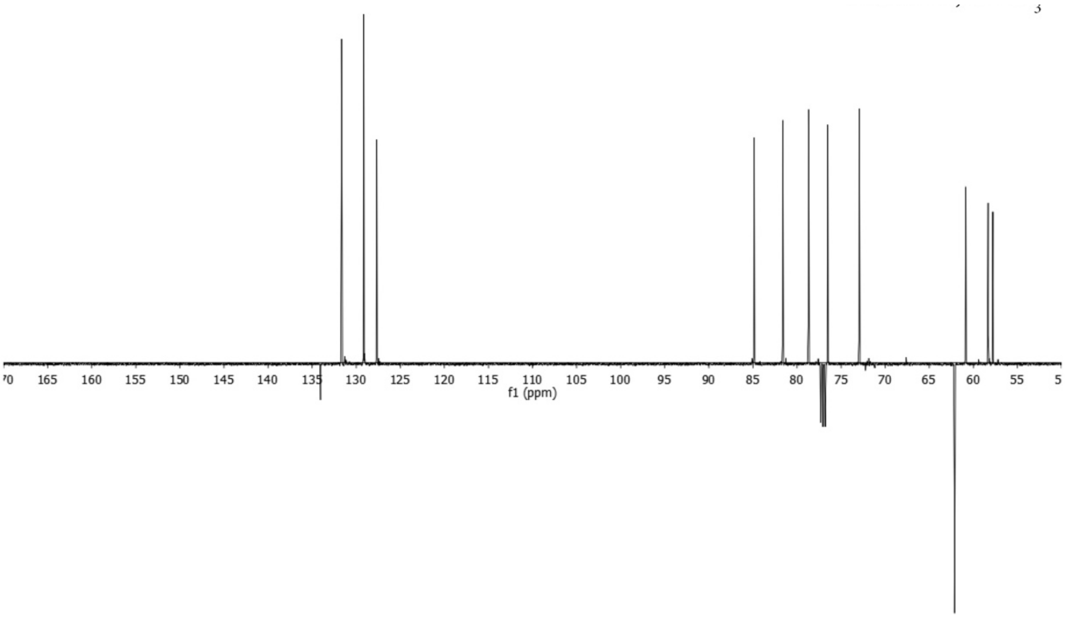

Supplementary Figure 112. ^{13}C NMR, 126 MHz, $CDCl_3$ of compound **S19**

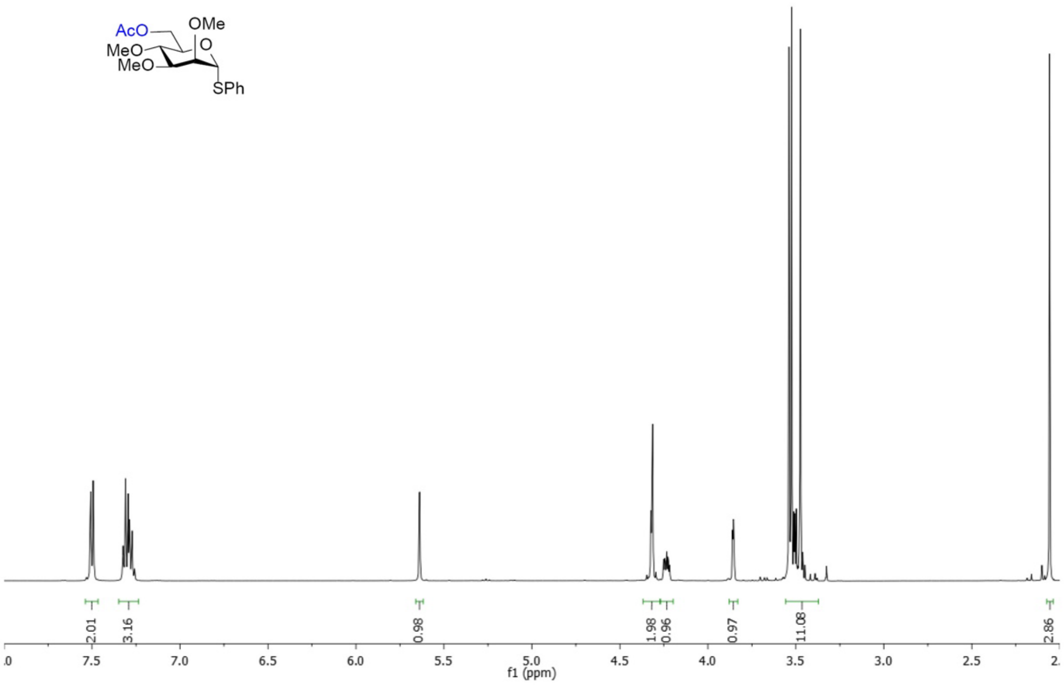

Supplementary Figure 113. ^{1}H NMR, 500 MHz, $CDCl_3$ of compound **6**

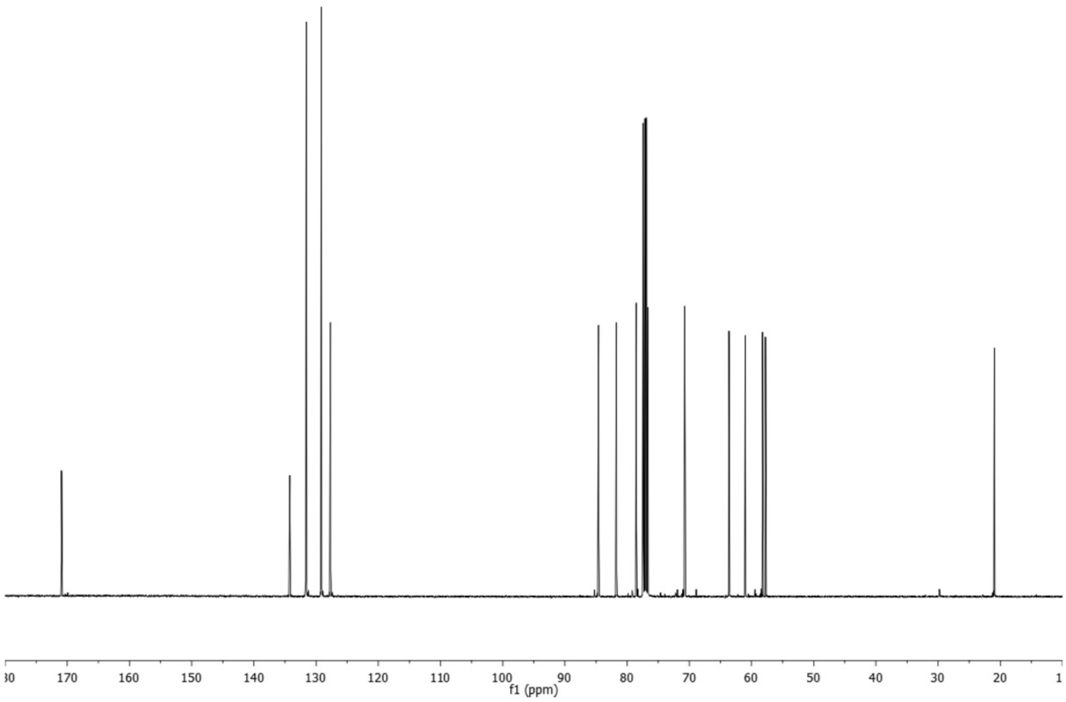

Supplementary Figure 114. ^{13}C NMR, 126 MHz, $CDCl_3$ of compound **6**

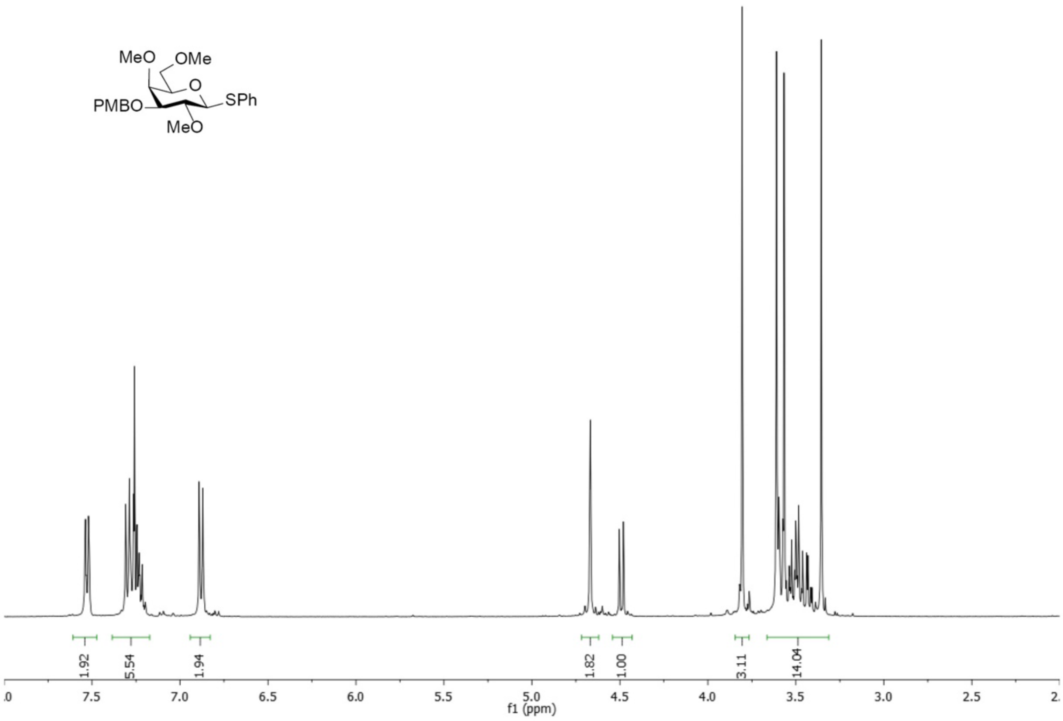

Supplementary Figure 115. ^{1}H NMR, 400 MHz, $CDCl_3$ of compound **S20**

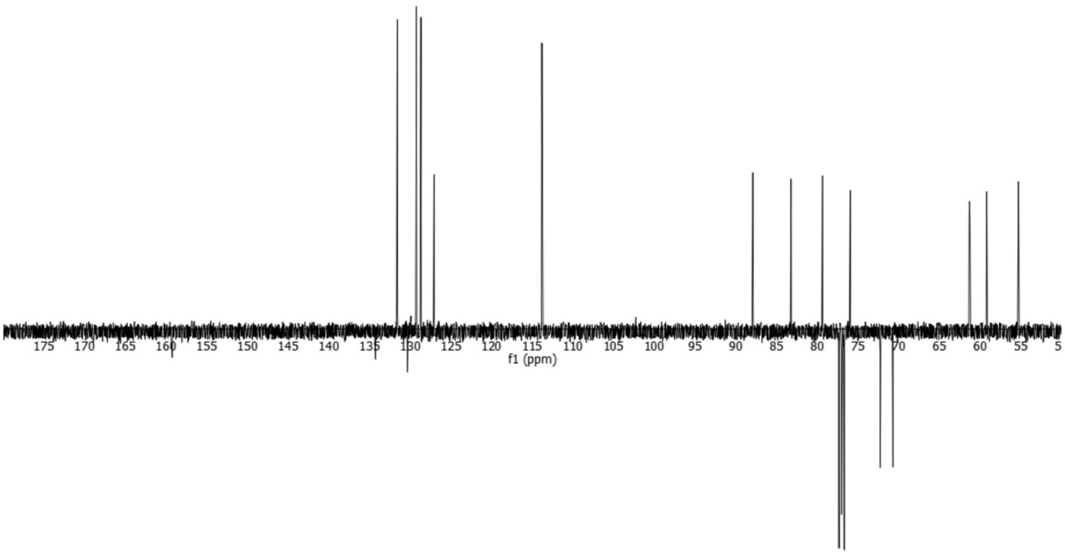

Supplementary Figure 116. ^{13}C NMR, 101 MHz, $CDCl_3$ of compound **S20**

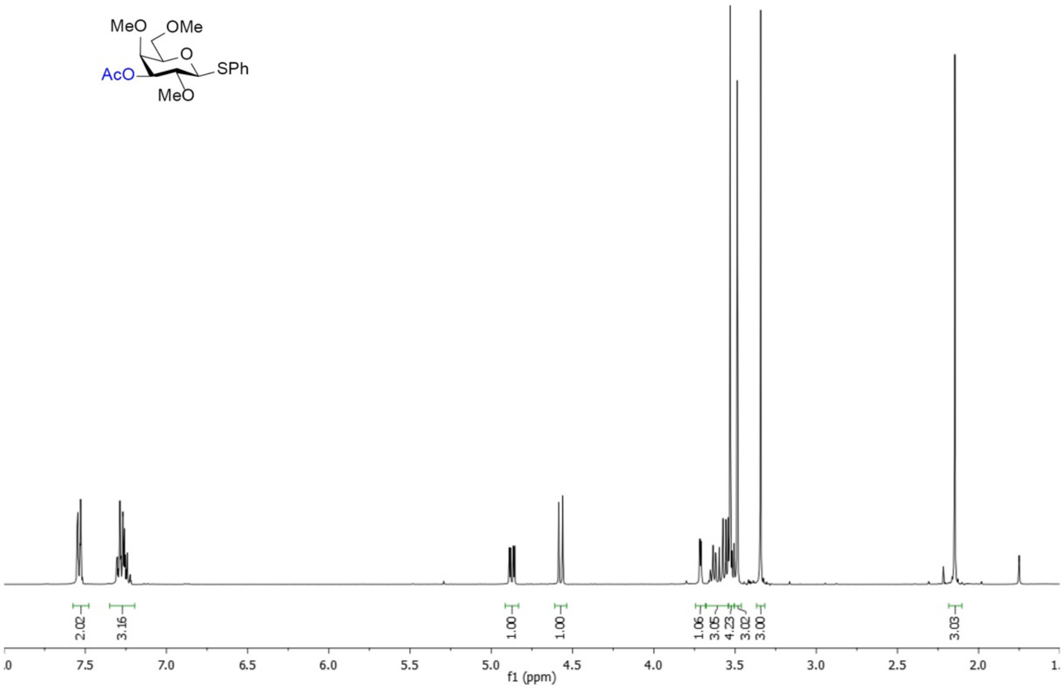

Supplementary Figure 117. ^{1}H NMR, 400 MHz, $CDCl_3$ of compound **7**

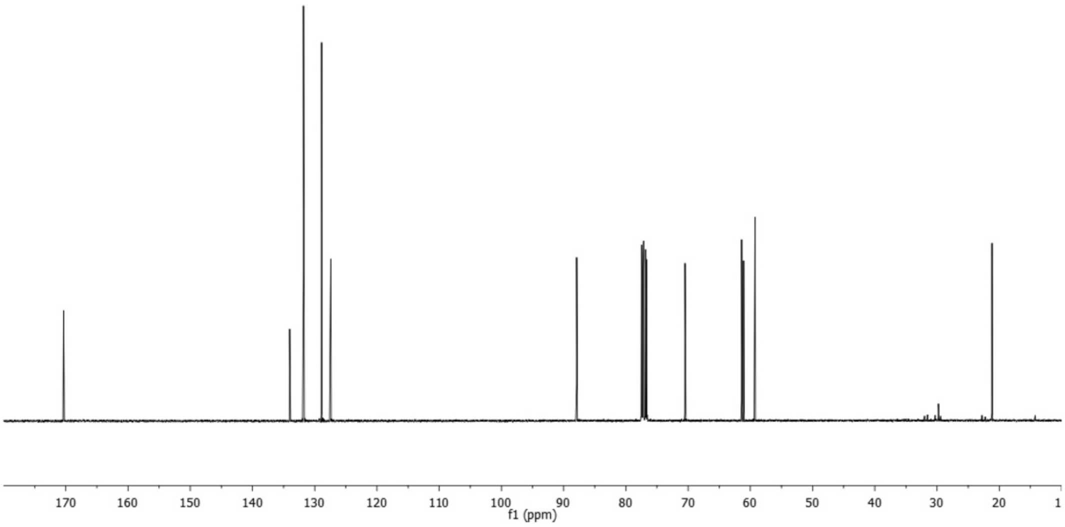

Supplementary Figure 118. ^{13}C NMR, 101 MHz, $CDCl_3$ of compound **7**

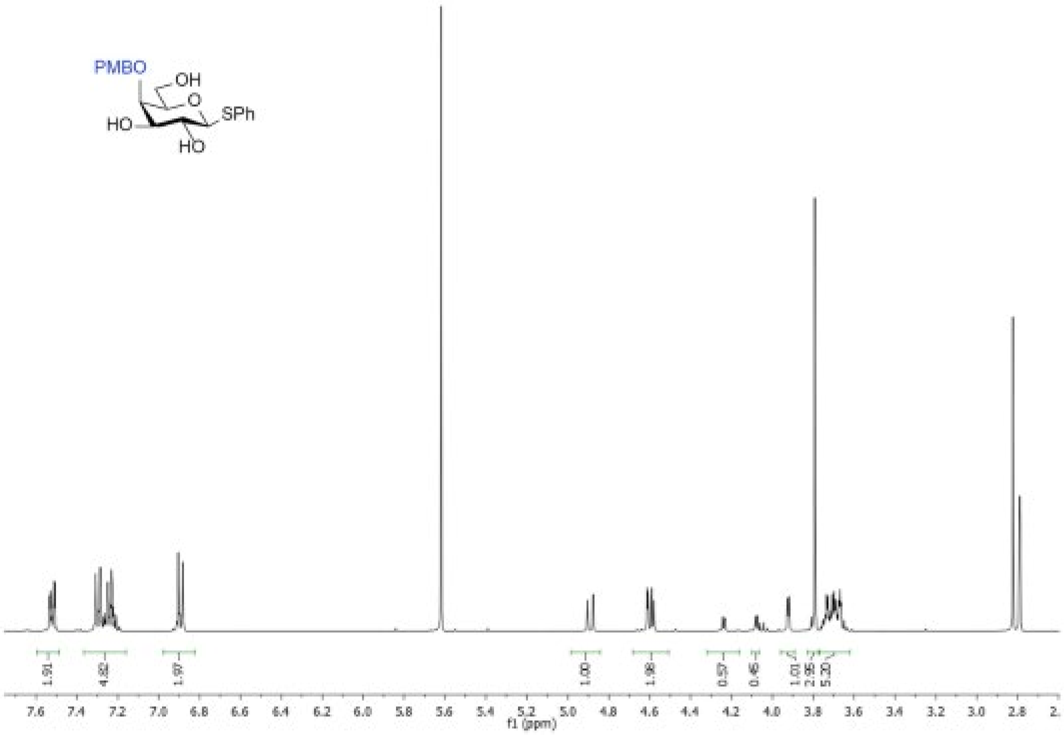

Supplementary Figure 119. ^{1}H NMR, 500 MHz, CD_3COCD_3 of compound **S22**

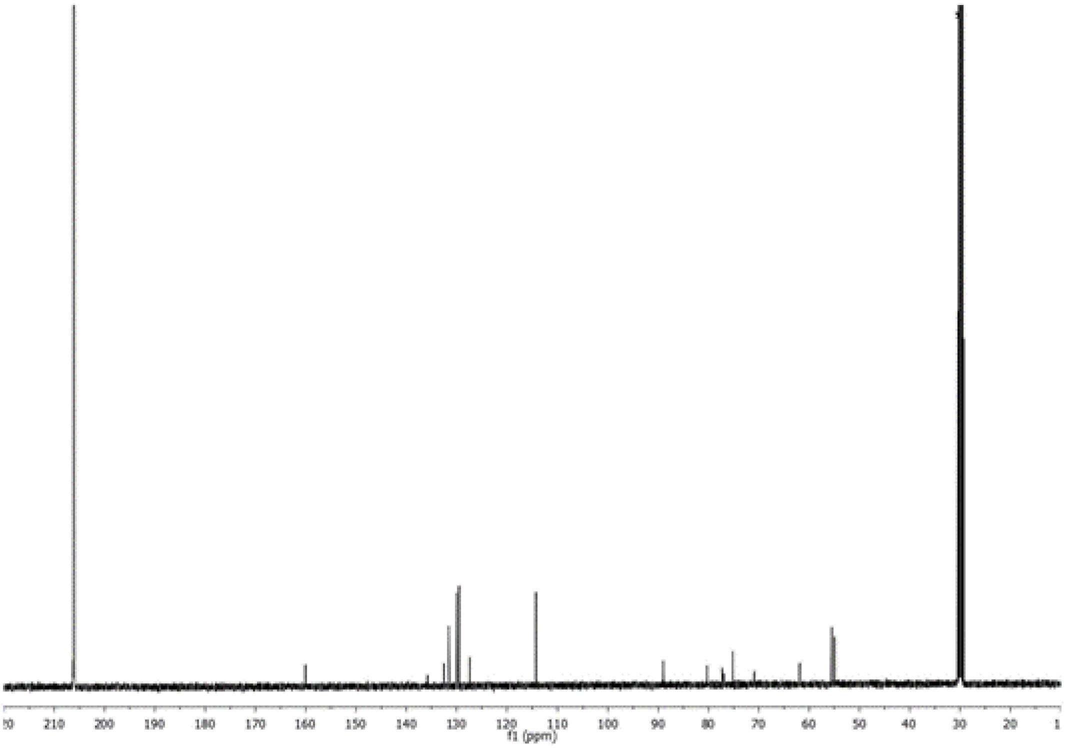

Supplementary Figure 120. ^{13}C NMR, 101 MHz, CD_3COCD_3 of compound **S22**

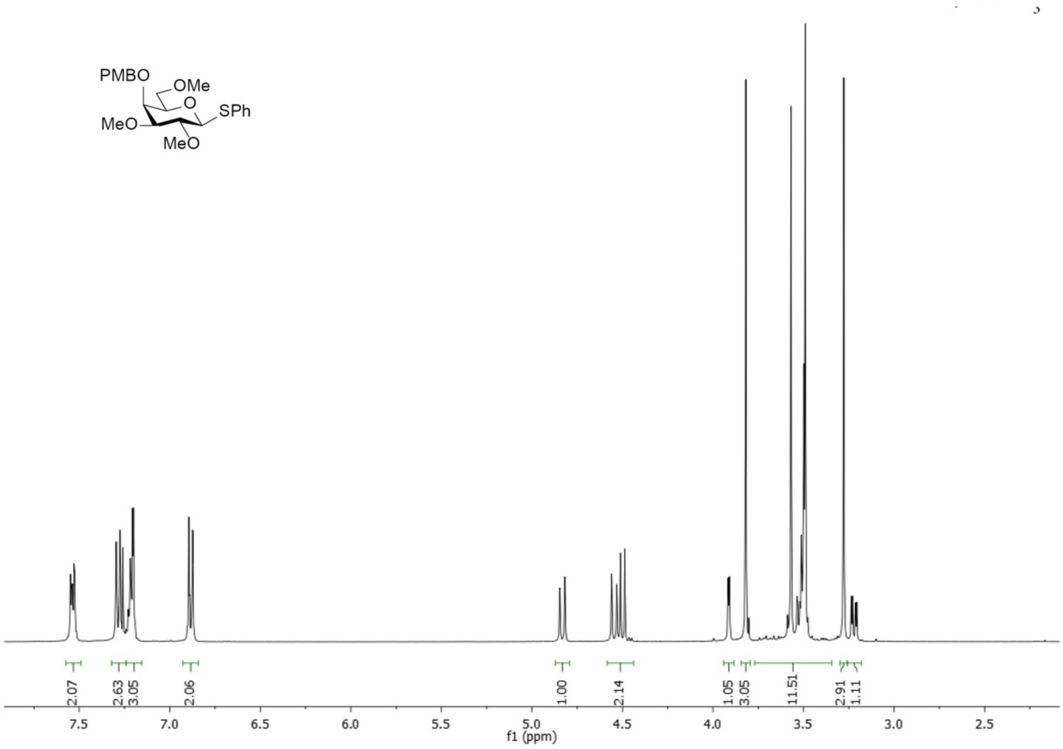

Supplementary Figure 121. ^{1}H NMR, 400 MHz, $CDCl_3$ of compound **S23**

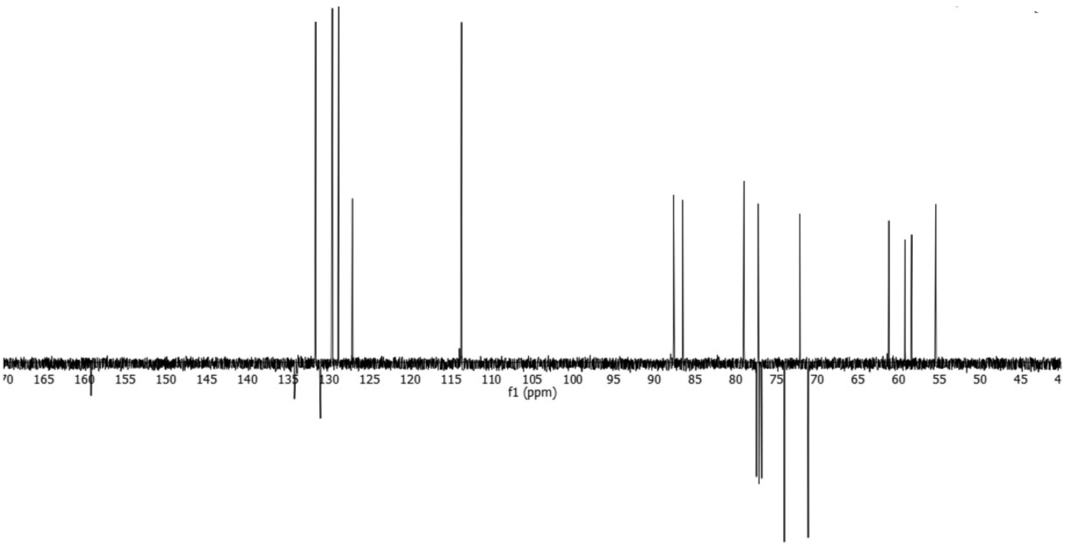

Supplementary Figure 122. ^{13}C NMR, 101 MHz, $CDCl_3$ of compound **S23**

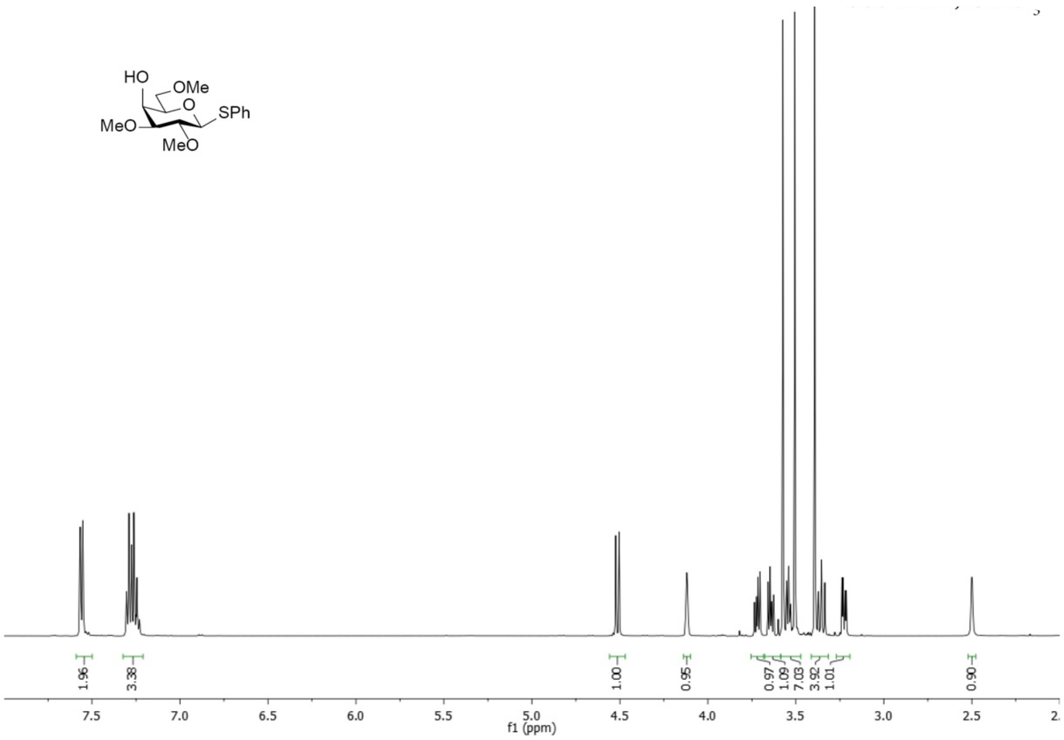

Supplementary Figure 123. ^{1}H NMR, 500 MHz, $CDCl_3$ of compound **S24**

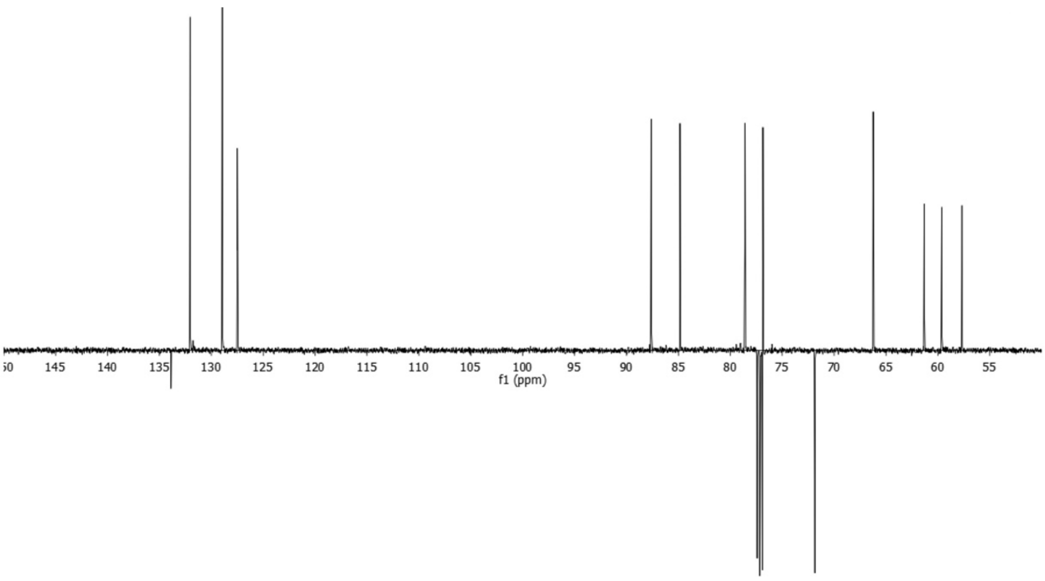

Supplementary Figure 124. ^{13}C NMR, 126 MHz, $CDCl_3$ of compound **S24**

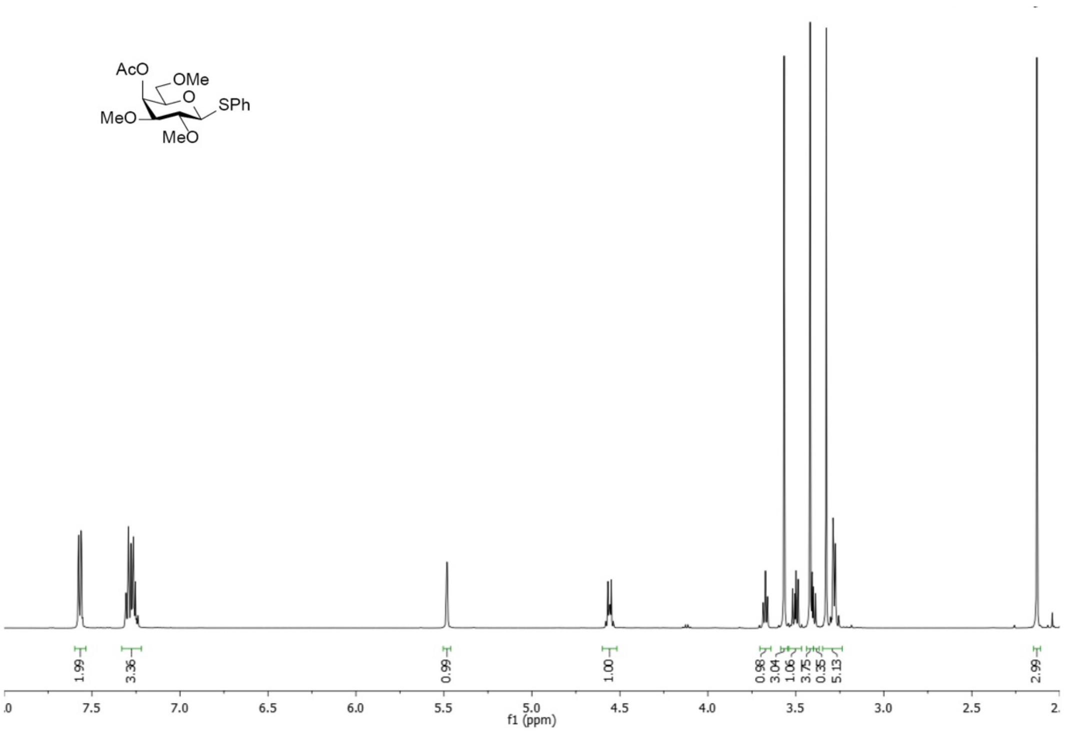

Supplementary Figure 125. ^{1}H NMR, 500 MHz, $CDCl_3$ of compound **8**

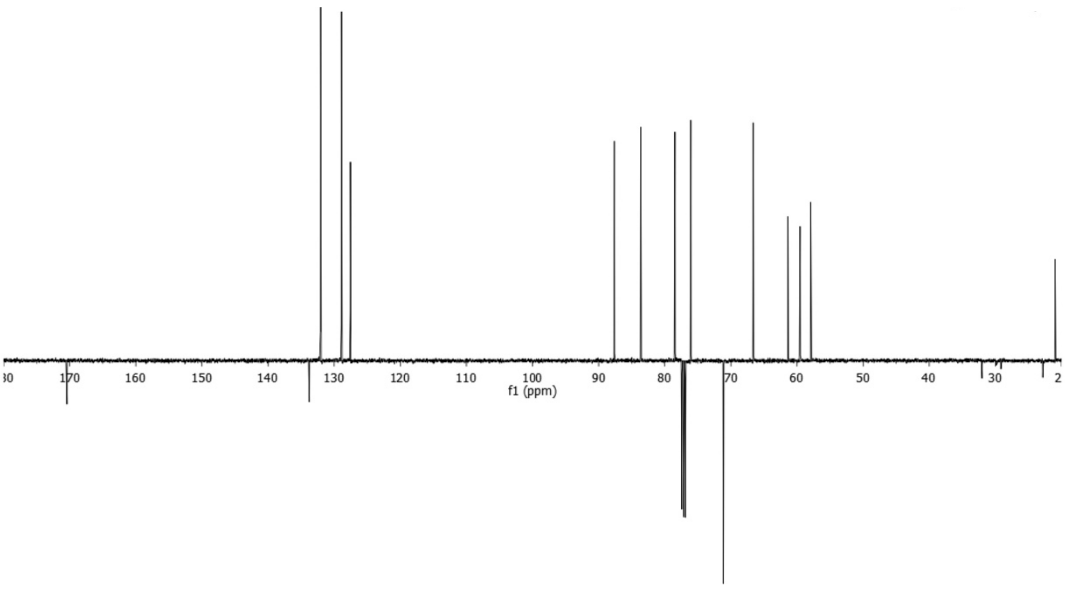

Supplementary Figure 126. ^{13}C NMR, 126 MHz, $CDCl_3$ of compound **8**

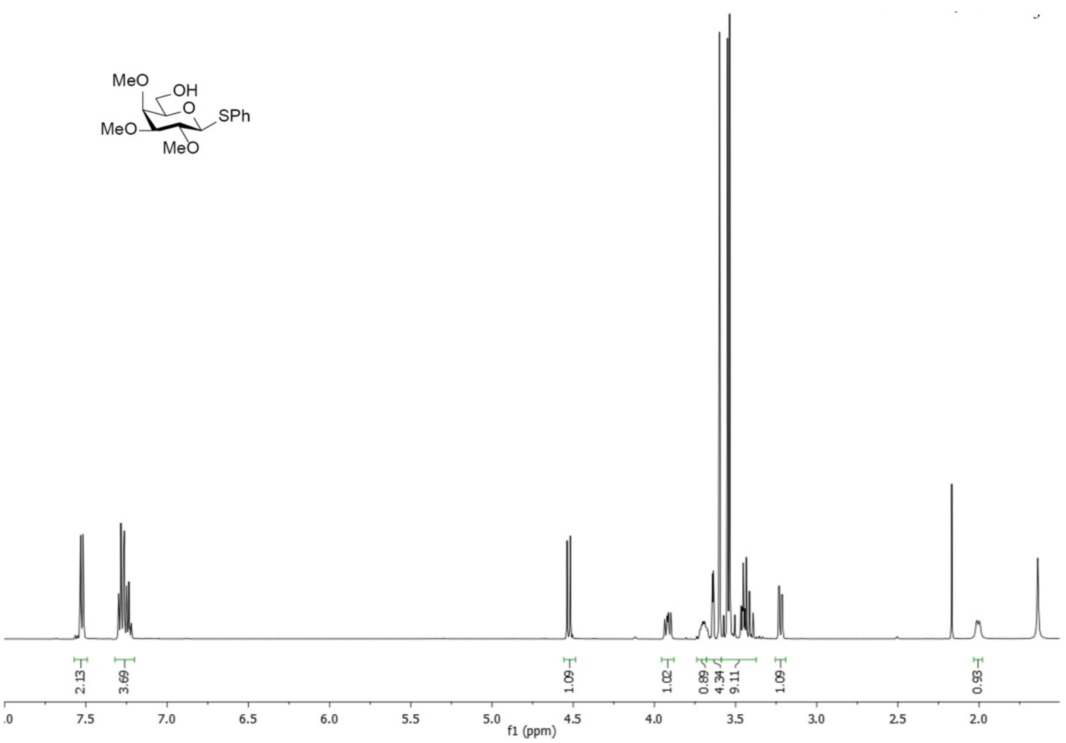

Supplementary Figure 127. ^{1}H NMR, 500 MHz, $CDCl_3$ of compound **S25**

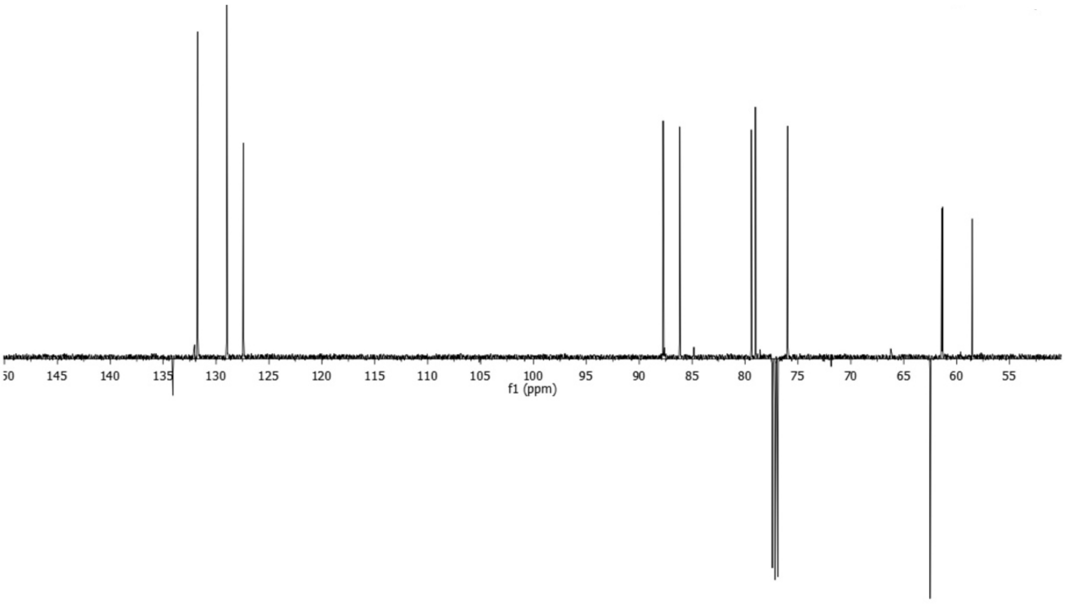

Supplementary Figure 128. ^{13}C NMR, 126 MHz, $CDCl_3$ of compound **S25**

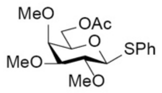

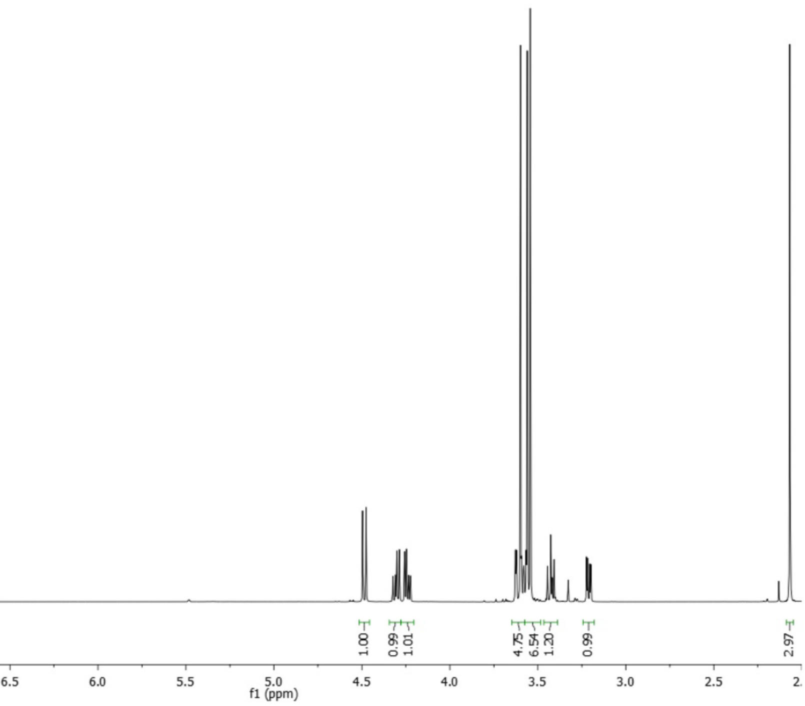

Supplementary Figure 129. ^{1}H NMR, 500 MHz, $CDCl_3$ of compound **9**

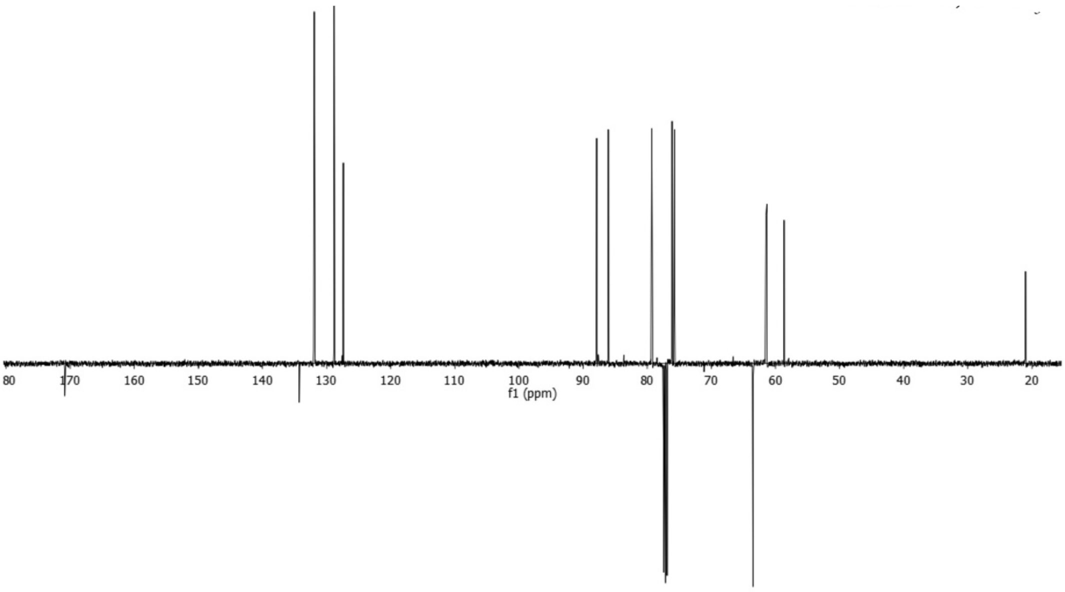

Supplementary Figure 130. ^{13}C NMR, 126 MHz, $CDCl_3$ of compound **9**

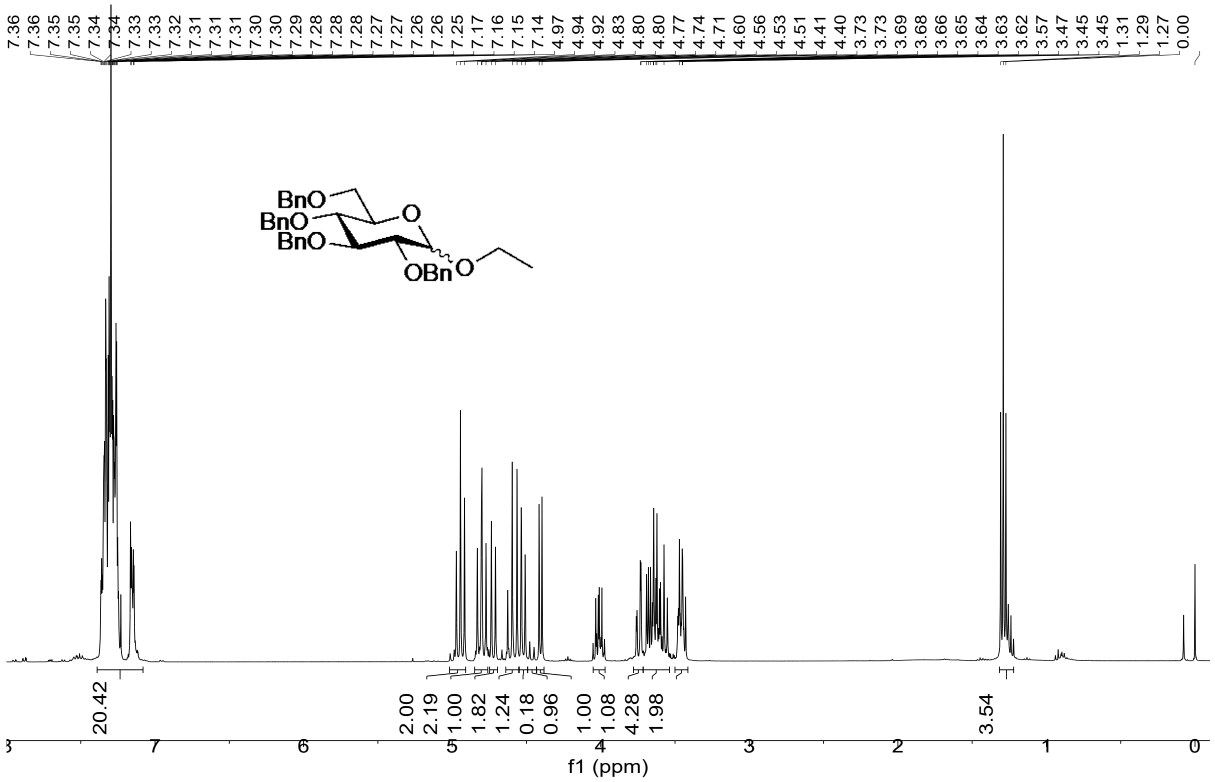

Supplementary Figure 131. ^{1}H NMR, 400 MHz, $CDCl_3$ of compound **S37**

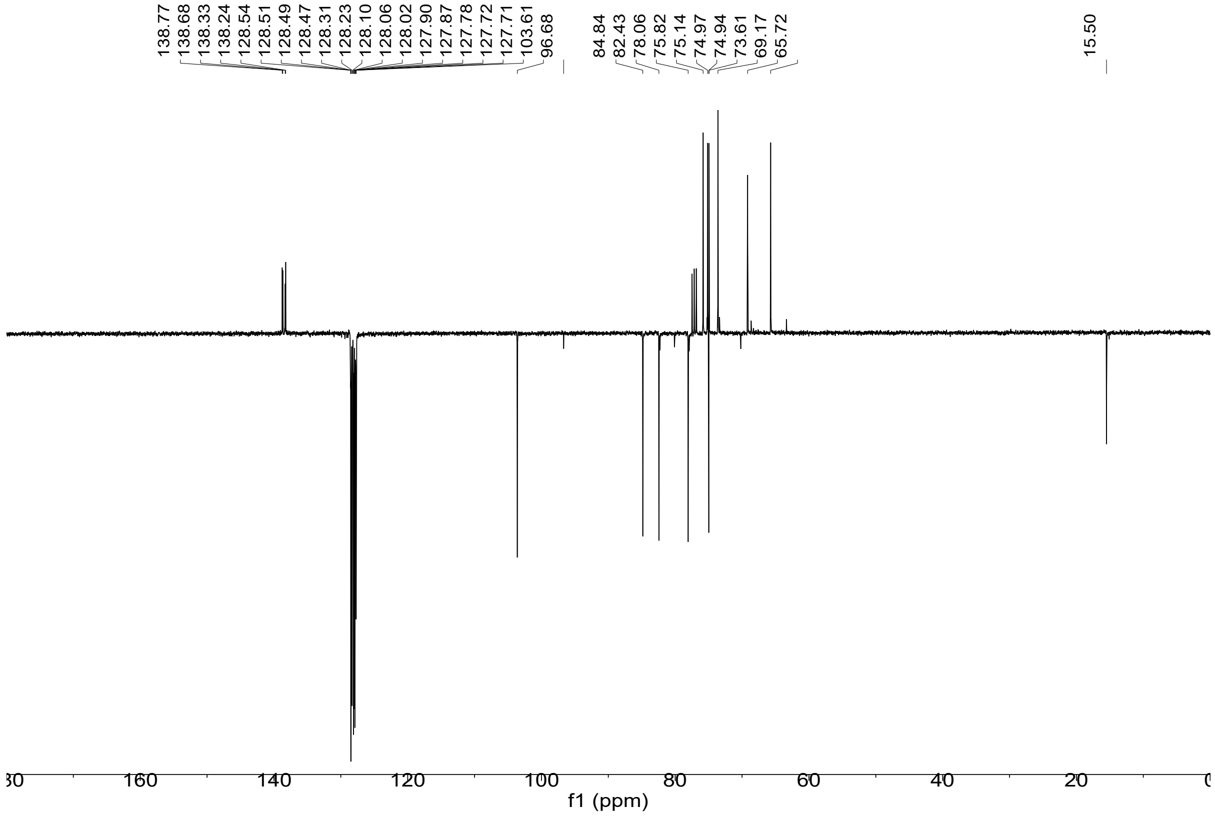

Supplementary Figure 132. ^{13}C NMR, 101 MHz, $CDCl_3$ of compound **S37**

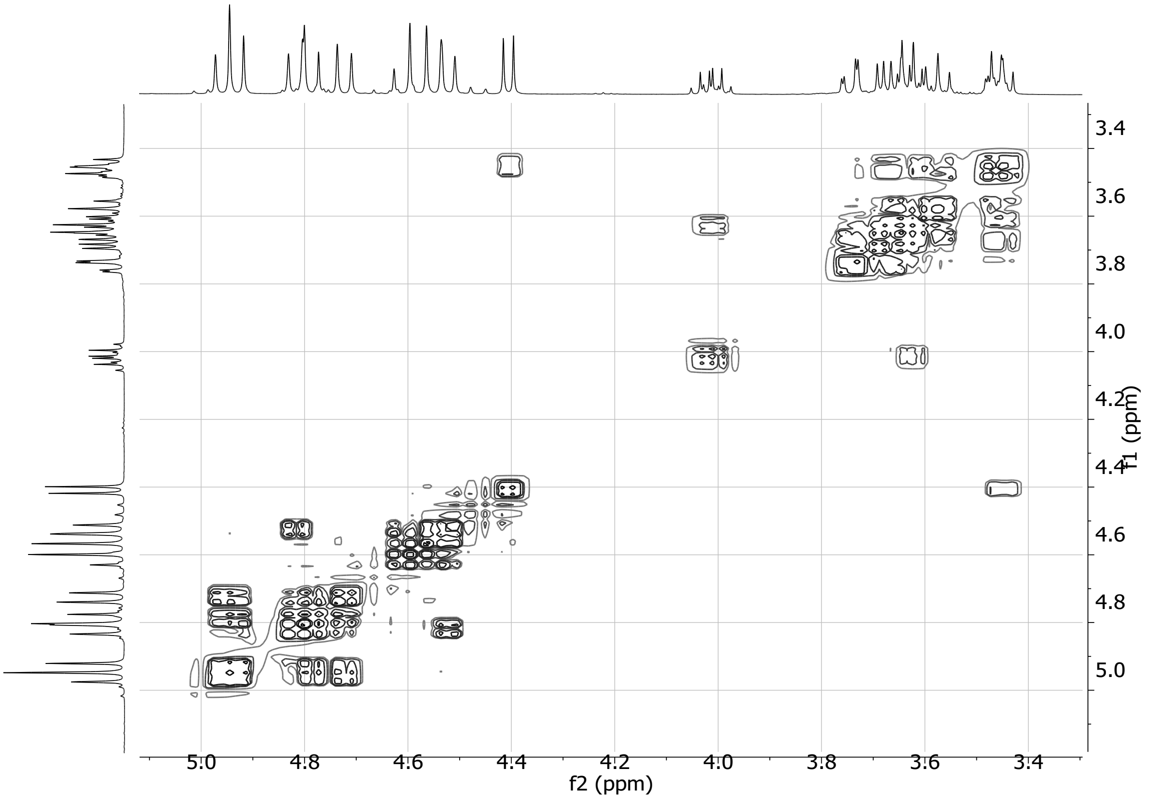

Supplementary Figure 133. HH-COSY NMR, $CDCl_3$ of compound **S37**

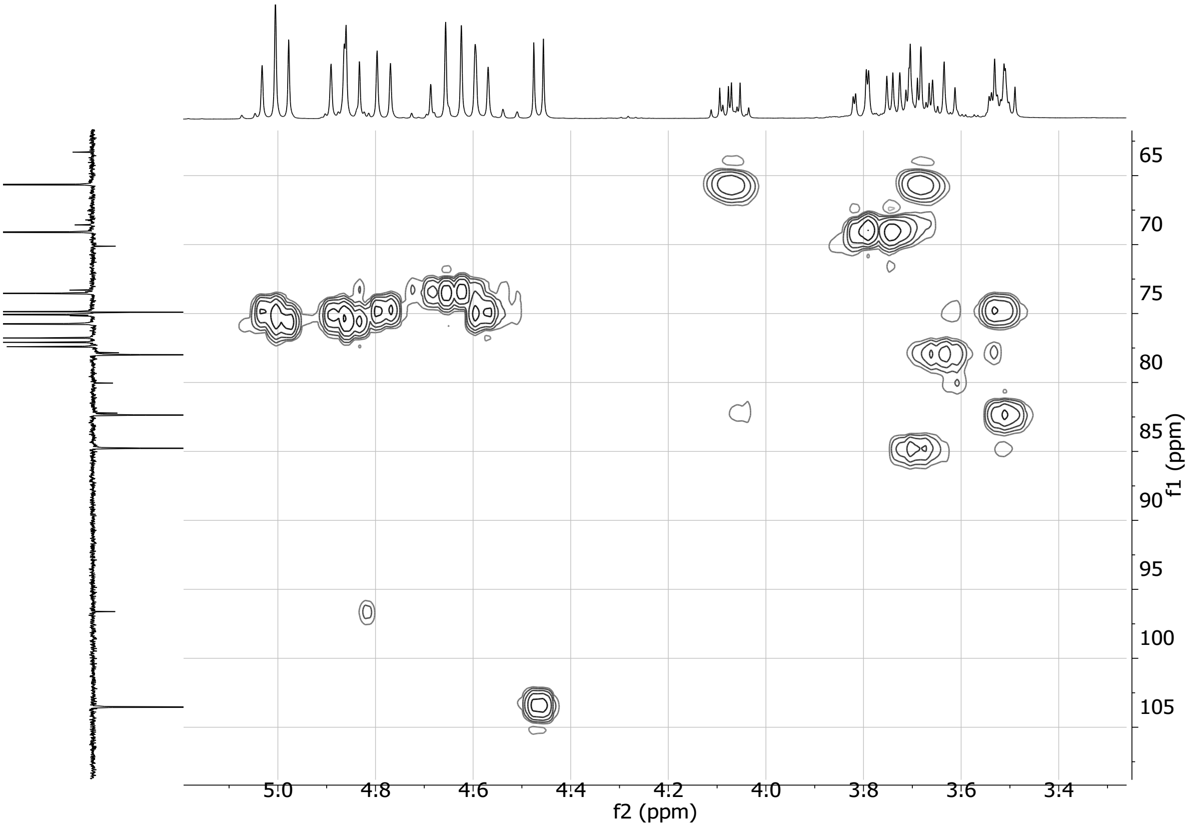

Supplementary Figure 134. HSQC NMR, $CDCl_3$ of compound **S37**

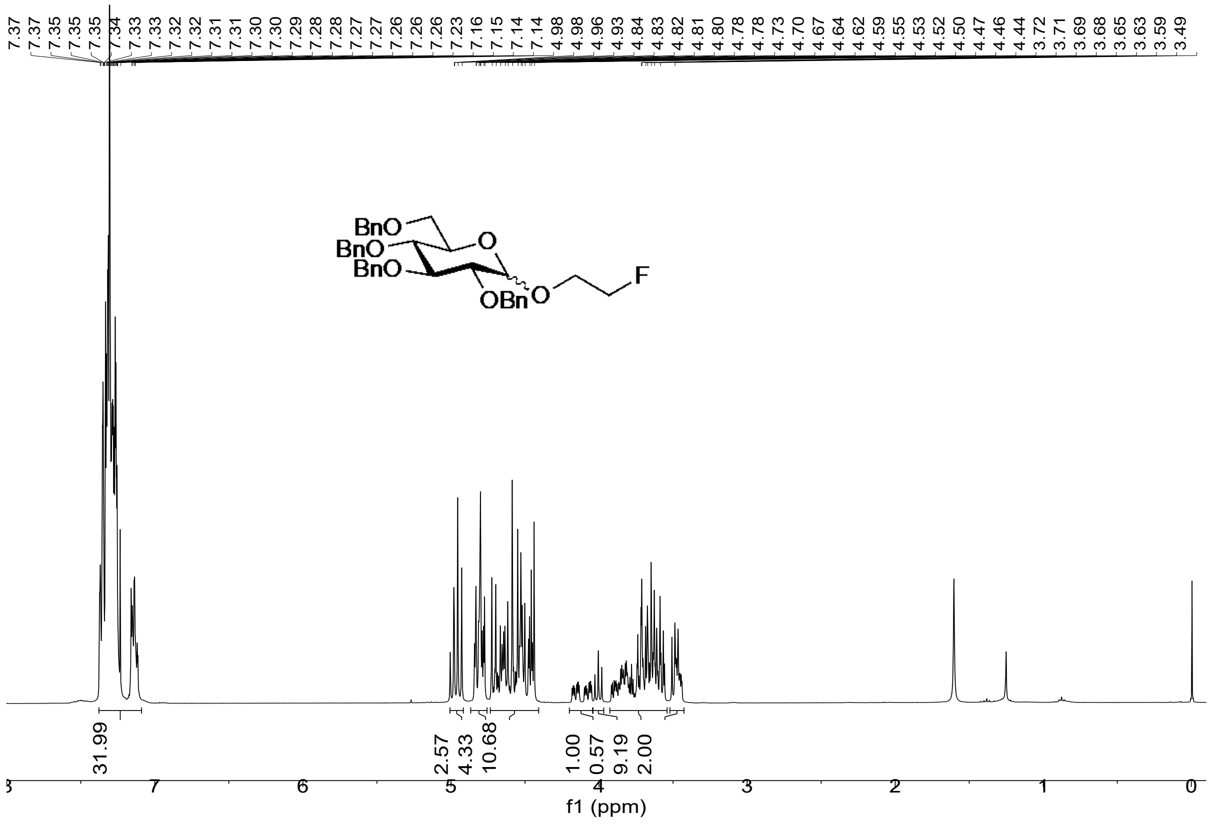

Supplementary Figure 135. ^{1}H NMR, 500 MHz, $CDCl_3$ of compound **S38**

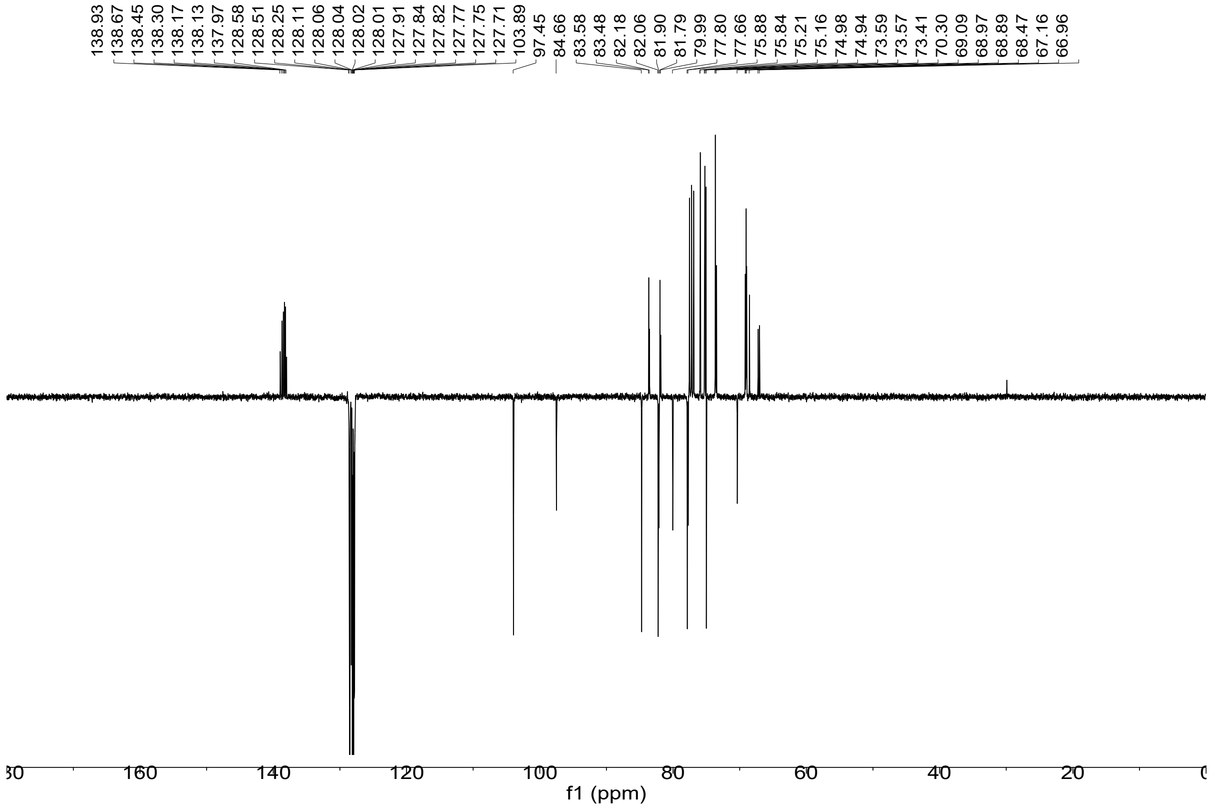

Supplementary Figure 136. ^{13}C NMR, 126 MHz, $CDCl_3$ of compound **S38**

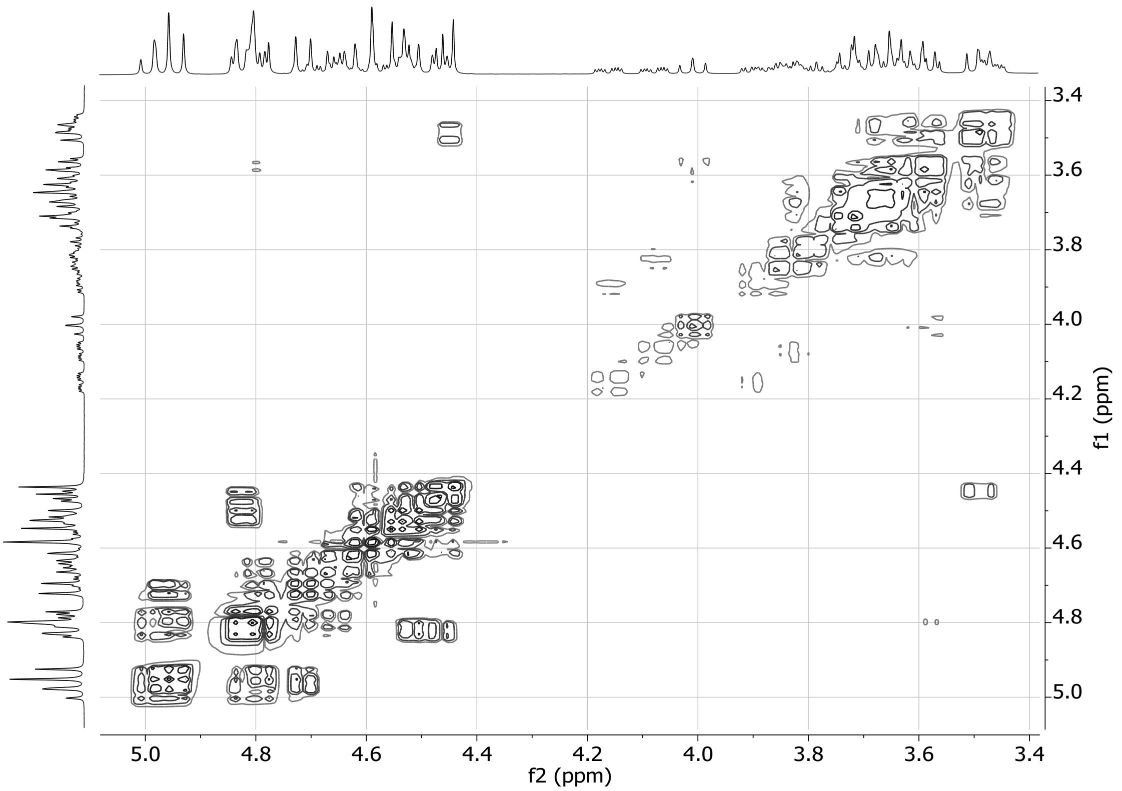

Supplementary Figure 137. HH-COSY NMR, $CDCl_3$ of compound **S38**

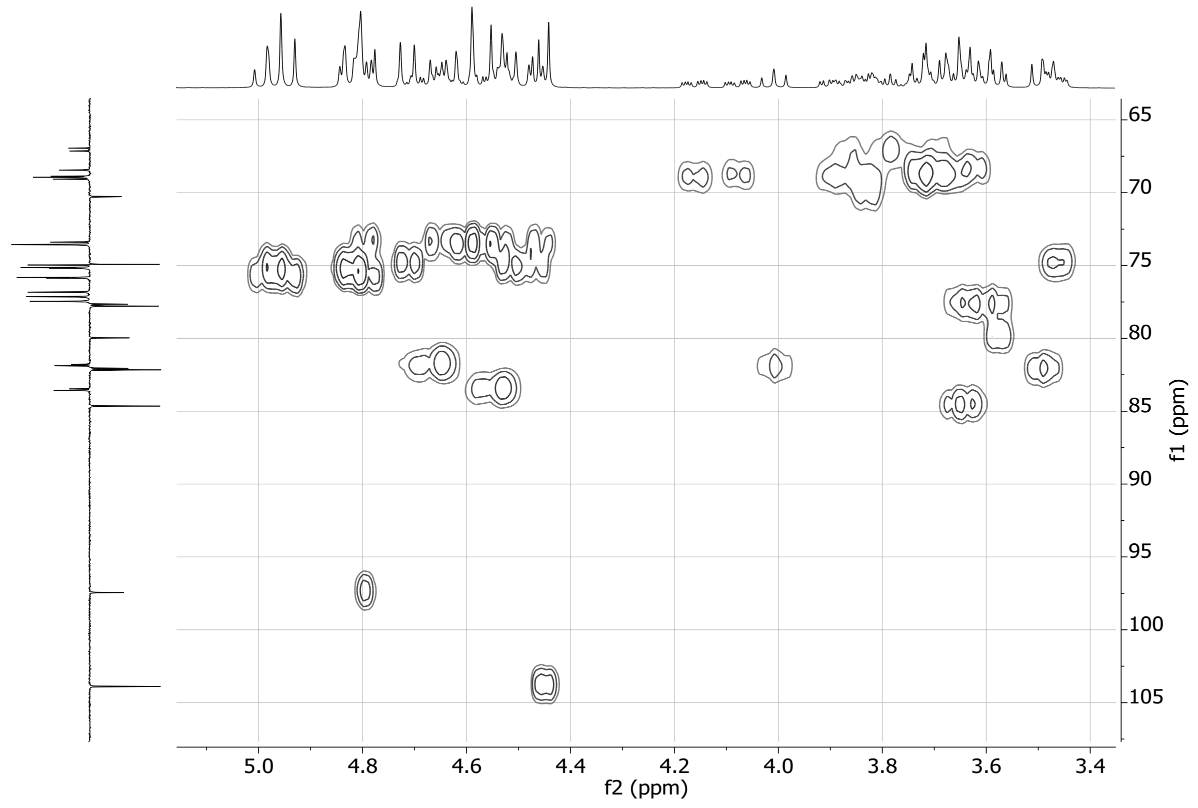

Supplementary Figure 138. HSQC NMR, $CDCl_3$ of compound **S38**

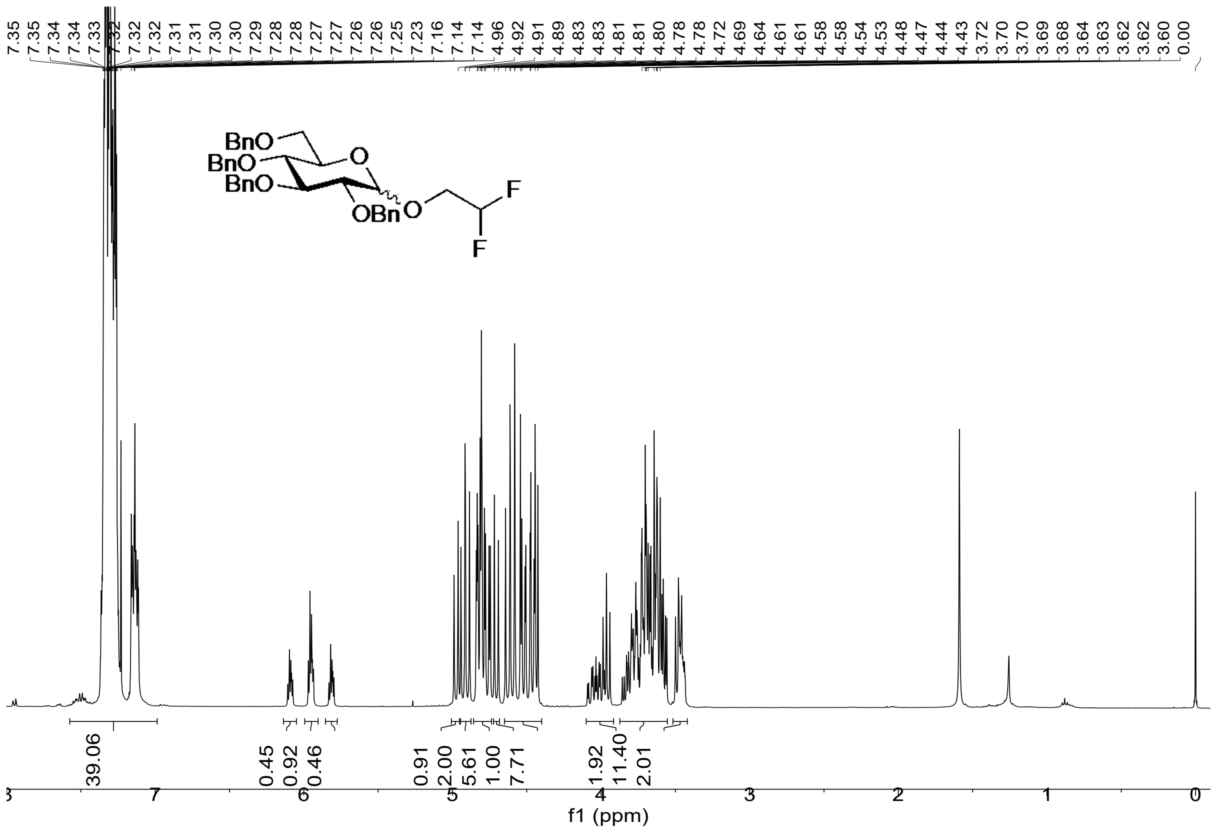

Supplementary Figure 139. ^{1}H NMR, 400 MHz, $CDCl_3$ of compound **S39**

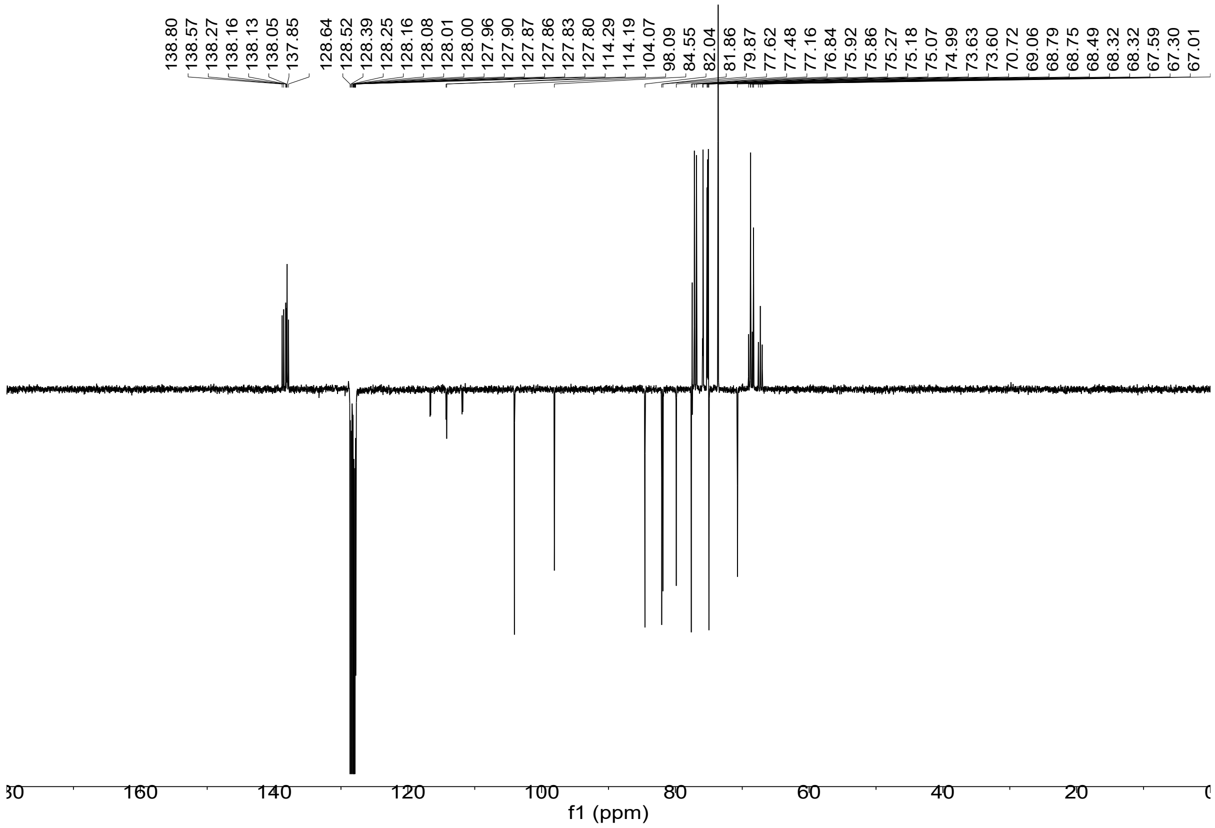

Supplementary Figure 140. ^{13}C NMR, 101 MHz, $CDCl_3$ of compound **S39**

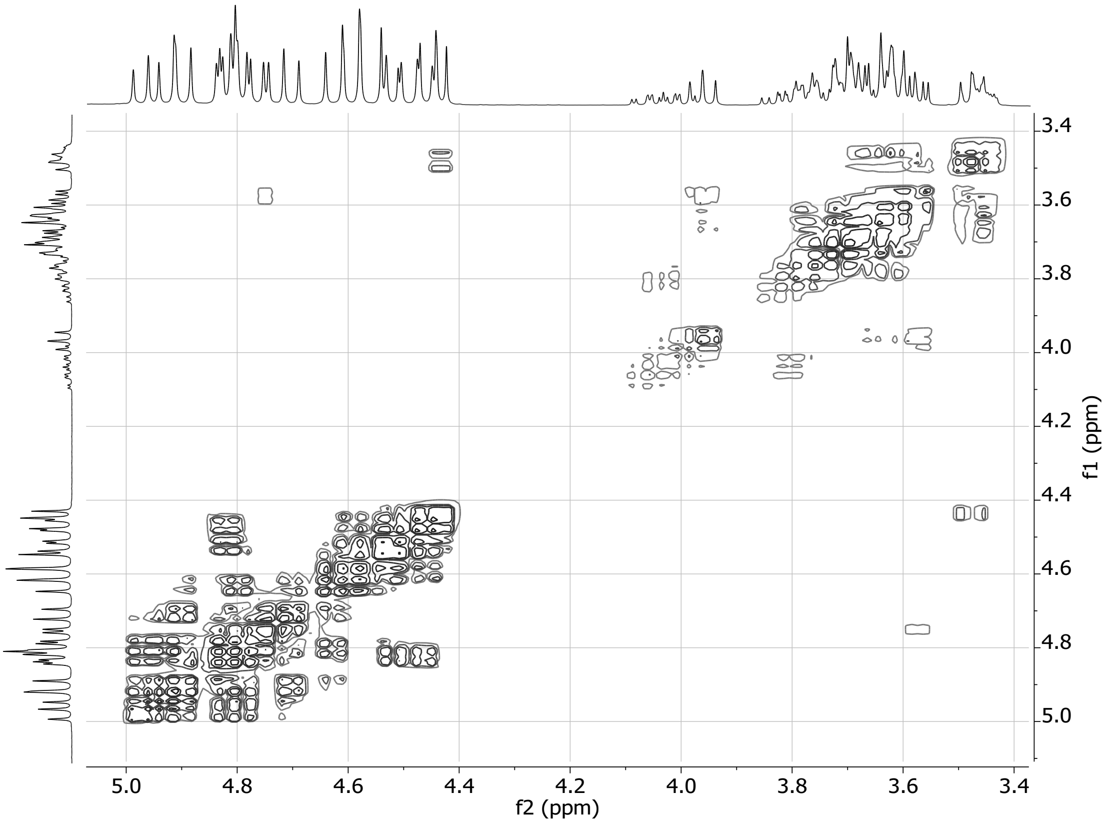

Supplementary Figure 141. HH-COSY NMR, $CDCl_3$ of compound **S39**

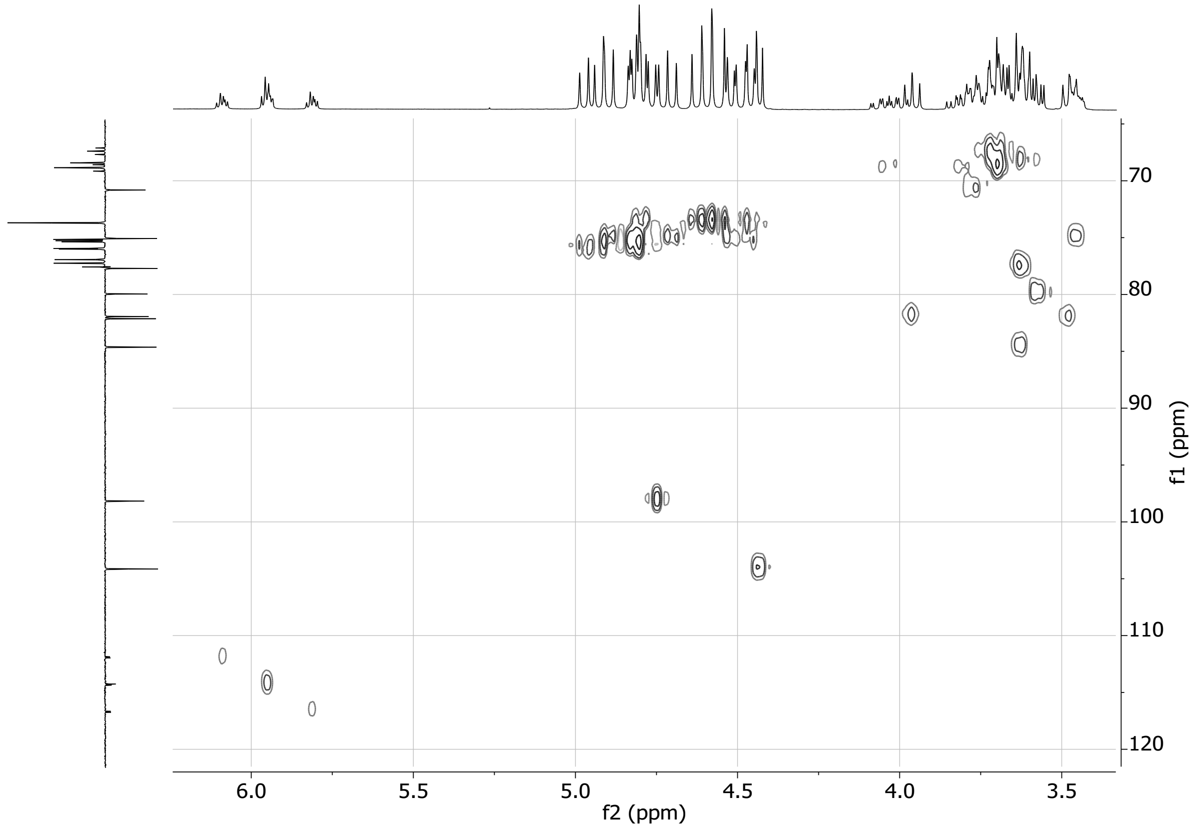

Supplementary Figure 142. HSQC NMR, $CDCl_3$ of compound **S39**

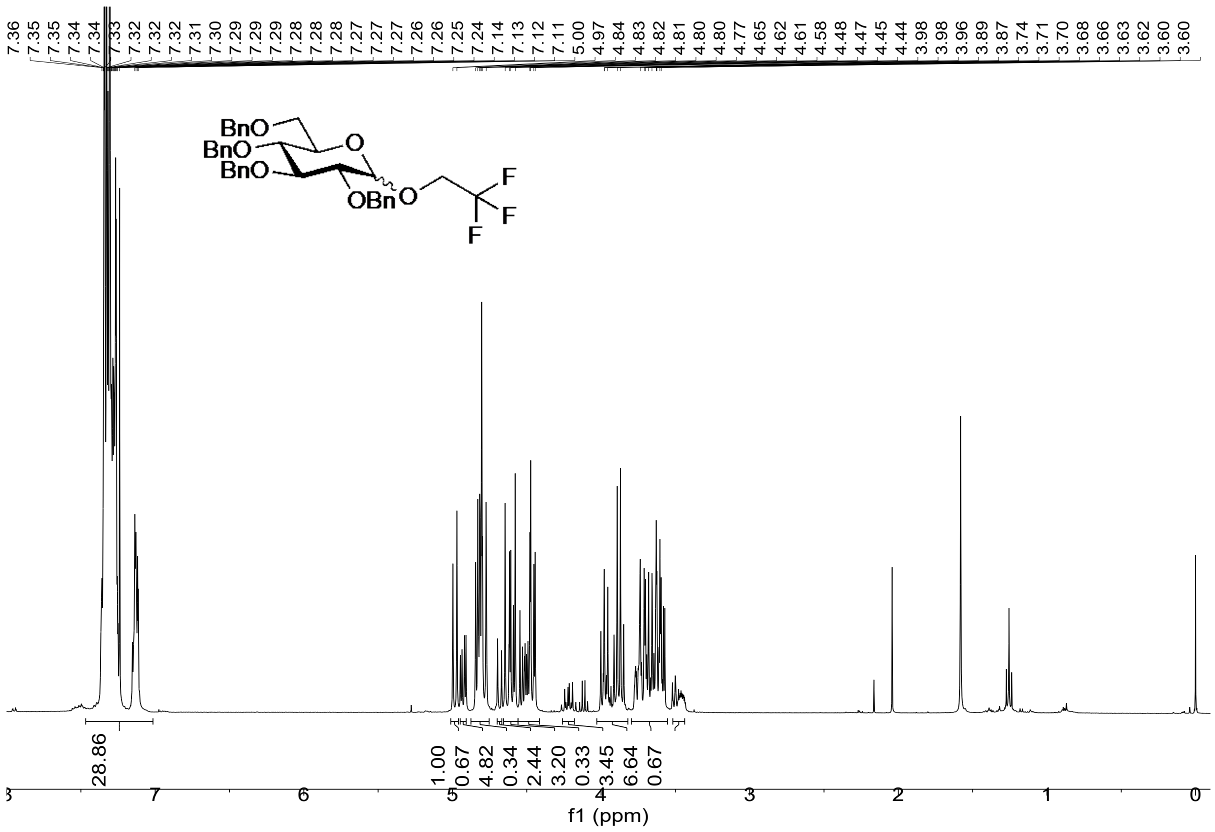

Supplementary Figure 143. ^{1}H NMR, 500 MHz, $CDCl_3$ of compound **S40**

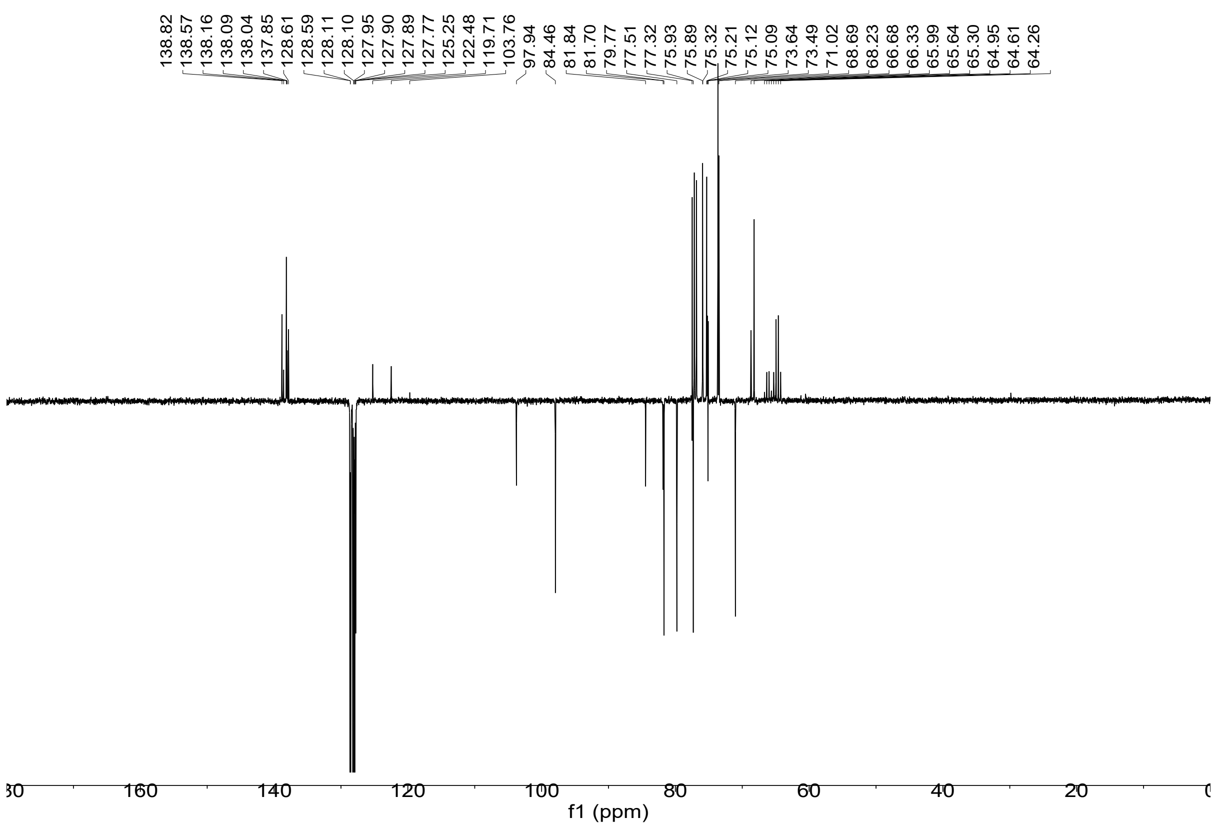

Supplementary Figure 144. ^{13}C NMR, 126 MHz, $CDCl_3$ of compound **S40**

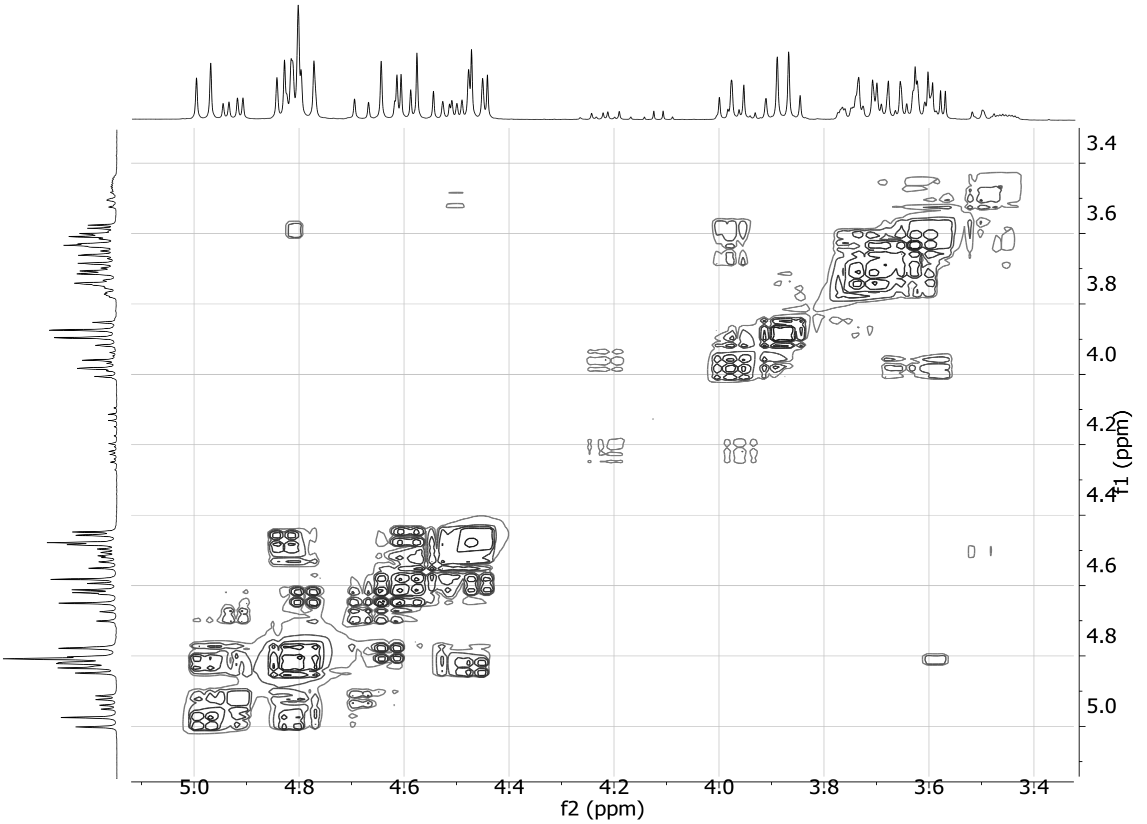

Supplementary Figure 145. HH-COSY NMR, $CDCl_3$ of compound **S40**

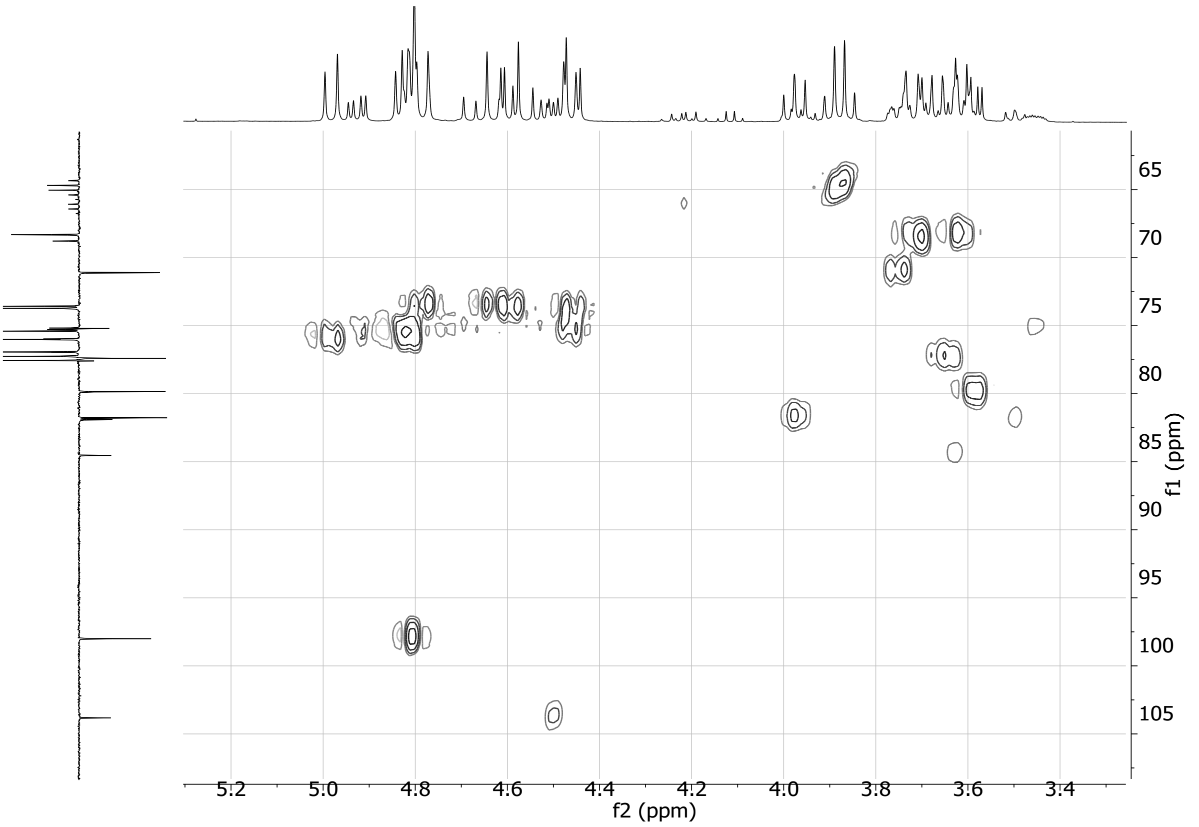

Supplementary Figure 146. HSQC NMR, $CDCl_3$ of compound **S40**

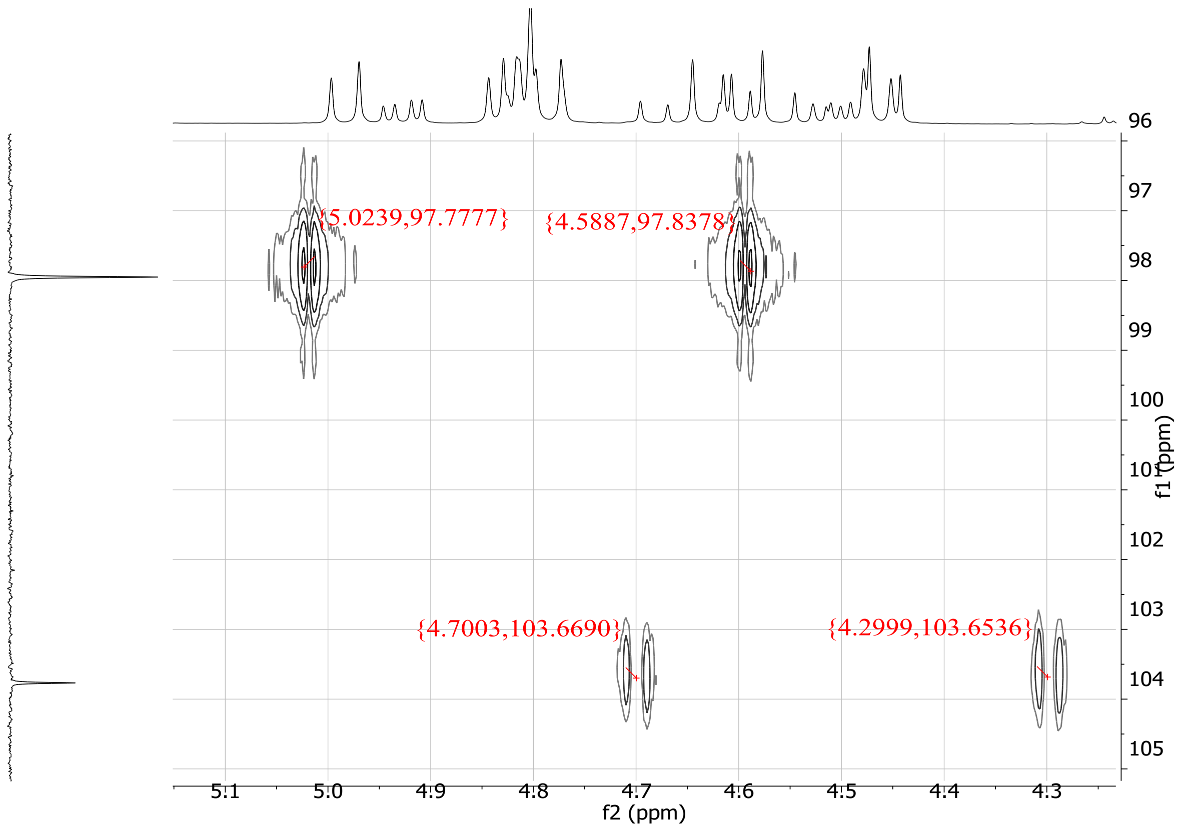

Supplementary Figure 147. HMBC-GATED NMR, $CDCl_3$ of compound **S40**

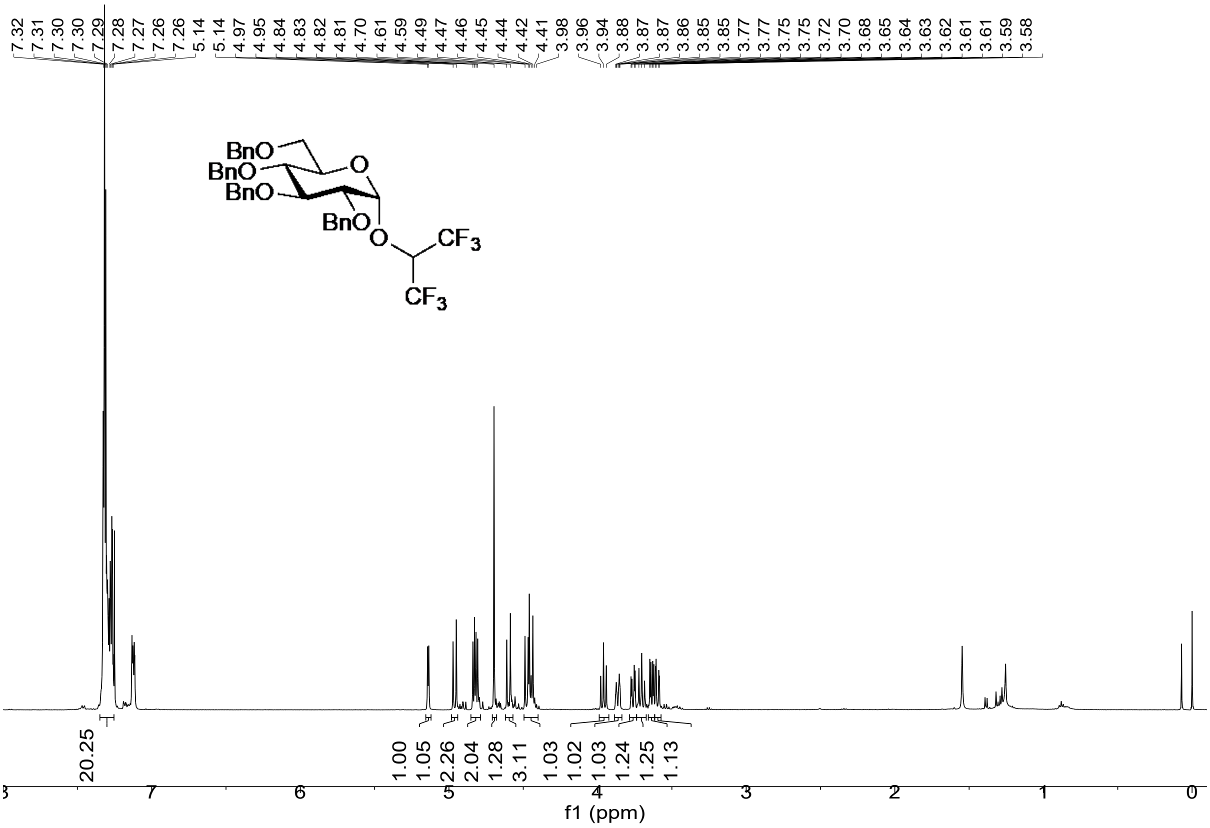

Supplementary Figure 148. ^{1}H NMR, 500 MHz, $CDCl_3$ of compound **S41**

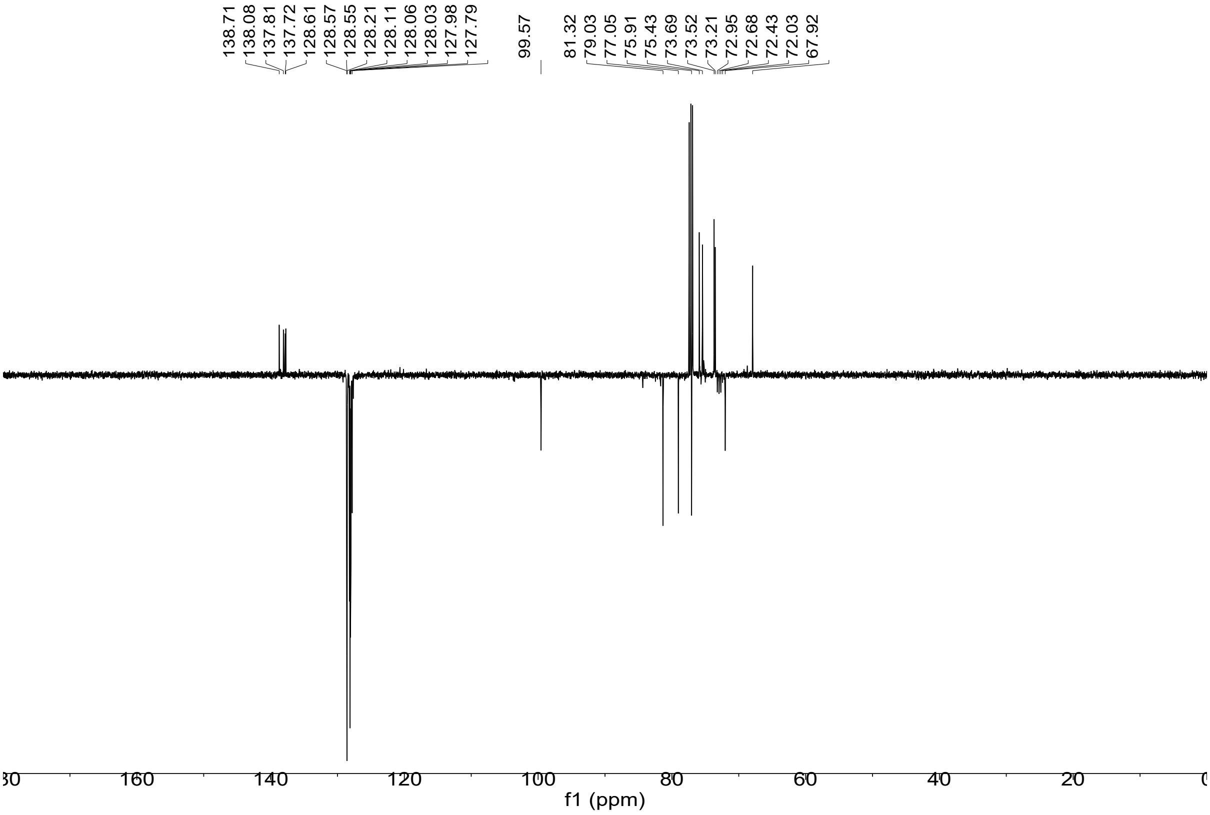

Supplementary Figure 149. ^{13}C NMR, 126 MHz, $CDCl_3$ of compound **S41**

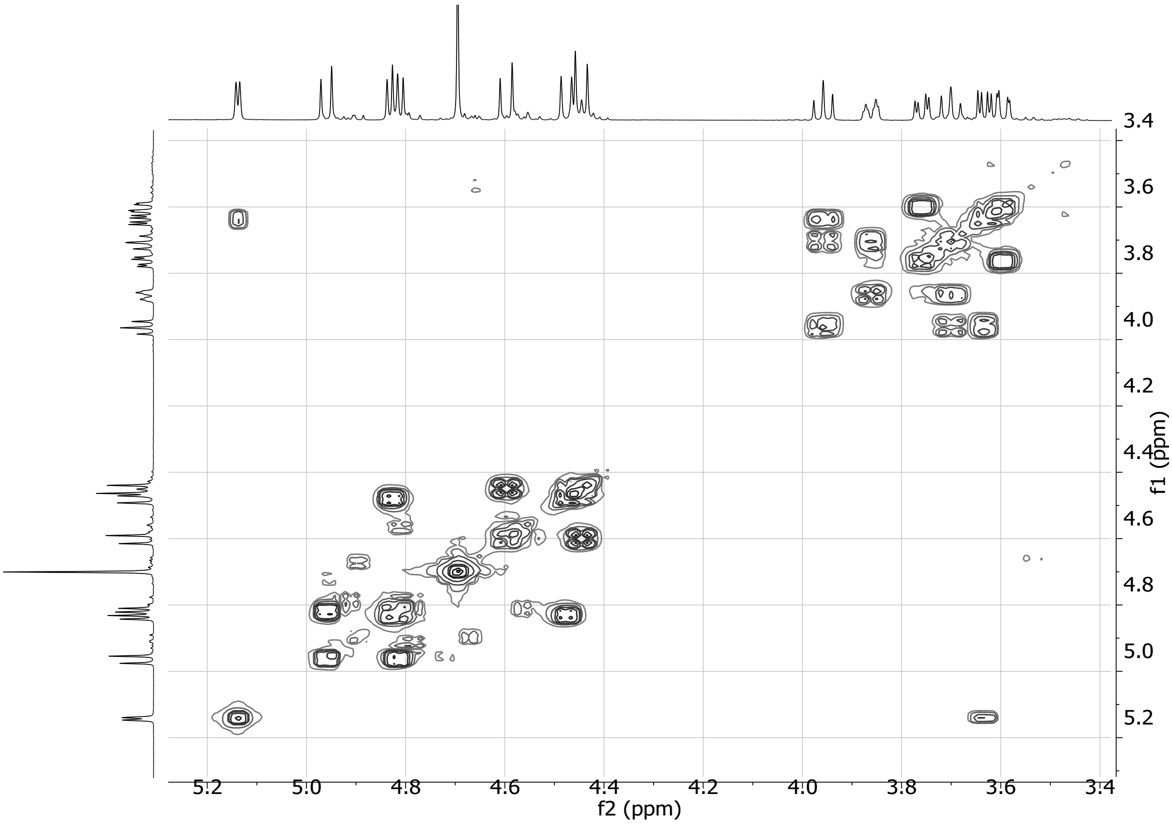

Supplementary Figure 150. HH-COSY NMR, $CDCl_3$ of compound **S41**

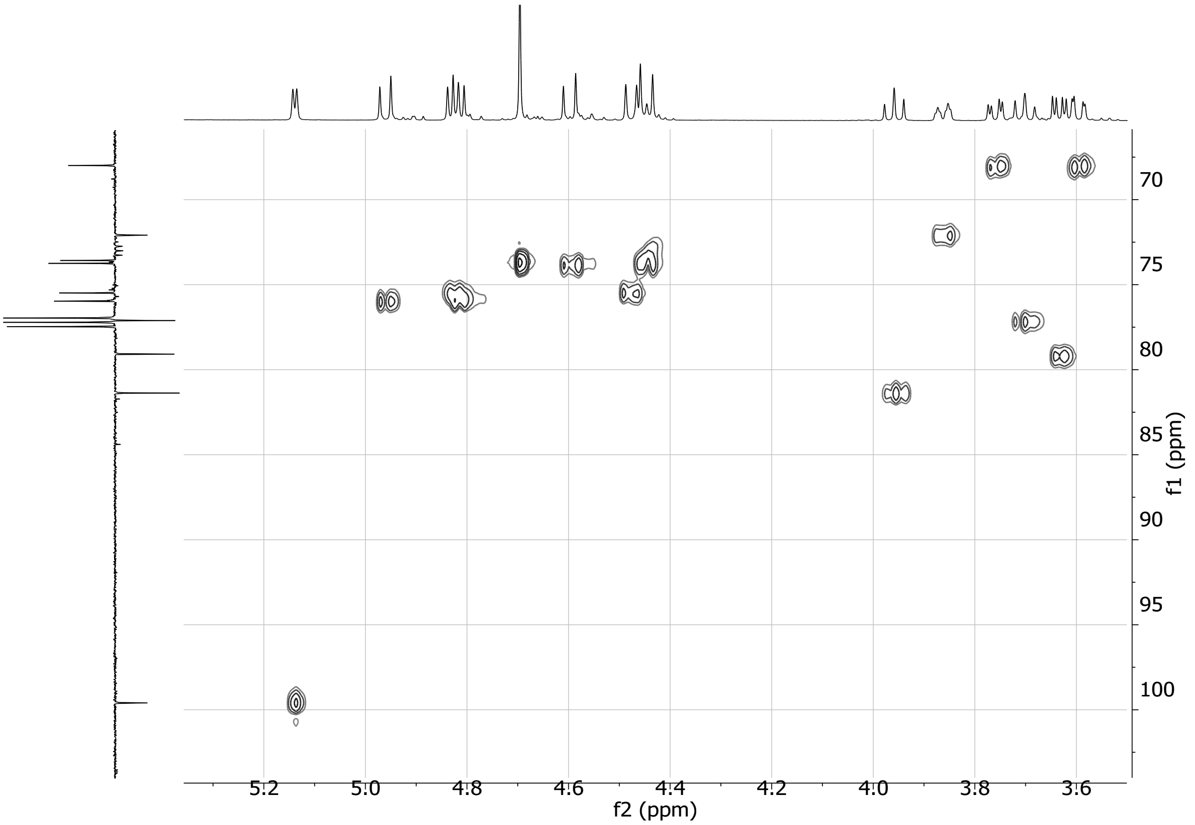

Supplementary Figure 151. HSQC NMR, $CDCl_3$ of compound **S41**

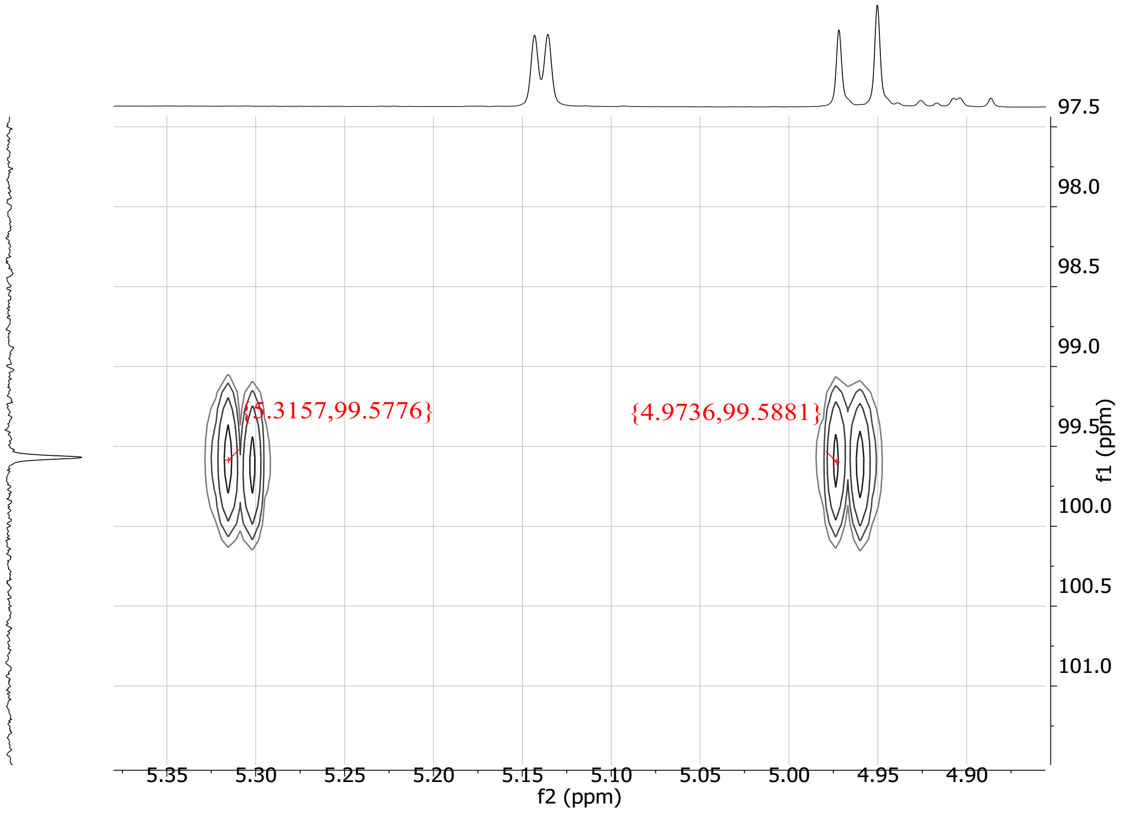

Supplementary Figure 152. HMBC-GATED NMR, $CDCl_3$ of compound **S4**

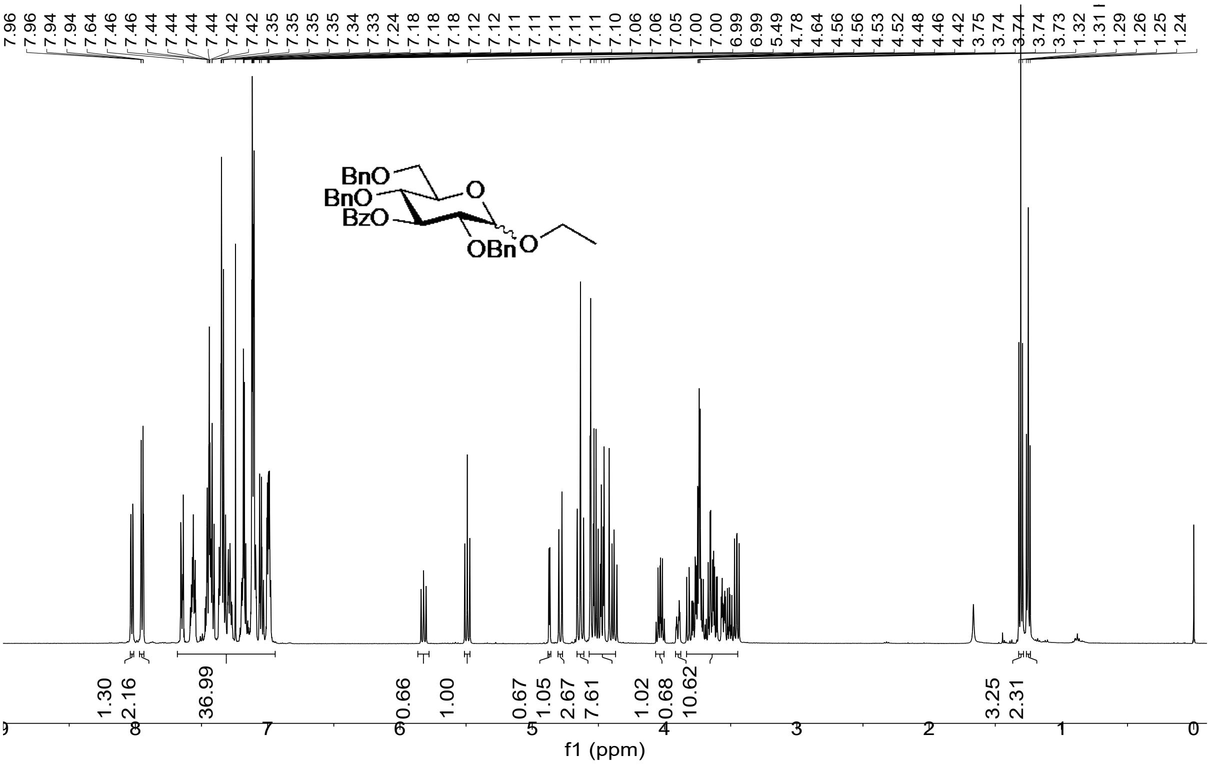

Supplementary Figure 153. ^{1}H NMR, 500 MHz, $CDCl_3$ of compound **S42**

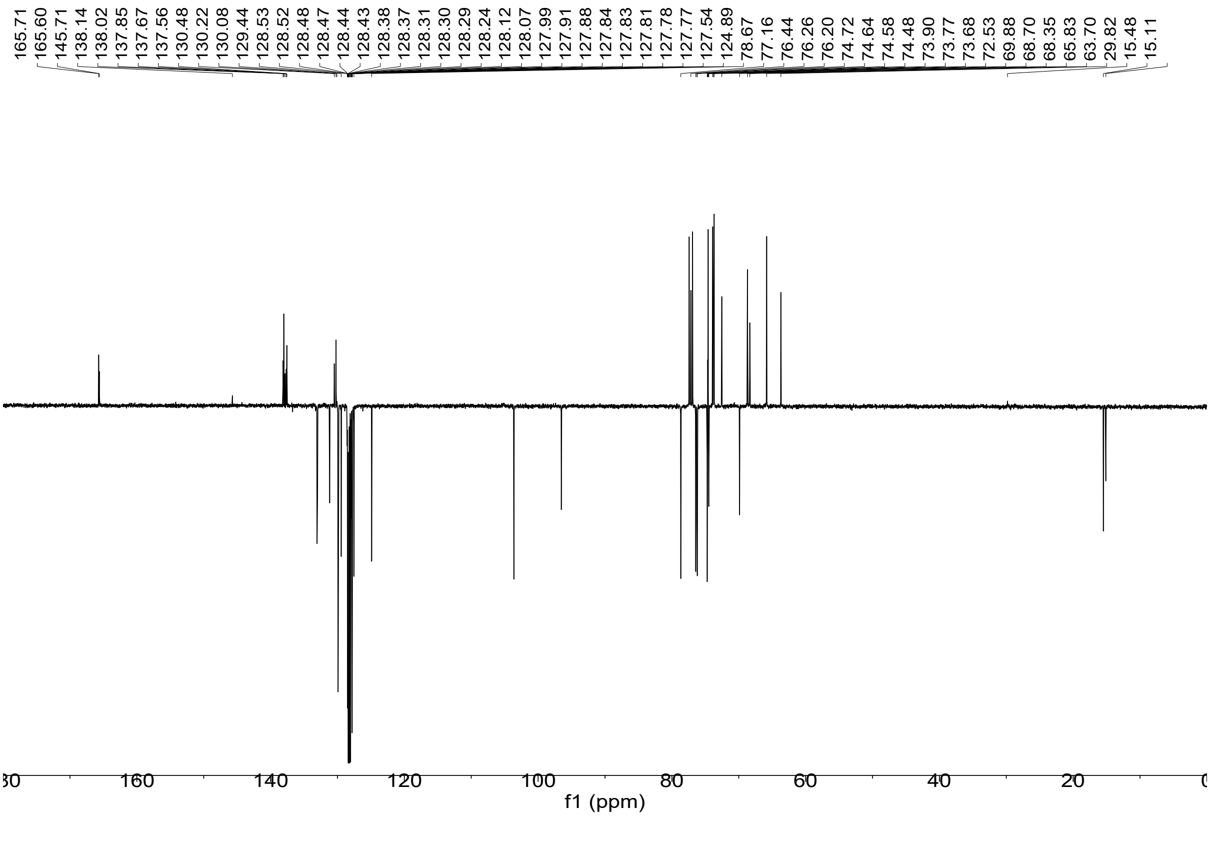

Supplementary Figure 154. ^{13}C NMR, 126 MHz, $CDCl_3$ of compound **S42**

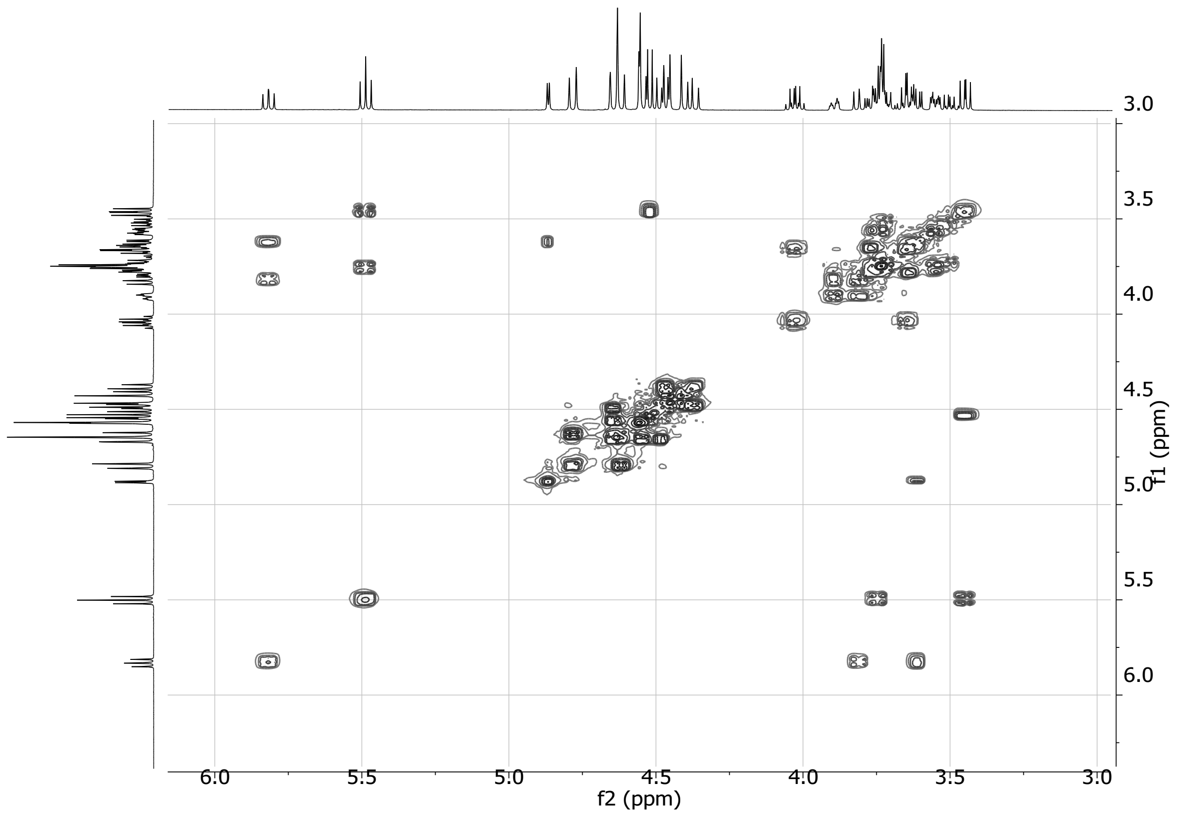

Supplementary Figure 155. HH-COSY NMR, $CDCl_3$ of compound **S42**

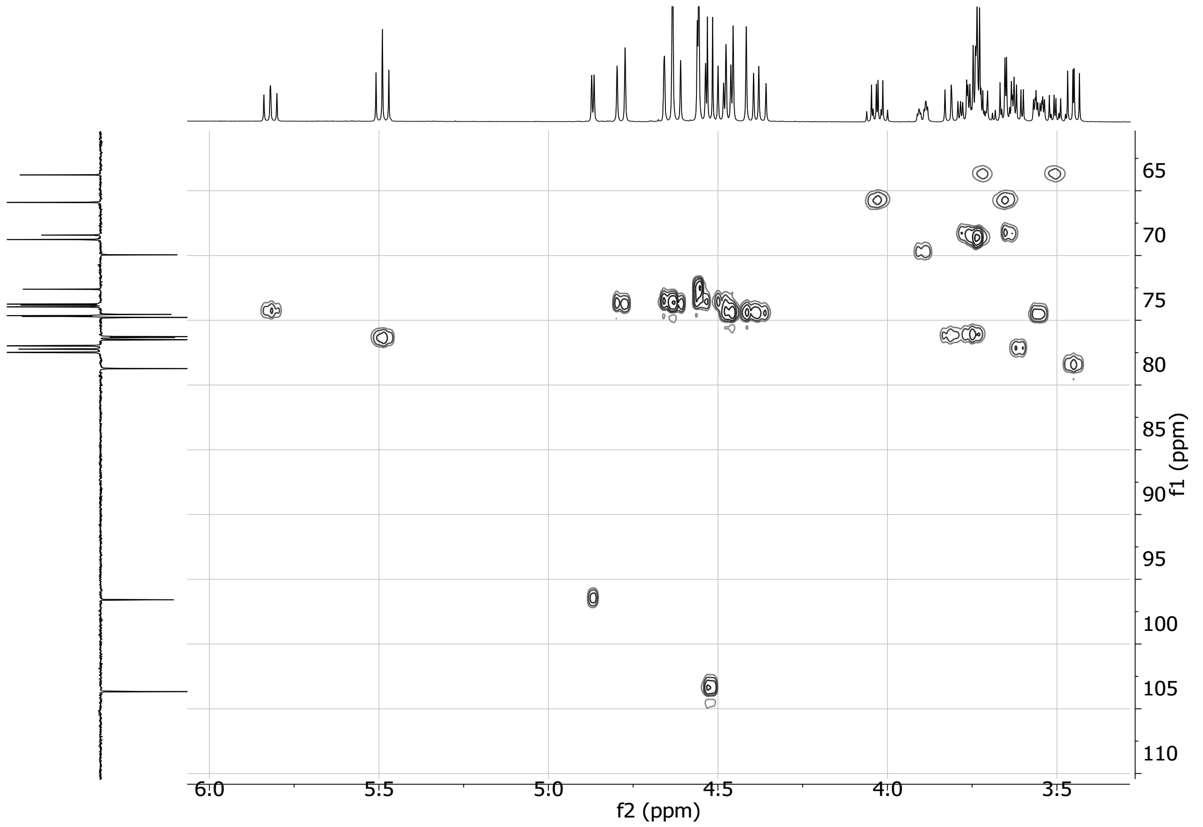

Supplementary Figure 156. HSQC NMR, $CDCl_3$ of compound **S42**

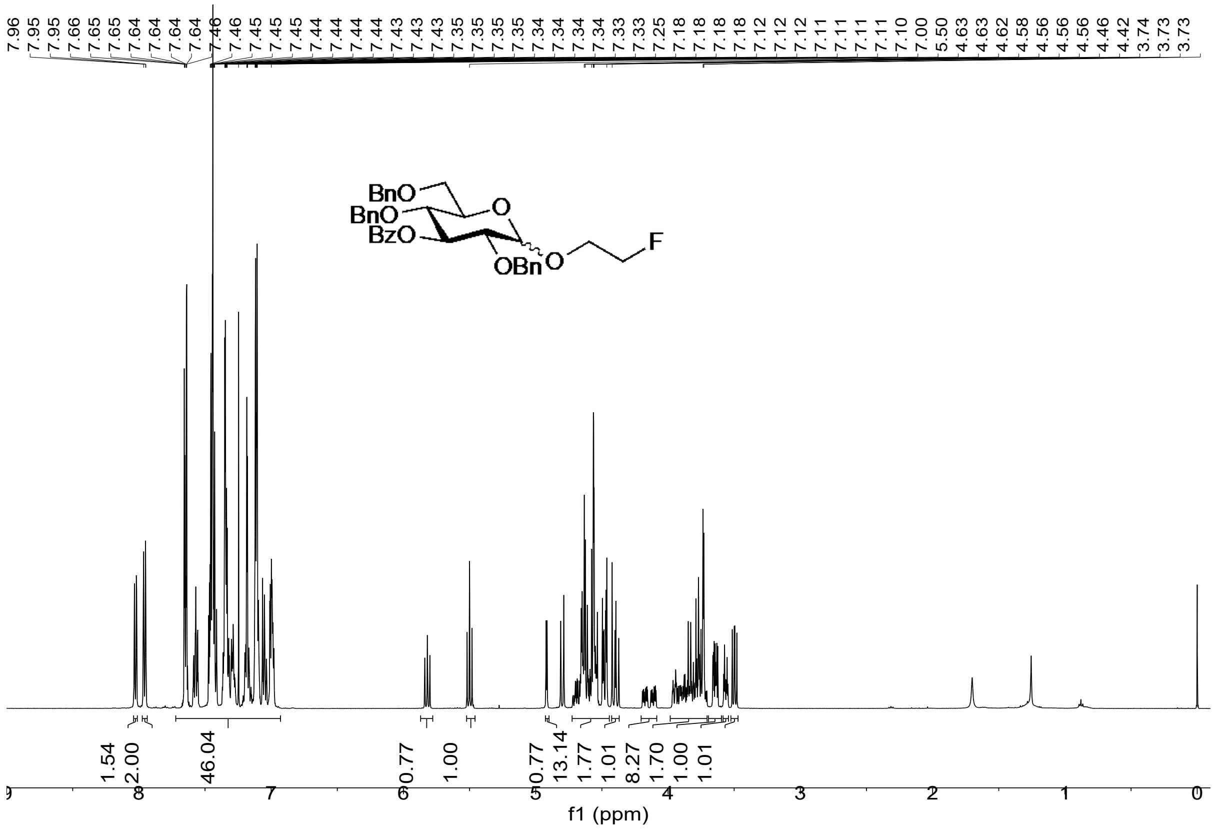

Supplementary Figure 157. ^{1}H NMR, 500 MHz, $CDCl_3$ of compound **S43**

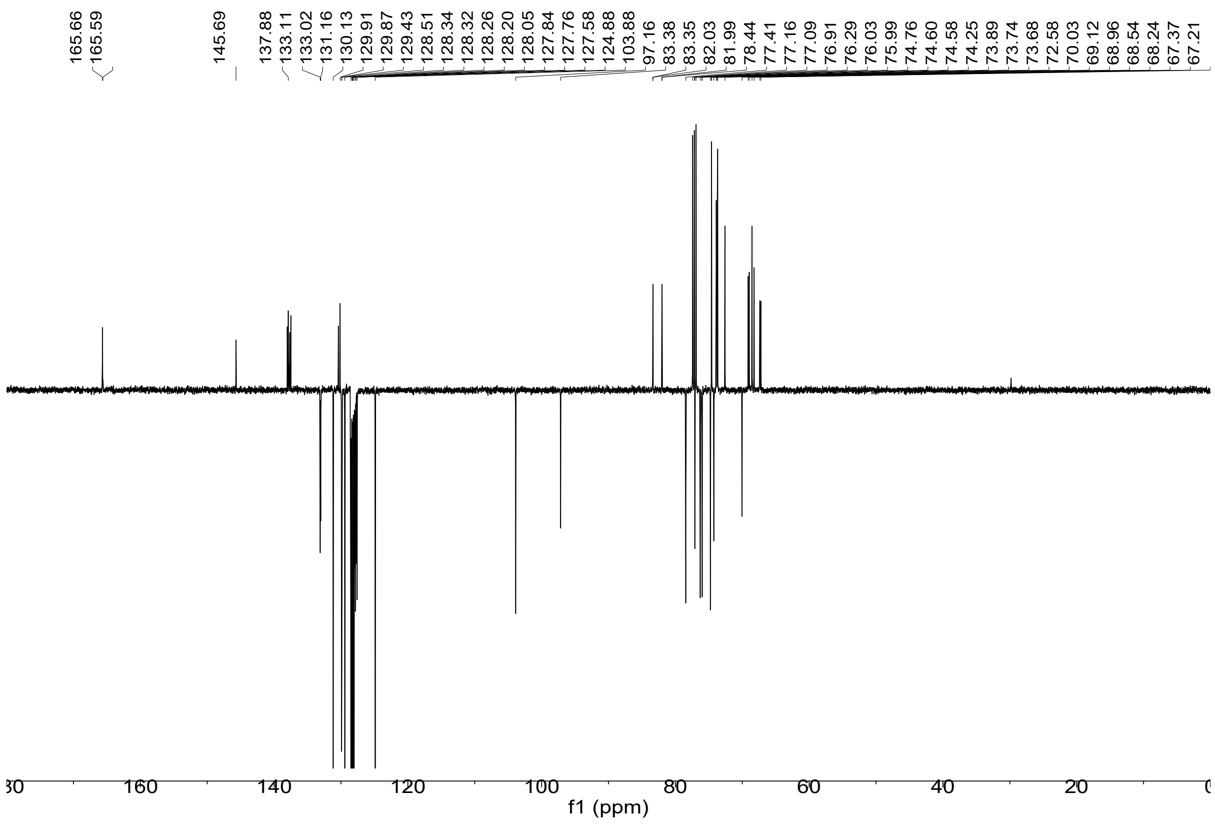

Supplementary Figure 158. ^{13}C NMR, 126 MHz, $CDCl_3$ of compound **S43**

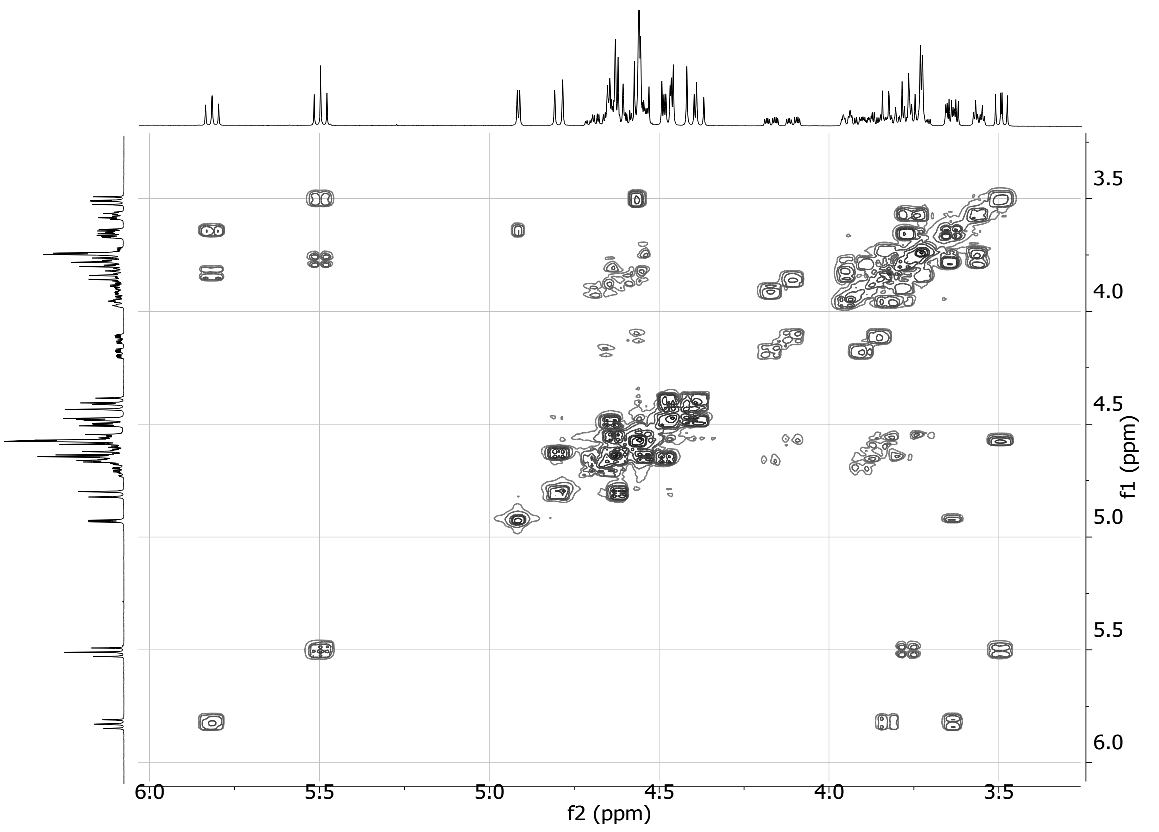

Supplementary Figure 159. HH-COSY NMR, $CDCl_3$ of compound **S43**

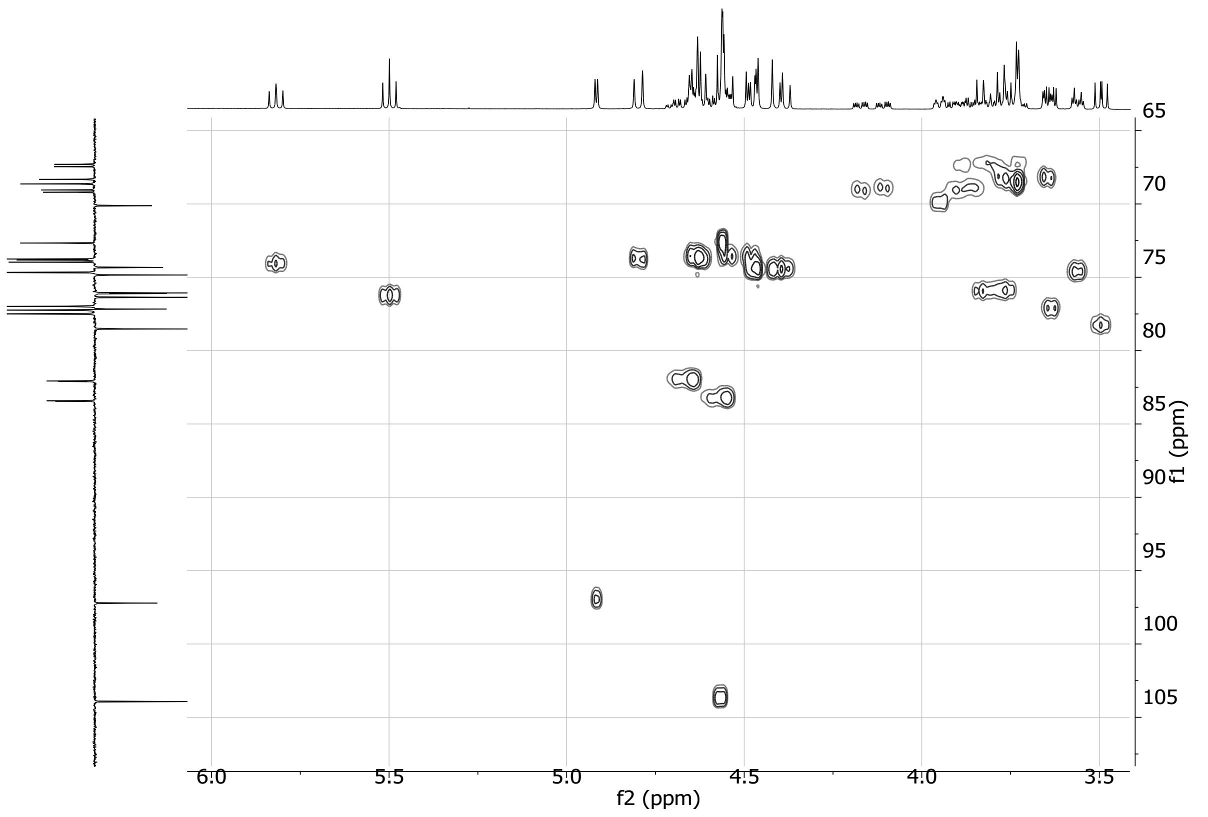

Supplementary Figure 160. HSQC NMR, $CDCl_3$ of compound **S43**

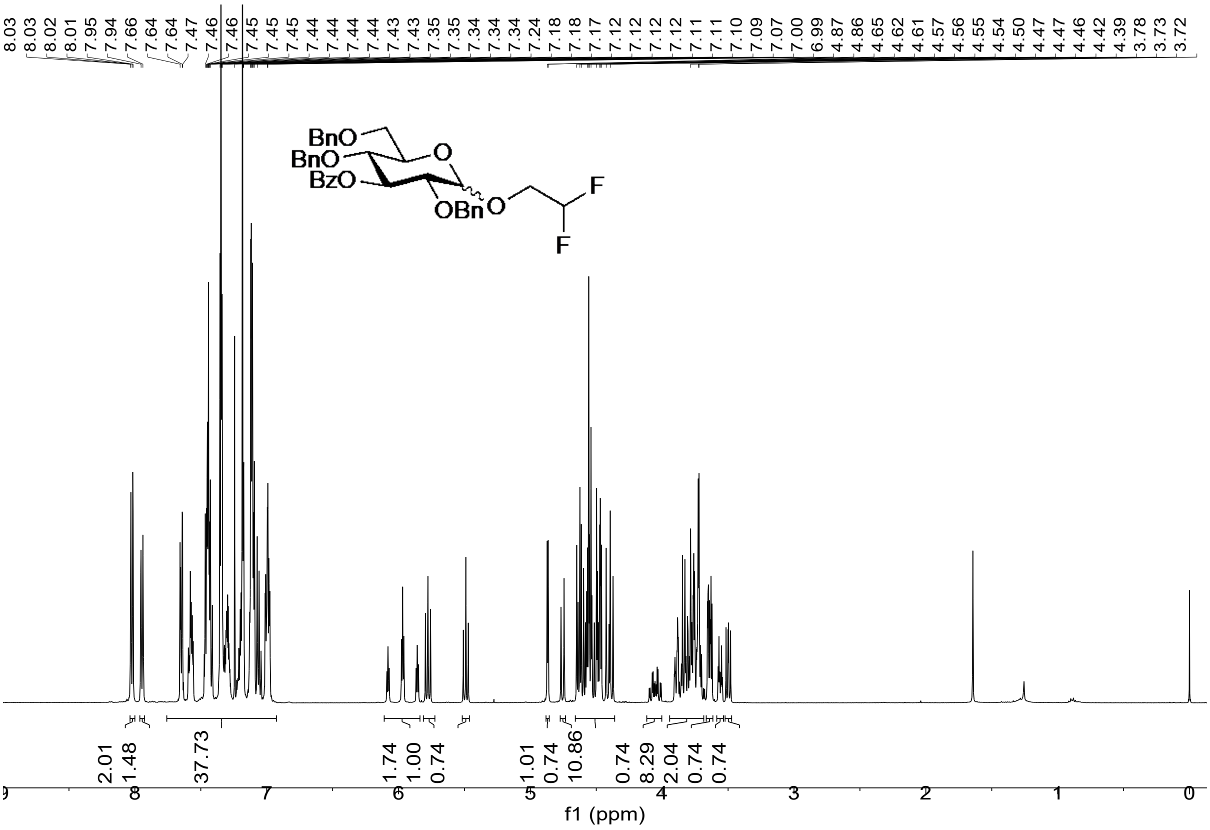

Supplementary Figure 161. ^{1}H NMR, 500 MHz, $CDCl_3$ of compound **S44**

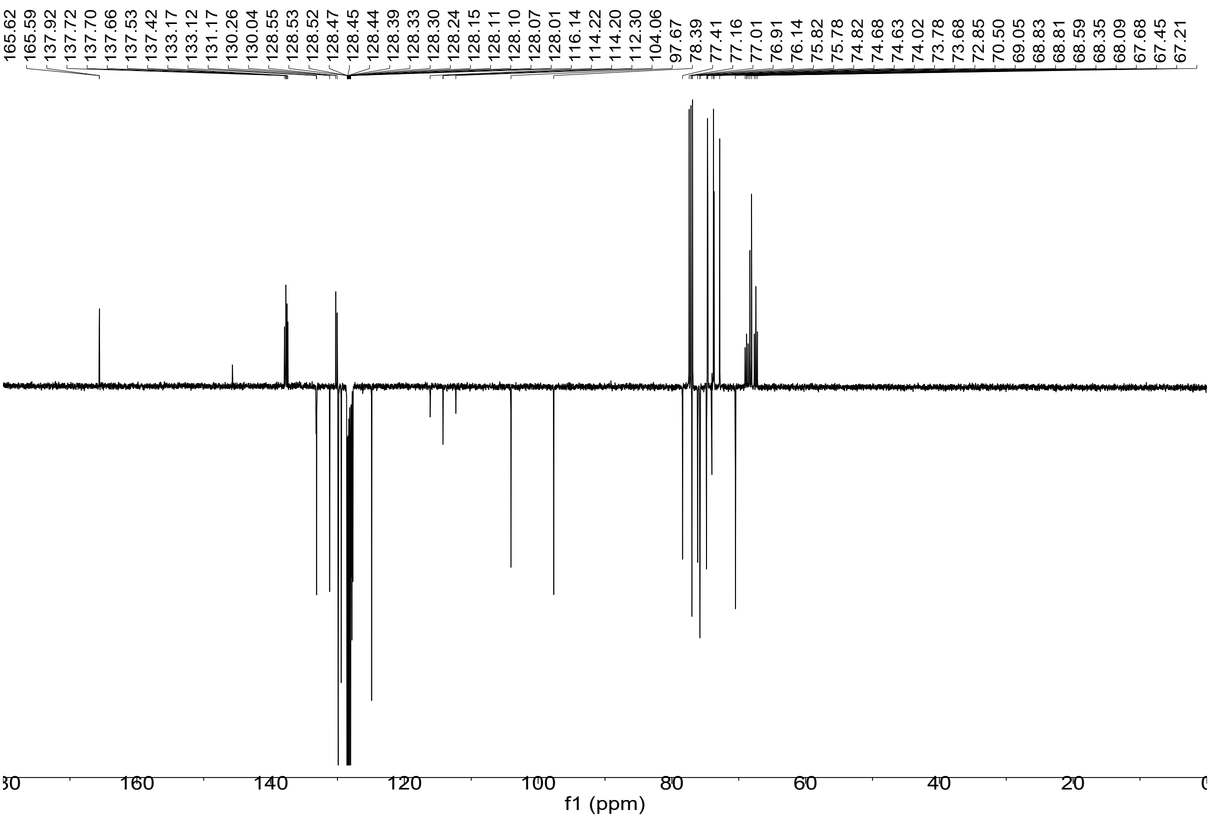

Supplementary Figure 162. ^{13}C NMR, 126 MHz, $CDCl_3$ of compound **S44**

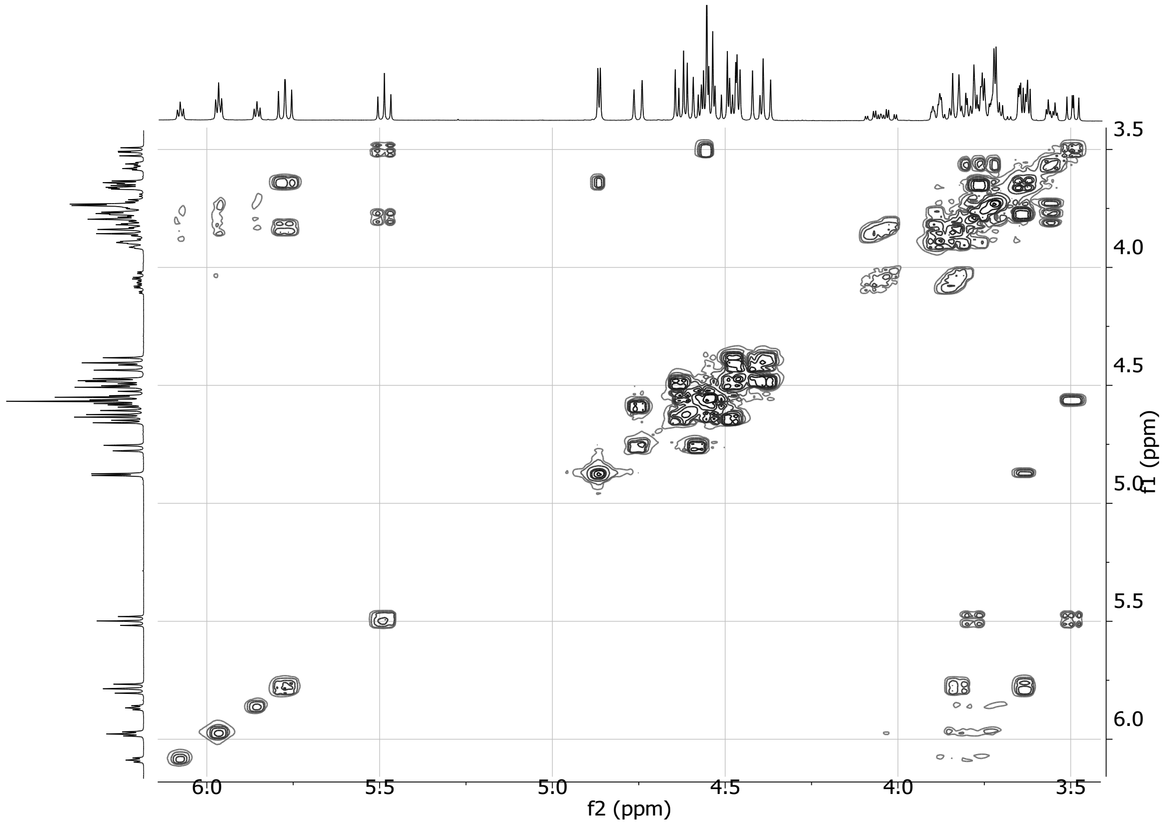

Supplementary Figure 163. HH-COSY NMR, $CDCl_3$ of compound **S44**

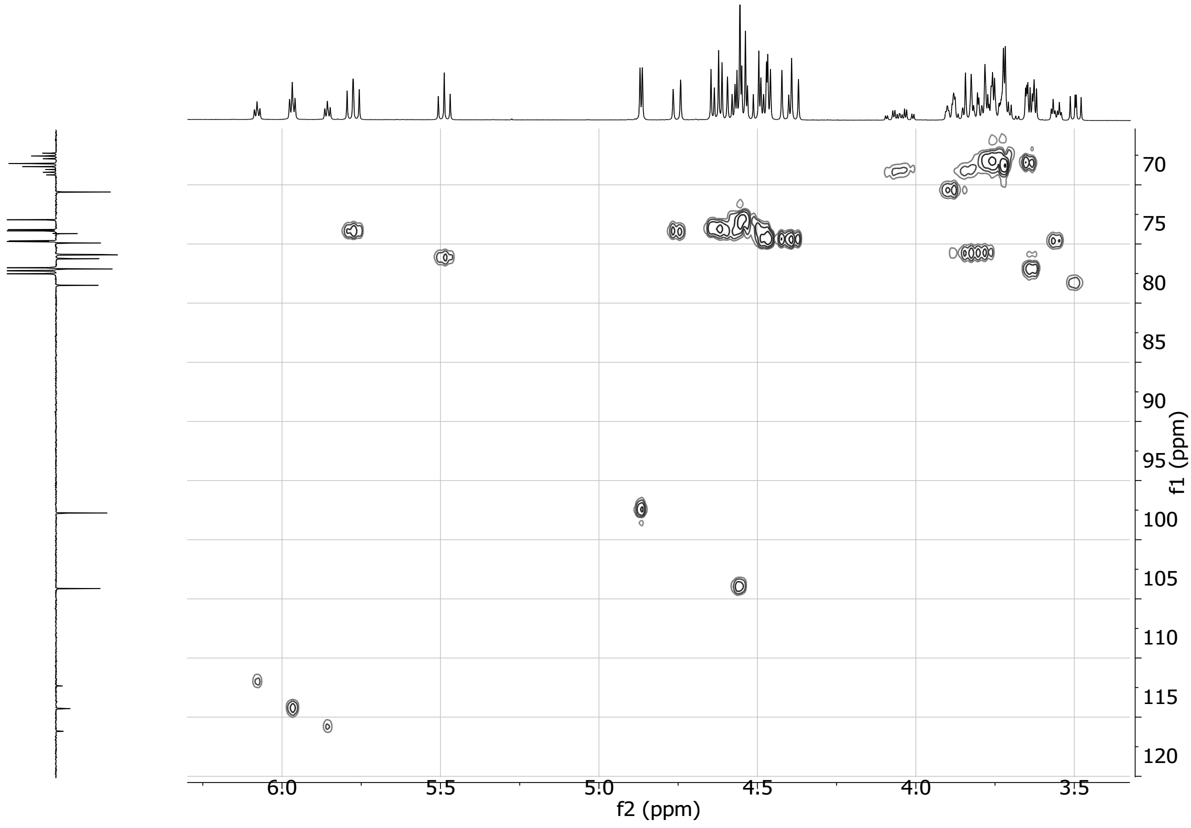

Supplementary Figure 164. HSQC NMR, $CDCl_3$ of compound **S44**

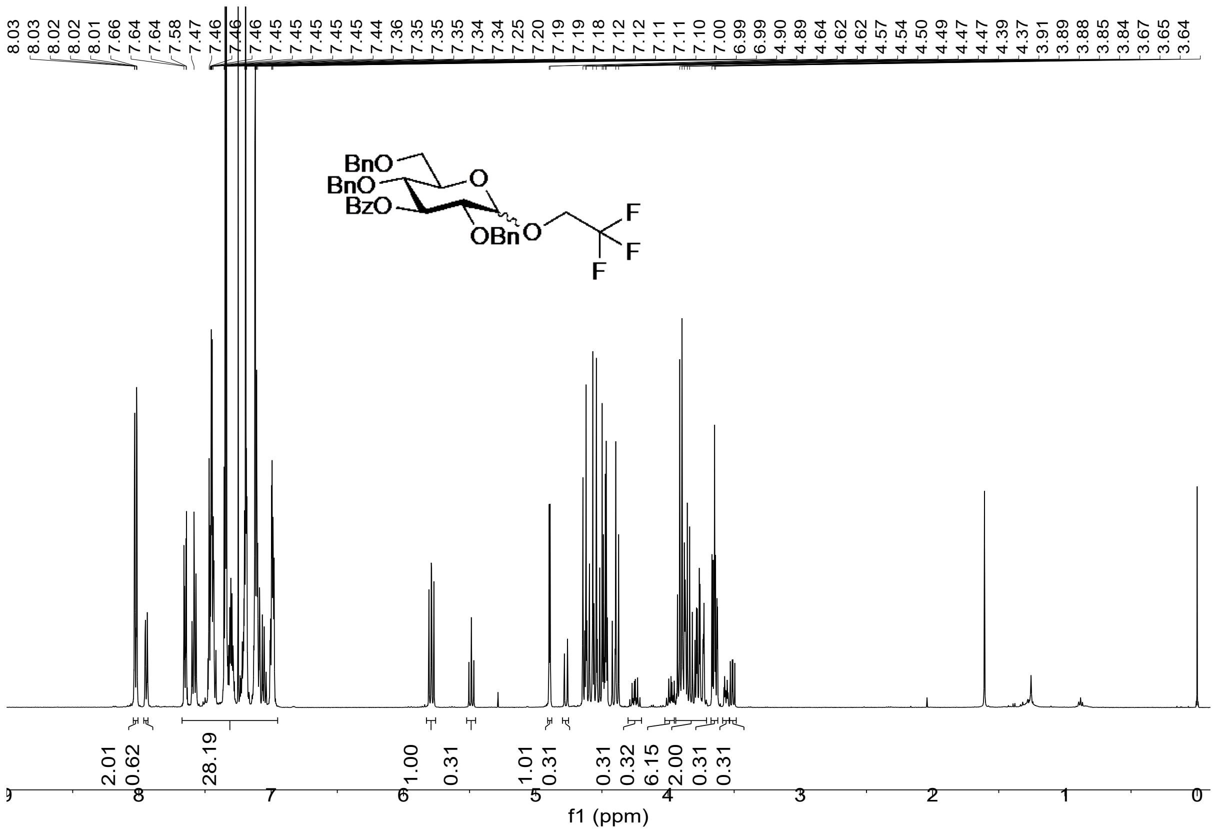

Supplementary Figure 165. ^{1}H NMR, 500 MHz, $CDCl_3$ of compound **S45**

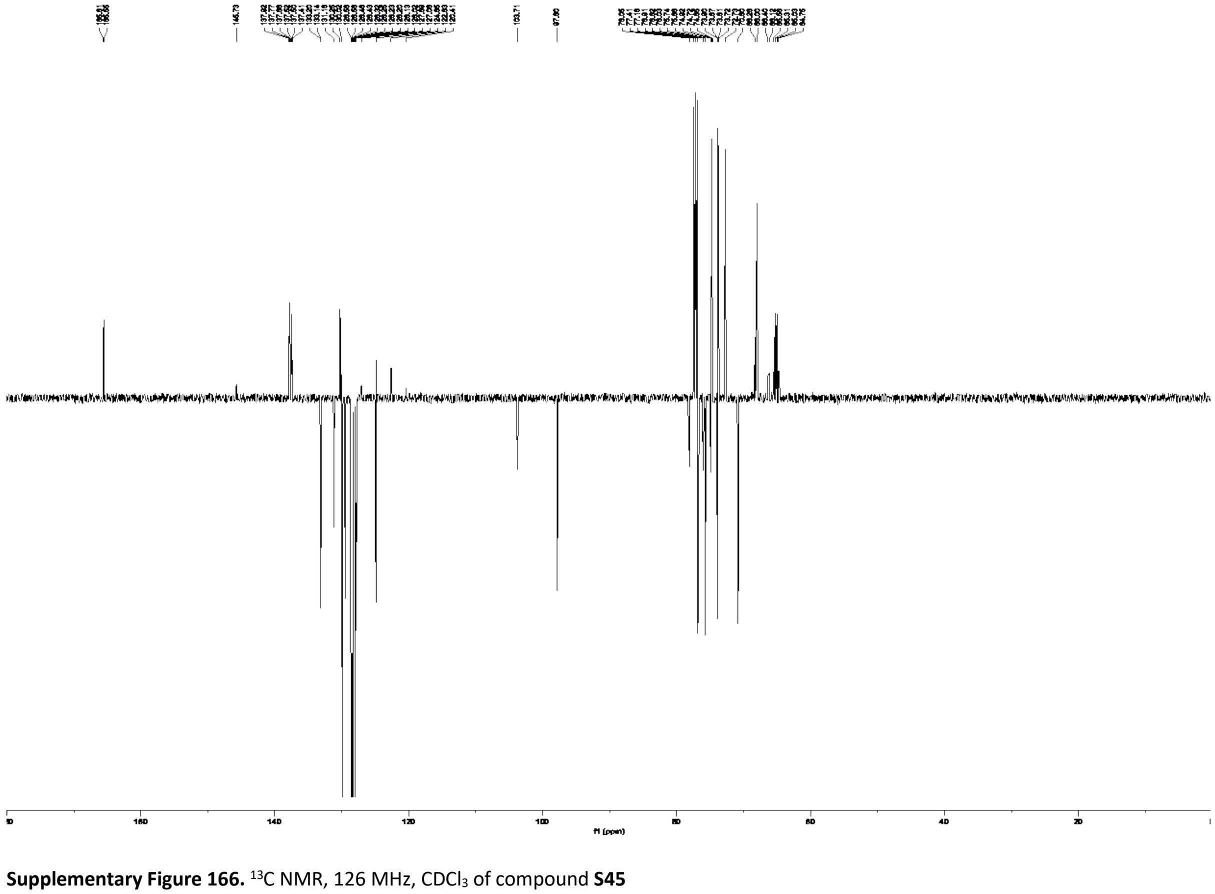

Supplementary Figure 166. ^{13}C NMR, 126 MHz, $CDCl_3$ of compound **S45**

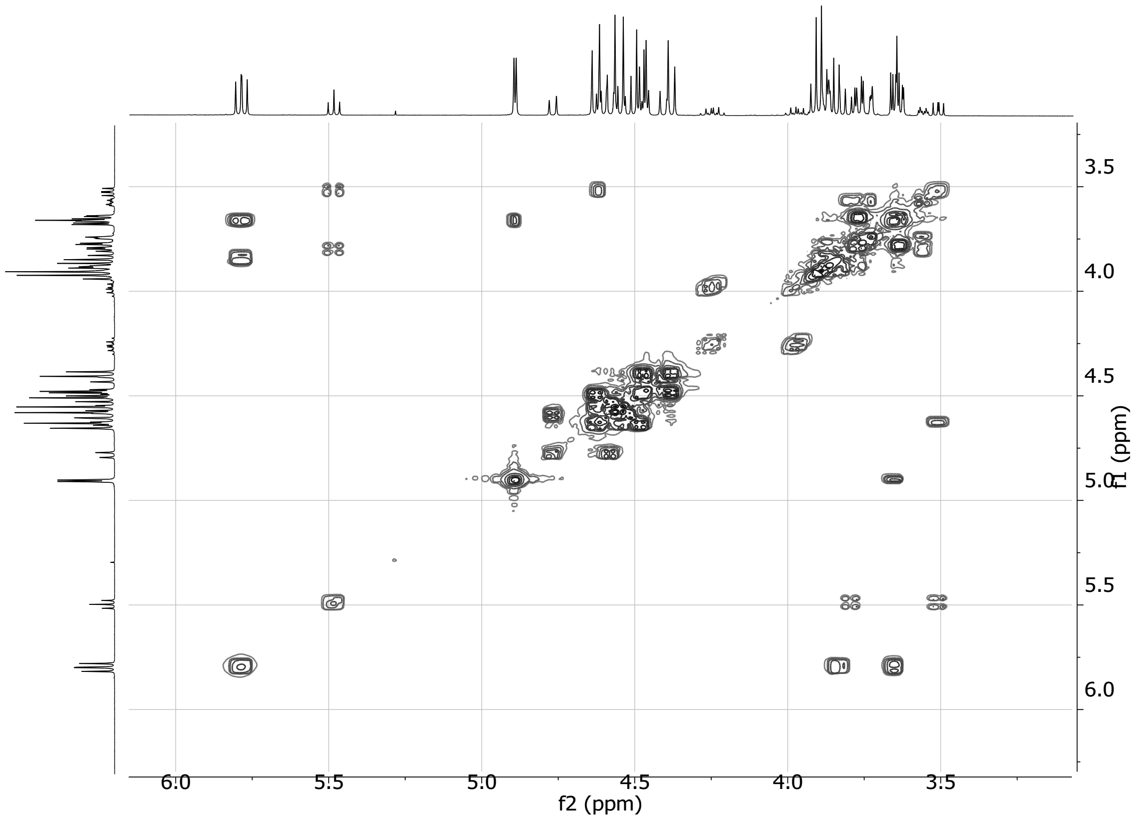

Supplementary Figure 167. HH-COSY NMR, $CDCl_3$ of compound **S45**

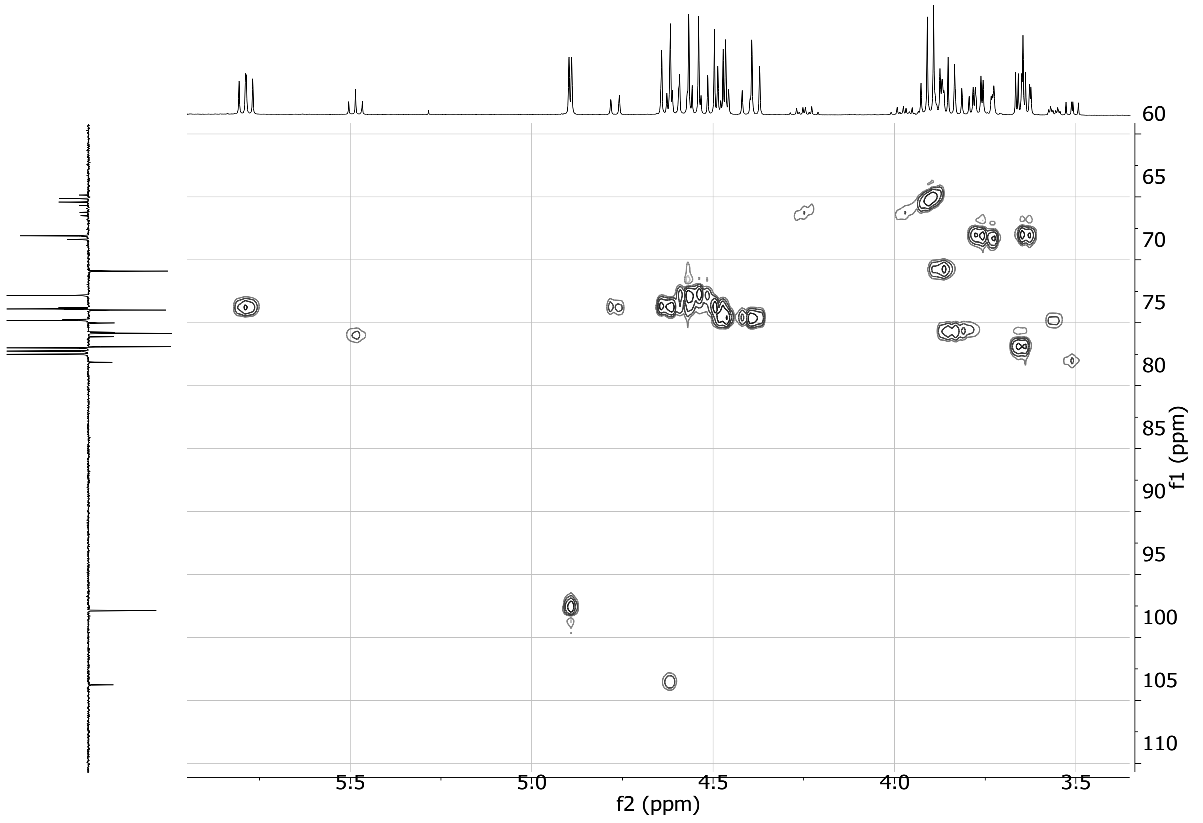

Supplementary Figure 168. HSQC NMR, $CDCl_3$ of compound **S45**

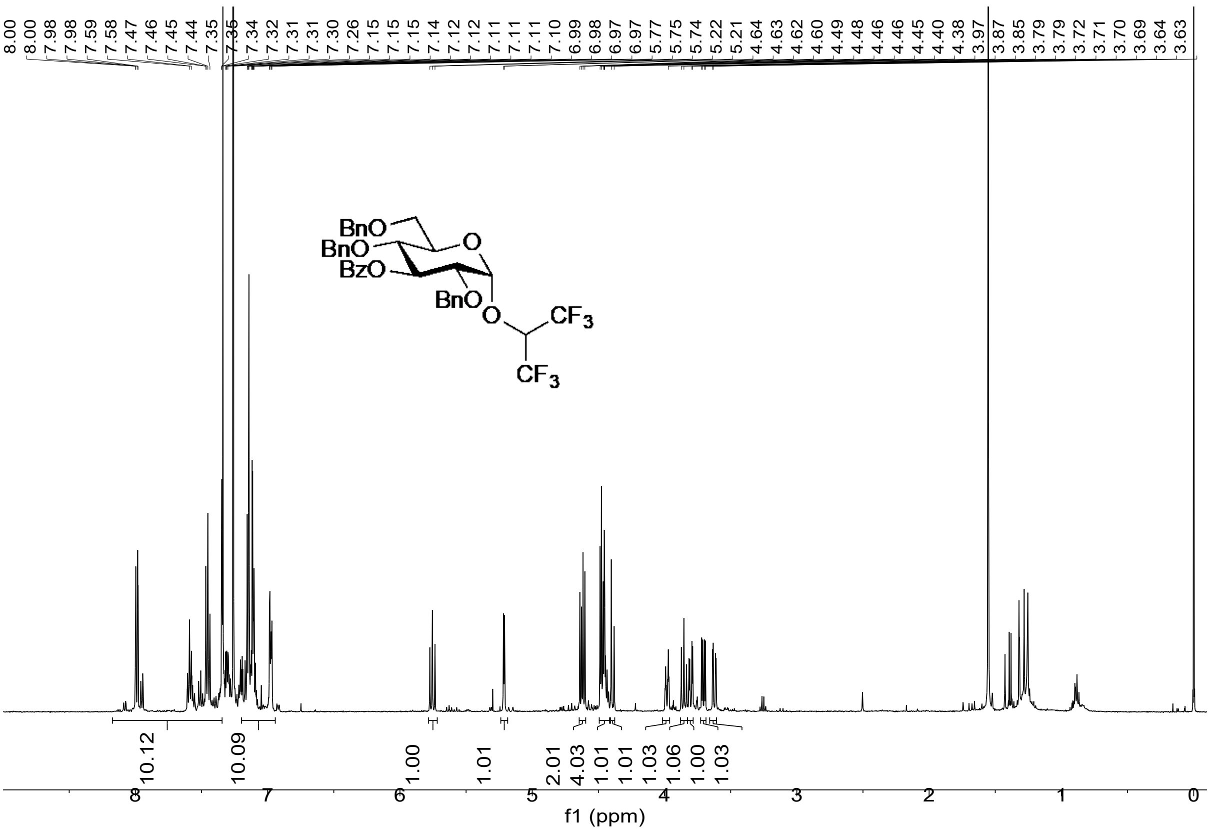

Supplementary Figure 169. ^{1}H NMR, 500 MHz, $CDCl_3$ of compound **S46**

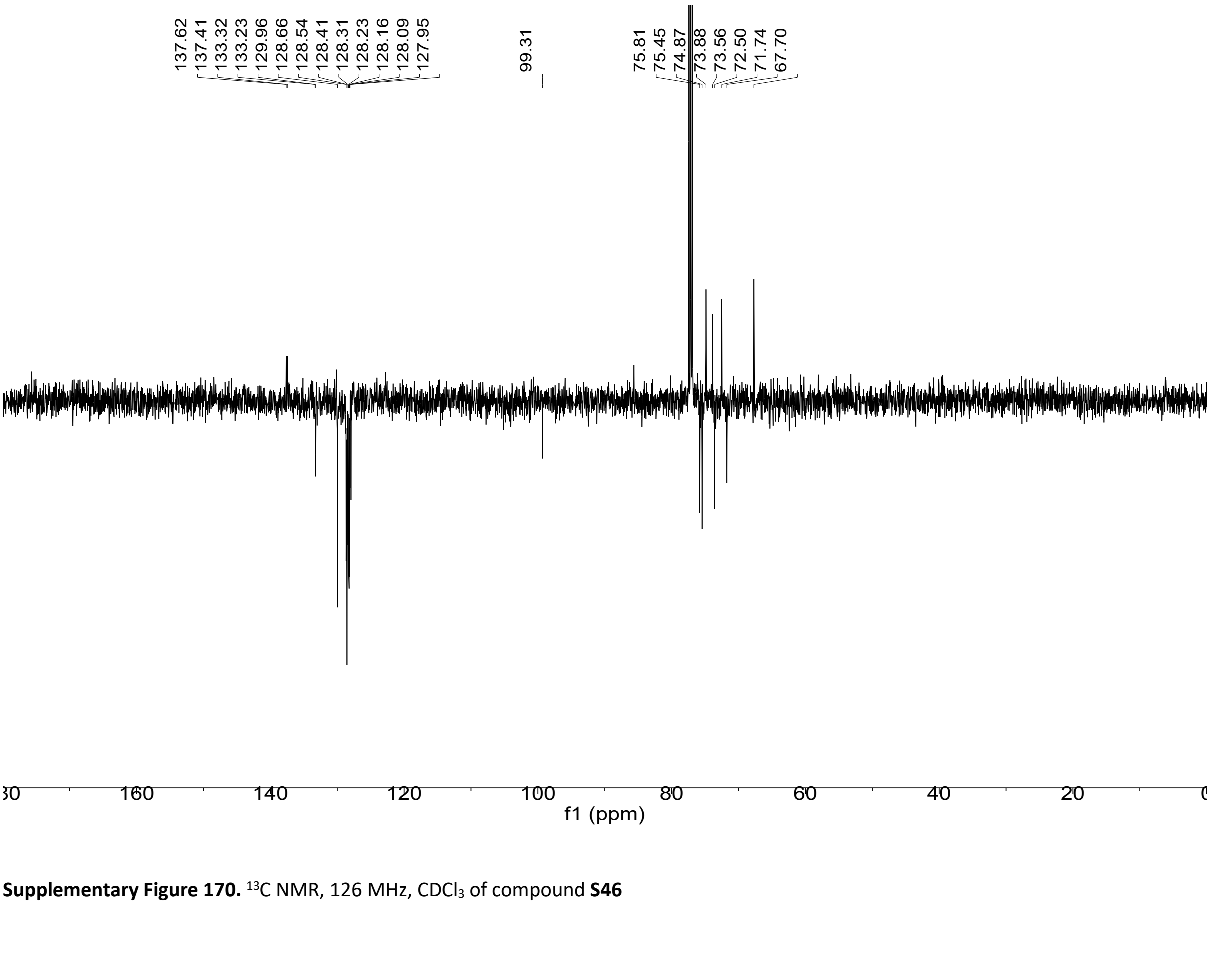

Supplementary Figure 170. ^{13}C NMR, 126 MHz, $CDCl_3$ of compound **S46**

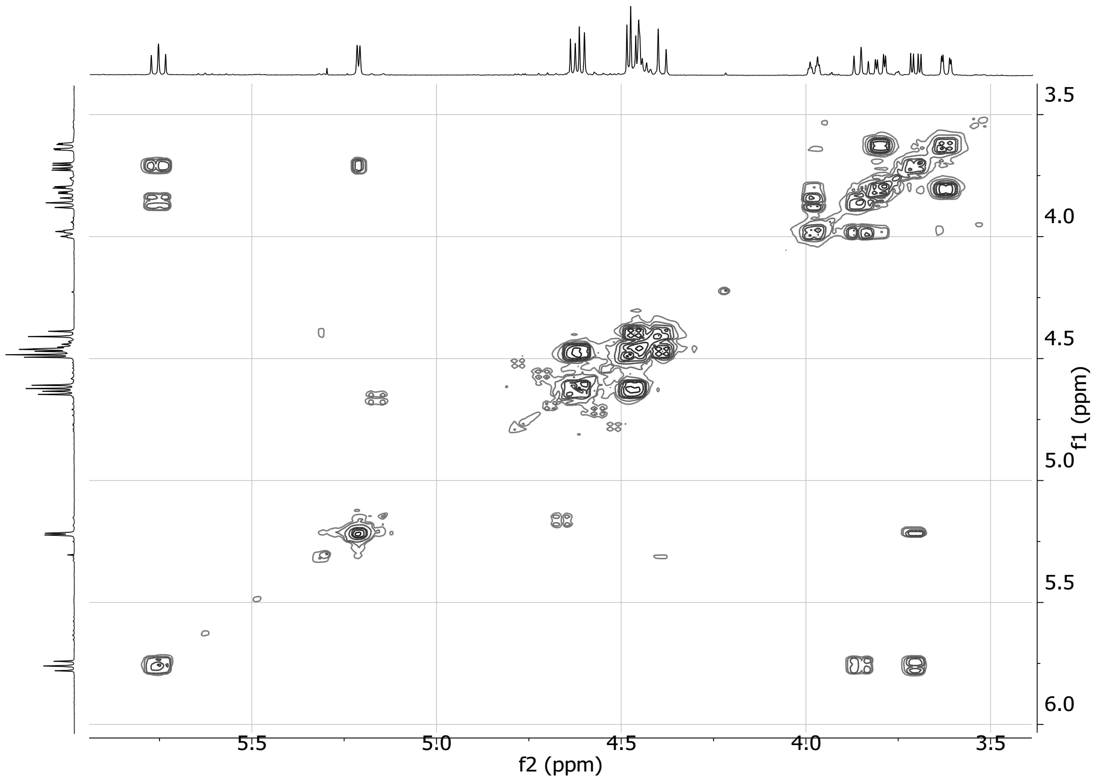

Supplementary Figure 171. HH-COSY NMR, $CDCl_3$ of compound **S46**

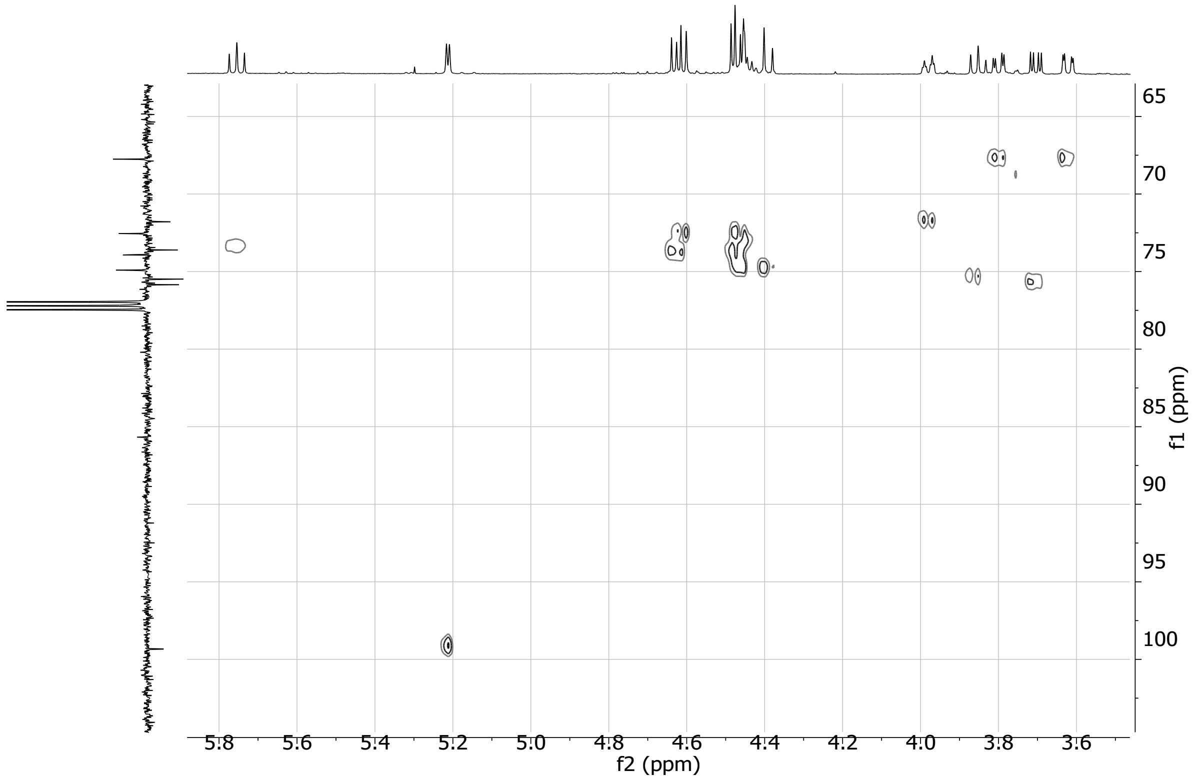

Supplementary Figure 172. HSQC NMR, $CDCl_3$ of compound **S46**

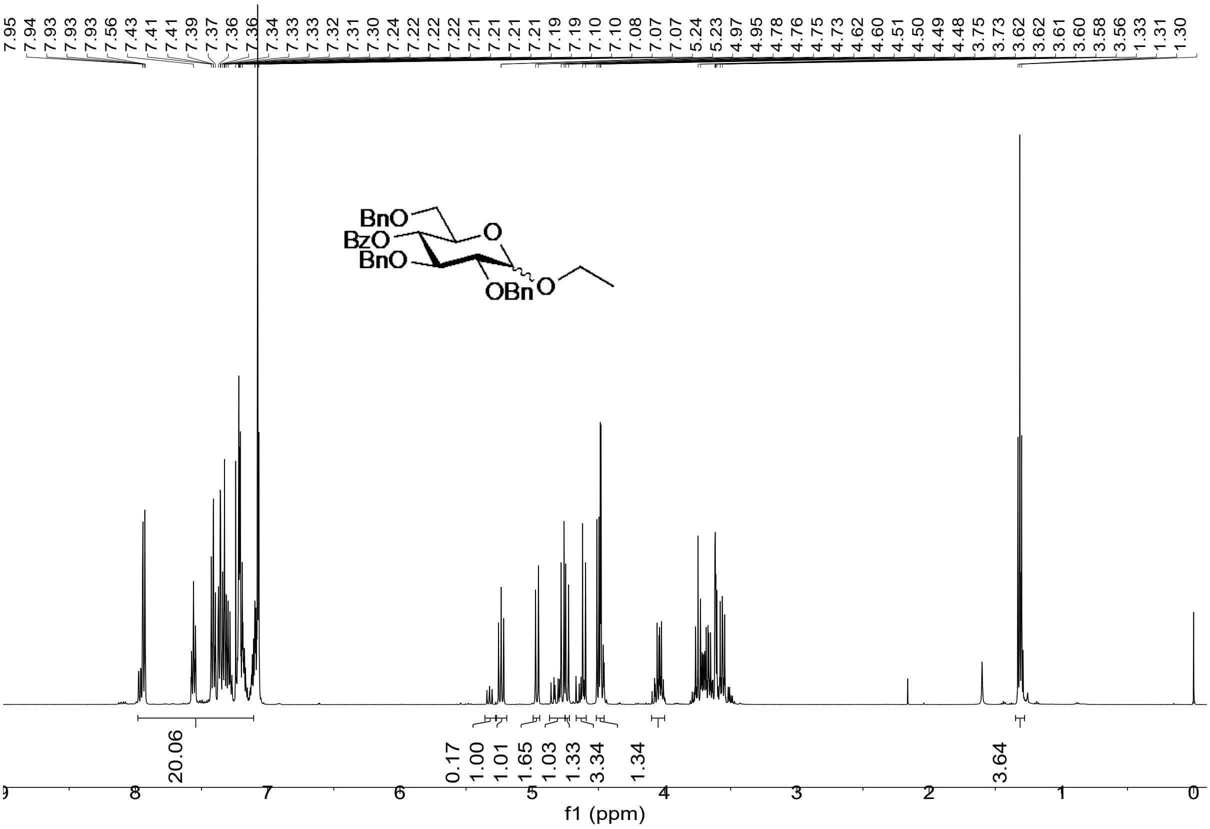

Supplementary Figure 173. ^{1}H NMR, 500 MHz, $CDCl_3$ of compound **S47**

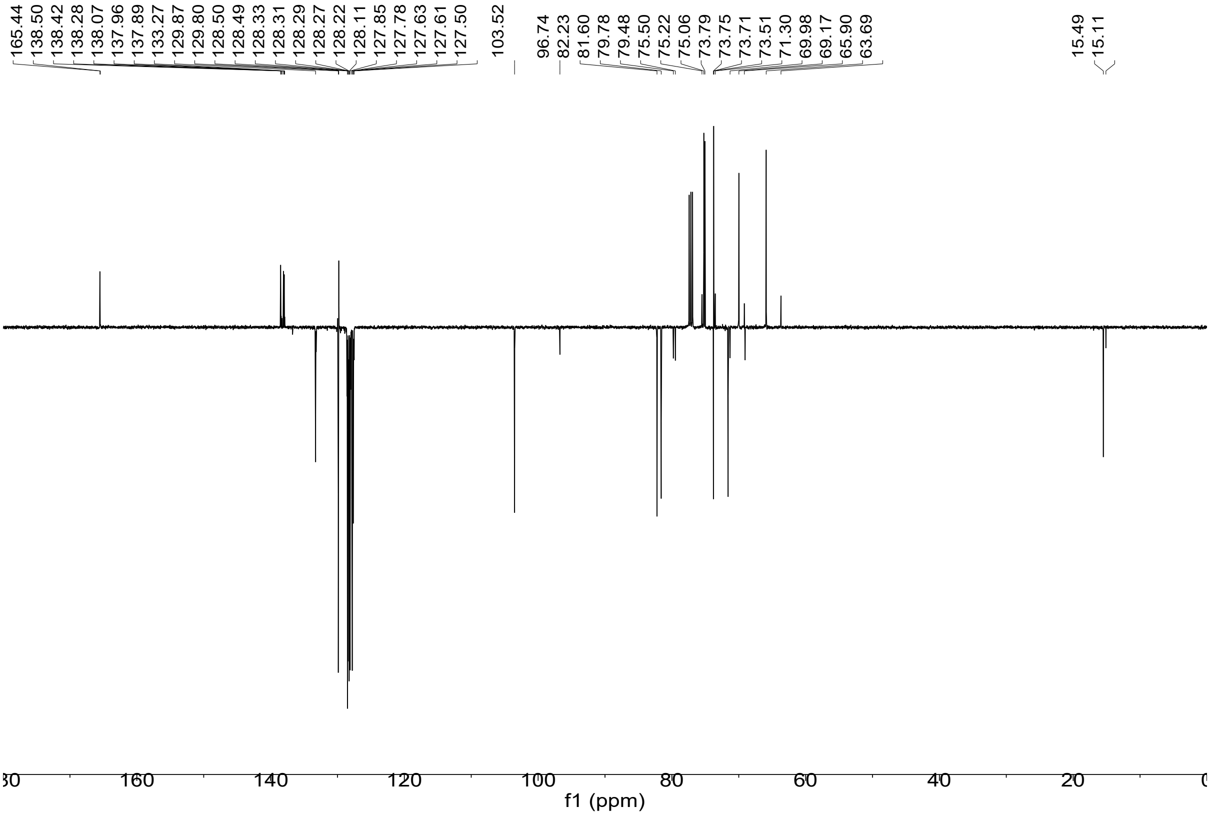

Supplementary Figure 174. ^{13}C NMR, 126 MHz, $CDCl_3$ of compound **S47**

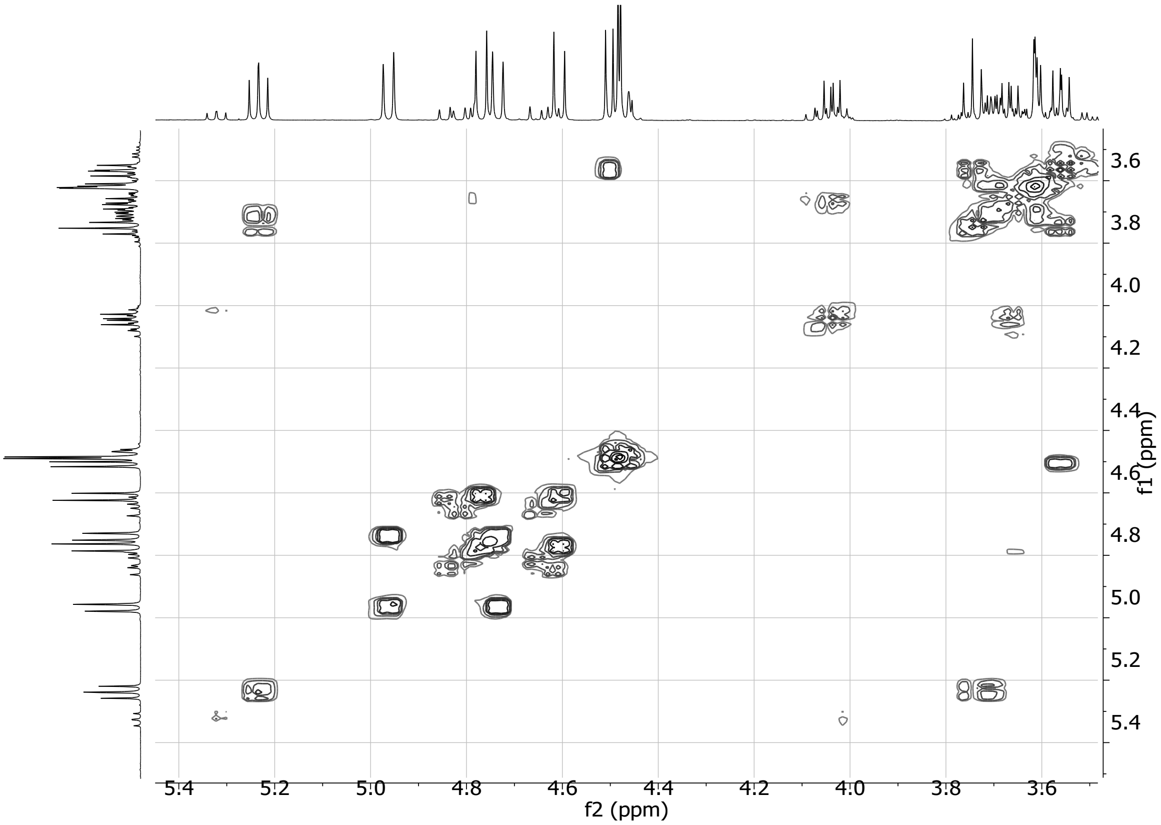

Supplementary Figure 175. HH-COSY NMR, $CDCl_3$ of compound **S47**

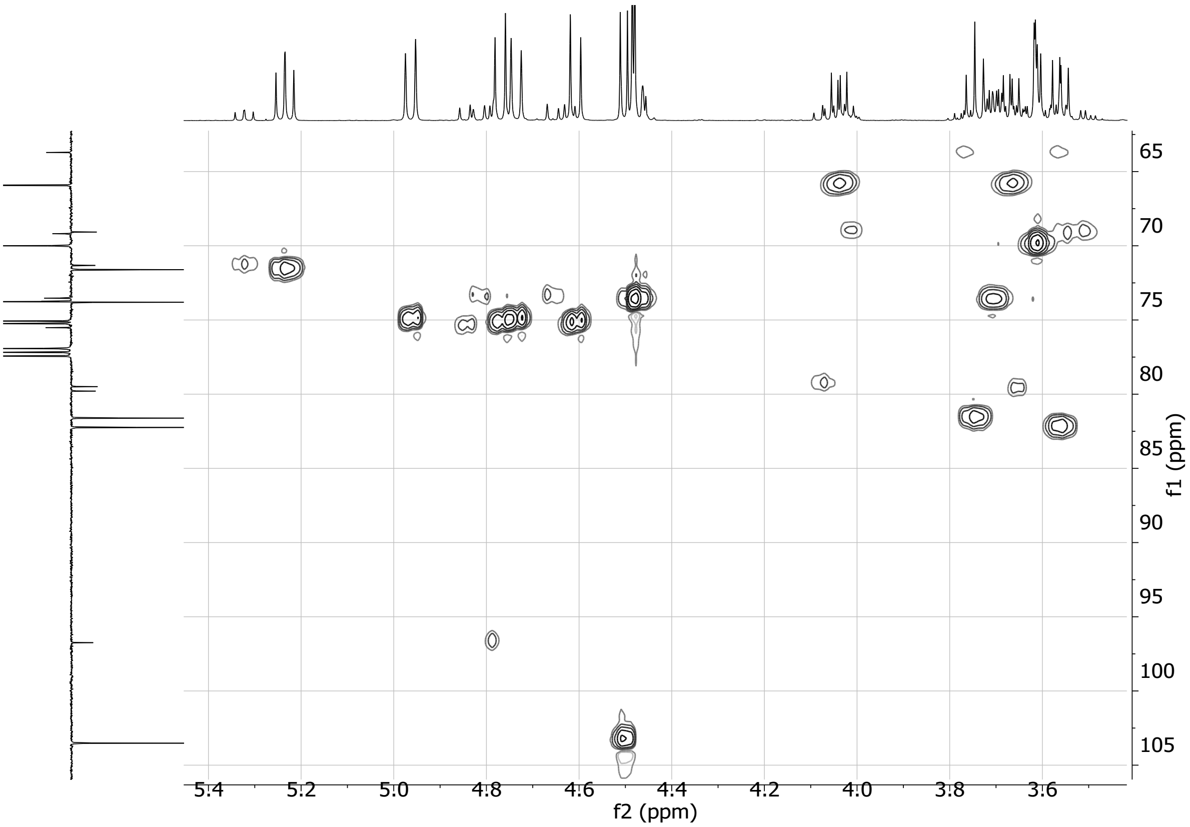

Supplementary Figure 176. HSQC NMR, $CDCl_3$ of compound **S47**

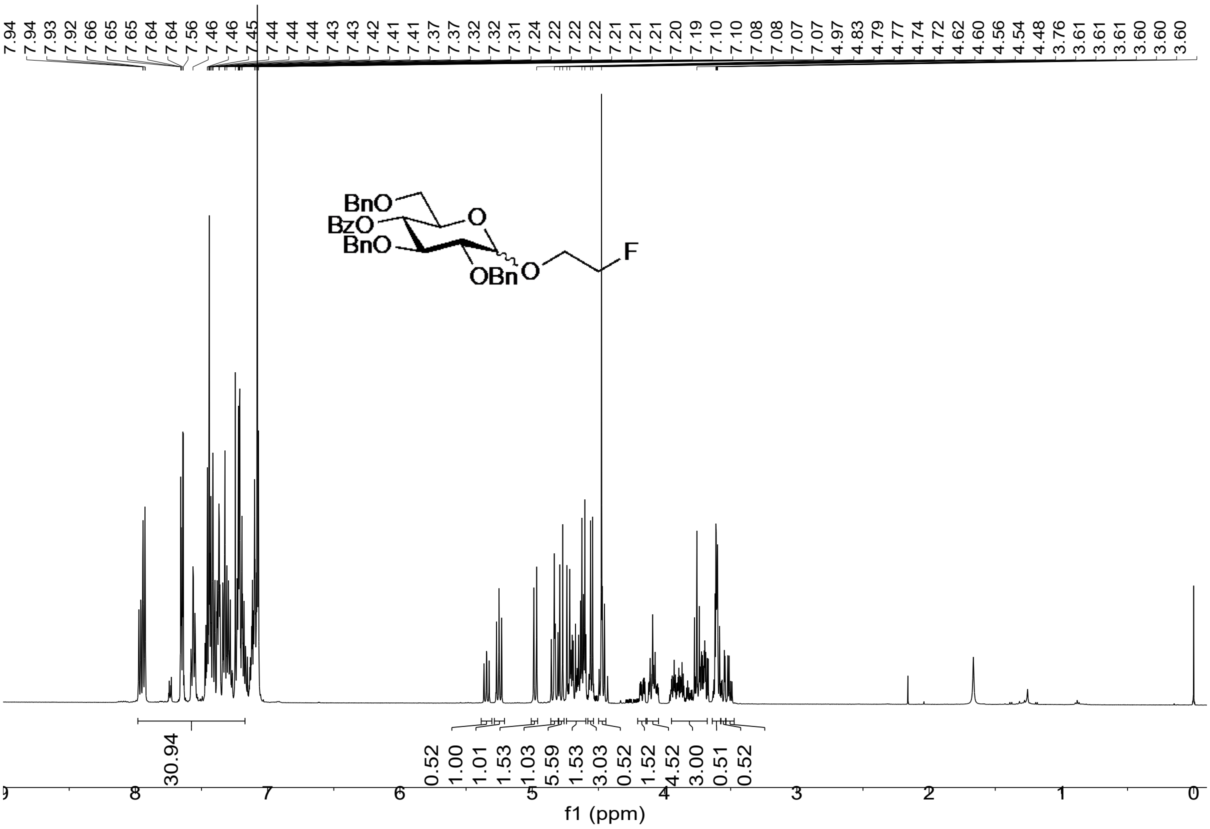

Supplementary Figure 177. ^{1}H NMR, 500 MHz, $CDCl_3$ of compound **S48**

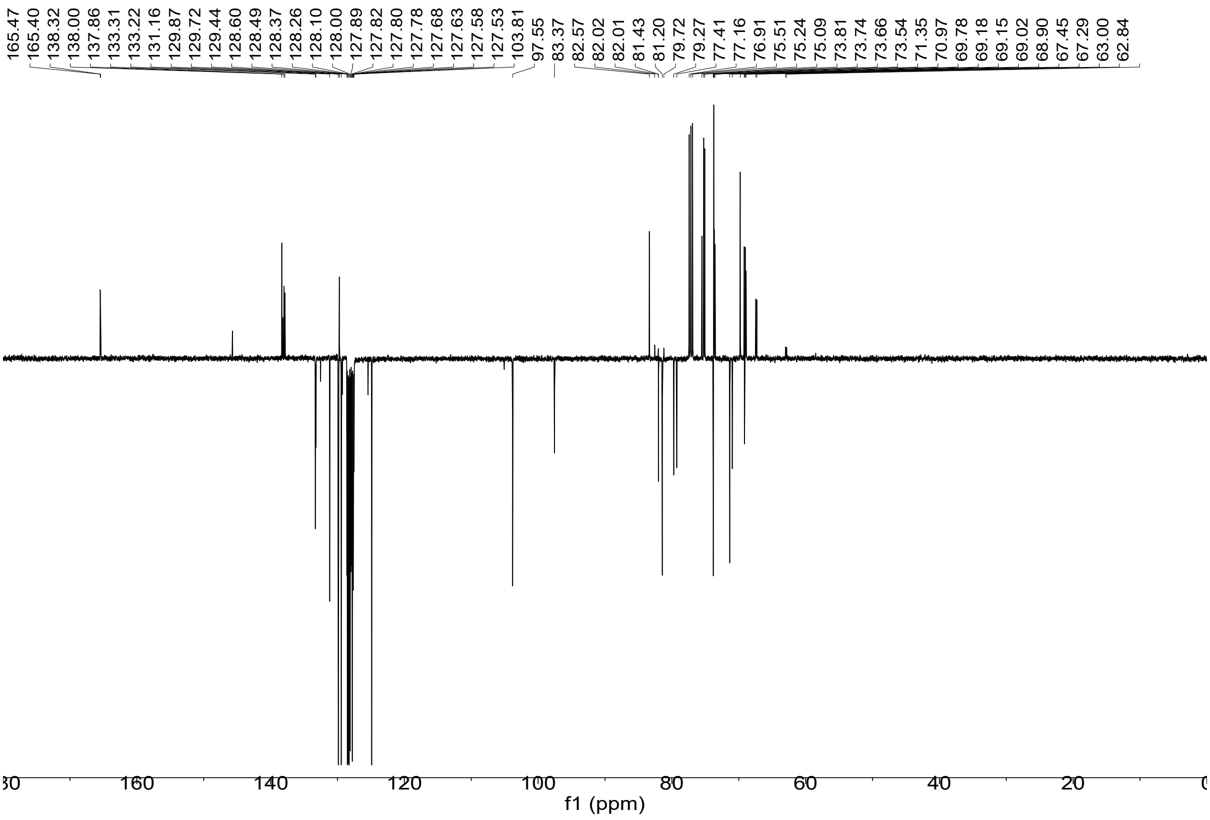

Supplementary Figure 178. ^{13}C NMR, 126 MHz, $CDCl_3$ of compound **S48**

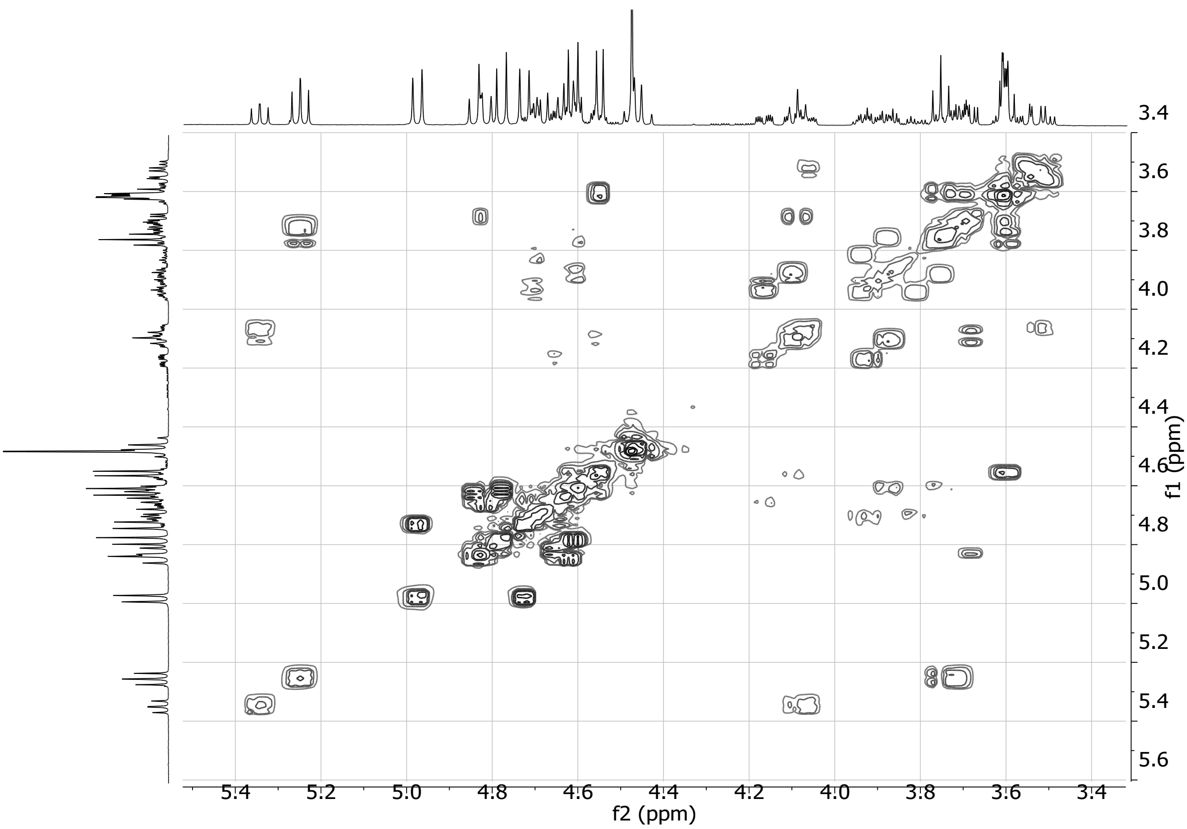

Supplementary Figure 179. HH-COSY NMR, $CDCl_3$ of compound **S48**

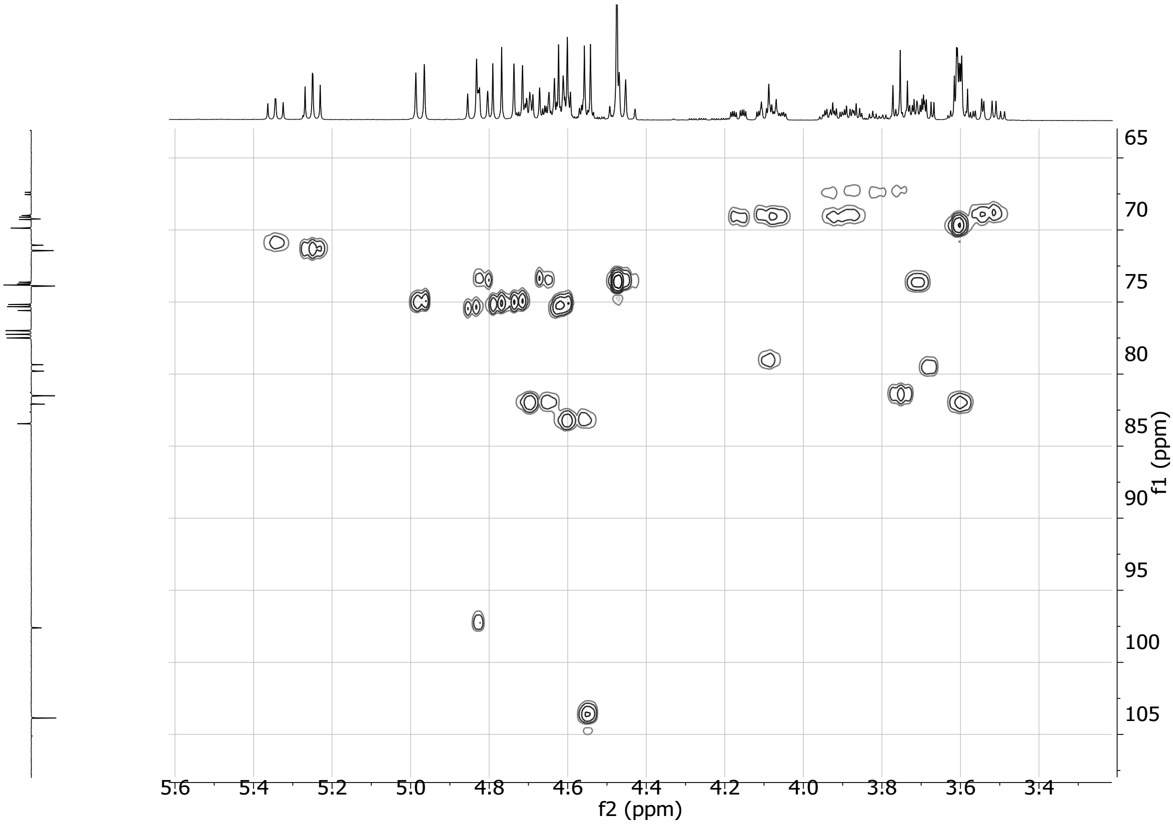

Supplementary Figure 180. HSQC NMR, $CDCl_3$ of compound **S48**

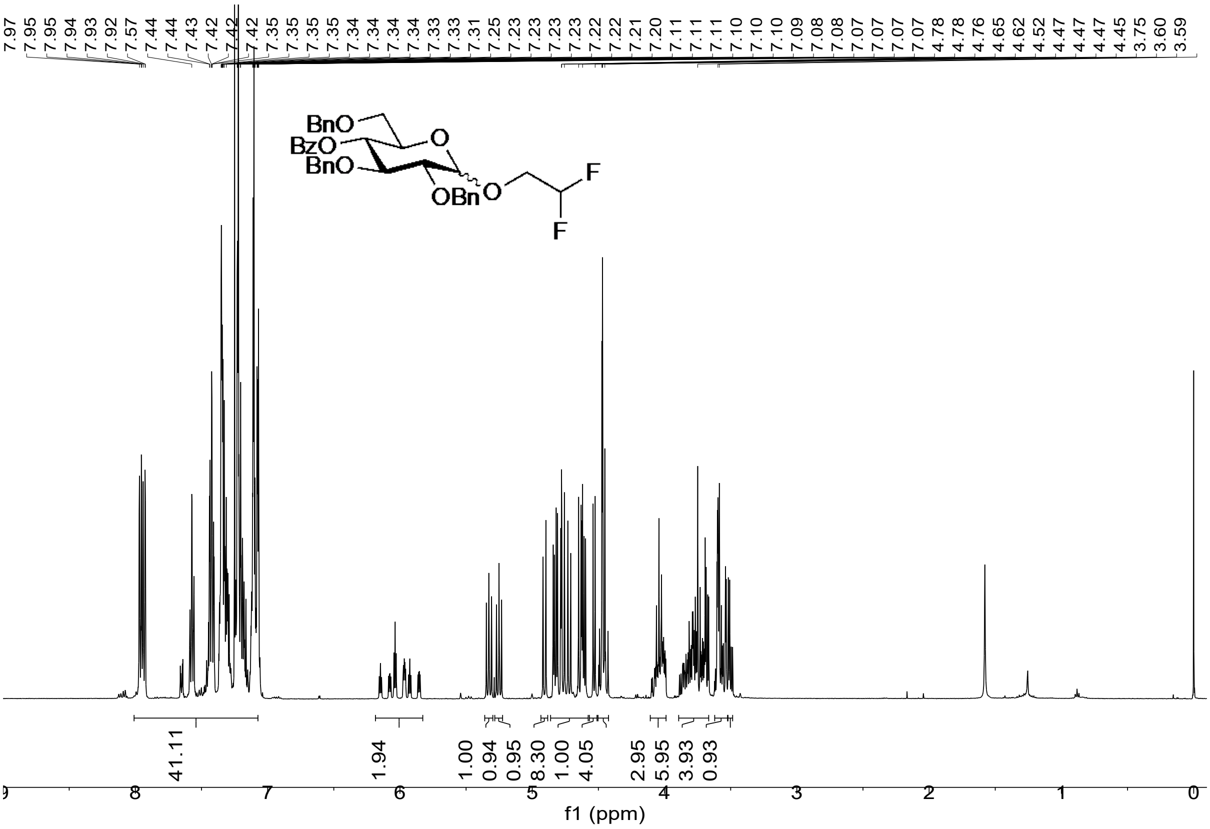

Supplementary Figure 181. ^{1}H NMR, 500 MHz, $CDCl_3$ of compound **S49**

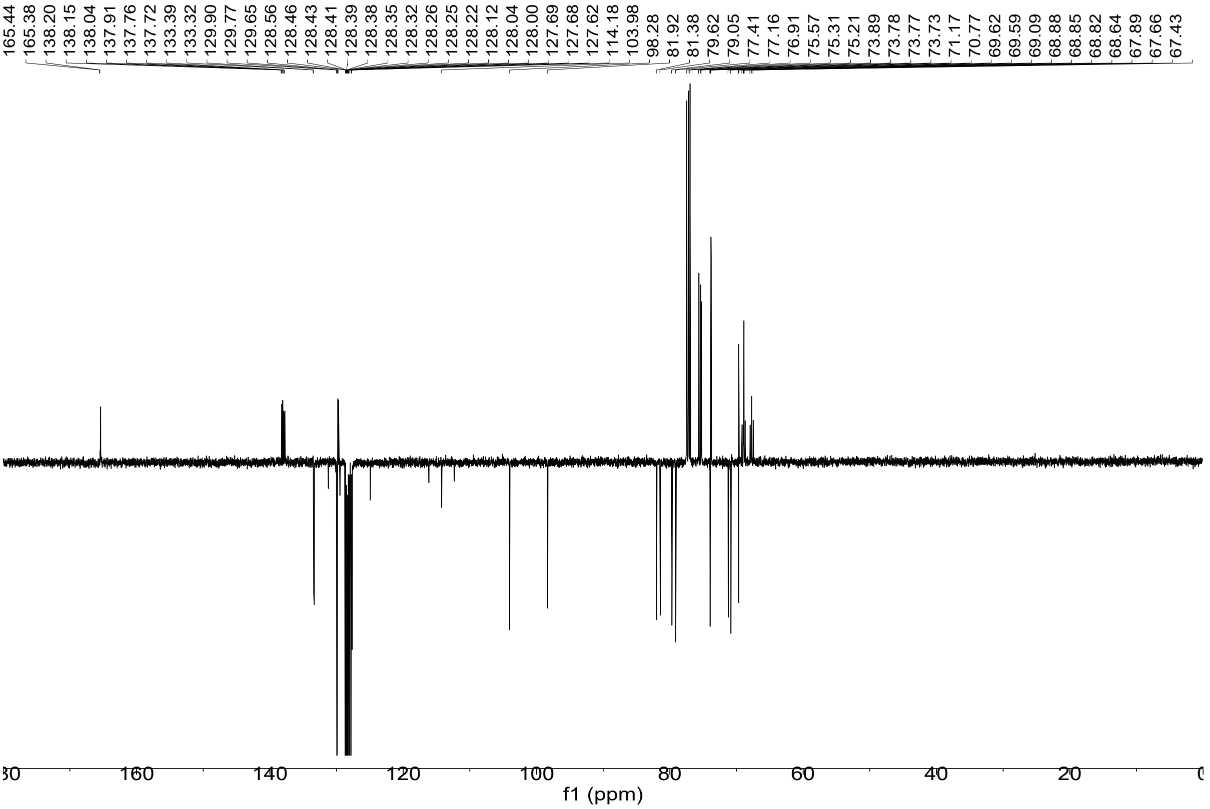

Supplementary Figure 182. ^{13}C NMR, 126 MHz, $CDCl_3$ of compound **S49**

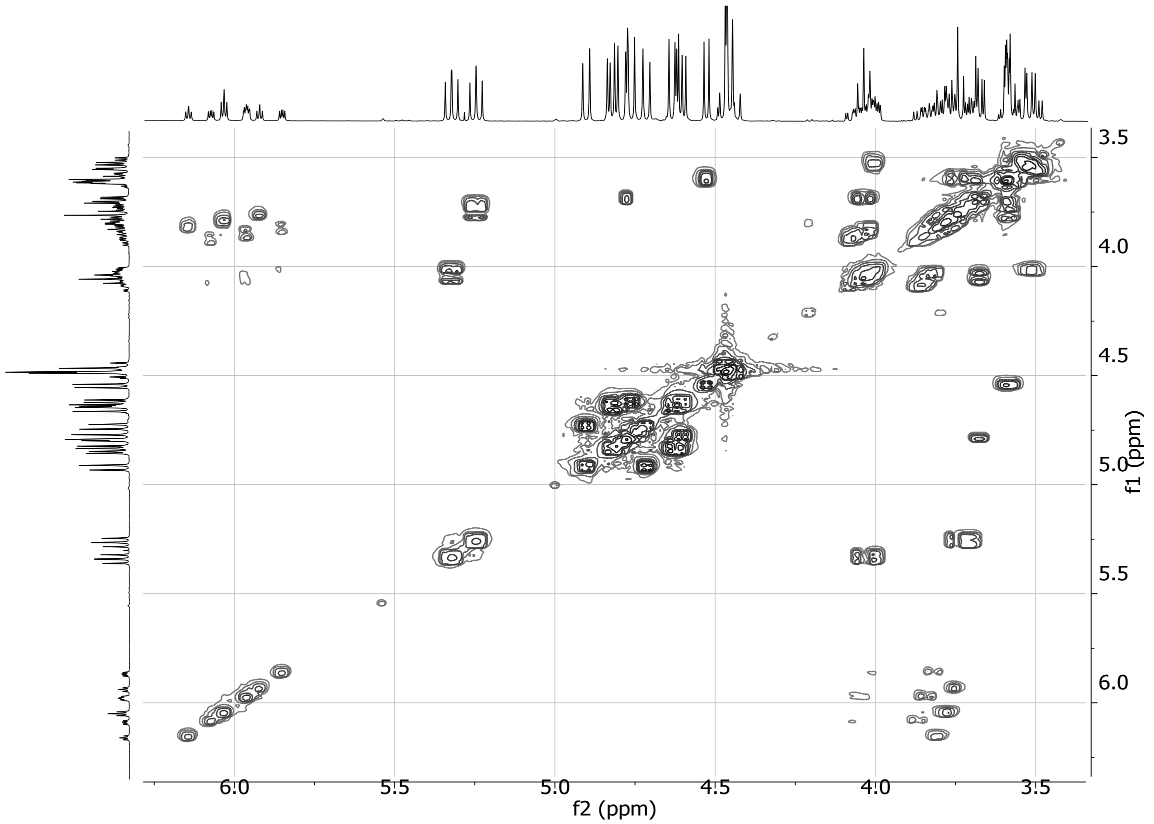

Supplementary Figure 183. HH-COSY NMR, $CDCl_3$ of compound **S49**

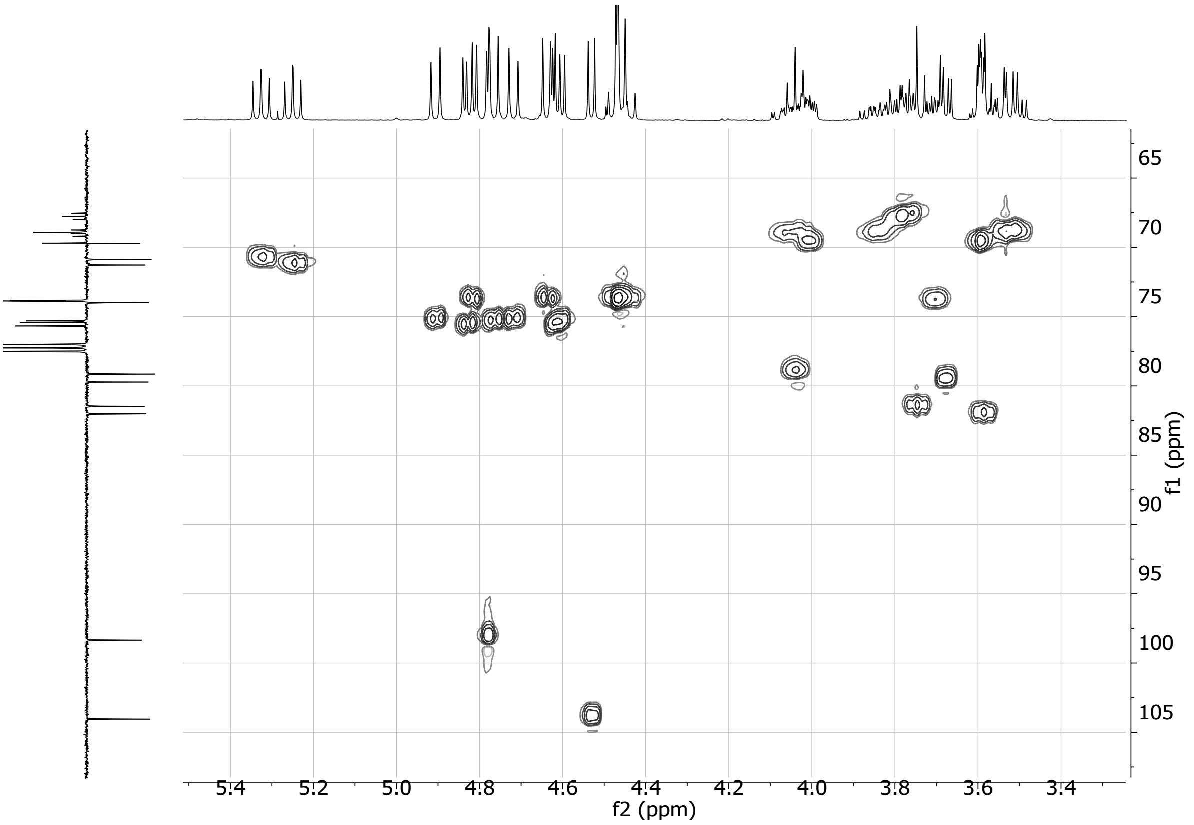

Supplementary Figure 184. HSQC NMR, $CDCl_3$ of compound **S49**

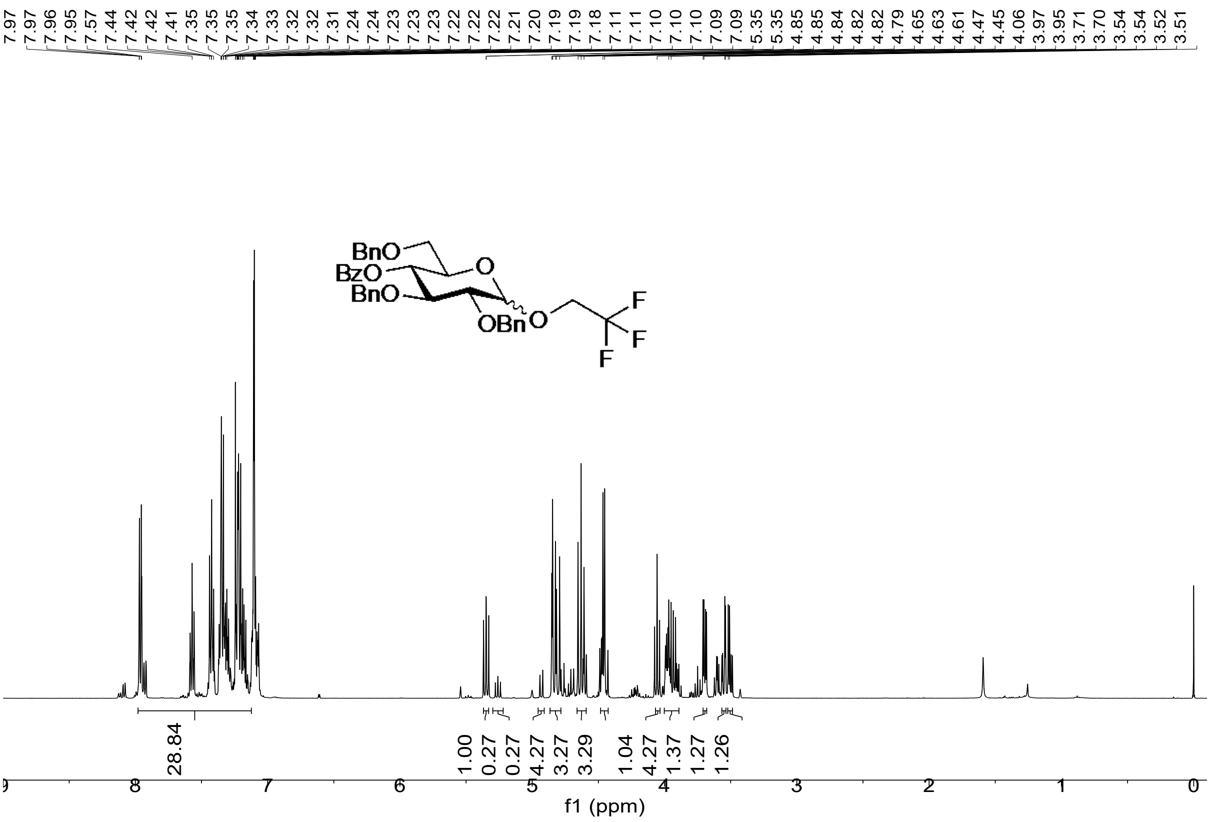

Supplementary Figure 185. ^{1}H NMR, 500 MHz, $CDCl_3$ of compound **S50**

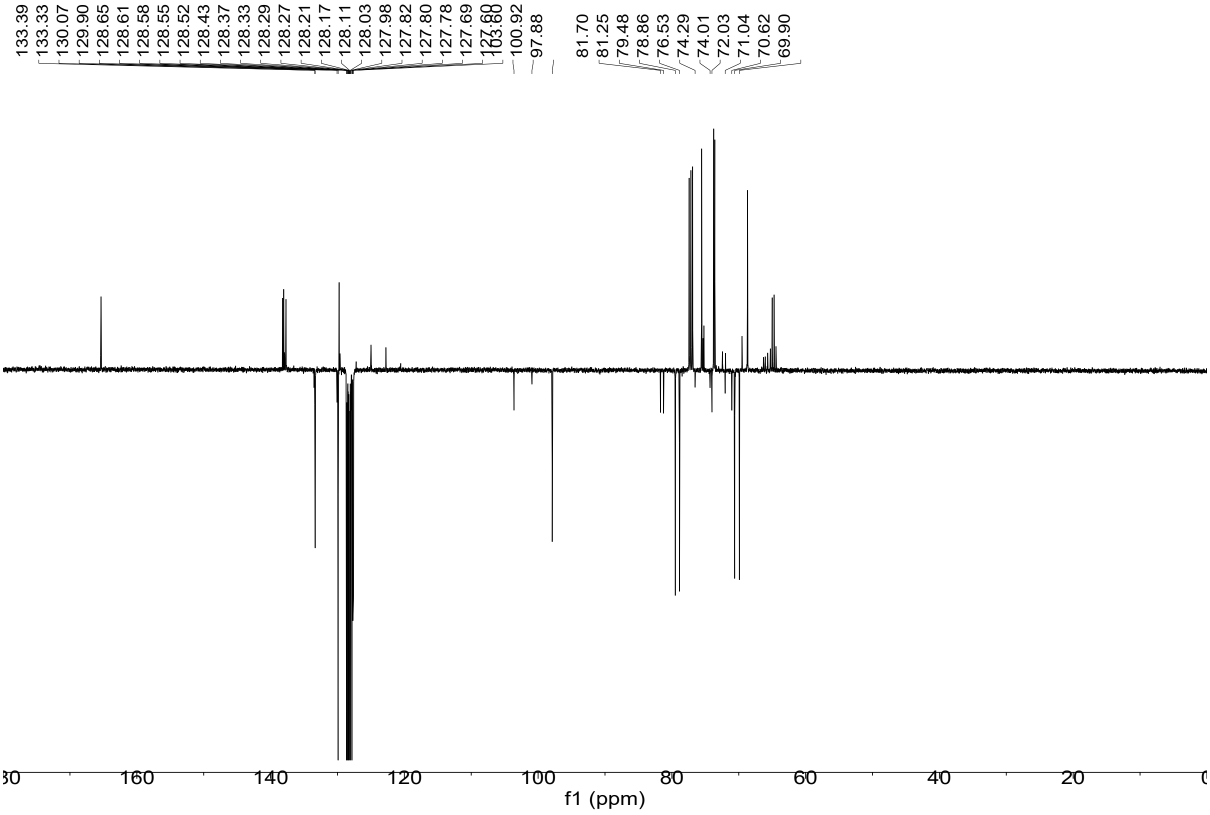

Supplementary Figure 186. ^{13}C NMR, 126 MHz, $CDCl_3$ of compound **S50**

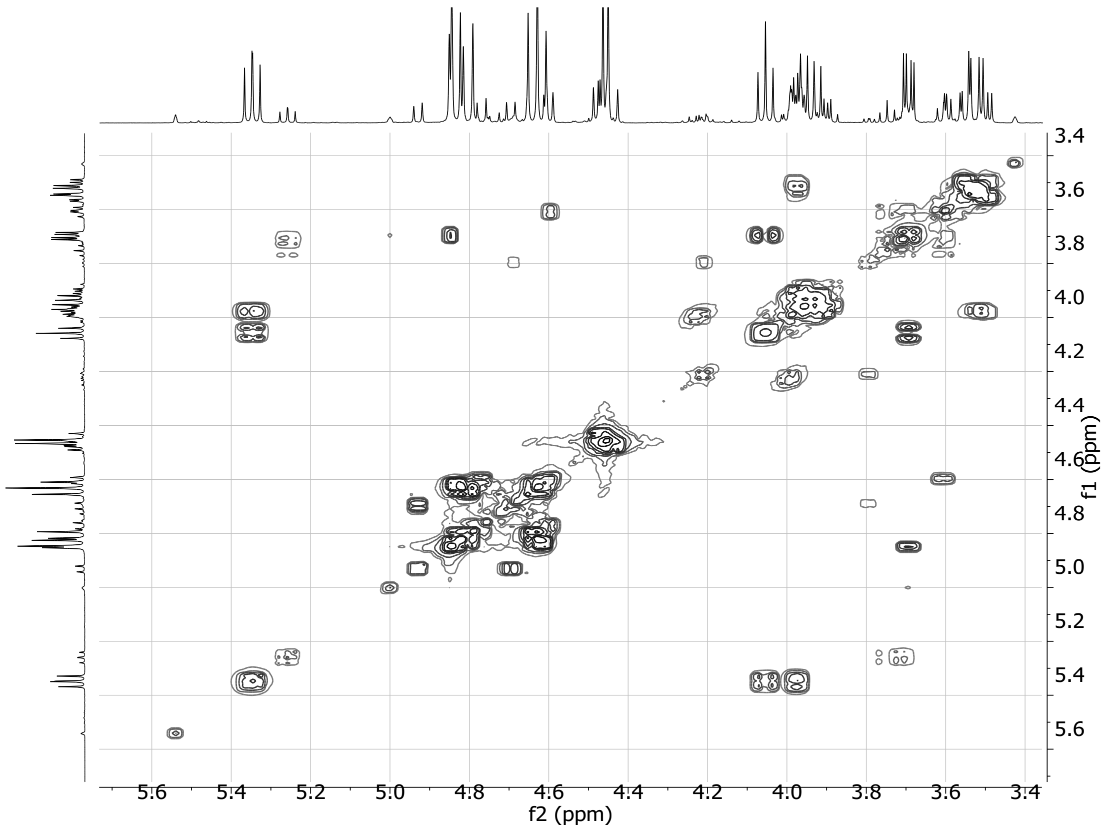

Supplementary Figure 187. HH-COSY NMR, $CDCl_3$ of compound **S50**

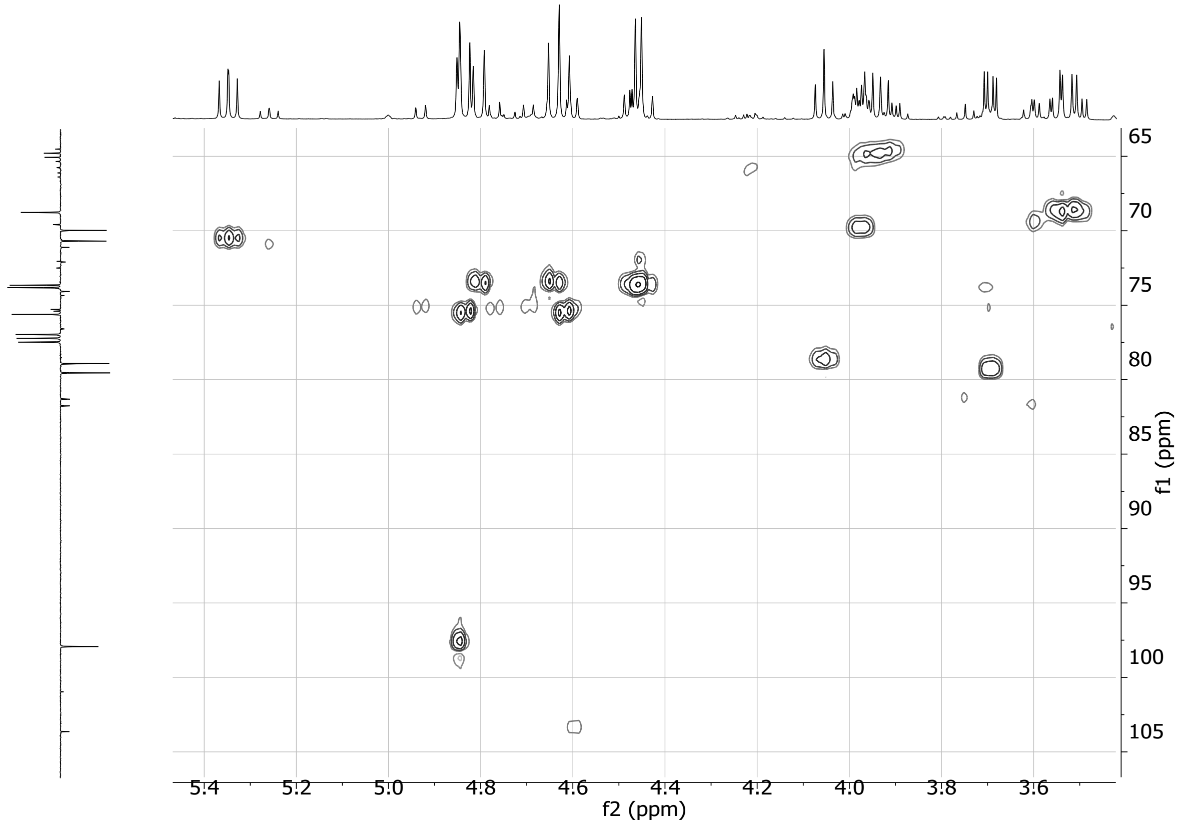

Supplementary Figure 188. HSQC NMR, $CDCl_3$ of compound **S50**

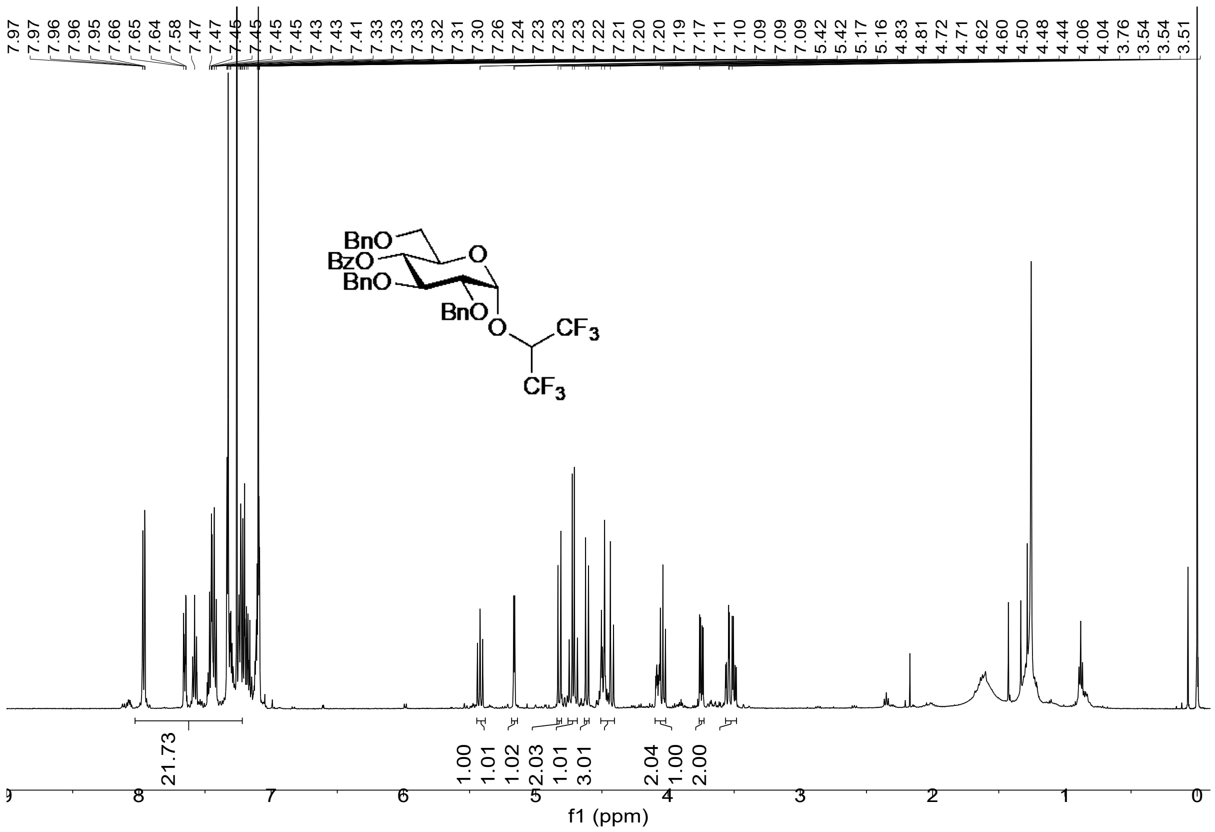

Supplementary Figure 189. ^{1}H NMR, 500 MHz, $CDCl_3$ of compound **S51**

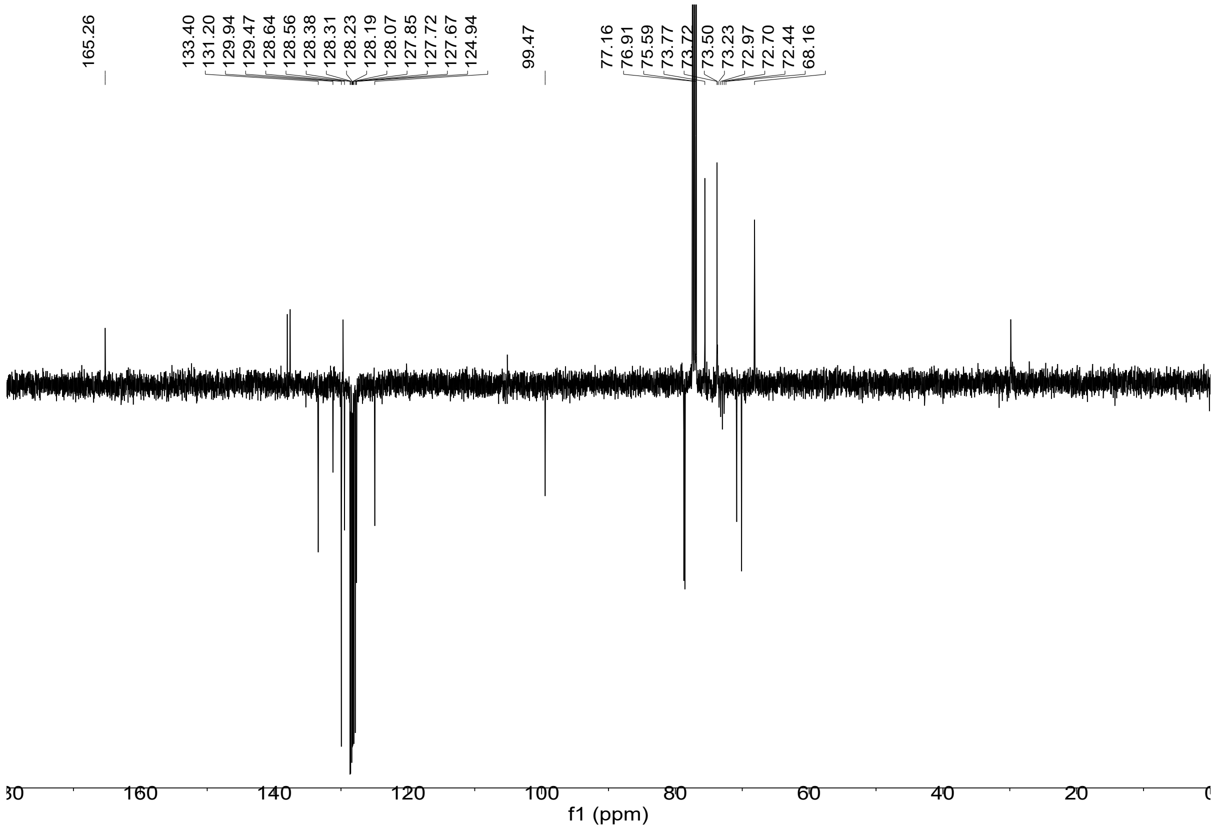

Supplementary Figure 190. ^{13}C NMR, 126 MHz, $CDCl_3$ of compound **S51**

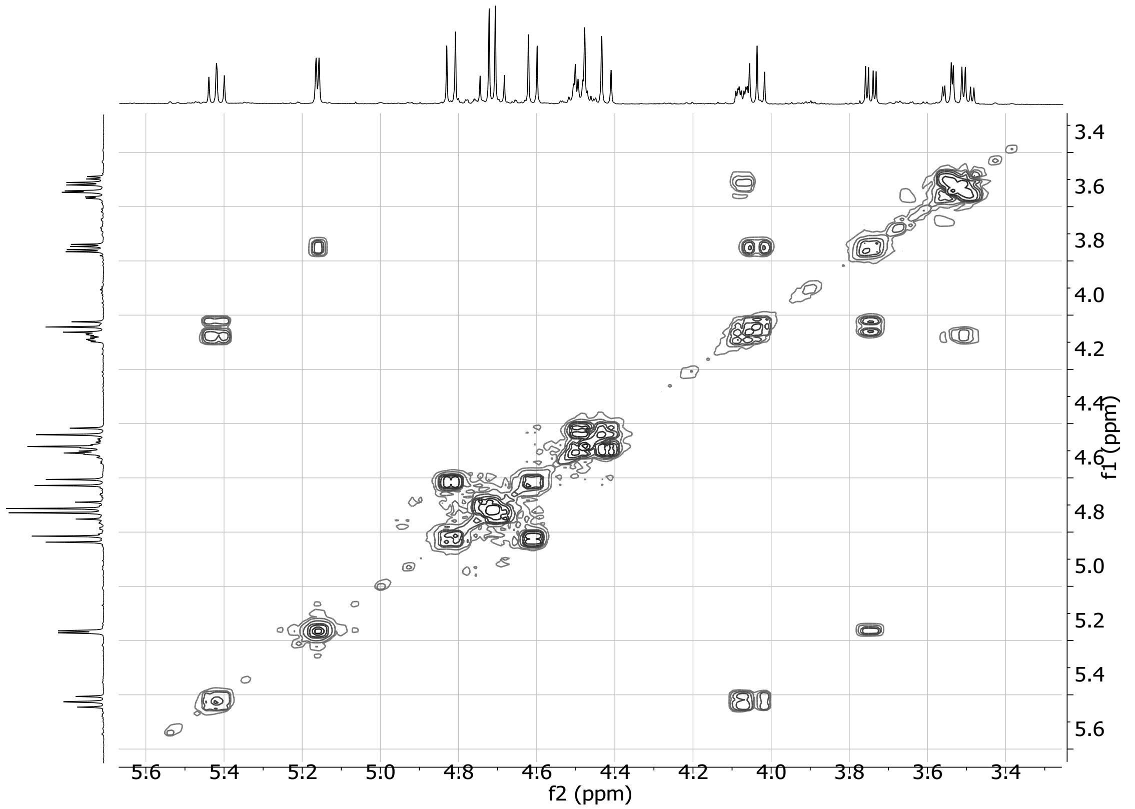

Supplementary Figure 191. HH-COSY NMR, $CDCl_3$ of compound **S51**

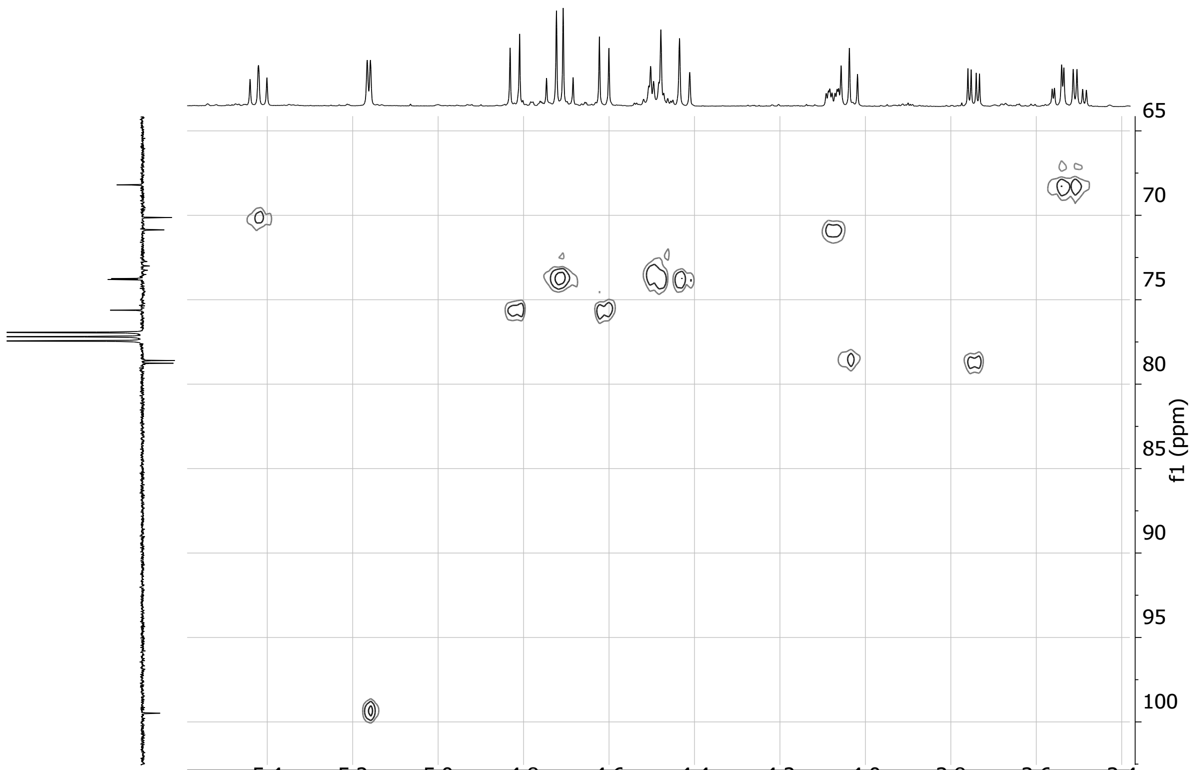

Supplementary Figure 192. HSQC NMR, $CDCl_3$ of compound **S51**

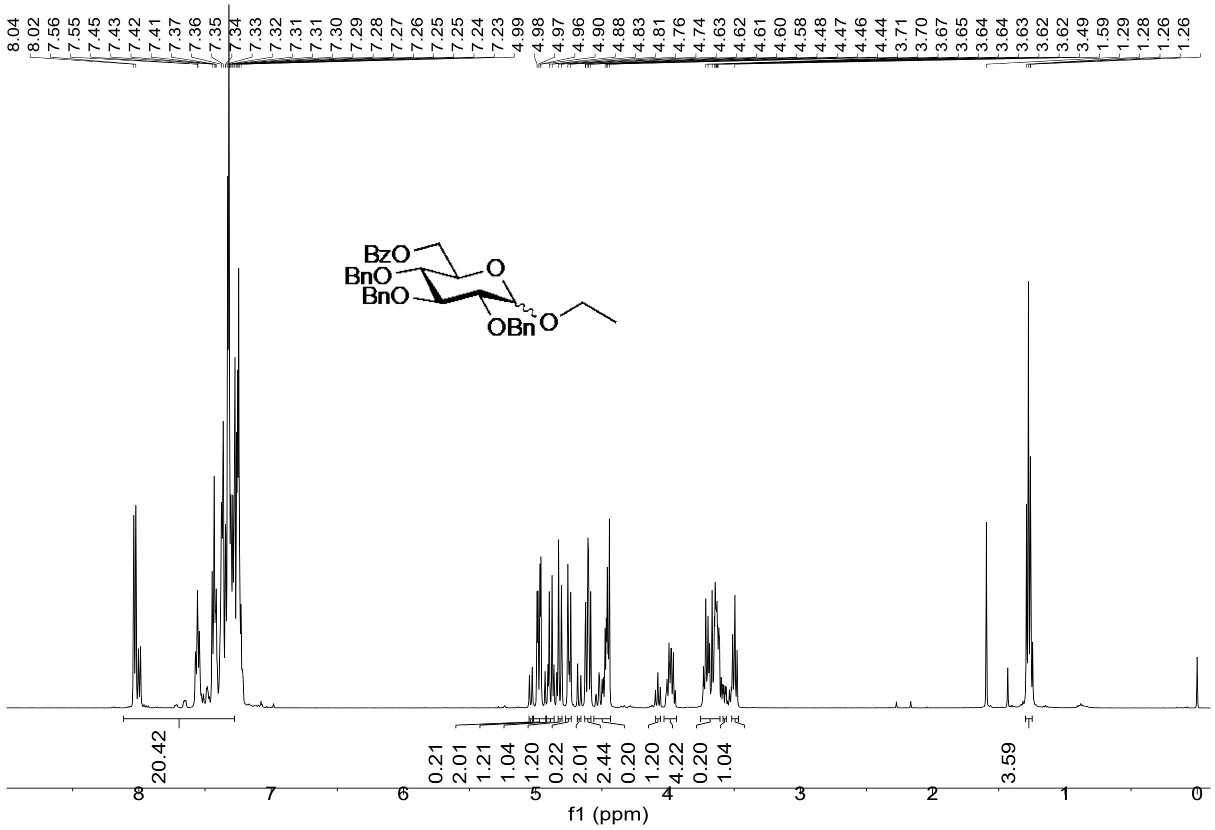

Supplementary Figure 193. ^{1}H NMR, 500 MHz, $CDCl_3$ of compound **S52**

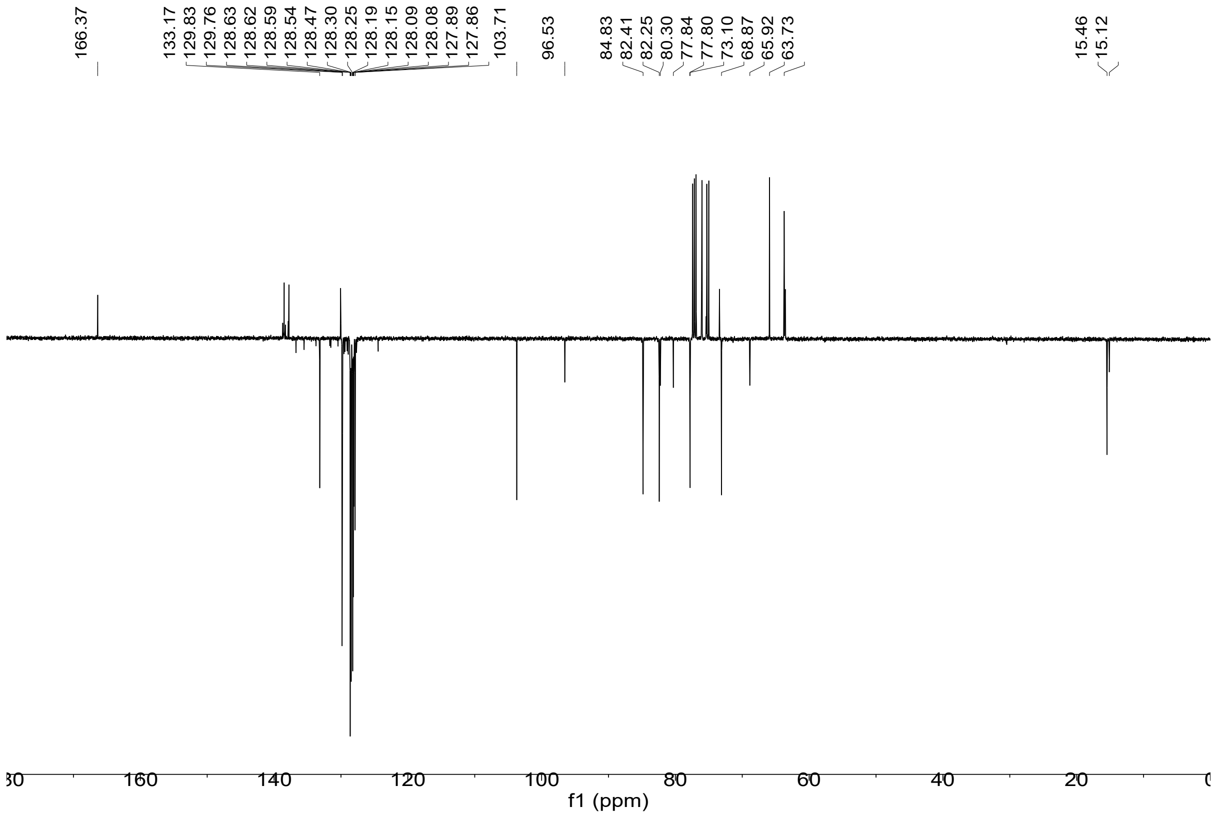

Supplementary Figure 194. ^{13}C NMR, 126 MHz, $CDCl_3$ of compound **S52**

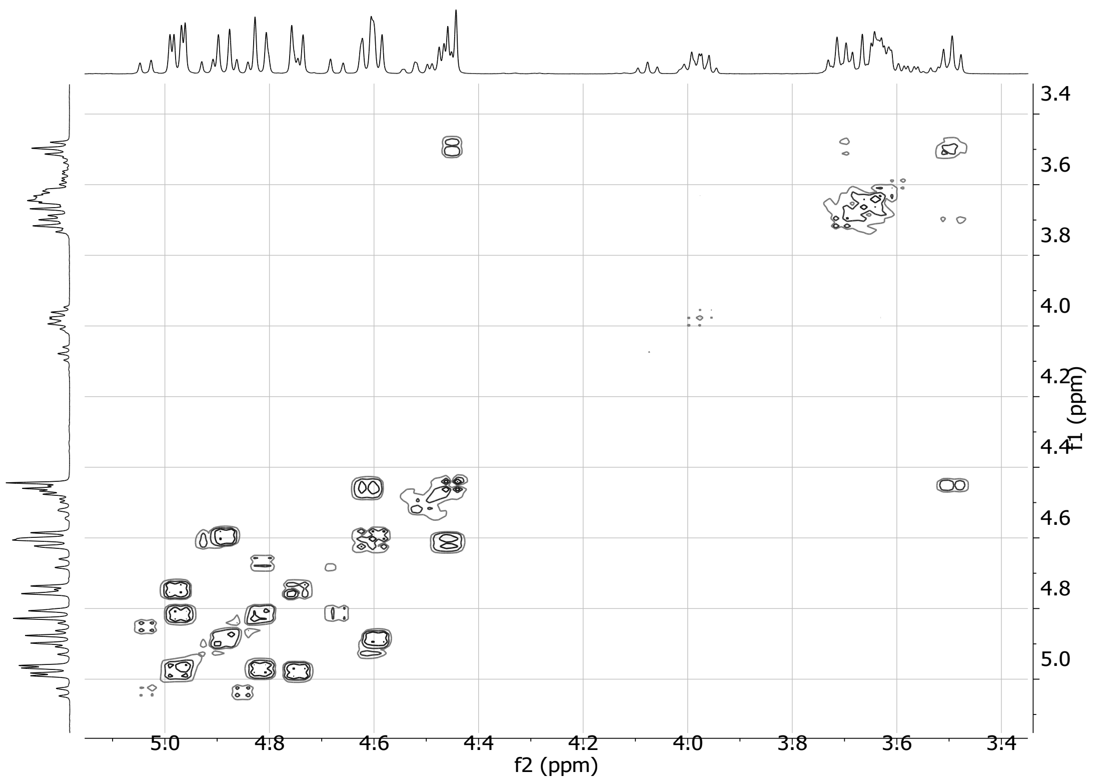

Supplementary Figure 195. HH-COSY NMR, $CDCl_3$ of compound **S52**

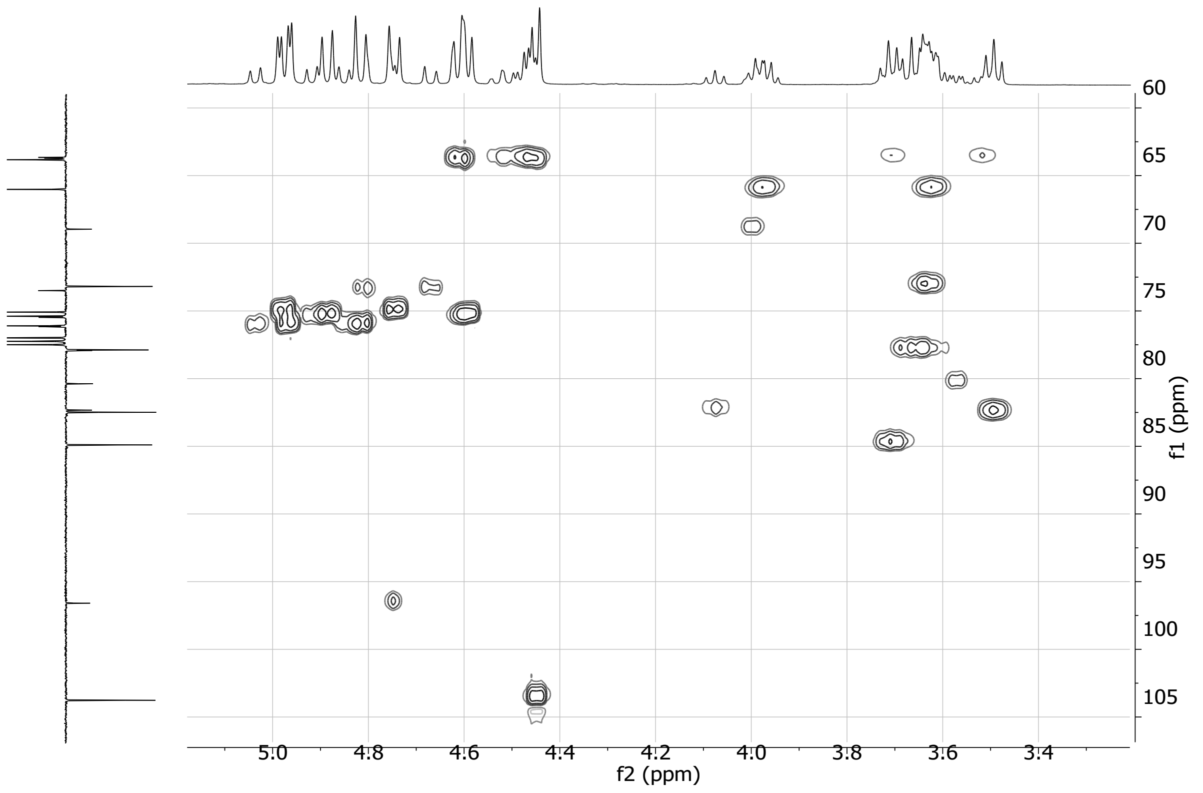

Supplementary Figure 196. HSQC NMR, $CDCl_3$ of compound **S52**

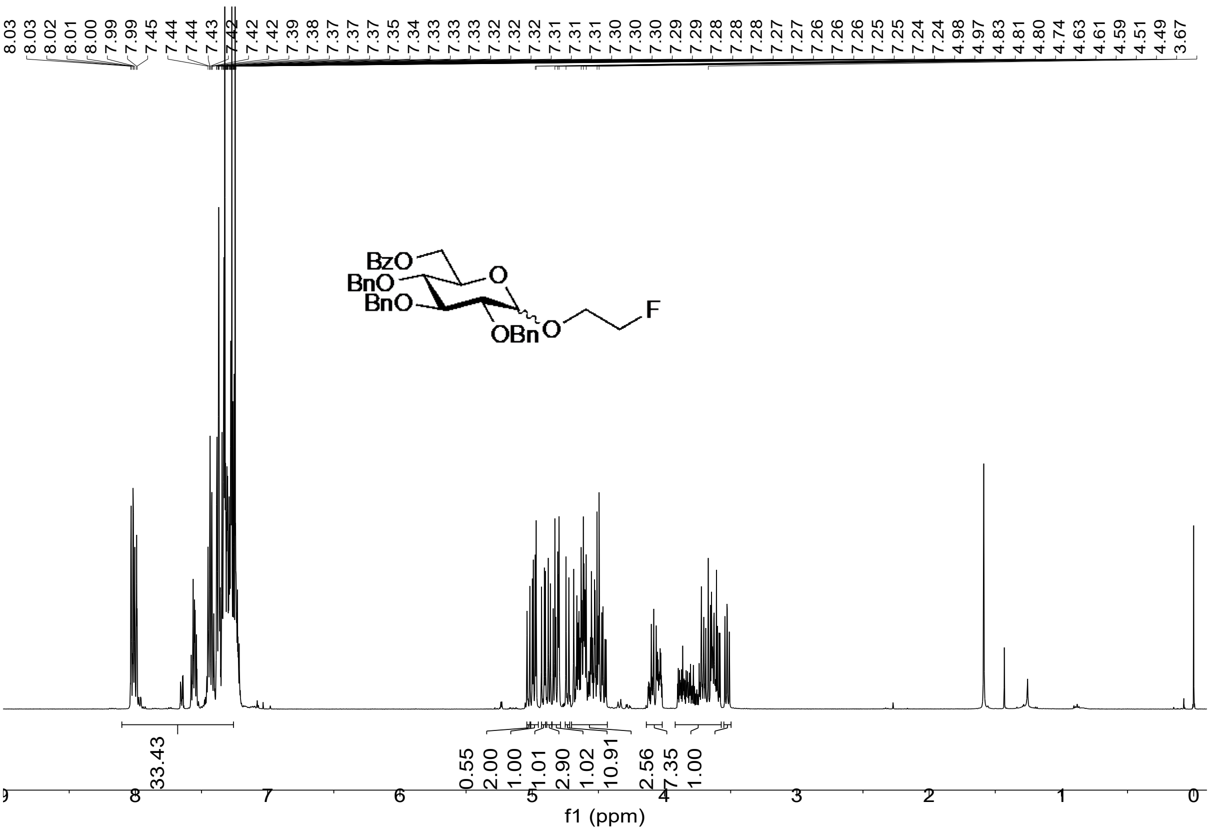

Supplementary Figure 197. ^{1}H NMR, 500 MHz, $CDCl_3$ of compound **S53**

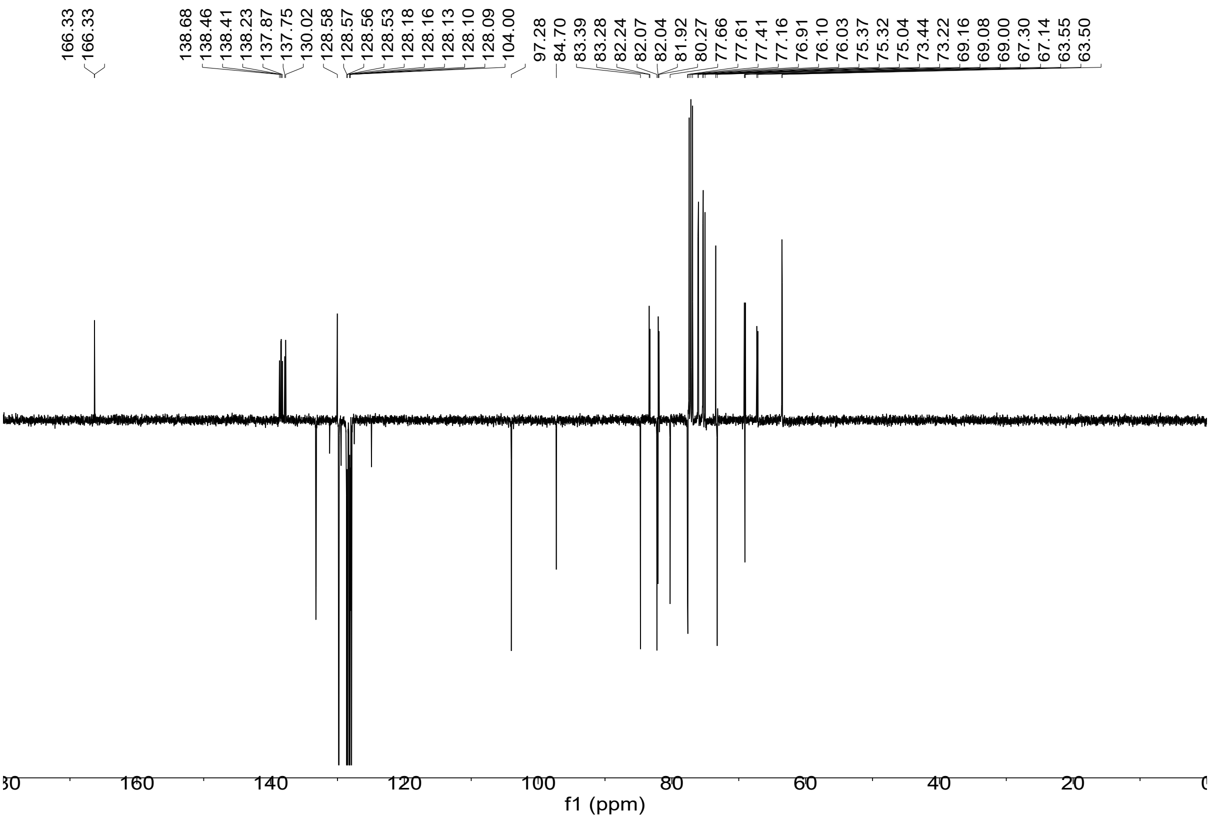

Supplementary Figure 198. ^{13}C NMR, 126 MHz, $CDCl_3$ of compound **S53**

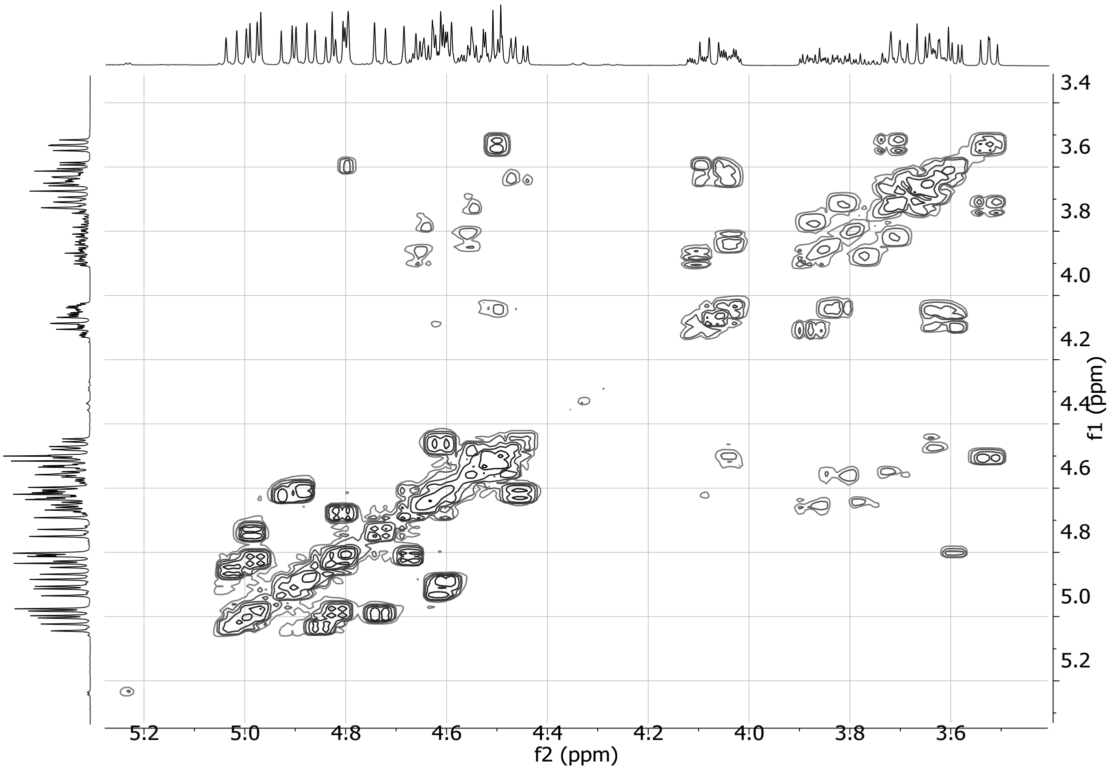

Supplementary Figure 199. HH-COSY NMR, $CDCl_3$ of compound **S53**

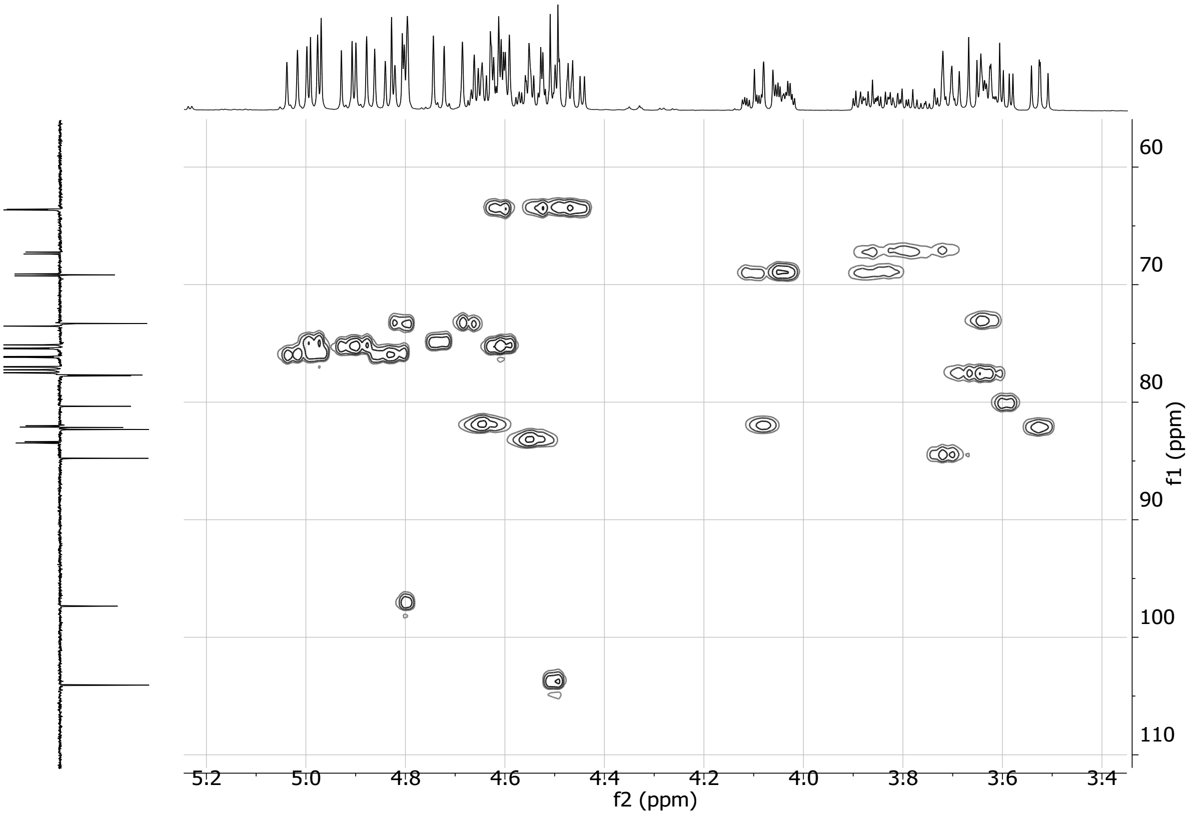

Supplementary Figure 200. HSQC NMR, $CDCl_3$ of compound **S53**

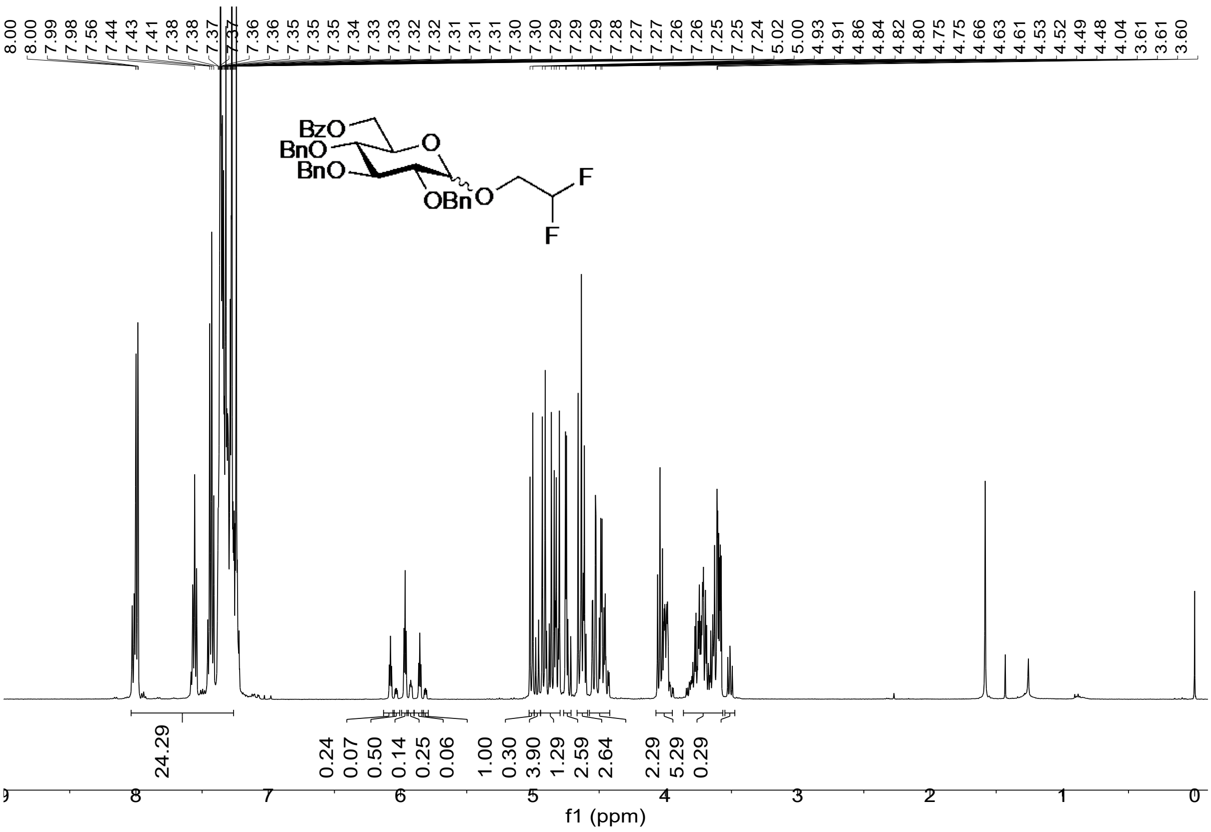

Supplementary Figure 201. ^{1}H NMR, 500 MHz, $CDCl_3$ of compound **S54**

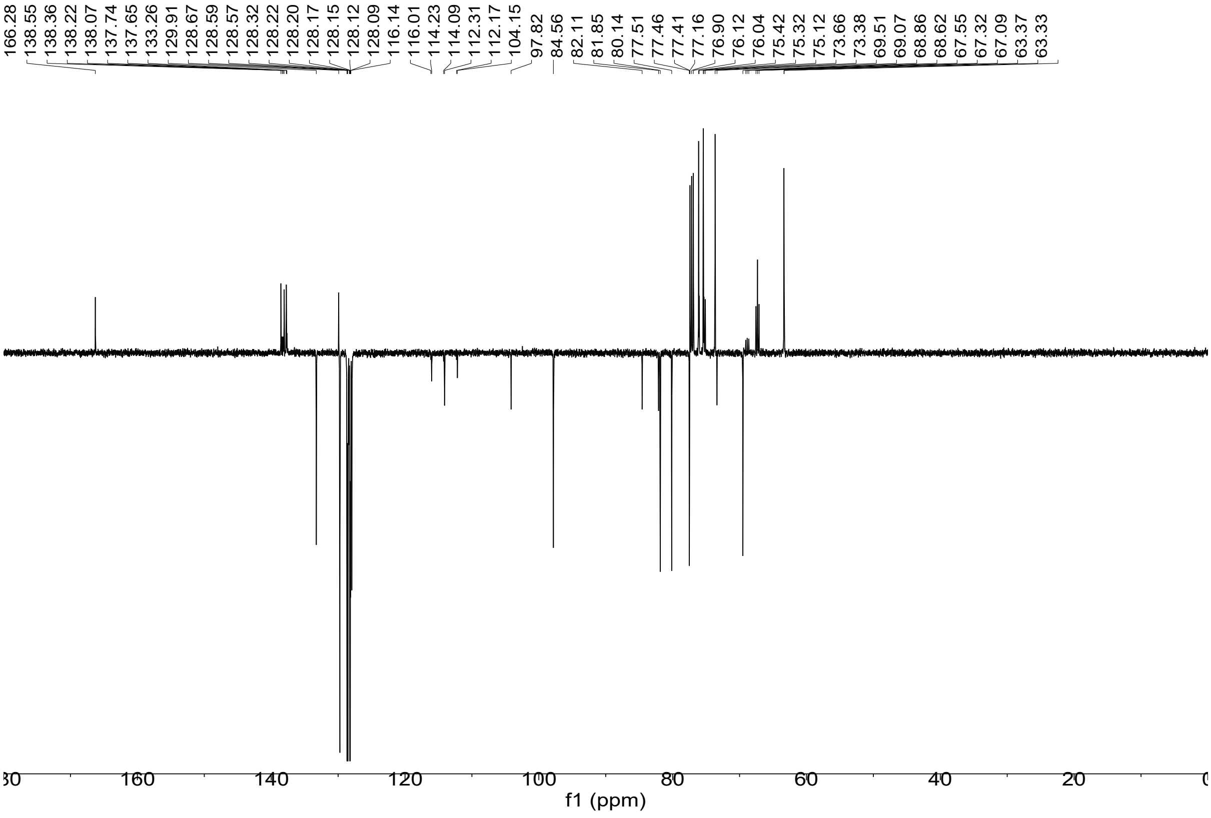

Supplementary Figure 202. ^{13}C NMR, 126 MHz, $CDCl_3$ of compound **S54**

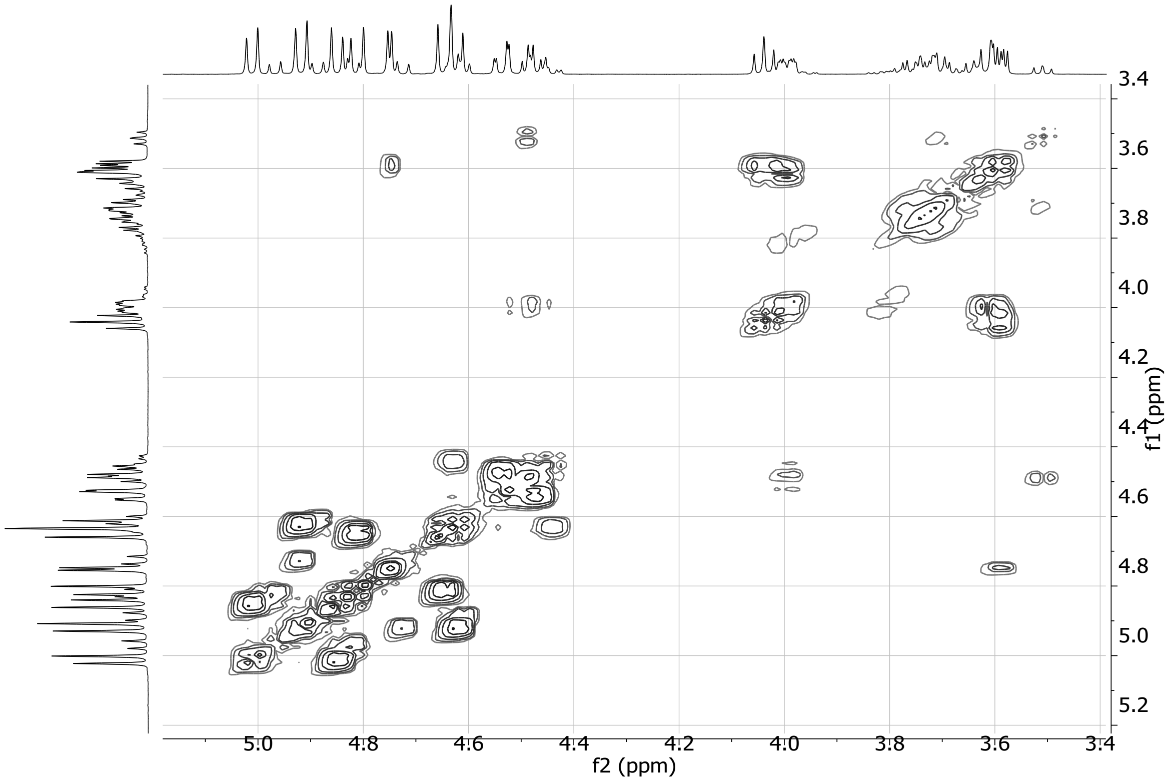

Supplementary Figure 203. HH-COSY NMR, $CDCl_3$ of compound **S54**

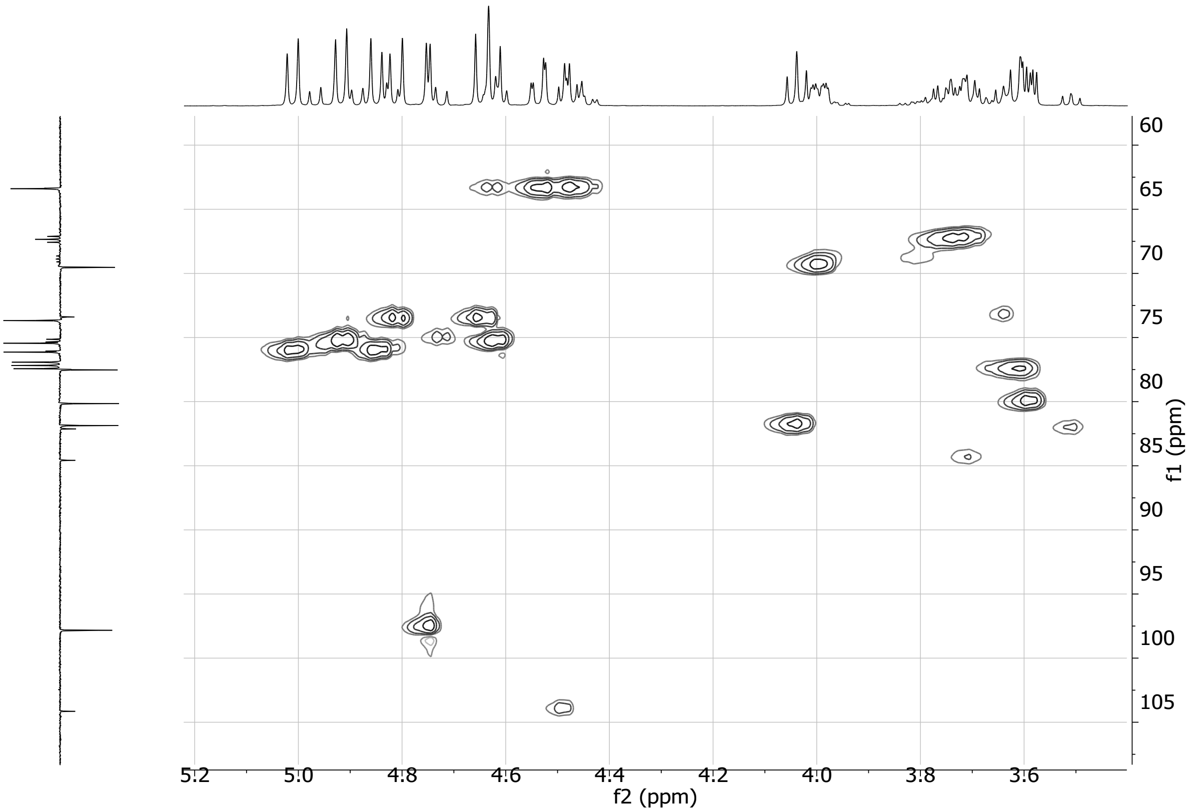

Supplementary Figure 204. HSQC NMR, $CDCl_3$ of compound **S54**

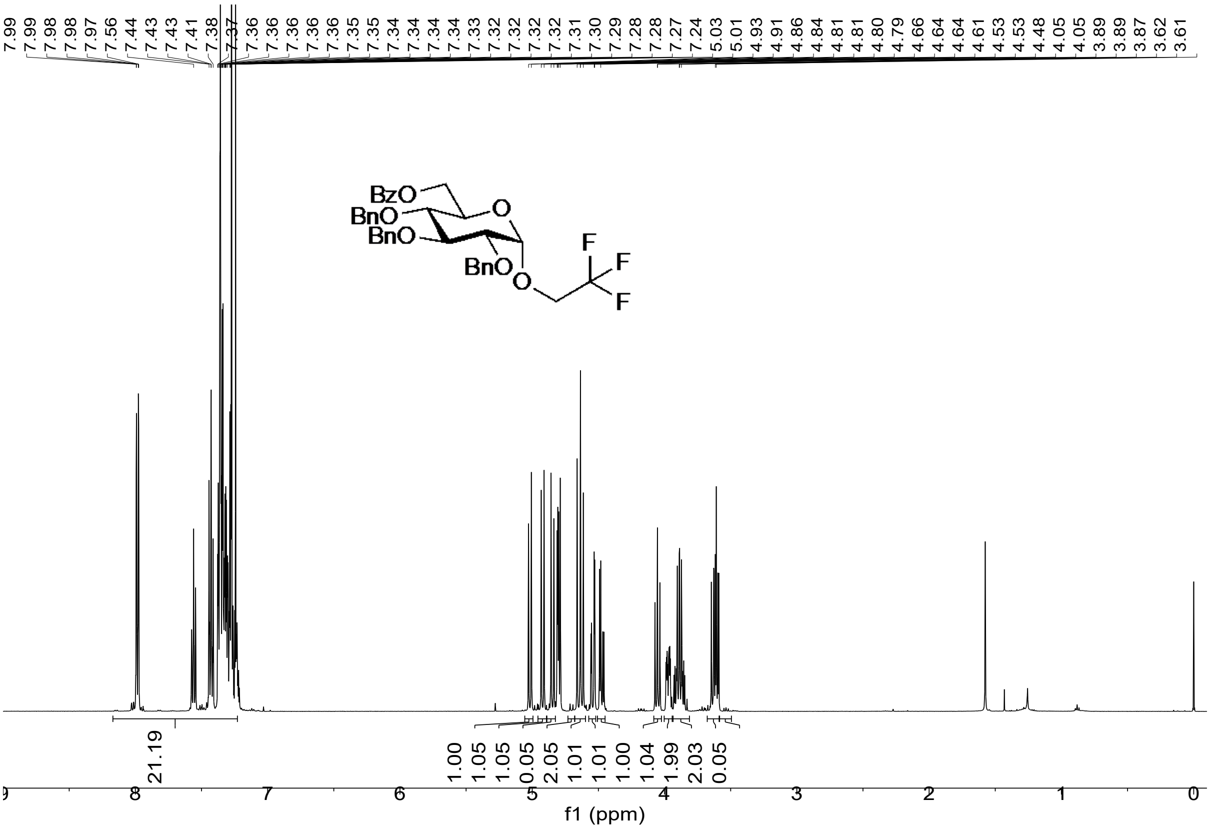

Supplementary Figure 205. ^{1}H NMR, 500 MHz, $CDCl_3$ of compound **S55**

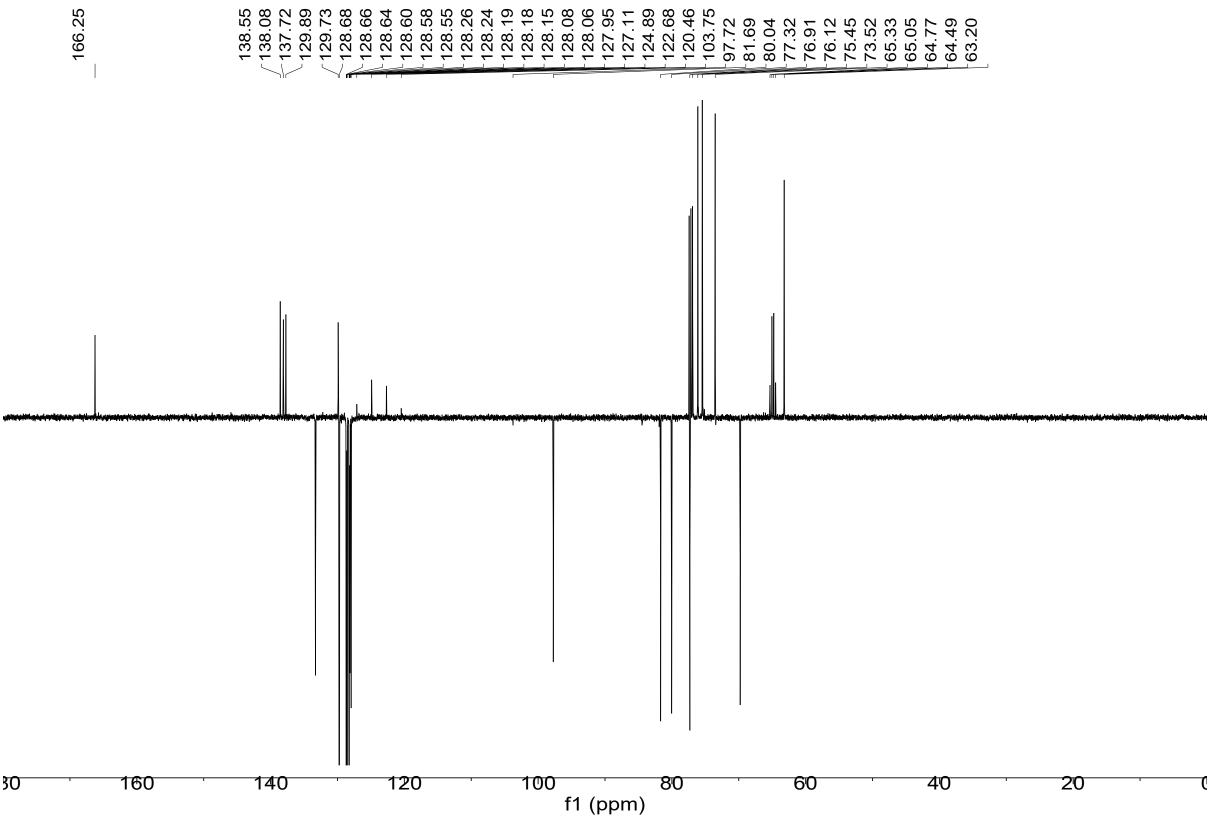

Supplementary Figure 206. ^{13}C NMR, 126 MHz, $CDCl_3$ of compound **S55**

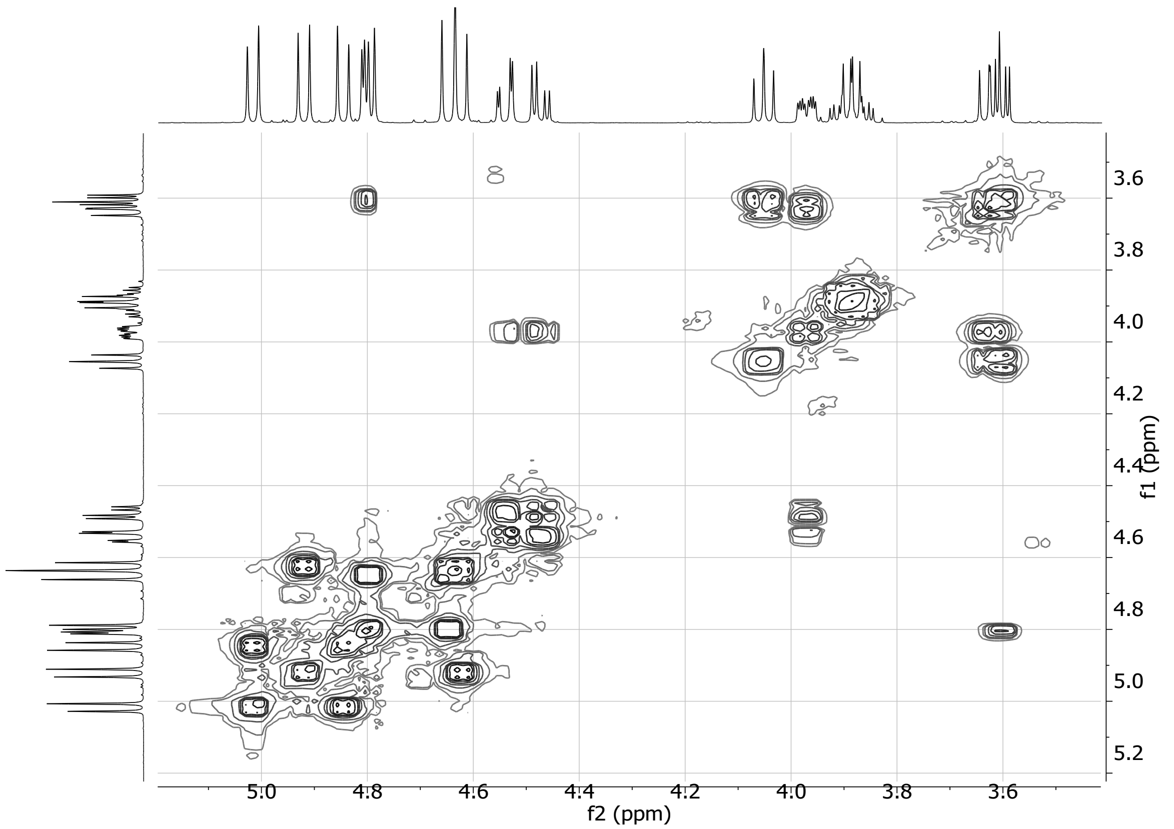

Supplementary Figure 207. HH-COSY NMR, $CDCl_3$ of compound **S55**

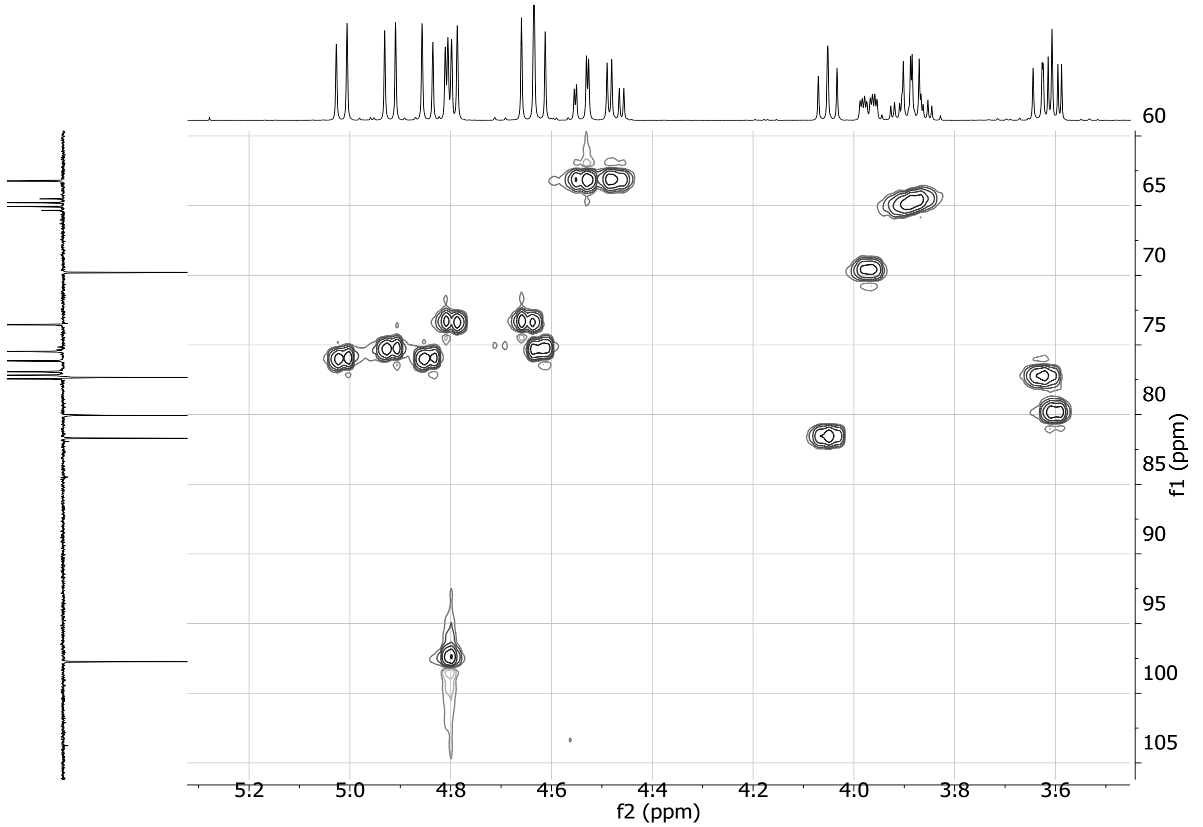

Supplementary Figure 208. HSQC NMR, $CDCl_3$ of compound **S55**

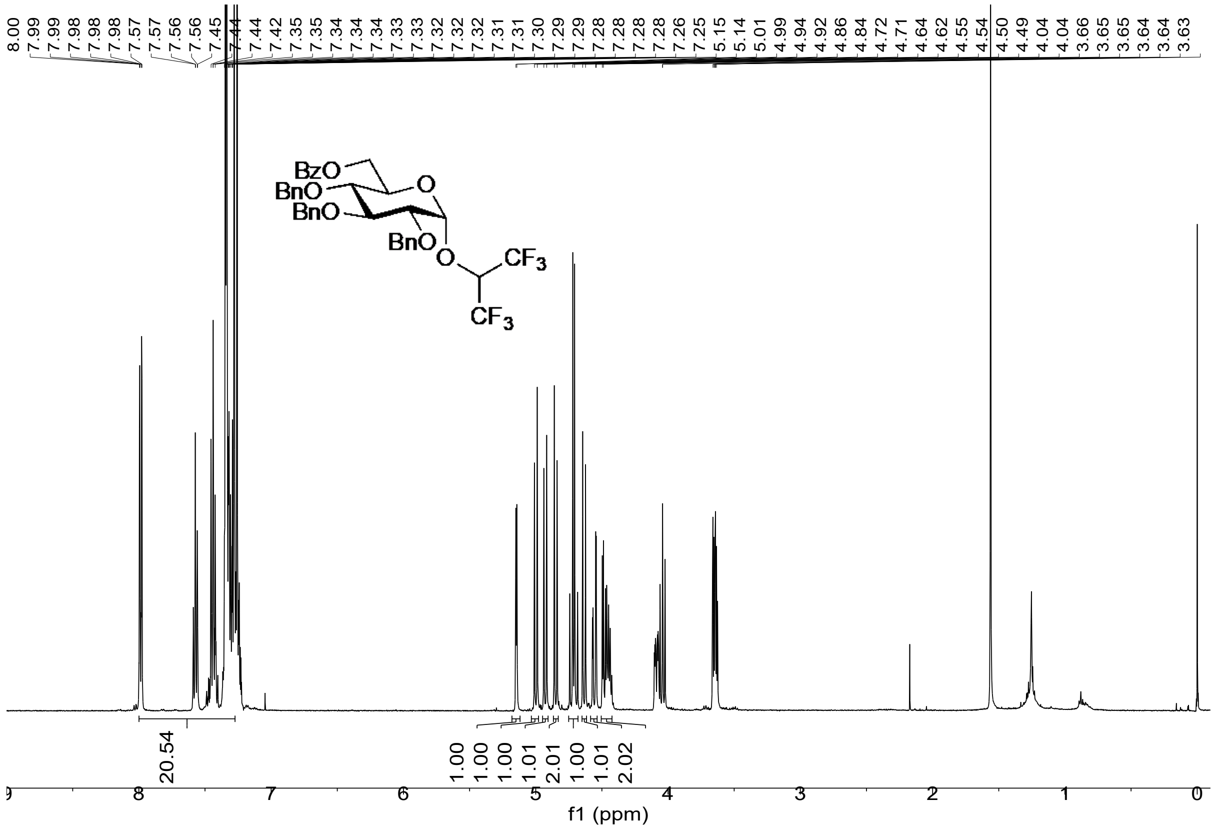

Supplementary Figure 209. ^{1}H NMR, 500 MHz, $CDCl_3$ of compound **S56**

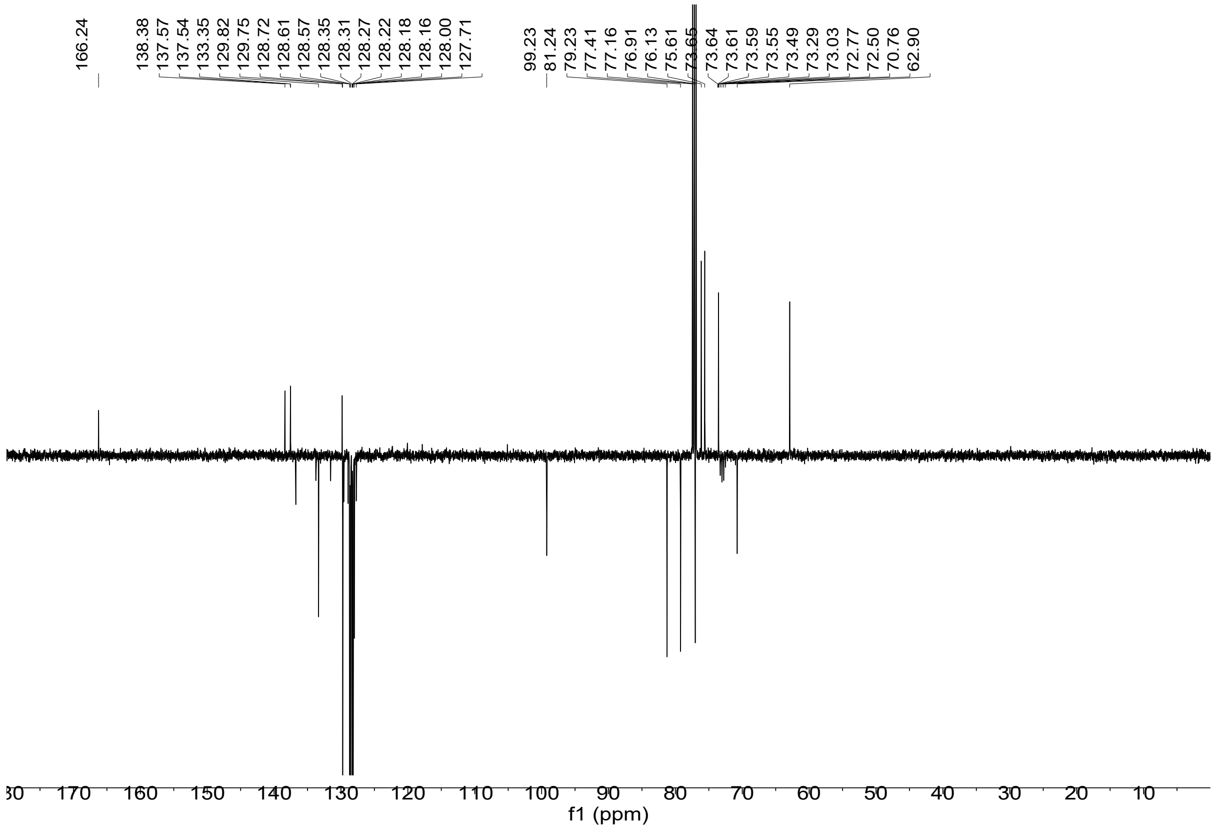

Supplementary Figure 210. ^{13}C NMR, 126 MHz, $CDCl_3$ of compound **S56**

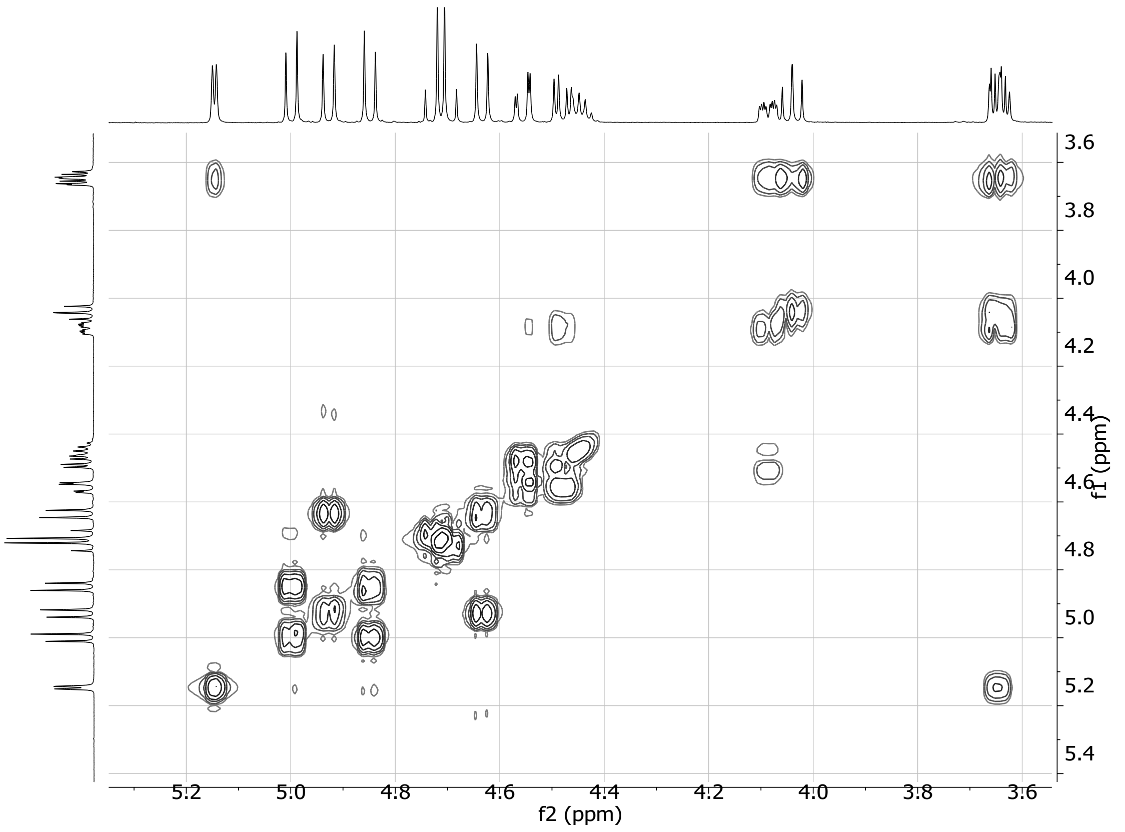

Supplementary Figure 211. HH-COSY NMR, $CDCl_3$ of compound **S56**

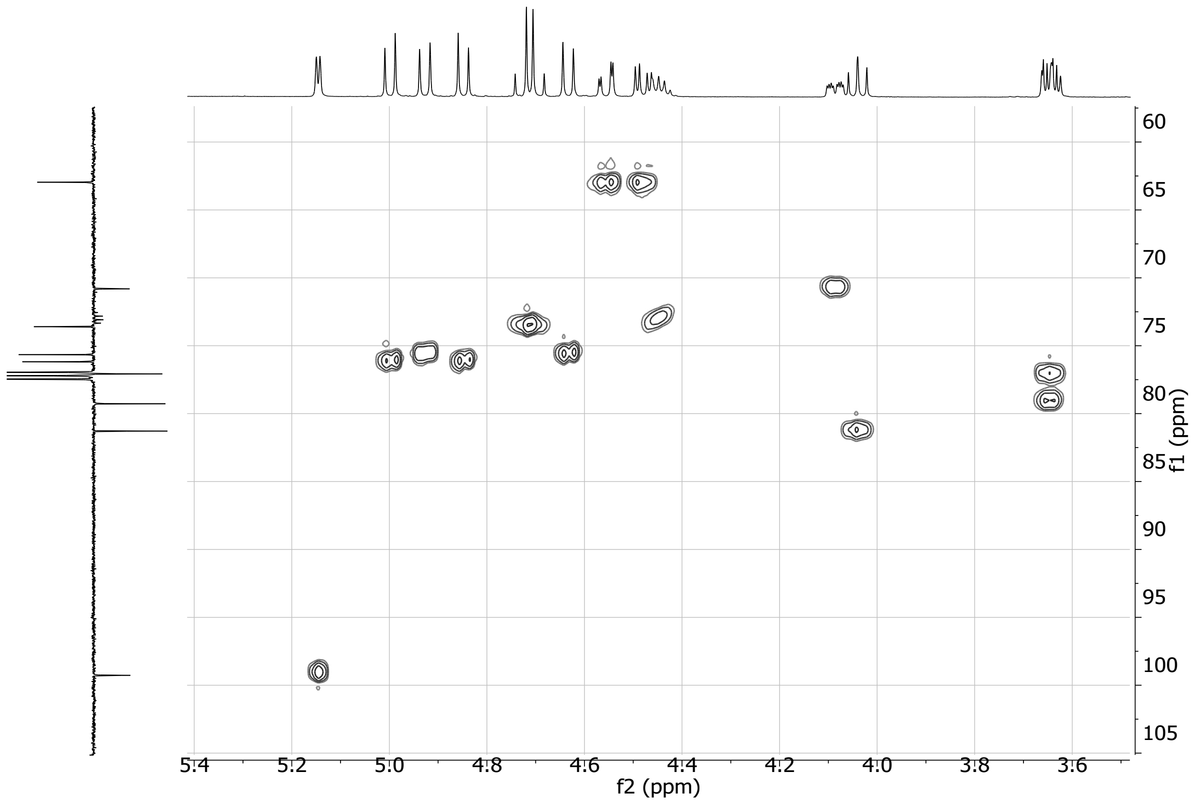

Supplementary Figure 212. HSQC NMR, $CDCl_3$ of compound **S56**

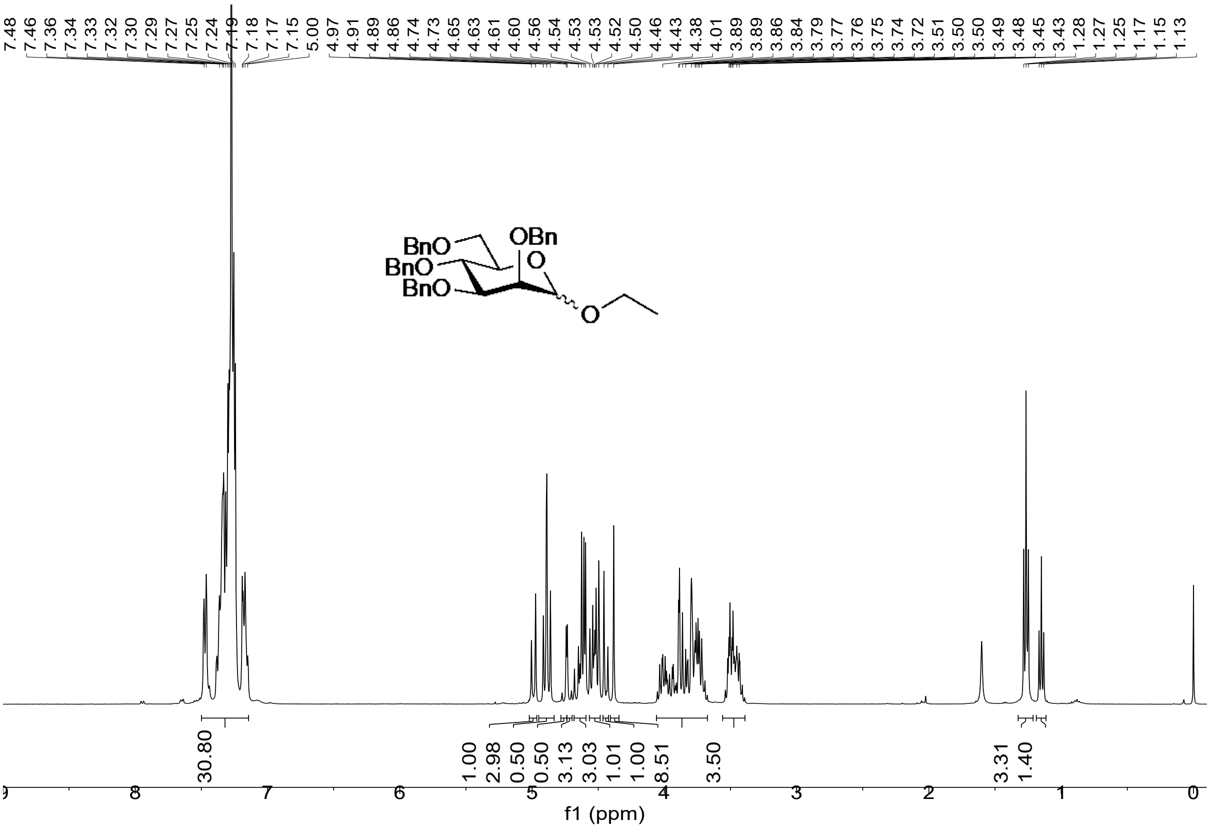

Supplementary Figure 213. ^{1}H NMR, 400 MHz, $CDCl_3$ of compound **S57**

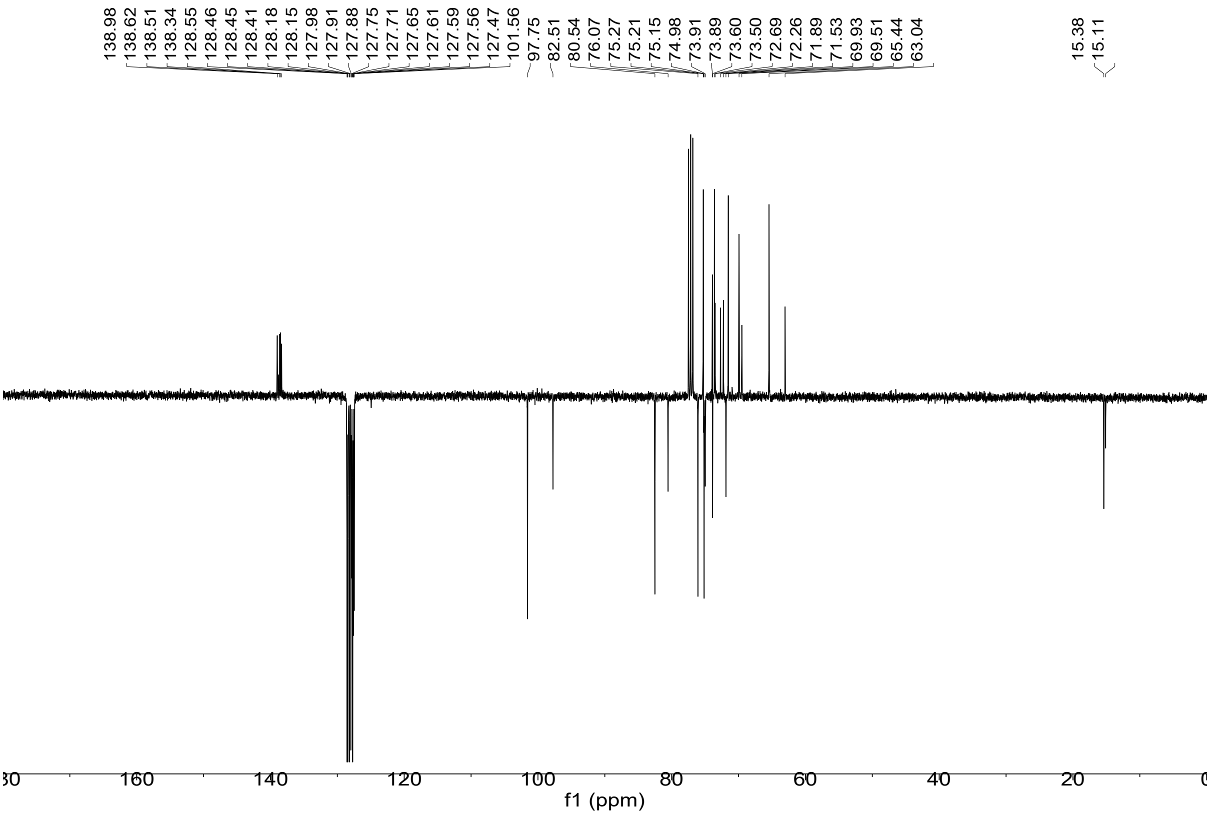

Supplementary Figure 214. ^{13}C NMR, 101 MHz, $CDCl_3$ of compound **S57**

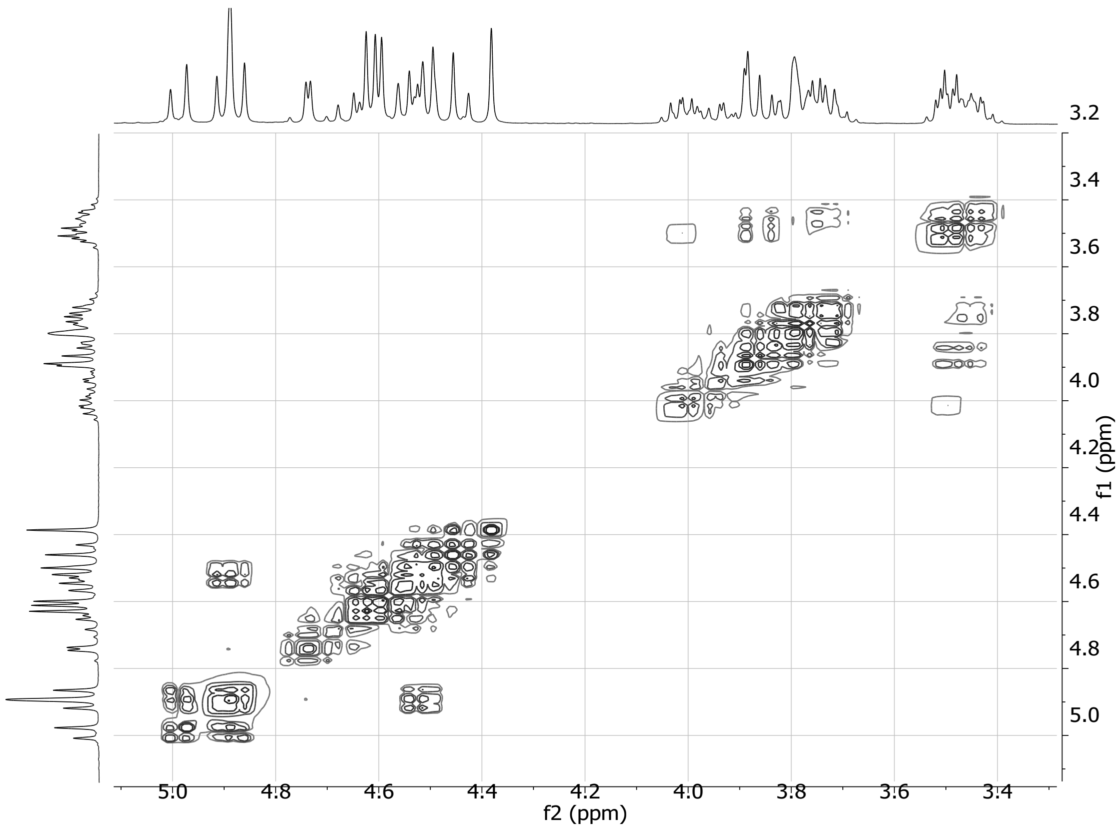

Supplementary Figure 215. HH-COSY NMR, $CDCl_3$ of compound **S57**

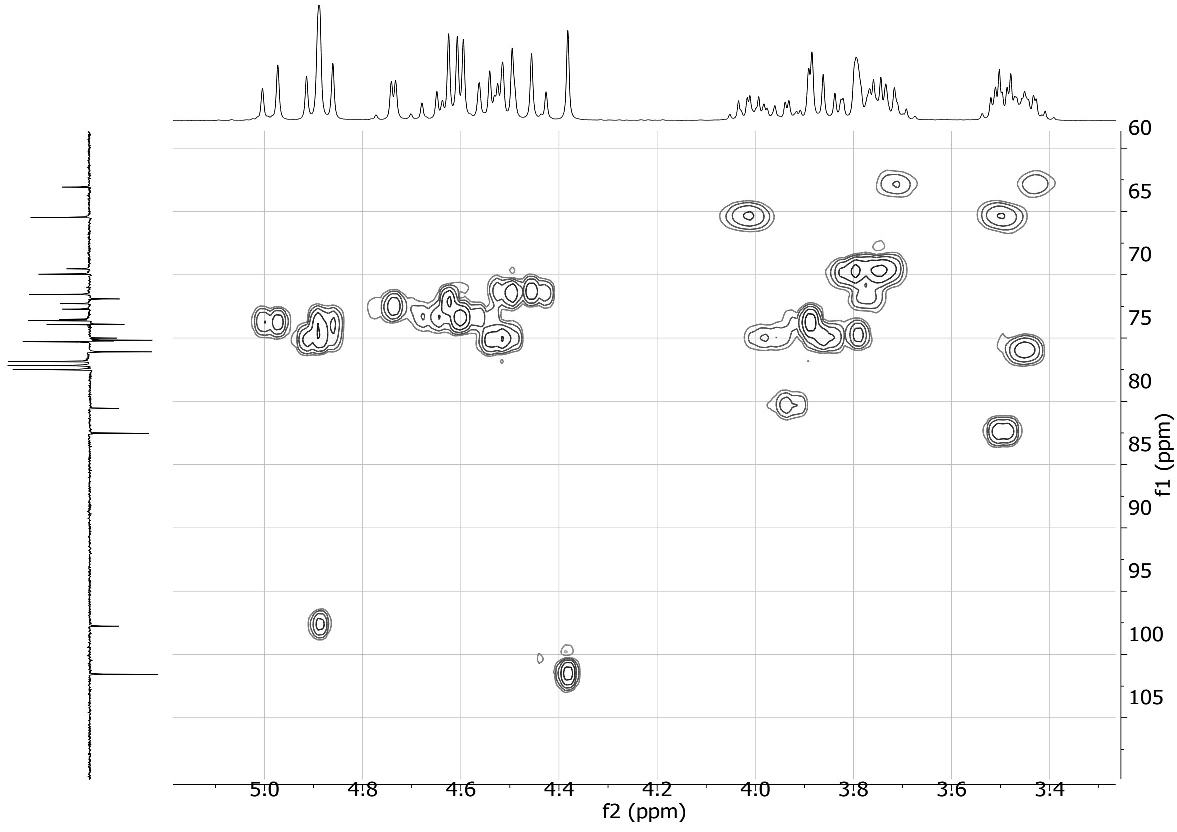

Supplementary Figure 216. HSQC NMR, $CDCl_3$ of compound **S57**

S

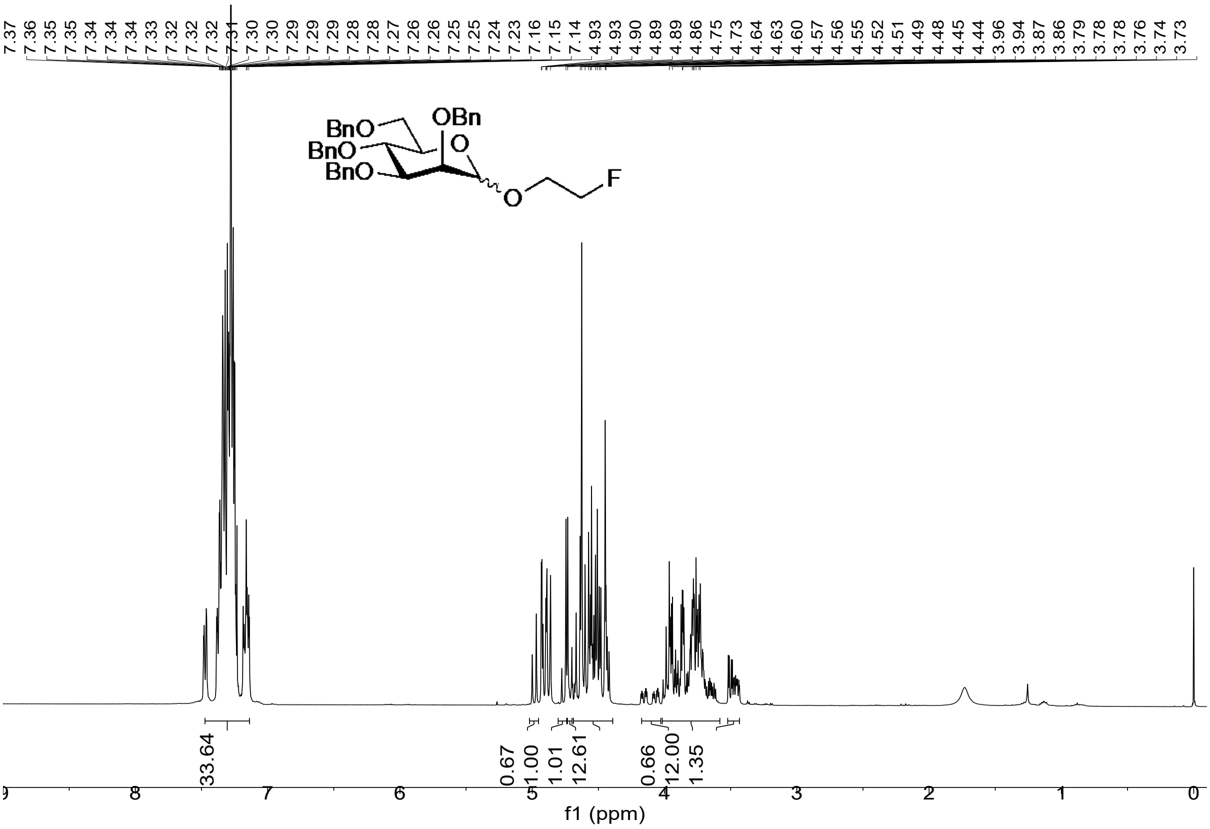

upplementary Figure 217. ^{1}H NMR, 400 MHz, $CDCl_3$ of compound **S58**

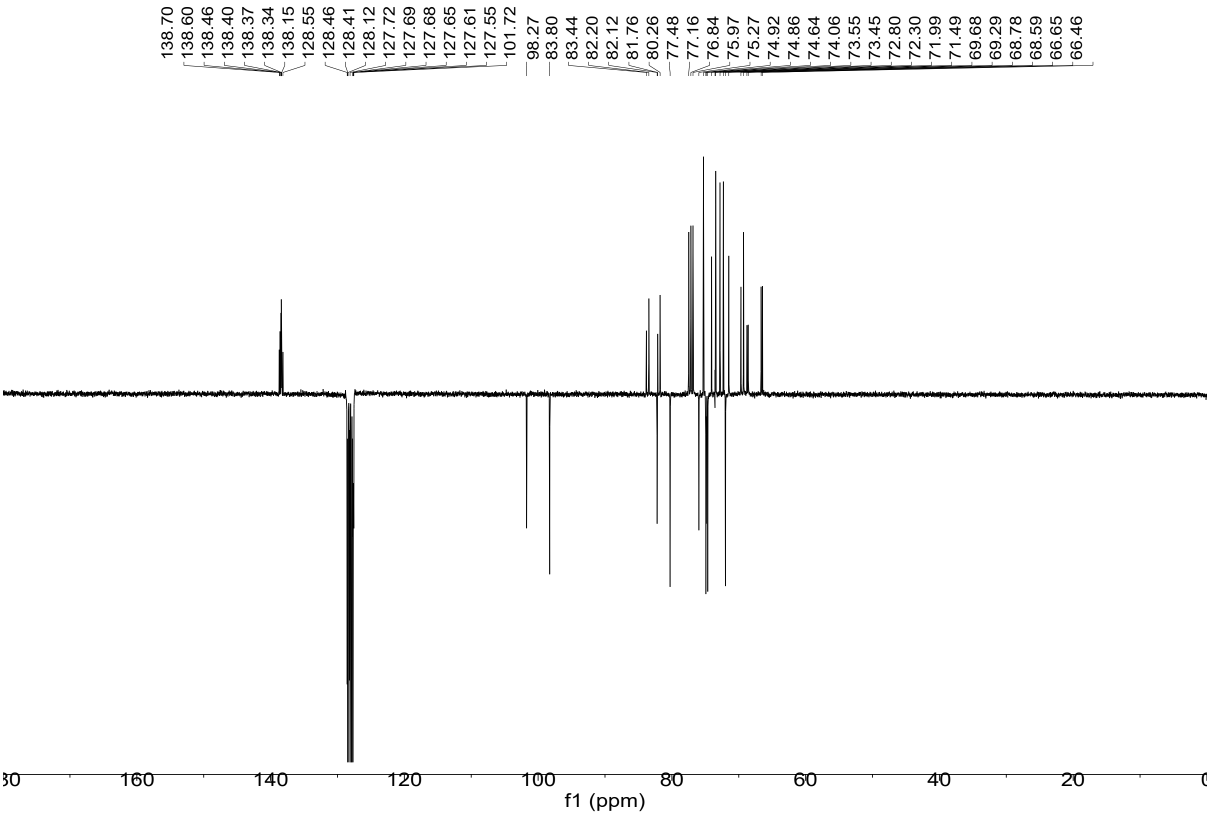

Supplementary Figure 218. ^{13}C NMR, 101 MHz, $CDCl_3$ of compound **S58**

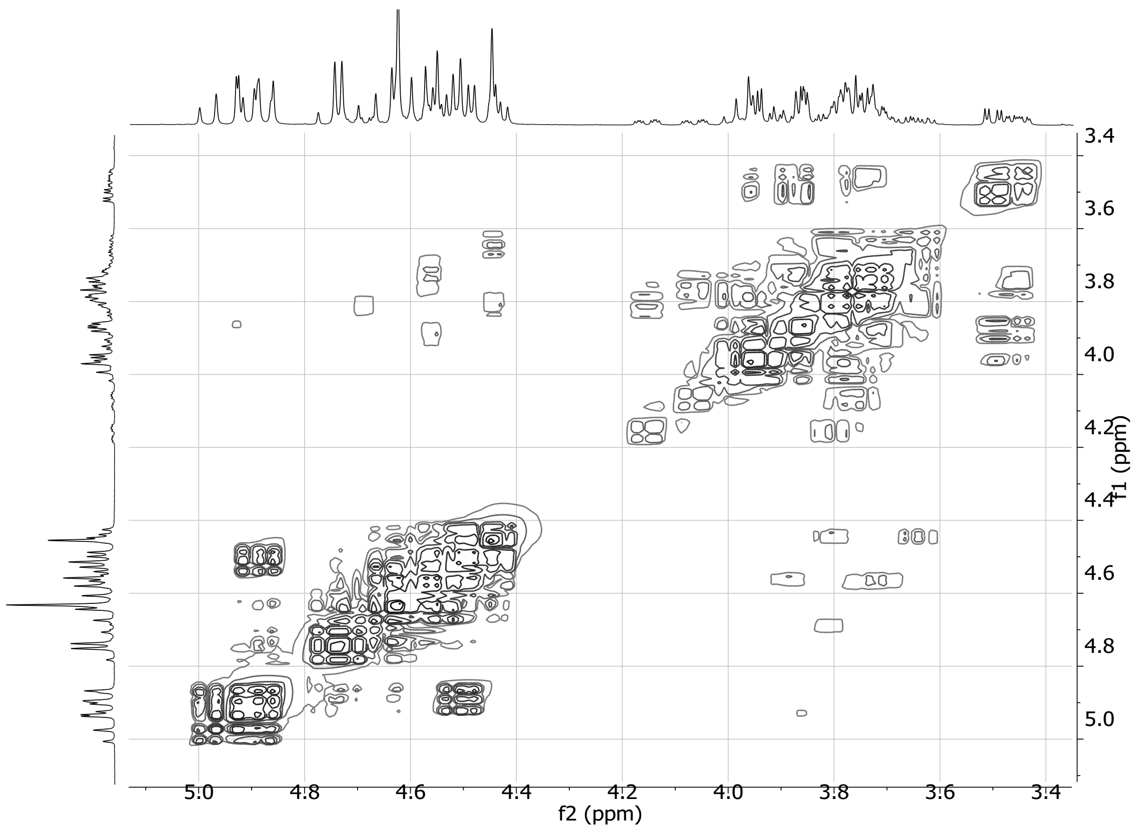

Supplementary Figure 219. HH-COSY NMR, $CDCl_3$ of compound b

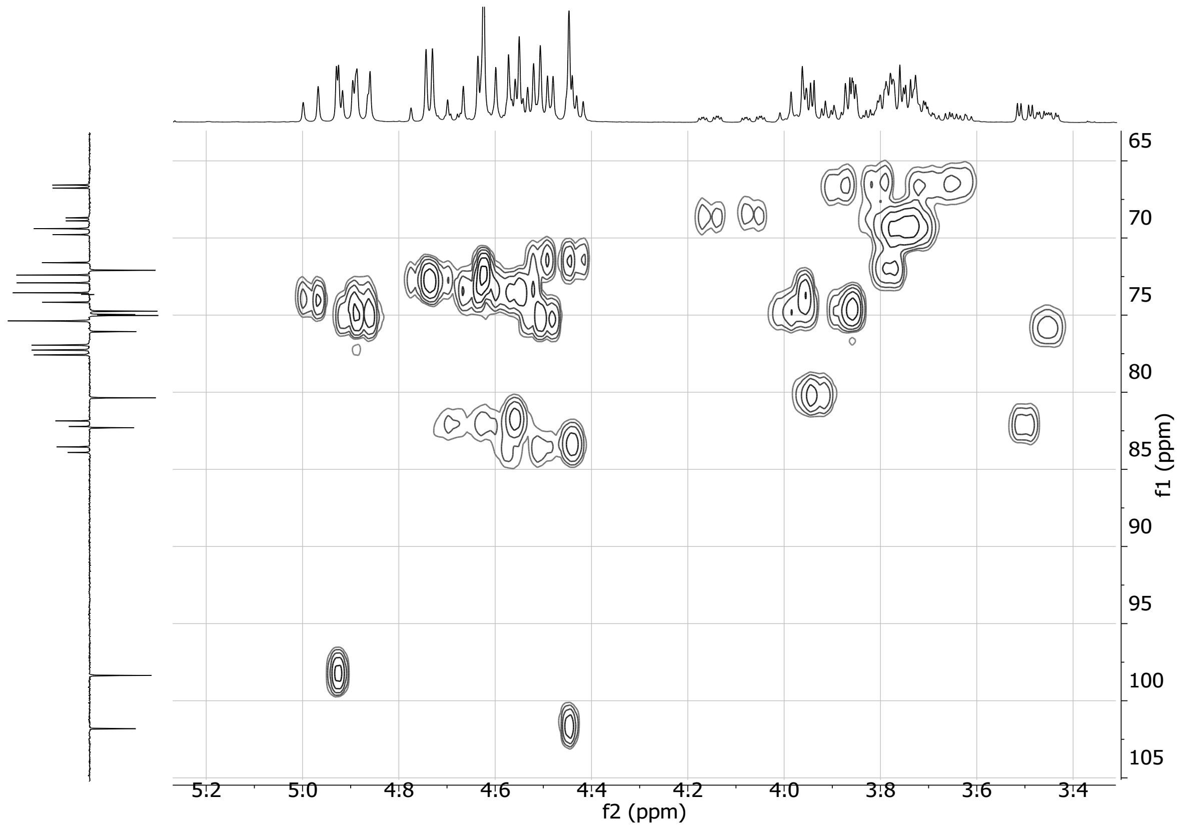

Supplementary Figure 220. HSQC NMR, $CDCl_3$ of compound **S58**

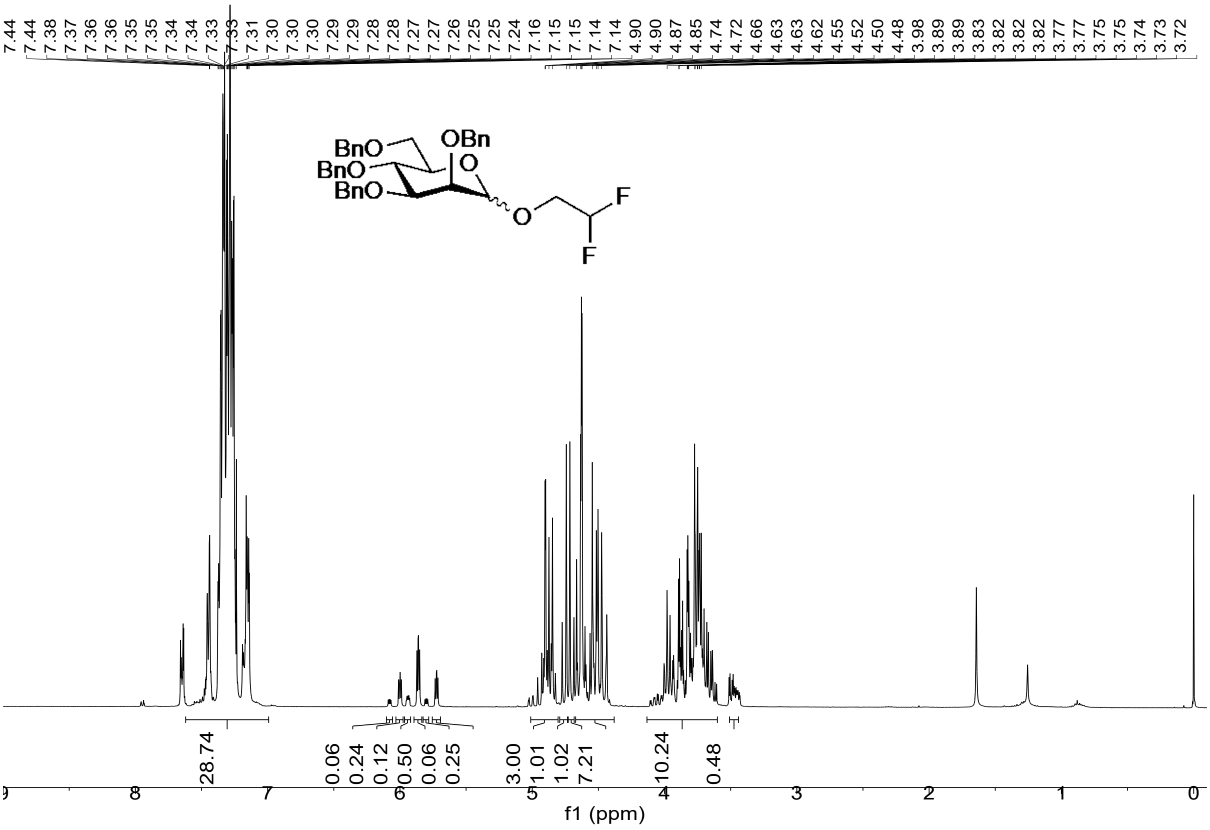

Supplementary Figure 221. ^{1}H NMR, 400 MHz, $CDCl_3$ of compound **S59**

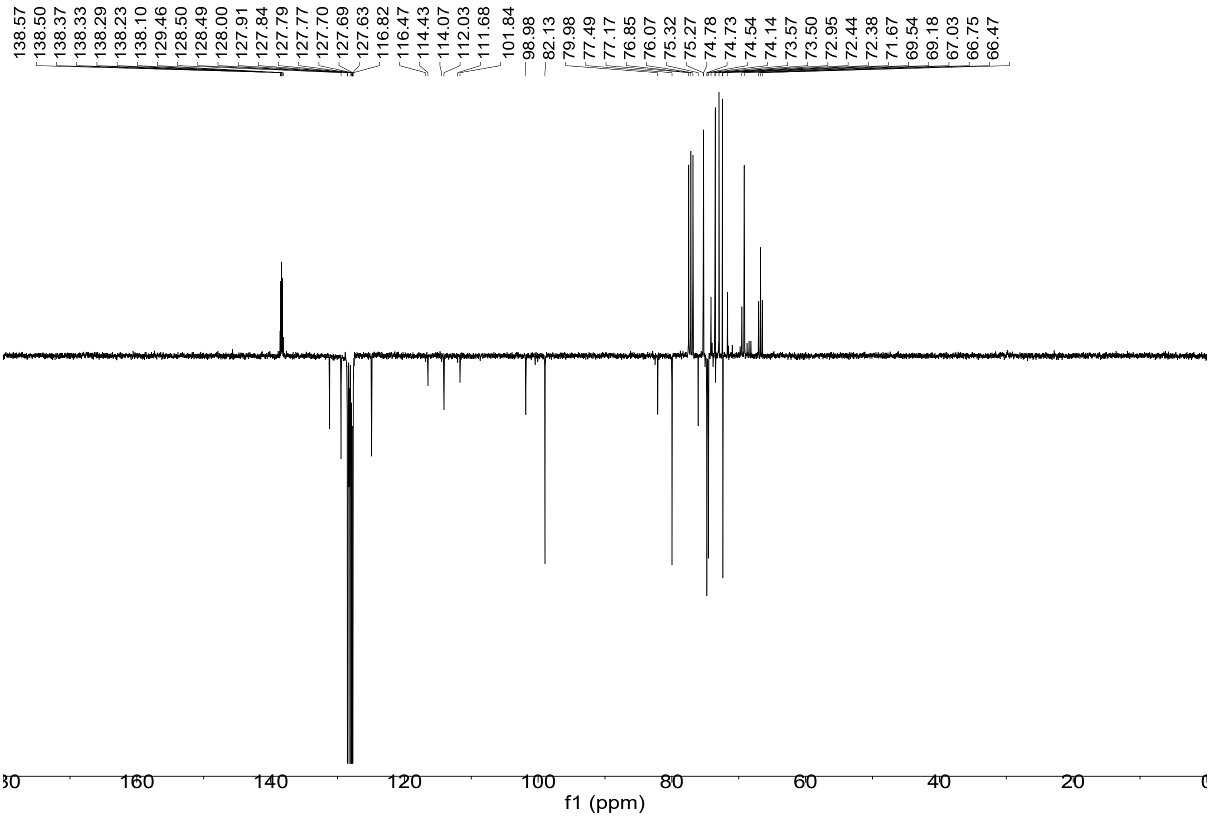

Supplementary Figure 222. ^{13}C NMR, 101 MHz, $CDCl_3$ of compound **S59**

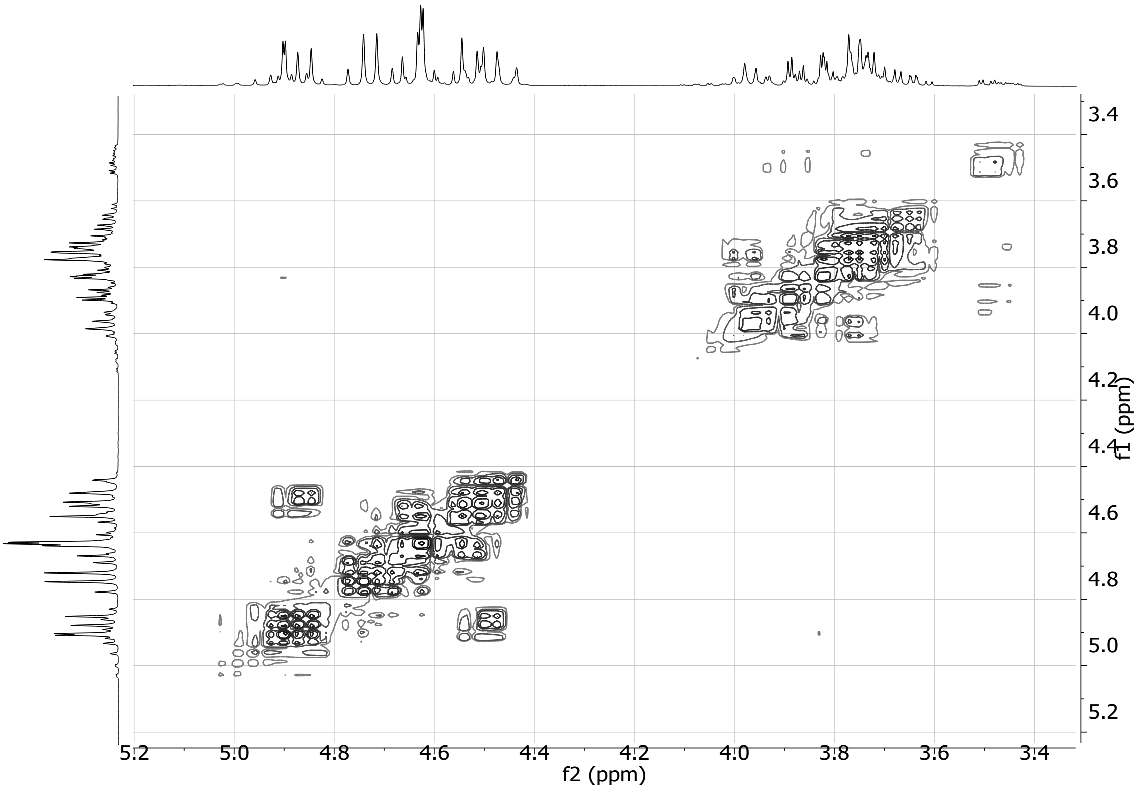

Supplementary Figure 223. HH-COSY NMR, $CDCl_3$ of compound **S59**

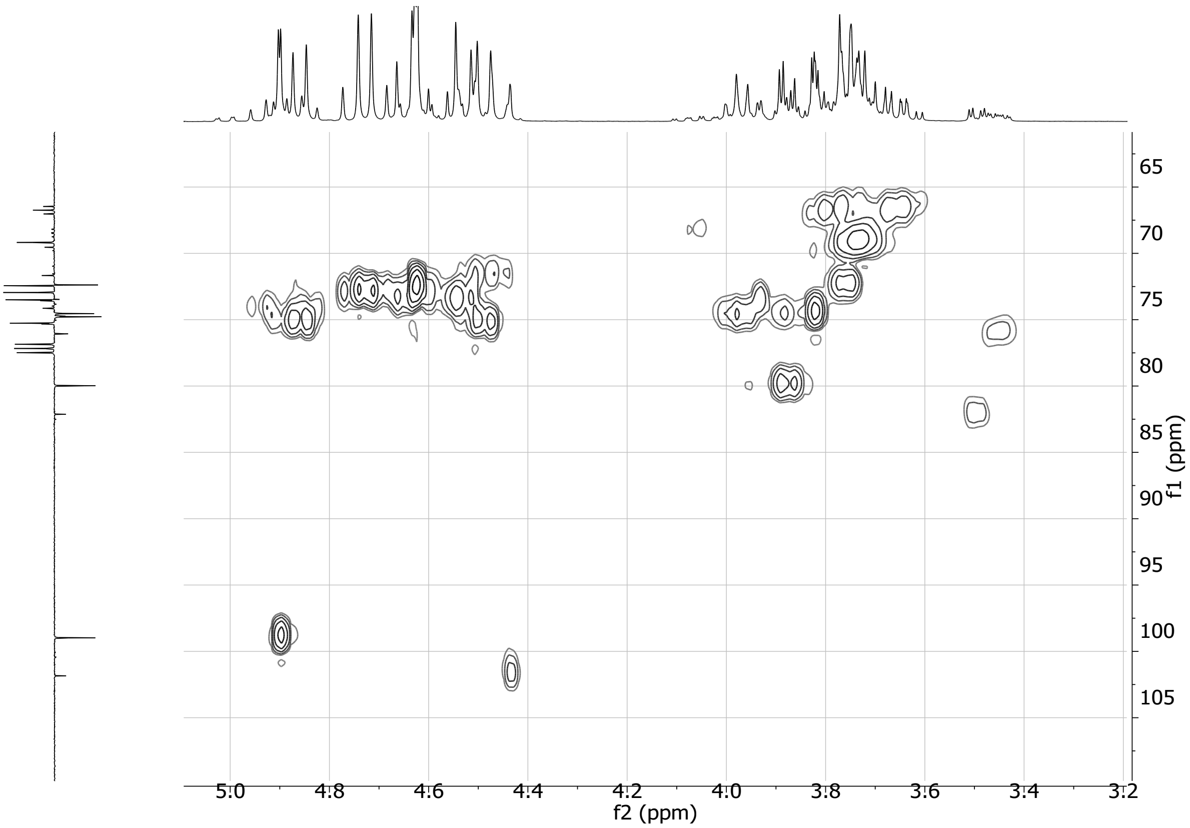

Supplementary Figure 224. HSQC NMR, $CDCl_3$ of compound **S59**

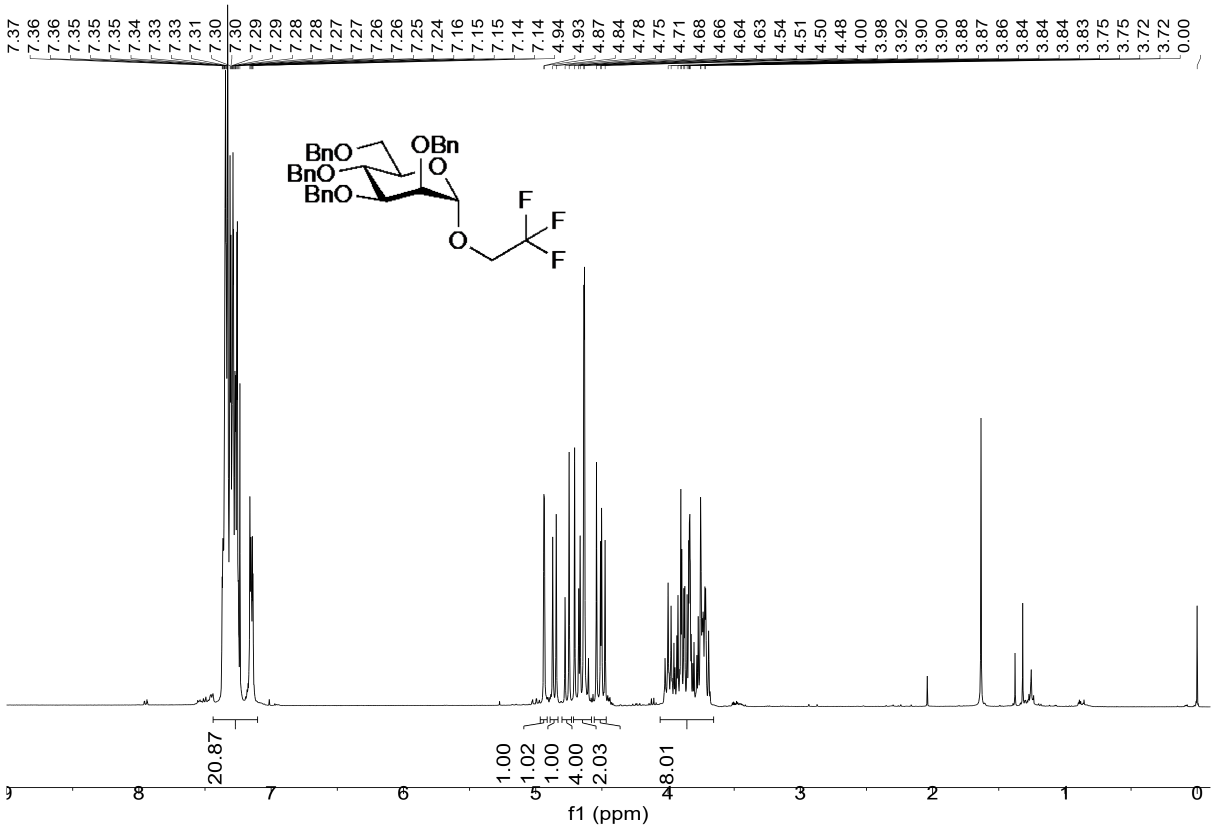

Supplementary Figure 225. ^{1}H NMR, 400 MHz, $CDCl_3$ of compound **S60**

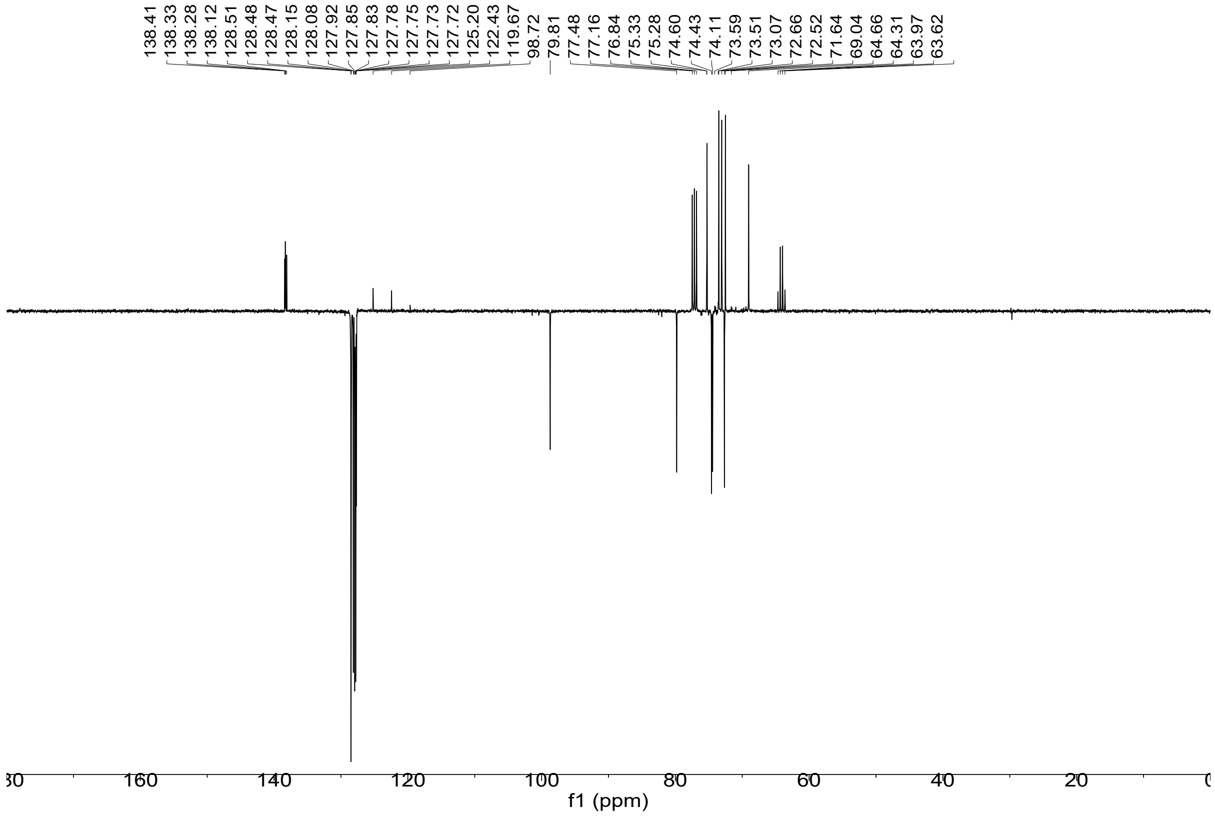

Supplementary Figure 226. ^{13}C NMR, 101 MHz, $CDCl_3$ of compound **S60**

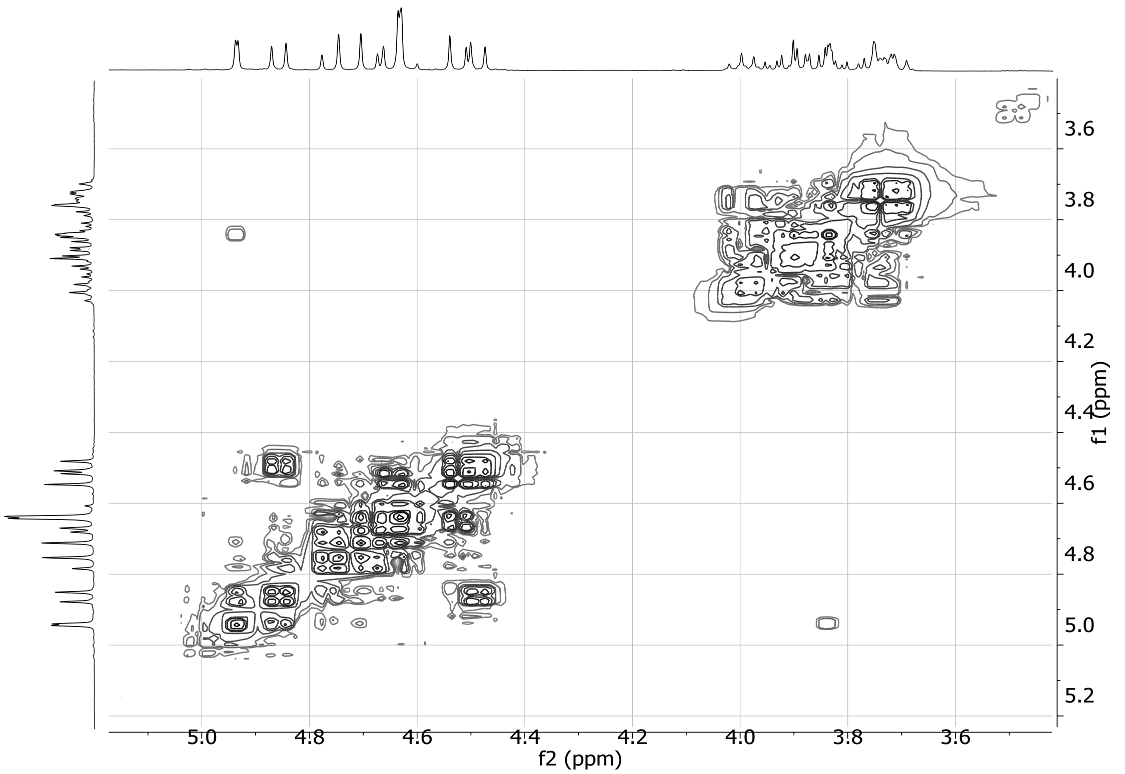

Supplementary Figure 227. HH-COSY NMR, $CDCl_3$ of compound **S60**

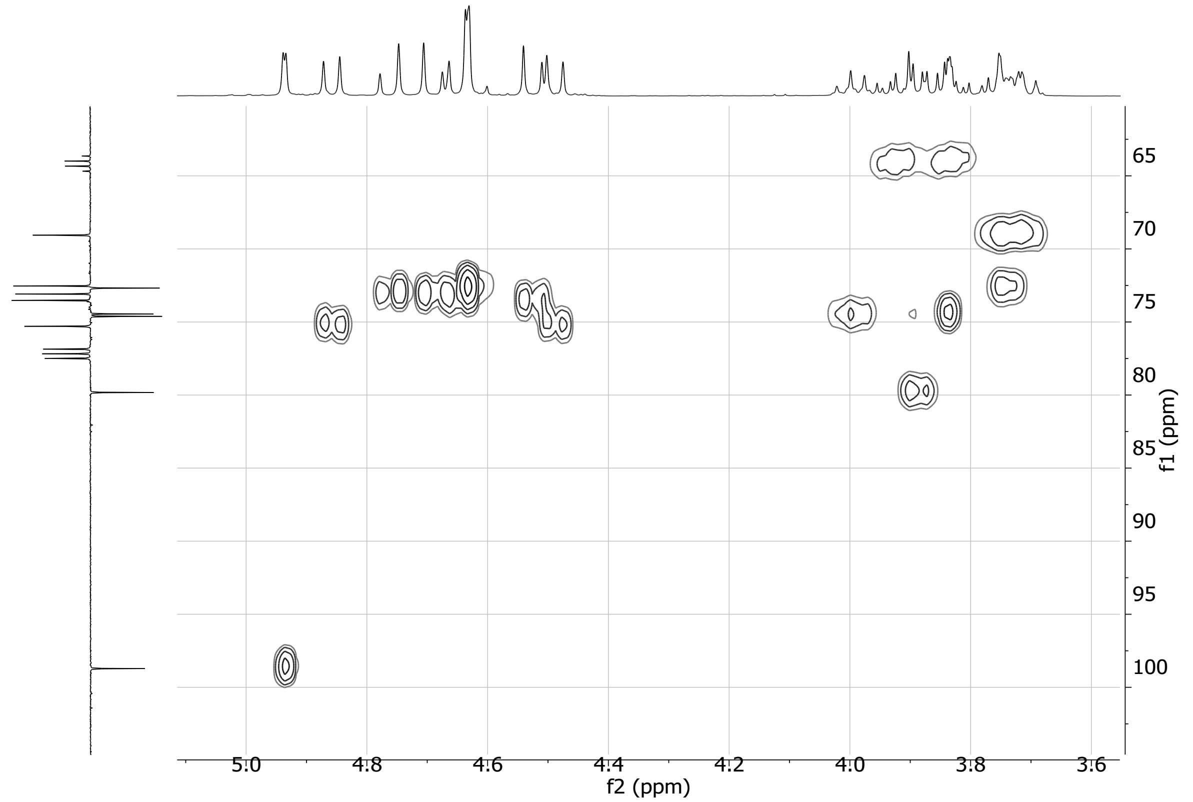

Supplementary Figure 228. HSQC NMR, $CDCl_3$ of compound **S60**

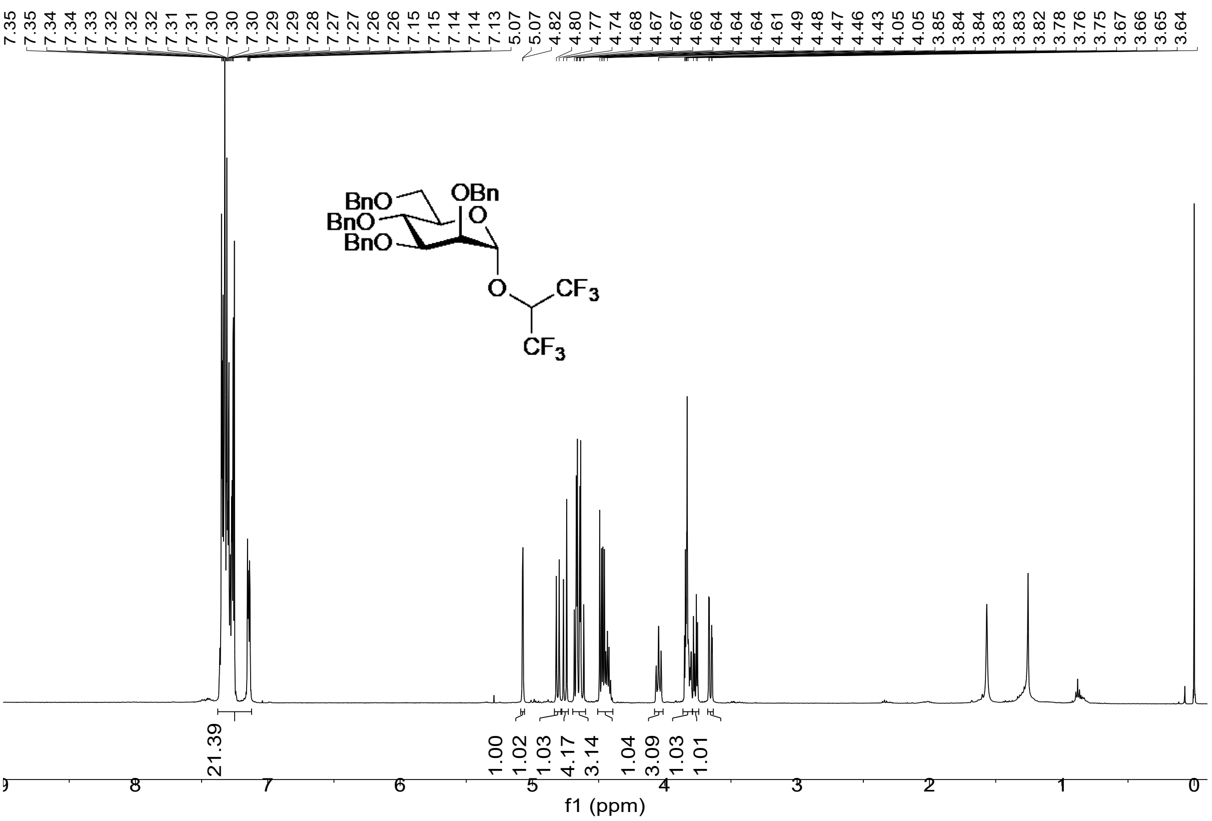

Supplementary Figure 229. ^{1}H NMR, 500 MHz, $CDCl_3$ of compound **S61**

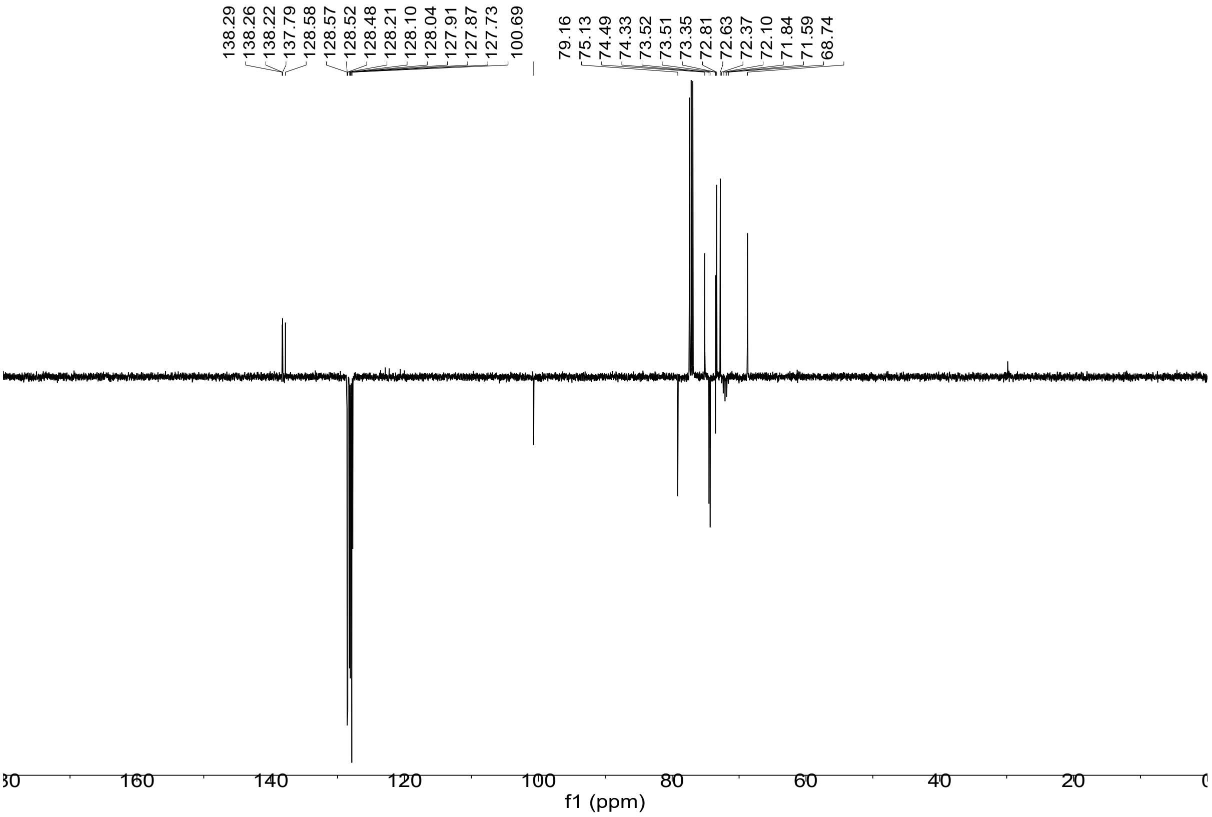

Supplementary Figure 230. ^{13}C NMR, 126 MHz, $CDCl_3$ of compound **S61**

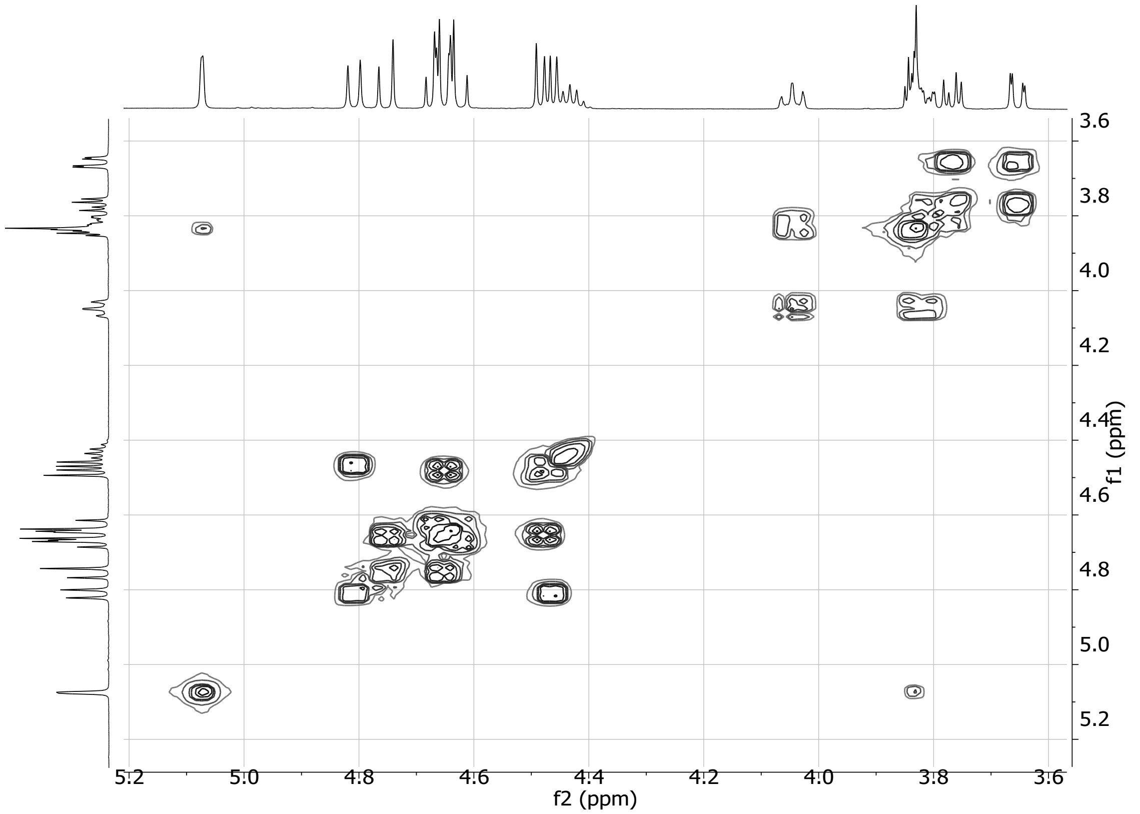

Supplementary Figure 231. HH-COSY NMR, $CDCl_3$ of compound **S61**

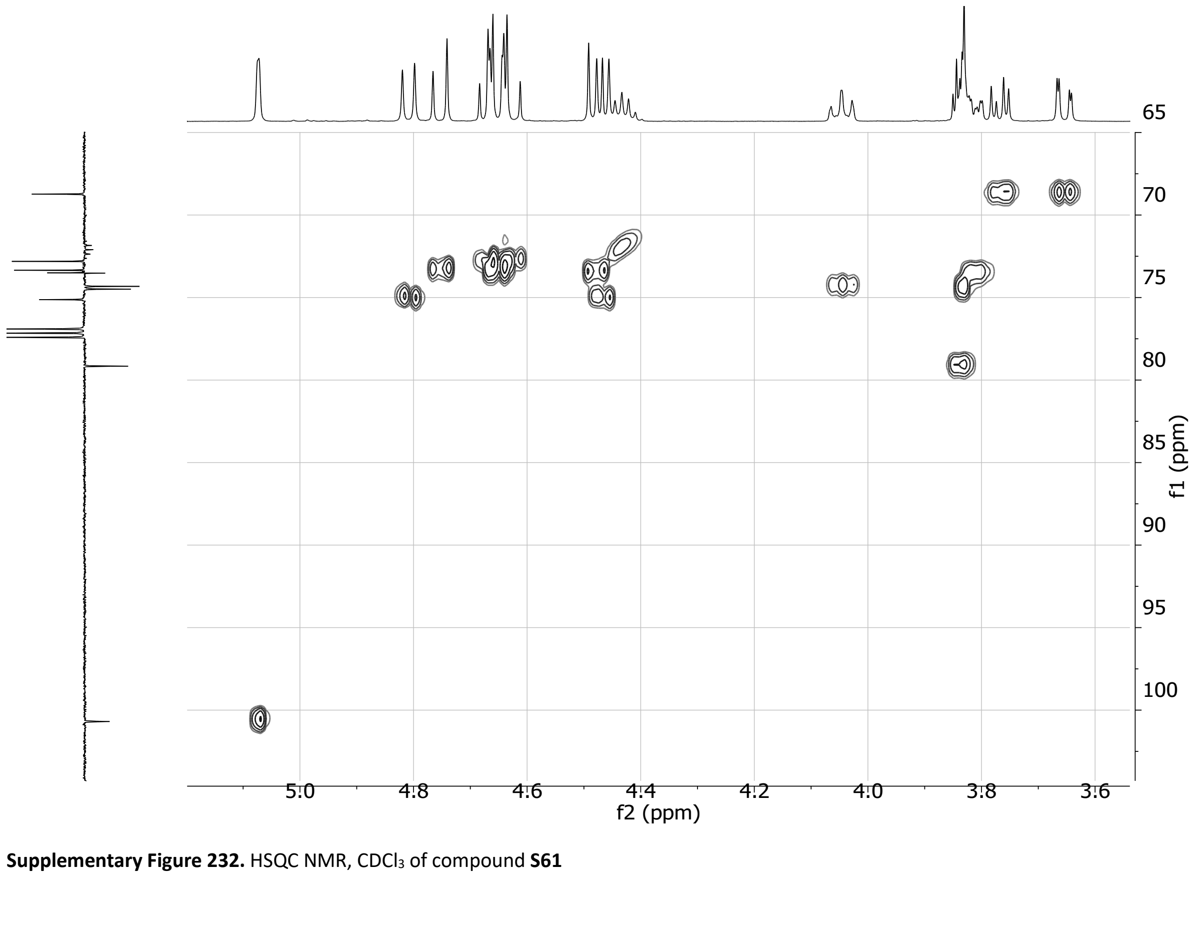

Supplementary Figure 232. HSQC NMR, $CDCl_3$ of compound **S61**

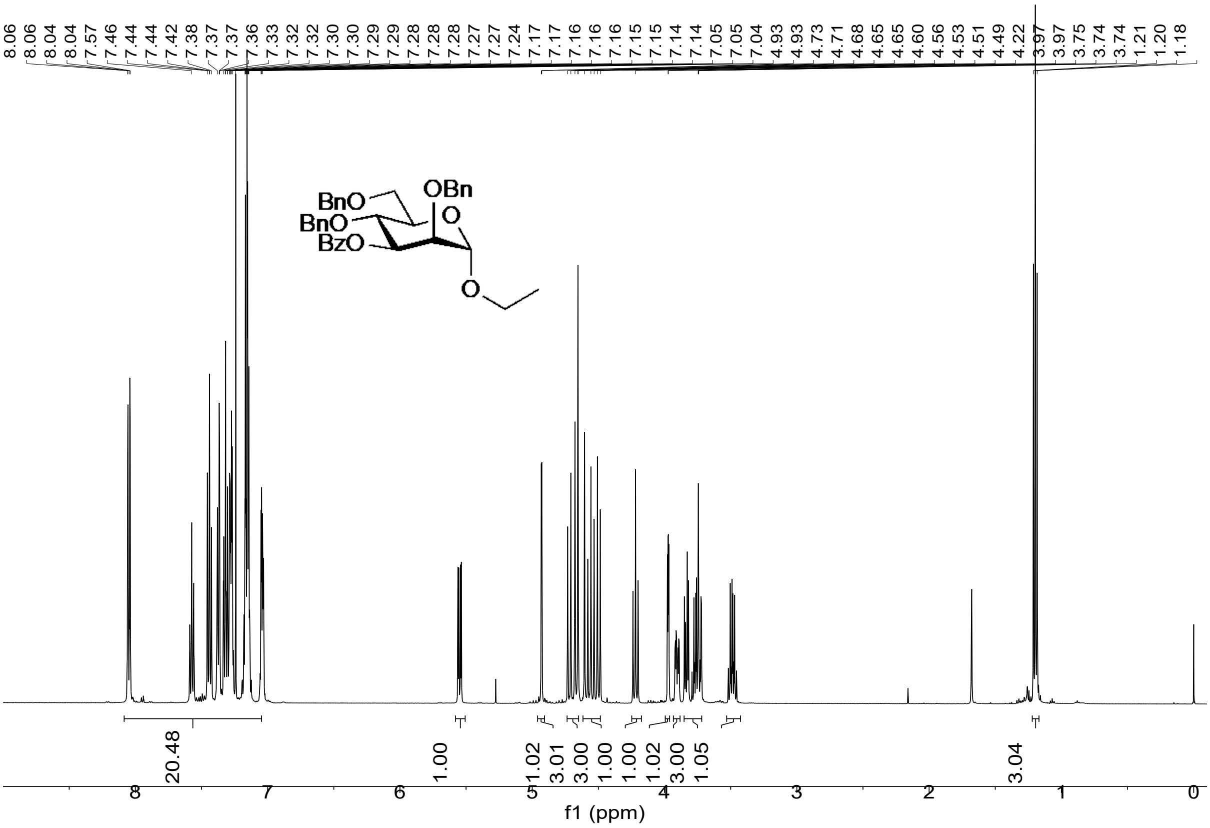

Supplementary Figure 233. ^{1}H NMR, 500 MHz, $CDCl_3$ of compound **S62**

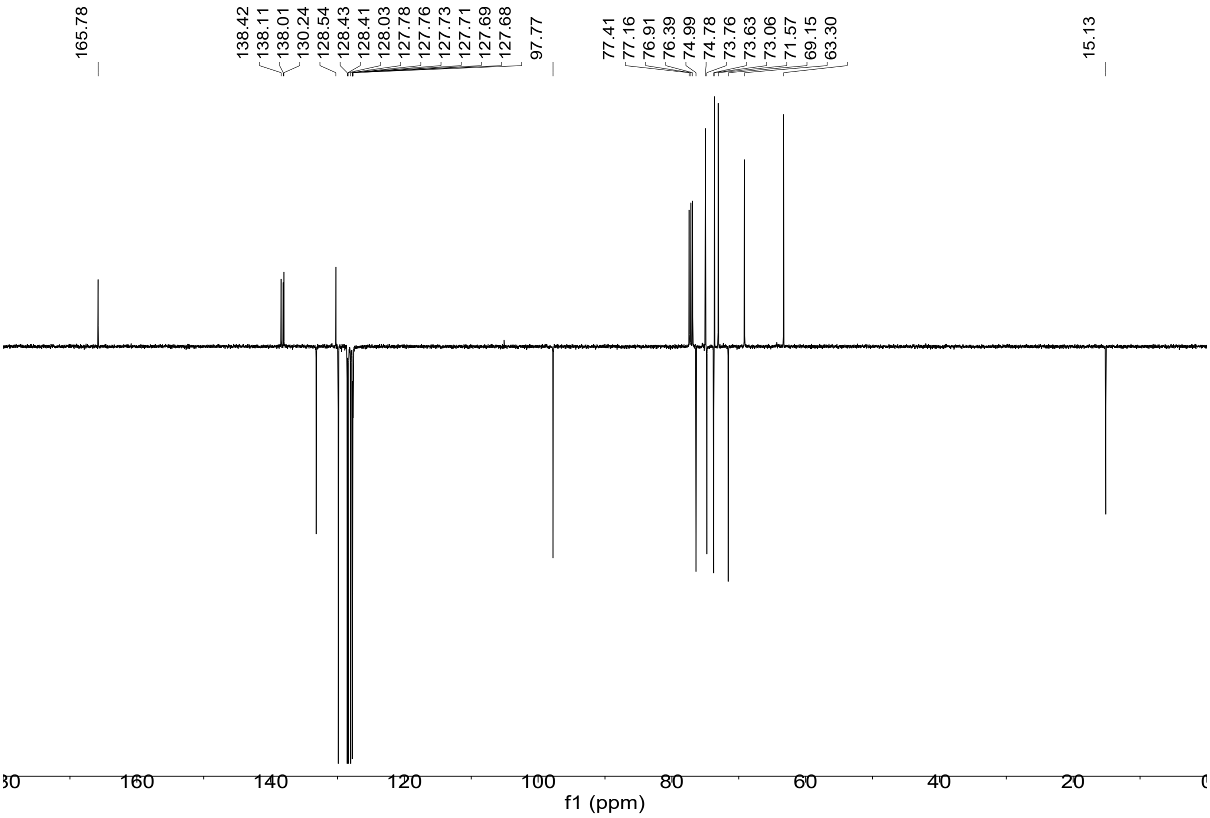

Supplementary Figure 234. ^{13}C NMR, 126 MHz, $CDCl_3$ of compound **S62**

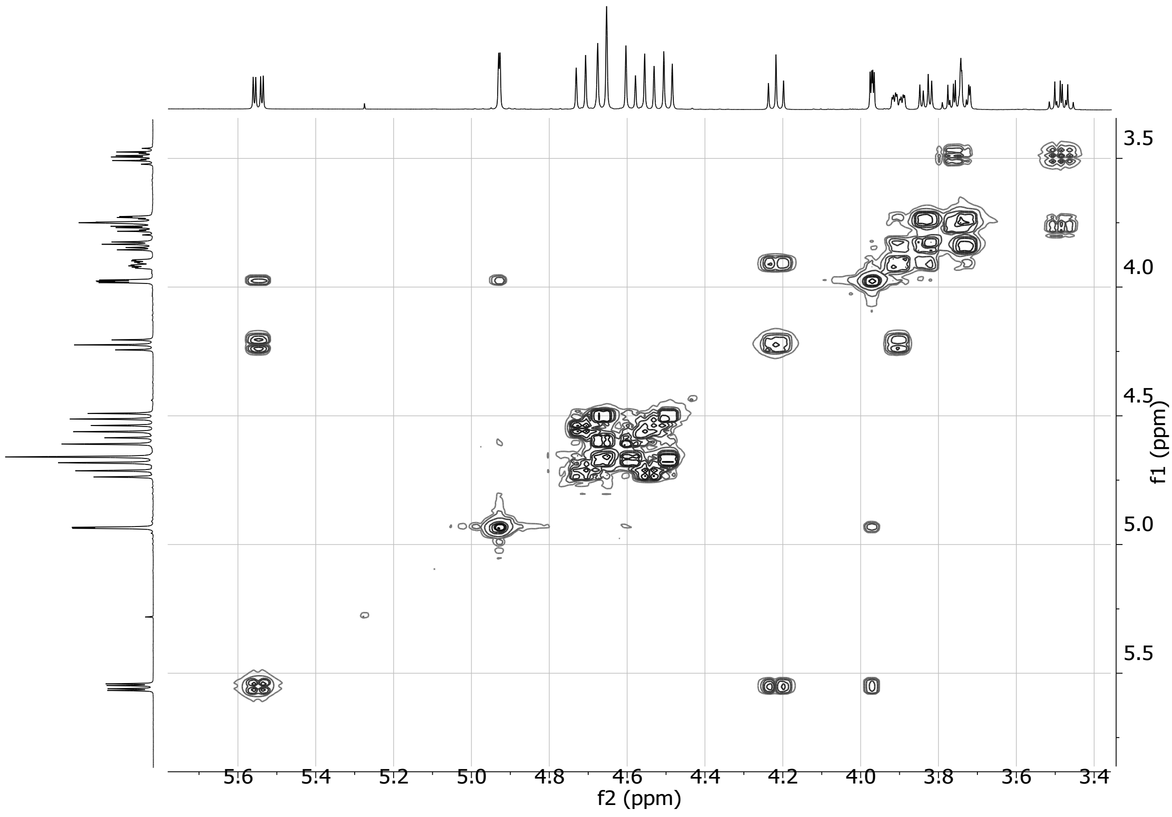

Supplementary Figure 235. HH-COSY NMR, $CDCl_3$ of compound **S62**

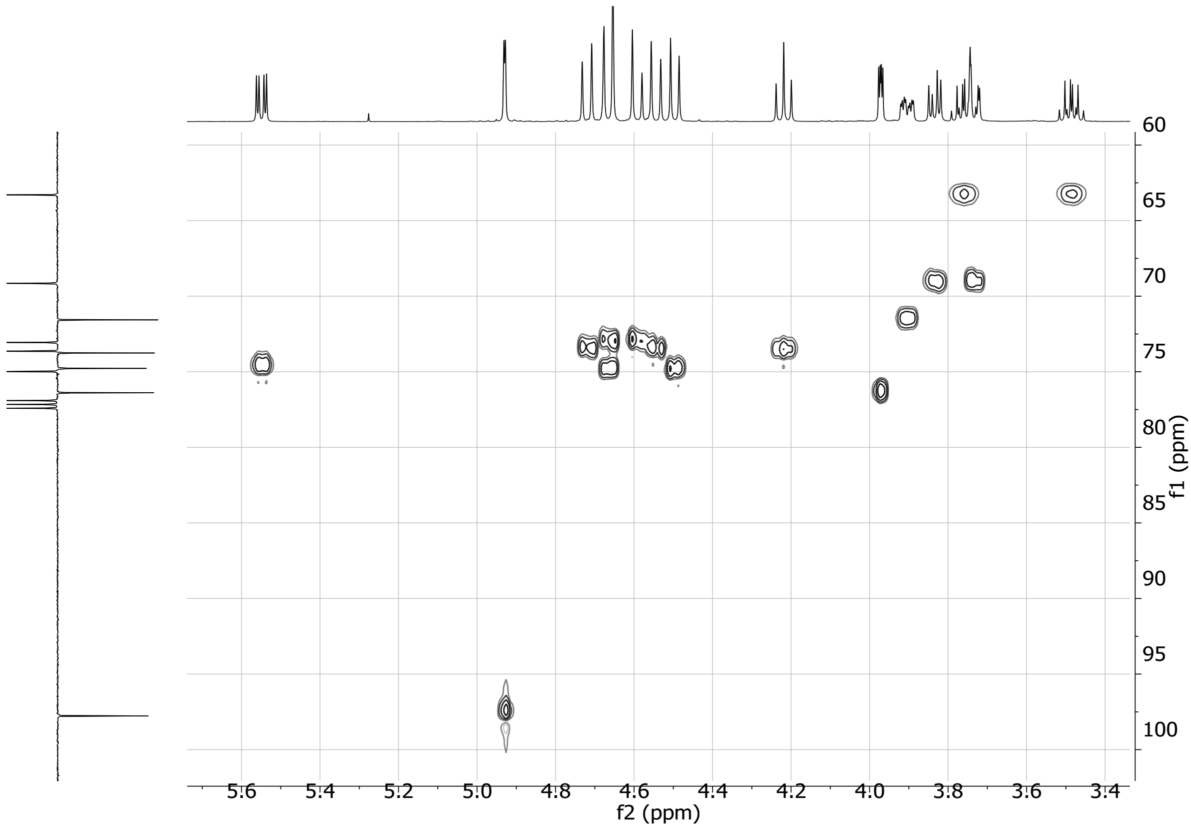

Supplementary Figure 236. HSQC NMR, $CDCl_3$ of compound **S62**

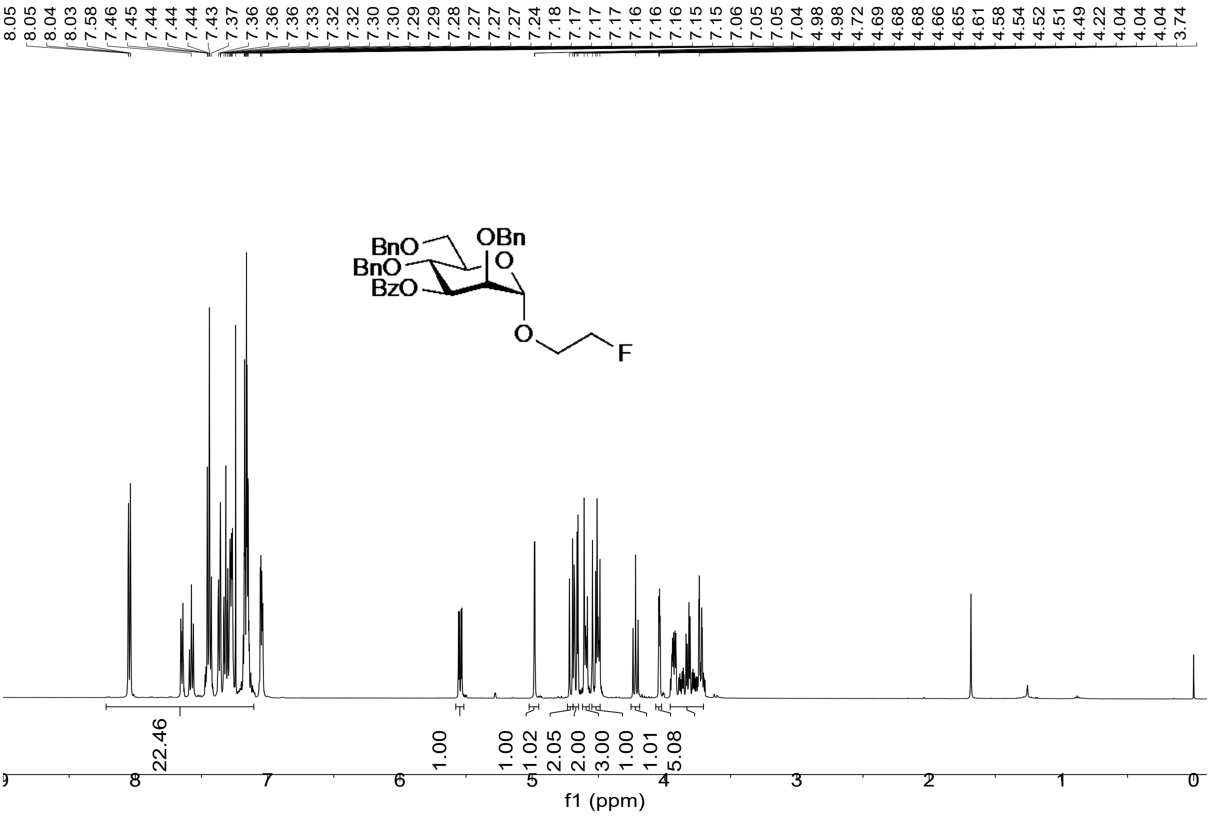

Supplementary Figure 237. ^{1}H NMR, 500 MHz, $CDCl_3$ of compound **S63**

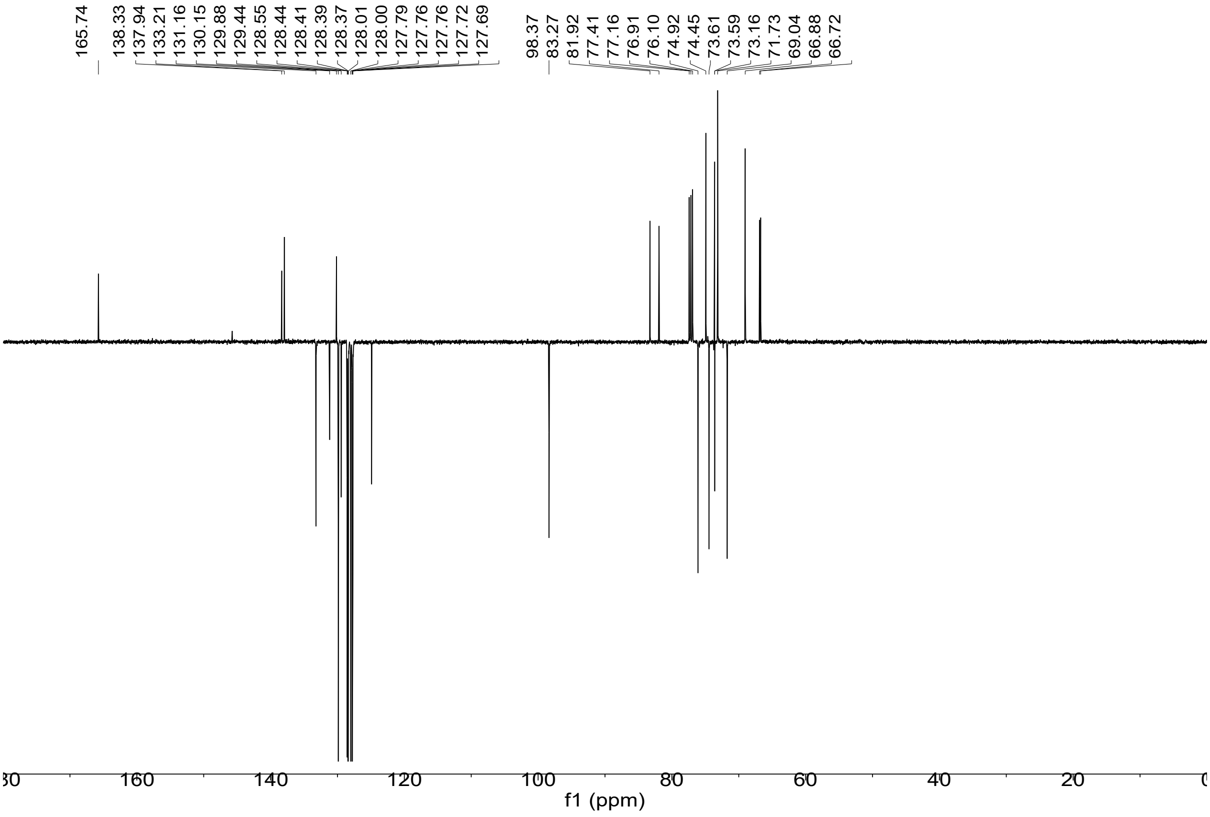

Supplementary Figure 238. ^{13}C NMR, 126 MHz, $CDCl_3$ of compound **S63**

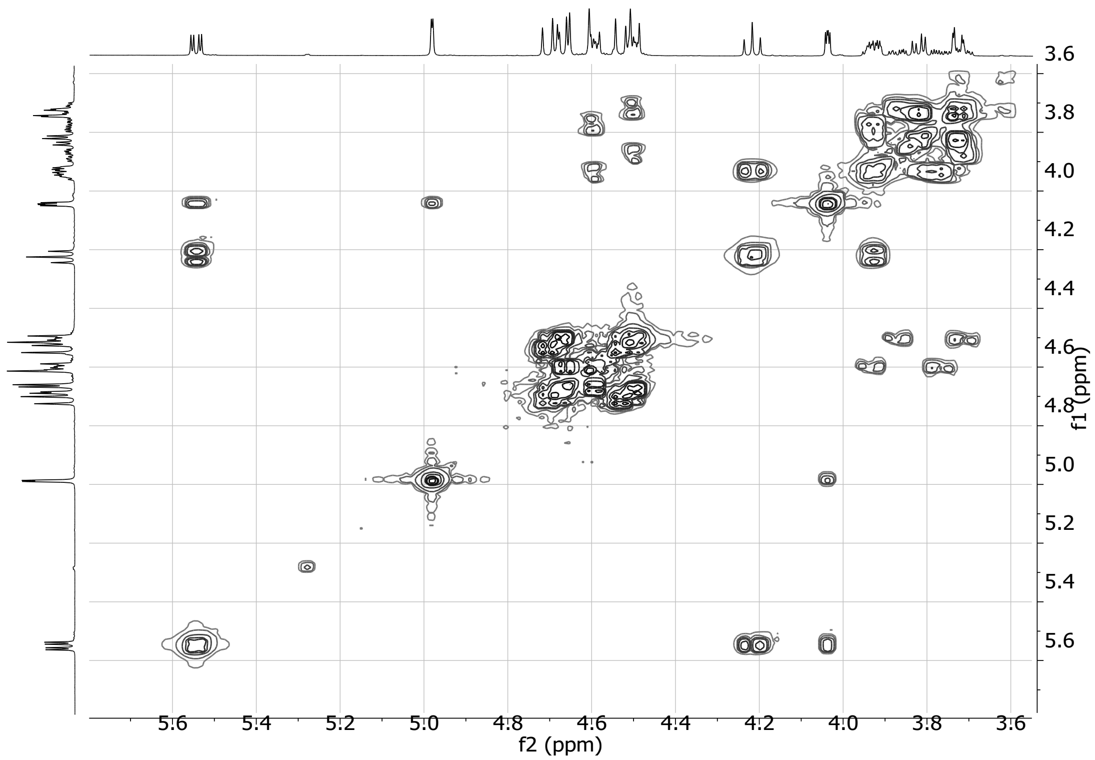

Supplementary Figure 239. HH-COSY NMR, $CDCl_3$ of compound **S63**

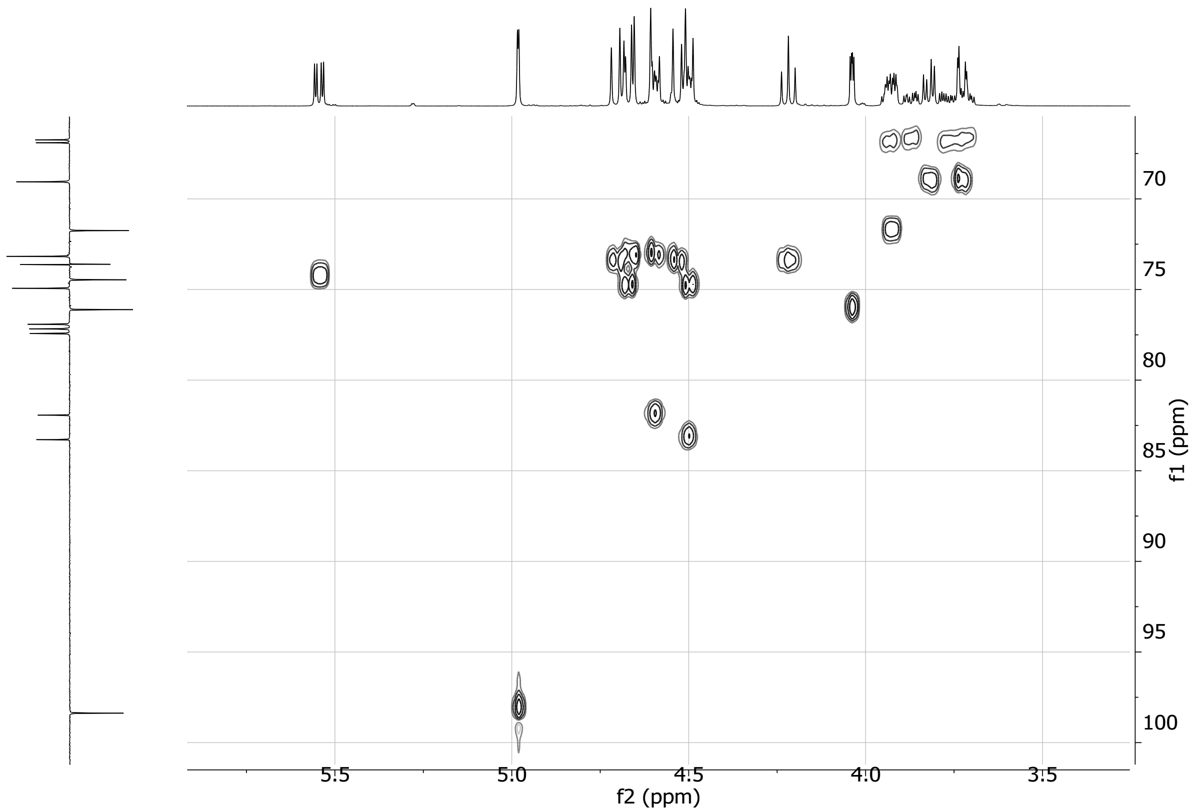

Supplementary Figure 240. HSQC NMR, $CDCl_3$ of compound **S63**

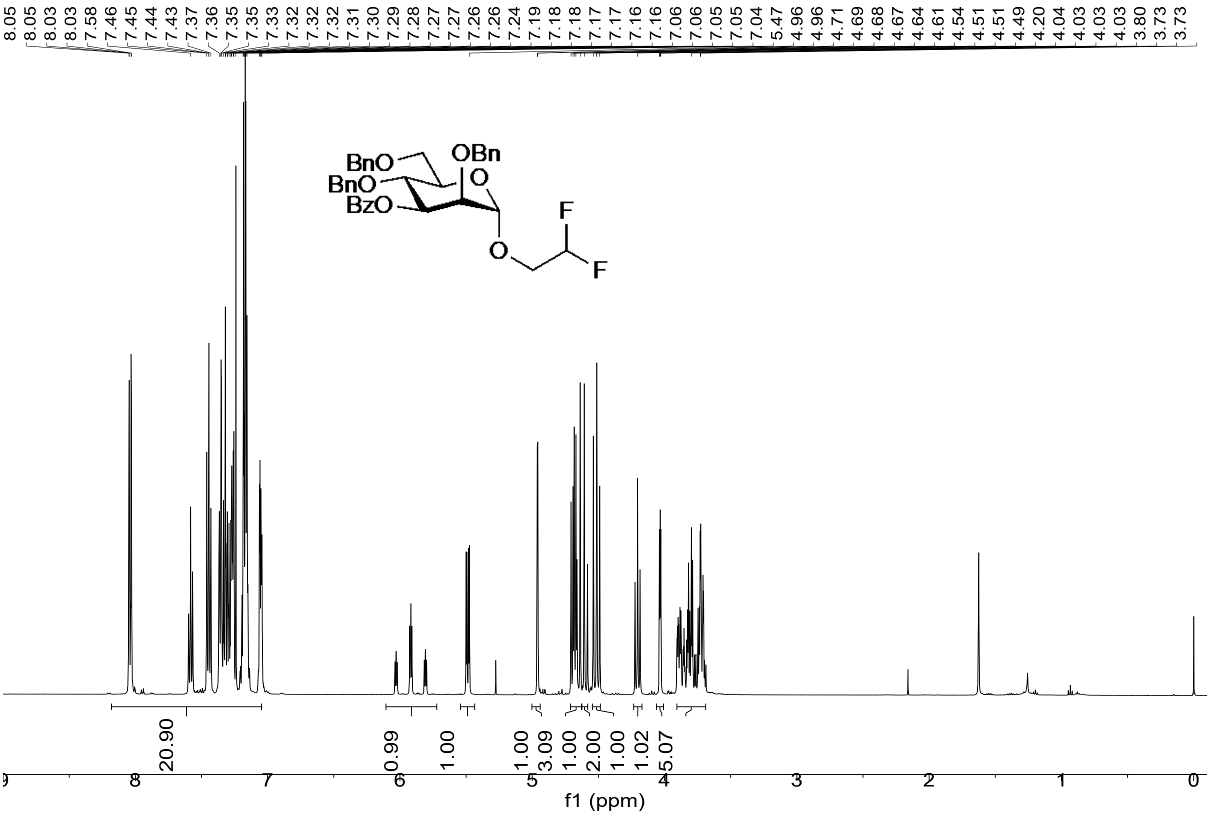

Supplementary Figure 241. ^1H NMR, 500 MHz, $CDCl_3$ of compound **S64**

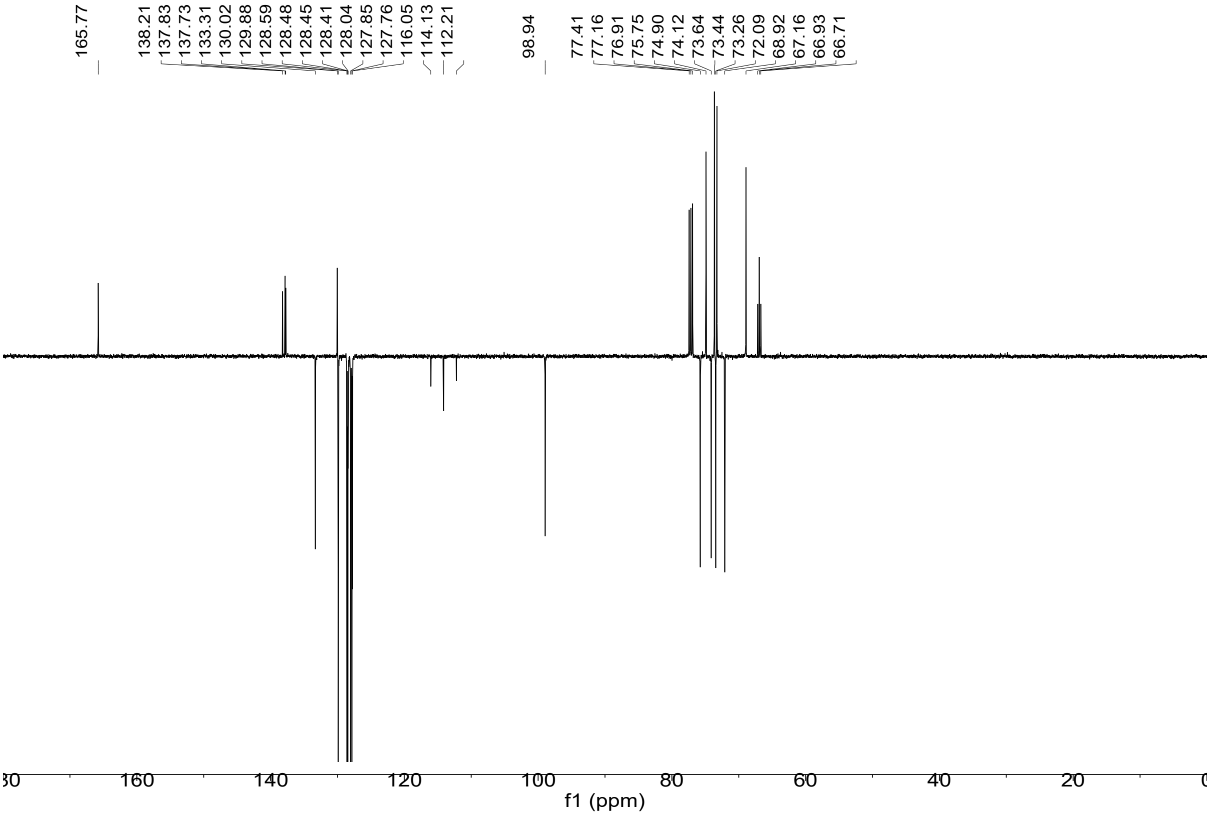

Supplementary Figure 242. ^{13}C NMR, 126 MHz, $CDCl_3$ of compound **S64**

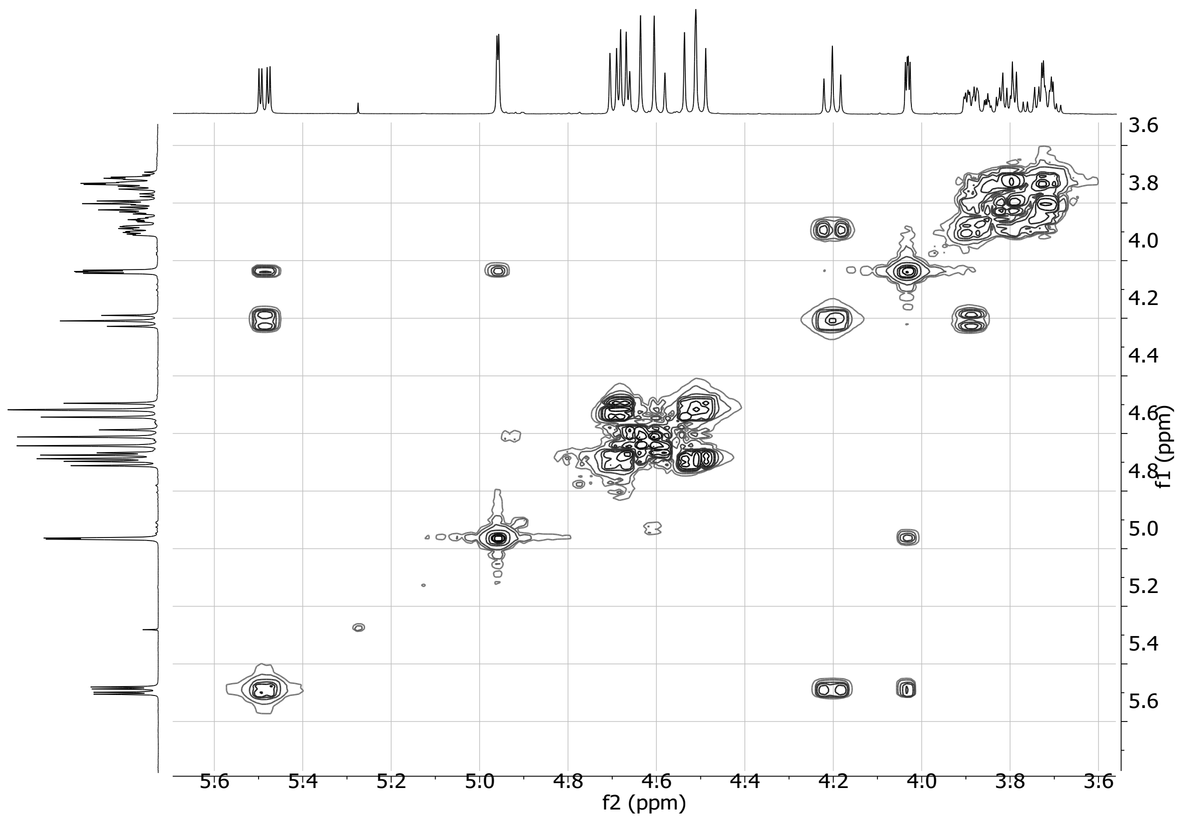

Supplementary Figure 243. HH-COSY NMR, $CDCl_3$ of compound **S64**

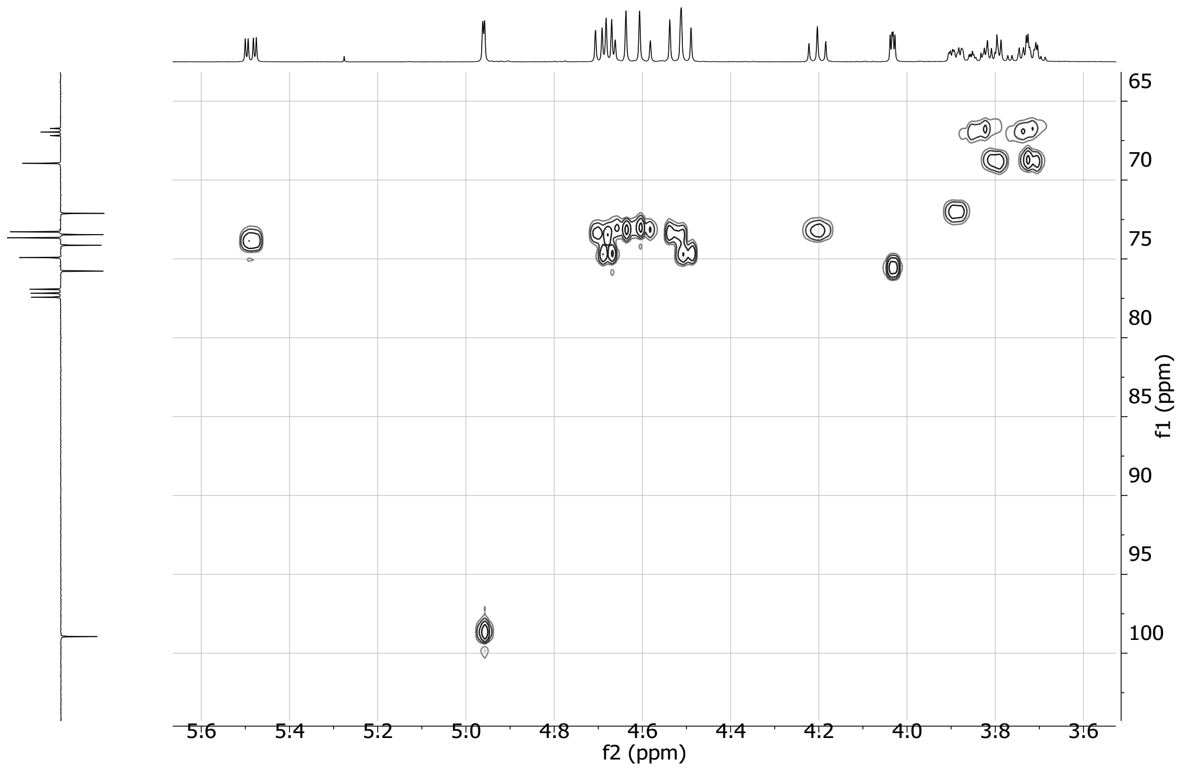

Supplementary Figure 244. HSQC NMR, $CDCl_3$ of compound **S64**

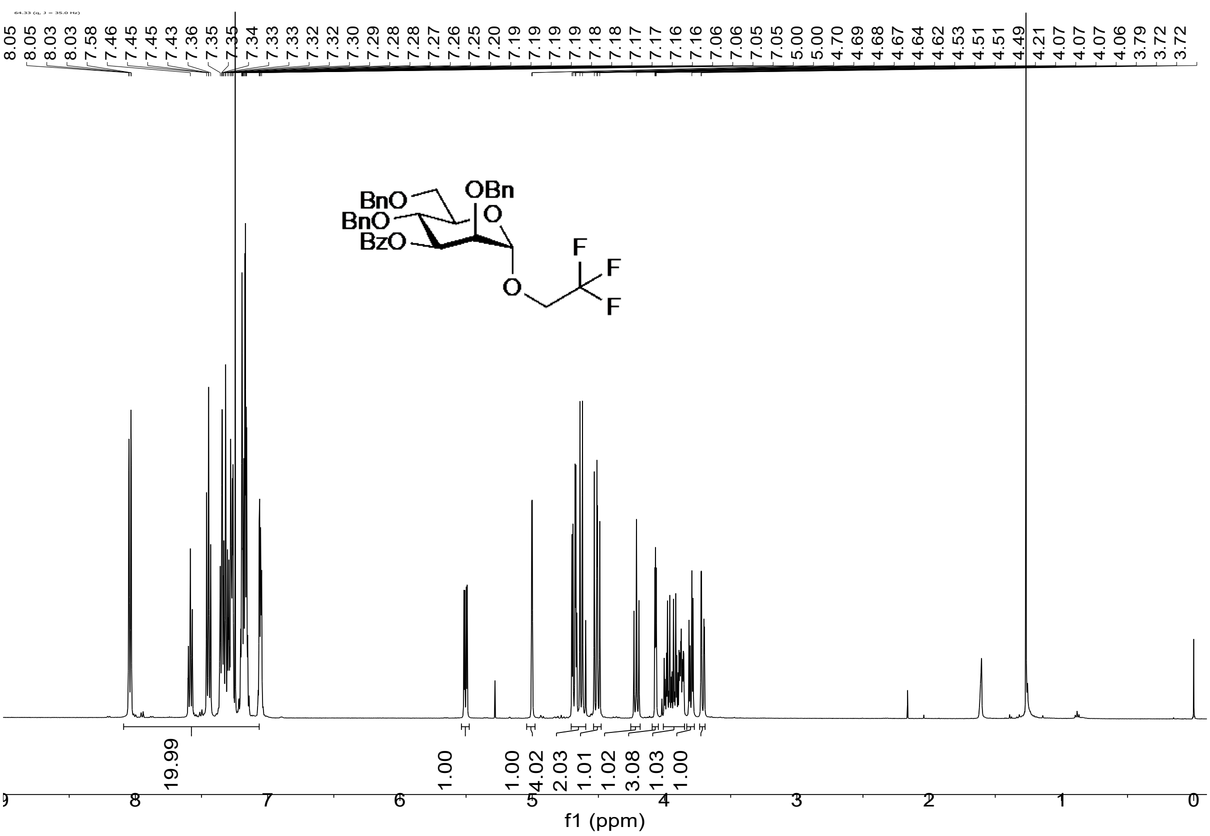

Supplementary Figure 245. ^{1}H NMR, 500 MHz, $CDCl_3$ of compound **S65**

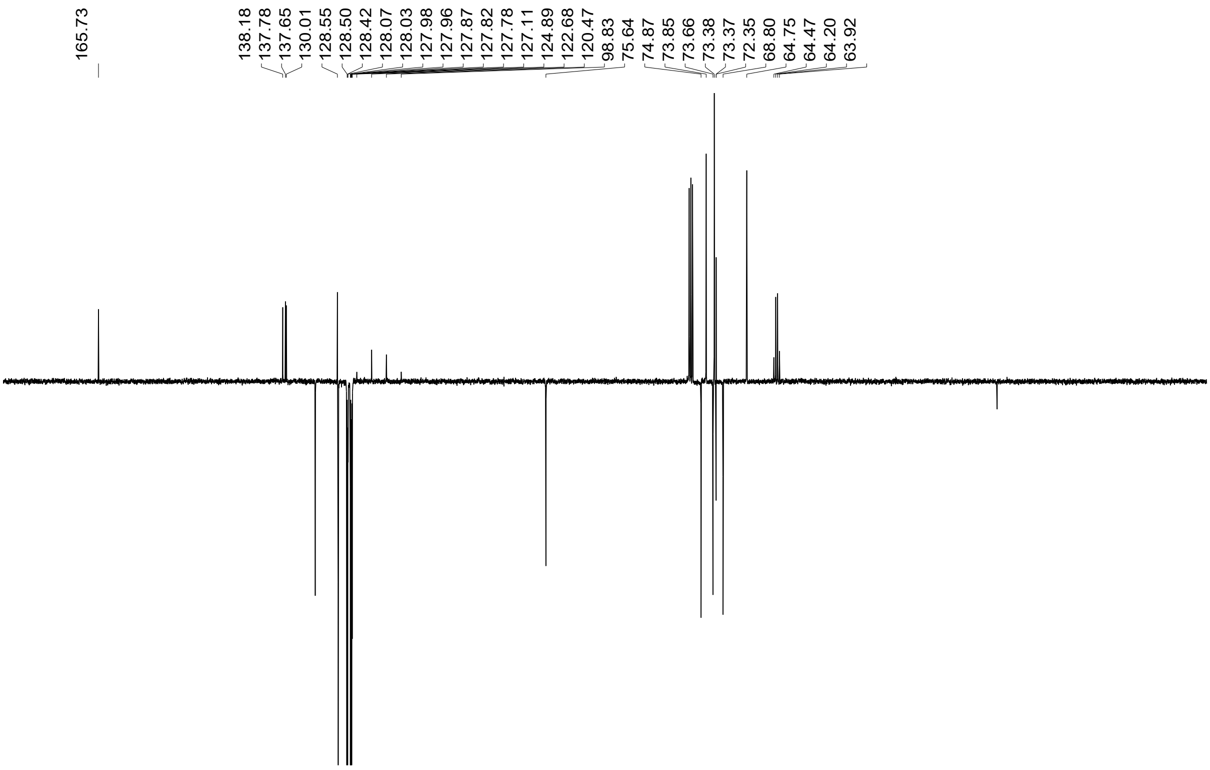

Supplementary Figure 246. ^{13}C NMR, 126 MHz, $CDCl_3$ of compound **S65**

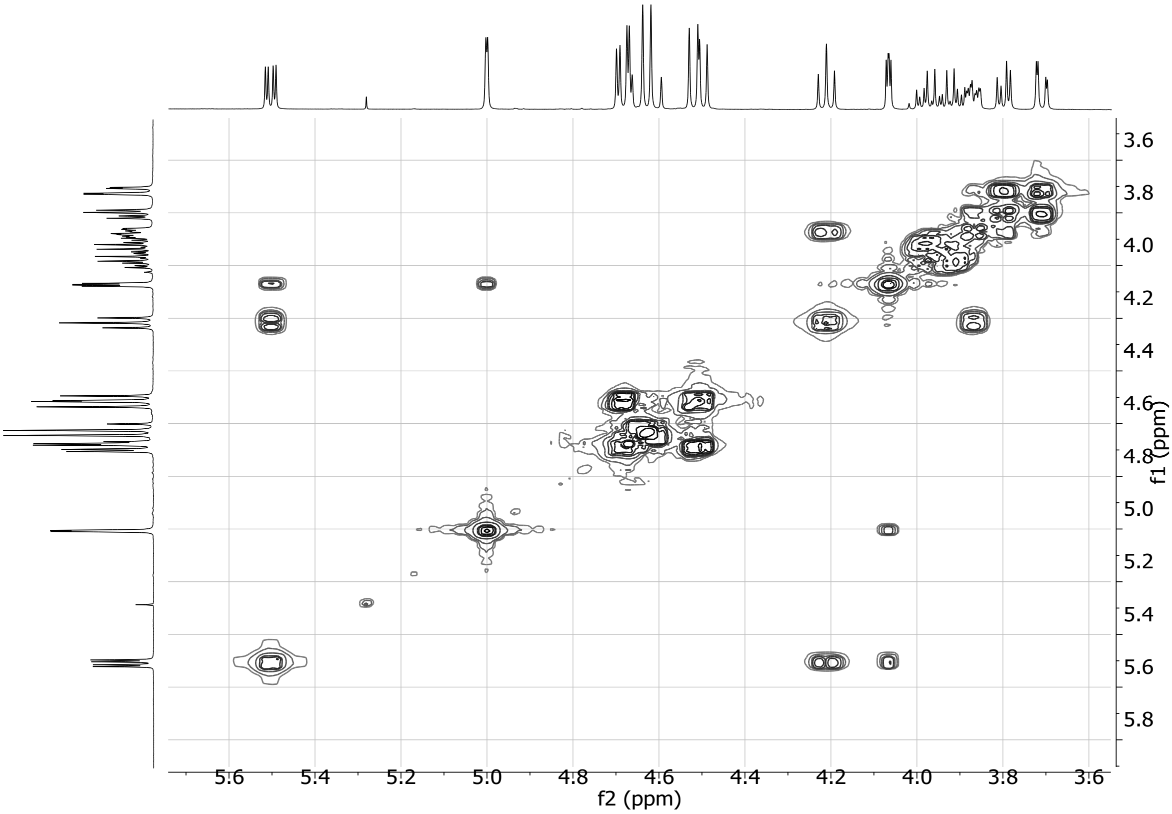

Supplementary Figure 247. HH-COSY NMR, $CDCl_3$ of compound **S65**

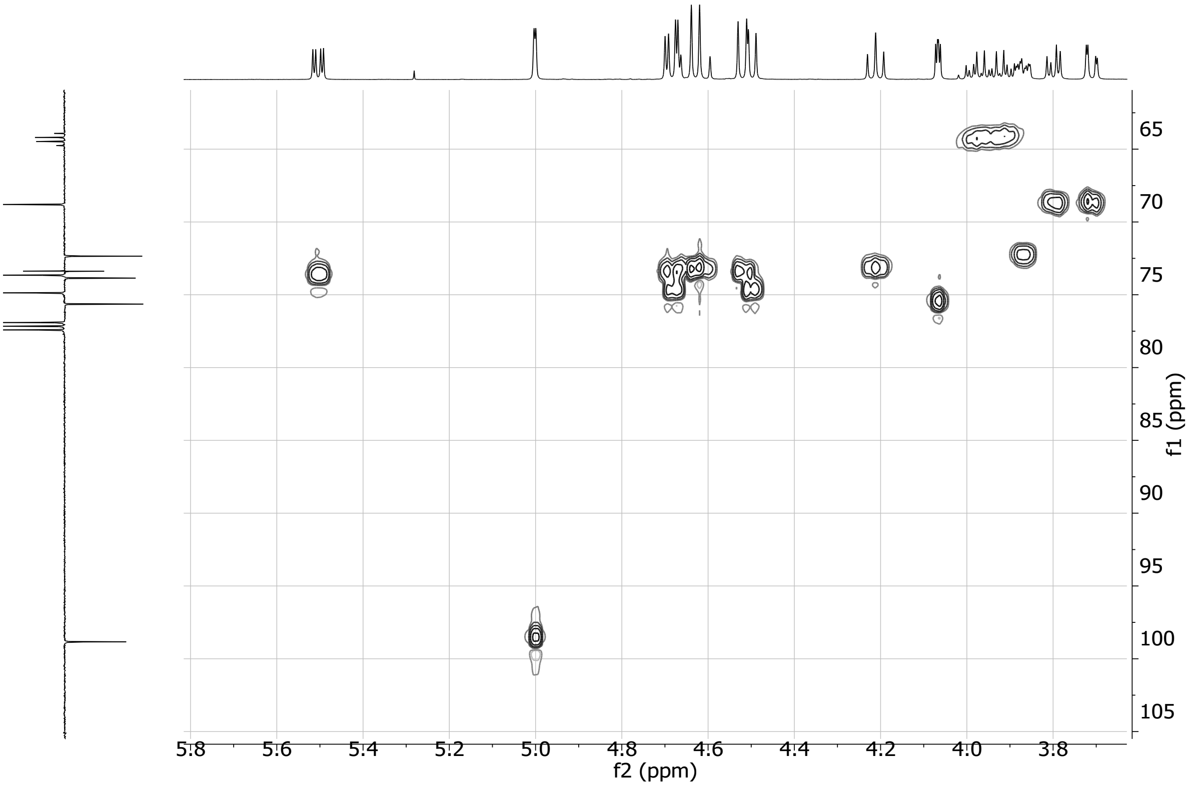

Supplementary Figure 248. HSQC NMR, $CDCl_3$ of compound **S65**

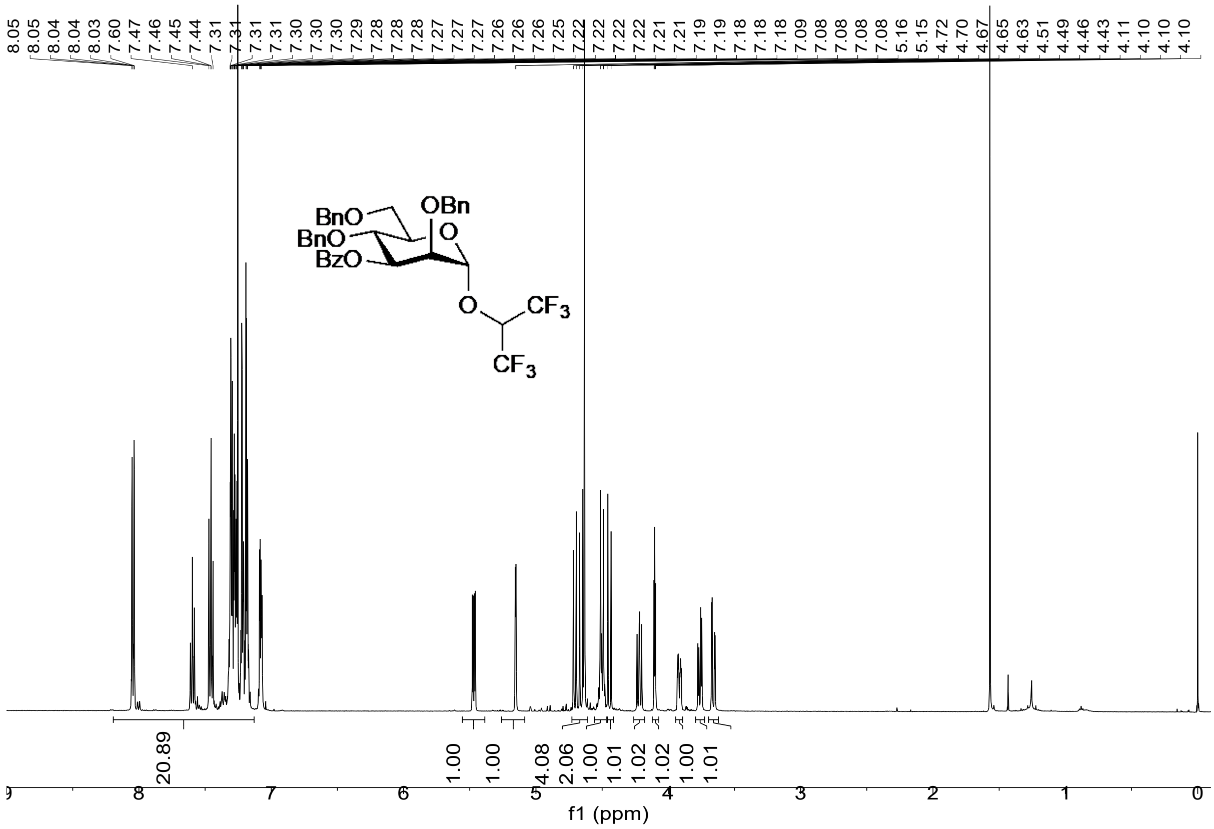

Supplementary Figure 249. ^{1}H NMR, 500 MHz, $CDCl_3$ of compound **S66**

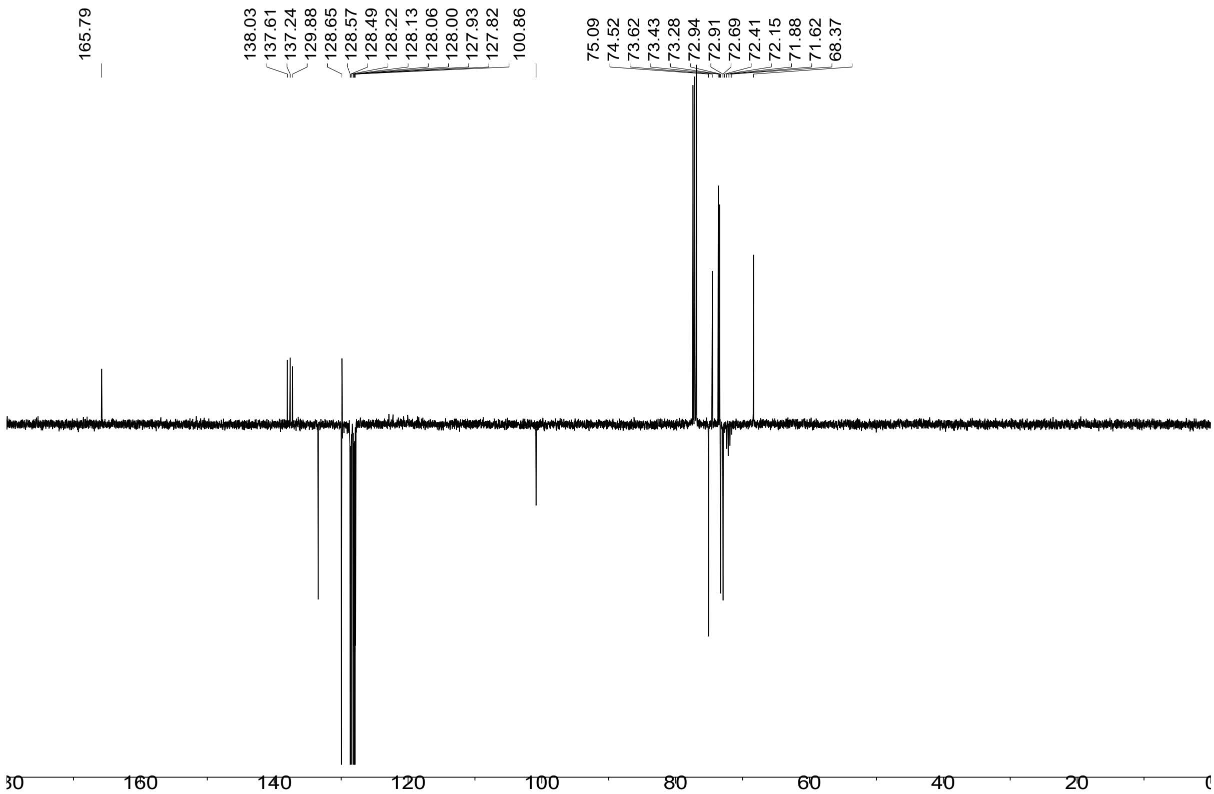

Supplementary Figure 250. ^{13}C NMR, 126 MHz, $CDCl_3$ of compound **S66**

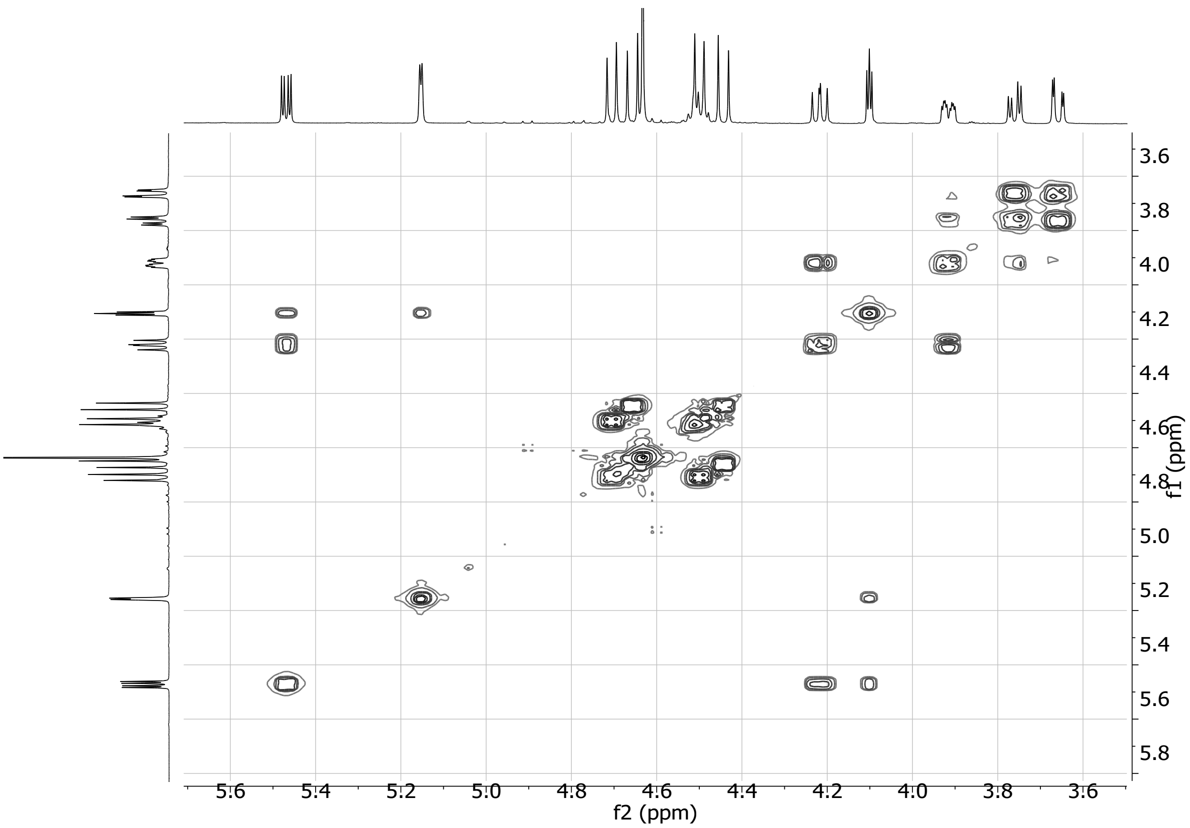

Supplementary Figure 251. HH-COSY NMR, $CDCl_3$ of compound **S66**

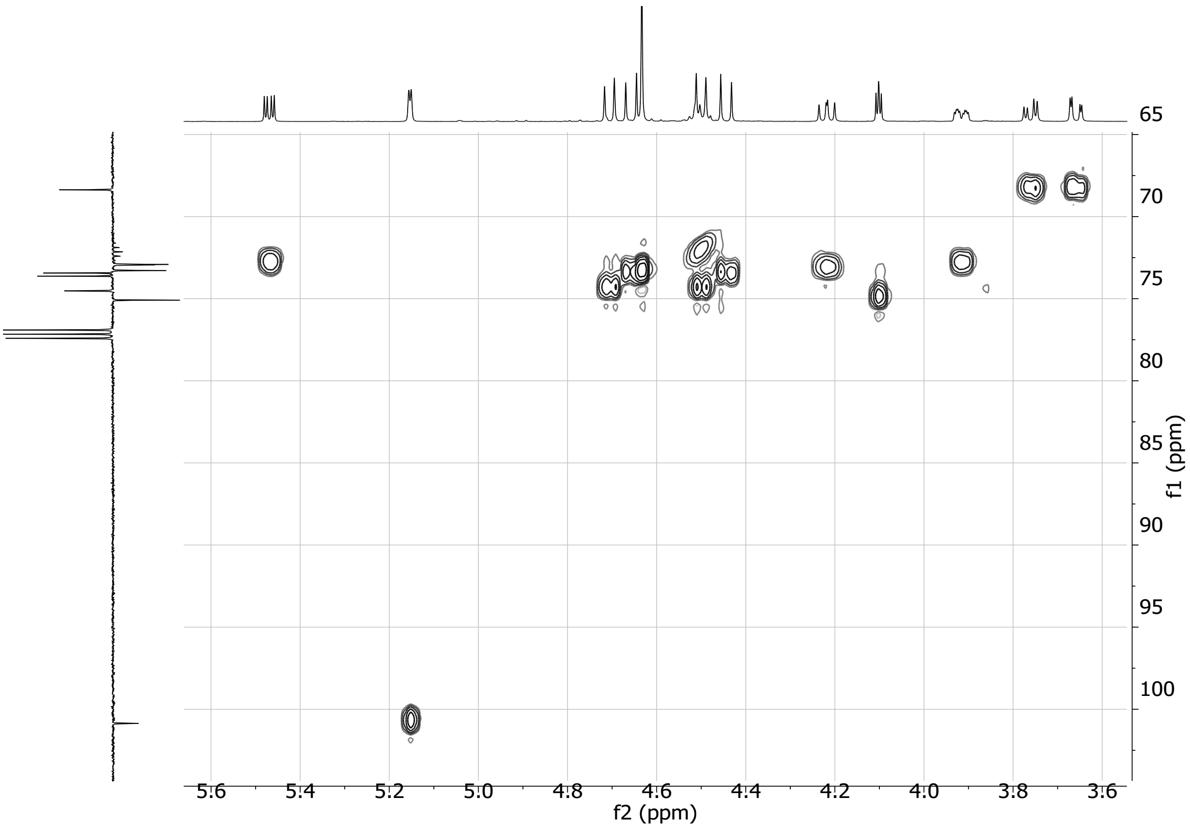

Supplementary Figure 252. HSQC NMR, $CDCl_3$ of compound **S66**

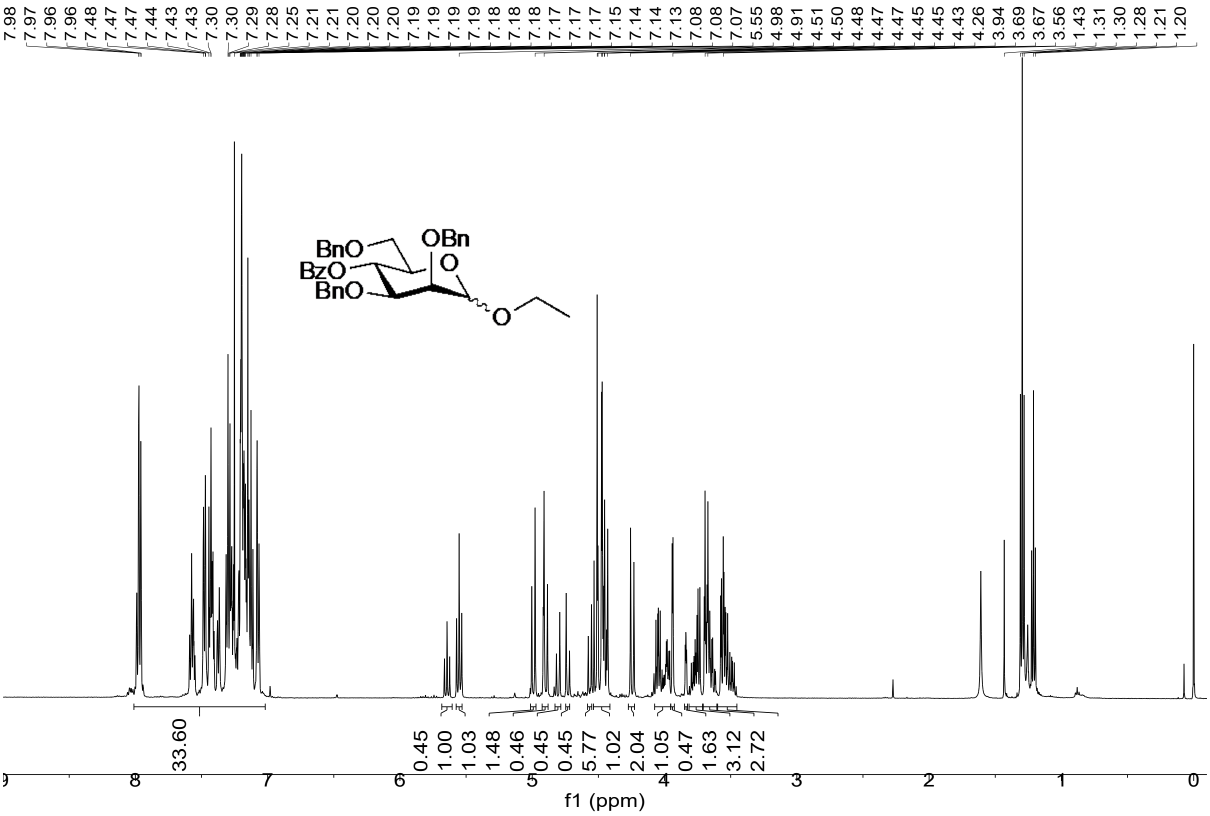

Supplementary Figure 253. ^{1}H NMR, 500 MHz, $CDCl_3$ of compound **S67**

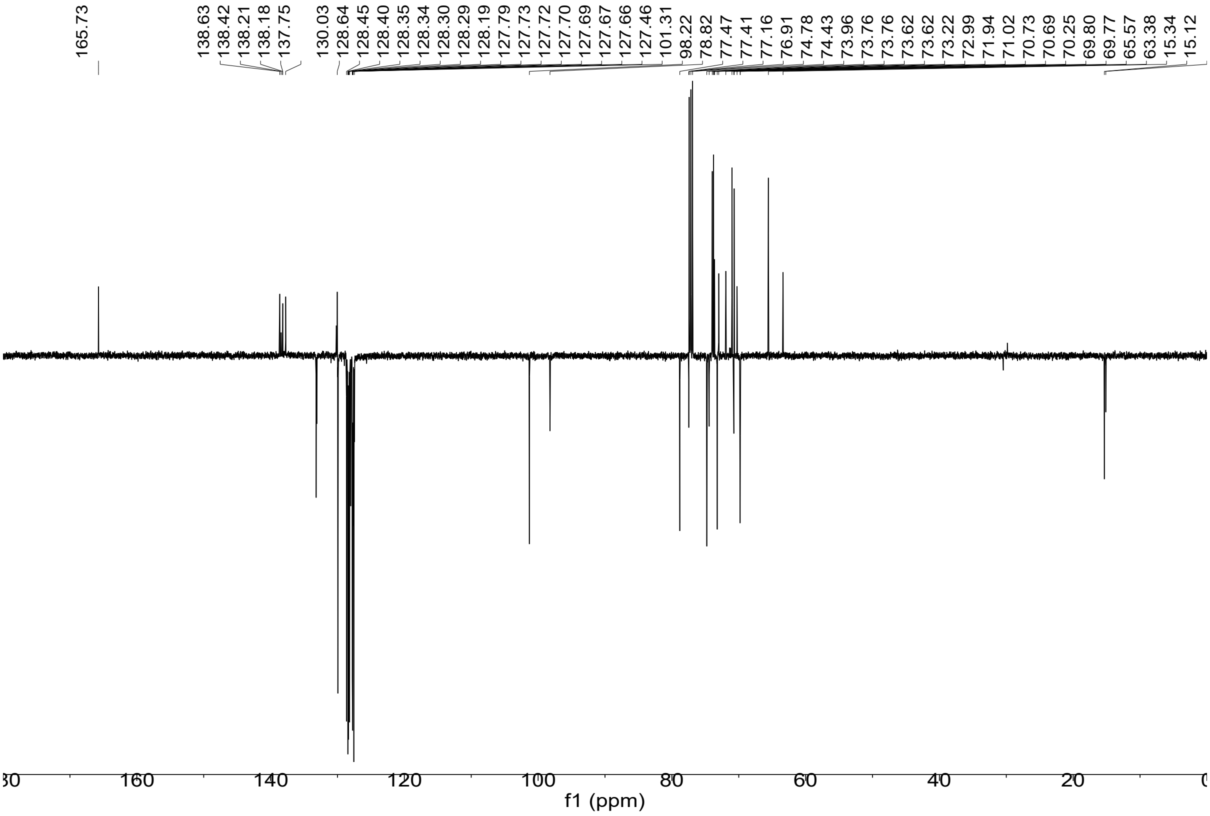

Supplementary Figure 254. ^{13}C NMR, 126 MHz, $CDCl_3$ of compound **S67**

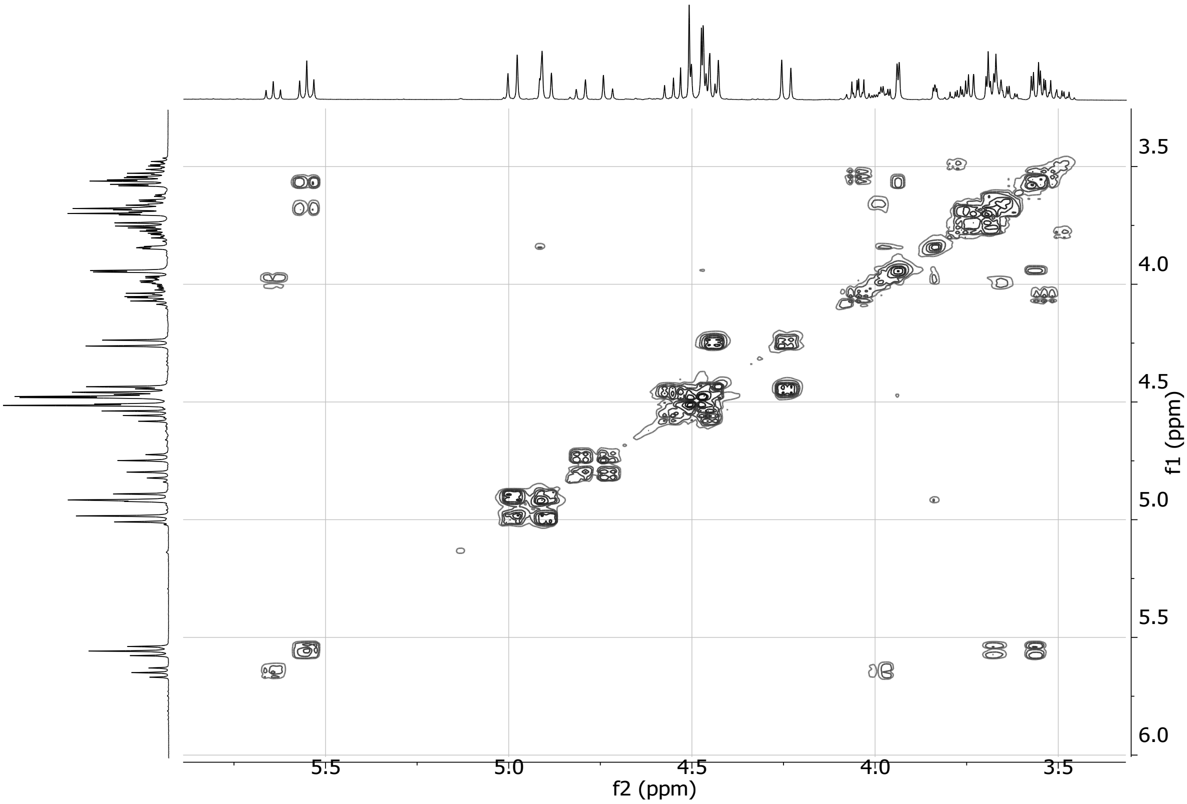

Supplementary Figure 255. HH-COSY NMR, $CDCl_3$ of compound **S67**

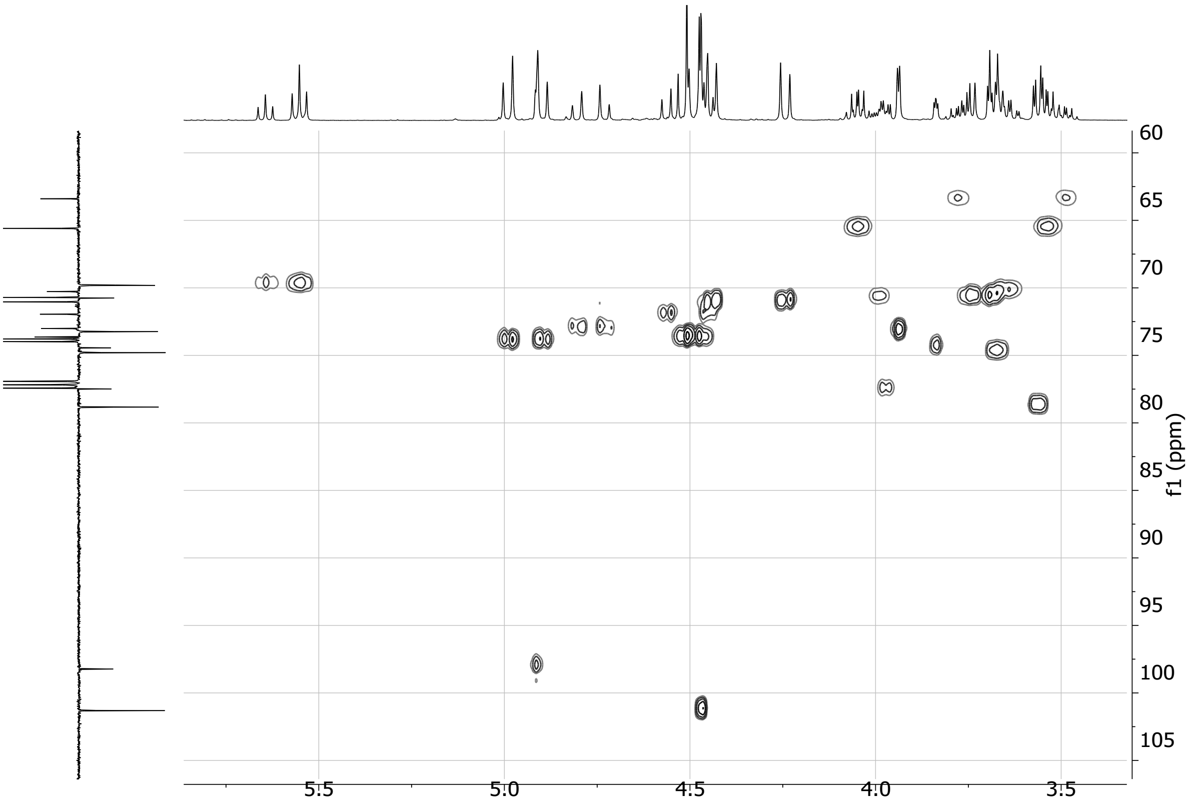

Supplementary Figure 256. HSQC NMR, $CDCl_3$ of compound **S67**

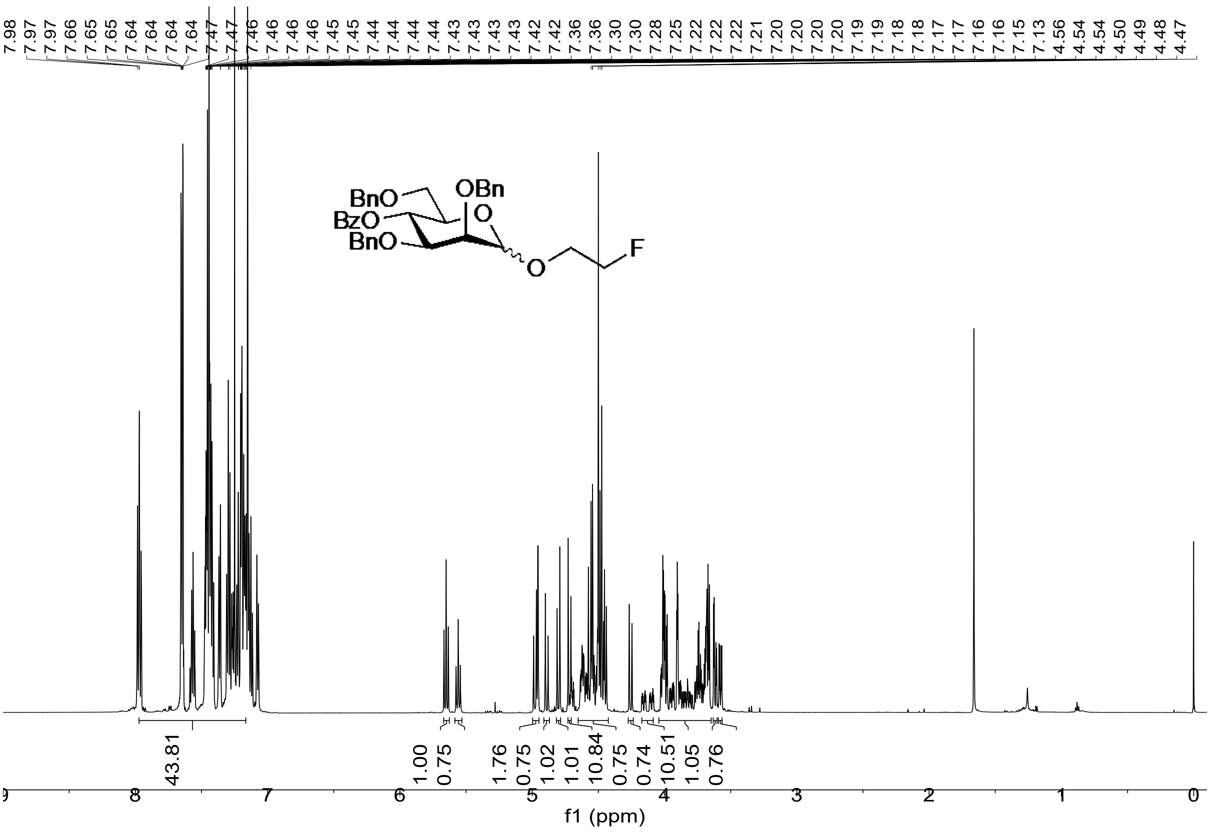

Supplementary Figure 257. ^{1}H NMR, 600 MHz, $CDCl_3$ of compound **S68**

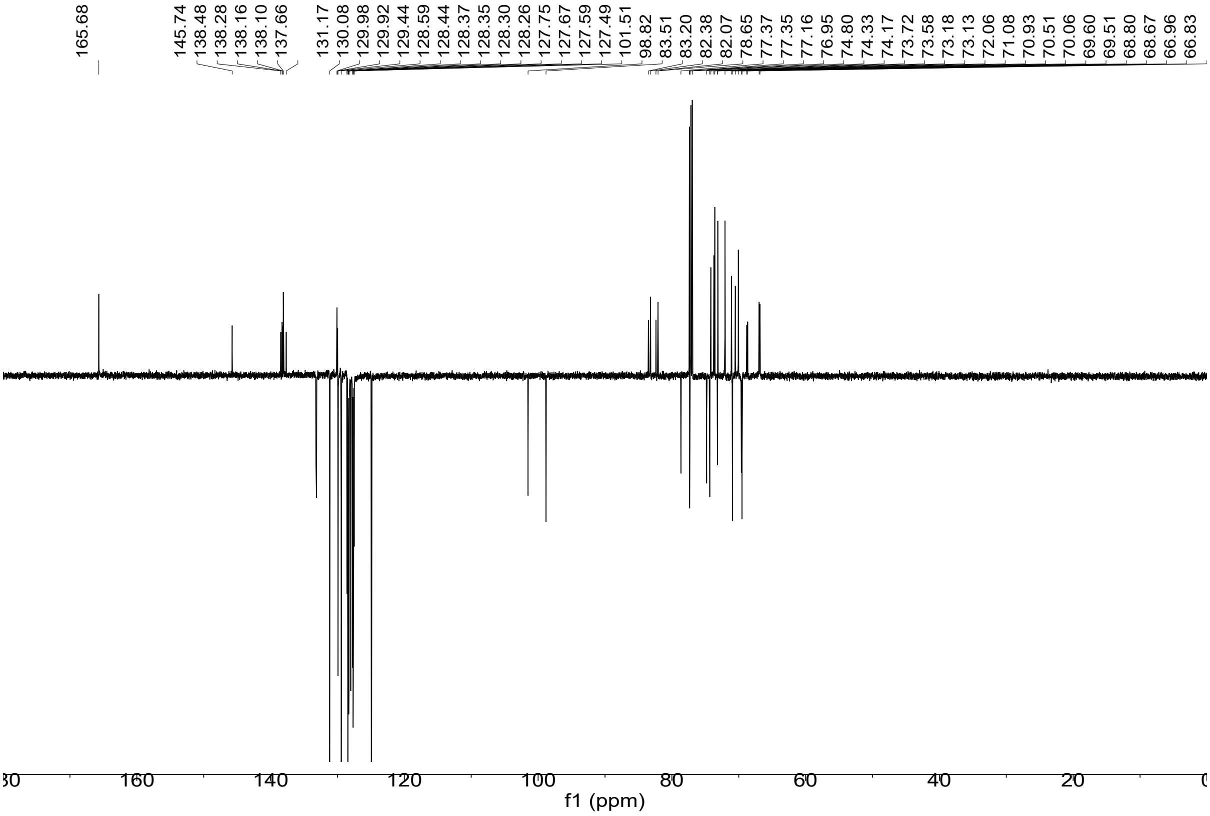

Supplementary Figure 258. ^{13}C NMR, 151 MHz, $CDCl_3$ of compound **S68**

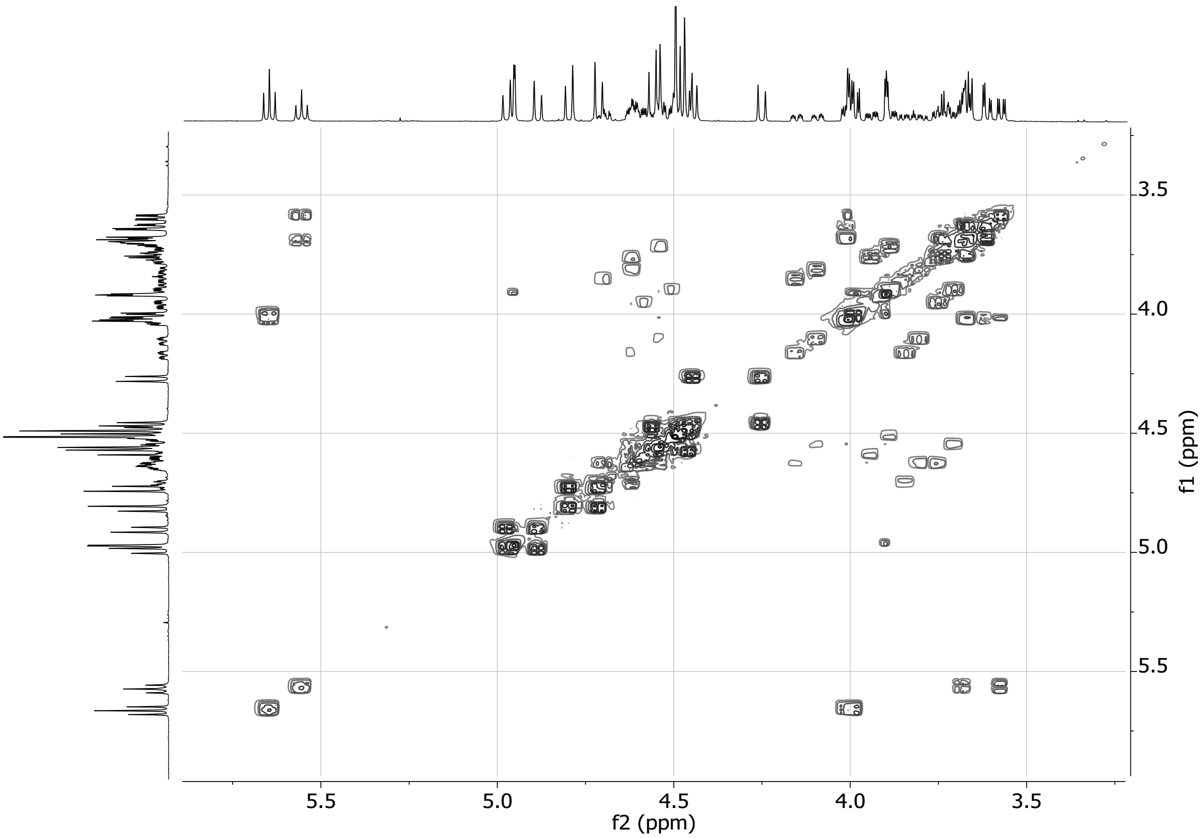

Supplementary Figure 259. HH-COSY NMR, $CDCl_3$ of compound **S68**

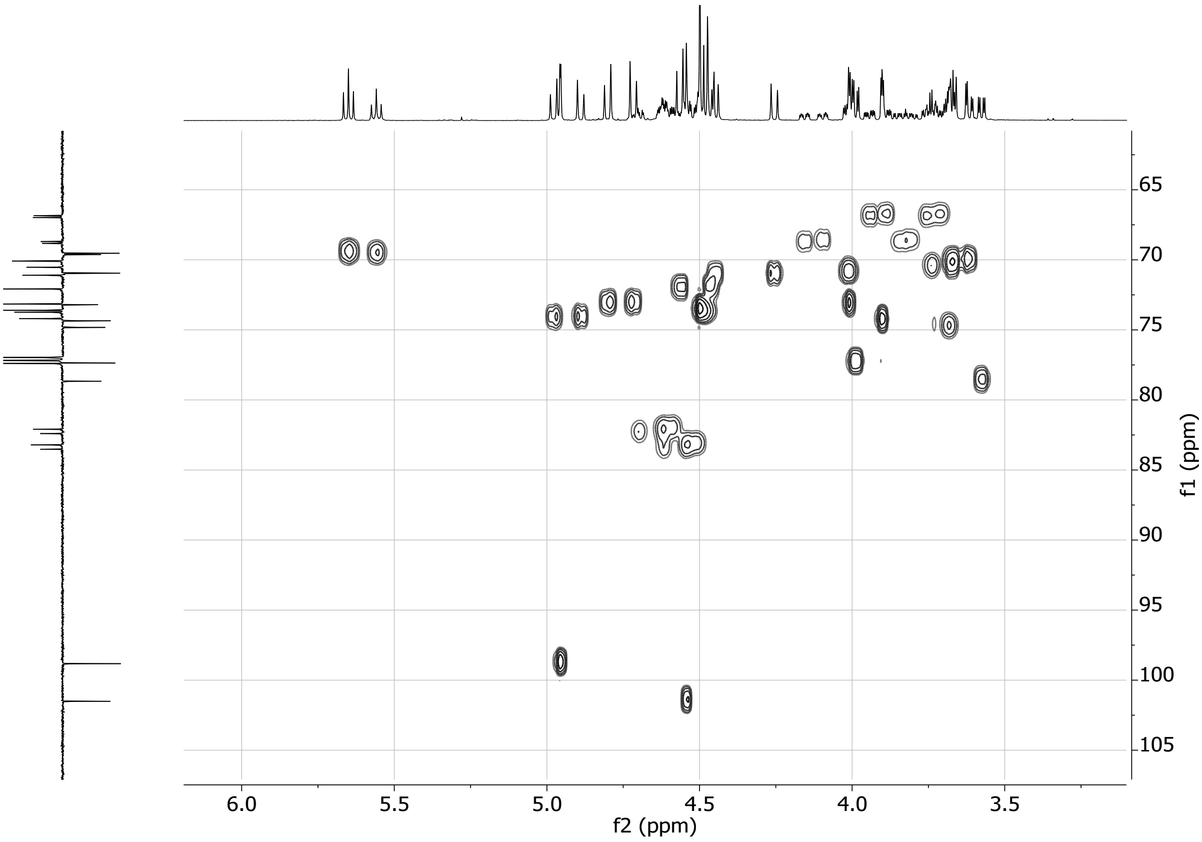

Supplementary Figure 260. HSQC NMR, $CDCl_3$ of compound **S68**

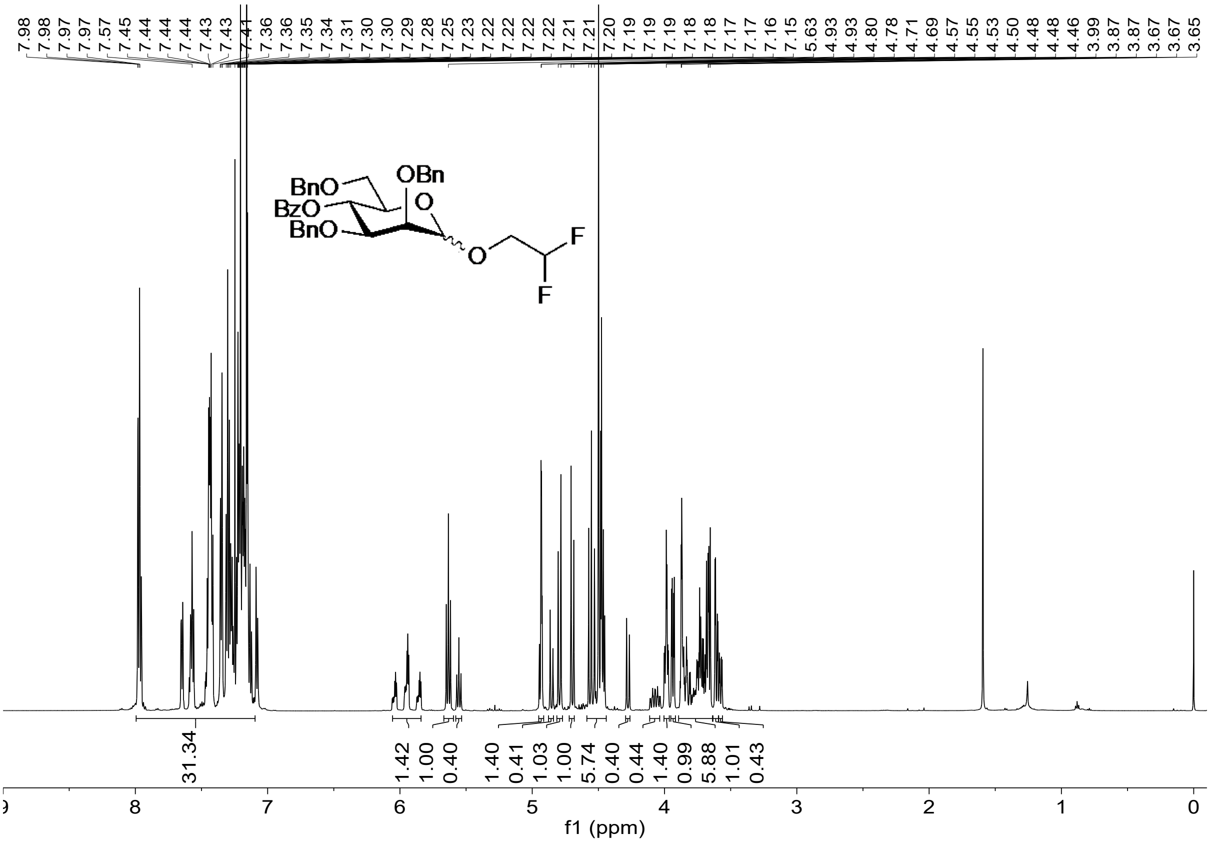

Supplementary Figure 261. ^{1}H NMR, 600 MHz, $CDCl_3$ of compound **S69**

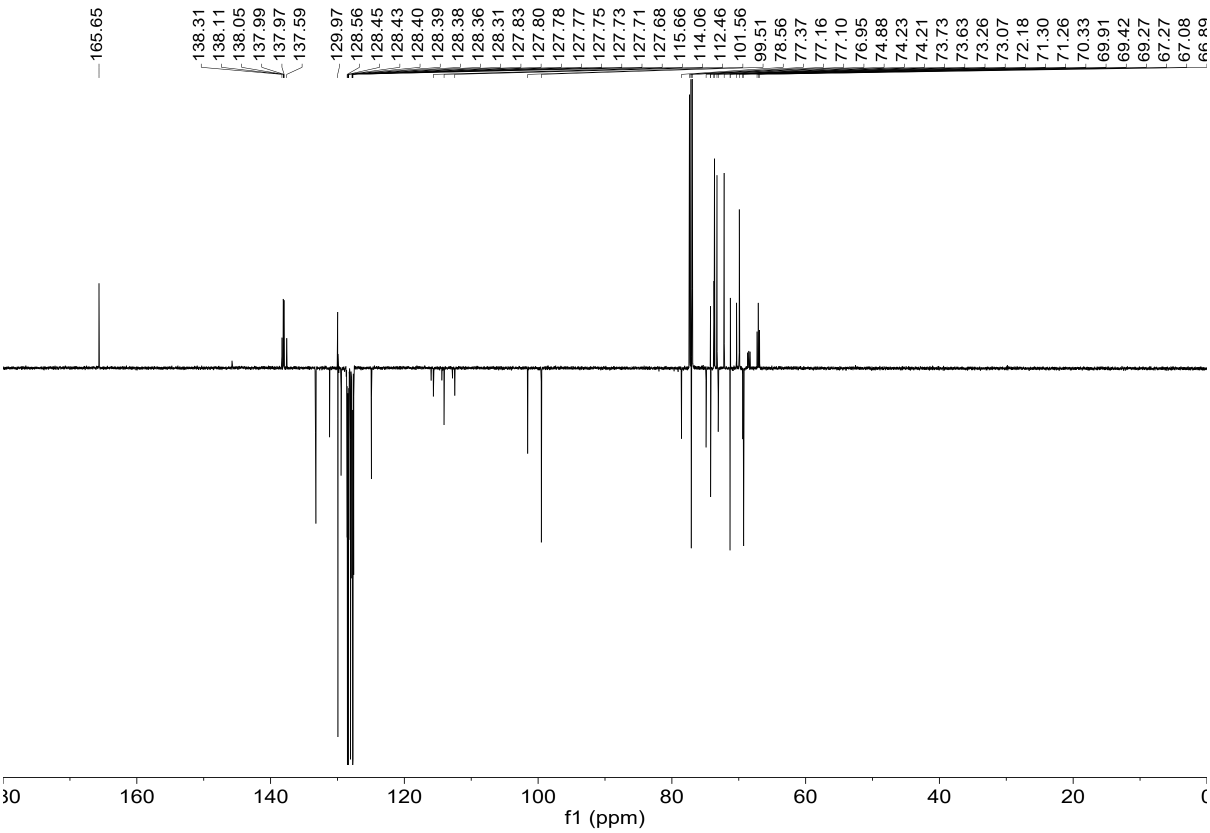

Supplementary Figure 262. ^{13}C NMR, 151 MHz, $CDCl_3$ of compound **S69**

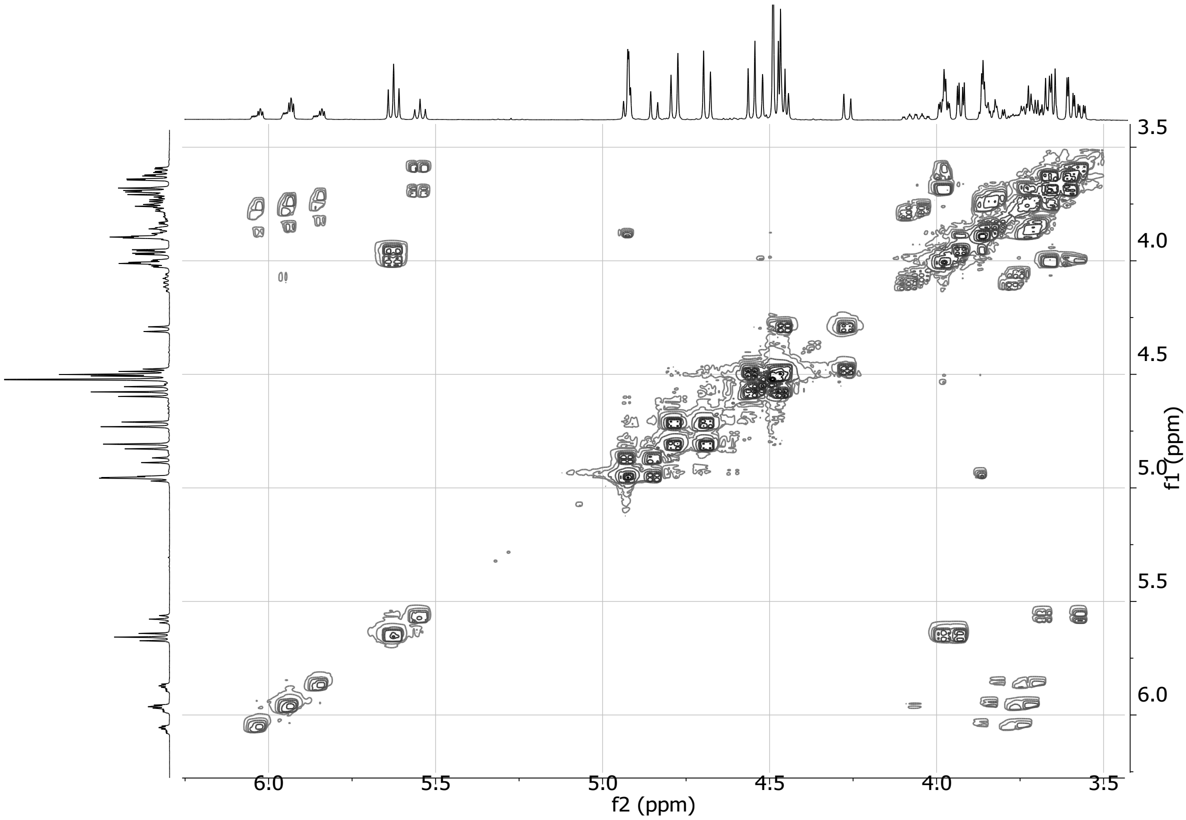

Supplementary Figure 263. HH-COSY NMR, $CDCl_3$ of compound **S69**

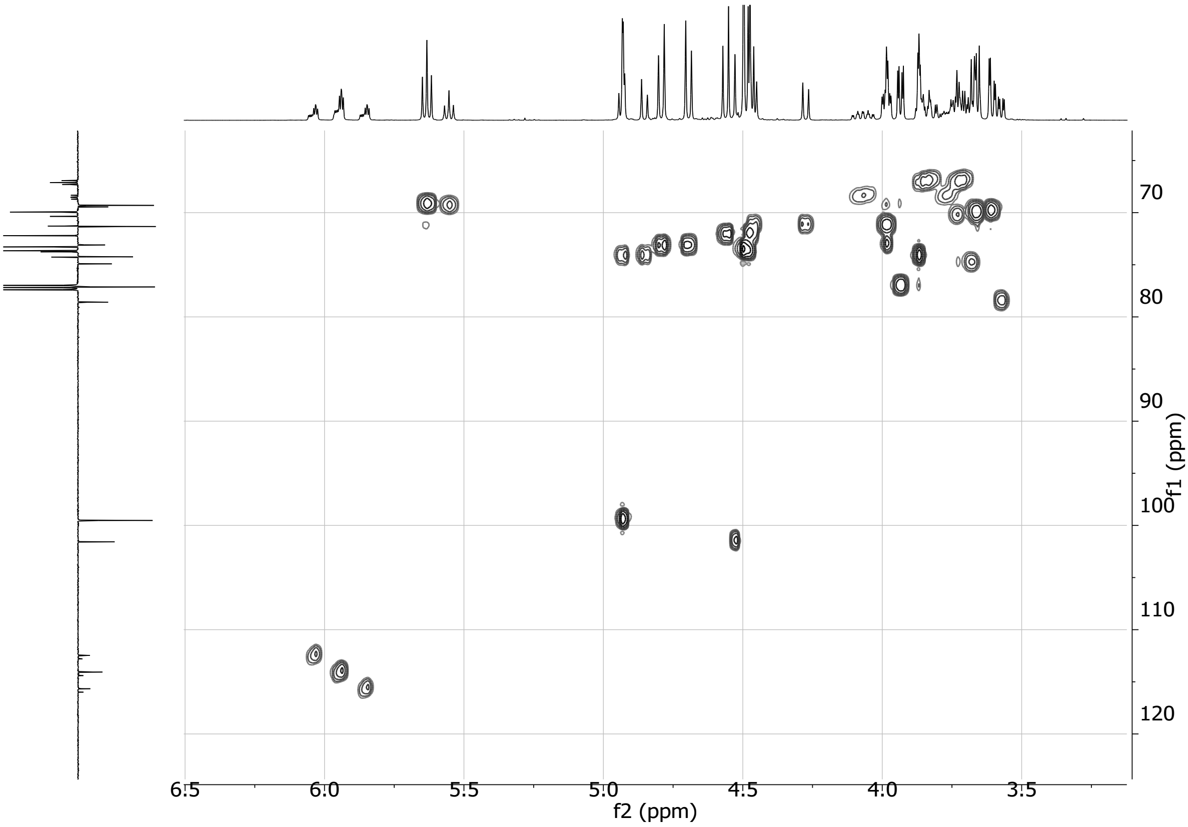

Supplementary Figure 264. HSQC NMR, $CDCl_3$ of compound **S69**

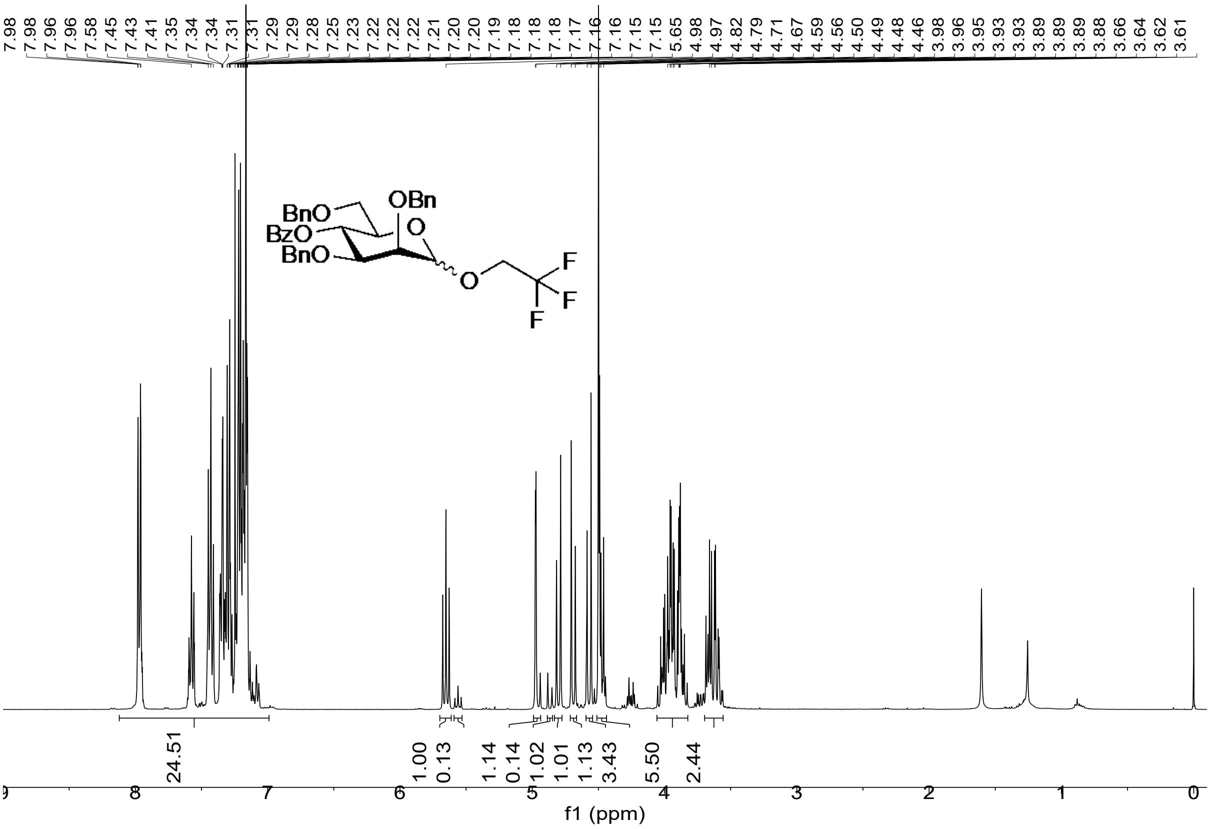

Supplementary Figure 265. ^{1}H NMR, 400 MHz, $CDCl_3$ of compound **S70**

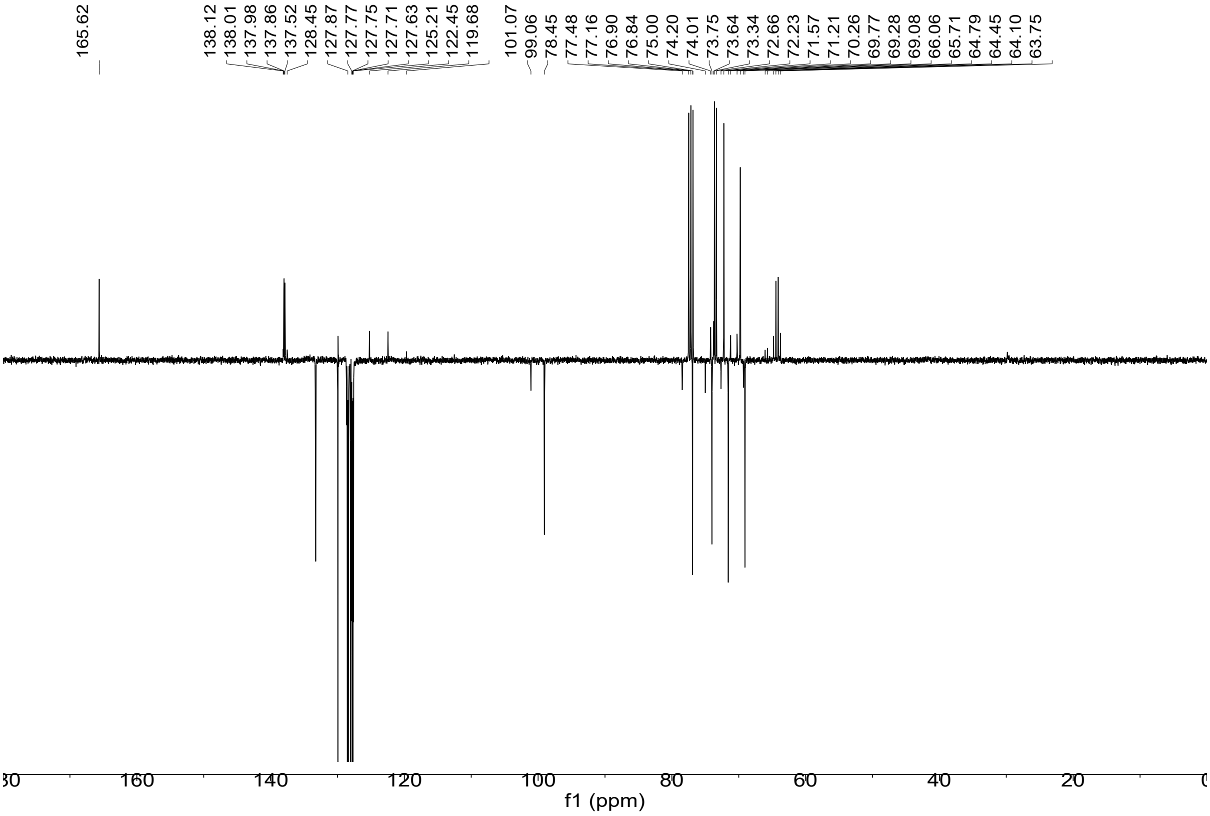

Supplementary Figure 266. ^{13}C NMR, 101 MHz, $CDCl_3$ of compound **S70**

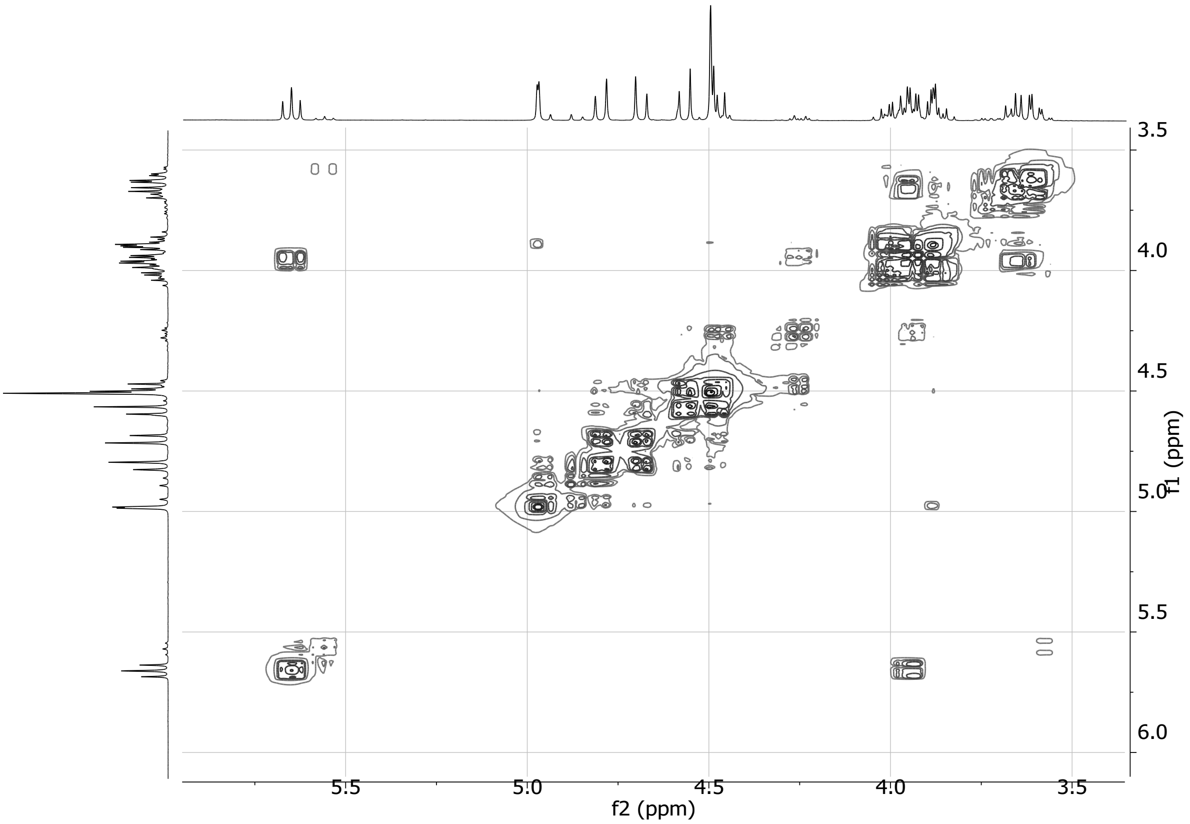

Supplementary Figure 267. HH-COSY NMR, $CDCl_3$ of compound **S70**

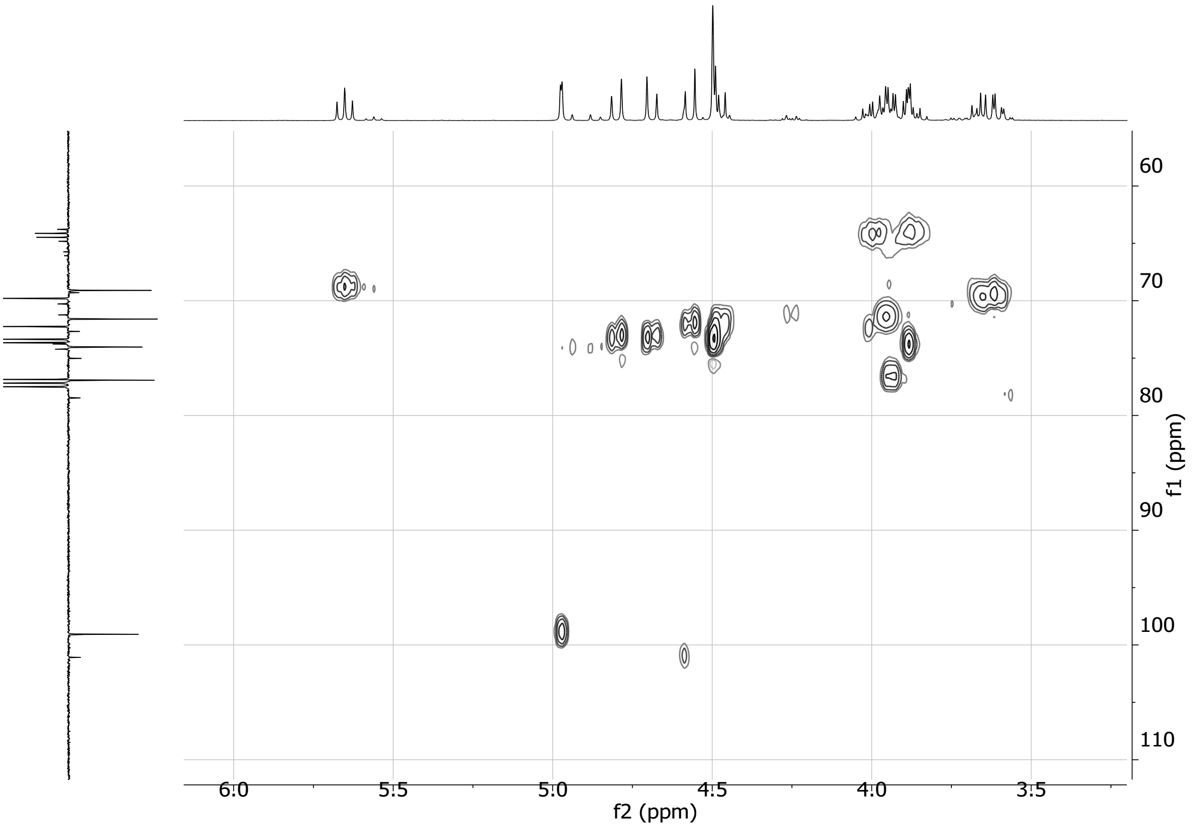

Supplementary Figure 268. HSQC NMR, $CDCl_3$ of compound **S70**

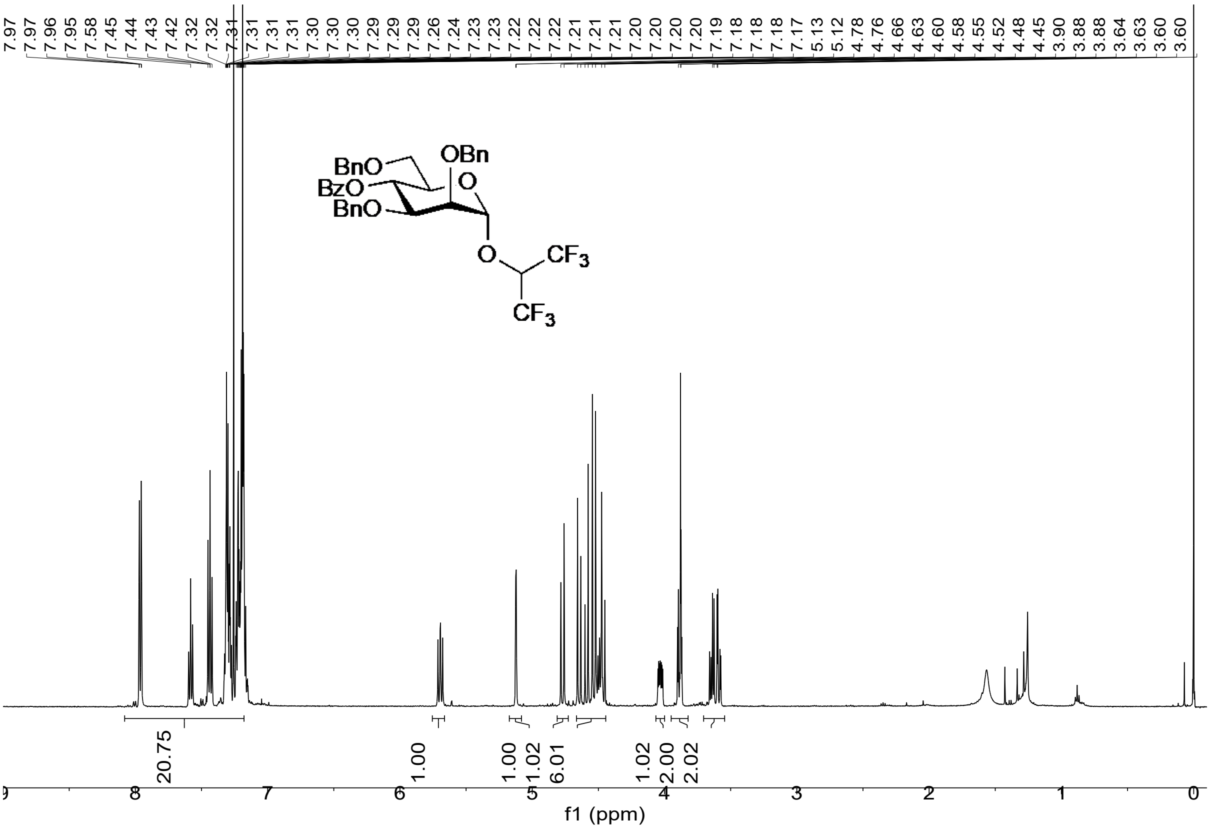

Supplementary Figure 269. ^{1}H NMR, 500 MHz, $CDCl_3$ of compound **S71**

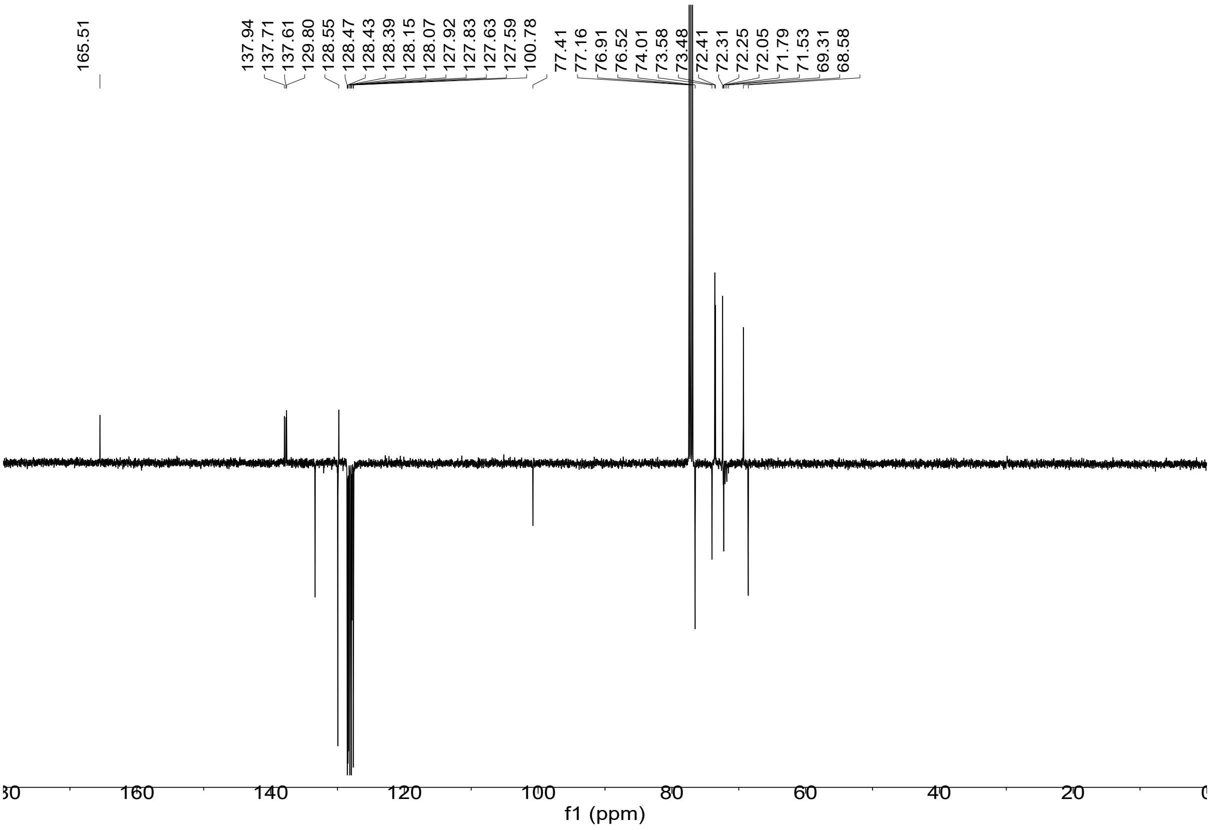

Supplementary Figure 270. ^{13}C NMR, 126 MHz, $CDCl_3$ of compound **S71**

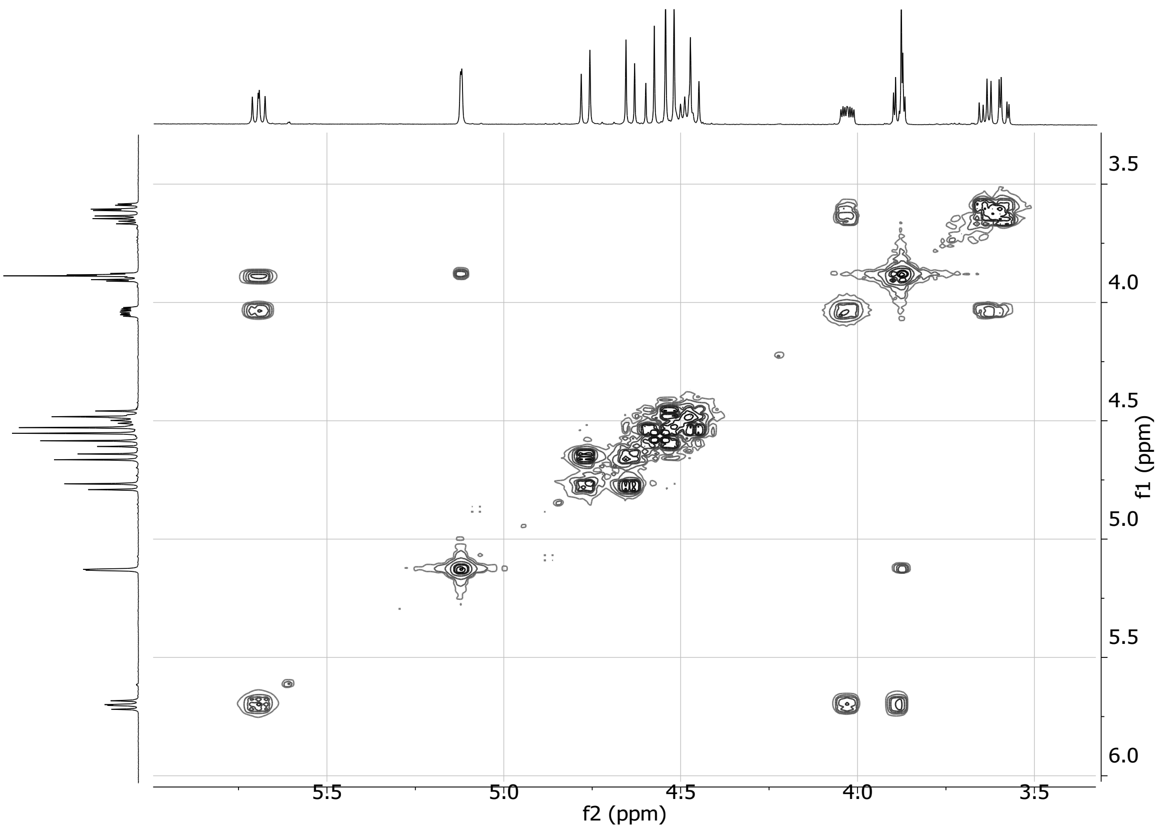

Supplementary Figure 271. HH-COSY NMR, $CDCl_3$ of compound **S71**

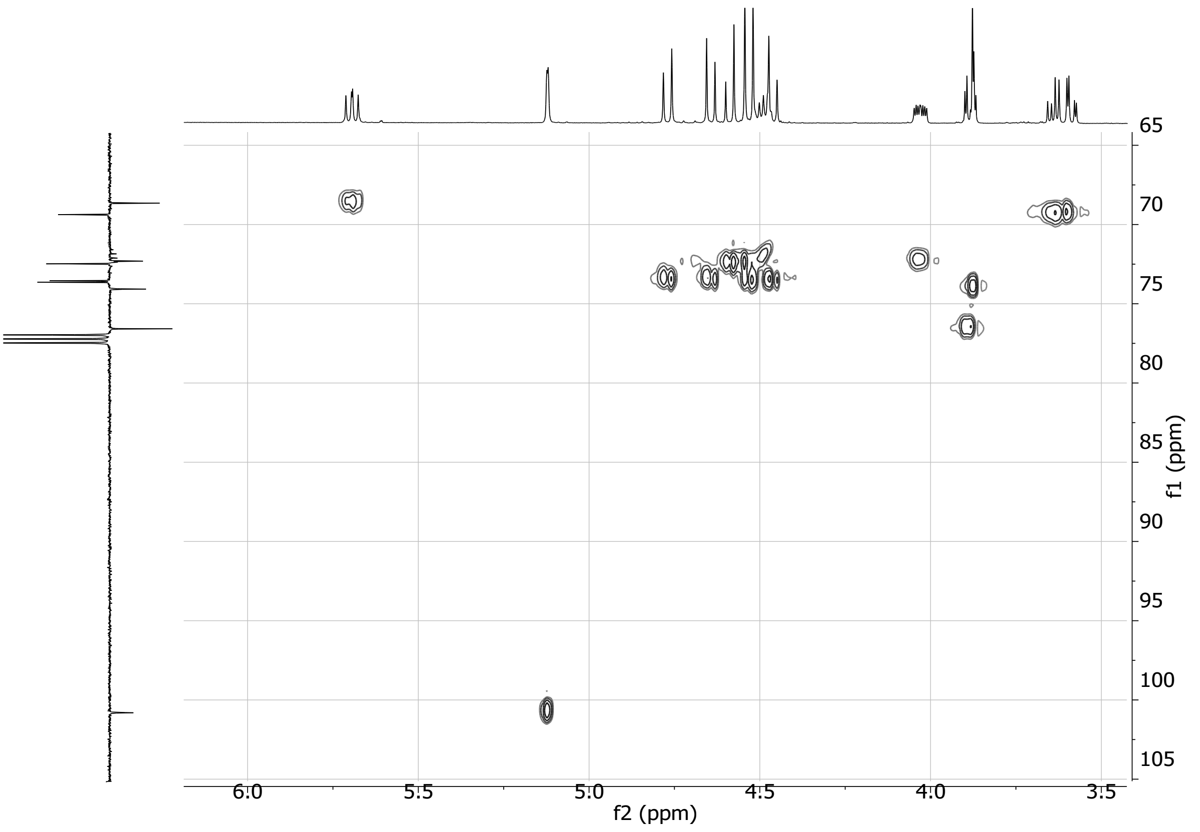

Supplementary Figure 272. HSQC NMR, $CDCl_3$ of compound **S71**

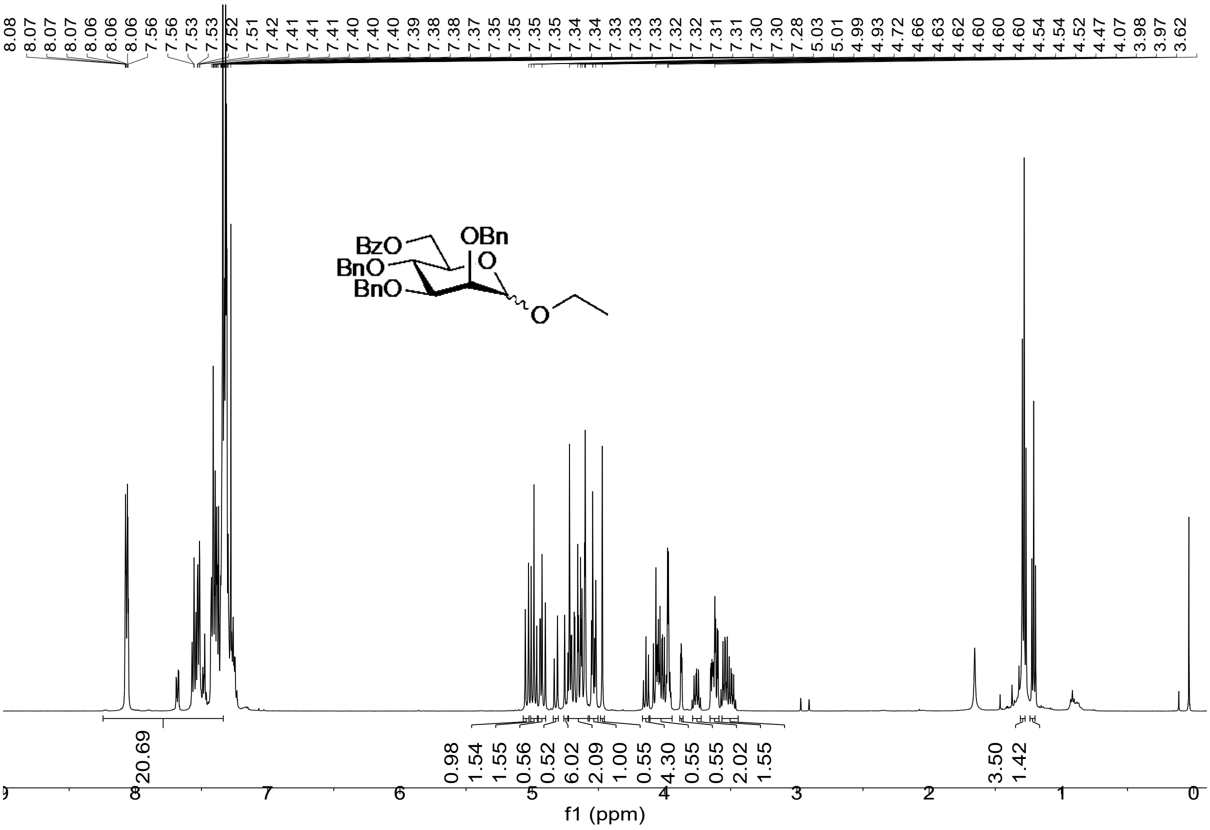

Supplementary Figure 273. ^{1}H NMR, 500 MHz, $CDCl_3$ of compound **S72**

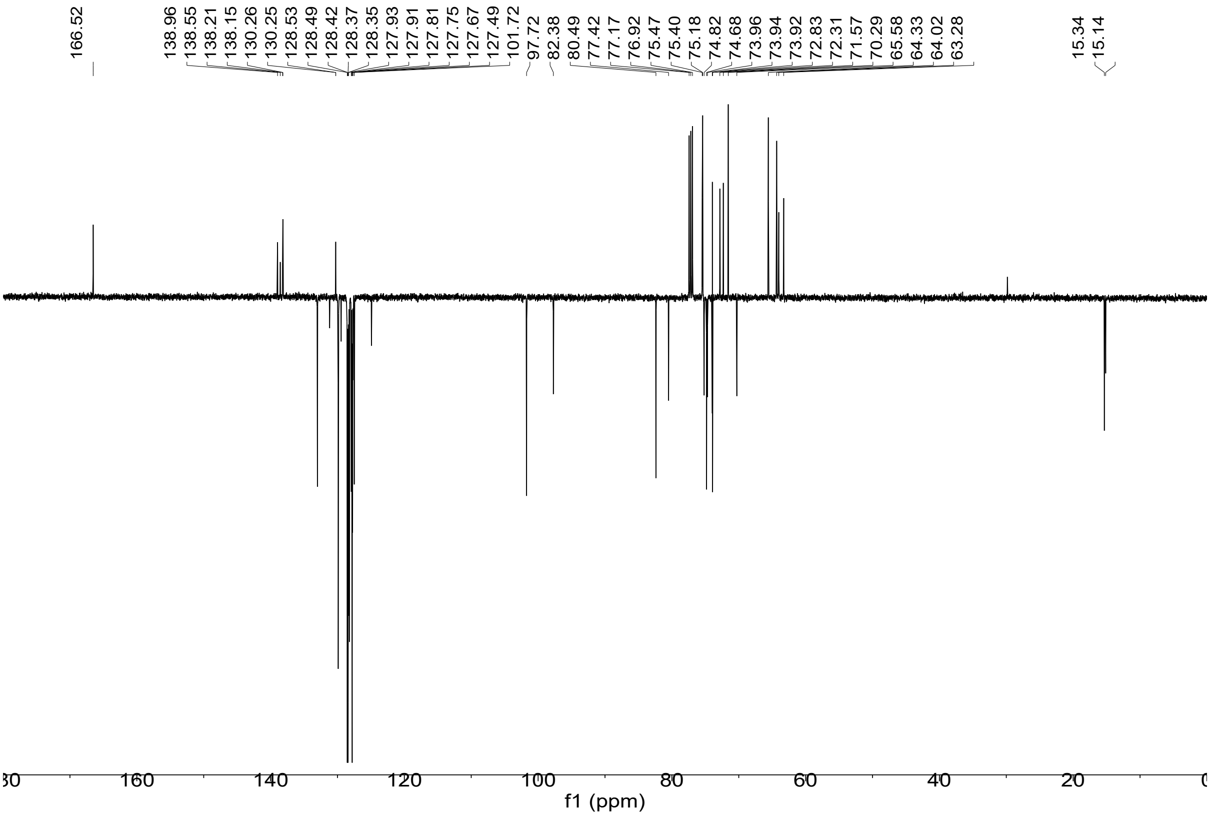

Supplementary Figure 274. ^{13}C NMR, 126 MHz, $CDCl_3$ of compound **S72**

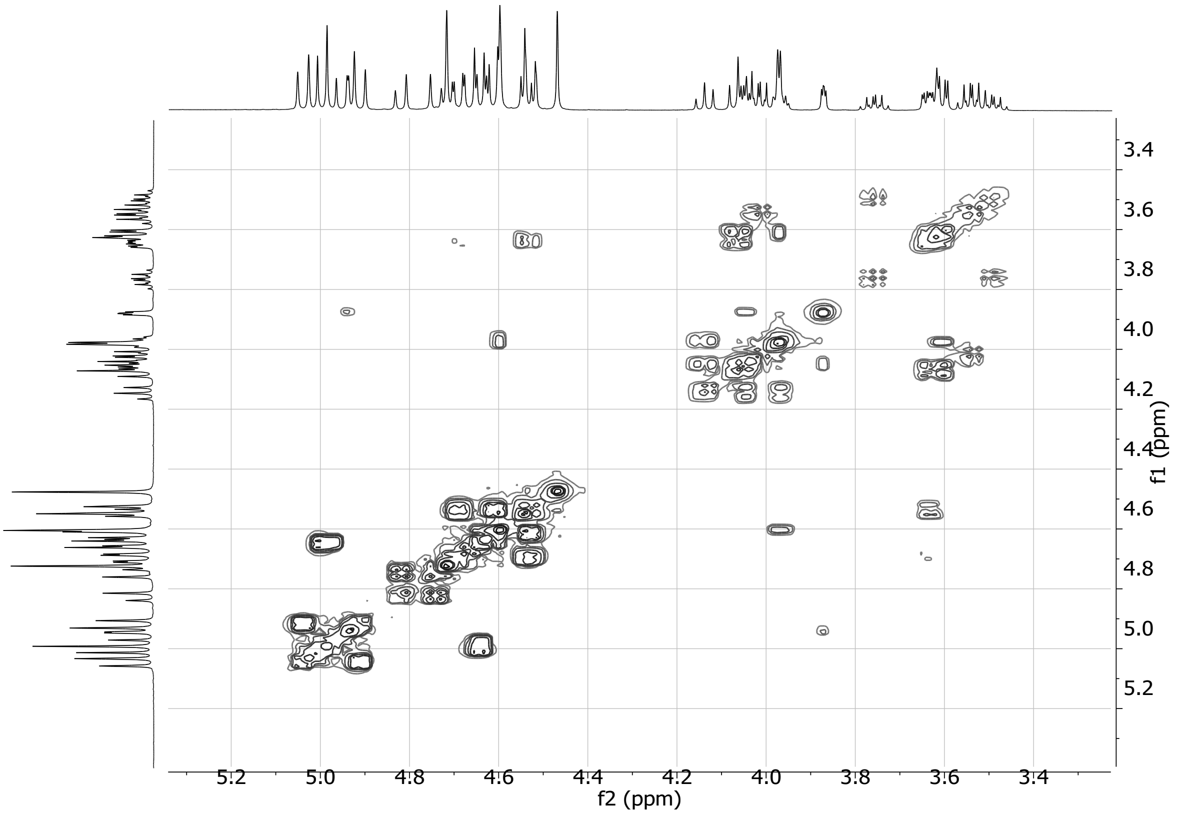

Supplementary Figure 275. HH-COSY NMR, $CDCl_3$ of compound **S72**

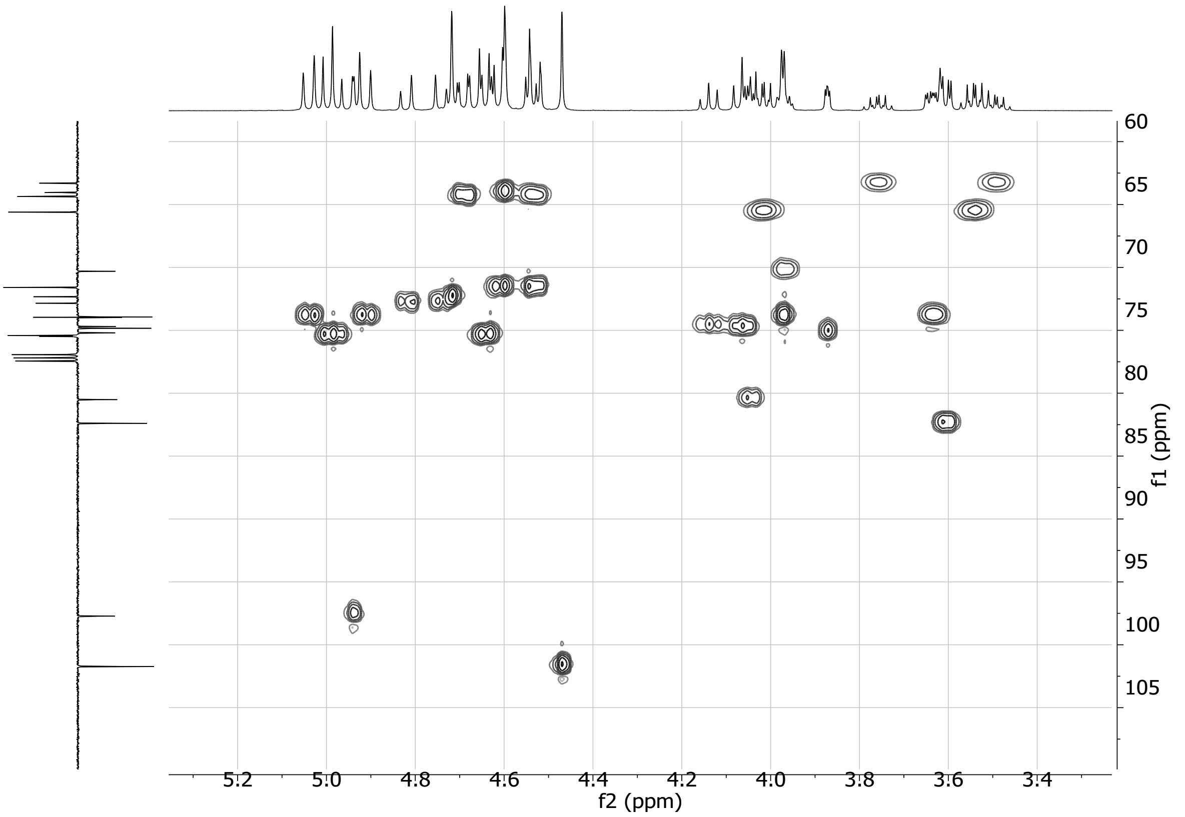

Supplementary Figure 276. HSQC NMR, $CDCl_3$ of compound **S72**

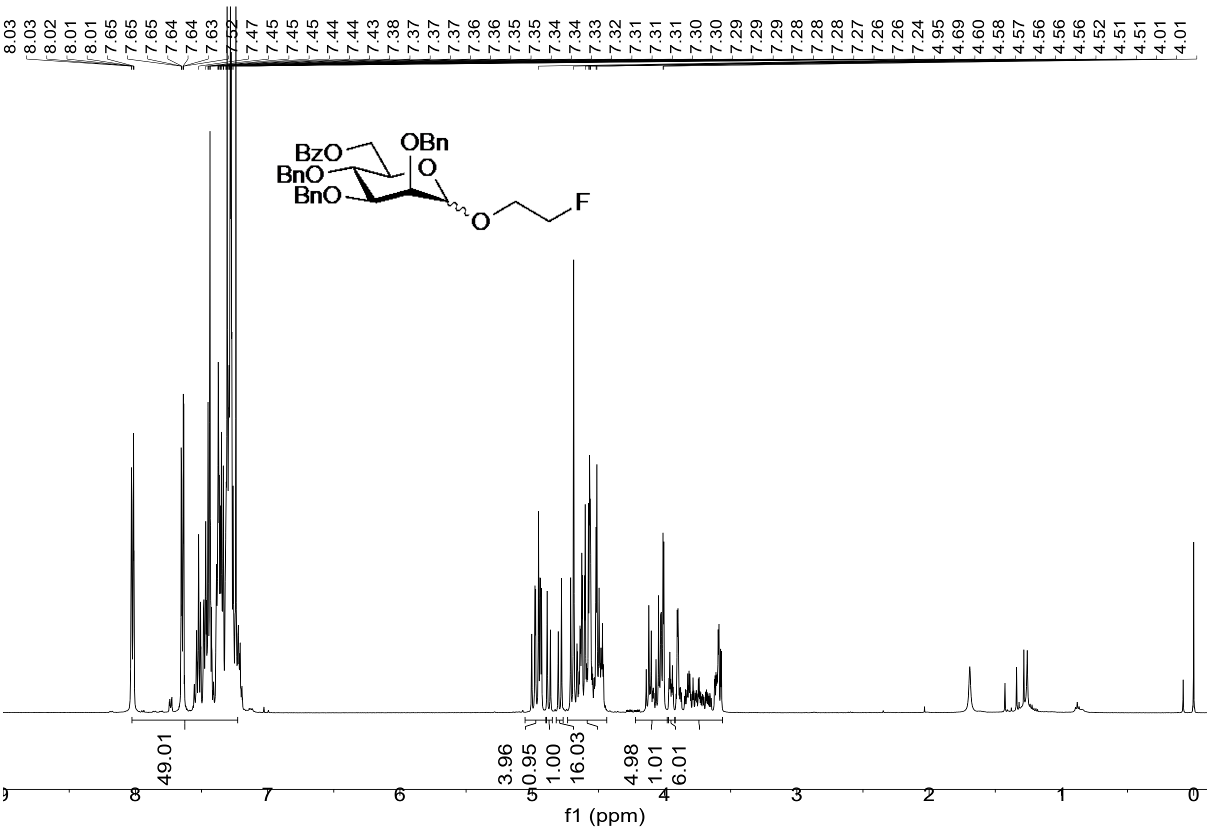

Supplementary Figure 277. ^{1}H NMR, 500 MHz, $CDCl_3$ of compound **S73**

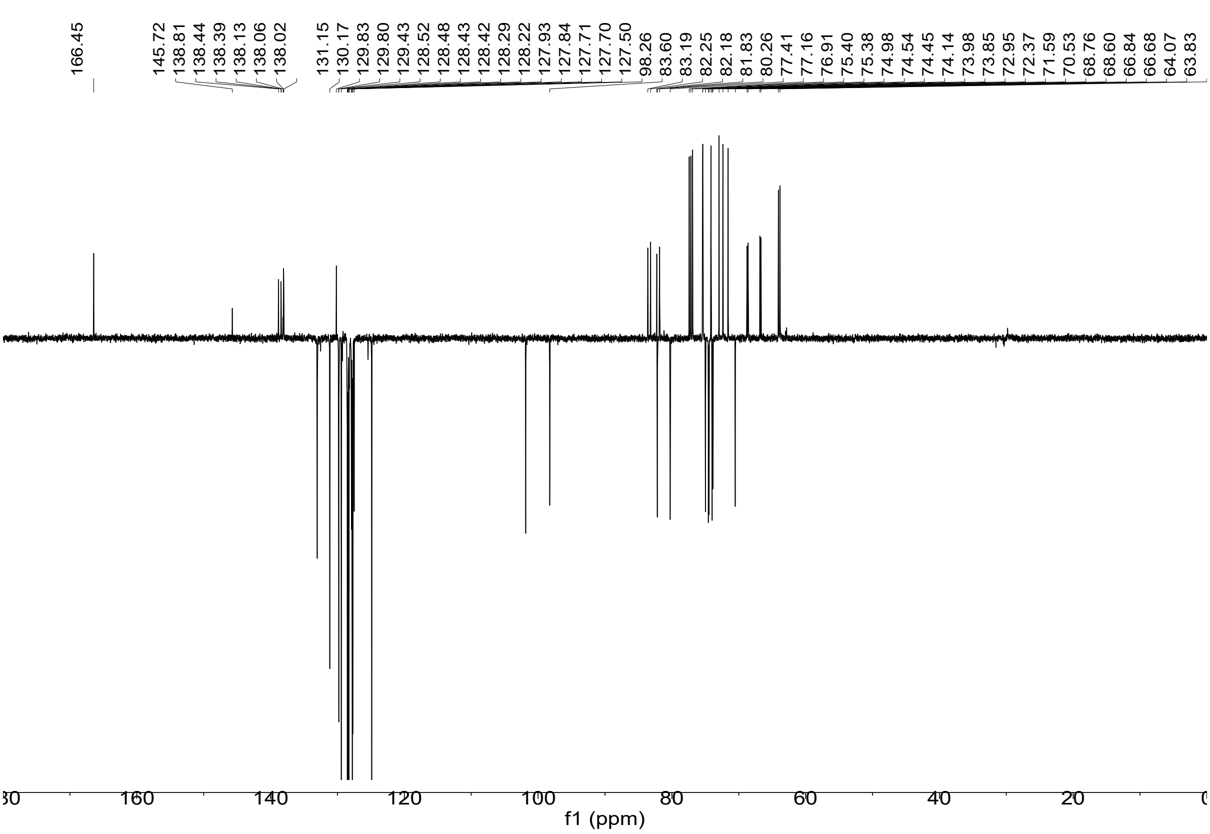

Supplementary Figure 278. ^{13}C NMR, 126 MHz, $CDCl_3$ of compound **S73**

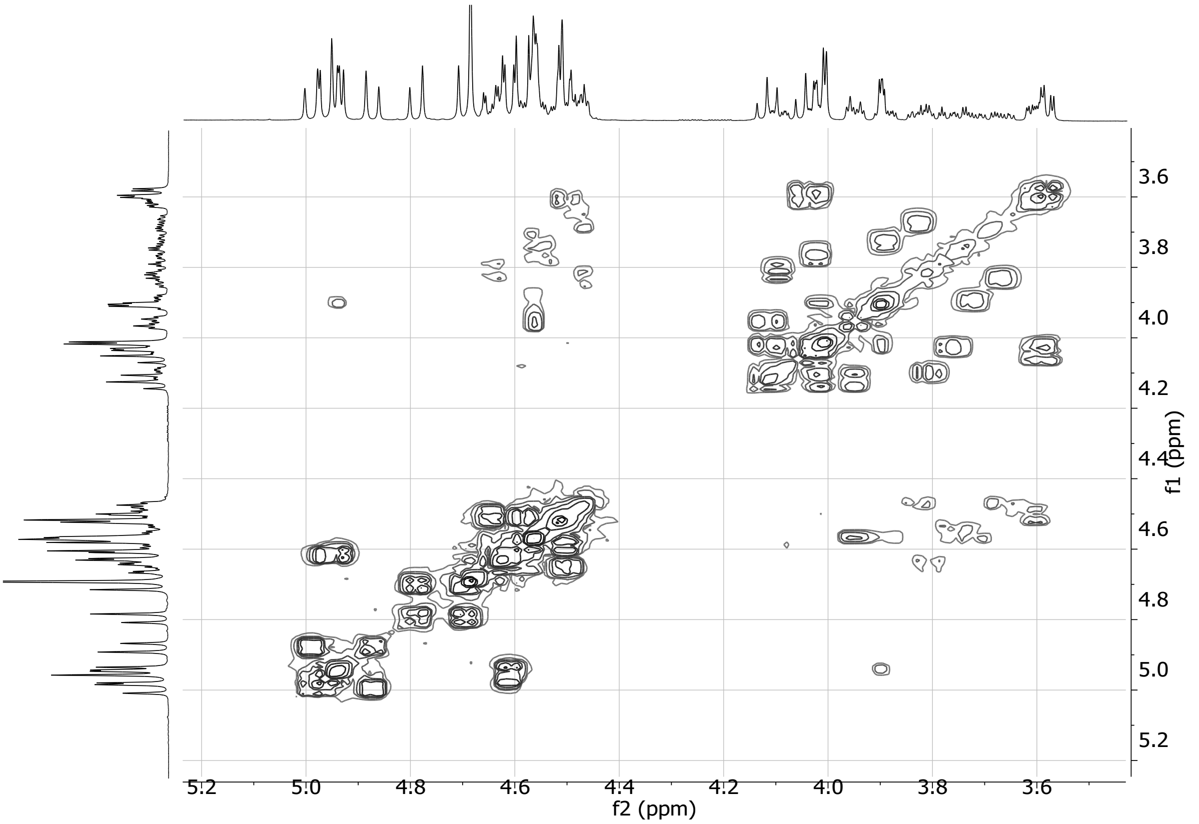

Supplementary Figure 279. HH-COSY NMR, $CDCl_3$ of compound **S73**

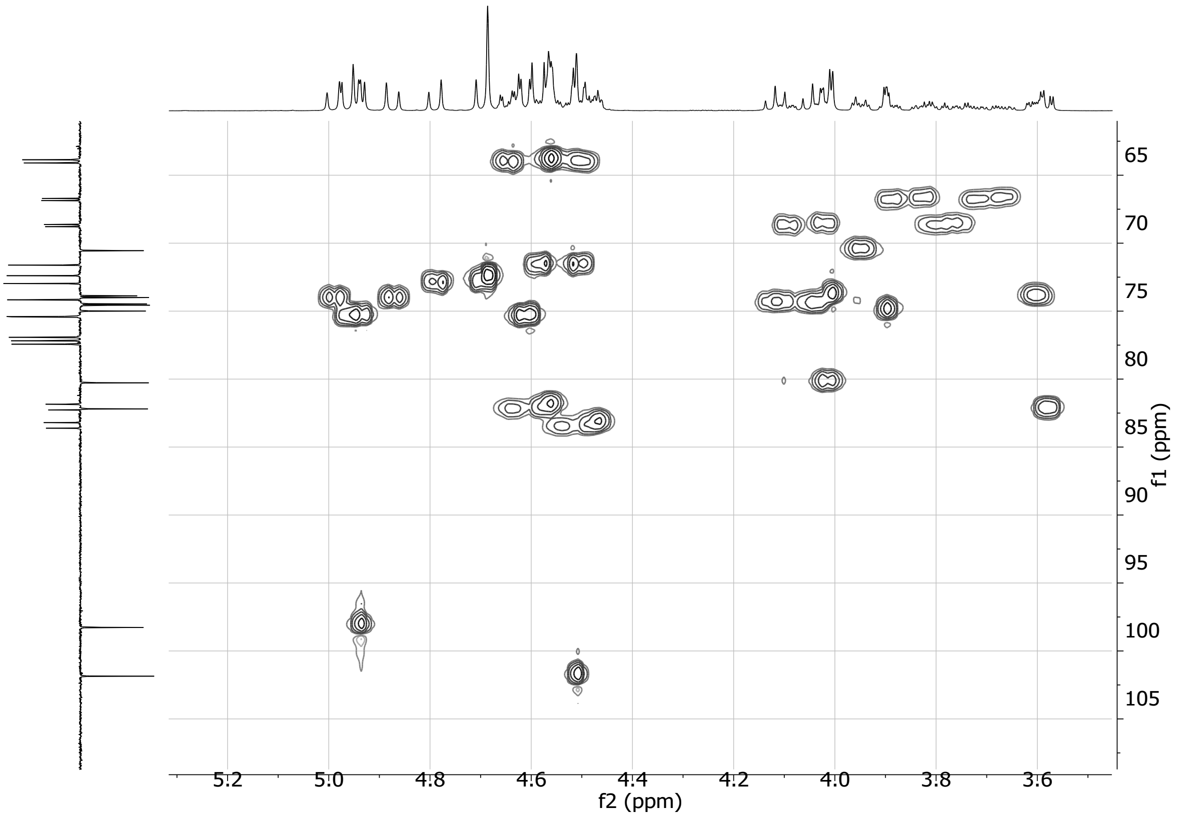

Supplementary Figure 280. HSQC NMR, $CDCl_3$ of compound **S73**

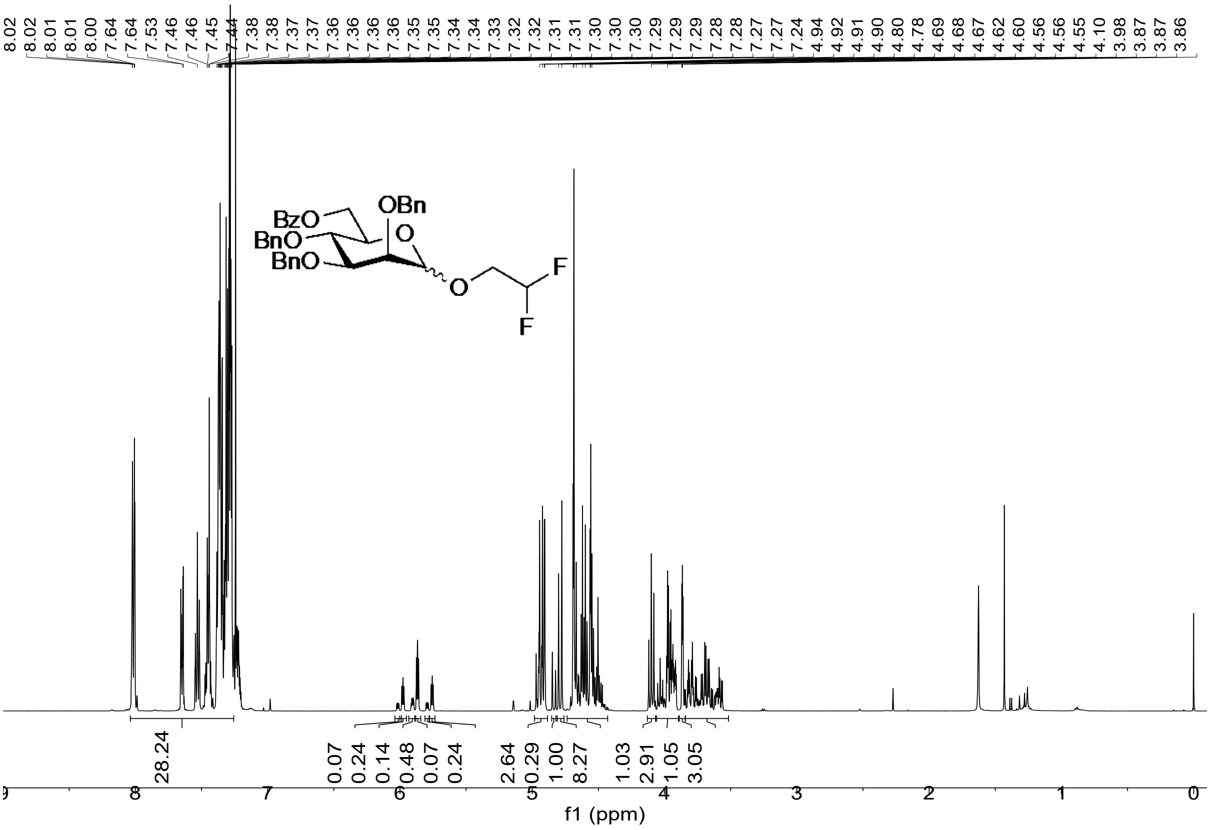

Supplementary Figure 281. ^{1}H NMR, 500 MHz, $CDCl_3$ of compound **S74**

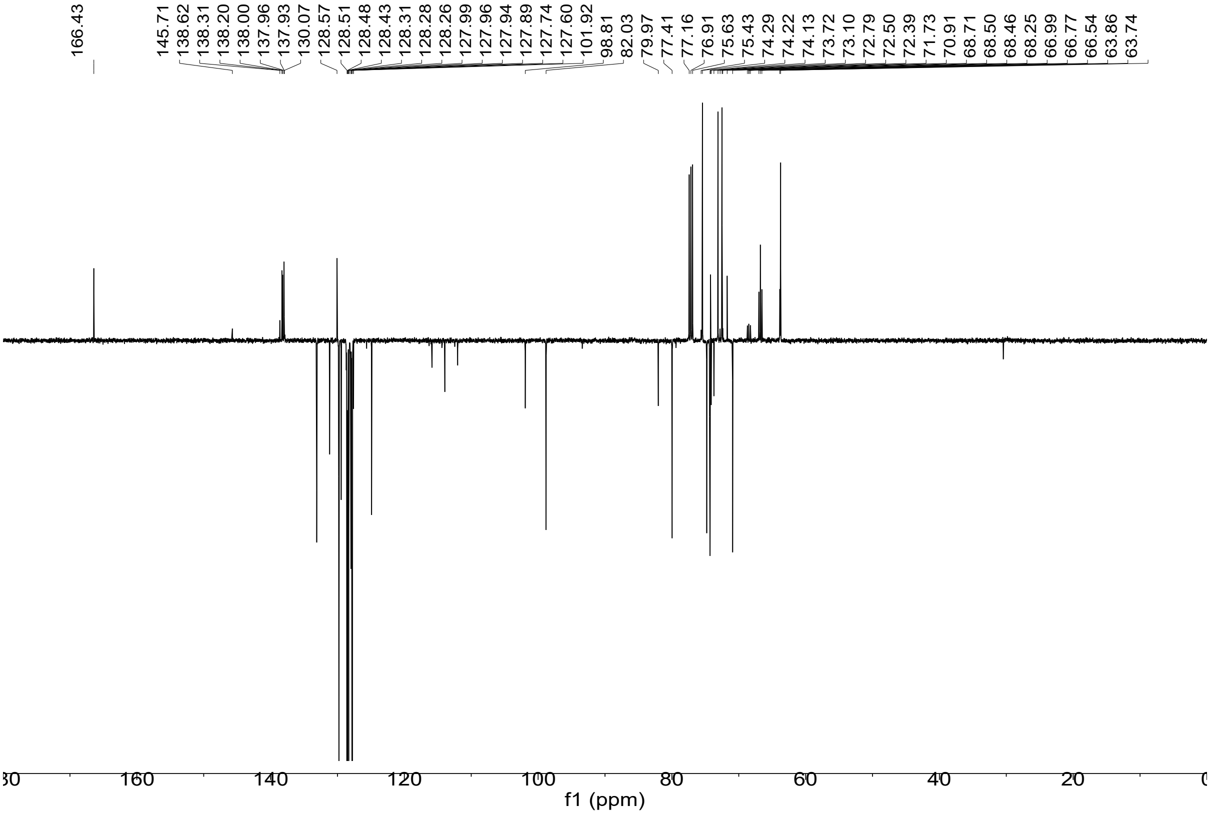

Supplementary Figure 282. ^{13}C NMR, 126 MHz, $CDCl_3$ of compound **S74**

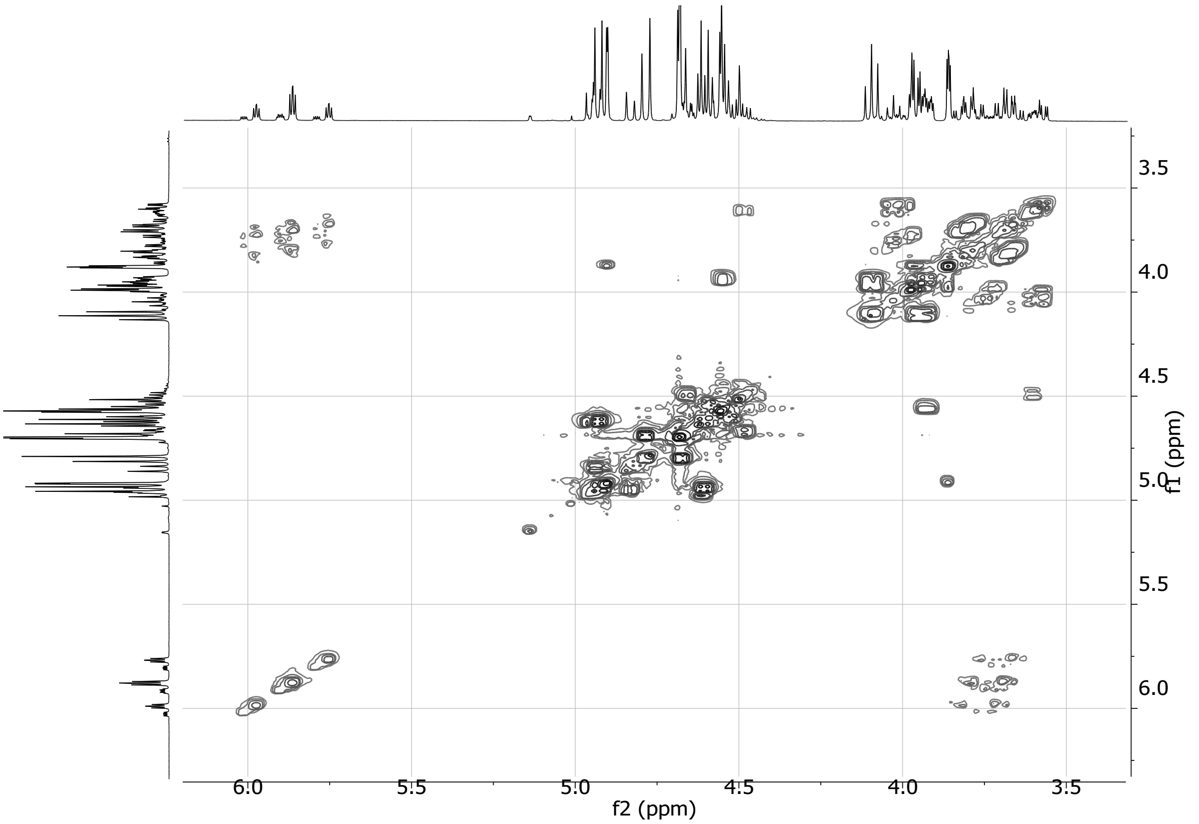

Supplementary Figure 283. HH-COSY NMR, $CDCl_3$ of compound **S74**

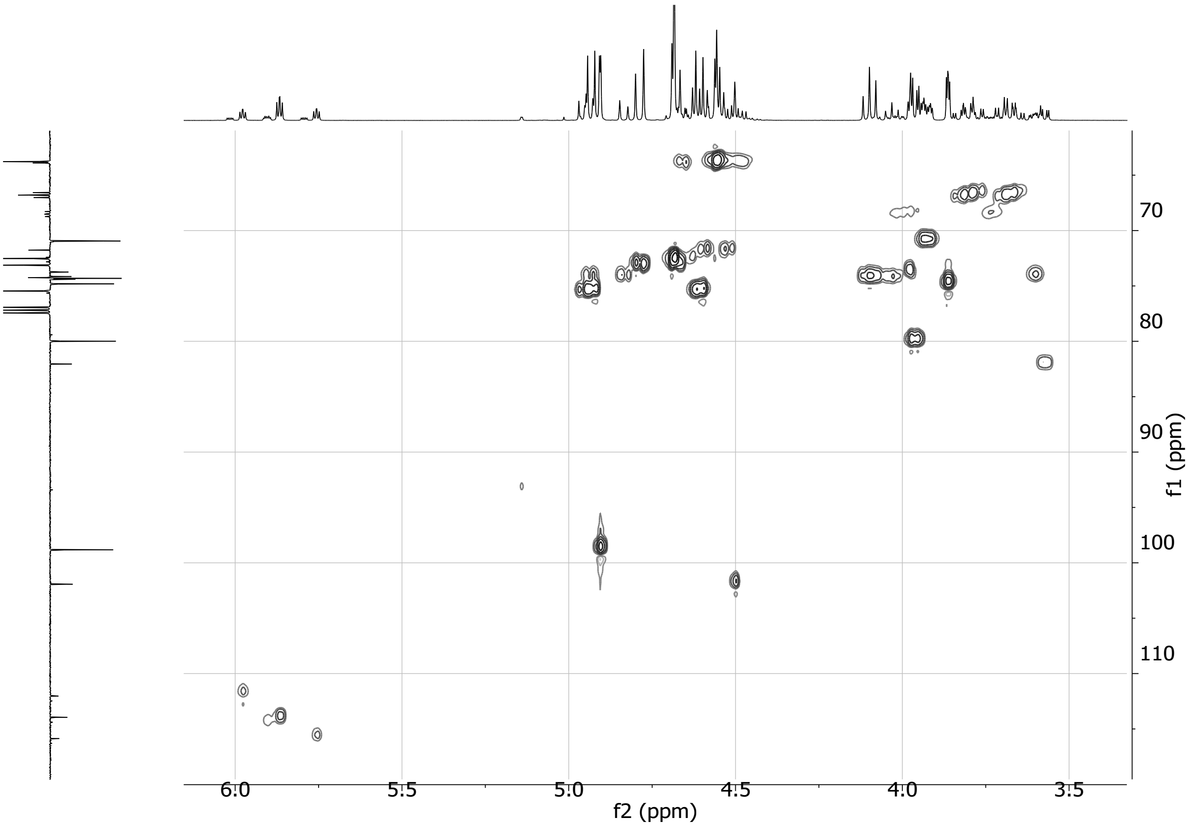

Supplementary Figure 284. HSQC NMR, $CDCl_3$ of compound **S74**

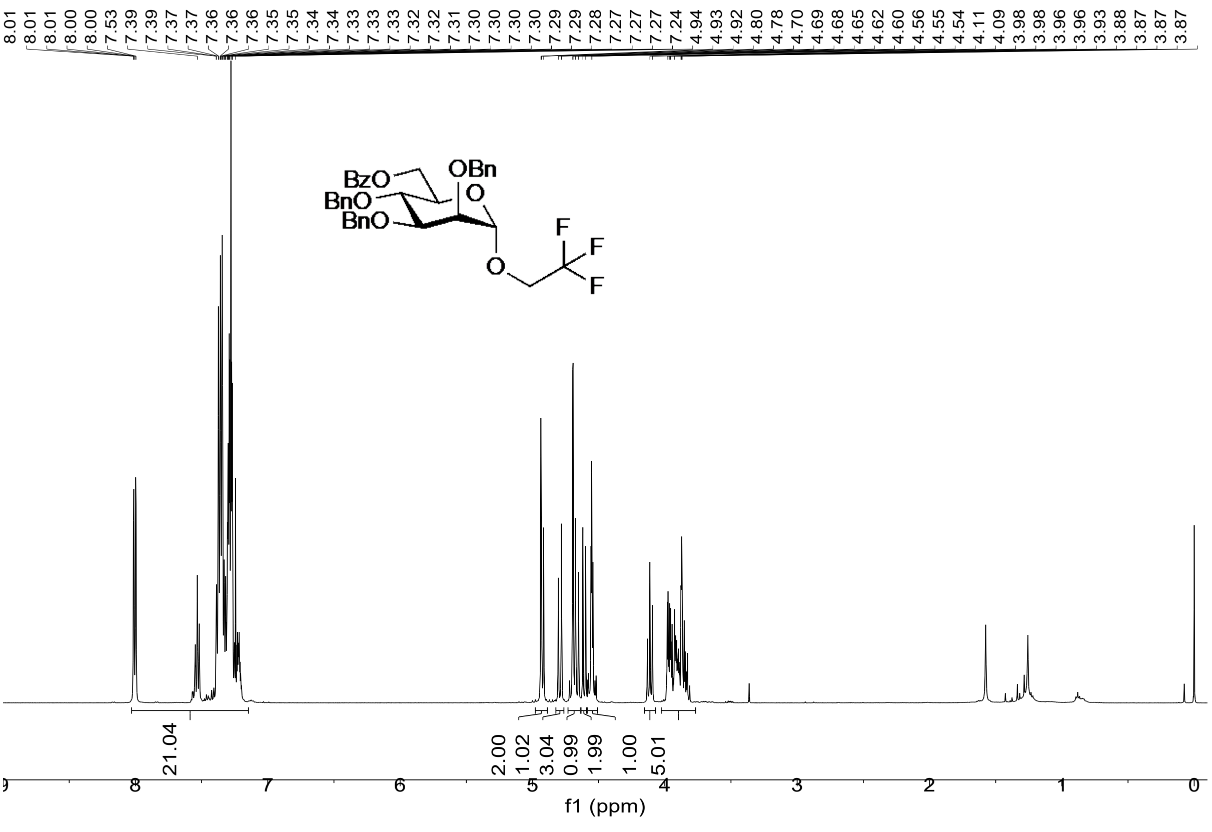

Supplementary Figure 285. ^{1}H NMR, 500 MHz, $CDCl_3$ of compound **S75**

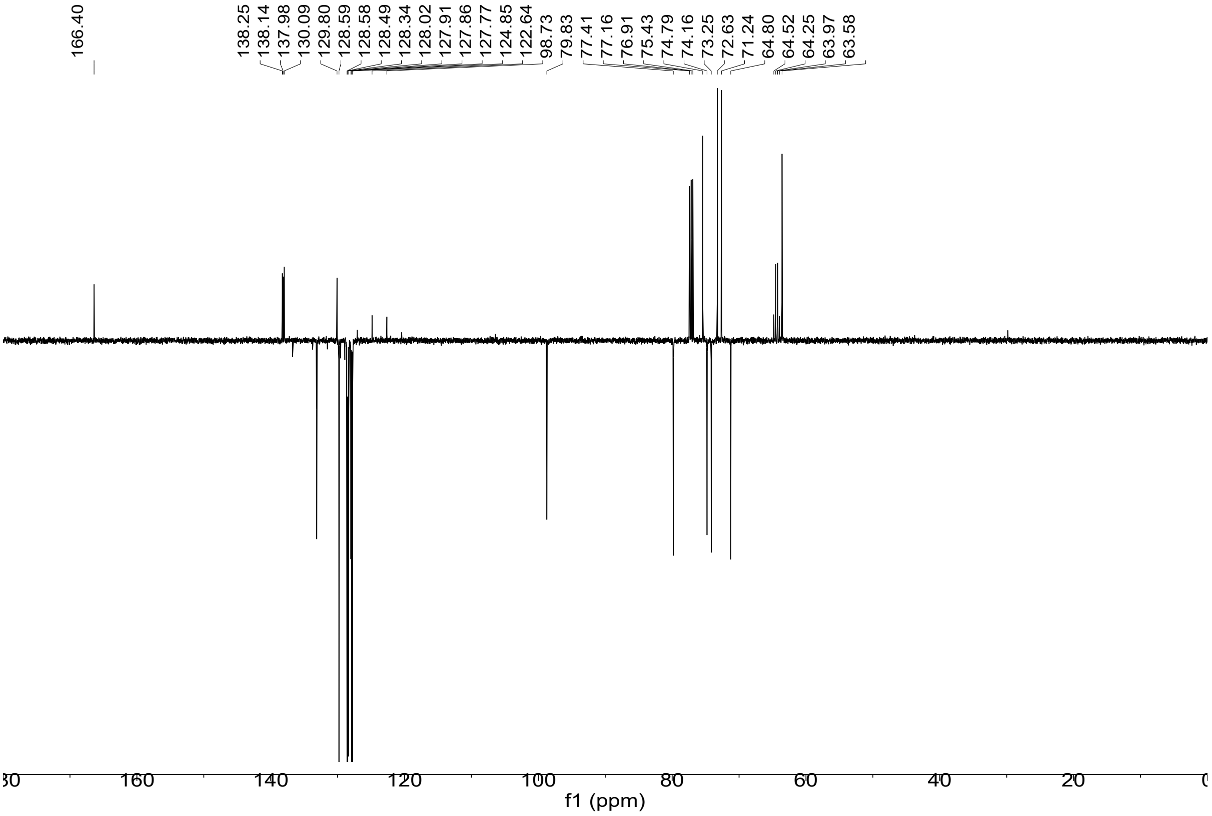

Supplementary Figure 286. ^{13}C NMR, 126 MHz, $CDCl_3$ of compound **S75**

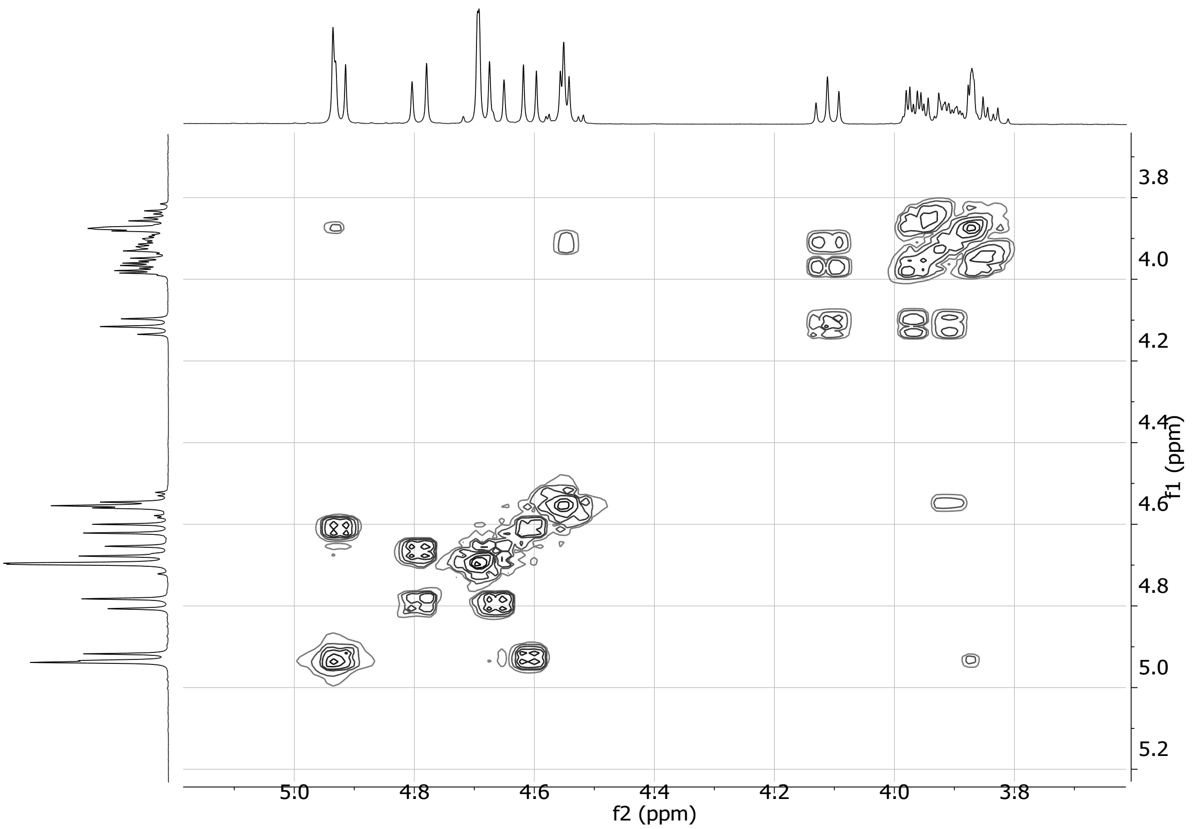

Supplementary Figure 287. HH-COSY NMR, $CDCl_3$ of compound **S75**

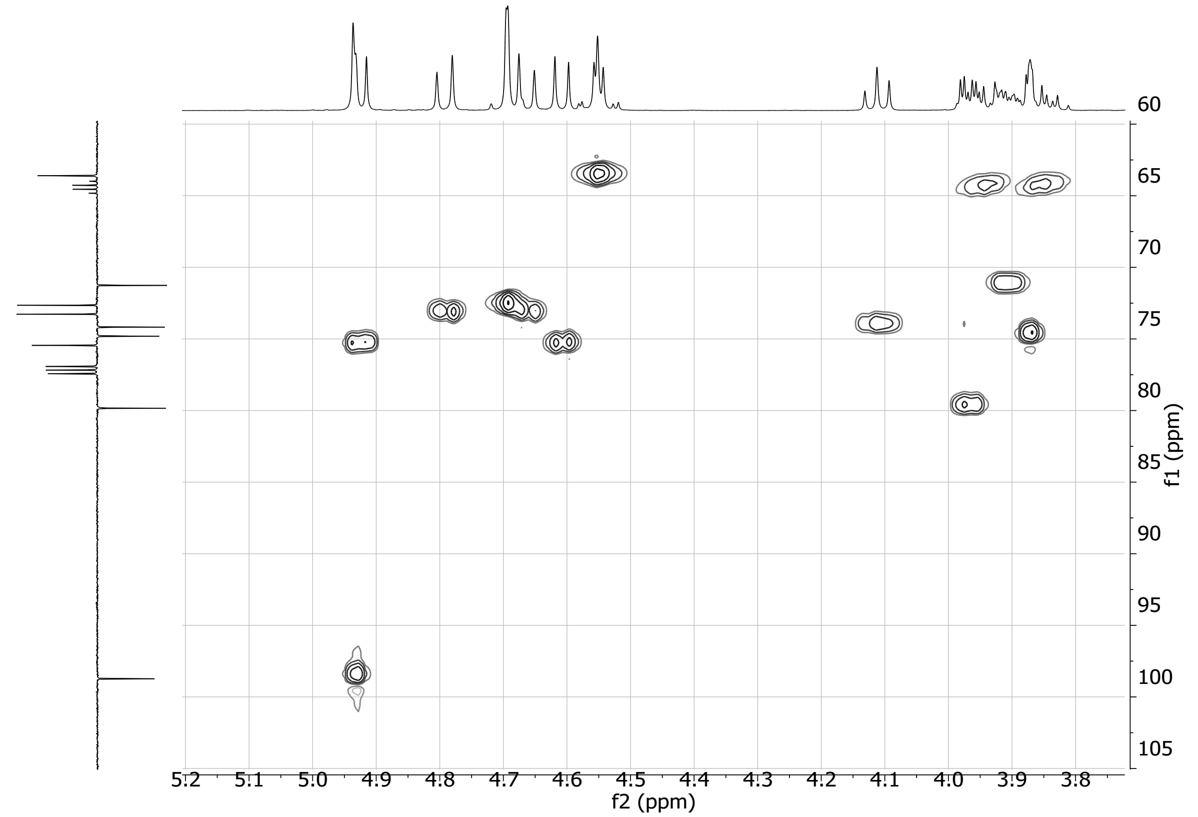

Supplementary Figure 288. HSQC NMR, $CDCl_3$ of compound **S75**

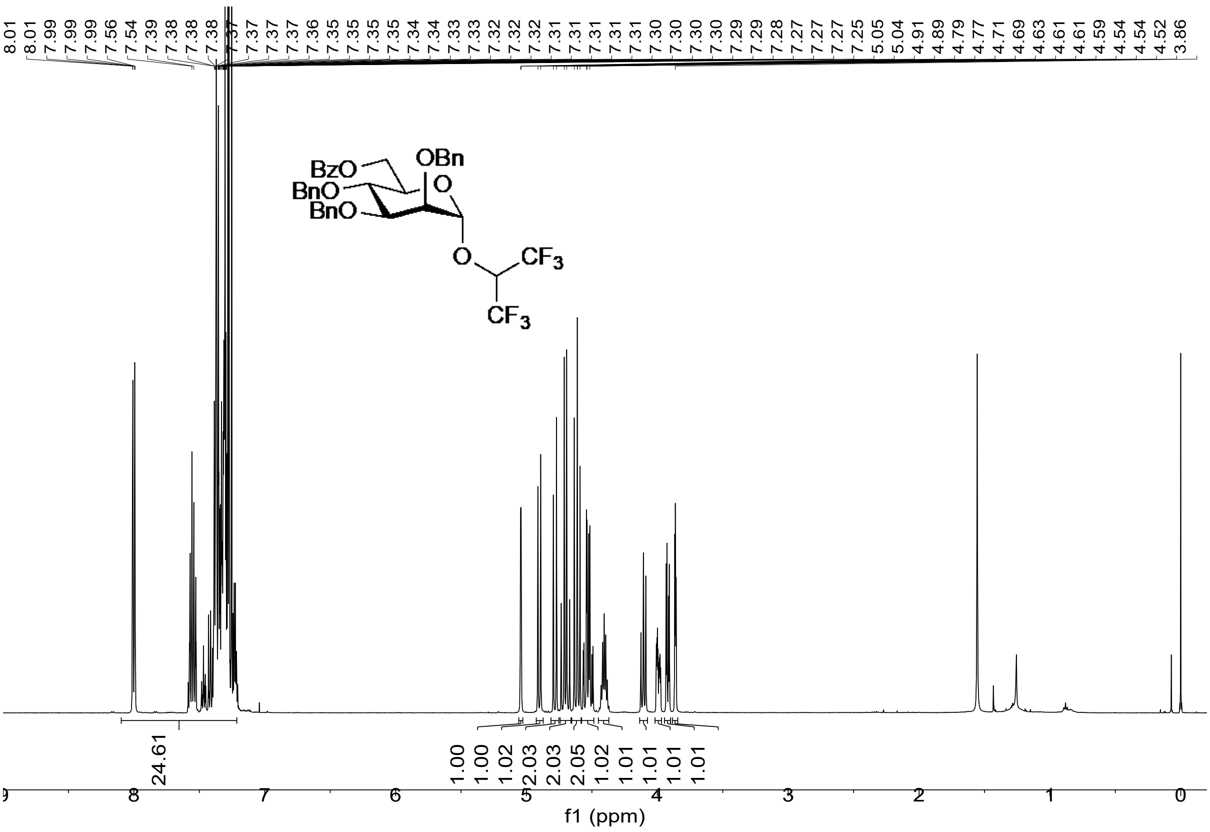

Supplementary Figure 289. ^{1}H NMR, 500 MHz, $CDCl_3$ of compound **S76**

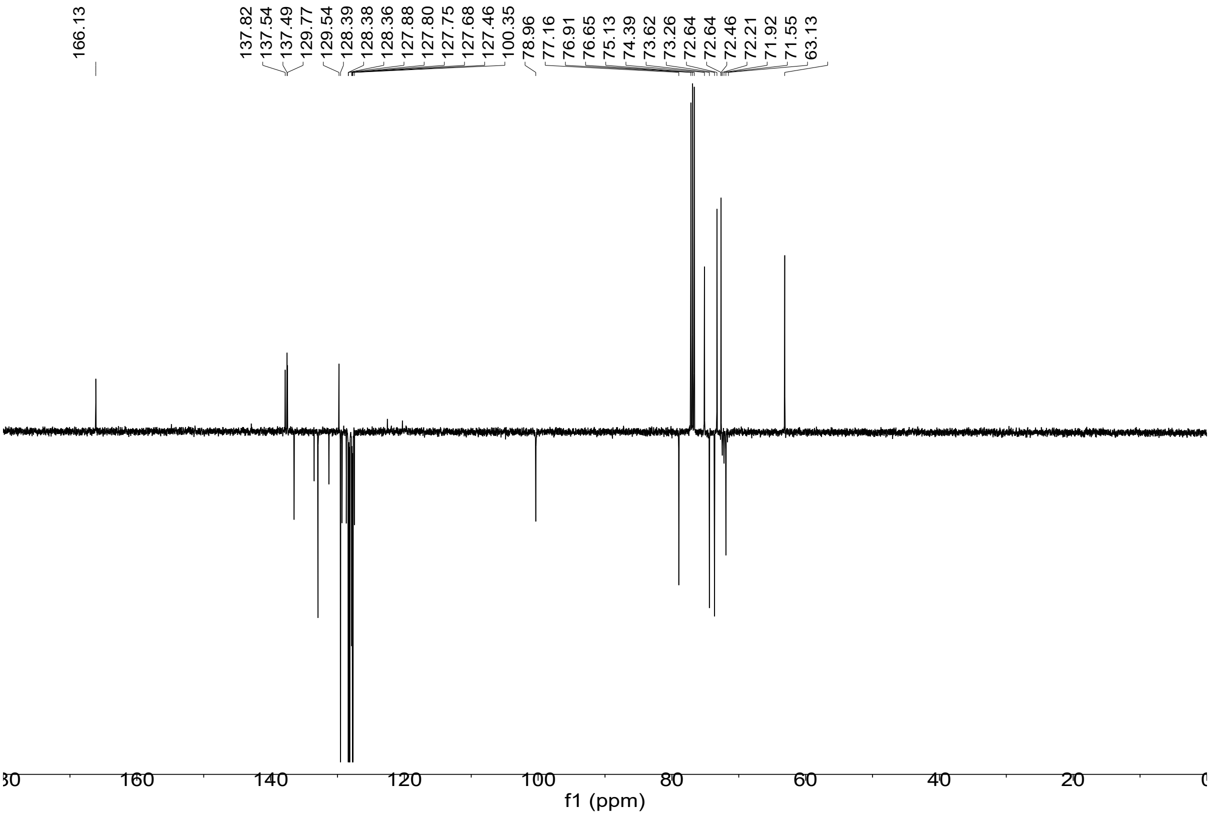

Supplementary Figure 290. ^{13}C NMR, 126 MHz, $CDCl_3$ of compound **S76**

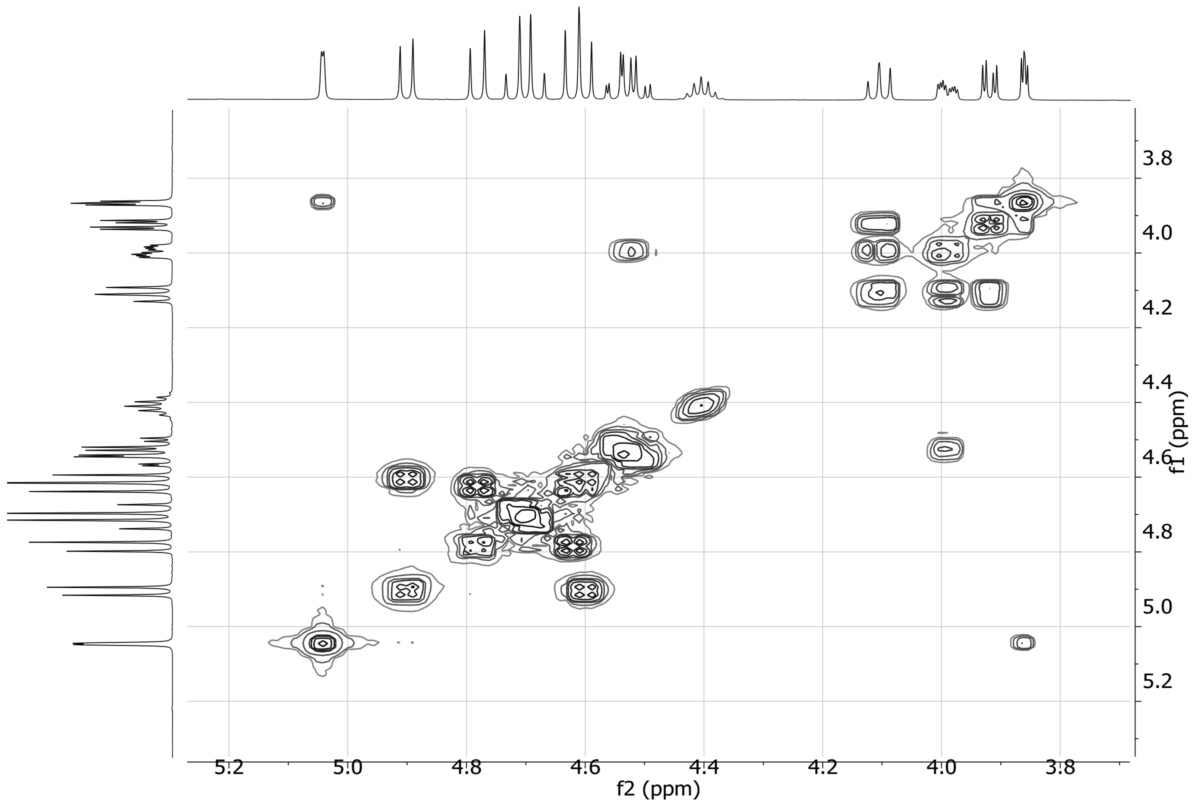

Supplementary Figure 291. HH-COSY NMR, $CDCl_3$ of compound **S76**

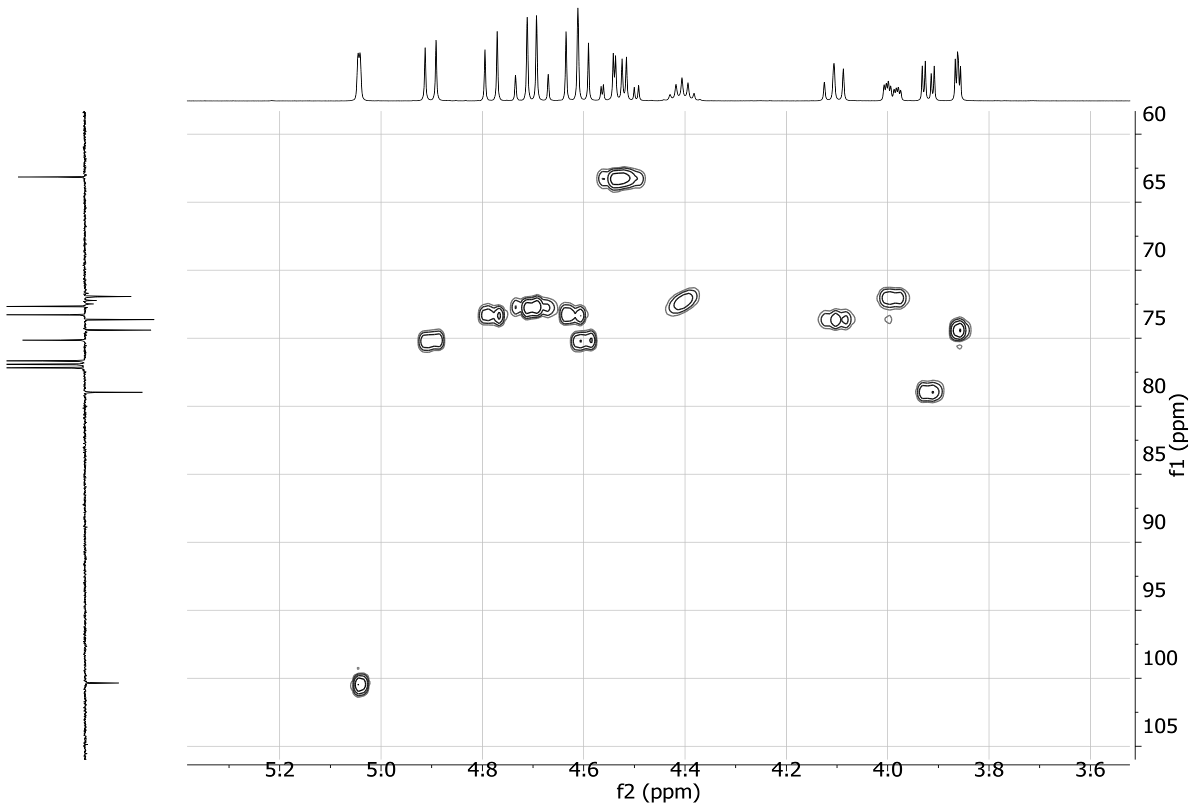

Supplementary Figure 292. HSQC NMR, $CDCl_3$ of compound **S76**

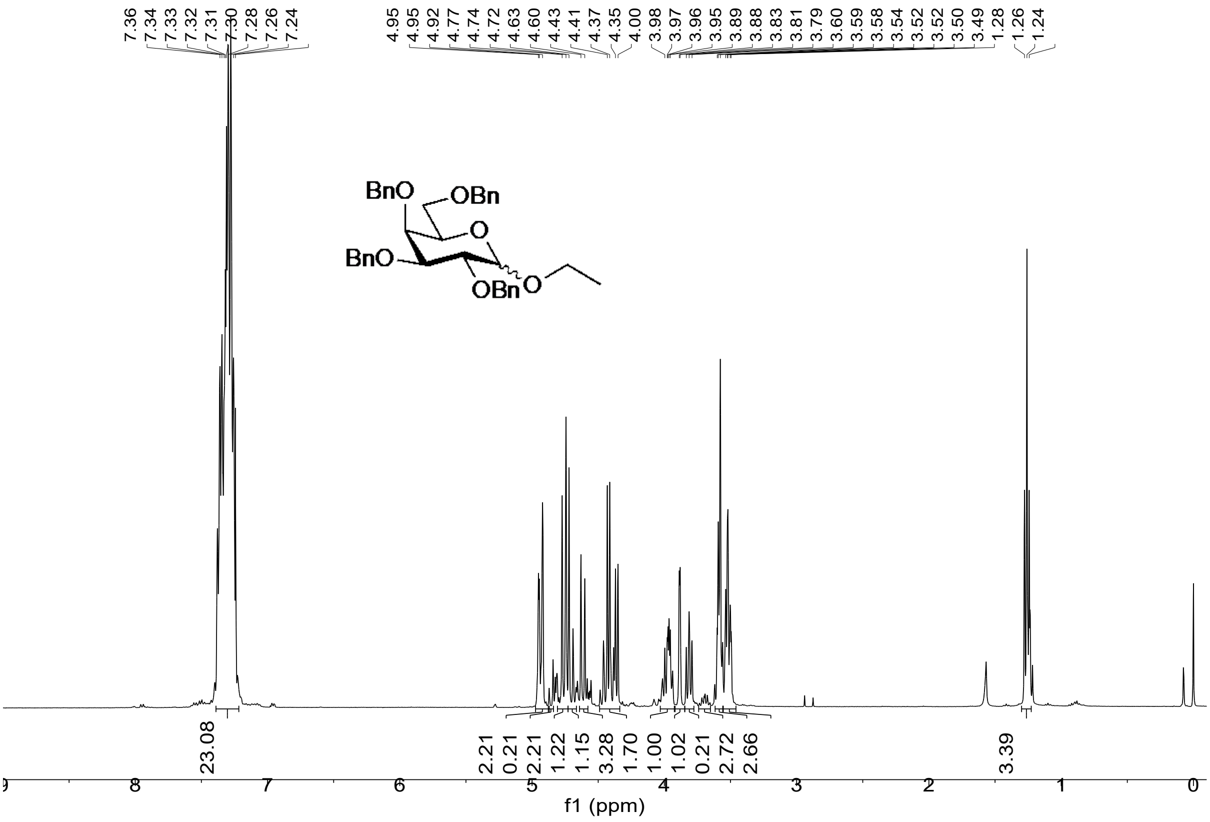

Supplementary Figure 293. ^{1}H NMR, 500 MHz, $CDCl_3$ of compound **S77**

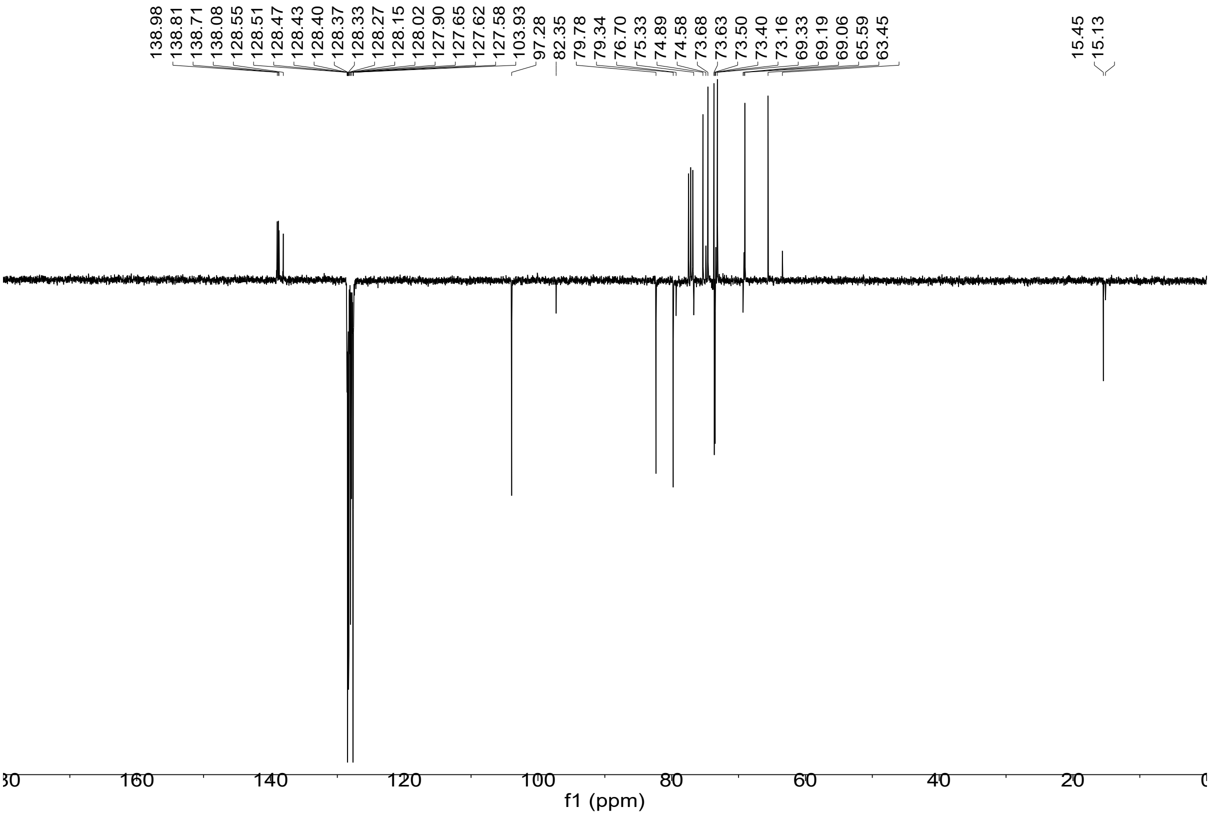

Supplementary Figure 294. ^{13}C NMR, 126 MHz, $CDCl_3$ of compound **S77**

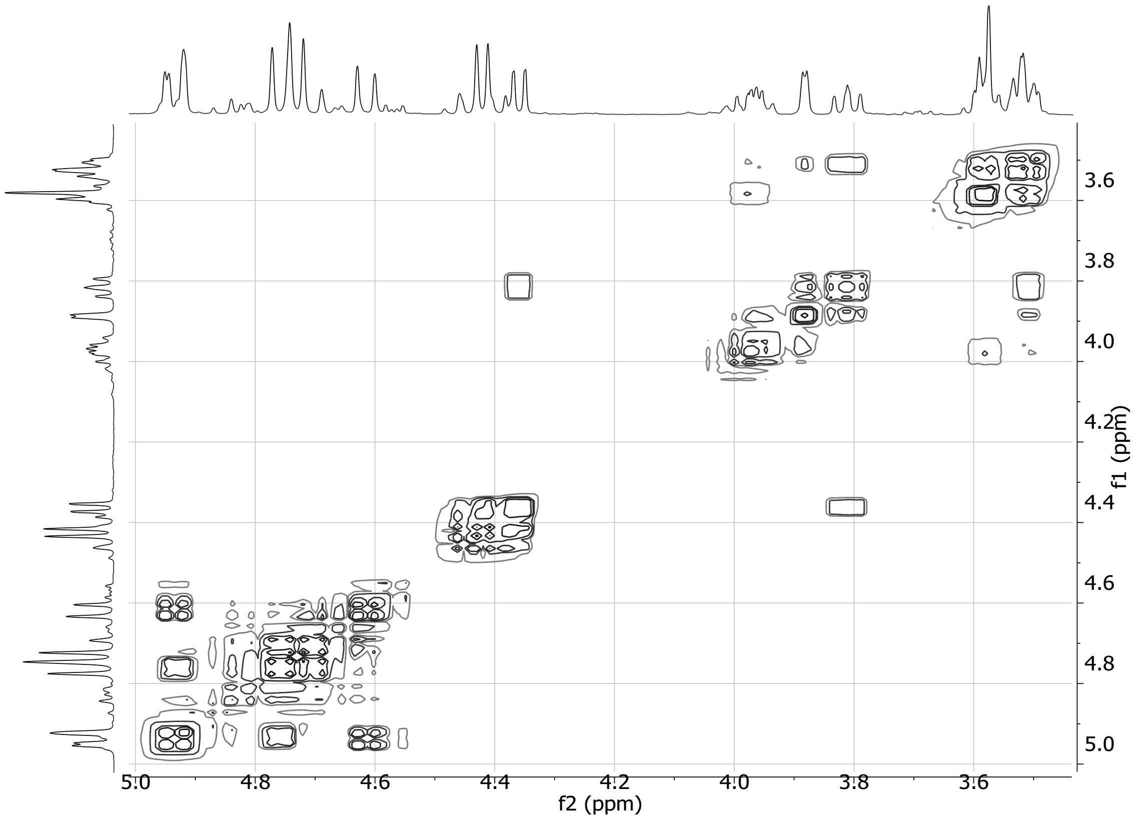

Supplementary Figure 295. HH-COSY NMR, $CDCl_3$ of compound **S77**

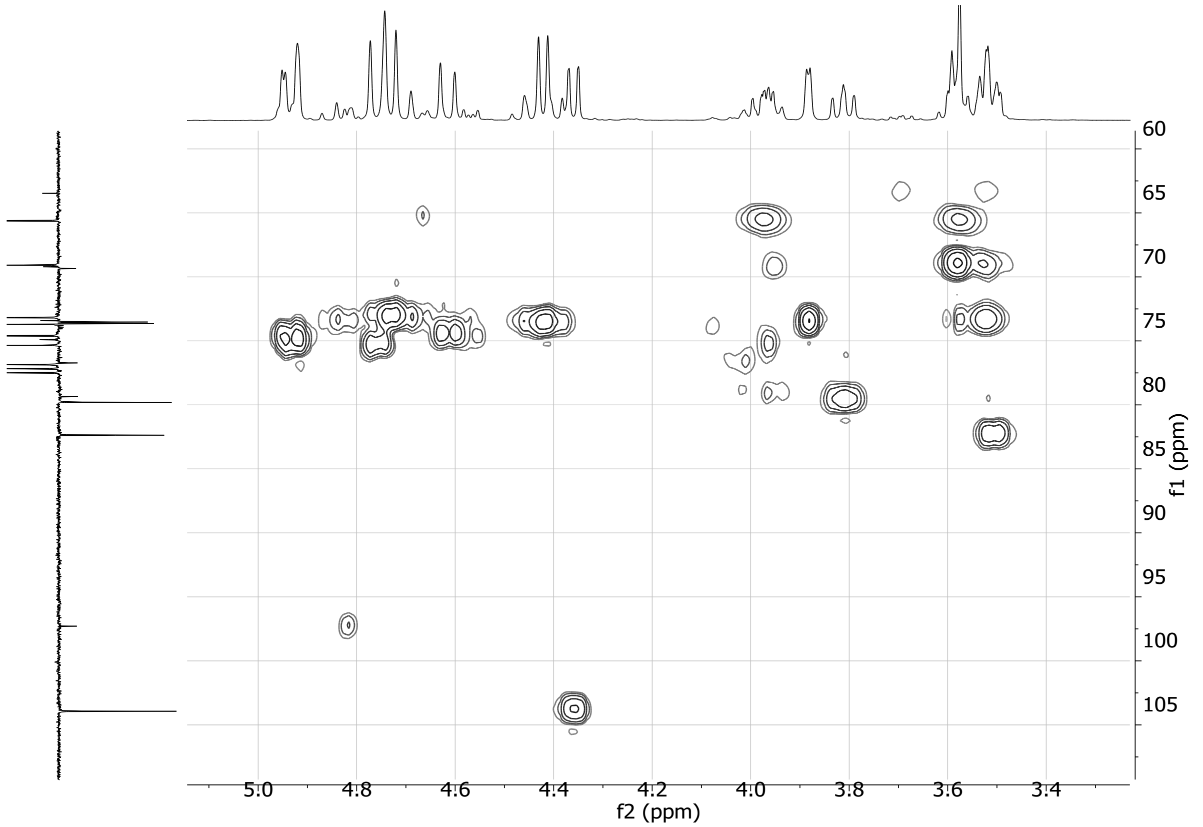

Supplementary Figure 296. HSQC NMR, $CDCl_3$ of compound **S7**

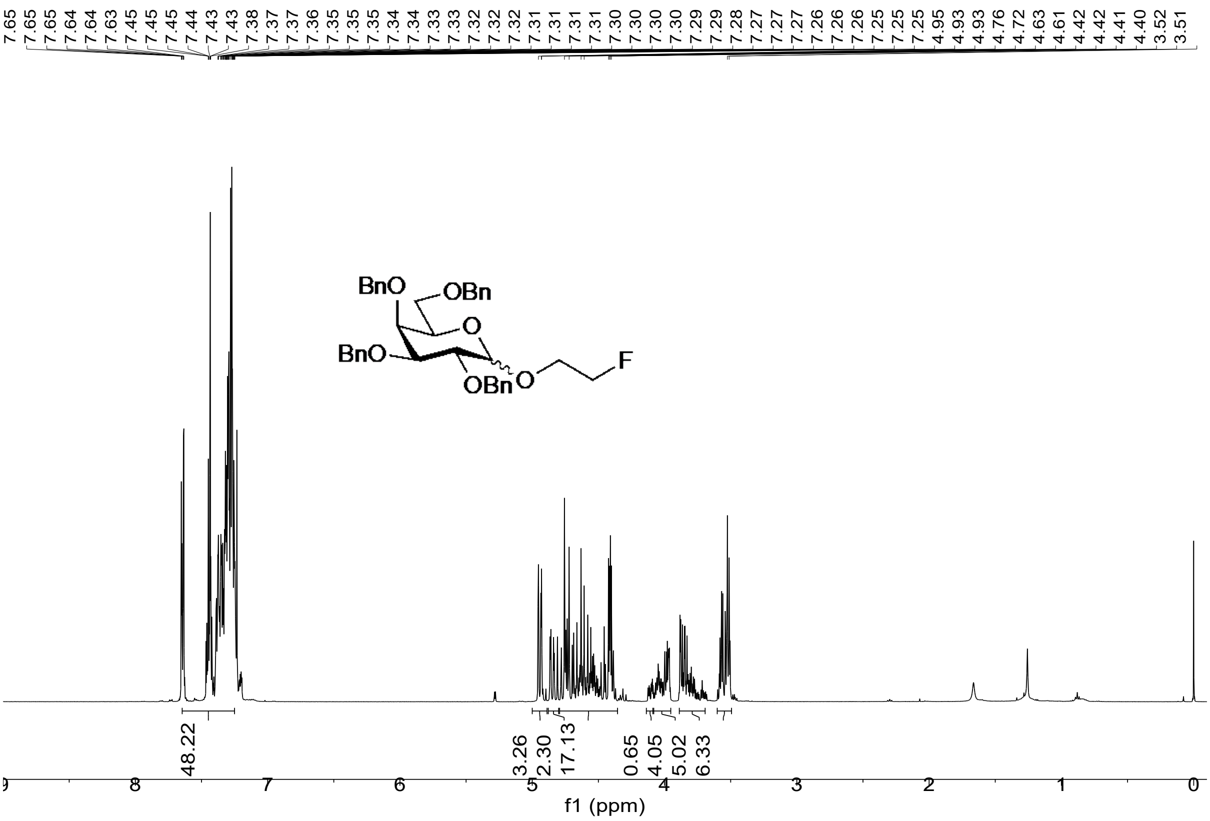

Supplementary Figure 297. ^{1}H NMR, 400 MHz, $CDCl_3$ of compound **S78**

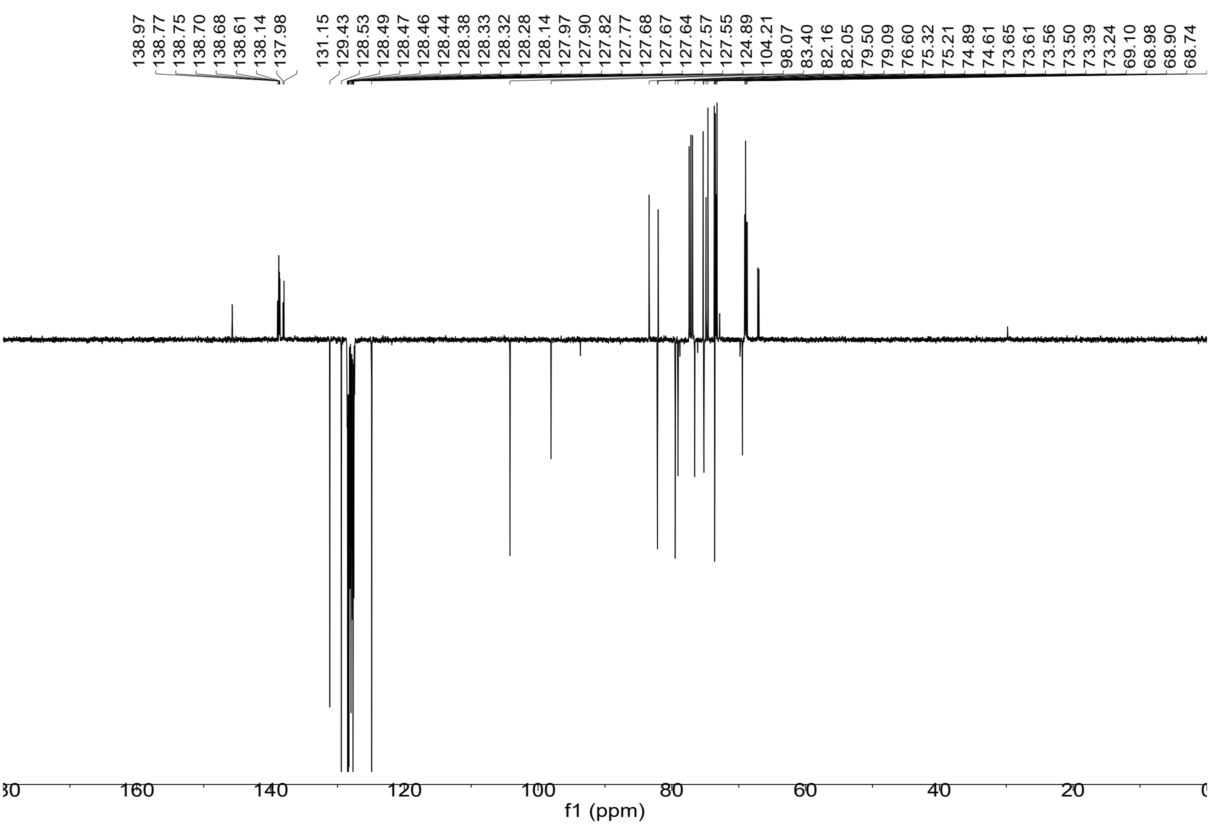

Supplementary Figure 298. ^{13}C NMR, 101 MHz, $CDCl_3$ of compound **S78**

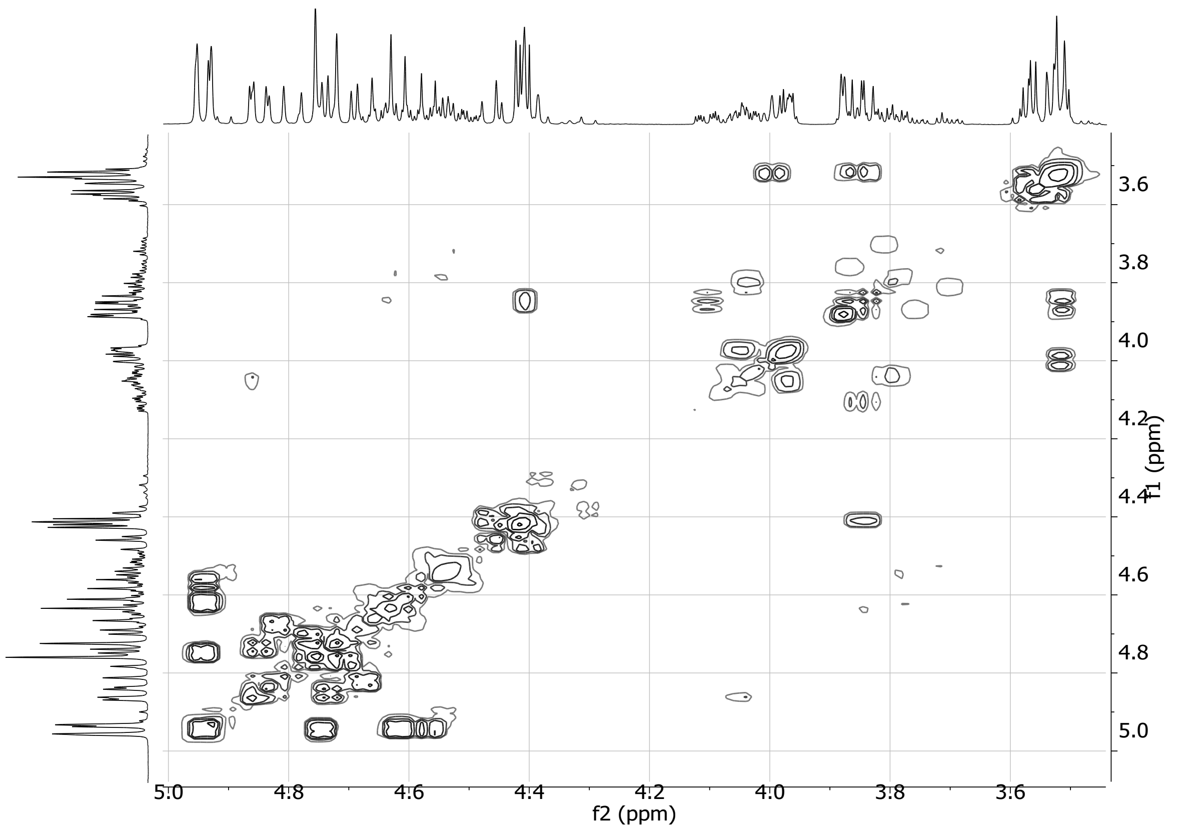

Supplementary Figure 299. HH-COSY NMR, $CDCl_3$ of compound **S78**

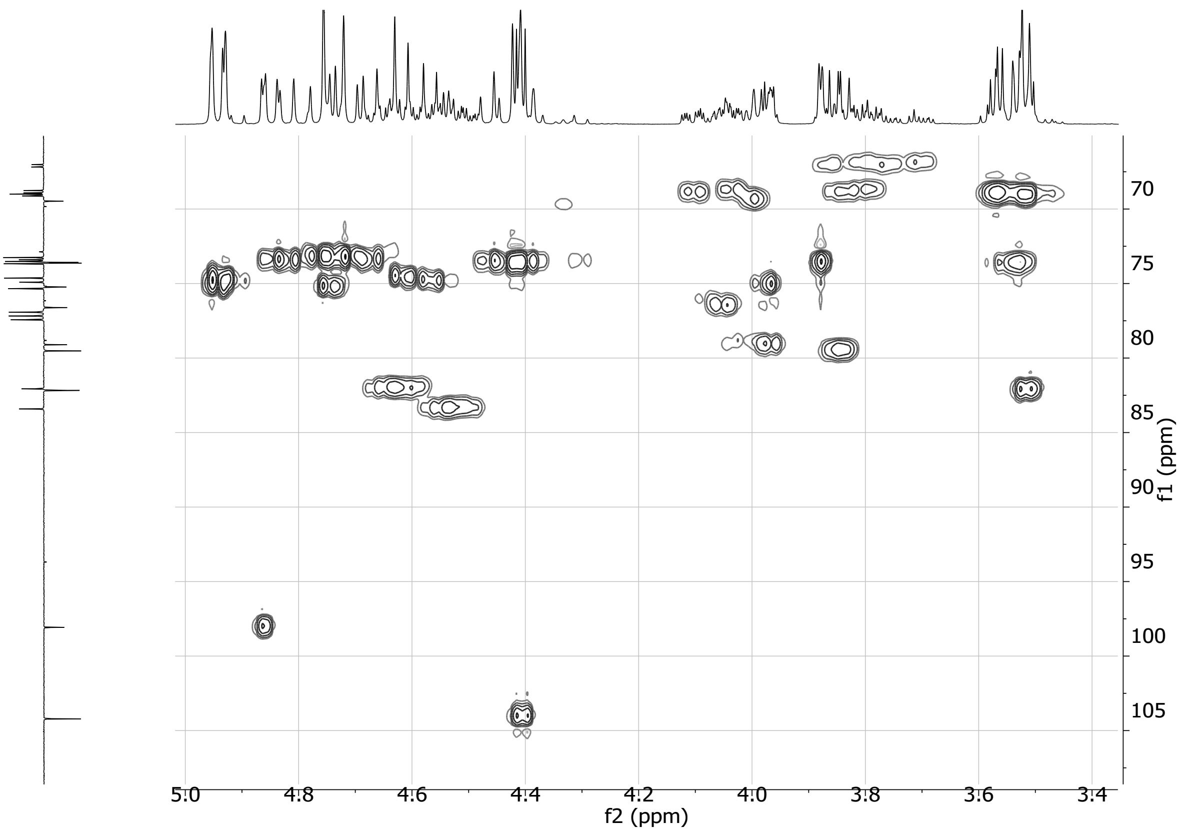

Supplementary Figure 300. HSQC NMR, $CDCl_3$ of compound **S78**

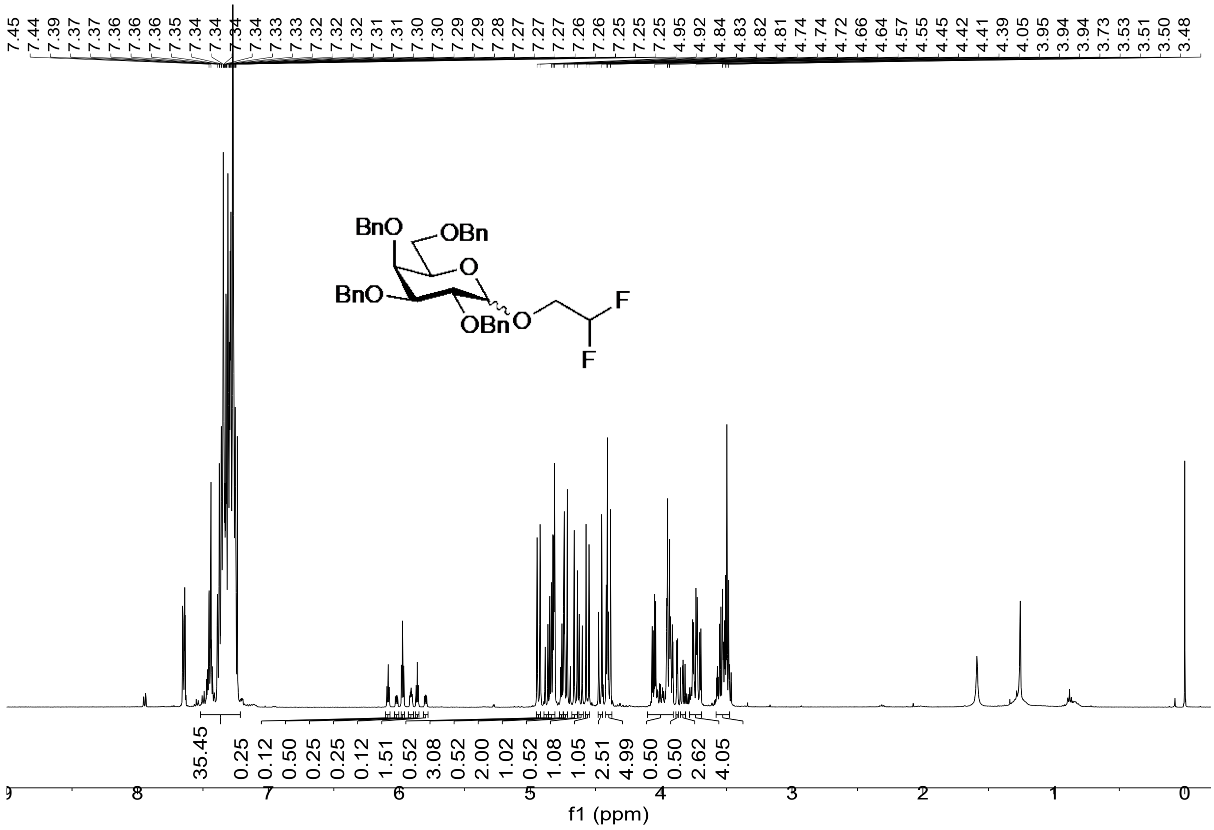

Supplementary Figure 301. ^{1}H NMR, 500 MHz, $CDCl_3$ of compound **S79**

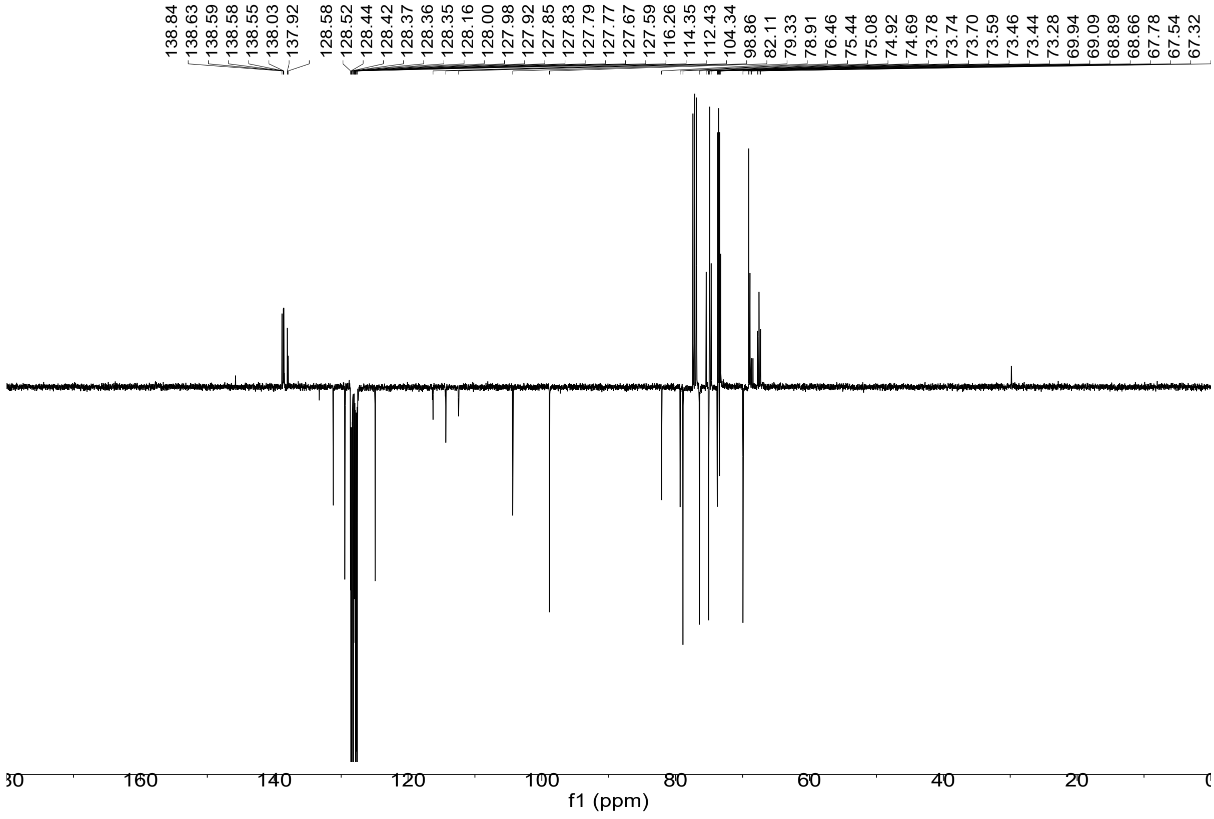

Supplementary Figure 302. ^{13}C NMR, 126 MHz, $CDCl_3$ of compound **S79**

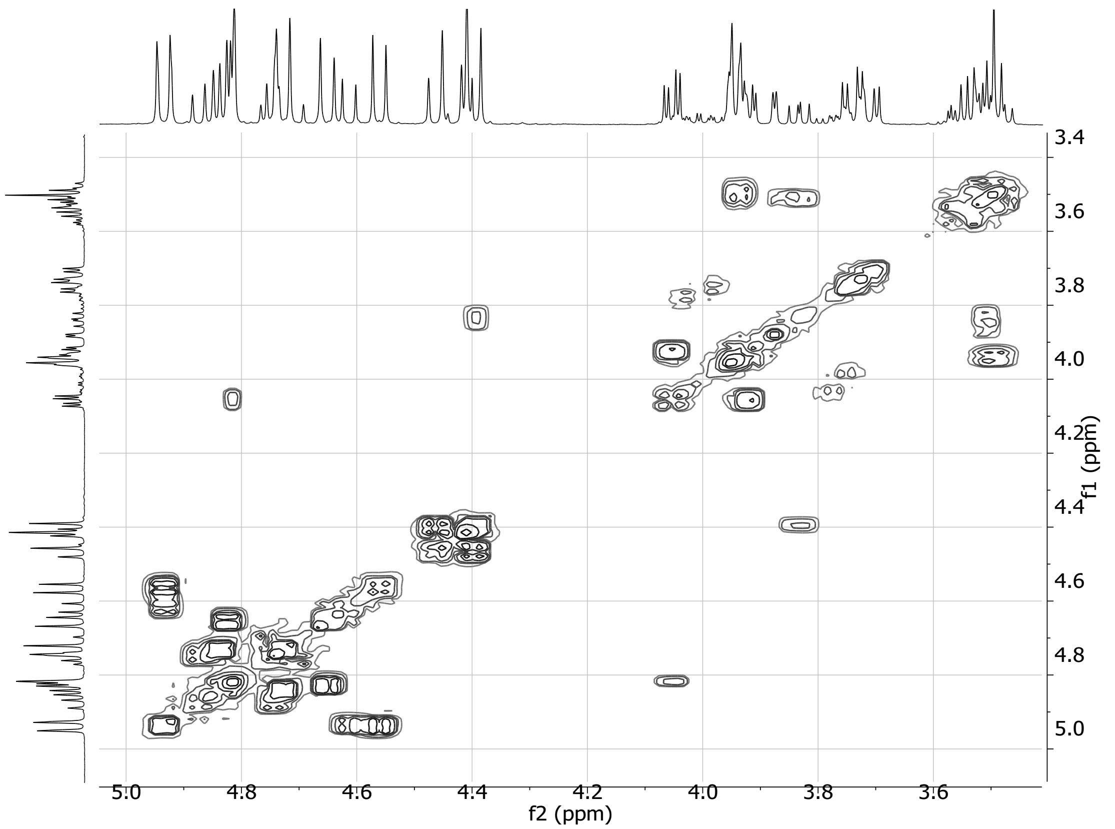

Supplementary Figure 303. HH-COSY NMR, $CDCl_3$ of compound **S79**

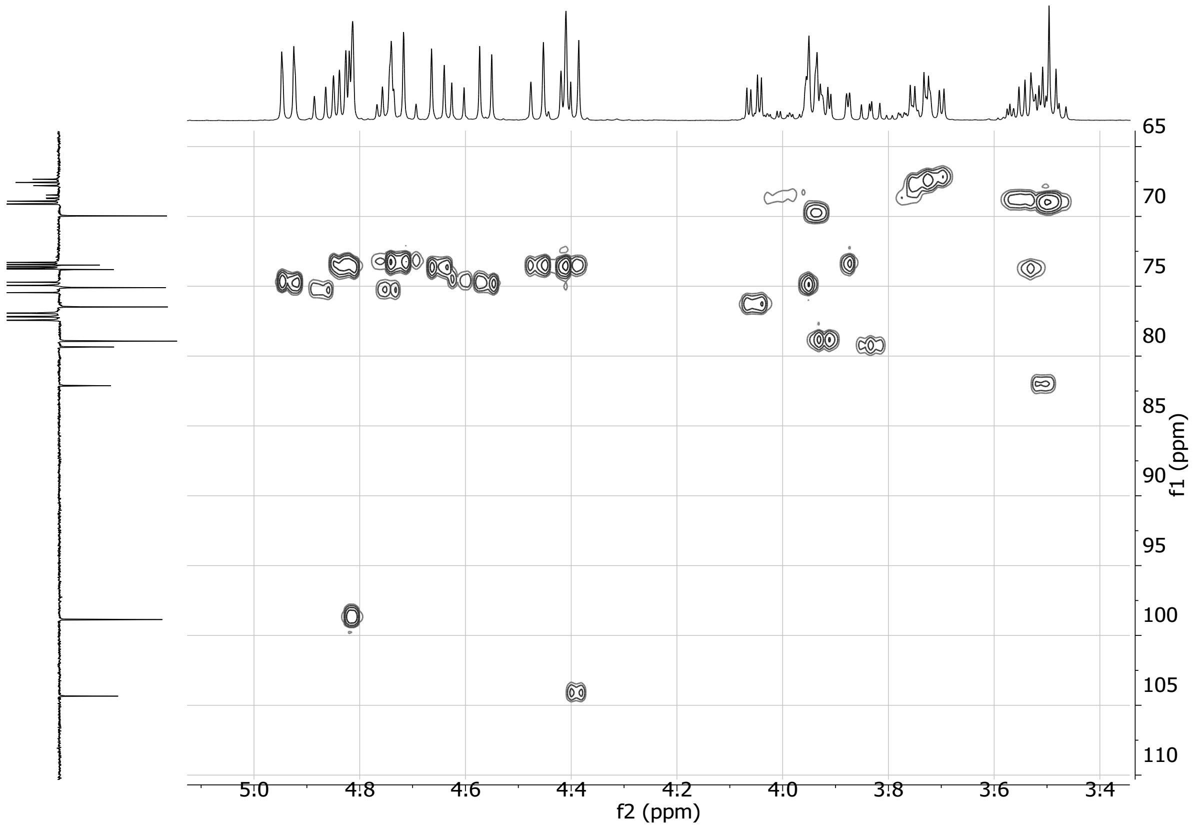

Supplementary Figure 304. HSQC NMR, $CDCl_3$ of compound **S79**

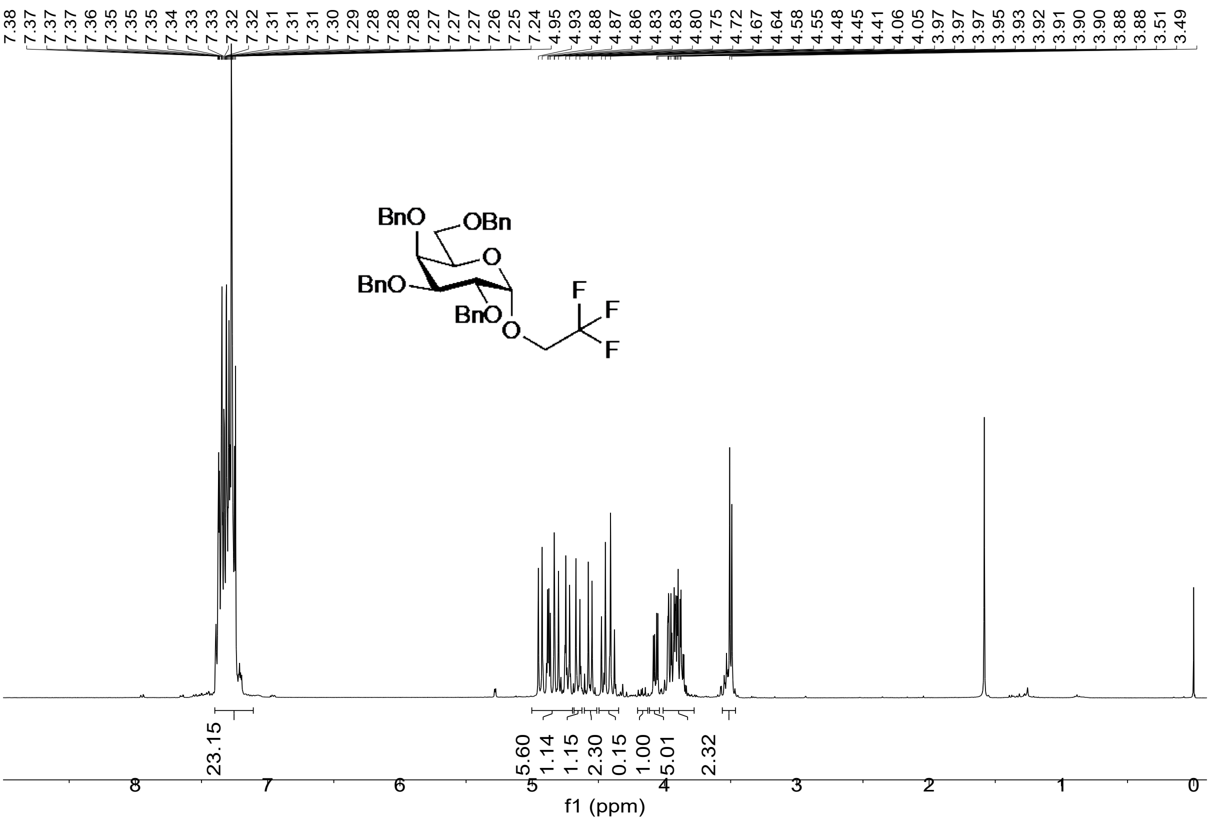

Supplementary Figure 305. ^{1}H NMR, 400 MHz, $CDCl_3$ of compound **S80**

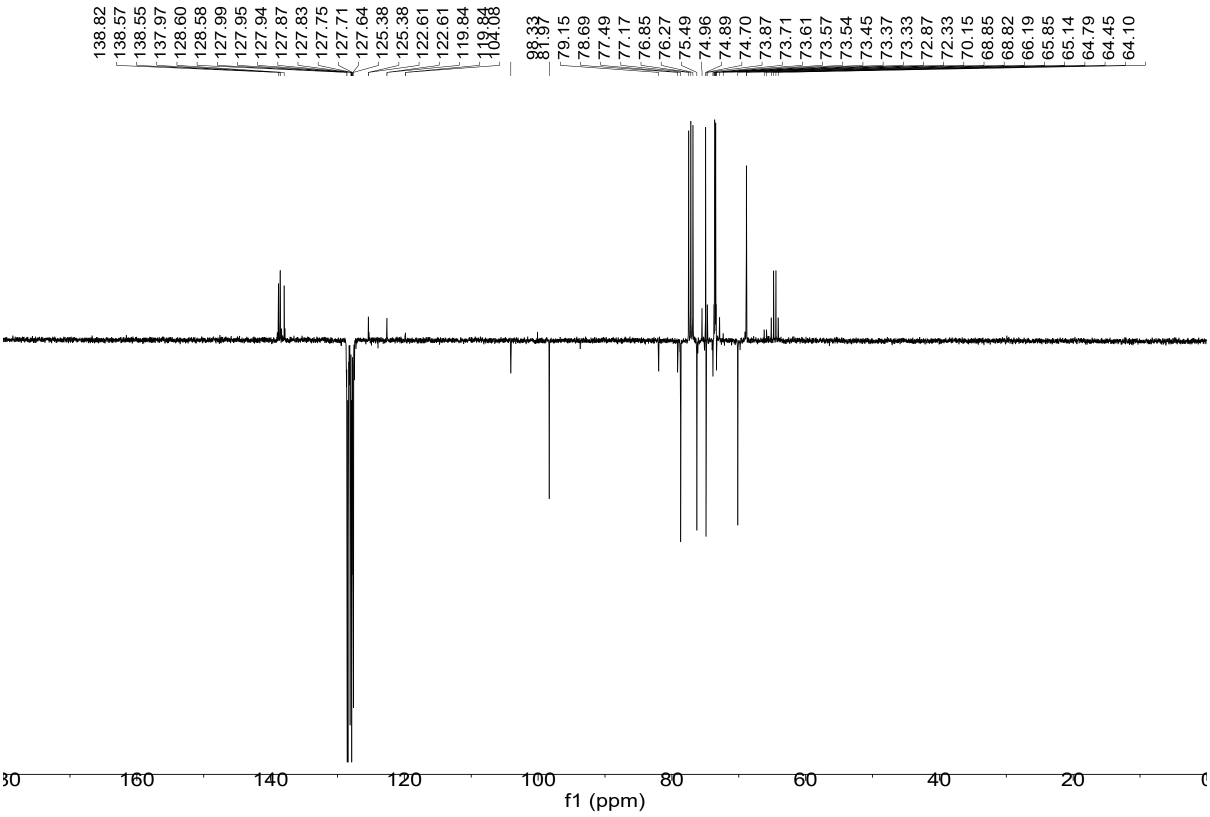

Supplementary Figure 306. ^{13}C NMR, 101 MHz, $CDCl_3$ of compound **S80**

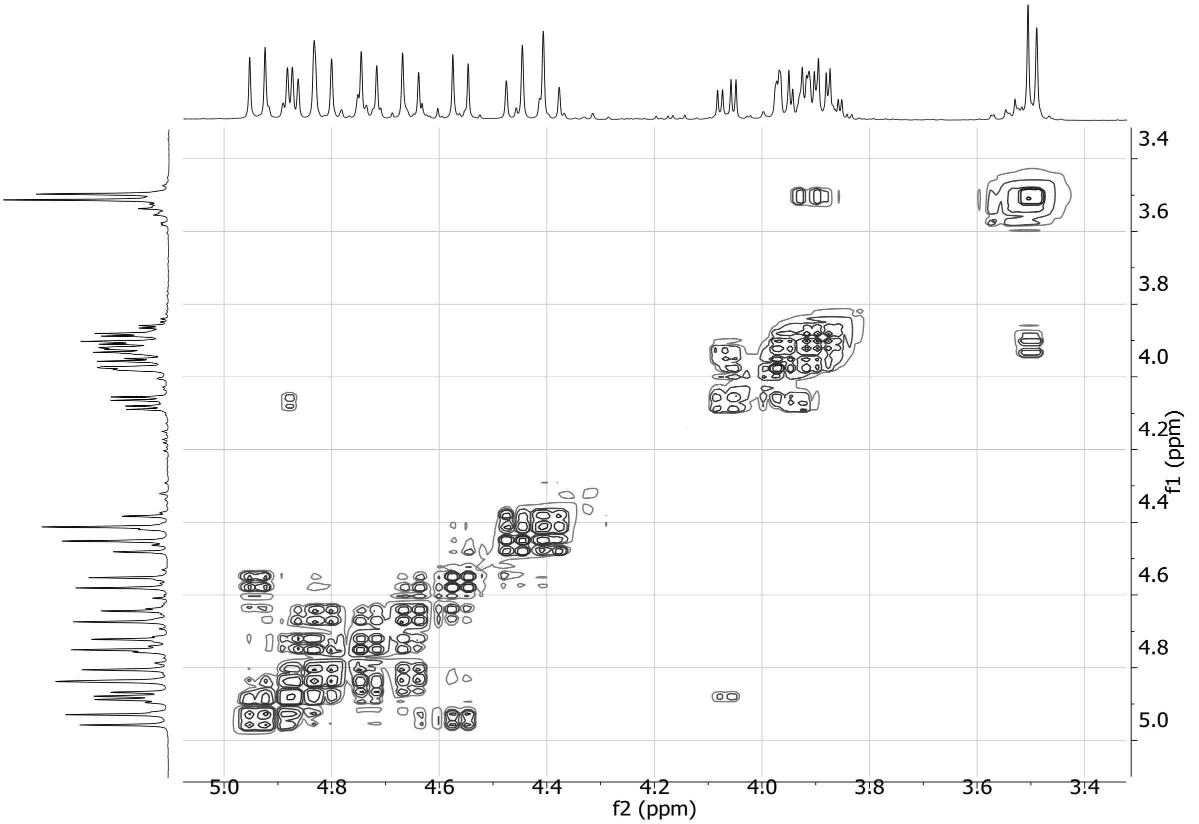

Supplementary Figure 307. HH-COSY NMR, $CDCl_3$ of compound **S80**

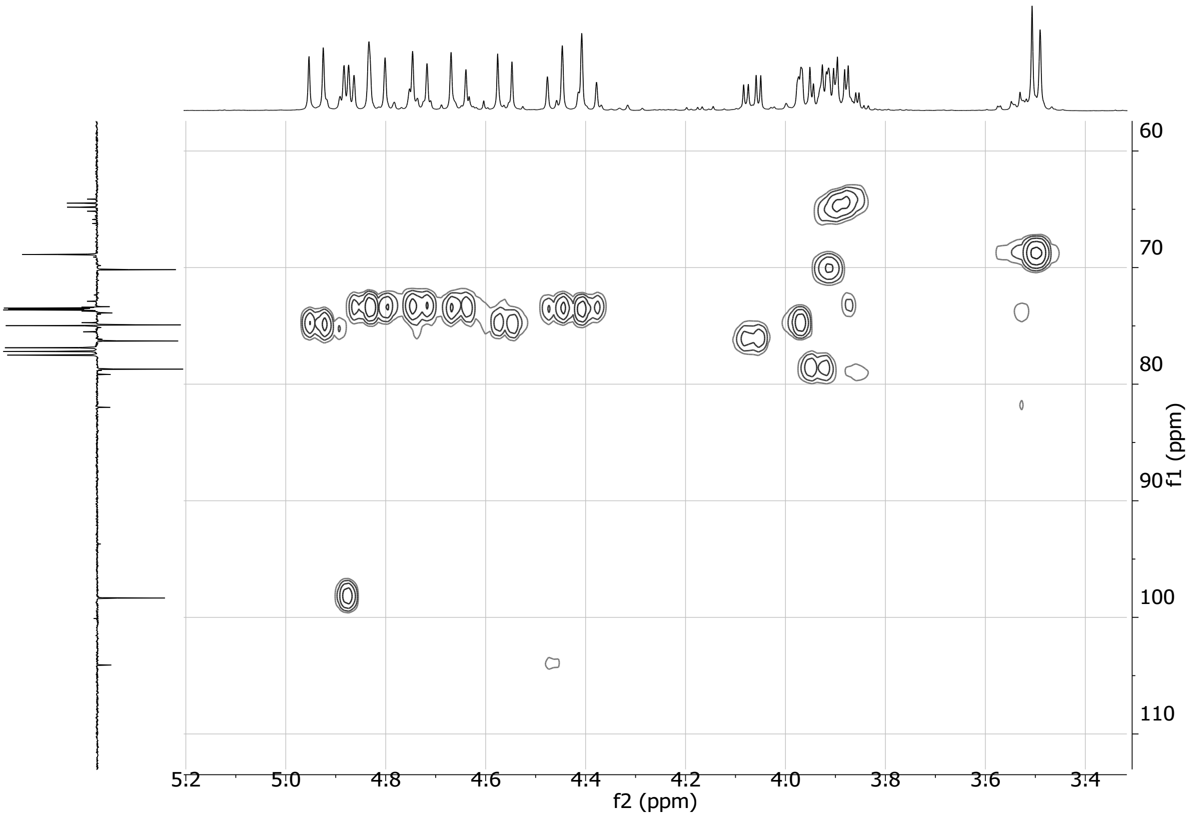

Supplementary Figure 308. HSQC NMR, $CDCl_3$ of compound **S80**

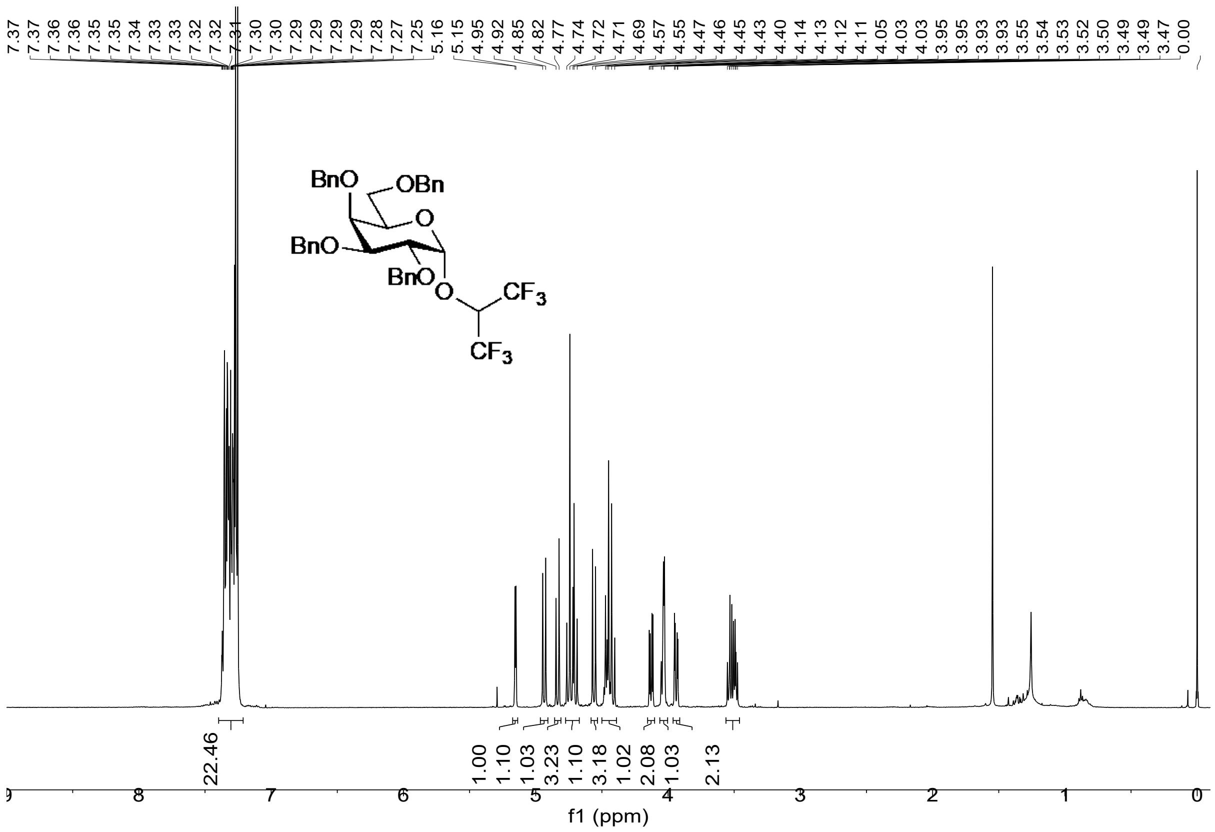

Supplementary Figure 309. ^{1}H NMR, 500 MHz, $CDCl_3$ of compound **S81**

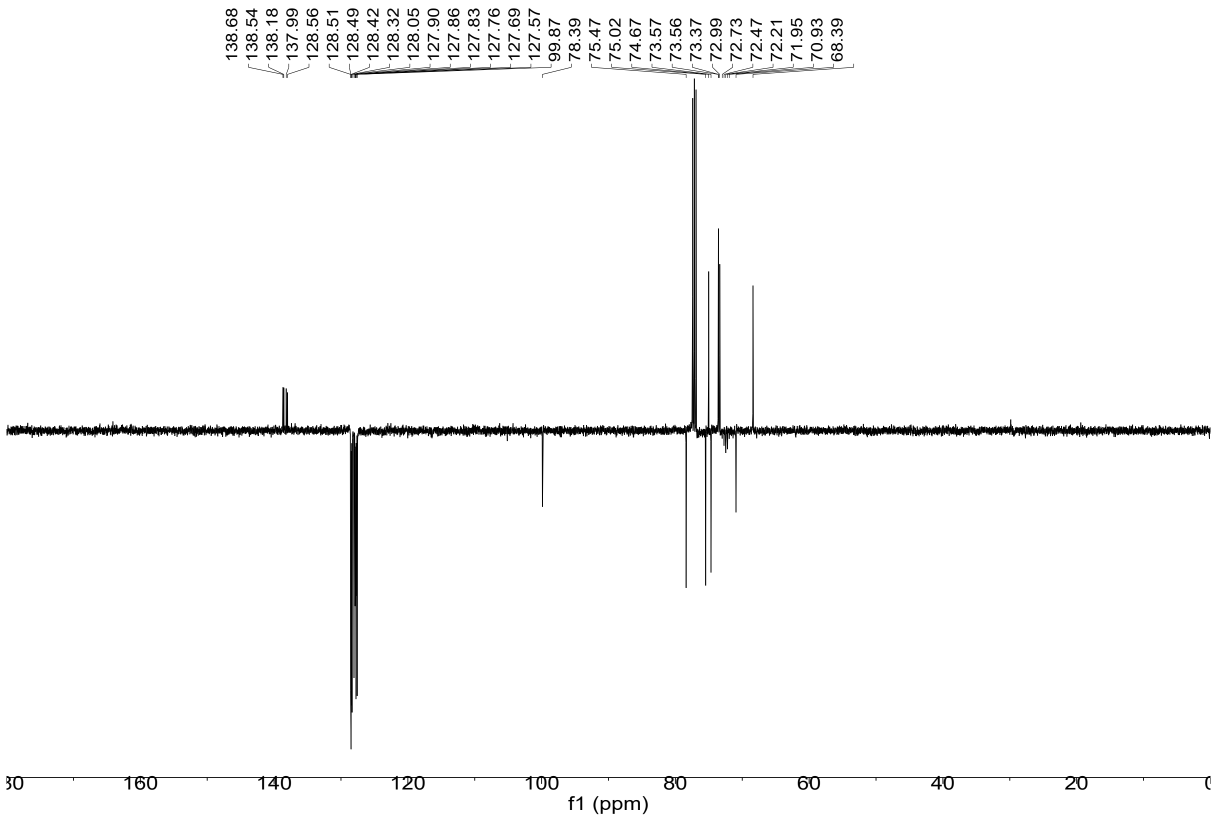

Supplementary Figure 310. ^{13}C NMR, 126 MHz, $CDCl_3$ of compound **S81**

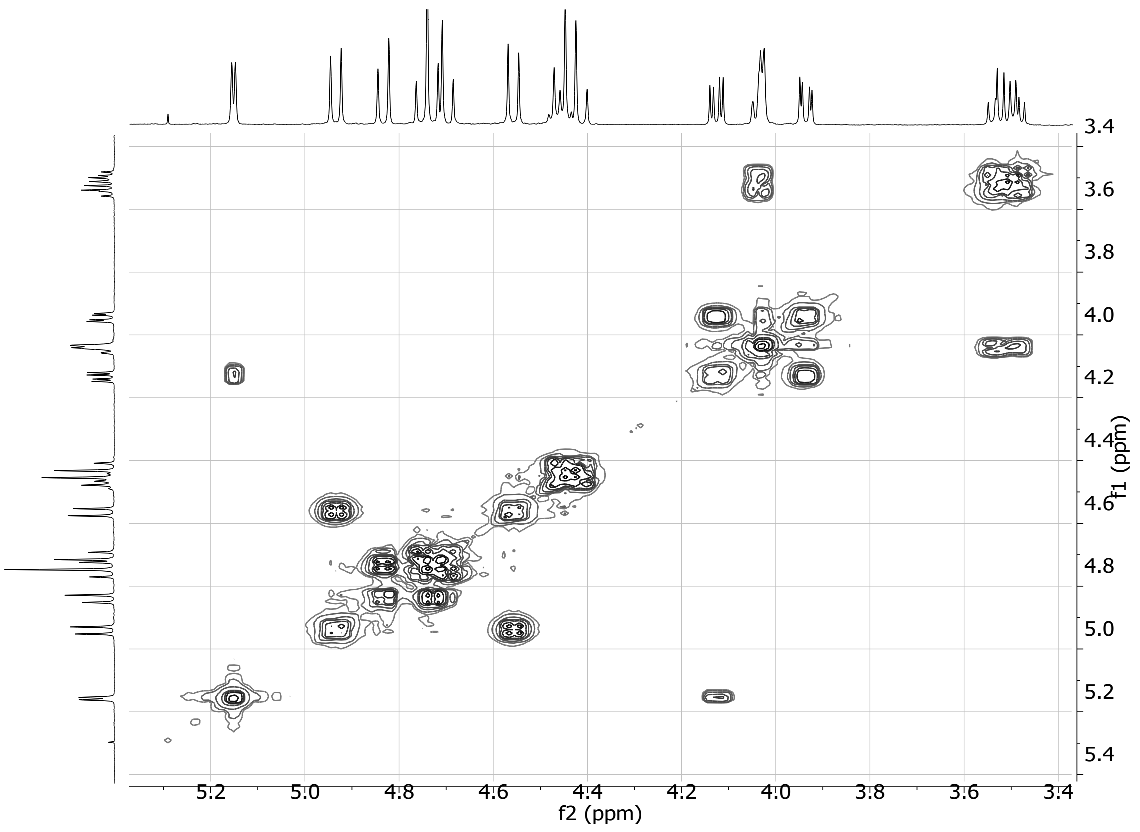

Supplementary Figure 311. HH-COSY NMR, $CDCl_3$ of compound **S81**

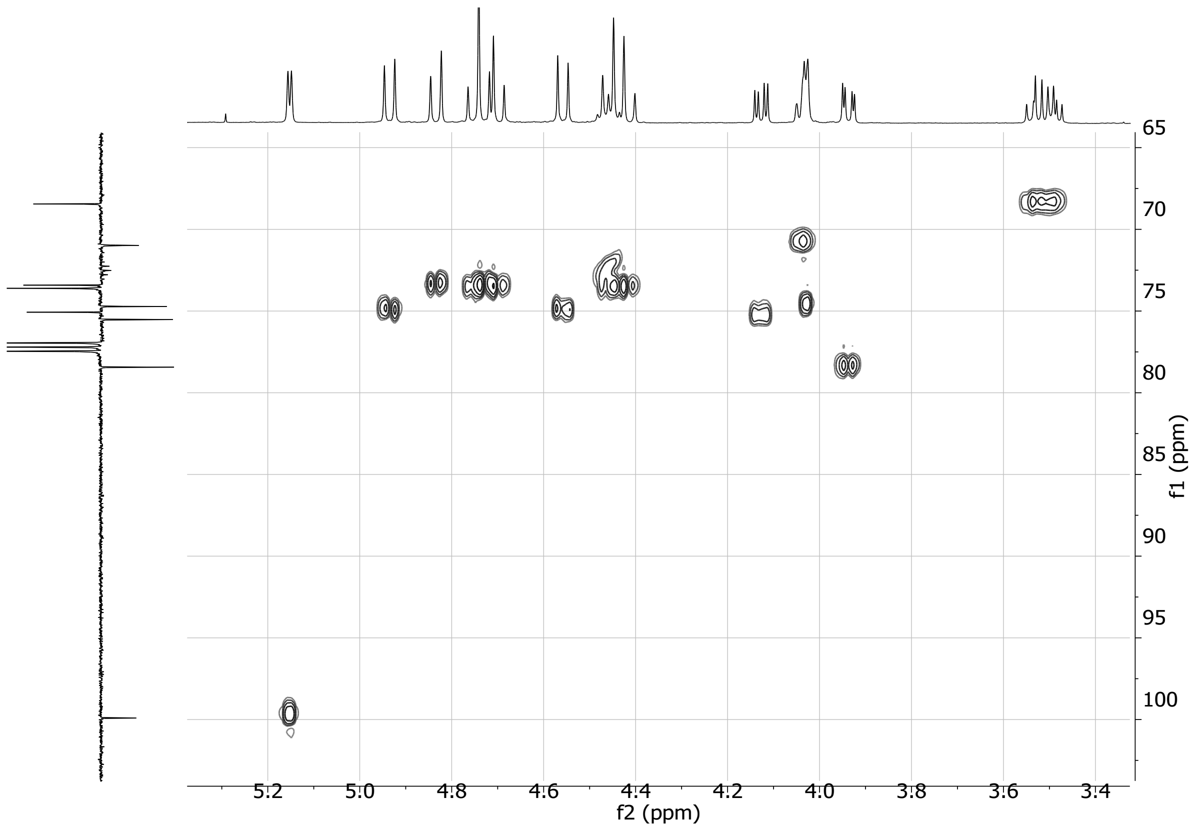

Supplementary Figure 312. HSQC NMR, $CDCl_3$ of compound **S81**

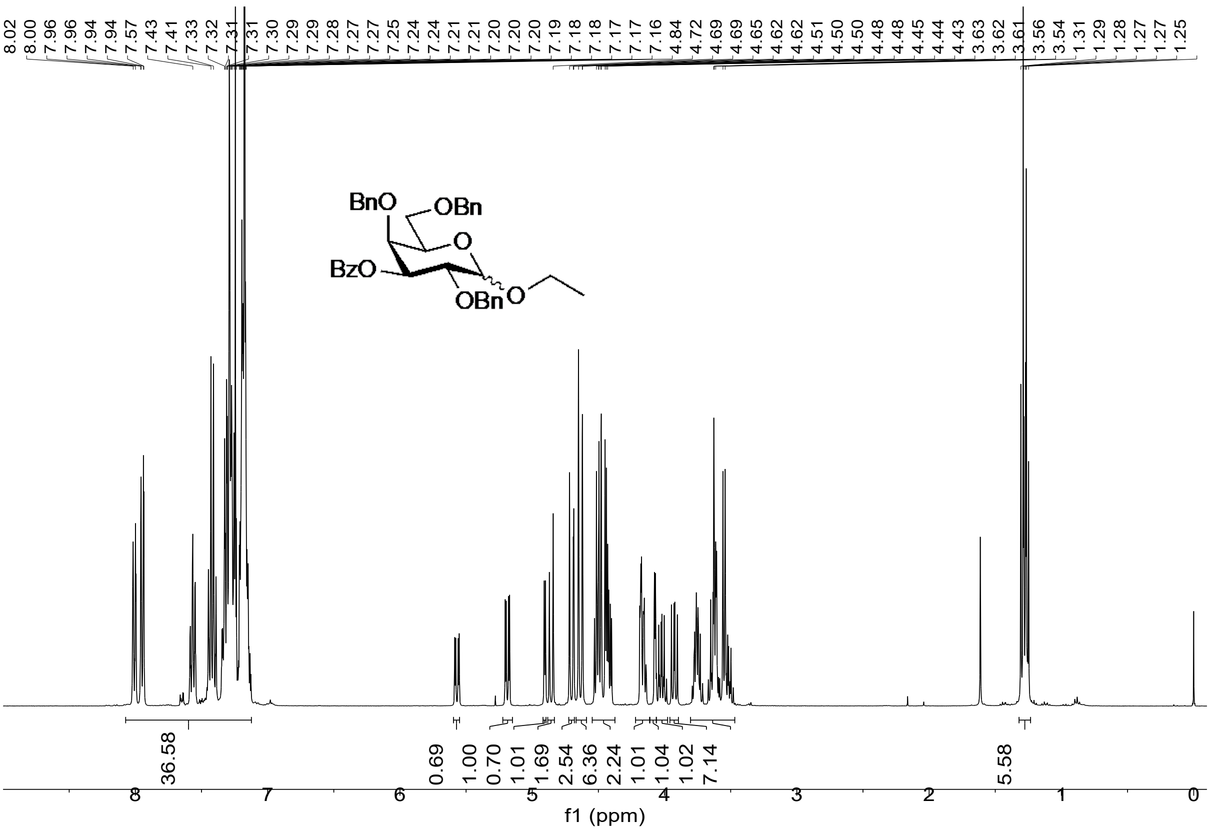

Supplementary Figure 313. ^{1}H NMR, 400 MHz, $CDCl_3$ of compound **S82**

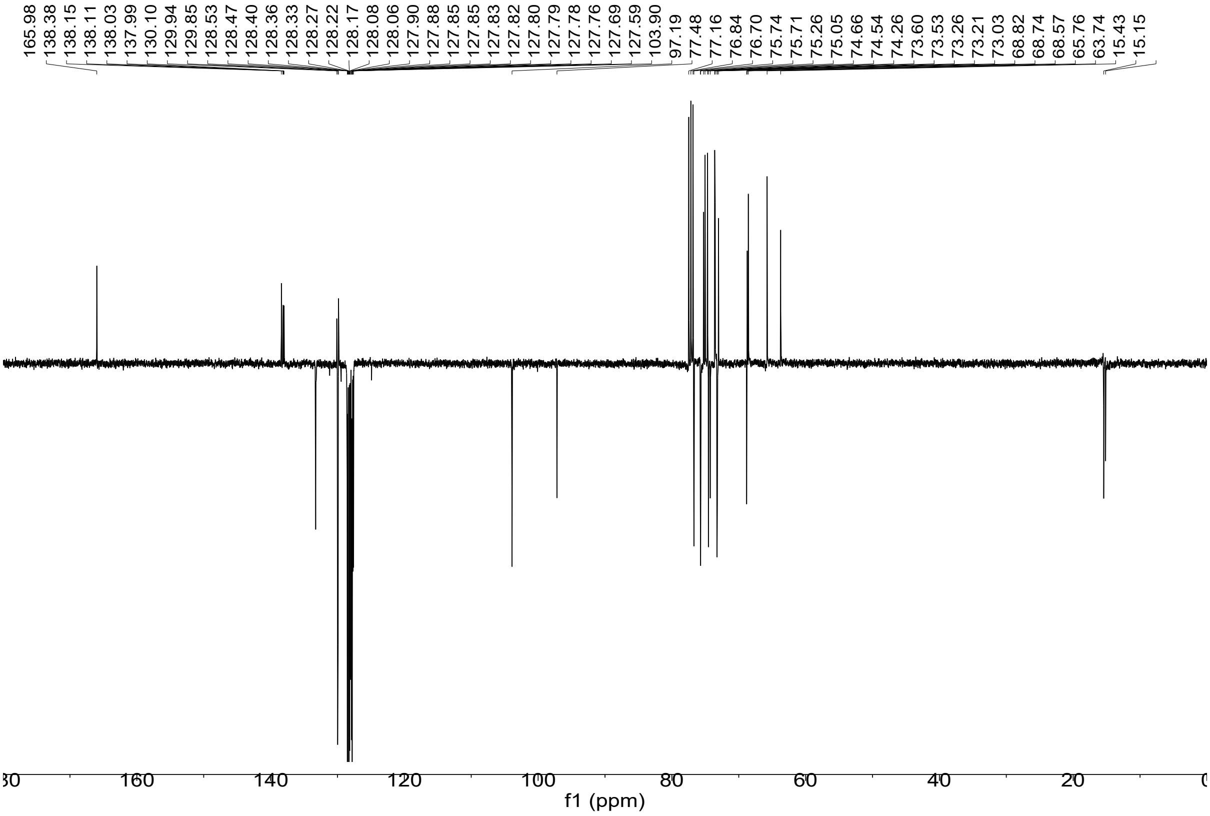

Supplementary Figure 314. ^{13}C NMR, 101MHz, $CDCl_3$ of compound **S82**

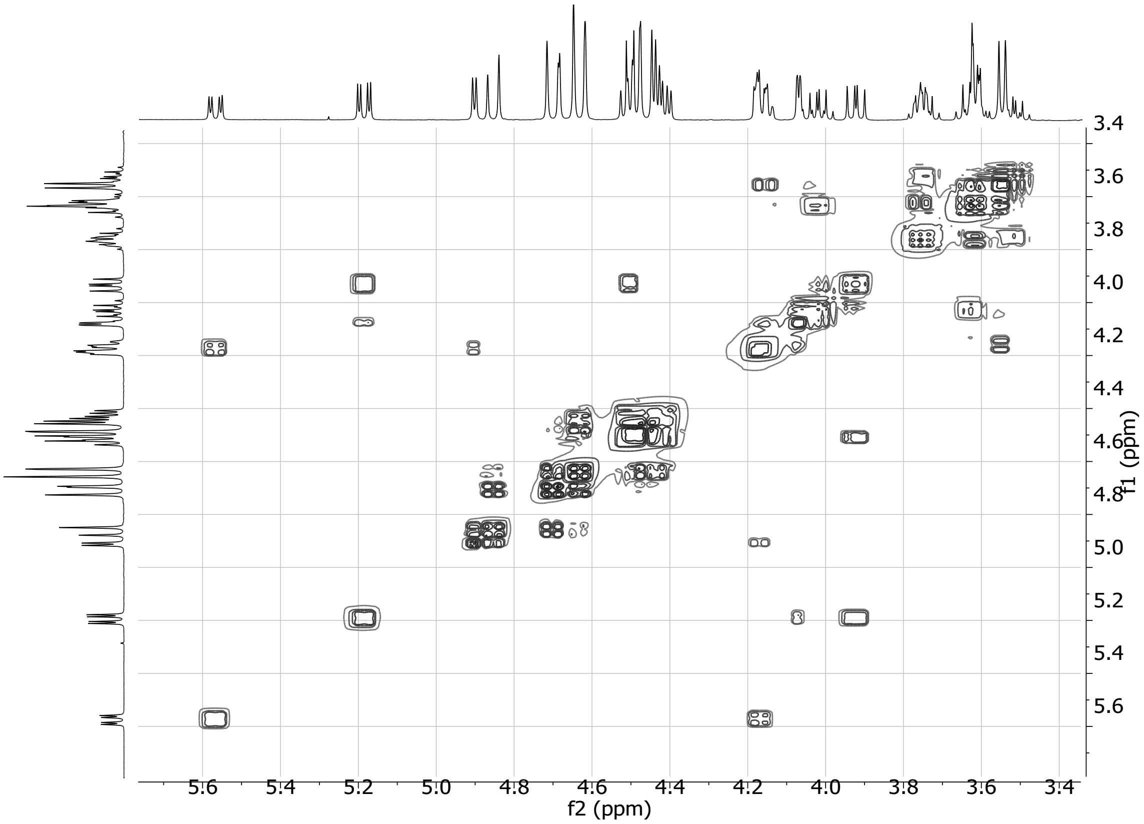

Supplementary Figure 315. HH-COSY NMR, $CDCl_3$ of compound **S82**

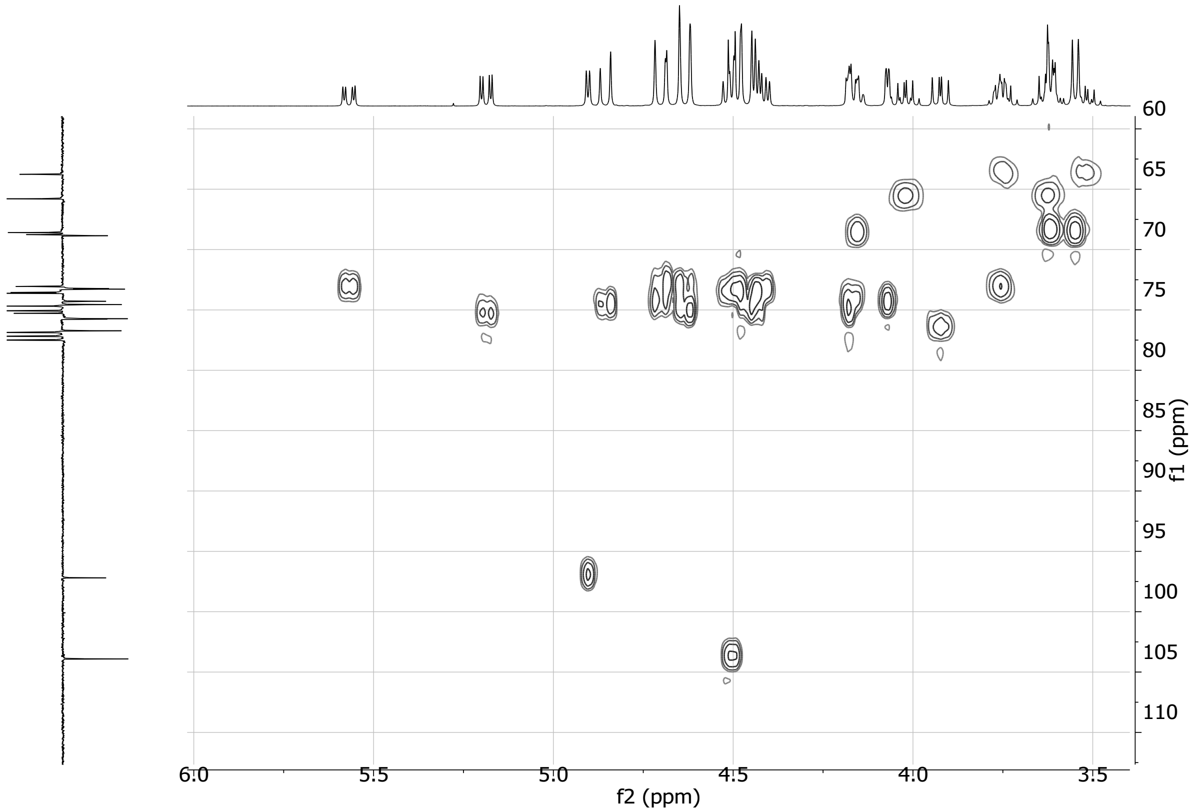

Supplementary Figure 316. HSQC NMR, $CDCl_3$ of compound **S82**

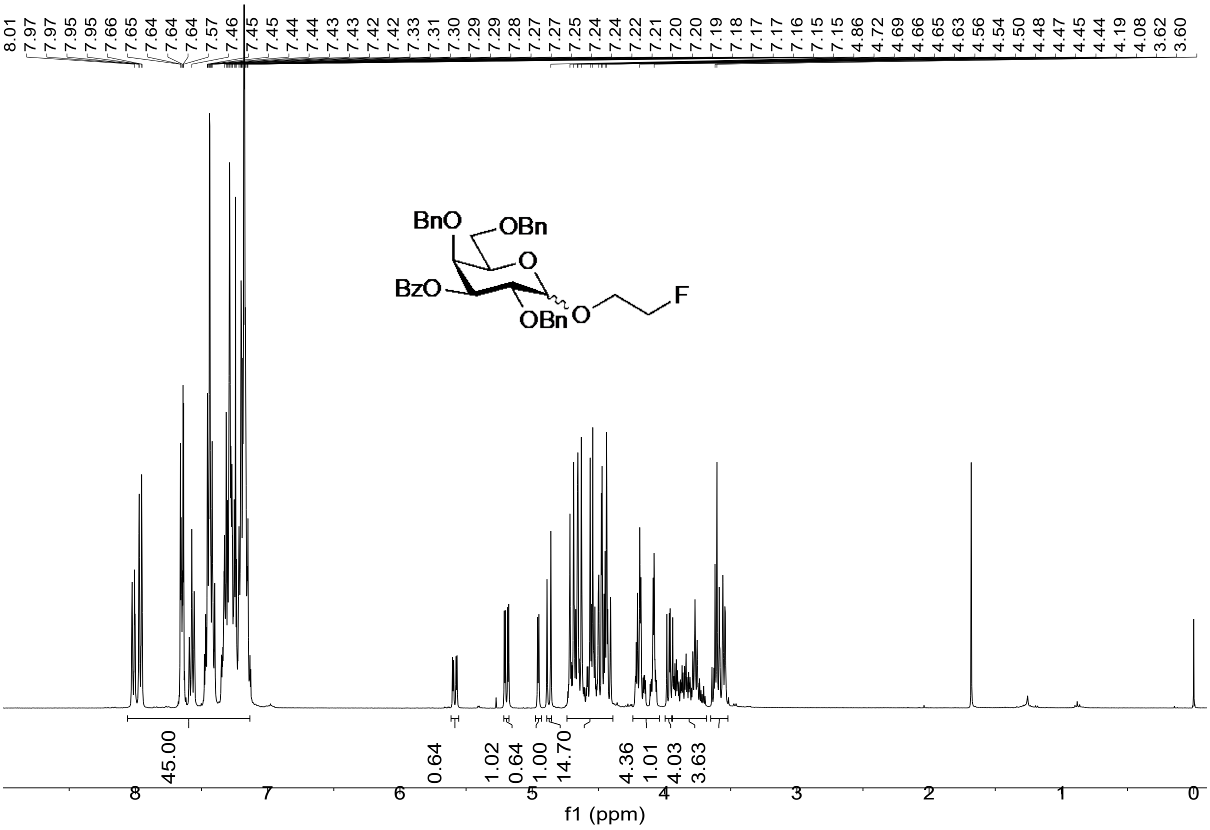

Supplementary Figure 317. ^{1}H NMR, 400 MHz, $CDCl_3$ of compound **S83**

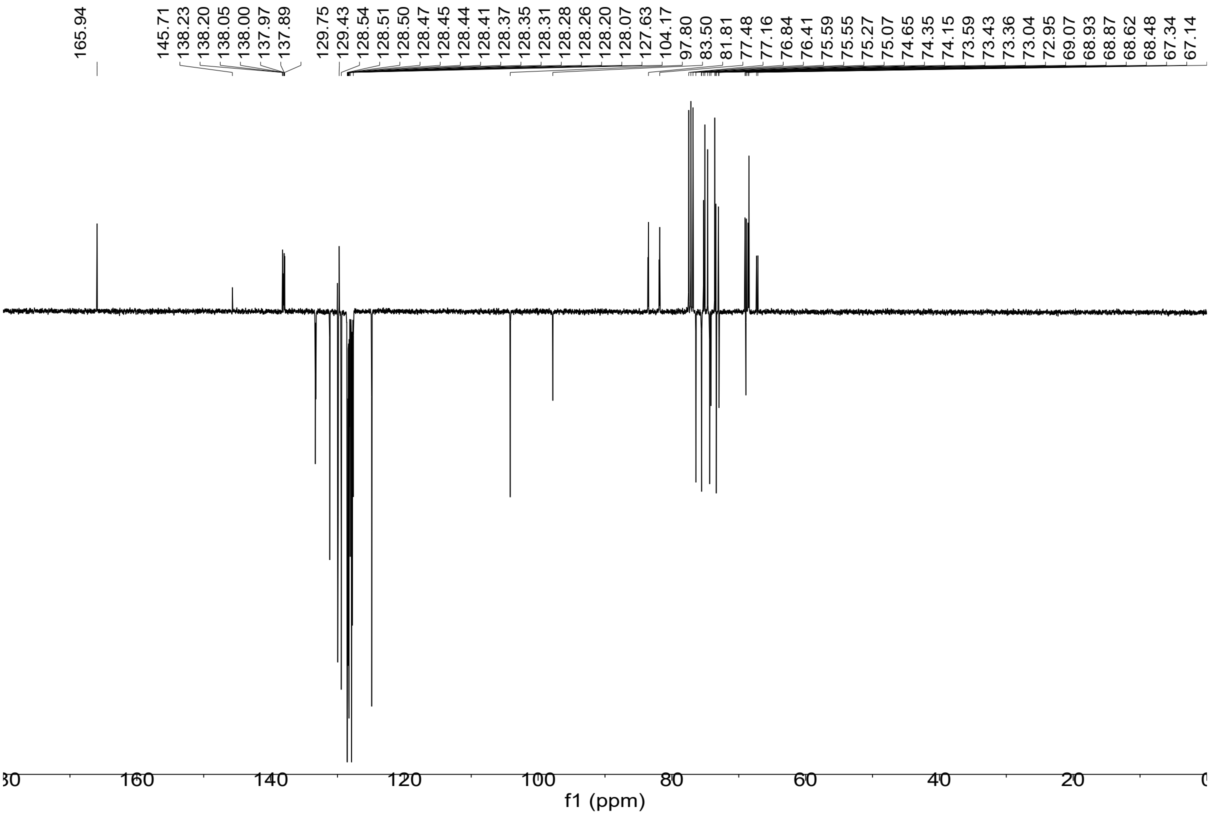

Supplementary Figure 318. ^{13}C NMR, 101 MHz, $CDCl_3$ of compound **S83**

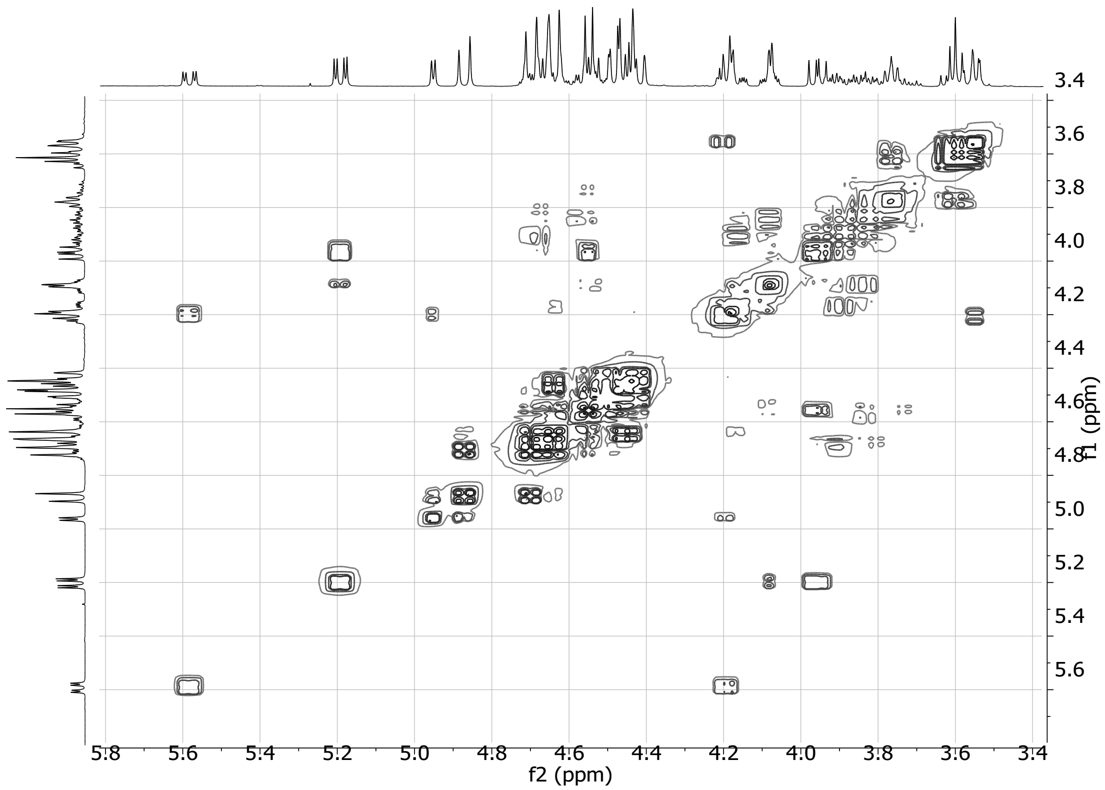

Supplementary Figure 319. HH-COSY NMR, $CDCl_3$ of compound **S83**

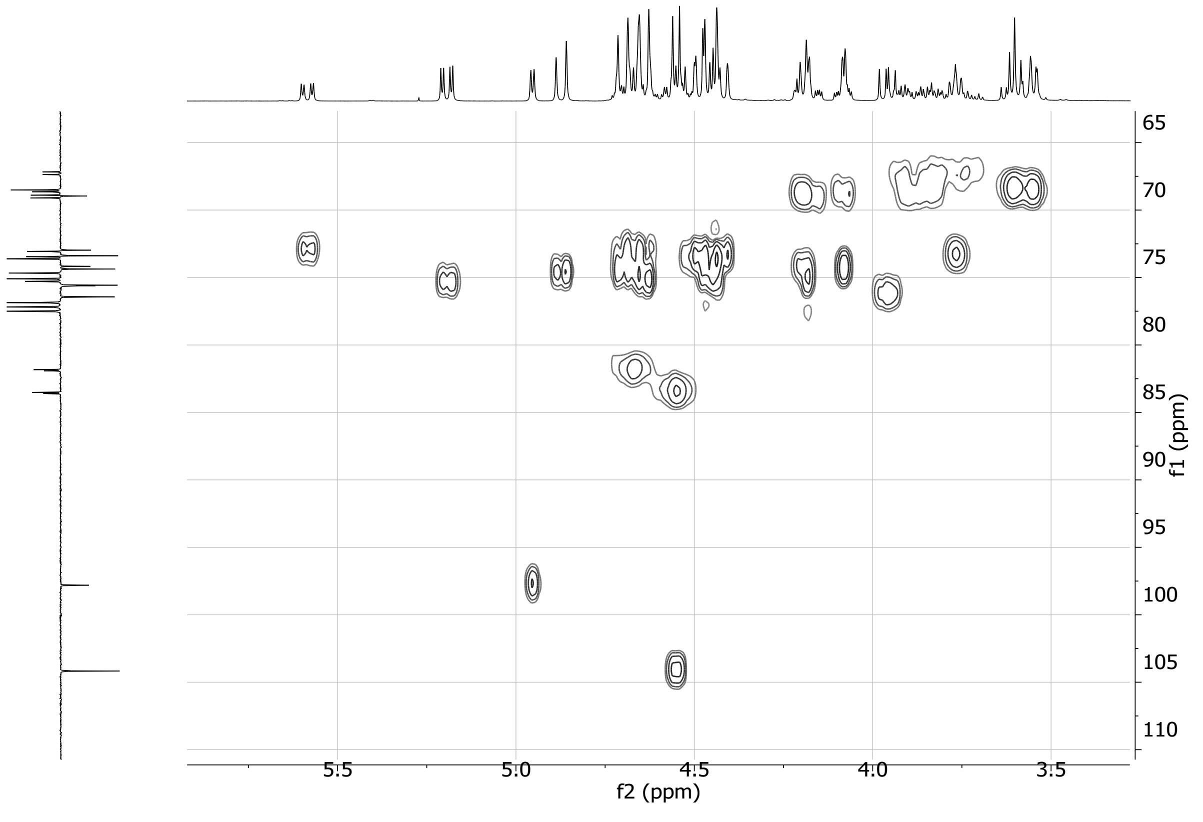

Supplementary Figure 320. HSQC NMR, $CDCl_3$ of compound **S83**

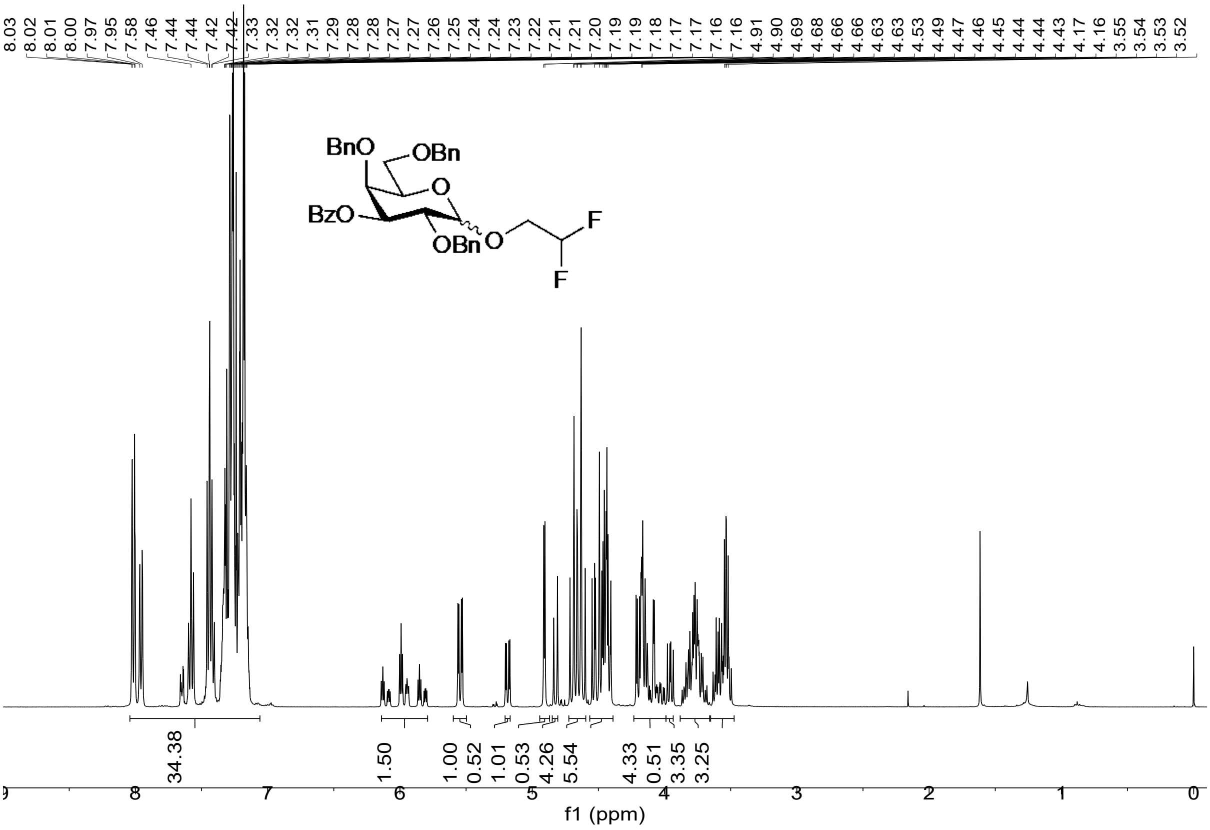

Supplementary Figure 321. ^{1}H NMR, 400 MHz, $CDCl_3$ of compound **S84**

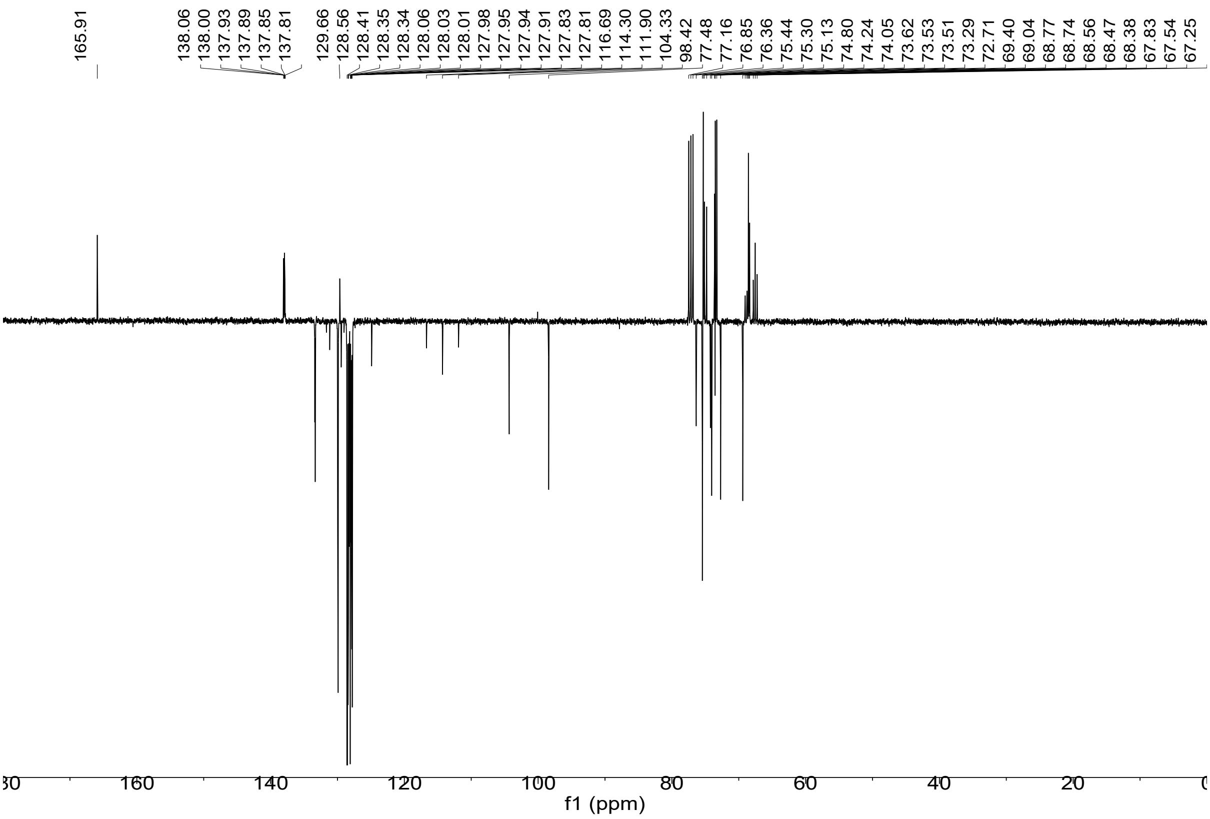

Supplementary Figure 322. ^{13}C NMR, 101 MHz, $CDCl_3$ of compound **S84**

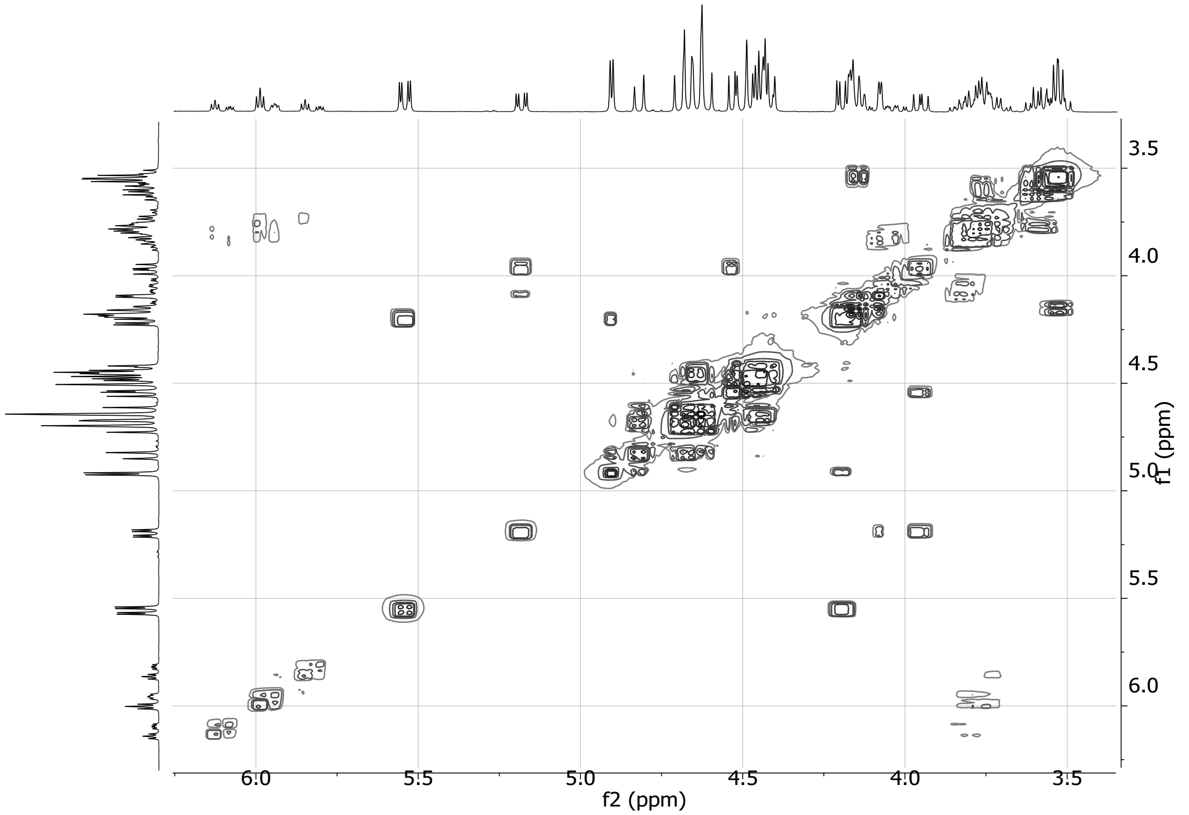

Supplementary Figure 323. HH-COSY NMR, $CDCl_3$ of compound **S84**

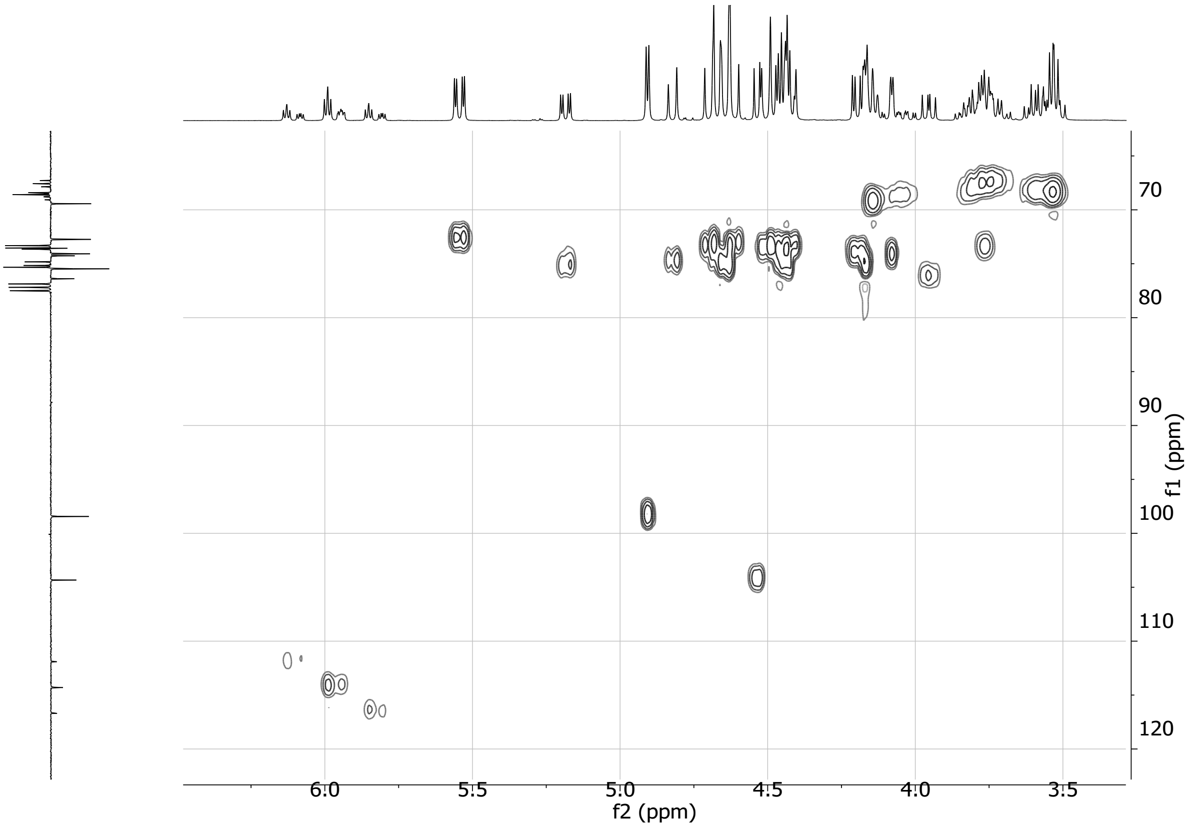

Supplementary Figure 324. HSQC NMR, $CDCl_3$ of compound **S84**

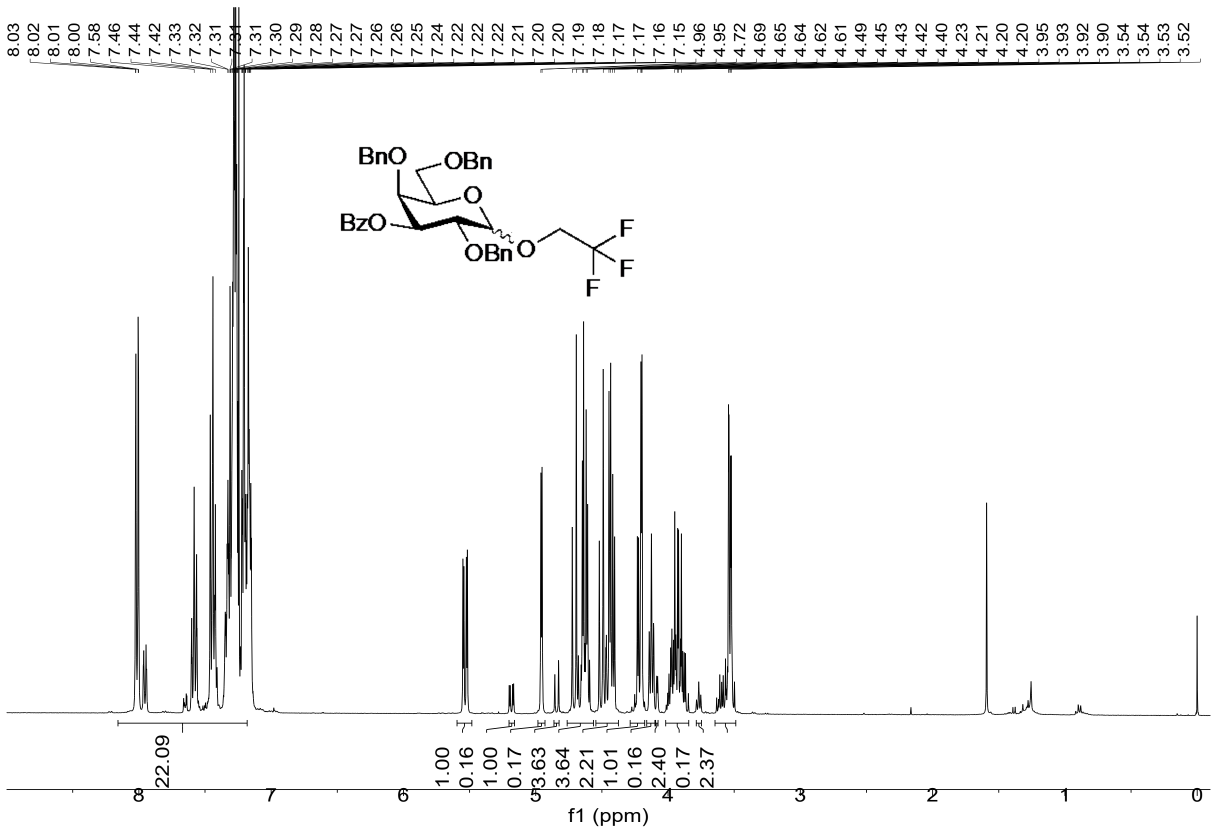

Supplementary Figure 325. ^{1}H NMR, 400 MHz, $CDCl_3$ of compound **S85**

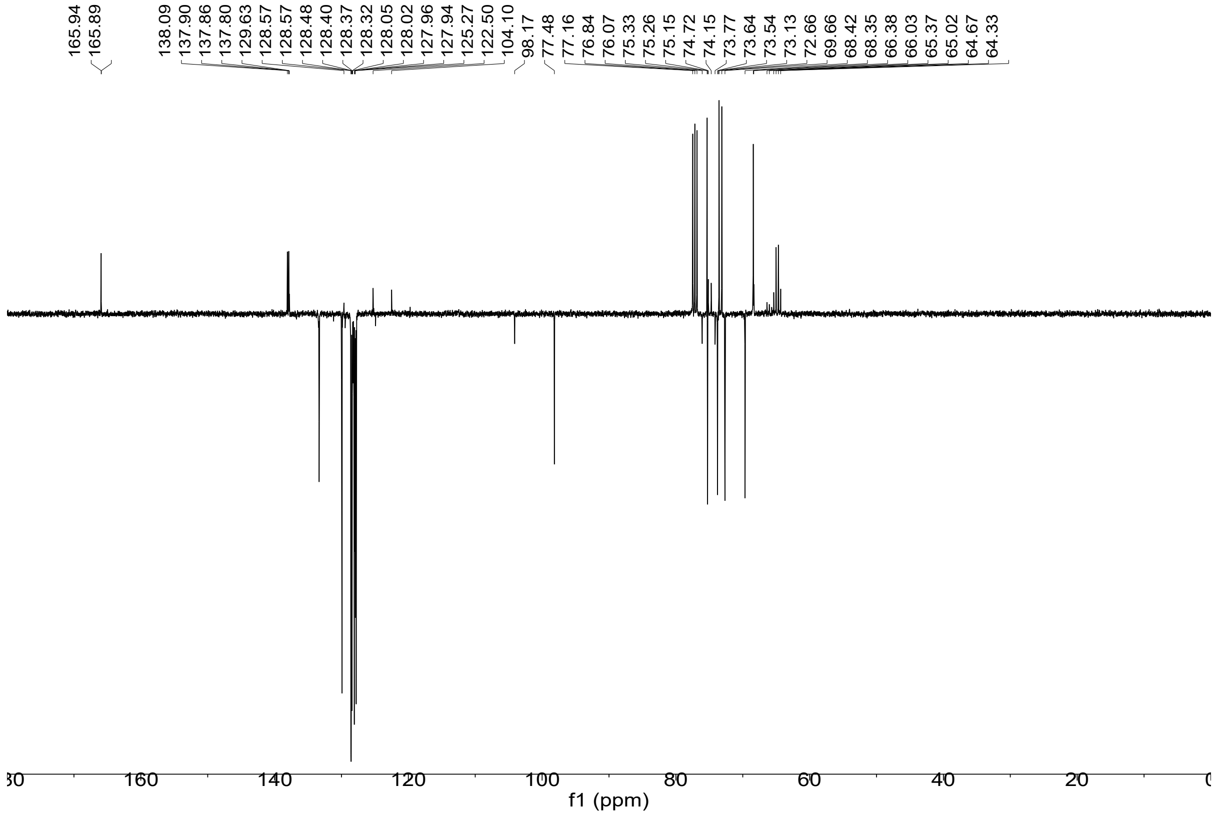

Supplementary Figure 326. ^{13}C NMR, 101 MHz, $CDCl_3$ of compound **S85**

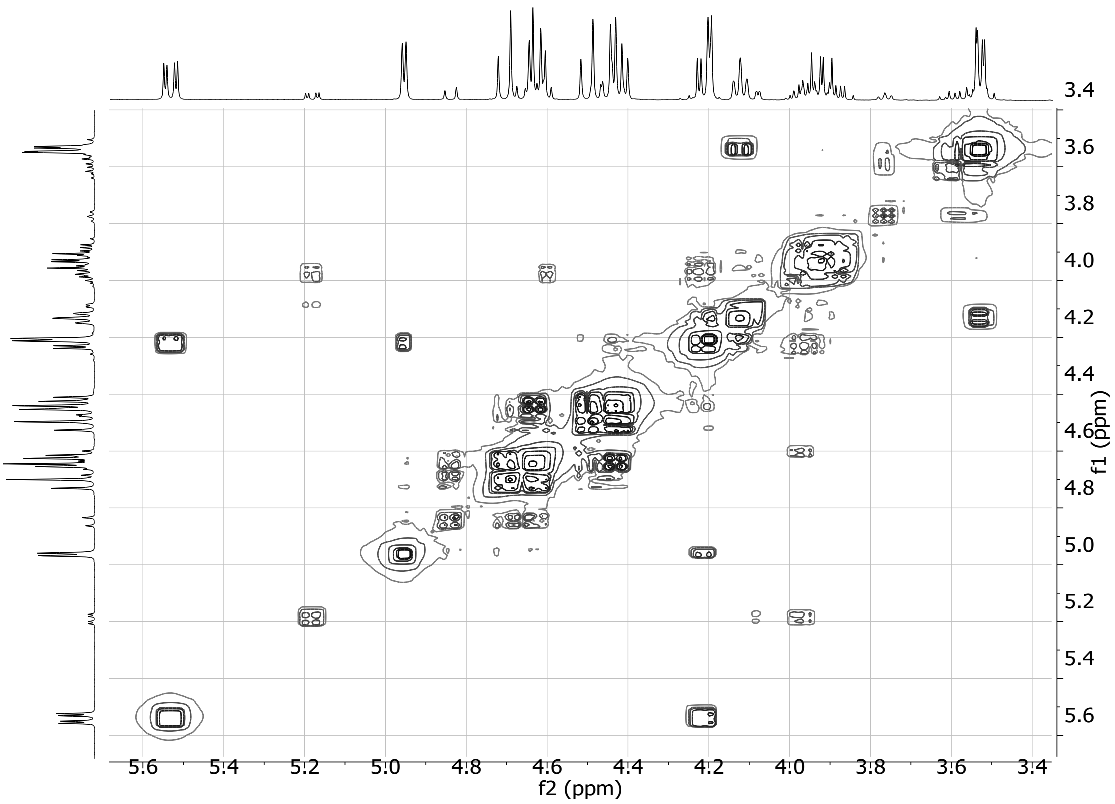

Supplementary Figure 327. HH-COSY NMR, $CDCl_3$ of compound **S85**

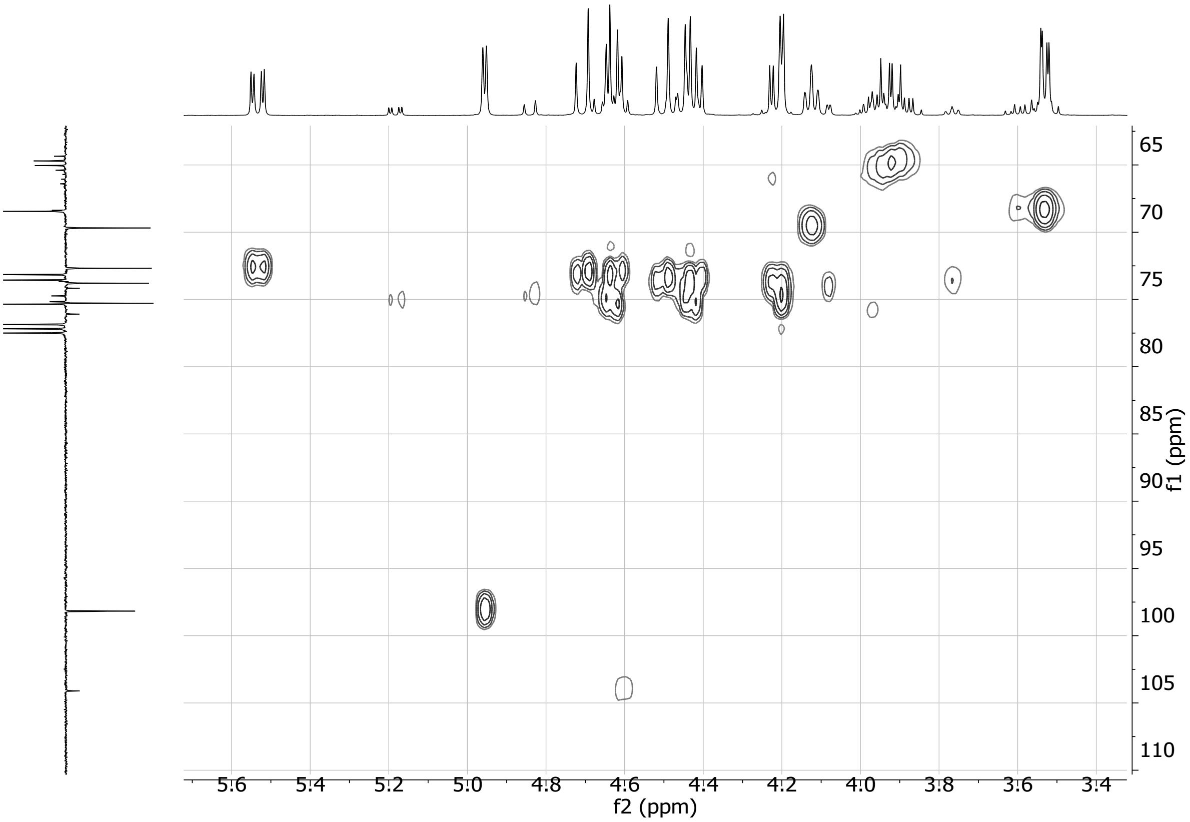

Supplementary Figure 328. HSQC NMR, $CDCl_3$ of compound **S85**

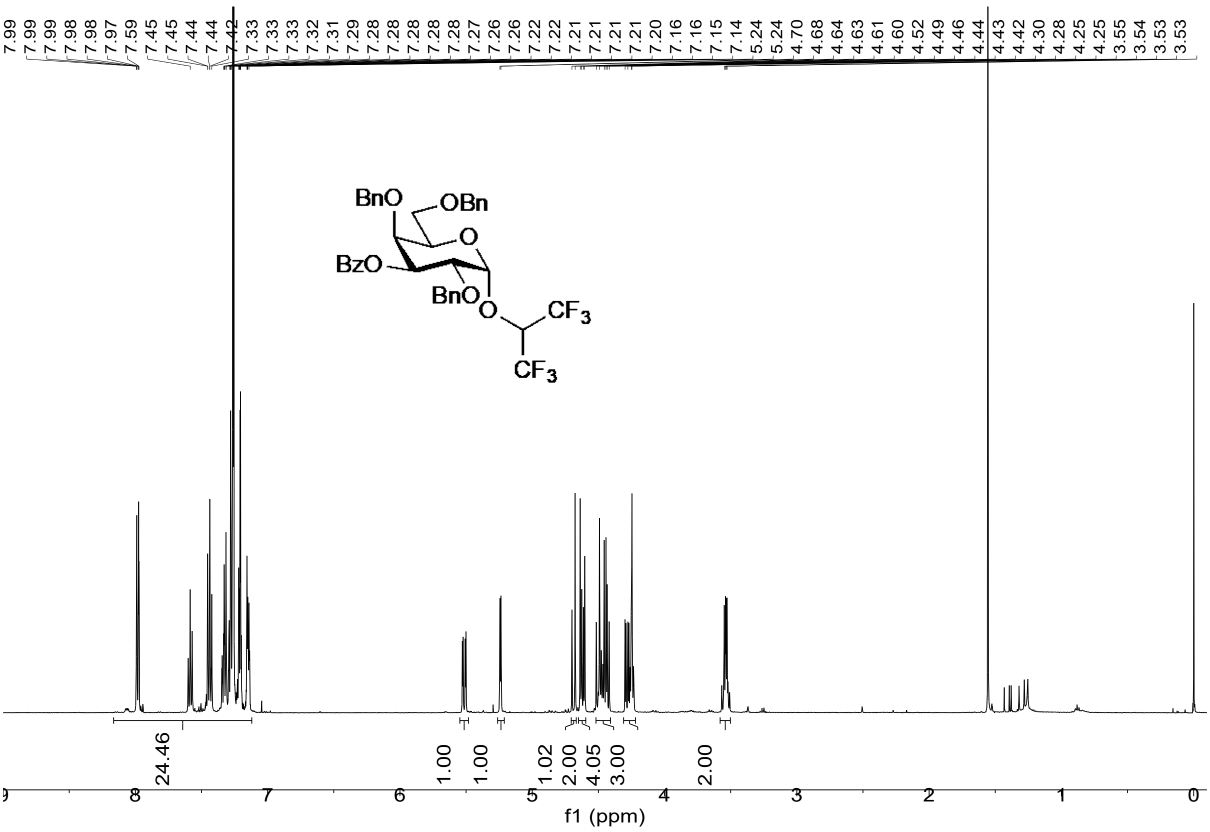

Supplementary Figure 329. ^1H NMR, 500 MHz, $CDCl_3$ of compound **S86**

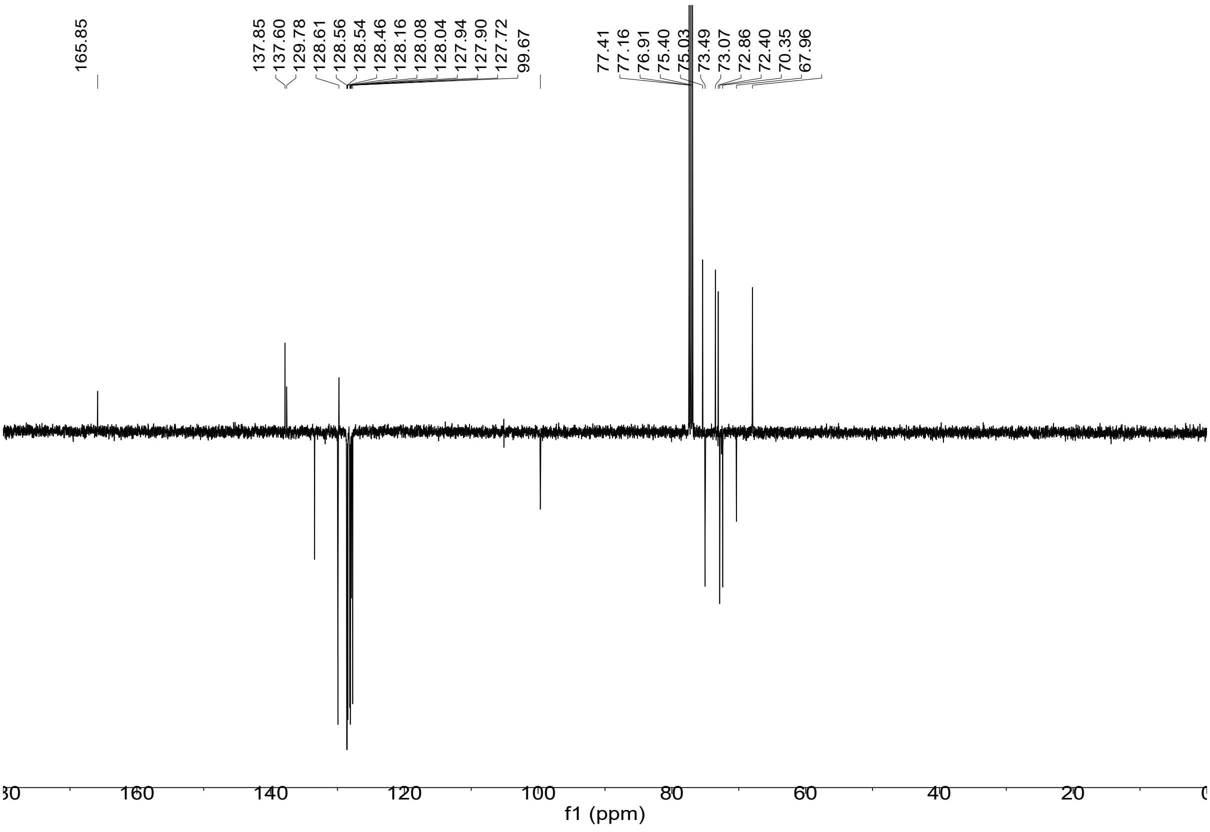

Supplementary Figure 330. ^{13}C NMR, 126 MHz, $CDCl_3$ of compound **S86**

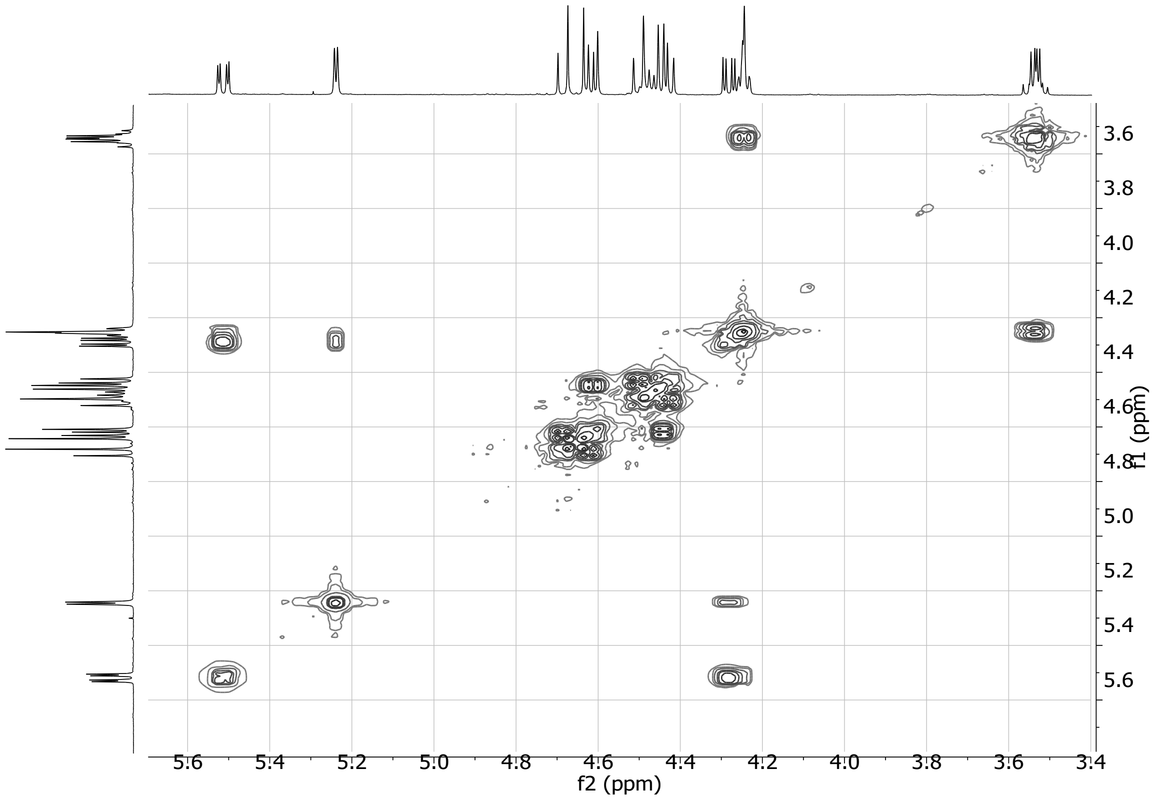

Supplementary Figure 331. HH-COSY NMR, $CDCl_3$ of compound **S86**

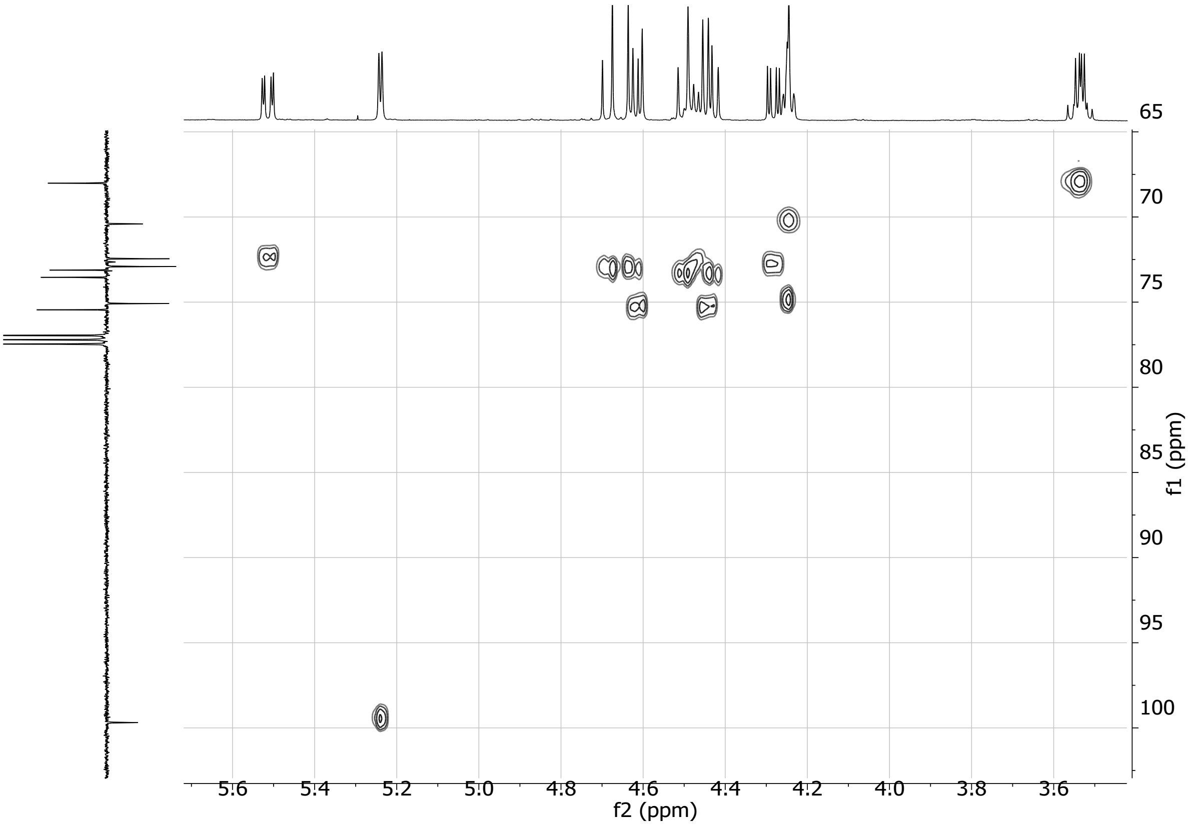

Supplementary Figure 332. HSQC NMR, $CDCl_3$ of compound **S86**

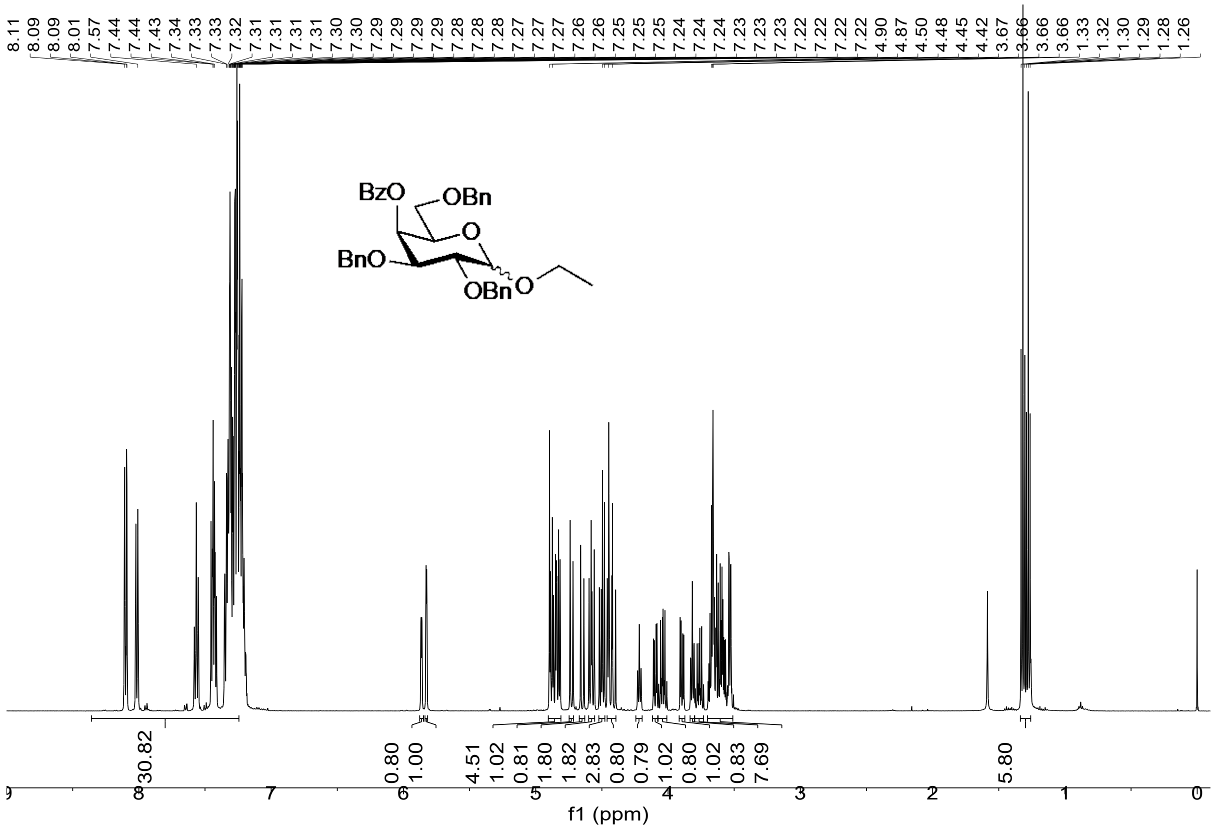

Supplementary Figure 333. ^{1}H NMR, 500 MHz, $CDCl_3$ of compound **S87**

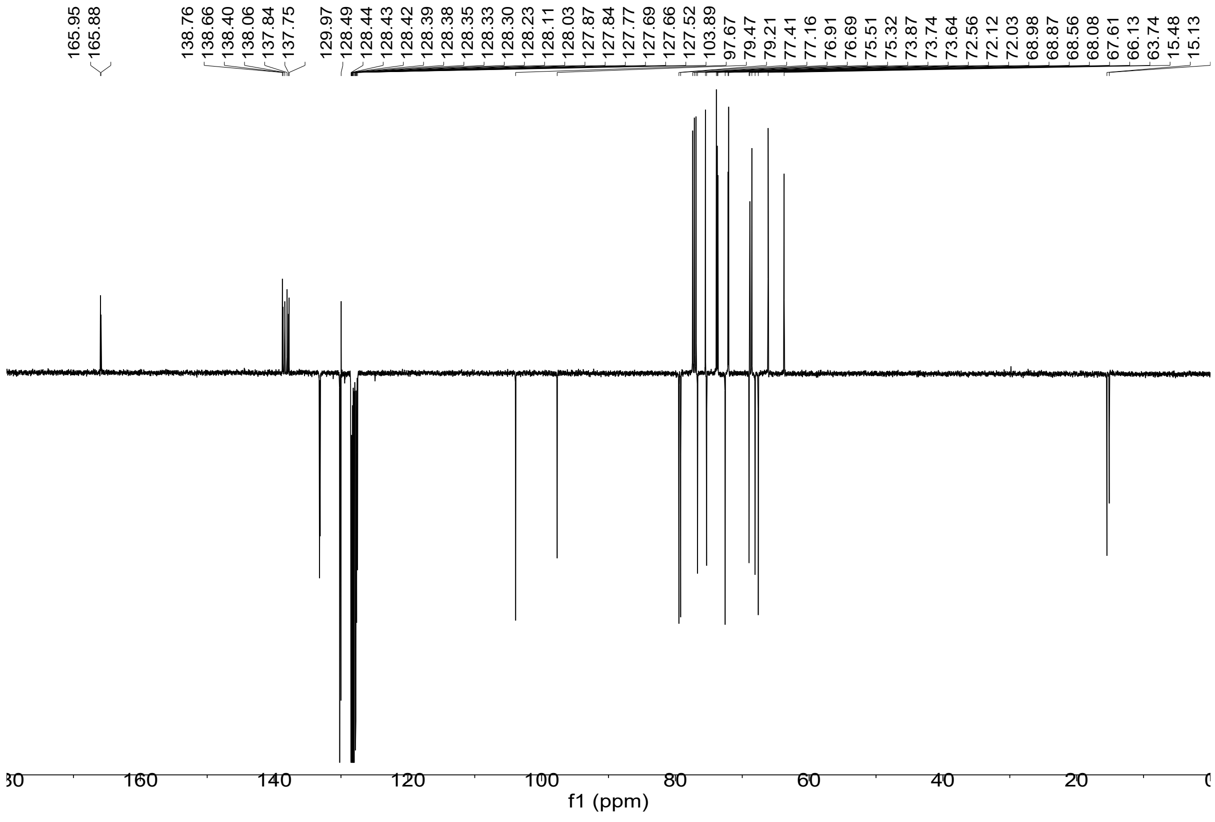

Supplementary Figure 334. ^{13}C NMR, 126 MHz, $CDCl_3$ of compound **S87**

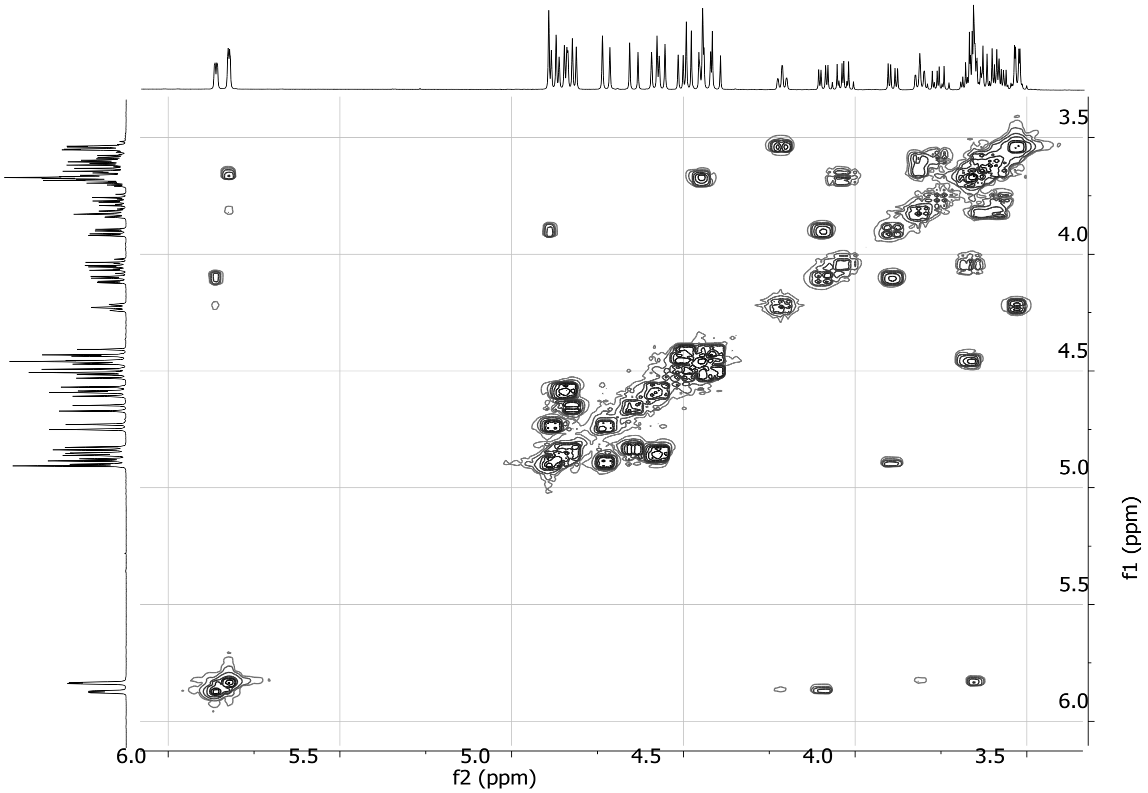

Supplementary Figure 335. HH-COSY NMR, $CDCl_3$ of compound **S87**

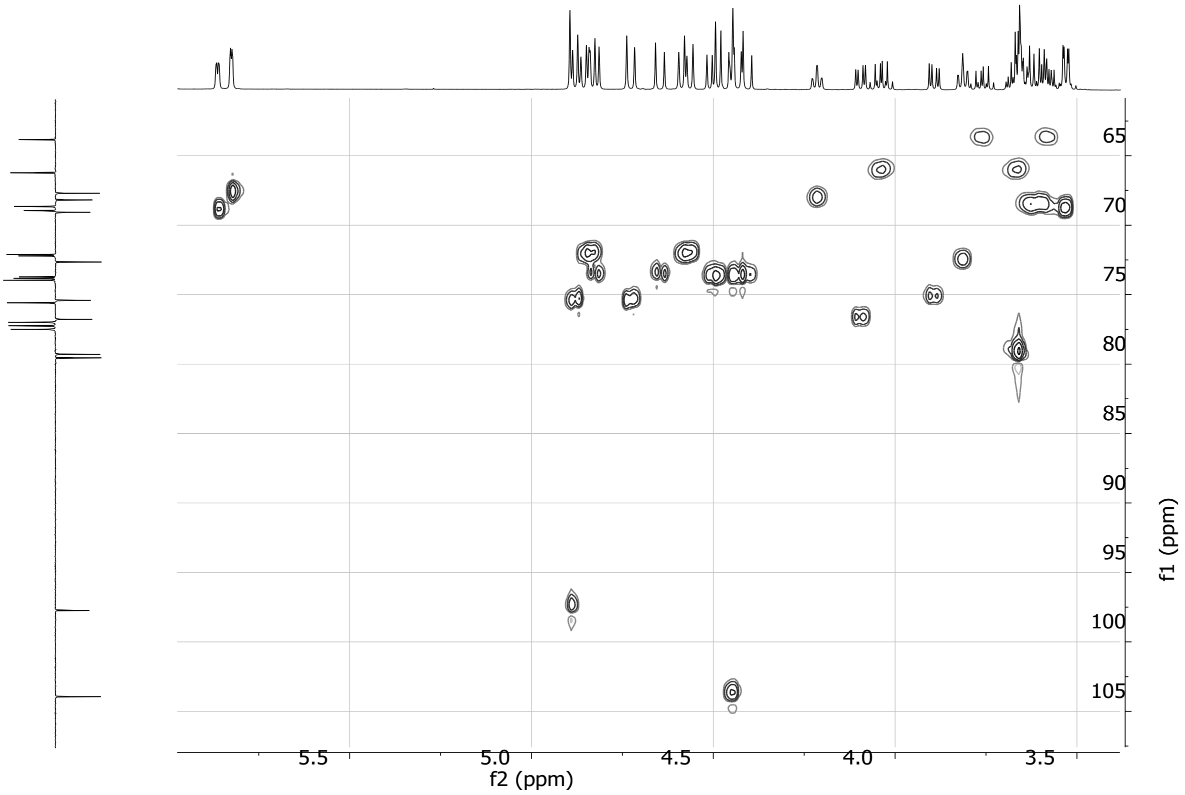

Supplementary Figure 336. HSQC NMR, $CDCl_3$ of compound **S87**

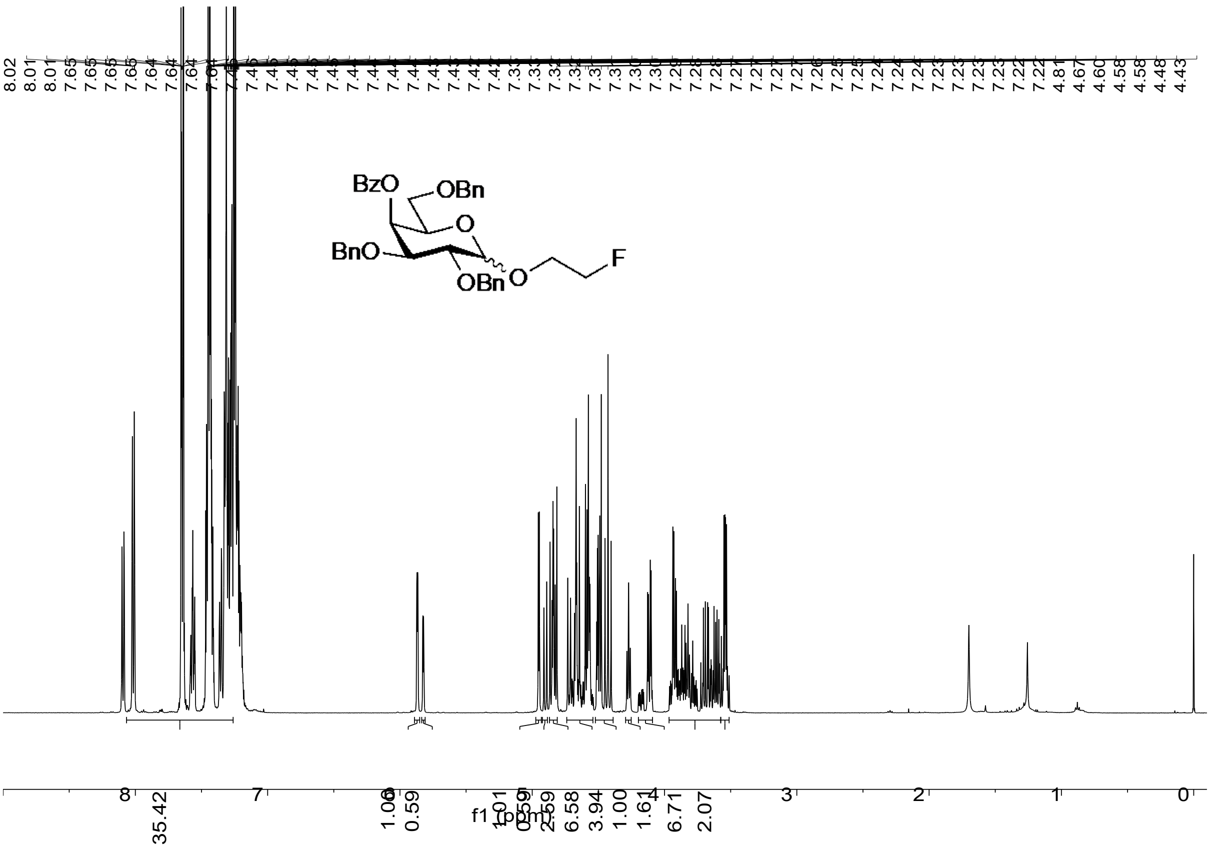

Supplementary Figure 337. ^{1}H NMR, 500 MHz, $CDCl_3$ of compound **S88**

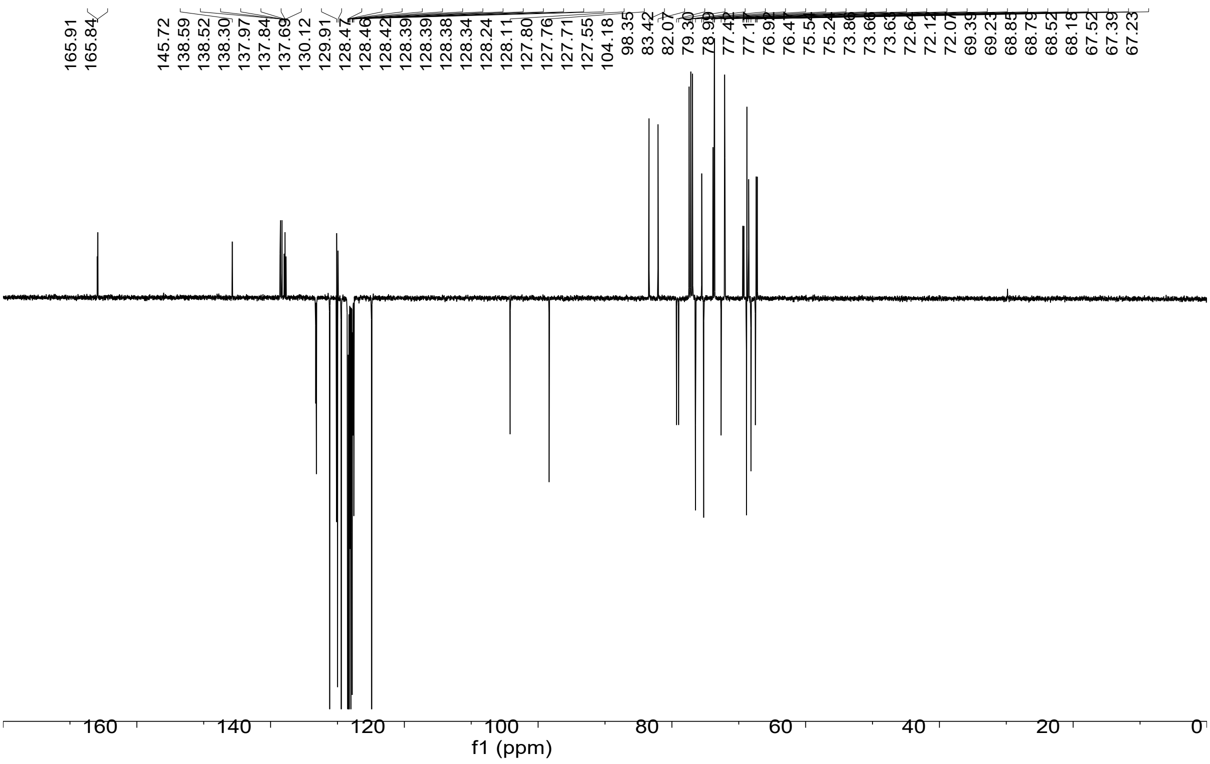

Supplementary Figure 338. ^{13}C NMR, 126 MHz, $CDCl_3$ of compound **S88**

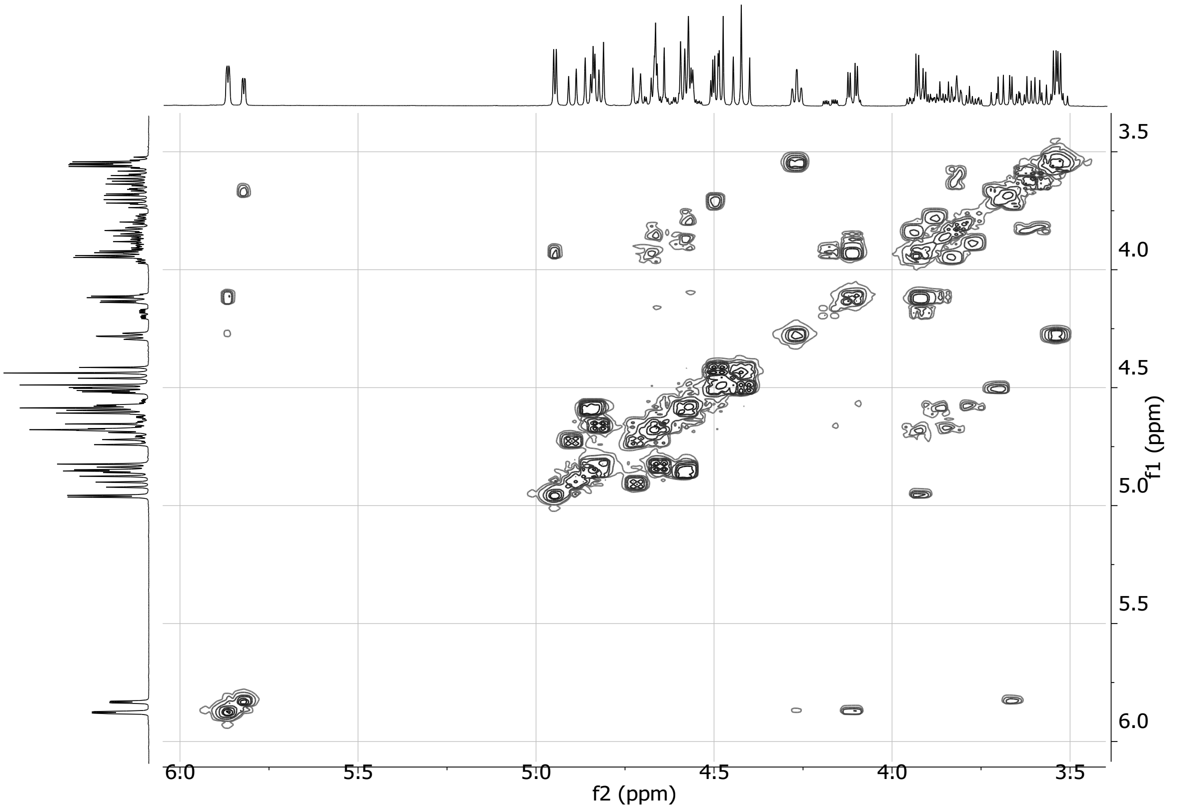

Supplementary Figure 339. HH-COSY NMR, $CDCl_3$ of compound **S88**

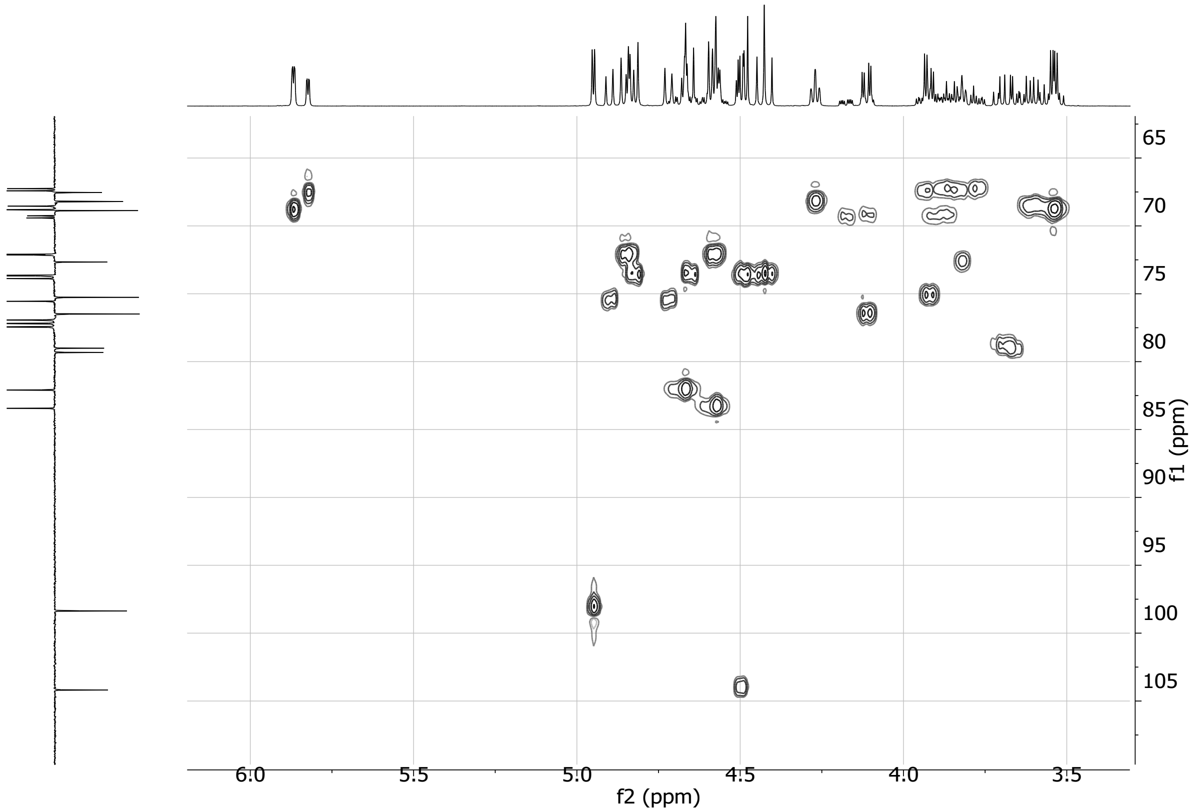

Supplementary Figure 340. HSQC NMR, $CDCl_3$ of compound **S88**

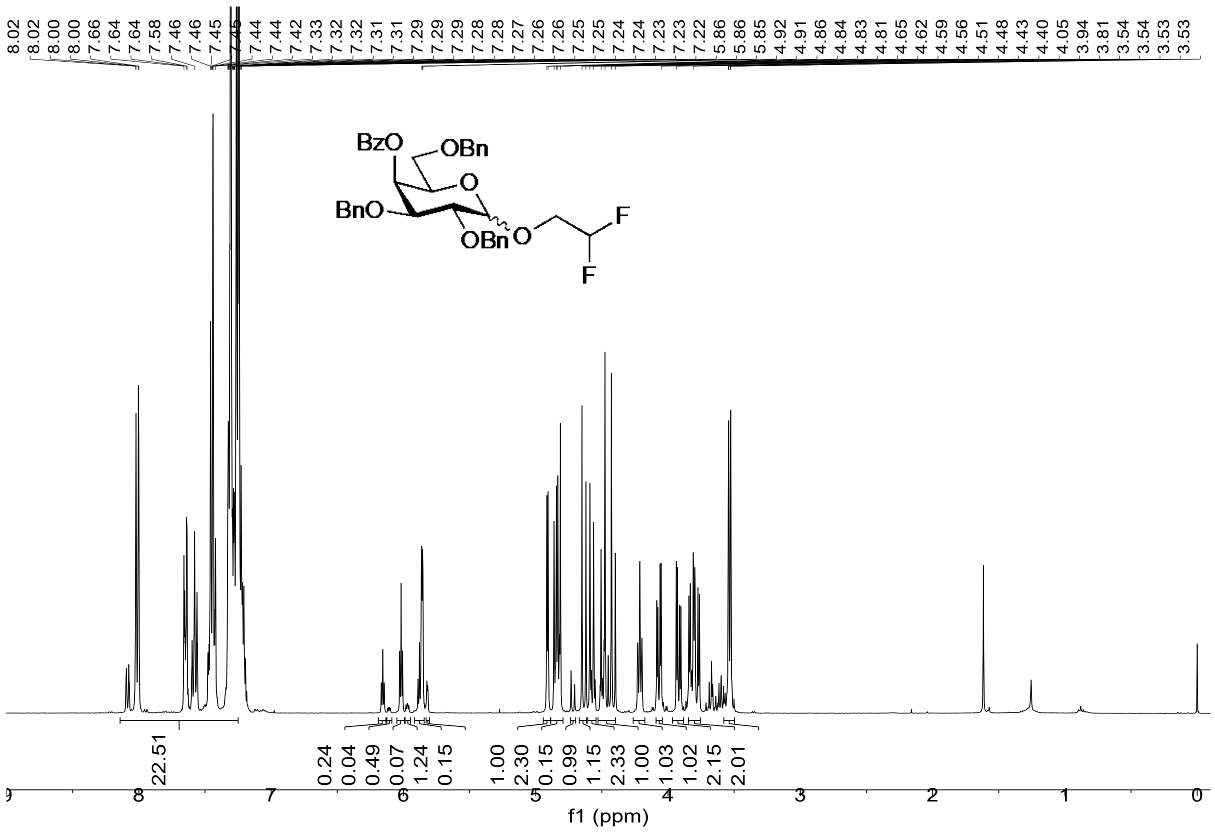

Supplementary Figure 341. ^{1}H NMR, 400 MHz, $CDCl_3$ of compound **S89**

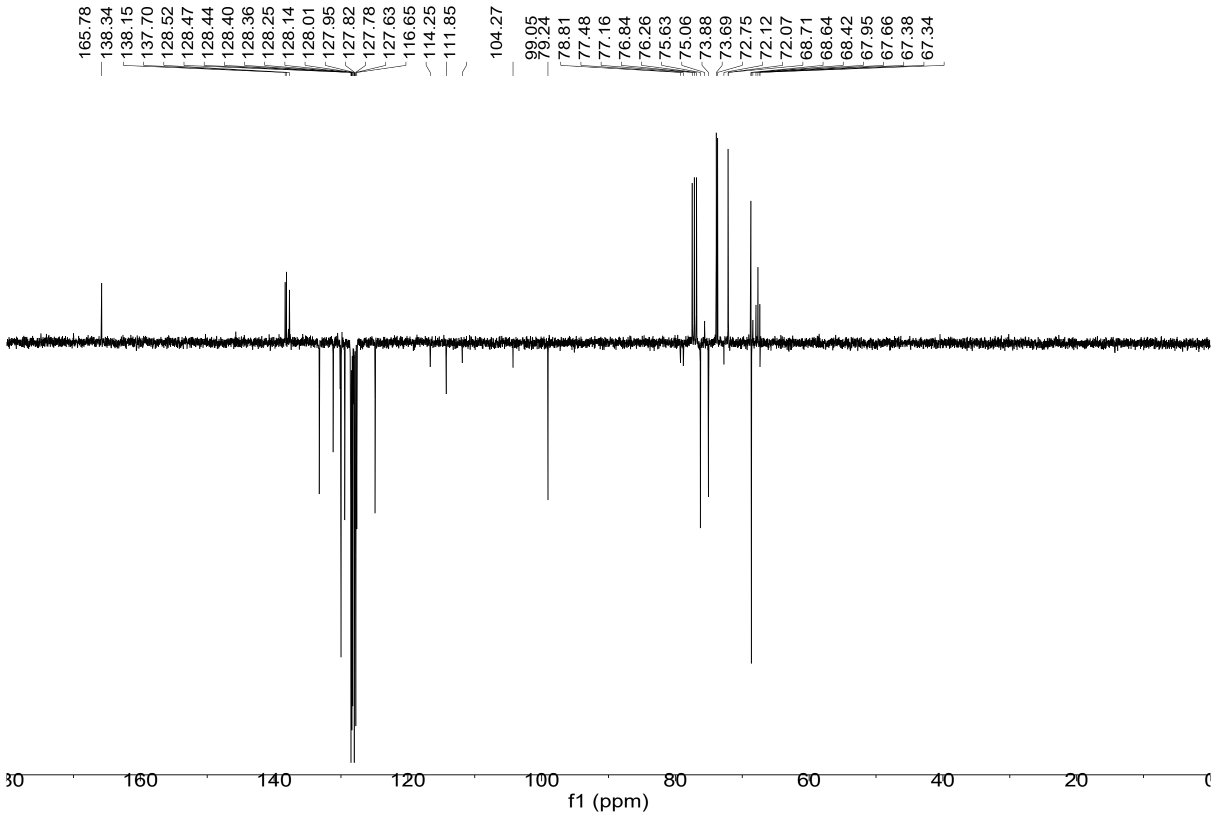

Supplementary Figure 342. ^{13}C NMR, 101 MHz, $CDCl_3$ of compound **S89**

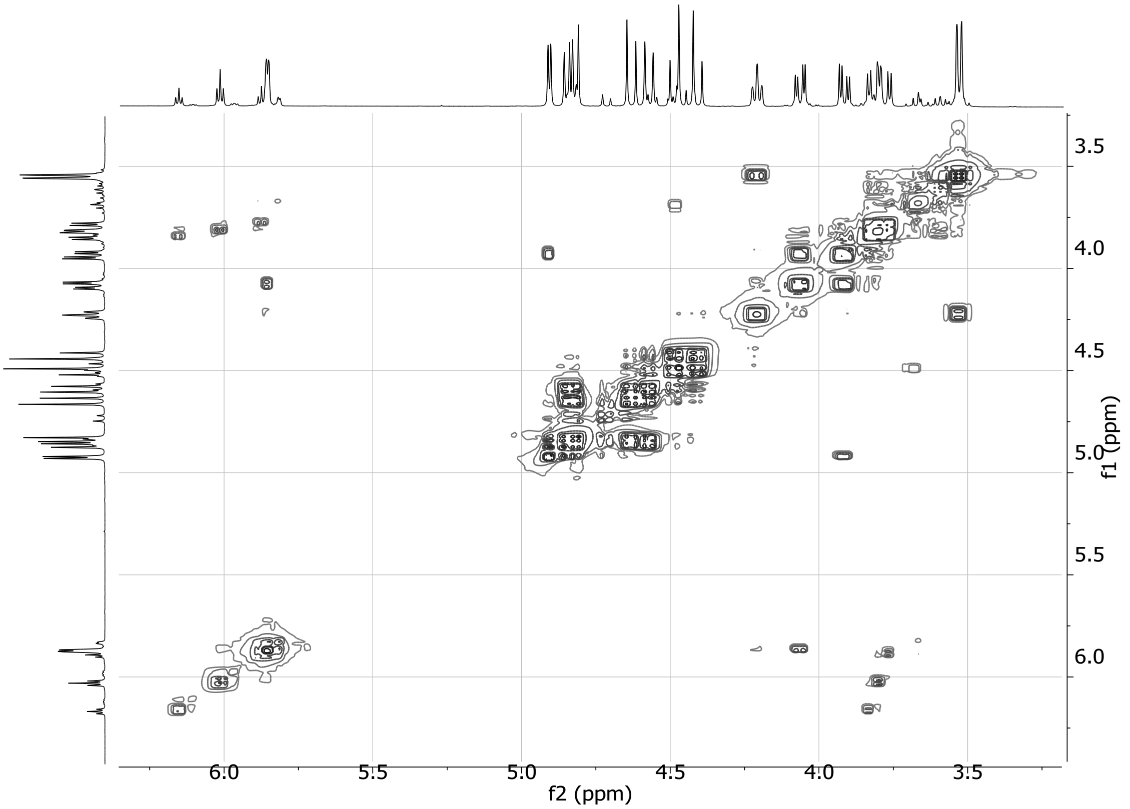

Supplementary Figure 343. HH-COSY NMR, $CDCl_3$ of compound **S89**

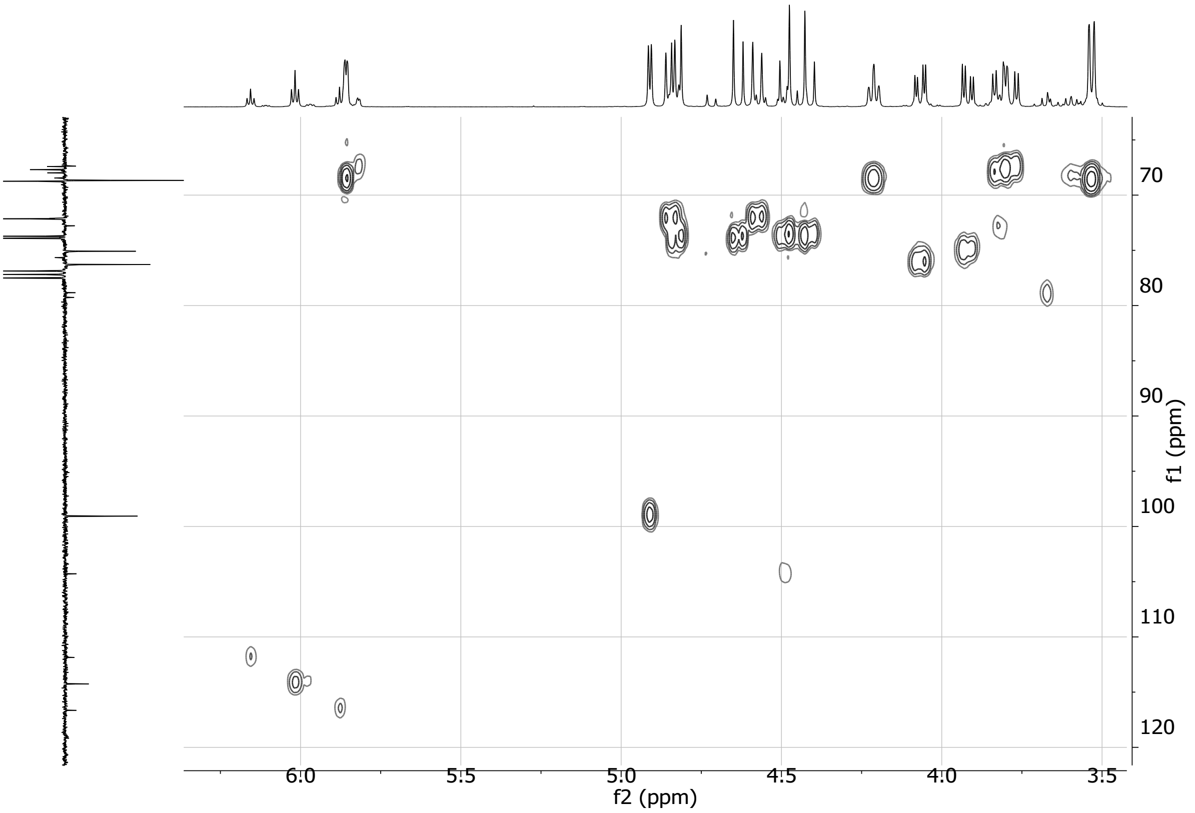

Supplementary Figure 344. HSQC NMR, $CDCl_3$ of compound **S89**

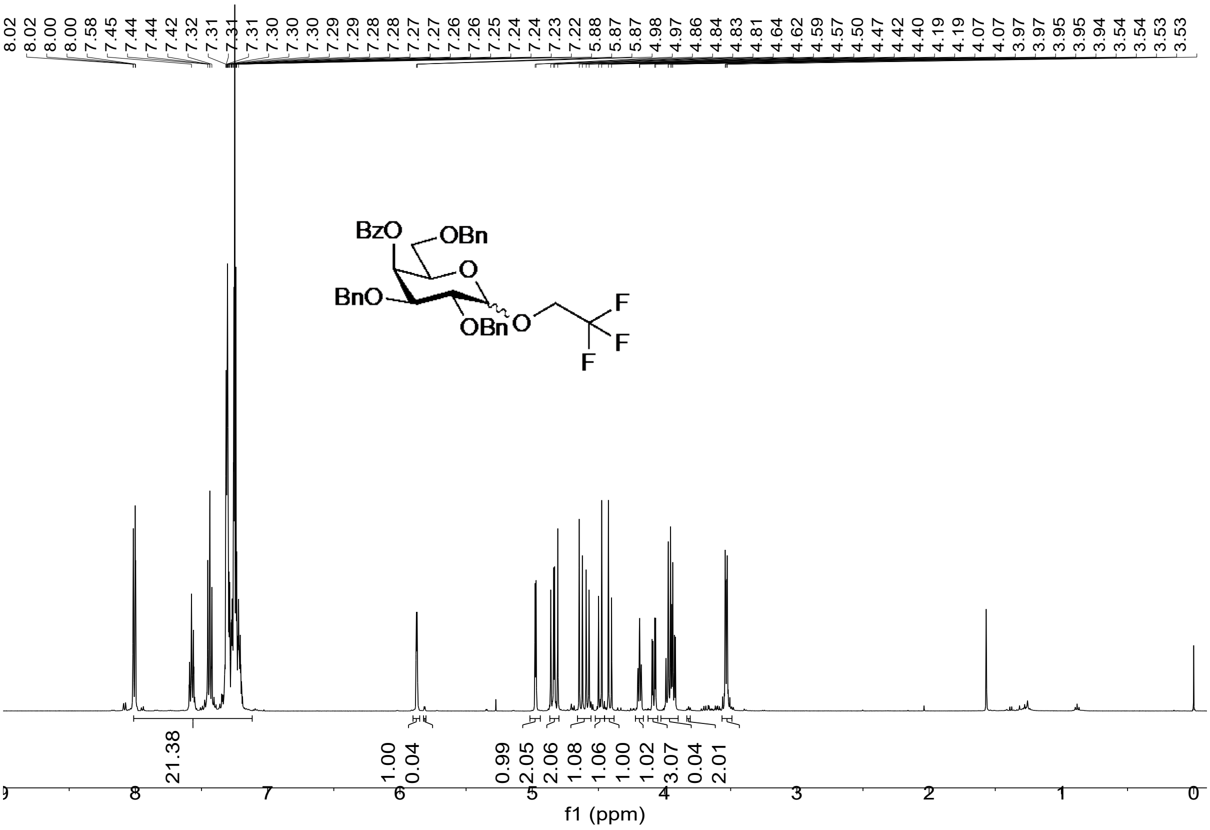

Supplementary Figure 345. ^{1}H NMR, 500 MHz, $CDCl_3$ of compound **S90**

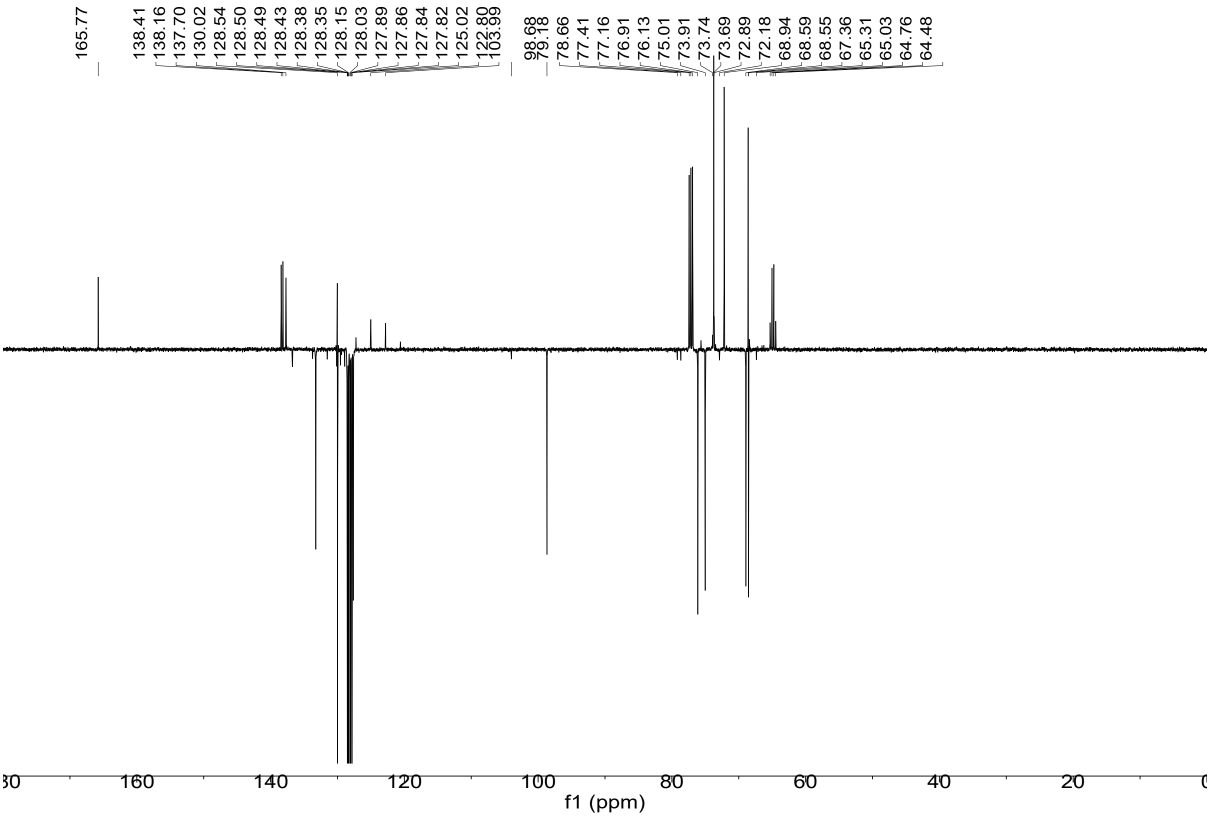

Supplementary Figure 346. ^{13}C NMR, 126 MHz, $CDCl_3$ of compound **S90**

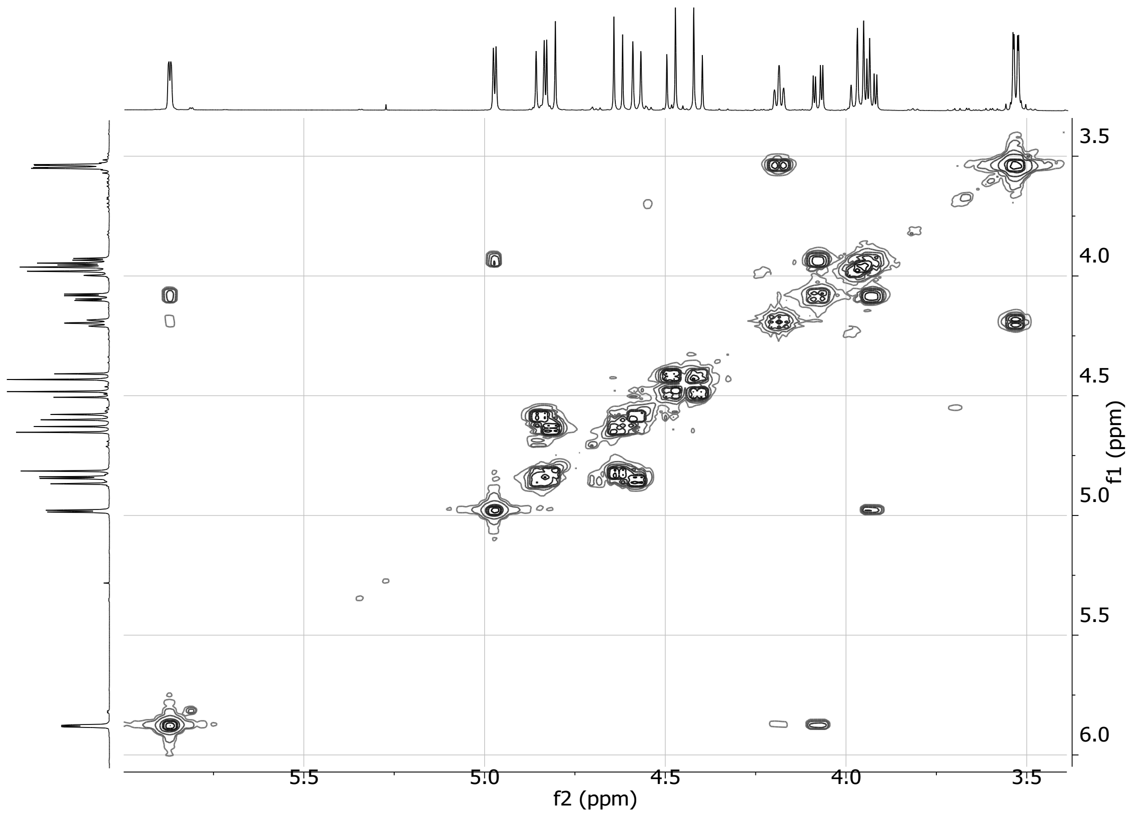

Supplementary Figure 347. HH-COSY NMR, $CDCl_3$ of compound **S90**

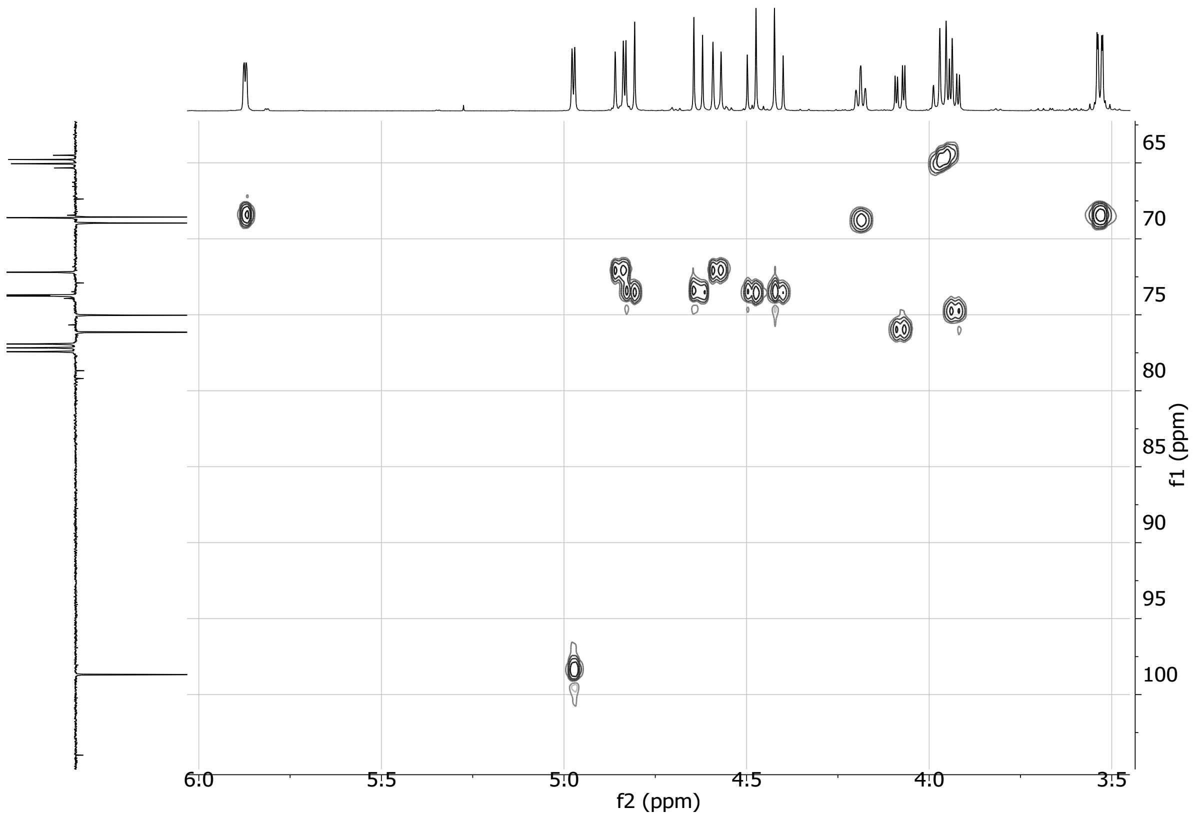

Supplementary Figure 348. HSQC NMR, $CDCl_3$ of compound **S90**

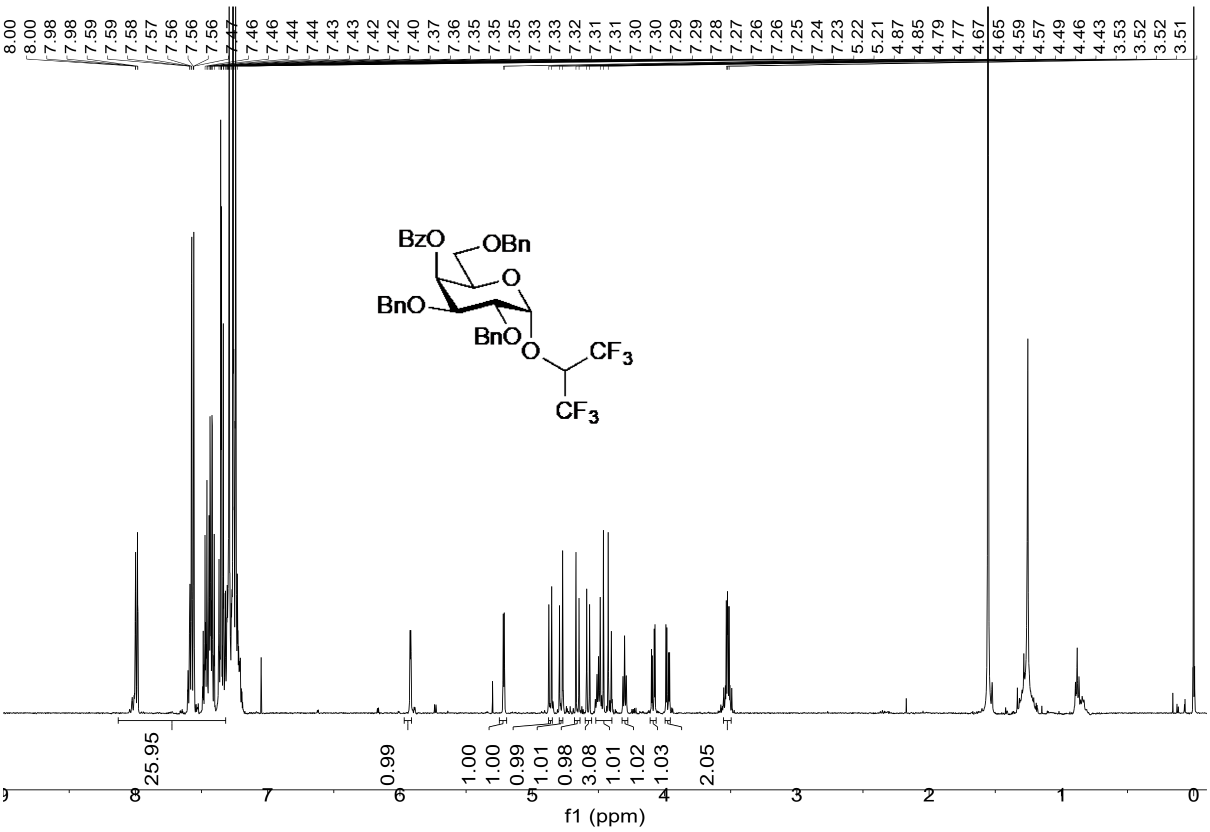

Supplementary Figure 349. ^{1}H NMR, 500 MHz, $CDCl_3$ of compound **S91**

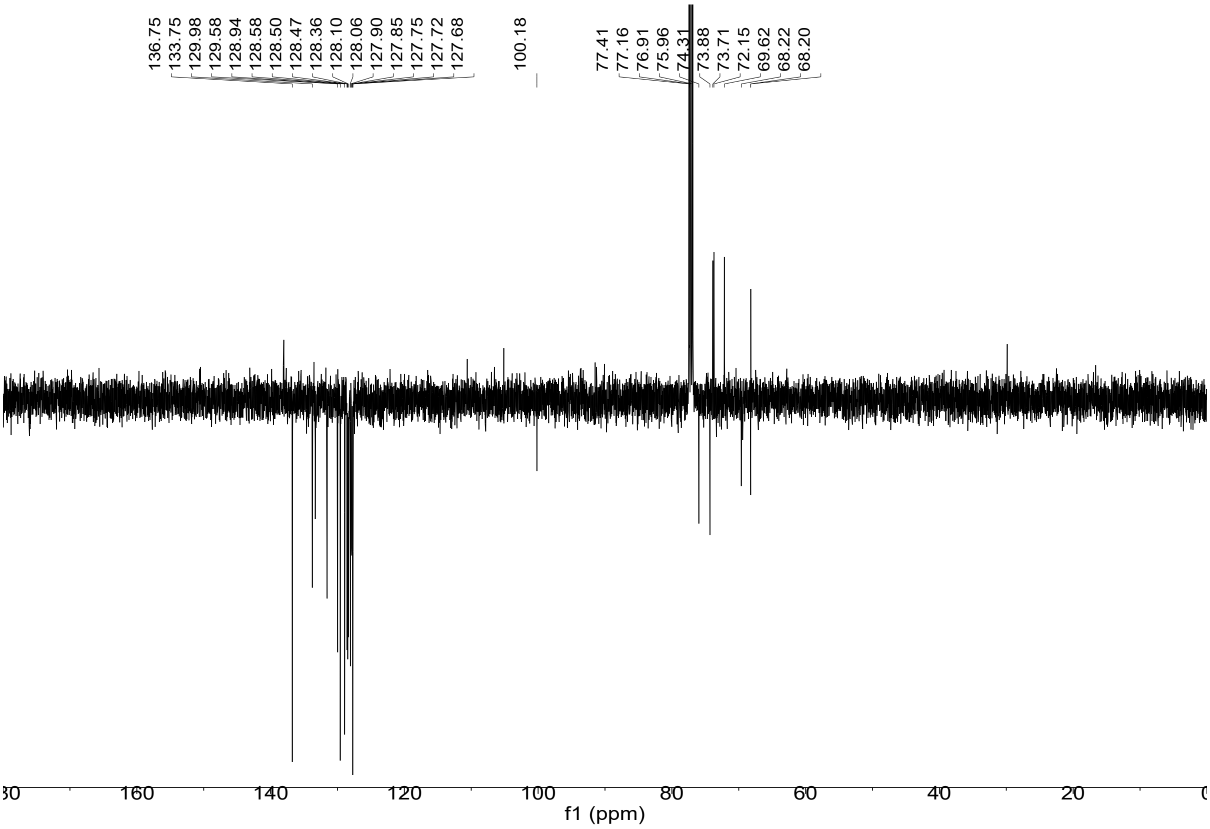

Supplementary Figure 350. ^{13}C NMR, 126 MHz, $CDCl_3$ of compound **S91**

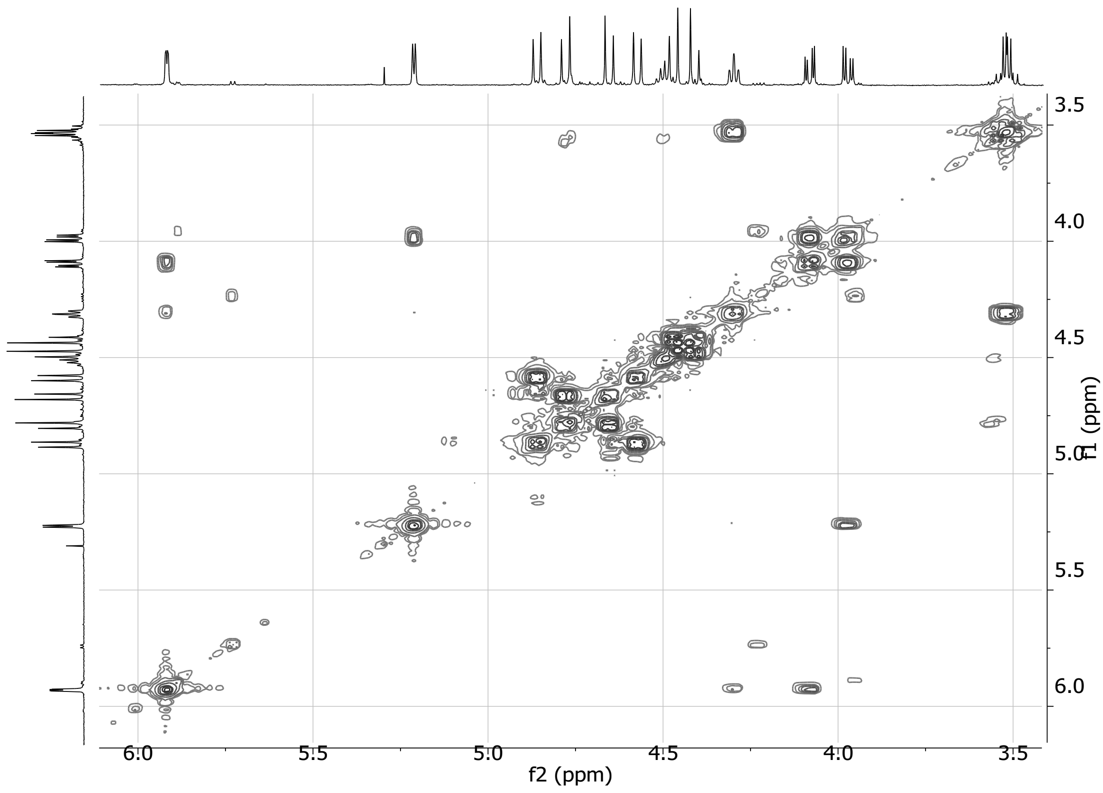

Supplementary Figure 351. HH-COSY NMR, $CDCl_3$ of compound **S91**

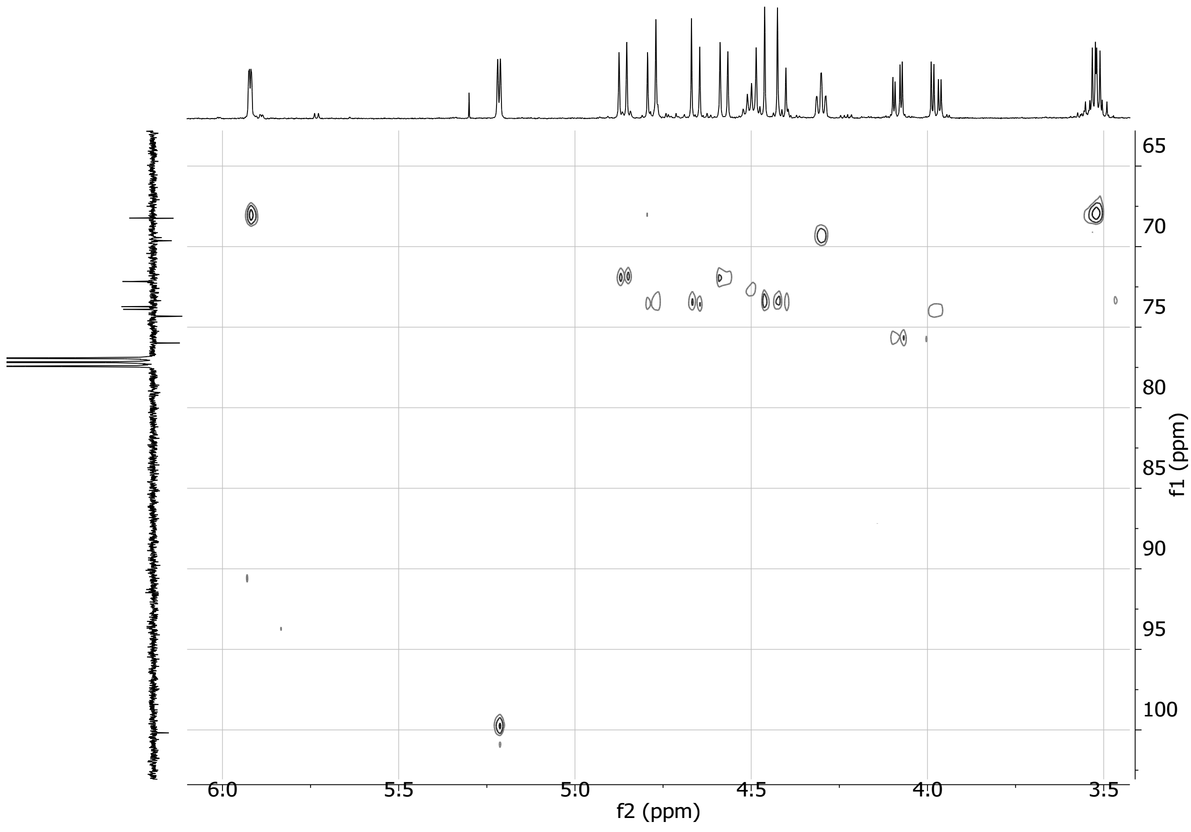

Supplementary Figure 352. HSQC NMR, $CDCl_3$ of compound **S91**

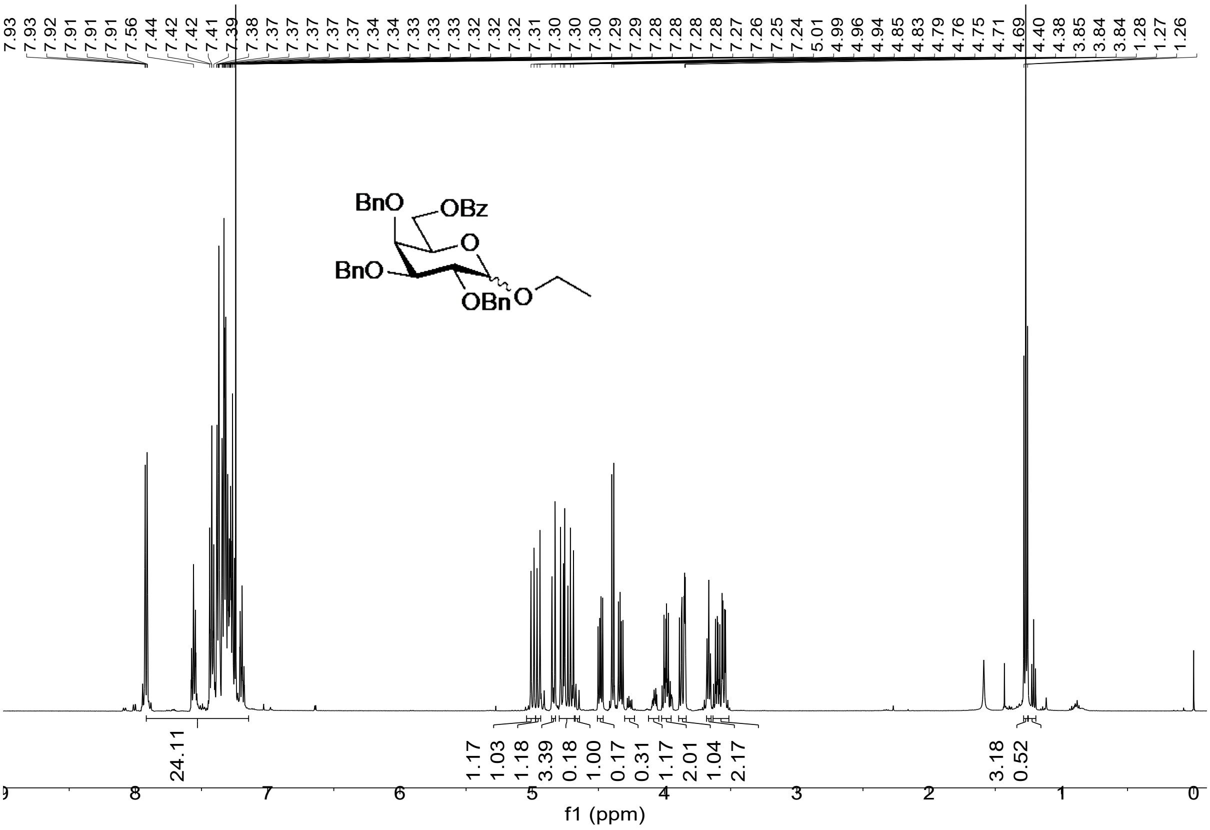

Supplementary Figure 353. ^{1}H NMR, 500 MHz, $CDCl_3$ of compound **S92**

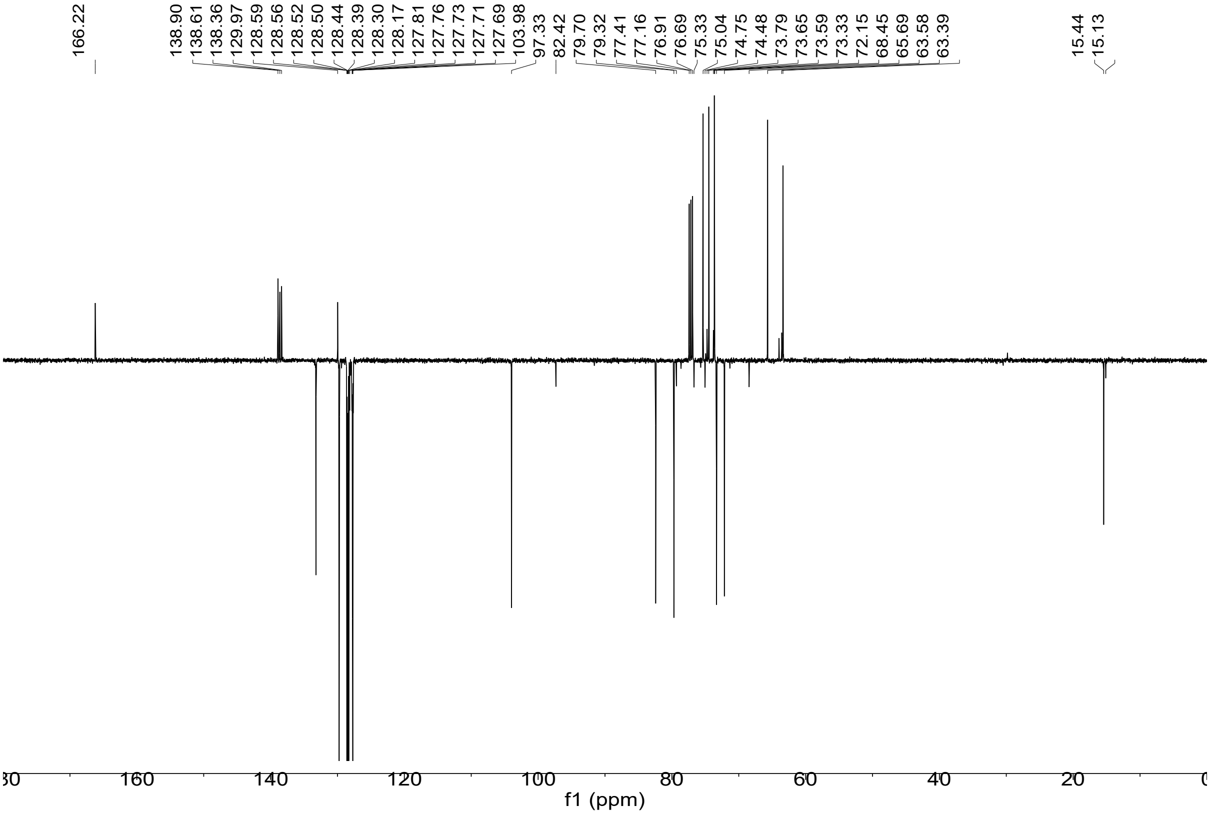

Supplementary Figure 354. ^{13}C NMR, 126 MHz, $CDCl_3$ of compound **S92**

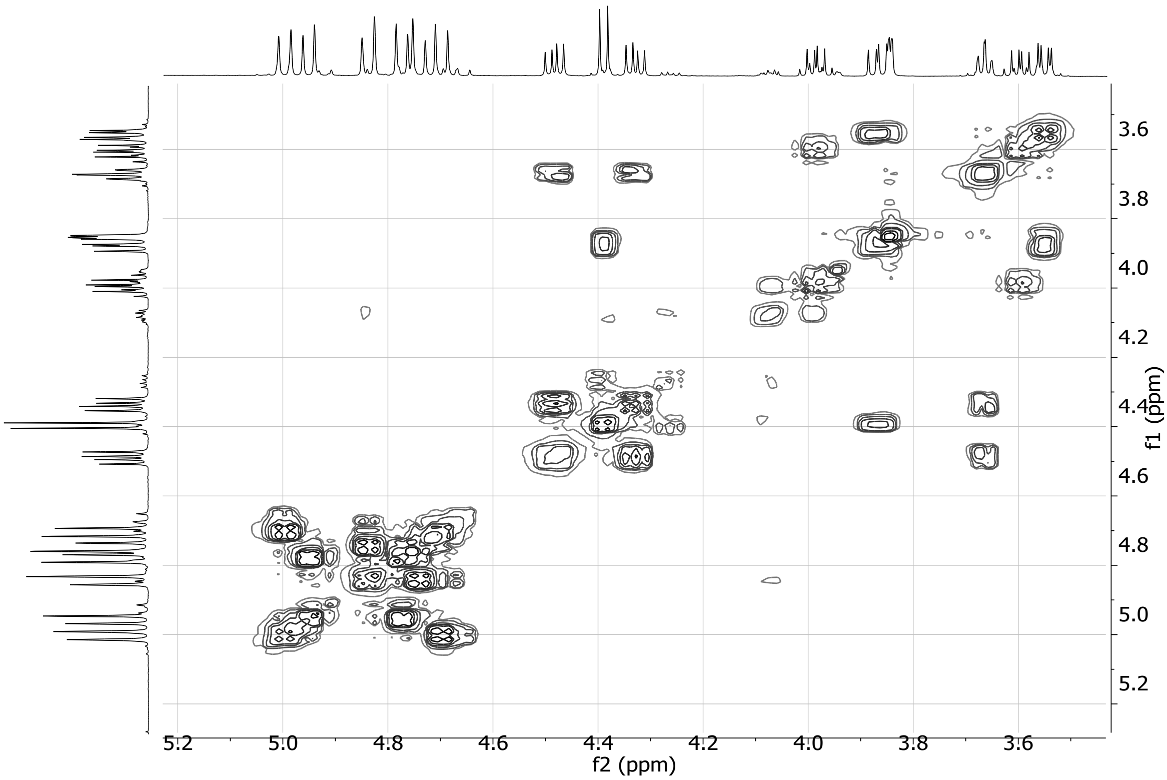

Supplementary Figure 355. HH-COSY NMR, $CDCl_3$ of compound **S92**

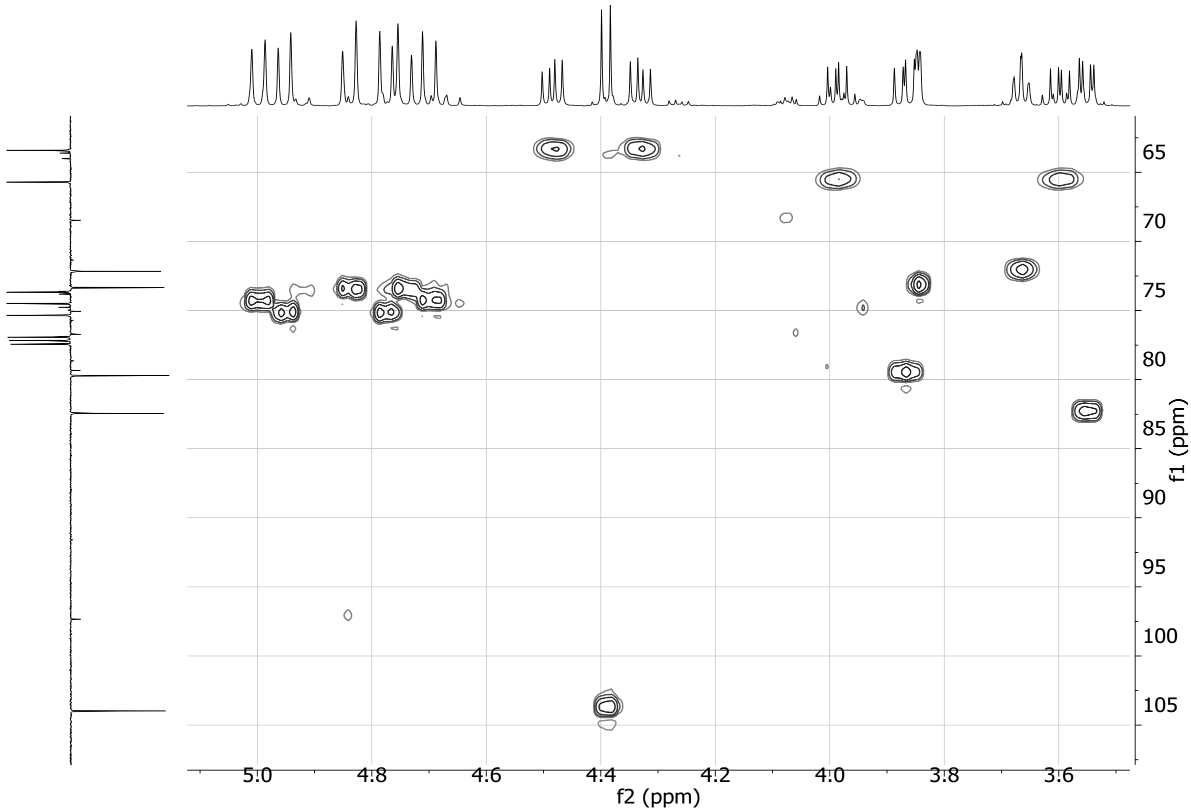

Supplementary Figure 356. HSQC NMR, $CDCl_3$ of compound **S92**

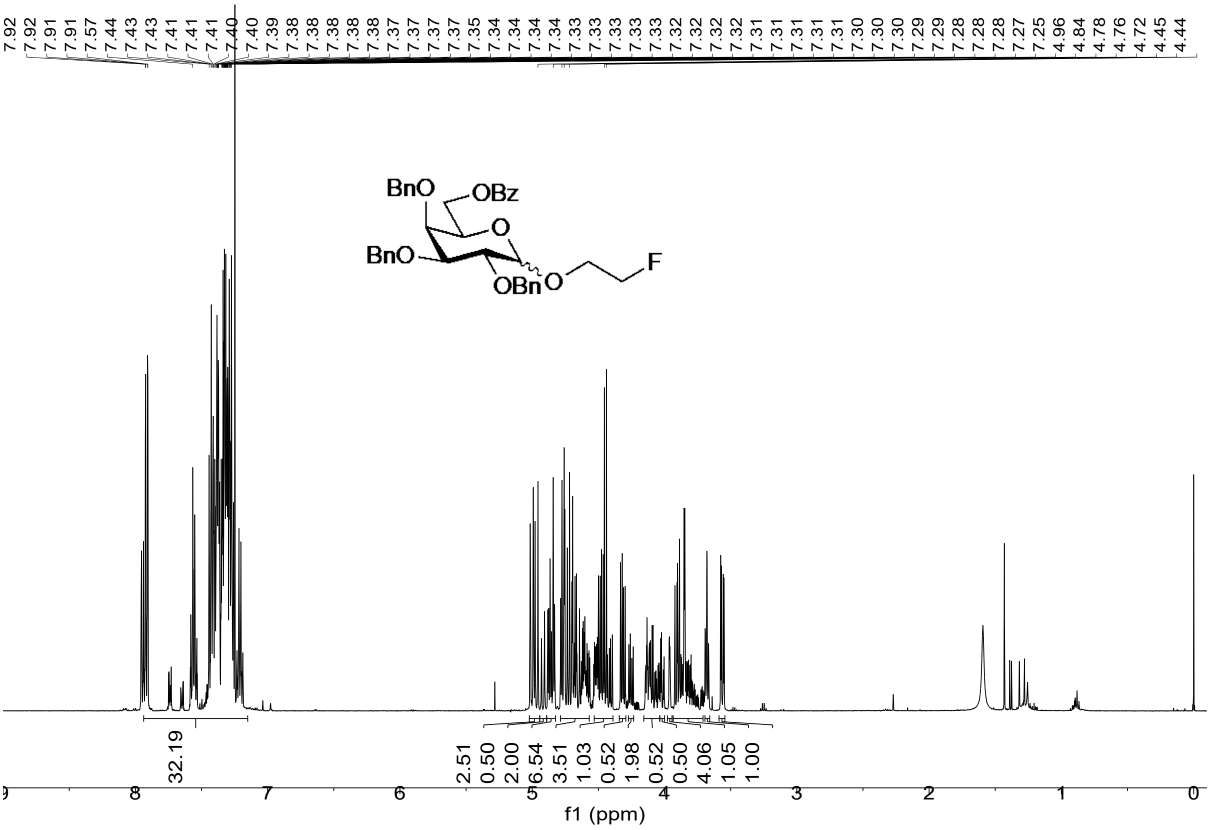

Supplementary Figure 357. ^{1}H NMR, 500 MHz, $CDCl_3$ of compound **S93**

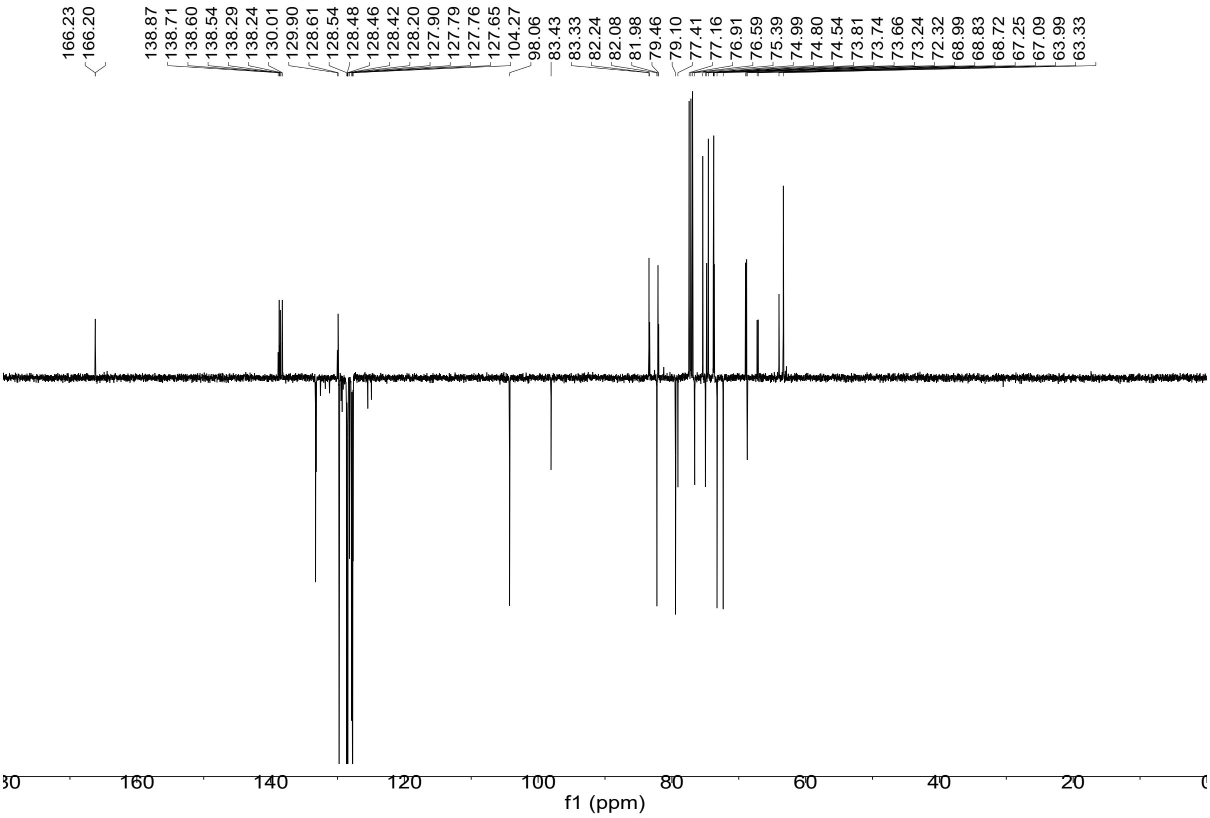

Supplementary Figure 358. ^{13}C NMR, 126 MHz, $CDCl_3$ of compound **S93**

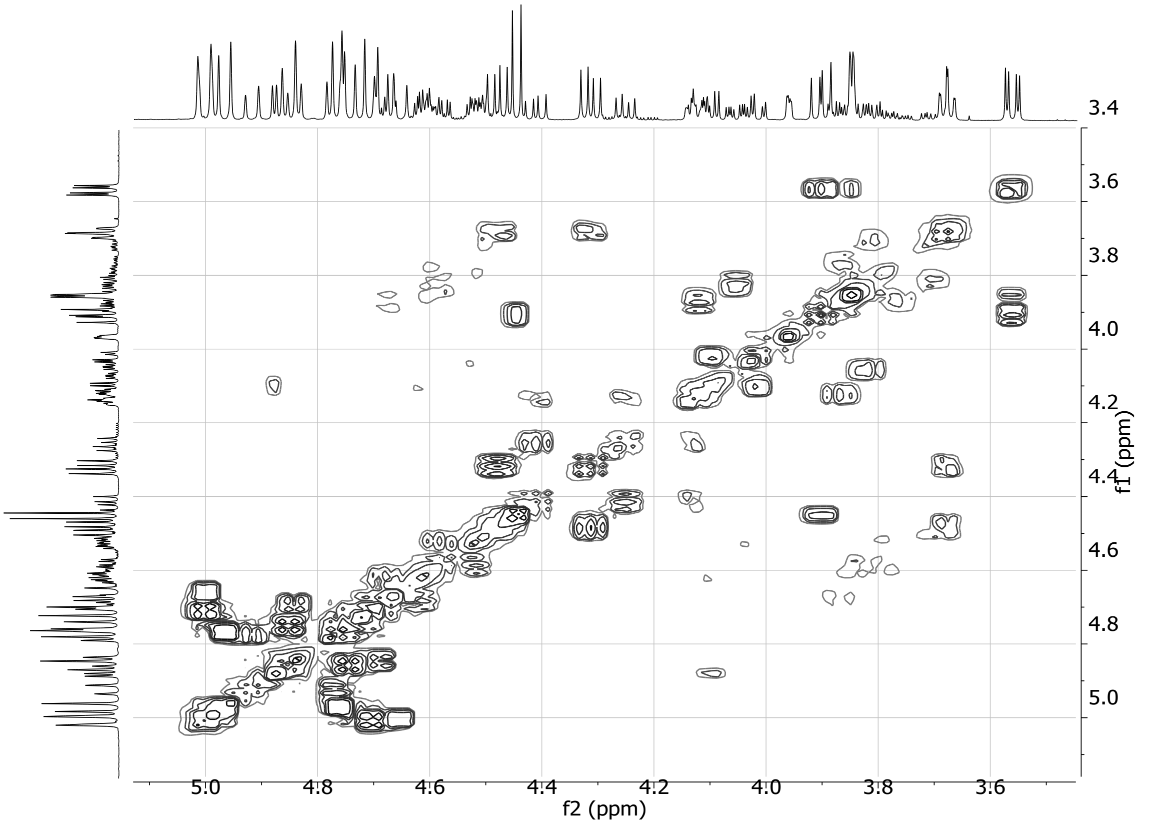

Supplementary Figure 359. HH-COSY NMR, $CDCl_3$ of compound **S93**

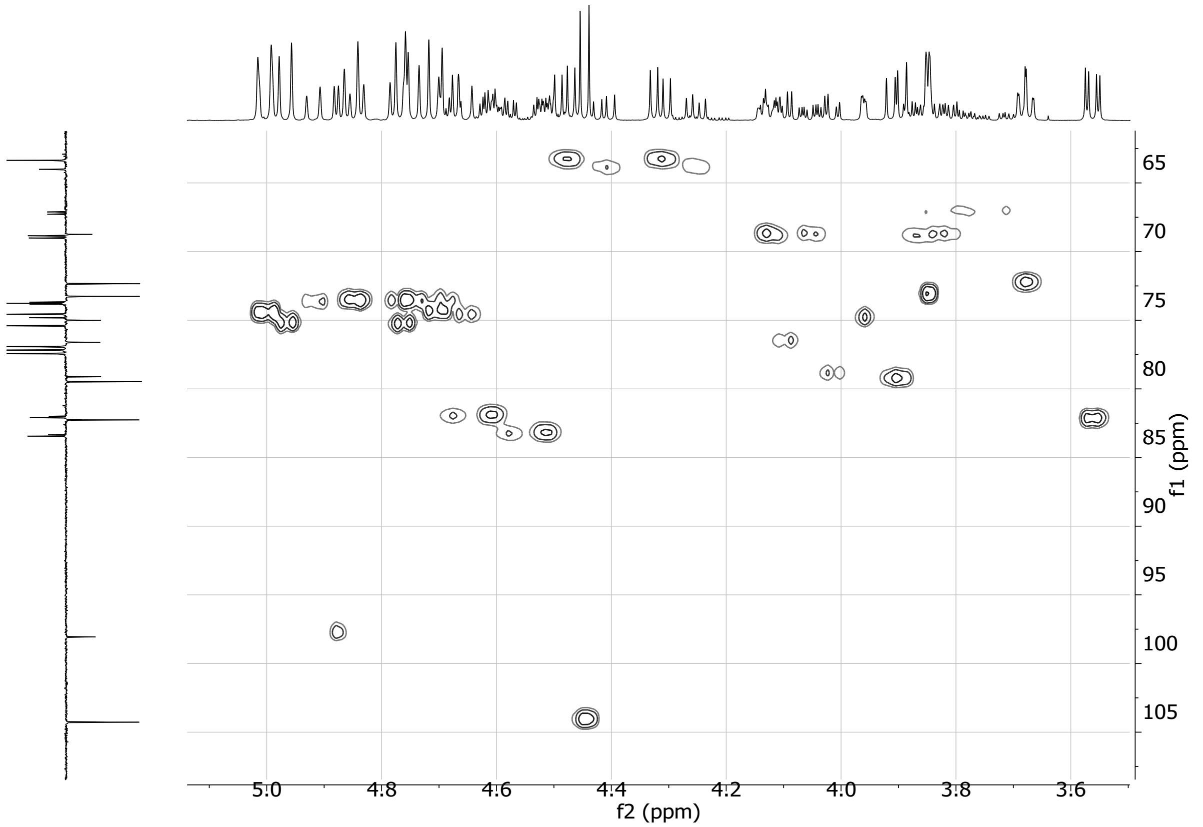

Supplementary Figure 360. HSQC NMR, $CDCl_3$ of compound **S93**

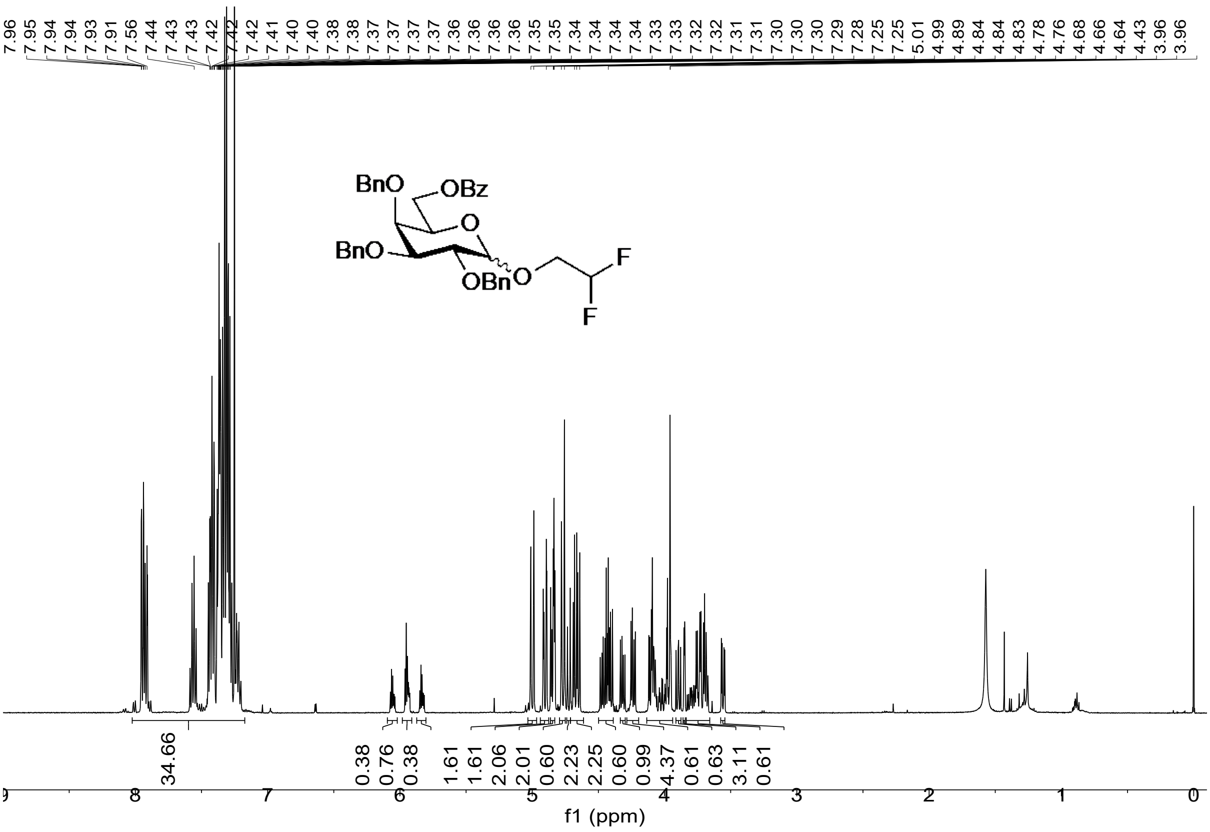

Supplementary Figure 361. ^{1}H NMR, 500 MHz, $CDCl_3$ of compound **S94**

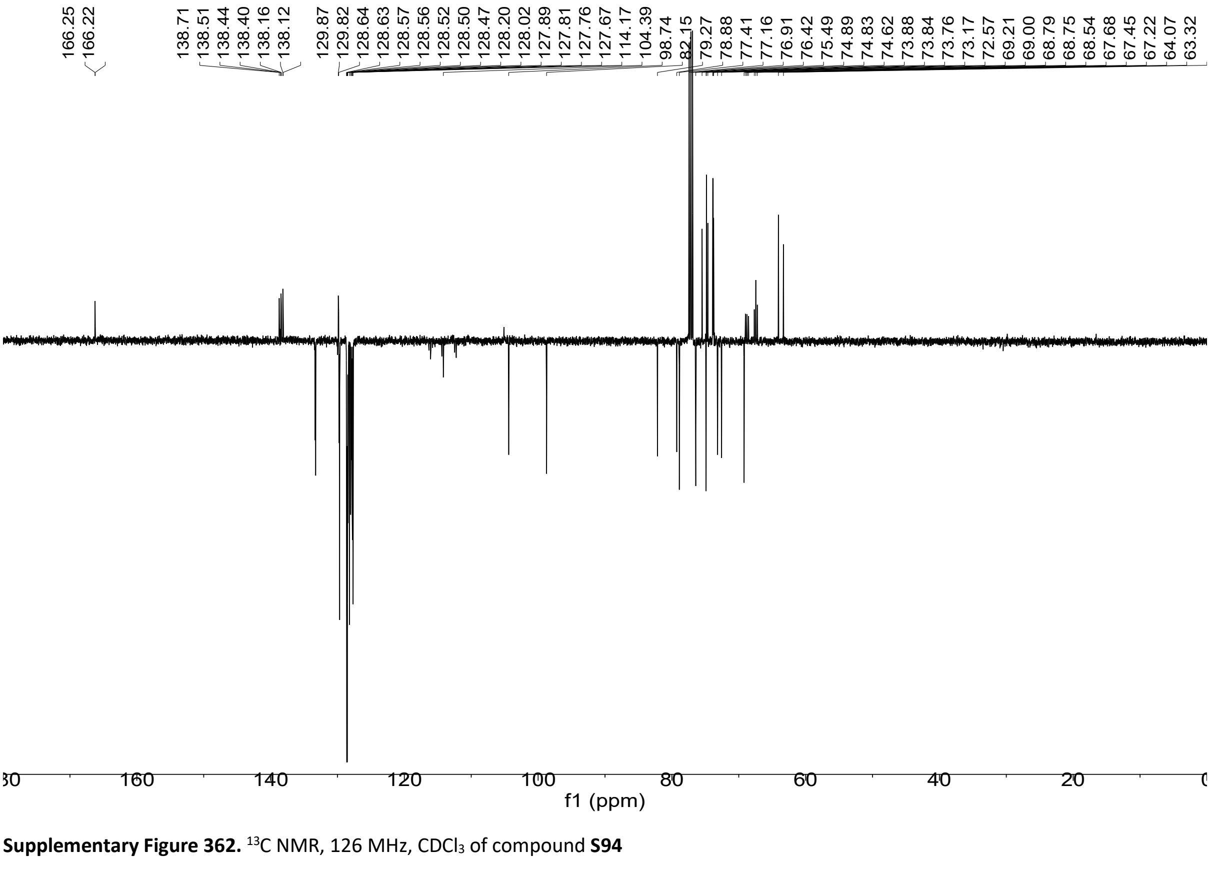

Supplementary Figure 362. ^{13}C NMR, 126 MHz, $CDCl_3$ of compound **S94**

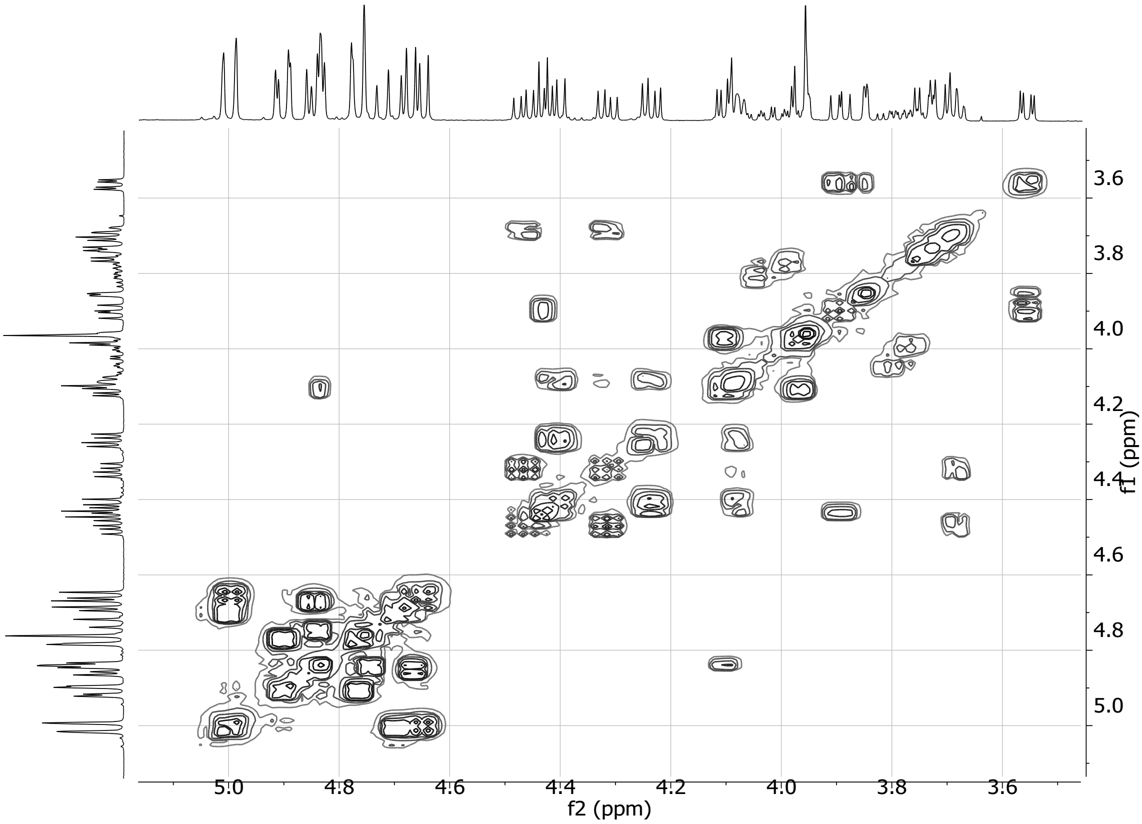

Supplementary Figure 363. HH-COSY NMR, $CDCl_3$ of compound **S94**

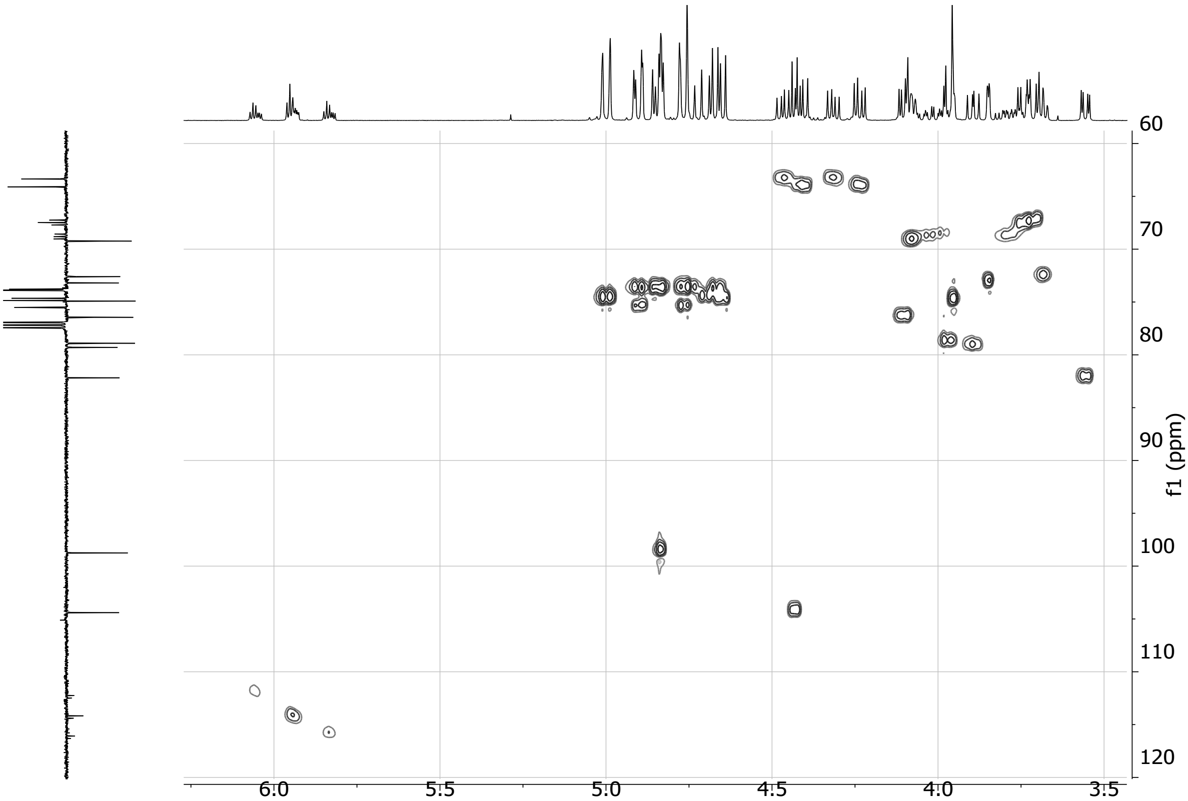

Supplementary Figure 364. HSQC NMR, $CDCl_3$ of compound **S94**

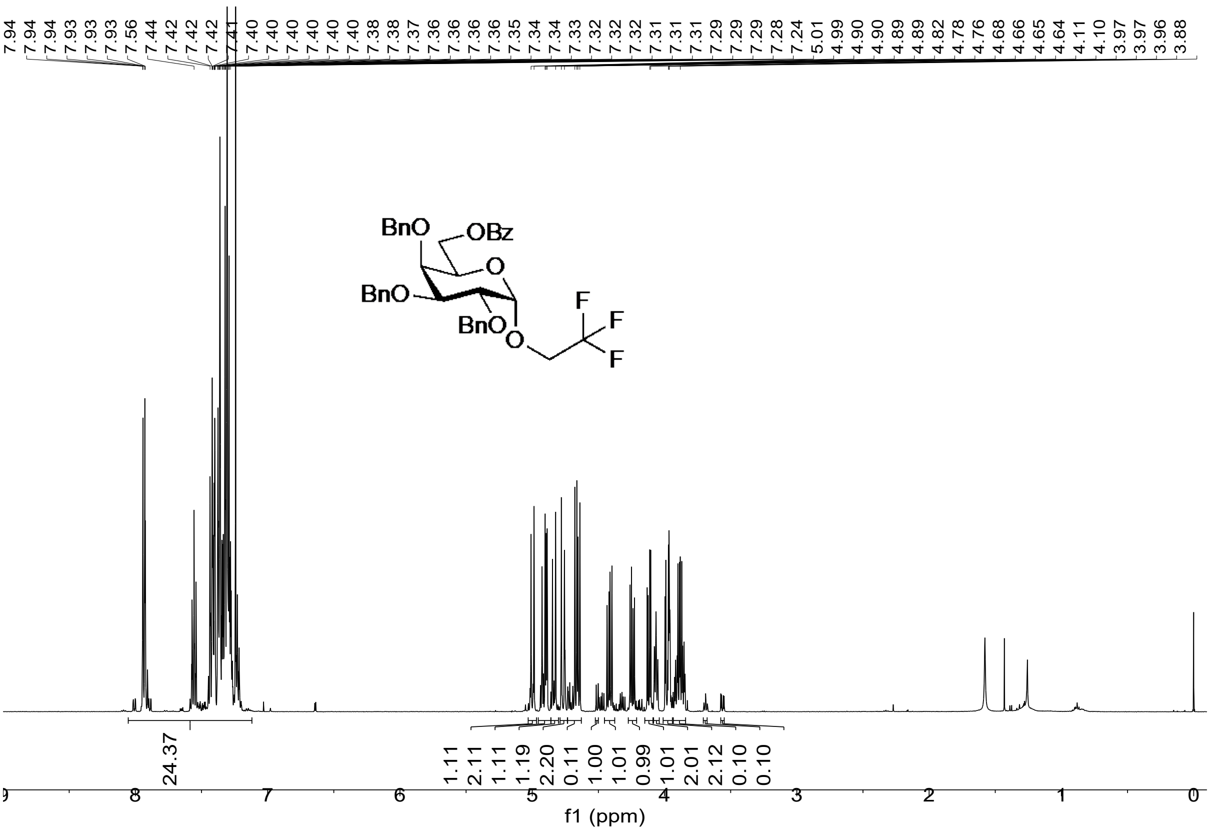

Supplementary Figure 365. ^{1}H NMR, 500 MHz, $CDCl_3$ of compound **S95**

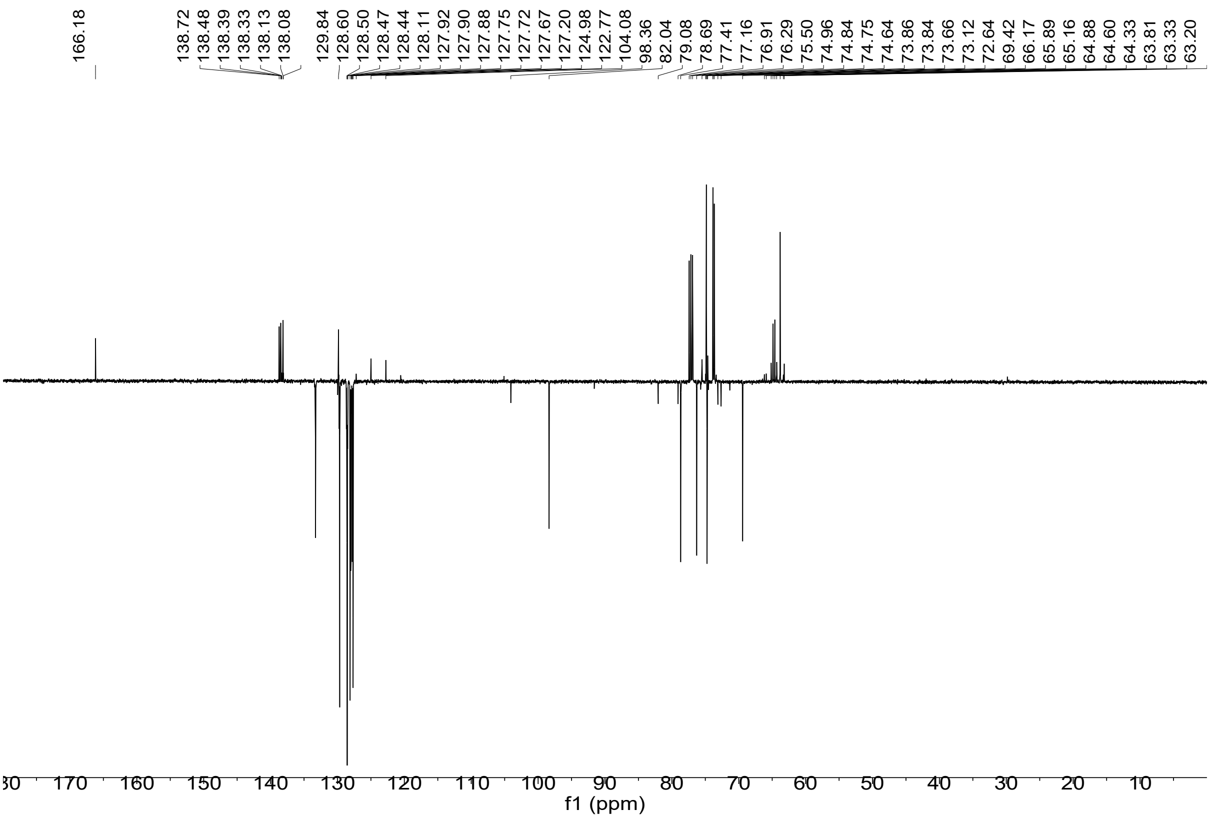

Supplementary Figure 366. ^{13}C NMR, 126 MHz, $CDCl_3$ of compound **S95**

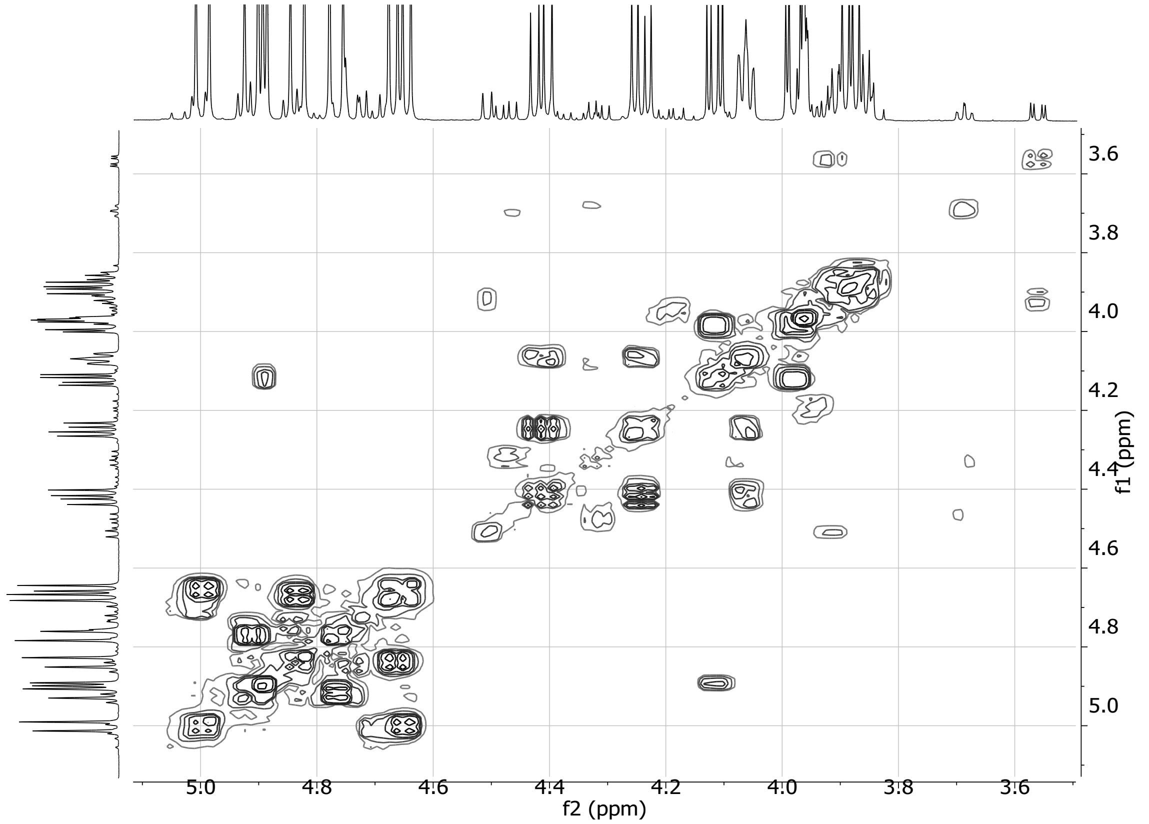

Supplementary Figure 367. HH-COSY NMR, $CDCl_3$ of compound **S95**

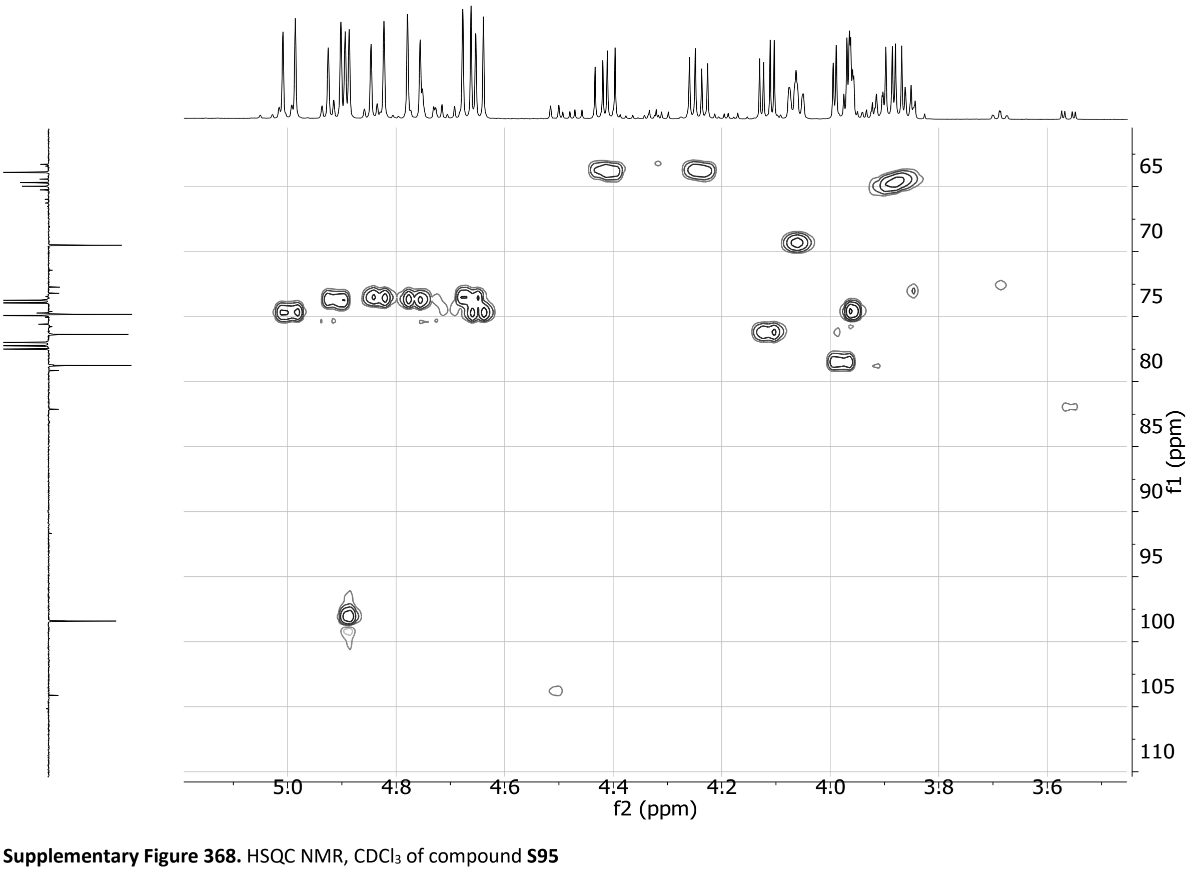

Supplementary Figure 368. HSQC NMR, $CDCl_3$ of compound **S95**

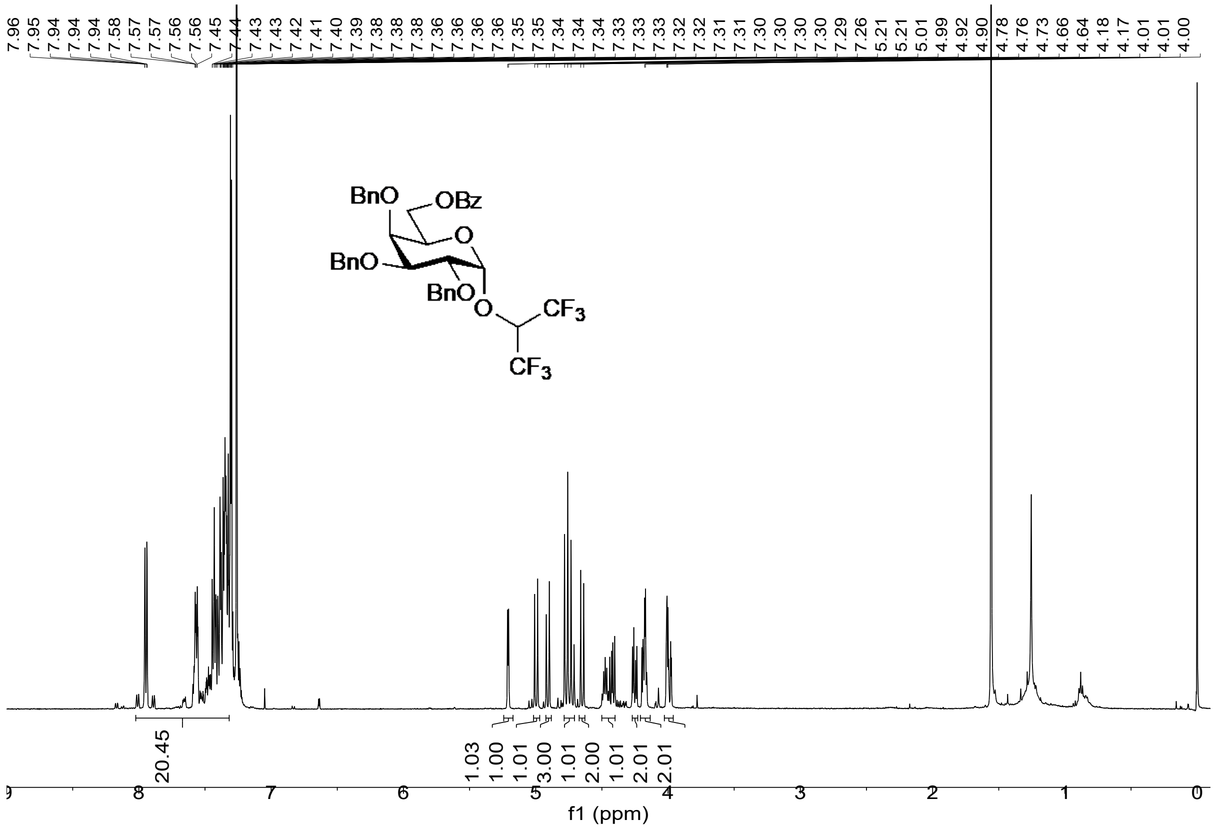

Supplementary Figure 369. ^{1}H NMR, 500 MHz, $CDCl_3$ of compound **S96**

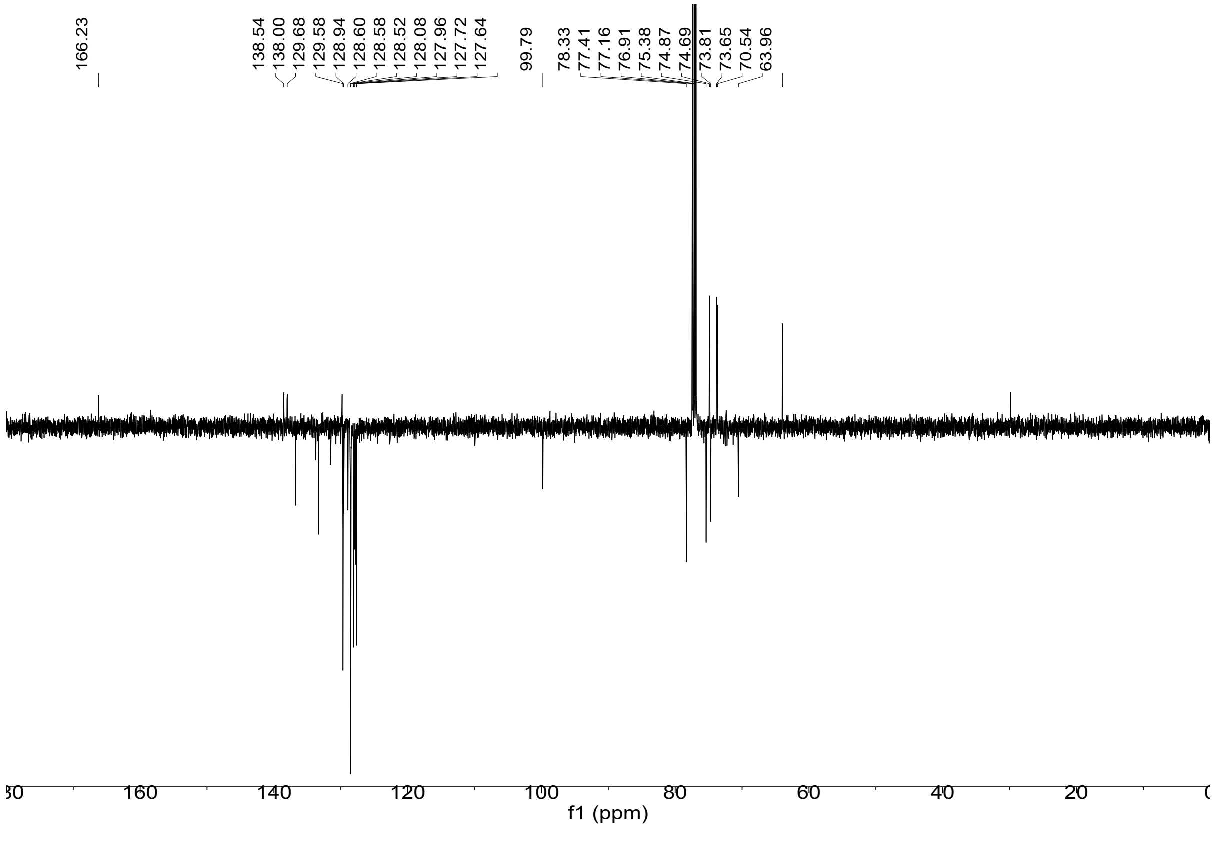

Supplementary Figure 370. ^{13}C NMR, 126 MHz, $CDCl_3$ of compound **S96**

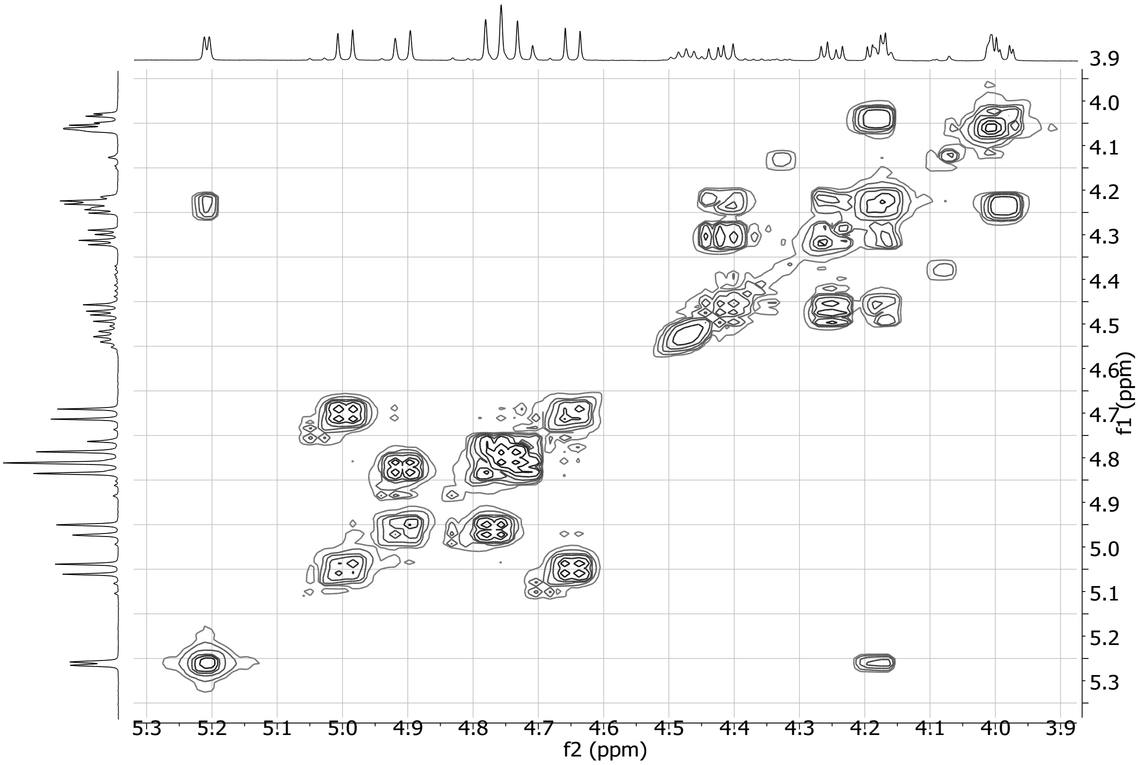

Supplementary Figure 371. HH-COSY NMR, $CDCl_3$ of compound **S96**

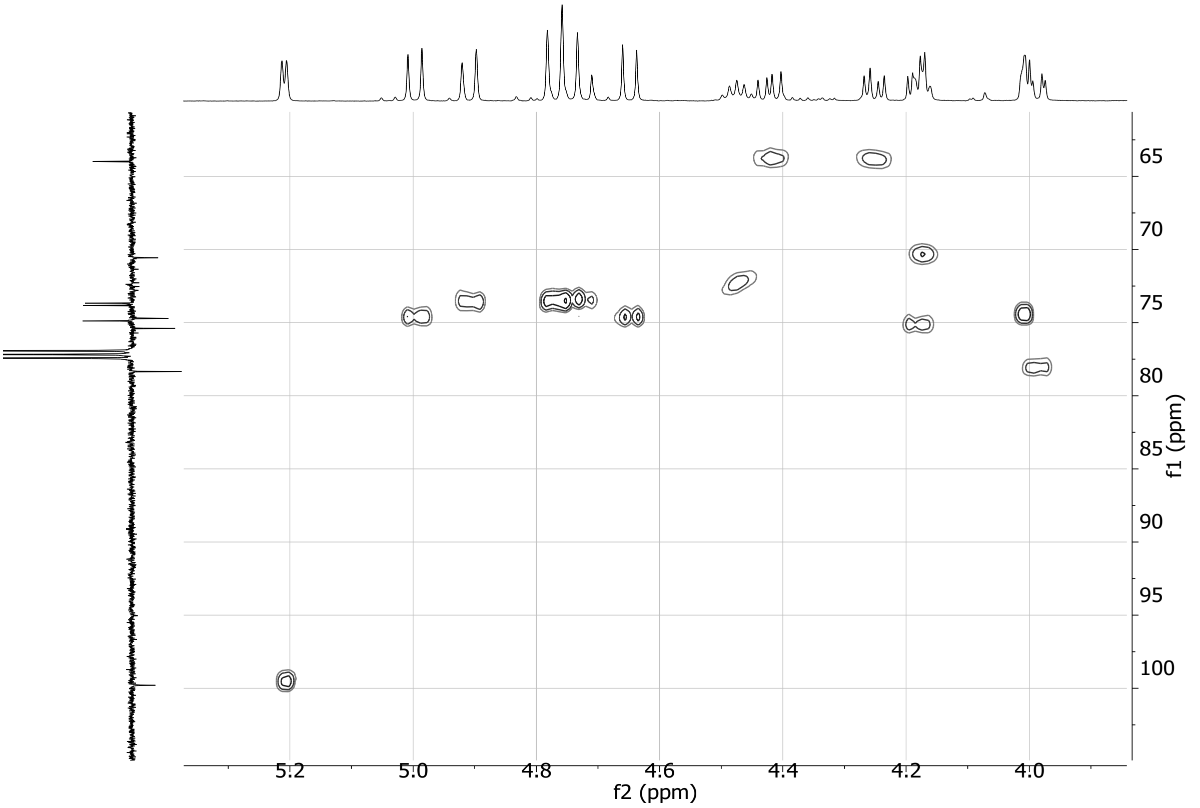

Supplementary Figure 372. HSQC NMR, $CDCl_3$ of compound **S96**

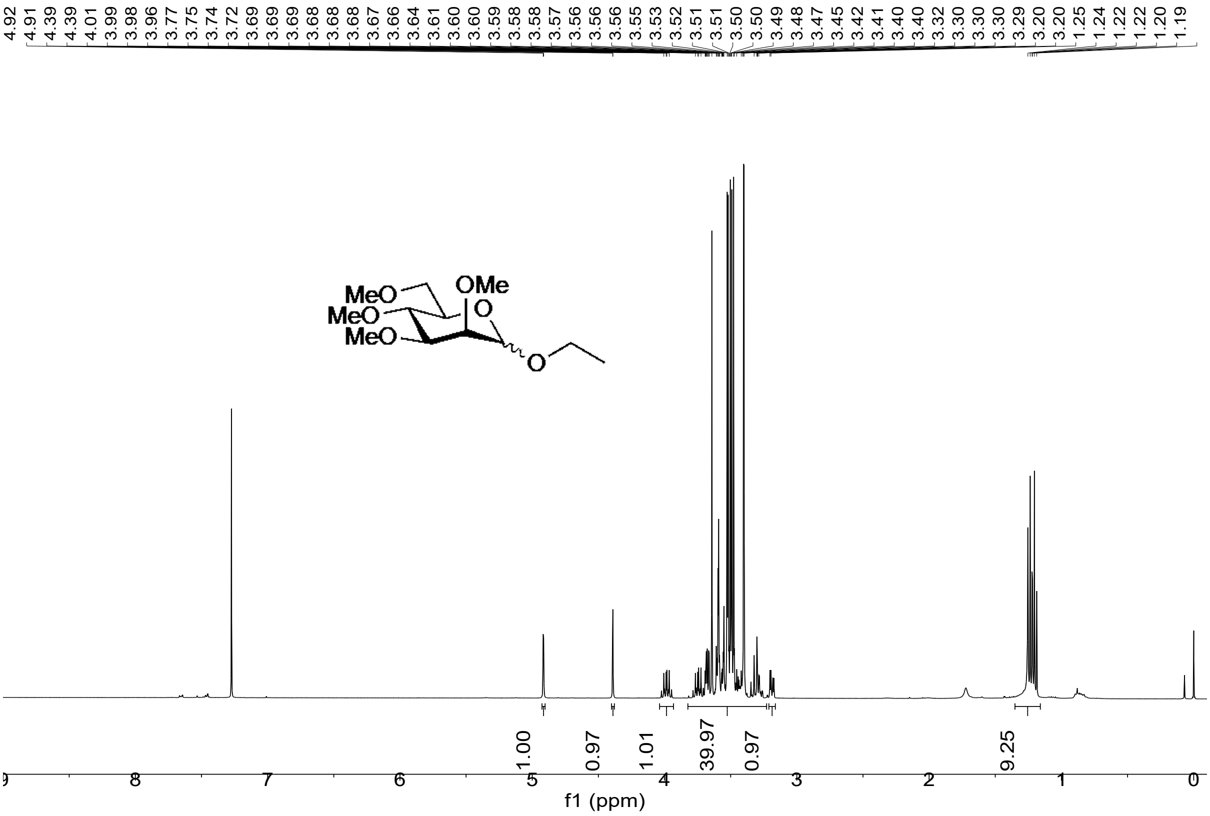

Supplementary Figure 373. ^{1}H NMR, 400 MHz, $CDCl_3$ of compound **S97**

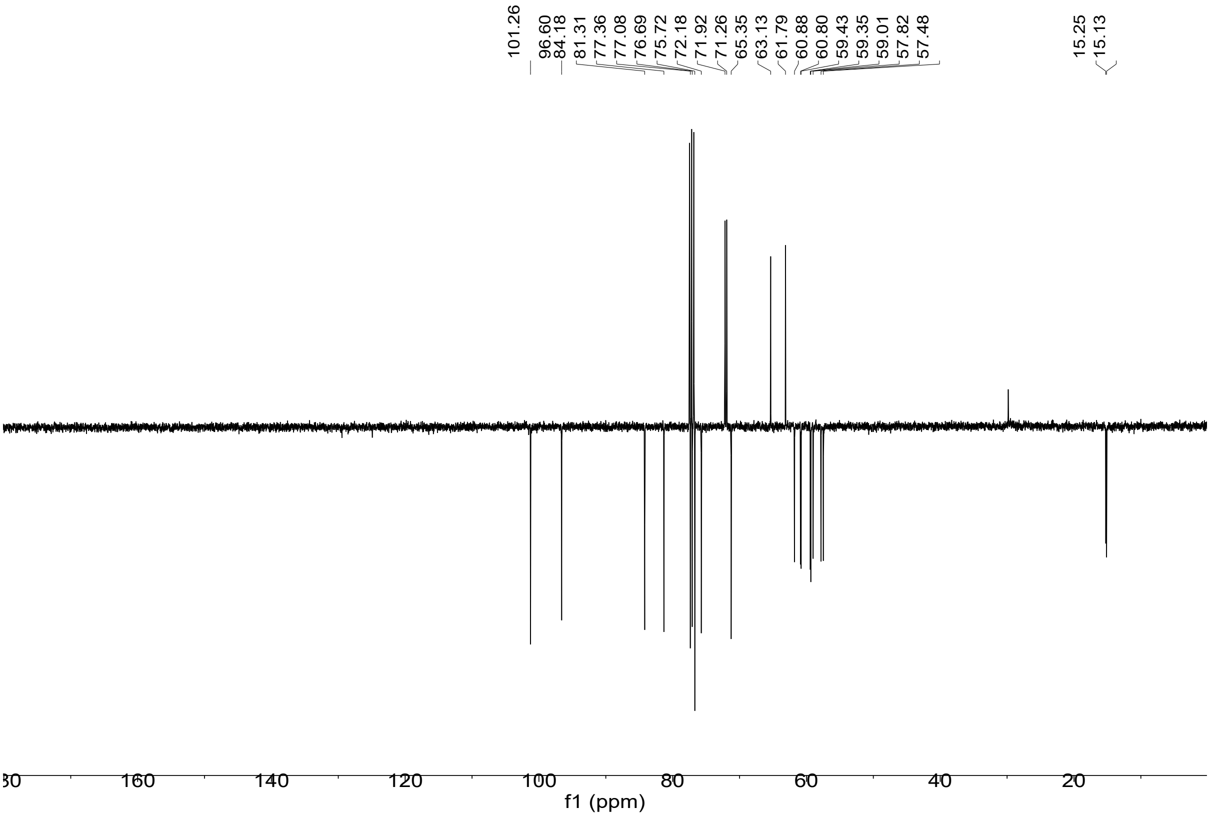

Supplementary Figure 374. ^{13}C NMR, 101 MHz, $CDCl_3$ of compound **S97**

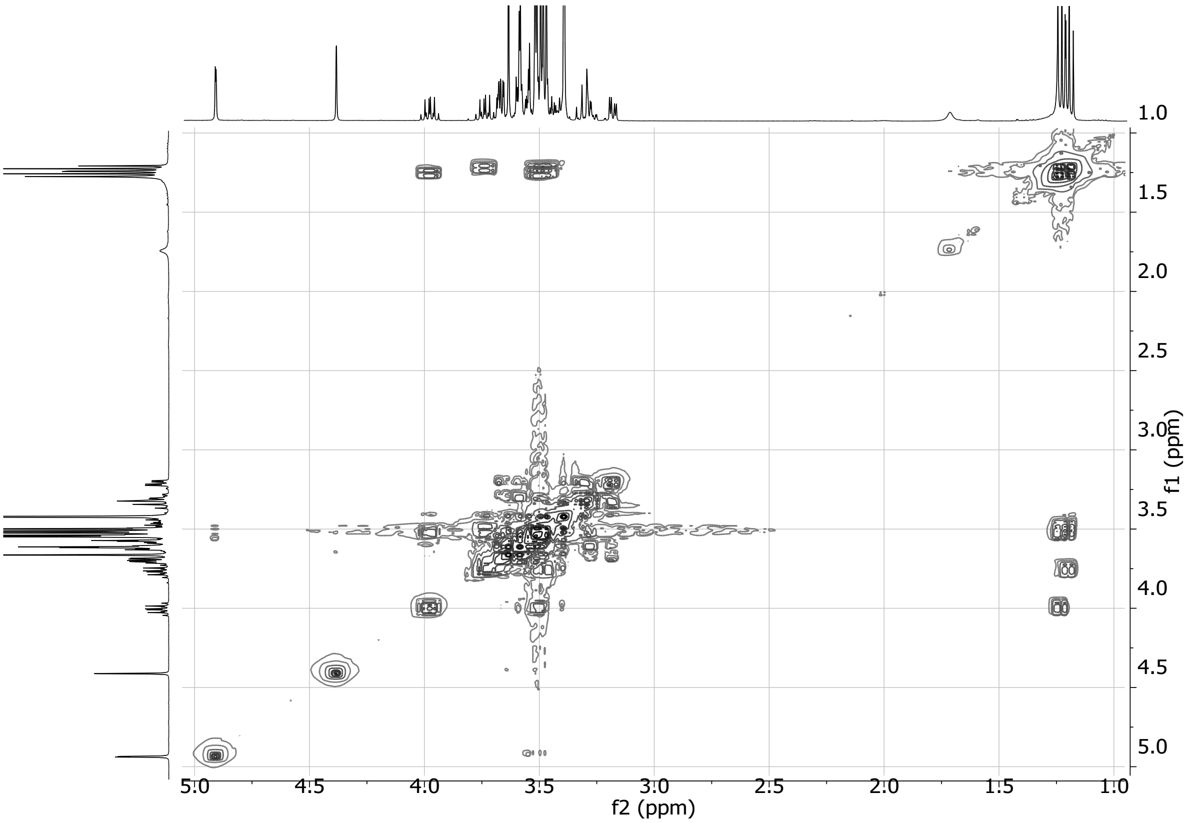

Supplementary Figure 375. HH-COSY NMR, $CDCl_3$ of compound **S97**

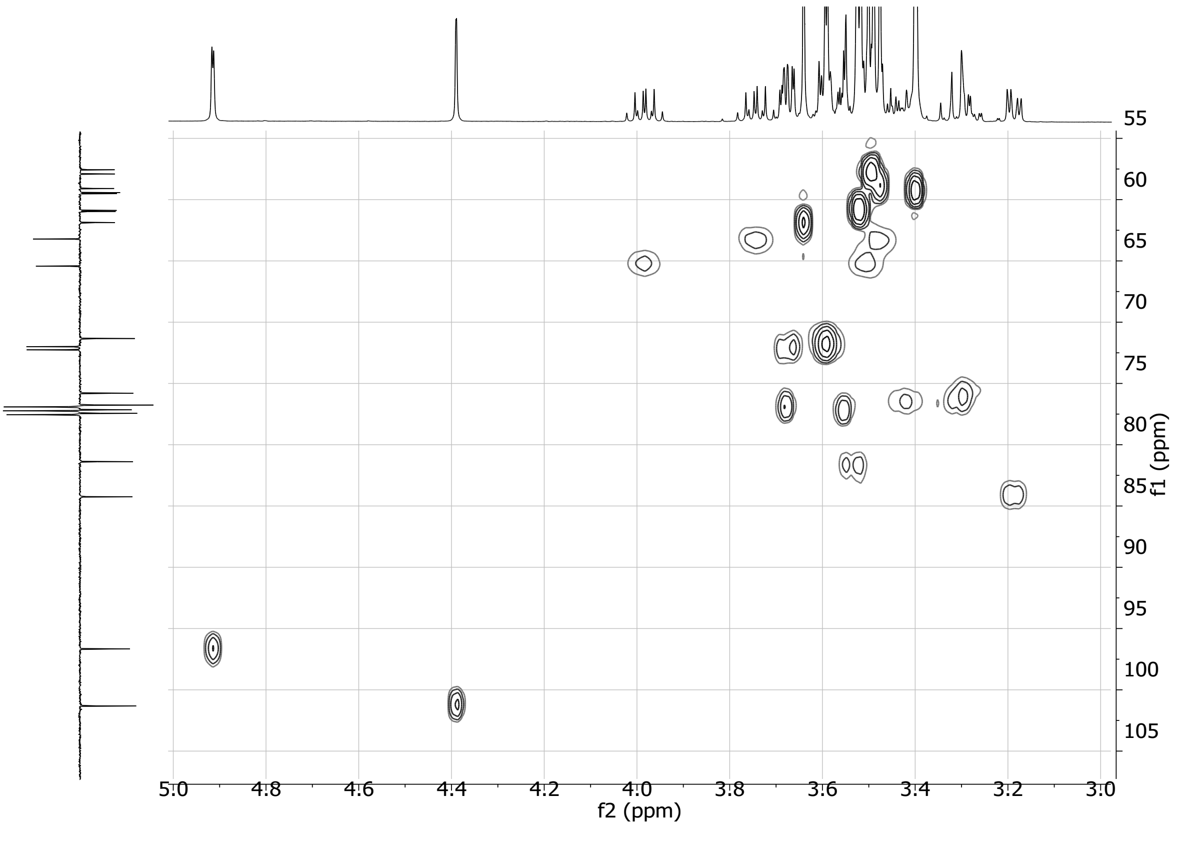

Supplementary Figure 376. HSQC NMR, $CDCl_3$ of compound **S97**

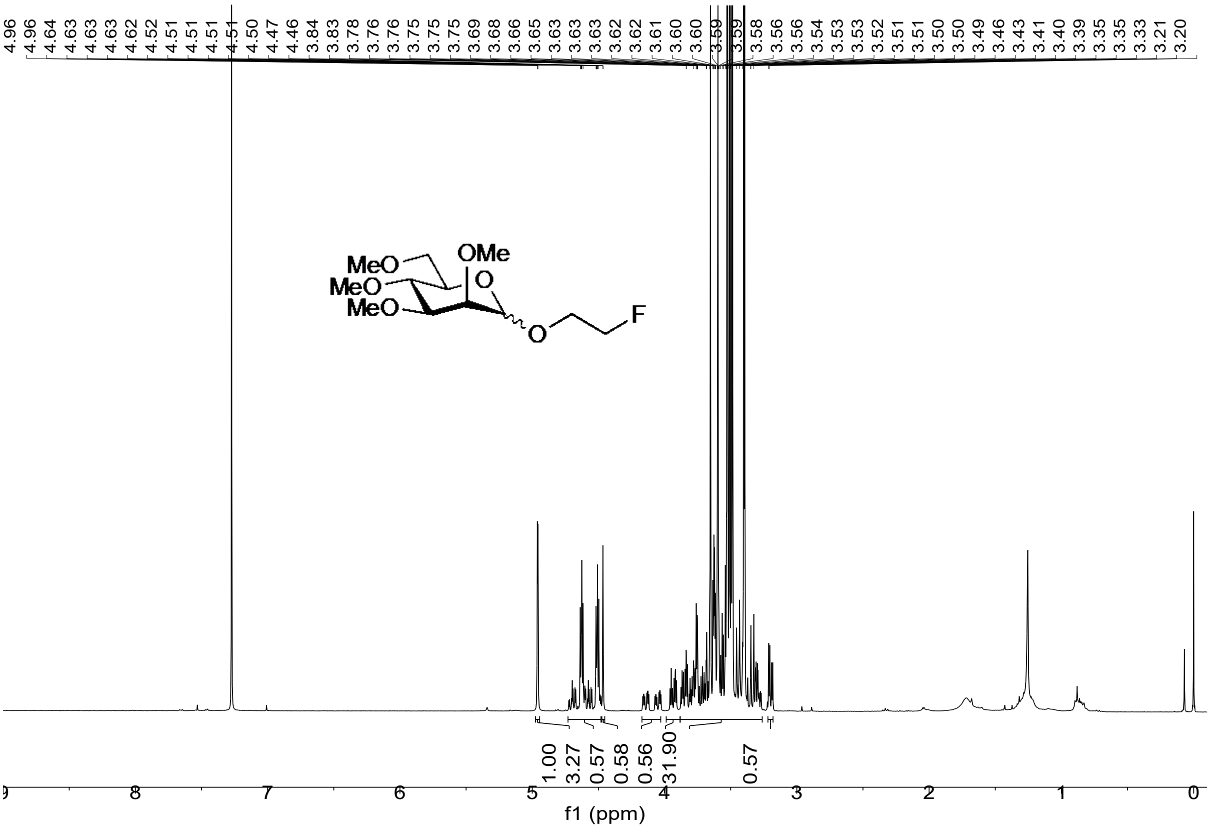

Supplementary Figure 377. ^{1}H NMR, 400 MHz, $CDCl_3$ of compound **S98**

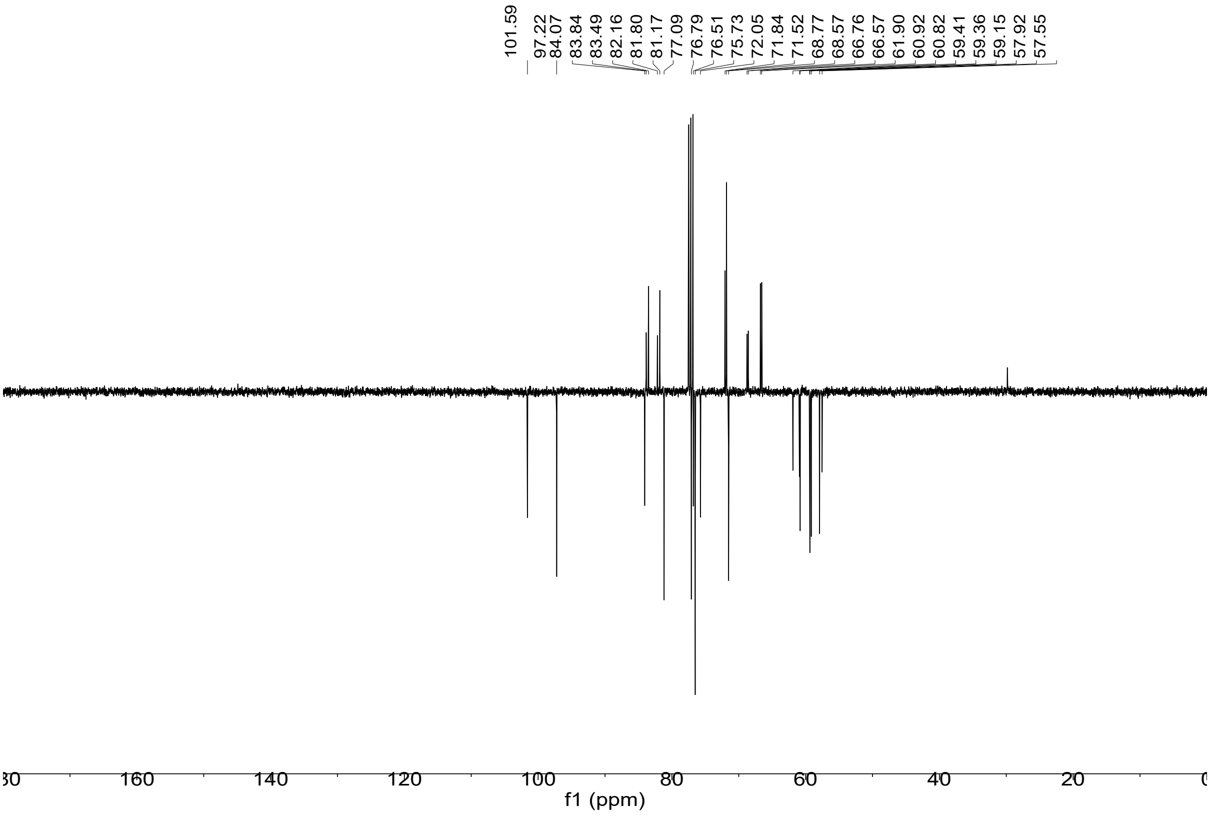

Supplementary Figure 378. ^{13}C NMR, 101 MHz, $CDCl_3$ of compound **S98**

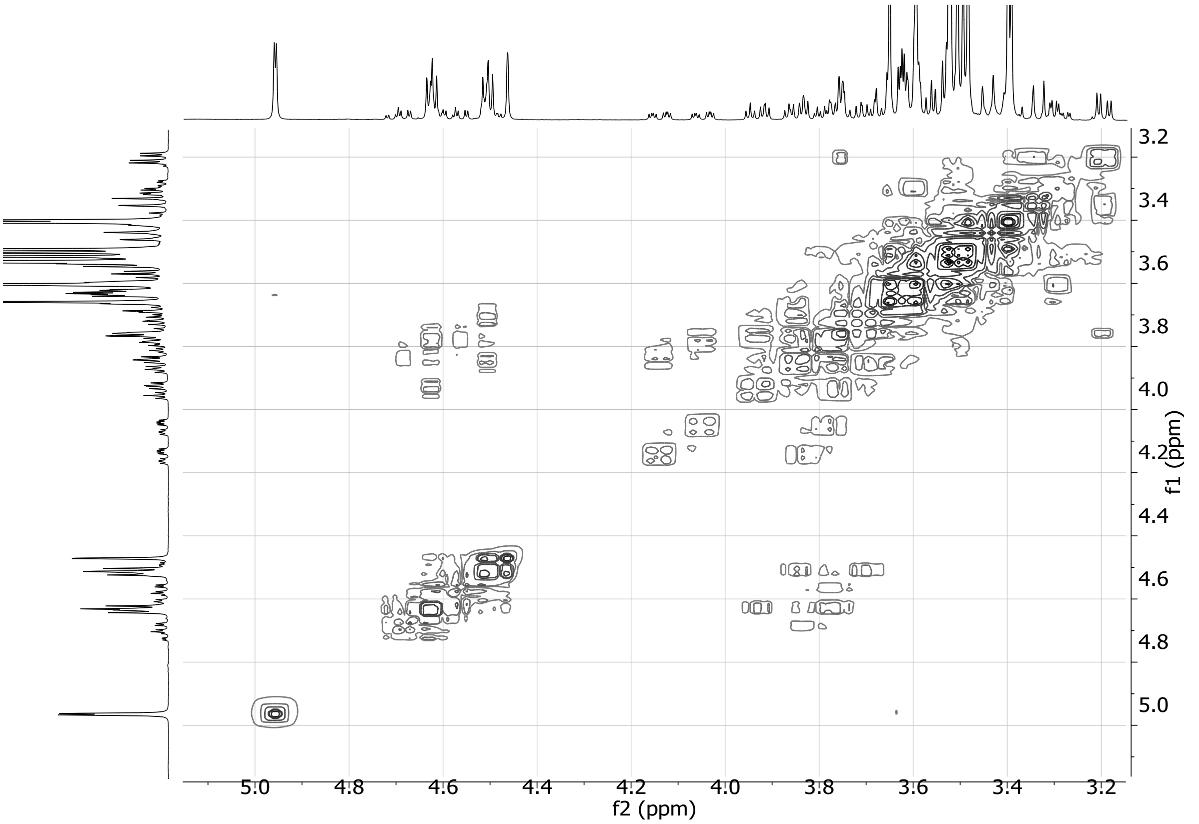

Supplementary Figure 379. HH-COSY NMR, $CDCl_3$ of compound **S98**

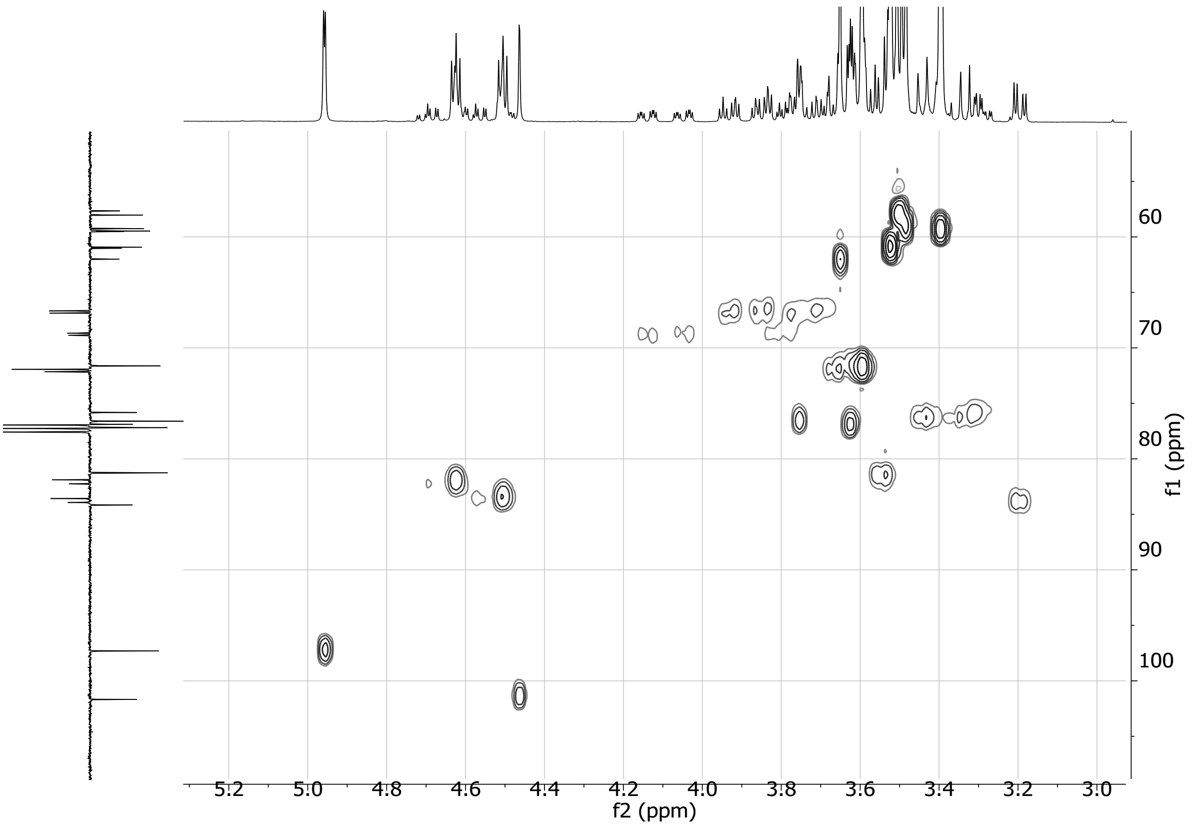

Supplementary Figure 380. HSQC NMR, $CDCl_3$ of compound **S98**

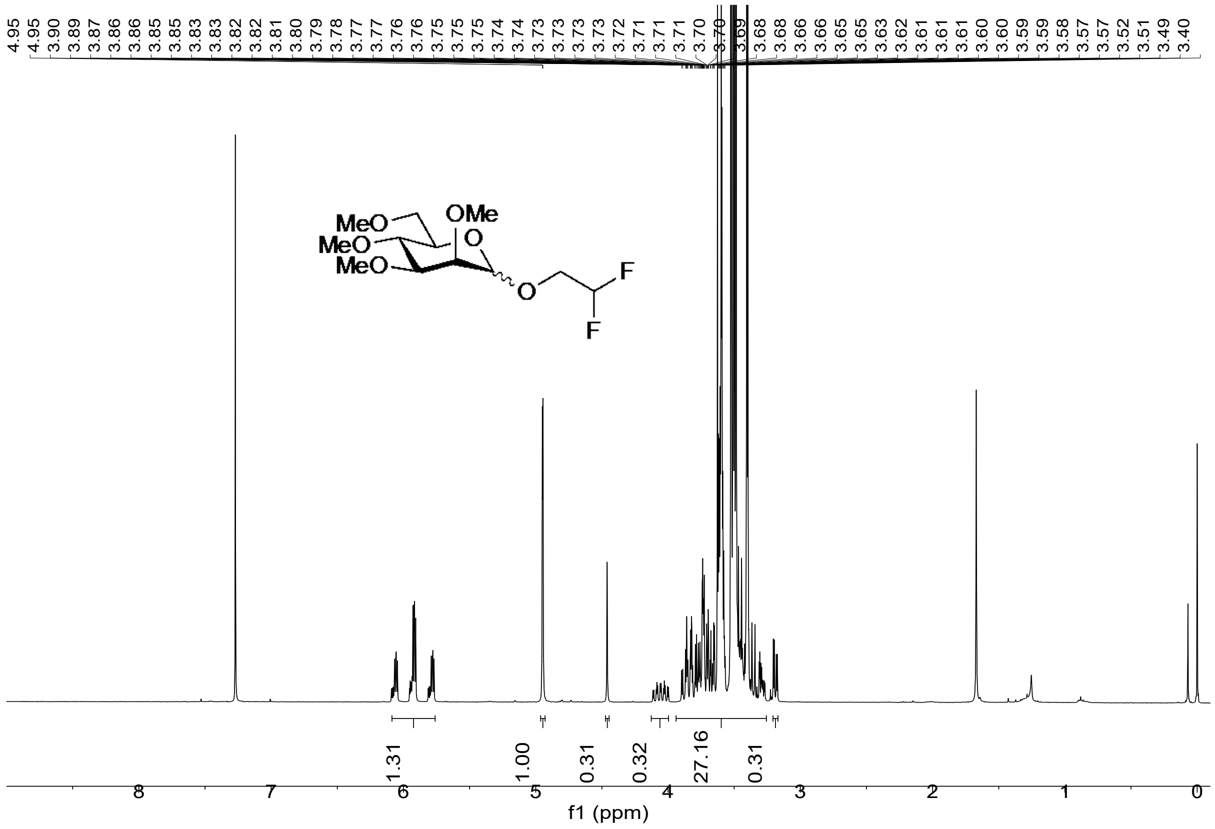

Supplementary Figure 381. ^{1}H NMR, 400 MHz, $CDCl_3$ of compound **S99**

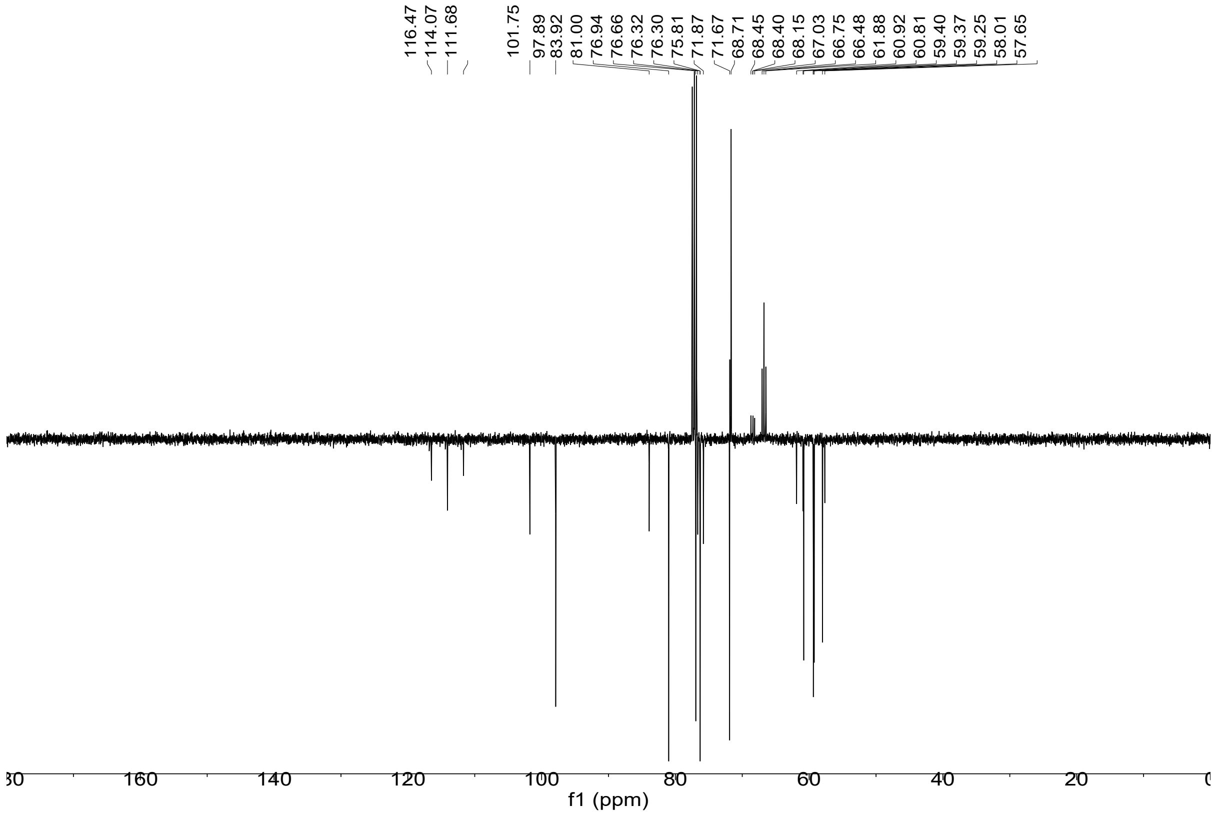

Supplementary Figure 382. ^{13}C NMR, 101 MHz, $CDCl_3$ of compound **S99**

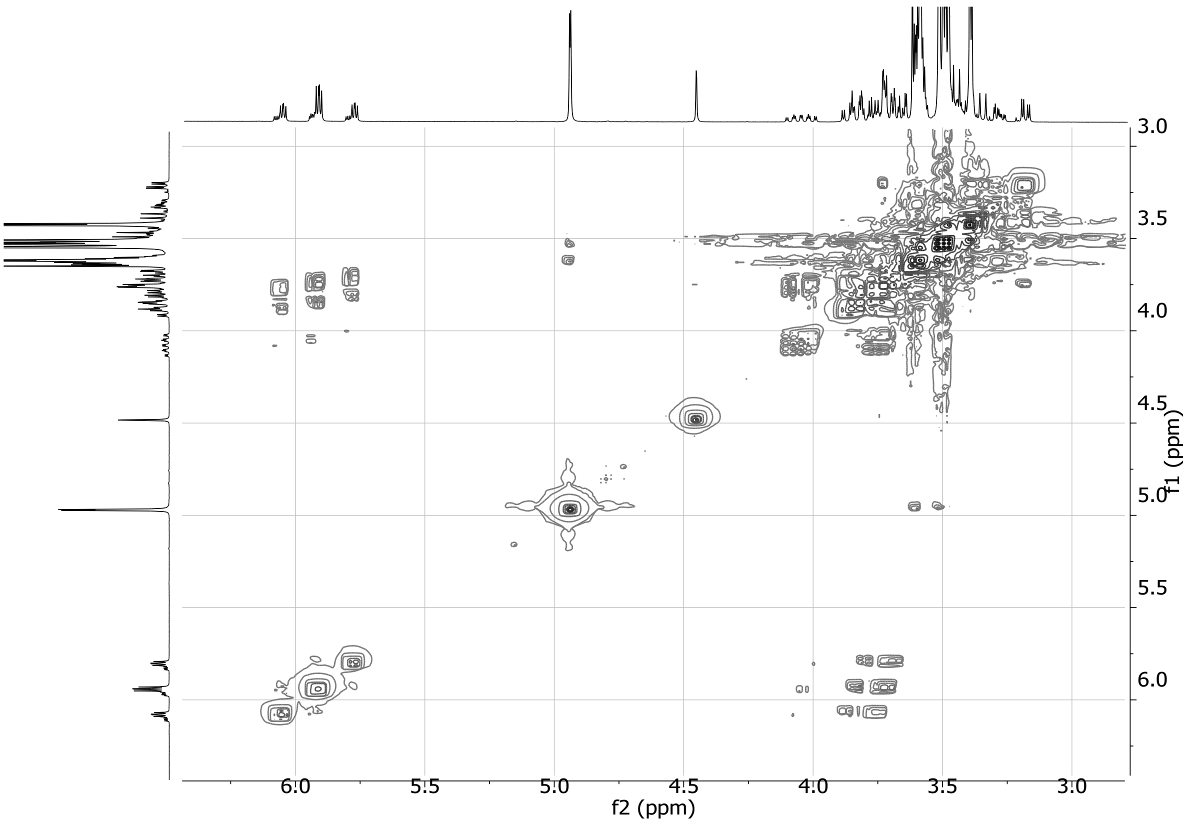

Supplementary Figure 383. HH-COSY NMR, $CDCl_3$ of compound **S99**

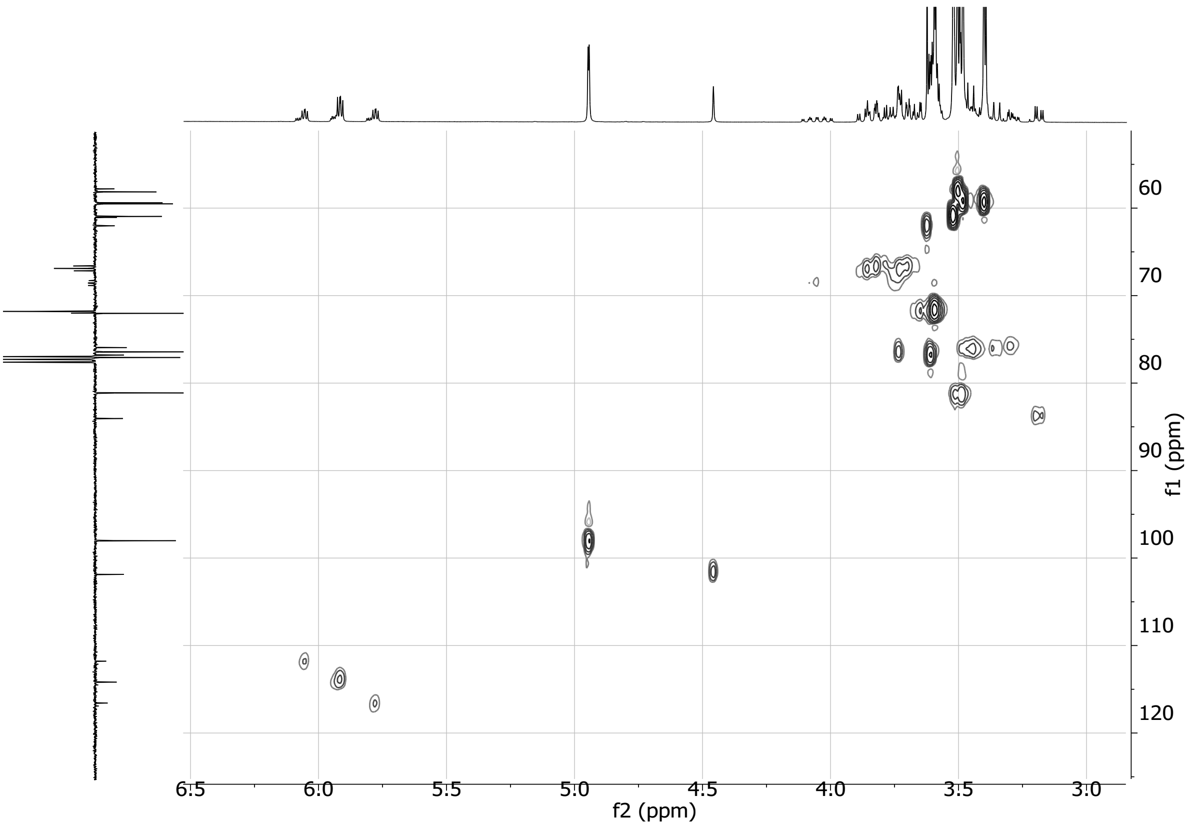

Supplementary Figure 384. HSQC NMR, $CDCl_3$ of compound **S99**

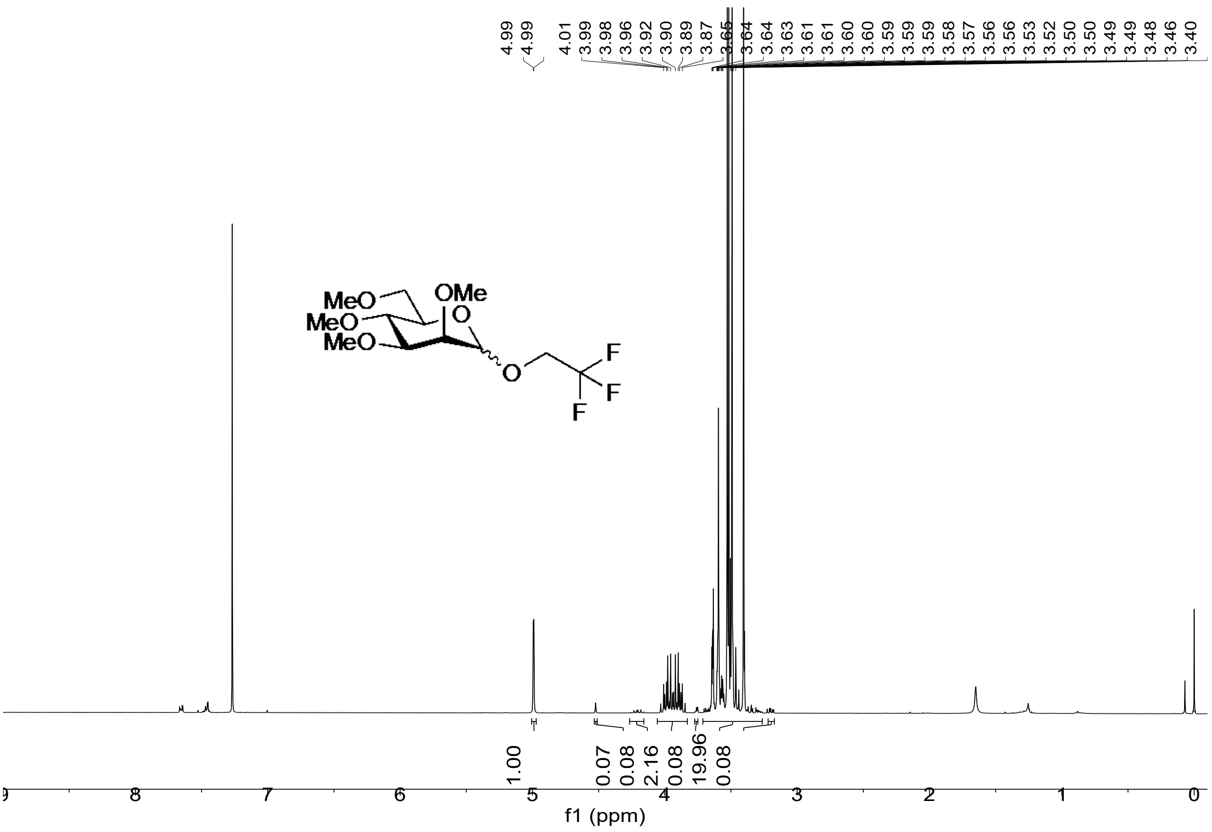

Supplementary Figure 385. ^{1}H NMR, 400 MHz, $CDCl_3$ of compound **S100**

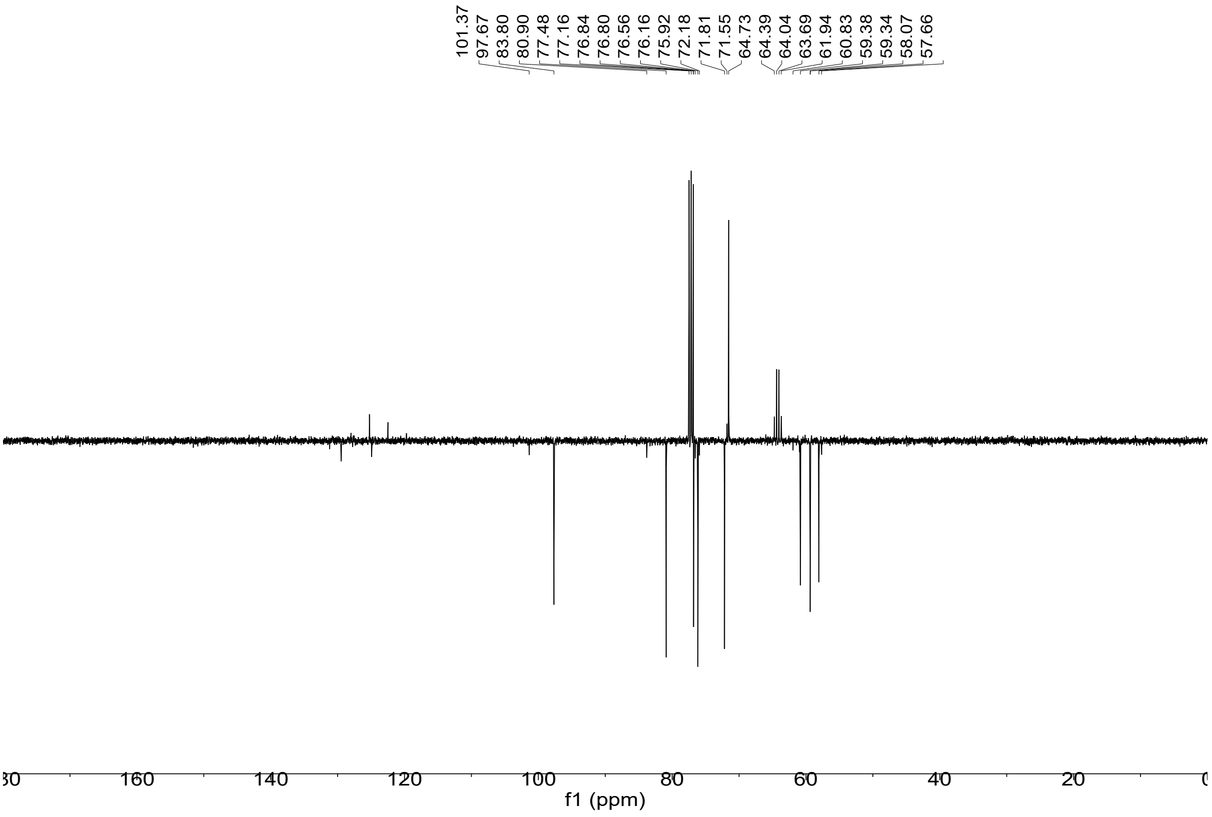

Supplementary Figure 386. ^{13}C NMR, 101 MHz, $CDCl_3$ of compound **S100**

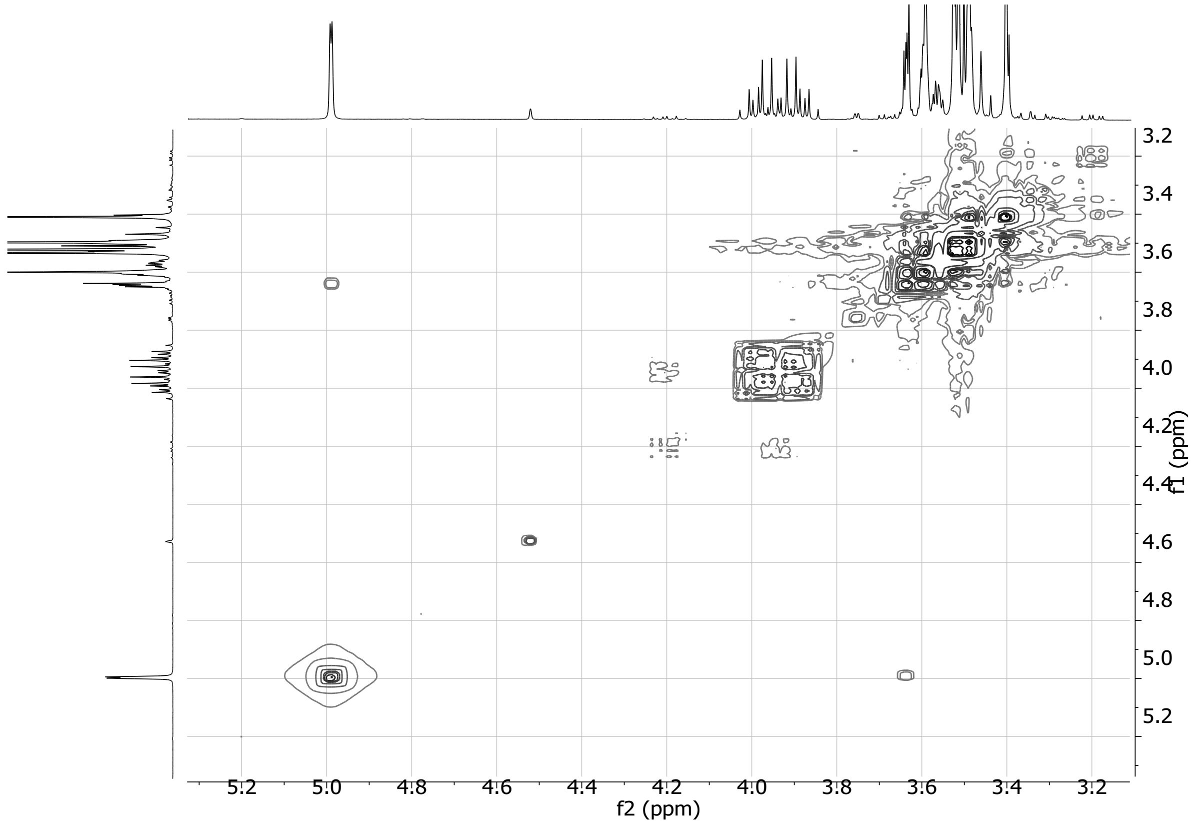

Supplementary Figure 387. HH-COSY NMR, $CDCl_3$ of compound **S100**

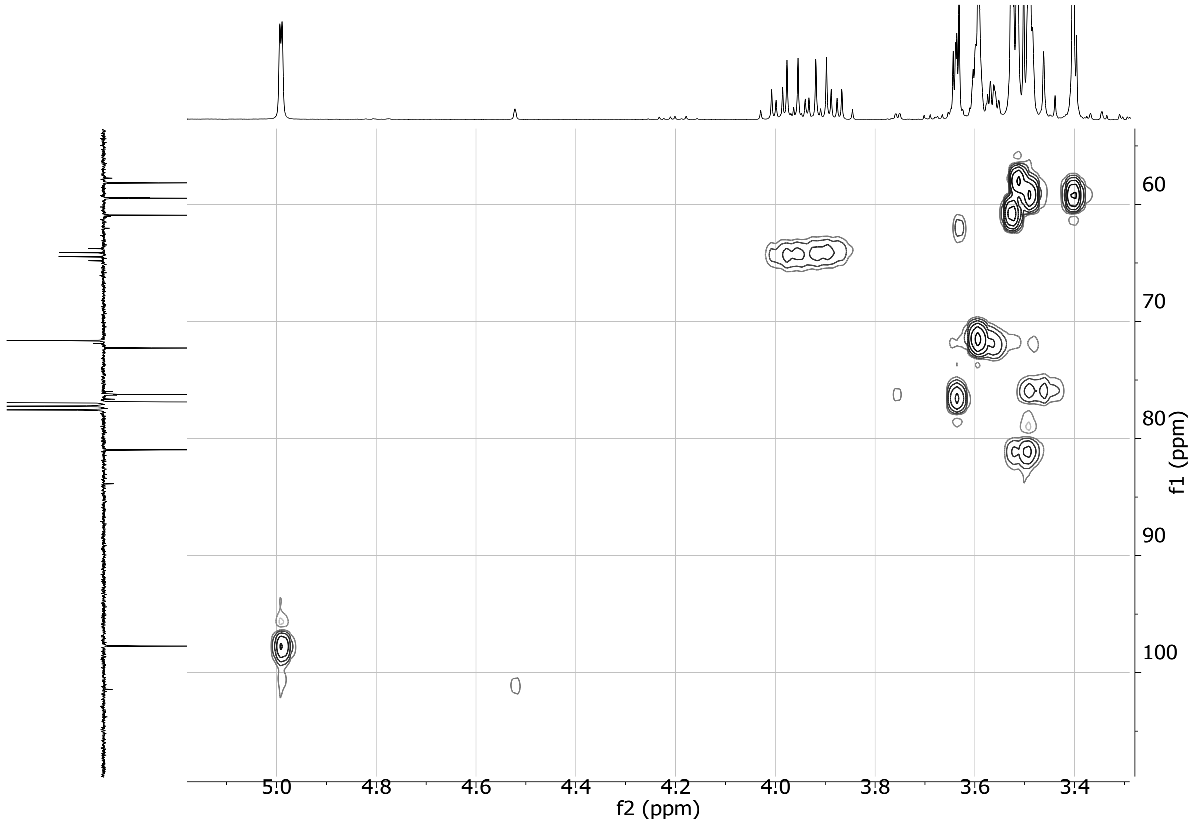

Supplementary Figure 388. HSQC NMR, $CDCl_3$ of compound **S100**

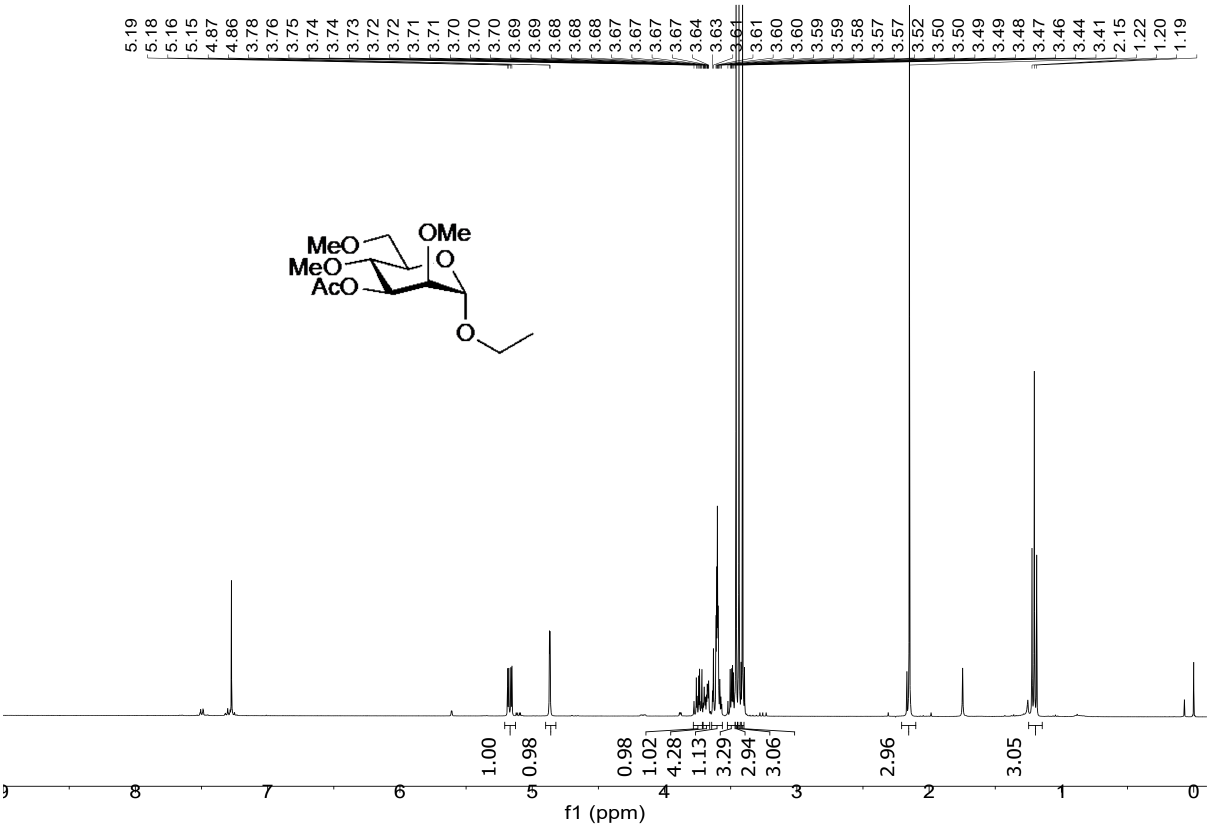

Supplementary Figure 389. ^{1}H NMR, 400 MHz, $CDCl_3$ of compound **S101**

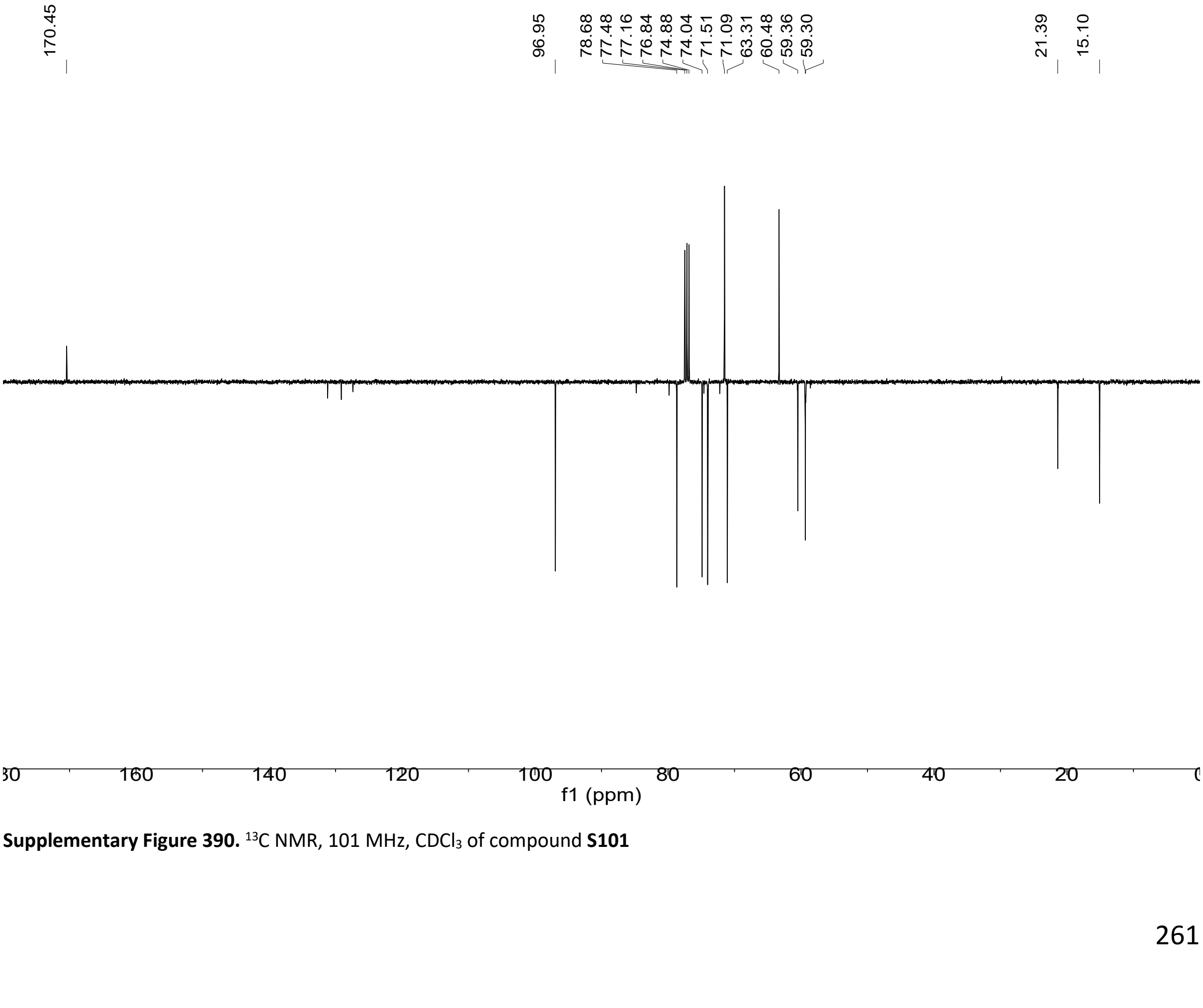

Supplementary Figure 390. ^{13}C NMR, 101 MHz, $CDCl_3$ of compound **S101**

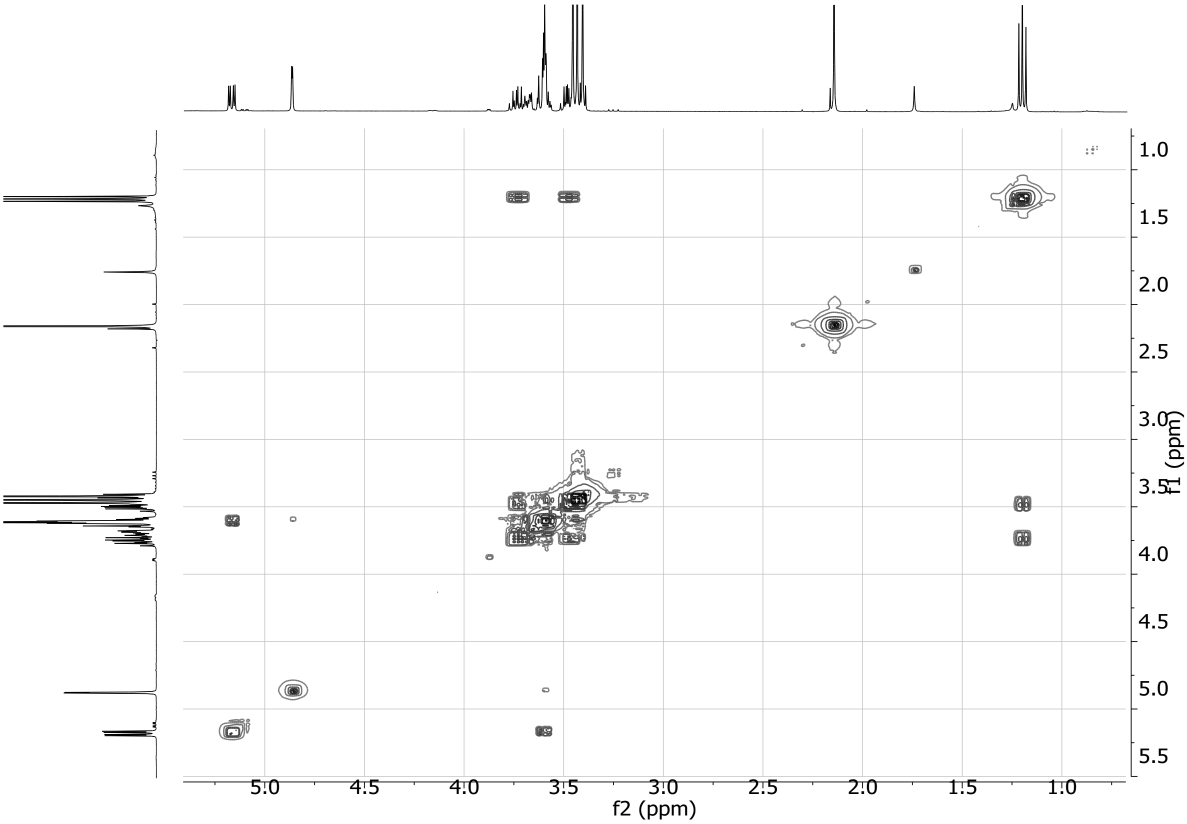

Supplementary Figure 391. HH-COSY NMR, $CDCl_3$ of compound **S101**

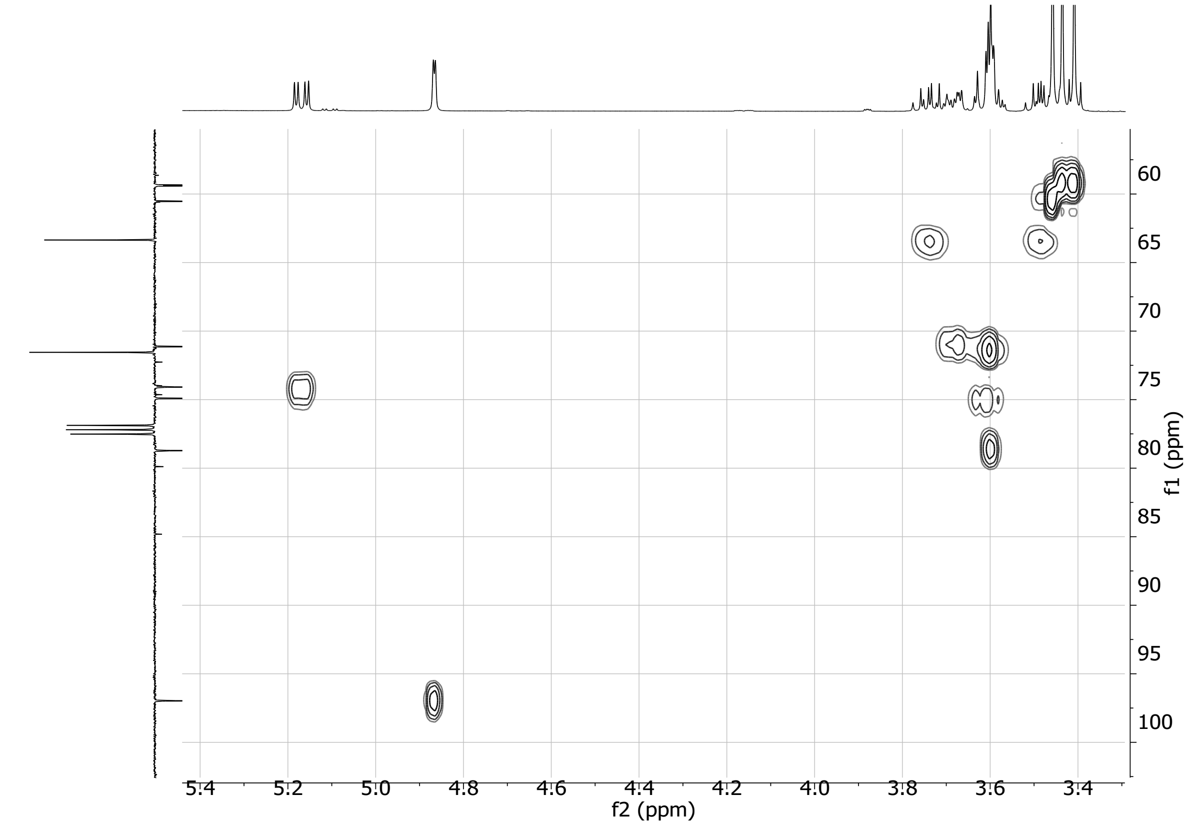

Supplementary Figure 392. HSQC NMR, $CDCl_3$ of compound **S101**

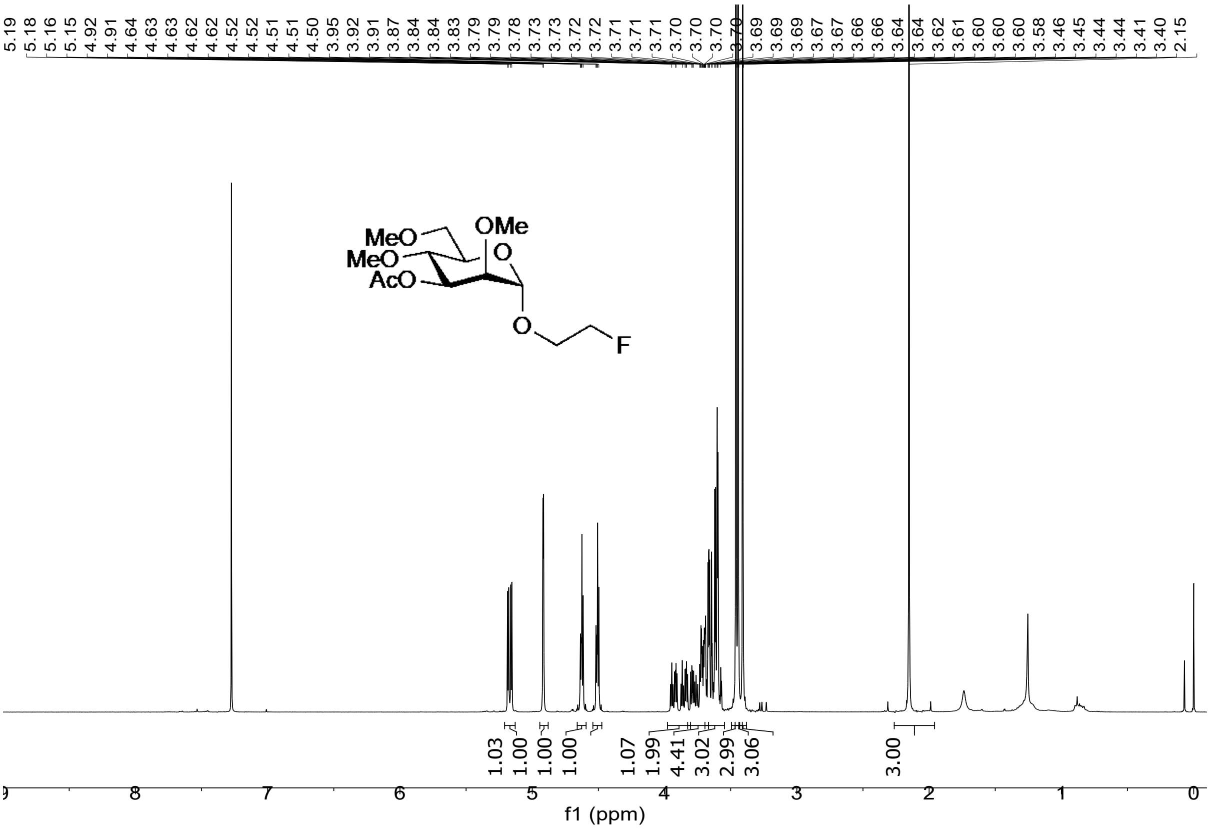

Supplementary Figure 393. ^{1}H NMR, 400 MHz, $CDCl_3$ of compound **S102**

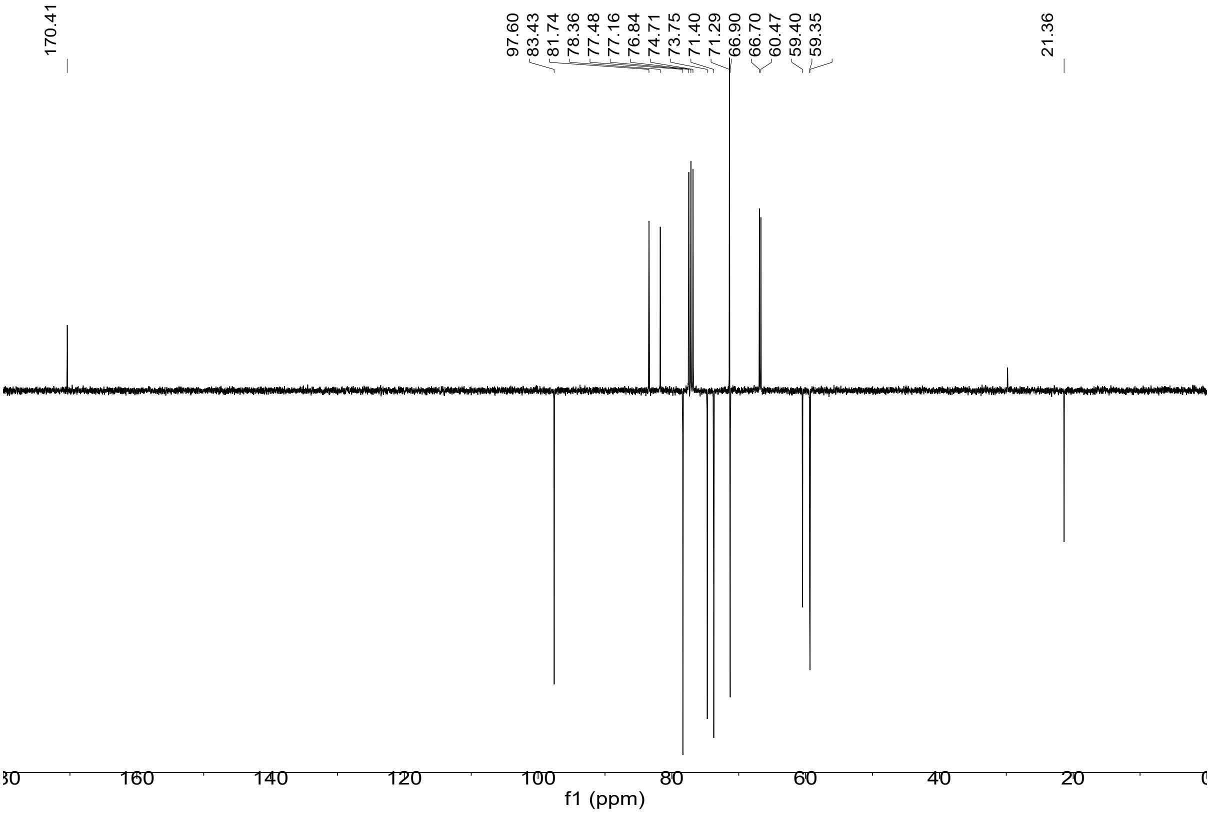

Supplementary Figure 394. ^{13}C NMR, 101 MHz, $CDCl_3$ of compound **S102**

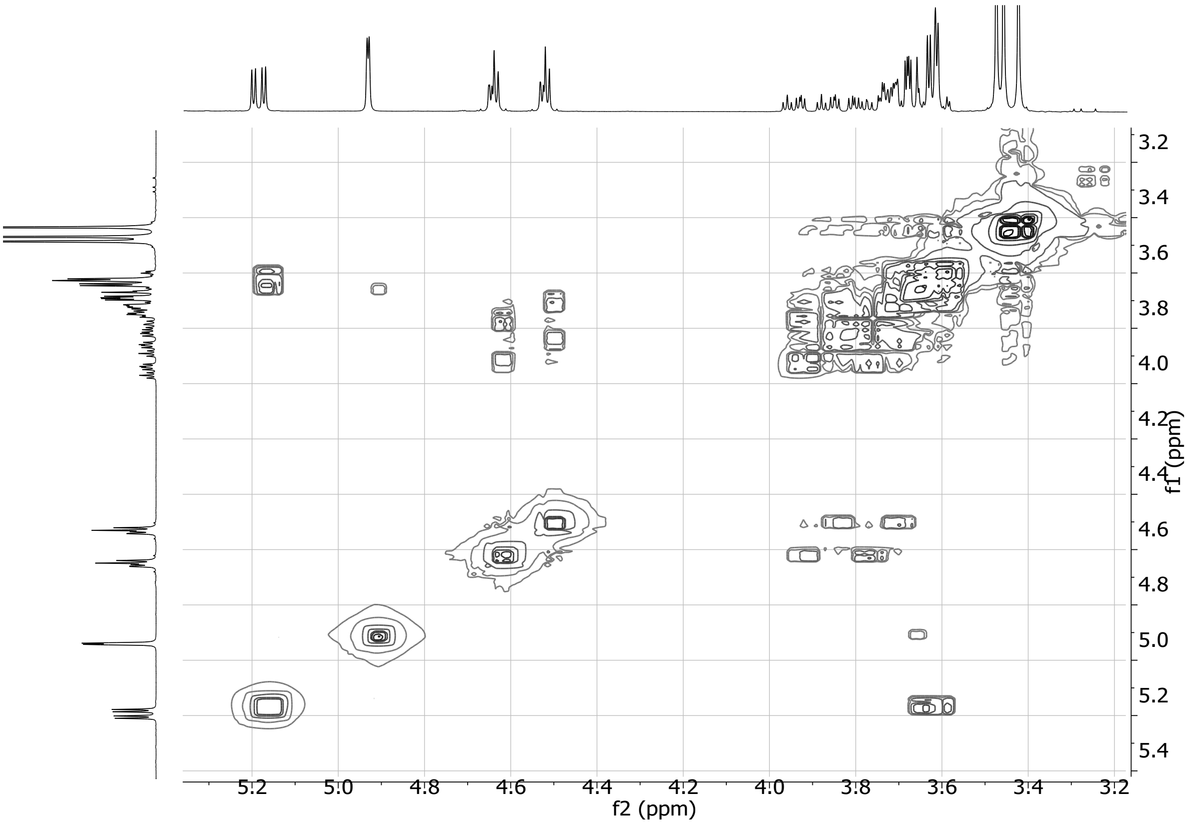

Supplementary Figure 395. HH-COSY NMR, $CDCl_3$ of compound **S102**

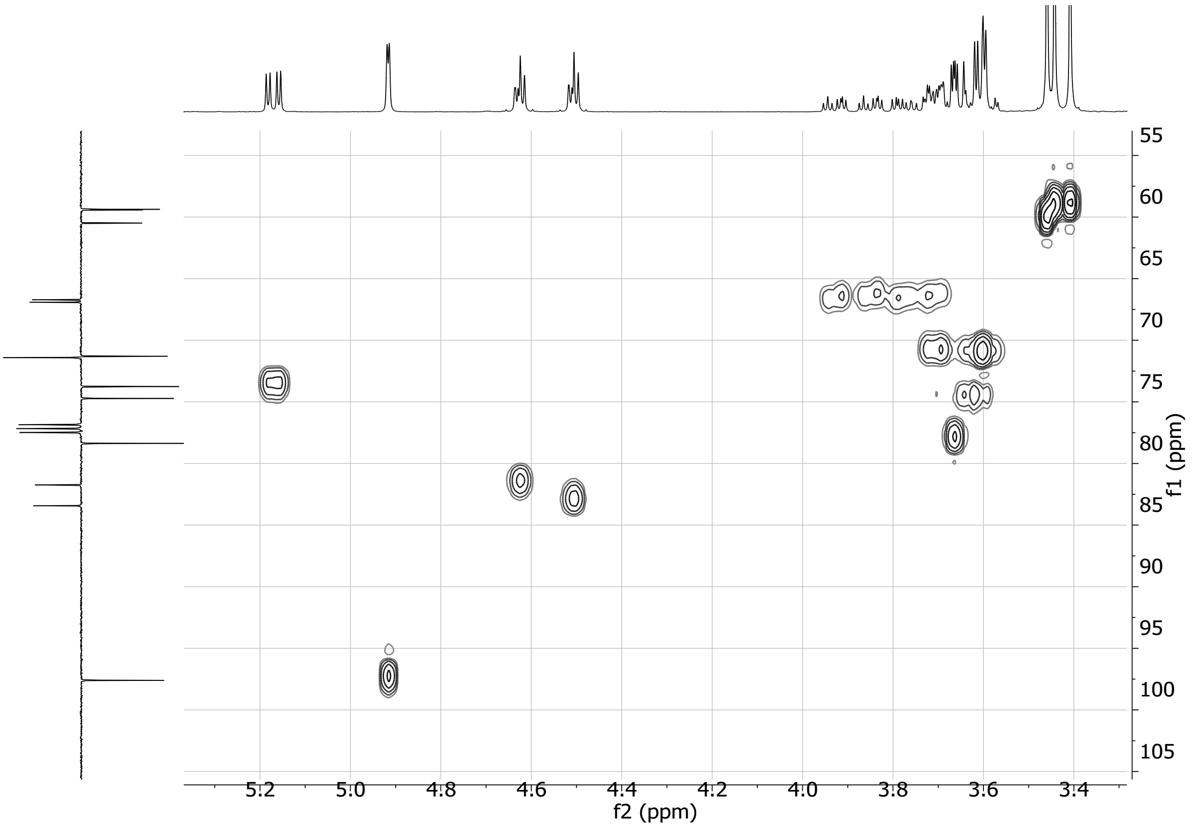

Supplementary Figure 396. HSQC NMR, $CDCl_3$ of compound **S102**

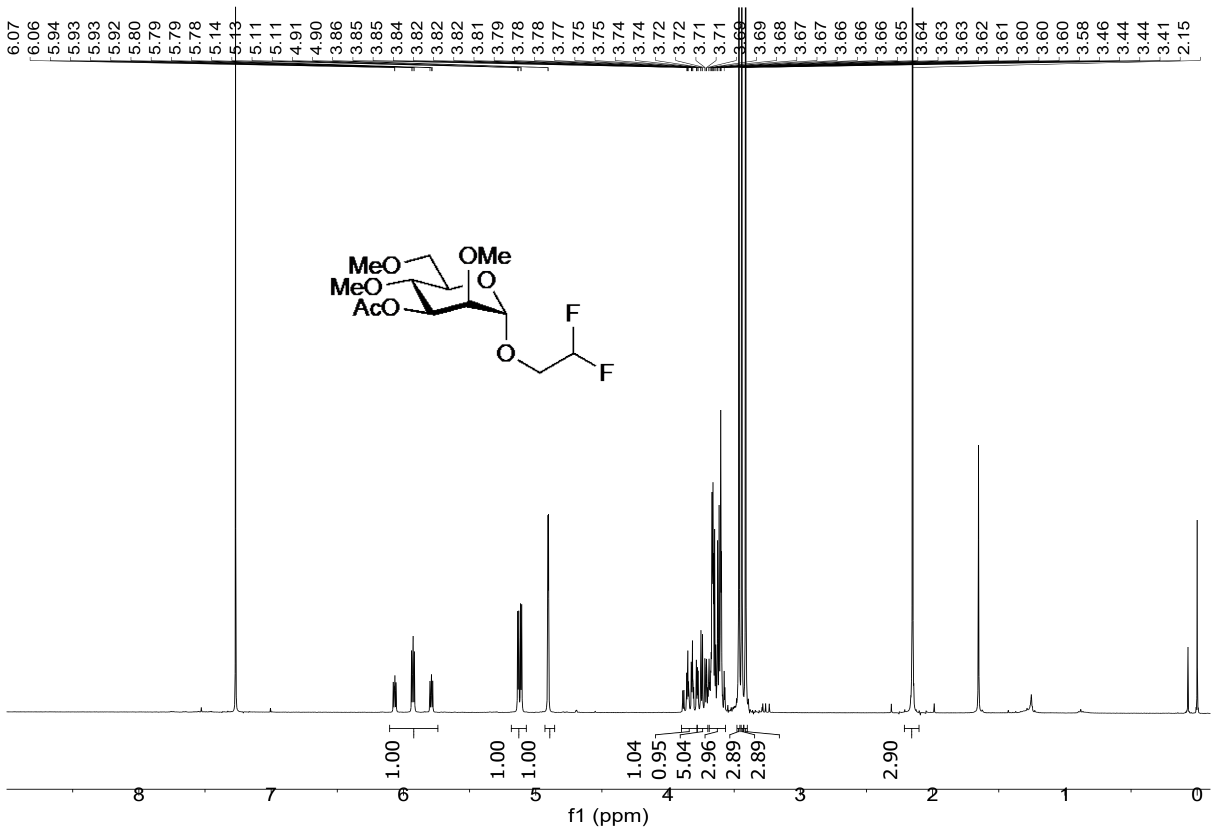

Supplementary Figure 397. ^{1}H NMR, 400 MHz, $CDCl_3$ of compound **S103**

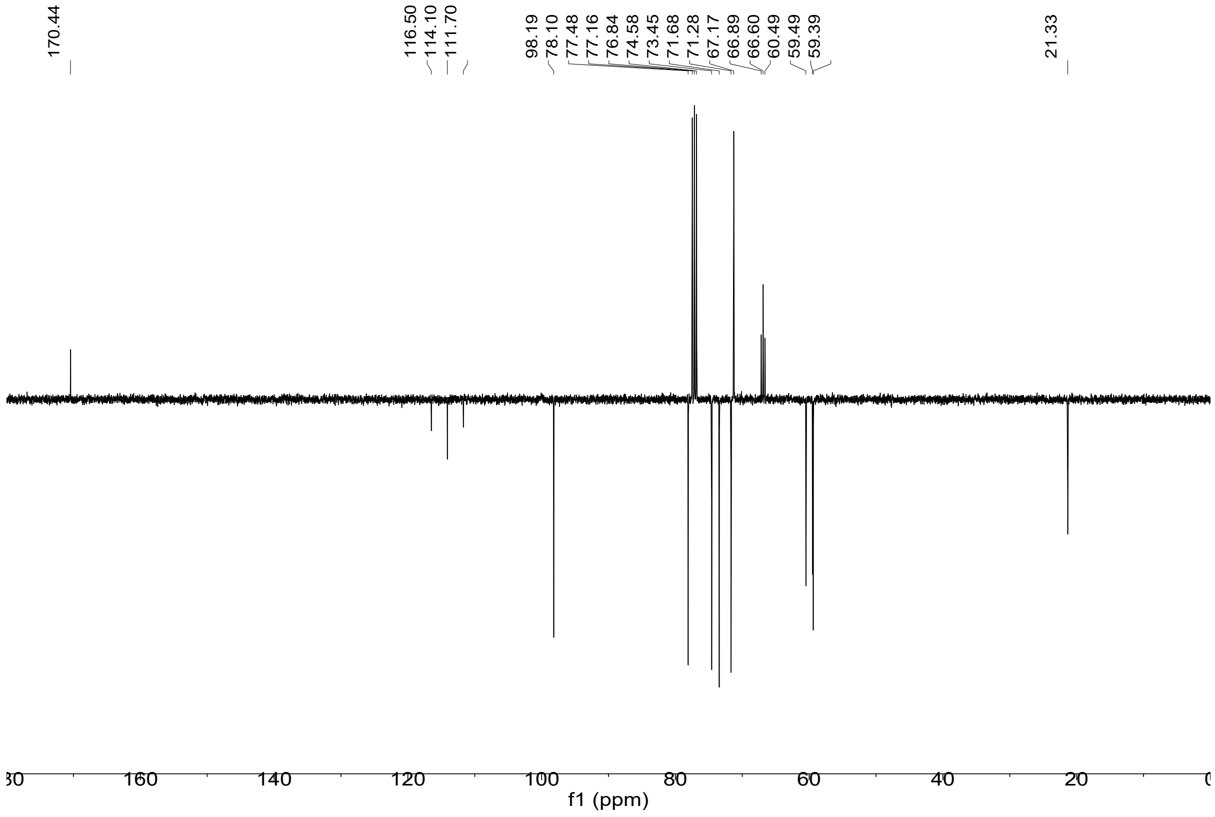

Supplementary Figure 398. ^{13}C NMR, 101 MHz, $CDCl_3$ of compound **S103**

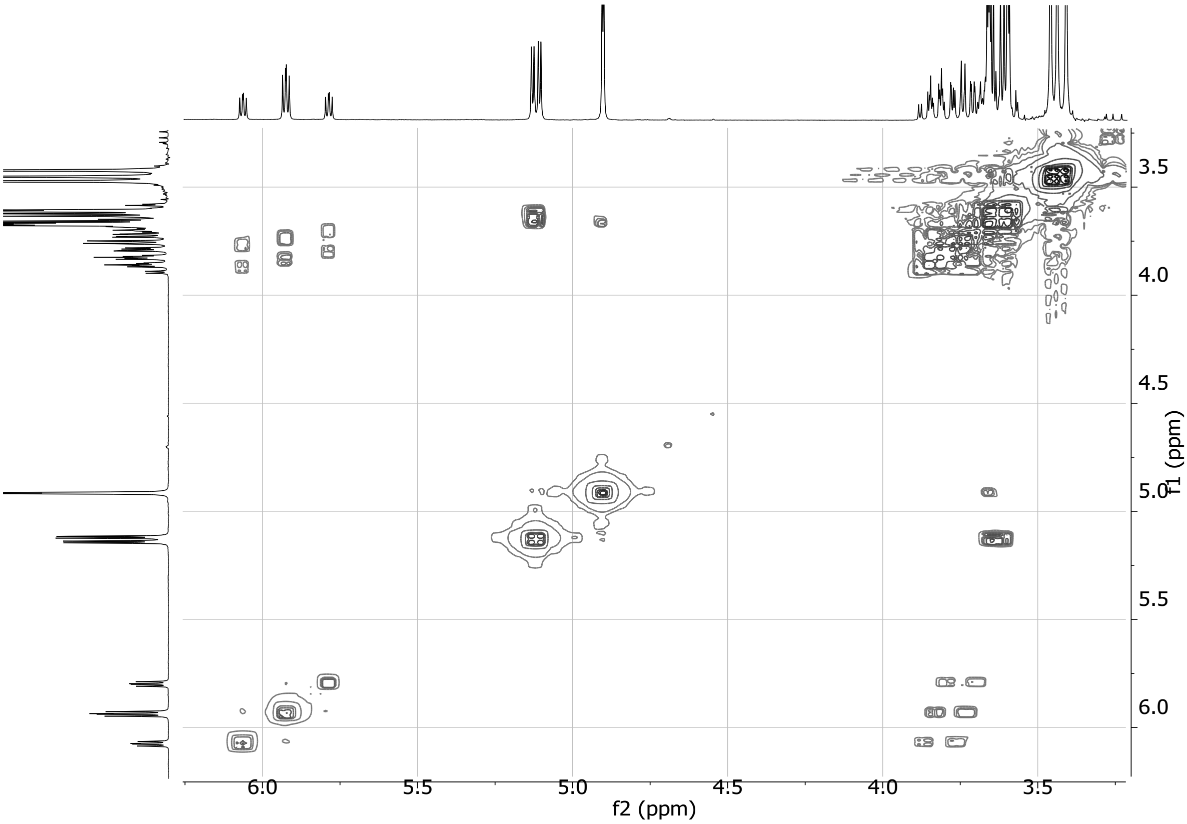

Supplementary Figure 399. HH-COSY NMR, $CDCl_3$ of compound **S103**

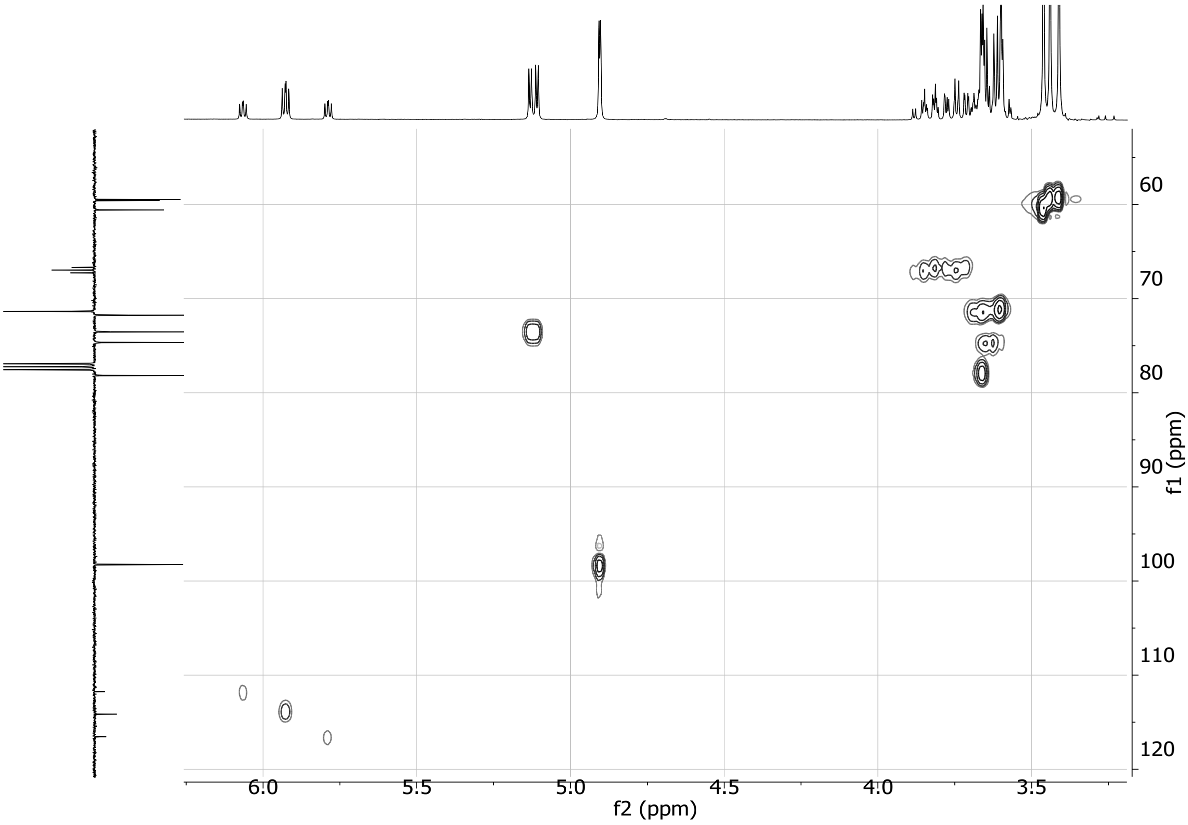

Supplementary Figure 400. HSQC NMR, $CDCl_3$ of compound **S103**

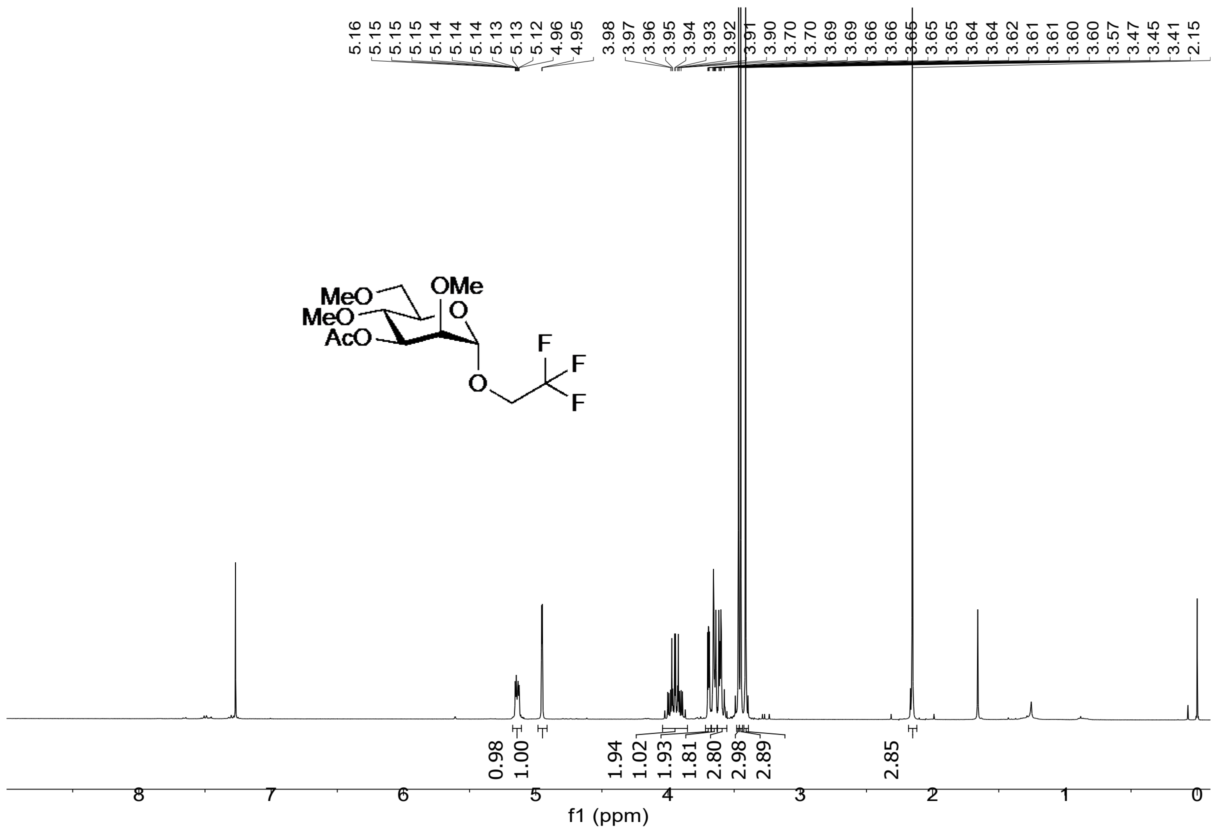

Supplementary Figure 401. ^{1}H NMR, 400 MHz, $CDCl_3$ of compound **S104**

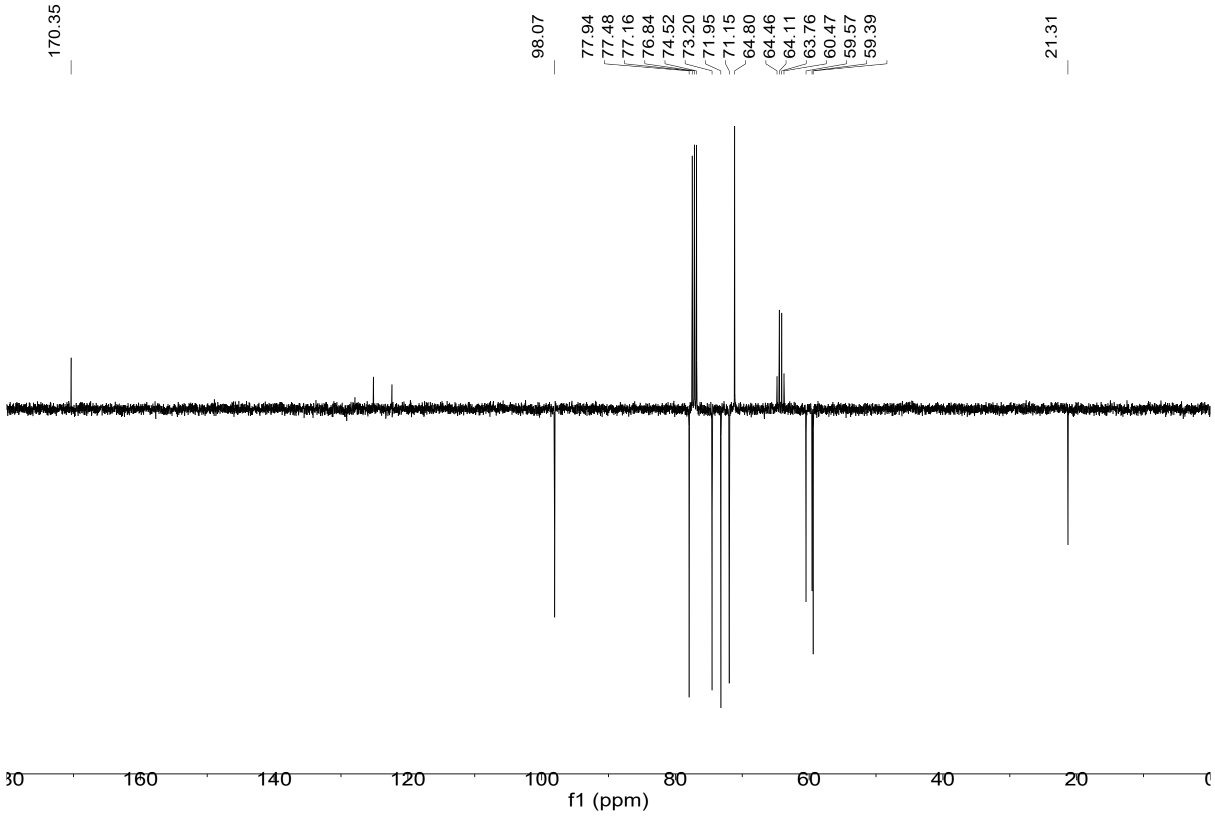

Supplementary Figure 402. ^{13}C NMR, 101 MHz, $CDCl_3$ of compound **S104**

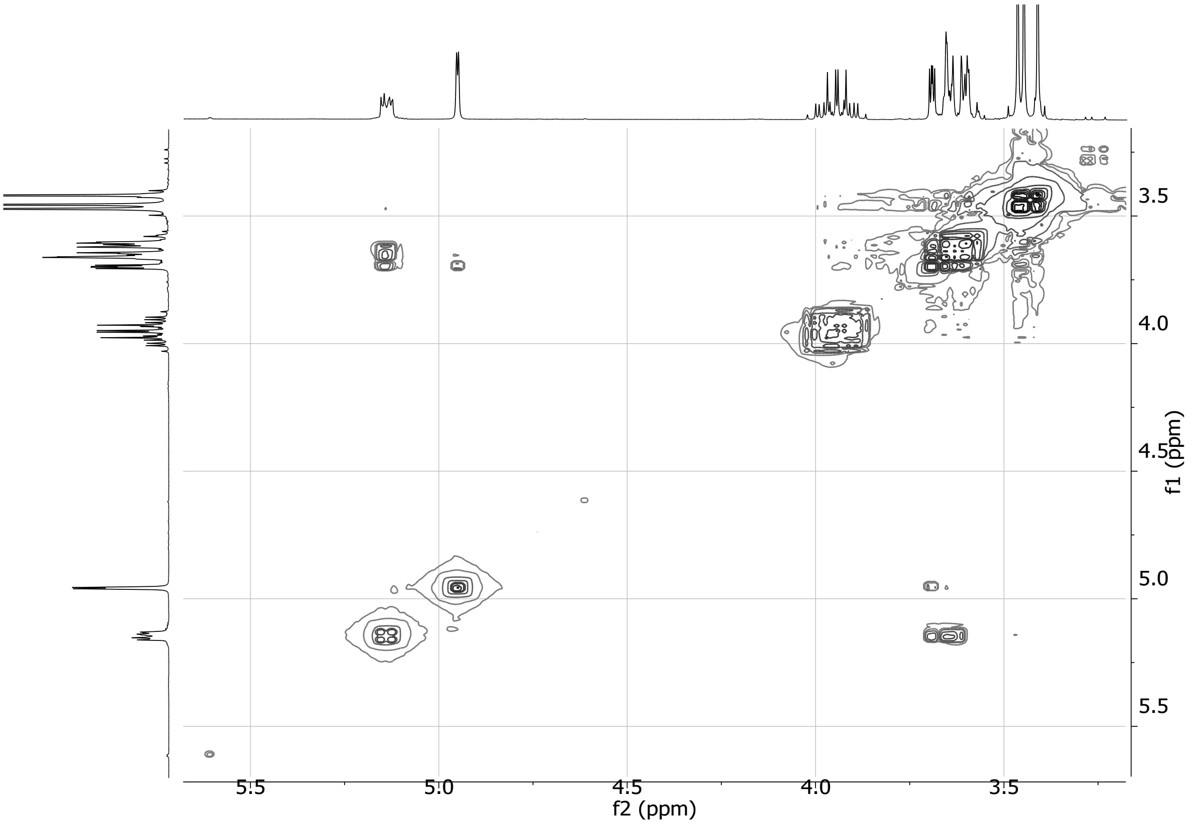

Supplementary Figure 403. HH-COSY NMR, $CDCl_3$ of compound **S104**

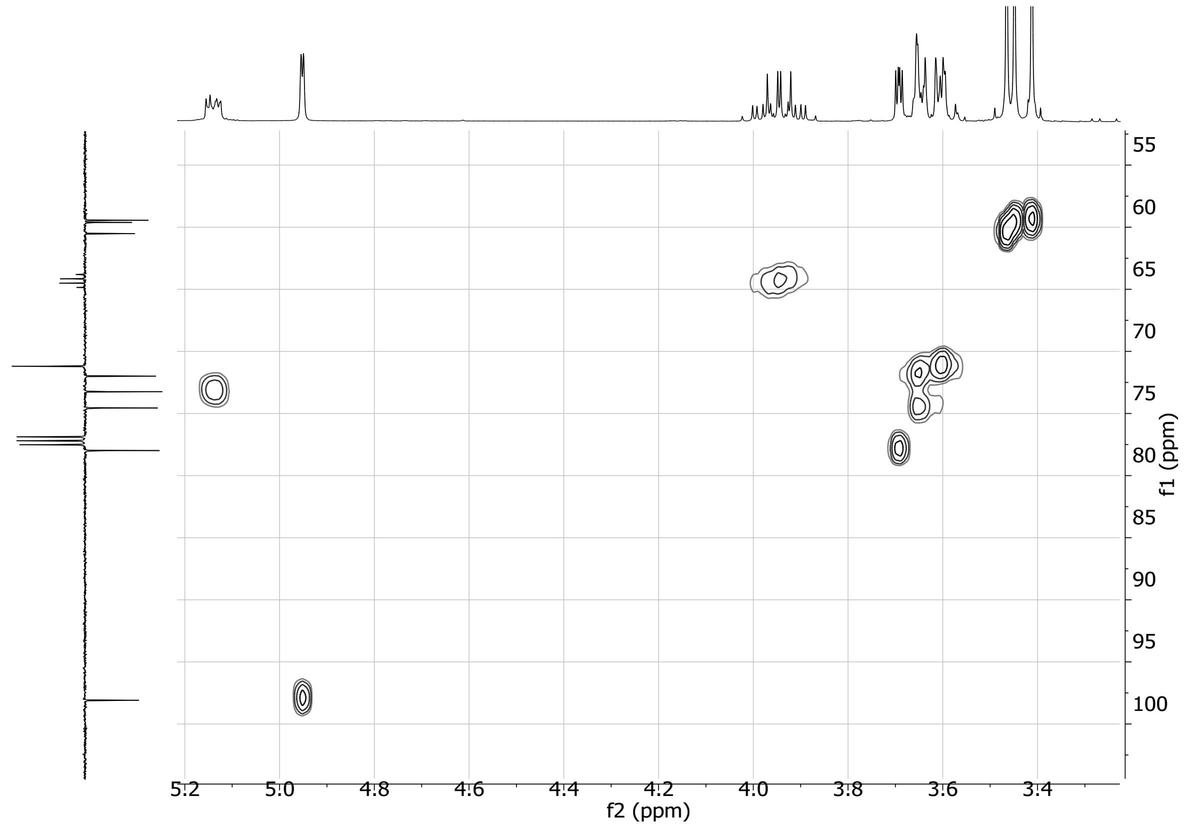

Supplementary Figure 404. HSQC NMR, $CDCl_3$ of compound **S104**

Supplementary References

1. Martens, J., Berden, G., Gebhardt, C. R. & Oomens, J. Infrared ion spectroscopy in a modified quadrupole ion trap mass spectrometer at the FELIX free electron laser laboratory. *Review of Scientific Instruments* **87**, 103108 (2016).
2. van Outersterp, R. E. *et al.* Reference-standard free metabolite identification using infrared ion spectroscopy. *International Journal of Mass Spectrometry* **443**, 77–85 (2019).
3. Landrum, G. (2006). RDKit: Open-source cheminformatics.
4. Tosco, P., Stiefl, N. & Landrum, G. Bringing the MMFF force field to the RDKit: implementation and validation. *Journal of Cheminformatics* **6**, 37 (2014).
5. Ebejer, J.-P., Morris, G. M. & Deane, C. M. Freely Available Conformer Generation Methods: How Good Are They? *J. Chem. Inf. Model.* **52**, 1146–1158 (2012).
6. Gaussian 16, Revision A.03, M. J. Frisch, G. W. Trucks, H. B. Schlegel, G. E. Scuseria, M. A. Robb, J. R. Cheeseman, G. Scalmani, V. Barone, G. A. Petersson, H. Nakatsuji, X. Li, M. Caricato, A. V. Marenich, J. Bloino, B. G. Janesko, R. Gomperts, B. Mennucci, H. P. Hratchian, J. V. Ortiz, A. F. Izmaylov, J. L. Sonnenberg, D. Williams-Young, F. Ding, F. Lipparini, F. Egidi, J. Goings, B. Peng, A. Petrone, T. Henderson, D. Ranasinghe, V. G. Zakrzewski, J. Gao, N. Rega, G. Zheng, W. Liang, M. Hada, M. Ehara, K. Toyota, R. Fukuda, J. Hasegawa, M. Ishida, T. Nakajima, Y. Honda, O. Kitao, H. Nakai, T. Vreven, K. Throssell, J. A. Montgomery, Jr., J. E. Peralta, F. Ogliaro, M. J. Bearpark, J. J. Heyd, E. N. Brothers, K. N. Kudin, V. N. Staroverov, T. A. Keith, R. Kobayashi, J. Normand, K. Raghavachari, A. P. Rendell, J. C. Burant, S. S. Iyengar, J. Tomasi, M. Cossi, J. M. Millam, M. Klene, C. Adamo, R. Cammi, J. W. Ochterski, R. L. Martin, K. Morokuma, O. Farkas, J. B. Foresman, and D. J. Fox, Gaussian, Inc., Wallingford CT, 2016.
7. van Rijssel, E. R. *et al.* Furanosyl Oxocarbenium Ion Stability and Stereoselectivity. *Angew. Chem. Int. Ed.* **53**, 10381–10385 (2014).
8. van der Vorm, S. *et al.* Furanosyl Oxocarbenium Ion Conformational Energy Landscape Maps as a Tool to Study the Glycosylation Stereoselectivity of 2-Azidofuranoses, 2-Fluorofuranoses and Methyl Furanosyl Uronates. *Chemistry – A European Journal* **25**, 7149–7157 (2019).
9. Hansen, T. *et al.* Defining the S_N1 Side of Glycosylation Reactions: Stereoselectivity of Glycopyranosyl Cations. *ACS Cent. Sci.* **5**, 781–788 (2019).
10. Madern, J. M. *et al.* Synthesis, Reactivity, and Stereoselectivity of 4-Thiofuranosides. *J. Org. Chem.* **84**, 1218–1227 (2019).
11. Ribeiro, R. F., Marenich, A. V., Cramer, C. J. & Truhlar, D. G. Use of Solution-Phase Vibrational Frequencies in Continuum Models for the Free Energy of Solvation. *J. Phys. Chem. B* **115**, 14556–14562 (2011).
12. OriginPro, 9.0.0. OriginLab Corporation, Northampton, MA, USA.
13. Gómez, A. M., Casillas, M., Barrio, A., Gawel, A. & López, J. C. Synthesis of Pyranoid and Furanoid Glycals from Glycosyl Sulfoxides by Treatment with Organolithium Reagents. *European Journal of Organic Chemistry* **2008**, 3933–3942 (2008).
14. Baek, J. Y. *et al.* Directing effect by remote electron-withdrawing protecting groups at O-3 or O-4 position of donors in glucosylations and galactosylations. *Tetrahedron* **71**, 5315–5320 (2015).
15. Chaube, M. A., Sarpe, V. A., Jana, S. & Kulkarni, S. S. First total synthesis of trehalose containing tetrasaccharides from Mycobacterium smegmatis. *Org. Biomol. Chem.* **14**, 5595–5598 (2016).
16. Pozsgay, V. Large Scale Synthesis of 2-Azidodeoxy Glucosyl Donors. *Journal of Carbohydrate Chemistry* **20**, 659–665 (2001).
17. Vorm, S. van der, Hansen, T., Overkleeft, H. S., Marel, G. A. van der & Codée, J. D. C. The influence of acceptor nucleophilicity on the glycosylation reaction mechanism. *Chem. Sci.* **8**, 1867–1875 (2017).
18. Deng, S., Gangadharmath, U. & Chang, C.-W. T. Sonochemistry: A Powerful Way of Enhancing the Efficiency of Carbohydrate Synthesis. *J. Org. Chem.* **71**, 5179–5185 (2006).
19. Elferink, H. *et al.* The Glycosylation Mechanisms of 6,3-Uronic Acid Lactones. *Angewandte Chemie International Edition* **58**, 8746–8751 (2019).
20. Blom, P. *et al.* A Convergent Ring-Closing Metathesis Approach to Carbohydrate-Based Macrolides with Potential Antibiotic Activity. *J. Org. Chem.* **70**, 10109–10112 (2005).
21. Elferink, H. *et al.* Direct Experimental Characterization of Glycosyl Cations by Infrared Ion Spectroscopy. *J. Am. Chem. Soc.* **140**, 6034–6038 (2018).
22. Uriel, C., Ventura, J., Gómez, A. M., López, J. C. & Fraser-Reid, B. Methyl 1,2-Orthoesters as Useful Glycosyl Donors in Glycosylation Reactions: A Comparison with n-Pent-4-enyl 1,2-Orthoesters. *European Journal of Organic Chemistry* **2012**, 3122–3131 (2012).
