# Supplementary material for: Characterization of glycosyl dioxolenium ions and their role in glycosylation reactions
Source: Nat Commun. 2020 May 29;11:2664. doi: 10.1038/s41467-020-16362-x (PMC7260182; doi:10.1038/s41467-020-16362-x)
Supplement: Supplementary file 2 — Description of Additional Supplementary Files [file 41467_2020_16362_MOESM2_ESM.pdf]

Description of Additional Supplementary Files

File Name: Supplementary Data 1
Description: xyz coordinates of DFT calculated structures.
