# Supplementary material for: Characterization of glycosyl dioxolenium ions and their role in glycosylation reactions
Source: Nat Commun. 2020 May 29;11:2664. doi: 10.1038/s41467-020-16362-x (PMC7260182; doi:10.1038/s41467-020-16362-x)
Supplement: Supplementary file 3 — Supplementary Data 1 [file 41467_2020_16362_MOESM3_ESM.zip › 237872_2_supp_4529928_q87741.docx]

**Supplementary Data - Cartesian coordinates**

*“Characterization of Glycosyl Dioxolenium Ions and Their Role in Glycosylation Reactions”*

Hansen *et al.*

**Cartesian coordinates (in Å)**

**3-*O*-Acetyl-2,4,6-tri-*O*-methyl-gluco-d-pyranosyl cation**

**Participation_*E*_2_**

8 0.274998000 -0.204621000 -1.691264000

6 0.076002000 0.918185000 0.577165000

6 -1.813484000 -0.486664000 -0.481022000

6 -1.081969000 -0.086482000 0.809327000

6 -0.728769000 -1.028496000 -1.401130000

6 0.522271000 1.010001000 -0.900562000

1 0.925412000 0.592393000 1.191098000

1 -2.555015000 -1.274094000 -0.279154000

1 -1.758569000 0.294173000 1.572729000

1 -1.062400000 -1.569608000 -2.284138000

1 -0.060835000 1.803301000 -1.372414000

8 -2.383060000 0.612192000 -1.132261000

8 -0.526546000 -1.315931000 1.432173000

8 -0.324813000 2.224051000 0.930687000

6 2.006100000 1.296978000 -1.039647000

1 2.201894000 2.313167000 -0.663768000

1 2.277326000 1.265733000 -2.104386000

6 -3.681633000 1.010206000 -0.660053000

1 -4.382955000 0.168566000 -0.705832000

1 -4.013082000 1.798380000 -1.335233000

1 -3.633306000 1.407384000 0.359680000

6 -0.059690000 -2.275044000 0.694164000

6 -0.109616000 2.572149000 2.303252000

1 -0.700942000 1.943164000 2.982258000

1 -0.432961000 3.607919000 2.408219000

1 0.952294000 2.489097000 2.565125000

8 2.728524000 0.329625000 -0.300524000

6 4.143414000 0.454263000 -0.446754000

1 4.490823000 1.437429000 -0.102249000

1 4.443933000 0.310106000 -1.492636000

1 4.595721000 -0.323938000 0.169559000

6 0.600446000 -3.407307000 1.395217000

1 0.473455000 -4.327291000 0.823075000

1 0.212139000 -3.509986000 2.409089000

1 1.673630000 -3.184963000 1.451569000

8 -0.129727000 -2.283775000 -0.575353000


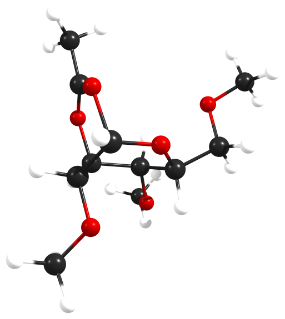


**Participation_*B*_2,5_**

C -3.0892118088 -1.3646302293 -1.7091667590

C -2.2844826270 -0.5892922863 -0.7290977091

O -2.3053539731 0.6812928136 -0.7719311501

C -1.3622512190 1.5786182732 0.1881907697

C -1.2824380996 0.7598515202 1.4688307553

O -0.4747840076 1.4775952079 2.3568331644

C -0.6042555443 1.1192395687 3.7433771119

C -0.6487106427 -0.5761423117 1.0519745605

O -1.5822329597 -1.2672029922 0.1251816982

C 0.7226067201 -0.4162003104 0.3462462451

O 1.7748734398 -0.7088703117 1.2395528995

C 2.1636786202 -2.0865932228 1.2856749600

C 0.9928902885 1.0227011005 -0.1515337535

C 1.8227866543 1.0486617943 -1.4219856861

O 1.1938109606 0.2338702682 -2.3938253253

C 1.8474232408 0.2690519112 -3.6628340547

O -0.2160802646 1.8048406774 -0.4486860580

H -3.9821609731 -0.8035656282 -1.9878932603

H -2.4768497654 -1.5022440155 -2.6093959103

H -3.3428004547 -2.3460136177 -1.3068665045

H -1.9798440938 2.4738774747 0.2182906987

H -2.2944537059 0.6013759696 1.8705453871

H -1.6484818007 1.2031410922 4.0671941198

H -0.2294773855 0.1075311836 3.9329294364

H 0.0065210692 1.8352676674 4.2921952279

H -0.5615093497 -1.2747888242 1.8824915876

H 0.7367703543 -1.1028600625 -0.5098195942

H 2.4868686339 -2.4342663995 0.2968381144

H 2.9981395208 -2.1434626013 1.9848395427

H 1.3500526072 -2.7292530998 1.6480834000

H 1.5107717920 1.5563196358 0.6478608498

H 1.9083308433 2.0873953227 -1.7711650381

H 2.8331493996 0.6798514718 -1.1865992271

H 1.2923103645 -0.3977437240 -4.3240856442

H 1.8394076692 1.2842697291 -4.0802420462

H 2.8850864970 -0.0813230452 -3.5823028082

**Oxocarbenium_*E*_4_/^2^*S*_0_**

8 2.060782000 -1.382627000 -0.081241000

6 -0.219624000 -1.065824000 -0.917745000

6 0.530923000 -0.124107000 1.359495000

6 -0.627024000 -0.257523000 0.339520000

6 1.683955000 -1.021314000 1.062750000

6 1.261236000 -0.978131000 -1.298720000

1 -0.807257000 -0.719151000 -1.778754000

1 -1.428403000 -0.809814000 0.833129000

1 2.286766000 -1.417913000 1.883747000

1 1.479968000 -1.797437000 -1.984652000

8 -1.073411000 1.071599000 0.050649000

8 -0.420625000 -2.441929000 -0.655196000

6 1.866583000 0.305322000 -1.845449000

1 1.271186000 0.594249000 -2.726872000

1 2.890937000 0.095464000 -2.188593000

6 -2.400004000 1.192386000 -0.311544000

6 -1.750331000 -2.939569000 -0.905543000

1 -1.993357000 -2.846963000 -1.970324000

1 -2.500926000 -2.407569000 -0.314468000

1 -1.728667000 -3.991771000 -0.623218000

8 1.866520000 1.306975000 -0.855981000

6 2.442975000 2.541681000 -1.302465000

1 2.403930000 3.229621000 -0.457714000

1 1.868596000 2.957102000 -2.139153000

1 3.486159000 2.395144000 -1.608470000

8 -3.110094000 0.224727000 -0.457848000

6 -2.794909000 2.631645000 -0.470408000

1 -2.888569000 3.090412000 0.519947000

1 -3.756480000 2.686475000 -0.979887000

1 -2.034529000 3.188746000 -1.022980000

1 0.942927000 0.891887000 1.215238000

8 0.084713000 -0.358756000 2.666856000

6 0.551688000 0.568821000 3.661484000

1 1.639685000 0.505911000 3.791351000

1 0.062714000 0.281589000 4.591777000

1 0.269422000 1.593783000 3.393725000


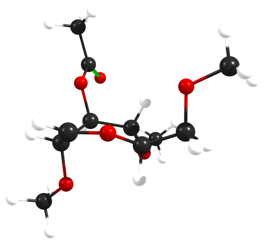


**Oxocarbenium_*B*_2,5_**

C -0.5540701757 -2.3448695312 -3.2952474746

O 0.0491530890 -2.1134683642 -2.0039452990

C -0.8504607158 -1.7364241768 -0.9802723318

C -0.9455723923 -0.2191624485 -0.7542889236

O -1.8544232149 -0.1189070370 0.3586766250

C -2.5980522905 1.0483244607 0.4413759885

C -3.4989899553 1.0390068939 1.6400134608

O -2.4831023002 1.9212759805 -0.3840671863

C 0.4258834810 0.4226882866 -0.4105268136

O 1.0379723251 1.0119011973 -1.5355417676

C 0.7271360656 2.4034284882 -1.7376614063

C 1.4868443022 -0.5906732627 0.0734978268

C 2.4571728975 -0.0746069570 1.1097914060

O 1.7213446200 0.4373145533 2.1941667405

C 2.5416887926 0.9233077544 3.2608861275

O 0.8370178402 -1.8178247757 0.7247073505

C -0.2011024919 -2.3238756579 0.2459796923

H -1.2894836683 -3.1544815988 -3.2373462925

H 0.2650191817 -2.6364455320 -3.9516055846

H -1.0220932371 -1.4300066755 -3.6720839561

H -1.8474746517 -2.1711886254 -1.1144785758

H -1.3560768259 0.2815864459 -1.6339778306

H -2.9053356709 0.9398124699 2.5539436221

H -4.1797699999 0.1836673506 1.5935585906

H -4.0698744817 1.9663046021 1.6665081974

H 0.2585778317 1.1573071142 0.3852879892

H 1.2704252237 2.7013112351 -2.6343096282

H -0.3466940436 2.5571445899 -1.8815622156

H 1.0694939279 3.0004693054 -0.8838515420

H 2.0013418428 -1.0120412155 -0.7911963510

H 3.1307462795 -0.8868518553 1.4228948619

H 3.0691977636 0.6999786331 0.6195956755

H 3.1635029352 0.1189693820 3.6742316032

H 3.1851951796 1.7436184258 2.9177374120

H 1.8640345819 1.2909065569 4.0316156018

H -0.5589720451 -3.2170960125 0.7629944076

**4-*O*-Acetyl-2,3,6-tri-*O*-methyl-gluco-d-pyranosyl cation**


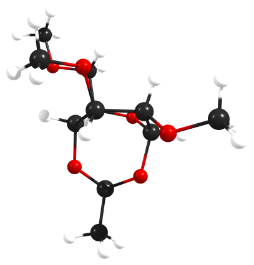


**Participation_*B*_1,4_**

C -2.8885198526 -2.2792491684 -1.8716222778

C -1.8043186124 -1.6671541603 -1.0572777987

O -1.8051932180 -1.8887937369 0.2005293257

C -0.7241996021 -1.3331478512 1.1882021523

C -0.9039600170 0.1886254928 1.2778701391

O -2.2173519499 0.5523624193 1.6128781048

C -2.5136287802 0.5946743598 3.0171765751

C -0.5321574671 0.8625018937 -0.0585256312

O 0.3248650271 1.9412880327 0.2187065012

C 0.2937187983 3.0123576292 -0.7354811264

C 0.1417553101 -0.1544834926 -1.0059293910

O -0.9421088428 -0.9396231718 -1.6872460838

C 1.1100699855 -1.1230923715 -0.3479185919

C 2.4302049742 -0.4683731147 0.0726476369

O 3.0097464154 0.0328978500 -1.1111551322

C 4.3319396624 0.5447246987 -0.9266370741

O 0.5036805564 -1.7644581135 0.8098117621

H -3.7937351355 -1.6729568909 -1.7414021801

H -2.6165260271 -2.2906136155 -2.9267620031

H -3.1056212845 -3.2844399596 -1.5051317751

H -1.0230713803 -1.8677033395 2.0868019234

H -0.1818847436 0.5276537944 2.0357790114

H -2.4540593726 -0.3990885953 3.4776966040

H -3.5366333623 0.9617617421 3.0980724815

H -1.8348711090 1.2838012430 3.5331355651

H -1.4635121422 1.2105707725 -0.5282569462

H 0.6625823767 2.6933648331 -1.7178276404

H 0.9544220433 3.7849207711 -0.3423856560

H -0.7227025602 3.4126374632 -0.8305991375

H 0.6367792293 0.3369429260 -1.8413521453

H 1.3370516645 -1.9291215440 -1.0524758971

H 3.0568648477 -1.2438043257 0.5373384038

H 2.2592672865 0.3312497383 0.8066108804

H 4.6700078165 0.8943106564 -1.9025039038

H 4.3333699276 1.3812711376 -0.2158748382

H 5.0078295379 -0.2416140026 -0.5668918368


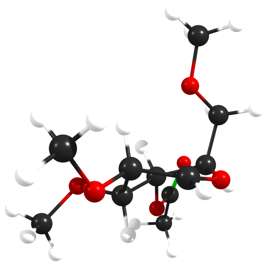


**Oxocarbenium_*E*_3_**

C 2.9354946748 2.2531898615 -1.5554363500

O 2.4303832356 1.7603285480 -0.3014409280

C 1.4957249805 0.7257693797 -0.3976997069

C 0.3326201336 0.9467640818 0.6315331830

O -0.3938996791 2.0856154093 0.2917633860

C -0.1172540408 3.2701470000 1.0606698136

C -0.6204469203 -0.2589385137 0.5986946868

O -1.3125672162 -0.2231981722 1.8560539852

C -2.4810644760 -0.9458675431 1.9156016148

C -3.2283387476 -0.6913508727 3.1899189598

O -2.8105210840 -1.6811888474 1.0101026631

C 0.0418162437 -1.6427450318 0.3826400565

C -0.2959623571 -2.3408615592 -0.9250725724

O 0.1208671629 -1.5037621250 -1.9853640011

C -0.1872704122 -2.0343000972 -3.2834977664

O 1.5537710277 -1.5636960628 0.4113350876

C 2.1588303303 -0.5542135547 -0.0405384888

H 3.6052545170 3.0762552706 -1.3083601460

H 3.4960921665 1.4764572578 -2.0904721942

H 2.1127495665 2.6159379682 -2.1820450914

H 1.0321736739 0.6326773577 -1.3919751096

H 0.7740863378 1.0354370817 1.6338280478

H 0.9179933951 3.5949093073 0.9224306302

H -0.7989184091 4.0323274980 0.6831155898

H -0.3193185216 3.0920911685 2.1229632303

H -1.3355239976 -0.0954697186 -0.2114758239

H -2.5519193412 -0.7073460144 4.0481296334

H -3.6828175765 0.3043930821 3.1456590720

H -4.0124284230 -1.4390134150 3.3045459483

H -0.1581687818 -2.3035716195 1.2245864941

H -1.3824959218 -2.5016544803 -0.9234346200

H 0.2075301365 -3.3177455957 -0.9703077390

H -1.2699278862 -2.1554422297 -3.4036573874

H 0.3119559660 -2.9988259516 -3.4358359852

H 0.1821851859 -1.3125011883 -4.0122211191

H 3.2492150580 -0.6306076792 -0.0150370530

**Oxocarbenium_*E*_3_/^2^S_0_**

8 -0.502117000 1.081900000 1.805005000

6 0.758427000 -0.174641000 -0.010719000

6 -1.760698000 -0.311910000 0.264607000

6 -0.436408000 -1.135899000 0.096451000

6 -1.591913000 0.547808000 1.463783000

6 0.683486000 1.102407000 0.863196000

1 0.843742000 0.134452000 -1.055523000

1 -1.860472000 0.322765000 -0.629463000

1 -0.313830000 -1.772522000 0.983602000

1 -2.392057000 0.638687000 2.203334000

1 1.517056000 1.142651000 1.562554000

8 -2.842403000 -1.171594000 0.474419000

8 -0.491984000 -1.893511000 -1.071106000

8 1.905043000 -0.961689000 0.345774000

6 0.568588000 2.416072000 0.106457000

1 1.472590000 2.502423000 -0.511391000

1 0.541263000 3.256712000 0.815282000

6 -4.050781000 -0.823742000 -0.225136000

1 -4.444726000 0.140950000 0.118410000

1 -4.769449000 -1.609660000 0.004883000

1 -3.869312000 -0.787597000 -1.305366000

6 -0.789336000 -3.292132000 -0.908913000

1 -1.786193000 -3.434501000 -0.481917000

1 -0.034493000 -3.772464000 -0.276067000

1 -0.750152000 -3.719760000 -1.910777000

6 3.122314000 -0.455425000 -0.045547000

8 -0.610616000 2.368004000 -0.671917000

6 -0.809607000 3.541048000 -1.475621000

1 -0.891793000 4.433675000 -0.843794000

1 -1.741834000 3.392241000 -2.020907000

1 0.015189000 3.666068000 -2.186496000

6 4.233918000 -1.432399000 0.193074000

1 5.191273000 -0.922443000 0.091334000

1 4.171406000 -2.234434000 -0.550463000

1 4.144363000 -1.890837000 1.181071000

8 3.215762000 0.655200000 -0.521936000


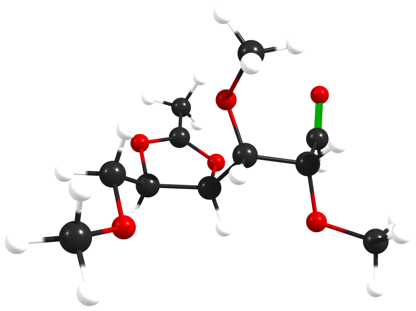


**Ring Opening (*R*)**

O 2.0221231787 -0.2788357894 -2.7878339506

C 1.7260376695 -1.1560047735 -2.0077070799

C 1.7639952228 -0.9700095336 -0.4866895892

O 1.6100889679 -2.1974691072 0.1925339263

C 2.8112290656 -2.9730544057 0.3116895290

C 0.6255502928 -0.0439876489 0.0555219129

O 0.5026081997 1.1923996996 -0.6059881595

C 1.6215564589 2.0897357992 -0.4966657095

C -0.7426259894 -0.7176384581 -0.0325327245

C -1.9679268127 0.0611442485 0.4877749234

C -1.7728253355 1.1167588075 1.5687980201

O -1.2881453231 0.3965374815 2.6801251618

C -1.2368701912 1.1672489991 3.8860579644

O -2.4685135956 0.7141550015 -0.7555738013

C -1.9311782119 0.1258441287 -1.7661213420

C -2.2572105055 0.4918459163 -3.1562184055

O -1.0899385453 -0.8093273791 -1.4905615587

H 1.3993913471 -2.1615950636 -2.3476735546

H 2.7217950758 -0.4916558229 -0.2343945662

H 2.5399694828 -3.8786368006 0.8544541415

H 3.5726337576 -2.4202321971 0.8756027704

H 3.2114520752 -3.2458071359 -0.6724069049

H 0.8275037428 0.0877945026 1.1297663463

H 1.2678451574 3.0582224662 -0.8513526808

H 1.9449216450 2.1800181653 0.5477818405

H 2.4491167819 1.7550289604 -1.1278082757

H -0.6957967857 -1.7387583352 0.3397040933

H -2.7612436048 -0.6297544204 0.7846634200

H -1.0716848952 1.8930059064 1.2361812815

H -2.7443852016 1.5875573530 1.7794542202

H -0.5653847610 2.0281955932 3.7735407882

H -2.2382281116 1.5173773017 4.1659385930

H -0.8534535757 0.5032746794 4.6607752890

H -2.3690477017 -0.4132363699 -3.7590196097

H -1.4060101056 1.0549131241 -3.5600412251

H -3.1570488681 1.1048451070 -3.1917750840


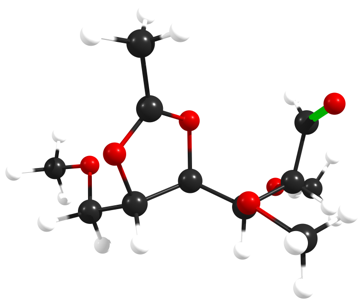


**Ring Opening (*S*)**

O 1.8354088912 2.9276073101 -0.7914772081

C 1.8602549071 1.7826785034 -1.1828725950

C 2.2248014339 0.6002597392 -0.2775362007

O 2.4369457735 -0.5828729579 -1.0179517387

C 3.7351142331 -0.6869429622 -1.6199626356

C 1.1115574016 0.2472175445 0.7589997952

O 0.6463825308 1.3220861824 1.5326563720

C 1.5990280969 1.9387431929 2.4161149827

C -0.1249269513 -0.3648813360 0.1043452868

C -1.2630403118 -0.7046521948 1.0908988834

C -1.9951227788 -2.0031476255 0.8151159217

O -2.3356966838 -2.0180397176 -0.5498385348

C -3.0600987006 -3.1864269425 -0.9533273741

O -2.2158080856 0.4181561361 0.8610949942

C -1.8512361623 1.0690097313 -0.1843394852

C -2.6227554012 2.2125875646 -0.7065143650

O -0.7617543261 0.6785190393 -0.7452327773

H 1.6154118162 1.5113934755 -2.2314847962

H 3.1241001620 0.8828344952 0.2894181342

H 4.5185068417 -0.6769418833 -0.8520422443

H 3.7535990769 -1.6398679716 -2.1490533580

H 3.9126843537 0.1270562354 -2.3332537124

H 1.5419599625 -0.5477100550 1.3936347354

H 2.1328416003 1.1788242060 3.0003577439

H 1.0195964931 2.5713570568 3.0887754891

H 2.3035920834 2.5582068204 1.8544964974

H 0.1294217406 -1.1824947481 -0.5670519558

H -0.9581256653 -0.6159652703 2.1337412782

H -2.8833421067 -2.0689214385 1.4614442948

H -1.3215589395 -2.8357041008 1.0810608348

H -3.2574350043 -3.0761961431 -2.0196130447

H -4.0099869027 -3.2626116058 -0.4098605386

H -2.4647970717 -4.0921444046 -0.7829218178

H -3.6199842820 2.2315362569 -0.2684755826

H -2.0850074666 3.1319804600 -0.4427411483

H -2.6704305584 2.1554674078 -1.7968041308

**6-*O*-Acetyl-2,3,4-tri-*O*-methyl-gluco-d-pyranosyl cation**


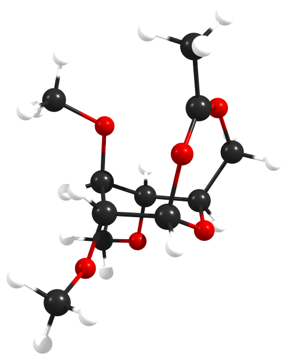


**Participation_^1^*C*_4_**

C 2.7735796254 -0.0487236340 -2.6344338914

C 2.2431761767 -0.3105891475 -1.2650228625

O 2.0744574160 0.7079139380 -0.5112621178

C 1.2519446253 0.8705523454 0.8077047035

C -0.1758754798 1.2462716312 0.3583319364

O -0.8472525308 1.8112298391 1.4568422748

C -0.6677475058 3.2214199347 1.6331744995

C -0.9575608819 0.0088745027 -0.1222043020

O -0.5431894974 -0.3400543780 -1.4441217689

C -1.3820355847 0.1770758222 -2.4848868724

C -0.7383597410 -1.2285609411 0.7815823729

O -1.4605390681 -1.1536030657 1.9914736558

C -2.8669304898 -1.3824030896 1.8951184555

C 0.7268166956 -1.4176751880 1.2181275087

C 1.6780541599 -2.1683058859 0.3051351123

O 2.0264258414 -1.5470602276 -0.9843339701

O 1.3807287624 -0.1958606226 1.6251110314

H 3.4483234729 0.8086854088 -2.6144759206

H 1.9233131232 0.2029914035 -3.2789344486

H 3.2690668888 -0.9333493170 -3.0333700436

H 1.7869913415 1.7065580368 1.2534012934

H -0.0961755370 1.9603763371 -0.4734597644

H -0.9892433033 3.7702338688 0.7386875334

H 0.3730525220 3.4792308530 1.8666033831

H -1.2960717523 3.5015424668 2.4786865291

H -2.0215885080 0.2660580513 -0.1090580182

H -2.4081240069 -0.1926127925 -2.3743773799

H -0.9725570143 -0.1864697827 -3.4286597770

H -1.3891911238 1.2745228957 -2.4905548709

H -1.0424800315 -2.1131253997 0.1992079225

H -3.3859735861 -0.5607224434 1.3873350976

H -3.2319142869 -1.4443594847 2.9206605262

H -3.0761313957 -2.3279443432 1.3764236697

H 0.6847529058 -2.0169679476 2.1331160740

H 1.2729991998 -3.1364230909 0.0097710557

H 2.6352585683 -2.3089265532 0.8125613726

**Oxocarbenium_*E*_3_/^2^*S*_0_**

8 -0.613368000 -0.657362000 -1.336163000

6 1.082969000 -1.455903000 0.204816000

6 0.838565000 1.047700000 -0.368595000

6 1.547859000 -0.022677000 0.547943000

6 -0.006425000 0.439689000 -1.431848000

6 -0.411727000 -1.519881000 -0.112762000

1 1.279400000 -2.096961000 1.075074000

1 0.114919000 1.584091000 0.274437000

1 2.617547000 0.065806000 0.319403000

1 -0.129892000 0.942263000 -2.393534000

1 -0.681111000 -2.497998000 -0.510621000

8 1.781054000 1.908335000 -0.951813000

8 1.292099000 0.196091000 1.910269000

8 1.733494000 -1.915429000 -0.961484000

6 -1.363302000 -1.093003000 1.002369000

1 -1.074037000 -0.149290000 1.462650000

1 -1.334768000 -1.876412000 1.764279000

6 1.355661000 3.272022000 -1.119228000

1 0.509355000 3.350175000 -1.813000000

1 2.210840000 3.804543000 -1.533888000

1 1.075186000 3.710055000 -0.153617000

6 1.996652000 1.296430000 2.502462000

1 3.075985000 1.197694000 2.338175000

1 1.780204000 1.257486000 3.569855000

1 1.652546000 2.257476000 2.099995000

6 3.025075000 -2.509125000 -0.756550000

1 3.373149000 -2.817686000 -1.742051000

1 2.945308000 -3.385852000 -0.103334000

1 3.738197000 -1.793853000 -0.330432000

8 -2.720627000 -1.032309000 0.545415000

6 -3.169997000 0.185892000 0.119853000

8 -2.424788000 1.140429000 -0.008112000

6 -4.645282000 0.168352000 -0.143215000

1 -4.937908000 1.087092000 -0.650040000

1 -5.179213000 0.092160000 0.810098000

1 -4.918909000 -0.705784000 -0.739814000


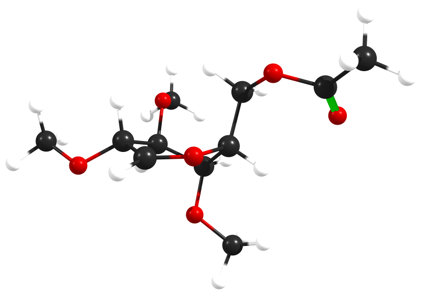


**Oxocarbenium_*E*_3_**

C -4.0651098326 1.1900390844 -1.1023973602

O -3.1842311827 1.1808344442 0.0354028663

C -1.8379055688 0.9661791979 -0.2589673206

C -1.4158025420 -0.5040559080 -0.5692277109

O -0.9500221353 -0.5280759661 -1.9026844526

C -1.0936348122 -1.7873190450 -2.5783659855

C -0.3458640605 -0.9825798946 0.4348537570

O -1.0331017606 -1.1856659391 1.6492697809

C -0.4281732450 -2.1000974650 2.5787471811

C 0.7722521182 0.0514320472 0.6291233452

C 1.6401571008 0.4015955539 -0.5743874811

O 2.7079288684 1.2831631341 -0.2212290018

C 3.7846793945 0.6976859289 0.4076422940

C 4.9068113908 1.6729960748 0.6007011022

O 3.7665108115 -0.4693314653 0.7331402713

O 0.1161509377 1.3170437435 1.1542363524

C -1.0586350205 1.6158222753 0.8268185089

H -3.7652536161 1.9662651017 -1.8166095336

H -4.0816544585 0.2136739882 -1.5986658812

H -5.0554040451 1.4130710464 -0.7075737497

H -1.5190385706 1.5502821152 -1.1541528211

H -2.3039581075 -1.1325026857 -0.4301007151

H -0.7441534566 -1.6251053998 -3.5979178412

H -0.4881458714 -2.5730612275 -2.1112459335

H -2.1451304494 -2.0974338246 -2.5958446239

H 0.1189815128 -1.9072647001 0.0650174537

H 0.5453505686 -1.7410859884 2.9325008958

H -0.3120961759 -3.0891667939 2.1207310696

H -1.1137162004 -2.1664906028 3.4231754890

H 1.4078379956 -0.2131640408 1.4726547935

H 1.0786146201 0.9055945408 -1.3606275323

H 2.0361057637 -0.5384403246 -0.9709705682

H 5.3525525511 1.9081709114 -0.3716434796

H 4.5360421345 2.6094055788 1.0257808182

H 5.6629001770 1.2312946748 1.2487630808

H -1.4917448336 2.4660918301 1.3597529321


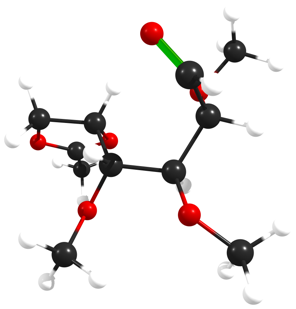


**Ring Opening (*S1*)**

O -2.4351723723 1.5082428808 -1.3162102265

C -2.7114517094 0.6016057251 -0.5571307386

C -1.9875221183 0.3929317699 0.7792126913

O -1.3700444644 1.5619898197 1.2645224262

C -2.2754325330 2.5539753847 1.7693146737

C -0.8803221471 -0.6981577269 0.6328275340

O -1.4747222830 -1.9659805641 0.4285248400

C -1.7854125789 -2.6940315961 1.6214512124

C 0.0807822447 -0.4858317105 -0.5621437666

O 1.1867543464 -1.3706105897 -0.5102309238

C 1.0525280191 -2.6185931755 -1.2158919907

C 0.6802485100 0.9193645576 -0.6578818193

O 1.6007461616 1.1007856663 0.5094666164

C 2.8132431025 1.1080928071 0.0931807761

C 3.9599966796 1.1040209113 1.0224906617

O 2.9802540085 1.1192591673 -1.1842582754

C 1.6535451529 1.1028278737 -1.8365917073

H -3.4857160104 -0.1473033481 -0.8134878587

H -2.7206214472 0.0066200923 1.5053949142

H -1.6574567982 3.3495113387 2.1868799559

H -2.9042944928 2.9621876880 0.9712787808

H -2.9044739742 2.1307228281 2.5626746292

H -0.2904120829 -0.6802059816 1.5594594159

H -0.8761295189 -2.8960335407 2.2011731742

H -2.2302890367 -3.6360105913 1.2989591930

H -2.5063947772 -2.1579591225 2.2509335705

H -0.4907458580 -0.6379883367 -1.4912400844

H 2.0154139029 -3.1215273310 -1.1195430706

H 0.8385710302 -2.4413896141 -2.2777573200

H 0.2608856342 -3.2305717670 -0.7808925585

H -0.0708515962 1.7017890410 -0.5713882213

H 4.8335678033 1.5539545066 0.5497199488

H 4.1892171681 0.0586278952 1.2661263576

H 3.6939601022 1.6204234139 1.9463038030

H 1.6650946024 0.2696705464 -2.5370156114

H 1.5526573303 2.0557910820 -2.3561310018


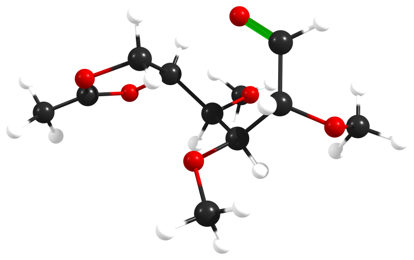


**Ring Opening_(*S2*)**

O -1.9188140858 0.7655577466 1.7776296959

C -2.5078000239 0.7640208485 0.7123682281

C -1.8193357019 1.0238716744 -0.6218522379

O -2.7030305502 0.8527084390 -1.7109360412

C -3.1883426027 -0.4721092906 -1.9589159429

C -0.4082535619 0.3744660245 -0.8455856332

O 0.6334392932 1.3426674427 -0.8968723626

C 0.7329860281 2.0476604062 -2.1430960210

C 0.0701201869 -0.7236316226 0.1326294650

O -0.9671236610 -1.6756172127 0.1962286040

C -0.5605212303 -3.0224449331 0.4589953018

C 0.5211247264 -0.3053643932 1.5654713948

O 1.9234866632 -0.8583906366 1.7614548305

C 2.7239080042 0.0947596766 2.0805556483

C 4.1650803141 -0.1384924728 2.3132464739

O 2.2005665506 1.2581442111 2.2100960162

C 0.7270291212 1.1649248814 1.9836435374

H -3.6081164006 0.6660827609 0.6695214856

H -1.6388099145 2.1093766535 -0.5995664668

H -2.3878883680 -1.1474581711 -2.2815947165

H -3.6765547598 -0.9021060663 -1.0763860866

H -3.9196401779 -0.3716205872 -2.7612491693

H -0.4784458195 -0.1290110601 -1.8181405066

H 0.9251993722 1.3479244596 -2.9655863551

H 1.5722974797 2.7365171640 -2.0428706274

H -0.1797841894 2.6149883743 -2.3579495631

H 0.9593243193 -1.1630948300 -0.3436688442

H -1.4631807155 -3.6314707365 0.4071705394

H 0.1588563232 -3.3664682076 -0.2950034167

H -0.1187844749 -3.1272402678 1.4585304742

H -0.0846070096 -0.8417300664 2.2924727552

H 4.5062841040 -1.0009776172 1.7396580546

H 4.3112042121 -0.3489106925 3.3804379136

H 4.7331620685 0.7584399780 2.0611068823

H 0.2610774720 1.4090609946 2.9349654181

H 0.5033870084 1.8950671282 1.2146912723


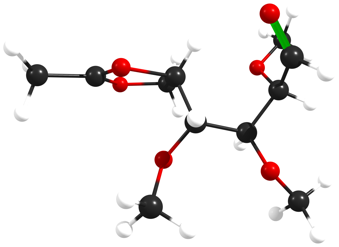


**Ring Opening_(*R1*)**

O -0.3287132381 -1.5203420011 -2.5710792995

C -1.2665096591 -1.5689799699 -1.8021167514

C -1.7679806201 -0.3465903340 -1.0254529289

O -1.3281735464 0.8825082673 -1.5624682116

C -1.9553790927 1.2606584663 -2.7990713708

C -1.2780872392 -0.3833685124 0.4518148241

O -1.8530247084 -1.4721890424 1.1462719955

C -3.1289300794 -1.2136084477 1.7439877713

C 0.2555399985 -0.5313725565 0.6325893980

O 0.6479645970 -0.1213156257 1.9320491441

C 0.7223419319 -1.1530875973 2.9321513233

C 1.0845005095 0.3122347650 -0.3406363094

O 2.5215749602 -0.0188034478 -0.0574424265

C 3.1069638723 1.0141984777 0.4282588268

C 4.4964869916 0.9647994641 0.9261233844

O 2.4171103686 2.0992876430 0.4620808948

C 1.0684984454 1.8314268565 -0.0951293013

H -1.7983515231 -2.5184290160 -1.6000447500

H -2.8687283771 -0.3911505358 -1.0043070499

H -1.6886302096 0.5700548475 -3.6047744886

H -3.0449221178 1.2965924119 -2.6777486758

H -1.5889814550 2.2594836259 -3.0390135822

H -1.5794370997 0.5721729995 0.9045879165

H -3.0571779365 -0.4005517955 2.4770806131

H -3.8883415258 -0.9615113466 0.9937119223

H -3.4224529309 -2.1350830633 2.2476862538

H 0.5166448825 -1.5854894071 0.4627704843

H 1.0761952740 -0.6641846383 3.8406849887

H 1.4348872013 -1.9310747684 2.6306875885

H -0.2568065623 -1.6031972805 3.1074283034

H 0.9196120196 0.0357059325 -1.3796509072

H 5.0550917422 0.1875430088 0.4026794668

H 4.4646870799 0.7116719775 1.9935170514

H 4.9714137633 1.9406874445 0.8165299597

H 0.3579037322 2.1562518773 0.6610452197

H 0.9832105514 2.4250513209 -1.0030012774


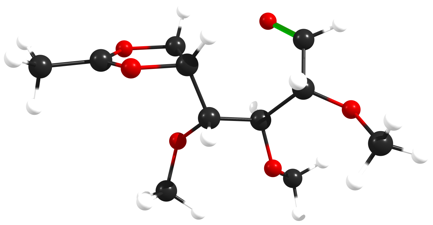


**Ring Opening_(*R2*)**

O -1.4077893685 1.9549846259 1.9226019658

C -2.1895115165 1.8767791015 0.9919998070

C -1.9086666000 1.0515135662 -0.2582879665

O -3.1523914229 0.7912267842 -0.8529735857

C -3.1838305253 0.8179526273 -2.2860665757

C -1.1085496342 -0.2628024395 0.0916343887

O -1.4282830743 -1.2918160973 -0.8153593289

C -2.4646963867 -2.1884334780 -0.3865915856

C 0.4332990576 -0.1103896360 -0.0029882358

O 1.0927730608 -1.2774436969 0.4581131918

C 1.4576324244 -2.2490497529 -0.5387402438

C 1.0297804354 1.0299946357 0.8304898959

O 2.4848105304 1.0864666985 0.4631184768

C 3.1891020189 0.7128776211 1.4725622277

C 4.6507346848 0.5245330078 1.3765724238

O 2.5526229530 0.5117257521 2.5684003146

C 1.1085857769 0.7861780378 2.3490548775

H -3.1634633868 2.3992857666 1.0016677259

H -1.2954622930 1.6850728863 -0.9274980670

H -2.8516067975 1.7959481228 -2.6591037181

H -4.2256885833 0.6636382726 -2.5688851998

H -2.5622519057 0.0231694290 -2.7091755096

H -1.3617689870 -0.5718503440 1.1152909256

H -3.4106032426 -1.6580062082 -0.2455308394

H -2.1740317382 -2.6936766305 0.5432526074

H -2.5733948238 -2.9282088708 -1.1809829162

H 0.6808126134 0.0845532810 -1.0574791893

H 2.1241828536 -1.8008211155 -1.2868827525

H 1.9861038899 -3.0398047587 -0.0045108705

H 0.5727155917 -2.6548132517 -1.0309279240

H 0.6380722210 2.0113565531 0.5787878801

H 4.8398501796 -0.5178529824 1.0900034959

H 5.1180817949 0.7064678144 2.3452892150

H 5.0643342198 1.1755488400 0.6049702840

H 0.5778573939 -0.1000184741 2.6866389645

H 0.8607385862 1.6554143127 2.9530358404

**3-*O*-Acetyl-2,4,6-tri-*O*-methyl-manno-d-pyranosyl cation**


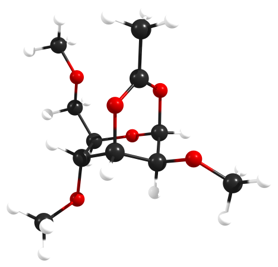


**Partcipation_^1^*C*_4_**

C 0.2734209825 2.7598126585 -1.7339607247

C -0.1702185106 1.6404019093 -0.8684051175

O -0.5024132854 1.8955177570 0.3408135523

C -1.0115114543 0.7696722000 1.2654513685

C -1.7983188747 -0.1811065081 0.3655819618

O -2.8502103799 0.4599272055 -0.3065301364

C -4.0893800412 0.5328519916 0.4180815270

C -0.7917804470 -0.6774358216 -0.6543589566

O -0.2643828699 0.4733242078 -1.4115994260

C 0.3556358484 -1.4543845120 0.0287273272

O -0.1791580097 -2.7029310974 0.4326066287

C -0.0802194670 -3.7576169741 -0.5342846591

C 0.9224490106 -0.7575365284 1.2919647143

C 2.2897064647 -0.1174087492 1.1002968258

O 2.2779981824 0.7735936261 -0.0045590691

C 3.5478345412 1.3977770445 -0.2191598764

O 0.0274560805 0.2101869780 1.9301446667

H -0.6193644985 3.2105979336 -2.1853929781

H 0.9157261276 2.3877936954 -2.5323947530

H 0.7773442048 3.5182015167 -1.1343401722

H -1.6060889001 1.3457265752 1.9715207229

H -2.1346346282 -1.0368465682 0.9662612431

H -3.9941881963 1.1419764589 1.3246565041

H -4.8042045194 1.0044551817 -0.2557400352

H -4.4409256767 -0.4715458526 0.6813960685

H -1.2631970249 -1.2851436724 -1.4253141145

H 1.1699230919 -1.5897024128 -0.6945381685

H -0.4995784207 -4.6436550788 -0.0575502395

H 0.9680789035 -3.9446960860 -0.7967496198

H -0.6521190173 -3.5379268173 -1.4446517776

H 1.0295835427 -1.5417628217 2.0449952630

H 3.0235097200 -0.9217885091 0.9325690363

H 2.5655997755 0.4091994261 2.0242596036

H 3.8369189795 2.0047869740 0.6479231212

H 4.3235129613 0.6461423813 -0.4124105300

H 3.4470958050 2.0398422887 -1.0954097809


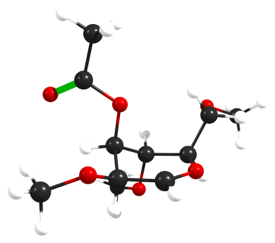


**Oxocarbenium_^3^*E***

C -3.2237368229 0.3648406238 2.2929256695

O -2.1934232102 1.1963701394 1.7172382037

C -0.9541578931 0.5850929990 1.5451562892

C -0.7857092766 -0.3057655498 0.2902974967

O -1.1177721028 0.4426674448 -0.8770096254

C -2.3675076176 0.1972889347 -1.4409799601

C -2.5927415717 1.0642244237 -2.6429625633

O -3.1202225286 -0.6230338702 -0.9789807511

C 0.6867718575 -0.7583411011 0.2165822136

O 0.9787279813 -1.5538615158 1.3536878697

C 0.7867577505 -2.9688420438 1.1780387126

C 1.6766542983 0.4065796562 0.2070246164

C 2.0311484139 0.9882379407 -1.1779185924

O 2.6636820195 -0.0202402351 -1.9269220181

C 4.0972385427 -0.0036423489 -1.8923875218

O 1.1973870309 1.5926147098 1.0207560919

C 0.0563893201 1.6727929677 1.5411515338

H -4.0720119828 1.0267445157 2.4626089084

H -3.5132976918 -0.4299710932 1.5997112590

H -2.8867052845 -0.0532643674 3.2489701831

H -0.6621841049 -0.0509110589 2.4109715564

H -1.4416606463 -1.1741019980 0.3721140802

H -3.5567984018 0.8182592563 -3.0862764722

H -2.5786603502 2.1187165903 -2.3500618122

H -1.7945871712 0.9114698668 -3.3754575509

H 0.8538880149 -1.3098382792 -0.7178160580

H -0.2581778718 -3.2110525664 0.9518691395

H 1.0666136263 -3.4309081338 2.1246267793

H 1.4349179215 -3.3466765383 0.3792889844

H 2.5781734947 0.1156970548 0.7498807086

H 2.6673038494 1.8729074604 -1.0465889679

H 1.1181783351 1.2811104708 -1.7021100980

H 4.4823730656 0.9405684277 -2.2948598704

H 4.4258381710 -0.8283343067 -2.5250490457

H 4.4839769493 -0.1553935720 -0.8760198689

H -0.1665661135 2.6278950962 2.0247004806

**4-*O*-Acetyl-2,3,6-tri-*O*-methyl-manno-d-pyranosyl cation**


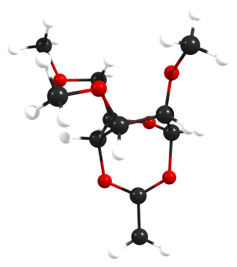


**Participation_^5^*S*_1_**

C -2.1478218445 -3.1070065065 -2.2447487714

C -1.4485238365 -2.1968164643 -1.2972525586

O -1.7424645425 -2.2756402978 -0.0567823197

C -0.9001678746 -1.5633778128 1.1016370884

C -1.3148496634 -0.0898941627 1.0764186550

O -0.6608549128 0.4596723083 2.1868974917

C -1.3497188510 1.5372558921 2.8450143686

C -0.8936843454 0.5407366041 -0.2952957753

O -0.3924400689 1.8273293760 -0.0692122478

C -0.4721722567 2.7200930875 -1.1887869797

C 0.1578032955 -0.3599227602 -0.9936191564

O -0.5715947335 -1.3933869629 -1.8036848893

C 1.1299539890 -1.0771999411 -0.0737667864

C 2.1417418171 -0.1411162227 0.5953501373

O 2.8812617446 0.4317517743 -0.4639271337

C 3.9685591706 1.2453454003 -0.0199523876

O 0.4036642286 -1.8411880393 0.9324543446

H -2.1496546059 -2.6892498300 -3.2515323819

H -3.1609400882 -3.3068698644 -1.8918288701

H -1.6037082174 -4.0601066029 -2.2603366182

H -1.2824966890 -2.1144245107 1.9570325654

H -2.4054528982 -0.0306264021 1.1911886405

H -0.7477063272 1.7830703374 3.7198934623

H -1.4263200968 2.4081467484 2.1893192118

H -2.3462974339 1.2129787210 3.1695320536

H -1.7752926936 0.5838937699 -0.9543276297

H 0.1501125786 2.3820711615 -2.0260438931

H -0.0962471964 3.6803061219 -0.8357680839

H -1.5115460890 2.8324301609 -1.5222535943

H 0.7139963787 0.1866573743 -1.7527999273

H 1.6834564221 -1.8203041518 -0.6546576290

H 1.6369114140 0.6198773702 1.2020498291

H 2.7788806726 -0.7507980059 1.2529471402

H 4.4613349902 1.6251040123 -0.9155785596

H 3.6079186569 2.0867615711 0.5860186882

H 4.6843599069 0.6546467464 0.5663025164


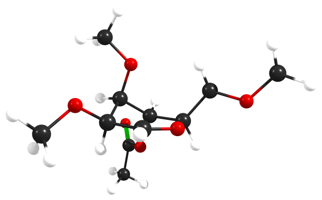


**Oxocarbenium_^3^*E***

C 3.0297180824 -0.2036762506 2.7783060171

O 2.5530997840 -0.4030593183 1.4355277724

C 1.1968751454 -0.1250251571 1.2299044847

C 0.9437153424 0.5402208513 -0.1477289486

O 1.3998745767 -0.2719527390 -1.1972074315

C 2.7052983273 0.0428939197 -1.7259380160

C -0.5812937304 0.6973245179 -0.3144008403

O -1.1058014247 1.5298699024 0.7345844436

C -1.1728008643 2.8916196619 0.4539974780

C -1.8414691192 3.6424912128 1.5667513103

O -0.7431094941 3.3414982796 -0.5787782103

C -1.3296285903 -0.6318210641 -0.2484006001

C -1.5237718819 -1.3733201964 -1.5636065028

O -2.3579051592 -2.4709321753 -1.3037117615

C -2.6480925509 -3.2522165343 -2.4658043732

O -0.6463119026 -1.6300213988 0.6789525425

C 0.4264211704 -1.4042301281 1.2876341368

H 2.5319358787 -0.8801826518 3.4845385998

H 4.0954990625 -0.4282881748 2.7565135636

H 2.8749165105 0.8359685516 3.0907534883

H 0.7727535569 0.5446444469 2.0007558742

H 1.4112388668 1.5309940990 -0.1547333671

H 3.4800840165 -0.1304240879 -0.9751423337

H 2.8509147186 -0.6264962345 -2.5737136462

H 2.7327470688 1.0820928878 -2.0718902055

H -0.7673369000 1.1688740555 -1.2829813521

H -1.6901850800 4.7113471744 1.4194830923

H -1.4546715629 3.3313565698 2.5404202137

H -2.9151615701 3.4257684123 1.5536037935

H -2.2943129739 -0.5111354670 0.2486200829

H -0.5548207402 -1.6704600668 -1.9882444632

H -1.9949835056 -0.6590256392 -2.2601249995

H -3.2961758460 -4.0645424131 -2.1369835235

H -1.7294341606 -3.6689423339 -2.8990057034

H -3.1702947080 -2.6518703549 -3.2222587635

H 0.8122696569 -2.2594421569 1.8501081484


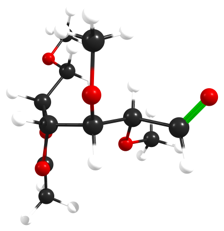


**Ring Opening_(*R1*)**

O -0.5911468129 1.1923452796 3.7866939592

C -1.2624541869 0.8375958868 2.8481229014

C -0.6458865707 0.4083437346 1.5067517022

O -1.2034138820 -0.8135831992 1.0347162333

C -0.9219189814 -1.9465620413 1.8758095678

C -0.9942667264 1.4719538664 0.4450934773

O -0.5548765245 2.7693549492 0.7618213639

C 0.8402059872 3.0139211892 0.9835682363

C -0.6845312762 1.1131877436 -1.0412208032

C 0.4370016047 0.1732617781 -1.5657931503

C 1.6299828227 -0.2415078118 -0.7234524739

O 2.5542332932 -0.8016500096 -1.6196271172

C 3.7468589930 -1.2797146561 -0.9907069462

O -0.3278324497 -1.0562007189 -1.9432981410

C -1.5797591298 -0.7840043091 -1.9280186617

C -2.6078982853 -1.7735317643 -2.3031283057

O -1.8957111147 0.4092187068 -1.5621517149

H -2.3715409752 0.7969896811 2.8944683790

H 0.4348637717 0.3077592394 1.6668690354

H -1.3851587684 -1.8364700787 2.8618845071

H 0.1597425267 -2.0822703957 1.9994078847

H -1.3454362640 -2.8184658112 1.3764240117

H -2.0881447756 1.5620305149 0.4461277744

H 1.2268186012 2.4490129725 1.8374590618

H 0.9125951647 4.0786013298 1.2054259828

H 1.4375970315 2.8053032047 0.0855240772

H -0.6601167886 2.0641318230 -1.5762288215

H 0.8202243679 0.5457916760 -2.5197287056

H 1.3255067260 -0.9624942792 0.0473908174

H 2.0431930857 0.6494896411 -0.2246683808

H 4.2758811102 -0.4639927133 -0.4807451939

H 4.3754989871 -1.6777114805 -1.7869990287

H 3.5196662260 -2.0774793149 -0.2715588952

H -3.3738721602 -1.2935124467 -2.9177308152

H -3.0911502835 -2.1242077104 -1.3830300961

H -2.1544543435 -2.6152344759 -2.8256717219


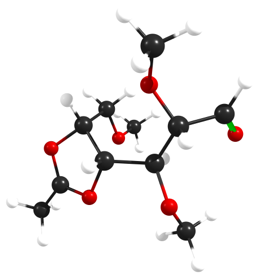


**Ring Opening_(*R2*)**

O -1.6231896937 0.8855348883 2.8826095357

C -1.4213442940 -0.3072027210 2.8404667970

C -0.3209802925 -0.9505469231 1.9866310711

O -0.9025857088 -1.9711493151 1.1717817003

C -0.9692072993 -3.2718746121 1.7797313412

C 0.3909453500 0.1044585670 1.1119256176

O 1.4577001229 0.6193687597 1.8797250878

C 1.5129297282 2.0503926040 2.0023012923

C 0.9740534257 -0.5527533547 -0.1488907410

C 0.0506845978 -0.9866036471 -1.3060721777

C -1.2782752837 -0.2732568298 -1.4870918841

O -1.0271338032 1.1156472211 -1.5331799446

C -2.2043269571 1.9014836066 -1.7628309930

O 0.8925470815 -0.6013048252 -2.4917560625

C 1.7421744827 0.2852758499 -2.1044836704

C 2.5440663924 1.0651344848 -3.0674453478

O 1.8513890103 0.4500251276 -0.8357660903

H -2.0593907413 -1.0170479095 3.4019887926

H 0.4414049084 -1.3820328624 2.6532765123

H -1.6124221962 -3.2713889758 2.6656245426

H -1.3985173750 -3.9405907807 1.0324907947

H 0.0335767012 -3.6197311984 2.0577137968

H -0.3035142936 0.8981020236 0.8226658175

H 0.5896738117 2.4343205236 2.4472834647

H 2.3552110106 2.2581912409 2.6627795469

H 1.6886636223 2.5247377267 1.0298497766

H 1.6634756654 -1.3528379641 0.1325680959

H -0.0692941289 -2.0659277797 -1.3562974199

H -1.9347951138 -0.5520358251 -0.6512348141

H -1.7429411336 -0.6259980255 -2.4202605351

H -2.9322643266 1.7575320622 -0.9552714856

H -2.6636642009 1.6401709167 -2.7242062231

H -1.8849146857 2.9435650169 -1.7816269236

H 3.4516672400 1.4395802843 -2.5938461396

H 1.9348871697 1.9157037887 -3.3986944900

H 2.7735112071 0.4530588567 -3.9420586410


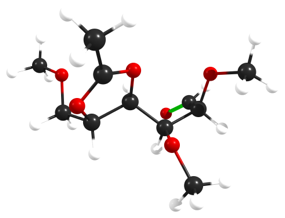


**Ring Opening_(*S1*)**

O -0.3422399595 0.8111073373 3.2519510586

C 0.0732269578 -0.3267335687 3.1941564796

C 0.9825441948 -0.8418092529 2.0701620662

O 0.4956657458 -2.1160510721 1.7172666742

C 1.4824508119 -3.1134773010 1.4266553327

C 1.0436382202 0.1620137573 0.8973662031

O 2.0351585341 -0.1316050289 -0.0712087119

C 3.3709556641 0.2558371768 0.2750876658

C -0.2727772978 0.2967160158 0.1316103721

C -0.2824693077 1.4406645277 -0.9091747467

C -1.5221944549 2.3135004090 -0.8841635932

O -2.6389682323 1.4572764786 -0.9088159155

C -3.8960010719 2.1432301577 -0.8556902035

O -0.2651000714 0.6973063802 -2.1979516500

C -0.4315368137 -0.5554922474 -1.9569221465

C -0.5852502907 -1.5376417917 -3.0484876318

O -0.4824072511 -0.9005503505 -0.7235692154

H -0.1907770283 -1.0912451181 3.9492709274

H 1.9934088101 -0.9155465911 2.5071083256

H 2.0579552664 -2.8545740261 0.5310397117

H 0.9352988016 -4.0416368446 1.2573414285

H 2.1610338012 -3.2488916557 2.2783078789

H 1.2188711960 1.1535521451 1.3461443983

H 3.4202845661 1.3271373114 0.5079062687

H 3.7514552432 -0.3188923544 1.1272395812

H 3.9878648954 0.0435133046 -0.5984479415

H -1.1367303748 0.3404973881 0.7921265840

H 0.6340279604 2.0311870917 -0.9016379171

H -1.5104959497 2.9984838461 -1.7451903781

H -1.4896064730 2.9216259770 0.0355617089

H -4.0145520193 2.8059466662 -1.7218094857

H -4.6693469539 1.3753405474 -0.8751236942

H -3.9819420164 2.7263667801 0.0694936017

H 0.0600037000 -1.2655134386 -3.8862243061

H -0.3655067726 -2.5418839909 -2.6869930244

H -1.6258420301 -1.5000586652 -3.3943857056


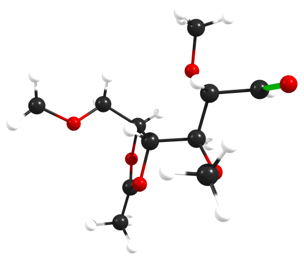


**Ring Opening_(*S2*)**

O 3.7831704427 -1.8027772111 -1.8134089390

C 3.0708095471 -0.8292428928 -1.8660420724

C 2.0830252740 -0.4579769609 -0.7496461696

O 2.1047038245 0.9358264923 -0.4750777387

C 3.3288743221 1.3971655925 0.1231159123

C 0.6430265024 -0.7906652562 -1.2121694278

O 0.4624802280 -2.1293323056 -1.5906978722

C 0.8053857495 -3.1681222023 -0.6554684142

C -0.3771700103 -0.3333273956 -0.1392261972

C -0.7357438555 1.1711421461 -0.1382570694

C -0.8410486369 1.8091934819 1.2327063758

O -1.6484921711 0.9709813232 2.0278002290

C -1.8682045559 1.4677270752 3.3538120952

O -2.1116105536 1.1584083326 -0.7318270129

C -2.5357183511 -0.0543727022 -0.7755098743

C -3.9235535275 -0.3831847035 -1.1556208929

O -1.6860456322 -0.9644271632 -0.4619936186

H 3.0847764783 -0.1311546470 -2.7301105126

H 2.3494377464 -1.0344781385 0.1479867924

H 3.2013679415 2.4651307277 0.3022765400

H 4.1821741426 1.2449548158 -0.5467503712

H 3.5153753675 0.8794073758 1.0719219014

H 0.4436254400 -0.2173161675 -2.1276126121

H 1.8873007535 -3.2352673954 -0.5110564323

H 0.4498041626 -4.0914814557 -1.1125465224

H 0.3001461682 -3.0350481180 0.3091493415

H -0.1397303612 -0.6927760185 0.8617332271

H -0.1219866226 1.7608196135 -0.8158184599

H 0.1779095246 1.9072307955 1.6388612603

H -1.2700950409 2.8175362096 1.1363459649

H -0.9184012425 1.5735847933 3.8926493015

H -2.3848150322 2.4350101326 3.3264520335

H -2.4941816654 0.7336600776 3.8612461876

H -4.5383163052 -0.3486520147 -0.2469191957

H -3.9718979134 -1.3890271966 -1.5741791875

H -4.3063821380 0.3608509601 -1.8562185697

**6-*O*-Acetyl-2,3,4-tri-*O*-methyl-manno-d-pyranosyl cation**


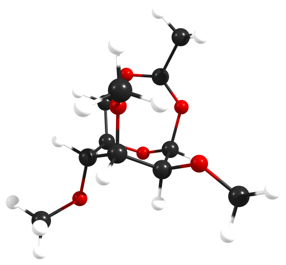


**Participation_^1^*C*_4_**

C 2.4336208985 -0.6702088814 -2.6123337955

C 1.8956640591 -0.8650497176 -1.2359747940

O 2.0848310379 0.0961548527 -0.4132056800

C 1.4199584885 0.4060067732 0.9312475179

C 0.1163244160 1.1755319465 0.6442995228

O 0.3135260728 2.2734542256 -0.2080679130

C 0.7696210179 3.4753849331 0.4273683411

C -0.9673146223 0.2566937666 0.0607777458

O -0.6760289810 -0.1517312895 -1.2695962340

C -1.3368097302 0.6015007481 -2.3015556808

C -1.0890207690 -1.0048701915 0.9466443097

O -1.6646171180 -0.6679254261 2.1970598281

C -3.0957531073 -0.6803570518 2.2328416630

C 0.2696637721 -1.6515901867 1.2828768199

C 0.9131739979 -2.5871475142 0.2739347759

O 1.3681383664 -2.0146041719 -1.0019478741

O 1.2693510725 -0.7018387077 1.7099226771

H 3.5287152788 -0.6837294824 -2.5612735045

H 2.1309823119 0.3113858112 -2.9826311341

H 2.0933357255 -1.4652802607 -3.2740844983

H 2.1913948077 1.0287456077 1.3798786264

H -0.2296567228 1.4921309833 1.6407004174

H 0.0818957322 3.7792672401 1.2260117255

H 0.7868484857 4.2419381944 -0.3476336054

H 1.7812094495 3.3633098278 0.8368495463

H -1.9222082642 0.7942514300 0.0854533889

H -0.9973930981 1.6404387952 -2.3056463707

H -1.0826959104 0.1166224699 -3.2454732554

H -2.4240602030 0.5627765802 -2.1641554612

H -1.7023757353 -1.7463262107 0.4143157527

H -3.5308354918 0.0639393512 1.5540800963

H -3.4828425909 -1.6755120778 1.9793859160

H -3.3760170188 -0.4330140387 3.2569898330

H 0.0939905710 -2.2729151366 2.1670058343

H 0.2280116329 -3.3750408255 -0.0400340565

H 1.8076721684 -3.0325923659 0.7156695192


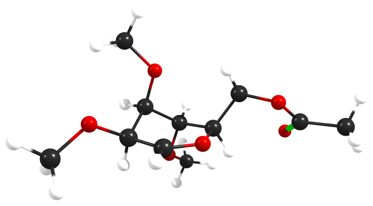


**Oxocarbenium_^3^*E***

C -4.1486598486 1.3276449392 1.0709372789

O -3.0846831094 1.2551982183 0.1041420004

C -1.9979484473 0.4480160336 0.4648027789

C -1.4687074132 -0.3994806249 -0.7210611611

O -1.0713263759 0.4031448379 -1.8027794713

C -2.0584667777 0.6234772030 -2.8309027377

C -0.2245478235 -1.1490792396 -0.2060580242

O -0.6456367996 -1.9185875928 0.9030558790

C 0.1111997791 -3.1188856028 1.1407921328

C 0.8962093223 -0.1789007556 0.2032017084

C 1.8311070113 0.3152170763 -0.8979229684

O 2.9567380924 1.0227028962 -0.3732562581

C 3.9407098107 0.2479199355 0.1910920089

C 5.1429156719 1.0641477601 0.5615453624

O 3.7990944299 -0.9479194508 0.3388842511

O 0.3320496647 1.0749488963 0.8590684219

C -0.8952707217 1.3251620077 0.9554972019

H -4.9225253332 1.9490447454 0.6215903754

H -3.8081338136 1.7919372385 2.0048705983

H -4.5471717654 0.3277783315 1.2790610448

H -2.2329023681 -0.2636310213 1.2776288857

H -2.2401603009 -1.1254787546 -1.0010716675

H -2.8983454426 1.2067981012 -2.4462428641

H -1.5483105730 1.1768925759 -3.6193672285

H -2.4116596195 -0.3349626737 -3.2282927209

H 0.1580908975 -1.7853272825 -1.0149020665

H 1.1649563437 -2.8981334088 1.3474061476

H -0.3375269554 -3.5915425172 2.0141148911

H 0.0381846925 -3.7920905918 0.2788159513

H 1.4783650478 -0.6040713804 1.0206492629

H 1.3214003868 1.0023039810 -1.5715735790

H 2.1665850348 -0.5645398301 -1.4552299112

H 5.6685930988 1.3639990964 -0.3514159365

H 4.8454336924 1.9777469094 1.0825596498

H 5.8101291053 0.4671359507 1.1822671788

H -1.1201785932 2.3016139929 1.3939935848


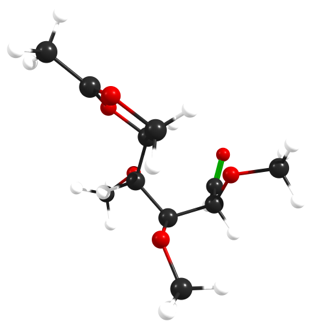


**Ring Opening_(*S1*)**

O -2.7003282354 1.4685761221 -0.8512532932

C -2.3732097993 1.4684479997 0.3136787833

C -1.8450366359 0.2180378565 1.0313185002

O -1.9030002064 -0.9417112451 0.2366762202

C -3.2218713199 -1.4722016728 0.0254321792

C -0.3775220955 0.4108070710 1.4972529434

O 0.1491563327 -0.7315480617 2.1427989332

C -0.3816755043 -1.0178043747 3.4399847011

C 0.6114964421 0.7659802916 0.3544350308

O 0.4134579769 2.0965852972 -0.0749568564

C 1.1966721468 3.0862456803 0.6021802176

C 0.4669256828 -0.0987233589 -0.9015286249

O 1.6441925641 0.2257400510 -1.7877457986

C 2.3229747771 -0.8414596653 -2.0046148298

C 3.5330579243 -0.8400662859 -2.8529962703

O 1.8819069325 -1.9208540312 -1.4626189769

C 0.6085439138 -1.6237461813 -0.7437690635

H -2.4691878063 2.3744490577 0.9461221396

H -2.4671414976 0.0981031961 1.9366482893

H -3.8272273768 -0.7956766496 -0.5832128448

H -3.0923392713 -2.4206915565 -0.4979440353

H -3.7141464366 -1.6595328059 0.9879690435

H -0.3620947112 1.2713866695 2.1842011117

H -0.3255674633 -0.1332106426 4.0875896992

H 0.2402238887 -1.8100720070 3.8578782525

H -1.4182846899 -1.3697560294 3.3850652319

H 1.6227025037 0.6250561174 0.7655394818

H 2.2679386142 2.9022911917 0.4529687207

H 0.9787132173 3.1154269438 1.6769697529

H 0.9262958480 4.0434215915 0.1560779183

H -0.4086980036 0.1989549923 -1.4761036081

H 4.2407281003 -1.5907249634 -2.4964905265

H 3.9800372067 0.1542441632 -2.8724911508

H 3.2347634837 -1.1100723580 -3.8742407403

H 0.7346067967 -1.9565816270 0.2840102245

H -0.1670632990 -2.1893207763 -1.2532307555


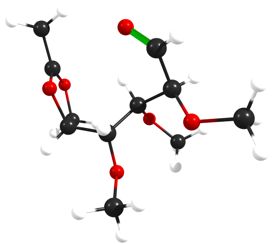


**Ring Opening_(*S*2)**

O -1.2299823306 0.7517576241 -2.2004924586

C -1.8962883784 0.0368053765 -1.4772028591

C -1.7874738285 0.0263426373 0.0556513958

O -2.0711168282 -1.2765024696 0.5330059254

C -3.4515670648 -1.5338613418 0.8163373134

C -0.3842981857 0.4917766016 0.4862199783

O -0.3056443843 1.1521337240 1.7347732229

C -1.0032804425 0.6032582808 2.8655277795

C 0.6938230022 -0.6269036014 0.3325430062

O 0.9507512564 -1.2647702266 1.5640548054

C 0.9679992855 -2.6990054798 1.5173927637

C 2.0570346362 -0.0765743571 -0.1355426483

O 1.9503935252 0.3189235778 -1.5737633717

C 2.0723905147 1.5962183617 -1.6769299332

C 1.8388216475 2.2958140051 -2.9525322935

O 2.3791550495 2.2204360045 -0.5934402936

C 2.5421075589 1.2357561516 0.5104569451

H -2.6176836555 -0.6881348741 -1.9006429071

H -2.5168720771 0.7598503287 0.4387047629

H -3.8200260776 -0.8554856550 1.5966001546

H -4.0783296442 -1.4307931310 -0.0778218295

H -3.5070923834 -2.5627691884 1.1728101185

H -0.1531027867 1.2814292463 -0.2367317277

H -0.7233888975 1.2349323113 3.7095226778

H -0.7111003785 -0.4295818047 3.0564697343

H -2.0882339532 0.6713727258 2.7253191648

H 0.3634984161 -1.3478111309 -0.4260825317

H 1.7526676144 -3.0705735142 0.8451249546

H 1.1835322449 -3.0336512201 2.5325173005

H -0.0072006109 -3.0812521805 1.1976957348

H 2.8054022380 -0.8682804501 -0.1067450166

H 2.1682164113 3.3320490987 -2.8883137046

H 0.7607731270 2.2491833691 -3.1571016021

H 2.3501145185 1.7660491939 -3.7610421719

H 1.9157614916 1.5748539665 1.3353824100

H 3.6003393694 1.2470080402 0.7703752006


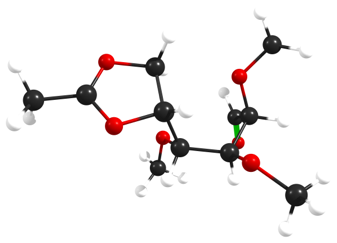


**Ring Opening_(*R1*)**

O -3.0890495596 1.4739752090 -0.8356448516

C -2.6903473192 0.3740557321 -0.5220698828

C -1.9516787571 0.1109111338 0.7936832515

O -1.5079273510 -1.2337444160 0.8786439339

C -2.4560976704 -2.1366545082 1.4631449967

C -0.7547481459 1.0688920487 0.9643289646

O -0.2223927130 1.0351692729 2.2743903465

C -0.8532777768 1.9081622808 3.2174708725

C 0.4484472541 0.7814155593 0.0266499300

O 0.1070296198 0.6965936494 -1.3415060381

C -0.0249234887 1.9526854070 -2.0324361381

C 1.2012495946 -0.4998279310 0.3973309841

O 2.6327271342 -0.2958272589 -0.0371663961

C 2.9559981203 -1.1920928682 -0.8975864657

C 4.2749214933 -1.1960463491 -1.5641883785

O 2.0647481999 -2.0784206212 -1.1580384225

C 0.8369282490 -1.7885031528 -0.3645138553

H -2.8387222043 -0.5142640064 -1.1704722973

H -2.6566478776 0.3340357006 1.6089825158

H -3.3884518681 -2.1668996077 0.8867837265

H -1.9991608515 -3.1274983426 1.4529091664

H -2.6744027390 -1.8476950076 2.4981917768

H -1.1247166334 2.0744994818 0.7229750954

H -0.7891139568 2.9520321387 2.8863277795

H -0.3120154088 1.7887428748 4.1564301258

H -1.9056592425 1.6413977668 3.3746651606

H 1.1557312899 1.6056774154 0.1983777648

H 0.8793841794 2.5586897662 -1.8995045408

H -0.9043844391 2.5057386003 -1.6896114510

H -0.1491718325 1.7049328767 -3.0868146556

H 1.2468312740 -0.6198912860 1.4778723499

H 5.0236212297 -0.7339679224 -0.9192524480

H 4.5555531573 -2.2149582257 -1.8347653307

H 4.1947703008 -0.6033104473 -2.4843766257

H 0.0350593228 -1.6647804178 -1.0843786639

H 0.6795894161 -2.6531245452 0.2747677003


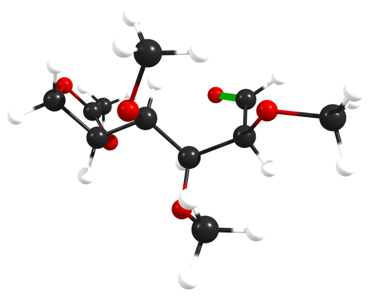


**Ring Opening_(*R2*)**

O 0.9012997344 -2.2327688630 1.3297834271

C 0.1555186519 -1.6803828286 2.1150950462

C -1.0214317805 -0.7817476672 1.7107205643

O -0.9902051637 0.3652492559 2.5489573398

C -1.7779745511 0.2712031237 3.7421045678

C -0.9610095372 -0.4429664579 0.2112647719

O -2.1723110007 -0.0196451564 -0.3796427685

C -2.9442425108 1.0142754892 0.2515365254

C 0.2344218177 0.4470128640 -0.2339040235

O 0.0406138323 1.8435385954 -0.1318318792

C 0.6929532499 2.5084486165 0.9631835789

C 0.4685250933 0.2699323457 -1.7458976070

O 0.7452794663 -1.1722641154 -2.0418158150

C 1.9970796248 -1.3148271393 -2.2962116073

C 2.6207356378 -2.6450386196 -2.4211110254

O 2.6750512904 -0.2241841054 -2.4106171316

C 1.7461981422 0.9300125962 -2.2759896555

H 0.3210964121 -1.7604124632 3.2069528644

H -1.9525259407 -1.3512322256 1.8700306076

H -2.8364749271 0.1167307983 3.4961001798

H -1.4362004610 -0.5411460316 4.3943793747

H -1.6606894936 1.2208446807 4.2653567587

H -0.7714362289 -1.4196403011 -0.2475183976

H -3.7573616470 1.2280881597 -0.4436657911

H -2.3540936526 1.9163380042 0.4225057950

H -3.3747417448 0.6639574995 1.1960104343

H 1.1422086905 0.1335612597 0.3024203780

H 1.7783416391 2.3413209199 0.9309422280

H 0.4887671903 3.5714037295 0.8303685767

H 0.2937115789 2.1626191586 1.9191227345

H -0.4340493424 0.5235722914 -2.3011021023

H 3.6153240600 -2.5633496777 -2.8579997611

H 1.9771104210 -3.2964609353 -3.0185094592

H 2.6879589016 -3.0805786599 -1.4154369051

H 1.6596178154 1.3661305901 -3.2716954500

H 2.2031347326 1.6323052692 -1.5822863736

**3-*O*-Acetyl-2,4,6-tri-*O*-methyl-galacto-d-pyranosyl cation**


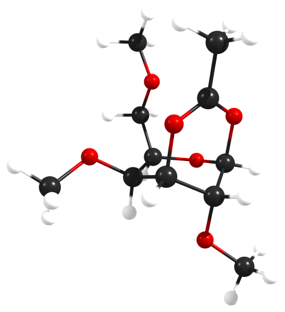


**Participation_^1^*C*_4_**

C -2.5947418014 -1.5741365482 -1.3608587284

C -1.7638828027 -0.6825218333 -0.5149733570

O -1.9699135789 0.5829770397 -0.5710103353

C -1.1039176601 1.5479165837 0.2717778292

C -0.9243973304 0.8268585852 1.6143946605

O -0.1460294354 1.5541147132 2.5220117305

C -0.8384757744 2.5912597162 3.2379327530

C -0.1526440892 -0.4452164607 1.2687272973

O -0.9614987756 -1.2426120818 0.3268791624

C 1.2228780717 -0.0877459694 0.6763122213

O 1.9453661692 -1.2100280960 0.2402912810

C 2.7058365174 -1.8802650715 1.2531151091

C 1.1593043236 0.9376513575 -0.4916091034

C 1.2416872431 0.3838133433 -1.9071193951

O 0.1610742997 -0.4850289922 -2.2119988601

C 0.2811048529 -1.0527991073 -3.5186954123

O 0.0302366003 1.8662686939 -0.3885547297

H -2.0674323657 -2.5097905688 -1.5467397225

H -3.5210862986 -1.7967961621 -0.8166027646

H -2.8534330252 -1.0676922088 -2.2905633741

H -1.7446479458 2.4273220405 0.2936428373

H -1.9136735752 0.5850940936 2.0323784660

H -1.7121234555 2.1826083843 3.7597222189

H -1.1457553865 3.4043001579 2.5708419160

H -0.1261370238 2.9771401352 3.9664733595

H -0.0440873935 -1.1081548048 2.1262414585

H 1.7387571373 0.4053397448 1.5117192827

H 2.0599926808 -2.3353612418 2.0143797691

H 3.4097130124 -1.1898472305 1.7342399900

H 3.2595463546 -2.6690954637 0.7436977031

H 2.0265011991 1.5950955238 -0.3867328654

H 1.2447377159 1.2386381618 -2.5986879045

H 2.1964052140 -0.1490325323 -2.0014427008

H -0.5844112040 -1.6986654033 -3.6743199321

H 1.1961366863 -1.6526175835 -3.5981078703

H 0.2890108433 -0.2687909144 -4.2865619898

**Oxocarbenium_^3^*H*_4_**

8 -1.581672000 1.409846000 -0.153016000

6 -0.326206000 -0.730545000 0.125410000

6 0.840004000 1.471146000 0.278180000

6 0.905747000 0.074682000 -0.340305000

6 -0.552286000 2.029150000 0.203475000

6 -1.574861000 -0.089499000 -0.469494000

1 0.895905000 0.152656000 -1.432909000

1 -0.708901000 3.094535000 0.390434000

1 -1.562930000 -0.093203000 -1.562567000

8 2.140980000 -0.502213000 0.077686000

6 -2.908263000 -0.607442000 0.028776000

1 -3.059258000 -0.330009000 1.081745000

1 -2.866614000 -1.709354000 -0.023586000

6 2.652129000 -1.482987000 -0.756141000

8 -3.899665000 -0.080438000 -0.812581000

6 -5.227721000 -0.453485000 -0.435957000

1 -5.895902000 0.014834000 -1.158558000

1 -5.466585000 -0.091267000 0.572542000

1 -5.355019000 -1.543245000 -0.474161000

8 2.028499000 -1.884460000 -1.709887000

6 4.015914000 -1.921977000 -0.313413000

1 4.742126000 -1.138621000 -0.556242000

1 4.287764000 -2.836529000 -0.839468000

1 4.046976000 -2.077031000 0.767972000

1 1.119312000 1.407526000 1.342226000

8 1.624311000 2.393458000 -0.442291000

6 2.487778000 3.237837000 0.343381000

1 1.911340000 3.904748000 0.995544000

1 3.051573000 3.833345000 -0.373874000

1 3.169058000 2.624766000 0.943433000

1 -0.261079000 -1.748231000 -0.282382000

8 -0.462951000 -0.727720000 1.532962000

6 0.099797000 -1.851529000 2.233910000

1 -0.222240000 -1.749708000 3.270511000

1 1.192303000 -1.842654000 2.185162000

1 -0.286033000 -2.792443000 1.824257000


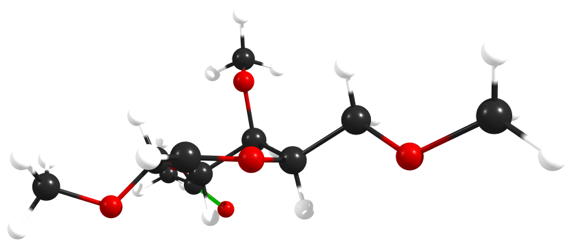


**Oxocarbenium_*E*_3_**

C 0.9012348803 -4.0326943543 -0.5318972018

O 0.0353529702 -2.9224285866 -0.8375003473

C 0.5022065113 -1.6504876151 -0.4513741143

C -0.5822963096 -0.8494081698 0.2690991927

O -0.6370546235 -1.3417940305 1.6062160193

C -1.8489474210 -1.1700773155 2.2545119851

C -1.8546020252 -1.8461470302 3.5929920014

O -2.7514161261 -0.5488195159 1.7452824624

C -0.2233593075 0.6499352075 0.1908371819

O 1.0883435816 0.9080359664 0.6520631710

C 1.2167448757 1.3080319400 2.0284619041

C -0.3044035012 1.0901822339 -1.2664218072

C 0.2073058737 2.4795162754 -1.5864275662

O -0.1875246122 2.7757130341 -2.8997697967

C 0.2647619217 4.0519878302 -3.3594470616

O 0.5308405984 0.1435624820 -2.1350505477

C 0.8924828696 -0.9901985801 -1.7425458801

H 1.8451397051 -3.9695661448 -1.0863571097

H 1.1013217709 -4.0685642120 0.5445500260

H 0.3590394473 -4.9249848259 -0.8430110649

H 1.3996144099 -1.6968682741 0.1864038327

H -1.5500603037 -1.0162288877 -0.2162518076

H -1.9250479027 -2.9296781021 3.4480995324

H -0.9289959266 -1.6456281102 4.1383769430

H -2.7173831913 -1.5053705174 4.1644113669

H -0.9776483142 1.2257439013 0.7437500982

H 0.5714668622 2.1686648848 2.2404230498

H 2.2596513632 1.5971579534 2.1602488056

H 0.9748216545 0.4836958710 2.7051963494

H -1.3062801832 0.9608841724 -1.6841423279

H -0.2486603436 3.1682032774 -0.8539728332

H 1.2975806645 2.5275550428 -1.4541142435

H -0.1428301825 4.8599895805 -2.7379622882

H 1.3613716907 4.1045912262 -3.3568522661

H -0.1022955677 4.1596430425 -4.3800451937

H 1.4792241906 -1.5541496499 -2.4720804640

**4-*O*-Acetyl-2,3,6-tri-*O*-methyl-galacto-d-pyranosyl cation**


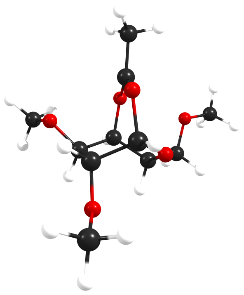


**Participation_^1^*S*_5_**

C -0.1862664833 2.2378429561 -2.5003249682

C -0.2339552397 1.3431157978 -1.3150951712

O -0.7682777380 1.7991381406 -0.2434252893

C -0.6778831003 1.0698512541 1.1322265879

C -1.6856161468 -0.0888384913 1.0687518010

O -1.6981618350 -0.7668052817 2.3032241617

C -2.4941757885 -0.1665019131 3.3364690993

C -1.2532590401 -1.1005168956 -0.0193768268

O -2.1293101383 -0.9627142458 -1.1103336881

C -2.1456046841 -2.0643326952 -2.0292158488

C 0.2314084576 -0.8739148398 -0.4106118813

O 0.3047689471 0.1822571779 -1.4438810421

C 1.0715586069 -0.5267162620 0.8161263599

C 2.5735323858 -0.4108700812 0.6101374236

O 2.8562869435 0.5154826043 -0.4129638158

C 4.2556856091 0.7621348560 -0.5663907190

O 0.6067260856 0.7272721820 1.3836945565

H -0.2012111921 1.6539320320 -3.4208405346

H -1.0100969432 2.9514116607 -2.4660232360

H 0.7578489278 2.7955781732 -2.4566600311

H -0.9601211316 1.8897277928 1.7889687272

H -2.6758305335 0.3032470089 0.8028760859

H -2.0657420248 0.7809705200 3.6842108222

H -3.5210192389 -0.0051764773 2.9870155786

H -2.4980399481 -0.8770044193 4.1629305062

H -1.3190005539 -2.1020302196 0.4296615314

H -1.2045511379 -2.1414255077 -2.5878007230

H -2.3413401032 -3.0048764734 -1.5003988043

H -2.9591879622 -1.8654191695 -2.7268018343

H 0.6525377525 -1.7334738998 -0.9317688315

H 0.8924610839 -1.3185151503 1.5561168280

H 2.9695164467 -1.4106876363 0.3654982832

H 3.0202278861 -0.0985364981 1.5663724836

H 4.3620385133 1.4807687611 -1.3797165072

H 4.7923236708 -0.1602151373 -0.8251329100

H 4.6816296473 1.1857403769 0.3523818265


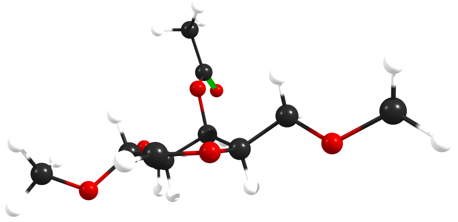


**Oxocarbenium_^4^*H*_3_**

C 3.9721656460 -1.6319791994 -0.3298293981

O 2.6046343557 -2.0743502875 -0.1828136866

C 1.6192238301 -1.0976422506 -0.3736908911

C 0.9908176998 -0.5825372445 0.9320380522

O 1.9273644502 0.2819021753 1.5069960867

C 1.7757518026 0.5107700092 2.9194802435

C -0.3537059013 0.1076226939 0.5976569729

O -0.1180793466 1.2067944659 -0.2895599979

C -0.2008440763 2.4801554712 0.2668865807

C 0.1991009074 3.5298585042 -0.7259250400

O -0.5516988240 2.6510684070 1.4074144457

C -1.3213030771 -0.8635418228 -0.0505445444

C -2.5774338299 -0.2771368268 -0.6637063509

O -3.4453916643 -1.3449946746 -0.9335206474

C -4.6736106608 -0.9445532556 -1.5483327389

O -0.6360930611 -1.6355636107 -1.1818223763

C 0.6113023737 -1.7224873751 -1.2830891884

H 4.5872445638 -2.4975064736 -0.0862283678

H 4.1693337654 -1.3212955022 -1.3621317203

H 4.1790571034 -0.8124720528 0.3637899289

H 1.9957769940 -0.2140374600 -0.9248440943

H 0.7901066988 -1.4455785463 1.5879260199

H 1.7773511228 -0.4408069540 3.4645813025

H 2.6396445006 1.1032338276 3.2200214763

H 0.8624069630 1.0748335462 3.1353025455

H -0.8193773930 0.4786598010 1.5175462384

H -0.2297723704 3.3261241789 -1.7099435572

H 1.2898799062 3.5273537575 -0.8282027397

H -0.1201265138 4.5065845943 -0.3635520519

H -1.5967191304 -1.6718804557 0.6325952407

H -2.3307114485 0.2985562102 -1.5684154320

H -3.0024330416 0.4245747099 0.0746305232

H -5.2522822698 -1.8553830202 -1.7009693347

H -5.2332637615 -0.2606401041 -0.8972408775

H -4.4885815172 -0.4615994395 -2.5166906418

H 0.9604652043 -2.3525057965 -2.1057119799


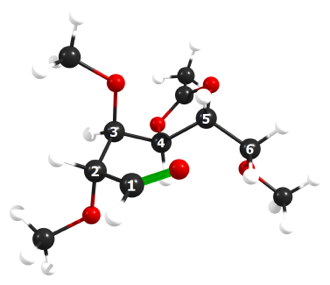


**Ring Opening_(*R*)**

O 1.9193534147 1.6997809515 -0.6341725070

C 2.5631487203 0.7157560744 -0.9342542708

C 2.1426260459 -0.7114331327 -0.5951871106

O 1.7437399780 -1.2828945573 -1.8358591389

C 2.5952874909 -2.3119966992 -2.3624937820

C 0.9708478548 -0.8624671610 0.4057217924

O 1.2900531067 -0.4537065436 1.7202989291

C 2.0252190706 -1.3993632174 2.5064606901

C -0.3049512921 -0.1236888346 -0.0152134978

C -0.5710836405 1.2735646349 0.5942768246

C -1.1590073850 2.2896993458 -0.3646165155

O -2.2503956908 1.6731191703 -1.0170699848

C -2.9018140997 2.5201389531 -1.9687084829

O -1.6384839027 0.9741198229 1.6070416813

C -2.0736436157 -0.2142519520 1.4153516767

C -3.2412545804 -0.7446480607 2.1491472506

O -1.4506207394 -0.9180206290 0.5375669126

H 3.4771174638 0.7986437149 -1.5526636071

H 3.0096410297 -1.2432604264 -0.1786429570

H 3.5939759923 -1.9237536710 -2.5920371712

H 2.1221892062 -2.6550783557 -3.2828919018

H 2.6768432086 -3.1487100686 -1.6574616024

H 0.7174607138 -1.9336510606 0.3853120415

H 1.4944433686 -2.3585269828 2.5605707709

H 3.0341978766 -1.5623139191 2.1104678145

H 2.1048378579 -0.9686114363 3.5048202127

H -0.4479831065 -0.1476954225 -1.0945822834

H 0.2699653124 1.6611178606 1.1601228244

H -1.4761952531 3.1800041555 0.1984135878

H -0.3713388355 2.5886402079 -1.0696009692

H -2.2047814081 2.8292259422 -2.7577468101

H -3.7102736346 1.9344190738 -2.4070571412

H -3.3176375685 3.4099295433 -1.4792663323

H -3.2815198486 -0.3103179447 3.1495579437

H -4.1449523310 -0.4400551319 1.6063699351

H -3.2051107796 -1.8335142439 2.1880251782


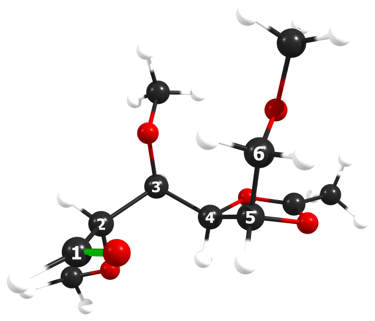


**Ring Opening_(*S*)**

O -0.3436403376 -2.2145335126 1.7908331564

C -1.5242242402 -1.9330667612 1.8125990719

C -2.1436965409 -0.8360263622 0.9485393224

O -2.8663724014 -1.5214128411 -0.0680932242

C -4.2984583386 -1.4501610332 0.0195156352

C -1.1270490784 0.1293999335 0.2943049390

O -0.4974089625 0.8629122630 1.3135722111

C -0.5129325387 2.2872231734 1.1503795248

C -0.1789314323 -0.6041650736 -0.6708674386

C 1.2116095276 -1.1437333326 -0.2916895651

C 2.0909753131 -0.4121250777 0.7071760841

O 2.3037072062 0.9054282154 0.2383031807

C 3.2026207052 1.6522790741 1.0620911825

O 1.9115145438 -0.9927006453 -1.6244045714

C 1.3235851970 -0.0492284613 -2.2694248460

C 1.9266694668 0.5674130016 -3.4674270244

O 0.1788152185 0.3197555819 -1.8124937689

H -2.2403782234 -2.5221061314 2.4159009697

H -2.8085089429 -0.2201463681 1.5688344449

H -4.6825499832 -1.9880671207 -0.8475473472

H -4.6656985178 -1.9293535297 0.9336720878

H -4.6378637269 -0.4075816406 -0.0134884366

H -1.7287848934 0.7849362970 -0.3512562629

H -1.5399185618 2.6565431196 1.0373867475

H 0.0927252165 2.5927040470 0.2907433634

H -0.0844427995 2.7034590429 2.0626219791

H -0.7628279881 -1.3891632461 -1.1608136260

H 1.1849005336 -2.2093095080 -0.0836625154

H 1.5915111506 -0.4281927579 1.6817870016

H 3.0445201172 -0.9564799305 0.7829718485

H 3.3015583246 2.6392259591 0.6084004765

H 4.1876184762 1.1697262345 1.1028619173

H 2.8066695650 1.7551258923 2.0800976478

H 1.1640477430 1.0538663240 -4.0752288978

H 2.6522712983 1.3163741473 -3.1268716638

H 2.4683679046 -0.1891189728 -4.0394236043

**6-*O*-Acetyl-2,3,4-tri-*O*-methyl-galacto-d-pyranosyl cation**


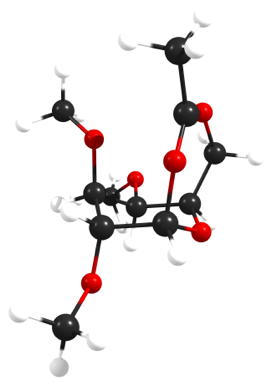


**Participation_^1^*C*_4_**

C 3.3832919681 1.1066293494 1.2097843184

C 2.2998803174 1.0104151972 0.1898677653

O 2.2780263359 -0.0692561458 -0.5031400585

C 1.1821820357 -0.6445220536 -1.4027737853

C 0.2771850431 -1.5138771147 -0.5009203423

O -0.5391472003 -2.2962965817 -1.3430390552

C -0.0049700458 -3.5747586577 -1.7128347372

C -0.6488070677 -0.6452769250 0.3714149230

O 0.1507694567 -0.0591852923 1.3910819678

C -0.5097295195 0.1504845259 2.6491986865

C -1.3372584453 0.3946501770 -0.5342326695

O -2.0791908953 1.3645266853 0.1773284064

C -3.4654769043 1.0514939331 0.3689665832

C -0.3528491601 1.1778515829 -1.4309801099

C 0.4278040543 2.3360229063 -0.8341115400

O 1.5283809873 2.0298046950 0.0999285966

O 0.5574287698 0.3138236945 -2.1384582754

H 3.0721365010 0.5164068092 2.0794170544

H 3.5406128526 2.1412489489 1.5115887926

H 4.3021150930 0.6657256709 0.8178906369

H 1.7819167210 -1.2452481030 -2.0841222689

H 0.9105591601 -2.1313074968 0.1486192188

H -0.7724116633 -4.0595564906 -2.3162295678

H 0.9094365929 -3.4783023074 -2.3111830368

H 0.2001327437 -4.1810698780 -0.8222398442

H -1.4038575168 -1.3070919177 0.8134934200

H 0.2525192165 0.5116541733 3.3415066706

H -0.9174230794 -0.7946012586 3.0282352597

H -1.3048576284 0.8945781321 2.5579520335

H -1.9844791137 -0.1688376136 -1.2205764733

H -3.9033241558 1.9043715572 0.8884701830

H -3.5950686849 0.1508496320 0.9812092264

H -3.9702070255 0.9128656738 -0.5947455902

H -0.9531392505 1.6493408703 -2.2156442520

H -0.2252997361 2.9850095159 -0.2523309770

H 0.9135192435 2.9013341063 -1.6322911596


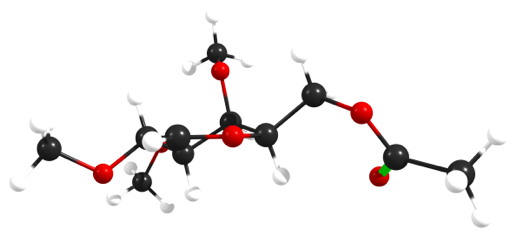


**Oxocarbenium_^4^*H*_3_**

C -3.7863122437 2.0706474408 0.4444055472

O -2.5817751408 1.5302059391 1.0205699068

C -1.7065975797 0.8987024886 0.1214552518

C -1.1896131472 -0.4559575240 0.6646045390

O -2.2071584971 -1.4158629930 0.6372696899

C -2.8641153185 -1.6726706701 1.8921478750

C -0.0285134061 -0.8941822282 -0.2568431097

O -0.4170333691 -0.9401014426 -1.6137243748

C -0.8956177920 -2.2128345527 -2.0979982621

C 1.1334541672 0.0877332798 -0.1315435684

C 2.2073887575 -0.0461152128 -1.2003313276

O 3.3612948396 0.7473489420 -0.9193619689

C 4.1912456804 0.2689768593 0.0658123587

C 5.4518770648 1.0745682125 0.1665547038

O 3.8878914346 -0.6968147031 0.7340798189

O 0.6375645561 1.5311319875 -0.2199590482

C -0.5748750532 1.8450296952 -0.1155129861

H -4.3649880115 2.4684121393 1.2774718630

H -4.3510891864 1.2812709251 -0.0642647807

H -3.5628749724 2.8806067679 -0.2606338228

H -2.1684202592 0.6867752025 -0.8590274591

H -0.8089485535 -0.3095721881 1.6869757631

H -3.3648750481 -0.7745587370 2.2658815319

H -3.6004553942 -2.4504824729 1.6900155016

H -2.1456588474 -2.0371141127 2.6359527845

H 0.3429276433 -1.8690351614 0.0917248279

H -1.0533916896 -2.0811871962 -3.1686048808

H -0.1390978256 -2.9891656856 -1.9338475231

H -1.8304663844 -2.4921467985 -1.6074856423

H 1.5768083988 0.0594455268 0.8648983020

H 2.4758327822 -1.1057876717 -1.2567701956

H 1.8373897176 0.2842271674 -2.1702841403

H 5.9793305307 0.8106144813 1.0825666667

H 6.0898085700 0.8509707465 -0.6953752931

H 5.2319333496 2.1448245536 0.1417206555

H -0.7627697728 2.9220969955 -0.1368392037


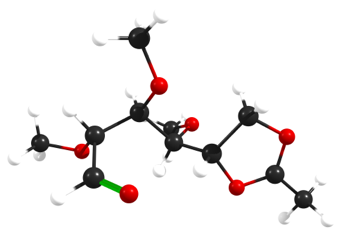


**Ring Opening_(*S1*)**

O -0.5929927180 -0.6219358603 2.9363242636

C -1.0868566080 0.4808770746 2.8260034130

C -0.7589685244 1.5013530948 1.7357471208

O -1.9961170157 1.8465011091 1.1215991248

C -2.5793228990 3.0867317804 1.5414952293

C 0.2078562436 1.0615006766 0.6107889953

O 1.5321338529 0.8429719370 1.0825003027

C 2.3336675740 2.0183852988 1.2420675104

C -0.2360742918 -0.2063487683 -0.1697479353

O -0.0060809320 -0.0867332499 -1.5657127465

C -1.0448538659 0.5758313887 -2.2973273114

C 0.5629515096 -1.4293834911 0.2766216838

O -0.0945990189 -2.6332354130 -0.3344923569

C 0.7493124455 -3.2541891082 -1.0741941470

C 0.3906950488 -4.4896984227 -1.8015285916

O 1.9369830157 -2.7657309276 -1.1224458888

C 1.9921721785 -1.5355306739 -0.2844042722

H -1.8800992930 0.8167107982 3.5231136640

H -0.3172634015 2.3788449025 2.2338432319

H -3.4938109571 3.2075980232 0.9598444104

H -1.9004067174 3.9247030665 1.3371347204

H -2.8318410577 3.0729489093 2.6082331819

H 0.2038522327 1.8846507596 -0.1168646532

H 1.9418581812 2.6745589726 2.0280349613

H 3.3274625224 1.6781781302 1.5357436886

H 2.3993876312 2.5757460119 0.2990510516

H -1.3000998664 -0.4003177793 0.0203480295

H -1.1852792274 1.6066741896 -1.9530790205

H -1.9935420102 0.0332316627 -2.2015439695

H -0.7274307690 0.5833267884 -3.3404233269

H 0.4940082950 -1.5702229830 1.3528834661

H 0.9643788067 -4.5568212097 -2.7273866699

H -0.6821220445 -4.5144957748 -1.9947467243

H 0.6536376054 -5.3488174119 -1.1714075369

H 2.7532660772 -1.7102397778 0.4728254024

H 2.2641379977 -0.7275237232 -0.9569983008


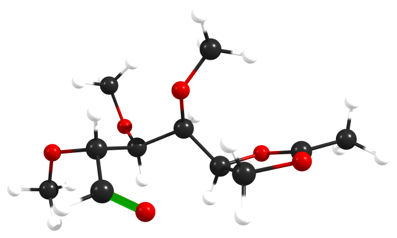


**Ring Opening_(*S*2)**

O -2.0481960441 -1.2283828512 -0.8994258092

C -2.5741560668 -1.0428376770 0.1849417581

C -1.9853691675 -0.1702022607 1.2793381935

O -3.0187278677 0.3878934910 2.0600155795

C -3.8322982675 1.3822229894 1.4219677428

C -0.9603767995 0.8704670193 0.7360667056

O -0.7234503016 1.9170388843 1.6540166820

C -0.3187595371 1.5822236892 2.9880436691

C 0.4005561004 0.2558456959 0.2616991707

O 0.7474013340 -0.8693033608 1.0474003490

C 2.1195890092 -0.9858397887 1.4376160360

C 0.4561539250 -0.0581110534 -1.2578062137

O 1.8108169568 0.4018918955 -1.7706523619

C 2.4083067265 -0.5742713025 -2.3535155988

C 3.7598707814 -0.4313802143 -2.9346327425

O 1.7761508755 -1.6915371670 -2.3806560258

C 0.4538229129 -1.5297117283 -1.7056133110

H -3.5348554450 -1.5270480683 0.4434282356

H -1.4600753878 -0.8612180908 1.9537444974

H -4.6539561053 1.5801923610 2.1107321744

H -3.2684631978 2.3053953069 1.2542718648

H -4.2493460530 1.0194791675 0.4719617129

H -1.4012267971 1.3831977143 -0.1260481959

H -0.2012161941 2.5370833868 3.5015373967

H 0.6403928835 1.0510684019 3.0039802397

H -1.0824318165 0.9899693097 3.5002428591

H 1.1222203354 1.0697163260 0.4186531830

H 2.1634584791 -1.7864987611 2.1767782269

H 2.7669528046 -1.2549881148 0.5917637029

H 2.4831516337 -0.0541001361 1.8867725251

H -0.2479207445 0.5543484610 -1.8177265928

H 3.9446105684 0.6082864067 -3.2074231049

H 3.8675020146 -1.0945293492 -3.7947881335

H 4.4961134156 -0.7311409592 -2.1777377574

H -0.2983865078 -1.7711693993 -2.4500258998

H 0.4422415441 -2.2339502234 -0.8770207576


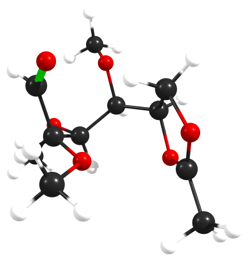


**Ring Opening_(*R1*)**

O -1.1287704876 0.5462359236 2.7878757067

C -1.5267164393 0.8666377424 1.6874178121

C -1.4058715592 -0.0444626794 0.4608138499

O -0.8395030257 -1.3085574879 0.7560989237

C -1.7112413889 -2.1940847557 1.4826744837

C -0.5885405842 0.5919845177 -0.6941787365

O -1.3016154247 1.6927080858 -1.2307371616

C -2.2081303061 1.3777864207 -2.2927668449

C 0.8054868867 1.1582467397 -0.3314120757

O 0.7592885958 2.1061696835 0.7181866652

C 0.7295832708 3.4957396320 0.3344009320

C 1.8931804838 0.1771267931 0.1509820033

O 1.8384118531 -1.1123232865 -0.5948926489

C 1.8717920807 -2.0864764714 0.2488837965

C 1.8220952508 -3.4928828477 -0.1957569424

O 1.9612148395 -1.7672044588 1.4859447189

C 1.8460078064 -0.2913137027 1.6211593054

H -2.0451919781 1.8266429170 1.5098012660

H -2.4306381545 -0.1728288879 0.0719048091

H -1.1777217334 -3.1399705737 1.5904201851

H -2.6349651462 -2.3642353836 0.9158388420

H -1.9423188958 -1.7953246002 2.4731175935

H -0.4513552442 -0.1906112351 -1.4551105914

H -1.6732003899 0.9414532890 -3.1457223830

H -2.9966249197 0.6883156388 -1.9651529317

H -2.6656269321 2.3202456722 -2.5950132808

H 1.1704034378 1.6244999468 -1.2561004447

H 1.6093420201 3.7439989358 -0.2710577780

H 0.7597230824 4.0597243785 1.2668984583

H -0.1832816181 3.7246673008 -0.2196116614

H 2.8692319076 0.5975650790 -0.0970257325

H 2.4077829477 -3.6182194235 -1.1089885658

H 0.7771761283 -3.7334814475 -0.4266497505

H 2.1771773770 -4.1527460273 0.5957998854

H 0.9099165860 -0.1043536122 2.1445193374

H 2.7037996732 0.0291281848 2.2091389555


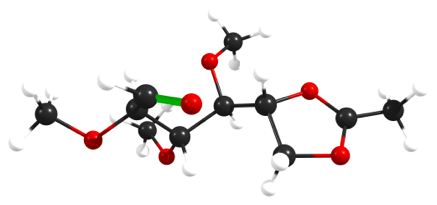


**Ring Opening_(*R2*)**

O 0.0469337086 1.9209223530 -1.6398981517

C -0.9552458937 1.3174512466 -1.9816173551

C -1.7009875417 0.3199521704 -1.1042115758

O -3.0528336732 0.7486714632 -1.0971426209

C -3.8913501660 0.1580818863 -2.0943441840

C -1.1755090385 0.3270862116 0.3565299166

O -2.0703416357 -0.2387338570 1.2885075645

C -2.5607876915 -1.5606708308 1.0566888345

C 0.2383390994 -0.3315864498 0.5232545356

O 0.3557028344 -1.4193729598 -0.3693062939

C 1.1247027804 -2.5480118608 0.0666733437

C 1.4266749775 0.6325218986 0.3477926791

O 2.6376290796 0.0254198242 1.0453199336

C 3.2388452198 0.9389025559 1.7162636553

C 4.5302848839 0.7018527519 2.3932244513

O 2.6638712323 2.0907636131 1.7497930169

C 1.3634668080 2.0029713101 1.0371405430

H -1.4266540371 1.5258267345 -2.9593934208

H -1.6002560640 -0.6845132371 -1.5402996480

H -4.8962135618 0.5390659125 -1.9092791342

H -3.8922828460 -0.9367321688 -2.0080368170

H -3.5853627232 0.4374829258 -3.1101960080

H -1.1234209255 1.3748357640 0.6677318507

H -3.1404110683 -1.6197277014 0.1300740441

H -3.2210242040 -1.7767950851 1.8973940904

H -1.7558214143 -2.3044570287 1.0306214246

H 0.2582671194 -0.6795323295 1.5679914226

H 2.1976531221 -2.3294212811 0.0828102342

H 0.9331070332 -3.3411667212 -0.6567016938

H 0.8017797955 -2.8787071023 1.0620658745

H 1.7312781791 0.6978822678 -0.6938224163

H 4.5919759227 1.2989239111 3.3048621493

H 5.3339835276 1.0243271527 1.7183348321

H 4.6571479710 -0.3608342232 2.6023281584

H 0.6002538960 2.0924725837 1.8115829867

H 1.3164052939 2.8347482997 0.3389637778

**3-*O*-Benzoyl-2,4,6-tri-*O*-benzyl-manno-d-pyranosyl cation**


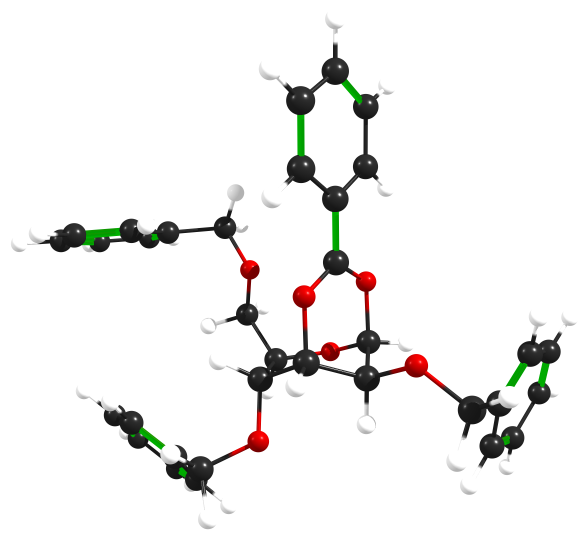


**Participation_^1^*C*_4_**

C 1.0327235882 -0.1586169726 -0.7503862395

C 1.6264305834 0.5157023035 0.4773564272

O 0.6229561500 0.7482700694 1.4558950570

C 1.1183009090 1.3586808805 2.6650801358

C 1.5738424654 2.7894826059 2.4797405615

C 2.9139162235 3.1480294012 2.6710424828

C 3.3270774313 4.4749654338 2.5123222428

C 2.4006636951 5.4563024435 2.1544743010

C 1.0588716279 5.1084029412 1.9595201155

C 0.6496556825 3.7847075397 2.1243245863

C -0.1805770998 0.5758634231 -1.3817084756

O -0.1087286210 0.5410507592 -2.8019272615

C 0.2714745875 1.7783315483 -3.4555411925

C 1.7178597193 2.1591352151 -3.2432936545

C 2.0615844574 3.2616875166 -2.4498312015

C 3.4029010875 3.6047775405 -2.2484979818

C 4.4130041102 2.8448225992 -2.8412522532

C 4.0799750228 1.7446477424 -3.6405841861

C 2.7415464212 1.4067323692 -3.8422340874

C -1.5420002469 -0.0869201389 -1.0848372801

O -1.9646751072 0.1470129412 0.3034050363

C -1.6896318716 -0.7030831962 1.2465028528

C -2.2320953128 -0.4492665420 2.5714432447

C -3.0650144645 0.6653750707 2.7914998847

C -3.5889606016 0.8901902283 4.0602750715

C -3.2866287044 0.0125187172 5.1084747886

C -2.4590952274 -1.0957426983 4.8914095574

C -1.9304729954 -1.3324251453 3.6267108087

O -1.0018607381 -1.7742404415 1.0495090226

C -1.4737678126 -1.5814583267 -1.3376220539

O -2.7447806182 -2.1489310480 -1.1328658352

C -3.1096584924 -3.2398972228 -2.0211036093

C -2.3036001425 -4.4979921836 -1.8051179943

C -2.4918568722 -5.2685046321 -0.6458844418

C -1.7351353069 -6.4208680650 -0.4308776264

C -0.7834035914 -6.8217437523 -1.3761522776

C -0.5958234508 -6.0686134137 -2.5368740024

C -1.3534852286 -4.9118926245 -2.7486805420

C -0.4406715025 -2.0984180702 -0.3371955683

O 0.8086553120 -1.5795230385 -0.4736180718

H 1.8130261683 -0.1713839206 -1.5134217616

H 2.4187857170 -0.1296567102 0.8828189557

H 2.0833634469 1.4617943632 0.1558079668

H 0.2747208009 1.3063360514 3.3588618171

H 1.9348817246 0.7459139287 3.0711526806

H 3.6391532732 2.3890465251 2.9548150538

H 4.3680424183 4.7397251683 2.6715701155

H 2.7177024924 6.4881578459 2.0368753764

H 0.3330641930 5.8715538739 1.6938917455

H -0.3958676588 3.5201659378 1.9860106583

H -0.2000912357 1.6064618129 -1.0103170504

H 0.0649244507 1.5831386413 -4.5104724874

H -0.3941187490 2.5836027277 -3.1174956338

H 1.2796724150 3.8662767841 -1.9960518278

H 3.6541075252 4.4649451241 -1.6355707689

H 5.4546456764 3.1124135617 -2.6919618317

H 4.8629484729 1.1605391931 -4.1148281512

H 2.4860955186 0.5590277477 -4.4732001196

H -2.3210497333 0.3949600499 -1.6732097702

H -3.2987447818 1.3368457128 1.9735487995

H -4.2343034937 1.7447771880 4.2343850614

H -3.6986884476 0.1918319095 6.0968112472

H -2.2295996094 -1.7725238559 5.7077151190

H -1.2889390267 -2.1877225251 3.4485897172

H -1.0921933633 -1.7437496230 -2.3523006770

H -3.0214948017 -2.8945652425 -3.0589621699

H -4.1667973578 -3.4010532465 -1.7996466965

H -3.2387875021 -4.9662631310 0.0840020576

H -1.8949246569 -7.0144240182 0.4642974833

H -0.2008477985 -7.7231188710 -1.2122134619

H 0.1317260852 -6.3823103227 -3.2791918314

H -1.2139550693 -4.3378039547 -3.6618824189

H -0.3656721585 -3.1813905027 -0.2834275371


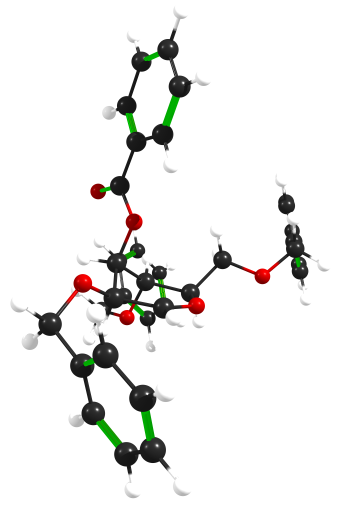


**Oxocarbenium_^3^*E***

O 3.2730146861 -0.0178842222 0.4321431341

C 2.6630755274 1.0066456076 0.2034249407

O 1.5678097116 1.0080646073 -0.6439044588

C 1.2930105100 -0.2319296930 -1.2986949122

C 0.3349613895 -1.1117718184 -0.4659766659

O 0.1361008699 -2.3428527371 -1.1402208484

C 1.0402527016 -3.4247688449 -0.7554698373

C 0.8406007209 -3.8800408275 0.6676895000

C -0.3266400370 -4.5714803902 1.0310233429

C -0.5198291771 -4.9882832397 2.3479058698

C 0.4546588129 -4.7228015335 3.3172398605

C 1.6206082870 -4.0406628910 2.9647600362

C 1.8119078926 -3.6193854499 1.6446837280

C -1.0405577533 -0.4655983284 -0.2536392923

C -1.2206862085 0.3093585754 1.0405915869

O -2.5559156213 0.7444983585 1.1007737403

C -2.8858606444 1.4767348671 2.3019615346

C -2.8255629814 0.6354283591 3.5564029292

C -3.7188448571 -0.4335301938 3.7315296090

C -3.6715581538 -1.2134641884 4.8872519433

C -2.7333538895 -0.9308572896 5.8867555911

C -1.8429595488 0.1320866366 5.7237863493

C -1.8887965758 0.9088951459 4.5615077332

O -1.4180314789 0.5089496725 -1.3618555753

C -0.7002187542 0.7471276076 -2.3637522477

C 0.6107194740 0.0832293463 -2.6368558547

O 1.4105216782 0.8748906498 -3.4636280619

C 1.3356615356 0.5720414459 -4.8876104989

C -0.0084388741 0.9098649051 -5.4863304481

C -0.3647098108 2.2516238948 -5.7039243311

C -1.6227265709 2.5759645989 -6.2140384965

C -2.5408262407 1.5624768096 -6.5159816022

C -2.1936820354 0.2254300303 -6.3125170111

C -0.9325119728 -0.0981554018 -5.7987618418

C 2.9568078412 2.3401946324 0.7675392786

C 3.9805780880 2.4283259275 1.7263344386

C 4.3024464445 3.6617397295 2.2875544064

C 3.6090512179 4.8116112191 1.8944903508

C 2.5928488139 4.7286085256 0.9367792177

C 2.2640940636 3.4973149496 0.3721303935

H 2.2218551172 -0.7740413158 -1.4810025632

H 0.7805328485 -1.2704274626 0.5230995157

H 2.0783974751 -3.1136167541 -0.9215188794

H 0.7973957721 -4.2146263610 -1.4693498240

H -1.0789692148 -4.7906745579 0.2773374275

H -1.4216054435 -5.5294764136 2.6178113654

H 0.3068210554 -5.0547694377 4.3403405481

H 2.3826248358 -3.8396742779 3.7115671863

H 2.7248746014 -3.0948154663 1.3727257274

H -1.8255034521 -1.2172229752 -0.3537692451

H -0.5138139853 1.1519364861 1.0908127184

H -0.9790029360 -0.3871287145 1.8582030483

H -3.9001561643 1.8371903432 2.1144588478

H -2.2206663545 2.3473266044 2.3897754406

H -4.4555549860 -0.6481534089 2.9615977187

H -4.3727462858 -2.0326308431 5.0161916167

H -2.7038309485 -1.5320894554 6.7904563299

H -1.1176688573 0.3601567230 6.4990575553

H -1.1992305876 1.7417333394 4.4429215945

H -1.0888419103 1.5163995960 -3.0349297419

H 0.3714018059 -0.9030764524 -3.0772298538

H 2.1351447056 1.1776531300 -5.3174088415

H 1.5791104811 -0.4881096629 -5.0342051504

H 0.3506817013 3.0400465685 -5.4835401033

H -1.8834478684 3.6151290216 -6.3901925327

H -3.5157172798 1.8158910081 -6.9207454558

H -2.8954654451 -0.5646860934 -6.5612571848

H -0.6597711633 -1.1427456683 -5.6646471066

H 4.5113189163 1.5277492746 2.0156518707

H 5.0934580478 3.7287771184 3.0275868309

H 3.8632464687 5.7727538372 2.3309783160

H 2.0623068053 5.6237861161 0.6279330467

H 1.4858031640 3.4333971021 -0.3793077529

**4-*O*-Benzoyl-2,3,6-tri-*O*-benzyl-manno-d-pyranosyl cation**


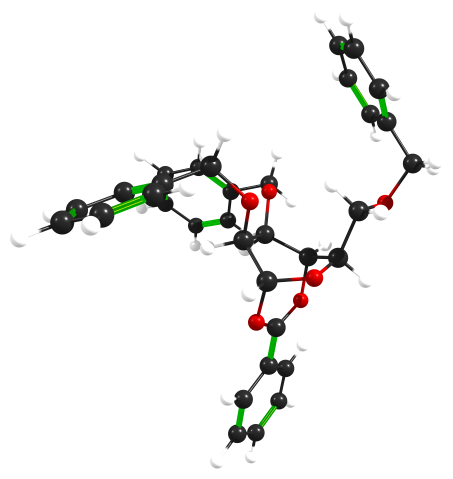


**Participation_^5^*S*_1_**

C -1.7149330084 0.8417989612 1.9197719124

C -2.7652836561 1.3200426873 0.9129752107

O -2.6832431569 2.7350607892 0.9320212737

C -3.7189946542 3.4039462541 0.1832591074

C -3.5609990142 3.2814619875 -1.3154147862

C -4.3735162855 2.4165264088 -2.0600036047

C -4.2186556353 2.3068451471 -3.4455431562

C -3.2477968024 3.0667563213 -4.1015291743

C -2.4346197033 3.9386973309 -3.3685278051

C -2.5927518188 4.0449087033 -1.9859307660

C -0.2849579910 1.1235691841 1.4913254196

O 0.6013965253 0.8953553694 2.6727737755

C 0.8653480331 -0.2894372868 3.1426127574

C 1.8547159458 -0.3830926314 4.2037532458

C 2.1191642049 -1.6333931128 4.8014028224

C 3.0628771847 -1.7218328233 5.8181160857

C 3.7427834565 -0.5742618396 6.2440879970

C 3.4815419172 0.6688882102 5.6542526195

C 2.5414259431 0.7714318340 4.6357781714

O 0.3100671920 -1.3744999564 2.7210371981

C 0.2443572278 0.2800526530 0.3039426617

O -0.0747984291 0.8830083766 -0.9217596924

C 0.8410602126 1.9106722034 -1.3784174295

C 2.1921330765 1.3669855104 -1.7780494519

C 2.3150758230 0.5490038931 -2.9130531547

C 3.5580658269 0.0394180893 -3.2890465845

C 4.6984004739 0.3444198032 -2.5366484191

C 4.5898600032 1.1636220991 -1.4114988501

C 3.3413369439 1.6704451157 -1.0354694492

C -0.3768884562 -1.1538493282 0.3485038456

O -1.4931358396 -1.3179712732 -0.4887559989

C -1.2254242576 -1.9013378316 -1.7932022156

C -0.7853401830 -3.3435115747 -1.7117511293

C 0.5081482513 -3.7224314132 -2.0961446083

C 0.9121530903 -5.0597677000 -2.0195757000

C 0.0244146053 -6.0301698650 -1.5514903087

C -1.2698850112 -5.6623075234 -1.1647600715

C -1.6717232200 -4.3289088304 -1.2465052798

C -0.9202591989 -1.4146880669 1.7538518394

O -1.8829726500 -0.5761276886 2.2008138524

H -1.9032918062 1.3489365777 2.8704237992

H -3.7473192099 0.9726804188 1.2673155472

H -2.5828525023 0.9062010334 -0.0834192086

H -3.6438143034 4.4477325765 0.4978214510

H -4.6972789434 3.0176728257 0.5015678290

H -5.1429031439 1.8350674459 -1.5571937705

H -4.8623746495 1.6395034975 -4.0109346437

H -3.1343021221 2.9930526747 -5.1790169462

H -1.6941422683 4.5485923573 -3.8783333116

H -1.9718936440 4.7364059255 -1.4211967244

H -0.1676224830 2.1899007414 1.3058013179

H 1.5846560763 -2.5155235350 4.4687941023

H 3.2684679805 -2.6807614081 6.2818068391

H 4.4776336644 -0.6483340356 7.0398271115

H 4.0112878554 1.5536470083 5.9910957566

H 2.3333724100 1.7304507674 4.1759837592

H 1.3344669657 0.1945090573 0.4203243062

H 0.9506476520 2.6878727070 -0.6104166740

H 0.3164678131 2.3528886398 -2.2278403279

H 1.4345564168 0.3252053072 -3.5099747190

H 3.6428230945 -0.5820231009 -4.1755693524

H 5.6668070808 -0.0451945873 -2.8352264360

H 5.4734821561 1.4146674258 -0.8324877436

H 3.2655290848 2.3247394212 -0.1694541611

H 0.3947986848 -1.9014329590 0.1306982151

H -0.4864615466 -1.2880058689 -2.3167808387

H -2.1834183994 -1.8055106125 -2.3092035640

H 1.1996574240 -2.9711129534 -2.4707002435

H 1.9145457175 -5.3410076409 -2.3282993156

H 0.3334484015 -7.0695661603 -1.4959367875

H -1.9667620704 -6.4171720854 -0.8128667406

H -2.6815488547 -4.0481491247 -0.9577332353

H -1.2447094969 -2.4412605231 1.9040225504


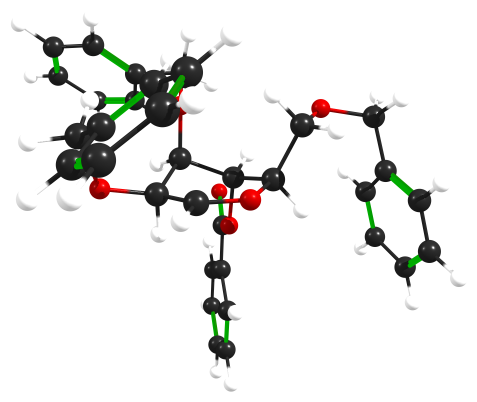


**Oxocarbenium_^3^*E***

O 1.2849025587 0.0817307656 2.7486847560

C 0.1248454722 0.4374191289 2.6988865424

O -0.5199895727 0.6072559637 1.4862081794

C 0.2843157644 0.3870330264 0.3116249808

C -0.1866318266 1.3700825825 -0.7487541938

C 0.8361844564 1.7105981769 -1.8551094472

O 2.0334034038 2.1743526790 -1.2731808279

C 2.2272502189 3.6103644582 -1.2567410647

C 1.2983156542 4.3406324604 -0.3150358191

C 0.2624219180 5.1467332045 -0.8073188789

C -0.6066050502 5.8059132406 0.0691110043

C -0.4456670233 5.6631850650 1.4490783740

C 0.5900458577 4.8663533557 1.9511858514

C 1.4574458415 4.2126493747 1.0749104950

O -1.4131896032 0.8896646393 -1.4867461181

C -1.8328799871 -0.3050273234 -1.4428270531

C -1.3099076577 -1.3433572592 -0.5043553773

O -1.7288100426 -2.6401315831 -0.8051360259

C -1.2484571964 -3.2681792864 -2.0361680958

C -1.7177842289 -2.5323265260 -3.2643593611

C -0.8537305175 -1.6724262261 -3.9666177658

C -1.3121468291 -0.9574274084 -5.0812005542

C -2.6356259122 -1.0936497437 -5.5002242067

C -3.5036935652 -1.9542712602 -4.8109905120

C -3.0480037984 -2.6678000658 -3.7041074347

C 0.1625265271 -1.0972808832 -0.1151476951

O 1.0121300818 -1.3800966403 -1.2056015982

C 2.4035876821 -1.6821841408 -0.8542182300

C 2.5684391563 -3.0038008490 -0.1495405624

C 2.5574871912 -4.2015736476 -0.8821241255

C 2.7001335537 -5.4298774140 -0.2363907805

C 2.8600070126 -5.4746130496 1.1529916179

C 2.8831006705 -4.2887525639 1.8900230671

C 2.7399627463 -3.0581499497 1.2417073844

C -0.7440960048 0.7266064440 3.8596386850

C -0.2071419858 0.5140267779 5.1408233951

C -0.9801930851 0.7720840790 6.2703859837

C -2.2894966619 1.2443277296 6.1291326564

C -2.8265294027 1.4601219611 4.8556769739

C -2.0587978616 1.2031057054 3.7209947724

H 1.3263381022 0.6214949964 0.5374469363

H -0.5528775807 2.2803395752 -0.2728532776

H 1.0673417711 0.8060568583 -2.4227021184

H 0.3889686635 2.4513541310 -2.5295026739

H 2.1250716038 3.9954965550 -2.2793239634

H 3.2694957982 3.7235863370 -0.9498457688

H 0.1447536508 5.2774238482 -1.8806769119

H -1.3975854433 6.4365111483 -0.3256443602

H -1.1129987988 6.1807838113 2.1314846695

H 0.7294032225 4.7685661453 3.0236570079

H 2.2697976264 3.6067973819 1.4689332956

H -2.7318115012 -0.4858156687 -2.0314825321

H -1.8770578336 -1.0876337263 0.4151292828

H -1.6884097168 -4.2649998973 -1.9788783705

H -0.1623272936 -3.3494887244 -2.0035659514

H 0.1803486369 -1.5845747113 -3.6476905647

H -0.6322578422 -0.3091736676 -5.6258108921

H -2.9914029790 -0.5468728701 -6.3680256231

H -4.5283424143 -2.0748384642 -5.1491386322

H -3.7194293809 -3.3412928623 -3.1778366977

H 0.3898844378 -1.7359727358 0.7453833933

H 2.9094347151 -1.6811050152 -1.8218956046

H 2.8107128250 -0.8616412090 -0.2541922363

H 2.4547571114 -4.1703984549 -1.9645103815

H 2.6999797127 -6.3493794318 -0.8139224544

H 2.9779294351 -6.4299226843 1.6552779045

H 3.0199411870 -4.3185957667 2.9666895963

H 2.7718963549 -2.1374585358 1.8195141924

H 0.8100248008 0.1487776096 5.2321886831

H -0.5645466991 0.6059310570 7.2590770328

H -2.8908733703 1.4449069244 7.0106364120

H -3.8414563610 1.8298704756 4.7489477546

H -2.4717303948 1.3780525733 2.7343638623


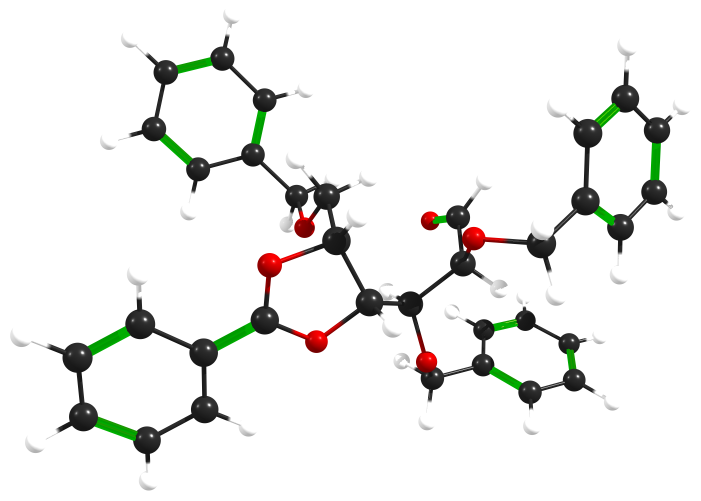


**Ring Opening_(*R*)**

O 2.0404065573 1.3326607946 0.1079877501

C 2.1754045365 0.2196302588 -0.3547390712

C 1.1608030724 -0.4434260908 -1.2931004329

O 1.1221662355 -1.8447832686 -1.0302910254

C 1.7695257237 -2.7052572225 -2.0094469134

C 3.2760018676 -2.6241952800 -1.9834660411

C 3.9762796870 -1.9440902941 -2.9890736946

C 5.3712933262 -1.8537737236 -2.9475919587

C 6.0783239102 -2.4461887562 -1.8994344749

C 5.3888147551 -3.1345757732 -0.8945819683

C 3.9973327213 -3.2240666937 -0.9386735767

C -0.2641792184 0.1768071033 -1.2031953946

O -0.8155961187 0.4488988311 -2.4830800384

C -0.6130664758 1.7811626025 -3.0094844958

C 0.7244340455 1.9756421230 -3.6890762100

C 0.9845850100 1.3453489922 -4.9171230781

C 2.2121835242 1.5188144599 -5.5575967974

C 3.1947834860 2.3318696042 -4.9801691401

C 2.9432629476 2.9678533367 -3.7627314919

C 1.7130464609 2.7897958660 -3.1209985215

C -1.2607069574 -0.8118998523 -0.5899764149

C -1.0860047029 -1.3955185085 0.8261708989

C -0.3394615012 -0.5986690935 1.8794199579

O -0.8545320986 0.7136896954 1.8853991750

C -0.2262907887 1.6040112156 2.8431514726

C -0.4916691160 1.2200953659 4.2797827472

C 0.5375724891 0.7280314459 5.0932512279

C 0.2879503224 0.3716050463 6.4224421288

C -0.9982385877 0.5027628540 6.9496588945

C -2.0328741663 0.9966327046 6.1468809465

C -1.7794931873 1.3546806607 4.8223567034

O -2.5103592133 -1.4566974710 1.2711390377

C -3.1935111529 -0.6065808479 0.5633616590

C -4.5528960448 -0.2596869057 0.8813340130

C -5.1463774998 -0.7664684754 2.0568186309

C -6.4623375560 -0.4315725126 2.3517795834

C -7.1828396528 0.4019764114 1.4863512656

C -6.5933406181 0.9062672863 0.3196525472

C -5.2790625650 0.5791630123 0.0094501013

O -2.5750309088 -0.1197934220 -0.4636970820

H 3.0623472143 -0.4034043135 -0.1308749152

H 1.5192161300 -0.2492256473 -2.3126163852

H 1.4259934214 -3.7074883262 -1.7410490446

H 1.3816688395 -2.4569758175 -3.0049431010

H 3.4331956086 -1.4896170898 -3.8143036584

H 5.9022247540 -1.3269038071 -3.7345235906

H 7.1616585804 -2.3814210662 -1.8684310752

H 5.9369875430 -3.6084009812 -0.0858284328

H 3.4658496210 -3.7684363511 -0.1618006626

H -0.2205870067 1.0873051266 -0.5953323402

H -0.7487159481 2.5143310857 -2.2043287888

H -1.4279890734 1.9038002529 -3.7279679454

H 0.2178950487 0.7246834369 -5.3744985108

H 2.3977562059 1.0361080738 -6.5126918492

H 4.1457542358 2.4766796404 -5.4840205343

H 3.6984401057 3.6062029596 -3.3141414080

H 1.5232599715 3.2889968491 -2.1744100970

H -1.4393673733 -1.6122748657 -1.3106924817

H -0.7307400383 -2.4233501076 0.7911589741

H 0.7343218647 -0.6197025412 1.6453043389

H -0.4755909176 -1.0955332271 2.8504104626

H 0.8500101255 1.6432978421 2.6366337088

H -0.6561647044 2.5805083877 2.6083114529

H 1.5437950806 0.6360241675 4.6908746930

H 1.0965510319 -0.0012712865 7.0437965833

H -1.1932469952 0.2323543776 7.9829666012

H -3.0303804583 1.1155924059 6.5596828931

H -2.5815101812 1.7532372997 4.2056851882

H -4.5756806254 -1.4063665463 2.7205961599

H -6.9292335282 -0.8155867386 3.2524640471

H -8.2107774216 0.6598098984 1.7222843072

H -7.1617213798 1.5494946410 -0.3435606055

H -4.8115222791 0.9576767883 -0.8928149028


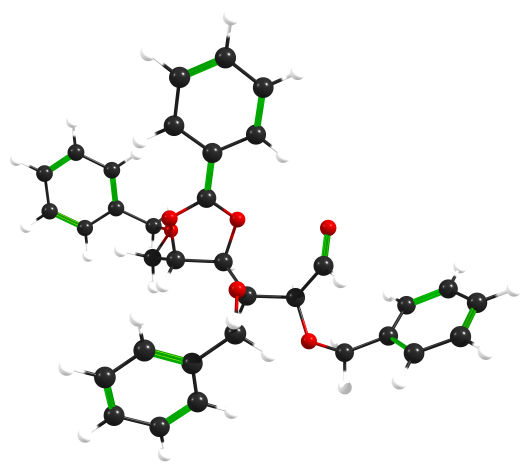


**Ring Opening_(*S*)**

O 2.6273911731 -2.2028662773 0.0175736224

C 2.0992144639 -2.1355368586 -1.0707608335

C 1.7552608012 -0.7983367870 -1.7543138405

O 1.7027695755 -0.9274701033 -3.1658095936

C 2.9771424611 -0.8681105337 -3.8518688100

C 3.8467370253 -2.0886544218 -3.6599609457

C 3.4760734657 -3.3167774894 -4.2323397512

C 4.2723239338 -4.4485048255 -4.0586395733

C 5.4553889417 -4.3654925596 -3.3147406513

C 5.8377237674 -3.1477273940 -2.7494279476

C 5.0357611329 -2.0154323247 -2.9226113077

C 0.3488584262 -0.2998726203 -1.3183153889

O -0.5757283389 -1.3556750692 -1.1219384250

C -1.2884789569 -1.8268357148 -2.3049918966

C -2.3153785470 -0.8345918597 -2.7931372252

C -3.5239722619 -0.6667800085 -2.0961855621

C -4.4702684380 0.2643423018 -2.5279374085

C -4.2208793915 1.0394719245 -3.6665786315

C -3.0266925448 0.8750765724 -4.3718632053

C -2.0790968570 -0.0573610203 -3.9363752427

C 0.3443114252 0.5365322641 -0.0393347512

C -1.0592344672 1.0157391307 0.3971135011

C -1.1483675168 2.4711413223 0.8101010668

O -0.0721257295 2.7443885555 1.6794073900

C -0.0405203490 4.0965732060 2.1950796151

C -1.1758015920 4.4047436271 3.1430081711

C -1.2285590319 3.7884929843 4.4033211462

C -2.2756869496 4.0633737331 5.2833493604

C -3.2823879861 4.9637196041 4.9161302419

C -3.2357636812 5.5870659628 3.6676710273

C -2.1868805477 5.3064552065 2.7860535792

O -1.3256646780 0.1680921085 1.5833774427

C -0.2446304559 -0.4790238223 1.8944163764

C -0.1549425814 -1.3492856362 3.0339254909

C 1.0081270391 -2.1301953070 3.2103622819

C 1.0910555442 -2.9751371777 4.3110886951

C 0.0319346481 -3.0422884832 5.2249907175

C -1.1231173115 -2.2684060552 5.0457243982

C -1.2254817178 -1.4202777268 3.9506074279

O 0.7624138510 -0.2902166091 1.1024883461

H 1.8183187354 -3.0381214577 -1.6444393081

H 2.5145886073 -0.0584384404 -1.4569755143

H 3.5099314176 0.0376538560 -3.5314847803

H 2.6977781447 -0.7481827816 -4.9016252019

H 2.5641734821 -3.3813665760 -4.8208253244

H 3.9793330293 -5.3914253591 -4.5105662840

H 6.0782452673 -5.2452787224 -3.1848335620

H 6.7576168607 -3.0765048384 -2.1772156576

H 5.3419613604 -1.0679468706 -2.4854093101

H 0.0117759913 0.3809864700 -2.1151007936

H -1.7614583626 -2.7499244779 -1.9630386027

H -0.5660579104 -2.0666097316 -3.0900895674

H -3.7305823234 -1.2840334079 -1.2247366253

H -5.4077128576 0.3737877612 -1.9905932074

H -4.9614373744 1.7557760156 -4.0092351739

H -2.8356402672 1.4635711822 -5.2641875456

H -1.1569151526 -0.1936548927 -4.4955933307

H 1.0601229768 1.3578017468 -0.0851231919

H -1.8315146534 0.7555166432 -0.3284455366

H -2.1210383331 2.6549000891 1.2858676403

H -1.0970694580 3.0876235583 -0.1040186703

H -0.0370042702 4.8015656620 1.3524243864

H 0.9259706159 4.1599103274 2.7006463218

H -0.4399527056 3.1000713485 4.6969551069

H -2.3011483342 3.5893281457 6.2601160023

H -4.0922310145 5.1844936587 5.6048030472

H -4.0089084218 6.2936531293 3.3815131490

H -2.1490116554 5.8037413101 1.8194362696

H 1.8121136963 -2.0799447035 2.4830532784

H 1.9762739284 -3.5849226332 4.4565491199

H 0.1046660775 -3.7043585979 6.0825183925

H -1.9367102213 -2.3305267969 5.7605082738

H -2.1113046189 -0.8128924344 3.8003872930

**6-*O*-Benzoyl-2,3,4-tri-*O*-benzyl-manno-d-pyranosyl cation**


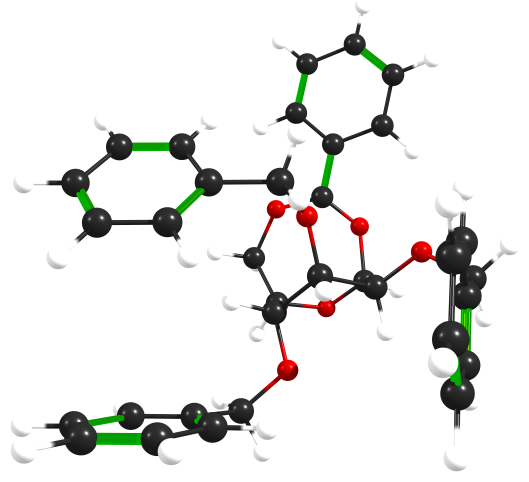


**Participation_^1^*C*_4_**

C 0.3502191938 -2.0709737731 1.7904196371

O -0.1014512052 -3.2595728022 1.1104786921

C 0.9921377406 -1.0437579527 0.8293309796

O 2.1970359040 -1.6021230061 0.3181724854

C 3.3875265061 -1.4069004367 1.1022293526

C 3.9408970461 0.0008344720 1.0387861624

C 4.1259461903 0.6362502398 -0.1991095682

C 4.6963788039 1.9082780308 -0.2621308061

C 5.0983684147 2.5582169222 0.9107891918

C 4.9187136134 1.9325985870 2.1458723600

C 4.3370786048 0.6619947160 2.2076124019

C 0.1132263701 -0.7479802991 -0.4089683681

O -1.0545077184 -0.0235917016 -0.0659142493

C -1.0544196169 1.4021840190 -0.3187574473

C -0.5675229069 2.2214718137 0.8542562879

C 0.7264829158 2.7590609737 0.8761632586

C 1.1687188992 3.5070981352 1.9717887628

C 0.3179696000 3.7291018870 3.0569117529

C -0.9794863248 3.2051912624 3.0418210418

C -1.4177037535 2.4597941651 1.9459777645

C -0.2975616802 -2.0921281664 -1.0365655607

O -1.2204896634 -1.9681674339 -2.0937600960

C -0.6939959807 -2.1873968870 -3.4236676210

C 0.2166017158 -1.0852300711 -3.9144150499

C 1.5827310450 -1.3191262897 -4.1173692674

C 2.4202674754 -0.2983161536 -4.5801193372

C 1.8959005264 0.9688452995 -4.8423091697

C 0.5309826981 1.2119303520 -4.6461534862

C -0.3021830576 0.1907669127 -4.1883413873

C -0.8830146171 -3.0554293338 0.0070169150

O -2.2813839252 -2.6004722344 0.3726771773

C -2.6897372197 -1.7103125901 1.2086599383

C -4.0605615203 -1.2378432533 1.0478529124

C -4.6926246095 -0.5177819171 2.0817434828

C -6.0025821940 -0.0822720518 1.9137473206

C -6.6835316478 -0.3545328199 0.7206878338

C -6.0558612215 -1.0643328810 -0.3095348761

C -4.7479015371 -1.5107763215 -0.1516464212

O -2.0349453908 -1.2313026084 2.2176890660

C -0.7381633083 -1.6334945158 2.7606779088

H 1.1498465177 -2.4309968889 2.4468430641

H 1.1807671894 -0.1085272284 1.3713125824

H 4.0986085731 -2.1220353084 0.6779659489

H 3.2172906126 -1.6992652969 2.1483150096

H 3.8292042957 0.1280596822 -1.1124365314

H 4.8425358746 2.3876476852 -1.2257232602

H 5.5554655010 3.5418731382 0.8588378112

H 5.2312662107 2.4287970707 3.0598225296

H 4.2048000754 0.1786324188 3.1730065877

H 0.7191319608 -0.1919184817 -1.1312329205

H -2.0983737590 1.6385032485 -0.5430859476

H -0.4579840787 1.6036811689 -1.2156771101

H 1.3939995006 2.6006783761 0.0329080069

H 2.1735654416 3.9170037930 1.9718352861

H 0.6572202089 4.3177924381 3.9039213773

H -1.6512566351 3.3932329792 3.8743941824

H -2.4335427113 2.0724712274 1.9272713929

H 0.6273502811 -2.5870305721 -1.3620722552

H -1.5892946796 -2.2593822411 -4.0463095758

H -0.1759808360 -3.1552849593 -3.4566186056

H 1.9946703154 -2.3076207742 -3.9280228946

H 3.4750841207 -0.4972534653 -4.7447298238

H 2.5414525815 1.7601418700 -5.2114168279

H 0.1167283850 2.1914525688 -4.8665347863

H -1.3641432593 0.3777276100 -4.0501197968

H -1.1104594164 -4.0257232384 -0.4306207972

H -4.1624656641 -0.3166704201 3.0053979387

H -6.4965197548 0.4642815726 2.7102951672

H -7.7061314673 -0.0122791432 0.5944167376

H -6.5873047536 -1.2673329262 -1.2333624723

H -4.2466671280 -2.0507907872 -0.9463009930

H -0.4276470662 -0.7588034356 3.3341291881

H -0.9588706005 -2.4604639687 3.4409918130


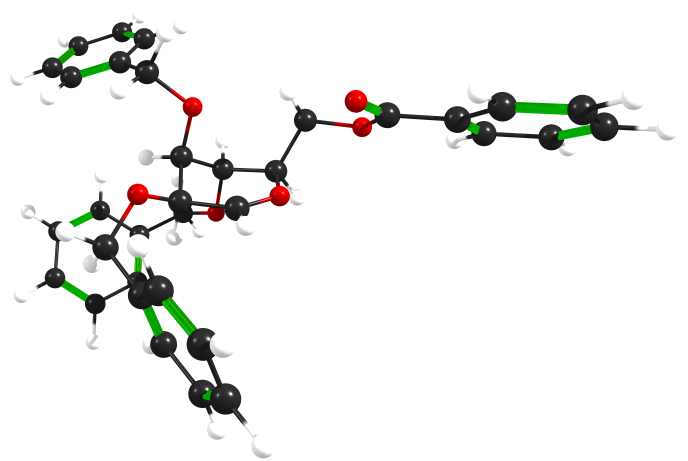


**Oxocarbenium_^3^*E***

O 1.2650727779 3.1264004890 -2.2000722117

C 2.4512563139 2.8805504163 -2.3043343559

O 2.9305564815 1.5891809523 -2.2662038204

C 1.9609576014 0.5446548287 -2.2082382058

C 1.8134755513 0.0238769580 -0.7800092149

C 0.9665285532 -1.2405343566 -0.5932520934

O 1.4790849470 -1.8787591527 0.5625209785

C 1.1966070582 -3.2961916403 0.7232094497

C 0.0877931244 -3.5642595392 1.7145944427

C 0.2710145290 -3.2443899986 3.0701965525

C -0.7397600495 -3.4929309132 4.0003090430

C -1.9442152827 -4.0753553328 3.5882029627

C -2.1301895020 -4.4084311418 2.2447932089

C -1.1186622969 -4.1528184712 1.3127997414

C -0.5398064688 -0.9058734626 -0.4719749090

O -0.9191793936 -0.2539134667 -1.6600641162

C -2.2844039137 -0.4664090993 -2.1251172168

C -2.5503936173 -1.8959997761 -2.5313762297

C -3.5167951450 -2.6605850896 -1.8644280291

C -3.7749133979 -3.9797618725 -2.2529672795

C -3.0605665537 -4.5484860014 -3.3091740697

C -2.0927117978 -3.7930268266 -3.9820997119

C -1.8435414304 -2.4751605314 -3.5982261756

C -0.7068219034 0.0038448949 0.7675404369

O -2.0363268337 0.4086097849 0.9479700247

C -2.4964014883 0.4729307462 2.3303444124

C -1.8353811007 1.5782294237 3.1181196142

C -0.8561110190 1.2897084595 4.0796187472

C -0.2198846566 2.3209740690 4.7799969563

C -0.5575784913 3.6508920869 4.5211178025

C -1.5381174424 3.9491241658 3.5669059455

C -2.1737602964 2.9194993232 2.8715048127

C 0.1881531999 1.1895746290 0.6364392227

O 1.2859766432 1.1897236277 0.0235619792

C 3.5286758853 3.8802448139 -2.4767649423

C 4.8791279495 3.5159411179 -2.6104363148

C 5.8495897153 4.5031146364 -2.7760610807

C 5.4800876540 5.8515350570 -2.8107791375

C 4.1357531349 6.2167305282 -2.6801600387

C 3.1609699087 5.2358440022 -2.5136492326

H 2.3573437365 -0.2752653188 -2.8139804246

H 1.0015040027 0.8745719922 -2.6060443074

H 2.7946306845 -0.1280194429 -0.3264222079

H 1.0987481535 -1.8692545010 -1.4848587929

H 2.1391930856 -3.7240709934 1.0763602365

H 0.9654735435 -3.7364930345 -0.2538210262

H 1.2147879513 -2.8152434510 3.3978681073

H -0.5820857876 -3.2549531951 5.0482068362

H -2.7246855052 -4.2823279096 4.3140832900

H -3.0556876254 -4.8755338397 1.9221456526

H -1.2684312828 -4.4272788333 0.2711969822

H -1.1127717319 -1.8227917741 -0.3043992561

H -2.9832129447 -0.1367920719 -1.3514481183

H -2.3619720242 0.2115914020 -2.9782930733

H -4.0811621554 -2.2183782277 -1.0469635546

H -4.5362733256 -4.5569870779 -1.7368046848

H -3.2627816737 -5.5701382575 -3.6160989093

H -1.5472575170 -4.2272197997 -4.8146935094

H -1.1068422612 -1.8847625420 -4.1373642098

H -0.3137711696 -0.5759326547 1.6244065697

H -2.3287859493 -0.5038830720 2.8005644116

H -3.5709542048 0.6373824826 2.2316605860

H -0.6033275819 0.2545887543 4.2978114561

H 0.5292977819 2.0844136077 5.5294066623

H -0.0697717620 4.4524030099 5.0671601918

H -1.8140989840 4.9816223135 3.3756527749

H -2.9422245833 3.1544907565 2.1392515676

H -0.0875661702 2.1530365749 1.0700367796

H 5.1622184586 2.4701703490 -2.5886713618

H 6.8927192920 4.2220056657 -2.8812766515

H 6.2388800392 6.6170770745 -2.9421246244

H 3.8500899972 7.2634287898 -2.7111972065

H 2.1136185654 5.5003448852 -2.4158081331


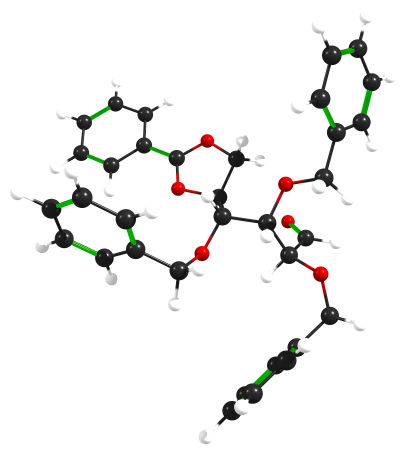


**Ring Opening_(*S*)**

O -1.6814125566 -0.0220079349 3.0402332486

C -2.6296867292 -0.0452424365 2.2804959159

C -2.5412318918 -0.4658658189 0.8161307089

O -3.8148376928 -0.3186138334 0.2038740946

C -4.7000420735 -1.4535551113 0.3409669297

C -4.2629148416 -2.6512999718 -0.4722993051

C -4.2223351340 -2.5728682062 -1.8738581608

C -3.8295164845 -3.6744686542 -2.6343086370

C -3.4741870914 -4.8727603717 -2.0028323718

C -3.5147552357 -4.9621968362 -0.6102643694

C -3.9063096710 -3.8544835843 0.1496596822

C -1.5204234313 0.3693350219 -0.0090582643

O -1.4381525434 1.7342112839 0.3585065727

C -2.5239814652 2.5855353002 -0.0948397655

C -2.1798003531 4.0095101556 0.2460543966

C -1.4016309287 4.7817758938 -0.6284986616

C -1.0600079048 6.0961333721 -0.3034773625

C -1.4956121946 6.6530330037 0.9029634236

C -2.2752932124 5.8932338272 1.7794058553

C -2.6153879602 4.5787923418 1.4504814036

C -0.0642395111 -0.1747152392 -0.0090932119

O -0.0490111441 -1.5575126472 -0.3070355650

C 0.0354991218 -1.9106737661 -1.7106919514

C 1.4364634089 -1.7977578919 -2.2631320886

C 1.7877391381 -0.7633693266 -3.1407008161

C 3.0877808218 -0.6668027909 -3.6488385147

C 4.0493115394 -1.6115961873 -3.2856554315

C 3.7070507913 -2.6537708486 -2.4152701998

C 2.4105585286 -2.7444576283 -1.9074306421

C 0.6805252091 -0.0192200835 1.3164651911

O 2.1075891366 -0.4089762138 1.0837197232

C 2.8714819508 0.3837836141 1.7671392260

C 4.2905568708 0.1797551222 1.8819571763

C 4.8893209379 -0.9240130921 1.2377689242

C 6.2599674499 -1.1169354064 1.3590097409

C 7.0292616359 -0.2226427361 2.1146548142

C 6.4349822682 0.8745607828 2.7521114299

C 5.0663277101 1.0838907736 2.6391902774

O 2.2661275109 1.3768972742 2.3454643745

C 0.8496117128 1.3871329180 1.9020535628

H -3.6434539306 0.2491021430 2.6203254898

H -2.2037698927 -1.5124968938 0.7745580695

H -5.6662393293 -1.0749128054 -0.0020768609

H -4.8026631739 -1.7281369186 1.3999919964

H -4.5115506111 -1.6482210448 -2.3669074628

H -3.8155452866 -3.6068774705 -3.7182705566

H -3.1801561110 -5.7336595238 -2.5958376331

H -3.2498230081 -5.8915227381 -0.1149431993

H -3.9478362968 -3.9337463868 1.2336220886

H -1.8781879739 0.2783563660 -1.0444646021

H -2.6383440756 2.4512035380 -1.1786007676

H -3.4572505343 2.2738581893 0.3863620175

H -1.0729124412 4.3559658662 -1.5736255902

H -0.4661948638 6.6882741851 -0.9934366977

H -1.2382474074 7.6778444445 1.1530672451

H -2.6254399156 6.3258989193 2.7117437243

H -3.2312032728 3.9945704897 2.1298530755

H 0.4867761196 0.4020379274 -0.7651752855

H -0.6628480071 -1.2988207493 -2.2942612846

H -0.3193597293 -2.9430405876 -1.7413071093

H 1.0364988571 -0.0398840414 -3.4497503051

H 3.3424932665 0.1344013440 -4.3360627540

H 5.0545915320 -1.5487814893 -3.6914436224

H 4.4450272490 -3.4068417483 -2.1532831294

H 2.1435051686 -3.5620604500 -1.2424576320

H 0.3074584630 -0.7285258033 2.0551506661

H 4.2819521365 -1.6042758510 0.6507722521

H 6.7325858582 -1.9608903286 0.8681356455

H 8.0993925067 -0.3810948505 2.2070246717

H 7.0410103521 1.5608598640 3.3337285544

H 4.5937042441 1.9289009279 3.1275769361

H 0.2335827552 1.5654582333 2.7787344113

H 0.7526616602 2.1955831759 1.1800362956


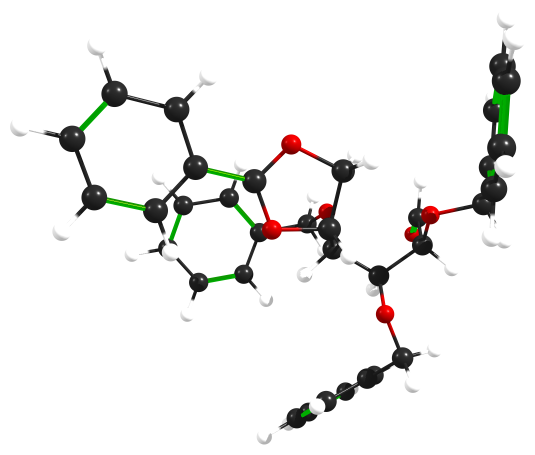


**Ring Opening_(*R*)**

O -2.1469154038 1.0245060208 -3.6441802989

C -2.4636576716 0.2978989964 -2.7274497272

C -2.5857268807 0.7967636673 -1.2854339641

O -2.9665724695 -0.2464676719 -0.4037736177

C -4.3940686479 -0.4092429411 -0.2288511641

C -4.6332636339 -1.5039621838 0.7759547656

C -4.5740891869 -1.2264535570 2.1502094097

C -4.7644373181 -2.2437200205 3.0882276321

C -5.0198415120 -3.5508148531 2.6600907921

C -5.0869839296 -3.8354031927 1.2933320809

C -4.8942678320 -2.8157112117 0.3570361197

C -1.2626341777 1.4240443336 -0.8001221237

O -1.4435804721 2.1562928554 0.4005664168

C -1.7203801753 3.5681134964 0.2359402606

C -0.5328306769 4.3510457645 -0.2744409736

C -0.5270666560 4.8917663198 -1.5673944980

C 0.5781789215 5.6109758995 -2.0346403549

C 1.6894291485 5.7954388388 -1.2095702464

C 1.6925521985 5.2618040337 0.0847871073

C 0.5884586501 4.5457472773 0.5482788837

C -0.1317996257 0.4016201067 -0.5250977064

O 0.0943847516 -0.4565291544 -1.6296142124

C 1.0010274129 0.0417706319 -2.6544990288

C 2.4446095205 0.0332120901 -2.2154090641

C 3.1473121988 -1.1797334408 -2.1295980861

C 4.4836296627 -1.1999179516 -1.7285163053

C 5.1368724267 -0.0039284239 -1.4085022337

C 4.4489791732 1.2083209395 -1.4944635944

C 3.1096188659 1.2256569118 -1.8975224993

C -0.3857716414 -0.4583054485 0.7193206897

O 0.9500605857 -0.7576255548 1.3313394447

C 1.1453611146 -2.0371591515 1.3572256043

C 2.3616722863 -2.6221396732 1.8605839549

C 3.4050177332 -1.7859923879 2.3093269829

C 4.5692636337 -2.3595366938 2.8060942772

C 4.6974339849 -3.7535235352 2.8540790483

C 3.6625667427 -4.5844510717 2.4051013315

C 2.4913145398 -4.0263292567 1.9076366834

O 0.1638459494 -2.7588086329 0.9179498151

C -0.9535729294 -1.8736822056 0.5093190028

H -2.6912687600 -0.7776676708 -2.8792943598

H -3.3382682614 1.6000707543 -1.2835227845

H -4.8114244405 0.5446578064 0.1204060938

H -4.8572115556 -0.6574470791 -1.1927903331

H -4.3922647739 -0.2079078149 2.4844783857

H -4.7302164913 -2.0153990326 4.1493742801

H -5.1804197655 -4.3399343725 3.3884543541

H -5.2999753971 -4.8456095256 0.9570001656

H -4.9585218228 -3.0387398162 -0.7051536517

H -0.9119919939 2.0809177080 -1.6048930566

H -2.0101269016 3.8963757670 1.2370719850

H -2.5838488823 3.7026812910 -0.4289253217

H -1.3940421352 4.7616516513 -2.2108127146

H 0.5660061196 6.0300547463 -3.0362134058

H 2.5442937782 6.3619682069 -1.5665467665

H 2.5484039759 5.4182911589 0.7349173538

H 0.5882962197 4.1439891685 1.5585883552

H 0.7624206911 0.9965511181 -0.3000397765

H 0.6885752249 1.0429770232 -2.9690723455

H 0.8383079583 -0.6387757544 -3.4927608187

H 2.6489343298 -2.1078153241 -2.3997877228

H 5.0222692189 -2.1420822099 -1.6852836387

H 6.1814133959 -0.0172863169 -1.1116457331

H 4.9549430836 2.1405510846 -1.2620859980

H 2.5871944916 2.1754942944 -1.9831486072

H -0.9199917785 0.1175278996 1.4722038400

H 3.2958866950 -0.7083947289 2.2611959423

H 5.3775691597 -1.7257841774 3.1548990898

H 5.6091360203 -4.1955305239 3.2442968132

H 3.7723961355 -5.6628000910 2.4472125167

H 1.6814674719 -4.6585099936 1.5605908632

H -1.1672890650 -2.1079252480 -0.5281071177

H -1.7944806061 -2.1119899676 1.1561735096

**3-Acetyloxy-pyranosyl cation**

*Local minima*

**^1^*C*_4_ conformation [R1] (0.0 kcal / mol)**

D1 = 60°

D3 = 45°

D5 = 45°

H -2.555624 1.273076 0.022801

C -1.569861 1.209401 -0.443143

C -0.760196 -0.080707 1.511539

O -1.326515 -1.272916 -0.553251

C -0.572259 -1.248398 0.567735

C -1.451244 -0.035296 -1.314199

C -0.548044 1.172031 0.686392

H -0.066585 -0.157781 2.349902

H -0.566600 -2.233739 1.019268

H -2.337636 -0.177288 -1.926917

H -0.542598 2.083070 1.276751

H -1.775972 -0.093372 1.909167

H -0.587756 0.025754 -1.982129

H -1.436025 2.095151 -1.066787

O 0.835899 1.137286 0.122181

C 1.458564 0.025013 -0.064686

C 2.873466 0.107871 -0.499545

H 3.119183 -0.745563 -1.128622

H 3.500950 0.068073 0.396916

H 3.055953 1.049851 -1.012149

O 0.941886 -1.125210 0.115724

**^3^*H*_4_ conformation [R2] (1.4 kcal / mol)**

D1 = 30°

D3 = 45°

D5 = 0°

H 1.356605 1.534853 -1.423996

C 0.740356 1.241862 -0.569319

C 1.217237 -1.231211 -0.765310

O 2.497502 0.100148 0.802506

C 2.319124 -0.959906 0.163661

C 1.567601 1.279010 0.695279

C 0.153274 -0.142232 -0.846909

H 0.788503 -2.204247 -0.497885

H 3.085739 -1.713253 0.342947

H 2.246391 2.123840 0.755781

H -0.339408 -0.155329 -1.816457

H 1.700709 -1.409324 -1.738720

H 0.979179 1.206420 1.608392

H -0.058753 1.978651 -0.482373

O -0.813866 -0.481623 0.178636

C -2.086990 -0.020712 -0.000842

C -2.979078 -0.461703 1.123234

H -2.985186 -1.551736 1.186217

H -3.987464 -0.091936 0.953422

H -2.596042 -0.078619 2.071574

O -2.406062 0.647728 -0.950851

***B_2,_*_5_ conformation [R1] (1.9 kcal / mol)**

D1 = 45°

D3 = -45°

D5 = 15°

H -1.577883 2.258923 -0.715993

C -1.084829 1.286769 -0.679560

C -0.730111 -0.069479 1.454276

O -1.664295 -1.127183 -0.490334

C -0.854420 -1.218621 0.513314

C -2.158775 0.217067 -0.837147

C -0.286153 1.148780 0.638903

H -0.037747 -0.292481 2.264076

H -0.681033 -2.237913 0.833769

H -3.017542 0.397338 -0.189546

H -0.322078 2.051573 1.238861

H -1.713224 0.127474 1.894042

H -2.498348 0.123935 -1.863981

H -0.393583 1.255283 -1.522724

O 1.144907 1.021742 0.334466

C 1.598214 -0.129012 -0.108447

C 3.036889 -0.125469 -0.496051

H 3.430643 -1.138873 -0.469990

H 3.601674 0.535367 0.160457

H 3.114334 0.260431 -1.517008

O 0.890625 -1.144718 -0.222094

**^4^*H*_3_ conformation [R2] (2.1 kcal / mol)**

D1 = -30°

D3 = -45°

D5 = 15°

H -0.098663 -1.892776 0.471893

C -0.698647 -0.988597 0.582299

C -1.029094 1.362047 -0.249335

O -2.952849 -0.087613 -0.035278

C -2.452354 1.059091 -0.078254

C -2.114795 -1.322091 0.173139

C -0.114587 0.139855 -0.271541

H -0.745857 2.060824 0.550731

H -3.189349 1.861817 -0.080872

H -2.190156 -1.837755 -0.783230

H 0.029591 -0.211148 -1.294176

H -0.968182 1.966932 -1.165507

H -2.677679 -1.860216 0.930137

H -0.668875 -0.691285 1.633853

O 1.147676 0.578269 0.251649

C 2.253388 -0.128363 -0.129359

C 3.495388 0.469193 0.464963

H 3.623625 1.489619 0.097010

H 4.357035 -0.134944 0.191255

H 3.401088 0.520145 1.551431

O 2.191626 -1.092908 -0.848121

**^3^*H*_4_ conformation [R1] (3.3 kcal / mol)**

D1 = 30°

D3 = 45°

D5 = 15°

H -1.991336 1.923548 -0.186574

C -1.089422 1.414889 -0.538690

C -0.796223 -0.073705 1.482686

O -1.952977 -0.925541 -0.481074

C -1.524807 -1.072178 0.687078

C -1.485551 0.200422 -1.349714

C -0.227503 1.105728 0.688708

H -0.060655 -0.578910 2.110678

H -1.841177 -2.006279 1.150127

H -2.335935 0.379462 -2.000532

H -0.198940 1.983220 1.328747

H -1.555219 0.295526 2.193906

H -0.670895 -0.251499 -1.909763

H -0.563257 2.112047 -1.193179

O 1.175188 0.923057 0.350473

C 1.630165 -0.259097 -0.112791

C 3.113915 -0.228883 -0.325242

H 3.441328 -1.173896 -0.751639

H 3.614643 -0.057005 0.630287

H 3.377652 0.597538 -0.987839

O 0.910332 -1.215867 -0.316452

**4-Acetyloxy-pyranosyl cation**

|  |
| --- |

*Local minima*

**^4^*H*_3_ conformation [R2] (0.0 kcal / mol)**

D1 = -30°

D3 = -45°

D5 = 0°

H -1.285334 2.081536 0.766658

C -0.712905 1.412000 0.126321

C -1.263492 -0.786276 1.239342

C -2.273596 -0.079669 -0.965181

C -2.135422 -1.139009 0.032397

O -1.678172 1.023377 -0.952532

C -0.171127 0.212606 0.875168

H -0.807906 -1.684942 1.651092

H -2.926762 -0.208751 -1.827708

H -3.150146 -1.456315 0.304316

H 0.048261 1.967205 -0.411323

H -1.885126 -0.345916 2.021587

H -1.746458 -1.997040 -0.540641

H 0.345070 0.582779 1.760945

O 0.765722 -0.503490 0.034422

C 2.038938 -0.011893 -0.027337

O 2.365884 1.002702 0.534655

C 2.917837 -0.898496 -0.859686

H 3.899908 -0.443388 -0.963735

H 2.468580 -1.056891 -1.841750

H 3.011045 -1.874560 -0.377948

**^1^*S*_5_ conformation [R1] (1.4 kcal / mol)**

D1 = 15°

D3 = -60°

D5 = 30°

H -2.364852 1.062357 -1.024816

C -1.288633 0.893498 -1.105958

C -1.098521 0.549578 1.392551

C -0.778231 -1.284070 -0.292553

C -1.388001 -0.920772 1.032954

O -1.038352 -0.513678 -1.356737

C -0.609295 1.322257 0.175955

H -0.365278 0.634979 2.195872

H -0.798590 -2.330796 -0.572536

H -2.463739 -1.094647 0.928358

H -0.902377 1.421999 -1.974198

H -2.007494 1.037046 1.748783

H -1.025989 -1.610462 1.794236

H -0.672236 2.397833 0.312861

O 0.871960 1.118447 0.002887

C 1.443366 -0.042276 -0.006801

O 0.850048 -1.158880 -0.033083

C 2.929614 -0.024885 0.044621

H 3.330896 -0.940695 -0.382318

H 3.228893 0.039703 1.095620

H 3.309720 0.855585 -0.471006

**^3^*H*_4_ conformation [R1] (1.7 kcal / mol)**

D1 = 30°

D3 = 45°

D5 = 0°

H 0.203778 -1.980681 -0.160792

C 0.644625 -1.057417 -0.523111

C 1.043602 1.387035 0.013408

C 2.882635 -0.350096 0.045431

C 2.502591 1.033731 0.314345

O 2.108138 -1.268185 -0.314934

C 0.128415 0.182799 0.200500

H 0.960651 1.729396 -1.020755

H 3.915088 -0.677069 0.162756

H 2.773962 1.184194 1.373370

H 0.534142 -0.977625 -1.603738

H 0.715503 2.201761 0.657256

H 3.219769 1.670316 -0.219213

H 0.003645 -0.050431 1.259796

O -1.149111 0.515595 -0.359099

C -2.246154 -0.112541 0.170589

O -2.157901 -0.967996 1.012690

C -3.506858 0.416825 -0.446066

H -4.363405 -0.109381 -0.031790

H -3.473068 0.285004 -1.529431

H -3.592207 1.487192 -0.247292

**^1,4^*B* conformation [R1] (2.3 kcal / mol)**

D1 = 0°

D3 = -45°

D5 = 45°

H -2.183200 1.157934 -1.380833

C -1.164484 0.790115 -1.242288

C -1.156788 0.709291 1.316898

C -0.818376 -1.303683 -0.156031

C -1.274850 -0.819513 1.191045

O -1.219697 -0.659842 -1.255637

C -0.609696 1.342763 0.050441

H -0.524835 1.000056 2.156231

H -0.864047 -2.374211 -0.318461

H -2.316670 -1.138472 1.286586

H -0.559291 1.091747 -2.096198

H -2.137710 1.152284 1.501517

H -0.713593 -1.346616 1.961988

H -0.674175 2.426663 0.059018

O 0.890847 1.125294 0.027423

C 1.441085 -0.043623 -0.018386

O 0.834150 -1.152008 -0.094514

C 2.927838 -0.057560 0.048713

H 3.314212 -0.950278 -0.437763

H 3.214509 -0.081426 1.105023

H 3.334019 0.848035 -0.397638

**3-*O*-Acetyl-2,4,6-tri-*O*-methyl-gluco-d-pyranosyl cation**

*Local minima*

***E*_2_ conformation [R1] (0.0 kcal / mol)**

D1 = 60°

D3 = 0°

D5 = 30°

O 0.170122 -0.175714 -1.783128

C -0.497420 1.060290 0.379617

C -1.354690 -1.268112 -0.244505

C -0.853792 -0.353758 0.875598

C -0.212142 -1.339813 -1.242526

C 0.054527 1.102985 -1.070487

H 0.267912 1.453830 1.055402

H -1.570713 -2.275484 0.144577

H -1.541360 -0.293530 1.713609

H -0.322969 -2.088537 -2.019481

H -0.646122 1.693460 -1.657792

O -2.441507 -0.726186 -0.938026

O 0.359594 -0.952261 1.480995

O -1.650099 1.880283 0.410406

C 1.439100 1.720800 -1.115106

H 1.373092 2.770749 -0.797340

H 1.822127 1.691049 -2.141829

C -3.711091 -0.865776 -0.286645

H -3.907990 -1.914464 -0.042554

H -4.452780 -0.512419 -0.999782

H -3.767449 -0.255373 0.618387

C 1.182388 -1.649860 0.778325

C -1.872915 2.540469 1.658911

H -2.083686 1.832503 2.468274

H -2.743175 3.178460 1.515301

H -1.009423 3.155518 1.934419

O 2.273644 0.976558 -0.241620

C 3.613841 1.458308 -0.201427

H 3.648377 2.498820 0.142474

H 4.085986 1.391456 -1.188419

H 4.159619 0.829899 0.501262

C 2.382300 -2.178847 1.466146

H 2.183039 -3.218227 1.745587

H 2.589086 -1.600975 2.363638

H 3.227974 -2.167546 0.780559

O 0.996723 -1.943842 -0.444090

***B*_2,5_ conformation [R1] (0.3 kcal / mol)**

D1 = 45°

D3 = -45°

D5 = 15°

O 0.274521 0.090207 -1.717526

C 0.399818 0.515362 0.721295

C -1.850421 0.290755 -0.552017

C -1.114649 0.164526 0.789321

C -0.950427 -0.367463 -1.577123

C 0.831833 0.970325 -0.679727

H 0.977713 -0.372732 0.997010

H -2.817864 -0.234105 -0.514297

H -1.606976 0.720591 1.580518

H -1.387083 -0.668582 -2.522361

H 0.438747 1.968761 -0.866453

O -1.997015 1.632028 -0.941777

O -1.238967 -1.252773 1.205286

O 0.715396 1.594019 1.571477

C 2.333662 0.960791 -0.869617

H 2.761024 1.752874 -0.241325

H 2.559445 1.193225 -1.918620

C -3.191685 2.268744 -0.462803

H -4.078265 1.734220 -0.816429

H -3.181300 3.275030 -0.875476

H -3.205589 2.326364 0.629105

C -1.035523 -2.169907 0.308548

C 0.938338 1.226600 2.935658

H 0.032064 0.822062 3.400620

H 1.225591 2.138242 3.456213

H 1.744373 0.489324 3.012915

O 2.837803 -0.309217 -0.510440

C 4.249457 -0.404306 -0.655600

H 4.764552 0.326703 -0.019971

H 4.552351 -0.244958 -1.697879

H 4.534400 -1.410322 -0.349805

C -1.002013 -3.573082 0.791902

H -1.439483 -4.231406 0.043347

H -1.514824 -3.658176 1.747343

H 0.047289 -3.855473 0.924523

O -0.848802 -1.924977 -0.915520

**^1^*C*_4_ conformation [R1] (1.8 kcal / mol)**

D1 = 60°

D3 = 30°

D5 = 45°

O 0.021532 -0.064615 1.838949

C 0.655817 1.123974 -0.313417

C 1.247448 -1.310778 0.133711

C 0.757226 -0.296615 -0.911265

C 0.209150 -1.265026 1.244856

C -0.095875 1.155949 1.035202

H 0.081683 1.738695 -1.015418

H 1.255199 -2.318814 -0.309743

H 1.369728 -0.280643 -1.807870

H 0.339807 -2.020051 2.012359

H 0.435651 1.890333 1.640859

O 2.469032 -1.006252 0.733741

O -0.549977 -0.764629 -1.404866

O 1.959778 1.640583 -0.160470

C -1.542155 1.619866 0.985392

H -1.538903 2.690459 0.738406

H -1.976926 1.501623 1.985269

C 3.621200 -1.228013 -0.085286

H 3.609537 -2.237687 -0.509768

H 4.482191 -1.120723 0.571404

H 3.689971 -0.487342 -0.886424

C -1.363898 -1.405019 -0.638134

C 2.371793 2.517566 -1.214003

H 2.401293 2.006653 -2.182548

H 3.376130 2.850500 -0.958135

H 1.706488 3.384840 -1.281841

O -2.297855 0.905001 0.022161

C -3.649557 1.357967 -0.052747

H -3.686967 2.418060 -0.326817

H -4.165392 1.213738 0.903108

H -4.145304 0.771483 -0.824501

C -2.619884 -1.897096 -1.245665

H -2.442390 -2.921522 -1.589818

H -2.893028 -1.281737 -2.099289

H -3.408473 -1.918820 -0.496645

O -1.107245 -1.724548 0.571178

**^4^*H*_3_ conformation [R2] (2.9 kcal / mol)**

D1 = -45°

D3 = -45°

D5 = 0°

O -1.463233 1.704490 -0.803861

C -0.609544 -0.576128 -0.147960

C 0.743613 1.496756 0.232303

C 0.779790 0.052794 -0.278023

C -0.455650 2.227158 -0.285998

C -1.628659 0.200487 -0.991722

H -0.930700 -0.569763 0.897796

H 1.116597 0.034913 -1.315638

H -0.469489 3.316679 -0.262488

H -1.433695 0.069138 -2.055715

O 1.702823 -0.678577 0.525504

O -0.611496 -1.882996 -0.675769

C -3.077820 -0.082412 -0.673440

H -3.278063 -1.119738 -0.974739

H -3.719110 0.575614 -1.273648

C 2.805392 -1.217784 -0.095201

C -0.799934 -2.929132 0.288864

H -1.766900 -2.829240 0.791892

H 0.002749 -2.927603 1.030585

H -0.777458 -3.862030 -0.271364

O -3.275816 0.116802 0.706740

C -4.611941 -0.156398 1.124418

H -4.651355 0.031759 2.196049

H -4.879157 -1.201278 0.928224

H -5.325846 0.499731 0.613496

O 3.026336 -1.089846 -1.268840

C 3.642626 -1.969795 0.896931

H 4.573395 -2.277956 0.426528

H 3.094582 -2.851819 1.237172

H 3.844879 -1.348213 1.770448

H 0.647266 1.479842 1.329146

O 1.875944 2.212495 -0.188802

C 2.518006 3.005009 0.825332

H 1.865544 3.808550 1.178762

H 3.398278 3.435394 0.353434

H 2.816748 2.373753 1.667279

**3-*O*-Acetyl-2,4,6-tri-*O*-methyl-manno-d-pyranosyl cation**

*Local minima*

**^1^*C*_4_ conformation [R1] (0.0 kcal / mol)**

D1 = 60°

D3 = 30°

D5 = 45°

O -0.184088 0.507981 1.830406

C 0.015547 1.457086 -0.496116

C 1.724188 -0.130116 0.445321

C 0.925501 0.249771 -0.794182

C 0.685066 -0.472864 1.498325

C -0.824595 1.320306 0.793859

H -0.660199 1.604866 -1.345125

H 1.539556 0.471795 -1.660688

H 1.100237 -0.907128 2.401395

H -0.848662 2.310180 1.250682

O 0.143457 -0.924889 -1.208031

O 0.942856 2.523634 -0.391952

C -2.276454 0.906069 0.609448

H -2.808974 1.752586 0.153695

H -2.714147 0.721873 1.598300

C -0.282302 -1.775624 -0.339698

C 0.425217 3.803283 -0.763928

H -0.389195 4.119240 -0.102849

H 0.068599 3.791789 -1.799401

H 1.250244 4.506792 -0.669756

O -2.393826 -0.239516 -0.216228

C -3.752105 -0.618666 -0.434292

H -4.240195 -0.891356 0.508091

H -3.743943 -1.479548 -1.100973

H -4.310886 0.196607 -0.907333

H 2.293475 0.746148 0.784265

O 2.528493 -1.265672 0.266776

C 3.790545 -1.012326 -0.363525

H 4.321937 -1.961671 -0.369374

H 4.364006 -0.273784 0.205167

H 3.666220 -0.665276 -1.393853

O -0.092182 -1.676843 0.917793

C -0.956500 -2.986650 -0.855525

H -1.666480 -3.358007 -0.119972

H -0.186492 -3.749345 -1.012754

H -1.437428 -2.774940 -1.807764

**^3^*H*_4_ conformation [R2] (6.7 kcal / mol)**

D1 = 45°

D3 = 45°

D5 = 0°

O 1.176919 -0.692963 1.455273

C 0.466049 -1.031744 -0.925227

C -1.199549 -0.877097 0.909178

C -0.870521 -0.396431 -0.517703

C -0.014051 -0.803757 1.809993

C 1.613894 -0.613118 -0.004761

H 0.717541 -0.718458 -1.944323

H -1.652761 -0.707266 -1.205784

H -0.174144 -0.825746 2.888192

H 2.401365 -1.365046 -0.014360

O -0.734729 1.024151 -0.532103

O 0.251106 -2.426900 -0.845176

C 2.249390 0.751567 -0.191070

H 1.557448 1.552809 0.088133

H 2.477527 0.856832 -1.262286

C -1.822885 1.757617 -0.946861

C 1.054315 -3.220011 -1.728002

H 2.121007 -3.122255 -1.502908

H 0.871657 -2.938182 -2.769403

H 0.751251 -4.252769 -1.569191

O 3.420977 0.774358 0.590713

C 4.115938 2.015469 0.508325

H 4.991311 1.928579 1.149949

H 3.489819 2.844417 0.859623

H 4.439258 2.221198 -0.519373

**4-*O*-Acetyl-2,3,6-tri-*O*-methyl-gluco-d-pyranosyl cation**

*Local minima*

***E*_3_ / ^2^*S*_O_ conformation [R2] (0.0 kcal / mol)**

D1 = -60°

D3 = 0°

D5 = 15°

O -0.667590 1.096455 1.805018

C 0.731562 -0.133634 0.073486

C -1.760115 -0.335079 0.188760

C -0.420365 -1.133147 0.183707

C -1.718879 0.573162 1.365319

C 0.597253 1.114408 0.978321

H 0.799825 0.193601 -0.963281

H -1.789797 0.268110 -0.729534

H -0.337605 -1.698789 1.118553

H -2.593931 0.702996 2.001764

H 1.368104 1.119408 1.743789

O -2.846752 -1.203833 0.319893

O -0.366052 -1.975717 -0.929501

O 1.920588 -0.858034 0.421243

C 0.552123 2.434501 0.234138

H 1.507499 2.536998 -0.295005

H 0.444764 3.263905 0.943574

C -3.978818 -0.884521 -0.505513

H -4.410782 0.083453 -0.232035

H -4.711287 -1.669221 -0.330356

H -3.690367 -0.874394 -1.560798

C -0.661398 -3.355683 -0.671846

H -1.680632 -3.476173 -0.299184

H 0.051365 -3.774508 0.045572

H -0.556408 -3.867715 -1.626732

C 3.089021 -0.429733 -0.144575

O -0.543895 2.370208 -0.652418

C -0.749753 3.571811 -1.399454

H -0.947768 4.416208 -0.730545

H -1.616067 3.400392 -2.035594

H 0.122029 3.796176 -2.022293

C 4.216926 -1.359440 0.190030

H 5.149600 -0.953082 -0.193662

H 4.025573 -2.337482 -0.257251

H 4.280021 -1.497959 1.270853

O 3.153767 0.569945 -0.814492

***B*_1,4_ conformation [R1] (0.1 kcal / mol)**

D1 = 0°

D3 = 45°

D5 = -60°

O -0.010651 -0.636069 -1.724659

C -0.469564 -0.491171 0.680036

C 1.079515 1.138750 -0.484766

C 0.095353 0.944709 0.694521

C 1.192468 -0.156415 -1.307857

C -0.958896 -0.970997 -0.676815

H -1.271204 -0.612704 1.401988

H 0.645147 1.880522 -1.173282

H 0.662379 1.082374 1.621908

H 1.826855 -0.047860 -2.180802

H -1.043442 -2.060701 -0.656203

O 2.308075 1.569403 0.041646

O -0.933183 1.897311 0.584434

O 0.574519 -1.412650 1.218275

C -2.315779 -0.385198 -1.066583

H -2.589410 -0.793415 -2.048192

H -2.256085 0.705411 -1.142161

C 3.174475 2.235913 -0.884398

H 3.585118 1.548179 -1.630400

H 3.992741 2.644640 -0.294577

H 2.646041 3.050815 -1.389852

C -1.591513 2.219555 1.815535

H -0.865840 2.555388 2.563125

H -2.158596 1.370438 2.209218

H -2.281217 3.029735 1.586980

C 1.669054 -1.677014 0.604199

O -3.229959 -0.783585 -0.066430

C -4.563287 -0.358000 -0.331075

H -5.177109 -0.704397 0.499157

H -4.937238 -0.793901 -1.265069

H -4.623844 0.734818 -0.396537

C 2.619790 -2.595993 1.270870

H 3.011564 -3.302891 0.539135

H 2.139578 -3.108651 2.099761

H 3.459114 -1.998982 1.640734

O 1.996920 -1.209868 -0.536631

**4-*O*-Acetyl-2,3,6-tri-*O*-methyl-manno-d-pyranosyl cation**

*Local minima*

**^3^*E* conformation [R2] (0.0 kcal / mol)**

D1 = 60°

D3 = 30°

D5 = 0°

O -0.336889 0.910548 -1.391159

C -0.229545 -0.643705 0.616218

C 1.808817 0.215868 -0.493390

C 1.078234 0.117519 0.868035

C 0.911471 0.953774 -1.427589

C -1.134957 0.092046 -0.380135

H -0.774442 -0.745954 1.553964

H 1.705164 -0.456366 1.555426

H 1.319910 1.632743 -2.176343

H -1.653619 -0.603632 -1.036082

O 0.737487 1.378360 1.379450

O 0.140066 -1.939707 0.127009

C -2.135364 1.059009 0.221544

H -1.615630 1.881094 0.726699

H -2.703969 0.494056 0.974183

C 1.694281 1.968656 2.275986

H 1.231152 2.879531 2.650211

H 2.621722 2.208125 1.754225

H 1.898101 1.292946 3.112177

C -0.804026 -2.929365 0.253366

O -2.967161 1.514795 -0.817857

C -3.946620 2.447363 -0.369488

H -4.615245 1.994183 0.372357

H -4.525440 2.740241 -1.244039

H -3.475712 3.335817 0.068204

H 1.927455 -0.810130 -0.879785

O 3.022277 0.904434 -0.380880

C 4.092571 0.386004 -1.186700

H 4.963198 0.998247 -0.963216

H 3.861417 0.460415 -2.253934

H 4.296332 -0.657306 -0.926978

O -1.900718 -2.706203 0.694456

C -0.271731 -4.243688 -0.230263

H 0.634078 -4.503941 0.320579

H -0.005738 -4.165935 -1.286835

H -1.028020 -5.012844 -0.094467

**^5^*S*_1_ conformation [R1] (1.9 kcal / mol)**

D1 = -15°

D3 = 60°

D5 = -30°

O 0.661214 -0.509747 -1.732150

C 0.242257 -0.857469 0.623637

C 0.445738 1.505277 -0.292471

C 0.085961 0.640473 0.957946

C 1.185959 0.658380 -1.325646

C -0.163636 -1.244841 -0.785897

H -0.270952 -1.481138 1.347925

H 0.785039 0.873827 1.770095

H 1.561958 1.218838 -2.173205

H 0.093176 -2.291390 -0.951586

O -1.230508 0.926292 1.347669

O 1.677260 -1.239912 0.814504

C -1.648965 -1.055249 -1.088685

H -1.818326 -1.339358 -2.135860

H -1.949272 -0.012919 -0.954502

C -1.514540 0.663051 2.726046

H -1.436737 -0.402671 2.963385

H -2.540544 0.985964 2.890454

H -0.840768 1.231326 3.375766

C 2.668443 -0.621684 0.265171

O -2.335037 -1.916201 -0.200782

C -3.749209 -1.847391 -0.349590

H -4.178908 -2.537531 0.375343

H -4.054390 -2.147141 -1.359524

H -4.118506 -0.834404 -0.150169

H 1.115850 2.322808 0.004156

O -0.670061 2.005684 -0.985172

C -1.104116 3.307056 -0.567231

H -0.292494 4.034422 -0.675988

H -1.455023 3.287549 0.465742

H -1.924111 3.578617 -1.229667

O 2.574763 0.308478 -0.596134

C 4.024718 -1.066336 0.671836

H 4.009995 -1.456796 1.686938

H 4.731038 -0.245298 0.566208

H 4.326270 -1.869060 -0.009690

**4-*O*-Acetyl-2,3,6-tri-*O*-methyl-galacto-d-pyranosyl cation**

*Local minima*

**^1^*S*_5_ conformation [R1] (0.0 kcal / mol)**

D1 = 15°

D3 = -60°

D5 = 30°

O -0.232023 -1.188634 -1.250850

C -0.487954 0.212950 0.713473

C 1.808038 -0.638256 0.027083

C 0.997610 0.089409 1.111203

C 1.018428 -0.705044 -1.278798

C -0.999383 -1.004977 -0.034912

H 1.036843 -0.534222 2.012001

H 1.548316 -1.098647 -2.138457

H -0.846221 -1.878746 0.604769

O 1.600913 1.343171 1.326654

C -2.465373 -0.933119 -0.441219

H -2.717591 -1.842806 -1.000920

H -2.642501 -0.068868 -1.095052

C 1.198672 2.007089 2.531114

H 0.157129 2.339755 2.482527

H 1.845768 2.876274 2.630045

H 1.333403 1.349625 3.395925

O -3.201475 -0.835569 0.757220

C -4.608795 -0.776067 0.544655

H -5.074022 -0.705996 1.526718

H -4.968817 -1.678423 0.036437

H -4.882073 0.103749 -0.050250

H 2.736829 -0.086222 -0.147744

O 2.051724 -1.952712 0.483709

C 3.169540 -2.611462 -0.125197

H 2.999508 -2.812700 -1.187347

H 3.284138 -3.558383 0.398757

H 4.079746 -2.014820 -0.006568

H -1.139449 0.446272 1.549398

O -0.641238 1.414368 -0.164895

C 0.133156 1.663621 -1.167574

O 0.933902 0.839538 -1.710865

C 0.063382 3.038979 -1.719644

H 0.777711 3.651072 -1.159243

H -0.932364 3.452584 -1.570124

H 0.345281 3.040458 -2.769757

**^.4^*H*_3_ conformation [R2] (1.8 kcal / mol)**

D1 = -45°

D3 = -45°

D5 = 0°

O 1.006641 -1.820928 0.234623

C 0.164569 0.389339 -0.615052

C -1.371745 -1.289715 0.401222

C -1.198761 -0.317832 -0.772657

C -0.137846 -2.083786 0.659150

C 1.308546 -0.604095 -0.639498

H -1.201274 -0.887591 -1.710968

H -0.213830 -3.001513 1.242344

H 1.416289 -1.059029 -1.623542

O -2.280122 0.570393 -0.713715

C 2.646666 -0.088794 -0.159877

H 2.615615 0.096661 0.921060

H 2.825073 0.873495 -0.662616

C -2.596423 1.223167 -1.950557

H -1.801865 1.911128 -2.251576

H -3.507230 1.790132 -1.768230

H -2.775830 0.486843 -2.740440

O 3.620190 -1.043561 -0.501798

C 4.921947 -0.696082 -0.036497

H 5.591199 -1.495208 -0.350777

H 4.941374 -0.614782 1.056857

H 5.258819 0.251908 -0.472873

H -1.505396 -0.680878 1.313950

O -2.409996 -2.209972 0.213927

C -3.595033 -1.962678 0.993020

H -3.388234 -2.072031 2.061704

H -4.316589 -2.715827 0.683909

H -3.983637 -0.964260 0.782660

H 0.319896 1.093397 -1.434789

O 0.180800 1.095902 0.632180

C -0.022401 2.457077 0.587832

O -0.111429 3.064026 -0.445089

C -0.105645 3.021469 1.973557

H -1.029333 2.676582 2.444951

H -0.106459 4.107649 1.923483

H 0.729486 2.668027 2.580012

**6-*O*-Acetyl-2,3,4-tri-*O*-methyl-gluco-d-pyranosyl cation**

*Local minima*

***E*_3_ conformation [R2] (0.0 kcal / mol)**

D1 = -60°

D3 = -30°

D5 = 0°

O 0.210700 -0.965278 -1.755194

C -0.213743 1.012891 -0.187623

C -1.533513 -1.082819 -0.077822

C -1.646032 0.463943 -0.228343

C -0.719104 -1.601159 -1.208959

C 0.636926 0.432531 -1.334070

H 0.233096 0.761872 0.779480

H -0.992248 -1.283383 0.860985

H -2.122417 0.712070 -1.183438

H -0.945133 -2.563874 -1.667572

H 0.448640 1.005152 -2.241113

O -2.792349 -1.692482 -0.108484

O -2.360379 0.995292 0.850757

O -0.170119 2.402832 -0.415524

C 2.121235 0.353562 -1.078926

H 2.489629 1.366549 -0.903099

H 2.643334 -0.063270 -1.941188

C -2.985902 -2.732975 0.863992

H -2.310654 -3.575580 0.685046

H -4.015389 -3.064869 0.750772

H -2.829898 -2.343045 1.874301

C -3.734996 1.308603 0.581867

H -3.810546 2.069911 -0.200979

H -4.141662 1.703495 1.511203

H -4.289372 0.415131 0.287769

C -0.056297 3.211495 0.765137

H 0.002222 4.241448 0.417427

H 0.856307 2.962827 1.317288

H -0.926378 3.085392 1.411180

O 2.322242 -0.474429 0.075130

C 3.599351 -0.523126 0.556938

O 4.499816 0.091584 0.047659

C 3.687404 -1.411416 1.764005

H 4.727258 -1.509311 2.066572

H 3.265211 -2.393023 1.542191

H 3.106751 -0.976830 2.581229

**^4^*H*_3_ conformation [R2] (0.2 kcal / mol)**

D1 = -45°

D3 = -45°

D5 = 0°

O -0.053376 -1.676630 -1.253920

C -0.053025 0.659222 -0.298448

C -1.901023 -0.952922 0.172476

C -1.577846 0.498441 -0.218113

C -1.086874 -1.936813 -0.600003

C 0.538567 -0.278691 -1.358100

H 0.382966 0.441197 0.682254

H -1.613872 -1.084897 1.228260

H -2.021224 0.707354 -1.198100

H -1.412383 -2.975086 -0.659170

H 0.233969 0.037476 -2.356063

O -3.249573 -1.267458 -0.054533

O -2.116062 1.338705 0.772275

O 0.283516 1.959236 -0.731848

C 2.035488 -0.468837 -1.322425

H 2.510807 0.497175 -1.493815

H 2.346999 -1.167225 -2.100180

C -3.975534 -1.736377 1.095150

H -3.600433 -2.706829 1.432300

H -5.009591 -1.839529 0.773687

H -3.909049 -1.006765 1.906868

C -2.886412 2.443095 0.278944

H -2.276953 3.100638 -0.346102

H -3.230004 2.988764 1.156178

H -3.751780 2.088851 -0.289634

C 0.906509 2.796702 0.255253

H 1.039871 3.768121 -0.217503

H 1.884371 2.400216 0.545054

H 0.268077 2.901836 1.135534

O 2.422277 -1.004025 -0.049601

C 3.482743 -0.418266 0.586579

O 4.041831 0.559579 0.161441

C 3.821598 -1.164353 1.843763

H 4.601050 -0.632744 2.384310

H 4.169214 -2.167526 1.586069

H 2.933918 -1.273500 2.469090

**^4^*H*_3_ conformation [R1] (0.5 kcal / mol)**

D1 = -45°

D3 = -45°

D5 = 0°

O 0.781688 -1.038443 -0.222674

C -0.546704 1.065102 0.205171

C -1.612674 -1.196123 0.237151

C -1.761669 0.253280 -0.268825

C -0.232041 -1.726116 0.028530

C 0.751307 0.469473 -0.354286

H -0.517540 1.073423 1.302243

H -1.785531 -1.181619 1.325481

H -1.785016 0.251669 -1.364268

H -0.054192 -2.801293 0.038668

H 0.809980 0.614097 -1.432489

O -2.493689 -2.066693 -0.428559

O -2.930107 0.823208 0.261789

O -0.576344 2.375320 -0.312987

C 2.008225 0.972576 0.325880

H 2.071263 0.614848 1.353592

H 1.971868 2.061138 0.307320

C -3.181243 -3.007839 0.414625

H -2.482143 -3.695895 0.898645

H -3.843310 -3.568974 -0.240835

H -3.765405 -2.480027 1.174107

C -4.045230 0.878076 -0.639316

H -3.806457 1.491513 -1.513893

H -4.860093 1.340659 -0.085062

H -4.342562 -0.123100 -0.960331

C -1.055014 3.381781 0.592667

H -1.024352 4.319416 0.040518

H -0.402212 3.455228 1.468751

H -2.077602 3.167845 0.906514

O 3.169452 0.576691 -0.410711

C 3.949488 -0.412530 0.119412

O 3.691209 -0.972050 1.154016

C 5.137915 -0.670100 -0.760852

H 5.728675 -1.484259 -0.347912

H 5.746024 0.234660 -0.828023

H 4.806771 -0.919075 -1.770957

***E*_4_–^2^*S*_O_ conformation [R1] (0.5 kcal / mol)**

D1 = -15°

D3 = 60°

D5 = 0°

O 0.614516 0.688677 -1.340538

C -1.112865 1.438996 0.183190

C -0.747797 -1.066155 -0.372717

C -1.604305 -0.007236 0.416276

C 0.096892 -0.448744 -1.426400

C 0.384029 1.515345 -0.093970

H -1.329077 2.015452 1.089001

H -0.035531 -1.505023 0.344656

H -2.620649 -0.104462 0.021892

H 0.263332 -0.955767 -2.375921

H 0.655754 2.504448 -0.450295

O -1.590257 -2.032138 -0.943934

O -1.561240 -0.244727 1.799283

O -1.730439 1.992472 -0.961399

C 1.292428 1.070677 1.042706

H 1.013325 0.107571 1.462807

H 1.206676 1.826581 1.823581

C -1.026726 -3.349255 -1.047502

H -0.164146 -3.368635 -1.721366

H -1.812447 -3.982160 -1.453397

H -0.724190 -3.718009 -0.062256

C -2.296931 -1.388916 2.245823

H -3.330438 -1.346342 1.887869

H -2.282561 -1.355070 3.333398

H -1.835738 -2.323361 1.911119

C -3.026097 2.554612 -0.727939

H -3.364015 2.947515 -1.684865

H -2.967627 3.367428 0.003108

H -3.740328 1.801923 -0.378601

O 2.671905 1.064207 0.648830

C 3.181333 -0.117303 0.209953

O 2.495236 -1.098600 0.035822

C 4.660120 -0.020315 -0.011015

H 5.023252 -0.938301 -0.466523

H 5.158392 0.140069 0.948041

H 4.887762 0.836787 -0.647173

**6-*O*-Acetyl-2,3,4-tri-*O*-methyl-manno-d-pyranosyl cation**

*Local minima*

**^3^*E* conformation [R1] (0.0 kcal / mol)**

D1 = 60°

D3 = 30°

D5 = 0°

O -0.211359 -0.737675 1.001758

C 0.379901 1.160687 -0.587674

C 2.104399 -0.098615 0.662906

C 1.664056 0.334384 -0.754982

C 1.003138 -0.908532 1.247991

C -0.743968 0.330234 0.061837

H 0.042037 1.468995 -1.582749

H 2.444023 0.969128 -1.182664

H 1.216053 -1.753612 1.903049

H -1.330379 0.944407 0.741661

O 1.360008 -0.767443 -1.573071

O 0.716816 2.275585 0.208998

C -1.647417 -0.410636 -0.912664

H -1.081099 -1.142143 -1.483190

H -2.080053 0.333128 -1.584052

C 2.448828 -1.287697 -2.353514

H 2.889920 -0.495691 -2.966603

H 2.017804 -2.049221 -3.000647

H 3.212884 -1.730520 -1.713228

C -0.104756 3.431993 0.008508

H -0.053672 3.763775 -1.033399

H 0.294328 4.207041 0.659495

H -1.148691 3.240722 0.278140

O -2.684594 -1.138648 -0.245445

C -3.764454 -0.419511 0.170150

H 2.188200 0.834108 1.270269

O 3.282054 -0.853506 0.642180

C 4.178742 -0.607300 1.736856

H 4.475712 0.445821 1.758992

H 5.050849 -1.232907 1.560817

H 3.723636 -0.881641 2.693917

C -4.809968 -1.316315 0.765461

H -5.179051 -2.003469 0.000657

H -5.630607 -0.715904 1.150687

H -4.373780 -1.918156 1.564972

O -3.829064 0.779688 0.052407

**^3^*H*_4_ conformation [R2] (0.8 kcal / mol)**

D1 = 45°

D3 = 45°

D5 = 0°

O 0.234030 -0.410089 -1.283108

C -0.361890 1.181312 0.557307

C -2.114315 -0.087314 -0.663143

C -1.643385 0.349931 0.740673

C -0.984908 -0.591082 -1.495940

C 0.763856 0.379908 -0.099452

H -0.015879 1.512108 1.542080

H -2.413653 0.978582 1.193529

H -1.198933 -1.219599 -2.360789

H 1.460038 1.053519 -0.593657

O -1.325481 -0.757243 1.547939

O -0.718194 2.275131 -0.267528

C 1.525536 -0.609304 0.771223

H 0.885545 -1.423166 1.095987

H 1.898324 -0.062842 1.638862

C -2.367310 -1.205192 2.427185

H -2.689366 -0.390995 3.084485

H -1.934204 -2.003730 3.026961

H -3.217785 -1.587256 1.860615

C 0.023043 3.478347 -0.033776

H -0.119121 3.821977 0.995727

H -0.371204 4.220295 -0.725209

H 1.091775 3.340984 -0.227452

O 2.613436 -1.210926 0.056260

C 3.756548 -0.477368 -0.049600

H -2.451348 0.829487 -1.180097

O -3.098562 -1.083832 -0.624867

C -4.257122 -0.831344 -1.438754

H -4.724986 0.115728 -1.154124

H -4.942880 -1.653945 -1.249185

H -3.999608 -0.811116 -2.501974

C 4.834745 -1.259276 -0.741214

H 4.464544 -1.648928 -1.691132

H 5.116990 -2.114510 -0.122638

H 5.700548 -0.622173 -0.905304

O 3.846323 0.648693 0.374587

**^1^*C*_4_ conformation [R1] (0.9 kcal / mol)**

D1 = 45°

D3 = 60°

D5 = 45°

O -0.747014 0.650755 -1.679318

C -1.765354 0.210063 0.510476

C -0.061483 -1.358934 -0.359843

C -0.648892 -0.753381 0.923328

C 0.193775 -0.310262 -1.475861

C -1.257423 1.270612 -0.480359

H -2.134315 0.718717 1.409057

H -1.078290 -1.560120 1.523190

H 0.425370 -0.763329 -2.433594

H -2.139631 1.814281 -0.828055

O 0.313373 -0.027242 1.665620

O -2.782028 -0.562733 -0.094784

C -0.327162 2.392385 -0.049476

H -0.666806 2.854557 0.874182

H -0.290911 3.141704 -0.839617

C 0.859294 -0.724388 2.791346

H 0.067219 -0.993406 3.498250

H 1.550584 -0.034274 3.273425

H 1.393041 -1.622342 2.474289

C -4.099104 -0.021119 0.011928

H -4.378044 0.120021 1.061953

H -4.768453 -0.747157 -0.446388

H -4.196776 0.932037 -0.519357

O 1.090337 2.100490 0.215401

C 1.867685 1.238586 -0.320216

H -0.856938 -2.011242 -0.793005

O 1.084049 -2.099659 -0.040281

C 1.463774 -3.092348 -0.997421

H 0.612933 -3.733251 -1.252513

H 2.239993 -3.692608 -0.525945

H 1.870414 -2.644707 -1.909981

C 3.302293 1.338826 0.060013

H 3.490502 2.245806 0.627139

H 3.914531 1.308351 -0.842262

H 3.552169 0.459273 0.659072

O 1.569906 0.321821 -1.153305

**^3^*E* conformation [R2] (0.9 kcal / mol)**

D1 = 60°

D3 = 30°

D5 = -15°

O 0.167178 -0.728305 -0.999298

C -0.374493 1.115461 0.660785

C -2.126975 -0.010153 -0.647264

C -1.650654 0.269433 0.800317

C -1.035421 -0.748433 -1.345820

C 0.737238 0.298224 -0.031227

H -0.026203 1.380444 1.664694

H -2.421809 0.855845 1.306885

H -1.271525 -1.487991 -2.111022

H 1.309908 0.930580 -0.706633

O -1.318397 -0.894717 1.507251

O -0.711783 2.263218 -0.085014

C 1.659823 -0.477340 0.892963

H 1.112685 -1.249279 1.427142

H 2.089516 0.234012 1.600171

C -2.393146 -1.512056 2.234339

H -2.867517 -0.785044 2.900847

H -1.939684 -2.305066 2.825909

H -3.136182 -1.931206 1.554605

C 0.111414 3.409046 0.165876

H 0.059015 3.695731 1.220819

H -0.286091 4.211684 -0.451744

H 1.154831 3.227039 -0.110946

O 2.701714 -1.148933 0.172966

C 3.767018 -0.389458 -0.205850

H -2.257836 0.975069 -1.130554

O -3.278933 -0.805784 -0.690953

C -4.232688 -0.431930 -1.698497

H -4.562403 0.600272 -1.546181

H -5.074730 -1.111057 -1.586166

H -3.812181 -0.538299 -2.703553

C 4.821353 -1.231516 -0.862484

H 4.385816 -1.801998 -1.684860

H 5.217547 -1.948026 -0.139155

H 5.623076 -0.594409 -1.228217

O 3.815590 0.801275 -0.015061

**6-*O*-Acetyl-2,3,4-tri-*O*-methyl-galacto-d-pyranosyl cation**

*Local minima*

**^4^*H*_3_ conformation [R1] (0.0 kcal / mol)**

D1 = -45°

D3 = -45°

D5 = 0°

O -0.546240 -1.407387 -0.409036

C 0.336203 0.908132 0.028595

C 1.861651 -1.051102 -0.223079

C 1.575731 0.196111 0.620781

C 0.626552 -1.823532 -0.521586

C -0.885589 -0.001368 0.066372

H 2.200044 -0.693137 -1.208931

H 1.360305 -0.122273 1.649854

H 0.716140 -2.863300 -0.836257

H -1.220854 -0.177514 1.086744

O 2.767168 -1.935962 0.373634

O 2.731769 0.992689 0.580438

C -2.033046 0.456020 -0.814371

H -1.768067 0.377518 -1.864602

H -2.243327 1.497864 -0.566808

C 4.074077 -1.971176 -0.228596

H 4.024174 -2.363013 -1.248882

H 4.668358 -2.641428 0.388823

H 4.516960 -0.972891 -0.228810

C 2.960916 1.774560 1.758598

H 2.165416 2.509367 1.919316

H 3.901846 2.297815 1.599541

H 3.045602 1.129735 2.639030

O -3.204625 -0.348842 -0.636631

C -3.962442 -0.085127 0.464668

O -3.635461 0.726765 1.295432

C -5.207196 -0.922803 0.475428

H -5.752919 -0.754298 1.400734

H -4.948741 -1.978524 0.374922

H -5.833548 -0.654994 -0.378634

O 0.564460 1.262899 -1.323290

H 0.090067 1.786666 0.636735

C 1.028044 2.602446 -1.548781

H 1.044182 2.738421 -2.628860

H 0.337535 3.325964 -1.103150

H 2.030850 2.743758 -1.143313

**^4^*H*_3_ conformation [R2] (0.6 kcal / mol)**

D1 = -45°

D3 = -45°

D5 = 0°

O 0.773691 -0.974110 -0.185880

C -0.518742 1.177747 -0.358070

C -1.633590 -0.975447 0.231441

C -1.746051 0.289045 -0.641114

C -0.261069 -1.555827 0.204387

C 0.777405 0.455473 -0.705279

H -1.798275 -0.647422 1.270591

H -1.736854 -0.010367 -1.696910

H -0.110259 -2.595278 0.495901

H 0.884009 0.301693 -1.778456

O -2.531330 -1.978120 -0.168582

O -2.918639 0.993183 -0.329758

C 2.027011 1.087624 -0.125527

H 2.040552 1.001084 0.957816

H 2.036953 2.140266 -0.411057

C -3.273269 -2.595134 0.898839

H -2.613387 -3.133261 1.586098

H -3.951751 -3.300407 0.424272

H -3.842772 -1.841152 1.449959

C -4.011288 0.788370 -1.236401

H -3.746450 1.121229 -2.245437

H -4.833885 1.394777 -0.861607

H -4.310755 -0.261885 -1.261446

O 3.190633 0.481448 -0.699599

C 3.934917 -0.342408 0.097628

O 3.647110 -0.592914 1.239537

C 5.126196 -0.861158 -0.654915

H 5.703976 -1.521887 -0.013023

H 5.745742 -0.024886 -0.985444

H 4.796389 -1.398503 -1.546511

O -0.455738 1.538396 1.008915

H -0.572767 2.062572 -1.003504

C -1.050784 2.801604 1.348230

H -0.824277 2.967082 2.400101

H -0.606420 3.604503 0.750852

H -2.130001 2.779449 1.195424

**^1^*C*_4_ conformation [R1] (1.6 kcal / mol)**

D1 = 45°

D3 = 45°

D5 = 45°

O 0.182457 -0.840888 -1.821310

C -1.263453 0.731680 -0.522954

C -0.552918 -1.466683 0.462262

C -0.950915 -0.021147 0.781540

C 0.439021 -1.562812 -0.709080

C -0.226889 0.530320 -1.644622

H -0.095146 -1.918985 1.356924

H -1.842376 -0.054382 1.414802

H 0.636924 -2.588203 -1.004025

H -0.710600 0.803996 -2.583457

O -1.726018 -2.151499 0.062703

O 0.143025 0.551385 1.489900

C 1.031034 1.377246 -1.603741

H 0.793586 2.422797 -1.434589

H 1.585499 1.256751 -2.533162

C -1.764406 -3.537681 0.416265

H -0.963376 -4.105827 -0.068223

H -2.724553 -3.915511 0.069479

H -1.692045 -3.662008 1.501779

C -0.202083 1.522745 2.482418

H -0.631205 2.417939 2.029764

H 0.723932 1.781247 2.994644

H -0.905448 1.097325 3.206671

O 2.021738 1.076270 -0.549880

C 2.343641 -0.020837 0.022626

O 1.812659 -1.175084 -0.106621

C 3.504724 0.058547 0.950181

H 4.120237 -0.834031 0.843858

H 3.107306 0.075949 1.969240

H 4.082059 0.960967 0.768621

H -2.196647 0.299791 -0.906019

O -1.399689 2.126771 -0.349466

C -2.743449 2.577418 -0.153676

H -2.698388 3.663514 -0.091386

H -3.377050 2.287500 -0.998253

H -3.171924 2.182753 0.773401
